# Integrating Sequence-based GWAS and RNA-Seq Provides Novel Insights into the Genetic Basis of Mastitis and Milk Production in Dairy Cattle

**DOI:** 10.1038/srep45560

**Published:** 2017-03-30

**Authors:** Lingzhao Fang, Goutam Sahana, Guosheng Su, Ying Yu, Shengli Zhang, Mogens Sandø Lund, Peter Sørensen

**Affiliations:** 1 Center for Quantitative Genetics and Genomics, Department of Molecular Biology and Genetics, Aarhus University, 8830 Tjele, Denmark; 2 Key Laboratory of Animal Genetics, Breeding and Reproduction, Ministry of Agriculture & National Engineering Laboratory for Animal Breeding, College of Animal Science and Technology, China Agricultural University, 100193, Beijing, China

## Abstract

Connecting genome-wide association study (GWAS) to biological mechanisms underlying complex traits is a major challenge. Mastitis resistance and milk production are complex traits of economic importance in the dairy sector and are associated with intra-mammary infection (IMI). Here, we integrated IMI-relevant RNA-Seq data from Holstein cattle and sequence-based GWAS data from three dairy cattle breeds (*i.e*., Holstein, Nordic red cattle, and Jersey) to explore the genetic basis of mastitis resistance and milk production using post-GWAS analyses and a genomic feature linear mixed model. At 24 h post-IMI, genes responsive to IMI in the mammary gland were preferentially enriched for genetic variants associated with mastitis resistance rather than milk production. Response genes in the liver were mainly enriched for variants associated with mastitis resistance at an early time point (3 h) post-IMI, whereas responsive genes at later stages were enriched for associated variants with milk production. The up- and down-regulated genes were enriched for associated variants with mastitis resistance and milk production, respectively. The patterns were consistent across breeds, indicating that different breeds shared similarities in the genetic basis of these traits. Our approaches provide a framework for integrating multiple layers of data to understand the genetic architecture underlying complex traits.

A better understanding of the genetic architecture underlying complex traits and diseases would be beneficial for the genomic prediction of disease risk in personalized medicine and would support the genomic selection in livestock and plant breeding^1^,^2^,^3^,^4^. Genome-wide association studies (GWAS) have had limited successes in illustrating the genetic architecture (*e.g*., the distribution of causal variants and their effects) underlying complex traits and diseases, even with large sample sizes (n > 100,000), due to a huge number of loci with small effects^2^,^5^,^6^,^7^,^8^. Extending GWAS results to biological and genetic mechanistic hypotheses of variation in complex traits and diseases has been a major challenge^9^. To overcome this challenge, one approach could be to assess the collective evidence of the association of a phenotype with all genomic variants in a group of genes defined by prior biological knowledge^9^,^10^,^11^,^12^,^13^, as causal variants have been proposed to preferentially cluster in genes interconnected in biological processes^14^,^15^. Over the last decade, transcriptomic studies have been commonly conducted on small-scale experimental populations to identify genes involved in biological processes underlying complex traits and diseases. Genomic variants affect complex phenotypes often through modulating gene expression^16^, therefore integrating genomic variants and gene expression data could contribute to a better understanding of the genetic architecture underlying the trait variation^16^. Compared to most existing pathway annotation databases (*e.g*., Gene ontology (GO) and Kyoto Encyclopedia of Genes and Genomes), these transcriptomic studies could provide more reliable gene clusters that are functionally related to traits of interest^9^. This information is of particular interest in livestock and plant genomics due to the lack of genome annotation. Moreover, patterns of gene expression have been suggested to be more consistent across breeds and populations compared to genome-wide significant loci of GWAS^17^,^18^,^19^.

Mastitis is most often caused by invading pathogens and frequently occurs in all lactating mammals and is a significant health problem in both human and veterinary medicine^20^. Gram-negative *Escherichia coli* (*E. coli*) is one of the most common mastitis-causing pathogens^21^. In the dairy industry, mastitis is one of the costliest diseases owing to its consequences of reduced milk production and quality and the need for the treatment and replacement of animals^20^. In addition, mastitis is unfavourably genetically correlated with milk production^22^,^23^. Due to its heavy toll on the health and productivity of animals, many transcriptome profiling studies have been conducted *in vivo* or *in vitro* during intra-mammary infection (IMI) to gain a better understanding of the molecular mechanisms underlying the host response to pathogen invasion. These studies have revealed that many genes with significantly affected expression levels are involved in both inflammatory responses and overall metabolism^24^,^25^,^26^,^27^. However, few studies have investigated whether the genomic variants associated with mastitis resistance and milk production are enriched in these active transcriptome regions during IMI. As both mastitis resistance and milk production are typical complex traits controlled by a minimum of 400–4000 effective loci in cattle^2^,^28^, the genetic architecture underlying them is currently poorly elucidated. We hypothesized that integrating sequence-based GWAS results with IMI-relevant transcriptome data from different tissues could contribute to a deeper insight into the genetic architecture underlying these economically important traits.

In the present study, mastitis resistance and three milk production traits (milk, fat, and protein yields) of Nordic Holstein (HOL), Nordic red cattle (RDC) and Jersey (JER) were analysed for their associations with imputed sequence-level genotype variants. The genotype data consisted of approximately 13–15 million single nucleotide polymorphisms (SNPs) from 10,597 animals. The RNA-Seq data were generated from IMI experiments of nine HOL animals involving two tissues (liver and mammary gland) and two pathogenic factors (*E. coli* and *E. coli* endotoxin (LPS)). We assumed that the gene expression patterns induced by IMI were similar across breeds. The major objectives of this study were (1) to investigate the distributions of association signals of mastitis resistance and milk production traits in gene regions responsive to IMI, (2) to gain novel immuno-biological insights into the genetic basis of mastitis resistance and milk production, and (3) to provide a general framework for extending GWAS results to biological mechanistic hypotheses of variation in complex traits and diseases by integrative analysis with biological information from small-scale independent experimental populations.

## Results

### Single-marker GWAS based on imputed sequence markers

A single-marker GWAS using imputed sequence markers (~13–15 M SNPs) was conducted for mastitis resistance, milk, fat, and protein yields in HOL, RDC, and JER separately. The –log_10_*P*-values of the tested SNPs from GWAS analyses for the four traits in the three breeds are shown as Manhattan plots (see Supplementary Figs S1–12). The genomic inflation statistics (lambda) of all the GWAS analyses ranged from 1.04 to 1.23, indicating that the residual population-stratification effects were very small and that the GWAS test statistics were not inflated. Detailed information of the top genome-wide significant SNP on each chromosome is shown in Table 1 for each trait in the three breeds.

The SNPs with the largest effect on fat and milk yields in the three breeds were in very close proximity to the well-known fat/milk-associated *DGAT1* gene on chromosome 14 and explained 18.3% and 13.9% (HOL), 6.3% and 7.2% (RDC), and 3.1% and 2.8% (JER) of the genomic variance of fat and milk yields, respectively. By contrast, no large-effect SNPs were observed for mastitis resistance or protein yield in any of the three breeds. Notably, the top SNPs on the significantly associated chromosomes jointly explained 9.7%, 17.4%, 22.3%, and 23.9% of the variance for mastitis resistance and protein, fat, and milk yields, respectively, in HOL; 6.8%, 8.4%, 12.6%, and 13.82% in RDC; and 0%, 0%, 3.1%, and 3.9% in JER. Hence, although the GWAS results demonstrated the importance of a small number of loci with moderate to large effects, they collectively explained only a small fraction of the total genomic variance. Loci with small effects remained undetectable by GWAS due to limited sample size (especially in JER) and very stringent genome-wide significance thresholds.

### Genomic features construction based on RNA-Seq analyses of bovine liver and mammary gland data

The complete datasets with statistical results for each of the 24,616 bovine genes at different time points (*i.e*., 3, 6, 9, 12, and 48 h) post-IMI with LPS compared with a time point before IMI (*i.e*., −22 h) in the liver are available in Supplementary Table S1. The detailed results of different time points (*i.e*., 12 and 24 h) post-IMI with *E. coli* compared with a time point before IMI (*i.e*., −144 h) in the liver and that of infected mammary quarters compared with controls at 24 h post-IMI are available in Supplementary Table S2. The genomic features (*i.e*., the sets of response genes) were constructed using six FDR cut-offs (*i.e*., 0.05, 0.01, 1e-3, 1e-6, 1e-8, and 1e-10) in each experimental comparison. Ultimately, a total of 48 genomic features containing 11,446 unique genes were included for the following post-GWAS analyses (Table 2). Table 2 shows that the expression levels of many more genes in the liver were affected at 6–12 h compared with 3 (early) and 48 h (late) post-IMI with LPS, and more genes responded in the liver than in the mammary gland at 24 h post-IMI with *E. coli*.

### Post-GWAS enrichment analyses and biological interpretation

**Figure 1:**
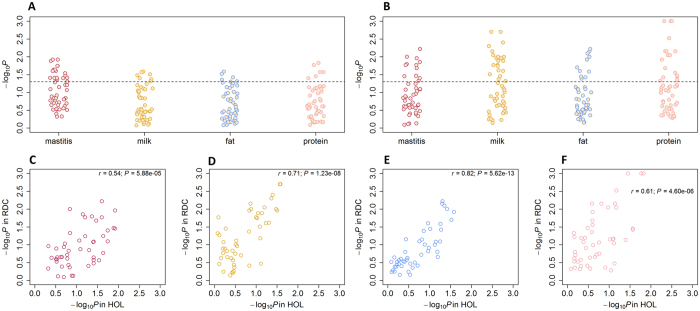
Post-GWAS analysis results for the 48 genomic features identified from RNA-Seq based on six FDR cut-offs in Holstein (HOL) and Nordic red cattle (RDC). –log_10_*P* indicates the –log_10_-transformed *P*-values from the post-GWAS analysis. *r* is the Pearson correlation of the –log_10_*P* of genomic features between HOL and RDC. *P* is the significance for the Pearson correlation test. Each point is one of the 48 genomic features. (**A**) post-GWAS results in HOL, (**B**) post-GWAS results in RDC, (**C**) Pearson correlation for mastitis resistance between HOL and RDC, (**D**) Pearson correlation for milk yield, (**E**) Pearson correlation for fat yield, (**F**) Pearson correlation for protein yield.

To investigate the distributions of association signals for mastitis resistance and milk production traits in gene regions responsive to IMI, a post-GWAS analysis of the 48 genomic features identified from RNA-Seq was applied to each trait in each of the three breeds separately. The average number of SNPs mapped in each genomic feature was 443,359 (ranging from 1,668 to 1,755,179). The –log_10_*P*-values of the genomic features from the post-GWAS analysis in HOL and RDC are shown in Fig. 1A,B, respectively, demonstrating that association signals for both mastitis resistance and milk production were significantly enriched (*P* < 0.05) in a subset of genes responsive to IMI, and the averaged Pearson correlation of –log_10_*P*-values between HOL and RDC was 0.67 across the four traits with high significance (*P* < 0.01) (Fig. 1C–F). A similar pattern was also observed between HOL and JER (see Supplementary Fig. S13). These findings indicated that certain similarities of the genetic basis underlying mastitis resistance and milk production are shared among breeds. The detailed statistical results for all the post-GWAS analyses in HOL, RDC and JER are summarized in Supplementary Tables S3, respectively.

#### Tissue differences in the enrichment of association signals for mastitis resistance and milk production

**Figure 2:**
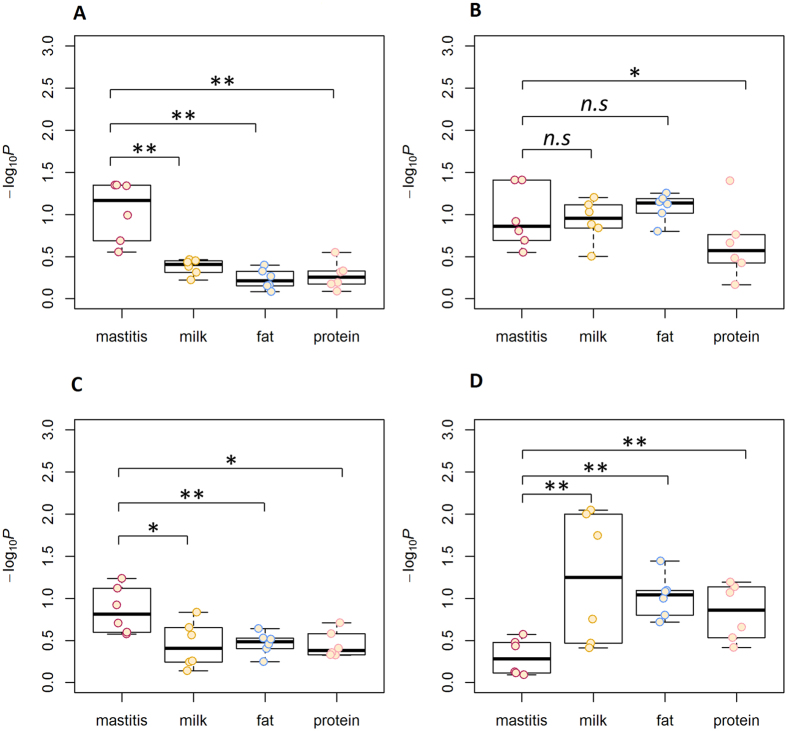
Tissue differences in the enrichment of association signals for mastitis resistance and milk production traits in Holstein (HOL) and Nordic red cattle (RDC). The genomic features were identified using six FDR cut-offs (*i.e*., 0.05, 0.01, 1e-3, 1e-6, 1e-8, 1e-10) from the mammary gland data of 24i *vs*. 24c and the liver data of 24 h *vs*. −144 h after IMI with *E. coli*, respectively. (**A** and **C**) are the analyses conducted in HOL and RDC, respectively, using genomic features defined from the mammary gland data of 24i *vs*. 24c. (**B** and **D**) are the analysis conducted in HOL and RDC, respectively, using genomic features defined from the liver data of 24 h *vs*. −144 h. Student’s *t*-test (paired) was used to test the significance of differences. *n.s* represents *P* ≥ 0.1, *represents *P* < 0.05, **represents *P* < 0.01.

The liver data from six HOL animals at 24 h post-IMI with *E. coli* compared with a time point before IMI (*i.e*., −144 h) and the mammary gland data from the same animals at 24 h post-IMI compared with the control were analysed. Figure 2A and C show that in the mammary gland, more association signals of mastitis resistance were enriched in response gene regions compared with those of milk production traits (*P* < 0.05) in both HOL and RDC, indicating that IMI mainly influenced the immune response in the mammary gland. A similar pattern was also observed in JER (see Supplementary Fig. S14). In the liver, more association signals of milk production traits tended to be enriched in response gene regions compared with those of mastitis resistance, particularly in RDC (*P* < 0.01) (Fig. 2B,D), suggesting that IMI affected the overall metabolism in the liver.

#### Dynamic impact of the hepatic transcriptome during IMI with LPS

**Figure 3:**
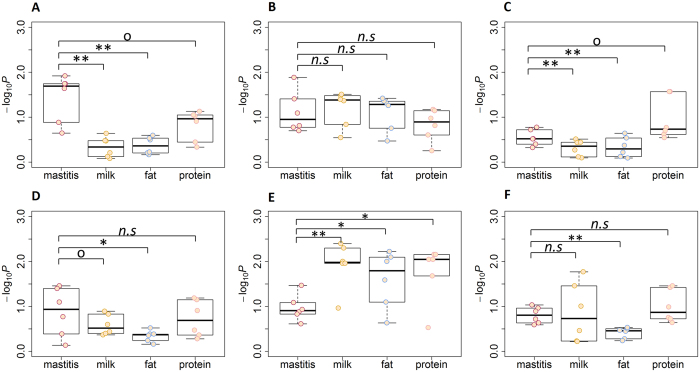
Dynamic impact on the hepatic transcriptome during intra-mammary infection (IMI) with LPS in Holstein (HOL) and Nordic red cattle (RDC). The genomic features identified in the liver data of 3 h *vs*. −22 h, 9 h *vs*. −22 h, and 48 h *vs*. −22 h comparisons post-IMI (pi) with LPS based on six cut-offs (*i.e*., 0.05, 0.01, 1e-3, 1e-6, 1e-8, 1e-10) were used for analyses. (**A** and **D**) are the analyses conducted in HOL and RDC, respectively, using genomic features defined from the liver data of 3 h *vs*. −22 h. B and E are the analyses conducted in HOL and RDC, respectively, using genomic features defined from the liver data of 9 h *vs*. −22 h. (**C** and **F**) are the analyses conducted in HOL and RDC, respectively, using genomic features defined from the liver data of 48 h *vs*. −22 h. Student’s *t*-test (paired) was used to test the significance of differences. *n.s* represents *P* ≥ 0.1, o represents *P* < 0.1, *represents *P* < 0.05, **represents *P* < 0.01.

At a very early time point (3 h) post-IMI with LPS, response genes in the liver were mainly enriched for association signals in mastitis resistance rather than in milk production, whereas at 9 h post-IMI, the response genes were enriched for association signals in both mastitis resistance and milk production (Fig. 3), except for JER milk production traits that were less associated with the response genes (see Supplementary Fig. S15). Notably, the response genes at 48 h post-IMI were more associated with protein yield compared with other traits in the three breeds (Fig. 3, see Supplementary Fig. S15). These observations provided genomic evidence that genes associated with mastitis resistance were activated initially in the liver and then genes associated with milk production traits was affected.

#### Differences in up- and down-regulated genes in the enrichment of association signals

**Figure 4:**
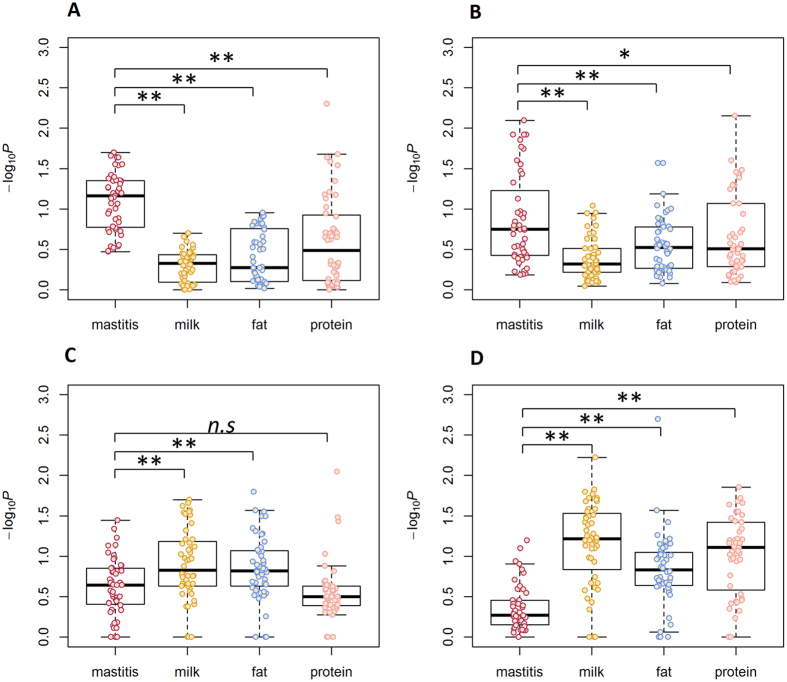
Differences in up- and down-regulated genes in the enrichment of association signals for mastitis resistance and milk production traits in Holstein (HOL) and Nordic red cattle (RDC). (**A** and **B**) are the analyses conducted in HOL and RDC, respectively, using the genomic features identified based on a log2 (fold-change) > 2 and six different FDR cut-offs (*i.e*., 0.05, 0.01, 1e-3, 1e-6, 1e-8, 1e-10). (**C** and **D**) are the analyses conducted in HOL and RDC, respectively, using the genomic features identified based on a log2 (fold-change) < −2 and six different FDR cut-offs. Student’s *t*-test (paired) was used to test the significance of differences. *n.s* represents *P* ≥ 0.1, *represents *P* < 0.05, **represents *P* < 0.01.

To explore the distributions of association signals in up- and down-regulated gene regions, we further divided each of the 48 genomic features into four subsets of up- or down-regulated genomic features based on four log_2_(fold-change) values (*i.e*., *>*2, >1, <−1, <−2). The detailed statistical information of the post-GWAS analysis for these genomic features in the three breeds is also summarized in Supplementary Tables S3–5. At 48 h post-IMI with LPS, there were no genes with an FDR < 1e-8 and log2 (fold-change) < −1 in the liver. The average number of markers mapped in the up-regulated features was 121,027 (ranging from 1,587 to 741,975), whereas the average number of markers mapped in the down-regulated features was 161,798 (ranging from 85 to 1,103,205). More association signals of mastitis resistance were enriched in the highly up-regulated features (log_2_(fold-change) > 2) compared with those of milk production with high significance (*P* < 0.01), whereas more association signals of milk production were enriched in the highly down-regulated genes (log_2_(fold-change) < −2) compared with those of mastitis resistance with high significance (Fig. 4). The patterns were consistent across the three breeds (Fig. 4, see Supplementary Fig. S16), except for JER down-regulated genes that were less associated with milk production compared with mastitis resistance (see Supplementary Fig. S16). These patterns were also observed for up- (down-) regulated genomic features with log_2_(fold-change) > 1 (<−1) (see Supplementary Figs S16–17). These observations provided genomic evidence that genes associated with mastitis resistance were activated by IMI but at the same time genes associated with the overall metabolism were inhibited.

### Explanation of genomic variance and biological interpretation for the top genomic feature in each trait

The genomic feature with the smallest *P*-value from the post-GWAS analysis in HOL was identified as the top feature for each trait. A genomic feature linear mixed model (GFLM) was applied to estimate the explained genomic variance by each of the top features (details in the Methods section).

#### Mastitis resistance

**Figure 5:**
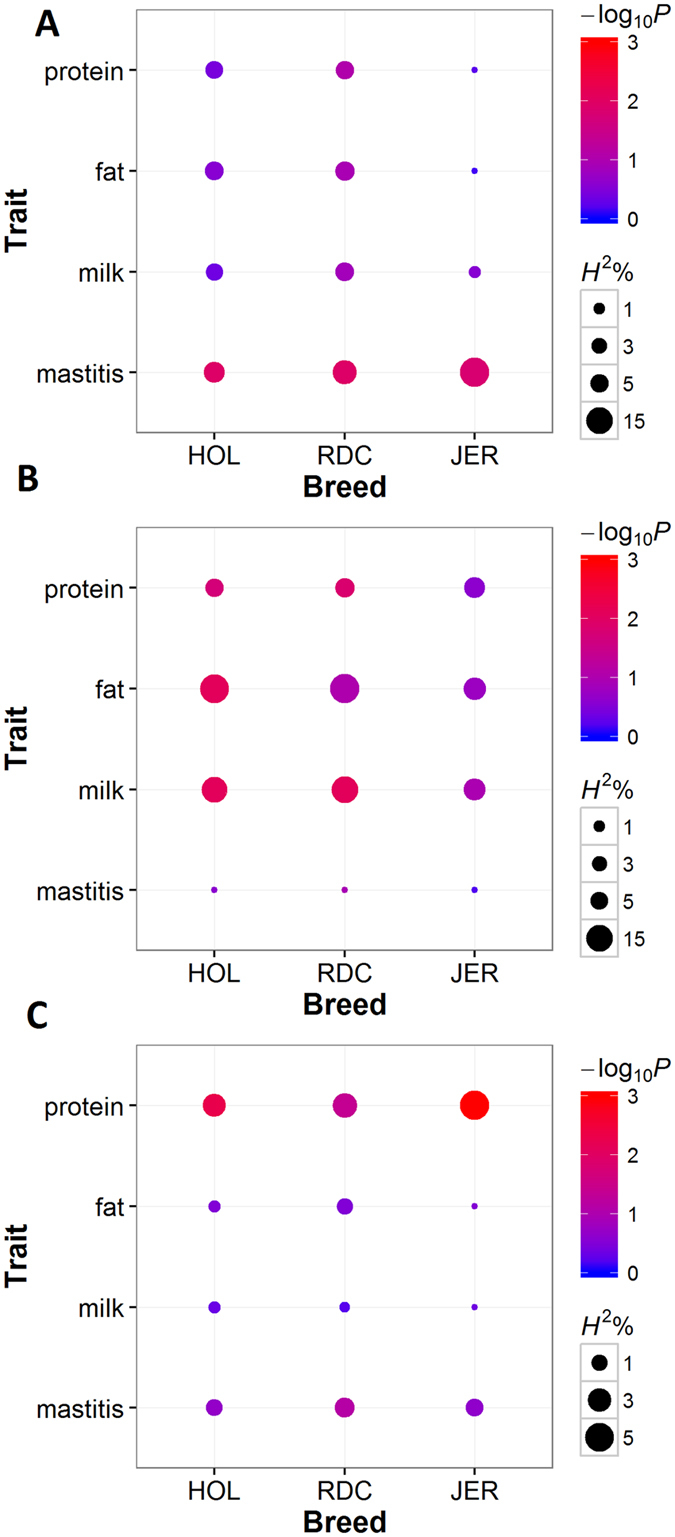
Genome feature linear mixed model (GFLM) analyses for the top genomic features in mastitis resistance and milk production traits. The point size represents the explained proportion of genomic variance by the feature (*H*^2^%); the colour represents the *P*-values of the feature from post-GWAS analyses. (**A**) the top genomic feature (FDR < 1e-6, log2(fold-change) > 1) in mastitis resistance; (**B**) the top genomic feature (FDR < 0.01, log2(fold-change) < −1) in milk and fat yield; (**C**) the top genomic feature (FDR < 1e-3, log2(fold-change) > 2) in protein yield.

**Figure 6:**
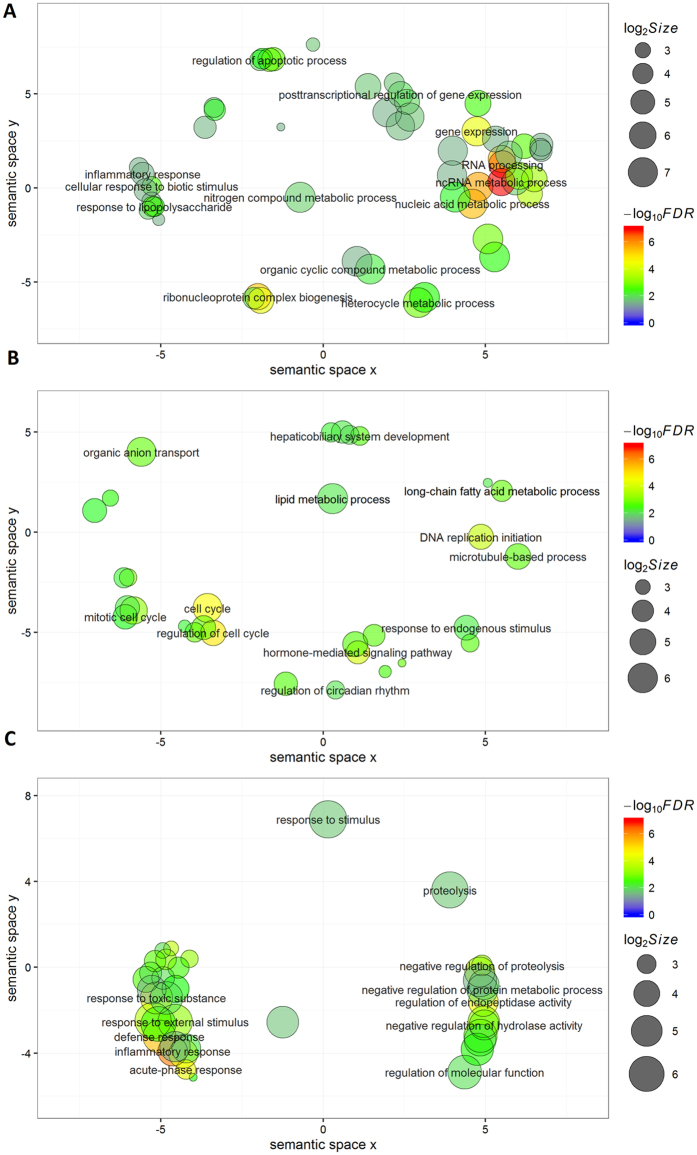
Scatterplots of enriched (FDR < 0.05) Gene Ontology (GO) terms from the functional enrichment analysis of top genomic features in mastitis resistance and milk production traits. The scatterplots show the enriched GO terms in a two-dimensional space (x and y coordinates) derived using multidimensional scaling to the matrix of semantic similarities of the GO terms. The closeness of the GO terms on a plot indicates their closeness in the GO graph structure (*i.e*., semantic similarity). The point size indicates the frequency of the GO term in the EBI GOA database; the colours represent the FDR values of GO terms from the functional enrichment analysis of genes in the top genomic feature using KOBAS2.0. (**A**) the top genomic feature (FDR < 1e-6, log2(fold-change) > 1) in mastitis resistance; (**B**) the top genomic feature (FDR < 0.01, log2(fold-change) < −1) in milk and fat yield; (**C**) the top genomic feature (FDR < 1e-3, log2(fold-change) > 2) in protein yield.

The top genomic feature (FDR < 1e-6, log2(fold-change) > 1) was identified in the liver at 6 h post-IMI with LPS, containing 1790 up-regulated genes with approximately 1% of SNPs over the whole genome. This feature explained 7.53%, 10.89%, and 18.88% of the genomic variance (*H *^2^) for mastitis resistance in HOL, RDC, and JER, respectively, approximately 5% of the variance for three milk production traits in HOL and RDC, but less than 1% of the variance for milk production traits in JER (Fig. 5A). A functional enrichment analysis of this feature demonstrated that these up-regulated genes were mainly engaged (FDR < 0.05) in RNA processing, the regulation of gene expression, the regulation of apoptotic processes, the inflammatory response, and metabolism processes (Fig. 6A). The detailed information of the top three enriched (FDR < 0.05) GO terms relevant to the immune response is summarized in Table 3.

#### Milk and fat yield

Milk and fat yield shared the same top genomic feature (FDR < 0.01, log2(fold-change) < −1), which was identified in the liver at 12 h post-IMI with *E. coli*, containing 654 down-regulated genes with approximately 0.5% of SNPs over the whole genome. This feature explained 13.34% (17.79%), 14.95% (19.32%), and 8.79% (9.49%) of the genomic variance for milk (fat) yield in HOL, RDC, and JER, respectively, and approximately 6% of the variance for protein yield and less than 0.01% of the variance for mastitis resistance in the three breeds (Fig. 5B). A functional enrichment analysis of this feature revealed that these down-regulated genes participated in multiple biological functions, including cell cycle regulation, hepatobiliary system development, lipid metabolic processes and long-chain fatty acid metabolic processes (Fig. 6B). The details of the enriched GO terms relevant to metabolic processes are summarized in Table 4.

#### Protein yield

The top genomic feature for protein yield (FDR < 1e-3, log2(fold-change) > 2) was identified in the liver at 48 h post-IMI with LPS, containing 48 highly up-regulated genes with less than 0.01% of SNPs over the whole genome. This feature explained 2.67%, 3.31%, and 5.33% of the genomic variance for protein yield in HOL, RDC and JER, respectively, 1.09%, 1.89%, and 1.34% of the variance for mastitis resistance, respectively, but less than 1% of the variance for milk and fat yield in the three breeds (Fig. 5C). A functional enrichment analysis of this feature revealed that these up-regulated genes were involved (FDR < 0.05) in multiple biological processes that were mainly relevant to inflammatory and defence responses and the regulation of protein metabolic processes (Fig. 6C). The details of the top three enriched GO terms relevant to metabolic processes and the top three enriched GO terms for immune response are summarized in Table 5.

## Discussion

To the best of our knowledge, this study is the first to integrate sequence-based GWAS and IMI-relevant transcriptome data to exploit the genetic basis underpinning mastitis resistance and milk production in dairy cattle. We provide genomic evidence that genes in the mammary gland responding to IMI were more associated with mastitis resistance than milk production. Moreover, responsive genes in the liver played roles not only in the regulation of the immune response but also in the dysregulation of overall metabolism, providing novel immuno-biological insights into the genetic mechanisms underlying the unfavourable correlation between mastitis and milk production. The patterns were consistent across breeds, revealing that different breeds could share similarities in genetic architecture underlying mastitis resistance and milk production. This finding is in line with previous observations that the innate immune response to IMI remains highly conserved among breeds^29^,^30^. Our findings here might indicate that it is possible to improve multi-breed genomic predictions by borrowing information across breeds, which is currently a major ongoing challenge in the animal breeding area^31^. However, in several analyses, slightly different results for Jersey compared to Nordic Red and Holstein were observed. These differences are probably due to the breed differences in segregating QTLs, minor allele frequencies, and SNP effects. In addition, the smaller sample size for Jersey animals may also have resulted in lower power to detect the associated SNPs compared to Nordic red and Holstein cattle.

The global gene expression alterations in the mammary gland and liver during IMI with *E. coli* and LPS as determined by microarray analyses have been previously reported using the same samples as those in the current study^24^,^27^,^32^. Compared to microarray technology, RNA-Seq has several advantages, including a greater dynamic range, higher reproducibility, less bias, and a lower frequency of false-positive signals^33^. A previous study^34^ re-analysed the microarray dataset of Jiang *et al*.^24^ using a dynamic impact approach (DIA) and found that at 3 h post-IMI with LPS, all pathways activated in the liver were primarily related to the innate immune system, with this activation persisting for up to 12 h. The authors found that between 6 and 12 h post-IMI, pathways related to metabolism were strongly inhibited, whereas the transcriptional response subsided at 48 h post-IMI. This result is in agreement with our current findings. Together, these findings from both transcriptome functional annotation and genome association analyses confirm that soon after IMI, the liver initially increases its immune response (*e.g*., increased production of acute phase proteins) and then decreases its overall metabolism, particularly of lipids and cholesterol^35^. There is clear evidence to indicate that the immune response in the liver is highly integrated with metabolic regulation and that the biological dysfunction of either could severely impact the other^36^, as the liver is a crucial organ for host immune responses and metabolism, including lipogenesis, gluconeogenesis, and cholesterol metabolism^37^,^38^.

Single-marker GWAS has limitations for deciphering the genetic and biological mechanisms underlying complex traits, therefore many studies using different strategies have been conducted to investigate the distributions of causal genomic variants contributing to complex phenotypes along the genome^1^,^13^,^28^,^39^.

Secondary analyses of GWAS results (*i.e*., post-GWAS) based on prior biological knowledge have been suggested as a computationally simple way to extract additional information from genome-wide marker data^12^. This approach has the potential to identify joint effects of multiple markers with independent subtle effects in a genomic feature that may be missed when estimated individually. Furthermore, statistical analysis incorporating external biological information can provide novel insights into the mechanisms causing phenotype variation, helping to open the “black box” of the genetic architecture underlying complex traits. A host of methods for this type of post-GWAS analysis have been developed to date^40^. A commonly used approach is count-based; that is, to compare the proportion of associations over a certain pre-defined significance threshold in the genomic feature to the proportion of such associations in the remaining genome^41^,^42^,^43^. One major limitation of this type approach is the dichotomization of associations into significant and non-significant groups based on a pre-specified significance cut-off, which ignores information about the strength of association^44^,^45^. Our post-GWAS approach assessed the enrichment of association signals in a genomic feature by comparing the sum of squared single marker test statistics (*i.e*., *t*^2^) within the region to an empirically derived distribution under a competitive null hypothesis. This approach is more likely to match the genetic architecture underlying complex phenotypes, whereby genetic variation is governed by many loci with small effects. Our previous studies^44^,^45^ using simulations have shown that the performance of this procedure is better or similar to other approaches (*e.g*., count or score-based) in most scenarios, and the number of false positives could be effectively controlled when the following criteria are met: 1) the average number of markers in each gene is approximately the same among the genomic features, and 2) the average linkage disequilibrium (LD) between markers in different genes is approximately the same^44^,^45^.

Our current GFLM approach could be an alternative way to examine the collective contribution of markers in a genomic feature to the phenotypic variation. It is based on partitioning genomic variance into two components: markers within and outside a genomic feature. We previously applied this approach to partition the genomic variance in health and milk production traits based on pathways^13^. This approach is similar to those proposed by other investigators, who used multiple variance components based on markers belonging to different chromosomes or sequence ontologies^39^,^46^. Here, we examined genomic markers in response gene regions detected from IMI-transcriptomic studies, which were more likely to be associated with mastitis and milk production. Moreover, our GFLM approach builds on a solid statistical modelling framework that is commonly applied to predict genetic values in animals and plants in genomic selection programmes^47^. Compared to the standard genomic best linear unbiased prediction (GBLUP) model, whereby the genetic marker relationships are weighted equally^47^, our GFLM approach could improve the ability to predict genomic values for complex traits through differential weighting of the individual genetic marker relationships based on the estimated genomic parameters, provided that causal genomic variants are enriched in the genomic feature^48^. The GFLM approach is more likely to match the genetic architecture of complex traits compared to GBLUP^48^. Additionally, the multiple-trait GFLM can be used to further disentangle the negative genetic correlation between mastitis and milk production in future studies.

In principle, many genomic features can be constructed using prior information from different sources, such as single genes, haplotypes, chromosomes, sequence ontologies, biological pathways, and experimental studies. The gain in knowledge generated by their use relies heavily on the quality of the genomic feature classification strategies on which the marker sets are based. As trait-associated genomic markers are not uniformly, or necessarily physically, clustered in the genome^14^,^39^, dissecting genomic variance using adjacent genomic regions is not an ideal way to detect the joint effect of many loci with small effects and does not facilitate the interpretation of biological mechanisms underlying the trait. Biological interpretation may be better served by the use of biological pathways as genomic features; however, the quantity and quality of genes that are functionally annotated in current pathway databases are limited^9^, particularly for livestock and plant genomes. Here, we used information from our transcriptomic studies of a small-scale experimental population to define genomic features, providing novel insights into the genetic and biological basis of mastitis and milk production traits. Our approaches can be easily extended to use diverse types of biological knowledge obtained from costly high-throughput technologies (*e.g*., RNA-Seq, methylation-Seq, and ChIP-Seq) in small-scale samples to assist in the understanding of the genetic architecture and biological mechanisms underlying complex traits at the population level. However, because gene expression patterns are highly time- and tissue-dependent, some trait-associated genes might not show differential expression in some tissues at a certain time. Therefore, incorporating more biological information (*e.g*., protein and metabolite levels) related to the studied complex traits could be important for understanding the flow of biological information underpinning complex traits, which will help us identify the appropriate genomic features that are highly enriched for causal variants. Our current genomic feature modelling approaches provide a general framework to investigate and integrate multiple layers of omics data from high-throughput technologies or existing pathway annotation databases, potentially leading to a better understanding of the genetic and biological basis underlying complex traits and diseases.

## Materials and Methods

### Animal biopsy samples for IMI experiments

All experimental procedures involving animals were approved by the Danish Animal Experiments Inspectorate and complied with Danish Ministry of Justice laws concerning animal experimentation and care of experimental animals. The animal experiments were conducted in strict accordance with regulations and guidelines established by these committees. An inspection was carried out by members of these committees during the animal infection experiments.

In total, three and six healthy Holstein animals at the early stage of their first lactation were used in the following two IMI experiments, respectively. For the first IMI experiment, the udder health of the three Holstein cows was evaluated based on bacteriological examinations and somatic cell count (SCC) before LPS treatment. All cows were free of mastitis-causing pathogens and had SCCs < 138,000 cells/ml in all the quarters. At the start of the trial, the right front quarter of all the cows was inoculated with 200 μg of *E. coli* LPS (0111:B4) (Sigma-Aldrich, Brøndby, Denmark) dissolved in 10 ml of a 0.9% NaCl solution, whereas the left front quarter received 10 ml of the 0.9% NaCl solution as a control. The following clinical findings verified the induction of mastitis by LPS, such as fever and high SCCs in the milk of the infected quarters. Liver biopsies were collected at −22, 3, 6, 9, 12, and 48 h relative to LPS treatment in all the studied cows. The detailed information of the liver biopsy samples from the three Holstein cows post-IMI with LPS has been previously described by Jiang *et al*.^24^.

For the second IMI experiment, prior to the *E. coli* treatment, all six Holstein animals were evaluated and had normal body temperature and white blood cell count, a negative glutaraldehyde test, and low Californian Mastitis Test (CMT; (Kruuse, Marslev, DK)) scores ranging from 1 to 5. They were found to be free from mastitis-causing pathogens and had SCCs in milk <27,000 cells/ml in all the quarters. One of the front quarters from each of the six animals was infused with 10 ml of a 0.9% NaCl solution containing ~20–40 CFU of live *E. coli*, whereas another quarter serving as a control received 10 ml of the 0.9% NaCl solution. The details of the liver biopsy samples from the six cows at −144, 12 and 24 h post-IMI with *E. coli* have been previously described by Jorgensen *et al*.^32^, whereas the mammary gland samples of the infected and control quarters from the same animals at 24 h post-IMI have been described previously by Buitenhuis *et al*.^27^.

### RNA sequencing and statistical analysis

RNA-Seq libraries were prepared from liver samples collected at −22, 3, 6, 9, 12, and 48 h post-IMI with LPS (each condition with three biological replicates), liver tissue samples from −144, 12, and 24 h post-IMI with *E. coli* (each condition with six biological replicates), and mammary gland tissues collected from udder quarters at 24 h post-IMI with and without *E. coli* infection (each condition with six biological replicates). The RNA extraction was performed as described previously^50^. In total, 48 RNA libraries were constructed for RNA-Seq. Each sample (*i.e*., each library) was then sequenced using a 100 bp paired-end method with Illumina HiSeq 2000 sequencing technology by AROS Applied Biotechnology (Aarhus, Denmark).

The approaches applied for RNA-Seq bioinformatics analyses have been previously described^49^. Briefly, the indices of the bovine reference genome (UMD3.1) were first built using the Build-Index function implemented in the Rsubread package^51^. The sequence reads of each sample were then mapped to the bovine reference genome assembly with an efficient mapping program using the seed-and-vote algorithm^51^ implemented in the Rsubread package in R/Bioconductor. The number of reads mapped to 24,616 Ensemble genes was counted using the function Feature-Counts in this package. The averaged uniquely mapping rate across all samples was 87.11%. The analysis of differential gene expression was conducted using edgeR^52^. The weighted trimmed means of M-values were used to normalize the count data. As the count data follow non-normal distributions and commonly exhibit a quadratic mean-variance relationship, a negative binomial generalized linear model (GLM) was used. To ensure stable inference, an empirical Bayes approach was applied to shrink gene-wise dispersions towards a common dispersion for all tested genes. The GLM allow adjustment for relevant factors in the experimental design, and the differential expression of each gene was determined based on a likelihood ratio test. Time (*i.e*., different time-points post-IMI) was considered as the only effect for the liver samples, whereas infection status (*i.e*., Infected and Control) was included in the model for the mammary gland samples. The statistical tests for each analysis were adjusted for multiple testing using the FDR method^53^.

### Single-marker GWAS based on imputed sequence markers

The definitions of milk production traits (milk, protein, and fat yields) and mastitis resistance were standardized among the Nordic countries. The phenotypes used for the single-marker association analysis were de-regressed proofs (*i.e*., de-regressed breeding values) of 5,056 HOL, 4,310 RDC, and 1,231 JER cattle from routine genetic evaluation by Nordic Cattle Genetic Evaluation (http://www.nordicebv.info/). The average reliabilities of the de-regressed proofs for milk, fat, and protein yields and mastitis resistance were 0.95, 0.95, 0.95, and 0.85, respectively, in HOL; 0.95, 0.95, 0.95, and 0.83, respectively, in RDC; and 0.92, 0.92, 0.92, and 0.76, respectively, in JER. The details of the imputation from 50 K and High-Density (HD, 700 K) genotypes of these cattle to whole-genome sequence data have been described previously^54^. Briefly, a multi-breed reference of 3,383 individuals (1,222 HOL, 1,326 RDC, and 835 JER) with HD genotypes was used to impute individuals with 50 K genotypes to the HD level. Individuals with the imputed HD genotypes were then imputed to the whole-sequence level using a multi-breed reference of 1,222 individuals from *run4* of the 1,000 Bull Genome project (288 HOL, 56 RDC, 61 JER and 743 individuals from different breeds)^55^ and private sequence data from Aarhus University (23 HOL, 30 RDC and 27 JER)^54^. IMPUTE2 v2.3.1^56^ was applied to impute 50 K to the HD level, and Minimac2^57^ was used to impute HD to the whole-sequence level. Ultimately, a total of 15,355,382, 15,243,827, and 13,403,916 SNPs (minor allele frequency, MAF > 0.01 and deviation from Hardy-Weinberg proportions >10e-6) were included for GWAS in HOL, RDC, and JER, respectively.

An association analysis for the imputed sequence SNPs was performed using a two-step variance component-based approach, to account for population stratification, implemented in EMMAX^58^. The detailed information about this model was described by Kang *et al*.^58^. In the first step, the polygenic and residual variances were assessed using the following linear model:


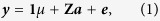


where ***y*** is a vector of phenotype (*i.e*., de-regressed proofs); **1** is a vector of ones; *μ* is the overall mean; **Z** is a design matrix relating phenotypes to random polygenic effects; ***a*** is a vector of random polygenic effects (*i.e*., breeding values), where ***a***~N(**0**, 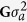), and **G** is the genome relationship matrix constructed by EMMAX using HD genotypes, excluding the chromosome harbouring the candidate SNP for controlling double fitting (*i.e*., fitting the SNP as a fixed effect for testing association and a random effect as part of the **G**)^59^, and 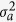 is the additive genetic variance; and ***e*** is the vector of residuals, where ***e***~N(**0**,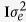), and **I** is an identity matrix. In the next step, the individual sequence-level SNP effect was assessed using the following linear regression model:


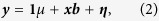


where ***y**, μ*, and **1** are as described above, ***x*** is a vector of imputed genotype dosages (ranging from 0 to 2), ***b*** is the allele substitution effect, and ***η*** is a vector of random residual deviates with (co)variance structure 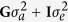. A Bonferroni approach was used to correct multiple testing. After correction, the genome-wide significance thresholds corresponding to an error rate of 0.05 were set at 3.3 × 10^−9^, 3.3 × 10^−9^, and 3.7 × 10^−9^. Manhattan plots were created using *qqman* v.0.1.2 in the R package^60^. The genomic inflation statistic (lambda) of GWAS was calculated as the median of the resulting chi-squared test statistics divided by the expected median of the chi-squared distribution with one degree of freedom (*i.e*., 0.454). The variance explained by an individual SNP was calculated as follows:


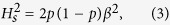


where 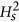 is the additive genomic variance explained by one SNP, *p* is the allele frequency, and *β* is the SNP effect estimated from GWAS.

### Post-GWAS enrichment analysis

Considering the genes detected in RNA-Seq as genomic features, a post-GWAS analysis was performed based on the GWAS results, where test statistics were obtained for the association of each individual SNP. The imputed sequence SNPs were mapped to the bovine reference genome (UMD3.1). A genetic marker was considered as relevant to a gene if the chromosome position of the marker was between the start and end positions of the gene^13^. The following summary test statistic was calculated for a genomic feature:


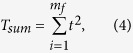


where *m*_*f*_ is the number of markers located in a genomic feature, and *t*^2^ is the squared of *t* (*i.e*., the estimated effect of a marker divided by its standard error). The approach applied to test the association between a phenotype and a genomic feature has been described previously^44^,^45^. Briefly, the observed test statistic (*e.g*., *t*^2^) was first ranked based on the chromosome position of the markers, and a test statistic was then randomly chosen from this vector. All test statistics were moved to the new positions, with the remaining markers maintaining the original order, whereby the chosen statistic became the first. This uncoupled any associations between markers and genomic features while maintaining the correlation structure among the test statistics. Afterward, a new summary statistic of a genomic feature was calculated based on the original position of the feature. The permutation was repeated 1,000 times for each studied genomic feature, and an empirical *P*-value was then calculated based on one-tailed tests of the proportion of randomly sampled summary statistics larger than that observed. Here, we used response genes detected in RNA-Seq to define genomic features. Genes were thus used as the sampling units in the permutation procedure. Our previous simulation studies demonstrated that this post-GWAS method performs better than or equal to other commonly used methods (*e.g*., count or score-based approaches) in most situations, whereby the genetic variations of the traits are controlled by a large number of loci with small effects^44^,^45^.

### Genomic feature-variance component analysis

By grouping markers into two sets (the genomic feature and the remaining genome), a genomic feature linear mixed model (GFLM) was used to assess the joint contribution of a set of markers in a genomic feature to a phenotype. The model is


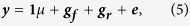


where ***y*** is the vector of phenotype (*i.e*., de-regressed proofs), **1** is a vector of ones, *μ* is the overall mean, ***g***_***f***_ is the vector of genomic values captured by markers in the genomic feature, ***g***_***r***_ is the vector of genomic values captured by markers in the remaining genome, and ***e*** is the vector of residuals. The assumptions for all the random effects are given by


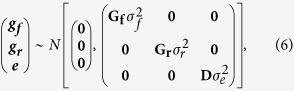


where **G**_**f**_ and **G**_**r**_ are genomic relationship matrices, built based on the markers located in the genomic feature and the remaining genome, respectively. These **G** matrices were built using the second method described by VanRaden (2008)^61^. **D** is a diagonal matrix with diagonal elements equal to 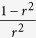, where *r*^2^ is the reliability of the de-regressed proof, and 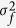, 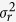, and 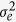 are the variance components accounted for by the markers in the genomic feature and the remaining genome and residuals, respectively.

The proportion of genomic variance explained by a genomic feature was calculated as


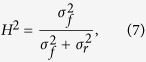


### Downstream bioinformatics analyses of DEG sets of interest

The genomic feature with the smallest *P*-value detected in HOL for each of the studied traits was considered to be of interest, and these features were subjected to functional enrichment analyses using a web-based tool, KOBAS2.0^62^ (http://kobas.cbi.pku.edu.cn/home.do). A hypergeometric gene set enrichment test, based on a gene ontology (GO) database, was applied, and the *P*-values for each cluster were corrected using the FDR method. The semantic similarities among the enriched GO terms (FDR < 0.05) were calculated using the SimRel approach^63^ implemented in REVIGO^64^ (http://revigo.irb.hr/). The detailed information for assigning x and y coordinates to each GO term to ensure more semantically similar terms are close in the scatterplots has been previously described^64^. Briefly, a multidimensional scaling procedure was applied to initially place the GO terms based on an eigenvalue decomposition of the pairwise distance matrix of the GO terms, followed by a stress minimization step that iteratively enhances the agreement between the terms’ closeness and their semantic similarities in the two-dimensional space.

### Availability of data

The RNA-Seq data were submitted to NCBI with the accession number GSE75379. All genomic annotation data defining gene regions are publicly available in Ensembl (ftp://ftp.ensembl.org/pub/release-84/gtf/bos_taurus). The whole-genome sequencing data from the 1000 Bull Genomes Project are publicly available as sequence data at NCBI with SRA no. SRP039339 (http://www.ncbi.nlm.nih.gov/bioproject/PRJNA238491) and variations in dbSNP (http://www.ncbi.nlm.nih.gov/projects/SNP/). The phenotype and imputed sequence genotype data are available only upon agreement with the commercial breeding organization (http://www.vikinggenetics.com/) and should be requested directly from the authors or the breeding organization.

## Additional Information

**How to cite this article**: Fang, L. *et al*. Integrating Sequence-based GWAS and RNA-Seq Provides Novel Insights into the Genetic Basis of Mastitis and Milk Production in Dairy Cattle. *Sci. Rep.* **7**, 45560; doi: 10.1038/srep45560 (2017).

**Publisher's note:** Springer Nature remains neutral with regard to jurisdictional claims in published maps and institutional affiliations.

## Supplementary Material

Supplementary Information

## Figures and Tables

**Table 1:** Summary of the top SNP on each chromosome with genome-wide significance determined by single-marker genome-wide association analyses (GWAS) of each trait in three cattle breeds.

Trait	Breed	Chromosome	Position of the top SNP (bp)	*P*-value	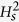 (%)
Mastitis resistance	HOL	6	88,840,407	5.55e-19	4.1
		13	59,260,175	2.09e-11	2.0
		18	43,909,571	2.44e-10	1.9
		23	11,477,969	1.10e-13	1.3
		25	35,353,527	2.89e-10	0.4
	RDC	6	88,723,742	2.31e-10	2.3
		24	60,959,835	3.72e-10	4.5
Protein yield	HOL	6	88,478,678	9.79e-10	1.5
		14	1,892,784	6.93e-22	2.9
		18	57,015,407	4.23e-11	1.2
		23	10,504,197	4.80e-11	5.0
		25	36,403,719	8.36e-14	0.9
		26	41,231,611	4.72e-19	1.4
		28	10,749,791	1.91e-09	1.9
		29	12,741,604	3.38e-10	2.6
	RDC	5	112,450,860	1.32e-09	1.1
		14	1,802,667	3.02e-09	1.1
		23	8,581,891	1.22e-09	0.8
		25	3,498,960	1.98e-12	1.0
		26	10,268,885	4.48e-10	4.4
Fat yield	HOL	5	93,945,991	8.95e-26	1.8
		14	1,810,124	3.64e-132	18.3
		26	20,547,445	2.06e-22	1.5
		29	17,696,734	1.82e-10	0.7
	RDC	5	93,945,694	3.23e-28	2.6
		14	1,807,140	9.80e-43	6.3
		23	28,567,796	4.35e-10	0.8
		25	9,870,005	3.98e-15	1.3
		26	24,379,571	5.20e-15	1.6
	JER	14	1,802,667	9.36e-15	3.1
Milk yield	HOL	5	93,944,849	8.79e-16	2.2
		14	1,825,125	6.16e-86	13.9
		20	29,996,727	1.79e-12	2.3
		23	17,821,120	1.55e-09	4.3
		26	37,869,380	3.80e-15	1.2
	RDC	5	112,343,204	2.00e-09	1.1
		14	1,743,939	9.75e-34	7.2
		16	1,322,611	2.63e-09	0.95
		19	61,449,096	1.19e-09	0.55
		20	31,909,478	2.78e-16	3.2
		25	3,498,960	4.83e-10	0.82
	JER	14	1,828,456	1.21e-21	2.8
		20	33,922,713	3.38e-09	1.1

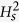
 (%) represents the proportion of genomic variance explained by the top SNP. HOL, Nordic Holstein; RDC, Nordic red; JER, Jersey.

**Table 2:** The number of genes responsive to intra-mammary infection in each of 48 genomic features defined at six FDR cut-offs (*i.e*. 0.05, 0.01, 1e-3, 1e-6, 1e-8, 1e-10) in eight experimental comparisons.

Tissue	Pathogenic factor	Comparison (h)	No._0.05_	No._0.01_	No._1e-3_	No._1e-6_	No._1e-8_	No._1e-10_
Liver	LPS	3 *vs*. −22	1163	826	583	289	192	139
Liver	LPS	6 *vs*. −22	7888	6800	5422	3047	2172	1638
Liver	LPS	9 *vs*. −22	8014	6859	5559	3312	2504	1923
Liver	LPS	12 *vs*. −22	6998	5809	4430	2402	1768	1319
Liver	LPS	48 *vs*. −22	483	240	128	48	32	25
Liver	*E. coli*	12 *vs*. −144	4183	2835	1576	390	199	102
Liver	*E. coli*	24 *vs*. −144	4650	3537	2228	837	525	316
Mammary	*E. coli*	24i *vs*. 24c	2308	1533	996	365	227	146

24i *vs*. 24c: comparison between infected mammary glands and controls at 24 h post IMI with *E. coli*.

**Table 3:** The top three enriched (FDR < 0.05) Gene Ontology (GO) terms relevant to the immune response detected by a functional enrichment analysis of the top genomic feature (FDR < 1e-6, log2(fold-change) > 1) in Holstein (HOL) mastitis resistance.

GO term	GO ID	FDR	Gene
Cellular response to biotic stimulus	GO:0071216	2.79E-3	*PPP1R15B, TLR2, MAPK14, CCL2, SCARB1, PRDM1, NFKBIA, TNFAIP3, EIF2AK3, TP53, DDIT3, XBP1, TRAF6, LITAF, ANKRD1, PLSCR4, PDE4B, TLR4, SERPINE1, ZC3H12A, LBP, TRIB1, RELA, MYD88, IRAK2, NLRP3, SBNO2*
Response to lipopolysaccharide	GO:0032496	4.29E-3	*NOCT, IFNAR1, MAPK14, CCL2, SCARB1, PRDM1, NFKBIA, AKIRIN2, SRR, TNFAIP3, CYP27B1, XBP1, TRAF6, LITAF, ANKRD1, PLSCR4, PDE4B, TLR4, SERPINE1, LTBR, TNFRSF1A, TNFRSF6B, ZC3H12A, LBP, CD40, TRIB1, RELA, MYD88, IRAK2, NLRP3, SBNO2, GCH1, JUNB*
Inflammatory response	GO:0006954	4.30E-3	*CHI3L1, CASP4, TLR2, IL10, SMAD1, MAPK14, ALOX5AP, CCL2, NLRC4, S100A8, S100A12, S100A9, TNFAIP3, APOD, BCL6, IL1B, IL1RN, ENSBTAG00000006354, B4GALT1, CCL19, NR1H4, S1PR3, HMOX1, IL20RB, TLR4, IL4R, RELB, OLR1, A2M, CD163, SERPINE1, LTBR, TNFRSF1A, TNFRSF6B, ZC3H12A, TNFAIP6, ENSBTAG00000022394, ENSBTAG00000002963, LBP, ENSBTAG00000037558, CD40, RELA, CCL20, MYD88, SEH1L, NLRP3, ITIH4, SBNO2, TNIP1, SNAP23, HIF1A, SOCS3*

**Table 4:** The three enriched (FDR < 0.05) Gene Ontology (GO) terms relevant to metabolism processes detected by a functional enrichment analysis of the top genomic feature (FDR < 0.01, log2(fold-change) < −1) in Holstein (HOL) milk and fat yield.

GO term	GO ID	FDR	Gene
long-chain fatty acid metabolic process	GO:0001676	2.06E-3	*ACSBG1, ENSBTAG00000031933, ENSBTAG00000003272, ENSBTAG00000013693, SLC27A1, CPT1A*
bile acid biosynthetic process	GO:0006699	7.69E-3	*HNF1A, NR1D1, CYP7A1*
lipid metabolic process	GO:0006629	8.10E-3	*ST3GAL2, SDR42E1, OXSM, ACSBG1, PIGS, SRD5A1, RUBCN, SMPD3, NPC1L1, ENSBTAG00000031933, HNF1A, ABCD3, SLC35C1, ENSBTAG00000003272, ID2, ENSBTAG00000013693, INPP1, HSD3B7, NR1D1, DOLPP1, GPAM, PDK1, SLC27A1, INSIG2, FITM2, HNF4A, SNAI2, CYP7A1, CPT1A, PIGV, PLPP2, AJUBA, IP6K2, IRS1*

**Table 5:** The top six enriched (FDR < 0.05) Gene Ontology (GO) terms relevant to metabolism processes and the immune response detected by a functional enrichment analysis of the top genomic feature (FDR < 1e-3, log2(fold-change) > 2) in Holstein (HOL) protein yield.

GO term	GO ID	FDR	Gene
negative regulation of cellular protein metabolic process	GO:0032269	1.51E-5	*ENSBTAG00000046540, SERPINA3*-*6, ENSBTAG00000007043, ENSBTAG00000007041, A2M, ITIH4, SOCS3*
regulation of endopeptidase activity	GO:0052548	2.50E-5	*S100A8, S100A9, ENSBTAG00000046540, SERPINA3*-*6, ENSBTAG00000007043, ENSBTAG00000007041, A2M, ITIH4*
enzyme inhibitor activity	GO:0004857	3.02E-5	*ENSBTAG00000046540, SERPINA3*-*6, ENSBTAG00000007043, ENSBTAG00000007041, A2M, SCGB1A1, ITIH4, SOCS3*
inflammatory response	GO:0006954	4.78E-7	*CHI3L1, ALOX5AP, S100A8, S100A12, S100A9, ENSBTAG00000006354, A2M, ENSBTAG00000022394, LBP, ITIH4, SOCS3*
detoxification	GO:0098754	2.42E-6	*ENSBTAG00000001595, S100A8, S100A9, ENSBTAG00000006354, CSN1S1, GPX3*
defense response	GO:0006952	3.22E-6	*CHI3L1, ALOX5AP, S100A8, S100A12, S100A9, ENSBTAG00000006354, A2M, ENSBTAG00000022394, LBP, ENSBTAG00000048250, ENSBTAG00000005005, FGFBP1, ITIH4, SOCS3*
