# Supplementary material for: Integrating Sequence-based GWAS and RNA-Seq Provides Novel Insights into the Genetic Basis of Mastitis and Milk Production in Dairy Cattle
Source: Sci Rep. 2017 Mar 30;7:45560. doi: 10.1038/srep45560 (PMC5372096; doi:10.1038/srep45560)
Supplement: Supplementary Information [file srep45560-s1.pdf]

Integrating Sequence-based GWAS and RNA-Seq Provides Novel Insights into the Genetic Basis of Mastitis and Milk Production in Dairy Cattle

Lingzhao Fang[1,2,*], Goutam Sahana[1], Guosheng Su[1], Ying Yu[2], Shengli Zhang[2], Mogens Sandø Lund[1], Peter Sørensen[1]

[1]Center for Quantitative Genetics and Genomics, Department of Molecular Biology and Genetics, Aarhus University, 8830 Tjele, Denmark

[2]Key Laboratory of Animal Genetics, Breeding and Reproduction, Ministry of Agriculture & National Engineering Laboratory for Animal Breeding, College of Animal Science and Technology, China Agricultural University, 100193, Beijing, China

[*] **Corresponding Author Information:**

Lingzhao Fang

Blichers Allé 20

8830 Tjele

Denmark

+4587157971

lingzhao.fang@mbg.au.dk

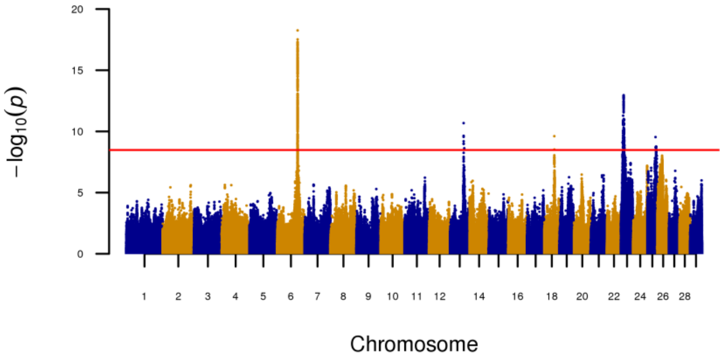

Figure S1. Manhattan plots of –log$_{10}$*P*-value for each of imputed sequence SNPs from signal-marker GWAS in Holstein (HOL) mastitis

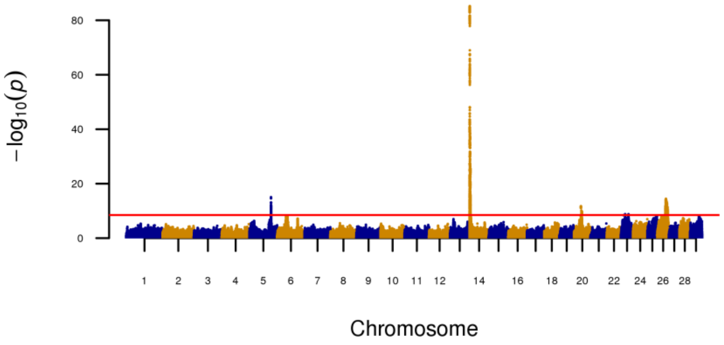

Figure S2. Manhattan plots of –log$_{10}$*P*-value for each of imputed sequence SNPs from signal-marker GWAS in Holstein (HOL) milk yield

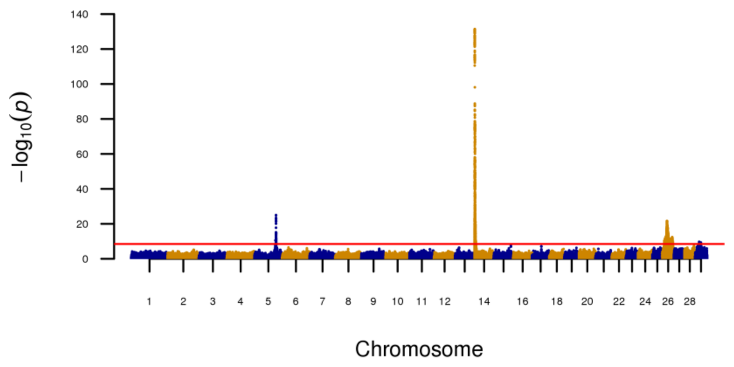

Figure S3. Manhattan plots of –log$_{10}$*P*-value for each of imputed sequence SNPs from signal-marker GWAS in Holstein (HOL) fat yield

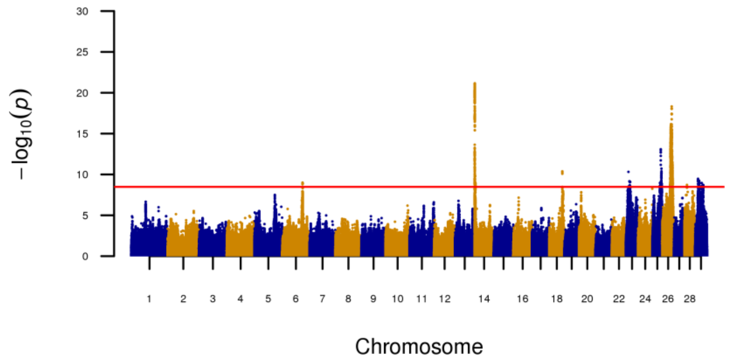

Figure S4. Manhattan plots of –log$_{10}$*P*-value for each of imputed sequence SNPs from signal-marker GWAS in Holstein (HOL) protein yield

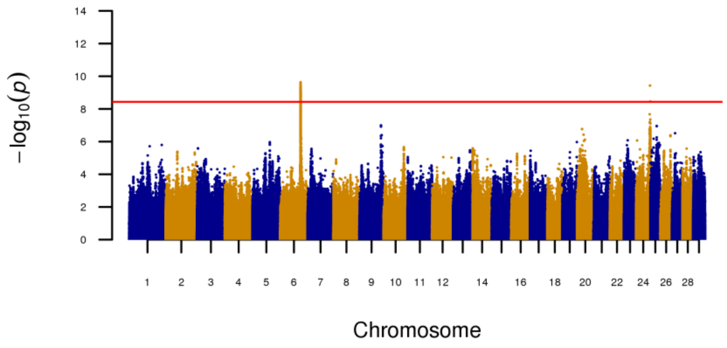

Figure S5. Manhattan plots of –log$_{10}$*P*-value for each of imputed sequence SNPs from signal-marker GWAS in Nordic red cattle (RDC) mastitis

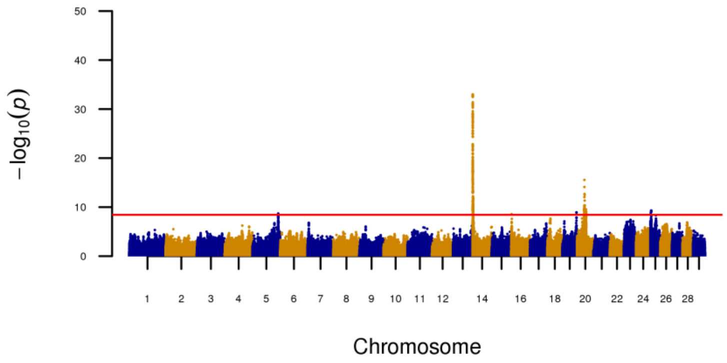

Figure S6. Manhattan plots of –log$_{10}$*P*-value for each of imputed sequence SNPs from signal-marker GWAS in Nordic red cattle (RDC) milk yield

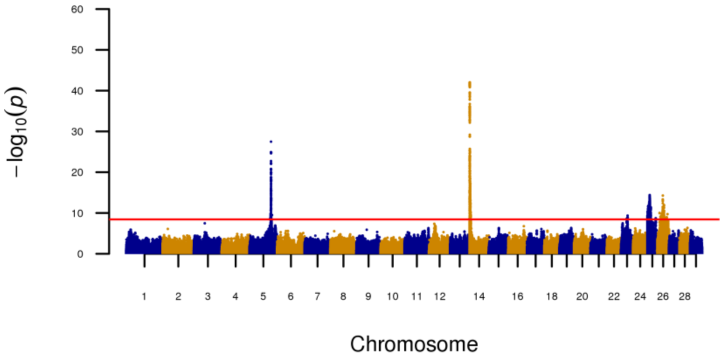

Figure S7. Manhattan plots of –log$_{10}$*P*-value for each of imputed sequence SNPs from signal-marker GWAS in Nordic red cattle (RDC) fat yield

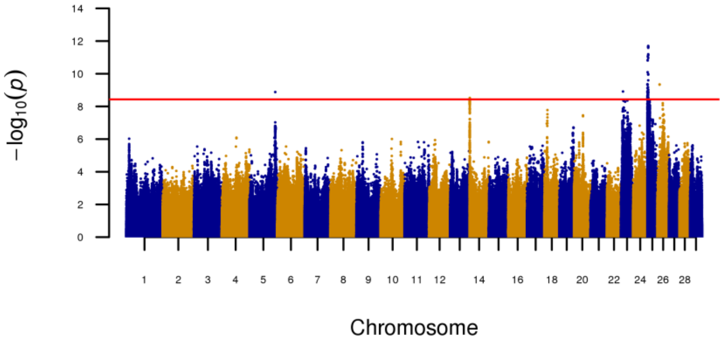

Figure S8. Manhattan plots of –log$_{10}$*P*-value for each of imputed sequence SNPs from signal-marker GWAS in Nordic red cattle (RDC) protein yield

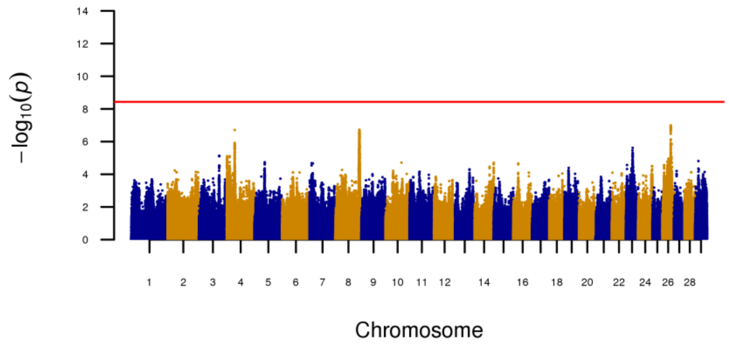

Figure S9. Manhattan plots of –log$_{10}$*P*-value for each of imputed sequence SNPs from signal-marker GWAS in Jersey (JER) mastitis

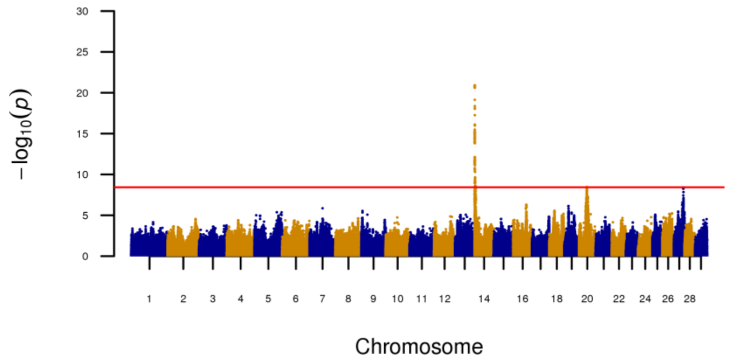

Figure S10. Manhattan plots of –log$_{10}$*P*-value for each of imputed sequence SNPs from signal-marker GWAS in Jersey (JER) milk yield

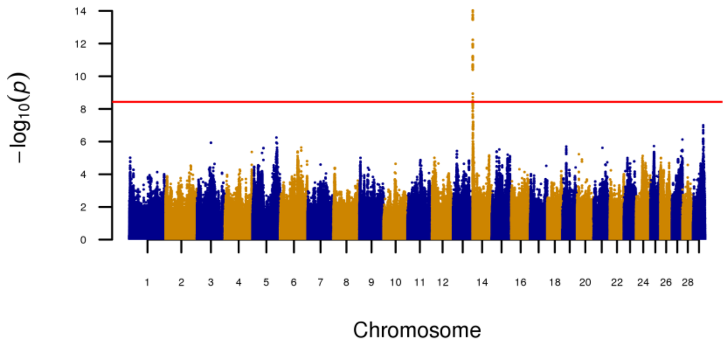

Figure S11. Manhattan plots of –log$_{10}$*P*-value for each of imputed sequence SNPs from signal-marker GWAS in Jersey (JER) fat yield

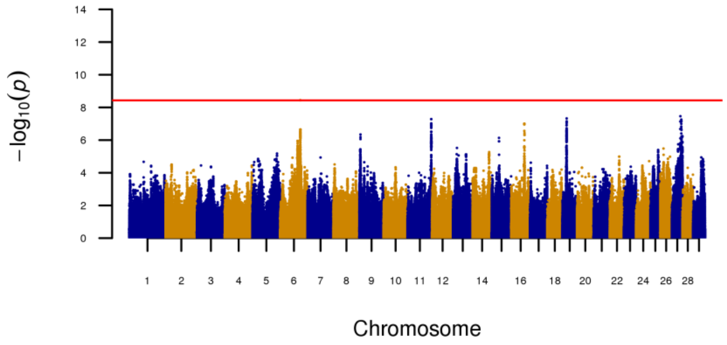

Figure S12. Manhattan plots of –log$_{10}$*P*-value for each of imputed sequence SNPs from signal-marker GWAS in Jersey (JER) protein yield

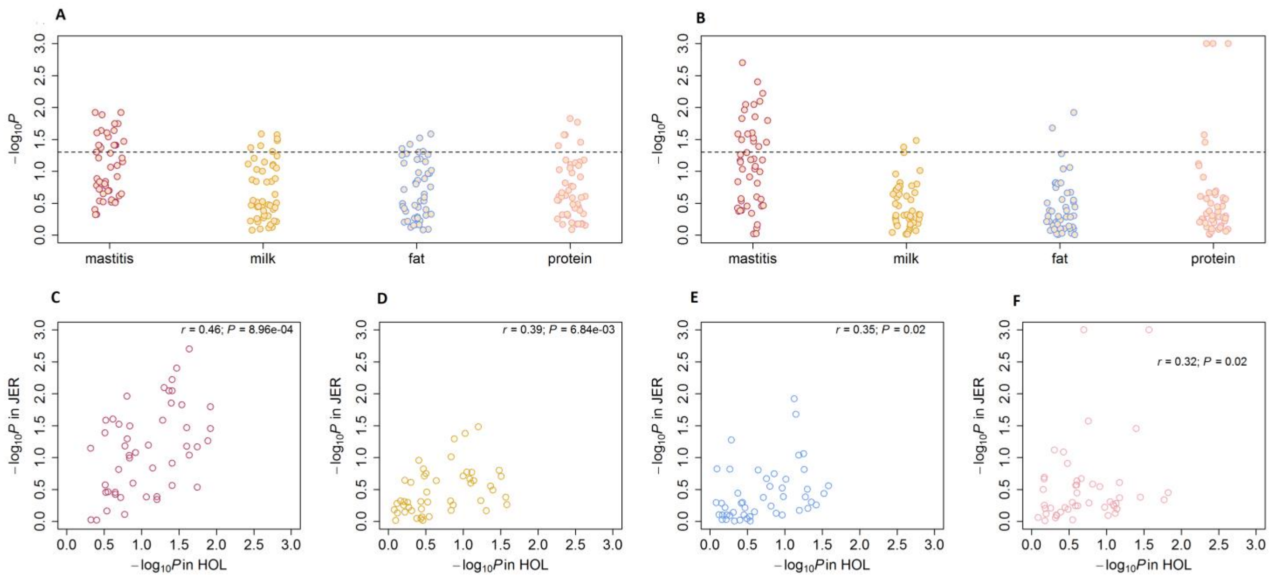

Figure S13. Post-GWAS results for the 48 genomic features identified from RNA-Seq based on six FDR-cutoffs in Holstein (HOL) and Jersey (JER). $-\log_{10}P$ is the $-\log_{10}$-transformed P-values from post-GWAS analysis. r is the Pearson correlation of $-\log_{10}P$ of genomic features between HOL and JER. P is the significance for Pearson correlation test. Each point is each of the 48 genomic features. A: post-GWAS results in HOL, B: post-GWAS results in JER, C: Pearson-correlation for mastitis resistance between HOL and JER, D: Pearson-correlation for milk yield, E: Pearson-correlation for fat yield, F: Pearson-correlation for protein yield.

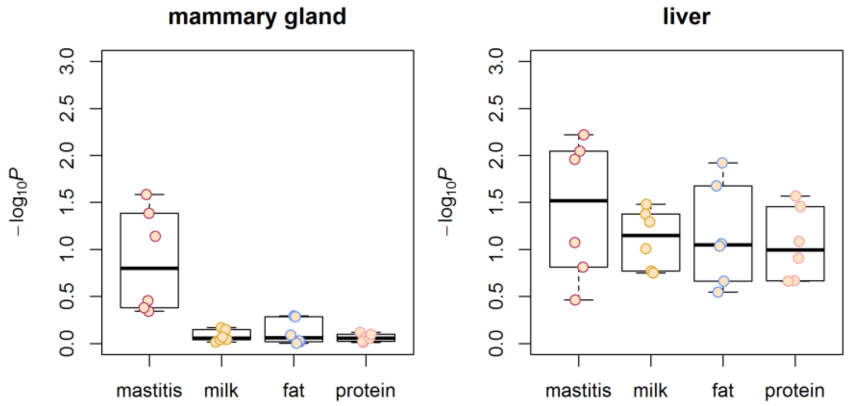

Figure S14. Tissue difference in enrichment of association signals for mastitis and milk production traits in Jersey (JER) cattle. The genomic features identified from mammary gland and liver at 24 h post-IMI with *E. coli* were used for analysis.

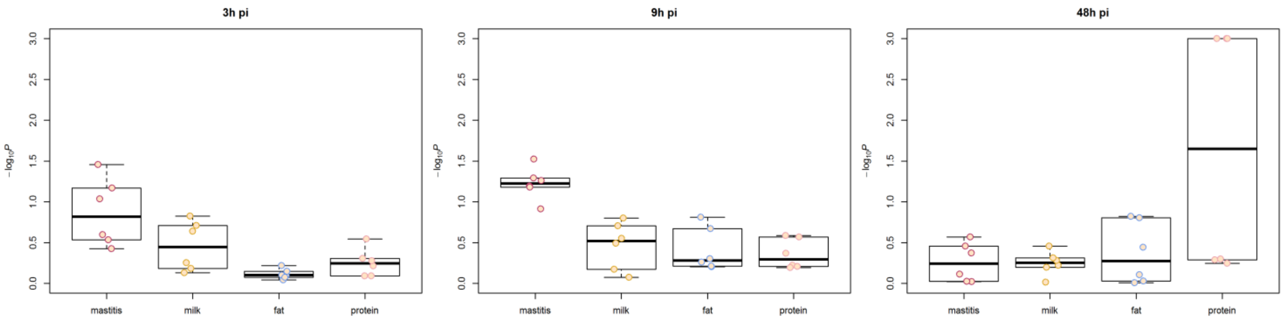

Figure S15. Dynamic impact of hepatic transcriptome during IMI with LPS in Jersey (JER) cattle. The genomic features identified from liver at 3, 9, and 48 h post-IMI with LPS were used for analysis.

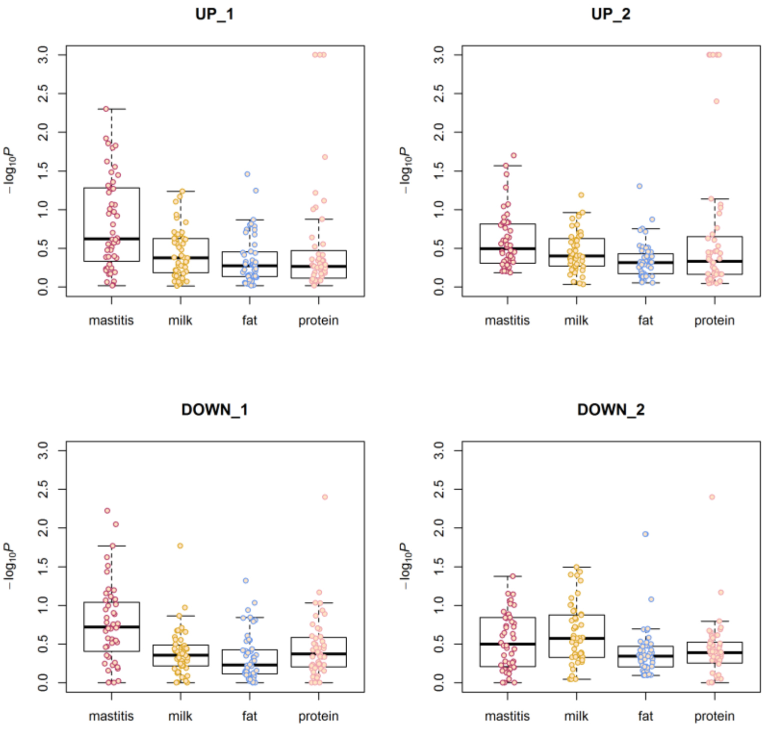

Fiugre S16. Difference of up- and down-regulated genes in enrichment of association signals for mastitis and milk production traits in Jersey (JER) cattle. HOL_UP1 and HOL_UP2 means the genomic features identified based on log2 (fold-change) > 1 and 2 in JER respectively. HOL_DOWN1 and HOL_DOWN2 means the genomic features identified based on log2 (fold-change) < -1 and -2 in JER respectively.

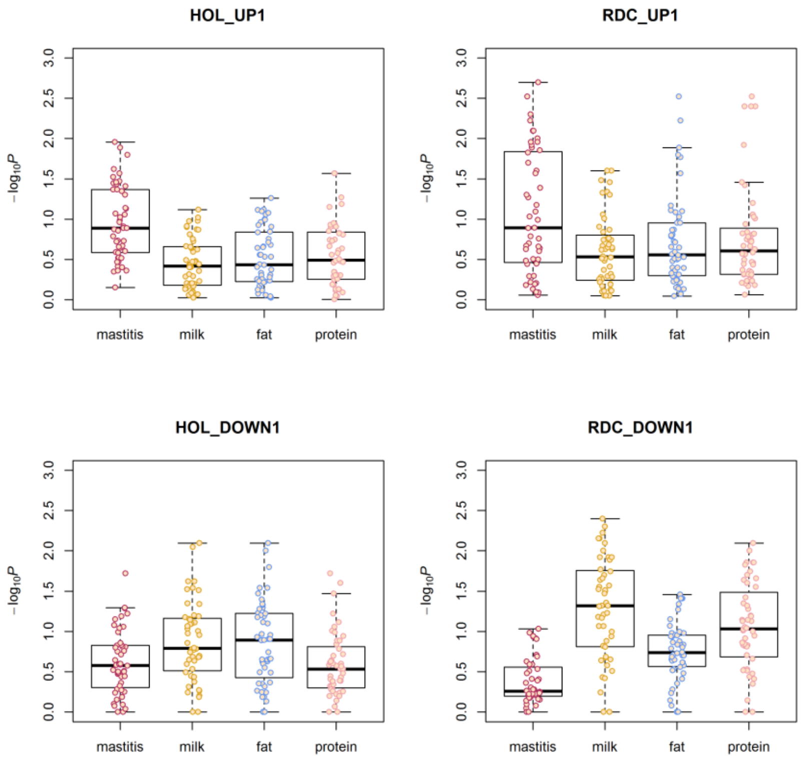

Fiugre S17. Difference of up- and down-regulated genes in enrichment of association signals for mastitis and milk production traits in Holstein (HOL) and Nordic red cattle (RDC). HOL_UP1 and RDC_UP1 means the genomic features identified based on log2 (fold-change) > 1 in HOL and RDC respectively. HOL_DOWN1 and RDC_ DOWN1 means the genomic features identified based on log2 (fold-change) < -1 in HOL and RDC respectively.

Table S1. Results of differential gene expression analysis in liver post-IMI with LPS

ENSID	log_2(FC3vs.-22)	FDR_3vs.-22	log_2(FC6vs.-22)	FDR_6vs.-22	log_2(FC9vs.-22)	FDR_9vs.-22	log_2(FC12vs.-22)	FDR_12vs.-22	log_2(FC48vs.-22)	FDR_48vs.-22
ENSBTAG00000046619	-1.91E+00	8.23E-01	-1.91E+00	4.69E-01	-1.91E+00	4.28E-01	-1.91E+00	4.39E-01	-1.91E+00	8.00E-01
ENSBTAG00000006858	0.00E+00	1.00E+00	0.00E+00	1.00E+00	-5.13E-15	1.00E+00	0.00E+00	1.00E+00	0.00E+00	1.00E+00
ENSBTAG00000039257	0.00E+00	1.00E+00	0.00E+00	1.00E+00	-5.13E-15	1.00E+00	0.00E+00	1.00E+00	0.00E+00	1.00E+00
ENSBTAG00000035349	0.00E+00	1.00E+00	0.00E+00	1.00E+00	-5.13E-15	1.00E+00	0.00E+00	1.00E+00	0.00E+00	1.00E+00
ENSBTAG00000001753	-2.95E-01	1.00E+00	-2.05E-01	1.00E+00	-8.29E-01	6.75E-01	3.79E-02	1.00E+00	-3.03E-01	1.00E+00
ENSBTAG00000005540	-7.45E-02	1.00E+00	-7.53E-01	3.88E-01	-1.53E+00	3.52E-02	-6.72E-01	4.39E-01	-2.25E-01	1.00E+00
ENSBTAG00000020035	4.64E+00	4.57E-27	6.00E+00	5.41E-41	4.95E+00	3.32E-31	2.64E+00	2.34E-11	-8.17E-01	3.11E-01
ENSBTAG00000001150	-1.18E+00	8.64E-01	-5.88E-01	8.82E-01	7.82E-01	5.60E-01	8.75E-01	4.95E-01	2.49E-01	1.00E+00
ENSBTAG00000026259	-7.93E-01	1.00E+00	-7.23E-01	9.45E-01	-9.24E-01	8.08E-01	-9.45E-01	8.05E-01	-2.71E+00	6.01E-01
ENSBTAG00000013841	-1.91E+00	8.23E-01	-1.91E+00	4.69E-01	-1.26E-01	1.00E+00	-1.91E+00	4.39E-01	7.99E-01	1.00E+00
ENSBTAG00000011528	-2.53E-01	9.58E-01	-9.55E-01	1.70E-03	-1.21E+00	4.17E-05	-1.23E+00	4.13E-05	-1.48E-01	1.00E+00
ENSBTAG00000026260	-7.95E-03	1.00E+00	-2.71E+00	2.42E-01	-9.35E-01	8.05E-01	-1.79E-01	1.00E+00	-7.98E-01	1.00E+00
ENSBTAG00000045984	0.00E+00	1.00E+00	0.00E+00	1.00E+00	-5.13E-15	1.00E+00	0.00E+00	1.00E+00	0.00E+00	1.00E+00
ENSBTAG00000012594	6.59E-01	1.93E-01	2.64E+00	5.62E-19	1.40E+00	2.06E-06	1.44E+00	1.43E-06	-9.50E-02	1.00E+00
ENSBTAG00000046118	0.00E+00	1.00E+00	0.00E+00	1.00E+00	-5.13E-15	1.00E+00	0.00E+00	1.00E+00	0.00E+00	1.00E+00
ENSBTAG00000047487	0.00E+00	1.00E+00	0.00E+00	1.00E+00	-5.13E-15	1.00E+00	0.00E+00	1.00E+00	0.00E+00	1.00E+00
ENSBTAG00000018278	-1.11E-01	1.00E+00	-4.16E-01	1.72E-01	-4.61E-01	1.16E-01	-3.65E-01	2.64E-01	-3.73E-01	6.10E-01
ENSBTAG00000021997	9.18E-02	1.00E+00	6.96E-01	2.36E-02	3.89E-01	2.87E-01	-1.26E-01	9.69E-01	-7.75E-02	1.00E+00
ENSBTAG00000008490	-4.56E-02	1.00E+00	-7.07E-01	6.96E-04	-8.39E-01	3.68E-05	-8.59E-01	2.99E-05	-2.60E-01	7.48E-01
ENSBTAG00000035175	-3.19E-01	5.76E-01	-4.22E-01	8.00E-02	-4.30E-01	7.21E-02	-4.30E-01	7.92E-02	-9.70E-02	1.00E+00
ENSBTAG00000008482	1.55E-01	9.40E-01	9.45E-01	2.63E-08	9.29E-01	3.96E-08	6.10E-01	6.30E-04	-5.49E-02	1.00E+00
ENSBTAG00000009188	5.71E-02	1.00E+00	6.37E-01	4.28E-03	9.76E-01	4.02E-06	1.06E+00	6.92E-07	-1.09E-01	1.00E+00
ENSBTAG00000009187	-1.39E-01	1.00E+00	-1.07E+00	1.09E-05	-1.02E+00	1.94E-05	-4.03E-01	1.59E-01	1.20E-01	1.00E+00
ENSBTAG00000001258	2.08E-01	1.00E+00	1.07E+00	1.08E-04	5.90E-01	5.17E-02	4.90E-01	1.35E-01	-5.15E-01	4.31E-01
ENSBTAG00000043399	5.60E-01	1.00E+00	-1.60E+00	4.10E-01	-1.80E+00	2.98E-01	-1.81E+00	3.03E-01	6.58E-03	1.00E+00
ENSBTAG00000012899	1.51E-01	1.00E+00	-3.61E-01	1.36E-01	-9.70E-01	1.99E-06	-1.27E+00	4.56E-10	5.91E-02	1.00E+00
ENSBTAG00000021819	1.76E+00	8.53E-07	2.99E+00	5.58E-19	3.18E+00	4.03E-21	3.24E+00	1.23E-21	1.42E+00	6.49E-04
ENSBTAG00000019404	9.76E-01	4.87E-04	1.46E+00	9.85E-10	1.35E+00	1.25E-08	8.24E-01	1.07E-03	3.00E-01	7.77E-01
ENSBTAG00000015212	6.74E-01	4.77E-02	1.07E+00	9.47E-06	8.98E-01	2.41E-04	6.82E-02	1.00E+00	3.65E-01	6.01E-01
ENSBTAG00000047288	0.00E+00	1.00E+00	0.00E+00	1.00E+00	-5.13E-15	1.00E+00	0.00E+00	1.00E+00	1.91E+00	8.00E-01
ENSBTAG00000037547	-9.97E-01	6.45E-01	-9.24E-01	3.26E-01	-1.36E+00	1.01E-01	-9.20E-01	3.09E-01	3.11E-02	1.00E+00
ENSBTAG00000043993	4.13E-01	9.74E-01	1.22E+00	1.63E-02	2.06E-01	9.80E-01	5.39E-01	4.39E-01	-1.01E-01	1.00E+00

ENSBTAG00000003064	-1.83E-02	1.00E+00	-1.93E-01	6.17E-01	2.90E-01	3.38E-01	-4.15E-01	1.26E-01	-2.09E-02	1.00E+00
ENSBTAG00000003063	3.17E-01	7.31E-01	4.07E-01	1.64E-01	2.30E-01	5.34E-01	4.43E-01	1.25E-01	3.94E-01	5.57E-01
ENSBTAG00000003059	-1.82E-01	9.13E-01	-4.60E-01	2.93E-02	-2.55E-01	3.13E-01	-5.99E-01	3.34E-03	1.74E-01	9.02E-01
ENSBTAG00000017310	1.64E+00	4.17E-03	1.92E+00	4.65E-05	9.57E-01	6.59E-02	7.00E-01	2.33E-01	-6.49E-01	6.86E-01
ENSBTAG00000012412	-5.63E-02	1.00E+00	1.50E+00	6.72E-09	2.02E+00	3.22E-15	1.30E+00	7.88E-07	-1.80E-02	1.00E+00
ENSBTAG00000023718	3.54E+00	6.80E-11	4.62E+00	2.04E-18	3.20E+00	3.79E-10	3.13E+00	1.31E-09	3.10E-01	1.00E+00
ENSBTAG00000016619	2.71E-01	1.00E+00	-5.22E-02	1.00E+00	-8.37E-02	1.00E+00	4.44E-01	5.35E-01	3.22E-01	1.00E+00
ENSBTAG00000020762	5.62E-01	8.98E-01	1.16E+00	6.85E-02	1.87E-01	1.00E+00	6.84E-01	4.00E-01	4.24E-01	1.00E+00
ENSBTAG00000018855	-1.45E-01	1.00E+00	8.02E-01	1.03E-04	8.22E-01	6.13E-05	3.70E-02	1.00E+00	-3.41E-01	5.42E-01
ENSBTAG00000018854	-1.22E-01	1.00E+00	-5.81E-01	9.54E-03	-1.38E+00	2.69E-11	-1.63E+00	4.40E-15	-4.43E-01	3.13E-01
ENSBTAG00000017839	2.14E-01	1.00E+00	8.68E-02	1.00E+00	-6.38E-02	1.00E+00	1.05E-01	1.00E+00	-3.14E-01	1.00E+00
ENSBTAG00000044299	0.00E+00	1.00E+00	0.00E+00	1.00E+00	-5.13E-15	1.00E+00	0.00E+00	1.00E+00	0.00E+00	1.00E+00
ENSBTAG00000046072	0.00E+00	1.00E+00	0.00E+00	1.00E+00	-5.13E-15	1.00E+00	0.00E+00	1.00E+00	0.00E+00	1.00E+00
ENSBTAG00000004530	0.00E+00	1.00E+00	0.00E+00	1.00E+00	-5.13E-15	1.00E+00	0.00E+00	1.00E+00	0.00E+00	1.00E+00
ENSBTAG00000034869	0.00E+00	1.00E+00	0.00E+00	1.00E+00	-5.13E-15	1.00E+00	0.00E+00	1.00E+00	0.00E+00	1.00E+00
ENSBTAG00000046804	0.00E+00	1.00E+00	0.00E+00	1.00E+00	-5.13E-15	1.00E+00	0.00E+00	1.00E+00	0.00E+00	1.00E+00
ENSBTAG00000015812	0.00E+00	1.00E+00	0.00E+00	1.00E+00	-5.13E-15	1.00E+00	0.00E+00	1.00E+00	0.00E+00	1.00E+00
ENSBTAG00000044154	0.00E+00	1.00E+00	0.00E+00	1.00E+00	-5.13E-15	1.00E+00	0.00E+00	1.00E+00	0.00E+00	1.00E+00
ENSBTAG00000045424	0.00E+00	1.00E+00	0.00E+00	1.00E+00	-5.13E-15	1.00E+00	0.00E+00	1.00E+00	0.00E+00	1.00E+00
ENSBTAG00000039602	0.00E+00	1.00E+00	0.00E+00	1.00E+00	-5.13E-15	1.00E+00	0.00E+00	1.00E+00	0.00E+00	1.00E+00
ENSBTAG00000037933	2.70E+00	5.76E-01	0.00E+00	1.00E+00	-5.13E-15	1.00E+00	0.00E+00	1.00E+00	0.00E+00	1.00E+00
ENSBTAG00000039597	0.00E+00	1.00E+00	0.00E+00	1.00E+00	-5.13E-15	1.00E+00	0.00E+00	1.00E+00	0.00E+00	1.00E+00
ENSBTAG00000046384	0.00E+00	1.00E+00	0.00E+00	1.00E+00	-5.13E-15	1.00E+00	0.00E+00	1.00E+00	0.00E+00	1.00E+00
ENSBTAG00000039820	-1.91E+00	8.23E-01	-1.91E+00	4.69E-01	-1.91E+00	4.28E-01	-1.91E+00	4.39E-01	2.93E+00	1.38E-01
ENSBTAG00000039255	0.00E+00	1.00E+00	0.00E+00	1.00E+00	-5.13E-15	1.00E+00	0.00E+00	1.00E+00	0.00E+00	1.00E+00
ENSBTAG00000030124	-7.85E-01	1.00E+00	5.94E-01	9.47E-01	7.29E-01	8.22E-01	6.94E-01	8.57E-01	-7.92E-01	1.00E+00
ENSBTAG00000034854	-1.23E+00	6.82E-01	4.70E-01	7.85E-01	-3.11E-01	9.96E-01	6.26E-01	5.67E-01	-4.74E-01	1.00E+00
ENSBTAG00000045873	0.00E+00	1.00E+00	0.00E+00	1.00E+00	-5.13E-15	1.00E+00	0.00E+00	1.00E+00	0.00E+00	1.00E+00
ENSBTAG00000042595	2.70E+00	5.76E-01	0.00E+00	1.00E+00	2.55E+00	2.55E-01	0.00E+00	1.00E+00	0.00E+00	1.00E+00
ENSBTAG00000004139	5.38E-01	7.33E-01	1.50E+00	4.21E-04	1.14E+00	9.10E-03	1.06E+00	1.91E-02	2.97E-01	1.00E+00
ENSBTAG00000007444	2.25E+00	4.38E-01	3.85E+00	4.90E-04	1.50E+00	4.48E-01	3.16E+00	1.01E-02	2.29E+00	4.76E-01
ENSBTAG00000014233	1.24E+00	3.92E-09	2.44E+00	1.16E-34	1.95E+00	3.85E-23	1.04E+00	2.34E-07	-2.38E-01	8.00E-01
ENSBTAG00000020122	5.70E-02	1.00E+00	7.70E-01	5.99E-06	9.01E-01	7.07E-08	6.65E-01	1.35E-04	-1.55E-01	9.07E-01
ENSBTAG00000020121	6.96E-03	1.00E+00	-1.21E-01	9.26E-01	-5.25E-02	1.00E+00	-2.57E-01	4.88E-01	1.00E-01	1.00E+00
ENSBTAG00000000201	4.49E-01	5.65E-01	9.81E-01	8.38E-04	1.15E+00	6.45E-05	9.74E-01	1.08E-03	1.66E-01	1.00E+00

ENSBTAG00000038433	1.34E-02	1.00E+00	2.41E+00	1.83E-04	1.14E-02	1.00E+00	-1.79E-01	1.00E+00	-1.12E+00	8.00E-01
ENSBTAG00000001412	-3.18E-01	9.32E-01	-4.55E-01	3.33E-01	2.58E-01	6.77E-01	1.21E-02	1.00E+00	1.52E-01	1.00E+00
ENSBTAG00000045479	0.00E+00	1.00E+00	0.00E+00	1.00E+00	-5.13E-15	1.00E+00	0.00E+00	1.00E+00	0.00E+00	1.00E+00
ENSBTAG00000040155	1.91E+00	8.23E-01	2.79E+00	2.10E-01	2.55E+00	2.54E-01	0.00E+00	1.00E+00	0.00E+00	1.00E+00
ENSBTAG00000042331	0.00E+00	1.00E+00	0.00E+00	1.00E+00	-5.13E-15	1.00E+00	0.00E+00	1.00E+00	0.00E+00	1.00E+00
ENSBTAG00000000648	1.83E+00	8.87E-03	2.34E+00	3.05E-05	8.15E-01	2.35E-01	1.51E+00	1.09E-02	3.23E-01	1.00E+00
ENSBTAG00000000706	5.92E-01	6.22E-01	-1.08E+00	1.66E-02	3.09E-01	6.91E-01	1.02E+00	2.52E-02	8.66E-02	1.00E+00
ENSBTAG00000045024	0.00E+00	1.00E+00	0.00E+00	1.00E+00	-5.13E-15	1.00E+00	0.00E+00	1.00E+00	0.00E+00	1.00E+00
ENSBTAG00000005742	-1.34E+00	1.40E-01	-3.47E-01	7.80E-01	-1.54E+00	9.90E-03	-1.43E+00	1.93E-02	-6.50E-01	7.85E-01
ENSBTAG00000043488	0.00E+00	1.00E+00	0.00E+00	1.00E+00	-5.13E-15	1.00E+00	0.00E+00	1.00E+00	0.00E+00	1.00E+00
ENSBTAG00000017753	-1.62E-01	1.00E+00	1.52E-01	8.55E-01	-1.61E-01	8.15E-01	-1.33E-01	9.21E-01	-3.67E-01	6.78E-01
ENSBTAG00000042557	0.00E+00	1.00E+00	0.00E+00	1.00E+00	-5.13E-15	1.00E+00	0.00E+00	1.00E+00	0.00E+00	1.00E+00
ENSBTAG00000019043	1.73E-01	9.69E-01	5.51E-01	9.92E-03	5.22E-01	1.52E-02	4.43E-01	5.20E-02	4.70E-01	2.14E-01
ENSBTAG00000000605	-7.42E-02	1.00E+00	-2.66E-01	4.56E-01	-3.59E-01	2.52E-01	-1.86E-01	7.02E-01	-3.42E-01	6.78E-01
ENSBTAG00000000603	1.39E-01	1.00E+00	1.15E+00	4.12E-04	5.19E-01	1.81E-01	1.76E-01	8.77E-01	-6.92E-01	3.00E-01
ENSBTAG00000019542	-7.59E-02	1.00E+00	-4.55E-01	5.52E-02	-1.59E-01	6.88E-01	1.10E-02	1.00E+00	-3.06E-01	6.32E-01
ENSBTAG00000036404	-1.91E+00	8.23E-01	8.76E-01	8.53E-01	-1.91E+00	4.28E-01	-1.91E+00	4.39E-01	-1.91E+00	8.00E-01
ENSBTAG00000042261	0.00E+00	1.00E+00	0.00E+00	1.00E+00	-5.13E-15	1.00E+00	0.00E+00	1.00E+00	0.00E+00	1.00E+00
ENSBTAG00000043064	0.00E+00	1.00E+00	0.00E+00	1.00E+00	-5.13E-15	1.00E+00	0.00E+00	1.00E+00	0.00E+00	1.00E+00
ENSBTAG00000038900	3.12E-01	1.00E+00	2.76E-01	7.94E-01	6.22E-01	2.34E-01	1.10E+00	1.32E-02	-3.41E-01	9.94E-01
ENSBTAG00000048165	0.00E+00	1.00E+00	0.00E+00	1.00E+00	-5.13E-15	1.00E+00	0.00E+00	1.00E+00	0.00E+00	1.00E+00
ENSBTAG00000045128	0.00E+00	1.00E+00	0.00E+00	1.00E+00	-5.13E-15	1.00E+00	0.00E+00	1.00E+00	0.00E+00	1.00E+00
ENSBTAG00000003301	-3.88E-01	1.00E+00	-5.71E-01	7.41E-01	-1.14E+00	2.89E-01	6.68E-01	5.58E-01	-4.19E-01	1.00E+00
ENSBTAG00000036841	0.00E+00	1.00E+00	0.00E+00	1.00E+00	-5.13E-15	1.00E+00	0.00E+00	1.00E+00	0.00E+00	1.00E+00
ENSBTAG00000039704	0.00E+00	1.00E+00	0.00E+00	1.00E+00	-5.13E-15	1.00E+00	0.00E+00	1.00E+00	0.00E+00	1.00E+00
ENSBTAG00000047337	0.00E+00	1.00E+00	0.00E+00	1.00E+00	-5.13E-15	1.00E+00	0.00E+00	1.00E+00	0.00E+00	1.00E+00
ENSBTAG00000044598	0.00E+00	1.00E+00	0.00E+00	1.00E+00	-5.13E-15	1.00E+00	0.00E+00	1.00E+00	0.00E+00	1.00E+00
ENSBTAG00000043929	0.00E+00	1.00E+00	0.00E+00	1.00E+00	-5.13E-15	1.00E+00	0.00E+00	1.00E+00	0.00E+00	1.00E+00
ENSBTAG00000000597	-1.28E+00	7.67E-01	-8.39E-01	6.07E-01	-3.36E-01	1.00E+00	4.70E-01	8.40E-01	-1.81E+00	5.43E-01
ENSBTAG00000000588	-5.26E-01	1.00E+00	-4.13E+00	1.96E-02	2.09E-01	1.00E+00	-1.11E+00	4.73E-01	8.39E-01	8.24E-01
ENSBTAG00000001395	8.84E-01	2.09E-01	1.58E+00	1.12E-04	6.25E-01	2.11E-01	7.15E-01	1.44E-01	9.40E-01	2.21E-01
ENSBTAG00000045008	0.00E+00	1.00E+00	0.00E+00	1.00E+00	-5.13E-15	1.00E+00	0.00E+00	1.00E+00	0.00E+00	1.00E+00
ENSBTAG00000047943	3.61E+00	2.28E-01	3.98E+00	2.57E-02	1.78E+00	4.79E-01	0.00E+00	1.00E+00	0.00E+00	1.00E+00
ENSBTAG00000018704	3.01E+00	7.84E-11	4.61E+00	2.43E-23	2.07E+00	1.80E-06	1.23E+00	8.07E-03	-4.43E-01	8.33E-01
ENSBTAG00000004869	-3.67E-01	7.82E-01	-8.46E-01	8.36E-03	-1.79E+00	3.96E-09	-8.51E-01	8.83E-03	8.57E-02	1.00E+00

ENSBTAG00000043726	0.00E+00	1.00E+00	0.00E+00	1.00E+00	-5.13E-15	1.00E+00	0.00E+00	1.00E+00	0.00E+00	1.00E+00
ENSBTAG00000040418	5.45E-01	9.28E-01	-7.44E-02	1.00E+00	-4.33E-01	7.51E-01	3.03E-01	9.11E-01	-8.07E-01	8.00E-01
ENSBTAG00000029827	1.83E+00	4.45E-01	9.76E-01	6.52E-01	3.50E-01	1.00E+00	-2.71E+00	2.05E-01	1.48E-03	1.00E+00
ENSBTAG00000029912	0.00E+00	1.00E+00	0.00E+00	1.00E+00	-5.13E-15	1.00E+00	0.00E+00	1.00E+00	0.00E+00	1.00E+00
ENSBTAG00000029768	0.00E+00	1.00E+00	0.00E+00	1.00E+00	-5.13E-15	1.00E+00	0.00E+00	1.00E+00	0.00E+00	1.00E+00
ENSBTAG00000047183	-1.91E+00	8.23E-01	-1.91E+00	4.69E-01	-1.91E+00	4.28E-01	-1.91E+00	4.39E-01	-1.91E+00	8.00E-01
ENSBTAG00000044220	0.00E+00	1.00E+00	0.00E+00	1.00E+00	-5.13E-15	1.00E+00	0.00E+00	1.00E+00	0.00E+00	1.00E+00
ENSBTAG00000019314	1.51E-01	1.00E+00	3.44E-01	2.25E-01	6.17E-01	1.01E-02	4.06E-01	1.37E-01	-1.72E-01	1.00E+00
ENSBTAG00000047293	8.89E-01	3.20E-01	2.81E+00	1.76E-09	1.98E+00	2.04E-05	1.81E+00	1.32E-04	1.07E-01	1.00E+00
ENSBTAG00000002623	1.44E+00	3.68E-04	1.06E+00	3.88E-03	4.23E-01	3.87E-01	1.28E+00	4.19E-04	-4.85E-01	7.08E-01
ENSBTAG00000012908	1.05E+00	1.61E-02	3.01E+00	8.33E-19	2.62E+00	5.46E-15	2.51E+00	8.76E-14	4.93E-01	6.13E-01
ENSBTAG00000034347	-5.18E-01	8.35E-01	-1.09E+00	7.04E-02	6.75E-01	2.13E-01	1.04E+00	2.75E-02	9.58E-02	1.00E+00
ENSBTAG00000038512	-2.95E-01	1.00E+00	-5.78E-01	8.91E-01	-8.33E-01	6.72E-01	2.53E-01	1.00E+00	-6.45E-01	1.00E+00
ENSBTAG00000038913	-4.66E-02	1.00E+00	-3.40E-01	3.84E-01	-2.08E-01	7.02E-01	-2.10E-01	7.07E-01	-3.68E-01	7.33E-01
ENSBTAG00000046369	0.00E+00	1.00E+00	0.00E+00	1.00E+00	-5.13E-15	1.00E+00	0.00E+00	1.00E+00	0.00E+00	1.00E+00
ENSBTAG00000047674	0.00E+00	1.00E+00	0.00E+00	1.00E+00	-5.13E-15	1.00E+00	1.76E+00	4.97E-01	0.00E+00	1.00E+00
ENSBTAG00000048144	0.00E+00	1.00E+00	1.98E+00	4.43E-01	-5.13E-15	1.00E+00	0.00E+00	1.00E+00	0.00E+00	1.00E+00
ENSBTAG00000046755	4.71E-01	1.00E+00	1.45E+00	4.82E-01	2.63E-01	1.00E+00	2.19E+00	1.89E-01	2.83E+00	3.11E-01
ENSBTAG00000010462	1.43E-01	1.00E+00	-5.06E-01	1.00E+00	-2.43E+00	1.71E-01	-2.35E+00	1.99E-01	1.05E+00	1.00E+00
ENSBTAG00000028813	0.00E+00	1.00E+00	0.00E+00	1.00E+00	-5.13E-15	1.00E+00	0.00E+00	1.00E+00	0.00E+00	1.00E+00
ENSBTAG00000043751	0.00E+00	1.00E+00	0.00E+00	1.00E+00	-5.13E-15	1.00E+00	0.00E+00	1.00E+00	0.00E+00	1.00E+00
ENSBTAG00000009851	5.56E-01	7.40E-01	1.82E+00	3.27E-05	1.18E+00	9.62E-03	-3.20E-02	1.00E+00	3.71E-01	9.27E-01
ENSBTAG00000037354	0.00E+00	1.00E+00	0.00E+00	1.00E+00	-5.13E-15	1.00E+00	0.00E+00	1.00E+00	0.00E+00	1.00E+00
ENSBTAG00000005101	0.00E+00	1.00E+00	0.00E+00	1.00E+00	-5.13E-15	1.00E+00	0.00E+00	1.00E+00	0.00E+00	1.00E+00
ENSBTAG00000008359	6.90E-01	6.15E-01	8.49E-02	1.00E+00	8.53E-02	1.00E+00	-2.47E-01	9.26E-01	1.45E-01	1.00E+00
ENSBTAG00000036262	-1.71E-01	1.00E+00	-4.64E-01	1.18E-01	-1.26E+00	6.56E-07	-1.53E+00	1.61E-09	1.91E-04	1.00E+00
ENSBTAG00000046383	-3.36E-01	1.00E+00	-3.24E-02	1.00E+00	7.58E-01	2.05E-01	4.97E-01	4.97E-01	-1.45E-01	1.00E+00
ENSBTAG00000045043	0.00E+00	1.00E+00	0.00E+00	1.00E+00	-5.13E-15	1.00E+00	0.00E+00	1.00E+00	0.00E+00	1.00E+00
ENSBTAG00000043669	0.00E+00	1.00E+00	0.00E+00	1.00E+00	-5.13E-15	1.00E+00	0.00E+00	1.00E+00	0.00E+00	1.00E+00
ENSBTAG00000038026	0.00E+00	1.00E+00	0.00E+00	1.00E+00	-5.13E-15	1.00E+00	0.00E+00	1.00E+00	0.00E+00	1.00E+00
ENSBTAG00000045974	2.81E-01	1.00E+00	5.61E-01	5.90E-01	1.41E+00	2.32E-02	6.76E-01	4.52E-01	4.96E-01	9.90E-01
ENSBTAG00000009219	-5.50E-01	1.00E+00	-4.03E-02	1.00E+00	-6.06E-01	6.75E-01	5.30E-01	7.38E-01	-7.99E-01	8.45E-01
ENSBTAG00000034284	-1.12E+00	6.29E-02	-1.19E+00	7.67E-03	-3.41E-01	5.75E-01	-4.27E-01	4.44E-01	-5.30E-01	7.07E-01
ENSBTAG00000026156	-2.70E+00	5.76E-01	8.97E-02	1.00E+00	-1.53E-01	1.00E+00	1.23E+00	4.57E-01	-7.85E-01	1.00E+00
ENSBTAG00000007939	5.86E-02	1.00E+00	7.97E-02	1.00E+00	2.17E-01	4.63E-01	1.59E-01	6.75E-01	-3.92E-01	4.14E-01

ENSBTAG00000009128	2.71E+00	5.76E-01	1.98E+00	4.43E-01	-5.13E-15	1.00E+00	3.04E+00	1.49E-01	0.00E+00	1.00E+00
ENSBTAG00000039151	0.00E+00	1.00E+00	0.00E+00	1.00E+00	-5.13E-15	1.00E+00	0.00E+00	1.00E+00	0.00E+00	1.00E+00
ENSBTAG00000046393	-1.91E+00	8.23E-01	-1.91E+00	4.69E-01	-1.31E-01	1.00E+00	-1.54E-01	1.00E+00	-1.91E+00	8.00E-01
ENSBTAG00000046959	5.00E-01	1.40E-01	1.21E+00	4.95E-09	6.83E-01	1.62E-03	3.24E-01	2.24E-01	-1.82E-02	1.00E+00
ENSBTAG00000023675	1.66E-01	1.00E+00	1.02E+00	4.07E-07	5.93E-01	5.74E-03	6.79E-01	1.48E-03	-1.16E-01	1.00E+00
ENSBTAG00000007970	5.45E-01	1.29E-01	1.91E+00	1.30E-18	1.36E+00	4.89E-10	1.04E+00	3.96E-06	-2.89E-01	7.74E-01
ENSBTAG00000045590	0.00E+00	1.00E+00	0.00E+00	1.00E+00	1.78E+00	4.79E-01	0.00E+00	1.00E+00	0.00E+00	1.00E+00
ENSBTAG00000006811	1.00E+00	2.30E-01	1.54E-01	1.00E+00	-2.77E-01	8.56E-01	4.56E-01	5.40E-01	5.39E-01	8.00E-01
ENSBTAG00000023652	2.93E-01	8.06E-01	-1.29E-01	8.96E-01	-5.43E-01	4.52E-02	-5.21E-01	6.39E-02	6.03E-01	1.74E-01
ENSBTAG00000005155	1.75E+00	4.86E-09	2.27E+00	7.89E-16	1.53E+00	5.04E-08	8.59E-01	4.54E-03	1.18E-01	1.00E+00
ENSBTAG00000005162	0.00E+00	1.00E+00	0.00E+00	1.00E+00	-5.13E-15	1.00E+00	0.00E+00	1.00E+00	0.00E+00	1.00E+00
ENSBTAG00000015728	8.25E-01	3.05E-03	1.94E+00	6.04E-18	1.48E+00	3.55E-11	1.37E-01	8.37E-01	-1.54E-01	1.00E+00
ENSBTAG00000046492	0.00E+00	1.00E+00	0.00E+00	1.00E+00	-5.13E-15	1.00E+00	0.00E+00	1.00E+00	0.00E+00	1.00E+00
ENSBTAG00000045781	-5.71E+00	1.83E-04	-3.72E+00	6.06E-04	-1.99E+00	1.98E-02	-5.71E+00	9.37E-06	-3.00E+00	3.02E-02
ENSBTAG00000034182	2.10E-01	1.00E+00	2.40E+00	2.36E-12	2.59E+00	2.95E-14	2.55E+00	1.03E-13	-7.51E-01	3.49E-01
ENSBTAG00000044111	-1.38E-01	1.00E+00	-1.15E+00	2.27E-03	-2.56E+00	5.16E-12	-2.44E+00	6.00E-11	-9.16E-03	1.00E+00
ENSBTAG00000042804	0.00E+00	1.00E+00	0.00E+00	1.00E+00	-5.13E-15	1.00E+00	0.00E+00	1.00E+00	0.00E+00	1.00E+00
ENSBTAG00000043097	0.00E+00	1.00E+00	0.00E+00	1.00E+00	-5.13E-15	1.00E+00	0.00E+00	1.00E+00	0.00E+00	1.00E+00
ENSBTAG00000010091	5.26E-02	1.00E+00	1.61E-01	6.17E-01	7.09E-01	1.98E-04	6.35E-01	1.24E-03	9.70E-02	1.00E+00
ENSBTAG00000047147	9.61E-02	1.00E+00	5.82E-01	3.42E-01	2.58E-01	8.54E-01	9.63E-01	5.58E-02	-6.45E-01	7.33E-01
ENSBTAG00000008346	5.65E-01	4.34E-01	7.85E-01	2.58E-02	7.90E-01	2.34E-02	5.44E-01	1.71E-01	-1.35E-01	1.00E+00
ENSBTAG00000044771	0.00E+00	1.00E+00	0.00E+00	1.00E+00	-5.13E-15	1.00E+00	0.00E+00	1.00E+00	0.00E+00	1.00E+00
ENSBTAG00000015546	3.24E-02	1.00E+00	3.82E-01	2.12E-01	1.09E+00	9.55E-06	7.36E-01	5.20E-03	-2.11E-01	9.41E-01
ENSBTAG00000001416	-3.60E+00	2.30E-01	7.87E-02	1.00E+00	-3.60E+00	4.47E-02	-1.07E+00	5.88E-01	-1.69E+00	7.78E-01
ENSBTAG00000023668	0.00E+00	1.00E+00	0.00E+00	1.00E+00	-5.13E-15	1.00E+00	0.00E+00	1.00E+00	0.00E+00	1.00E+00
ENSBTAG00000047095	0.00E+00	1.00E+00	1.98E+00	4.43E-01	-5.13E-15	1.00E+00	0.00E+00	1.00E+00	0.00E+00	1.00E+00
ENSBTAG00000040456	0.00E+00	1.00E+00	0.00E+00	1.00E+00	-5.13E-15	1.00E+00	0.00E+00	1.00E+00	0.00E+00	1.00E+00
ENSBTAG00000022908	0.00E+00	1.00E+00	0.00E+00	1.00E+00	-5.13E-15	1.00E+00	0.00E+00	1.00E+00	0.00E+00	1.00E+00
ENSBTAG00000027113	0.00E+00	1.00E+00	0.00E+00	1.00E+00	-5.13E-15	1.00E+00	0.00E+00	1.00E+00	0.00E+00	1.00E+00
ENSBTAG00000033437	0.00E+00	1.00E+00	0.00E+00	1.00E+00	-5.13E-15	1.00E+00	0.00E+00	1.00E+00	0.00E+00	1.00E+00
ENSBTAG00000047371	0.00E+00	1.00E+00	0.00E+00	1.00E+00	-5.13E-15	1.00E+00	0.00E+00	1.00E+00	0.00E+00	1.00E+00
ENSBTAG00000044603	0.00E+00	1.00E+00	0.00E+00	1.00E+00	-5.13E-15	1.00E+00	0.00E+00	1.00E+00	0.00E+00	1.00E+00
ENSBTAG00000037820	0.00E+00	1.00E+00	0.00E+00	1.00E+00	1.78E+00	4.79E-01	0.00E+00	1.00E+00	0.00E+00	1.00E+00
ENSBTAG00000027782	0.00E+00	1.00E+00	0.00E+00	1.00E+00	-5.13E-15	1.00E+00	0.00E+00	1.00E+00	0.00E+00	1.00E+00
ENSBTAG00000020996	4.31E-01	1.19E-01	8.38E-01	1.55E-06	8.84E-01	2.99E-07	6.77E-01	1.65E-04	-1.15E-01	1.00E+00

ENSBTAG00000020997	9.82E-01	8.10E-01	3.20E-01	1.00E+00	-4.12E+00	1.25E-02	-1.58E+00	2.83E-01	4.35E-01	1.00E+00
ENSBTAG00000028582	0.00E+00	1.00E+00	0.00E+00	1.00E+00	-5.13E-15	1.00E+00	0.00E+00	1.00E+00	0.00E+00	1.00E+00
ENSBTAG00000004124	3.88E-01	7.99E-01	1.11E+00	8.93E-04	7.68E-01	2.93E-02	7.87E-01	2.80E-02	2.86E-01	9.14E-01
ENSBTAG00000044934	3.75E+00	4.07E-05	2.98E+00	1.62E-03	2.08E+00	6.55E-02	2.07E+00	7.47E-02	-7.75E-01	1.00E+00
ENSBTAG00000023666	1.87E+00	6.55E-13	1.67E+00	2.56E-11	2.14E-01	6.19E-01	-4.52E-01	1.47E-01	6.41E-01	1.53E-01
ENSBTAG00000004471	1.07E-03	1.00E+00	4.35E-01	8.86E-02	1.34E-01	8.22E-01	-5.00E-01	4.79E-02	-5.20E-01	2.20E-01
ENSBTAG00000014889	-1.51E-01	1.00E+00	7.66E-01	4.16E-03	-1.82E-01	7.31E-01	-2.39E-01	5.61E-01	9.09E-02	1.00E+00
ENSBTAG00000013662	3.88E-01	1.00E+00	-9.29E-01	1.10E-01	-1.57E+00	3.04E-03	-1.52E+00	4.80E-03	1.25E+00	1.57E-01
ENSBTAG00000013663	-2.42E-01	9.49E-01	1.93E-01	7.33E-01	5.54E-01	6.72E-02	4.78E-01	1.40E-01	-8.44E-01	4.80E-02
ENSBTAG00000013664	0.00E+00	1.00E+00	0.00E+00	1.00E+00	-5.13E-15	1.00E+00	0.00E+00	1.00E+00	0.00E+00	1.00E+00
ENSBTAG00000013667	2.20E-02	1.00E+00	9.68E-02	1.00E+00	1.58E+00	4.57E-02	8.69E-01	4.47E-01	-1.42E+00	7.63E-01
ENSBTAG00000015010	3.64E-01	4.69E-01	1.28E+00	1.71E-09	7.12E-01	1.32E-03	5.07E-01	3.54E-02	-1.02E-01	1.00E+00
ENSBTAG00000004833	-1.28E-02	1.00E+00	-1.89E-01	5.84E-01	-7.24E-01	8.86E-04	-1.11E+00	1.64E-07	-3.29E-01	6.01E-01
ENSBTAG00000014302	1.80E-01	8.87E-01	1.25E+00	2.35E-12	1.08E+00	1.49E-09	9.77E-01	7.43E-08	-4.80E-01	1.28E-01
ENSBTAG00000034442	4.24E-02	1.00E+00	3.11E-01	2.51E-01	-5.51E-01	1.80E-02	-1.57E+00	5.99E-13	5.37E-01	1.56E-01
ENSBTAG00000045333	0.00E+00	1.00E+00	0.00E+00	1.00E+00	-5.13E-15	1.00E+00	0.00E+00	1.00E+00	0.00E+00	1.00E+00
ENSBTAG00000013321	1.20E+00	6.14E-01	1.52E+00	1.02E-01	6.48E-01	7.03E-01	8.80E-01	4.94E-01	2.65E-01	1.00E+00
ENSBTAG00000013806	8.69E-02	1.00E+00	1.12E-01	9.16E-01	-5.54E-01	2.09E-02	-1.14E+00	3.93E-07	2.72E-01	7.79E-01
ENSBTAG00000046385	4.91E-01	1.66E-01	1.85E+00	5.27E-19	1.37E+00	4.20E-11	8.26E-01	1.60E-04	-1.62E-01	1.00E+00
ENSBTAG00000004061	5.44E-01	7.51E-01	1.77E+00	1.57E-05	1.05E+00	1.85E-02	2.08E-01	9.31E-01	-1.65E-01	1.00E+00
ENSBTAG00000000746	5.87E-02	1.00E+00	-7.49E-01	2.86E-03	-1.33E+00	2.79E-08	-1.44E+00	2.35E-09	5.05E-01	3.11E-01
ENSBTAG00000000752	4.19E-01	1.00E+00	-4.00E-01	7.59E-01	-6.82E-01	4.09E-01	2.42E-01	9.60E-01	5.16E-01	8.60E-01
ENSBTAG00000004538	-9.81E-02	1.00E+00	3.87E-02	1.00E+00	7.54E-01	2.25E-02	2.32E-01	7.08E-01	-3.04E-01	8.87E-01
ENSBTAG00000047076	0.00E+00	1.00E+00	0.00E+00	1.00E+00	1.78E+00	4.79E-01	0.00E+00	1.00E+00	2.72E+00	6.01E-01
ENSBTAG00000004459	-2.28E-01	1.00E+00	4.03E-01	5.59E-01	4.63E-01	4.60E-01	5.22E-01	3.95E-01	-5.60E-01	7.82E-01
ENSBTAG00000012140	2.71E-02	1.00E+00	8.55E-01	4.43E-01	-1.77E+00	1.74E-01	-1.85E-01	1.00E+00	-1.10E+00	8.00E-01
ENSBTAG00000016363	9.29E-01	2.37E-03	2.86E+00	2.38E-29	2.07E+00	1.06E-16	1.21E+00	2.15E-06	-2.61E-01	8.24E-01
ENSBTAG00000048157	9.53E-01	2.41E-01	2.63E+00	1.26E-10	2.38E+00	5.96E-09	1.47E+00	1.17E-03	4.99E-01	8.07E-01
ENSBTAG00000026836	-1.69E-01	1.00E+00	1.59E-01	8.37E-01	3.90E-01	2.39E-01	6.72E-01	1.94E-02	-5.65E-01	3.24E-01
ENSBTAG00000015662	-2.91E-01	9.36E-01	2.88E-01	5.66E-01	-3.40E-01	4.64E-01	-1.26E+00	2.44E-04	-2.28E-02	1.00E+00
ENSBTAG00000016932	8.83E-02	1.00E+00	2.96E-01	3.73E-01	1.03E-01	9.78E-01	-2.89E-01	4.07E-01	-2.10E-01	9.30E-01
ENSBTAG00000018731	-2.31E-01	1.00E+00	6.34E-01	2.53E-01	-2.80E-01	7.98E-01	-7.27E-01	1.93E-01	-2.05E-01	1.00E+00
ENSBTAG00000024027	3.52E-01	4.60E-01	1.15E+00	1.41E-08	9.36E-01	4.66E-06	3.66E-01	1.42E-01	-4.27E-01	3.44E-01
ENSBTAG00000015069	1.83E-01	8.23E-01	3.50E-01	6.77E-02	2.74E-01	1.85E-01	1.82E-01	4.73E-01	-5.12E-01	5.09E-02
ENSBTAG00000013460	3.90E-01	5.79E-01	9.34E-01	4.38E-04	7.75E-01	4.30E-03	4.34E-01	1.76E-01	1.55E-01	1.00E+00

ENSBTAG00000013461	1.50E-01	1.00E+00	5.65E-01	3.36E-02	1.68E-01	7.45E-01	9.52E-02	1.00E+00	-4.26E-01	4.79E-01
ENSBTAG00000013464	2.89E-01	8.38E-01	1.46E+00	5.14E-08	2.30E-01	6.21E-01	7.10E-01	1.67E-02	3.91E-01	6.51E-01
ENSBTAG00000013465	-5.56E-02	1.00E+00	8.58E-02	1.00E+00	1.28E-01	9.54E-01	2.89E-01	4.97E-01	-5.42E-01	4.07E-01
ENSBTAG00000010987	1.19E+00	6.35E-06	9.58E-01	8.70E-05	2.15E-01	5.80E-01	9.05E-02	1.00E+00	-9.06E-02	1.00E+00
ENSBTAG00000045162	0.00E+00	1.00E+00	0.00E+00	1.00E+00	-5.13E-15	1.00E+00	1.76E+00	4.97E-01	1.93E+00	8.00E-01
ENSBTAG00000024806	0.00E+00	1.00E+00	0.00E+00	1.00E+00	-5.13E-15	1.00E+00	0.00E+00	1.00E+00	0.00E+00	1.00E+00
ENSBTAG00000047093	0.00E+00	1.00E+00	0.00E+00	1.00E+00	-5.13E-15	1.00E+00	0.00E+00	1.00E+00	0.00E+00	1.00E+00
ENSBTAG00000044510	0.00E+00	1.00E+00	0.00E+00	1.00E+00	-5.13E-15	1.00E+00	0.00E+00	1.00E+00	0.00E+00	1.00E+00
ENSBTAG00000003666	-5.03E-01	1.00E+00	8.66E-02	1.00E+00	-1.58E-01	1.00E+00	1.80E-01	1.00E+00	3.96E-01	1.00E+00
ENSBTAG00000038252	0.00E+00	1.00E+00	0.00E+00	1.00E+00	-5.13E-15	1.00E+00	0.00E+00	1.00E+00	0.00E+00	1.00E+00
ENSBTAG00000000088	1.72E-01	1.00E+00	4.55E-01	1.57E-01	2.33E-01	5.96E-01	2.71E-03	1.00E+00	-2.24E-02	1.00E+00
ENSBTAG00000013057	2.78E-01	9.45E-01	6.20E-01	7.83E-02	1.57E-01	9.09E-01	4.61E-01	2.45E-01	-2.25E-01	1.00E+00
ENSBTAG00000005414	-1.08E-01	1.00E+00	-5.65E-01	2.97E-02	-8.37E-01	5.90E-04	-6.54E-01	1.09E-02	-8.49E-01	1.03E-02
ENSBTAG00000045897	0.00E+00	1.00E+00	0.00E+00	1.00E+00	-5.13E-15	1.00E+00	0.00E+00	1.00E+00	0.00E+00	1.00E+00
ENSBTAG00000047234	0.00E+00	1.00E+00	0.00E+00	1.00E+00	-5.13E-15	1.00E+00	0.00E+00	1.00E+00	0.00E+00	1.00E+00
ENSBTAG00000045335	-4.16E+00	8.48E-02	-4.16E+00	1.92E-02	-2.38E+00	9.83E-02	-4.16E+00	1.20E-02	-4.16E+00	1.35E-01
ENSBTAG00000046313	2.71E+00	5.76E-01	0.00E+00	1.00E+00	-5.13E-15	1.00E+00	0.00E+00	1.00E+00	3.21E+00	4.34E-01
ENSBTAG00000039575	0.00E+00	1.00E+00	0.00E+00	1.00E+00	-5.13E-15	1.00E+00	0.00E+00	1.00E+00	0.00E+00	1.00E+00
ENSBTAG00000006893	2.26E-01	9.67E-01	1.70E-01	7.91E-01	1.63E-01	8.07E-01	4.87E-01	1.16E-01	2.29E-01	9.24E-01
ENSBTAG00000003585	-1.22E-02	1.00E+00	2.85E-02	1.00E+00	7.99E-03	1.00E+00	-8.72E-02	1.00E+00	5.01E-02	1.00E+00
ENSBTAG00000005787	-4.46E-02	1.00E+00	-5.44E-01	2.19E-02	-2.95E-01	2.90E-01	5.18E-02	1.00E+00	-1.08E-01	1.00E+00
ENSBTAG00000018397	-3.22E+00	3.75E-01	-1.24E+00	6.08E-01	-1.70E-01	1.00E+00	-1.46E+00	4.92E-01	9.28E-01	9.12E-01
ENSBTAG00000018399	-8.07E-01	7.80E-01	-8.73E-01	3.30E-01	-9.91E-01	2.23E-01	-5.46E-01	5.93E-01	-1.51E-01	1.00E+00
ENSBTAG00000017140	-3.86E-01	9.45E-01	-9.02E-01	8.20E-02	8.49E-01	6.25E-02	8.47E-01	6.96E-02	-3.79E-01	9.24E-01
ENSBTAG00000006158	4.56E-01	7.42E-01	-4.48E-01	3.66E-01	-5.66E-01	1.99E-01	-9.30E-01	2.01E-02	6.76E-01	4.04E-01
ENSBTAG00000047012	0.00E+00	1.00E+00	0.00E+00	1.00E+00	-5.13E-15	1.00E+00	0.00E+00	1.00E+00	0.00E+00	1.00E+00
ENSBTAG00000000183	-1.64E-02	1.00E+00	5.97E-01	1.12E-01	-6.62E-01	8.45E-02	-9.82E-01	7.04E-03	-8.40E-01	1.80E-01
ENSBTAG00000015475	-5.29E-03	1.00E+00	-6.28E-01	2.91E-01	-1.64E+00	9.94E-04	-1.43E+00	4.50E-03	6.32E-01	6.08E-01
ENSBTAG00000044520	0.00E+00	1.00E+00	0.00E+00	1.00E+00	-5.13E-15	1.00E+00	0.00E+00	1.00E+00	0.00E+00	1.00E+00
ENSBTAG00000020460	0.00E+00	1.00E+00	0.00E+00	1.00E+00	1.78E+00	4.79E-01	1.76E+00	4.97E-01	2.71E+00	6.01E-01
ENSBTAG00000038103	0.00E+00	1.00E+00	1.99E+00	4.43E-01	2.56E+00	2.54E-01	0.00E+00	1.00E+00	1.93E+00	8.00E-01
ENSBTAG00000022275	9.54E-02	1.00E+00	6.47E-02	1.00E+00	-1.42E-01	8.50E-01	-2.67E-01	4.73E-01	-2.93E-01	8.00E-01
ENSBTAG00000000609	8.40E-02	1.00E+00	2.14E-01	4.86E-01	3.74E-02	1.00E+00	8.78E-02	9.94E-01	-1.30E-01	1.00E+00
ENSBTAG00000042390	0.00E+00	1.00E+00	0.00E+00	1.00E+00	-5.13E-15	1.00E+00	0.00E+00	1.00E+00	0.00E+00	1.00E+00
ENSBTAG00000045855	0.00E+00	1.00E+00	0.00E+00	1.00E+00	-5.13E-15	1.00E+00	0.00E+00	1.00E+00	0.00E+00	1.00E+00

ENSBTAG00000033360	4.24E-02	1.00E+00	-3.76E-01	6.10E-01	-2.50E-01	8.39E-01	1.32E-01	1.00E+00	3.47E-02	1.00E+00
ENSBTAG00000045820	0.00E+00	1.00E+00	0.00E+00	1.00E+00	-5.13E-15	1.00E+00	0.00E+00	1.00E+00	0.00E+00	1.00E+00
ENSBTAG00000038651	0.00E+00	1.00E+00	0.00E+00	1.00E+00	-5.13E-15	1.00E+00	0.00E+00	1.00E+00	0.00E+00	1.00E+00
ENSBTAG00000013769	-1.60E-01	1.00E+00	-1.62E+00	7.08E-11	-1.35E+00	4.01E-08	3.47E-02	1.00E+00	2.05E-01	9.44E-01
ENSBTAG00000014441	-2.32E-01	1.00E+00	-8.22E-01	4.84E-02	-5.43E-01	2.35E-01	-1.05E+00	8.57E-03	-8.95E-01	1.97E-01
ENSBTAG00000046637	0.00E+00	1.00E+00	0.00E+00	1.00E+00	-5.13E-15	1.00E+00	0.00E+00	1.00E+00	0.00E+00	1.00E+00
ENSBTAG00000012646	-3.31E-02	1.00E+00	1.49E+00	4.87E-03	1.31E+00	1.47E-02	9.77E-01	9.97E-02	-2.84E-01	1.00E+00
ENSBTAG00000001209	3.06E-01	7.67E-01	-3.91E-01	1.92E-01	-1.82E+00	6.25E-14	-1.89E+00	8.72E-15	3.43E-01	6.61E-01
ENSBTAG00000004601	-3.22E-04	1.00E+00	-3.75E-01	1.62E-01	-3.57E-01	1.90E-01	-5.44E-01	2.75E-02	-1.92E-01	9.31E-01
ENSBTAG00000047422	0.00E+00	1.00E+00	0.00E+00	1.00E+00	-5.13E-15	1.00E+00	0.00E+00	1.00E+00	0.00E+00	1.00E+00
ENSBTAG00000004599	4.57E+00	1.70E-12	6.84E+00	7.58E-26	6.24E+00	1.36E-22	3.81E+00	8.25E-10	2.05E+00	3.13E-02
ENSBTAG00000015065	2.61E-01	1.00E+00	-5.69E-02	1.00E+00	-6.17E-01	1.28E-01	-1.69E+00	1.03E-05	8.28E-01	1.28E-01
ENSBTAG00000016554	-5.61E-01	8.98E-01	7.65E-01	2.24E-01	-9.26E-02	1.00E+00	2.60E-01	9.20E-01	-5.67E-01	8.60E-01
ENSBTAG00000016556	5.18E-02	1.00E+00	-8.70E-02	1.00E+00	-2.04E-01	1.00E+00	-9.11E-01	2.22E-01	-6.97E-01	8.00E-01
ENSBTAG00000019332	9.40E-01	7.31E-02	1.68E+00	1.09E-06	7.31E-01	7.02E-02	-1.19E+00	6.58E-03	-2.85E-01	1.00E+00
ENSBTAG00000033319	6.82E-01	7.43E-01	-3.75E-01	7.46E-01	-1.50E+00	1.09E-02	-1.91E+00	1.29E-03	-4.99E-02	1.00E+00
ENSBTAG00000011871	1.45E+00	1.65E-01	-1.90E+00	1.18E-01	-5.02E-01	8.06E-01	-7.46E-01	5.84E-01	1.06E+00	5.85E-01
ENSBTAG00000009084	4.81E-01	3.98E-01	4.32E-01	1.87E-01	1.96E-01	7.09E-01	2.02E-01	7.00E-01	-6.14E-02	1.00E+00
ENSBTAG00000009085	2.64E-01	6.81E-01	1.70E-01	5.94E-01	1.40E-01	7.15E-01	-3.62E-01	1.15E-01	1.86E-01	8.71E-01
ENSBTAG00000010793	3.74E-02	1.00E+00	1.98E+00	3.45E-03	1.57E+00	2.37E-02	1.49E+00	3.85E-02	2.21E+00	1.71E-02
ENSBTAG00000040144	-6.29E-01	1.00E+00	-5.34E-01	8.37E-01	4.09E-01	8.75E-01	-2.75E+00	3.47E-02	8.09E-01	8.00E-01
ENSBTAG00000001235	2.57E-01	1.00E+00	-3.21E-01	7.36E-01	-6.85E-01	2.27E-01	-5.08E-01	4.39E-01	-5.01E-01	8.00E-01
ENSBTAG00000006534	-2.42E-01	8.48E-01	9.27E-01	4.21E-05	1.37E+00	2.73E-10	9.98E-01	9.67E-06	-8.15E-01	1.68E-02
ENSBTAG00000005723	-1.82E-01	9.71E-01	5.18E-01	2.27E-02	9.72E-01	2.67E-06	7.54E-01	4.79E-04	-6.60E-01	4.36E-02
ENSBTAG00000000242	1.13E-02	1.00E+00	8.21E-01	1.89E-04	1.01E+00	2.78E-06	8.71E-01	8.21E-05	-2.56E-01	8.00E-01
ENSBTAG00000013909	1.38E-01	1.00E+00	-2.73E-01	7.67E-01	2.67E-02	1.00E+00	2.27E-04	1.00E+00	-9.98E-02	1.00E+00
ENSBTAG00000013920	2.39E-01	1.00E+00	-7.26E-01	2.83E-01	-1.45E+00	1.15E-02	-2.00E+00	6.51E-04	3.86E-01	9.87E-01
ENSBTAG00000013937	-7.67E-02	1.00E+00	-1.89E+00	4.12E-11	-9.88E-01	5.67E-04	-1.01E+00	5.17E-04	1.49E-01	1.00E+00
ENSBTAG00000013292	-1.92E-01	1.00E+00	-1.50E+00	3.69E-03	-1.08E+00	2.91E-02	-1.06E+00	3.77E-02	-1.30E-01	1.00E+00
ENSBTAG00000042593	0.00E+00	1.00E+00	0.00E+00	1.00E+00	-5.13E-15	1.00E+00	0.00E+00	1.00E+00	0.00E+00	1.00E+00
ENSBTAG00000021000	-1.01E-01	1.00E+00	5.67E-01	2.68E-01	4.73E-01	3.98E-01	1.11E+00	1.03E-02	1.08E+00	1.07E-01
ENSBTAG00000007784	2.17E-01	8.23E-01	1.70E+00	7.63E-19	1.17E+00	1.08E-09	1.07E+00	3.59E-08	-1.73E-01	9.19E-01
ENSBTAG00000002703	3.32E-01	6.82E-01	1.18E+00	1.02E-06	6.32E-01	1.49E-02	7.16E-01	5.72E-03	8.97E-03	1.00E+00
ENSBTAG00000004971	-2.61E-01	7.97E-01	-1.67E+00	2.35E-14	-1.32E+00	8.84E-10	-4.24E-01	9.34E-02	1.14E-01	1.00E+00
ENSBTAG00000023550	0.00E+00	1.00E+00	0.00E+00	1.00E+00	-5.13E-15	1.00E+00	0.00E+00	1.00E+00	0.00E+00	1.00E+00

ENSBTAG00000044668	0.00E+00	1.00E+00	0.00E+00	1.00E+00	-5.13E-15	1.00E+00	0.00E+00	1.00E+00	0.00E+00	1.00E+00
ENSBTAG00000004974	1.55E+00	7.39E-02	2.87E+00	1.40E-07	2.57E+00	3.04E-06	3.47E+00	7.00E-11	5.14E-01	9.73E-01
ENSBTAG00000004975	5.17E-01	5.86E-01	6.08E-01	1.39E-01	6.95E-01	7.40E-02	5.72E-01	1.79E-01	5.69E-01	5.50E-01
ENSBTAG00000004977	5.37E-01	3.59E-01	2.87E+00	6.00E-29	2.33E+00	9.18E-20	1.60E+00	1.67E-09	-5.10E-03	1.00E+00
ENSBTAG00000042839	0.00E+00	1.00E+00	0.00E+00	1.00E+00	-5.13E-15	1.00E+00	0.00E+00	1.00E+00	0.00E+00	1.00E+00
ENSBTAG00000002132	-5.59E-01	1.00E+00	-1.62E-01	1.00E+00	9.82E-01	1.94E-01	-8.07E-02	1.00E+00	-9.70E-01	7.98E-01
ENSBTAG00000011921	6.13E-03	1.00E+00	1.26E+00	2.90E-02	5.52E-01	4.91E-01	-3.94E-02	1.00E+00	3.28E-01	1.00E+00
ENSBTAG00000036373	-1.37E-01	1.00E+00	-8.99E-01	1.67E-02	-2.38E+00	6.29E-11	-2.16E+00	2.55E-09	-5.60E-01	5.58E-01
ENSBTAG00000011928	1.86E-01	1.00E+00	-9.62E-01	1.79E-03	-1.71E+00	9.68E-09	-1.60E+00	9.68E-08	-6.02E-01	3.40E-01
ENSBTAG00000006451	-2.79E-01	1.00E+00	-1.95E-01	1.00E+00	-4.48E-01	9.69E-01	1.02E+00	3.84E-01	-2.66E-01	1.00E+00
ENSBTAG00000044343	0.00E+00	1.00E+00	0.00E+00	1.00E+00	-5.13E-15	1.00E+00	0.00E+00	1.00E+00	0.00E+00	1.00E+00
ENSBTAG00000046931	2.11E-02	1.00E+00	1.01E+00	2.95E-04	2.00E+00	5.54E-14	1.53E+00	1.52E-08	-5.67E-01	3.49E-01
ENSBTAG00000040393	-1.05E-01	1.00E+00	-1.00E+00	1.03E-03	-2.61E+00	2.35E-17	-3.95E+00	3.89E-31	-3.71E-01	7.59E-01
ENSBTAG00000046854	1.53E-01	1.00E+00	2.49E+00	7.83E-15	2.26E+00	1.59E-12	1.84E+00	1.82E-08	-1.60E-01	1.00E+00
ENSBTAG00000045704	3.53E-02	1.00E+00	2.27E-01	7.61E-01	-8.27E-01	2.35E-02	-1.26E+00	3.44E-04	1.93E-01	1.00E+00
ENSBTAG00000014157	0.00E+00	1.00E+00	0.00E+00	1.00E+00	-5.13E-15	1.00E+00	0.00E+00	1.00E+00	0.00E+00	1.00E+00
ENSBTAG00000009014	-6.12E-01	1.00E+00	-4.44E-02	1.00E+00	-6.53E-01	6.44E-01	-5.74E-01	7.32E-01	-7.93E-01	8.78E-01
ENSBTAG00000001390	2.94E-01	9.50E-01	1.10E+00	1.09E-03	9.95E-01	3.59E-03	9.25E-01	8.65E-03	-1.40E-01	1.00E+00
ENSBTAG00000000210	5.19E-01	2.30E-01	1.37E+00	5.25E-09	4.28E-01	1.38E-01	3.03E-01	3.76E-01	-9.44E-02	1.00E+00
ENSBTAG00000000213	8.83E-01	3.82E-03	2.40E+00	1.34E-22	1.76E+00	5.14E-13	1.32E+00	1.06E-07	-1.57E-01	1.00E+00
ENSBTAG00000008562	3.66E-02	1.00E+00	6.46E-01	1.16E-02	1.10E+00	2.66E-06	1.18E+00	6.10E-07	6.24E-02	1.00E+00
ENSBTAG00000012581	1.22E-03	1.00E+00	3.99E-01	1.70E-01	-4.39E-02	1.00E+00	-4.79E-01	9.13E-02	6.54E-02	1.00E+00
ENSBTAG00000018059	1.81E+00	9.96E-06	-1.73E-01	9.45E-01	-1.97E+00	2.69E-07	-8.54E-01	4.32E-02	6.06E-01	5.50E-01
ENSBTAG00000009391	6.12E-01	3.77E-01	3.48E-01	4.73E-01	-7.40E-02	1.00E+00	4.48E-01	3.18E-01	5.15E-01	6.01E-01
ENSBTAG00000009392	4.96E-01	4.25E-01	1.19E-01	9.88E-01	-1.22E+00	1.96E-05	-1.83E+00	1.23E-10	5.82E-01	3.32E-01
ENSBTAG00000005604	1.99E+00	5.76E-01	1.40E+00	5.16E-01	6.40E-01	9.79E-01	6.26E-01	9.94E-01	2.94E+00	1.39E-01
ENSBTAG00000002182	-2.42E-02	1.00E+00	-7.33E-02	1.00E+00	-5.37E-01	5.36E-02	-7.02E-01	9.00E-03	-4.32E-01	4.91E-01
ENSBTAG00000001293	-8.06E-01	6.80E-01	-3.00E-01	8.88E-01	-6.61E-01	4.06E-01	-9.22E-01	2.05E-01	-3.78E-01	1.00E+00
ENSBTAG00000044376	0.00E+00	1.00E+00	1.98E+00	4.43E-01	-5.13E-15	1.00E+00	0.00E+00	1.00E+00	0.00E+00	1.00E+00
ENSBTAG00000019557	-2.26E-01	1.00E+00	-3.14E+00	1.96E-24	-3.79E+00	2.08E-33	-2.32E+00	1.11E-14	1.06E-01	1.00E+00
ENSBTAG00000040597	-1.48E-01	1.00E+00	-1.58E+00	1.25E-06	-1.53E+00	1.44E-06	-6.06E-01	8.73E-02	-7.14E-01	2.28E-01
ENSBTAG00000048057	-7.92E-02	1.00E+00	-7.93E-01	8.90E-04	-9.65E-01	3.21E-05	-1.19E-01	8.98E-01	-1.89E-01	9.52E-01
ENSBTAG00000001464	3.87E-01	9.19E-01	4.97E-01	3.86E-01	-7.34E-01	1.44E-01	-3.41E-02	1.00E+00	8.32E-01	3.47E-01
ENSBTAG00000033170	3.00E-01	9.62E-01	7.19E-01	6.31E-02	2.79E-01	6.45E-01	1.19E+00	8.11E-04	6.55E-01	4.15E-01
ENSBTAG00000022155	1.12E+00	3.65E-04	2.12E+00	4.86E-15	2.63E+00	4.31E-22	2.72E+00	3.77E-23	1.83E-01	1.00E+00

ENSBTAG00000015892	-1.37E-01	1.00E+00	1.80E-01	6.92E-01	1.43E-01	8.20E-01	1.08E-01	9.60E-01	6.24E-02	1.00E+00
ENSBTAG00000015895	-2.52E-01	9.33E-01	-1.28E+00	3.59E-06	-3.27E+00	7.09E-31	-3.63E+00	1.74E-36	5.50E-02	1.00E+00
ENSBTAG00000015898	-4.39E-01	4.44E-01	-2.99E-01	4.04E-01	9.62E-02	1.00E+00	5.32E-01	6.45E-02	-2.14E-01	9.43E-01
ENSBTAG00000016848	-1.90E-01	1.00E+00	2.42E-01	5.65E-01	7.55E-01	5.70E-03	7.97E-01	3.83E-03	-3.14E-01	8.00E-01
ENSBTAG00000045436	0.00E+00	1.00E+00	0.00E+00	1.00E+00	-5.13E-15	1.00E+00	0.00E+00	1.00E+00	0.00E+00	1.00E+00
ENSBTAG00000001918	-5.42E-01	8.23E-01	-4.96E-02	1.00E+00	-3.40E-02	1.00E+00	1.55E-01	1.00E+00	-1.75E-01	1.00E+00
ENSBTAG00000001920	-6.48E-01	5.76E-01	-6.68E-01	2.07E-01	-2.48E-01	8.07E-01	-4.08E-01	5.00E-01	-1.23E+00	9.93E-02
ENSBTAG00000002273	1.92E+00	8.23E-01	1.99E+00	4.43E-01	-5.13E-15	1.00E+00	0.00E+00	1.00E+00	1.91E+00	8.00E-01
ENSBTAG00000014508	-1.03E+00	3.29E-01	-3.36E-01	7.61E-01	-3.23E-02	1.00E+00	-1.14E-01	1.00E+00	9.35E-02	1.00E+00
ENSBTAG00000001009	7.45E-02	1.00E+00	1.08E-01	9.51E-01	6.16E-01	1.32E-02	4.82E-01	7.38E-02	-2.70E-01	8.00E-01
ENSBTAG00000008862	5.33E-01	3.16E-01	1.74E+00	1.39E-10	2.04E+00	4.18E-14	1.55E+00	1.41E-08	4.63E-01	5.08E-01
ENSBTAG00000013069	-1.55E-01	1.00E+00	9.01E-02	1.00E+00	1.35E+00	1.38E-06	8.79E-01	3.47E-03	-5.96E-02	1.00E+00
ENSBTAG00000013070	3.71E-01	8.23E-01	-2.46E+00	9.93E-12	-3.83E+00	9.03E-23	-2.63E+00	2.03E-13	5.10E-01	6.01E-01
ENSBTAG00000033166	-1.46E-01	1.00E+00	-2.61E+00	3.35E-21	-3.58E+00	8.76E-36	-2.73E+00	3.53E-23	4.46E-01	5.22E-01
ENSBTAG00000001442	2.31E-01	1.00E+00	-1.42E+00	7.21E-02	-4.78E+00	2.77E-07	-2.49E+00	1.31E-03	6.88E-02	1.00E+00
ENSBTAG00000004705	1.78E-01	1.00E+00	-6.06E-01	3.17E-01	-9.29E-01	7.46E-02	-4.29E-01	5.18E-01	8.74E-01	2.74E-01
ENSBTAG00000013118	4.03E-01	4.37E-01	-3.26E-01	2.95E-01	-2.41E-02	1.00E+00	7.36E-02	1.00E+00	-6.07E-01	1.57E-01
ENSBTAG00000003865	2.22E-01	1.00E+00	1.53E+00	1.16E-02	1.32E+00	3.48E-02	1.13E+00	9.35E-02	8.30E-02	1.00E+00
ENSBTAG00000045101	0.00E+00	1.00E+00	0.00E+00	1.00E+00	-5.13E-15	1.00E+00	0.00E+00	1.00E+00	0.00E+00	1.00E+00
ENSBTAG00000014133	0.00E+00	1.00E+00	1.98E+00	4.43E-01	-5.13E-15	1.00E+00	1.76E+00	4.97E-01	0.00E+00	1.00E+00
ENSBTAG00000010688	0.00E+00	1.00E+00	1.99E+00	4.43E-01	-5.13E-15	1.00E+00	1.76E+00	4.97E-01	1.93E+00	8.00E-01
ENSBTAG00000011138	-2.01E-01	9.48E-01	-7.48E-03	1.00E+00	1.64E-01	7.11E-01	3.23E-02	1.00E+00	-1.75E-01	9.93E-01
ENSBTAG00000011140	0.00E+00	1.00E+00	0.00E+00	1.00E+00	-5.13E-15	1.00E+00	0.00E+00	1.00E+00	0.00E+00	1.00E+00
ENSBTAG00000011143	2.69E-01	8.23E-01	2.16E+00	5.20E-19	2.36E+00	1.93E-22	1.78E+00	1.91E-13	1.23E-01	1.00E+00
ENSBTAG00000042660	0.00E+00	1.00E+00	0.00E+00	1.00E+00	-5.13E-15	1.00E+00	0.00E+00	1.00E+00	0.00E+00	1.00E+00
ENSBTAG00000021791	1.01E-01	1.00E+00	4.74E-01	5.41E-01	8.82E-02	1.00E+00	-5.54E-01	4.55E-01	-3.48E-02	1.00E+00
ENSBTAG00000009933	3.19E-01	1.00E+00	1.54E+00	1.82E-02	7.22E-01	3.81E-01	-8.34E-02	1.00E+00	-3.94E-02	1.00E+00
ENSBTAG00000016656	7.85E-01	7.08E-01	2.62E+00	2.41E-05	1.87E+00	2.73E-03	2.83E-01	9.37E-01	-1.17E-02	1.00E+00
ENSBTAG00000005066	1.07E-01	1.00E+00	8.87E-01	2.47E-02	1.12E+00	2.93E-03	5.25E-01	2.68E-01	4.09E-01	8.00E-01
ENSBTAG00000005071	-4.93E-02	1.00E+00	1.04E+00	3.60E-07	1.15E+00	8.11E-09	6.74E-01	1.80E-03	-1.73E-01	9.64E-01
ENSBTAG00000018765	-6.08E-01	7.41E-01	-2.06E+00	1.21E-04	-2.46E+00	3.60E-06	-2.11E+00	5.56E-05	-1.60E-01	1.00E+00
ENSBTAG00000018877	-2.26E-01	8.84E-01	-1.53E-01	7.74E-01	-5.72E-01	2.07E-02	-6.81E-01	5.34E-03	2.77E-01	7.93E-01
ENSBTAG00000014797	-4.52E-02	1.00E+00	5.82E-01	1.57E-02	2.07E-01	5.57E-01	1.97E-01	6.04E-01	3.07E-03	1.00E+00
ENSBTAG00000018777	5.15E-01	2.82E-01	-6.32E-01	2.74E-02	-3.04E-01	4.04E-01	-2.50E-01	5.32E-01	2.77E-01	8.00E-01
ENSBTAG00000043908	0.00E+00	1.00E+00	0.00E+00	1.00E+00	-5.13E-15	1.00E+00	0.00E+00	1.00E+00	0.00E+00	1.00E+00

ENSBTAG00000044318	2.23E-02	1.00E+00	-5.73E-01	8.94E-01	-3.88E+00	2.29E-02	-8.51E-01	6.68E-01	2.50E-01	1.00E+00
ENSBTAG00000023607	4.13E-02	1.00E+00	4.09E-01	1.10E-01	4.54E-01	6.73E-02	5.38E-01	2.70E-02	1.93E-01	9.22E-01
ENSBTAG00000023608	-2.79E+00	1.40E-01	-4.71E+00	3.29E-03	-4.71E+00	1.47E-03	-4.71E+00	1.66E-03	-2.79E+00	2.01E-01
ENSBTAG00000014567	1.86E-02	1.00E+00	-7.49E-01	8.57E-03	-8.71E-01	1.74E-03	-6.83E-01	2.09E-02	1.42E-01	1.00E+00
ENSBTAG00000000566	-5.89E-02	1.00E+00	-1.61E+00	1.90E-08	-1.63E+00	6.34E-09	-9.21E-01	1.48E-03	-1.91E-01	1.00E+00
ENSBTAG00000006947	3.08E-01	1.00E+00	8.59E-01	5.80E-01	-5.32E-01	9.39E-01	9.06E-01	5.16E-01	-3.67E-01	1.00E+00
ENSBTAG00000044700	0.00E+00	1.00E+00	0.00E+00	1.00E+00	-5.13E-15	1.00E+00	0.00E+00	1.00E+00	0.00E+00	1.00E+00
ENSBTAG00000002640	8.71E-01	6.05E-01	-1.30E+00	5.24E-02	-3.18E+00	5.16E-07	-2.01E+00	1.77E-03	8.79E-01	6.13E-01
ENSBTAG00000045961	0.00E+00	1.00E+00	0.00E+00	1.00E+00	-5.13E-15	1.00E+00	0.00E+00	1.00E+00	0.00E+00	1.00E+00
ENSBTAG00000046935	0.00E+00	1.00E+00	0.00E+00	1.00E+00	-5.13E-15	1.00E+00	0.00E+00	1.00E+00	0.00E+00	1.00E+00
ENSBTAG00000013727	-7.31E-02	1.00E+00	7.68E-01	2.57E-03	1.00E+00	4.60E-05	5.11E-01	7.02E-02	-2.54E-01	8.26E-01
ENSBTAG00000013755	-3.88E-02	1.00E+00	-9.76E-01	5.05E-06	-2.07E+00	1.15E-22	-1.89E+00	2.93E-19	2.71E-01	7.62E-01
ENSBTAG00000010382	2.27E-01	1.00E+00	-1.33E+00	4.38E-01	6.69E-01	6.91E-01	1.39E+00	1.73E-01	-9.17E-01	9.52E-01
ENSBTAG00000021396	-3.53E-01	7.50E-01	-4.93E-01	1.32E-01	-2.46E-01	5.73E-01	2.94E-01	4.61E-01	1.82E-01	1.00E+00
ENSBTAG00000000496	3.24E-01	1.00E+00	-1.47E-01	1.00E+00	-2.02E-01	9.69E-01	-1.14E+00	3.01E-02	3.49E-01	9.95E-01
ENSBTAG00000040031	-2.24E-01	8.23E-01	-1.07E+00	2.20E-07	-9.05E-01	1.18E-05	-4.83E-01	3.57E-02	-5.54E-01	1.08E-01
ENSBTAG00000011383	-9.52E-02	1.00E+00	-8.03E-01	5.87E-04	-6.37E-01	7.68E-03	5.21E-03	1.00E+00	-2.35E-01	8.17E-01
ENSBTAG00000006471	-1.78E-01	1.00E+00	-6.70E-01	1.02E-02	-7.79E-01	2.17E-03	-7.66E-01	3.13E-03	-5.10E-01	3.20E-01
ENSBTAG00000003383	5.70E-01	5.68E-01	1.54E+00	2.23E-05	2.06E+00	6.98E-09	2.13E+00	3.03E-09	7.07E-01	3.92E-01
ENSBTAG00000044172	-1.72E-02	1.00E+00	2.23E-01	8.53E-01	8.43E-01	3.83E-02	1.66E+00	4.92E-06	4.65E-01	7.89E-01
ENSBTAG00000045455	1.12E-02	1.00E+00	8.79E-01	8.52E-01	-1.26E-01	1.00E+00	-1.91E+00	4.39E-01	-1.91E+00	8.00E-01
ENSBTAG00000014208	1.95E-01	1.00E+00	6.22E-01	2.95E-02	5.34E-01	7.13E-02	4.91E-01	1.13E-01	-6.04E-01	2.36E-01
ENSBTAG00000018025	2.15E-01	1.00E+00	-1.23E+00	1.32E-03	-1.02E+00	6.06E-03	-4.08E-01	4.03E-01	1.06E-01	1.00E+00
ENSBTAG00000032839	2.97E-01	8.23E-01	1.68E-01	8.28E-01	2.91E-01	4.66E-01	2.37E-01	6.10E-01	1.76E-01	1.00E+00
ENSBTAG00000042610	0.00E+00	1.00E+00	0.00E+00	1.00E+00	-5.13E-15	1.00E+00	0.00E+00	1.00E+00	0.00E+00	1.00E+00
ENSBTAG00000043376	0.00E+00	1.00E+00	0.00E+00	1.00E+00	-5.13E-15	1.00E+00	0.00E+00	1.00E+00	0.00E+00	1.00E+00
ENSBTAG00000014874	-6.40E-02	1.00E+00	1.80E-01	6.40E-01	5.28E-01	2.56E-02	7.10E-01	1.89E-03	2.59E-02	1.00E+00
ENSBTAG00000001890	1.69E-02	1.00E+00	-6.00E-01	7.79E-02	-4.23E-02	1.00E+00	-9.08E-02	1.00E+00	4.19E-01	6.38E-01
ENSBTAG00000032819	5.17E-01	8.56E-01	-2.02E+00	2.67E-04	-2.34E+00	2.08E-05	-2.83E-01	8.72E-01	1.20E+00	2.01E-01
ENSBTAG00000014721	2.24E-01	1.00E+00	-8.25E-01	6.67E-01	5.56E-02	1.00E+00	-2.37E+00	1.04E-01	5.50E-01	1.00E+00
ENSBTAG00000021364	-2.17E-01	9.06E-01	-5.74E-01	2.05E-02	-7.96E-01	6.66E-04	-7.65E-01	1.37E-03	-5.28E-02	1.00E+00
ENSBTAG00000032719	2.34E-03	1.00E+00	-8.35E-01	6.43E-02	8.20E-03	1.00E+00	-2.27E-01	8.59E-01	1.46E-01	1.00E+00
ENSBTAG00000021515	1.41E+00	3.01E-03	4.33E+00	2.19E-27	5.71E+00	2.81E-42	5.21E+00	1.11E-36	4.81E-01	7.90E-01
ENSBTAG00000012347	2.31E+00	6.32E-04	6.17E+00	2.23E-22	6.47E+00	3.94E-24	5.11E+00	5.78E-17	1.66E+00	6.81E-02
ENSBTAG00000016979	-9.23E-02	1.00E+00	7.75E-01	1.31E-03	8.84E-01	1.88E-04	3.95E-01	1.68E-01	-2.75E-01	8.00E-01

ENSBTAG00000032684	-2.38E-01	1.00E+00	-4.79E-01	2.10E-01	-1.41E+00	9.24E-06	-1.30E+00	5.54E-05	-9.34E-04	1.00E+00
ENSBTAG00000040161	4.76E-01	8.72E-01	1.63E+00	5.24E-04	5.09E-01	4.54E-01	-9.33E-02	1.00E+00	6.86E-01	6.27E-01
ENSBTAG00000007577	1.64E-01	1.00E+00	7.48E-01	6.77E-02	7.77E-01	5.46E-02	7.83E-01	5.81E-02	1.66E-01	1.00E+00
ENSBTAG00000013317	-6.44E-01	7.73E-02	-2.76E-01	4.38E-01	2.31E-01	5.28E-01	4.24E-01	1.48E-01	-1.90E-01	9.90E-01
ENSBTAG00000013319	8.42E-01	8.23E-01	5.80E-01	6.83E-01	-2.01E-01	1.00E+00	-1.51E+00	1.60E-01	7.70E-01	8.16E-01
ENSBTAG00000014660	-3.70E-01	6.01E-01	7.77E-01	2.51E-03	9.18E-01	2.37E-04	5.89E-02	1.00E+00	-3.30E-01	7.34E-01
ENSBTAG00000006388	-5.12E-01	8.23E-01	6.53E-01	2.16E-01	1.07E+00	1.41E-02	1.09E+00	1.40E-02	6.84E-01	5.65E-01
ENSBTAG00000009614	3.40E-01	7.41E-01	-4.31E-01	1.93E-01	-6.66E-01	2.23E-02	-7.02E-01	1.72E-02	-4.28E-01	5.82E-01
ENSBTAG00000013192	7.36E-01	2.51E-02	2.43E-01	5.13E-01	-1.37E+00	9.97E-08	-6.50E-01	1.84E-02	-2.07E-02	1.00E+00
ENSBTAG00000048195	1.54E-01	1.00E+00	-3.18E-01	1.96E-01	-6.22E-01	2.79E-03	-6.78E-01	1.19E-03	-4.71E-01	2.09E-01
ENSBTAG00000047856	2.61E-01	1.00E+00	8.86E-01	1.19E-02	9.74E-01	4.84E-03	1.12E+00	1.19E-03	1.33E-01	1.00E+00
ENSBTAG00000038422	0.00E+00	1.00E+00	0.00E+00	1.00E+00	-5.13E-15	1.00E+00	0.00E+00	1.00E+00	0.00E+00	1.00E+00
ENSBTAG00000032674	6.11E-01	1.74E-01	1.57E+00	2.54E-09	1.21E+00	5.85E-06	1.05E+00	1.21E-04	3.93E-01	6.27E-01
ENSBTAG00000008873	2.79E-04	1.00E+00	3.98E-01	1.40E-01	6.25E-01	9.56E-03	8.66E-01	2.13E-04	-1.45E-01	1.00E+00
ENSBTAG00000011659	-4.01E-01	9.62E-01	-1.64E+00	7.90E-04	-3.28E+00	3.00E-11	-2.05E+00	2.53E-05	1.05E-01	1.00E+00
ENSBTAG00000002998	-4.20E-01	1.00E+00	6.96E-01	2.67E-01	-2.47E-01	9.51E-01	4.33E-01	5.97E-01	1.27E-02	1.00E+00
ENSBTAG00000020378	1.20E-01	1.00E+00	-6.99E-01	1.14E-02	-8.06E-01	2.76E-03	-1.77E-01	7.60E-01	1.20E-01	1.00E+00
ENSBTAG00000045354	0.00E+00	1.00E+00	0.00E+00	1.00E+00	-5.13E-15	1.00E+00	0.00E+00	1.00E+00	0.00E+00	1.00E+00
ENSBTAG00000043278	0.00E+00	1.00E+00	0.00E+00	1.00E+00	-5.13E-15	1.00E+00	0.00E+00	1.00E+00	1.91E+00	8.00E-01
ENSBTAG00000048225	-1.24E-01	1.00E+00	-6.15E-02	1.00E+00	-1.42E-01	7.35E-01	1.22E-01	8.28E-01	-7.67E-01	5.47E-03
ENSBTAG00000039659	0.00E+00	1.00E+00	0.00E+00	1.00E+00	-5.13E-15	1.00E+00	0.00E+00	1.00E+00	0.00E+00	1.00E+00
ENSBTAG00000000448	5.13E-01	1.00E+00	-3.59E-01	1.00E+00	-3.67E-01	9.86E-01	-4.01E-01	9.60E-01	-2.00E-01	1.00E+00
ENSBTAG00000023600	1.99E+00	1.37E-01	3.58E+00	3.69E-07	3.12E+00	1.80E-05	2.54E+00	1.65E-03	1.30E+00	6.50E-01
ENSBTAG00000006724	1.57E-01	1.00E+00	4.56E-01	1.44E-01	1.38E+00	6.22E-08	1.44E+00	2.02E-08	1.61E-01	1.00E+00
ENSBTAG00000003378	8.09E-02	1.00E+00	1.74E-01	7.22E-01	4.03E-01	1.62E-01	9.61E-01	8.21E-05	-7.20E-03	1.00E+00
ENSBTAG00000036946	0.00E+00	1.00E+00	0.00E+00	1.00E+00	-5.13E-15	1.00E+00	0.00E+00	1.00E+00	0.00E+00	1.00E+00
ENSBTAG00000014564	4.99E-02	1.00E+00	-2.80E-01	4.92E-01	-3.70E-01	3.03E-01	-2.50E-01	5.74E-01	8.23E-01	4.64E-02
ENSBTAG00000044535	0.00E+00	1.00E+00	0.00E+00	1.00E+00	-5.13E-15	1.00E+00	0.00E+00	1.00E+00	0.00E+00	1.00E+00
ENSBTAG00000008462	1.33E+00	1.10E-02	4.12E+00	2.41E-24	4.63E+00	9.48E-30	3.43E+00	7.02E-18	-3.25E-01	1.00E+00
ENSBTAG00000008461	4.92E-01	1.34E-01	2.20E+00	5.21E-28	1.74E+00	1.90E-18	8.70E-01	2.83E-05	-1.10E-01	1.00E+00
ENSBTAG00000002394	-5.67E-01	9.04E-01	-2.96E-01	9.14E-01	2.43E-02	1.00E+00	-2.43E-01	9.83E-01	1.96E-01	1.00E+00
ENSBTAG00000045117	0.00E+00	1.00E+00	0.00E+00	1.00E+00	-5.13E-15	1.00E+00	0.00E+00	1.00E+00	0.00E+00	1.00E+00
ENSBTAG00000013204	8.16E-01	7.46E-02	9.54E-01	3.20E-03	1.47E-01	9.40E-01	3.36E-01	4.65E-01	5.08E-01	5.49E-01
ENSBTAG00000013203	2.33E+00	1.11E-15	1.51E+00	1.31E-07	1.11E+00	1.50E-04	1.91E+00	1.45E-11	9.83E-01	1.84E-02
ENSBTAG00000038600	2.43E+00	3.23E-01	2.72E+00	4.35E-02	1.81E+00	2.89E-01	3.82E+00	3.49E-04	3.06E+00	1.16E-01

ENSBTAG00000032656	5.80E-01	8.08E-02	5.13E-01	3.64E-02	2.90E-02	1.00E+00	-6.92E-02	1.00E+00	3.95E-01	4.70E-01
ENSBTAG00000000569	-8.65E-02	1.00E+00	1.55E+00	2.56E-06	1.10E+00	1.23E-03	9.34E-01	8.34E-03	8.72E-01	1.18E-01
ENSBTAG00000048136	1.26E-02	1.00E+00	-7.14E-01	9.45E-01	-1.42E-01	1.00E+00	-2.70E+00	2.05E-01	-7.85E-01	1.00E+00
ENSBTAG00000019514	-7.51E-02	1.00E+00	1.97E-01	5.11E-01	5.78E-01	5.56E-03	5.68E-01	7.71E-03	-7.06E-02	1.00E+00
ENSBTAG00000016305	-2.86E-01	1.00E+00	-2.71E-01	9.45E-01	-7.03E-01	3.87E-01	-4.10E-01	7.29E-01	-5.41E-01	8.99E-01
ENSBTAG00000018657	1.57E-02	1.00E+00	-1.47E-01	1.00E+00	-3.99E-01	9.79E-01	-1.60E+00	2.67E-01	-2.21E+00	4.77E-01
ENSBTAG00000035844	2.96E-01	1.00E+00	-2.34E-01	1.00E+00	3.64E-01	9.13E-01	-3.38E-01	9.86E-01	1.07E+00	5.89E-01
ENSBTAG00000005430	7.06E-01	1.49E-01	5.67E-01	1.09E-01	5.46E-01	1.24E-01	5.56E-01	1.23E-01	-1.51E-01	1.00E+00
ENSBTAG00000012413	-3.93E-01	4.12E-01	-1.96E+00	2.32E-18	-3.52E+00	1.29E-48	-3.05E+00	6.70E-39	-3.12E-01	6.59E-01
ENSBTAG00000045796	0.00E+00	1.00E+00	0.00E+00	1.00E+00	-5.13E-15	1.00E+00	0.00E+00	1.00E+00	0.00E+00	1.00E+00
ENSBTAG00000006945	1.79E-01	1.00E+00	-1.00E+00	7.28E-05	-1.97E-01	6.50E-01	-1.33E-01	8.87E-01	6.87E-03	1.00E+00
ENSBTAG00000044148	1.11E-02	1.00E+00	8.82E-01	8.52E-01	-1.91E+00	4.28E-01	-1.47E-01	1.00E+00	-1.91E+00	8.00E-01
ENSBTAG00000018703	-1.30E-01	1.00E+00	8.85E-01	1.29E-01	-3.74E-01	7.64E-01	-8.24E-01	2.88E-01	-2.79E-01	1.00E+00
ENSBTAG00000023593	-7.87E-01	1.00E+00	-2.71E+00	2.42E-01	-2.71E+00	1.99E-01	-2.71E+00	2.05E-01	-2.71E+00	6.01E-01
ENSBTAG00000006813	2.60E-03	1.00E+00	9.48E-02	1.00E+00	-6.36E-01	2.80E-01	-6.36E-01	2.91E-01	-1.23E-01	1.00E+00
ENSBTAG00000013205	1.32E-01	1.00E+00	-6.93E-01	1.89E-02	-1.75E+00	1.17E-10	-1.71E+00	4.81E-10	-2.37E-01	9.26E-01
ENSBTAG00000013456	5.21E-01	1.00E+00	-7.17E-01	9.45E-01	-9.18E-01	8.09E-01	-1.73E-01	1.00E+00	5.13E-01	1.00E+00
ENSBTAG00000006494	-8.14E-02	1.00E+00	-1.43E+00	1.14E-01	-2.59E+00	4.16E-03	-5.65E+00	1.24E-05	9.39E-01	5.70E-01
ENSBTAG00000013148	-3.72E-01	1.00E+00	-2.32E+00	1.25E-06	-3.71E+00	8.31E-14	-1.56E+00	1.44E-03	9.74E-01	3.09E-01
ENSBTAG00000021025	-2.09E-01	1.00E+00	-1.46E+00	1.39E-07	-2.04E+00	2.27E-13	-9.54E-01	7.67E-04	-4.62E-01	5.04E-01
ENSBTAG00000015460	1.32E-01	1.00E+00	-4.24E-01	7.62E-01	-1.27E+00	9.81E-02	9.60E-01	1.29E-01	3.95E-02	1.00E+00
ENSBTAG00000032538	-4.95E-01	5.76E-01	-1.47E+00	1.99E-05	-2.14E+00	4.46E-10	-2.93E+00	3.94E-16	-6.69E-01	3.49E-01
ENSBTAG00000044001	2.94E-01	7.80E-01	9.03E-01	1.83E-04	9.63E-01	5.45E-05	5.34E-01	4.71E-02	-3.79E-01	5.97E-01
ENSBTAG00000036372	0.00E+00	1.00E+00	0.00E+00	1.00E+00	-5.13E-15	1.00E+00	0.00E+00	1.00E+00	0.00E+00	1.00E+00
ENSBTAG00000001511	3.37E+00	5.78E-26	4.96E+00	1.47E-49	3.60E+00	3.75E-30	2.13E+00	3.40E-12	2.08E-01	1.00E+00
ENSBTAG00000003179	1.91E+00	8.23E-01	0.00E+00	1.00E+00	2.55E+00	2.55E-01	0.00E+00	1.00E+00	0.00E+00	1.00E+00
ENSBTAG00000017312	0.00E+00	1.00E+00	1.99E+00	4.43E-01	-5.13E-15	1.00E+00	0.00E+00	1.00E+00	0.00E+00	1.00E+00
ENSBTAG00000032265	-5.44E-01	1.00E+00	4.68E-01	8.73E-01	3.43E-01	9.97E-01	-8.12E-01	5.43E-01	-6.48E-01	1.00E+00
ENSBTAG00000012467	2.60E-01	7.50E-01	-7.18E-01	5.66E-04	-1.40E+00	2.11E-12	-7.92E-01	1.45E-04	1.36E-01	1.00E+00
ENSBTAG00000012706	0.00E+00	1.00E+00	0.00E+00	1.00E+00	-5.13E-15	1.00E+00	0.00E+00	1.00E+00	0.00E+00	1.00E+00
ENSBTAG00000044843	0.00E+00	1.00E+00	0.00E+00	1.00E+00	-5.13E-15	1.00E+00	0.00E+00	1.00E+00	0.00E+00	1.00E+00
ENSBTAG00000010357	4.63E-01	4.40E-01	2.64E+00	9.13E-23	2.65E+00	4.23E-23	2.93E+00	2.78E-27	1.23E+00	1.72E-04
ENSBTAG00000006872	-4.65E-01	7.69E-01	-1.49E-01	9.99E-01	-4.01E-01	4.35E-01	-2.81E-01	6.75E-01	2.30E-01	1.00E+00
ENSBTAG00000019813	-1.29E+00	9.38E-01	-3.21E+00	1.26E-01	-6.55E-01	8.97E-01	1.43E+00	2.38E-01	-1.29E+00	9.02E-01
ENSBTAG00000014727	7.10E-02	1.00E+00	5.24E-01	4.32E-02	1.06E+00	3.28E-06	9.10E-01	1.15E-04	-4.25E-01	4.77E-01

ENSBTAG00000014724	2.05E-01	7.97E-01	4.83E-01	7.08E-03	8.02E-01	1.95E-06	3.04E-01	1.37E-01	-5.13E-01	5.10E-02
ENSBTAG00000042702	0.00E+00	1.00E+00	0.00E+00	1.00E+00	-5.13E-15	1.00E+00	0.00E+00	1.00E+00	0.00E+00	1.00E+00
ENSBTAG00000042475	0.00E+00	1.00E+00	0.00E+00	1.00E+00	-5.13E-15	1.00E+00	0.00E+00	1.00E+00	0.00E+00	1.00E+00
ENSBTAG00000044788	0.00E+00	1.00E+00	0.00E+00	1.00E+00	-5.13E-15	1.00E+00	0.00E+00	1.00E+00	0.00E+00	1.00E+00
ENSBTAG00000044311	0.00E+00	1.00E+00	0.00E+00	1.00E+00	-5.13E-15	1.00E+00	0.00E+00	1.00E+00	0.00E+00	1.00E+00
ENSBTAG00000005122	-8.30E-02	1.00E+00	-4.77E-01	9.56E-03	-1.03E+00	1.17E-09	-1.31E+00	1.33E-14	-1.25E-01	1.00E+00
ENSBTAG00000001265	-2.23E-01	1.00E+00	-1.14E+00	7.44E-05	-2.13E+00	4.71E-14	-2.81E+00	1.33E-22	1.71E-01	1.00E+00
ENSBTAG00000017531	2.00E-02	1.00E+00	-8.27E-01	7.73E-03	-1.55E+00	1.16E-07	-1.86E+00	2.69E-10	-4.92E-01	5.20E-01
ENSBTAG00000000522	-1.61E-01	1.00E+00	-7.81E-01	7.64E-04	-1.70E+00	1.93E-14	-2.08E+00	1.72E-20	-2.56E-01	8.00E-01
ENSBTAG00000003151	1.94E+00	3.83E-09	4.64E+00	5.51E-43	3.91E+00	4.33E-33	2.79E+00	8.49E-19	3.46E-01	8.00E-01
ENSBTAG00000003143	9.86E-01	3.13E-04	1.49E+00	9.77E-11	2.83E-01	4.12E-01	5.71E-01	3.32E-02	-2.78E-01	8.00E-01
ENSBTAG00000003140	1.62E+00	1.80E-04	3.07E+00	1.27E-18	1.52E+00	7.14E-05	1.16E+00	4.91E-03	2.98E-01	1.00E+00
ENSBTAG00000005091	-1.97E-01	1.00E+00	-1.76E+00	5.25E-05	-1.38E+00	7.89E-04	-9.60E-01	2.39E-02	-3.56E-02	1.00E+00
ENSBTAG00000014915	7.37E-01	1.35E-01	8.21E-01	1.24E-02	4.98E-01	1.85E-01	4.68E-01	2.33E-01	-1.47E+00	6.49E-04
ENSBTAG00000011305	8.76E-01	1.00E+00	-7.23E-01	9.45E-01	-1.59E-01	1.00E+00	-1.78E-01	1.00E+00	1.24E-02	1.00E+00
ENSBTAG00000047841	0.00E+00	1.00E+00	0.00E+00	1.00E+00	-5.13E-15	1.00E+00	0.00E+00	1.00E+00	0.00E+00	1.00E+00
ENSBTAG00000043831	0.00E+00	1.00E+00	0.00E+00	1.00E+00	-5.13E-15	1.00E+00	0.00E+00	1.00E+00	0.00E+00	1.00E+00
ENSBTAG00000001697	5.59E-01	3.58E-02	1.18E+00	4.22E-10	4.53E-01	3.18E-02	1.32E-01	7.61E-01	-9.61E-02	1.00E+00
ENSBTAG00000007666	2.46E-01	1.00E+00	1.55E+00	4.99E-02	9.11E-01	3.40E-01	1.78E+00	2.17E-02	-5.26E-03	1.00E+00
ENSBTAG00000001749	9.34E-02	1.00E+00	1.71E-01	7.19E-01	-6.17E-02	1.00E+00	-3.81E-01	1.94E-01	6.23E-02	1.00E+00
ENSBTAG00000001535	2.12E-01	1.00E+00	-5.53E-02	1.00E+00	-1.64E+00	3.03E-08	-1.44E+00	1.64E-06	5.32E-01	4.34E-01
ENSBTAG00000029106	0.00E+00	1.00E+00	0.00E+00	1.00E+00	-5.13E-15	1.00E+00	0.00E+00	1.00E+00	0.00E+00	1.00E+00
ENSBTAG00000017115	-5.04E-01	2.39E-01	-6.31E-01	1.47E-02	2.81E-01	3.95E-01	1.62E-01	7.60E-01	1.23E-01	1.00E+00
ENSBTAG00000017103	7.99E-01	3.37E-01	-2.14E+00	6.28E-07	-2.37E+00	3.13E-08	-1.67E+00	1.20E-04	5.24E-01	7.68E-01
ENSBTAG00000019625	-2.43E-01	8.91E-01	-1.07E+00	2.09E-05	-3.09E+00	9.40E-34	-3.74E+00	7.32E-46	3.39E-01	6.97E-01
ENSBTAG00000007296	4.47E-01	9.78E-01	-6.31E-01	4.69E-01	-3.72E-01	7.72E-01	-9.48E-02	1.00E+00	-4.85E-01	9.50E-01
ENSBTAG00000005212	2.77E-01	1.00E+00	4.45E-01	5.22E-01	2.05E-01	9.65E-01	7.82E-01	1.42E-01	3.35E-01	1.00E+00
ENSBTAG00000005209	4.41E-02	1.00E+00	-7.98E-02	1.00E+00	3.37E-01	1.99E-01	4.33E-01	8.06E-02	-2.36E-02	1.00E+00
ENSBTAG00000042639	0.00E+00	1.00E+00	0.00E+00	1.00E+00	-5.13E-15	1.00E+00	0.00E+00	1.00E+00	0.00E+00	1.00E+00
ENSBTAG00000019014	-4.72E-01	4.17E-01	-1.67E+00	3.23E-09	-1.19E+00	1.35E-05	-6.06E-01	4.37E-02	3.43E-01	7.28E-01
ENSBTAG00000005313	4.01E-01	1.00E+00	8.83E-01	1.89E-01	3.07E+00	2.03E-09	2.28E+00	1.66E-05	-4.85E-02	1.00E+00
ENSBTAG00000008343	-3.21E+00	3.75E-01	-4.23E-01	1.00E+00	-3.21E+00	9.45E-02	-3.21E+00	9.92E-02	-3.21E+00	4.34E-01
ENSBTAG00000046790	-1.91E+00	8.23E-01	-1.91E+00	4.69E-01	-1.91E+00	4.28E-01	-1.91E+00	4.39E-01	-1.91E+00	8.00E-01
ENSBTAG00000002878	5.95E-01	8.00E-02	8.02E-01	6.12E-04	3.50E-01	2.15E-01	-4.58E-01	8.53E-02	5.11E-01	2.57E-01
ENSBTAG00000000476	8.36E-01	5.85E-04	1.23E+00	2.00E-09	1.33E+00	5.62E-11	8.25E-01	1.10E-04	4.39E-01	3.20E-01

ENSBTAG00000040199	1.94E-01	9.17E-01	5.21E-01	1.83E-02	-2.57E-01	3.58E-01	-2.68E-01	3.39E-01	-1.46E-01	1.00E+00
ENSBTAG00000012284	-2.93E-02	1.00E+00	1.57E+00	5.24E-07	-7.25E-01	3.75E-02	-1.32E+00	5.75E-05	-2.29E-01	1.00E+00
ENSBTAG00000012273	5.22E-01	5.76E-01	2.68E+00	9.43E-17	1.71E+00	1.54E-07	4.98E-01	2.51E-01	1.41E-02	1.00E+00
ENSBTAG00000012881	2.62E-01	7.68E-01	9.36E-01	7.54E-06	1.17E+00	1.21E-08	9.92E-01	2.30E-06	2.97E-01	6.50E-01
ENSBTAG00000042094	0.00E+00	1.00E+00	0.00E+00	1.00E+00	-5.13E-15	1.00E+00	0.00E+00	1.00E+00	0.00E+00	1.00E+00
ENSBTAG00000005660	-6.87E-02	1.00E+00	-2.28E-01	4.08E-01	-2.71E-02	1.00E+00	2.79E-01	2.71E-01	-2.73E-02	1.00E+00
ENSBTAG00000005658	4.94E-02	1.00E+00	2.07E+00	2.64E-21	2.45E+00	2.80E-29	2.45E+00	3.76E-29	-1.51E-02	1.00E+00
ENSBTAG00000005657	0.00E+00	1.00E+00	0.00E+00	1.00E+00	-5.13E-15	1.00E+00	0.00E+00	1.00E+00	0.00E+00	1.00E+00
ENSBTAG00000005652	-6.88E-04	1.00E+00	1.04E+00	1.27E-05	1.17E+00	6.28E-07	1.14E+00	2.04E-06	2.73E-01	8.00E-01
ENSBTAG00000040399	2.45E-01	1.00E+00	3.53E+00	1.69E-15	2.45E+00	4.95E-07	1.80E+00	1.06E-03	4.28E-01	1.00E+00
ENSBTAG00000040245	0.00E+00	1.00E+00	0.00E+00	1.00E+00	-5.13E-15	1.00E+00	0.00E+00	1.00E+00	0.00E+00	1.00E+00
ENSBTAG00000006920	1.48E-01	1.00E+00	5.49E-01	2.76E-02	9.02E-01	9.02E-05	9.88E-01	2.00E-05	4.43E-03	1.00E+00
ENSBTAG00000020106	-8.40E-02	1.00E+00	1.88E-01	5.13E-01	3.64E-01	9.87E-02	4.55E-01	3.29E-02	2.56E-03	1.00E+00
ENSBTAG00000019336	-3.34E-01	5.10E-01	-4.51E-01	4.73E-02	-7.66E-03	1.00E+00	-1.80E-01	5.97E-01	3.90E-01	4.10E-01
ENSBTAG00000011414	1.00E-01	1.00E+00	1.10E+00	2.45E-11	1.39E+00	1.17E-17	1.15E+00	2.12E-12	-1.08E-01	1.00E+00
ENSBTAG00000040304	3.72E-01	9.01E-01	-8.12E-01	7.89E-02	-8.39E-01	6.29E-02	-1.14E+00	9.47E-03	8.01E-01	3.20E-01
ENSBTAG00000016624	-1.67E+00	7.79E-01	-1.60E+00	4.08E-01	-1.81E+00	2.94E-01	-1.82E+00	3.01E-01	-8.69E-01	1.00E+00
ENSBTAG00000039011	-6.67E-01	1.00E+00	-1.89E+00	2.58E-01	-1.32E+00	4.17E-01	-3.88E+00	2.48E-02	-6.52E-01	1.00E+00
ENSBTAG00000038131	-5.37E-02	1.00E+00	-1.95E+00	7.94E-07	-3.65E+00	3.86E-19	-4.03E+00	3.92E-22	7.50E-01	3.93E-01
ENSBTAG00000017666	1.64E-01	1.00E+00	-2.26E+00	1.52E-11	-4.02E+00	9.48E-30	-4.18E+00	2.26E-31	6.42E-01	3.89E-01
ENSBTAG00000014460	3.64E-02	1.00E+00	-9.70E-02	9.62E-01	-2.50E-03	1.00E+00	9.58E-02	9.70E-01	5.53E-03	1.00E+00
ENSBTAG00000014459	-4.91E-02	1.00E+00	1.26E-01	1.00E+00	7.15E-01	4.88E-02	-2.50E-01	7.09E-01	-3.46E-01	8.34E-01
ENSBTAG00000014451	4.27E-01	3.75E-01	7.00E-01	3.70E-03	1.23E+00	5.46E-08	9.15E-01	1.00E-04	1.34E-01	1.00E+00
ENSBTAG00000014449	1.26E-01	1.00E+00	2.75E-01	5.02E-01	9.31E-03	1.00E+00	4.00E-02	1.00E+00	-8.42E-01	5.53E-02
ENSBTAG00000017450	3.61E-01	1.00E+00	-1.13E+00	5.07E-02	-3.28E-01	7.85E-01	5.82E-01	4.34E-01	1.01E+00	3.65E-01
ENSBTAG00000037951	-1.38E+00	8.06E-02	-1.67E+00	4.13E-03	-9.29E-01	9.98E-02	-2.39E-01	8.99E-01	-6.45E-01	7.26E-01
ENSBTAG00000042699	-2.25E+00	1.80E-01	-4.96E+00	1.12E-03	-2.40E+00	2.48E-02	-3.20E+00	6.52E-03	4.51E-01	1.00E+00
ENSBTAG00000046222	-2.67E+00	4.40E-02	-5.39E+00	1.33E-04	-5.39E+00	3.78E-05	-2.86E+00	3.63E-03	-8.54E-01	8.00E-01
ENSBTAG00000047590	-2.33E+00	8.66E-04	-2.44E+00	8.50E-05	-3.45E+00	1.96E-07	-1.99E+00	4.61E-04	-7.85E-01	5.72E-01
ENSBTAG00000032217	3.78E-01	7.59E-01	6.60E-03	1.00E+00	-4.57E-01	2.16E-01	-6.17E-01	7.41E-02	8.73E-01	6.28E-02
ENSBTAG00000010394	4.05E-01	6.99E-01	-1.99E-02	1.00E+00	-7.59E-01	2.79E-02	-7.78E-01	2.63E-02	2.84E-01	8.92E-01
ENSBTAG00000010397	0.00E+00	1.00E+00	0.00E+00	1.00E+00	-5.13E-15	1.00E+00	0.00E+00	1.00E+00	0.00E+00	1.00E+00
ENSBTAG00000006552	1.80E-01	1.00E+00	5.94E-01	7.71E-02	-1.19E-01	1.00E+00	-4.94E-01	1.96E-01	-5.68E-01	4.40E-01
ENSBTAG00000006548	8.33E-02	1.00E+00	-5.33E-01	2.23E-02	-8.60E-01	7.56E-05	-8.13E-01	2.45E-04	2.36E-01	8.00E-01
ENSBTAG00000012797	1.69E-01	1.00E+00	-1.98E-01	7.03E-01	-3.85E-01	2.52E-01	-5.11E-01	1.02E-01	-2.79E-02	1.00E+00

ENSBTAG00000000290	1.34E-01	1.00E+00	3.17E-01	3.15E-01	5.84E-01	2.31E-02	9.95E-01	3.62E-05	1.56E-01	1.00E+00
ENSBTAG00000045138	0.00E+00	1.00E+00	0.00E+00	1.00E+00	-5.13E-15	1.00E+00	0.00E+00	1.00E+00	0.00E+00	1.00E+00
ENSBTAG00000011598	3.86E+00	1.79E-01	5.19E+00	1.07E-03	7.20E+00	2.35E-08	5.58E+00	2.18E-04	1.95E+00	8.00E-01
ENSBTAG00000043803	0.00E+00	1.00E+00	0.00E+00	1.00E+00	-5.13E-15	1.00E+00	0.00E+00	1.00E+00	0.00E+00	1.00E+00
ENSBTAG00000023513	-1.99E-01	9.29E-01	-9.98E-01	6.44E-06	-9.06E-01	3.47E-05	-6.22E-01	7.15E-03	2.97E-01	6.65E-01
ENSBTAG00000016457	1.88E-01	9.44E-01	8.92E-01	2.34E-05	-1.12E-01	8.85E-01	-1.10E-01	9.00E-01	-2.13E-01	8.34E-01
ENSBTAG00000001969	8.51E-01	6.17E-01	3.31E+00	2.27E-08	1.82E+00	2.68E-03	1.06E+00	1.29E-01	2.86E-01	1.00E+00
ENSBTAG00000001968	-4.98E-02	1.00E+00	2.90E-01	3.03E-01	4.12E-01	9.68E-02	-9.49E-02	9.73E-01	-8.84E-02	1.00E+00
ENSBTAG00000013219	5.68E-03	1.00E+00	3.00E-01	1.00E+00	-1.74E-01	1.00E+00	7.54E-01	5.47E-01	1.96E-01	1.00E+00
ENSBTAG00000005946	-3.25E-01	1.00E+00	-1.48E+00	2.02E-02	-6.18E-01	4.32E-01	4.61E-01	6.21E-01	-2.98E-01	1.00E+00
ENSBTAG00000002463	-7.75E-02	1.00E+00	-2.89E-01	4.59E-01	-4.19E-01	2.07E-01	-4.42E-02	1.00E+00	-5.68E-01	3.12E-01
ENSBTAG00000002462	-9.89E-02	1.00E+00	5.69E-01	1.10E-02	5.02E-01	2.80E-02	2.00E-01	5.47E-01	-4.66E-01	2.80E-01
ENSBTAG00000002460	-2.49E-01	8.22E-01	-3.75E-01	1.34E-01	2.28E-01	4.49E-01	3.10E-01	2.51E-01	-4.67E-01	2.76E-01
ENSBTAG00000002767	-3.81E-01	8.23E-01	-5.02E-01	2.65E-01	-2.25E-01	7.66E-01	-2.57E-01	6.94E-01	-1.52E-01	1.00E+00
ENSBTAG00000010485	-3.09E-01	6.56E-01	-8.51E-01	1.55E-04	-3.26E-01	2.40E-01	-3.10E-01	2.85E-01	-8.46E-02	1.00E+00
ENSBTAG00000037393	3.46E-03	1.00E+00	-9.04E-03	1.00E+00	3.22E-01	4.79E-01	2.69E-01	6.18E-01	-2.86E-01	9.06E-01
ENSBTAG00000032114	9.39E-01	9.42E-01	-1.22E+00	6.14E-01	9.56E-01	5.38E-01	-6.77E-01	8.88E-01	-3.21E+00	4.34E-01
ENSBTAG00000009235	-2.11E-01	1.00E+00	-3.98E-01	4.43E-01	-2.16E-01	8.09E-01	-7.25E-01	8.48E-02	-2.45E-01	1.00E+00
ENSBTAG00000009232	1.82E-01	1.00E+00	4.56E-01	1.26E-01	5.71E-01	4.10E-02	7.37E-01	6.38E-03	-5.98E-02	1.00E+00
ENSBTAG00000043271	3.23E+00	3.75E-01	0.00E+00	1.00E+00	3.42E+00	7.48E-02	0.00E+00	1.00E+00	3.21E+00	4.34E-01
ENSBTAG00000012463	-2.50E-01	1.00E+00	1.08E+00	2.89E-03	8.40E-01	2.65E-02	1.00E+00	6.90E-03	2.83E-01	9.76E-01
ENSBTAG00000011377	-3.83E-01	1.00E+00	7.88E-02	1.00E+00	-1.82E+00	2.93E-01	-1.07E+00	5.89E-01	-8.93E-01	1.00E+00
ENSBTAG00000045258	0.00E+00	1.00E+00	0.00E+00	1.00E+00	-5.13E-15	1.00E+00	0.00E+00	1.00E+00	0.00E+00	1.00E+00
ENSBTAG00000036933	0.00E+00	1.00E+00	0.00E+00	1.00E+00	-5.13E-15	1.00E+00	0.00E+00	1.00E+00	0.00E+00	1.00E+00
ENSBTAG00000021910	-1.77E-02	1.00E+00	-9.93E-02	1.00E+00	4.11E-02	1.00E+00	4.04E-01	3.71E-01	-2.25E-02	1.00E+00
ENSBTAG00000042772	0.00E+00	1.00E+00	0.00E+00	1.00E+00	-5.13E-15	1.00E+00	0.00E+00	1.00E+00	0.00E+00	1.00E+00
ENSBTAG00000044913	-1.67E+00	7.77E-01	-3.59E+00	6.63E-02	-3.59E+00	4.51E-02	-5.56E-01	9.24E-01	-3.59E+00	3.09E-01
ENSBTAG00000043126	0.00E+00	1.00E+00	0.00E+00	1.00E+00	-5.13E-15	1.00E+00	0.00E+00	1.00E+00	0.00E+00	1.00E+00
ENSBTAG00000044291	0.00E+00	1.00E+00	0.00E+00	1.00E+00	-5.13E-15	1.00E+00	1.76E+00	4.97E-01	0.00E+00	1.00E+00
ENSBTAG00000029452	0.00E+00	1.00E+00	0.00E+00	1.00E+00	-5.13E-15	1.00E+00	0.00E+00	1.00E+00	0.00E+00	1.00E+00
ENSBTAG00000031891	4.82E-01	1.00E+00	-4.64E-01	7.37E-01	-3.41E+00	2.20E-04	-2.26E+00	6.72E-03	1.54E+00	8.57E-02
ENSBTAG00000046279	0.00E+00	1.00E+00	0.00E+00	1.00E+00	-5.13E-15	1.00E+00	1.77E+00	4.97E-01	3.22E+00	4.34E-01
ENSBTAG00000012401	-5.42E-03	1.00E+00	-2.03E-02	1.00E+00	-3.92E-02	1.00E+00	6.19E-02	1.00E+00	-1.29E+00	4.46E-03
ENSBTAG00000047651	0.00E+00	1.00E+00	0.00E+00	1.00E+00	-5.13E-15	1.00E+00	0.00E+00	1.00E+00	0.00E+00	1.00E+00
ENSBTAG00000031802	-2.62E-01	1.00E+00	6.64E-02	1.00E+00	3.93E-01	8.31E-01	9.03E-01	3.01E-01	-6.89E-01	8.46E-01

ENSBTAG00000021987	0.00E+00	1.00E+00	0.00E+00	1.00E+00	-5.13E-15	1.00E+00	0.00E+00	1.00E+00	0.00E+00	1.00E+00
ENSBTAG00000012023	0.00E+00	1.00E+00	0.00E+00	1.00E+00	-5.13E-15	1.00E+00	0.00E+00	1.00E+00	0.00E+00	1.00E+00
ENSBTAG00000037965	0.00E+00	1.00E+00	0.00E+00	1.00E+00	-5.13E-15	1.00E+00	0.00E+00	1.00E+00	0.00E+00	1.00E+00
ENSBTAG00000023814	8.66E-02	1.00E+00	9.95E-01	7.88E-02	9.80E-01	8.31E-02	6.78E-01	3.04E-01	-1.08E+00	3.39E-01
ENSBTAG00000020073	-4.89E-01	7.03E-01	-1.62E+00	3.48E-05	-8.38E-01	3.84E-02	-5.60E-01	2.24E-01	-7.39E-01	3.62E-01
ENSBTAG00000018994	-9.22E-01	6.66E-01	-1.29E+00	9.56E-02	-9.26E-01	2.77E-01	-1.60E+00	3.39E-02	2.81E-01	1.00E+00
ENSBTAG00000010874	-1.08E+00	2.40E-01	-1.74E+00	3.57E-03	-9.01E-01	1.16E-01	-1.56E+00	5.97E-03	-1.01E+00	3.81E-01
ENSBTAG00000019376	9.94E-01	1.87E-03	3.66E+00	6.26E-41	3.84E+00	1.76E-44	4.05E+00	1.96E-48	1.23E+00	1.48E-04
ENSBTAG00000045646	0.00E+00	1.00E+00	0.00E+00	1.00E+00	-5.13E-15	1.00E+00	0.00E+00	1.00E+00	0.00E+00	1.00E+00
ENSBTAG00000006174	0.00E+00	1.00E+00	0.00E+00	1.00E+00	3.71E+00	4.08E-02	3.40E+00	8.38E-02	0.00E+00	1.00E+00
ENSBTAG00000017490	-1.59E-02	1.00E+00	5.02E-02	1.00E+00	-1.02E-02	1.00E+00	6.08E-01	1.13E-02	5.07E-01	2.38E-01
ENSBTAG00000011742	1.85E-02	1.00E+00	5.47E-02	1.00E+00	4.73E-02	1.00E+00	2.71E-01	7.45E-01	-3.68E-01	8.89E-01
ENSBTAG00000031795	0.00E+00	1.00E+00	0.00E+00	1.00E+00	-5.13E-15	1.00E+00	0.00E+00	1.00E+00	0.00E+00	1.00E+00
ENSBTAG00000005386	-1.05E-01	1.00E+00	-1.32E+00	7.18E-07	-1.91E+00	4.90E-13	-1.13E+00	3.27E-05	2.20E-01	9.56E-01
ENSBTAG00000010550	-1.89E+00	1.33E-01	-1.25E+00	1.57E-01	4.06E-01	7.75E-01	9.84E-01	1.82E-01	-5.60E-01	9.56E-01
ENSBTAG00000007394	6.32E-01	1.30E-01	1.94E+00	1.85E-14	2.45E+00	2.90E-22	1.66E+00	7.31E-11	-2.64E-01	8.54E-01
ENSBTAG00000016323	-2.00E-01	1.00E+00	-6.77E-01	5.57E-01	-1.34E+00	1.41E-01	-5.38E-01	6.74E-01	-2.59E+00	1.01E-01
ENSBTAG00000019060	4.82E-01	8.76E-01	8.76E-01	1.07E-01	6.26E-02	1.00E+00	-2.06E-01	1.00E+00	-1.44E-01	1.00E+00
ENSBTAG00000004154	3.10E-02	1.00E+00	5.65E-01	1.77E-01	1.19E+00	6.65E-04	1.15E+00	1.30E-03	-1.86E-01	1.00E+00
ENSBTAG00000001403	-5.46E-02	1.00E+00	-6.87E-01	9.70E-03	-1.08E-01	9.70E-01	1.56E-01	7.99E-01	1.44E-02	1.00E+00
ENSBTAG00000043872	0.00E+00	1.00E+00	0.00E+00	1.00E+00	-5.13E-15	1.00E+00	0.00E+00	1.00E+00	0.00E+00	1.00E+00
ENSBTAG00000013938	-2.39E-01	1.00E+00	-3.95E-01	4.01E-01	-4.94E-01	2.36E-01	-5.34E-02	1.00E+00	1.05E-01	1.00E+00
ENSBTAG00000045411	0.00E+00	1.00E+00	0.00E+00	1.00E+00	-5.13E-15	1.00E+00	0.00E+00	1.00E+00	0.00E+00	1.00E+00
ENSBTAG00000048011	-4.40E-01	9.42E-01	-1.30E+00	2.68E-02	-5.60E-01	4.16E-01	-6.01E-01	3.76E-01	-1.06E+00	3.46E-01
ENSBTAG00000042535	0.00E+00	1.00E+00	0.00E+00	1.00E+00	-5.13E-15	1.00E+00	0.00E+00	1.00E+00	0.00E+00	1.00E+00
ENSBTAG00000045991	-2.59E-01	1.00E+00	2.80E-01	9.22E-01	-4.46E-01	6.88E-01	-9.41E-01	2.38E-01	1.45E-01	1.00E+00
ENSBTAG00000039442	1.28E-02	1.00E+00	-4.42E-01	4.37E-02	-7.87E-01	8.21E-05	-1.06E+00	9.16E-08	-2.51E-01	7.65E-01
ENSBTAG00000018228	8.04E-01	1.00E+00	7.78E-02	1.00E+00	-1.91E+00	4.28E-01	1.13E+00	6.66E-01	-1.91E+00	8.00E-01
ENSBTAG00000010679	1.99E+00	5.76E-01	1.77E+00	3.26E-01	-1.91E+00	4.28E-01	1.49E+00	4.56E-01	-1.91E+00	8.00E-01
ENSBTAG00000006415	5.16E-03	1.00E+00	6.91E-02	1.00E+00	6.33E-01	9.81E-01	-1.53E-01	1.00E+00	1.18E-02	1.00E+00
ENSBTAG00000006409	-3.67E-01	8.23E-01	6.68E-01	5.37E-02	-6.17E-03	1.00E+00	-8.94E-01	1.34E-02	-4.36E-02	1.00E+00
ENSBTAG00000013142	4.34E-01	3.75E-01	1.62E+00	1.72E-12	1.24E+00	8.14E-08	8.56E-01	4.14E-04	-2.43E-01	8.31E-01
ENSBTAG00000013141	-9.45E-02	1.00E+00	2.21E+00	2.79E-09	8.77E-01	3.60E-02	3.09E-01	6.65E-01	5.18E-01	7.13E-01
ENSBTAG00000043157	1.91E+00	8.23E-01	0.00E+00	1.00E+00	1.78E+00	4.79E-01	3.40E+00	8.33E-02	1.91E+00	8.00E-01
ENSBTAG00000043452	0.00E+00	1.00E+00	0.00E+00	1.00E+00	-5.13E-15	1.00E+00	0.00E+00	1.00E+00	0.00E+00	1.00E+00

ENSBTAG00000047144	7.96E-01	1.00E+00	-1.91E+00	4.69E-01	-1.91E+00	4.28E-01	1.11E+00	6.72E-01	8.04E-01	1.00E+00
ENSBTAG00000005871	1.33E+00	9.44E-02	1.04E+00	8.26E-02	7.88E-01	2.29E-01	7.40E-01	2.82E-01	1.74E-01	1.00E+00
ENSBTAG00000030029	0.00E+00	1.00E+00	0.00E+00	1.00E+00	-5.13E-15	1.00E+00	0.00E+00	1.00E+00	0.00E+00	1.00E+00
ENSBTAG00000018727	-2.11E-01	1.00E+00	-1.04E+00	9.46E-05	-1.96E+00	5.04E-14	-1.99E+00	2.80E-14	5.04E-01	4.02E-01
ENSBTAG00000031704	1.35E-02	1.00E+00	8.81E-01	8.52E-01	-1.91E+00	4.28E-01	1.12E+00	6.71E-01	7.13E-03	1.00E+00
ENSBTAG00000012708	-2.76E-01	1.00E+00	-4.59E-01	4.21E-01	-1.14E+00	9.58E-03	-8.89E-01	5.20E-02	-9.88E-02	1.00E+00
ENSBTAG00000031709	1.98E-03	1.00E+00	4.70E-01	2.51E-02	4.82E-01	2.03E-02	4.64E-01	3.00E-02	-2.89E-01	6.15E-01
ENSBTAG00000011427	5.18E-01	1.00E+00	2.09E+00	7.64E-02	7.24E-01	8.24E-01	2.07E+00	7.45E-02	-2.70E+00	6.01E-01
ENSBTAG00000031618	-4.48E-01	1.00E+00	1.00E-01	1.00E+00	8.86E-03	1.00E+00	8.46E-01	4.47E-01	3.74E-01	1.00E+00
ENSBTAG00000011423	-7.93E-01	1.00E+00	8.15E-02	1.00E+00	1.47E+00	3.01E-01	-1.77E-01	1.00E+00	1.45E+00	7.47E-01
ENSBTAG00000035710	7.18E-03	1.00E+00	-3.10E-01	1.00E+00	3.73E-01	9.30E-01	6.65E-01	5.84E-01	-9.48E-01	8.41E-01
ENSBTAG00000043293	0.00E+00	1.00E+00	0.00E+00	1.00E+00	1.78E+00	4.79E-01	0.00E+00	1.00E+00	0.00E+00	1.00E+00
ENSBTAG00000011139	1.93E-01	1.00E+00	-3.85E-01	4.99E-01	-1.87E+00	1.47E-05	-6.58E-01	1.57E-01	-1.06E-01	1.00E+00
ENSBTAG00000044337	0.00E+00	1.00E+00	0.00E+00	1.00E+00	-5.13E-15	1.00E+00	0.00E+00	1.00E+00	0.00E+00	1.00E+00
ENSBTAG00000017180	1.91E+00	8.23E-01	0.00E+00	1.00E+00	1.78E+00	4.79E-01	1.76E+00	4.97E-01	0.00E+00	1.00E+00
ENSBTAG00000006975	4.16E-01	1.00E+00	-8.20E-01	6.70E-01	-6.98E-01	7.25E-01	-1.86E-01	1.00E+00	-5.23E-01	1.00E+00
ENSBTAG00000044737	0.00E+00	1.00E+00	0.00E+00	1.00E+00	-5.13E-15	1.00E+00	0.00E+00	1.00E+00	0.00E+00	1.00E+00
ENSBTAG00000018312	3.12E-02	1.00E+00	-1.12E-02	1.00E+00	-1.58E-01	8.02E-01	1.57E-01	8.08E-01	-3.96E-02	1.00E+00
ENSBTAG00000043587	0.00E+00	1.00E+00	0.00E+00	1.00E+00	-5.13E-15	1.00E+00	0.00E+00	1.00E+00	0.00E+00	1.00E+00
ENSBTAG00000027213	1.18E-01	1.00E+00	3.02E-01	4.56E-01	3.69E-01	3.15E-01	3.85E-01	2.96E-01	-4.14E-01	6.21E-01
ENSBTAG00000025847	2.71E+00	5.76E-01	1.98E+00	4.43E-01	-5.13E-15	1.00E+00	0.00E+00	1.00E+00	0.00E+00	1.00E+00
ENSBTAG00000011051	-2.08E-02	1.00E+00	-1.47E+00	3.24E-02	-1.02E+00	1.25E-01	-7.95E-01	2.65E-01	-5.85E-01	8.02E-01
ENSBTAG00000043595	0.00E+00	1.00E+00	0.00E+00	1.00E+00	-5.13E-15	1.00E+00	0.00E+00	1.00E+00	0.00E+00	1.00E+00
ENSBTAG00000018489	-4.59E-01	1.00E+00	-2.36E+00	9.92E-02	-1.28E+00	3.39E-01	-9.47E-01	5.11E-01	-2.42E+00	3.79E-01
ENSBTAG00000047250	-1.01E+00	6.48E-02	-1.33E+00	6.96E-04	-2.03E+00	1.47E-07	-2.85E+00	1.67E-12	3.80E-01	8.15E-01
ENSBTAG00000004713	1.53E-01	1.00E+00	-2.54E-03	1.00E+00	4.08E-01	3.30E-01	1.14E+00	3.63E-04	3.68E-01	8.00E-01
ENSBTAG00000016228	-1.06E-01	1.00E+00	-1.05E+00	2.19E-06	1.05E+00	1.11E-06	8.03E-01	3.41E-04	-3.68E-01	5.26E-01
ENSBTAG00000046104	9.56E-01	8.56E-01	-3.58E+00	6.72E-02	-1.02E+00	6.07E-01	-1.05E+00	5.99E-01	5.70E-03	1.00E+00
ENSBTAG00000001274	-2.81E-01	8.23E-01	-1.21E+00	8.09E-07	-2.28E+00	2.66E-20	-2.58E+00	7.66E-25	-6.40E-02	1.00E+00
ENSBTAG00000017322	0.00E+00	1.00E+00	0.00E+00	1.00E+00	2.55E+00	2.55E-01	2.53E+00	2.71E-01	0.00E+00	1.00E+00
ENSBTAG00000017416	5.86E-01	2.00E-01	1.60E+00	1.01E-09	1.79E+00	6.71E-12	1.73E+00	4.57E-11	1.29E-01	1.00E+00
ENSBTAG00000045275	0.00E+00	1.00E+00	0.00E+00	1.00E+00	-5.13E-15	1.00E+00	0.00E+00	1.00E+00	0.00E+00	1.00E+00
ENSBTAG00000008078	-2.78E-01	1.00E+00	-4.71E-01	3.74E-01	-5.90E-01	2.04E-01	6.27E-01	1.27E-01	-2.08E-01	1.00E+00
ENSBTAG00000005862	1.22E-01	1.00E+00	1.94E-01	8.55E-01	7.15E-01	5.86E-02	8.54E-01	2.14E-02	-5.61E-01	5.50E-01
ENSBTAG00000029916	0.00E+00	1.00E+00	0.00E+00	1.00E+00	-5.13E-15	1.00E+00	0.00E+00	1.00E+00	0.00E+00	1.00E+00

ENSBTAG00000029929	0.00E+00	1.00E+00	0.00E+00	1.00E+00	-5.13E-15	1.00E+00	0.00E+00	1.00E+00	0.00E+00	1.00E+00
ENSBTAG00000031572	5.70E-02	1.00E+00	-4.03E-01	2.42E-01	1.85E-01	7.34E-01	2.61E-01	5.14E-01	-9.56E-03	1.00E+00
ENSBTAG00000047247	1.45E+00	7.46E-01	9.84E-01	6.48E-01	-1.42E-01	1.00E+00	-1.65E-01	1.00E+00	2.01E+00	3.99E-01
ENSBTAG00000015150	2.72E+00	5.76E-01	4.63E+00	3.84E-03	4.37E+00	7.45E-03	1.76E+00	4.98E-01	2.71E+00	6.01E-01
ENSBTAG00000014960	-4.64E-01	9.17E-01	-1.21E+00	3.11E-02	-1.27E-01	1.00E+00	1.44E-02	1.00E+00	-7.00E-01	6.61E-01
ENSBTAG00000042564	0.00E+00	1.00E+00	0.00E+00	1.00E+00	-5.13E-15	1.00E+00	0.00E+00	1.00E+00	0.00E+00	1.00E+00
ENSBTAG00000003135	1.69E+00	7.70E-01	7.51E-02	1.00E+00	6.39E-01	9.79E-01	-1.42E-01	1.00E+00	-1.91E+00	8.00E-01
ENSBTAG00000038395	0.00E+00	1.00E+00	1.98E+00	4.43E-01	-5.13E-15	1.00E+00	0.00E+00	1.00E+00	0.00E+00	1.00E+00
ENSBTAG00000009263	3.17E-02	1.00E+00	3.79E-01	4.53E-01	-2.29E-01	7.78E-01	-6.20E-01	1.44E-01	2.33E-03	1.00E+00
ENSBTAG00000014713	6.51E-01	3.75E-01	-5.62E-02	1.00E+00	-6.07E-01	1.49E-01	-8.72E-01	2.60E-02	-3.11E-01	9.17E-01
ENSBTAG00000027795	7.13E-02	1.00E+00	4.94E-01	5.86E-02	7.24E-01	2.71E-03	7.59E-01	1.89E-03	-3.45E-02	1.00E+00
ENSBTAG00000009336	0.00E+00	1.00E+00	0.00E+00	1.00E+00	-5.13E-15	1.00E+00	0.00E+00	1.00E+00	0.00E+00	1.00E+00
ENSBTAG00000004126	5.98E-01	8.92E-01	1.28E+00	4.29E-02	4.93E-01	6.46E-01	9.22E-02	1.00E+00	1.13E-01	1.00E+00
ENSBTAG00000031561	-2.66E-01	7.65E-01	-8.45E-01	8.74E-05	-1.04E+00	8.23E-07	-9.33E-01	1.44E-05	-4.30E-01	3.46E-01
ENSBTAG00000047963	0.00E+00	1.00E+00	0.00E+00	1.00E+00	-5.13E-15	1.00E+00	0.00E+00	1.00E+00	0.00E+00	1.00E+00
ENSBTAG00000047570	0.00E+00	1.00E+00	0.00E+00	1.00E+00	-5.13E-15	1.00E+00	0.00E+00	1.00E+00	0.00E+00	1.00E+00
ENSBTAG00000044346	0.00E+00	1.00E+00	0.00E+00	1.00E+00	-5.13E-15	1.00E+00	0.00E+00	1.00E+00	0.00E+00	1.00E+00
ENSBTAG00000005230	-2.58E+00	1.94E-02	-1.36E+00	9.65E-02	-5.80E+00	2.14E-06	-1.66E+00	3.43E-02	-6.99E-01	8.00E-01
ENSBTAG00000045446	0.00E+00	1.00E+00	0.00E+00	1.00E+00	-5.13E-15	1.00E+00	0.00E+00	1.00E+00	0.00E+00	1.00E+00
ENSBTAG00000024643	2.17E-01	1.00E+00	2.83E+00	5.25E-14	3.10E+00	1.83E-16	2.67E+00	1.25E-12	3.02E-01	9.70E-01
ENSBTAG00000009012	0.00E+00	1.00E+00	0.00E+00	1.00E+00	-5.13E-15	1.00E+00	0.00E+00	1.00E+00	0.00E+00	1.00E+00
ENSBTAG00000042513	0.00E+00	1.00E+00	0.00E+00	1.00E+00	-5.13E-15	1.00E+00	0.00E+00	1.00E+00	0.00E+00	1.00E+00
ENSBTAG00000042623	-1.91E+00	8.23E-01	8.82E-01	8.52E-01	-1.91E+00	4.28E-01	-1.42E-01	1.00E+00	6.47E-03	1.00E+00
ENSBTAG00000003851	2.76E+00	5.72E-27	3.25E+00	5.20E-37	2.61E+00	1.57E-25	2.10E+00	2.68E-17	-2.70E-01	8.00E-01
ENSBTAG00000018009	-6.11E-01	8.85E-01	8.60E-01	1.98E-01	3.44E-01	8.07E-01	-4.43E-01	7.09E-01	-5.87E-01	8.85E-01
ENSBTAG00000012120	2.38E+00	1.21E-18	2.37E+00	1.88E-19	1.38E+00	1.56E-07	1.24E+00	4.01E-06	7.28E-02	1.00E+00
ENSBTAG00000018471	5.19E-01	2.61E-01	1.27E+00	4.82E-07	1.51E+00	1.46E-09	1.68E+00	2.16E-11	2.54E-01	8.39E-01
ENSBTAG00000018465	1.52E-01	1.00E+00	1.54E+00	7.06E-05	3.17E-01	6.48E-01	3.36E-01	6.20E-01	-1.68E-02	1.00E+00
ENSBTAG00000037274	0.00E+00	1.00E+00	0.00E+00	1.00E+00	-5.13E-15	1.00E+00	0.00E+00	1.00E+00	0.00E+00	1.00E+00
ENSBTAG00000044278	0.00E+00	1.00E+00	0.00E+00	1.00E+00	-5.13E-15	1.00E+00	0.00E+00	1.00E+00	0.00E+00	1.00E+00
ENSBTAG00000009622	9.40E-01	9.43E-01	1.85E+00	8.18E-02	9.59E-01	5.39E-01	-6.78E-01	8.89E-01	9.59E-01	9.00E-01
ENSBTAG00000013013	1.93E-01	8.62E-01	9.64E-01	2.31E-07	1.35E+00	1.55E-13	9.64E-01	2.68E-07	-6.99E-02	1.00E+00
ENSBTAG00000014318	8.50E-01	6.86E-02	2.42E+00	4.66E-14	2.21E+00	4.05E-12	1.61E+00	5.92E-07	3.49E-01	8.00E-01
ENSBTAG00000005203	4.33E-02	1.00E+00	-1.31E+00	1.27E-02	-1.29E+00	1.10E-02	-1.10E-01	1.00E+00	1.38E-01	1.00E+00
ENSBTAG00000008307	-1.44E-01	1.00E+00	7.06E-01	1.68E-01	1.37E+00	1.52E-03	3.61E-02	1.00E+00	2.07E-02	1.00E+00

ENSBTAG00000045003	0.00E+00	1.00E+00	0.00E+00	1.00E+00	-5.13E-15	1.00E+00	0.00E+00	1.00E+00	0.00E+00	1.00E+00
ENSBTAG00000042550	0.00E+00	1.00E+00	0.00E+00	1.00E+00	-5.13E-15	1.00E+00	0.00E+00	1.00E+00	0.00E+00	1.00E+00
ENSBTAG00000044667	0.00E+00	1.00E+00	0.00E+00	1.00E+00	-5.13E-15	1.00E+00	0.00E+00	1.00E+00	0.00E+00	1.00E+00
ENSBTAG00000002075	-2.41E-01	1.00E+00	9.31E-02	1.00E+00	-4.09E-01	9.78E-01	6.14E-01	7.11E-01	5.51E-01	1.00E+00
ENSBTAG00000024623	2.82E-01	8.77E-01	-6.03E-01	7.82E-02	-9.35E-01	2.85E-03	-1.21E+00	1.26E-04	3.06E-01	8.00E-01
ENSBTAG00000006147	-1.40E+00	7.70E-01	-4.43E-01	9.62E-01	-3.93E-01	9.84E-01	-1.10E+00	4.80E-01	-2.21E+00	4.81E-01
ENSBTAG00000006142	-8.26E-02	1.00E+00	5.52E-01	8.89E-03	6.28E-01	2.24E-03	7.20E-01	4.54E-04	-2.32E-01	8.00E-01
ENSBTAG00000013439	-7.96E-01	2.59E-02	-2.16E+00	7.31E-16	-3.08E+00	5.44E-29	-3.60E+00	8.31E-37	-3.90E-01	6.13E-01
ENSBTAG00000046896	0.00E+00	1.00E+00	0.00E+00	1.00E+00	-5.13E-15	1.00E+00	0.00E+00	1.00E+00	0.00E+00	1.00E+00
ENSBTAG00000011631	0.00E+00	1.00E+00	0.00E+00	1.00E+00	1.78E+00	4.79E-01	0.00E+00	1.00E+00	0.00E+00	1.00E+00
ENSBTAG00000047341	1.54E-01	1.00E+00	2.49E-01	4.76E-01	9.16E-01	1.10E-04	1.18E+00	4.44E-07	5.96E-02	1.00E+00
ENSBTAG00000016961	7.17E-01	1.46E-01	3.64E+00	9.63E-31	3.42E+00	6.80E-28	1.67E+00	4.48E-08	-9.74E-02	1.00E+00
ENSBTAG00000046577	0.00E+00	1.00E+00	0.00E+00	1.00E+00	-5.13E-15	1.00E+00	0.00E+00	1.00E+00	0.00E+00	1.00E+00
ENSBTAG00000001465	-1.00E+00	2.75E-02	-6.22E-01	1.05E-01	-4.86E-01	2.34E-01	-5.78E-01	1.43E-01	4.16E-01	7.42E-01
ENSBTAG00000033740	-8.91E-02	1.00E+00	-7.81E-02	1.00E+00	-1.41E-01	1.00E+00	2.89E-01	8.89E-01	1.43E-02	1.00E+00
ENSBTAG00000004564	-1.66E-02	1.00E+00	-6.99E-01	1.41E-02	-9.20E-01	7.44E-04	-1.00E+00	2.68E-04	-2.64E-01	8.44E-01
ENSBTAG00000043899	0.00E+00	1.00E+00	0.00E+00	1.00E+00	-5.13E-15	1.00E+00	0.00E+00	1.00E+00	0.00E+00	1.00E+00
ENSBTAG00000019670	3.44E-01	9.62E-01	-2.13E-02	1.00E+00	-7.58E-01	1.22E-01	-9.20E-01	5.53E-02	-3.59E-01	9.23E-01
ENSBTAG00000011569	-1.32E-01	1.00E+00	-1.22E+00	1.71E-04	-3.07E+00	4.75E-21	-3.29E+00	1.84E-23	4.87E-01	6.01E-01
ENSBTAG00000013378	6.07E-03	1.00E+00	5.99E-01	9.45E-01	7.17E-01	8.28E-01	-9.46E-01	8.04E-01	-2.71E+00	6.01E-01
ENSBTAG00000042814	0.00E+00	1.00E+00	2.79E+00	2.10E-01	-5.13E-15	1.00E+00	0.00E+00	1.00E+00	0.00E+00	1.00E+00
ENSBTAG00000010385	-1.91E+00	8.23E-01	-1.91E+00	4.69E-01	-1.91E+00	4.28E-01	1.49E+00	4.55E-01	-1.91E+00	8.00E-01
ENSBTAG00000023447	-1.91E+00	8.23E-01	-1.91E+00	4.69E-01	-1.91E+00	4.28E-01	6.26E-01	9.94E-01	-1.91E+00	8.00E-01
ENSBTAG00000001728	1.92E+00	8.23E-01	1.98E+00	4.43E-01	3.43E+00	7.35E-02	3.03E+00	1.50E-01	3.21E+00	4.34E-01
ENSBTAG00000021703	4.47E-01	6.98E-01	-3.28E-01	5.31E-01	-1.19E+00	1.08E-03	-5.96E-01	1.51E-01	-4.50E-01	7.35E-01
ENSBTAG00000015837	0.00E+00	1.00E+00	0.00E+00	1.00E+00	-5.13E-15	1.00E+00	0.00E+00	1.00E+00	0.00E+00	1.00E+00
ENSBTAG00000046051	0.00E+00	1.00E+00	0.00E+00	1.00E+00	-5.13E-15	1.00E+00	0.00E+00	1.00E+00	0.00E+00	1.00E+00
ENSBTAG00000031397	0.00E+00	1.00E+00	0.00E+00	1.00E+00	-5.13E-15	1.00E+00	0.00E+00	1.00E+00	0.00E+00	1.00E+00
ENSBTAG00000011192	0.00E+00	1.00E+00	0.00E+00	1.00E+00	-5.13E-15	1.00E+00	0.00E+00	1.00E+00	0.00E+00	1.00E+00
ENSBTAG00000020990	0.00E+00	1.00E+00	0.00E+00	1.00E+00	-5.13E-15	1.00E+00	0.00E+00	1.00E+00	0.00E+00	1.00E+00
ENSBTAG00000009848	0.00E+00	1.00E+00	0.00E+00	1.00E+00	-5.13E-15	1.00E+00	0.00E+00	1.00E+00	0.00E+00	1.00E+00
ENSBTAG00000011267	1.32E+00	9.30E-01	2.07E+00	1.95E-01	1.82E+00	2.78E-01	1.78E+00	3.03E-01	-1.91E+00	8.00E-01
ENSBTAG00000038069	-3.58E+00	2.33E-01	-7.93E-01	7.87E-01	-1.02E+00	6.06E-01	-1.82E-01	1.00E+00	-8.63E-01	1.00E+00
ENSBTAG00000023445	-9.78E-01	7.42E-05	1.99E-01	5.60E-01	-6.92E-01	2.16E-03	1.26E-01	8.49E-01	3.02E-01	6.67E-01
ENSBTAG00000001331	-1.11E+00	8.23E-01	-1.54E+00	2.72E-01	9.05E-01	3.73E-01	-3.97E-01	9.63E-01	-1.94E-01	1.00E+00

ENSBTAG00000031435	-9.03E-02	1.00E+00	9.88E-02	9.37E-01	3.33E-01	1.80E-01	4.53E-01	5.02E-02	-4.29E-01	3.14E-01
ENSBTAG00000009106	1.33E-01	1.00E+00	6.86E-01	1.21E-04	5.82E-01	1.33E-03	3.93E-01	4.90E-02	-2.41E-01	6.95E-01
ENSBTAG00000042474	0.00E+00	1.00E+00	0.00E+00	1.00E+00	-5.13E-15	1.00E+00	0.00E+00	1.00E+00	0.00E+00	1.00E+00
ENSBTAG00000009104	9.58E-01	7.83E-03	3.13E+00	4.15E-27	3.03E+00	7.66E-26	2.61E+00	7.03E-20	7.76E-01	9.88E-02
ENSBTAG00000018402	8.50E-01	3.73E-03	1.98E+00	2.37E-17	2.01E+00	6.37E-18	1.70E+00	3.87E-13	-2.47E-01	8.33E-01
ENSBTAG00000011150	-9.15E-02	1.00E+00	-1.90E-01	9.98E-01	1.65E-01	1.00E+00	5.00E-01	4.61E-01	-2.45E-01	1.00E+00
ENSBTAG00000011147	-8.09E-02	1.00E+00	-7.95E-01	4.54E-05	-1.31E+00	4.83E-12	-7.98E-01	4.95E-05	-2.87E-01	6.07E-01
ENSBTAG00000031346	2.57E-02	1.00E+00	1.36E+00	2.28E-01	-1.03E+00	6.05E-01	5.64E-01	8.49E-01	1.14E+00	7.78E-01
ENSBTAG00000005068	-3.13E-02	1.00E+00	-6.13E-01	5.91E-02	4.13E-01	2.29E-01	5.57E-01	7.99E-02	8.45E-02	1.00E+00
ENSBTAG00000007814	5.91E-03	1.00E+00	-1.24E-01	1.00E+00	2.47E-01	7.53E-01	5.30E-01	2.54E-01	4.39E-02	1.00E+00
ENSBTAG00000001893	1.02E-02	1.00E+00	9.62E-02	9.78E-01	-2.03E-01	5.66E-01	1.55E-01	7.52E-01	-1.39E-01	1.00E+00
ENSBTAG00000015163	4.23E-01	8.69E-01	2.33E+00	5.71E-08	2.12E+00	6.55E-07	2.03E+00	2.52E-06	-6.86E-01	5.60E-01
ENSBTAG00000019960	-8.06E-02	1.00E+00	8.73E-01	6.70E-03	1.14E+00	1.88E-04	1.09E+00	4.65E-04	-1.82E-01	1.00E+00
ENSBTAG00000012164	1.22E+00	9.00E-07	1.76E+00	1.03E-14	1.47E+00	1.01E-10	1.39E+00	1.37E-09	1.02E+00	3.54E-04
ENSBTAG00000042551	0.00E+00	1.00E+00	0.00E+00	1.00E+00	-5.13E-15	1.00E+00	0.00E+00	1.00E+00	0.00E+00	1.00E+00
ENSBTAG00000002395	1.06E+00	3.08E-05	2.60E+00	3.96E-30	9.27E-01	6.06E-05	9.31E-02	1.00E+00	-3.32E-01	6.44E-01
ENSBTAG00000048150	0.00E+00	1.00E+00	0.00E+00	1.00E+00	-5.13E-15	1.00E+00	0.00E+00	1.00E+00	0.00E+00	1.00E+00
ENSBTAG00000019704	2.43E-01	9.70E-01	-5.49E-01	9.71E-02	-6.10E-01	5.67E-02	4.87E-01	1.57E-01	1.67E-01	1.00E+00
ENSBTAG00000001721	-3.16E-01	8.23E-01	-8.78E-01	5.92E-03	-5.77E-01	8.92E-02	1.69E-01	8.49E-01	-2.29E-01	9.90E-01
ENSBTAG00000015008	5.18E-01	1.00E+00	2.49E+00	1.77E-02	2.22E+00	4.12E-02	2.41E+00	2.20E-02	8.88E-01	1.00E+00
ENSBTAG00000000456	-2.71E+00	5.76E-01	1.74E+00	1.95E-01	3.49E-01	1.00E+00	6.89E-01	8.59E-01	-7.91E-01	1.00E+00
ENSBTAG00000045633	5.95E-01	4.66E-01	-6.51E-01	1.18E-01	-2.97E-01	6.18E-01	7.70E-01	5.46E-02	9.78E-01	8.70E-02
ENSBTAG00000046008	7.52E-02	1.00E+00	8.81E-01	2.64E-03	7.65E-01	1.03E-02	3.09E-01	4.62E-01	-4.26E-01	6.28E-01
ENSBTAG00000042362	0.00E+00	1.00E+00	0.00E+00	1.00E+00	-5.13E-15	1.00E+00	2.54E+00	2.71E-01	0.00E+00	1.00E+00
ENSBTAG00000039334	-9.17E-01	6.41E-01	-9.34E-01	2.66E-01	-1.65E+00	2.89E-02	-2.60E+00	1.45E-03	-5.68E-01	9.02E-01
ENSBTAG00000025146	8.58E-02	1.00E+00	1.38E+00	2.43E-06	1.24E+00	2.25E-05	9.86E-01	1.37E-03	2.62E-01	9.40E-01
ENSBTAG00000014751	-1.91E+00	8.23E-01	-1.91E+00	4.69E-01	-1.91E+00	4.28E-01	-1.47E-01	1.00E+00	-1.91E+00	8.00E-01
ENSBTAG00000014749	-1.91E+00	8.23E-01	8.81E-01	8.52E-01	1.14E+00	6.46E-01	-1.48E-01	1.00E+00	1.69E+00	7.74E-01
ENSBTAG00000039176	0.00E+00	1.00E+00	0.00E+00	1.00E+00	-5.13E-15	1.00E+00	0.00E+00	1.00E+00	0.00E+00	1.00E+00
ENSBTAG00000005522	2.72E+00	5.76E-01	1.98E+00	4.43E-01	2.56E+00	2.54E-01	1.76E+00	4.97E-01	0.00E+00	1.00E+00
ENSBTAG00000022227	1.16E+00	1.47E-02	2.33E+00	1.41E-10	2.25E+00	5.08E-10	1.49E+00	6.52E-05	-3.34E-01	9.06E-01
ENSBTAG00000031306	-1.71E-01	1.00E+00	-2.42E-01	1.00E+00	-2.97E-01	9.76E-01	-3.85E-01	8.67E-01	-2.16E-01	1.00E+00
ENSBTAG00000011986	2.05E+00	4.32E-12	4.57E+00	5.04E-50	3.59E+00	2.46E-34	1.90E+00	2.66E-11	-3.52E-01	7.87E-01
ENSBTAG00000007331	4.42E-01	6.32E-01	4.78E-01	2.14E-01	7.32E-01	2.98E-02	8.20E-01	1.45E-02	-6.85E-01	2.95E-01
ENSBTAG00000043802	0.00E+00	1.00E+00	0.00E+00	1.00E+00	-5.13E-15	1.00E+00	0.00E+00	1.00E+00	0.00E+00	1.00E+00

ENSBTAG00000047669	0.00E+00	1.00E+00	0.00E+00	1.00E+00	-5.13E-15	1.00E+00	0.00E+00	1.00E+00	0.00E+00	1.00E+00
ENSBTAG00000007933	-9.66E-03	1.00E+00	-1.86E+00	6.26E-07	3.84E-01	4.42E-01	7.32E-01	6.87E-02	8.72E-01	1.63E-01
ENSBTAG00000031178	1.73E-01	1.00E+00	-7.19E-01	1.45E-02	-1.81E+00	7.92E-11	-1.31E+00	2.71E-06	-3.90E-02	1.00E+00
ENSBTAG00000011584	9.47E-01	1.28E-02	1.76E+00	8.56E-10	1.09E+00	2.23E-04	1.35E+00	4.11E-06	-4.86E-01	6.01E-01
ENSBTAG00000009154	2.90E-01	6.59E-01	1.01E+00	9.19E-07	9.06E-01	1.19E-05	4.75E-01	4.15E-02	-1.18E-01	1.00E+00
ENSBTAG00000011116	-5.25E-01	7.23E-01	-3.04E+00	1.24E-08	-4.61E+00	8.20E-14	-2.28E+00	1.23E-06	1.57E+00	2.00E-04
ENSBTAG00000038111	9.87E-02	1.00E+00	-2.00E-01	7.18E-01	-3.86E-01	2.69E-01	-2.62E-01	5.37E-01	-2.95E-01	8.00E-01
ENSBTAG00000016026	8.41E-02	1.00E+00	8.09E-02	1.00E+00	-3.80E-01	3.48E-01	-3.72E-01	3.78E-01	1.91E-01	1.00E+00
ENSBTAG00000002232	6.04E-01	4.48E-01	1.55E-01	9.83E-01	-4.62E-01	3.56E-01	-3.21E-01	6.00E-01	-4.22E-01	8.00E-01
ENSBTAG00000023429	-3.50E-01	7.71E-01	-1.68E+00	3.59E-09	-2.80E+00	9.38E-22	-2.40E+00	1.03E-16	2.85E-01	8.31E-01
ENSBTAG00000011529	-1.23E-01	1.00E+00	-8.60E-01	2.80E-04	-2.76E-01	3.89E-01	-4.48E-01	9.86E-02	-3.31E-01	6.43E-01
ENSBTAG00000011524	3.78E-01	6.97E-01	1.37E+00	6.41E-07	5.57E-01	7.90E-02	5.46E-01	9.44E-02	8.29E-02	1.00E+00
ENSBTAG00000018138	4.14E-01	6.50E-01	-8.20E-01	8.89E-03	-9.74E-01	1.43E-03	2.63E-02	1.00E+00	6.61E-01	2.54E-01
ENSBTAG00000006222	4.68E-01	4.54E-01	1.66E+00	1.15E-10	9.21E-01	7.38E-04	6.50E-01	2.98E-02	3.51E-01	7.62E-01
ENSBTAG00000019007	1.82E+00	4.52E-01	1.27E+00	4.46E-01	-1.59E-01	1.00E+00	-1.84E-01	1.00E+00	-2.71E+00	6.01E-01
ENSBTAG00000014140	1.40E+00	2.58E-13	2.43E+00	7.89E-39	2.17E+00	5.79E-32	1.64E+00	4.61E-19	-4.55E-02	1.00E+00
ENSBTAG00000021886	1.51E-01	1.00E+00	2.21E+00	3.88E-04	-5.79E-01	7.55E-01	-6.04E-01	7.41E-01	-2.63E+00	2.71E-01
ENSBTAG00000044773	0.00E+00	1.00E+00	0.00E+00	1.00E+00	-5.13E-15	1.00E+00	0.00E+00	1.00E+00	0.00E+00	1.00E+00
ENSBTAG00000002185	6.76E-01	5.50E-03	8.63E-01	1.09E-05	3.72E-01	1.01E-01	1.67E-02	1.00E+00	-2.87E-01	6.30E-01
ENSBTAG00000045597	0.00E+00	1.00E+00	0.00E+00	1.00E+00	-5.13E-15	1.00E+00	0.00E+00	1.00E+00	0.00E+00	1.00E+00
ENSBTAG00000015413	7.60E-01	5.57E-02	1.02E+00	2.67E-04	7.45E-01	1.12E-02	6.92E-01	2.32E-02	-3.93E-01	6.95E-01
ENSBTAG00000040061	-7.04E-01	4.36E-01	-7.63E-01	1.05E-01	-4.41E-01	4.41E-01	8.38E-02	1.00E+00	2.88E-01	1.00E+00
ENSBTAG00000014034	2.99E-02	1.00E+00	-1.56E+00	6.23E-06	-3.62E-03	1.00E+00	3.03E-01	4.97E-01	-7.87E-02	1.00E+00
ENSBTAG00000009115	9.27E-01	9.45E-01	1.41E+00	2.58E-01	1.13E+00	4.18E-01	1.81E-01	1.00E+00	1.14E-02	1.00E+00
ENSBTAG00000015310	-6.14E-02	1.00E+00	-2.10E-01	8.54E-01	1.12E-01	1.00E+00	6.04E-01	1.78E-01	-5.13E-01	6.68E-01
ENSBTAG00000018346	-1.29E+00	9.40E-01	-4.20E-01	1.00E+00	-6.54E-01	8.98E-01	7.20E-01	7.60E-01	-3.21E+00	4.35E-01
ENSBTAG00000008714	3.60E-01	1.00E+00	8.18E-01	2.94E-01	-2.24E-02	1.00E+00	3.84E-01	8.05E-01	-6.90E-01	8.43E-01
ENSBTAG00000044978	0.00E+00	1.00E+00	0.00E+00	1.00E+00	-5.13E-15	1.00E+00	0.00E+00	1.00E+00	0.00E+00	1.00E+00
ENSBTAG00000048200	1.91E+00	8.23E-01	0.00E+00	1.00E+00	-5.13E-15	1.00E+00	0.00E+00	1.00E+00	0.00E+00	1.00E+00
ENSBTAG00000047216	-4.09E-01	6.83E-01	-1.79E+00	3.01E-08	-3.15E+00	1.69E-19	-2.74E+00	4.29E-16	-3.59E-01	8.00E-01
ENSBTAG00000043742	0.00E+00	1.00E+00	1.98E+00	4.43E-01	1.78E+00	4.79E-01	1.76E+00	4.97E-01	0.00E+00	1.00E+00
ENSBTAG00000002896	-1.09E-01	1.00E+00	5.07E-01	4.95E-02	9.20E-01	7.63E-05	7.35E-01	2.54E-03	-3.34E-02	1.00E+00
ENSBTAG00000020028	0.00E+00	1.00E+00	0.00E+00	1.00E+00	-5.13E-15	1.00E+00	0.00E+00	1.00E+00	0.00E+00	1.00E+00
ENSBTAG00000012867	2.11E-02	1.00E+00	1.20E+00	2.39E-06	8.54E-01	1.15E-03	1.09E+00	2.50E-05	4.12E-01	5.69E-01
ENSBTAG00000015056	-1.14E+00	3.15E-01	-1.85E+00	4.36E-03	-2.52E+00	9.89E-05	-2.33E+00	3.24E-04	-1.20E+00	3.31E-01

ENSBTAG00000043381	0.00E+00	1.00E+00	0.00E+00	1.00E+00	-5.13E-15	1.00E+00	0.00E+00	1.00E+00	0.00E+00	1.00E+00
ENSBTAG00000014843	2.41E-01	8.23E-01	7.59E-01	5.73E-04	9.81E-01	4.72E-06	9.05E-01	3.52E-05	1.56E-01	1.00E+00
ENSBTAG00000047608	0.00E+00	1.00E+00	0.00E+00	1.00E+00	-5.13E-15	1.00E+00	0.00E+00	1.00E+00	0.00E+00	1.00E+00
ENSBTAG00000046040	0.00E+00	1.00E+00	0.00E+00	1.00E+00	-5.13E-15	1.00E+00	1.77E+00	4.97E-01	0.00E+00	1.00E+00
ENSBTAG00000046738	0.00E+00	1.00E+00	0.00E+00	1.00E+00	-5.13E-15	1.00E+00	0.00E+00	1.00E+00	0.00E+00	1.00E+00
ENSBTAG00000045637	-1.91E+00	8.23E-01	-1.91E+00	4.69E-01	-1.91E+00	4.28E-01	-1.91E+00	4.39E-01	-1.91E+00	8.00E-01
ENSBTAG00000031277	3.23E+00	3.75E-01	3.68E+00	5.08E-02	1.78E+00	4.79E-01	1.77E+00	4.97E-01	0.00E+00	1.00E+00
ENSBTAG00000043968	0.00E+00	1.00E+00	0.00E+00	1.00E+00	-5.13E-15	1.00E+00	0.00E+00	1.00E+00	0.00E+00	1.00E+00
ENSBTAG00000006909	-4.72E-02	1.00E+00	-4.44E-01	4.00E-01	2.54E-02	1.00E+00	3.42E-01	5.66E-01	1.93E-01	1.00E+00
ENSBTAG00000023426	-1.01E-01	1.00E+00	-5.55E-01	5.07E-01	7.54E-01	1.89E-01	6.42E-01	3.08E-01	6.53E-01	7.03E-01
ENSBTAG00000018538	4.11E-02	1.00E+00	-2.85E-01	2.23E-01	-1.08E-02	1.00E+00	1.89E-01	4.97E-01	-2.23E-01	8.00E-01
ENSBTAG00000008111	1.52E-02	1.00E+00	2.57E-02	1.00E+00	1.92E-01	1.00E+00	-1.32E+00	9.96E-02	-2.51E-01	1.00E+00
ENSBTAG00000001497	-4.56E-02	1.00E+00	-8.60E-01	3.42E-05	-1.30E+00	1.33E-10	-4.24E-01	7.13E-02	-2.48E-01	7.90E-01
ENSBTAG00000012850	-5.55E-01	9.36E-01	2.07E-01	1.00E+00	1.87E-01	1.00E+00	-6.27E-01	4.90E-01	8.67E-01	5.50E-01
ENSBTAG00000019952	4.49E-01	4.03E-01	9.06E-02	1.00E+00	-3.18E-01	3.49E-01	-3.18E-01	3.63E-01	2.88E-01	8.00E-01
ENSBTAG00000019949	-7.57E-02	1.00E+00	1.01E+00	1.14E-06	1.10E+00	7.69E-08	6.81E-01	1.82E-03	-1.01E-01	1.00E+00
ENSBTAG00000001451	6.04E-03	1.00E+00	-1.91E+00	4.69E-01	-1.91E+00	4.28E-01	-1.91E+00	4.39E-01	-1.91E+00	8.00E-01
ENSBTAG00000015198	-9.12E-02	1.00E+00	-6.17E-01	5.39E-02	-1.52E-02	1.00E+00	-8.59E-01	4.64E-03	-6.15E-03	1.00E+00
ENSBTAG00000014589	0.00E+00	1.00E+00	0.00E+00	1.00E+00	1.78E+00	4.79E-01	1.76E+00	4.97E-01	2.71E+00	6.01E-01
ENSBTAG00000019535	0.00E+00	1.00E+00	0.00E+00	1.00E+00	-5.13E-15	1.00E+00	0.00E+00	1.00E+00	1.91E+00	8.00E-01
ENSBTAG00000043591	0.00E+00	1.00E+00	0.00E+00	1.00E+00	-5.13E-15	1.00E+00	0.00E+00	1.00E+00	0.00E+00	1.00E+00
ENSBTAG00000008299	1.04E+00	4.04E-01	2.74E+00	3.12E-08	1.68E+00	2.62E-03	6.56E-01	4.52E-01	2.09E+00	2.33E-03
ENSBTAG00000046781	0.00E+00	1.00E+00	0.00E+00	1.00E+00	-5.13E-15	1.00E+00	0.00E+00	1.00E+00	0.00E+00	1.00E+00
ENSBTAG00000018427	3.86E-01	2.20E-01	1.34E+00	7.72E-15	6.96E-01	1.04E-04	5.13E-01	7.15E-03	5.41E-03	1.00E+00
ENSBTAG00000003833	-2.51E-01	1.00E+00	-2.45E+00	8.71E-07	-2.42E+00	3.85E-07	-2.17E+00	4.15E-06	1.31E-01	1.00E+00
ENSBTAG00000048305	0.00E+00	1.00E+00	0.00E+00	1.00E+00	-5.13E-15	1.00E+00	0.00E+00	1.00E+00	0.00E+00	1.00E+00
ENSBTAG00000016306	-1.32E-01	1.00E+00	2.42E-01	9.72E-01	4.82E-01	5.87E-01	3.24E-01	8.43E-01	-7.68E-01	7.04E-01
ENSBTAG00000035437	9.97E-02	1.00E+00	1.95E-01	5.58E-01	9.72E-02	9.51E-01	2.06E-01	5.22E-01	-1.16E-01	1.00E+00
ENSBTAG00000015221	-4.58E-02	1.00E+00	-3.21E-01	2.97E-01	-1.11E+00	2.74E-06	-1.50E+00	1.83E-10	-1.80E-01	1.00E+00
ENSBTAG00000000750	4.32E-01	3.01E-01	1.57E+00	2.74E-14	9.21E-01	1.55E-05	5.51E-01	1.98E-02	2.31E-01	8.08E-01
ENSBTAG00000023416	-5.60E-02	1.00E+00	-1.21E+00	1.03E-07	-1.51E+00	1.84E-11	-8.21E-01	4.28E-04	-2.41E-01	8.00E-01
ENSBTAG00000045461	0.00E+00	1.00E+00	0.00E+00	1.00E+00	-5.13E-15	1.00E+00	0.00E+00	1.00E+00	0.00E+00	1.00E+00
ENSBTAG00000042460	0.00E+00	1.00E+00	0.00E+00	1.00E+00	-5.13E-15	1.00E+00	0.00E+00	1.00E+00	0.00E+00	1.00E+00
ENSBTAG00000015377	-3.50E-02	1.00E+00	7.36E-01	2.72E-01	-5.31E-02	1.00E+00	6.71E-01	3.34E-01	-7.09E-02	1.00E+00
ENSBTAG00000007014	-1.52E-01	1.00E+00	-4.63E-01	1.32E-01	-1.44E-01	8.57E-01	-2.22E-01	6.09E-01	-1.19E-01	1.00E+00

ENSBTAG00000014461	-2.48E-01	7.43E-01	-1.02E+00	1.19E-07	-1.38E+00	3.60E-13	-1.38E+00	3.84E-13	-2.35E-01	7.80E-01
ENSBTAG00000000742	-5.37E-01	5.76E-01	-4.10E-02	1.00E+00	-8.95E-01	1.67E-02	-3.33E-01	5.35E-01	5.82E-01	5.16E-01
ENSBTAG00000016999	3.73E-02	1.00E+00	-6.95E-03	1.00E+00	4.13E-01	5.90E-02	4.47E-01	4.18E-02	-1.53E-01	9.93E-01
ENSBTAG00000001324	6.61E-02	1.00E+00	-1.76E-01	9.53E-01	-9.01E-02	1.00E+00	5.31E-01	2.61E-01	-6.76E-02	1.00E+00
ENSBTAG00000000905	3.73E-01	1.00E+00	2.93E+00	2.00E-03	1.48E+00	1.76E-01	2.83E-01	1.00E+00	-8.51E-01	9.31E-01
ENSBTAG00000044075	4.03E-01	5.88E-01	1.81E+00	1.51E-11	2.22E+00	1.40E-16	2.22E+00	2.19E-16	-1.26E-01	1.00E+00
ENSBTAG00000007273	-1.02E-01	1.00E+00	-5.15E-01	5.66E-02	-1.31E+00	3.98E-08	-1.46E+00	1.39E-09	3.45E-01	6.45E-01
ENSBTAG00000023411	-4.26E-02	1.00E+00	-3.60E-01	2.56E-01	-1.18E+00	2.18E-06	-1.32E+00	1.41E-07	8.76E-01	1.21E-02
ENSBTAG00000000526	-1.21E-01	1.00E+00	-4.64E-01	4.82E-02	-3.89E-01	1.13E-01	-4.02E-02	1.00E+00	7.68E-02	1.00E+00
ENSBTAG00000035556	7.24E-02	1.00E+00	1.02E+00	6.60E-09	1.51E+00	5.00E-18	1.15E+00	5.86E-11	-3.51E-01	3.90E-01
ENSBTAG00000043328	0.00E+00	1.00E+00	0.00E+00	1.00E+00	-5.13E-15	1.00E+00	0.00E+00	1.00E+00	0.00E+00	1.00E+00
ENSBTAG00000017659	-9.50E-01	1.67E-01	-1.30E+00	4.47E-03	-1.25E+00	4.50E-03	-1.24E+00	5.36E-03	-3.76E-01	8.77E-01
ENSBTAG00000018508	-1.09E+00	4.85E-01	-1.16E+00	1.40E-01	-1.78E+00	1.63E-02	-1.31E+00	8.11E-02	-6.06E-01	8.49E-01
ENSBTAG00000046329	0.00E+00	1.00E+00	1.98E+00	4.43E-01	2.56E+00	2.54E-01	0.00E+00	1.00E+00	1.91E+00	8.00E-01
ENSBTAG00000035654	2.46E-01	9.44E-01	5.75E-01	6.28E-02	6.18E-01	4.09E-02	5.32E-01	9.84E-02	2.53E-01	8.99E-01
ENSBTAG00000045260	0.00E+00	1.00E+00	0.00E+00	1.00E+00	-5.13E-15	1.00E+00	0.00E+00	1.00E+00	0.00E+00	1.00E+00
ENSBTAG00000005769	-5.26E-01	2.61E-01	-9.09E-01	5.83E-04	-1.27E-01	9.14E-01	-4.54E-01	1.40E-01	5.56E-02	1.00E+00
ENSBTAG00000004495	2.99E-01	7.20E-01	6.75E-01	4.30E-03	6.19E-01	9.70E-03	7.95E-01	6.97E-04	1.82E-01	9.72E-01
ENSBTAG00000031010	-1.90E-02	1.00E+00	-1.67E-01	8.58E-01	-7.83E-01	1.25E-02	-6.37E-01	5.73E-02	4.03E-01	6.84E-01
ENSBTAG00000019577	0.00E+00	1.00E+00	0.00E+00	1.00E+00	-5.13E-15	1.00E+00	0.00E+00	1.00E+00	0.00E+00	1.00E+00
ENSBTAG00000002966	2.87E-01	8.23E-01	6.40E-01	2.69E-02	9.17E-01	7.66E-04	8.66E-01	1.95E-03	1.25E-01	1.00E+00
ENSBTAG00000048110	0.00E+00	1.00E+00	0.00E+00	1.00E+00	-5.13E-15	1.00E+00	0.00E+00	1.00E+00	0.00E+00	1.00E+00
ENSBTAG00000011634	3.57E-01	8.25E-01	-4.90E-01	2.67E-01	-1.10E+00	2.56E-03	-8.28E-01	3.19E-02	3.43E-01	8.31E-01
ENSBTAG00000012798	1.06E+00	5.86E-01	1.15E+00	1.87E-01	7.85E-01	4.48E-01	1.69E+00	1.74E-02	5.60E-01	1.00E+00
ENSBTAG00000010448	-5.39E-01	1.00E+00	-1.04E+00	6.92E-01	-2.01E+00	2.62E-01	-1.83E+00	3.36E-01	-5.73E-01	1.00E+00
ENSBTAG00000030942	3.76E-02	1.00E+00	4.40E-02	1.00E+00	-1.29E-01	7.30E-01	-9.49E-02	9.02E-01	-1.81E-01	8.45E-01
ENSBTAG00000018749	-9.12E-02	1.00E+00	-6.25E-01	1.40E-01	-8.39E-01	3.20E-02	-8.90E-01	2.44E-02	1.72E-01	1.00E+00
ENSBTAG00000009258	5.44E-01	6.05E-01	-6.18E-01	1.96E-01	-5.95E-01	2.09E-01	-2.53E-01	7.74E-01	2.87E-01	9.98E-01
ENSBTAG00000045294	0.00E+00	1.00E+00	0.00E+00	1.00E+00	-5.13E-15	1.00E+00	0.00E+00	1.00E+00	0.00E+00	1.00E+00
ENSBTAG00000007462	-9.68E-03	1.00E+00	2.94E-01	4.69E-01	7.35E-01	1.27E-02	4.64E-01	1.76E-01	2.59E-01	8.97E-01
ENSBTAG00000011626	-2.35E-03	1.00E+00	-2.81E-01	3.38E-01	-4.08E-01	1.09E-01	-1.45E-01	7.80E-01	4.88E-03	1.00E+00
ENSBTAG00000035701	0.00E+00	1.00E+00	0.00E+00	1.00E+00	-5.13E-15	1.00E+00	0.00E+00	1.00E+00	0.00E+00	1.00E+00
ENSBTAG00000046620	-7.10E-02	1.00E+00	-2.82E-01	6.18E-01	-3.13E-01	5.37E-01	-4.54E-01	3.04E-01	-7.07E-01	3.15E-01
ENSBTAG00000013600	2.48E-03	1.00E+00	4.58E-01	3.00E-02	7.03E-01	3.24E-04	4.61E-01	3.17E-02	-3.32E-01	5.06E-01
ENSBTAG00000013602	1.45E-01	1.00E+00	-4.47E-01	4.68E-02	-1.66E-01	6.35E-01	1.48E-01	7.21E-01	-4.77E-01	2.06E-01

ENSBTAG00000018768	-1.75E-01	1.00E+00	-3.47E-01	2.24E-01	-9.10E-01	8.33E-05	-7.98E-01	8.21E-04	-2.97E-01	7.44E-01
ENSBTAG00000023106	-2.03E-01	1.00E+00	-5.57E-01	5.03E-02	-4.41E-01	1.40E-01	1.27E-01	9.12E-01	-1.28E-01	1.00E+00
ENSBTAG00000013629	-1.58E-01	1.00E+00	-5.74E-01	1.37E-01	-2.15E-01	7.68E-01	2.73E-01	6.23E-01	-4.08E-03	1.00E+00
ENSBTAG00000030993	3.60E+00	2.29E-01	3.68E+00	5.07E-02	3.43E+00	7.35E-02	2.53E+00	2.71E-01	1.93E+00	8.00E-01
ENSBTAG00000043046	0.00E+00	1.00E+00	0.00E+00	1.00E+00	-5.13E-15	1.00E+00	0.00E+00	1.00E+00	0.00E+00	1.00E+00
ENSBTAG00000042463	0.00E+00	1.00E+00	0.00E+00	1.00E+00	-5.13E-15	1.00E+00	0.00E+00	1.00E+00	0.00E+00	1.00E+00
ENSBTAG00000007068	-9.68E-02	1.00E+00	-3.58E-01	4.62E-01	-8.00E-01	3.05E-02	-5.70E-01	1.60E-01	9.29E-02	1.00E+00
ENSBTAG00000043182	0.00E+00	1.00E+00	0.00E+00	1.00E+00	-5.13E-15	1.00E+00	0.00E+00	1.00E+00	0.00E+00	1.00E+00
ENSBTAG00000023407	-2.71E-01	1.00E+00	-5.77E+00	3.69E-18	-5.51E+00	1.98E-19	-1.56E+00	2.88E-04	9.99E-01	1.11E-01
ENSBTAG00000002029	2.03E-01	9.58E-01	1.07E-01	9.50E-01	-1.43E-01	8.12E-01	-1.23E-02	1.00E+00	3.83E-01	5.40E-01
ENSBTAG00000004754	-3.54E-01	8.84E-01	9.38E-02	1.00E+00	-1.23E+00	1.43E-03	-8.96E-01	2.65E-02	-6.78E-01	4.34E-01
ENSBTAG00000042467	0.00E+00	1.00E+00	0.00E+00	1.00E+00	-5.13E-15	1.00E+00	0.00E+00	1.00E+00	0.00E+00	1.00E+00
ENSBTAG00000047593	0.00E+00	1.00E+00	0.00E+00	1.00E+00	-5.13E-15	1.00E+00	0.00E+00	1.00E+00	1.91E+00	8.00E-01
ENSBTAG00000043030	0.00E+00	1.00E+00	0.00E+00	1.00E+00	-5.13E-15	1.00E+00	0.00E+00	1.00E+00	0.00E+00	1.00E+00
ENSBTAG00000000394	-7.49E-02	1.00E+00	1.78E-01	9.63E-01	-2.09E-01	8.92E-01	-2.74E-01	7.59E-01	5.06E-02	1.00E+00
ENSBTAG00000030921	-4.83E-01	1.00E+00	-2.38E-01	1.00E+00	-6.35E-01	6.13E-01	-1.73E+00	4.94E-02	-8.26E-02	1.00E+00
ENSBTAG00000008471	-4.15E-01	1.00E+00	6.12E-02	1.00E+00	4.86E-01	9.67E-01	-1.05E+00	5.46E-01	-2.72E-01	1.00E+00
ENSBTAG00000030913	-6.28E-01	9.50E-01	-5.03E-01	7.37E-01	-7.57E-01	4.65E-01	-1.29E+00	1.37E-01	-4.70E-01	1.00E+00
ENSBTAG00000009132	2.34E+00	1.51E-09	1.18E+00	1.85E-03	6.16E-01	1.61E-01	1.43E+00	1.47E-04	1.11E+00	4.73E-02
ENSBTAG00000045771	0.00E+00	1.00E+00	0.00E+00	1.00E+00	-5.13E-15	1.00E+00	0.00E+00	1.00E+00	0.00E+00	1.00E+00
ENSBTAG00000046015	0.00E+00	1.00E+00	0.00E+00	1.00E+00	-5.13E-15	1.00E+00	2.53E+00	2.71E-01	0.00E+00	1.00E+00
ENSBTAG00000021251	-1.10E+00	2.51E-03	-3.05E+00	3.95E-22	-2.62E+00	9.88E-18	-1.08E+00	4.81E-04	-4.81E-01	5.62E-01
ENSBTAG00000021253	1.11E-01	1.00E+00	3.10E-01	3.81E-01	4.89E-01	9.40E-02	4.83E-01	1.06E-01	7.37E-01	6.37E-02
ENSBTAG00000021259	2.42E-01	9.79E-01	2.59E-01	5.76E-01	1.12E+00	4.93E-05	1.54E+00	9.73E-09	6.22E-02	1.00E+00
ENSBTAG00000030836	1.06E-01	1.00E+00	1.24E+00	2.61E-06	7.88E-01	4.36E-03	3.41E-01	3.49E-01	-1.88E-01	1.00E+00
ENSBTAG00000021270	2.15E-01	1.00E+00	9.71E-02	1.00E+00	-1.58E-01	1.00E+00	9.65E-01	3.80E-01	-2.36E-01	1.00E+00
ENSBTAG00000021272	-2.34E-01	1.00E+00	-1.50E+00	4.18E-03	-3.37E-01	6.64E-01	2.52E-03	1.00E+00	-4.36E-01	8.53E-01
ENSBTAG00000021276	-4.04E-01	1.00E+00	-1.32E+00	1.63E-01	-1.25E+00	1.84E-01	-1.49E+00	1.06E-01	-4.26E-01	1.00E+00
ENSBTAG00000030814	-1.91E+00	8.23E-01	7.58E-02	1.00E+00	-1.25E-01	1.00E+00	-1.91E+00	4.39E-01	-1.91E+00	8.00E-01
ENSBTAG00000048276	0.00E+00	1.00E+00	0.00E+00	1.00E+00	-5.13E-15	1.00E+00	0.00E+00	1.00E+00	1.91E+00	8.00E-01
ENSBTAG00000021280	-3.66E-01	1.00E+00	6.49E-01	7.84E-01	1.28E-01	1.00E+00	-1.83E-01	1.00E+00	3.23E-01	1.00E+00
ENSBTAG00000023398	-2.64E-01	1.00E+00	-1.08E+00	1.34E-02	-5.86E-01	2.21E-01	-1.37E+00	1.35E-03	-7.01E-01	4.70E-01
ENSBTAG00000021284	3.22E+00	3.75E-01	0.00E+00	1.00E+00	1.78E+00	4.79E-01	3.40E+00	8.33E-02	0.00E+00	1.00E+00
ENSBTAG00000021286	2.19E-01	1.00E+00	7.49E-02	1.00E+00	8.63E-01	1.19E-02	5.78E-01	1.41E-01	3.53E-02	1.00E+00
ENSBTAG00000021899	-2.19E-01	1.00E+00	1.01E-01	1.00E+00	8.30E-01	2.71E-02	-8.37E-02	1.00E+00	-2.29E-01	1.00E+00

ENSBTAG00000046859	0.00E+00	1.00E+00	0.00E+00	1.00E+00	-5.13E-15	1.00E+00	0.00E+00	1.00E+00	0.00E+00	1.00E+00
ENSBTAG00000021490	6.89E-01	4.22E-02	2.00E+00	1.42E-17	2.12E+00	9.38E-20	1.51E+00	1.93E-10	3.04E-01	7.77E-01
ENSBTAG00000021491	-4.49E-02	1.00E+00	-4.14E-01	1.01E-01	-7.33E-02	1.00E+00	1.90E-01	6.04E-01	3.27E-02	1.00E+00
ENSBTAG00000014153	8.41E-02	1.00E+00	1.75E+00	1.57E-11	3.70E-01	2.84E-01	4.01E-01	2.43E-01	2.80E-01	8.20E-01
ENSBTAG00000000160	-4.19E-02	1.00E+00	-6.06E-01	1.46E-02	-1.91E+00	8.48E-17	-1.34E+00	7.76E-09	3.28E-01	6.52E-01
ENSBTAG00000011645	1.26E-02	1.00E+00	4.19E-01	3.73E-02	3.61E-01	8.49E-02	-3.64E-02	1.00E+00	-3.28E-01	4.60E-01
ENSBTAG00000003134	0.00E+00	1.00E+00	0.00E+00	1.00E+00	-5.13E-15	1.00E+00	2.53E+00	2.71E-01	0.00E+00	1.00E+00
ENSBTAG00000017321	-2.33E-01	8.52E-01	-1.42E+00	2.79E-10	-1.10E+00	1.01E-06	-3.76E-01	1.78E-01	-1.47E-01	1.00E+00
ENSBTAG00000021771	-1.64E-01	9.31E-01	-5.00E-01	7.84E-03	-4.34E-01	2.45E-02	-2.10E-01	4.13E-01	-1.54E-01	9.26E-01
ENSBTAG00000017060	-1.77E-01	1.00E+00	-4.17E-01	1.73E-01	-2.74E-01	4.46E-01	-3.25E-01	3.43E-01	-2.15E-01	9.37E-01
ENSBTAG00000017010	8.72E-02	1.00E+00	8.14E-01	4.18E-04	8.48E-01	1.96E-04	9.05E-01	7.81E-05	-2.79E-01	8.00E-01
ENSBTAG00000017486	1.59E-01	1.00E+00	3.71E-01	3.33E-01	1.20E+00	9.37E-06	1.28E+00	2.29E-06	2.56E-01	9.13E-01
ENSBTAG00000007818	-3.44E-02	1.00E+00	-7.24E-03	1.00E+00	-4.00E-02	1.00E+00	-1.32E-01	7.60E-01	3.20E-01	5.25E-01
ENSBTAG00000014880	8.05E-01	1.70E-01	1.21E-01	1.00E+00	8.83E-01	1.96E-02	9.26E-01	1.53E-02	1.55E-02	1.00E+00
ENSBTAG00000014882	1.19E+00	8.55E-01	1.27E+00	4.43E-01	2.20E+00	4.39E-02	1.23E+00	4.57E-01	1.14E-02	1.00E+00
ENSBTAG00000023393	-3.59E+00	2.31E-01	-8.03E-01	7.80E-01	-5.37E-01	9.33E-01	1.07E-01	1.00E+00	-3.68E-01	1.00E+00
ENSBTAG00000010658	-1.02E-01	1.00E+00	3.61E-01	1.66E-01	8.41E-01	1.15E-04	1.15E+00	1.04E-07	-1.68E-01	9.93E-01
ENSBTAG00000019589	-3.50E-01	6.15E-01	-1.27E+00	3.01E-07	-1.14E+00	3.34E-06	-4.57E-01	1.08E-01	1.26E-02	1.00E+00
ENSBTAG00000021901	-1.26E-01	1.00E+00	-1.03E+00	1.72E-05	-7.08E-01	4.04E-03	-7.29E-01	3.52E-03	-2.66E-01	8.00E-01
ENSBTAG00000046909	0.00E+00	1.00E+00	0.00E+00	1.00E+00	-5.13E-15	1.00E+00	0.00E+00	1.00E+00	0.00E+00	1.00E+00
ENSBTAG00000017882	-1.96E-01	1.00E+00	-3.39E-01	3.41E-01	-1.41E+00	9.41E-08	-1.28E+00	1.97E-06	7.50E-01	7.31E-02
ENSBTAG00000037662	0.00E+00	1.00E+00	0.00E+00	1.00E+00	-5.13E-15	1.00E+00	0.00E+00	1.00E+00	0.00E+00	1.00E+00
ENSBTAG00000040291	0.00E+00	1.00E+00	0.00E+00	1.00E+00	-5.13E-15	1.00E+00	0.00E+00	1.00E+00	0.00E+00	1.00E+00
ENSBTAG00000018607	-4.79E-03	1.00E+00	-1.68E+00	4.33E-03	-1.35E+00	1.78E-02	-9.21E-01	1.32E-01	9.50E-01	3.46E-01
ENSBTAG00000018610	0.00E+00	1.00E+00	0.00E+00	1.00E+00	-5.13E-15	1.00E+00	0.00E+00	1.00E+00	0.00E+00	1.00E+00
ENSBTAG00000030862	0.00E+00	1.00E+00	0.00E+00	1.00E+00	-5.13E-15	1.00E+00	0.00E+00	1.00E+00	0.00E+00	1.00E+00
ENSBTAG00000011012	0.00E+00	1.00E+00	0.00E+00	1.00E+00	-5.13E-15	1.00E+00	2.53E+00	2.71E-01	0.00E+00	1.00E+00
ENSBTAG00000000972	2.72E+00	5.76E-01	0.00E+00	1.00E+00	-5.13E-15	1.00E+00	2.53E+00	2.71E-01	0.00E+00	1.00E+00
ENSBTAG00000048090	-7.18E-02	1.00E+00	5.57E-01	6.60E-03	6.08E-01	2.53E-03	5.25E-01	1.26E-02	3.52E-01	4.50E-01
ENSBTAG00000011437	3.01E+00	5.23E-24	3.00E+00	8.12E-25	4.42E+00	3.73E-48	3.95E+00	8.05E-40	2.64E-01	9.23E-01
ENSBTAG00000017407	4.71E-01	4.33E-01	-8.41E-01	5.04E-03	-9.92E-01	6.32E-04	-9.00E-01	2.52E-03	3.89E-01	6.43E-01
ENSBTAG00000017418	3.40E-01	5.76E-01	7.88E-01	6.56E-04	1.37E+00	4.06E-10	8.72E-01	1.58E-04	1.20E-01	1.00E+00
ENSBTAG00000018186	2.39E+00	2.05E-12	3.96E+00	2.72E-31	3.37E+00	2.91E-24	2.79E+00	1.63E-17	5.30E-01	5.56E-01
ENSBTAG00000018189	3.13E+00	3.46E-03	3.74E+00	2.84E-05	4.20E+00	2.76E-06	3.95E+00	1.25E-05	3.51E-01	1.00E+00
ENSBTAG00000000524	4.16E-01	4.85E-01	4.49E-01	1.26E-01	6.37E-01	1.73E-02	8.68E-01	8.00E-04	-1.86E-01	1.00E+00

ENSBTAG00000003981	4.06E-01	3.33E-01	1.67E+00	2.06E-16	1.65E+00	3.73E-16	1.10E+00	1.16E-07	1.32E-04	1.00E+00
ENSBTAG00000020456	-9.41E-02	1.00E+00	-6.66E-01	1.55E-02	-6.81E-01	1.26E-02	-3.92E-01	2.26E-01	2.91E-01	8.00E-01
ENSBTAG00000007100	1.54E-01	1.00E+00	-3.67E-01	3.51E-01	1.93E-01	7.63E-01	5.57E-01	9.81E-02	3.86E-01	7.08E-01
ENSBTAG00000007105	6.74E-02	1.00E+00	1.77E+00	9.47E-18	1.89E+00	3.45E-20	1.27E+00	1.29E-09	-1.26E-01	1.00E+00
ENSBTAG00000007106	-2.90E-01	7.51E-01	-7.78E-01	8.51E-04	-1.37E+00	8.25E-10	-1.54E+00	6.28E-12	-6.26E-01	8.94E-02
ENSBTAG00000048134	0.00E+00	1.00E+00	0.00E+00	1.00E+00	-5.13E-15	1.00E+00	0.00E+00	1.00E+00	0.00E+00	1.00E+00
ENSBTAG00000023907	-2.00E-01	9.46E-01	-5.05E-01	4.16E-02	-1.36E+00	6.88E-10	-1.56E+00	2.27E-12	3.36E-01	6.03E-01
ENSBTAG00000024162	-3.04E-01	9.32E-01	-7.30E-01	5.49E-02	1.28E+00	6.93E-05	1.28E+00	9.16E-05	9.87E-02	1.00E+00
ENSBTAG00000030775	1.80E-01	1.00E+00	-6.99E-01	6.00E-02	-3.53E-01	4.28E-01	-4.13E-01	3.32E-01	-3.94E-02	1.00E+00
ENSBTAG00000030764	0.00E+00	1.00E+00	0.00E+00	1.00E+00	-5.13E-15	1.00E+00	0.00E+00	1.00E+00	0.00E+00	1.00E+00
ENSBTAG00000011802	-1.14E-01	1.00E+00	-5.02E-01	1.21E-01	-4.23E-01	2.15E-01	-1.99E-01	7.29E-01	1.33E-01	1.00E+00
ENSBTAG00000019269	-7.72E-02	1.00E+00	-3.56E-01	3.40E-01	-4.08E-01	2.44E-01	-2.53E-01	5.74E-01	1.00E-01	1.00E+00
ENSBTAG00000020404	-1.06E-01	1.00E+00	-1.30E+00	1.91E-08	-2.77E+00	1.83E-32	-3.42E+00	4.47E-46	2.69E-01	8.00E-01
ENSBTAG00000020409	-3.98E-01	6.43E-01	-1.58E+00	1.10E-07	-4.18E+00	1.88E-31	-2.30E+00	3.01E-14	6.19E-01	2.48E-01
ENSBTAG00000018936	-6.11E-01	5.95E-01	-1.43E+00	9.15E-04	-1.74E+00	4.06E-05	-1.12E+00	1.34E-02	-2.05E-01	1.00E+00
ENSBTAG00000003148	6.68E-02	1.00E+00	5.62E-01	5.91E-03	5.43E-01	8.04E-03	2.28E-01	4.17E-01	1.14E-01	1.00E+00
ENSBTAG00000003161	4.31E-03	1.00E+00	-4.95E-01	1.15E-01	-4.65E-01	1.45E-01	-3.46E-01	3.43E-01	-5.42E-02	1.00E+00
ENSBTAG00000021984	-1.43E+00	7.65E-01	2.63E+00	1.25E-04	1.28E+00	1.69E-01	3.39E-01	1.00E+00	5.36E-01	1.00E+00
ENSBTAG00000030670	-1.36E-03	1.00E+00	-1.28E+00	1.90E-09	-5.92E-01	8.11E-03	-9.68E-02	9.56E-01	2.19E-01	8.18E-01
ENSBTAG00000011495	-2.56E-01	9.32E-01	-1.66E+00	5.93E-09	-7.92E-01	7.77E-03	-3.56E-01	3.56E-01	4.35E-02	1.00E+00
ENSBTAG00000004777	5.33E-02	1.00E+00	-2.20E-01	8.41E-01	-6.65E-01	1.29E-01	2.35E-01	7.90E-01	5.69E-01	5.85E-01
ENSBTAG00000044930	0.00E+00	1.00E+00	0.00E+00	1.00E+00	-5.13E-15	1.00E+00	0.00E+00	1.00E+00	0.00E+00	1.00E+00
ENSBTAG00000005503	-2.75E-01	1.00E+00	-6.17E-01	3.41E-01	-1.42E-01	1.00E+00	2.83E-01	8.36E-01	4.18E-01	9.15E-01
ENSBTAG00000007039	-6.72E-02	1.00E+00	-7.09E-01	3.30E-03	-9.49E-01	4.53E-05	-9.62E-01	4.51E-05	4.20E-01	4.34E-01
ENSBTAG00000043957	-3.68E-01	1.00E+00	-1.98E+00	1.01E-03	-1.07E+00	7.90E-02	1.91E-01	1.00E+00	6.87E-01	6.76E-01
ENSBTAG00000046447	2.70E+00	5.76E-01	1.98E+00	4.43E-01	1.78E+00	4.79E-01	0.00E+00	1.00E+00	0.00E+00	1.00E+00
ENSBTAG00000004742	2.38E+00	1.86E-07	5.42E+00	1.70E-33	3.98E+00	1.24E-20	2.53E+00	2.26E-09	-6.49E-02	1.00E+00
ENSBTAG00000044270	0.00E+00	1.00E+00	0.00E+00	1.00E+00	-5.13E-15	1.00E+00	0.00E+00	1.00E+00	0.00E+00	1.00E+00
ENSBTAG00000030105	0.00E+00	1.00E+00	0.00E+00	1.00E+00	-5.13E-15	1.00E+00	0.00E+00	1.00E+00	1.91E+00	8.00E-01
ENSBTAG00000018682	-1.09E+00	2.42E-03	-1.69E+00	1.99E-08	-7.43E-01	1.92E-02	-2.32E-01	6.68E-01	6.69E-01	2.28E-01
ENSBTAG00000023384	-5.12E-01	3.14E-01	-2.09E+00	1.02E-15	-4.53E+00	2.40E-57	-4.32E+00	2.33E-53	-2.81E-01	8.00E-01
ENSBTAG00000030629	8.85E-02	1.00E+00	-5.35E-01	1.26E-01	-1.72E+00	3.28E-08	-3.07E+00	9.44E-19	4.72E-01	5.32E-01
ENSBTAG00000018688	-3.76E-01	8.23E-01	-7.43E-01	5.38E-02	-6.42E-01	9.62E-02	-8.64E-01	2.07E-02	-3.87E-01	8.00E-01
ENSBTAG00000014306	7.36E-02	1.00E+00	-6.96E-02	1.00E+00	-4.86E-01	4.21E-02	-2.49E-01	4.30E-01	-4.92E-01	2.48E-01
ENSBTAG00000011876	8.34E-02	1.00E+00	5.94E-01	1.50E-02	1.07E+00	1.98E-06	5.62E-01	2.55E-02	-1.63E-02	1.00E+00

ENSBTAG00000011880	-5.00E-01	6.70E-01	9.63E-01	4.73E-03	1.70E+00	2.54E-08	1.52E+00	1.24E-06	-5.25E-01	6.34E-01
ENSBTAG00000030585	-1.63E-01	1.00E+00	-1.92E+00	8.88E-08	-1.03E+00	5.18E-03	-2.14E+00	2.54E-09	2.62E-01	1.00E+00
ENSBTAG00000037096	0.00E+00	1.00E+00	1.98E+00	4.43E-01	-5.13E-15	1.00E+00	0.00E+00	1.00E+00	0.00E+00	1.00E+00
ENSBTAG00000021447	2.24E+00	4.13E-01	1.77E+00	3.29E-01	-1.31E-01	1.00E+00	2.03E+00	2.01E-01	7.92E-01	1.00E+00
ENSBTAG00000033679	-2.95E-01	8.51E-01	-2.18E+00	7.48E-14	-2.72E+00	2.62E-20	-2.38E+00	3.18E-16	9.67E-03	1.00E+00
ENSBTAG00000036019	-2.19E+00	2.81E-02	-1.85E+00	1.57E-02	-3.03E+00	1.88E-04	-1.44E+00	4.75E-02	-4.10E-01	1.00E+00
ENSBTAG00000043298	0.00E+00	1.00E+00	0.00E+00	1.00E+00	-5.13E-15	1.00E+00	0.00E+00	1.00E+00	0.00E+00	1.00E+00
ENSBTAG00000011512	5.21E-02	1.00E+00	-2.06E-01	7.66E-01	-1.64E+00	2.75E-07	-1.25E+00	1.11E-04	4.92E-01	5.36E-01
ENSBTAG00000008167	3.55E-01	3.63E-01	4.03E-01	5.71E-02	2.87E-01	2.24E-01	3.46E-01	1.26E-01	7.61E-02	1.00E+00
ENSBTAG00000004791	-5.83E-01	7.23E-02	-1.35E+00	1.31E-09	-4.66E-01	5.74E-02	-1.28E-01	8.45E-01	-5.79E-02	1.00E+00
ENSBTAG00000001771	1.48E-01	1.00E+00	4.94E-01	7.04E-02	6.44E-01	1.18E-02	7.12E-01	5.41E-03	2.92E-01	7.93E-01
ENSBTAG00000037616	0.00E+00	1.00E+00	0.00E+00	1.00E+00	-5.13E-15	1.00E+00	0.00E+00	1.00E+00	0.00E+00	1.00E+00
ENSBTAG00000042581	0.00E+00	1.00E+00	0.00E+00	1.00E+00	-5.13E-15	1.00E+00	0.00E+00	1.00E+00	0.00E+00	1.00E+00
ENSBTAG00000045272	0.00E+00	1.00E+00	0.00E+00	1.00E+00	-5.13E-15	1.00E+00	0.00E+00	1.00E+00	0.00E+00	1.00E+00
ENSBTAG00000000973	1.69E+00	2.01E-01	4.05E+00	2.19E-12	4.76E+00	4.47E-19	3.93E+00	1.12E-11	1.10E+00	7.32E-01
ENSBTAG00000011001	-2.92E-01	8.77E-01	-5.79E-01	8.62E-02	-4.75E-01	1.84E-01	-5.94E-01	8.22E-02	-4.42E-01	6.01E-01
ENSBTAG00000043221	6.06E-03	1.00E+00	-7.22E-01	9.45E-01	-2.71E+00	1.99E-01	-2.71E+00	2.05E-01	-7.91E-01	1.00E+00
ENSBTAG00000009214	2.96E+00	4.43E-21	3.25E+00	9.63E-26	2.70E+00	6.67E-19	2.04E+00	1.50E-11	1.08E+00	1.10E-02
ENSBTAG00000020787	2.28E-01	8.20E-01	1.35E+00	6.29E-13	1.14E+00	1.55E-09	5.99E-01	3.29E-03	7.69E-02	1.00E+00
ENSBTAG00000012837	-6.75E-02	1.00E+00	-8.37E-01	2.99E-02	-1.51E+00	2.81E-05	-7.53E-01	5.87E-02	4.34E-02	1.00E+00
ENSBTAG00000021906	-3.77E-02	1.00E+00	-6.94E-01	3.17E-02	-1.21E+00	5.58E-05	-6.65E-01	4.17E-02	-2.29E-01	9.79E-01
ENSBTAG00000021139	1.92E+00	8.23E-01	0.00E+00	1.00E+00	3.43E+00	7.36E-02	1.76E+00	4.97E-01	1.91E+00	8.00E-01
ENSBTAG00000044519	0.00E+00	1.00E+00	0.00E+00	1.00E+00	-5.13E-15	1.00E+00	0.00E+00	1.00E+00	0.00E+00	1.00E+00
ENSBTAG00000006479	3.89E-02	1.00E+00	6.26E-01	2.03E-03	7.30E-01	2.26E-04	5.34E-01	1.17E-02	1.81E-01	8.99E-01
ENSBTAG00000023377	-1.01E-01	1.00E+00	-9.21E-01	1.28E-02	-1.80E+00	2.89E-07	-1.02E+00	5.93E-03	7.02E-02	1.00E+00
ENSBTAG00000030592	-7.90E-02	1.00E+00	-3.99E-01	4.21E-01	-4.83E-01	2.76E-01	-4.95E-01	2.72E-01	-2.69E-01	9.90E-01
ENSBTAG00000023375	2.10E-01	8.87E-01	6.16E-01	5.88E-03	2.37E-01	4.32E-01	-7.55E-02	1.00E+00	-4.07E-02	1.00E+00
ENSBTAG00000019202	-7.45E-03	1.00E+00	1.01E+00	7.94E-07	1.18E+00	3.93E-09	1.08E+00	1.13E-07	1.22E-01	1.00E+00
ENSBTAG00000040193	1.70E-01	1.00E+00	-3.85E-02	1.00E+00	-1.04E+00	7.49E-03	-1.31E+00	6.83E-04	-1.30E-01	1.00E+00
ENSBTAG00000003718	-7.86E-02	1.00E+00	-1.73E+00	5.82E-19	-3.02E+00	7.46E-51	-3.44E+00	2.32E-63	2.86E-01	6.35E-01
ENSBTAG00000019129	-2.18E-01	9.46E-01	-1.99E+00	7.07E-16	-3.15E+00	8.47E-35	-2.31E+00	1.20E-20	5.37E-01	2.74E-01
ENSBTAG00000024815	-8.42E-02	1.00E+00	2.74E-01	3.55E-01	7.90E-02	1.00E+00	6.66E-01	3.66E-03	-6.04E-02	1.00E+00
ENSBTAG00000038697	0.00E+00	1.00E+00	0.00E+00	1.00E+00	-5.13E-15	1.00E+00	0.00E+00	1.00E+00	0.00E+00	1.00E+00
ENSBTAG00000008240	-4.85E-01	1.00E+00	3.32E-01	9.58E-01	1.75E-01	1.00E+00	-1.83E+00	9.88E-02	-1.27E+00	6.63E-01
ENSBTAG00000003916	2.56E-01	7.28E-01	1.37E+00	2.38E-13	1.94E+00	1.76E-25	1.71E+00	4.94E-20	3.09E-02	1.00E+00

ENSBTAG00000004891	1.97E-01	9.20E-01	1.08E+00	1.17E-07	1.07E+00	1.53E-07	6.38E-01	3.67E-03	1.63E-01	9.91E-01
ENSBTAG00000030593	-3.97E-02	1.00E+00	4.50E-01	2.89E-01	4.16E-02	1.00E+00	-3.26E-01	5.28E-01	-4.90E-01	6.45E-01
ENSBTAG00000000502	1.43E+00	3.55E-01	2.43E+00	1.24E-03	2.28E+00	2.49E-03	1.86E+00	2.25E-02	-8.13E-01	9.19E-01
ENSBTAG00000009942	1.09E-01	1.00E+00	-5.17E-01	2.06E-01	-8.49E-01	1.72E-02	-6.77E-01	7.77E-02	-1.11E-01	1.00E+00
ENSBTAG00000042360	0.00E+00	1.00E+00	0.00E+00	1.00E+00	-5.13E-15	1.00E+00	0.00E+00	1.00E+00	0.00E+00	1.00E+00
ENSBTAG00000030581	2.14E-01	9.19E-01	-2.72E-01	4.04E-01	-8.02E-01	7.89E-04	-1.31E-01	8.55E-01	-1.72E-01	1.00E+00
ENSBTAG00000021158	-1.54E-01	1.00E+00	-7.42E-01	1.10E-02	-9.86E-01	4.22E-04	-7.34E-01	1.33E-02	-1.93E-01	1.00E+00
ENSBTAG00000047978	0.00E+00	1.00E+00	0.00E+00	1.00E+00	-5.13E-15	1.00E+00	0.00E+00	1.00E+00	0.00E+00	1.00E+00
ENSBTAG00000047105	-1.91E+00	8.23E-01	8.83E-01	8.52E-01	-1.24E-01	1.00E+00	-1.91E+00	4.39E-01	7.05E-03	1.00E+00
ENSBTAG00000017396	-1.91E+00	8.23E-01	-1.91E+00	4.69E-01	-1.91E+00	4.28E-01	-1.91E+00	4.39E-01	-1.91E+00	8.00E-01
ENSBTAG00000003493	0.00E+00	1.00E+00	1.99E+00	4.43E-01	-5.13E-15	1.00E+00	0.00E+00	1.00E+00	0.00E+00	1.00E+00
ENSBTAG00000046432	-1.37E-01	1.00E+00	5.32E-01	1.16E-01	1.06E+00	3.19E-04	1.29E+00	1.08E-05	-1.49E-01	1.00E+00
ENSBTAG00000045430	0.00E+00	1.00E+00	0.00E+00	1.00E+00	-5.13E-15	1.00E+00	0.00E+00	1.00E+00	0.00E+00	1.00E+00
ENSBTAG00000035858	1.34E-01	1.00E+00	-2.97E-01	3.78E-01	-1.43E+00	4.23E-09	-2.30E+00	3.88E-20	9.93E-01	1.18E-03
ENSBTAG00000000767	7.84E-02	1.00E+00	1.65E+00	7.88E-08	1.77E+00	4.99E-09	1.25E+00	7.48E-05	-1.59E-01	1.00E+00
ENSBTAG00000012804	3.24E-01	1.00E+00	1.09E+00	2.51E-02	2.15E+00	2.32E-06	2.54E+00	3.43E-08	-3.75E-02	1.00E+00
ENSBTAG00000012803	1.37E-01	1.00E+00	1.33E+00	1.09E-11	1.49E+00	1.19E-14	9.54E-01	1.84E-06	-2.46E-01	7.87E-01
ENSBTAG00000018852	-1.99E-01	8.28E-01	-1.61E+00	3.39E-18	-1.17E+00	2.57E-10	-9.07E-01	1.57E-06	-3.87E-01	3.30E-01
ENSBTAG00000014750	-5.81E-02	1.00E+00	-1.10E+00	2.20E-02	-7.90E-01	1.24E-01	-1.52E+00	1.19E-03	-4.18E-01	8.71E-01
ENSBTAG00000043295	-1.36E-03	1.00E+00	6.50E-01	4.06E-01	1.73E+00	8.31E-04	3.99E-01	7.28E-01	-1.04E+00	5.90E-01
ENSBTAG00000047622	0.00E+00	1.00E+00	0.00E+00	1.00E+00	-5.13E-15	1.00E+00	0.00E+00	1.00E+00	0.00E+00	1.00E+00
ENSBTAG00000011338	-5.55E-01	7.81E-01	-2.24E+00	2.81E-06	-1.57E+00	9.65E-04	-5.82E-01	3.46E-01	-4.44E-01	8.55E-01
ENSBTAG00000014099	2.81E-02	1.00E+00	-1.35E-01	8.82E-01	2.64E-01	4.60E-01	5.17E-02	1.00E+00	-3.68E-01	6.14E-01
ENSBTAG00000011076	-3.34E-01	6.18E-01	-2.30E+00	9.49E-23	-4.73E+00	1.55E-70	-4.35E+00	2.50E-63	-2.89E-01	7.73E-01
ENSBTAG00000023186	3.89E-01	7.00E-01	3.34E-01	4.22E-01	2.31E-01	6.50E-01	1.52E-01	8.99E-01	8.35E-03	1.00E+00
ENSBTAG00000014027	-9.89E-01	6.02E-01	-4.32E-01	7.69E-01	-7.05E-03	1.00E+00	4.45E-01	6.94E-01	3.77E-01	1.00E+00
ENSBTAG00000001895	2.47E-01	8.23E-01	3.48E-01	2.16E-01	5.79E-01	1.62E-02	2.55E-01	4.39E-01	2.42E-01	8.01E-01
ENSBTAG00000001898	-1.80E-01	1.00E+00	5.70E-01	2.53E-01	3.72E-01	5.35E-01	5.24E-01	3.13E-01	-3.81E-01	9.00E-01
ENSBTAG00000008699	-5.82E-01	3.77E-01	5.65E-02	1.00E+00	3.77E-01	3.90E-01	4.61E-01	2.60E-01	-1.06E-01	1.00E+00
ENSBTAG00000002354	-1.31E+00	9.32E-01	4.61E-01	9.98E-01	-1.44E+00	4.79E-01	-1.46E+00	4.92E-01	3.69E-01	1.00E+00
ENSBTAG00000002357	-2.55E-01	1.00E+00	3.02E-01	6.88E-01	-2.54E-01	7.91E-01	-1.77E-01	9.68E-01	1.65E-01	1.00E+00
ENSBTAG00000035030	4.90E-01	1.40E-01	9.43E-01	4.91E-06	8.66E-01	2.91E-05	5.71E-01	1.06E-02	3.34E-01	5.63E-01
ENSBTAG00000017442	6.06E-01	8.86E-02	9.51E-01	6.80E-05	3.84E-01	1.81E-01	-5.11E-01	5.82E-02	-1.39E-01	1.00E+00
ENSBTAG00000048058	2.85E-01	9.17E-01	1.03E+00	9.79E-04	6.91E-01	3.80E-02	4.14E-03	1.00E+00	2.48E-01	9.60E-01
ENSBTAG00000017525	1.46E-01	1.00E+00	3.52E-01	1.32E-01	2.64E-01	3.08E-01	9.44E-02	9.42E-01	-2.08E-01	8.13E-01

ENSBTAG00000045118	0.00E+00	1.00E+00	0.00E+00	1.00E+00	-5.13E-15	1.00E+00	0.00E+00	1.00E+00	0.00E+00	1.00E+00
ENSBTAG00000016644	-6.31E-01	7.80E-01	5.20E-01	4.43E-01	7.84E-01	1.50E-01	4.25E-01	5.74E-01	-1.09E+00	3.47E-01
ENSBTAG00000035005	0.00E+00	1.00E+00	0.00E+00	1.00E+00	-5.13E-15	1.00E+00	0.00E+00	1.00E+00	0.00E+00	1.00E+00
ENSBTAG00000039618	2.72E+00	5.76E-01	2.79E+00	2.11E-01	3.42E+00	7.52E-02	4.14E+00	1.58E-02	2.71E+00	6.01E-01
ENSBTAG00000011608	-2.14E-01	9.78E-01	-7.59E-01	4.31E-03	-6.74E-01	1.27E-02	-3.31E-01	3.35E-01	-1.99E-02	1.00E+00
ENSBTAG00000034998	-3.96E-02	1.00E+00	-1.47E+00	9.23E-07	-8.23E-01	6.41E-03	-4.24E-01	2.42E-01	2.43E-01	9.25E-01
ENSBTAG00000035174	-3.22E-03	1.00E+00	-3.35E-01	4.39E-01	6.14E-01	5.70E-02	7.88E-01	1.11E-02	-4.65E-01	6.01E-01
ENSBTAG00000013741	-1.29E+00	9.39E-01	4.73E-01	9.90E-01	-1.43E+00	4.79E-01	-6.72E-01	8.91E-01	-3.21E+00	4.34E-01
ENSBTAG00000044071	1.39E+00	3.33E-01	7.71E-01	4.97E-01	6.12E-01	6.48E-01	1.25E+00	1.51E-01	6.96E-01	9.03E-01
ENSBTAG00000014536	-2.22E-01	9.87E-01	-2.00E+00	7.83E-14	-3.25E+00	9.39E-32	-3.15E+00	4.06E-30	-7.49E-02	1.00E+00
ENSBTAG00000047719	-3.82E-01	1.00E+00	3.83E-01	1.00E+00	-1.82E+00	2.93E-01	-1.07E+00	5.89E-01	-8.93E-01	1.00E+00
ENSBTAG00000047476	0.00E+00	1.00E+00	5.68E+00	1.77E-05	-5.13E-15	1.00E+00	1.76E+00	4.97E-01	1.93E+00	8.00E-01
ENSBTAG00000042300	0.00E+00	1.00E+00	0.00E+00	1.00E+00	-5.13E-15	1.00E+00	0.00E+00	1.00E+00	0.00E+00	1.00E+00
ENSBTAG00000002781	1.00E+00	8.28E-01	3.82E+00	1.95E-04	2.72E+00	8.04E-03	7.05E-02	1.00E+00	6.38E-01	1.00E+00
ENSBTAG00000024878	-5.28E-01	1.00E+00	-1.79E-01	1.00E+00	-9.63E-01	2.77E-01	-5.13E-01	6.80E-01	-9.14E-01	7.54E-01
ENSBTAG00000044809	0.00E+00	1.00E+00	0.00E+00	1.00E+00	-5.13E-15	1.00E+00	0.00E+00	1.00E+00	0.00E+00	1.00E+00
ENSBTAG00000028588	0.00E+00	1.00E+00	0.00E+00	1.00E+00	-5.13E-15	1.00E+00	0.00E+00	1.00E+00	1.91E+00	8.00E-01
ENSBTAG00000007840	-8.48E-01	5.43E-01	-1.84E+00	9.86E-04	-2.08E+00	1.76E-04	-2.25E+00	5.92E-05	-1.33E+00	1.69E-01
ENSBTAG00000000081	5.93E-02	1.00E+00	3.63E-01	3.63E-01	-2.74E-01	5.44E-01	3.80E-01	3.37E-01	2.45E-01	9.46E-01
ENSBTAG00000004028	3.43E-01	8.23E-01	1.88E+00	2.77E-10	9.16E-01	3.35E-03	3.07E-01	4.97E-01	-5.79E-02	1.00E+00
ENSBTAG00000044151	2.44E-01	1.00E+00	-1.72E-01	1.00E+00	6.48E-01	4.66E-01	-7.75E-02	1.00E+00	7.76E-01	7.63E-01
ENSBTAG00000043085	0.00E+00	1.00E+00	0.00E+00	1.00E+00	-5.13E-15	1.00E+00	0.00E+00	1.00E+00	0.00E+00	1.00E+00
ENSBTAG00000043356	0.00E+00	1.00E+00	0.00E+00	1.00E+00	-5.13E-15	1.00E+00	0.00E+00	1.00E+00	0.00E+00	1.00E+00
ENSBTAG00000007114	-5.04E-02	1.00E+00	-9.61E-03	1.00E+00	-2.99E-02	1.00E+00	-1.95E-01	6.84E-01	4.39E-02	1.00E+00
ENSBTAG00000017507	2.55E-02	1.00E+00	-4.44E-01	6.58E-01	-2.79E-01	8.87E-01	3.21E-01	7.87E-01	3.36E-01	1.00E+00
ENSBTAG00000000897	5.64E-03	1.00E+00	-6.47E-01	3.56E-02	-1.04E+00	2.55E-04	-2.66E-01	5.45E-01	1.44E-01	1.00E+00
ENSBTAG00000000898	0.00E+00	1.00E+00	0.00E+00	1.00E+00	-5.13E-15	1.00E+00	0.00E+00	1.00E+00	0.00E+00	1.00E+00
ENSBTAG00000020199	9.40E-01	2.94E-02	8.25E-01	1.56E-02	1.72E-04	1.00E+00	-1.66E-01	9.02E-01	-4.44E-01	6.78E-01
ENSBTAG00000034848	-7.97E-01	6.67E-01	-7.89E-01	2.99E-01	-7.39E-01	3.12E-01	-1.54E-01	1.00E+00	-1.10E-01	1.00E+00
ENSBTAG00000020201	1.91E+00	8.23E-01	0.00E+00	1.00E+00	2.55E+00	2.54E-01	2.54E+00	2.71E-01	0.00E+00	1.00E+00
ENSBTAG00000020202	-3.22E+00	3.75E-01	-3.22E+00	1.25E-01	-1.44E+00	4.79E-01	-1.46E+00	4.92E-01	-5.13E-01	1.00E+00
ENSBTAG00000034823	-2.06E-01	9.44E-01	-5.60E-01	2.58E-02	-1.18E+00	2.63E-07	-1.59E+00	4.04E-12	3.29E-02	1.00E+00
ENSBTAG00000045134	3.89E+00	1.40E-01	4.44E+00	6.71E-03	1.78E+00	4.79E-01	3.95E+00	2.62E-02	0.00E+00	1.00E+00
ENSBTAG00000042478	0.00E+00	1.00E+00	2.79E+00	2.10E-01	3.42E+00	7.40E-02	1.76E+00	4.97E-01	1.91E+00	8.00E-01
ENSBTAG00000020205	3.21E-02	1.00E+00	-6.60E-01	9.28E-02	-1.25E+00	4.02E-04	-7.29E-01	5.96E-02	3.18E-02	1.00E+00

ENSBTAG00000020212	-1.29E-01	1.00E+00	-8.18E-01	3.60E-03	4.56E-02	1.00E+00	-4.66E-02	1.00E+00	-1.14E-01	1.00E+00
ENSBTAG00000009249	-7.93E-01	1.00E+00	1.26E+00	4.53E-01	-1.55E-01	1.00E+00	-9.47E-01	8.05E-01	5.12E-01	1.00E+00
ENSBTAG00000003263	-6.33E-03	1.00E+00	-9.49E-02	9.45E-01	-3.79E-01	1.09E-01	-5.82E-01	7.58E-03	-4.17E-01	3.36E-01
ENSBTAG00000005016	1.88E-01	9.86E-01	1.16E-01	9.07E-01	4.09E-01	1.19E-01	7.62E-01	1.10E-03	-3.68E-02	1.00E+00
ENSBTAG00000043733	0.00E+00	1.00E+00	0.00E+00	1.00E+00	-5.13E-15	1.00E+00	0.00E+00	1.00E+00	0.00E+00	1.00E+00
ENSBTAG00000028061	0.00E+00	1.00E+00	0.00E+00	1.00E+00	-5.13E-15	1.00E+00	0.00E+00	1.00E+00	0.00E+00	1.00E+00
ENSBTAG00000007371	7.35E-02	1.00E+00	-9.44E-01	7.08E-05	-3.63E-01	1.99E-01	2.86E-01	3.67E-01	-2.59E-01	8.00E-01
ENSBTAG00000007374	-6.18E-01	8.23E-01	6.95E-01	2.84E-01	6.84E-01	2.76E-01	5.85E-01	4.11E-01	2.02E-02	1.00E+00
ENSBTAG00000008341	-5.01E-03	1.00E+00	-4.27E-01	1.25E-01	-3.38E-01	2.56E-01	2.76E-02	1.00E+00	-4.32E-01	4.41E-01
ENSBTAG00000002110	2.92E-01	8.23E-01	-4.41E-01	1.60E-01	-1.08E+00	4.24E-05	-1.35E+00	2.93E-07	5.44E-01	3.19E-01
ENSBTAG00000038234	-4.54E-02	1.00E+00	-3.16E+00	1.51E-11	-4.18E+00	5.62E-18	-2.34E+00	4.20E-07	4.50E-01	8.34E-01
ENSBTAG00000002255	9.44E-01	1.22E-01	-9.44E-01	2.50E-02	-3.71E+00	1.00E-19	-1.67E+00	3.06E-05	1.69E+00	5.87E-04
ENSBTAG00000025856	2.37E-01	1.00E+00	9.13E-01	1.40E-01	7.42E-01	2.63E-01	1.49E+00	7.80E-03	4.38E-01	9.50E-01
ENSBTAG00000025853	6.09E-02	1.00E+00	8.72E-01	7.84E-02	-3.45E-01	6.60E-01	-7.67E-01	1.52E-01	-2.25E-01	1.00E+00
ENSBTAG00000006751	1.26E-01	1.00E+00	5.12E-01	5.61E-02	9.28E-01	1.21E-04	3.34E-01	2.90E-01	-1.09E-01	1.00E+00
ENSBTAG00000044289	-1.91E+00	8.23E-01	-1.91E+00	4.69E-01	-1.91E+00	4.28E-01	-1.91E+00	4.39E-01	-1.91E+00	8.00E-01
ENSBTAG00000006823	5.64E-01	2.24E-01	1.18E+00	6.66E-06	2.01E-01	6.80E-01	-6.59E-01	2.53E-02	8.81E-01	1.94E-02
ENSBTAG00000013132	9.52E-02	1.00E+00	6.24E-02	1.00E+00	8.48E-01	1.65E-02	6.19E-01	1.15E-01	1.77E-01	1.00E+00
ENSBTAG00000012866	-3.04E-01	1.00E+00	-5.99E-01	7.27E-01	-1.78E+00	1.02E-01	-4.97E-01	8.05E-01	-4.80E-01	1.00E+00
ENSBTAG00000034463	0.00E+00	1.00E+00	0.00E+00	1.00E+00	-5.13E-15	1.00E+00	0.00E+00	1.00E+00	0.00E+00	1.00E+00
ENSBTAG00000001619	2.10E-01	1.00E+00	-4.56E-01	2.55E-01	-2.83E-01	5.69E-01	3.73E-01	4.02E-01	8.50E-01	9.73E-02
ENSBTAG00000005344	6.91E-01	2.30E-01	2.15E+00	3.64E-11	1.82E+00	1.77E-08	1.56E+00	2.28E-06	1.74E-01	1.00E+00
ENSBTAG00000046956	6.83E-01	2.28E-01	1.66E+00	1.68E-07	7.53E-01	3.06E-02	4.79E-01	2.42E-01	1.77E-01	1.00E+00
ENSBTAG00000015732	4.19E-01	2.08E-01	7.21E-01	2.04E-04	2.06E-01	4.59E-01	-7.06E-02	1.00E+00	-2.81E-01	6.27E-01
ENSBTAG00000015716	5.24E-01	6.79E-01	1.58E+00	4.64E-05	1.79E+00	3.04E-06	2.56E+00	3.37E-11	7.44E-01	3.89E-01
ENSBTAG00000000821	-5.03E-01	6.00E-01	-2.25E+00	3.68E-09	-3.82E+00	1.75E-19	-4.02E+00	1.38E-20	4.07E-01	7.84E-01
ENSBTAG00000010399	7.17E-02	1.00E+00	-9.17E-01	6.30E-03	-1.48E+00	4.34E-06	-1.32E+00	5.57E-05	4.60E-01	6.01E-01
ENSBTAG00000043148	0.00E+00	1.00E+00	0.00E+00	1.00E+00	-5.13E-15	1.00E+00	0.00E+00	1.00E+00	0.00E+00	1.00E+00
ENSBTAG00000002163	-2.79E-01	1.00E+00	3.41E-02	1.00E+00	3.78E-01	7.08E-01	3.58E-01	7.55E-01	-7.76E-01	7.51E-01
ENSBTAG00000019642	1.19E-02	1.00E+00	9.35E-02	1.00E+00	5.13E-01	9.39E-01	4.80E-01	9.69E-01	3.74E-01	1.00E+00
ENSBTAG00000011540	-3.28E-01	6.06E-01	-1.82E+00	3.68E-16	-2.16E+00	3.78E-22	-1.37E+00	8.41E-10	-2.36E-01	8.08E-01
ENSBTAG00000021630	-5.52E-01	2.03E-01	-1.81E+00	1.14E-12	-9.48E-01	2.15E-04	-4.32E-01	1.57E-01	-3.55E-01	6.52E-01
ENSBTAG00000003737	-6.72E-01	7.24E-02	-1.90E+00	8.30E-14	-3.58E+00	4.20E-40	-2.24E+00	1.73E-18	-1.76E-01	1.00E+00
ENSBTAG00000000062	-9.72E-01	3.18E-01	-1.79E+00	1.62E-03	-2.37E+00	2.95E-05	-1.25E+00	2.43E-02	5.11E-01	8.00E-01
ENSBTAG00000017471	-1.91E+00	8.23E-01	-1.91E+00	4.69E-01	-1.25E-01	1.00E+00	-1.91E+00	4.39E-01	-1.91E+00	8.00E-01

ENSBTAG00000039658	7.58E-01	3.64E-02	1.52E+00	3.98E-09	4.37E-01	1.68E-01	-3.29E-01	3.80E-01	7.71E-02	1.00E+00
ENSBTAG00000008386	-3.80E-01	3.93E-01	-1.87E+00	4.34E-19	-1.62E+00	5.86E-15	-8.99E-01	2.77E-05	1.58E-01	1.00E+00
ENSBTAG00000002821	8.47E-01	3.57E-01	8.11E-01	1.28E-01	-2.59E-01	8.81E-01	-1.13E+00	5.73E-02	1.63E+00	5.27E-03
ENSBTAG00000012352	-1.57E-01	1.00E+00	-3.65E-01	1.66E-01	-4.52E-01	6.57E-02	-6.05E-02	1.00E+00	2.84E-01	7.31E-01
ENSBTAG00000042594	0.00E+00	1.00E+00	0.00E+00	1.00E+00	-5.13E-15	1.00E+00	0.00E+00	1.00E+00	0.00E+00	1.00E+00
ENSBTAG00000042190	-3.23E+00	3.75E-01	-1.24E+00	6.06E-01	-3.23E+00	9.35E-02	-6.91E-01	8.81E-01	-1.31E+00	8.99E-01
ENSBTAG00000008828	-3.92E-01	1.00E+00	-1.23E+00	3.66E-01	-1.11E+00	3.87E-01	-3.84E-01	9.56E-01	-6.26E-01	1.00E+00
ENSBTAG00000006440	-2.22E-01	1.00E+00	-2.82E+00	7.66E-06	-4.13E+00	6.60E-10	-2.02E+00	1.22E-03	7.67E-01	6.95E-01
ENSBTAG00000014807	-7.50E-02	1.00E+00	-7.04E-01	2.07E-03	-5.75E-01	1.45E-02	-7.01E-01	2.50E-03	1.48E-01	1.00E+00
ENSBTAG00000015155	2.25E-01	9.48E-01	1.23E+00	1.32E-06	1.33E+00	1.10E-07	8.68E-01	1.04E-03	8.28E-02	1.00E+00
ENSBTAG00000042761	0.00E+00	1.00E+00	0.00E+00	1.00E+00	-5.13E-15	1.00E+00	0.00E+00	1.00E+00	0.00E+00	1.00E+00
ENSBTAG00000015158	-4.63E-02	1.00E+00	8.06E-02	1.00E+00	-1.44E-02	1.00E+00	-2.13E-01	7.06E-01	2.51E-01	9.14E-01
ENSBTAG00000025826	5.65E-01	9.60E-01	1.66E+00	6.70E-03	1.21E-01	1.00E+00	1.74E-01	1.00E+00	1.68E-02	1.00E+00
ENSBTAG00000017095	-2.39E-01	8.82E-01	-1.12E+00	4.32E-06	-8.13E-01	1.11E-03	-2.72E-01	4.39E-01	4.97E-01	3.26E-01
ENSBTAG00000044258	1.26E-02	1.00E+00	-1.91E+00	4.69E-01	-1.91E+00	4.28E-01	-1.91E+00	4.39E-01	-1.91E+00	8.00E-01
ENSBTAG00000018959	1.08E-01	1.00E+00	1.35E-02	1.00E+00	1.37E-01	8.14E-01	4.27E-01	1.05E-01	-7.50E-02	1.00E+00
ENSBTAG00000034140	9.94E-01	9.32E-01	4.15E-01	1.00E+00	-4.37E-01	9.98E-01	-1.85E-01	1.00E+00	4.57E-01	1.00E+00
ENSBTAG00000042164	0.00E+00	1.00E+00	0.00E+00	1.00E+00	-5.13E-15	1.00E+00	0.00E+00	1.00E+00	0.00E+00	1.00E+00
ENSBTAG00000000371	1.47E-01	1.00E+00	-1.19E+00	1.17E-10	-9.89E-01	8.13E-08	-7.47E-01	9.00E-05	1.82E-01	8.56E-01
ENSBTAG00000000372	3.18E-01	7.80E-01	2.07E+00	1.34E-16	1.30E+00	2.40E-07	7.26E-01	8.45E-03	-2.93E-01	8.00E-01
ENSBTAG00000044425	0.00E+00	1.00E+00	0.00E+00	1.00E+00	-5.13E-15	1.00E+00	0.00E+00	1.00E+00	0.00E+00	1.00E+00
ENSBTAG00000033983	1.86E-01	9.66E-01	7.33E-01	8.87E-04	5.57E-01	1.54E-02	1.79E-01	6.39E-01	-1.62E-01	1.00E+00
ENSBTAG00000005210	1.84E-01	1.00E+00	3.91E-01	1.82E-01	2.64E-01	4.37E-01	-1.99E-01	6.50E-01	-1.94E-01	9.81E-01
ENSBTAG00000005211	8.11E-02	1.00E+00	5.23E-01	1.27E-02	6.28E-01	2.04E-03	7.09E-01	5.01E-04	-6.38E-01	2.66E-02
ENSBTAG00000043531	0.00E+00	1.00E+00	0.00E+00	1.00E+00	-5.13E-15	1.00E+00	0.00E+00	1.00E+00	0.00E+00	1.00E+00
ENSBTAG00000042406	0.00E+00	1.00E+00	0.00E+00	1.00E+00	-5.13E-15	1.00E+00	0.00E+00	1.00E+00	0.00E+00	1.00E+00
ENSBTAG00000043377	0.00E+00	1.00E+00	0.00E+00	1.00E+00	-5.13E-15	1.00E+00	0.00E+00	1.00E+00	0.00E+00	1.00E+00
ENSBTAG00000042233	0.00E+00	1.00E+00	0.00E+00	1.00E+00	-5.13E-15	1.00E+00	0.00E+00	1.00E+00	0.00E+00	1.00E+00
ENSBTAG00000005213	2.12E-01	9.33E-01	1.63E-01	7.41E-01	4.71E-01	7.08E-02	1.82E-01	6.71E-01	-2.18E-02	1.00E+00
ENSBTAG00000005214	-4.69E-01	8.06E-01	-1.03E+00	2.03E-02	-2.74E-01	7.22E-01	-1.05E+00	1.71E-02	-6.10E-01	6.01E-01
ENSBTAG00000044351	0.00E+00	1.00E+00	0.00E+00	1.00E+00	-5.13E-15	1.00E+00	0.00E+00	1.00E+00	0.00E+00	1.00E+00
ENSBTAG00000000625	-9.22E-01	9.01E-02	-1.92E+00	2.97E-07	-4.75E-01	3.06E-01	-3.60E-01	4.97E-01	-5.34E-01	6.04E-01
ENSBTAG00000038176	0.00E+00	1.00E+00	0.00E+00	1.00E+00	-5.13E-15	1.00E+00	0.00E+00	1.00E+00	0.00E+00	1.00E+00
ENSBTAG00000012599	2.98E-01	8.81E-01	1.59E+00	9.28E-08	1.94E+00	4.27E-11	1.24E+00	4.77E-05	7.53E-01	1.56E-01
ENSBTAG00000040368	2.14E-01	1.00E+00	2.62E-01	5.12E-01	8.91E-01	9.92E-04	8.35E-01	2.66E-03	1.81E-01	1.00E+00

ENSBTAG00000013638	1.94E-01	1.00E+00	-1.34E-01	1.00E+00	-2.60E-01	8.10E-01	-2.19E-01	9.03E-01	1.30E-01	1.00E+00
ENSBTAG00000045628	-8.98E-01	1.14E-02	-8.72E-01	2.42E-03	-6.53E-01	2.68E-02	-1.24E+00	1.03E-05	-5.40E-01	3.69E-01
ENSBTAG00000000218	-1.23E-01	1.00E+00	-4.07E-01	9.17E-02	-9.20E-01	1.27E-05	-1.18E+00	2.28E-08	-1.18E-01	1.00E+00
ENSBTAG00000014145	1.04E-03	1.00E+00	-2.99E-01	1.00E+00	-5.45E-01	9.27E-01	-5.73E-01	9.11E-01	-8.82E-01	1.00E+00
ENSBTAG00000042586	0.00E+00	1.00E+00	0.00E+00	1.00E+00	-5.13E-15	1.00E+00	0.00E+00	1.00E+00	0.00E+00	1.00E+00
ENSBTAG00000044704	0.00E+00	1.00E+00	0.00E+00	1.00E+00	-5.13E-15	1.00E+00	0.00E+00	1.00E+00	0.00E+00	1.00E+00
ENSBTAG00000005564	4.68E-01	2.12E-01	5.57E-01	1.57E-02	6.19E-01	5.78E-03	4.46E-01	7.11E-02	1.10E-01	1.00E+00
ENSBTAG00000025822	-1.90E-01	1.00E+00	-1.06E+00	2.84E-04	-1.71E+00	1.92E-09	-7.89E-01	9.92E-03	1.16E-01	1.00E+00
ENSBTAG00000005565	-2.80E-01	9.24E-01	-1.14E+00	2.37E-04	-1.58E+00	2.06E-07	-1.27E+00	4.01E-05	1.03E-03	1.00E+00
ENSBTAG00000008378	-8.39E-01	7.84E-03	-1.87E+00	6.24E-14	-7.53E-01	3.87E-03	-5.34E-01	6.11E-02	-4.70E-01	4.27E-01
ENSBTAG00000008380	8.44E-01	3.75E-01	2.86E-01	8.38E-01	5.29E-01	4.20E-01	3.87E-01	6.35E-01	-5.35E-01	8.25E-01
ENSBTAG00000044379	0.00E+00	1.00E+00	0.00E+00	1.00E+00	-5.13E-15	1.00E+00	0.00E+00	1.00E+00	0.00E+00	1.00E+00
ENSBTAG00000045276	0.00E+00	1.00E+00	0.00E+00	1.00E+00	-5.13E-15	1.00E+00	0.00E+00	1.00E+00	0.00E+00	1.00E+00
ENSBTAG00000012681	1.95E-01	1.00E+00	-2.73E+00	1.65E-08	-1.54E+00	1.75E-04	-1.45E+00	4.84E-04	-6.79E-01	4.64E-01
ENSBTAG00000045866	0.00E+00	1.00E+00	0.00E+00	1.00E+00	-5.13E-15	1.00E+00	1.76E+00	4.97E-01	0.00E+00	1.00E+00
ENSBTAG00000012365	-2.67E-01	8.70E-01	-1.54E+00	6.86E-09	-1.64E+00	5.30E-10	-8.11E-01	4.05E-03	-9.44E-02	1.00E+00
ENSBTAG00000044691	0.00E+00	1.00E+00	0.00E+00	1.00E+00	-5.13E-15	1.00E+00	0.00E+00	1.00E+00	0.00E+00	1.00E+00
ENSBTAG00000005994	1.87E-02	1.00E+00	-3.88E+00	3.62E-02	-3.88E+00	2.26E-02	-8.41E-01	6.71E-01	-6.63E-01	1.00E+00
ENSBTAG00000009030	8.05E-01	1.00E+00	1.39E+00	5.18E-01	1.80E+00	2.83E-01	2.41E+00	8.56E-02	-4.45E-05	1.00E+00
ENSBTAG00000001405	7.12E-01	1.41E-01	-1.17E+00	1.51E-04	-1.02E+00	1.13E-03	5.63E-01	1.14E-01	7.99E-01	1.16E-01
ENSBTAG00000020352	8.26E-01	1.33E-01	7.20E-01	6.32E-02	4.04E-01	4.04E-01	3.19E-01	5.63E-01	1.37E+00	2.57E-03
ENSBTAG00000009983	5.76E-01	7.96E-01	7.65E-01	1.90E-01	4.22E-01	5.83E-01	-5.16E-01	4.67E-01	1.55E+00	2.88E-02
ENSBTAG00000018320	2.13E-02	1.00E+00	3.57E-02	1.00E+00	-4.09E-01	1.54E-01	-3.63E-01	2.37E-01	-1.65E-01	1.00E+00
ENSBTAG00000015580	7.53E-01	4.35E-02	1.72E+00	4.04E-11	2.31E+00	4.13E-19	1.02E+00	1.85E-04	-3.48E-01	7.58E-01
ENSBTAG00000030439	0.00E+00	1.00E+00	1.98E+00	4.43E-01	-5.13E-15	1.00E+00	0.00E+00	1.00E+00	2.71E+00	6.01E-01
ENSBTAG00000013982	-1.11E-01	1.00E+00	-1.44E+00	4.34E-10	-1.19E+00	2.13E-07	-8.90E-01	1.87E-04	-1.96E-01	9.40E-01
ENSBTAG00000003915	1.07E-01	1.00E+00	-7.39E-01	4.43E-01	7.45E-01	3.34E-01	7.15E-01	3.78E-01	-8.10E-01	7.92E-01
ENSBTAG00000040208	-3.21E+00	3.75E-01	-1.23E+00	6.11E-01	-1.44E+00	4.79E-01	-1.85E-01	1.00E+00	-4.96E-01	1.00E+00
ENSBTAG00000010944	-3.50E-02	1.00E+00	-1.89E-01	1.00E+00	2.32E-02	1.00E+00	2.84E-01	8.63E-01	-1.58E-01	1.00E+00
ENSBTAG00000011644	2.15E-02	1.00E+00	1.00E-01	1.00E+00	3.92E-01	8.22E-01	4.34E-01	7.74E-01	4.18E-01	1.00E+00
ENSBTAG00000014236	1.91E+00	8.23E-01	2.79E+00	2.10E-01	-5.13E-15	1.00E+00	2.53E+00	2.71E-01	0.00E+00	1.00E+00
ENSBTAG00000007433	2.63E-01	8.29E-01	-6.31E-01	2.48E-02	9.19E-04	1.00E+00	-2.75E-01	4.59E-01	-2.61E-01	8.29E-01
ENSBTAG00000033344	2.39E-01	1.00E+00	2.66E-01	6.67E-01	1.14E+00	5.09E-04	9.18E-01	8.16E-03	-3.64E-02	1.00E+00
ENSBTAG00000046391	4.84E+00	9.29E-03	3.30E+00	1.02E-01	3.42E+00	7.43E-02	1.77E+00	4.97E-01	1.91E+00	8.00E-01
ENSBTAG00000001601	2.96E-01	8.23E-01	8.52E-01	1.61E-03	1.22E+00	2.67E-06	1.49E+00	9.18E-09	-5.19E-02	1.00E+00

ENSBTAG00000005753	3.26E-02	1.00E+00	5.82E-01	7.79E-03	1.09E+00	8.03E-08	1.26E-01	8.27E-01	-1.25E-02	1.00E+00
ENSBTAG00000014092	-4.08E-01	6.17E-01	-4.79E-01	1.61E-01	-8.48E-01	4.44E-03	-3.29E-01	4.12E-01	2.05E-01	1.00E+00
ENSBTAG00000012981	9.86E-02	1.00E+00	-2.92E-01	3.23E-01	-4.69E-01	6.07E-02	-4.48E-01	8.35E-02	1.08E-01	1.00E+00
ENSBTAG00000025814	3.18E-01	1.00E+00	-7.85E-01	1.70E-01	-4.19E-01	5.41E-01	-3.24E-01	7.20E-01	1.72E-01	1.00E+00
ENSBTAG00000006708	9.53E-02	1.00E+00	-8.19E-03	1.00E+00	2.91E-03	1.00E+00	-4.71E-02	1.00E+00	2.32E-01	8.02E-01
ENSBTAG00000007614	-1.12E-01	1.00E+00	3.91E-01	7.56E-02	3.43E-01	1.33E-01	4.92E-02	1.00E+00	-2.72E-01	6.69E-01
ENSBTAG00000001578	-1.09E-02	1.00E+00	9.78E-01	5.28E-06	1.31E+00	4.14E-10	9.87E-01	4.89E-06	-1.32E-01	1.00E+00
ENSBTAG00000043475	0.00E+00	1.00E+00	0.00E+00	1.00E+00	-5.13E-15	1.00E+00	0.00E+00	1.00E+00	0.00E+00	1.00E+00
ENSBTAG00000004990	-1.25E-01	1.00E+00	-9.59E-01	1.38E-04	-6.54E-01	1.14E-02	3.62E-01	2.31E-01	2.93E-01	7.82E-01
ENSBTAG00000017466	8.02E-01	8.23E-01	1.07E+00	2.30E-01	2.13E+00	5.88E-04	2.78E+00	1.10E-06	1.09E+00	6.01E-01
ENSBTAG00000045823	0.00E+00	1.00E+00	0.00E+00	1.00E+00	-5.13E-15	1.00E+00	0.00E+00	1.00E+00	0.00E+00	1.00E+00
ENSBTAG00000008218	9.45E-02	1.00E+00	2.28E-02	1.00E+00	-5.96E-03	1.00E+00	2.37E-01	4.50E-01	1.85E-01	9.31E-01
ENSBTAG00000019734	1.11E-01	1.00E+00	1.38E+00	7.89E-03	2.29E+00	4.71E-06	2.40E+00	2.24E-06	5.02E-01	8.27E-01
ENSBTAG00000025803	-2.72E-01	1.00E+00	-1.16E+00	1.52E-03	-1.22E+00	6.24E-04	-5.58E-01	1.79E-01	-4.67E-01	6.83E-01
ENSBTAG00000007800	3.21E-01	1.00E+00	-1.53E+00	8.67E-02	-1.25E+00	1.42E-01	-9.91E-01	2.75E-01	5.57E-01	9.00E-01
ENSBTAG00000004788	-4.57E-01	2.18E-01	-1.12E+00	1.71E-07	-1.81E+00	3.20E-17	-2.08E+00	1.18E-21	4.58E-03	1.00E+00
ENSBTAG00000010666	-5.02E-01	3.75E-01	-6.70E-01	2.30E-02	-4.36E-01	1.89E-01	-1.35E-01	9.25E-01	3.33E-02	1.00E+00
ENSBTAG00000010665	-1.79E-01	1.00E+00	7.07E-01	5.81E-01	2.43E-01	1.00E+00	-4.63E-01	8.49E-01	-2.67E-01	1.00E+00
ENSBTAG00000007149	-1.72E+00	5.45E-03	-4.43E+00	2.25E-15	-2.91E+00	1.32E-08	-2.43E+00	1.96E-06	-2.50E-01	1.00E+00
ENSBTAG00000006366	-6.97E-01	6.28E-01	-2.46E+00	1.38E-06	-2.35E+00	3.51E-06	-1.02E+00	6.95E-02	-1.91E-01	1.00E+00
ENSBTAG00000018422	2.88E-01	8.23E-01	1.91E-01	6.95E-01	2.31E-01	5.52E-01	4.01E-02	1.00E+00	7.60E-02	1.00E+00
ENSBTAG00000018419	-3.05E-01	9.19E-01	-7.66E-01	4.11E-02	-5.81E-01	1.30E-01	-7.33E-01	4.92E-02	-6.79E-02	1.00E+00
ENSBTAG00000010238	-4.43E-01	7.80E-01	-1.58E+00	4.74E-05	-1.97E+00	2.93E-07	-1.39E+00	3.20E-04	-3.79E-01	8.09E-01
ENSBTAG00000040056	-7.33E-01	6.11E-01	-2.49E+00	3.43E-06	-2.95E+00	4.47E-08	-1.66E+00	2.20E-03	-3.35E-01	1.00E+00
ENSBTAG00000010236	-6.18E-01	2.48E-01	-1.72E+00	7.60E-09	-3.50E+00	1.40E-29	-2.44E+00	4.72E-16	-3.14E-02	1.00E+00
ENSBTAG00000010235	-2.24E-01	8.44E-01	1.66E+00	3.55E-16	1.44E+00	1.37E-12	9.48E-01	7.02E-06	-1.84E-01	9.37E-01
ENSBTAG00000010234	-1.58E-01	1.00E+00	-3.62E-01	2.59E-01	-6.68E-01	1.32E-02	-6.49E-01	1.90E-02	1.26E-01	1.00E+00
ENSBTAG00000018796	-4.32E-01	3.50E-01	-1.10E+00	2.21E-06	-1.09E+00	1.85E-06	-7.28E-01	2.45E-03	1.30E-02	1.00E+00
ENSBTAG00000003920	3.88E-01	9.96E-01	1.78E+00	4.74E-05	-1.85E-01	1.00E+00	-2.41E-01	9.27E-01	7.50E-02	1.00E+00
ENSBTAG00000002718	-2.78E-01	7.71E-01	1.36E-01	8.18E-01	4.84E-01	4.25E-02	3.18E-01	2.59E-01	1.85E-01	9.45E-01
ENSBTAG00000002715	-4.92E-02	1.00E+00	-1.99E-01	5.02E-01	-2.67E-01	3.03E-01	-4.16E-01	6.97E-02	-8.11E-02	1.00E+00
ENSBTAG00000038842	-9.64E-02	1.00E+00	-3.40E-01	2.58E-01	-4.02E-01	1.55E-01	-4.96E-01	6.84E-02	8.48E-03	1.00E+00
ENSBTAG00000002712	1.55E-01	1.00E+00	-6.57E-01	5.83E-02	-7.88E-01	1.72E-02	-9.90E-01	2.46E-03	-1.31E-01	1.00E+00
ENSBTAG00000039415	-5.24E-02	1.00E+00	-1.68E-01	7.47E-01	-5.51E-01	3.90E-02	-6.91E-01	8.11E-03	1.11E-01	1.00E+00
ENSBTAG00000002710	-2.39E-01	1.00E+00	3.05E-01	1.00E+00	-1.63E-01	1.00E+00	1.04E-02	1.00E+00	3.95E-01	1.00E+00

ENSBTAG00000002709	1.77E-01	1.00E+00	-1.02E-01	9.50E-01	3.26E-01	2.39E-01	7.57E-01	9.75E-04	2.51E-01	8.00E-01
ENSBTAG00000002707	6.20E-02	1.00E+00	1.80E+00	2.51E-13	2.58E+00	1.61E-25	2.31E+00	6.44E-21	-3.64E-01	6.45E-01
ENSBTAG00000002705	7.39E-01	6.42E-01	3.25E+00	7.04E-11	1.64E+00	1.69E-03	8.39E-02	1.00E+00	7.96E-01	6.01E-01
ENSBTAG00000005816	2.91E-01	9.90E-01	8.74E-01	2.16E-02	-2.04E-01	8.39E-01	-7.24E-01	7.56E-02	2.18E-01	1.00E+00
ENSBTAG00000005815	-3.26E-01	7.56E-01	-1.23E+00	1.99E-06	-2.34E-01	5.60E-01	-2.08E-01	6.51E-01	2.49E-01	8.60E-01
ENSBTAG00000005814	-4.28E-02	1.00E+00	2.87E-01	3.61E-01	4.69E-01	7.31E-02	3.84E-01	1.77E-01	-2.62E-01	8.00E-01
ENSBTAG00000025778	-2.83E-01	8.23E-01	-8.07E-01	2.06E-03	-1.62E+00	1.11E-10	-1.92E+00	4.36E-14	-3.10E-01	7.78E-01
ENSBTAG00000021395	-1.02E-02	1.00E+00	3.93E-01	1.64E-01	4.92E-01	6.22E-02	5.22E-01	4.97E-02	-2.12E-01	9.04E-01
ENSBTAG00000021394	-4.46E-01	7.04E-01	-1.42E+00	3.68E-05	-1.36E+00	7.31E-05	-1.31E+00	1.61E-04	4.95E-01	6.03E-01
ENSBTAG00000021392	-2.11E-01	9.06E-01	-1.53E+00	2.18E-12	-1.99E+00	7.20E-20	-1.24E+00	1.87E-08	5.40E-01	1.80E-01
ENSBTAG00000011934	-3.62E-01	8.23E-01	-8.73E-01	8.28E-03	-2.21E+00	1.41E-12	-2.48E+00	4.00E-15	4.04E-01	7.43E-01
ENSBTAG00000001871	-6.19E-04	1.00E+00	-1.91E+00	4.69E-01	-1.91E+00	4.28E-01	-1.91E+00	4.39E-01	-1.91E+00	8.00E-01
ENSBTAG00000001870	3.22E+00	3.75E-01	3.68E+00	5.09E-02	2.56E+00	2.54E-01	2.53E+00	2.71E-01	1.93E+00	8.00E-01
ENSBTAG00000015208	2.14E-02	1.00E+00	-9.58E-01	1.27E-02	-2.89E+00	7.97E-15	-3.50E+00	5.78E-20	5.81E-01	5.53E-01
ENSBTAG00000017665	-3.19E-01	6.66E-01	-8.57E-01	2.54E-04	-1.85E+00	3.41E-16	-2.50E+00	1.56E-27	-6.07E-02	1.00E+00
ENSBTAG00000045993	-4.84E-02	1.00E+00	-1.36E+00	6.36E-03	-2.78E+00	4.76E-08	-2.02E+00	4.13E-05	-1.53E-01	1.00E+00
ENSBTAG00000030841	-9.16E-01	9.56E-01	-2.16E+00	1.57E-01	-2.37E+00	9.72E-02	-2.39E+00	1.00E-01	-1.44E+00	7.58E-01
ENSBTAG00000007104	-7.79E-01	1.00E+00	-2.70E+00	2.42E-01	-1.42E-01	1.00E+00	-2.70E+00	2.05E-01	-2.70E+00	6.01E-01
ENSBTAG00000007099	-3.47E-01	6.21E-01	-1.25E+00	6.62E-07	-7.43E-01	3.31E-03	-7.53E-01	3.41E-03	2.80E-01	8.00E-01
ENSBTAG00000007097	-2.30E-01	9.28E-01	-1.14E+00	8.42E-06	-3.23E-01	3.22E-01	1.55E-01	8.06E-01	3.79E-01	6.01E-01
ENSBTAG00000007053	6.71E-01	6.03E-01	6.95E-01	2.25E-01	-2.08E-01	9.74E-01	4.96E-01	4.63E-01	5.80E-01	7.69E-01
ENSBTAG00000009595	2.53E-01	8.13E-01	1.50E+00	2.05E-13	1.24E+00	1.30E-09	7.76E-01	3.33E-04	-2.65E-01	7.81E-01
ENSBTAG00000009703	1.40E-02	1.00E+00	1.40E+00	3.78E-01	-1.04E+00	5.65E-01	1.14E+00	5.12E-01	5.39E-01	1.00E+00
ENSBTAG00000037339	0.00E+00	1.00E+00	0.00E+00	1.00E+00	-5.13E-15	1.00E+00	0.00E+00	1.00E+00	0.00E+00	1.00E+00
ENSBTAG00000040053	-1.23E+00	6.86E-01	5.25E-01	7.98E-01	-1.73E+00	1.06E-01	1.27E-01	1.00E+00	-6.66E-01	9.98E-01
ENSBTAG00000029863	0.00E+00	1.00E+00	0.00E+00	1.00E+00	-5.13E-15	1.00E+00	0.00E+00	1.00E+00	0.00E+00	1.00E+00
ENSBTAG00000032709	0.00E+00	1.00E+00	0.00E+00	1.00E+00	-5.13E-15	1.00E+00	1.77E+00	4.97E-01	0.00E+00	1.00E+00
ENSBTAG00000009701	0.00E+00	1.00E+00	1.98E+00	4.43E-01	-5.13E-15	1.00E+00	0.00E+00	1.00E+00	2.72E+00	6.01E-01
ENSBTAG00000038733	1.20E+00	5.72E-01	3.12E-01	1.00E+00	9.12E-01	4.09E-01	1.33E+00	1.34E-01	4.19E-01	1.00E+00
ENSBTAG00000016467	-6.55E-01	1.74E-01	-8.89E-01	3.96E-03	-1.80E+00	2.99E-09	-1.91E+00	5.51E-10	-6.69E-02	1.00E+00
ENSBTAG00000006884	-6.58E-02	1.00E+00	-1.70E-02	1.00E+00	1.91E-01	5.99E-01	-3.95E-01	1.39E-01	-7.58E-02	1.00E+00
ENSBTAG00000019692	-3.20E-02	1.00E+00	-5.88E-01	1.25E-01	-4.37E-01	3.03E-01	1.36E-03	1.00E+00	3.58E-01	8.00E-01
ENSBTAG00000037679	-6.72E-01	1.23E-01	7.17E-01	8.60E-03	1.05E+00	3.70E-05	9.86E-01	1.44E-04	-2.87E-01	8.02E-01
ENSBTAG00000032686	-1.51E-01	1.00E+00	-1.07E+00	3.83E-05	-9.79E-01	1.35E-04	-7.58E-01	4.50E-03	-2.36E-01	8.82E-01
ENSBTAG00000025755	2.00E-01	1.00E+00	-7.62E-01	4.72E-01	-1.04E+00	2.60E-01	-8.62E-01	3.98E-01	6.67E-01	8.27E-01

ENSBTAG00000007415	8.77E-01	8.58E-01	-6.00E-01	7.69E-01	4.14E-01	9.33E-01	-2.25E-01	1.00E+00	4.32E-01	1.00E+00
ENSBTAG00000043704	0.00E+00	1.00E+00	0.00E+00	1.00E+00	-5.13E-15	1.00E+00	0.00E+00	1.00E+00	0.00E+00	1.00E+00
ENSBTAG00000042621	0.00E+00	1.00E+00	0.00E+00	1.00E+00	-5.13E-15	1.00E+00	0.00E+00	1.00E+00	0.00E+00	1.00E+00
ENSBTAG00000005952	3.88E+00	1.42E-01	1.98E+00	4.43E-01	2.56E+00	2.54E-01	3.04E+00	1.49E-01	2.71E+00	6.01E-01
ENSBTAG00000047027	3.52E-01	4.65E-01	1.23E+00	1.20E-09	1.05E+00	2.09E-07	6.64E-01	2.18E-03	5.72E-02	1.00E+00
ENSBTAG00000011571	8.53E-02	1.00E+00	1.82E-01	5.37E-01	2.20E-01	4.20E-01	5.46E-02	1.00E+00	2.37E-01	7.79E-01
ENSBTAG00000011349	2.12E-01	1.00E+00	1.77E-01	9.68E-01	-7.19E-01	1.56E-01	-2.82E-01	7.53E-01	4.21E-01	8.00E-01
ENSBTAG00000012728	-2.71E+00	5.76E-01	-7.27E-01	9.45E-01	-9.29E-01	8.06E-01	-2.71E+00	2.05E-01	-2.71E+00	6.01E-01
ENSBTAG00000012726	-1.32E-01	1.00E+00	-8.95E-02	1.00E+00	-1.25E-02	1.00E+00	2.12E-01	5.65E-01	-3.78E-01	5.43E-01
ENSBTAG00000012725	-1.71E-01	1.00E+00	-2.63E+00	1.08E-14	-2.59E+00	6.83E-15	-1.56E+00	1.40E-06	1.44E-01	1.00E+00
ENSBTAG00000012724	-1.07E+00	1.00E-02	-2.15E+00	5.81E-11	-3.03E+00	1.24E-19	-1.09E+00	1.38E-03	-1.50E-01	1.00E+00
ENSBTAG00000012723	-3.47E-01	7.67E-01	-1.70E+00	1.21E-09	-1.80E+00	9.33E-11	-7.93E-01	7.72E-03	-2.24E-01	9.66E-01
ENSBTAG00000010890	2.35E-01	8.17E-01	1.97E+00	2.44E-24	1.71E+00	4.56E-19	1.52E+00	3.19E-15	-2.23E-01	8.00E-01
ENSBTAG00000038333	-1.87E-02	1.00E+00	6.86E-01	1.44E-03	5.21E-01	2.07E-02	4.23E-01	8.11E-02	2.60E-01	7.77E-01
ENSBTAG00000016381	2.92E-01	1.00E+00	2.34E+00	2.84E-07	8.36E-02	1.00E+00	-5.12E-01	6.08E-01	-4.89E-01	9.69E-01
ENSBTAG00000016378	-1.92E-01	9.62E-01	-1.62E-01	7.18E-01	3.08E-02	1.00E+00	4.62E-01	6.98E-02	3.61E-01	5.52E-01
ENSBTAG00000014824	3.65E-02	1.00E+00	1.37E-01	8.66E-01	2.64E-01	4.45E-01	1.45E-01	8.37E-01	-2.64E-01	8.00E-01
ENSBTAG00000014822	-2.81E-02	1.00E+00	5.58E-01	3.65E-02	6.88E-01	7.01E-03	4.90E-01	8.23E-02	-3.76E-01	6.01E-01
ENSBTAG00000014821	-2.23E-01	9.62E-01	3.06E-01	3.92E-01	2.29E-01	5.73E-01	-5.25E-02	1.00E+00	-6.76E-01	1.30E-01
ENSBTAG00000014820	-3.64E-02	1.00E+00	-1.41E-01	7.52E-01	-1.69E-01	6.27E-01	3.82E-02	1.00E+00	-2.24E-01	8.00E-01
ENSBTAG00000047305	1.12E-02	1.00E+00	8.83E-01	8.52E-01	-1.31E-01	1.00E+00	-1.42E-01	1.00E+00	6.45E-03	1.00E+00
ENSBTAG00000047672	0.00E+00	1.00E+00	1.98E+00	4.43E-01	-5.13E-15	1.00E+00	1.77E+00	4.97E-01	0.00E+00	1.00E+00
ENSBTAG00000046906	0.00E+00	1.00E+00	2.79E+00	2.10E-01	-5.13E-15	1.00E+00	0.00E+00	1.00E+00	0.00E+00	1.00E+00
ENSBTAG00000047893	1.28E-02	1.00E+00	8.83E-01	8.52E-01	1.51E+00	4.29E-01	-1.91E+00	4.39E-01	7.99E-01	1.00E+00
ENSBTAG00000045760	-1.91E+00	8.23E-01	8.82E-01	8.52E-01	-1.31E-01	1.00E+00	-1.48E-01	1.00E+00	-1.91E+00	8.00E-01
ENSBTAG00000009274	-1.16E-01	1.00E+00	-4.57E-01	4.54E-01	-9.14E-01	5.56E-02	-1.16E+00	1.38E-02	-1.54E-01	1.00E+00
ENSBTAG00000047306	1.92E+00	8.23E-01	0.00E+00	1.00E+00	-5.13E-15	1.00E+00	1.76E+00	4.97E-01	0.00E+00	1.00E+00
ENSBTAG00000025604	-1.26E-01	1.00E+00	-1.73E-01	9.45E-01	2.06E-01	8.45E-01	4.42E-02	1.00E+00	-1.29E+00	2.29E-02
ENSBTAG00000047064	-7.41E-02	1.00E+00	1.03E-01	1.00E+00	1.99E-01	7.49E-01	4.31E-01	2.48E-01	-6.45E-01	2.69E-01
ENSBTAG00000047744	-8.94E-02	1.00E+00	1.35E-01	9.72E-01	2.87E-01	5.47E-01	4.91E-01	2.04E-01	-7.60E-01	1.78E-01
ENSBTAG00000016658	-6.22E-01	6.77E-02	-2.23E+00	2.06E-20	-1.68E+00	4.92E-13	1.22E-03	1.00E+00	-6.26E-02	1.00E+00
ENSBTAG00000019077	2.98E-01	8.20E-01	1.09E+00	1.98E-05	1.12E+00	1.02E-05	1.07E+00	3.53E-05	1.28E-01	1.00E+00
ENSBTAG00000000432	-2.03E-01	1.00E+00	-4.37E-01	2.39E-01	-6.88E-01	3.36E-02	-1.03E+00	9.16E-04	-8.75E-01	6.01E-02
ENSBTAG00000000431	0.00E+00	1.00E+00	0.00E+00	1.00E+00	-5.13E-15	1.00E+00	0.00E+00	1.00E+00	0.00E+00	1.00E+00
ENSBTAG00000039402	0.00E+00	1.00E+00	0.00E+00	1.00E+00	-5.13E-15	1.00E+00	0.00E+00	1.00E+00	0.00E+00	1.00E+00

ENSBTAG00000047156	0.00E+00	1.00E+00	0.00E+00	1.00E+00	-5.13E-15	1.00E+00	0.00E+00	1.00E+00	0.00E+00	1.00E+00
ENSBTAG00000046962	0.00E+00	1.00E+00	0.00E+00	1.00E+00	-5.13E-15	1.00E+00	0.00E+00	1.00E+00	0.00E+00	1.00E+00
ENSBTAG00000045595	0.00E+00	1.00E+00	0.00E+00	1.00E+00	-5.13E-15	1.00E+00	0.00E+00	1.00E+00	0.00E+00	1.00E+00
ENSBTAG00000047258	0.00E+00	1.00E+00	0.00E+00	1.00E+00	-5.13E-15	1.00E+00	0.00E+00	1.00E+00	0.00E+00	1.00E+00
ENSBTAG00000045758	0.00E+00	1.00E+00	0.00E+00	1.00E+00	-5.13E-15	1.00E+00	0.00E+00	1.00E+00	0.00E+00	1.00E+00
ENSBTAG00000048302	0.00E+00	1.00E+00	0.00E+00	1.00E+00	-5.13E-15	1.00E+00	0.00E+00	1.00E+00	0.00E+00	1.00E+00
ENSBTAG00000046464	0.00E+00	1.00E+00	0.00E+00	1.00E+00	-5.13E-15	1.00E+00	0.00E+00	1.00E+00	0.00E+00	1.00E+00
ENSBTAG00000047041	0.00E+00	1.00E+00	0.00E+00	1.00E+00	-5.13E-15	1.00E+00	0.00E+00	1.00E+00	0.00E+00	1.00E+00
ENSBTAG00000039077	0.00E+00	1.00E+00	0.00E+00	1.00E+00	-5.13E-15	1.00E+00	0.00E+00	1.00E+00	0.00E+00	1.00E+00
ENSBTAG00000046530	0.00E+00	1.00E+00	0.00E+00	1.00E+00	-5.13E-15	1.00E+00	0.00E+00	1.00E+00	0.00E+00	1.00E+00
ENSBTAG00000047444	0.00E+00	1.00E+00	0.00E+00	1.00E+00	-5.13E-15	1.00E+00	0.00E+00	1.00E+00	0.00E+00	1.00E+00
ENSBTAG00000046682	0.00E+00	1.00E+00	0.00E+00	1.00E+00	-5.13E-15	1.00E+00	0.00E+00	1.00E+00	0.00E+00	1.00E+00
ENSBTAG00000038098	0.00E+00	1.00E+00	0.00E+00	1.00E+00	-5.13E-15	1.00E+00	0.00E+00	1.00E+00	0.00E+00	1.00E+00
ENSBTAG00000047276	0.00E+00	1.00E+00	0.00E+00	1.00E+00	-5.13E-15	1.00E+00	0.00E+00	1.00E+00	0.00E+00	1.00E+00
ENSBTAG00000048064	0.00E+00	1.00E+00	0.00E+00	1.00E+00	-5.13E-15	1.00E+00	0.00E+00	1.00E+00	0.00E+00	1.00E+00
ENSBTAG00000036202	0.00E+00	1.00E+00	0.00E+00	1.00E+00	-5.13E-15	1.00E+00	0.00E+00	1.00E+00	0.00E+00	1.00E+00
ENSBTAG00000038565	4.12E+00	8.63E-02	0.00E+00	1.00E+00	1.78E+00	4.79E-01	0.00E+00	1.00E+00	3.21E+00	4.34E-01
ENSBTAG00000047312	-3.21E+00	3.75E-01	4.78E-01	9.88E-01	-3.21E+00	9.53E-02	-1.44E+00	4.97E-01	-3.21E+00	4.34E-01
ENSBTAG00000047091	0.00E+00	1.00E+00	0.00E+00	1.00E+00	-5.13E-15	1.00E+00	0.00E+00	1.00E+00	0.00E+00	1.00E+00
ENSBTAG00000046477	0.00E+00	1.00E+00	0.00E+00	1.00E+00	-5.13E-15	1.00E+00	1.77E+00	4.97E-01	0.00E+00	1.00E+00
ENSBTAG00000047683	0.00E+00	1.00E+00	0.00E+00	1.00E+00	-5.13E-15	1.00E+00	0.00E+00	1.00E+00	0.00E+00	1.00E+00
ENSBTAG00000046897	0.00E+00	1.00E+00	1.98E+00	4.43E-01	-5.13E-15	1.00E+00	0.00E+00	1.00E+00	1.91E+00	8.00E-01
ENSBTAG00000047001	0.00E+00	1.00E+00	0.00E+00	1.00E+00	-5.13E-15	1.00E+00	0.00E+00	1.00E+00	0.00E+00	1.00E+00
ENSBTAG00000047981	0.00E+00	1.00E+00	0.00E+00	1.00E+00	-5.13E-15	1.00E+00	0.00E+00	1.00E+00	0.00E+00	1.00E+00
ENSBTAG00000046500	1.84E-02	1.00E+00	8.82E-02	1.00E+00	-2.70E+00	1.99E-01	-9.33E-01	8.08E-01	-2.70E+00	6.01E-01
ENSBTAG00000047097	0.00E+00	1.00E+00	0.00E+00	1.00E+00	-5.13E-15	1.00E+00	1.76E+00	4.97E-01	0.00E+00	1.00E+00
ENSBTAG00000045953	0.00E+00	1.00E+00	2.79E+00	2.10E-01	-5.13E-15	1.00E+00	0.00E+00	1.00E+00	0.00E+00	1.00E+00
ENSBTAG00000047909	0.00E+00	1.00E+00	0.00E+00	1.00E+00	-5.13E-15	1.00E+00	0.00E+00	1.00E+00	1.91E+00	8.00E-01
ENSBTAG00000047522	-7.93E-01	1.00E+00	-2.71E+00	2.42E-01	-2.71E+00	1.99E-01	-2.71E+00	2.05E-01	-5.70E-03	1.00E+00
ENSBTAG00000047398	-2.71E+00	5.76E-01	-2.71E+00	2.42E-01	-2.71E+00	1.99E-01	-9.46E-01	8.04E-01	-2.71E+00	6.01E-01
ENSBTAG00000048236	-1.91E+00	8.23E-01	-1.91E+00	4.69E-01	-1.91E+00	4.28E-01	-1.91E+00	4.39E-01	-1.91E+00	8.00E-01
ENSBTAG00000039807	-1.75E+00	3.50E-01	-2.99E+00	1.34E-02	-2.42E+00	2.27E-02	-1.94E+00	6.31E-02	-1.08E+00	7.47E-01
ENSBTAG00000037924	-1.54E-01	1.00E+00	-4.07E-01	9.25E-01	-3.52E-01	9.62E-01	-3.51E-01	9.65E-01	-9.58E-01	8.00E-01
ENSBTAG00000039038	-9.39E-01	8.22E-01	-5.09E+00	6.24E-04	-5.09E+00	2.18E-04	-5.09E+00	2.47E-04	-1.88E+00	3.34E-01
ENSBTAG00000048074	-1.67E+00	5.76E-01	-4.37E+00	1.05E-02	-2.59E+00	5.60E-02	-4.37E+00	6.16E-03	-2.46E+00	3.68E-01

ENSBTAG00000005941	-2.70E+00	5.76E-01	-2.70E+00	2.42E-01	-9.22E-01	8.08E-01	-2.70E+00	2.05E-01	-7.85E-01	1.00E+00
ENSBTAG00000047624	0.00E+00	1.00E+00	0.00E+00	1.00E+00	-5.13E-15	1.00E+00	0.00E+00	1.00E+00	0.00E+00	1.00E+00
ENSBTAG00000046102	-2.71E+00	5.76E-01	-2.71E+00	2.42E-01	-2.71E+00	1.99E-01	-2.71E+00	2.05E-01	-2.71E+00	6.01E-01
ENSBTAG00000047952	-2.70E+00	5.76E-01	-7.14E-01	9.45E-01	-9.17E-01	8.09E-01	-9.33E-01	8.08E-01	-7.85E-01	1.00E+00
ENSBTAG00000047639	0.00E+00	1.00E+00	0.00E+00	1.00E+00	1.78E+00	4.79E-01	1.77E+00	4.97E-01	1.91E+00	8.00E-01
ENSBTAG00000046305	6.62E-03	1.00E+00	6.97E-02	1.00E+00	-1.91E+00	4.28E-01	-1.91E+00	4.39E-01	-1.91E+00	8.00E-01
ENSBTAG00000047096	0.00E+00	1.00E+00	0.00E+00	1.00E+00	1.78E+00	4.79E-01	1.76E+00	4.97E-01	0.00E+00	1.00E+00
ENSBTAG00000046157	0.00E+00	1.00E+00	2.79E+00	2.10E-01	-5.13E-15	1.00E+00	1.76E+00	4.97E-01	0.00E+00	1.00E+00
ENSBTAG00000046546	1.91E+00	8.23E-01	3.68E+00	5.07E-02	-5.13E-15	1.00E+00	0.00E+00	1.00E+00	0.00E+00	1.00E+00
ENSBTAG00000047690	3.90E+00	1.40E-01	1.98E+00	4.43E-01	1.78E+00	4.79E-01	3.03E+00	1.50E-01	0.00E+00	1.00E+00
ENSBTAG00000046802	3.23E+00	3.75E-01	1.98E+00	4.43E-01	1.78E+00	4.79E-01	1.77E+00	4.97E-01	0.00E+00	1.00E+00
ENSBTAG00000047058	5.48E-03	1.00E+00	-2.70E+00	2.42E-01	-2.70E+00	1.99E-01	-2.70E+00	2.05E-01	-2.70E+00	6.01E-01
ENSBTAG00000027170	1.91E+00	8.23E-01	0.00E+00	1.00E+00	1.78E+00	4.79E-01	1.77E+00	4.97E-01	1.91E+00	8.00E-01
ENSBTAG00000035785	-3.88E+00	1.45E-01	-3.88E+00	3.67E-02	-2.10E+00	1.79E-01	-3.88E+00	2.51E-02	-1.96E+00	6.01E-01
ENSBTAG00000040141	0.00E+00	1.00E+00	0.00E+00	1.00E+00	-5.13E-15	1.00E+00	0.00E+00	1.00E+00	0.00E+00	1.00E+00
ENSBTAG00000046819	3.61E+00	2.28E-01	2.79E+00	2.11E-01	1.78E+00	4.79E-01	2.53E+00	2.71E-01	3.59E+00	3.09E-01
ENSBTAG00000038060	5.19E-03	1.00E+00	-1.91E+00	4.69E-01	-1.37E-01	1.00E+00	-1.91E+00	4.39E-01	-1.91E+00	8.00E-01
ENSBTAG00000024659	0.00E+00	1.00E+00	0.00E+00	1.00E+00	-5.13E-15	1.00E+00	0.00E+00	1.00E+00	0.00E+00	1.00E+00
ENSBTAG00000040504	0.00E+00	1.00E+00	0.00E+00	1.00E+00	-5.13E-15	1.00E+00	0.00E+00	1.00E+00	0.00E+00	1.00E+00
ENSBTAG00000022229	8.03E-01	1.00E+00	-1.91E+00	4.69E-01	-1.24E-01	1.00E+00	-1.91E+00	4.39E-01	7.17E-03	1.00E+00
ENSBTAG00000045863	1.92E+00	8.23E-01	0.00E+00	1.00E+00	-5.13E-15	1.00E+00	0.00E+00	1.00E+00	0.00E+00	1.00E+00
ENSBTAG00000046993	1.19E+00	8.55E-01	-2.70E+00	2.42E-01	-2.70E+00	1.99E-01	-2.70E+00	2.05E-01	-7.85E-01	1.00E+00
ENSBTAG00000045883	3.22E+00	3.75E-01	0.00E+00	1.00E+00	2.55E+00	2.54E-01	2.53E+00	2.71E-01	2.73E+00	6.01E-01
ENSBTAG00000047015	-1.91E+00	8.23E-01	-1.91E+00	4.69E-01	-1.91E+00	4.28E-01	-1.91E+00	4.39E-01	-1.91E+00	8.00E-01
ENSBTAG00000033045	-7.92E-01	1.00E+00	-2.71E+00	2.42E-01	-2.71E+00	1.99E-01	-9.45E-01	8.05E-01	1.28E-03	1.00E+00
ENSBTAG00000046995	2.70E+00	5.76E-01	2.79E+00	2.10E-01	-5.13E-15	1.00E+00	0.00E+00	1.00E+00	0.00E+00	1.00E+00
ENSBTAG00000046426	3.22E+00	3.75E-01	2.79E+00	2.10E-01	1.78E+00	4.79E-01	0.00E+00	1.00E+00	0.00E+00	1.00E+00
ENSBTAG00000038544	8.99E-01	1.00E+00	6.00E-01	9.45E-01	-9.17E-01	8.10E-01	6.93E-01	8.58E-01	-7.86E-01	1.00E+00
ENSBTAG00000046361	-1.91E+00	8.23E-01	7.11E-02	1.00E+00	-1.91E+00	4.28E-01	-1.91E+00	4.39E-01	-1.91E+00	8.00E-01
ENSBTAG00000046643	0.00E+00	1.00E+00	1.99E+00	4.43E-01	-5.13E-15	1.00E+00	0.00E+00	1.00E+00	0.00E+00	1.00E+00
ENSBTAG00000033036	5.16E-03	1.00E+00	8.73E-01	8.53E-01	-1.91E+00	4.28E-01	-1.53E-01	1.00E+00	-1.91E+00	8.00E-01
ENSBTAG00000033034	0.00E+00	1.00E+00	0.00E+00	1.00E+00	-5.13E-15	1.00E+00	0.00E+00	1.00E+00	0.00E+00	1.00E+00
ENSBTAG00000035530	1.91E+00	8.23E-01	0.00E+00	1.00E+00	-5.13E-15	1.00E+00	1.76E+00	4.97E-01	0.00E+00	1.00E+00
ENSBTAG00000046478	-1.91E+00	8.23E-01	-1.91E+00	4.69E-01	-1.91E+00	4.28E-01	-1.48E-01	1.00E+00	-1.91E+00	8.00E-01
ENSBTAG00000039546	-2.70E+00	5.76E-01	1.28E+00	4.43E-01	-1.41E-01	1.00E+00	-9.38E-01	8.06E-01	-2.70E+00	6.01E-01

ENSBTAG00000006514	1.91E+00	8.23E-01	0.00E+00	1.00E+00	-5.13E-15	1.00E+00	1.76E+00	4.97E-01	0.00E+00	1.00E+00
ENSBTAG00000011925	0.00E+00	1.00E+00	0.00E+00	1.00E+00	-5.13E-15	1.00E+00	0.00E+00	1.00E+00	0.00E+00	1.00E+00
ENSBTAG00000007029	0.00E+00	1.00E+00	0.00E+00	1.00E+00	-5.13E-15	1.00E+00	0.00E+00	1.00E+00	0.00E+00	1.00E+00
ENSBTAG00000047746	0.00E+00	1.00E+00	0.00E+00	1.00E+00	-5.13E-15	1.00E+00	0.00E+00	1.00E+00	0.00E+00	1.00E+00
ENSBTAG00000047253	0.00E+00	1.00E+00	0.00E+00	1.00E+00	-5.13E-15	1.00E+00	0.00E+00	1.00E+00	0.00E+00	1.00E+00
ENSBTAG00000024752	0.00E+00	1.00E+00	0.00E+00	1.00E+00	1.78E+00	4.79E-01	0.00E+00	1.00E+00	0.00E+00	1.00E+00
ENSBTAG00000013249	-1.28E+00	2.15E-01	-2.17E+00	1.27E-03	-1.70E+00	7.20E-03	-1.37E+00	3.51E-02	-2.33E-01	1.00E+00
ENSBTAG00000015148	1.82E-01	9.03E-01	1.47E+00	5.38E-16	1.08E+00	3.51E-09	2.96E-01	2.13E-01	2.57E-02	1.00E+00
ENSBTAG00000015146	4.03E-01	3.58E-01	1.77E+00	1.72E-17	1.61E+00	8.37E-15	1.04E+00	1.07E-06	-1.26E-01	1.00E+00
ENSBTAG00000044207	-4.40E-02	1.00E+00	3.45E-01	1.60E-01	2.85E-01	2.73E-01	1.07E-01	9.03E-01	6.54E-02	1.00E+00
ENSBTAG00000020422	-1.94E-01	8.78E-01	-2.72E-02	1.00E+00	3.15E-01	1.81E-01	2.18E-01	4.40E-01	2.35E-01	7.94E-01
ENSBTAG00000042579	0.00E+00	1.00E+00	0.00E+00	1.00E+00	-5.13E-15	1.00E+00	0.00E+00	1.00E+00	0.00E+00	1.00E+00
ENSBTAG00000043196	0.00E+00	1.00E+00	0.00E+00	1.00E+00	-5.13E-15	1.00E+00	0.00E+00	1.00E+00	0.00E+00	1.00E+00
ENSBTAG00000020421	2.09E-01	8.63E-01	8.28E-01	6.23E-05	9.21E-01	6.43E-06	9.60E-01	3.11E-06	7.54E-02	1.00E+00
ENSBTAG00000004694	-2.15E-01	1.00E+00	-4.15E+00	2.81E-08	-2.59E+00	6.34E-06	-1.65E+00	1.94E-03	-9.95E-01	3.30E-01
ENSBTAG00000011396	3.22E-01	4.19E-01	1.16E+00	9.24E-11	1.18E+00	3.78E-11	8.11E-01	1.04E-05	-1.92E-02	1.00E+00
ENSBTAG00000018919	0.00E+00	1.00E+00	0.00E+00	1.00E+00	1.78E+00	4.79E-01	2.53E+00	2.71E-01	0.00E+00	1.00E+00
ENSBTAG00000032908	1.45E-01	1.00E+00	1.43E+00	5.55E-03	1.99E+00	1.72E-05	1.46E+00	4.37E-03	6.03E-02	1.00E+00
ENSBTAG00000021008	3.85E-02	1.00E+00	1.44E+00	1.07E-05	2.22E+00	5.11E-12	2.30E+00	1.32E-12	-1.38E-01	1.00E+00
ENSBTAG00000013401	4.66E-02	1.00E+00	-9.39E-01	5.10E-02	4.89E-01	4.09E-01	4.96E-01	4.11E-01	-3.59E-01	9.43E-01
ENSBTAG00000046482	2.70E+00	5.76E-01	0.00E+00	1.00E+00	3.06E+00	1.36E-01	1.76E+00	4.97E-01	0.00E+00	1.00E+00
ENSBTAG00000046189	-6.73E-01	5.64E-01	-3.24E+00	7.76E-10	-8.55E+00	1.86E-21	-4.22E+00	5.42E-14	7.89E-01	4.03E-01
ENSBTAG00000000328	-3.21E-01	1.00E+00	-2.34E+00	3.54E-05	-3.18E+00	4.85E-08	-3.23E+00	3.95E-08	5.18E-01	8.00E-01
ENSBTAG00000000843	-6.23E-01	2.25E-01	-1.46E+00	5.21E-07	-3.11E+00	1.62E-25	-3.15E+00	5.35E-26	2.70E-02	1.00E+00
ENSBTAG00000000936	-1.81E-01	1.00E+00	-2.41E-01	8.80E-01	-1.83E+00	3.04E-04	-3.61E+00	2.09E-09	-7.90E-01	4.99E-01
ENSBTAG00000006613	-1.13E-01	1.00E+00	-4.40E-01	4.67E-02	-3.14E-01	1.99E-01	-7.55E-02	1.00E+00	-1.36E-01	1.00E+00
ENSBTAG00000025664	-1.40E+00	7.83E-01	-1.36E-01	1.00E+00	-3.89E-01	9.98E-01	-1.08E+00	4.97E-01	1.85E+00	1.69E-01
ENSBTAG00000025663	0.00E+00	1.00E+00	0.00E+00	1.00E+00	-5.13E-15	1.00E+00	1.77E+00	4.97E-01	0.00E+00	1.00E+00
ENSBTAG00000004950	1.08E-01	1.00E+00	-6.90E-01	4.59E-01	-2.02E+00	8.11E-03	-7.20E-01	4.32E-01	6.63E-01	8.00E-01
ENSBTAG00000032873	-2.70E+00	5.76E-01	-2.70E+00	2.42E-01	-2.70E+00	1.99E-01	-2.70E+00	2.05E-01	-2.70E+00	6.01E-01
ENSBTAG00000003769	2.06E-01	1.00E+00	1.25E+00	2.05E-06	1.73E+00	2.50E-11	1.79E+00	6.65E-12	2.33E-02	1.00E+00
ENSBTAG00000008793	2.72E+00	5.76E-01	2.79E+00	2.10E-01	2.55E+00	2.55E-01	1.76E+00	4.97E-01	5.09E+00	9.18E-03
ENSBTAG00000008792	-1.19E-01	1.00E+00	-6.03E-02	1.00E+00	-6.61E-01	9.85E-02	-6.78E-01	9.49E-02	2.14E-01	1.00E+00
ENSBTAG00000019612	-1.17E-01	1.00E+00	-4.76E-01	6.57E-02	-1.22E+00	7.41E-08	-1.60E+00	1.96E-12	-5.12E-01	2.61E-01
ENSBTAG00000045492	0.00E+00	1.00E+00	0.00E+00	1.00E+00	-5.13E-15	1.00E+00	0.00E+00	1.00E+00	0.00E+00	1.00E+00

ENSBTAG00000046668	6.74E-01	8.75E-01	2.29E-01	1.00E+00	-2.28E+00	3.75E-02	-1.80E+00	9.73E-02	1.05E+00	5.55E-01
ENSBTAG00000039241	-1.76E-01	1.00E+00	-1.30E+00	1.84E-01	-5.29E+00	6.95E-05	-3.53E+00	1.29E-03	1.13E+00	3.84E-01
ENSBTAG00000046058	0.00E+00	1.00E+00	4.62E+00	3.69E-03	2.56E+00	2.54E-01	1.76E+00	4.97E-01	1.91E+00	8.00E-01
ENSBTAG00000003006	0.00E+00	1.00E+00	0.00E+00	1.00E+00	-5.13E-15	1.00E+00	0.00E+00	1.00E+00	0.00E+00	1.00E+00
ENSBTAG00000032869	1.15E+00	1.82E-01	2.76E+00	3.65E-09	1.56E+00	2.29E-03	1.63E+00	1.53E-03	1.04E+00	3.51E-01
ENSBTAG00000012317	-1.43E-03	1.00E+00	2.82E-02	1.00E+00	-2.49E-01	5.91E-01	-1.03E-01	1.00E+00	3.01E-01	8.02E-01
ENSBTAG00000037452	2.71E-01	1.00E+00	-4.89E-01	3.26E-01	-4.10E-01	4.37E-01	-4.07E-01	4.57E-01	2.61E-01	1.00E+00
ENSBTAG00000002748	-5.31E-02	1.00E+00	-3.19E-01	3.39E-01	-4.19E-01	1.60E-01	-4.29E-01	1.56E-01	-3.05E-01	7.83E-01
ENSBTAG00000002745	2.26E-01	8.85E-01	1.83E+00	3.85E-16	2.26E+00	1.53E-23	1.93E+00	1.05E-17	-1.53E-01	1.00E+00
ENSBTAG00000002743	5.98E-01	1.47E-02	8.95E-01	1.75E-06	1.04E+00	1.85E-08	5.43E-01	7.03E-03	-2.04E-01	8.00E-01
ENSBTAG00000001183	-4.01E-01	9.57E-01	-1.94E+00	5.92E-04	-1.81E+00	6.59E-04	-9.31E-01	9.20E-02	3.73E-01	9.25E-01
ENSBTAG00000001179	7.88E-02	1.00E+00	-3.86E-01	2.42E-01	-2.30E-01	5.89E-01	-4.87E-01	1.18E-01	2.14E-01	9.61E-01
ENSBTAG00000009374	-1.26E-01	1.00E+00	-5.59E-01	2.10E-02	-6.94E-01	2.78E-03	-4.63E-01	7.15E-02	-1.02E-01	1.00E+00
ENSBTAG00000044427	-1.22E+00	1.13E-01	-1.23E+00	2.18E-02	-7.28E-01	2.30E-01	-2.08E+00	7.08E-05	-1.79E+00	1.15E-02
ENSBTAG00000025666	6.33E-02	1.00E+00	1.37E-01	8.61E-01	-1.68E-01	7.42E-01	-5.57E-03	1.00E+00	2.89E-01	8.00E-01
ENSBTAG00000043589	7.62E-01	1.00E+00	-7.97E-01	7.86E-01	1.64E+00	8.65E-02	1.60E+00	1.03E-01	-3.59E+00	3.09E-01
ENSBTAG00000008466	3.71E-01	1.00E+00	1.19E+00	3.07E-02	7.69E-01	2.23E-01	6.07E-01	4.07E-01	-2.79E-01	1.00E+00
ENSBTAG00000009372	-6.76E-02	1.00E+00	-5.77E-01	2.43E-02	-7.44E-01	2.24E-03	-6.68E-01	8.01E-03	-5.92E-02	1.00E+00
ENSBTAG00000038643	-1.91E+00	8.23E-01	-1.91E+00	4.69E-01	-1.91E+00	4.28E-01	-1.91E+00	4.39E-01	-1.91E+00	8.00E-01
ENSBTAG00000015983	-1.91E+00	8.23E-01	-1.91E+00	4.69E-01	-1.91E+00	4.28E-01	-1.91E+00	4.39E-01	-1.91E+00	8.00E-01
ENSBTAG00000032801	5.60E-01	1.00E+00	-5.69E-01	9.88E-01	1.51E-01	1.00E+00	-8.71E-01	7.92E-01	4.59E-01	1.00E+00
ENSBTAG00000039785	0.00E+00	1.00E+00	0.00E+00	1.00E+00	-5.13E-15	1.00E+00	0.00E+00	1.00E+00	0.00E+00	1.00E+00
ENSBTAG00000032799	0.00E+00	1.00E+00	0.00E+00	1.00E+00	-5.13E-15	1.00E+00	0.00E+00	1.00E+00	0.00E+00	1.00E+00
ENSBTAG00000032798	1.30E+00	9.32E-01	7.79E-02	1.00E+00	-1.30E-01	1.00E+00	-1.41E-01	1.00E+00	8.00E-01	1.00E+00
ENSBTAG00000006198	0.00E+00	1.00E+00	0.00E+00	1.00E+00	-5.13E-15	1.00E+00	0.00E+00	1.00E+00	0.00E+00	1.00E+00
ENSBTAG00000038868	-1.91E+00	8.23E-01	-1.91E+00	4.69E-01	-1.91E+00	4.28E-01	-1.91E+00	4.39E-01	-1.91E+00	8.00E-01
ENSBTAG00000037959	0.00E+00	1.00E+00	1.98E+00	4.43E-01	-5.13E-15	1.00E+00	0.00E+00	1.00E+00	0.00E+00	1.00E+00
ENSBTAG00000038227	0.00E+00	1.00E+00	0.00E+00	1.00E+00	-5.13E-15	1.00E+00	0.00E+00	1.00E+00	0.00E+00	1.00E+00
ENSBTAG00000039315	0.00E+00	1.00E+00	0.00E+00	1.00E+00	-5.13E-15	1.00E+00	0.00E+00	1.00E+00	0.00E+00	1.00E+00
ENSBTAG00000048109	0.00E+00	1.00E+00	1.98E+00	4.43E-01	-5.13E-15	1.00E+00	0.00E+00	1.00E+00	0.00E+00	1.00E+00
ENSBTAG00000047483	2.26E-02	1.00E+00	2.32E-01	8.80E-01	2.44E-01	8.34E-01	8.94E-01	4.32E-02	5.76E-01	6.61E-01
ENSBTAG00000045521	0.00E+00	1.00E+00	0.00E+00	1.00E+00	-5.13E-15	1.00E+00	0.00E+00	1.00E+00	0.00E+00	1.00E+00
ENSBTAG00000038485	0.00E+00	1.00E+00	0.00E+00	1.00E+00	-5.13E-15	1.00E+00	0.00E+00	1.00E+00	0.00E+00	1.00E+00
ENSBTAG00000046970	0.00E+00	1.00E+00	0.00E+00	1.00E+00	-5.13E-15	1.00E+00	1.76E+00	4.97E-01	0.00E+00	1.00E+00
ENSBTAG00000025068	0.00E+00	1.00E+00	0.00E+00	1.00E+00	-5.13E-15	1.00E+00	0.00E+00	1.00E+00	0.00E+00	1.00E+00

ENSBTAG00000039416	0.00E+00	1.00E+00	0.00E+00	1.00E+00	-5.13E-15	1.00E+00	0.00E+00	1.00E+00	0.00E+00	1.00E+00
ENSBTAG00000039120	0.00E+00	1.00E+00	0.00E+00	1.00E+00	-5.13E-15	1.00E+00	0.00E+00	1.00E+00	0.00E+00	1.00E+00
ENSBTAG00000038672	0.00E+00	1.00E+00	0.00E+00	1.00E+00	-5.13E-15	1.00E+00	0.00E+00	1.00E+00	0.00E+00	1.00E+00
ENSBTAG00000032792	1.12E-02	1.00E+00	7.57E-02	1.00E+00	-1.91E+00	4.28E-01	6.20E-01	9.94E-01	-1.91E+00	8.00E-01
ENSBTAG00000037411	0.00E+00	1.00E+00	0.00E+00	1.00E+00	-5.13E-15	1.00E+00	0.00E+00	1.00E+00	0.00E+00	1.00E+00
ENSBTAG00000046651	0.00E+00	1.00E+00	0.00E+00	1.00E+00	-5.13E-15	1.00E+00	0.00E+00	1.00E+00	0.00E+00	1.00E+00
ENSBTAG00000046390	0.00E+00	1.00E+00	0.00E+00	1.00E+00	-5.13E-15	1.00E+00	0.00E+00	1.00E+00	0.00E+00	1.00E+00
ENSBTAG00000047311	0.00E+00	1.00E+00	0.00E+00	1.00E+00	-5.13E-15	1.00E+00	0.00E+00	1.00E+00	0.00E+00	1.00E+00
ENSBTAG00000032789	0.00E+00	1.00E+00	0.00E+00	1.00E+00	-5.13E-15	1.00E+00	0.00E+00	1.00E+00	0.00E+00	1.00E+00
ENSBTAG00000011510	0.00E+00	1.00E+00	0.00E+00	1.00E+00	-5.13E-15	1.00E+00	0.00E+00	1.00E+00	0.00E+00	1.00E+00
ENSBTAG00000037511	0.00E+00	1.00E+00	0.00E+00	1.00E+00	-5.13E-15	1.00E+00	0.00E+00	1.00E+00	0.00E+00	1.00E+00
ENSBTAG00000032783	-2.71E+00	5.76E-01	-2.71E+00	2.42E-01	-2.71E+00	1.99E-01	-2.71E+00	2.05E-01	-2.71E+00	6.01E-01
ENSBTAG00000009039	0.00E+00	1.00E+00	0.00E+00	1.00E+00	-5.13E-15	1.00E+00	0.00E+00	1.00E+00	0.00E+00	1.00E+00
ENSBTAG00000032781	0.00E+00	1.00E+00	0.00E+00	1.00E+00	-5.13E-15	1.00E+00	0.00E+00	1.00E+00	0.00E+00	1.00E+00
ENSBTAG00000039370	0.00E+00	1.00E+00	0.00E+00	1.00E+00	-5.13E-15	1.00E+00	0.00E+00	1.00E+00	0.00E+00	1.00E+00
ENSBTAG00000035758	0.00E+00	1.00E+00	0.00E+00	1.00E+00	-5.13E-15	1.00E+00	0.00E+00	1.00E+00	0.00E+00	1.00E+00
ENSBTAG00000047802	0.00E+00	1.00E+00	0.00E+00	1.00E+00	-5.13E-15	1.00E+00	0.00E+00	1.00E+00	0.00E+00	1.00E+00
ENSBTAG00000022915	0.00E+00	1.00E+00	0.00E+00	1.00E+00	-5.13E-15	1.00E+00	0.00E+00	1.00E+00	0.00E+00	1.00E+00
ENSBTAG00000004821	0.00E+00	1.00E+00	0.00E+00	1.00E+00	-5.13E-15	1.00E+00	1.76E+00	4.97E-01	0.00E+00	1.00E+00
ENSBTAG00000046488	0.00E+00	1.00E+00	0.00E+00	1.00E+00	-5.13E-15	1.00E+00	0.00E+00	1.00E+00	0.00E+00	1.00E+00
ENSBTAG00000047984	0.00E+00	1.00E+00	0.00E+00	1.00E+00	-5.13E-15	1.00E+00	0.00E+00	1.00E+00	0.00E+00	1.00E+00
ENSBTAG00000047348	0.00E+00	1.00E+00	0.00E+00	1.00E+00	-5.13E-15	1.00E+00	0.00E+00	1.00E+00	0.00E+00	1.00E+00
ENSBTAG00000046061	0.00E+00	1.00E+00	0.00E+00	1.00E+00	-5.13E-15	1.00E+00	0.00E+00	1.00E+00	0.00E+00	1.00E+00
ENSBTAG00000046009	0.00E+00	1.00E+00	0.00E+00	1.00E+00	-5.13E-15	1.00E+00	0.00E+00	1.00E+00	0.00E+00	1.00E+00
ENSBTAG00000046370	0.00E+00	1.00E+00	0.00E+00	1.00E+00	-5.13E-15	1.00E+00	0.00E+00	1.00E+00	0.00E+00	1.00E+00
ENSBTAG00000047566	0.00E+00	1.00E+00	0.00E+00	1.00E+00	-5.13E-15	1.00E+00	2.53E+00	2.71E-01	0.00E+00	1.00E+00
ENSBTAG00000038188	0.00E+00	1.00E+00	0.00E+00	1.00E+00	-5.13E-15	1.00E+00	0.00E+00	1.00E+00	0.00E+00	1.00E+00
ENSBTAG00000013255	0.00E+00	1.00E+00	0.00E+00	1.00E+00	-5.13E-15	1.00E+00	0.00E+00	1.00E+00	0.00E+00	1.00E+00
ENSBTAG00000046207	0.00E+00	1.00E+00	0.00E+00	1.00E+00	-5.13E-15	1.00E+00	0.00E+00	1.00E+00	0.00E+00	1.00E+00
ENSBTAG00000047465	0.00E+00	1.00E+00	0.00E+00	1.00E+00	-5.13E-15	1.00E+00	0.00E+00	1.00E+00	0.00E+00	1.00E+00
ENSBTAG00000045618	0.00E+00	1.00E+00	0.00E+00	1.00E+00	-5.13E-15	1.00E+00	0.00E+00	1.00E+00	0.00E+00	1.00E+00
ENSBTAG00000039354	1.92E+00	8.23E-01	0.00E+00	1.00E+00	-5.13E-15	1.00E+00	0.00E+00	1.00E+00	0.00E+00	1.00E+00
ENSBTAG00000003549	0.00E+00	1.00E+00	0.00E+00	1.00E+00	-5.13E-15	1.00E+00	0.00E+00	1.00E+00	0.00E+00	1.00E+00
ENSBTAG00000035309	0.00E+00	1.00E+00	0.00E+00	1.00E+00	-5.13E-15	1.00E+00	0.00E+00	1.00E+00	0.00E+00	1.00E+00
ENSBTAG00000039554	0.00E+00	1.00E+00	0.00E+00	1.00E+00	2.56E+00	2.54E-01	1.76E+00	4.97E-01	0.00E+00	1.00E+00

ENSBTAG00000039707	0.00E+00	1.00E+00	0.00E+00	1.00E+00	-5.13E-15	1.00E+00	1.76E+00	4.97E-01	0.00E+00	1.00E+00
ENSBTAG00000040459	0.00E+00	1.00E+00	0.00E+00	1.00E+00	-5.13E-15	1.00E+00	0.00E+00	1.00E+00	0.00E+00	1.00E+00
ENSBTAG00000038083	0.00E+00	1.00E+00	0.00E+00	1.00E+00	-5.13E-15	1.00E+00	0.00E+00	1.00E+00	0.00E+00	1.00E+00
ENSBTAG00000037647	0.00E+00	1.00E+00	1.98E+00	4.43E-01	-5.13E-15	1.00E+00	0.00E+00	1.00E+00	0.00E+00	1.00E+00
ENSBTAG00000038963	0.00E+00	1.00E+00	0.00E+00	1.00E+00	-5.13E-15	1.00E+00	0.00E+00	1.00E+00	0.00E+00	1.00E+00
ENSBTAG00000039884	0.00E+00	1.00E+00	0.00E+00	1.00E+00	-5.13E-15	1.00E+00	0.00E+00	1.00E+00	0.00E+00	1.00E+00
ENSBTAG00000040609	0.00E+00	1.00E+00	0.00E+00	1.00E+00	-5.13E-15	1.00E+00	0.00E+00	1.00E+00	0.00E+00	1.00E+00
ENSBTAG00000038531	0.00E+00	1.00E+00	0.00E+00	1.00E+00	-5.13E-15	1.00E+00	2.53E+00	2.71E-01	0.00E+00	1.00E+00
ENSBTAG00000020040	-3.50E-01	6.83E-01	-1.55E+00	9.34E-09	-8.59E-01	1.33E-03	-9.56E-01	3.85E-04	-2.58E-01	8.38E-01
ENSBTAG00000014948	5.19E-03	1.00E+00	7.11E-02	1.00E+00	-1.37E-01	1.00E+00	-1.91E+00	4.39E-01	-1.91E+00	8.00E-01
ENSBTAG00000025632	-1.12E-01	1.00E+00	1.03E+00	1.19E-05	1.17E+00	4.97E-07	7.01E-01	5.08E-03	-4.31E-01	4.50E-01
ENSBTAG00000016236	-3.67E-01	5.92E-01	-2.19E+00	1.06E-17	-1.52E+00	9.56E-10	-4.77E-01	1.02E-01	2.86E-01	8.00E-01
ENSBTAG00000006416	4.68E-02	1.00E+00	1.71E-01	5.52E-01	1.39E-01	6.83E-01	1.88E-01	4.97E-01	-1.51E-01	9.62E-01
ENSBTAG00000006413	2.12E-01	1.00E+00	1.07E+00	5.34E-04	1.00E+00	1.17E-03	7.93E-01	1.63E-02	-6.27E-01	4.31E-01
ENSBTAG00000007385	-2.35E-02	1.00E+00	-1.14E-02	1.00E+00	-1.57E-01	6.40E-01	-4.81E-02	1.00E+00	3.17E-03	1.00E+00
ENSBTAG00000037985	2.71E+00	5.76E-01	3.98E+00	2.55E-02	1.78E+00	4.79E-01	0.00E+00	1.00E+00	0.00E+00	1.00E+00
ENSBTAG00000044029	2.72E-02	1.00E+00	7.20E-01	8.83E-04	1.47E+00	2.72E-13	1.53E+00	3.92E-14	7.15E-03	1.00E+00
ENSBTAG00000025642	1.59E-01	1.00E+00	8.11E-02	1.00E+00	-1.36E-01	1.00E+00	-2.22E-01	8.44E-01	4.50E-01	7.96E-01
ENSBTAG00000021414	0.00E+00	1.00E+00	1.98E+00	4.43E-01	-5.13E-15	1.00E+00	1.76E+00	4.97E-01	1.93E+00	8.00E-01
ENSBTAG00000025634	8.25E-02	1.00E+00	-2.41E-01	9.04E-01	-3.59E-04	1.00E+00	3.09E-01	7.31E-01	-2.51E-01	1.00E+00
ENSBTAG00000047645	3.87E-01	1.00E+00	1.02E+00	5.17E-01	2.19E-01	1.00E+00	9.31E-01	5.68E-01	5.47E-03	1.00E+00
ENSBTAG00000046604	0.00E+00	1.00E+00	0.00E+00	1.00E+00	-5.13E-15	1.00E+00	1.76E+00	4.97E-01	1.91E+00	8.00E-01
ENSBTAG00000032613	1.83E-01	1.00E+00	-9.63E-01	4.69E-01	-4.04E-01	9.21E-01	-1.95E-01	1.00E+00	-6.17E-01	1.00E+00
ENSBTAG00000020100	-8.76E-02	1.00E+00	-9.88E-01	4.43E-02	-5.27E-01	3.46E-01	-8.75E-01	7.75E-02	-1.53E+00	2.18E-02
ENSBTAG00000021730	-1.91E+00	8.23E-01	-1.91E+00	4.69E-01	-1.91E+00	4.28E-01	-1.91E+00	4.39E-01	-1.91E+00	8.00E-01
ENSBTAG00000005714	3.15E+00	1.77E-02	2.18E+00	7.10E-02	2.37E-01	1.00E+00	1.64E+00	2.36E-01	1.62E+00	6.25E-01
ENSBTAG00000012712	3.45E-01	5.32E-01	1.20E+00	9.17E-09	1.35E+00	6.47E-11	1.29E+00	6.07E-10	2.92E-01	6.98E-01
ENSBTAG00000040206	-9.27E-02	1.00E+00	8.99E-01	1.67E-02	1.68E+00	1.41E-06	1.38E+00	1.10E-04	1.28E-02	1.00E+00
ENSBTAG00000043323	-1.91E+00	8.23E-01	8.81E-01	8.52E-01	-1.91E+00	4.28E-01	1.12E+00	6.68E-01	-1.91E+00	8.00E-01
ENSBTAG00000001364	-3.12E-02	1.00E+00	-7.34E-01	1.30E-02	-1.89E-01	7.34E-01	2.68E-01	5.03E-01	-4.34E-01	5.85E-01
ENSBTAG00000043636	0.00E+00	1.00E+00	0.00E+00	1.00E+00	-5.13E-15	1.00E+00	0.00E+00	1.00E+00	0.00E+00	1.00E+00
ENSBTAG00000042615	0.00E+00	1.00E+00	0.00E+00	1.00E+00	-5.13E-15	1.00E+00	3.03E+00	1.50E-01	0.00E+00	1.00E+00
ENSBTAG00000002179	5.59E-02	1.00E+00	-1.01E+00	2.60E-04	-8.32E-01	2.87E-03	-7.37E-01	1.08E-02	-7.09E-02	1.00E+00
ENSBTAG00000047974	0.00E+00	1.00E+00	0.00E+00	1.00E+00	-5.13E-15	1.00E+00	0.00E+00	1.00E+00	0.00E+00	1.00E+00
ENSBTAG00000003172	-2.08E-01	1.00E+00	-4.24E-01	4.24E-01	8.29E-02	1.00E+00	-2.18E-01	8.37E-01	-6.47E-01	4.90E-01

ENSBTAG00000043625	0.00E+00	1.00E+00	0.00E+00	1.00E+00	-5.13E-15	1.00E+00	0.00E+00	1.00E+00	0.00E+00	1.00E+00
ENSBTAG00000027162	1.19E-02	1.00E+00	2.07E+00	1.95E-01	1.14E+00	6.46E-01	1.12E+00	6.70E-01	1.84E-02	1.00E+00
ENSBTAG00000045158	0.00E+00	1.00E+00	0.00E+00	1.00E+00	-5.13E-15	1.00E+00	0.00E+00	1.00E+00	0.00E+00	1.00E+00
ENSBTAG00000032518	2.80E-01	8.23E-01	-4.33E-01	1.33E-01	-7.07E-01	5.96E-03	-5.37E-01	5.29E-02	-7.87E-01	2.93E-02
ENSBTAG00000043481	0.00E+00	1.00E+00	0.00E+00	1.00E+00	-5.13E-15	1.00E+00	0.00E+00	1.00E+00	0.00E+00	1.00E+00
ENSBTAG00000042361	0.00E+00	1.00E+00	0.00E+00	1.00E+00	-5.13E-15	1.00E+00	0.00E+00	1.00E+00	0.00E+00	1.00E+00
ENSBTAG00000021870	-5.73E-02	1.00E+00	-7.79E-01	4.29E-03	-1.14E-01	9.66E-01	5.98E-01	3.56E-02	-1.44E-01	1.00E+00
ENSBTAG00000021872	2.29E-01	1.00E+00	2.30E-01	7.31E-01	1.26E-03	1.00E+00	-4.30E-01	3.31E-01	2.23E-02	1.00E+00
ENSBTAG00000045437	0.00E+00	1.00E+00	0.00E+00	1.00E+00	-5.13E-15	1.00E+00	0.00E+00	1.00E+00	0.00E+00	1.00E+00
ENSBTAG00000002006	2.24E+00	1.56E-04	1.90E+00	2.95E-04	2.30E+00	1.04E-05	2.67E+00	4.61E-07	-4.61E-01	9.01E-01
ENSBTAG00000012015	-8.47E-01	7.00E-01	-7.52E-01	3.96E-01	-1.28E+00	8.57E-02	-5.70E-01	5.35E-01	-8.36E-01	7.22E-01
ENSBTAG00000016826	3.55E-01	1.00E+00	2.38E+00	9.07E-08	3.35E+00	1.17E-15	1.81E+00	1.08E-04	1.04E-01	1.00E+00
ENSBTAG00000014351	2.66E-01	8.14E-01	1.27E+00	9.41E-09	1.76E+00	6.76E-16	1.18E+00	1.05E-07	1.56E-01	1.00E+00
ENSBTAG00000043106	0.00E+00	1.00E+00	0.00E+00	1.00E+00	-5.13E-15	1.00E+00	0.00E+00	1.00E+00	0.00E+00	1.00E+00
ENSBTAG00000014353	3.17E-01	6.26E-01	1.02E+00	3.23E-06	1.30E+00	1.38E-09	1.34E+00	6.08E-10	-6.94E-02	1.00E+00
ENSBTAG00000012180	7.68E-01	3.24E-01	6.24E-01	2.07E-01	-9.62E-01	4.69E-02	-1.40E+00	3.49E-03	4.92E-02	1.00E+00
ENSBTAG00000007237	-7.31E-01	4.51E-01	-1.30E+00	5.83E-03	-9.42E-01	4.62E-02	-7.01E-01	1.75E-01	-5.88E-01	6.78E-01
ENSBTAG00000019076	-1.41E+00	7.66E-01	1.03E-01	1.00E+00	-1.72E-01	1.00E+00	-1.59E+00	2.71E-01	5.80E-01	1.00E+00
ENSBTAG00000046052	-2.77E+00	1.38E-01	-2.71E+00	3.62E-02	-5.20E-01	7.88E-01	-1.66E+00	1.49E-01	5.27E-01	1.00E+00
ENSBTAG00000019079	-4.62E-01	4.15E-01	-1.44E+00	4.54E-08	-5.91E-01	3.98E-02	-6.52E-01	2.33E-02	-2.71E-02	1.00E+00
ENSBTAG00000013774	-9.86E-01	6.72E-03	-2.93E+00	5.66E-22	-3.14E+00	4.27E-25	-2.59E+00	2.86E-18	1.46E-01	1.00E+00
ENSBTAG00000000552	8.02E-01	1.00E+00	-1.91E+00	4.69E-01	-1.25E-01	1.00E+00	1.49E+00	4.57E-01	7.99E-01	1.00E+00
ENSBTAG00000032340	-2.40E-02	1.00E+00	-1.32E+00	2.33E-04	-7.54E-01	4.12E-02	-7.99E-01	3.23E-02	2.98E-02	1.00E+00
ENSBTAG00000004409	7.19E-03	1.00E+00	-1.04E+00	4.13E-08	-2.08E+00	2.60E-28	-1.24E+00	5.07E-11	4.12E-01	2.96E-01
ENSBTAG00000004420	-3.23E-01	7.75E-01	-8.55E-01	1.77E-03	4.39E-01	1.53E-01	8.03E-01	3.05E-03	1.71E-01	1.00E+00
ENSBTAG00000004421	-2.29E-01	9.74E-01	-6.38E-01	3.74E-02	2.19E-01	6.20E-01	2.99E-01	4.33E-01	2.81E-01	8.00E-01
ENSBTAG00000025593	-7.96E-02	1.00E+00	-9.04E-01	1.78E-04	-7.50E-02	1.00E+00	1.35E-01	8.53E-01	-6.44E-02	1.00E+00
ENSBTAG00000003193	-7.02E-01	7.89E-02	-2.76E-02	1.00E+00	7.05E-01	9.01E-03	3.76E-01	2.60E-01	-2.38E-01	9.06E-01
ENSBTAG00000045360	0.00E+00	1.00E+00	0.00E+00	1.00E+00	-5.13E-15	1.00E+00	0.00E+00	1.00E+00	0.00E+00	1.00E+00
ENSBTAG00000002918	2.40E-01	1.00E+00	8.18E-01	2.62E-02	-9.68E-01	1.05E-02	-1.03E+00	7.02E-03	-4.82E-01	6.88E-01
ENSBTAG00000002921	-2.96E-01	7.80E-01	-1.89E-01	6.71E-01	-1.37E-01	8.54E-01	-3.59E-01	2.47E-01	-2.58E-01	8.00E-01
ENSBTAG00000013477	-1.03E-01	1.00E+00	-9.77E-01	6.41E-05	-2.08E+00	5.48E-18	-1.82E+00	3.66E-14	8.46E-02	1.00E+00
ENSBTAG00000013480	-3.58E-01	3.75E-01	-1.44E+00	8.40E-14	-1.85E+00	6.12E-22	-1.36E+00	1.67E-12	2.10E-02	1.00E+00
ENSBTAG00000047281	0.00E+00	1.00E+00	0.00E+00	1.00E+00	-5.13E-15	1.00E+00	0.00E+00	1.00E+00	0.00E+00	1.00E+00
ENSBTAG00000013485	-1.03E+00	2.05E-03	-2.67E+00	1.87E-20	-3.54E+00	2.06E-32	-3.15E+00	3.78E-27	-3.93E-01	6.47E-01

ENSBTAG00000013488	3.41E-01	1.00E+00	5.01E-01	5.86E-01	3.89E-01	7.36E-01	-5.24E-01	6.22E-01	-2.02E-01	1.00E+00
ENSBTAG00000004086	-2.01E-01	1.00E+00	-5.65E-01	3.89E-01	-1.97E-01	9.75E-01	-2.71E-01	8.36E-01	-1.47E-01	1.00E+00
ENSBTAG00000016291	1.63E+00	3.51E-01	3.79E+00	1.21E-08	3.91E+00	1.22E-09	1.80E+00	5.36E-02	2.87E-01	1.00E+00
ENSBTAG00000011567	-9.30E-02	1.00E+00	2.61E-01	3.94E-01	5.91E-01	1.05E-02	4.41E-01	8.19E-02	-1.54E-01	1.00E+00
ENSBTAG00000010361	1.87E+00	6.87E-04	2.12E+00	8.31E-06	1.36E+00	5.44E-03	1.02E+00	5.38E-02	5.02E-01	8.00E-01
ENSBTAG00000010371	8.70E-01	8.02E-01	4.76E+00	5.29E-10	2.53E+00	6.76E-04	1.96E+00	1.21E-02	-5.44E-01	1.00E+00
ENSBTAG00000010380	-2.43E-02	1.00E+00	9.82E-01	2.63E-05	1.06E+00	4.07E-06	7.10E-01	3.96E-03	1.59E-01	1.00E+00
ENSBTAG00000038438	-1.19E-01	1.00E+00	-2.86E-01	2.63E-01	-3.03E-01	2.19E-01	-3.79E-01	1.07E-01	-2.43E-01	7.90E-01
ENSBTAG00000008363	-3.05E-01	1.00E+00	-1.30E+00	2.38E-03	-1.37E+00	1.18E-03	-3.74E-01	5.64E-01	-6.24E-01	6.01E-01
ENSBTAG00000027173	1.10E-01	1.00E+00	1.10E+00	2.31E-08	1.20E+00	7.44E-10	1.25E+00	1.80E-10	-1.12E-01	1.00E+00
ENSBTAG00000044604	0.00E+00	1.00E+00	0.00E+00	1.00E+00	-5.13E-15	1.00E+00	0.00E+00	1.00E+00	0.00E+00	1.00E+00
ENSBTAG00000010766	-4.45E-01	6.18E-01	7.10E-01	2.43E-02	1.14E-01	1.00E+00	-7.38E-01	3.21E-02	-4.48E-01	6.27E-01
ENSBTAG00000010774	2.67E-01	8.23E-01	1.45E+00	7.09E-10	6.72E-02	1.00E+00	-6.17E-01	2.92E-02	-8.71E-02	1.00E+00
ENSBTAG00000010778	-1.58E-01	1.00E+00	1.15E-01	1.00E+00	8.18E-01	5.39E-03	9.82E-01	7.54E-04	9.48E-02	1.00E+00
ENSBTAG00000001671	1.86E-01	9.89E-01	1.80E-01	6.52E-01	4.95E-01	4.44E-02	5.17E-01	3.83E-02	9.15E-03	1.00E+00
ENSBTAG00000001675	1.04E-01	1.00E+00	-8.67E-01	3.79E-02	-1.07E+00	7.61E-03	-4.42E-01	4.10E-01	6.33E-01	4.99E-01
ENSBTAG00000001693	-2.33E-01	8.23E-01	-3.26E-01	2.31E-01	2.00E-01	5.48E-01	4.34E-01	8.21E-02	2.38E-01	8.00E-01
ENSBTAG00000001694	-5.68E-01	6.41E-01	-2.42E+00	6.96E-07	-3.51E-01	5.72E-01	4.48E-02	1.00E+00	-5.33E-01	7.20E-01
ENSBTAG00000042188	-1.79E-03	1.00E+00	-7.21E-01	9.45E-01	-2.71E+00	1.99E-01	-2.71E+00	2.05E-01	-2.71E+00	6.01E-01
ENSBTAG00000015138	9.24E-02	1.00E+00	1.54E+00	4.93E-08	1.62E+00	6.39E-09	1.07E+00	2.51E-04	2.54E-01	9.12E-01
ENSBTAG00000014662	0.00E+00	1.00E+00	0.00E+00	1.00E+00	-5.13E-15	1.00E+00	0.00E+00	1.00E+00	0.00E+00	1.00E+00
ENSBTAG00000015142	8.69E-02	1.00E+00	6.68E-01	2.37E-02	8.37E-01	2.98E-03	4.40E-01	1.96E-01	4.89E-01	4.57E-01
ENSBTAG00000046607	-3.35E-01	7.16E-01	-3.07E-01	3.84E-01	-1.28E-01	9.01E-01	1.20E-01	9.32E-01	-2.10E-01	9.52E-01
ENSBTAG00000006784	1.63E-01	1.00E+00	-1.45E-01	8.41E-01	-3.74E-02	1.00E+00	-8.59E-01	8.65E-04	3.23E-01	7.03E-01
ENSBTAG00000006790	-1.41E-01	1.00E+00	1.72E-01	8.30E-01	-2.96E-01	4.79E-01	-1.16E+00	1.39E-04	-5.26E-03	1.00E+00
ENSBTAG00000006792	9.53E-01	1.31E-02	6.33E-01	6.34E-02	8.31E-01	7.93E-03	9.67E-01	1.74E-03	1.72E-01	1.00E+00
ENSBTAG00000021924	1.48E-02	1.00E+00	9.11E-02	1.00E+00	-3.59E+00	4.53E-02	1.07E-01	1.00E+00	-1.67E+00	7.79E-01
ENSBTAG00000005268	-5.04E-01	1.00E+00	4.67E-01	9.95E-01	-1.53E-01	1.00E+00	-3.21E+00	9.94E-02	1.16E+00	8.00E-01
ENSBTAG00000032068	-7.36E-01	1.00E+00	2.84E-01	1.00E+00	3.31E-01	9.88E-01	9.57E-01	3.35E-01	2.09E-01	1.00E+00
ENSBTAG00000032055	2.75E-02	1.00E+00	2.09E-01	5.69E-01	6.45E-01	7.32E-03	5.24E-01	4.12E-02	3.62E-01	5.90E-01
ENSBTAG00000008863	1.14E+00	6.83E-08	2.17E+00	1.68E-28	1.56E+00	9.44E-16	1.03E+00	2.91E-07	-8.88E-03	1.00E+00
ENSBTAG00000008866	1.74E-02	1.00E+00	-1.45E-01	9.27E-01	-9.99E-01	1.16E-03	-1.21E+00	8.21E-05	2.20E-01	1.00E+00
ENSBTAG00000008868	-2.12E-01	1.00E+00	5.65E-01	3.08E-01	8.47E-02	1.00E+00	-6.67E-01	2.65E-01	4.45E-01	8.13E-01
ENSBTAG00000004079	2.52E-01	9.56E-01	1.09E+00	1.71E-04	8.83E-01	2.89E-03	1.15E+00	8.02E-05	3.13E-01	8.00E-01
ENSBTAG00000005661	3.30E-01	3.93E-01	9.09E-01	4.61E-07	3.08E-01	1.60E-01	3.36E-01	1.23E-01	-1.86E-01	8.36E-01

ENSBTAG00000031981	1.53E-01	1.00E+00	-1.79E-01	6.99E-01	2.30E-01	5.10E-01	2.41E-01	4.97E-01	-2.24E-01	8.90E-01
ENSBTAG00000005663	1.44E-01	1.00E+00	-6.64E-02	1.00E+00	4.75E-01	6.78E-02	8.44E-01	3.45E-04	-2.53E-01	8.00E-01
ENSBTAG00000039789	8.89E-02	1.00E+00	-2.80E-01	8.91E-01	4.77E-02	1.00E+00	-1.77E-03	1.00E+00	1.32E-01	1.00E+00
ENSBTAG00000003754	2.25E-01	8.87E-01	-1.79E-01	6.84E-01	1.86E-01	6.42E-01	-1.78E-01	6.87E-01	-2.36E-01	8.34E-01
ENSBTAG00000005751	-3.70E-01	5.40E-01	-1.45E+00	4.48E-10	-7.13E-01	3.15E-03	-5.20E-01	4.72E-02	1.19E-01	1.00E+00
ENSBTAG00000006470	2.67E-01	9.95E-01	-6.31E-01	1.03E-01	-1.68E-01	8.99E-01	4.38E-01	3.07E-01	3.53E-01	8.00E-01
ENSBTAG00000021087	2.20E-01	1.00E+00	9.96E-01	4.25E-04	8.82E-01	2.03E-03	7.04E-01	2.02E-02	2.42E-01	9.23E-01
ENSBTAG00000013031	-2.78E-01	8.23E-01	-1.35E+00	3.89E-08	-9.22E-01	2.05E-04	-7.69E-01	2.89E-03	2.72E-01	8.00E-01
ENSBTAG00000013032	8.88E-03	1.00E+00	-1.51E-01	5.59E-01	-6.02E-02	1.00E+00	-7.58E-02	9.55E-01	1.67E-01	8.33E-01
ENSBTAG00000011439	2.13E-01	1.00E+00	9.24E-02	1.00E+00	-4.09E-01	9.74E-01	-7.28E-01	7.08E-01	2.42E-01	1.00E+00
ENSBTAG00000046363	5.33E-01	1.00E+00	1.78E+00	1.14E-02	-1.80E-01	1.00E+00	4.15E-01	8.72E-01	2.59E-03	1.00E+00
ENSBTAG00000004692	-2.68E-01	1.00E+00	-8.00E-01	2.08E-01	-1.51E+00	7.18E-03	-9.86E-01	9.77E-02	-3.39E+00	2.28E-05
ENSBTAG00000038155	8.09E-01	1.00E+00	8.80E-01	8.52E-01	-1.91E+00	4.28E-01	1.12E+00	6.68E-01	6.37E-03	1.00E+00
ENSBTAG00000024582	-1.91E+00	8.23E-01	8.81E-01	8.52E-01	6.51E-01	9.78E-01	-1.91E+00	4.39E-01	-1.91E+00	8.00E-01
ENSBTAG00000005697	1.42E-01	1.00E+00	3.72E-02	1.00E+00	-1.04E+00	1.87E-01	3.12E-01	9.04E-01	-8.39E-01	7.51E-01
ENSBTAG00000032894	0.00E+00	1.00E+00	0.00E+00	1.00E+00	-5.13E-15	1.00E+00	0.00E+00	1.00E+00	0.00E+00	1.00E+00
ENSBTAG00000047222	1.92E+00	8.23E-01	0.00E+00	1.00E+00	1.78E+00	4.79E-01	0.00E+00	1.00E+00	0.00E+00	1.00E+00
ENSBTAG00000045199	0.00E+00	1.00E+00	0.00E+00	1.00E+00	-5.13E-15	1.00E+00	0.00E+00	1.00E+00	0.00E+00	1.00E+00
ENSBTAG00000045661	-1.76E+00	2.07E-02	-1.74E+00	3.58E-03	-9.32E-01	1.68E-01	-2.17E+00	2.82E-04	-2.03E+00	1.18E-02
ENSBTAG00000004926	-1.50E-02	1.00E+00	9.80E-01	4.05E-03	1.68E+00	5.58E-08	1.09E+00	1.18E-03	-1.87E-01	1.00E+00
ENSBTAG00000025564	3.10E-01	7.06E-01	5.02E-01	5.21E-02	4.44E-01	9.68E-02	3.15E-01	3.07E-01	-1.10E-01	1.00E+00
ENSBTAG00000033891	6.05E-01	2.58E-01	2.44E+00	8.51E-17	2.66E+00	1.04E-19	1.92E+00	5.50E-11	3.18E-01	8.00E-01
ENSBTAG00000004930	-5.01E-01	1.61E-01	4.44E-01	6.01E-02	5.02E-01	2.68E-02	1.21E-01	8.56E-01	-3.36E-01	6.01E-01
ENSBTAG00000004931	8.03E-02	1.00E+00	1.16E+00	1.62E-06	8.40E-01	7.92E-04	1.31E-02	1.00E+00	3.65E-02	1.00E+00
ENSBTAG00000031697	-2.10E-01	1.00E+00	-4.04E-01	3.26E-01	-5.69E-01	1.12E-01	-1.39E-01	9.55E-01	-1.58E-01	1.00E+00
ENSBTAG00000004933	1.33E-02	1.00E+00	-1.45E-01	6.52E-01	-1.78E-01	5.00E-01	-3.72E-01	7.29E-02	1.12E-01	1.00E+00
ENSBTAG00000043867	0.00E+00	1.00E+00	0.00E+00	1.00E+00	-5.13E-15	1.00E+00	0.00E+00	1.00E+00	0.00E+00	1.00E+00
ENSBTAG00000004934	1.56E-01	1.00E+00	8.12E-01	5.51E-05	7.16E-01	4.29E-04	5.94E-01	5.26E-03	-2.87E-01	6.51E-01
ENSBTAG00000047908	-1.39E+00	1.10E-01	-1.12E+00	7.69E-02	-8.45E-01	2.12E-01	-2.03E+00	7.24E-04	-1.79E+00	3.77E-02
ENSBTAG00000046912	-9.09E-01	3.75E-01	-1.08E+00	5.29E-02	-5.02E-01	4.72E-01	-2.15E+00	7.16E-05	-1.88E+00	1.01E-02
ENSBTAG00000044296	0.00E+00	1.00E+00	0.00E+00	1.00E+00	-5.13E-15	1.00E+00	0.00E+00	1.00E+00	0.00E+00	1.00E+00
ENSBTAG00000045980	4.30E-01	2.33E-01	1.31E+00	5.00E-11	1.05E+00	1.45E-07	9.13E-01	8.08E-06	1.45E-02	1.00E+00
ENSBTAG00000005547	-3.27E-01	6.45E-01	2.93E-01	3.30E-01	7.86E-01	4.14E-04	7.44E-01	1.10E-03	-6.50E-01	9.42E-02
ENSBTAG00000005550	-2.78E-01	8.23E-01	-1.32E+00	5.88E-08	-2.76E-01	4.08E-01	1.85E-02	1.00E+00	-1.54E-01	1.00E+00
ENSBTAG00000043035	0.00E+00	1.00E+00	1.98E+00	4.43E-01	2.56E+00	2.54E-01	1.76E+00	4.97E-01	2.71E+00	6.01E-01

ENSBTAG00000029512	-9.13E-01	8.86E-01	-2.54E+00	6.10E-02	-8.00E-01	5.69E-01	-1.99E+00	1.06E-01	-9.32E-01	8.42E-01
ENSBTAG00000015200	-9.53E-02	1.00E+00	-1.19E+00	4.93E-03	-1.21E+00	4.07E-03	-4.05E-01	4.97E-01	-3.75E-01	8.86E-01
ENSBTAG00000015202	-1.08E-01	1.00E+00	-6.63E-01	4.42E-03	-1.14E+00	2.71E-07	-1.28E+00	8.80E-09	-3.12E-01	6.55E-01
ENSBTAG00000004780	-1.00E+00	2.74E-01	-1.98E+00	2.32E-04	-1.82E+00	4.98E-04	-2.35E+00	1.24E-05	-4.82E-01	8.39E-01
ENSBTAG00000014792	1.80E-01	9.44E-01	1.46E-01	7.23E-01	6.34E-01	2.02E-03	2.97E-01	2.48E-01	-2.87E-01	6.58E-01
ENSBTAG00000014312	1.37E+00	3.57E-01	2.83E+00	1.12E-04	1.39E+00	9.40E-02	2.42E-01	1.00E+00	-5.70E-01	1.00E+00
ENSBTAG00000001539	2.08E+00	7.87E-14	3.45E+00	8.51E-36	2.53E+00	3.32E-21	1.24E+00	5.95E-06	-1.65E-01	1.00E+00
ENSBTAG00000020281	2.24E-01	1.00E+00	3.16E-01	4.98E-01	4.08E-01	3.24E-01	6.07E-01	9.76E-02	-1.19E-01	1.00E+00
ENSBTAG00000044049	9.42E-02	1.00E+00	-7.92E-01	1.98E-03	-1.81E+00	1.06E-13	-2.07E+00	3.25E-17	4.73E-02	1.00E+00
ENSBTAG00000011494	1.47E-01	1.00E+00	-4.26E-01	1.80E-01	-1.36E+00	1.75E-07	-1.24E+00	2.47E-06	4.92E-01	4.26E-01
ENSBTAG00000010103	-1.76E-01	1.00E+00	-1.25E-01	9.46E-01	1.73E-01	7.74E-01	3.48E-02	1.00E+00	-2.92E-01	8.00E-01
ENSBTAG00000046184	0.00E+00	1.00E+00	0.00E+00	1.00E+00	-5.13E-15	1.00E+00	0.00E+00	1.00E+00	0.00E+00	1.00E+00
ENSBTAG00000011225	-1.25E-01	1.00E+00	-4.18E-01	6.72E-02	-5.15E-02	1.00E+00	-2.03E-02	1.00E+00	-1.90E-01	8.85E-01
ENSBTAG00000031707	7.19E-02	1.00E+00	8.53E-02	1.00E+00	-1.47E-01	1.00E+00	2.50E-01	8.79E-01	-3.44E-01	1.00E+00
ENSBTAG00000003043	8.78E-01	4.27E-03	1.31E+00	8.30E-08	1.08E+00	1.15E-05	4.31E-01	1.61E-01	-4.90E-01	4.34E-01
ENSBTAG00000021944	-9.12E-02	1.00E+00	-1.13E-01	8.77E-01	-2.73E-01	3.14E-01	-1.68E-01	6.50E-01	-5.17E-01	1.58E-01
ENSBTAG00000021945	-3.20E-02	1.00E+00	1.82E-01	7.25E-01	4.54E-01	1.26E-01	6.95E-01	1.09E-02	-3.25E-01	7.53E-01
ENSBTAG00000001423	-2.71E+00	5.76E-01	8.27E-02	1.00E+00	-2.71E+00	1.99E-01	-9.40E-01	8.06E-01	-2.71E+00	6.01E-01
ENSBTAG00000006703	-7.31E-01	2.68E-01	-7.80E-01	5.36E-02	-3.69E-01	4.63E-01	-4.94E-01	2.77E-01	-5.68E-01	5.77E-01
ENSBTAG00000008411	7.07E-01	2.36E-02	8.07E-01	7.37E-04	1.72E-01	7.04E-01	-1.74E-01	7.12E-01	-5.00E-01	3.09E-01
ENSBTAG00000007638	9.68E-02	1.00E+00	-9.52E-01	2.58E-02	-3.76E+00	5.90E-15	-4.54E+00	5.51E-18	8.93E-02	1.00E+00
ENSBTAG00000011037	5.28E-01	3.57E-01	2.52E-01	5.78E-01	-2.86E-01	4.83E-01	4.52E-01	1.95E-01	3.30E-01	8.00E-01
ENSBTAG00000015806	-3.29E-01	7.60E-01	-1.82E+00	3.71E-12	-1.37E+00	1.21E-07	-6.19E-01	3.30E-02	-1.82E-01	1.00E+00
ENSBTAG00000015808	-2.90E-01	8.29E-01	-1.29E+00	3.68E-06	-1.42E-01	8.97E-01	-2.90E-02	1.00E+00	2.34E-01	9.35E-01
ENSBTAG00000043656	0.00E+00	1.00E+00	0.00E+00	1.00E+00	-5.13E-15	1.00E+00	0.00E+00	1.00E+00	0.00E+00	1.00E+00
ENSBTAG00000017641	0.00E+00	1.00E+00	0.00E+00	1.00E+00	-5.13E-15	1.00E+00	0.00E+00	1.00E+00	0.00E+00	1.00E+00
ENSBTAG00000014493	-2.80E-01	7.65E-01	-1.74E+00	4.24E-15	-8.75E-01	1.00E-04	-1.07E+00	2.02E-06	-3.32E-01	6.05E-01
ENSBTAG00000039462	2.24E-02	1.00E+00	-7.35E-01	1.59E-01	-5.45E-01	3.38E-01	-1.01E+00	3.76E-02	-1.11E+00	1.53E-01
ENSBTAG00000016823	3.44E-01	6.56E-01	7.49E-01	3.10E-03	8.74E-01	3.61E-04	7.80E-01	2.10E-03	3.81E-02	1.00E+00
ENSBTAG00000016822	1.35E-01	1.00E+00	2.65E-01	3.84E-01	2.89E-03	1.00E+00	-1.18E-01	8.88E-01	3.83E-01	4.81E-01
ENSBTAG00000016821	2.51E-01	1.00E+00	1.08E-01	1.00E+00	-5.69E-01	3.55E-01	-8.10E-01	1.50E-01	1.40E-01	1.00E+00
ENSBTAG00000002014	3.55E-01	4.47E-01	2.59E-01	3.55E-01	5.28E-01	1.66E-02	6.37E-01	3.41E-03	-3.82E-03	1.00E+00
ENSBTAG00000043013	0.00E+00	1.00E+00	0.00E+00	1.00E+00	-5.13E-15	1.00E+00	0.00E+00	1.00E+00	0.00E+00	1.00E+00
ENSBTAG00000002012	-8.88E-02	1.00E+00	-4.60E-01	4.85E-02	-4.67E-01	4.34E-02	-3.39E-01	1.92E-01	-2.59E-01	7.79E-01
ENSBTAG00000011820	2.67E-01	1.00E+00	-4.95E-01	4.06E-01	-2.11E+00	1.53E-06	-2.36E+00	1.24E-07	4.39E-01	8.07E-01

ENSBTAG00000019474	1.66E-01	1.00E+00	-3.86E-01	1.06E-01	-3.03E-01	2.40E-01	-2.51E-01	3.86E-01	2.69E-01	7.33E-01
ENSBTAG00000044583	0.00E+00	1.00E+00	0.00E+00	1.00E+00	-5.13E-15	1.00E+00	0.00E+00	1.00E+00	0.00E+00	1.00E+00
ENSBTAG00000008807	-1.85E+00	3.73E-03	-2.34E+00	3.38E-05	-1.28E+00	1.11E-02	-1.58E+00	2.20E-03	-1.02E+00	2.97E-01
ENSBTAG00000008805	-1.17E-01	1.00E+00	-9.52E-01	6.01E-05	-6.26E-01	1.12E-02	-4.31E-01	1.18E-01	-2.96E-01	7.60E-01
ENSBTAG00000019390	1.84E-01	1.00E+00	-3.03E-01	1.00E+00	2.66E-01	1.00E+00	3.54E-01	9.56E-01	-9.42E-01	8.45E-01
ENSBTAG00000010641	2.96E-01	1.00E+00	1.60E-01	1.00E+00	-4.40E-01	5.52E-01	3.16E-01	7.39E-01	-2.34E-01	1.00E+00
ENSBTAG00000011146	1.62E-01	1.00E+00	4.08E-01	6.68E-02	2.81E-02	1.00E+00	4.34E-01	5.21E-02	-6.40E-01	2.97E-02
ENSBTAG00000012898	-6.33E-01	2.39E-01	-5.04E-01	1.65E-01	-1.56E+00	3.17E-07	-1.48E+00	1.74E-06	-5.67E-01	4.29E-01
ENSBTAG00000017373	4.97E-02	1.00E+00	-5.05E-01	5.47E-02	9.77E-02	9.83E-01	4.51E-01	9.63E-02	-1.52E-01	1.00E+00
ENSBTAG00000005373	-2.57E-01	8.87E-01	-8.95E-01	9.79E-04	-1.31E+00	6.00E-07	-1.10E+00	4.33E-05	3.06E-01	8.00E-01
ENSBTAG00000003667	-1.11E-01	1.00E+00	-5.43E-01	1.94E-01	-1.84E+00	2.46E-07	-1.03E+00	4.70E-03	-4.01E-01	8.00E-01
ENSBTAG00000030049	0.00E+00	1.00E+00	0.00E+00	1.00E+00	-5.13E-15	1.00E+00	0.00E+00	1.00E+00	0.00E+00	1.00E+00
ENSBTAG00000020814	6.58E+00	4.78E-06	3.70E+00	6.51E-03	2.50E+00	9.99E-02	5.82E+00	9.63E-06	4.92E-01	1.00E+00
ENSBTAG00000042813	0.00E+00	1.00E+00	0.00E+00	1.00E+00	-5.13E-15	1.00E+00	0.00E+00	1.00E+00	0.00E+00	1.00E+00
ENSBTAG00000042731	0.00E+00	1.00E+00	0.00E+00	1.00E+00	-5.13E-15	1.00E+00	0.00E+00	1.00E+00	0.00E+00	1.00E+00
ENSBTAG00000010952	-1.26E+00	2.59E-01	-3.10E+00	6.30E-05	-5.00E+00	5.32E-08	-3.09E+00	3.14E-05	6.72E-01	7.63E-01
ENSBTAG00000038920	-1.27E-01	1.00E+00	-1.21E+00	4.17E-09	-1.19E+00	5.59E-09	-6.00E-01	6.40E-03	-2.42E-01	8.00E-01
ENSBTAG00000015904	2.39E-01	8.56E-01	-9.04E-01	2.12E-04	-1.46E+00	8.94E-10	-2.22E-01	5.47E-01	1.71E-02	1.00E+00
ENSBTAG00000005328	3.27E-01	7.19E-01	-3.02E-01	3.83E-01	2.82E-01	4.25E-01	1.92E-01	6.81E-01	7.68E-02	1.00E+00
ENSBTAG00000009615	1.04E+00	8.93E-07	3.01E+00	4.02E-51	2.89E+00	4.16E-48	2.50E+00	3.90E-37	-2.90E-01	6.24E-01
ENSBTAG00000019114	0.00E+00	1.00E+00	3.30E+00	1.03E-01	-5.13E-15	1.00E+00	2.53E+00	2.71E-01	1.91E+00	8.00E-01
ENSBTAG00000013493	8.73E-02	1.00E+00	3.16E-01	4.40E-01	6.13E-01	4.64E-02	5.64E-01	8.04E-02	-5.09E-01	4.73E-01
ENSBTAG00000010298	1.16E-01	1.00E+00	6.23E-01	9.53E-03	5.05E-01	4.38E-02	3.43E-01	2.35E-01	-1.39E-01	1.00E+00
ENSBTAG00000043816	0.00E+00	1.00E+00	0.00E+00	1.00E+00	-5.13E-15	1.00E+00	0.00E+00	1.00E+00	0.00E+00	1.00E+00
ENSBTAG00000009443	3.11E+00	1.16E-04	5.15E+00	1.09E-18	3.45E+00	2.73E-07	3.80E+00	5.16E-09	2.16E+00	1.04E-01
ENSBTAG00000014699	-4.50E-01	8.87E-01	-3.09E-01	7.65E-01	-1.10E+00	3.68E-02	-1.40E+00	7.99E-03	-1.94E-01	1.00E+00
ENSBTAG00000021538	1.33E-01	1.00E+00	2.71E-01	4.12E-01	6.94E-01	4.18E-03	8.72E-01	2.55E-04	-1.13E-01	1.00E+00
ENSBTAG00000005269	1.38E-01	1.00E+00	1.36E-02	1.00E+00	-9.65E-01	1.92E-01	-5.07E-01	6.01E-01	-2.24E-01	1.00E+00
ENSBTAG00000014181	-3.75E-02	1.00E+00	2.56E-01	4.21E-01	3.93E-01	1.32E-01	1.30E-01	8.48E-01	-5.61E-02	1.00E+00
ENSBTAG00000011319	1.15E-01	1.00E+00	5.23E-02	1.00E+00	-2.50E-02	1.00E+00	-3.87E-01	8.58E-02	-1.50E-01	9.92E-01
ENSBTAG00000012391	3.55E-01	9.05E-01	-5.87E-01	2.18E-01	-1.18E-01	1.00E+00	8.66E-01	3.53E-02	1.44E-02	1.00E+00
ENSBTAG00000043145	0.00E+00	1.00E+00	0.00E+00	1.00E+00	-5.13E-15	1.00E+00	0.00E+00	1.00E+00	0.00E+00	1.00E+00
ENSBTAG00000005481	1.01E-01	1.00E+00	3.34E-01	3.98E-01	4.93E-01	1.36E-01	3.51E-01	3.66E-01	2.51E-01	9.11E-01
ENSBTAG00000005479	-2.47E-01	8.70E-01	-7.12E-01	5.89E-03	-1.41E+00	7.04E-09	-2.01E+00	1.61E-16	-2.81E-01	8.00E-01
ENSBTAG00000037226	0.00E+00	1.00E+00	0.00E+00	1.00E+00	-5.13E-15	1.00E+00	0.00E+00	1.00E+00	0.00E+00	1.00E+00

ENSBTAG00000044518	0.00E+00	1.00E+00	0.00E+00	1.00E+00	-5.13E-15	1.00E+00	0.00E+00	1.00E+00	0.00E+00	1.00E+00
ENSBTAG00000011764	0.00E+00	1.00E+00	0.00E+00	1.00E+00	-5.13E-15	1.00E+00	0.00E+00	1.00E+00	0.00E+00	1.00E+00
ENSBTAG00000043926	0.00E+00	1.00E+00	0.00E+00	1.00E+00	-5.13E-15	1.00E+00	0.00E+00	1.00E+00	0.00E+00	1.00E+00
ENSBTAG00000042541	0.00E+00	1.00E+00	0.00E+00	1.00E+00	-5.13E-15	1.00E+00	0.00E+00	1.00E+00	0.00E+00	1.00E+00
ENSBTAG00000013452	-1.35E-01	1.00E+00	-9.27E-01	1.92E-05	-2.40E+00	4.32E-29	-2.96E+00	1.10E-41	2.26E-01	8.12E-01
ENSBTAG00000010119	3.47E-02	1.00E+00	-4.54E-02	1.00E+00	-8.29E-01	2.14E-02	4.27E-02	1.00E+00	4.67E-02	1.00E+00
ENSBTAG00000043491	0.00E+00	1.00E+00	0.00E+00	1.00E+00	-5.13E-15	1.00E+00	0.00E+00	1.00E+00	0.00E+00	1.00E+00
ENSBTAG00000040384	1.41E-01	1.00E+00	3.39E-01	4.85E-01	2.79E-01	6.17E-01	4.38E-01	3.26E-01	-1.41E-02	1.00E+00
ENSBTAG00000045089	0.00E+00	1.00E+00	0.00E+00	1.00E+00	-5.13E-15	1.00E+00	0.00E+00	1.00E+00	0.00E+00	1.00E+00
ENSBTAG00000002194	2.94E-01	8.23E-01	8.49E-01	2.00E-03	2.87E-01	4.55E-01	-2.04E-01	6.97E-01	1.91E-01	1.00E+00
ENSBTAG00000008575	2.63E-01	1.00E+00	1.30E+00	1.16E-03	7.10E-01	1.15E-01	2.65E-01	7.60E-01	5.81E-01	6.01E-01
ENSBTAG00000002586	9.08E-02	1.00E+00	7.26E-01	3.79E-04	1.06E-01	8.90E-01	-2.49E-01	3.77E-01	-2.49E-01	7.79E-01
ENSBTAG00000043372	0.00E+00	1.00E+00	0.00E+00	1.00E+00	-5.13E-15	1.00E+00	0.00E+00	1.00E+00	0.00E+00	1.00E+00
ENSBTAG00000039235	-8.71E-01	1.00E+00	-3.58E+00	6.71E-02	-3.58E+00	4.57E-02	-3.58E+00	4.94E-02	-3.58E+00	3.09E-01
ENSBTAG00000001486	1.13E-01	1.00E+00	-1.61E-01	7.28E-01	5.16E-01	3.32E-02	2.70E-01	3.91E-01	-5.00E-02	1.00E+00
ENSBTAG00000000271	3.41E-02	1.00E+00	-8.86E-01	6.28E-02	-7.47E-01	1.25E-01	-7.74E-01	1.16E-01	-5.23E-01	7.68E-01
ENSBTAG00000031433	-6.69E-02	1.00E+00	-1.07E+00	2.58E-04	-1.44E+00	5.82E-07	-1.28E+00	9.13E-06	-4.61E-01	5.32E-01
ENSBTAG00000000565	-2.49E-01	9.17E-01	-2.48E+00	1.92E-19	-1.47E+00	3.38E-08	-8.95E-01	1.29E-03	-4.51E-01	5.08E-01
ENSBTAG00000031396	1.63E+00	4.92E-06	3.07E+00	3.24E-21	2.15E+00	1.81E-11	2.29E+00	1.17E-12	2.69E-01	9.69E-01
ENSBTAG00000013793	3.33E-01	9.59E-01	3.35E-01	5.98E-01	-1.62E-01	9.85E-01	2.22E-01	8.49E-01	-2.20E-01	1.00E+00
ENSBTAG00000005848	2.63E-02	1.00E+00	5.37E-01	7.49E-01	2.70E-01	1.00E+00	8.48E-01	4.13E-01	-5.97E-01	1.00E+00
ENSBTAG00000009151	-5.27E-02	1.00E+00	-8.76E-01	5.23E-02	5.16E-02	1.00E+00	1.03E+00	9.43E-03	1.03E+00	8.57E-02
ENSBTAG00000031388	6.02E-01	7.65E-01	-1.44E-01	1.00E+00	3.32E-01	7.45E-01	5.16E-01	4.60E-01	5.15E-01	8.00E-01
ENSBTAG00000012741	2.68E-01	8.29E-01	3.67E-01	2.53E-01	5.70E-01	4.13E-02	1.15E+00	6.82E-06	4.73E-01	4.34E-01
ENSBTAG00000030014	0.00E+00	1.00E+00	0.00E+00	1.00E+00	-5.13E-15	1.00E+00	0.00E+00	1.00E+00	0.00E+00	1.00E+00
ENSBTAG00000012740	-8.73E-02	1.00E+00	-2.04E-01	5.62E-01	-2.25E-01	4.83E-01	-2.96E-02	1.00E+00	-2.86E-01	7.53E-01
ENSBTAG00000038259	0.00E+00	1.00E+00	0.00E+00	1.00E+00	1.78E+00	4.79E-01	2.53E+00	2.71E-01	0.00E+00	1.00E+00
ENSBTAG00000012408	1.21E+00	6.72E-03	3.52E+00	3.86E-28	2.62E+00	4.64E-16	1.99E+00	2.81E-09	6.07E-01	5.58E-01
ENSBTAG00000001342	1.52E-01	1.00E+00	1.77E+00	3.24E-19	1.68E+00	1.37E-17	9.55E-01	2.61E-06	-5.36E-01	1.29E-01
ENSBTAG00000045196	0.00E+00	1.00E+00	0.00E+00	1.00E+00	-5.13E-15	1.00E+00	0.00E+00	1.00E+00	0.00E+00	1.00E+00
ENSBTAG00000025522	1.11E-01	1.00E+00	-2.04E-01	1.00E+00	-1.10E-01	1.00E+00	5.36E-01	4.97E-01	-9.13E-01	5.90E-01
ENSBTAG00000021876	-3.06E-01	1.00E+00	-1.33E+00	2.10E-02	-3.28E+00	1.03E-07	-2.05E+00	2.93E-04	-2.99E-01	1.00E+00
ENSBTAG00000046311	-1.59E-01	1.00E+00	1.93E-01	8.54E-01	8.84E-02	1.00E+00	3.99E-02	1.00E+00	-5.23E-01	6.25E-01
ENSBTAG00000046470	-5.66E-01	6.21E-01	-1.98E+00	1.16E-06	-1.33E+00	1.16E-03	-2.60E-01	7.78E-01	8.28E-01	3.09E-01
ENSBTAG00000024958	-7.58E-01	1.49E-02	-3.23E+00	3.65E-39	-3.44E+00	6.05E-44	-2.92E+00	2.59E-33	-7.09E-01	5.64E-02

ENSBTAG00000011022	2.24E-01	8.23E-01	6.95E-01	4.11E-04	6.95E-01	3.78E-04	7.58E-01	1.13E-04	-2.27E-01	8.00E-01
ENSBTAG00000006489	1.38E-01	1.00E+00	4.05E-01	4.18E-01	9.97E-02	1.00E+00	3.45E-01	5.11E-01	-2.21E-01	1.00E+00
ENSBTAG00000044748	0.00E+00	1.00E+00	0.00E+00	1.00E+00	-5.13E-15	1.00E+00	0.00E+00	1.00E+00	0.00E+00	1.00E+00
ENSBTAG00000003763	1.17E-01	1.00E+00	-2.39E+00	1.59E-12	-2.68E+00	1.98E-15	-1.74E+00	1.16E-07	-9.54E-02	1.00E+00
ENSBTAG00000003762	3.45E-01	9.32E-01	-3.25E-01	6.36E-01	2.42E-01	7.84E-01	1.38E-03	1.00E+00	-1.27E-01	1.00E+00
ENSBTAG00000005429	1.92E+00	8.23E-01	0.00E+00	1.00E+00	3.06E+00	1.36E-01	2.53E+00	2.71E-01	1.91E+00	8.00E-01
ENSBTAG00000042528	0.00E+00	1.00E+00	0.00E+00	1.00E+00	-5.13E-15	1.00E+00	0.00E+00	1.00E+00	0.00E+00	1.00E+00
ENSBTAG00000005865	-2.66E-01	8.23E-01	-1.32E+00	1.02E-07	-4.03E-01	1.82E-01	7.35E-02	1.00E+00	5.00E-01	3.52E-01
ENSBTAG00000013830	-1.09E-01	1.00E+00	-5.27E-03	1.00E+00	4.48E-01	5.12E-02	3.60E-01	1.51E-01	9.34E-03	1.00E+00
ENSBTAG00000019601	1.69E-01	1.00E+00	8.08E-01	4.38E-02	6.37E-01	1.36E-01	9.44E-01	1.60E-02	1.19E-01	1.00E+00
ENSBTAG00000047868	0.00E+00	1.00E+00	0.00E+00	1.00E+00	-5.13E-15	1.00E+00	0.00E+00	1.00E+00	0.00E+00	1.00E+00
ENSBTAG00000014011	-1.30E+00	2.45E-01	-1.63E+00	2.23E-02	-1.18E+00	8.57E-02	-8.96E-01	2.21E-01	-3.57E-01	1.00E+00
ENSBTAG00000017683	-7.86E-01	2.37E-01	1.56E+00	2.65E-06	1.97E+00	9.02E-10	3.63E-01	4.84E-01	-4.91E-01	7.12E-01
ENSBTAG00000017677	3.58E-02	1.00E+00	1.40E+00	5.66E-02	1.63E+00	1.54E-02	3.73E-01	9.02E-01	-4.48E-01	1.00E+00
ENSBTAG00000000737	1.16E+00	2.45E-01	3.46E+00	4.97E-10	1.72E+00	2.67E-03	2.32E+00	3.79E-05	4.37E-01	9.93E-01
ENSBTAG00000002576	9.16E-01	5.82E-01	1.28E+00	5.48E-02	9.98E-01	1.69E-01	5.64E-01	5.83E-01	3.84E-01	1.00E+00
ENSBTAG00000014890	-6.60E-01	1.00E+00	-5.82E-01	8.85E-01	2.89E-01	1.00E+00	-8.52E-01	6.64E-01	8.34E-01	8.72E-01
ENSBTAG00000010472	1.86E+00	4.87E-02	5.65E+00	1.75E-22	5.90E+00	1.77E-24	4.85E+00	5.47E-17	1.85E+00	8.66E-02
ENSBTAG00000013616	1.05E-01	1.00E+00	1.02E+00	2.11E-04	1.31E+00	1.00E-06	9.21E-01	1.12E-03	-1.18E-01	1.00E+00
ENSBTAG00000011831	2.98E-01	8.14E-01	2.11E-01	6.24E-01	2.48E-01	5.04E-01	6.99E-01	9.60E-03	6.85E-01	9.60E-02
ENSBTAG00000031165	3.73E-01	3.75E-01	1.10E+00	3.84E-08	1.31E+00	2.44E-11	1.90E+00	6.96E-22	-1.83E-02	1.00E+00
ENSBTAG00000044670	0.00E+00	1.00E+00	1.99E+00	4.43E-01	-5.13E-15	1.00E+00	0.00E+00	1.00E+00	1.91E+00	8.00E-01
ENSBTAG00000011929	3.55E-01	9.69E-01	2.52E+00	2.58E-11	1.14E+00	6.31E-03	4.81E-01	4.13E-01	-3.67E-01	9.47E-01
ENSBTAG00000045682	0.00E+00	1.00E+00	0.00E+00	1.00E+00	-5.13E-15	1.00E+00	0.00E+00	1.00E+00	0.00E+00	1.00E+00
ENSBTAG00000045913	0.00E+00	1.00E+00	0.00E+00	1.00E+00	-5.13E-15	1.00E+00	0.00E+00	1.00E+00	0.00E+00	1.00E+00
ENSBTAG00000011916	2.00E-01	8.89E-01	2.76E-01	3.04E-01	3.51E-01	1.50E-01	3.72E-01	1.28E-01	-1.17E-02	1.00E+00
ENSBTAG00000022801	-3.32E-01	7.05E-01	-1.63E-01	7.84E-01	-2.69E-02	1.00E+00	-7.14E-01	7.56E-03	-1.55E-01	1.00E+00
ENSBTAG00000009911	3.05E-01	8.70E-01	-1.35E+00	1.32E-04	-2.07E+00	6.64E-09	-3.00E+00	2.66E-14	9.81E-01	1.98E-02
ENSBTAG00000045591	-6.74E-01	1.00E+00	-1.90E+00	2.55E-01	-1.34E+00	4.12E-01	-1.35E+00	4.14E-01	-3.88E+00	2.06E-01
ENSBTAG00000045634	-3.21E+00	3.75E-01	-1.23E+00	6.11E-01	-1.44E+00	4.79E-01	-1.45E+00	4.94E-01	-3.21E+00	4.34E-01
ENSBTAG00000004303	-1.51E-01	1.00E+00	-9.97E-01	6.11E-05	-2.22E+00	8.19E-20	-3.39E+00	1.64E-40	2.28E-01	8.96E-01
ENSBTAG00000011936	-2.18E-01	1.00E+00	-7.32E-01	4.92E-02	-1.47E-01	9.52E-01	-1.48E-01	9.53E-01	-4.88E-01	6.07E-01
ENSBTAG00000046224	0.00E+00	1.00E+00	0.00E+00	1.00E+00	-5.13E-15	1.00E+00	0.00E+00	1.00E+00	0.00E+00	1.00E+00
ENSBTAG00000006201	-1.36E-01	1.00E+00	-9.24E-01	6.79E-04	2.13E-01	6.09E-01	5.86E-02	1.00E+00	-6.42E-02	1.00E+00
ENSBTAG00000004013	-2.64E-01	1.00E+00	-1.52E+00	1.60E-01	-2.35E-01	1.00E+00	-1.09E-01	1.00E+00	1.15E-01	1.00E+00

ENSBTAG00000004011	1.64E-01	1.00E+00	1.09E-01	1.00E+00	3.80E-03	1.00E+00	-6.42E-03	1.00E+00	4.16E-02	1.00E+00
ENSBTAG00000013425	1.67E-01	1.00E+00	4.51E-01	2.01E-01	1.01E-01	1.00E+00	-3.00E-01	4.97E-01	-5.74E-01	4.25E-01
ENSBTAG00000046553	-2.64E-01	1.00E+00	-7.49E-01	4.25E-02	-4.78E-01	2.54E-01	2.09E-01	8.02E-01	4.29E-01	7.33E-01
ENSBTAG00000046376	0.00E+00	1.00E+00	1.98E+00	4.43E-01	-5.13E-15	1.00E+00	0.00E+00	1.00E+00	0.00E+00	1.00E+00
ENSBTAG00000005217	-1.90E-01	1.00E+00	1.42E-01	8.50E-01	3.01E-01	3.58E-01	4.57E-01	1.10E-01	-4.62E-01	4.22E-01
ENSBTAG00000027764	-1.11E-01	1.00E+00	-1.75E+00	4.22E-12	-2.37E+00	1.15E-20	-2.19E+00	7.25E-18	-2.43E-01	8.65E-01
ENSBTAG00000022583	5.16E-01	1.00E+00	-7.15E-01	9.45E-01	-1.42E-01	1.00E+00	-9.39E-01	8.06E-01	5.24E-01	1.00E+00
ENSBTAG00000011661	3.11E-02	1.00E+00	1.77E-01	9.57E-01	7.51E-01	7.49E-02	1.06E+00	6.88E-03	8.70E-02	1.00E+00
ENSBTAG00000002278	1.00E-01	1.00E+00	-3.44E-01	2.80E-01	-6.79E-02	1.00E+00	2.52E-01	4.92E-01	7.46E-02	1.00E+00
ENSBTAG00000011998	-3.31E-01	8.23E-01	-8.42E-01	6.76E-03	-1.07E+00	3.56E-04	-1.00E+00	1.09E-03	-5.84E-01	3.59E-01
ENSBTAG00000042771	0.00E+00	1.00E+00	0.00E+00	1.00E+00	-5.13E-15	1.00E+00	0.00E+00	1.00E+00	0.00E+00	1.00E+00
ENSBTAG00000010145	-9.06E-01	9.60E-01	4.91E-01	8.57E-01	-1.58E+00	2.63E-01	-1.95E-01	1.00E+00	-9.09E-01	9.23E-01
ENSBTAG00000045847	0.00E+00	1.00E+00	0.00E+00	1.00E+00	-5.13E-15	1.00E+00	0.00E+00	1.00E+00	0.00E+00	1.00E+00
ENSBTAG00000008771	-6.01E-02	1.00E+00	-9.95E-01	9.35E-03	6.66E-01	7.56E-02	1.41E+00	1.85E-05	1.58E-02	1.00E+00
ENSBTAG00000004854	-5.04E-01	2.02E-01	-1.65E+00	2.98E-12	-6.14E-01	1.17E-02	6.74E-02	1.00E+00	-9.34E-01	2.07E-03
ENSBTAG00000016800	8.83E-02	1.00E+00	3.33E-01	4.82E-01	9.56E-01	3.45E-03	3.18E-01	5.15E-01	6.98E-02	1.00E+00
ENSBTAG00000006267	0.00E+00	1.00E+00	1.98E+00	4.43E-01	-5.13E-15	1.00E+00	0.00E+00	1.00E+00	0.00E+00	1.00E+00
ENSBTAG00000019879	1.92E+00	8.23E-01	0.00E+00	1.00E+00	-5.13E-15	1.00E+00	0.00E+00	1.00E+00	0.00E+00	1.00E+00
ENSBTAG00000036982	0.00E+00	1.00E+00	0.00E+00	1.00E+00	-5.13E-15	1.00E+00	0.00E+00	1.00E+00	0.00E+00	1.00E+00
ENSBTAG00000010365	1.14E-01	1.00E+00	4.28E-01	1.43E-01	1.21E-01	9.22E-01	-6.60E-02	1.00E+00	-3.04E-02	1.00E+00
ENSBTAG00000012250	2.76E-02	1.00E+00	-1.26E-01	8.86E-01	2.31E-02	1.00E+00	3.34E-01	2.72E-01	3.02E-02	1.00E+00
ENSBTAG00000021019	-1.15E-01	1.00E+00	-1.18E+00	9.99E-03	-4.06E-01	5.00E-01	4.32E-01	4.69E-01	3.38E-01	9.46E-01
ENSBTAG00000004558	1.64E-01	1.00E+00	5.14E-01	3.81E-01	-1.50E-02	1.00E+00	-6.94E-02	1.00E+00	-7.18E-01	5.25E-01
ENSBTAG00000036401	0.00E+00	1.00E+00	0.00E+00	1.00E+00	-5.13E-15	1.00E+00	0.00E+00	1.00E+00	0.00E+00	1.00E+00
ENSBTAG00000018279	-3.33E-01	5.76E-01	-3.63E-01	1.64E-01	-7.47E-01	8.06E-04	-4.39E-01	8.01E-02	-2.91E-01	7.08E-01
ENSBTAG00000005586	-8.02E-02	1.00E+00	-1.10E+00	7.48E-03	-1.74E+00	9.97E-06	-1.82E+00	4.72E-06	-2.46E-01	1.00E+00
ENSBTAG00000027251	1.44E+00	7.51E-01	-2.70E+00	2.42E-01	-2.70E+00	1.99E-01	-2.70E+00	2.05E-01	5.47E-01	1.00E+00
ENSBTAG00000016327	-3.22E-01	1.00E+00	7.54E-01	2.80E-01	5.79E-01	4.55E-01	-5.28E-01	6.09E-01	-2.37E-01	1.00E+00
ENSBTAG00000044398	0.00E+00	1.00E+00	0.00E+00	1.00E+00	-5.13E-15	1.00E+00	0.00E+00	1.00E+00	3.21E+00	4.34E-01
ENSBTAG00000020934	-6.14E-01	9.01E-02	-2.28E+00	6.86E-21	-2.31E+00	1.40E-21	-1.41E+00	4.92E-09	-3.45E-02	1.00E+00
ENSBTAG00000016246	-6.91E-01	9.22E-01	-1.70E+00	6.92E-02	-6.79E-01	5.41E-01	6.83E-01	4.93E-01	-2.53E-01	1.00E+00
ENSBTAG00000016242	-1.15E+00	8.82E-01	-1.59E+00	3.40E-01	-9.24E-01	6.25E-01	1.02E+00	4.59E-01	-4.38E+00	1.32E-01
ENSBTAG00000016239	-1.23E+00	9.18E-01	-1.60E+00	3.89E-01	-5.57E-01	9.71E-01	1.36E+00	4.68E-01	-8.63E-01	1.00E+00
ENSBTAG00000016234	-1.49E+00	7.41E-01	-2.09E+00	1.32E-01	-1.39E+00	3.76E-01	1.13E+00	5.02E-01	-1.08E-01	1.00E+00
ENSBTAG00000025496	-1.72E-01	1.00E+00	-6.37E-01	7.35E-03	-1.66E+00	5.26E-14	-2.25E+00	7.19E-24	-5.72E-02	1.00E+00

ENSBTAG00000038327	-1.14E-01	1.00E+00	-2.62E+00	3.05E-05	-2.13E+00	2.05E-04	-6.36E-01	3.43E-01	4.25E-01	9.00E-01
ENSBTAG00000047170	0.00E+00	1.00E+00	0.00E+00	1.00E+00	-5.13E-15	1.00E+00	0.00E+00	1.00E+00	0.00E+00	1.00E+00
ENSBTAG00000014501	6.43E-01	1.09E-02	4.80E-01	2.49E-02	-9.88E-02	9.14E-01	-1.40E-01	7.40E-01	-1.43E-01	1.00E+00
ENSBTAG00000037199	0.00E+00	1.00E+00	0.00E+00	1.00E+00	-5.13E-15	1.00E+00	0.00E+00	1.00E+00	0.00E+00	1.00E+00
ENSBTAG00000019924	-6.24E-03	1.00E+00	-2.94E-01	4.50E-01	7.82E-01	4.06E-03	8.44E-01	2.08E-03	-1.44E-01	1.00E+00
ENSBTAG00000044557	0.00E+00	1.00E+00	0.00E+00	1.00E+00	-5.13E-15	1.00E+00	0.00E+00	1.00E+00	0.00E+00	1.00E+00
ENSBTAG00000044632	0.00E+00	1.00E+00	0.00E+00	1.00E+00	-5.13E-15	1.00E+00	0.00E+00	1.00E+00	0.00E+00	1.00E+00
ENSBTAG00000043487	0.00E+00	1.00E+00	0.00E+00	1.00E+00	-5.13E-15	1.00E+00	0.00E+00	1.00E+00	0.00E+00	1.00E+00
ENSBTAG00000042311	0.00E+00	1.00E+00	0.00E+00	1.00E+00	-5.13E-15	1.00E+00	0.00E+00	1.00E+00	0.00E+00	1.00E+00
ENSBTAG00000047239	0.00E+00	1.00E+00	0.00E+00	1.00E+00	-5.13E-15	1.00E+00	0.00E+00	1.00E+00	0.00E+00	1.00E+00
ENSBTAG00000003835	4.01E-01	7.83E-01	-1.89E+00	3.68E-08	-1.42E+00	2.82E-05	-1.39E+00	5.25E-05	-1.58E-01	1.00E+00
ENSBTAG00000019496	1.78E-01	1.00E+00	1.90E+00	1.20E-07	1.56E+00	1.55E-05	6.42E-01	1.45E-01	-4.88E-01	7.47E-01
ENSBTAG00000019500	1.83E-02	1.00E+00	1.02E+00	2.62E-06	1.41E+00	2.70E-11	8.82E-01	6.58E-05	-3.33E-01	6.01E-01
ENSBTAG00000025311	-3.54E-02	1.00E+00	6.31E-01	3.89E-02	6.68E-01	2.66E-02	7.02E-01	2.13E-02	-6.20E-01	2.63E-01
ENSBTAG00000017141	-7.16E-01	1.01E-02	-4.38E-01	7.60E-02	2.97E-01	2.80E-01	-1.16E-02	1.00E+00	-7.89E-01	9.10E-03
ENSBTAG00000044921	0.00E+00	1.00E+00	0.00E+00	1.00E+00	-5.13E-15	1.00E+00	1.77E+00	4.97E-01	1.93E+00	8.00E-01
ENSBTAG00000001468	1.68E+00	4.91E-10	2.50E+00	2.71E-22	1.91E+00	5.70E-14	8.44E-01	2.03E-03	1.75E-01	1.00E+00
ENSBTAG00000040151	1.38E+00	3.08E-05	2.83E+00	5.45E-21	2.41E+00	4.09E-16	1.56E+00	1.75E-07	-2.41E-01	9.70E-01
ENSBTAG00000019120	1.76E-02	1.00E+00	1.21E+00	2.82E-06	1.63E+00	5.26E-11	1.18E+00	5.31E-06	-7.42E-01	1.39E-01
ENSBTAG00000021988	1.49E-01	1.00E+00	1.78E+00	2.21E-12	1.54E+00	1.31E-09	1.26E+00	1.25E-06	-6.71E-02	1.00E+00
ENSBTAG00000021986	5.69E-01	7.15E-02	1.77E+00	3.35E-17	1.24E+00	3.63E-09	5.60E-01	1.69E-02	-1.61E-01	1.00E+00
ENSBTAG00000002326	3.05E-01	1.00E+00	-4.46E-01	5.38E-01	-1.67E-01	1.00E+00	6.11E-01	3.17E-01	-4.07E-01	9.27E-01
ENSBTAG00000002331	2.69E-01	1.00E+00	-4.84E-01	6.09E-01	-3.91E-01	7.26E-01	-6.85E-01	3.86E-01	-1.08E+00	4.35E-01
ENSBTAG00000024206	1.69E+00	7.71E-01	8.76E-01	8.53E-01	2.60E+00	4.51E-02	-1.91E+00	4.39E-01	7.56E-04	1.00E+00
ENSBTAG00000020533	3.56E-02	1.00E+00	4.57E-01	1.02E-01	2.55E-01	4.70E-01	1.77E-01	7.21E-01	1.11E-01	1.00E+00
ENSBTAG00000010795	-2.44E-01	9.61E-01	-2.20E-01	6.71E-01	3.90E-01	2.75E-01	-4.07E-01	2.66E-01	2.46E-01	9.19E-01
ENSBTAG00000018277	-1.91E+00	8.23E-01	7.58E-02	1.00E+00	-1.91E+00	4.28E-01	6.21E-01	9.94E-01	-1.91E+00	8.00E-01
ENSBTAG00000047067	0.00E+00	1.00E+00	0.00E+00	1.00E+00	-5.13E-15	1.00E+00	0.00E+00	1.00E+00	0.00E+00	1.00E+00
ENSBTAG00000017713	2.46E-01	7.32E-01	9.89E-01	1.03E-07	1.65E+00	1.63E-19	1.53E+00	6.60E-17	-4.99E-01	1.24E-01
ENSBTAG00000045339	0.00E+00	1.00E+00	0.00E+00	1.00E+00	-5.13E-15	1.00E+00	0.00E+00	1.00E+00	0.00E+00	1.00E+00
ENSBTAG00000005141	-1.12E-01	1.00E+00	-9.41E-01	9.11E-02	-9.42E-01	8.55E-02	-5.90E-02	1.00E+00	1.10E-01	1.00E+00
ENSBTAG00000030881	2.19E-01	7.78E-01	5.80E-01	1.52E-03	6.80E-01	1.36E-04	6.35E-02	1.00E+00	-1.62E-02	1.00E+00
ENSBTAG00000042081	0.00E+00	1.00E+00	1.98E+00	4.43E-01	1.78E+00	4.79E-01	0.00E+00	1.00E+00	0.00E+00	1.00E+00
ENSBTAG00000014741	1.92E+00	8.23E-01	0.00E+00	1.00E+00	-5.13E-15	1.00E+00	0.00E+00	1.00E+00	0.00E+00	1.00E+00
ENSBTAG00000013042	2.97E-01	5.83E-01	9.54E-01	1.24E-06	8.12E-01	4.35E-05	6.80E-01	9.85E-04	-2.96E-01	6.18E-01

ENSBTAG00000013043	7.49E-02	1.00E+00	8.00E-01	3.88E-03	5.63E-01	5.77E-02	4.30E-01	1.94E-01	1.37E-01	1.00E+00
ENSBTAG00000043327	-5.02E-01	1.00E+00	7.73E-01	7.29E-01	-1.48E-01	1.00E+00	-6.73E-01	8.90E-01	1.49E+00	6.01E-01
ENSBTAG00000012998	-4.13E-01	5.72E-01	-4.41E-01	1.69E-01	-3.55E-03	1.00E+00	1.52E-01	8.54E-01	-2.84E-02	1.00E+00
ENSBTAG00000009862	-1.12E-02	1.00E+00	-8.09E-01	3.96E-02	-7.35E-01	6.51E-02	4.99E-01	2.71E-01	2.24E-01	1.00E+00
ENSBTAG00000044706	0.00E+00	1.00E+00	0.00E+00	1.00E+00	-5.13E-15	1.00E+00	0.00E+00	1.00E+00	0.00E+00	1.00E+00
ENSBTAG00000006341	-6.27E-01	1.00E+00	-5.38E-01	8.53E-01	-1.96E+00	1.24E-01	-3.81E-01	9.79E-01	-1.95E-01	1.00E+00
ENSBTAG00000044829	0.00E+00	1.00E+00	0.00E+00	1.00E+00	-5.13E-15	1.00E+00	0.00E+00	1.00E+00	0.00E+00	1.00E+00
ENSBTAG00000036635	0.00E+00	1.00E+00	0.00E+00	1.00E+00	-5.13E-15	1.00E+00	0.00E+00	1.00E+00	0.00E+00	1.00E+00
ENSBTAG00000020880	-7.87E-02	1.00E+00	-5.61E-01	1.64E-01	-6.28E-01	1.04E-01	-6.70E-01	8.38E-02	-1.38E-01	1.00E+00
ENSBTAG00000045091	0.00E+00	1.00E+00	0.00E+00	1.00E+00	-5.13E-15	1.00E+00	0.00E+00	1.00E+00	0.00E+00	1.00E+00
ENSBTAG00000014392	-3.87E-03	1.00E+00	-4.35E-01	3.25E-02	-4.01E-01	5.16E-02	-1.43E-01	6.86E-01	-1.50E-01	9.69E-01
ENSBTAG00000002808	2.94E-02	1.00E+00	8.43E-01	8.37E-06	6.09E-01	1.82E-03	3.65E-01	1.02E-01	-4.01E-01	3.10E-01
ENSBTAG00000018898	5.10E-02	1.00E+00	9.96E-02	9.96E-01	1.05E-01	9.75E-01	4.35E-01	1.30E-01	4.79E-02	1.00E+00
ENSBTAG00000010503	-5.35E-02	1.00E+00	6.38E-01	1.97E-02	1.01E+00	6.85E-05	9.17E-01	4.41E-04	-1.96E-01	1.00E+00
ENSBTAG00000004631	8.00E-02	1.00E+00	-9.36E-01	4.71E-05	-4.28E-01	9.53E-02	-5.29E-02	1.00E+00	-2.76E-02	1.00E+00
ENSBTAG00000043611	0.00E+00	1.00E+00	0.00E+00	1.00E+00	-5.13E-15	1.00E+00	0.00E+00	1.00E+00	0.00E+00	1.00E+00
ENSBTAG00000019421	-1.23E+00	2.30E-01	-1.24E+00	5.73E-02	-1.47E+00	1.80E-02	-6.54E-01	3.93E-01	-2.87E-01	1.00E+00
ENSBTAG00000045799	-1.91E+00	8.23E-01	7.57E-02	1.00E+00	-1.91E+00	4.28E-01	6.20E-01	9.94E-01	-1.91E+00	8.00E-01
ENSBTAG00000005984	4.10E-01	4.75E-01	-7.10E-01	6.14E-03	-1.27E+00	2.26E-07	-8.58E-01	8.02E-04	3.46E-01	6.53E-01
ENSBTAG00000045329	0.00E+00	1.00E+00	0.00E+00	1.00E+00	-5.13E-15	1.00E+00	0.00E+00	1.00E+00	0.00E+00	1.00E+00
ENSBTAG00000042389	0.00E+00	1.00E+00	0.00E+00	1.00E+00	-5.13E-15	1.00E+00	0.00E+00	1.00E+00	0.00E+00	1.00E+00
ENSBTAG00000003370	-8.96E-02	1.00E+00	-2.90E-01	9.88E-01	-1.59E-01	1.00E+00	-1.45E+00	1.07E-01	-4.69E-01	1.00E+00
ENSBTAG00000030824	-8.50E-02	1.00E+00	-7.22E-01	3.45E-03	-4.48E-01	9.18E-02	-6.10E-01	1.59E-02	3.54E-01	5.98E-01
ENSBTAG00000003371	-3.80E-01	6.13E-01	-9.55E-01	4.04E-04	-2.38E-01	5.62E-01	-6.26E-02	1.00E+00	-1.74E-01	1.00E+00
ENSBTAG00000011448	-7.88E-01	3.49E-01	-6.32E-01	2.19E-01	-1.13E+00	1.14E-02	-1.43E+00	1.34E-03	1.19E-01	1.00E+00
ENSBTAG00000011451	1.37E-01	1.00E+00	-2.97E-01	3.87E-01	-9.03E-02	1.00E+00	-4.49E-02	1.00E+00	-1.33E-01	1.00E+00
ENSBTAG00000040564	-1.45E-01	1.00E+00	-3.28E-01	3.25E-01	-4.83E-01	9.51E-02	-3.37E-01	3.17E-01	-4.07E-01	5.59E-01
ENSBTAG00000003090	-6.90E-01	8.23E-01	-2.41E+00	3.36E-03	-2.37E+00	2.16E-03	-1.47E+00	4.95E-02	-1.24E+00	4.34E-01
ENSBTAG00000014096	-1.15E-01	1.00E+00	-1.77E+00	8.32E-09	-6.74E-01	3.57E-02	1.43E-01	9.23E-01	-1.18E-01	1.00E+00
ENSBTAG00000020729	-2.52E-01	7.56E-01	-7.28E-01	3.03E-04	-1.57E+00	4.59E-16	-2.18E+00	6.91E-29	-1.43E-01	1.00E+00
ENSBTAG00000018127	-2.48E-01	7.31E-01	-1.22E+00	8.99E-11	-1.45E+00	6.57E-15	-8.94E-01	2.90E-06	8.97E-02	1.00E+00
ENSBTAG00000014657	1.12E-02	1.00E+00	5.95E-01	9.47E-01	3.49E-01	1.00E+00	6.86E-01	8.62E-01	-7.93E-01	1.00E+00
ENSBTAG00000013035	0.00E+00	1.00E+00	2.79E+00	2.10E-01	-5.13E-15	1.00E+00	3.03E+00	1.50E-01	0.00E+00	1.00E+00
ENSBTAG00000012139	1.37E+00	1.52E-01	1.93E-01	1.00E+00	8.44E-01	2.81E-01	1.70E+00	4.41E-03	4.40E-01	1.00E+00
ENSBTAG00000046309	-3.87E+00	1.45E-01	-1.09E+00	5.55E-01	-8.26E-01	6.76E-01	-2.11E+00	1.81E-01	-1.16E+00	8.46E-01

ENSBTAG00000030801	-3.39E-02	1.00E+00	1.72E-02	1.00E+00	8.53E-02	1.00E+00	-2.67E-01	3.98E-01	-3.21E-01	6.34E-01
ENSBTAG00000011796	-1.47E-01	1.00E+00	1.06E+00	2.39E-06	1.30E+00	4.22E-09	6.65E-01	5.83E-03	-4.89E-01	3.11E-01
ENSBTAG00000007931	-4.53E-01	3.34E-01	-4.51E-01	9.80E-02	-5.40E-01	3.50E-02	-4.59E-01	9.10E-02	-3.80E-01	5.60E-01
ENSBTAG00000042567	0.00E+00	1.00E+00	0.00E+00	1.00E+00	-5.13E-15	1.00E+00	0.00E+00	1.00E+00	0.00E+00	1.00E+00
ENSBTAG00000047000	-5.23E-02	1.00E+00	-6.01E-01	2.30E-01	-9.32E-01	3.43E-02	7.11E-01	1.41E-01	-1.47E-01	1.00E+00
ENSBTAG00000045169	-3.22E+00	3.75E-01	-3.22E+00	1.25E-01	-1.44E+00	4.79E-01	-3.22E+00	9.88E-02	3.79E-01	1.00E+00
ENSBTAG00000016713	-2.24E-01	1.00E+00	-2.65E+00	3.13E-12	-2.20E+00	3.38E-09	-5.44E-01	2.43E-01	6.09E-01	5.16E-01
ENSBTAG00000003276	6.34E-03	1.00E+00	1.73E-02	1.00E+00	-3.60E-01	2.28E-01	-3.80E-01	2.05E-01	-5.19E-01	3.05E-01
ENSBTAG00000036968	0.00E+00	1.00E+00	0.00E+00	1.00E+00	-5.13E-15	1.00E+00	0.00E+00	1.00E+00	0.00E+00	1.00E+00
ENSBTAG00000020935	1.19E+00	1.59E-03	1.92E+00	9.61E-10	1.52E+00	1.23E-06	1.68E+00	1.06E-07	-2.11E-01	1.00E+00
ENSBTAG00000020937	-5.03E-01	4.09E-01	-3.19E-01	4.33E-01	3.24E-01	3.96E-01	5.48E-01	8.19E-02	7.15E-03	1.00E+00
ENSBTAG00000018947	6.14E-03	1.00E+00	-3.84E-01	1.00E+00	-1.32E+00	3.38E-01	5.13E-01	8.06E-01	1.91E-01	1.00E+00
ENSBTAG00000043474	0.00E+00	1.00E+00	0.00E+00	1.00E+00	-5.13E-15	1.00E+00	0.00E+00	1.00E+00	0.00E+00	1.00E+00
ENSBTAG00000030792	6.62E-03	1.00E+00	6.92E-02	1.00E+00	-1.32E-01	1.00E+00	1.48E+00	4.57E-01	1.17E-02	1.00E+00
ENSBTAG00000045741	0.00E+00	1.00E+00	0.00E+00	1.00E+00	-5.13E-15	1.00E+00	1.76E+00	4.97E-01	0.00E+00	1.00E+00
ENSBTAG00000046304	2.42E+00	1.44E-01	1.73E+00	2.13E-01	1.96E+00	1.13E-01	3.53E+00	1.17E-04	2.26E+00	2.93E-01
ENSBTAG00000011379	-5.34E-01	7.97E-01	-7.65E-01	1.58E-01	-1.47E+00	2.41E-03	-7.73E-01	1.50E-01	-3.94E-01	9.07E-01
ENSBTAG00000017200	-5.98E-01	1.66E-01	-3.91E+00	6.87E-43	-4.44E+00	2.60E-52	-3.42E+00	2.16E-35	2.54E-01	8.58E-01
ENSBTAG00000044915	0.00E+00	1.00E+00	0.00E+00	1.00E+00	-5.13E-15	1.00E+00	0.00E+00	1.00E+00	0.00E+00	1.00E+00
ENSBTAG00000019784	2.91E-01	7.71E-01	7.96E-01	8.23E-04	6.67E-01	6.00E-03	8.59E-01	3.04E-04	1.95E-02	1.00E+00
ENSBTAG00000008815	-1.07E-02	1.00E+00	1.02E+00	2.19E-04	1.72E+00	8.70E-11	7.36E-01	1.22E-02	-5.81E-01	3.22E-01
ENSBTAG00000004430	-4.64E-01	6.66E-01	-1.50E+00	1.20E-05	-2.05E+00	1.45E-09	-1.14E+00	1.24E-03	-6.13E-01	4.36E-01
ENSBTAG00000025450	2.12E-01	9.20E-01	-2.03E+00	1.41E-19	-2.08E+00	1.15E-20	-8.15E-01	5.02E-04	3.23E-01	6.45E-01
ENSBTAG00000004498	1.90E-01	1.00E+00	-1.21E-01	1.00E+00	-1.75E-01	1.00E+00	2.95E-01	1.00E+00	6.26E-01	9.76E-01
ENSBTAG00000044963	0.00E+00	1.00E+00	0.00E+00	1.00E+00	-5.13E-15	1.00E+00	0.00E+00	1.00E+00	0.00E+00	1.00E+00
ENSBTAG00000013836	1.91E-01	1.00E+00	-2.25E+00	4.59E-18	-3.78E+00	3.98E-40	-3.26E+00	5.50E-33	-1.27E-01	1.00E+00
ENSBTAG00000009641	-3.71E-01	4.27E-01	-1.39E+00	2.80E-11	-2.62E+00	4.67E-35	-2.92E+00	3.75E-42	-2.02E-01	8.78E-01
ENSBTAG00000009642	-5.52E-02	1.00E+00	9.17E-02	1.00E+00	-7.39E-01	1.20E-01	-1.33E+00	2.51E-03	-2.38E-01	1.00E+00
ENSBTAG00000009643	2.75E-01	1.00E+00	-7.89E-01	6.84E-02	-1.47E+00	2.20E-04	-5.15E-01	2.91E-01	1.41E-02	1.00E+00
ENSBTAG00000040551	1.57E-01	1.00E+00	-9.04E-01	5.60E-03	-1.05E+00	9.64E-04	-3.65E-01	4.08E-01	1.60E-01	1.00E+00
ENSBTAG00000007807	-2.80E-01	8.84E-01	-8.20E-01	7.97E-03	-1.58E+00	1.05E-07	-1.01E+00	8.52E-04	-3.62E-01	7.62E-01
ENSBTAG00000007809	-5.62E-01	6.39E-01	-1.78E+00	7.13E-05	-1.33E+00	1.86E-03	-1.02E+00	2.08E-02	-8.12E-02	1.00E+00
ENSBTAG00000001551	3.63E-01	8.23E-01	5.49E-01	1.72E-01	1.57E+00	2.37E-06	1.09E+00	1.89E-03	4.96E-01	6.06E-01
ENSBTAG00000004732	6.07E-01	2.24E-01	2.49E-01	5.95E-01	-3.32E-01	4.10E-01	-4.82E-01	1.69E-01	8.98E-01	3.20E-02
ENSBTAG00000028814	0.00E+00	1.00E+00	0.00E+00	1.00E+00	-5.13E-15	1.00E+00	0.00E+00	1.00E+00	0.00E+00	1.00E+00

ENSBTAG00000003469	1.79E-01	9.82E-01	-1.17E-01	8.78E-01	3.51E-01	1.74E-01	3.72E-01	1.50E-01	-2.15E-01	8.38E-01
ENSBTAG00000048112	-8.18E-01	9.38E-01	-1.46E+00	1.99E-01	-1.15E+00	3.50E-01	-6.03E-01	7.73E-01	-1.77E+00	4.24E-01
ENSBTAG00000003474	2.10E-01	1.00E+00	2.58E+00	5.07E-07	3.39E+00	1.04E-11	3.35E+00	2.60E-11	1.08E+00	3.84E-01
ENSBTAG00000005897	8.96E-02	1.00E+00	2.28E-01	5.30E-01	2.04E-01	5.99E-01	-3.19E-03	1.00E+00	2.77E-02	1.00E+00
ENSBTAG00000017994	7.98E-02	1.00E+00	1.99E+00	8.97E-24	1.74E+00	1.01E-18	9.58E-01	2.77E-06	-9.82E-02	1.00E+00
ENSBTAG00000042192	3.58E+00	2.31E-01	0.00E+00	1.00E+00	-5.13E-15	1.00E+00	0.00E+00	1.00E+00	0.00E+00	1.00E+00
ENSBTAG00000038667	0.00E+00	1.00E+00	0.00E+00	1.00E+00	-5.13E-15	1.00E+00	0.00E+00	1.00E+00	0.00E+00	1.00E+00
ENSBTAG00000019819	4.25E-02	1.00E+00	-9.26E-01	2.10E-03	-7.74E-01	1.09E-02	-1.37E-01	9.32E-01	-5.20E-01	4.43E-01
ENSBTAG00000037625	-3.04E-01	1.00E+00	-9.91E-01	1.63E-01	-1.59E+00	1.44E-02	6.70E-02	1.00E+00	1.00E+00	4.16E-01
ENSBTAG00000014234	8.43E-01	1.00E+00	8.19E-01	1.00E+00	-6.85E-02	1.00E+00	4.29E-01	1.00E+00	2.49E-01	1.00E+00
ENSBTAG00000004171	3.89E-01	6.35E-01	1.38E+00	4.34E-07	7.23E-01	1.34E-02	1.04E-01	1.00E+00	1.93E-01	1.00E+00
ENSBTAG00000010239	4.84E-01	1.00E+00	1.20E+00	1.00E-01	1.12E+00	1.33E-01	2.42E-01	1.00E+00	1.10E+00	4.89E-01
ENSBTAG00000011767	-7.07E-02	1.00E+00	-8.66E-01	2.96E-04	-3.15E-01	2.93E-01	3.21E-01	2.88E-01	2.38E-01	8.22E-01
ENSBTAG00000016309	-9.53E-02	1.00E+00	4.11E-02	1.00E+00	-1.54E-01	6.42E-01	-9.99E-02	8.97E-01	-3.66E-01	4.10E-01
ENSBTAG00000016311	3.21E-01	5.81E-01	1.77E+00	6.87E-19	2.05E+00	2.74E-25	1.58E+00	2.24E-15	-6.90E-02	1.00E+00
ENSBTAG00000016255	-4.17E-01	5.76E-01	-1.37E+00	1.43E-06	7.58E-02	1.00E+00	1.40E+00	5.20E-08	-1.48E-02	1.00E+00
ENSBTAG00000012900	5.31E-01	5.07E-01	1.05E+00	1.27E-03	1.56E+00	2.44E-07	8.26E-01	1.63E-02	3.23E-01	8.56E-01
ENSBTAG00000018297	-2.72E-01	1.00E+00	-1.29E+00	3.24E-04	-1.89E+00	1.07E-07	-1.04E+00	4.72E-03	1.38E-01	1.00E+00
ENSBTAG00000018299	-3.35E-01	5.76E-01	-8.10E-01	4.25E-04	1.01E-01	9.52E-01	-5.40E-02	1.00E+00	9.90E-03	1.00E+00
ENSBTAG00000046048	0.00E+00	1.00E+00	0.00E+00	1.00E+00	-5.13E-15	1.00E+00	0.00E+00	1.00E+00	0.00E+00	1.00E+00
ENSBTAG00000045619	-2.15E-01	1.00E+00	-6.28E-02	1.00E+00	-7.57E-01	1.36E-01	-6.26E-01	2.55E-01	-1.92E-01	1.00E+00
ENSBTAG00000001945	5.02E-01	4.66E-01	9.68E-01	1.55E-03	1.13E+00	1.63E-04	1.96E+00	1.96E-11	5.83E-01	3.81E-01
ENSBTAG00000044420	0.00E+00	1.00E+00	0.00E+00	1.00E+00	-5.13E-15	1.00E+00	0.00E+00	1.00E+00	0.00E+00	1.00E+00
ENSBTAG00000001948	2.00E-02	1.00E+00	-2.93E-01	2.35E-01	5.39E-02	1.00E+00	3.10E-01	2.05E-01	-1.20E-01	1.00E+00
ENSBTAG00000001950	-6.17E-01	3.23E-01	-1.07E+00	1.24E-03	-1.64E+00	3.13E-07	-1.16E+00	4.25E-04	2.62E-01	9.51E-01
ENSBTAG00000012838	-2.71E+00	5.76E-01	-2.71E+00	2.42E-01	-1.53E-01	1.00E+00	-9.46E-01	8.04E-01	1.21E-02	1.00E+00
ENSBTAG00000014334	9.73E-02	1.00E+00	-3.40E-01	3.83E-01	3.07E-02	1.00E+00	5.98E-01	5.79E-02	6.31E-01	2.40E-01
ENSBTAG00000018971	2.61E-01	1.00E+00	2.72E-01	6.73E-01	-5.44E-01	2.12E-01	-1.17E+00	1.93E-03	3.93E-01	8.00E-01
ENSBTAG00000045041	0.00E+00	1.00E+00	0.00E+00	1.00E+00	-5.13E-15	1.00E+00	0.00E+00	1.00E+00	0.00E+00	1.00E+00
ENSBTAG00000025434	-5.93E-01	3.61E-01	3.64E-01	4.19E-01	7.45E-01	3.10E-02	-3.85E-02	1.00E+00	6.67E-02	1.00E+00
ENSBTAG00000017477	6.76E-01	1.00E+00	-3.21E+00	1.25E-01	-1.64E-01	1.00E+00	4.75E-01	9.71E-01	2.20E-02	1.00E+00
ENSBTAG00000018255	2.72E-01	6.79E-01	1.94E-01	5.16E-01	-2.46E-01	3.62E-01	-1.40E-02	1.00E+00	3.71E-01	4.32E-01
ENSBTAG00000042854	0.00E+00	1.00E+00	0.00E+00	1.00E+00	-5.13E-15	1.00E+00	0.00E+00	1.00E+00	0.00E+00	1.00E+00
ENSBTAG00000042977	0.00E+00	1.00E+00	0.00E+00	1.00E+00	-5.13E-15	1.00E+00	0.00E+00	1.00E+00	0.00E+00	1.00E+00
ENSBTAG00000021225	-9.87E-02	1.00E+00	-3.18E-01	3.58E-01	5.45E-01	5.31E-02	1.02E+00	8.42E-05	2.83E-01	8.00E-01

ENSBTAG00000010052	-5.85E-01	6.25E-01	-5.10E-01	3.58E-01	1.79E-01	9.60E-01	6.14E-01	2.12E-01	6.02E-02	1.00E+00
ENSBTAG00000009998	-1.66E-01	1.00E+00	-1.19E+00	1.50E-02	-1.72E+00	2.60E-04	-6.40E-01	2.71E-01	-4.03E-01	9.04E-01
ENSBTAG00000017798	-6.44E-02	1.00E+00	3.69E-01	2.91E-01	6.15E-01	3.68E-02	4.46E-01	1.78E-01	-4.25E-01	6.00E-01
ENSBTAG00000029544	0.00E+00	1.00E+00	0.00E+00	1.00E+00	-5.13E-15	1.00E+00	0.00E+00	1.00E+00	0.00E+00	1.00E+00
ENSBTAG00000017811	9.13E-02	1.00E+00	7.60E-01	9.58E-03	1.07E+00	1.37E-04	1.11E+00	9.15E-05	3.57E-01	7.46E-01
ENSBTAG00000008576	3.60E-01	1.00E+00	-4.22E-01	7.77E-01	-3.10E-01	9.01E-01	-5.04E-01	6.52E-01	3.99E-01	1.00E+00
ENSBTAG00000047101	0.00E+00	1.00E+00	0.00E+00	1.00E+00	2.56E+00	2.54E-01	0.00E+00	1.00E+00	3.24E+00	4.34E-01
ENSBTAG00000042896	-4.13E+00	8.50E-02	-8.30E-01	6.61E-01	-2.36E+00	9.83E-02	-4.13E+00	1.21E-02	-2.22E+00	4.75E-01
ENSBTAG00000003857	1.32E-01	1.00E+00	4.93E-01	2.14E-02	4.81E-01	2.51E-02	2.72E-01	3.02E-01	7.91E-02	1.00E+00
ENSBTAG00000011596	-3.67E-01	5.72E-01	-2.07E-01	5.91E-01	-4.93E-01	6.24E-02	-1.01E+00	2.53E-05	3.18E-01	7.02E-01
ENSBTAG00000001881	-4.00E-01	6.82E-01	-1.31E+00	8.40E-06	-3.01E+00	7.53E-24	-4.19E+00	7.84E-41	-5.05E-01	4.95E-01
ENSBTAG00000030599	1.19E-01	1.00E+00	-1.40E+00	5.93E-10	-2.28E+00	1.39E-23	-2.39E+00	1.61E-25	5.50E-01	1.90E-01
ENSBTAG00000044273	0.00E+00	1.00E+00	0.00E+00	1.00E+00	-5.13E-15	1.00E+00	0.00E+00	1.00E+00	0.00E+00	1.00E+00
ENSBTAG00000027080	-7.48E-01	7.71E-01	2.14E-01	1.00E+00	-7.48E-01	3.37E-01	-7.05E-01	3.88E-01	-1.33E-01	1.00E+00
ENSBTAG00000045774	0.00E+00	1.00E+00	0.00E+00	1.00E+00	-5.13E-15	1.00E+00	0.00E+00	1.00E+00	0.00E+00	1.00E+00
ENSBTAG00000007586	6.61E-02	1.00E+00	1.05E+00	5.54E-06	9.15E-01	8.44E-05	4.75E-01	7.59E-02	-7.30E-04	1.00E+00
ENSBTAG00000030595	6.82E-02	1.00E+00	1.81E-01	5.90E-01	-6.64E-02	1.00E+00	-3.63E-01	1.46E-01	-2.99E-01	6.30E-01
ENSBTAG00000037932	-1.91E+00	8.23E-01	8.83E-01	8.52E-01	-1.30E-01	1.00E+00	-1.46E-01	1.00E+00	1.70E+00	7.71E-01
ENSBTAG00000046600	0.00E+00	1.00E+00	0.00E+00	1.00E+00	-5.13E-15	1.00E+00	0.00E+00	1.00E+00	0.00E+00	1.00E+00
ENSBTAG00000003715	2.62E+00	5.58E-02	2.98E+00	1.68E-03	1.47E+00	2.99E-01	1.78E+00	1.61E-01	2.14E+00	3.11E-01
ENSBTAG00000038377	2.29E-01	1.00E+00	1.19E+00	4.97E-02	5.25E-01	5.49E-01	4.00E-01	7.48E-01	-6.77E-01	8.34E-01
ENSBTAG00000045679	-3.63E-01	9.14E-01	-3.81E-01	5.13E-01	-3.29E-02	1.00E+00	1.85E-01	9.12E-01	-4.86E-01	7.72E-01
ENSBTAG00000006786	0.00E+00	1.00E+00	0.00E+00	1.00E+00	-5.13E-15	1.00E+00	0.00E+00	1.00E+00	0.00E+00	1.00E+00
ENSBTAG00000039950	0.00E+00	1.00E+00	0.00E+00	1.00E+00	-5.13E-15	1.00E+00	0.00E+00	1.00E+00	0.00E+00	1.00E+00
ENSBTAG00000045604	-2.69E-01	1.00E+00	5.01E-01	8.52E-01	8.88E-02	1.00E+00	4.84E-01	8.56E-01	9.43E-01	8.00E-01
ENSBTAG00000005700	1.69E-01	1.00E+00	1.12E-02	1.00E+00	2.37E-01	9.87E-01	1.03E+00	9.28E-02	1.56E-01	1.00E+00
ENSBTAG00000012565	1.20E-02	1.00E+00	-1.12E+00	2.98E-06	-1.14E+00	1.38E-06	-4.47E-02	1.00E+00	2.40E-01	8.32E-01
ENSBTAG00000005395	-6.36E-01	1.00E+00	-5.51E-01	8.22E-01	-1.66E-01	1.00E+00	3.62E-01	9.41E-01	-6.49E-01	1.00E+00
ENSBTAG00000025385	-5.94E-03	1.00E+00	3.69E-02	1.00E+00	2.34E-01	6.68E-01	3.47E-01	4.29E-01	4.48E-01	6.06E-01
ENSBTAG00000015805	-6.14E-02	1.00E+00	-5.26E-01	4.08E-01	-8.45E-01	1.06E-01	-1.17E+00	1.89E-02	1.96E-01	1.00E+00
ENSBTAG00000048139	0.00E+00	1.00E+00	0.00E+00	1.00E+00	-5.13E-15	1.00E+00	0.00E+00	1.00E+00	0.00E+00	1.00E+00
ENSBTAG00000014189	2.86E-02	1.00E+00	-4.23E-01	8.10E-01	-5.59E-01	6.15E-01	4.82E-01	6.38E-01	-7.91E-02	1.00E+00
ENSBTAG00000025458	5.76E-02	1.00E+00	-1.59E+00	3.69E-07	-2.22E+00	1.32E-12	-1.82E+00	4.62E-09	-2.97E-01	8.52E-01
ENSBTAG00000046952	-5.90E-01	6.23E-01	-4.70E-01	4.23E-01	-7.14E-01	1.38E-01	-4.24E-01	4.70E-01	-1.14E+00	1.26E-01
ENSBTAG00000008713	-1.74E-01	1.00E+00	-1.24E+00	7.64E-08	6.04E-01	1.20E-02	6.43E-01	7.94E-03	3.50E-02	1.00E+00

ENSBTAG00000011041	1.51E-01	1.00E+00	6.87E-01	1.97E-03	5.69E-02	1.00E+00	-4.00E-01	1.18E-01	1.68E-01	9.89E-01
ENSBTAG00000017177	3.88E-02	1.00E+00	2.95E-01	3.47E-01	5.84E-01	1.95E-02	2.74E-01	4.13E-01	-1.29E-01	1.00E+00
ENSBTAG00000011757	-2.00E-02	1.00E+00	-5.52E-02	1.00E+00	-2.84E-02	1.00E+00	1.96E-01	4.97E-01	2.26E-02	1.00E+00
ENSBTAG00000011680	4.90E-01	1.00E+00	1.06E+00	1.42E-01	-1.93E-01	1.00E+00	5.13E-01	6.53E-01	5.06E-01	9.90E-01
ENSBTAG00000011683	2.07E-01	9.59E-01	-1.24E+00	1.88E-07	-1.13E+00	1.79E-06	-4.52E-01	1.07E-01	4.23E-02	1.00E+00
ENSBTAG00000002842	2.06E-01	1.00E+00	-1.50E+00	1.39E-04	-1.71E+00	8.36E-06	-1.85E+00	2.13E-06	-2.85E-01	9.70E-01
ENSBTAG00000025372	0.00E+00	1.00E+00	0.00E+00	1.00E+00	-5.13E-15	1.00E+00	0.00E+00	1.00E+00	0.00E+00	1.00E+00
ENSBTAG00000046814	-2.35E-01	9.83E-01	-1.49E+00	5.38E-07	-1.99E+00	1.81E-11	-1.34E+00	4.38E-06	3.63E-01	7.26E-01
ENSBTAG00000002843	-4.83E-01	2.65E-01	-2.41E+00	2.11E-23	-2.30E+00	3.95E-22	-2.08E+00	2.13E-18	-4.21E-01	4.60E-01
ENSBTAG00000010270	-3.27E-01	1.00E+00	-1.86E+00	5.10E-03	-1.45E+00	1.91E-02	-1.36E+00	3.03E-02	3.00E-01	1.00E+00
ENSBTAG00000009788	-5.21E-01	2.55E-01	-2.34E+00	3.23E-20	-4.28E+00	2.43E-54	-2.90E+00	2.65E-29	1.39E-01	1.00E+00
ENSBTAG00000009783	-1.70E-01	1.00E+00	7.91E-03	1.00E+00	2.92E-01	6.34E-01	1.52E-01	9.85E-01	-4.48E-01	7.68E-01
ENSBTAG00000015930	1.24E-01	1.00E+00	-1.04E+00	1.28E-02	-1.60E+00	6.81E-05	-6.50E-01	1.57E-01	5.79E-01	5.84E-01
ENSBTAG00000009271	-4.64E-01	7.83E-01	5.10E-01	2.62E-01	-2.15E-01	8.37E-01	4.89E-01	2.94E-01	-4.12E-01	8.00E-01
ENSBTAG00000009272	1.65E+00	6.74E-03	2.92E+00	4.46E-09	1.25E+00	1.52E-02	1.74E-01	1.00E+00	3.06E-01	1.00E+00
ENSBTAG00000003747	9.27E-01	4.54E-03	9.93E-01	2.25E-04	8.51E-02	1.00E+00	-3.58E-01	3.11E-01	-1.62E-01	1.00E+00
ENSBTAG00000020327	9.23E-02	1.00E+00	1.46E+00	5.04E-15	2.52E-01	3.23E-01	8.73E-02	9.63E-01	-9.01E-02	1.00E+00
ENSBTAG00000020329	9.58E-02	1.00E+00	2.03E+00	1.02E-11	1.56E+00	1.80E-07	1.53E-01	9.26E-01	-3.27E-01	8.17E-01
ENSBTAG00000020331	2.43E-01	8.78E-01	9.89E-01	5.90E-05	3.86E-01	1.98E-01	-6.25E-02	1.00E+00	-1.17E-01	1.00E+00
ENSBTAG00000020334	-3.05E-01	8.23E-01	-3.23E-01	4.34E-01	-5.94E-01	6.48E-02	-9.51E-01	1.52E-03	-4.06E-03	1.00E+00
ENSBTAG00000018467	3.60E-01	3.33E-01	8.37E-02	9.62E-01	-4.32E-01	3.54E-02	-7.71E-01	5.11E-05	3.76E-03	1.00E+00
ENSBTAG00000018469	4.19E-01	8.18E-01	3.84E-01	4.52E-01	1.17E-01	1.00E+00	2.70E-01	6.93E-01	1.13E+00	3.13E-02
ENSBTAG00000030557	1.17E+00	2.46E-05	1.01E+00	6.94E-05	9.45E-01	1.94E-04	1.05E+00	3.66E-05	3.95E-01	6.01E-01
ENSBTAG00000014632	-1.67E+00	7.80E-01	9.60E-02	1.00E+00	-3.59E+00	4.54E-02	-1.06E+00	5.95E-01	-3.59E+00	3.09E-01
ENSBTAG00000014633	-8.63E-03	1.00E+00	-6.40E-01	1.29E-01	-5.13E-01	2.58E-01	-5.48E-01	2.29E-01	4.97E-02	1.00E+00
ENSBTAG00000005774	0.00E+00	1.00E+00	2.79E+00	2.10E-01	1.78E+00	4.79E-01	1.76E+00	4.97E-01	0.00E+00	1.00E+00
ENSBTAG00000004218	-3.61E+00	2.29E-06	-4.20E+00	3.70E-08	-3.77E+00	4.99E-08	-1.75E+00	3.51E-03	6.49E-02	1.00E+00
ENSBTAG00000021955	-1.18E-01	1.00E+00	-5.99E-01	2.24E-03	-9.96E-01	9.02E-08	-7.62E-01	7.59E-05	-2.43E-01	7.51E-01
ENSBTAG00000043732	0.00E+00	1.00E+00	0.00E+00	1.00E+00	-5.13E-15	1.00E+00	0.00E+00	1.00E+00	0.00E+00	1.00E+00
ENSBTAG00000021956	1.37E-01	1.00E+00	-1.31E-01	8.51E-01	8.40E-01	1.05E-04	1.20E+00	1.43E-08	1.52E-01	1.00E+00
ENSBTAG00000021957	-2.11E-01	1.00E+00	-1.05E+00	2.80E-03	-1.44E+00	2.30E-05	-1.68E+00	1.05E-06	-2.23E-01	1.00E+00
ENSBTAG00000020379	-4.45E-01	3.10E-01	-1.86E+00	1.34E-16	-1.94E+00	4.93E-18	-2.07E+00	4.33E-20	6.89E-01	4.56E-02
ENSBTAG00000020381	2.16E-01	9.80E-01	1.23E+00	3.75E-07	1.18E+00	9.56E-07	6.07E-01	2.73E-02	-3.30E-01	7.77E-01
ENSBTAG00000008287	-9.15E-02	1.00E+00	3.18E-01	2.05E-01	2.47E-01	3.76E-01	9.47E-02	9.51E-01	8.72E-02	1.00E+00
ENSBTAG00000015052	4.53E-01	9.32E-01	1.70E+00	7.00E-04	8.01E-01	1.78E-01	-1.04E+00	8.68E-02	7.16E-01	6.25E-01

ENSBTAG00000006463	-1.43E-01	1.00E+00	-3.48E-01	3.27E-01	9.59E-02	1.00E+00	6.02E-01	4.53E-02	1.02E-01	1.00E+00
ENSBTAG00000006688	-9.69E-01	3.70E-01	-2.22E+00	1.07E-04	-2.32E+00	3.93E-05	-1.47E+00	9.96E-03	-2.17E-01	1.00E+00
ENSBTAG00000013688	-1.39E-01	1.00E+00	-2.70E+00	3.75E-02	-2.90E+00	1.82E-02	-3.54E-01	9.66E-01	3.02E-01	1.00E+00
ENSBTAG00000008664	-3.19E-01	7.68E-01	2.85E-01	4.36E-01	9.19E-01	3.04E-04	8.78E-01	7.35E-04	-9.64E-02	1.00E+00
ENSBTAG00000019279	-2.75E-01	7.67E-01	-1.28E+00	1.03E-08	-3.18E-01	2.48E-01	-5.17E-01	3.47E-02	-1.39E-01	1.00E+00
ENSBTAG00000013992	-1.27E-01	1.00E+00	-5.52E-01	1.69E-01	5.43E-01	1.38E-01	1.13E+00	3.21E-04	-5.45E-02	1.00E+00
ENSBTAG00000013983	-4.94E-01	1.00E+00	-6.43E-01	5.48E-01	-3.43E-01	8.80E-01	8.61E-01	2.20E-01	5.36E-03	1.00E+00
ENSBTAG00000016612	1.45E-01	1.00E+00	5.41E-01	5.07E-02	7.03E-01	7.20E-03	8.30E-01	1.40E-03	2.65E-01	8.00E-01
ENSBTAG00000005694	2.84E-01	8.40E-01	6.40E-01	3.15E-02	6.82E-01	2.01E-02	1.10E+00	8.06E-05	8.14E-01	5.32E-02
ENSBTAG00000004322	4.46E+00	1.05E-08	3.33E+00	2.78E-06	4.20E+00	6.02E-09	4.88E+00	5.78E-11	-8.47E-01	7.84E-01
ENSBTAG00000009451	1.24E+00	3.70E-04	1.35E-01	9.65E-01	-9.05E-01	4.68E-03	-1.23E+00	9.69E-05	-2.77E-01	9.00E-01
ENSBTAG00000025405	1.95E+00	5.49E-07	2.71E+00	5.58E-15	3.85E-01	5.14E-01	2.11E-01	8.97E-01	-1.07E-02	1.00E+00
ENSBTAG00000040078	6.15E-01	3.37E-01	2.21E+00	1.50E-12	2.06E+00	3.32E-11	1.55E+00	1.14E-06	6.15E-01	4.09E-01
ENSBTAG00000045886	-2.69E-01	1.00E+00	-9.37E-01	2.40E-02	-2.43E-01	7.91E-01	2.07E-01	8.83E-01	4.47E-01	8.00E-01
ENSBTAG00000035662	-7.54E-02	1.00E+00	-1.25E+00	4.56E-05	-2.49E+00	2.05E-15	-3.04E+00	1.56E-20	3.18E-01	8.00E-01
ENSBTAG00000026909	2.25E-01	8.23E-01	2.10E+00	6.89E-25	2.50E+00	4.75E-34	2.23E+00	8.92E-28	-1.13E-01	1.00E+00
ENSBTAG00000015797	1.92E+00	8.23E-01	3.68E+00	5.09E-02	-5.13E-15	1.00E+00	0.00E+00	1.00E+00	0.00E+00	1.00E+00
ENSBTAG00000005489	2.43E-01	8.97E-01	1.87E+00	1.97E-14	2.18E+00	3.56E-19	1.66E+00	1.24E-11	-1.04E-02	1.00E+00
ENSBTAG00000011985	3.72E-01	1.00E+00	5.23E-01	1.00E+00	1.09E+00	8.10E-01	1.15E+00	7.92E-01	4.76E-01	1.00E+00
ENSBTAG00000011987	-1.39E-01	1.00E+00	-6.64E-01	1.37E-01	-1.04E+00	9.67E-03	-3.82E-01	4.97E-01	4.50E-01	8.00E-01
ENSBTAG00000025403	-1.82E-01	1.00E+00	-4.57E-01	1.06E-01	2.12E-01	5.78E-01	5.83E-01	2.58E-02	1.51E-01	1.00E+00
ENSBTAG00000012004	1.87E+00	9.25E-03	2.19E+00	1.42E-04	1.68E+00	4.28E-03	1.27E+00	4.66E-02	-4.63E-01	9.87E-01
ENSBTAG00000012005	1.92E-01	1.00E+00	-8.31E-01	1.84E-02	-5.35E-01	1.61E-01	-4.71E-01	2.49E-01	-2.45E-01	9.93E-01
ENSBTAG00000003084	1.28E-01	1.00E+00	6.84E-01	3.56E-02	4.33E-02	1.00E+00	-2.40E-01	6.58E-01	5.32E-02	1.00E+00
ENSBTAG00000012285	3.82E-01	1.00E+00	4.84E-01	8.70E-01	-1.75E-01	1.00E+00	3.44E-01	1.00E+00	2.22E-01	1.00E+00
ENSBTAG00000044962	0.00E+00	1.00E+00	0.00E+00	1.00E+00	-5.13E-15	1.00E+00	0.00E+00	1.00E+00	0.00E+00	1.00E+00
ENSBTAG00000008735	1.87E-01	1.00E+00	3.94E-01	2.10E-01	1.35E-01	8.87E-01	-4.72E-01	1.35E-01	-3.49E-01	7.26E-01
ENSBTAG00000019454	-4.86E-01	7.28E-01	-1.88E+00	8.50E-07	-1.27E+00	9.81E-04	-7.46E-01	8.55E-02	-1.84E-01	1.00E+00
ENSBTAG00000013181	-1.17E+00	8.70E-01	9.98E-02	1.00E+00	-2.10E+00	1.75E-01	-1.85E-01	1.00E+00	-1.16E+00	8.42E-01
ENSBTAG00000025329	1.53E+00	4.44E-09	1.22E+00	8.35E-07	6.21E-01	2.09E-02	1.32E-01	8.96E-01	4.99E-01	3.64E-01
ENSBTAG00000047564	0.00E+00	1.00E+00	0.00E+00	1.00E+00	-5.13E-15	1.00E+00	0.00E+00	1.00E+00	0.00E+00	1.00E+00
ENSBTAG00000009923	-2.92E-01	8.03E-01	-4.28E-01	1.38E-01	-2.95E-02	1.00E+00	1.92E-01	6.70E-01	-1.56E-01	1.00E+00
ENSBTAG00000014384	3.22E+00	3.75E-01	0.00E+00	1.00E+00	1.78E+00	4.79E-01	0.00E+00	1.00E+00	2.72E+00	6.01E-01
ENSBTAG00000016283	-1.90E-01	1.00E+00	-1.42E-01	1.00E+00	-1.06E+00	1.30E-01	-2.53E-01	9.51E-01	-2.09E-01	1.00E+00
ENSBTAG00000016285	-7.87E-01	1.00E+00	2.70E+00	7.07E-03	-1.42E-01	1.00E+00	-9.40E-01	8.06E-01	-2.70E+00	6.01E-01

ENSBTAG00000002704	2.50E-01	9.66E-01	9.69E-01	1.21E-03	8.89E-01	3.26E-03	8.27E-01	8.01E-03	5.58E-01	4.05E-01
ENSBTAG00000002706	4.19E-02	1.00E+00	-7.74E-01	1.99E-03	-1.76E+00	1.45E-13	-1.52E+00	2.21E-10	2.16E-02	1.00E+00
ENSBTAG00000020469	3.63E-01	1.00E+00	5.80E-01	4.01E-01	1.60E+00	2.90E-04	1.91E+00	8.47E-06	1.54E+00	1.26E-02
ENSBTAG00000020471	-2.27E-01	1.00E+00	-1.14E+00	5.03E-02	-1.21E-01	1.00E+00	8.53E-01	8.90E-02	7.76E-01	4.73E-01
ENSBTAG00000020474	-2.11E-01	1.00E+00	1.59E+00	1.14E-01	7.75E-01	6.02E-01	-1.49E+00	3.15E-01	-9.44E-01	9.17E-01
ENSBTAG00000042489	0.00E+00	1.00E+00	0.00E+00	1.00E+00	-5.13E-15	1.00E+00	0.00E+00	1.00E+00	0.00E+00	1.00E+00
ENSBTAG00000020475	2.45E-01	8.23E-01	5.92E-01	9.18E-03	-1.86E-03	1.00E+00	3.05E-01	2.75E-01	2.37E-01	8.00E-01
ENSBTAG00000020477	1.03E+00	3.14E-05	2.62E+00	5.91E-32	1.48E+00	1.48E-11	6.69E-01	4.91E-03	1.34E-01	1.00E+00
ENSBTAG00000039353	-1.16E+00	8.77E-01	-3.88E+00	3.63E-02	-1.32E+00	4.19E-01	-1.34E+00	4.20E-01	1.68E-02	1.00E+00
ENSBTAG00000020480	3.43E-01	7.37E-01	9.72E-01	2.91E-04	3.85E-01	2.48E-01	7.01E-01	1.43E-02	3.09E-01	8.00E-01
ENSBTAG00000008133	2.93E-01	6.90E-01	1.17E+00	2.65E-08	1.31E+00	2.49E-10	6.95E-01	1.99E-03	-8.15E-03	1.00E+00
ENSBTAG00000008135	1.53E-01	1.00E+00	3.15E-01	4.54E-01	1.31E-01	9.62E-01	-8.02E-02	1.00E+00	-4.56E-01	6.01E-01
ENSBTAG00000008136	3.04E-01	5.00E-01	1.11E+00	7.96E-10	8.33E-01	4.98E-06	6.34E-01	9.05E-04	-5.84E-02	1.00E+00
ENSBTAG00000043716	0.00E+00	1.00E+00	0.00E+00	1.00E+00	-5.13E-15	1.00E+00	0.00E+00	1.00E+00	0.00E+00	1.00E+00
ENSBTAG00000012106	-4.52E-01	4.26E-01	-8.01E-01	3.47E-03	-8.08E-02	1.00E+00	-1.61E-01	8.03E-01	1.22E-01	1.00E+00
ENSBTAG00000025324	-6.41E-01	8.23E-01	1.39E-01	1.00E+00	-3.83E-01	7.70E-01	1.07E+00	9.52E-02	-1.24E-01	1.00E+00
ENSBTAG00000028362	0.00E+00	1.00E+00	0.00E+00	1.00E+00	-5.13E-15	1.00E+00	0.00E+00	1.00E+00	0.00E+00	1.00E+00
ENSBTAG00000001605	-1.91E+00	8.23E-01	-1.91E+00	4.69E-01	6.45E-01	9.78E-01	-1.46E-01	1.00E+00	1.83E-02	1.00E+00
ENSBTAG00000040167	-2.71E-01	9.82E-01	-1.35E-02	1.00E+00	3.38E-01	4.51E-01	2.76E-01	5.97E-01	-1.59E-01	1.00E+00
ENSBTAG00000047260	8.96E-02	1.00E+00	-1.15E+00	1.21E-01	-1.92E+00	6.67E-03	-2.84E-01	9.01E-01	-1.01E-02	1.00E+00
ENSBTAG00000021708	4.20E-01	8.22E-01	-3.59E-01	5.45E-01	5.02E-01	2.58E-01	8.34E-01	2.89E-02	-7.34E-02	1.00E+00
ENSBTAG00000047322	0.00E+00	1.00E+00	0.00E+00	1.00E+00	-5.13E-15	1.00E+00	0.00E+00	1.00E+00	0.00E+00	1.00E+00
ENSBTAG00000017489	-1.70E+00	3.76E-01	-5.04E-01	8.16E-01	-4.74E-01	8.20E-01	6.46E-01	5.74E-01	-2.25E-01	1.00E+00
ENSBTAG00000013652	-9.42E-02	1.00E+00	-1.26E+00	1.38E-05	2.20E-01	6.69E-01	3.72E-01	3.31E-01	-2.37E-02	1.00E+00
ENSBTAG00000042272	0.00E+00	1.00E+00	0.00E+00	1.00E+00	-5.13E-15	1.00E+00	0.00E+00	1.00E+00	0.00E+00	1.00E+00
ENSBTAG00000046331	0.00E+00	1.00E+00	0.00E+00	1.00E+00	-5.13E-15	1.00E+00	0.00E+00	1.00E+00	0.00E+00	1.00E+00
ENSBTAG00000025308	2.89E-01	8.23E-01	5.46E-01	5.75E-02	-8.56E-03	1.00E+00	7.73E-02	1.00E+00	2.59E-01	8.42E-01
ENSBTAG00000008083	9.44E-01	4.82E-02	3.59E+00	1.59E-24	2.68E+00	3.15E-15	2.18E+00	1.54E-10	1.41E+00	1.14E-03
ENSBTAG00000045343	0.00E+00	1.00E+00	0.00E+00	1.00E+00	-5.13E-15	1.00E+00	0.00E+00	1.00E+00	0.00E+00	1.00E+00
ENSBTAG00000018039	0.00E+00	1.00E+00	0.00E+00	1.00E+00	-5.13E-15	1.00E+00	0.00E+00	1.00E+00	0.00E+00	1.00E+00
ENSBTAG00000043728	0.00E+00	1.00E+00	0.00E+00	1.00E+00	1.78E+00	4.79E-01	0.00E+00	1.00E+00	0.00E+00	1.00E+00
ENSBTAG00000043070	0.00E+00	1.00E+00	0.00E+00	1.00E+00	-5.13E-15	1.00E+00	1.76E+00	4.97E-01	0.00E+00	1.00E+00
ENSBTAG00000021393	1.04E+00	5.55E-01	2.05E+00	2.90E-03	1.17E+00	1.31E-01	2.23E+00	1.24E-03	9.00E-01	6.95E-01
ENSBTAG00000002900	6.55E-01	1.52E-01	1.71E+00	1.03E-09	1.70E+00	1.01E-09	1.58E+00	2.05E-08	1.29E+00	1.83E-04
ENSBTAG00000001124	5.66E-01	2.28E-01	-5.55E-01	7.17E-02	-4.34E-02	1.00E+00	-3.67E-02	1.00E+00	6.48E-01	1.81E-01

ENSBTAG00000012737	1.13E+00	2.30E-02	9.45E-01	2.10E-02	3.95E-02	1.00E+00	-2.05E-01	9.13E-01	-4.89E-01	8.00E-01
ENSBTAG00000019355	-1.74E+00	1.44E-01	-1.68E+00	4.05E-02	-1.39E+00	7.27E-02	-1.59E+00	4.37E-02	-2.28E+00	7.31E-02
ENSBTAG00000030456	8.64E-02	1.00E+00	-1.68E+00	1.36E-11	-9.08E-01	3.14E-04	-9.07E-01	3.99E-04	9.64E-02	1.00E+00
ENSBTAG00000018596	-2.46E-01	1.00E+00	-1.00E-01	1.00E+00	-3.05E-01	6.12E-01	-1.53E-01	9.88E-01	1.24E-01	1.00E+00
ENSBTAG00000030453	3.33E-01	5.44E-01	1.17E+00	1.06E-08	1.20E+00	3.48E-09	5.80E-01	9.52E-03	2.11E-02	1.00E+00
ENSBTAG00000009994	-4.69E-02	1.00E+00	-1.25E-01	1.00E+00	1.10E+00	7.21E-04	1.09E+00	9.92E-04	1.58E-02	1.00E+00
ENSBTAG00000030432	-1.82E-01	1.00E+00	-2.31E+00	7.60E-11	-1.72E+00	3.17E-07	-1.15E+00	8.15E-04	-2.06E-03	1.00E+00
ENSBTAG00000044964	0.00E+00	1.00E+00	0.00E+00	1.00E+00	-5.13E-15	1.00E+00	0.00E+00	1.00E+00	0.00E+00	1.00E+00
ENSBTAG00000046684	-4.07E-02	1.00E+00	-6.45E-01	3.50E-02	-1.24E+00	1.06E-05	-3.35E-01	3.84E-01	6.70E-01	1.62E-01
ENSBTAG00000013136	-4.37E-02	1.00E+00	-9.58E-01	2.53E-05	-1.32E+00	3.09E-09	-1.10E+00	9.41E-07	-3.64E-01	5.37E-01
ENSBTAG00000013138	-1.22E-01	1.00E+00	-1.50E+00	1.71E-07	-9.11E-01	1.51E-03	-1.64E-01	8.30E-01	5.35E-01	3.68E-01
ENSBTAG00000045849	1.31E+00	9.31E-01	2.31E+00	1.14E-01	2.61E+00	4.42E-02	1.78E+00	3.04E-01	1.97E+00	6.01E-01
ENSBTAG00000005426	-8.36E-02	1.00E+00	2.00E-02	1.00E+00	2.71E-01	3.42E-01	1.81E-01	6.24E-01	-2.83E-01	7.23E-01
ENSBTAG00000005427	9.39E-02	1.00E+00	8.83E-01	2.85E-05	1.39E+00	5.81E-12	1.34E+00	4.87E-11	6.41E-01	4.64E-02
ENSBTAG00000045016	1.92E+00	8.23E-01	1.98E+00	4.43E-01	1.78E+00	4.79E-01	0.00E+00	1.00E+00	0.00E+00	1.00E+00
ENSBTAG00000025644	-6.32E-02	1.00E+00	-1.69E-01	5.83E-01	-1.66E-01	5.90E-01	-2.18E-01	4.33E-01	-2.09E-01	8.00E-01
ENSBTAG00000006392	1.43E-01	1.00E+00	-3.69E-01	3.35E-01	-6.03E-01	5.98E-02	-9.77E-01	1.19E-03	4.08E-02	1.00E+00
ENSBTAG00000043774	0.00E+00	1.00E+00	0.00E+00	1.00E+00	-5.13E-15	1.00E+00	0.00E+00	1.00E+00	0.00E+00	1.00E+00
ENSBTAG00000010906	-1.02E-01	1.00E+00	-7.21E-01	3.81E-02	-5.85E-01	1.06E-01	-3.69E-01	4.09E-01	-5.72E-02	1.00E+00
ENSBTAG00000044381	9.54E-01	8.23E-01	-5.77E-01	8.91E-01	-3.88E+00	2.26E-02	-1.35E+00	4.18E-01	4.73E-01	1.00E+00
ENSBTAG00000012545	4.04E-02	1.00E+00	-2.72E-01	2.67E-01	-3.53E-01	1.15E-01	-1.30E-01	7.65E-01	9.15E-02	1.00E+00
ENSBTAG00000045029	0.00E+00	1.00E+00	0.00E+00	1.00E+00	-5.13E-15	1.00E+00	0.00E+00	1.00E+00	0.00E+00	1.00E+00
ENSBTAG00000015385	1.88E-01	1.00E+00	3.44E-01	2.70E-01	6.49E-01	1.07E-02	7.70E-01	2.18E-03	-9.54E-02	1.00E+00
ENSBTAG00000000359	3.00E-01	5.14E-01	1.54E+00	6.64E-18	1.96E+00	3.58E-28	1.73E+00	2.57E-22	-2.08E-01	8.00E-01
ENSBTAG00000000362	-3.84E-03	1.00E+00	-8.51E-01	1.88E-05	-4.40E-01	4.27E-02	-2.94E-01	2.42E-01	2.25E-01	8.00E-01
ENSBTAG00000045234	0.00E+00	1.00E+00	0.00E+00	1.00E+00	-5.13E-15	1.00E+00	0.00E+00	1.00E+00	0.00E+00	1.00E+00
ENSBTAG00000044563	0.00E+00	1.00E+00	0.00E+00	1.00E+00	-5.13E-15	1.00E+00	0.00E+00	1.00E+00	0.00E+00	1.00E+00
ENSBTAG00000001558	-1.62E+00	5.76E-01	-2.49E+00	5.68E-02	-2.38E+00	5.74E-02	-6.23E-01	8.05E-01	-5.56E-01	1.00E+00
ENSBTAG00000012330	-5.29E-03	1.00E+00	-1.13E-02	1.00E+00	-4.14E-01	5.00E-01	-5.44E-01	3.37E-01	-3.10E-01	1.00E+00
ENSBTAG00000007516	-3.07E-01	7.18E-01	1.44E-01	8.12E-01	3.91E-01	1.49E-01	5.31E-01	3.62E-02	-1.72E-01	1.00E+00
ENSBTAG00000007518	5.30E-01	1.00E+00	-7.16E-01	9.45E-01	-2.70E+00	1.99E-01	1.78E+00	1.65E-01	-7.73E-01	1.00E+00
ENSBTAG00000005954	6.88E-01	1.00E+00	2.36E+00	1.12E-02	9.55E-01	5.44E-01	-1.46E+00	4.94E-01	3.94E-03	1.00E+00
ENSBTAG00000038257	0.00E+00	1.00E+00	0.00E+00	1.00E+00	1.78E+00	4.79E-01	0.00E+00	1.00E+00	0.00E+00	1.00E+00
ENSBTAG00000020646	4.13E-01	1.00E+00	-1.48E+00	1.34E-02	-1.94E+00	7.94E-04	-2.73E+00	4.77E-06	6.82E-01	7.57E-01
ENSBTAG00000015965	-2.35E-01	9.25E-01	6.58E-01	1.27E-02	1.58E+00	4.14E-11	1.07E+00	1.68E-05	-2.03E-01	9.69E-01

ENSBTAG00000009230	7.65E-01	1.00E+00	6.43E-01	7.89E-01	1.42E+00	1.68E-01	-1.82E-01	1.00E+00	5.50E-01	1.00E+00
ENSBTAG00000012099	2.40E-01	9.65E-01	1.90E-01	7.59E-01	4.28E-01	2.09E-01	6.50E-01	3.53E-02	3.34E-01	7.97E-01
ENSBTAG00000044282	0.00E+00	1.00E+00	0.00E+00	1.00E+00	-5.13E-15	1.00E+00	0.00E+00	1.00E+00	0.00E+00	1.00E+00
ENSBTAG00000045125	0.00E+00	1.00E+00	0.00E+00	1.00E+00	3.06E+00	1.36E-01	0.00E+00	1.00E+00	1.91E+00	8.00E-01
ENSBTAG00000005828	-4.68E-01	5.68E-01	-2.52E+00	2.61E-16	-1.91E+00	1.93E-10	-2.05E+00	1.60E-11	-4.34E-02	1.00E+00
ENSBTAG00000045153	-2.70E+00	5.76E-01	-2.70E+00	2.42E-01	-2.70E+00	1.99E-01	-2.70E+00	2.05E-01	-2.70E+00	6.01E-01
ENSBTAG00000027991	2.34E-01	9.44E-01	7.65E-01	5.17E-03	5.30E-01	7.29E-02	2.37E-01	5.78E-01	5.61E-02	1.00E+00
ENSBTAG00000042295	0.00E+00	1.00E+00	0.00E+00	1.00E+00	-5.13E-15	1.00E+00	0.00E+00	1.00E+00	0.00E+00	1.00E+00
ENSBTAG00000023163	2.07E+00	1.53E-01	2.62E+00	2.80E-03	1.13E+00	4.28E-01	-1.46E+00	4.93E-01	2.36E+00	9.47E-02
ENSBTAG00000024105	1.21E-01	1.00E+00	-7.98E-01	1.47E-01	-8.72E-01	9.96E-02	2.25E-01	9.17E-01	3.79E-01	9.37E-01
ENSBTAG00000004297	-3.00E-01	1.00E+00	-2.64E-01	8.50E-01	-5.23E-01	4.14E-01	-3.60E-01	6.58E-01	-7.57E-01	5.52E-01
ENSBTAG00000021181	-2.54E-01	1.00E+00	-1.21E+00	8.24E-03	-9.97E-01	2.62E-02	-5.22E-01	3.32E-01	-5.49E-01	6.98E-01
ENSBTAG00000046419	-1.16E+00	4.25E-01	-2.41E+00	2.51E-03	-1.85E+00	1.27E-02	-2.00E+00	8.39E-03	3.60E-01	1.00E+00
ENSBTAG00000044987	0.00E+00	1.00E+00	0.00E+00	1.00E+00	-5.13E-15	1.00E+00	0.00E+00	1.00E+00	0.00E+00	1.00E+00
ENSBTAG00000045538	-3.59E-01	9.72E-01	-5.50E-01	3.28E-01	-8.82E-01	6.45E-02	-1.03E+00	2.94E-02	-2.80E-01	1.00E+00
ENSBTAG00000007675	5.84E-02	1.00E+00	-2.69E-02	1.00E+00	5.49E-01	1.10E-01	9.04E-01	3.58E-03	3.63E-01	7.90E-01
ENSBTAG00000024091	6.99E-01	8.26E-01	-5.31E-01	7.67E-01	-3.09E-01	9.81E-01	2.32E-01	1.00E+00	5.21E-01	9.92E-01
ENSBTAG00000034586	-6.79E-01	9.97E-01	-2.04E+00	8.20E-02	-1.41E+00	1.96E-01	-1.43E+00	1.98E-01	-1.24E+00	6.79E-01
ENSBTAG00000034657	1.30E-02	1.00E+00	-2.71E+00	2.42E-01	7.22E-01	8.24E-01	1.24E+00	4.55E-01	5.19E-01	1.00E+00
ENSBTAG00000008241	4.46E-02	1.00E+00	2.81E-03	1.00E+00	5.70E-01	4.96E-02	6.10E-01	3.68E-02	-9.90E-03	1.00E+00
ENSBTAG00000001947	2.12E-01	1.00E+00	-7.32E-01	1.50E-01	-1.83E+00	8.38E-05	-2.30E+00	2.51E-06	8.98E-01	1.94E-01
ENSBTAG00000001949	-1.30E+00	2.67E-03	-1.85E+00	5.89E-07	-1.21E+00	8.14E-04	-5.17E-01	2.33E-01	-3.76E-01	8.00E-01
ENSBTAG00000047503	-1.91E+00	8.23E-01	6.92E-02	1.00E+00	-1.31E-01	1.00E+00	1.12E+00	6.70E-01	-1.91E+00	8.00E-01
ENSBTAG00000001952	1.14E+00	8.23E-01	1.23E+00	3.79E-01	-1.43E+00	4.79E-01	-1.74E-01	1.00E+00	1.13E+00	8.00E-01
ENSBTAG00000017955	-3.82E-01	1.00E+00	8.17E-01	3.61E-01	5.92E-02	1.00E+00	3.73E-02	1.00E+00	-5.39E-01	1.00E+00
ENSBTAG00000011553	-1.98E-01	8.64E-01	-7.15E-01	3.53E-04	-1.57E+00	2.99E-16	-2.14E+00	5.15E-28	-4.51E-01	2.29E-01
ENSBTAG00000011558	5.14E-01	1.00E+00	-1.25E+00	1.63E-01	-4.56E+00	1.46E-05	-2.19E+00	1.06E-02	1.38E-01	1.00E+00
ENSBTAG00000009510	-1.45E+00	7.10E-03	-4.22E+00	3.05E-15	-1.43E+00	1.01E-03	-3.50E-01	6.12E-01	-3.55E-02	1.00E+00
ENSBTAG00000020348	-6.53E-01	1.00E+00	-5.69E-01	8.98E-01	-1.33E+00	4.17E-01	-1.82E-01	1.00E+00	8.23E-01	8.90E-01
ENSBTAG00000020350	2.27E+00	1.55E-03	7.16E-01	3.83E-01	2.43E-01	9.93E-01	1.20E+00	7.90E-02	6.05E-01	8.29E-01
ENSBTAG00000018372	5.39E-01	9.62E-02	1.21E+00	8.30E-09	6.69E-01	2.46E-03	8.12E-01	2.08E-04	2.35E-01	8.00E-01
ENSBTAG00000015667	3.85E-01	7.69E-01	1.04E+00	1.06E-03	1.58E+00	2.22E-07	1.77E+00	8.38E-09	8.13E-01	1.18E-01
ENSBTAG00000015659	-4.77E-02	1.00E+00	-6.00E-01	9.67E-03	-4.28E-01	8.63E-02	-1.70E-01	6.75E-01	5.53E-02	1.00E+00
ENSBTAG00000000848	7.95E-02	1.00E+00	3.59E-01	1.57E-01	5.65E-01	1.23E-02	6.48E-01	3.88E-03	2.29E-01	8.00E-01
ENSBTAG00000047659	2.99E-01	9.05E-01	-3.11E-01	5.33E-01	-9.39E-01	7.37E-03	-3.51E-01	4.57E-01	2.41E-01	9.98E-01

ENSBTAG00000034995	1.86E-01	1.00E+00	1.44E+00	6.33E-06	5.69E-01	1.36E-01	1.92E-01	8.48E-01	-2.16E-01	1.00E+00
ENSBTAG00000012925	-2.74E-01	1.00E+00	-8.44E-02	1.00E+00	-2.34E-01	8.93E-01	-1.24E-01	1.00E+00	-6.04E-03	1.00E+00
ENSBTAG00000002965	3.04E+00	2.18E-02	3.54E+00	6.45E-04	4.36E-01	9.45E-01	-7.25E-01	6.84E-01	-2.09E-01	1.00E+00
ENSBTAG00000014090	2.45E+00	7.22E-06	4.17E+00	8.28E-16	3.44E+00	8.92E-12	2.14E+00	1.91E-05	-2.60E-01	1.00E+00
ENSBTAG00000004497	-1.19E-01	1.00E+00	-3.23E-01	3.02E-01	-3.61E-01	2.26E-01	-3.16E-01	3.28E-01	3.89E-01	5.52E-01
ENSBTAG00000042133	0.00E+00	1.00E+00	0.00E+00	1.00E+00	-5.13E-15	1.00E+00	0.00E+00	1.00E+00	0.00E+00	1.00E+00
ENSBTAG00000003963	-1.59E-01	1.00E+00	2.54E-01	6.05E-01	-1.06E+00	7.85E-04	-1.57E+00	6.81E-07	5.56E-01	4.15E-01
ENSBTAG00000003975	-1.42E-01	1.00E+00	-1.22E+00	4.88E-06	-1.39E+00	1.50E-07	-9.55E-01	5.07E-04	1.34E-01	1.00E+00
ENSBTAG00000019213	8.55E-01	1.54E-01	1.82E+00	6.13E-07	1.02E+00	8.82E-03	9.29E-01	2.12E-02	5.99E-01	5.65E-01
ENSBTAG00000010846	-3.50E-01	6.47E-01	-1.72E+00	5.61E-12	-1.81E+00	2.85E-13	-1.37E+00	4.87E-08	3.90E-01	5.91E-01
ENSBTAG00000010849	-3.05E-02	1.00E+00	-2.72E-01	5.79E-01	-8.03E-01	1.43E-02	-1.70E+00	1.27E-07	1.28E-01	1.00E+00
ENSBTAG00000035244	-6.50E-01	1.08E-01	-1.25E+00	2.31E-06	-7.66E-01	5.13E-03	-8.16E-01	3.14E-03	-3.02E-01	8.00E-01
ENSBTAG00000021920	4.75E-01	5.13E-01	-1.31E+00	1.04E-05	-1.17E+00	7.88E-05	-1.18E+00	8.88E-05	-7.54E-02	1.00E+00
ENSBTAG00000006019	-6.19E-02	1.00E+00	-1.59E+00	4.04E-03	-1.23E+00	1.97E-02	-8.94E-01	1.10E-01	-3.44E-02	1.00E+00
ENSBTAG00000018542	5.64E-02	1.00E+00	-4.66E-02	1.00E+00	-2.50E-01	4.50E-01	-2.57E-02	1.00E+00	-1.73E-02	1.00E+00
ENSBTAG00000048041	-2.50E-01	8.23E-01	-1.79E+00	1.23E-15	-7.54E-01	7.99E-04	-3.08E-01	2.80E-01	3.82E-02	1.00E+00
ENSBTAG00000005892	2.20E-01	1.00E+00	-2.28E-02	1.00E+00	-6.88E-01	4.31E-02	-8.48E-01	1.10E-02	-2.23E-01	1.00E+00
ENSBTAG00000006744	1.38E-01	1.00E+00	1.36E+00	4.91E-10	1.28E+00	4.26E-09	8.01E-01	4.79E-04	3.67E-01	5.38E-01
ENSBTAG00000034384	-3.07E-01	1.00E+00	-1.63E+00	5.63E-05	-2.68E+00	4.11E-11	-3.03E+00	3.05E-13	-2.43E-01	1.00E+00
ENSBTAG00000009834	1.06E+00	3.46E-01	-7.07E-01	4.95E-01	-3.41E-01	8.88E-01	-7.24E-01	4.69E-01	1.47E+00	9.53E-02
ENSBTAG00000004814	8.84E-02	1.00E+00	-2.58E-01	5.27E-01	-6.78E-01	1.89E-02	-6.55E-01	2.75E-02	2.02E-02	1.00E+00
ENSBTAG00000014873	-1.35E-01	1.00E+00	-4.17E-01	1.18E-01	-3.27E-01	2.56E-01	-4.61E-01	8.12E-02	-3.33E-01	6.24E-01
ENSBTAG00000014877	8.70E-02	1.00E+00	3.63E-01	1.11E-01	6.20E-01	2.05E-03	3.82E-01	9.41E-02	2.61E-01	7.15E-01
ENSBTAG00000010388	5.95E-02	1.00E+00	-2.01E-01	9.68E-01	-3.58E-01	6.58E-01	1.72E-01	1.00E+00	2.56E-01	1.00E+00
ENSBTAG00000045451	0.00E+00	1.00E+00	0.00E+00	1.00E+00	-5.13E-15	1.00E+00	0.00E+00	1.00E+00	0.00E+00	1.00E+00
ENSBTAG00000018991	7.09E-01	1.80E-01	3.75E-01	4.12E-01	1.18E+00	1.48E-04	7.75E-01	2.46E-02	2.64E-01	9.63E-01
ENSBTAG00000048207	-2.71E+00	5.76E-01	-2.71E+00	2.42E-01	-2.71E+00	1.99E-01	-2.71E+00	2.05E-01	-2.71E+00	6.01E-01
ENSBTAG00000003965	-4.68E-02	1.00E+00	9.07E-02	1.00E+00	1.39E+00	4.60E-07	8.63E-01	3.53E-03	-3.91E-01	7.07E-01
ENSBTAG00000003970	-1.39E-01	1.00E+00	5.59E-01	1.62E-02	3.03E-01	2.76E-01	-8.49E-02	1.00E+00	-5.05E-01	2.43E-01
ENSBTAG00000038949	-5.09E-02	1.00E+00	7.42E-02	1.00E+00	6.41E-03	1.00E+00	-1.92E-01	7.26E-01	-3.66E-01	6.84E-01
ENSBTAG00000002582	2.04E+00	2.86E-01	2.89E+00	5.26E-03	1.86E+00	1.21E-01	7.18E-01	7.92E-01	1.17E+00	8.00E-01
ENSBTAG00000004544	-5.24E-02	1.00E+00	4.24E-01	7.30E-02	-4.26E-02	1.00E+00	-1.78E-01	6.24E-01	-4.07E-01	3.95E-01
ENSBTAG00000034255	1.15E-01	1.00E+00	1.22E+00	2.09E-10	1.72E+00	2.35E-19	1.16E+00	2.36E-09	-3.22E-01	5.53E-01
ENSBTAG00000043999	-1.53E-01	1.00E+00	9.45E-02	9.15E-01	4.42E-02	1.00E+00	-4.52E-01	2.96E-02	-1.21E-01	1.00E+00
ENSBTAG00000012449	7.20E-02	1.00E+00	-1.99E-01	1.00E+00	-5.59E-01	5.32E-01	9.58E-02	1.00E+00	-2.00E-01	1.00E+00

ENSBTAG00000004327	1.69E-02	1.00E+00	1.12E+00	4.86E-02	6.55E-01	3.53E-01	1.08E+00	5.98E-02	3.24E-01	1.00E+00
ENSBTAG00000004328	4.94E-01	6.28E-01	9.45E-01	8.68E-03	1.37E+00	3.94E-05	1.52E+00	5.38E-06	5.55E-01	5.66E-01
ENSBTAG00000034184	-7.92E-01	1.00E+00	-7.29E-01	9.45E-01	-2.71E+00	1.99E-01	-2.71E+00	2.05E-01	-2.71E+00	6.01E-01
ENSBTAG00000009962	-1.98E-04	1.00E+00	6.41E-01	3.67E-03	9.35E-01	8.46E-06	8.53E-01	7.25E-05	-2.41E-01	8.00E-01
ENSBTAG00000046971	-2.36E-01	1.00E+00	-8.54E-01	1.10E-01	-5.39E-01	3.55E-01	2.47E-01	8.21E-01	-7.64E-01	5.16E-01
ENSBTAG00000042179	0.00E+00	1.00E+00	0.00E+00	1.00E+00	-5.13E-15	1.00E+00	0.00E+00	1.00E+00	0.00E+00	1.00E+00
ENSBTAG00000016794	5.16E-01	1.00E+00	1.28E+00	4.43E-01	-1.53E-01	1.00E+00	-9.41E-01	8.06E-01	1.20E+00	8.22E-01
ENSBTAG00000019697	-1.69E-01	1.00E+00	9.23E-01	6.71E-02	1.75E+00	1.28E-04	1.21E+00	1.28E-02	3.70E-01	9.54E-01
ENSBTAG00000019701	-1.42E-01	1.00E+00	-1.30E-01	9.45E-01	-2.89E-01	4.79E-01	-2.64E-01	5.45E-01	-2.16E-01	9.89E-01
ENSBTAG00000025898	6.21E-01	1.26E-02	1.90E-01	5.16E-01	-5.14E-01	1.41E-02	-7.28E-01	3.27E-04	2.11E-01	8.00E-01
ENSBTAG00000008267	1.18E-01	1.00E+00	1.02E+00	3.17E-07	1.01E+00	4.33E-07	6.10E-01	4.62E-03	4.31E-02	1.00E+00
ENSBTAG00000043158	-2.73E-01	1.00E+00	-2.01E-01	1.00E+00	1.61E+00	6.27E-02	2.71E-01	1.00E+00	-6.59E-01	1.00E+00
ENSBTAG00000008269	3.68E-01	6.70E-01	1.29E+00	1.02E-06	1.18E+00	8.00E-06	5.71E-01	5.97E-02	-3.79E-02	1.00E+00
ENSBTAG00000021161	-2.41E+00	3.10E-01	-1.54E+00	2.72E-01	-4.33E+00	5.77E-03	-6.40E-01	7.47E-01	-1.63E+00	6.07E-01
ENSBTAG00000033998	-2.09E-01	1.00E+00	-3.75E-01	9.99E-01	-3.84E-01	9.74E-01	5.35E-01	7.53E-01	-1.64E+00	6.01E-01
ENSBTAG00000013023	4.99E-01	3.79E-01	1.59E+00	4.07E-09	1.03E+00	1.92E-04	1.24E+00	6.81E-06	1.22E-01	1.00E+00
ENSBTAG00000006343	1.77E-02	1.00E+00	1.62E+00	8.20E-03	2.21E+00	2.05E-04	2.42E+00	5.78E-05	4.82E-01	9.40E-01
ENSBTAG00000043317	2.87E+00	1.77E-02	3.68E+00	1.01E-05	2.93E+00	1.53E-03	1.95E+00	1.07E-01	1.24E-03	1.00E+00
ENSBTAG00000005273	1.82E+00	4.26E-10	3.28E+00	8.33E-31	3.26E+00	9.16E-31	2.63E+00	3.99E-21	6.96E-01	1.53E-01
ENSBTAG00000033772	0.00E+00	1.00E+00	0.00E+00	1.00E+00	-5.13E-15	1.00E+00	0.00E+00	1.00E+00	0.00E+00	1.00E+00
ENSBTAG00000046579	-1.91E+00	8.23E-01	-1.91E+00	4.69E-01	-1.91E+00	4.28E-01	-1.91E+00	4.39E-01	-1.91E+00	8.00E-01
ENSBTAG00000005704	1.32E+00	9.30E-01	1.77E+00	3.26E-01	6.40E-01	9.79E-01	1.12E+00	6.71E-01	-1.91E+00	8.00E-01
ENSBTAG00000018571	5.89E-01	1.00E+00	2.42E+00	9.14E-03	1.84E+00	6.43E-02	2.22E+00	2.12E-02	-4.24E-01	1.00E+00
ENSBTAG00000001034	3.07E-01	1.00E+00	1.36E+00	9.33E-03	6.96E-01	2.85E-01	5.44E-01	4.74E-01	-3.96E-01	1.00E+00
ENSBTAG00000033748	1.04E+00	2.68E-02	6.55E-01	1.15E-01	7.21E-01	6.88E-02	-6.95E-02	1.00E+00	-3.99E-01	8.17E-01
ENSBTAG00000024075	-7.87E-01	1.00E+00	5.97E-01	9.45E-01	-1.53E-01	1.00E+00	-1.76E-01	1.00E+00	-7.91E-01	1.00E+00
ENSBTAG00000042997	0.00E+00	1.00E+00	0.00E+00	1.00E+00	-5.13E-15	1.00E+00	0.00E+00	1.00E+00	0.00E+00	1.00E+00
ENSBTAG00000001706	-1.45E+00	6.14E-01	-2.36E-01	1.00E+00	-2.12E+00	8.02E-02	5.96E-01	6.68E-01	-1.06E+00	8.00E-01
ENSBTAG00000001714	-3.85E-01	6.37E-01	-8.07E-01	5.37E-03	5.77E-01	4.88E-02	5.27E-01	8.68E-02	2.73E-01	8.29E-01
ENSBTAG00000038462	2.49E-01	1.00E+00	8.27E-01	4.46E-01	4.13E-02	1.00E+00	7.30E-01	5.20E-01	7.07E-01	8.97E-01
ENSBTAG00000042285	0.00E+00	1.00E+00	0.00E+00	1.00E+00	-5.13E-15	1.00E+00	0.00E+00	1.00E+00	0.00E+00	1.00E+00
ENSBTAG00000006150	2.15E-01	1.00E+00	-3.32E-01	4.43E-01	-2.08E-02	1.00E+00	3.35E-01	4.49E-01	9.81E-02	1.00E+00
ENSBTAG00000048142	-9.21E-04	1.00E+00	2.80E+00	4.39E-03	1.82E+00	1.41E-01	3.21E-01	1.00E+00	1.82E+00	5.01E-01
ENSBTAG00000021731	-1.93E-01	9.82E-01	4.05E-01	1.33E-01	9.78E-01	1.79E-05	5.84E-01	1.97E-02	1.79E-01	9.90E-01
ENSBTAG00000012332	1.75E-01	1.00E+00	1.01E+00	4.95E-06	8.03E-01	3.85E-04	3.62E-01	1.93E-01	2.39E-01	8.02E-01

ENSBTAG00000001086	6.00E-01	5.76E-01	1.70E+00	2.05E-05	9.00E-01	3.84E-02	6.62E-01	1.73E-01	-2.75E-01	1.00E+00
ENSBTAG00000015575	1.63E+00	2.02E-01	1.55E+00	6.84E-02	1.00E+00	3.40E-01	1.39E+00	1.15E-01	1.27E+00	5.47E-01
ENSBTAG00000017189	-2.02E-02	1.00E+00	-1.73E-01	6.03E-01	-2.67E-01	3.03E-01	3.26E-02	1.00E+00	-3.66E-01	4.39E-01
ENSBTAG00000043253	0.00E+00	1.00E+00	0.00E+00	1.00E+00	-5.13E-15	1.00E+00	0.00E+00	1.00E+00	0.00E+00	1.00E+00
ENSBTAG00000013108	1.51E+00	4.78E-01	2.19E+00	2.39E-02	2.81E+00	2.31E-03	4.58E+00	6.06E-07	1.82E+00	3.52E-01
ENSBTAG00000044983	0.00E+00	1.00E+00	0.00E+00	1.00E+00	-5.13E-15	1.00E+00	0.00E+00	1.00E+00	0.00E+00	1.00E+00
ENSBTAG00000006989	1.74E-01	1.00E+00	-1.77E+00	4.94E-12	-1.41E+00	3.32E-08	-5.18E-01	8.27E-02	3.80E-01	6.09E-01
ENSBTAG00000006988	-7.81E-02	1.00E+00	-9.37E-01	1.18E-01	-1.33E+00	1.67E-02	-2.14E+00	7.87E-05	6.38E-02	1.00E+00
ENSBTAG00000018579	-6.69E-01	7.22E-02	-1.47E+00	8.84E-09	-8.76E-01	7.15E-04	-3.41E-01	3.06E-01	-1.10E-02	1.00E+00
ENSBTAG00000018578	3.26E-01	8.23E-01	-1.87E-01	8.19E-01	-5.84E-01	9.75E-02	-8.47E-01	1.16E-02	5.82E-01	3.59E-01
ENSBTAG00000020124	-6.75E-03	1.00E+00	1.89E-01	5.44E-01	6.05E-01	3.65E-03	6.69E-01	1.34E-03	-5.08E-02	1.00E+00
ENSBTAG00000020123	-1.78E-01	9.87E-01	2.75E-01	3.36E-01	3.83E-01	1.27E-01	4.14E-01	9.88E-02	-3.34E-01	6.00E-01
ENSBTAG00000016609	1.00E+00	2.05E-01	3.97E+00	3.44E-18	2.61E+00	5.17E-09	1.22E+00	1.31E-02	4.93E-01	8.00E-01
ENSBTAG00000016607	-1.67E+00	2.05E-01	-3.22E+00	9.71E-04	-2.08E+00	1.21E-02	-2.96E+00	1.15E-03	-5.47E-01	9.66E-01
ENSBTAG00000016606	-4.27E-01	4.95E-01	5.87E-01	3.11E-02	9.39E-01	1.56E-04	5.96E-01	3.01E-02	1.79E-01	1.00E+00
ENSBTAG00000016602	-3.81E-01	1.00E+00	-2.94E-01	1.00E+00	3.62E-01	1.00E+00	1.00E-01	1.00E+00	3.05E-01	1.00E+00
ENSBTAG00000016600	2.69E-01	9.02E-01	1.21E+00	9.97E-06	1.14E+00	2.98E-05	6.04E-01	5.45E-02	1.43E-01	1.00E+00
ENSBTAG00000016599	1.27E-02	1.00E+00	5.77E-01	1.16E-02	6.74E-01	2.40E-03	6.05E-01	8.71E-03	-8.52E-02	1.00E+00
ENSBTAG00000001844	7.94E-01	7.26E-02	2.48E+00	1.81E-16	2.69E+00	5.37E-19	2.54E+00	4.09E-17	5.76E-01	4.09E-01
ENSBTAG00000001843	-3.66E-01	3.75E-01	-9.02E-01	6.03E-06	-1.94E+00	6.88E-23	-2.23E+00	3.69E-29	-1.78E-01	9.06E-01
ENSBTAG00000001840	-1.24E-01	1.00E+00	-1.02E+00	3.34E-05	-5.69E-01	3.02E-02	-2.71E-01	4.39E-01	-5.86E-02	1.00E+00
ENSBTAG00000000490	-3.78E-01	8.06E-01	-2.50E+00	2.12E-13	-1.71E+00	1.41E-07	-5.63E-01	1.40E-01	4.58E-02	1.00E+00
ENSBTAG00000000489	6.61E-02	1.00E+00	-7.90E-01	6.89E-02	-1.27E+00	1.60E-03	-1.15E+00	5.14E-03	-1.04E-02	1.00E+00
ENSBTAG00000040226	-6.08E-01	7.72E-01	-3.95E+00	3.92E-07	-4.96E+00	2.56E-09	-2.79E+00	1.12E-05	3.40E-01	9.98E-01
ENSBTAG00000037856	-1.06E-01	1.00E+00	-5.35E-01	1.78E-02	-1.09E+00	1.50E-07	-9.14E-01	1.72E-05	-2.44E-03	1.00E+00
ENSBTAG00000004887	-9.85E-02	1.00E+00	-8.40E-01	4.58E-05	-4.52E-01	4.53E-02	-1.31E-01	7.99E-01	1.00E-01	1.00E+00
ENSBTAG00000004884	8.27E-01	5.97E-01	3.85E+00	6.24E-14	1.57E+00	5.41E-03	1.20E+00	5.23E-02	5.10E-01	9.14E-01
ENSBTAG00000010072	0.00E+00	1.00E+00	0.00E+00	1.00E+00	-5.13E-15	1.00E+00	0.00E+00	1.00E+00	0.00E+00	1.00E+00
ENSBTAG00000004881	9.06E-01	7.39E-02	3.13E+00	9.14E-22	2.18E+00	2.19E-11	1.89E+00	1.17E-08	5.96E-01	5.03E-01
ENSBTAG00000006259	8.11E-02	1.00E+00	5.12E-01	9.20E-02	1.04E+00	1.00E-04	1.30E+00	9.34E-07	2.67E-02	1.00E+00
ENSBTAG00000015193	1.11E-01	1.00E+00	2.85E-02	1.00E+00	-3.60E-01	5.48E-01	-4.99E-01	3.45E-01	-8.73E-02	1.00E+00
ENSBTAG00000022381	-3.03E-02	1.00E+00	-9.74E-01	3.67E-03	-9.18E-01	5.82E-03	-9.36E-02	1.00E+00	8.09E-01	1.19E-01
ENSBTAG00000012037	-7.83E-04	1.00E+00	4.74E-01	1.70E-02	4.23E-01	3.74E-02	1.86E-02	1.00E+00	-2.19E-01	8.00E-01
ENSBTAG00000015441	2.35E-01	1.00E+00	1.12E+00	4.05E-02	-1.26E+00	3.51E-02	-5.34E-01	4.97E-01	7.50E-01	6.03E-01
ENSBTAG00000009600	-5.55E-03	1.00E+00	5.00E-01	1.80E-02	8.19E-01	2.82E-05	3.73E-01	1.07E-01	-1.31E-01	1.00E+00

ENSBTAG00000039878	-3.69E-01	1.00E+00	-3.59E+00	6.69E-02	-3.59E+00	4.55E-02	-1.83E+00	2.99E-01	7.49E-01	9.87E-01
ENSBTAG00000033255	-6.63E-02	1.00E+00	8.03E-01	9.69E-05	4.00E-01	8.72E-02	1.09E-01	8.94E-01	-4.67E-01	2.48E-01
ENSBTAG00000043164	-1.91E+00	8.23E-01	7.78E-02	1.00E+00	-1.91E+00	4.28E-01	6.21E-01	9.94E-01	1.31E+00	8.99E-01
ENSBTAG00000044622	-1.18E+00	8.62E-01	3.40E-01	1.00E+00	4.63E-01	8.95E-01	-2.13E+00	1.75E-01	-1.17E+00	8.35E-01
ENSBTAG00000011176	7.28E-02	1.00E+00	-7.10E-02	1.00E+00	2.17E-01	5.75E-01	3.40E-01	2.80E-01	-1.04E-01	1.00E+00
ENSBTAG00000047969	8.99E-01	1.00E+00	1.72E+00	2.03E-01	-1.56E-01	1.00E+00	-9.47E-01	8.05E-01	4.98E-01	1.00E+00
ENSBTAG00000011172	1.91E-01	9.90E-01	-1.14E+00	1.28E-06	-3.37E-01	2.50E-01	-2.58E-01	4.46E-01	-1.69E-01	1.00E+00
ENSBTAG00000024058	2.72E+00	5.76E-01	1.99E+00	4.43E-01	-5.13E-15	1.00E+00	3.03E+00	1.50E-01	0.00E+00	1.00E+00
ENSBTAG00000012759	-9.18E-01	9.53E-01	-8.27E-01	6.64E-01	-2.35E+00	1.01E-01	-7.32E-01	7.06E-01	-9.03E-01	9.27E-01
ENSBTAG00000012756	1.15E-01	1.00E+00	1.36E+00	3.35E-12	1.32E+00	1.19E-11	9.98E-01	6.04E-07	-2.13E-01	8.02E-01
ENSBTAG00000008765	-1.16E-01	1.00E+00	-1.82E-01	6.30E-01	-6.38E-01	5.51E-03	-7.65E-01	7.99E-04	7.45E-02	1.00E+00
ENSBTAG00000008764	-1.20E-01	1.00E+00	1.60E+00	9.83E-13	1.69E+00	2.73E-14	1.32E+00	5.41E-09	-3.26E-01	6.85E-01
ENSBTAG00000024061	-1.91E+00	8.23E-01	1.39E+00	5.17E-01	6.50E-01	9.78E-01	1.13E+00	6.66E-01	7.99E-01	1.00E+00
ENSBTAG00000008035	-1.24E-01	1.00E+00	-8.46E-02	1.00E+00	1.48E-01	8.85E-01	1.59E-01	8.57E-01	1.20E-01	1.00E+00
ENSBTAG00000043946	0.00E+00	1.00E+00	0.00E+00	1.00E+00	-5.13E-15	1.00E+00	0.00E+00	1.00E+00	0.00E+00	1.00E+00
ENSBTAG00000006162	-5.55E-01	3.84E-01	-1.75E+00	6.24E-08	4.98E-01	1.60E-01	1.38E+00	2.79E-06	-6.55E-02	1.00E+00
ENSBTAG00000044590	0.00E+00	1.00E+00	0.00E+00	1.00E+00	-5.13E-15	1.00E+00	0.00E+00	1.00E+00	0.00E+00	1.00E+00
ENSBTAG00000018555	-4.01E-02	1.00E+00	-8.26E-01	2.10E-03	-1.39E+00	6.72E-08	-1.62E+00	4.41E-10	-2.32E-01	9.04E-01
ENSBTAG00000001866	-8.63E-01	3.41E-01	-2.27E+00	2.49E-05	-2.93E+00	9.49E-08	-2.56E+00	1.50E-06	6.21E-01	6.01E-01
ENSBTAG00000021961	-3.41E-02	1.00E+00	-1.74E-01	6.62E-01	-5.79E-01	1.35E-02	-4.92E-01	4.73E-02	-3.07E-01	6.65E-01
ENSBTAG00000020799	5.64E-02	1.00E+00	4.00E-02	1.00E+00	2.33E-01	4.41E-01	2.50E-01	4.16E-01	-3.76E-01	4.86E-01
ENSBTAG00000043833	0.00E+00	1.00E+00	0.00E+00	1.00E+00	-5.13E-15	1.00E+00	0.00E+00	1.00E+00	0.00E+00	1.00E+00
ENSBTAG00000046679	0.00E+00	1.00E+00	0.00E+00	1.00E+00	-5.13E-15	1.00E+00	0.00E+00	1.00E+00	0.00E+00	1.00E+00
ENSBTAG00000039263	0.00E+00	1.00E+00	0.00E+00	1.00E+00	-5.13E-15	1.00E+00	0.00E+00	1.00E+00	0.00E+00	1.00E+00
ENSBTAG00000012212	8.46E-01	3.81E-01	-2.66E-01	8.75E-01	5.78E-01	3.58E-01	-3.68E-01	6.88E-01	-3.52E-01	1.00E+00
ENSBTAG00000043374	0.00E+00	1.00E+00	0.00E+00	1.00E+00	-5.13E-15	1.00E+00	0.00E+00	1.00E+00	1.91E+00	8.00E-01
ENSBTAG00000013290	5.18E-02	1.00E+00	-5.57E-01	2.02E-02	-6.05E-01	9.98E-03	-6.81E-01	3.79E-03	-9.21E-02	1.00E+00
ENSBTAG00000009242	4.32E-02	1.00E+00	1.08E-01	8.10E-01	1.99E-01	4.17E-01	2.02E-01	4.23E-01	-1.67E-01	8.65E-01
ENSBTAG00000014667	-3.28E-01	1.00E+00	-1.05E+00	2.82E-02	-3.33E-01	6.54E-01	1.90E+00	2.68E-06	8.02E-01	3.78E-01
ENSBTAG00000014668	-6.95E-01	5.81E-03	-6.26E-01	2.96E-03	-6.52E-02	1.00E+00	-6.67E-02	1.00E+00	1.43E-01	1.00E+00
ENSBTAG00000002105	-4.34E-01	3.62E-01	-7.31E-01	2.38E-03	-9.48E-01	4.89E-05	-8.93E-01	1.78E-04	-3.49E-01	6.01E-01
ENSBTAG00000010623	-1.06E+00	3.39E-01	-5.66E-01	4.58E-01	-1.40E+00	2.13E-02	-2.22E+00	5.78E-04	2.93E-02	1.00E+00
ENSBTAG00000010620	-2.60E-01	1.00E+00	-1.03E+00	1.73E-01	-1.37E+00	5.09E-02	-1.77E+00	1.03E-02	-9.15E-02	1.00E+00
ENSBTAG00000010605	1.04E-02	1.00E+00	-1.23E+00	6.13E-01	-6.55E-01	8.97E-01	1.27E+00	3.34E-01	-1.30E+00	9.01E-01
ENSBTAG00000004562	1.91E+00	8.23E-01	4.63E+00	4.30E-03	4.63E+00	3.27E-03	0.00E+00	1.00E+00	3.21E+00	4.36E-01

ENSBTAG00000004560	-7.50E-01	9.87E-01	-1.46E+00	1.98E-01	-1.99E+00	5.71E-02	-3.11E+00	2.91E-03	-7.65E-01	9.39E-01
ENSBTAG00000008905	1.92E+00	8.23E-01	1.99E+00	4.43E-01	1.78E+00	4.79E-01	0.00E+00	1.00E+00	0.00E+00	1.00E+00
ENSBTAG00000009076	-8.56E-01	6.68E-01	-6.67E-01	4.44E-01	-7.58E-02	1.00E+00	-1.19E+00	1.08E-01	-7.55E-01	7.78E-01
ENSBTAG00000000783	-4.30E-01	1.00E+00	5.98E-01	7.41E-01	1.01E+00	3.38E-01	1.72E+00	3.61E-02	-1.09E-02	1.00E+00
ENSBTAG00000008674	-8.10E-02	1.00E+00	3.18E-01	2.99E-01	8.07E-01	7.73E-04	7.81E-01	1.45E-03	-6.26E-01	1.17E-01
ENSBTAG00000012519	8.11E-01	6.50E-02	9.55E-01	2.38E-03	2.22E-01	7.05E-01	-1.13E-01	1.00E+00	2.69E-01	9.11E-01
ENSBTAG00000003108	-8.69E-01	1.00E+00	-2.84E-01	1.00E+00	-1.65E-01	1.00E+00	-1.94E-01	1.00E+00	-1.67E+00	7.79E-01
ENSBTAG00000007734	-6.31E-02	1.00E+00	3.33E-02	1.00E+00	3.48E-01	2.89E-01	5.40E-01	6.26E-02	-2.81E-01	8.00E-01
ENSBTAG00000033887	0.00E+00	1.00E+00	0.00E+00	1.00E+00	-5.13E-15	1.00E+00	0.00E+00	1.00E+00	0.00E+00	1.00E+00
ENSBTAG00000009246	-1.19E-01	1.00E+00	-5.36E-01	5.08E-02	-6.14E-01	2.08E-02	-5.11E-01	7.09E-02	-3.88E-01	5.88E-01
ENSBTAG00000021694	6.62E-03	1.00E+00	-6.26E-01	3.88E-03	-5.98E-01	5.73E-03	-3.59E-01	1.50E-01	-1.70E-01	9.56E-01
ENSBTAG00000010898	4.93E-03	1.00E+00	6.16E-02	1.00E+00	5.47E-01	5.34E-02	6.41E-01	2.15E-02	6.82E-03	1.00E+00
ENSBTAG00000008223	5.99E-01	2.17E-01	2.01E+00	3.40E-14	1.42E+00	1.08E-07	2.15E-01	6.87E-01	1.79E-02	1.00E+00
ENSBTAG00000033041	2.53E-01	7.89E-01	3.30E-01	1.98E-01	1.29E-01	8.08E-01	-2.40E-01	4.37E-01	-3.72E-04	1.00E+00
ENSBTAG00000027932	2.96E-01	6.99E-01	-1.18E-01	8.90E-01	-3.09E-01	2.75E-01	-6.95E-02	1.00E+00	1.98E-01	9.06E-01
ENSBTAG00000033010	2.34E-01	8.23E-01	1.39E+00	6.93E-13	1.70E+00	9.02E-19	1.39E+00	7.18E-13	-9.22E-02	1.00E+00
ENSBTAG00000037357	0.00E+00	1.00E+00	0.00E+00	1.00E+00	-5.13E-15	1.00E+00	0.00E+00	1.00E+00	0.00E+00	1.00E+00
ENSBTAG00000019839	2.86E-02	1.00E+00	4.53E-01	1.08E-01	3.62E-01	2.34E-01	5.23E-01	5.92E-02	-6.73E-02	1.00E+00
ENSBTAG00000045226	0.00E+00	1.00E+00	0.00E+00	1.00E+00	-5.13E-15	1.00E+00	0.00E+00	1.00E+00	0.00E+00	1.00E+00
ENSBTAG00000008006	-4.14E-01	7.45E-01	-1.08E+00	1.57E-03	-5.82E-01	1.22E-01	-4.83E-01	2.36E-01	-2.31E-02	1.00E+00
ENSBTAG00000045057	0.00E+00	1.00E+00	0.00E+00	1.00E+00	-5.13E-15	1.00E+00	0.00E+00	1.00E+00	0.00E+00	1.00E+00
ENSBTAG00000008548	3.58E-02	1.00E+00	1.08E+00	4.62E-07	1.22E+00	8.75E-09	1.01E+00	3.04E-06	-5.90E-02	1.00E+00
ENSBTAG00000044993	0.00E+00	1.00E+00	0.00E+00	1.00E+00	-5.13E-15	1.00E+00	0.00E+00	1.00E+00	0.00E+00	1.00E+00
ENSBTAG00000045038	0.00E+00	1.00E+00	0.00E+00	1.00E+00	-5.13E-15	1.00E+00	0.00E+00	1.00E+00	0.00E+00	1.00E+00
ENSBTAG00000009020	1.12E-01	1.00E+00	6.77E-02	1.00E+00	-7.66E-01	2.11E-02	-2.70E-01	5.96E-01	6.27E-01	3.29E-01
ENSBTAG00000009021	-2.03E-01	8.65E-01	-1.37E+00	1.96E-11	-1.14E+00	1.40E-08	-1.49E-01	7.08E-01	-3.11E-01	6.01E-01
ENSBTAG00000024980	-2.31E-01	1.00E+00	-1.70E-02	1.00E+00	-1.48E+00	1.11E-01	-3.07E-01	9.62E-01	-1.19E-01	1.00E+00
ENSBTAG00000018590	7.75E-03	1.00E+00	-2.13E-01	7.26E-01	1.39E-01	9.33E-01	8.69E-01	3.57E-03	2.08E-01	1.00E+00
ENSBTAG00000014310	-1.65E-01	1.00E+00	-1.27E+00	1.49E-06	-6.45E-01	2.27E-02	-2.00E-01	6.87E-01	2.14E-01	9.58E-01
ENSBTAG00000003861	1.18E-01	1.00E+00	-2.47E-01	5.98E-01	2.31E-01	6.22E-01	4.04E-01	2.56E-01	-2.41E-01	9.37E-01
ENSBTAG00000008703	2.67E-01	1.00E+00	1.49E+00	3.13E-05	1.06E+00	4.18E-03	2.77E-01	6.72E-01	-1.20E-01	1.00E+00
ENSBTAG00000008707	3.64E-01	8.35E-01	1.08E+00	2.68E-03	-9.90E-01	9.12E-03	-2.05E+00	4.99E-08	1.03E+00	5.23E-02
ENSBTAG00000039770	2.01E-02	1.00E+00	1.13E+00	3.18E-10	1.37E+00	1.56E-14	1.04E+00	9.00E-09	-2.76E-01	6.05E-01
ENSBTAG00000001113	1.15E-02	1.00E+00	-3.09E-01	3.12E-01	-1.14E+00	1.15E-06	-1.17E+00	7.01E-07	1.77E-01	9.93E-01
ENSBTAG00000001114	8.70E-02	1.00E+00	-1.50E-01	9.31E-01	1.35E-01	9.68E-01	2.43E-01	6.65E-01	2.67E-01	9.20E-01

ENSBTAG00000013923	-7.26E-01	9.36E-01	-1.42E+00	1.93E-01	-3.32E+00	3.93E-03	-3.40E-01	9.54E-01	4.03E-02	1.00E+00
ENSBTAG00000042902	0.00E+00	1.00E+00	0.00E+00	1.00E+00	-5.13E-15	1.00E+00	0.00E+00	1.00E+00	0.00E+00	1.00E+00
ENSBTAG00000023736	-8.26E-01	8.48E-02	-1.03E+00	2.43E-03	-3.45E-02	1.00E+00	3.60E-01	3.96E-01	-5.69E-01	4.48E-01
ENSBTAG00000032905	-3.98E-01	4.91E-01	-1.36E+00	1.15E-08	-2.39E+00	2.45E-23	-2.08E+00	2.98E-18	-7.84E-01	2.46E-02
ENSBTAG00000010531	2.17E+00	6.02E-03	4.96E+00	1.31E-14	3.77E+00	1.90E-09	3.68E+00	6.25E-09	7.57E-01	8.00E-01
ENSBTAG00000016167	-5.61E-02	1.00E+00	-1.50E+00	2.47E-09	-9.91E-01	1.11E-04	-7.00E-01	1.04E-02	1.43E-01	1.00E+00
ENSBTAG00000044256	0.00E+00	1.00E+00	0.00E+00	1.00E+00	-5.13E-15	1.00E+00	1.76E+00	4.97E-01	0.00E+00	1.00E+00
ENSBTAG00000020567	-1.16E-02	1.00E+00	6.28E-01	3.58E-03	3.72E-01	1.26E-01	3.58E-01	1.55E-01	-2.84E-01	7.00E-01
ENSBTAG00000042820	0.00E+00	1.00E+00	0.00E+00	1.00E+00	1.78E+00	4.79E-01	0.00E+00	1.00E+00	0.00E+00	1.00E+00
ENSBTAG00000044545	-1.91E+00	8.23E-01	6.91E-02	1.00E+00	6.39E-01	9.79E-01	-1.91E+00	4.39E-01	-1.91E+00	8.00E-01
ENSBTAG00000026809	1.65E-02	1.00E+00	5.64E-02	1.00E+00	4.54E-01	5.01E-01	6.50E-01	2.77E-01	-5.43E-01	8.00E-01
ENSBTAG00000021102	-3.58E-01	3.49E-01	-9.43E-01	4.85E-07	-2.09E+00	1.64E-29	-2.77E+00	3.82E-48	-5.39E-02	1.00E+00
ENSBTAG00000014891	1.29E-03	1.00E+00	8.58E-01	5.65E-07	1.05E+00	6.11E-10	2.33E-01	3.15E-01	-4.89E-01	7.92E-02
ENSBTAG00000012984	-7.82E-01	5.86E-01	-1.98E+00	2.20E-03	-1.93E+00	1.50E-03	-1.97E+00	1.46E-03	-1.39E+00	1.76E-01
ENSBTAG00000015521	2.57E-02	1.00E+00	6.05E-01	2.89E-02	5.88E-01	3.37E-02	4.45E-01	1.48E-01	-8.26E-02	1.00E+00
ENSBTAG00000014679	1.56E-02	1.00E+00	3.72E-01	2.30E-01	-9.04E-02	1.00E+00	-4.22E-02	1.00E+00	1.52E-01	1.00E+00
ENSBTAG00000024042	6.13E-02	1.00E+00	-6.90E-01	4.61E-02	-3.93E-01	3.26E-01	-9.74E-02	1.00E+00	-5.22E-02	1.00E+00
ENSBTAG00000017039	3.08E-01	1.00E+00	1.43E+00	3.93E-04	5.66E-01	2.93E-01	-1.06E-02	1.00E+00	5.06E-01	8.00E-01
ENSBTAG00000011643	4.94E-01	3.92E-01	1.19E+00	1.45E-05	5.52E-01	7.59E-02	9.62E-01	7.13E-04	1.84E-01	1.00E+00
ENSBTAG00000037586	-1.29E+00	9.36E-01	-1.23E+00	6.11E-01	-3.21E+00	9.44E-02	-3.21E+00	9.91E-02	-3.21E+00	4.34E-01
ENSBTAG00000024044	1.02E+00	9.81E-02	2.10E+00	2.45E-07	6.86E-01	1.55E-01	-8.34E-01	8.12E-02	1.04E-01	1.00E+00
ENSBTAG00000032775	0.00E+00	1.00E+00	0.00E+00	1.00E+00	-5.13E-15	1.00E+00	0.00E+00	1.00E+00	0.00E+00	1.00E+00
ENSBTAG00000016442	3.65E-01	5.66E-01	-2.84E-01	3.81E-01	-6.86E-02	1.00E+00	1.74E-01	7.07E-01	-4.14E-02	1.00E+00
ENSBTAG00000043391	0.00E+00	1.00E+00	0.00E+00	1.00E+00	-5.13E-15	1.00E+00	0.00E+00	1.00E+00	0.00E+00	1.00E+00
ENSBTAG00000017318	-4.64E-01	1.00E+00	1.32E+00	5.27E-02	2.77E+00	4.69E-06	2.68E+00	1.21E-05	1.86E-01	1.00E+00
ENSBTAG00000004722	-4.30E-01	6.75E-01	-1.73E+00	1.07E-07	-9.06E-02	1.00E+00	-6.03E-02	1.00E+00	1.41E-01	1.00E+00
ENSBTAG00000019422	9.36E-02	1.00E+00	3.02E-01	6.09E-01	-5.42E-02	1.00E+00	-1.23E-01	1.00E+00	-1.51E-01	1.00E+00
ENSBTAG00000013861	2.25E-01	1.00E+00	-9.06E-01	7.81E-03	-7.81E-02	1.00E+00	-1.71E-02	1.00E+00	-4.28E-01	6.72E-01
ENSBTAG00000037153	0.00E+00	1.00E+00	0.00E+00	1.00E+00	-5.13E-15	1.00E+00	0.00E+00	1.00E+00	0.00E+00	1.00E+00
ENSBTAG00000026034	-7.79E-01	1.00E+00	-2.70E+00	2.42E-01	-2.70E+00	1.99E-01	-9.39E-01	8.06E-01	-2.70E+00	6.01E-01
ENSBTAG00000043712	0.00E+00	1.00E+00	0.00E+00	1.00E+00	-5.13E-15	1.00E+00	0.00E+00	1.00E+00	0.00E+00	1.00E+00
ENSBTAG00000019382	-1.84E-01	1.00E+00	-8.68E-01	2.01E-02	-7.07E-02	1.00E+00	-1.11E-02	1.00E+00	1.72E-01	1.00E+00
ENSBTAG00000010935	-1.58E-02	1.00E+00	5.90E-02	1.00E+00	5.53E-01	1.68E-02	4.88E-01	4.51E-02	-4.54E-02	1.00E+00
ENSBTAG00000000705	6.11E-02	1.00E+00	9.84E-02	1.00E+00	1.97E-01	6.43E-01	3.95E-01	1.92E-01	-2.66E-01	8.00E-01
ENSBTAG00000032623	4.34E+00	5.10E-02	3.68E+00	5.11E-02	3.96E+00	2.29E-02	4.14E+00	1.59E-02	3.21E+00	4.34E-01

ENSBTAG00000004073	3.78E-01	8.23E-01	-1.09E+00	2.59E-03	-6.36E-01	1.07E-01	3.35E-01	5.06E-01	-8.65E-03	1.00E+00
ENSBTAG00000045766	0.00E+00	1.00E+00	0.00E+00	1.00E+00	-5.13E-15	1.00E+00	0.00E+00	1.00E+00	0.00E+00	1.00E+00
ENSBTAG00000004076	-1.62E-01	1.00E+00	-1.46E+00	1.28E-03	-3.22E+00	1.37E-12	-2.71E+00	2.04E-09	3.76E-01	9.23E-01
ENSBTAG00000004674	-3.78E-01	3.87E-01	-1.23E+00	2.66E-09	-3.35E+00	6.42E-55	-3.18E+00	3.64E-50	-3.91E-02	1.00E+00
ENSBTAG00000014836	0.00E+00	1.00E+00	0.00E+00	1.00E+00	-5.13E-15	1.00E+00	0.00E+00	1.00E+00	0.00E+00	1.00E+00
ENSBTAG00000003553	1.05E-01	1.00E+00	3.93E-01	2.98E-01	2.78E-01	5.23E-01	9.90E-01	1.02E-03	4.08E-01	6.58E-01
ENSBTAG00000003555	1.48E-01	1.00E+00	8.90E-01	2.15E-05	1.11E+00	5.58E-08	6.48E-01	3.37E-03	1.55E-01	1.00E+00
ENSBTAG00000032521	-7.93E-01	2.05E-01	-2.97E+00	1.87E-12	-2.42E+00	5.88E-10	-1.48E+00	9.63E-05	-3.18E-01	9.09E-01
ENSBTAG00000013676	-7.04E-01	1.51E-01	-3.01E+00	1.07E-19	-2.87E+00	4.88E-19	-2.43E+00	1.34E-14	-5.11E-01	5.10E-01
ENSBTAG00000016365	-6.78E-01	3.75E-01	-2.06E+00	3.93E-08	-2.96E+00	6.96E-15	-1.74E+00	3.59E-06	-5.19E-01	6.51E-01
ENSBTAG00000016366	-1.16E+00	7.79E-02	-4.72E+00	1.80E-21	-4.00E+00	5.07E-17	-2.43E+00	1.22E-07	-4.27E-01	8.66E-01
ENSBTAG00000016368	-6.13E-03	1.00E+00	1.08E+00	4.87E-08	8.64E-01	1.48E-05	1.11E+00	1.96E-08	-4.08E-01	3.46E-01
ENSBTAG00000043054	0.00E+00	1.00E+00	0.00E+00	1.00E+00	-5.13E-15	1.00E+00	0.00E+00	1.00E+00	0.00E+00	1.00E+00
ENSBTAG00000044493	0.00E+00	1.00E+00	0.00E+00	1.00E+00	-5.13E-15	1.00E+00	0.00E+00	1.00E+00	0.00E+00	1.00E+00
ENSBTAG00000000223	1.32E-01	1.00E+00	2.24E-01	4.70E-01	-2.21E-01	4.79E-01	-2.27E-01	4.84E-01	8.63E-02	1.00E+00
ENSBTAG00000017421	-1.72E-01	9.76E-01	-8.82E-01	2.08E-05	-6.11E-01	4.09E-03	-8.39E-01	5.80E-05	-5.21E-01	1.44E-01
ENSBTAG00000017429	-2.87E-01	9.28E-01	-1.09E+00	7.30E-04	-1.49E-01	9.31E-01	-3.53E-01	4.39E-01	-2.00E-01	1.00E+00
ENSBTAG00000043226	0.00E+00	1.00E+00	0.00E+00	1.00E+00	-5.13E-15	1.00E+00	0.00E+00	1.00E+00	0.00E+00	1.00E+00
ENSBTAG00000032519	-1.92E-01	1.00E+00	-1.55E+00	3.06E-07	-6.99E-02	1.00E+00	3.47E-01	3.93E-01	-6.15E-01	3.13E-01
ENSBTAG00000027017	1.92E+00	8.23E-01	2.79E+00	2.10E-01	-5.13E-15	1.00E+00	2.53E+00	2.71E-01	0.00E+00	1.00E+00
ENSBTAG00000004159	0.00E+00	1.00E+00	0.00E+00	1.00E+00	1.78E+00	4.79E-01	1.76E+00	4.97E-01	0.00E+00	1.00E+00
ENSBTAG00000042095	0.00E+00	1.00E+00	0.00E+00	1.00E+00	-5.13E-15	1.00E+00	0.00E+00	1.00E+00	0.00E+00	1.00E+00
ENSBTAG00000007508	2.93E-01	6.27E-01	9.03E-01	9.20E-06	5.71E-01	7.95E-03	3.82E-01	1.14E-01	-3.31E-01	5.67E-01
ENSBTAG00000032651	0.00E+00	1.00E+00	0.00E+00	1.00E+00	-5.13E-15	1.00E+00	0.00E+00	1.00E+00	0.00E+00	1.00E+00
ENSBTAG00000020614	4.03E-01	3.75E-01	4.38E-01	8.13E-02	-9.91E-03	1.00E+00	-1.80E-01	6.54E-01	-1.18E-01	1.00E+00
ENSBTAG00000003711	-1.40E-01	1.00E+00	-8.40E-02	1.00E+00	-5.29E-01	2.72E-02	-6.95E-01	2.81E-03	-2.23E-01	8.33E-01
ENSBTAG00000045935	0.00E+00	1.00E+00	0.00E+00	1.00E+00	-5.13E-15	1.00E+00	0.00E+00	1.00E+00	0.00E+00	1.00E+00
ENSBTAG00000004105	1.91E+00	8.23E-01	2.79E+00	2.10E-01	-5.13E-15	1.00E+00	0.00E+00	1.00E+00	0.00E+00	1.00E+00
ENSBTAG00000013734	2.20E-01	1.00E+00	3.07E-01	1.00E+00	7.75E-01	5.21E-01	5.01E-01	8.30E-01	2.46E-01	1.00E+00
ENSBTAG00000013749	9.30E-01	9.44E-02	3.17E-01	6.27E-01	2.52E+00	3.25E-13	2.72E+00	6.12E-15	-4.60E-01	8.00E-01
ENSBTAG00000015719	-5.61E-02	1.00E+00	-4.40E-01	1.00E-01	3.67E-01	1.86E-01	5.72E-01	2.22E-02	-4.14E-01	4.56E-01
ENSBTAG00000015723	-1.57E-02	1.00E+00	-3.19E-01	2.35E-01	-7.83E-02	1.00E+00	2.11E-01	5.06E-01	-3.70E-01	4.92E-01
ENSBTAG00000008987	2.62E-01	7.67E-01	1.13E+00	2.31E-08	9.37E-01	4.39E-06	1.16E+00	9.95E-09	4.84E-01	2.18E-01
ENSBTAG00000047160	0.00E+00	1.00E+00	0.00E+00	1.00E+00	-5.13E-15	1.00E+00	0.00E+00	1.00E+00	0.00E+00	1.00E+00
ENSBTAG00000003358	4.54E-01	7.43E-01	5.29E-01	2.28E-01	3.13E-01	5.78E-01	4.77E-01	3.09E-01	1.25E-01	1.00E+00

ENSBTAG00000032558	5.86E-01	4.18E-01	1.51E+00	5.81E-06	1.56E+00	2.79E-06	2.07E+00	5.22E-10	5.88E-01	4.70E-01
ENSBTAG00000001575	-2.47E-01	9.87E-01	-1.44E+00	1.49E-06	-9.01E-01	3.66E-03	-3.03E-01	4.97E-01	-2.83E-01	8.74E-01
ENSBTAG00000006474	-4.27E-01	9.39E-01	-1.13E+00	3.06E-02	-1.97E+00	6.27E-05	-1.53E+00	2.63E-03	-3.81E-01	9.70E-01
ENSBTAG00000042135	6.04E-03	1.00E+00	-1.91E+00	4.69E-01	-1.91E+00	4.28E-01	-1.47E-01	1.00E+00	-1.91E+00	8.00E-01
ENSBTAG00000002742	1.13E-02	1.00E+00	-3.24E-01	3.97E-01	-4.87E-01	1.28E-01	-5.53E-01	7.99E-02	1.46E-01	1.00E+00
ENSBTAG00000017500	2.14E+00	2.34E-01	2.22E+00	4.85E-02	1.01E+00	5.92E-01	2.06E+00	7.64E-02	1.20E+00	8.24E-01
ENSBTAG00000001424	-1.03E-01	1.00E+00	-5.41E-01	1.68E-02	-2.17E-02	1.00E+00	2.82E-01	3.10E-01	-5.59E-02	1.00E+00
ENSBTAG00000001425	6.09E-02	1.00E+00	2.56E-01	3.16E-01	-1.59E-01	6.36E-01	-3.29E-02	1.00E+00	-1.09E-01	1.00E+00
ENSBTAG00000015151	5.14E-01	6.55E-01	6.99E-01	9.91E-02	2.26E-01	8.11E-01	4.61E-01	3.65E-01	5.34E-01	6.27E-01
ENSBTAG00000002570	1.22E-01	1.00E+00	-6.91E-01	4.89E-03	-1.09E-02	1.00E+00	2.02E-01	5.96E-01	1.88E-02	1.00E+00
ENSBTAG00000004168	1.84E-01	1.00E+00	1.95E+00	2.06E-11	6.13E-01	6.62E-02	1.07E+00	4.38E-04	-3.98E-01	7.29E-01
ENSBTAG00000023999	6.33E-01	1.00E+00	1.94E+00	6.69E-03	1.37E+00	9.29E-02	8.54E-01	4.39E-01	-1.95E-01	1.00E+00
ENSBTAG00000016573	1.44E+00	7.62E-01	2.08E+00	8.46E-02	-1.47E-01	1.00E+00	2.06E+00	8.38E-02	8.98E-01	1.00E+00
ENSBTAG00000027015	1.00E-01	1.00E+00	-5.62E-03	1.00E+00	1.97E-02	1.00E+00	-1.44E-01	1.00E+00	-7.41E-01	4.50E-01
ENSBTAG00000032424	-7.91E-03	1.00E+00	-7.38E-01	5.38E-01	-4.51E-01	8.14E-01	6.69E-01	5.22E-01	-9.57E-02	1.00E+00
ENSBTAG00000046199	1.47E-01	1.00E+00	-1.50E-01	1.00E+00	-7.69E-01	7.35E-02	-4.19E-01	4.39E-01	-1.23E-01	1.00E+00
ENSBTAG00000044573	0.00E+00	1.00E+00	0.00E+00	1.00E+00	-5.13E-15	1.00E+00	0.00E+00	1.00E+00	0.00E+00	1.00E+00
ENSBTAG00000024021	-2.02E-01	1.00E+00	-2.94E-02	1.00E+00	-1.75E-01	9.86E-01	-2.45E-01	8.49E-01	-4.01E-01	8.93E-01
ENSBTAG00000014072	-1.65E-01	1.00E+00	5.01E-01	5.78E-01	1.76E-01	1.00E+00	8.48E-01	1.90E-01	8.13E-02	1.00E+00
ENSBTAG00000027786	0.00E+00	1.00E+00	1.98E+00	4.43E-01	-5.13E-15	1.00E+00	0.00E+00	1.00E+00	0.00E+00	1.00E+00
ENSBTAG00000002943	2.56E-01	1.00E+00	-1.51E+00	3.31E-03	-2.37E-01	8.51E-01	8.21E-01	6.20E-02	6.72E-01	5.03E-01
ENSBTAG00000019458	1.43E-01	1.00E+00	-2.60E-01	3.69E-01	-2.19E-01	4.79E-01	-3.43E-01	1.89E-01	1.09E-01	1.00E+00
ENSBTAG00000019459	0.00E+00	1.00E+00	0.00E+00	1.00E+00	-5.13E-15	1.00E+00	0.00E+00	1.00E+00	0.00E+00	1.00E+00
ENSBTAG00000008068	3.48E-01	6.13E-01	1.12E+00	2.89E-06	9.97E-01	3.23E-05	1.05E+00	1.30E-05	4.53E-01	4.21E-01
ENSBTAG00000000102	-2.84E-01	1.00E+00	1.80E+00	3.02E-02	1.91E+00	1.98E-02	1.02E+00	3.10E-01	1.16E+00	6.01E-01
ENSBTAG00000020262	-2.79E-01	6.00E-01	-3.68E-01	9.24E-02	-5.58E-01	5.04E-03	-4.44E-01	3.69E-02	-3.45E-01	4.43E-01
ENSBTAG00000024019	1.92E+00	8.23E-01	0.00E+00	1.00E+00	-5.13E-15	1.00E+00	0.00E+00	1.00E+00	1.91E+00	8.00E-01
ENSBTAG00000006852	-2.32E-01	8.28E-01	-7.01E-01	2.21E-03	-1.12E+00	3.22E-07	-1.61E+00	2.69E-13	-4.48E-01	3.43E-01
ENSBTAG00000046563	0.00E+00	1.00E+00	0.00E+00	1.00E+00	-5.13E-15	1.00E+00	0.00E+00	1.00E+00	0.00E+00	1.00E+00
ENSBTAG00000042750	0.00E+00	1.00E+00	0.00E+00	1.00E+00	-5.13E-15	1.00E+00	0.00E+00	1.00E+00	0.00E+00	1.00E+00
ENSBTAG00000006861	-9.32E-01	8.75E-01	2.55E-01	1.00E+00	-1.10E+00	3.95E-01	-2.77E+00	3.36E-02	-9.44E-01	8.34E-01
ENSBTAG00000006995	1.05E-01	1.00E+00	6.19E-01	1.97E-02	6.94E-03	1.00E+00	-1.85E-01	7.02E-01	2.66E-01	8.00E-01
ENSBTAG00000044469	0.00E+00	1.00E+00	0.00E+00	1.00E+00	-5.13E-15	1.00E+00	0.00E+00	1.00E+00	0.00E+00	1.00E+00
ENSBTAG00000045474	0.00E+00	1.00E+00	0.00E+00	1.00E+00	-5.13E-15	1.00E+00	0.00E+00	1.00E+00	0.00E+00	1.00E+00
ENSBTAG00000011101	-1.82E-01	1.00E+00	-8.18E-01	2.15E-01	-2.76E+00	7.16E-07	-4.37E+00	1.21E-13	2.88E-01	1.00E+00

ENSBTAG00000011104	-3.53E-01	6.49E-01	-1.20E+00	1.69E-06	-2.43E+00	2.94E-22	-2.51E+00	2.04E-23	2.33E-02	1.00E+00
ENSBTAG00000000295	1.61E-01	1.00E+00	-1.04E+00	5.94E-04	-3.62E-01	3.62E-01	-4.97E-01	1.66E-01	-6.22E-02	1.00E+00
ENSBTAG00000015473	1.42E-02	1.00E+00	2.59E-01	5.42E-01	2.66E-01	5.16E-01	2.85E-01	4.97E-01	-2.47E-01	9.06E-01
ENSBTAG00000015481	3.78E-03	1.00E+00	1.08E-01	9.57E-01	5.43E-01	3.62E-02	3.41E-01	2.70E-01	-7.87E-02	1.00E+00
ENSBTAG00000017460	2.12E-01	1.00E+00	1.56E+00	3.74E-09	1.95E+00	5.82E-14	1.15E+00	2.54E-05	-2.76E-01	8.57E-01
ENSBTAG00000017469	3.34E-02	1.00E+00	-4.36E-02	1.00E+00	-5.76E-01	3.56E-02	-2.98E-02	1.00E+00	-5.62E-01	2.58E-01
ENSBTAG00000000542	-4.29E-02	1.00E+00	-4.67E-01	2.12E-01	-5.02E-01	1.68E-01	-2.86E-01	5.46E-01	-4.25E-01	6.61E-01
ENSBTAG00000000545	2.81E-01	7.23E-01	7.92E-01	2.63E-04	1.07E+00	3.39E-07	1.00E+00	3.14E-06	2.94E-01	6.83E-01
ENSBTAG00000008909	6.27E-02	1.00E+00	8.28E-01	1.20E-02	6.28E-01	7.31E-02	3.74E-01	3.95E-01	-3.34E-01	8.00E-01
ENSBTAG00000046259	6.51E-01	6.06E-01	9.66E-01	4.51E-02	1.10E+00	1.42E-02	7.20E-01	1.80E-01	3.63E-01	9.82E-01
ENSBTAG00000017448	-8.30E-01	5.73E-01	-9.41E-01	1.39E-01	-1.32E+00	2.47E-02	-1.84E+00	1.69E-03	2.51E-03	1.00E+00
ENSBTAG00000036435	0.00E+00	1.00E+00	0.00E+00	1.00E+00	-5.13E-15	1.00E+00	0.00E+00	1.00E+00	0.00E+00	1.00E+00
ENSBTAG00000029903	2.72E+00	5.76E-01	1.99E+00	4.43E-01	-5.13E-15	1.00E+00	0.00E+00	1.00E+00	0.00E+00	1.00E+00
ENSBTAG00000030046	2.70E+00	5.76E-01	0.00E+00	1.00E+00	-5.13E-15	1.00E+00	0.00E+00	1.00E+00	0.00E+00	1.00E+00
ENSBTAG00000012394	-6.64E-01	9.73E-01	-2.63E-01	1.00E+00	-6.95E-01	5.67E-01	-3.91E-02	1.00E+00	-6.53E-01	9.44E-01
ENSBTAG00000033801	3.29E-02	1.00E+00	-1.82E-01	6.42E-01	2.40E-01	4.39E-01	3.98E-01	1.25E-01	1.32E-01	1.00E+00
ENSBTAG00000005870	1.49E-01	1.00E+00	5.15E-01	1.97E-02	5.32E-01	1.48E-02	3.36E-01	1.85E-01	-3.48E-01	5.24E-01
ENSBTAG00000016534	4.01E-01	1.00E+00	4.16E-01	7.01E-01	2.98E-02	1.00E+00	1.26E-01	1.00E+00	-1.09E-01	1.00E+00
ENSBTAG00000007414	5.65E-01	3.19E-01	1.91E+00	1.04E-11	1.71E+00	1.17E-09	1.20E+00	3.98E-05	1.88E-01	1.00E+00
ENSBTAG00000015386	1.93E+00	1.44E-07	1.88E+00	5.30E-08	8.86E-01	2.80E-02	1.49E+00	3.02E-05	6.37E-01	5.37E-01
ENSBTAG00000009694	3.87E-01	4.03E-01	-7.13E-01	1.97E-03	-1.11E+00	5.26E-07	-8.44E-01	2.06E-04	1.16E-02	1.00E+00
ENSBTAG00000005257	1.99E-01	1.00E+00	1.68E-01	7.90E-01	4.47E-01	1.46E-01	6.32E-01	2.71E-02	-8.33E-02	1.00E+00
ENSBTAG00000044218	0.00E+00	1.00E+00	0.00E+00	1.00E+00	-5.13E-15	1.00E+00	0.00E+00	1.00E+00	0.00E+00	1.00E+00
ENSBTAG00000008048	8.35E-01	4.00E-02	2.30E+00	3.22E-16	1.27E+00	1.02E-05	1.24E+00	2.34E-05	2.45E-03	1.00E+00
ENSBTAG00000002557	3.22E-02	1.00E+00	-4.01E-01	2.19E-01	-9.54E-01	3.75E-04	-8.75E-01	1.51E-03	-3.45E-01	7.29E-01
ENSBTAG00000018554	6.38E-02	1.00E+00	-3.80E-01	1.86E-01	-7.20E-01	3.37E-03	-3.46E-01	2.55E-01	1.32E-01	1.00E+00
ENSBTAG00000022461	-1.52E-01	1.00E+00	-5.19E-01	2.49E-02	-5.00E-01	3.09E-02	6.95E-02	1.00E+00	-3.94E-02	1.00E+00
ENSBTAG00000038011	2.49E-01	1.00E+00	-8.59E-03	1.00E+00	9.21E-02	1.00E+00	1.18E-01	1.00E+00	1.64E-01	1.00E+00
ENSBTAG00000019628	4.80E-01	1.00E+00	1.30E+00	2.10E-01	8.17E-02	1.00E+00	-4.92E-01	9.38E-01	-2.80E-01	1.00E+00
ENSBTAG00000000157	-6.32E-01	1.96E-01	-1.55E-01	8.77E-01	-1.57E-01	8.61E-01	2.20E-01	6.70E-01	2.84E-01	8.32E-01
ENSBTAG00000000152	2.46E-01	8.78E-01	1.83E+00	7.81E-14	1.76E+00	4.56E-13	1.11E+00	8.59E-06	-6.64E-02	1.00E+00
ENSBTAG00000010971	-5.69E-02	1.00E+00	-2.48E-01	4.68E-01	1.76E-01	6.79E-01	6.02E-01	1.51E-02	-1.11E-01	1.00E+00
ENSBTAG00000016038	3.68E-01	8.09E-01	5.11E-01	1.72E-01	1.26E+00	5.45E-05	1.47E+00	2.40E-06	3.68E-01	8.00E-01
ENSBTAG00000016032	-2.16E-01	1.00E+00	-9.15E-01	2.27E-01	-1.26E+00	6.73E-02	-1.47E+00	3.19E-02	6.38E-01	8.03E-01
ENSBTAG00000032051	3.92E-01	5.14E-01	-1.08E+00	8.50E-06	-3.47E+00	2.38E-43	-3.44E+00	1.10E-42	6.56E-01	9.77E-02

ENSBTAG00000038369	9.37E-01	1.95E-01	-5.08E-01	4.54E-01	-1.67E+00	1.53E-03	-1.50E+00	5.02E-03	1.75E+00	7.29E-04
ENSBTAG00000002803	7.12E-01	4.13E-01	-1.12E+00	2.06E-02	-2.13E+00	7.96E-06	-6.26E-01	2.51E-01	9.35E-01	2.12E-01
ENSBTAG00000043493	0.00E+00	1.00E+00	0.00E+00	1.00E+00	-5.13E-15	1.00E+00	0.00E+00	1.00E+00	0.00E+00	1.00E+00
ENSBTAG00000004352	0.00E+00	1.00E+00	2.79E+00	2.10E-01	2.56E+00	2.54E-01	0.00E+00	1.00E+00	3.22E+00	4.34E-01
ENSBTAG00000000703	1.21E+00	6.02E-01	1.18E-01	1.00E+00	7.07E-01	6.27E-01	1.03E+00	3.65E-01	1.23E+00	6.11E-01
ENSBTAG00000005614	6.23E-02	1.00E+00	7.52E-01	5.44E-04	1.10E+00	1.15E-07	9.91E-01	2.79E-06	1.77E-01	9.62E-01
ENSBTAG00000044532	0.00E+00	1.00E+00	0.00E+00	1.00E+00	-5.13E-15	1.00E+00	0.00E+00	1.00E+00	0.00E+00	1.00E+00
ENSBTAG00000012637	-5.65E-01	1.00E+00	3.66E-01	9.30E-01	-5.31E-01	7.79E-01	-1.01E+00	4.19E-01	-5.66E-01	1.00E+00
ENSBTAG00000015205	-1.74E-01	1.00E+00	7.42E-01	6.64E-02	1.05E+00	5.10E-03	7.80E-01	5.61E-02	1.71E-02	1.00E+00
ENSBTAG00000042106	0.00E+00	1.00E+00	0.00E+00	1.00E+00	-5.13E-15	1.00E+00	0.00E+00	1.00E+00	0.00E+00	1.00E+00
ENSBTAG00000014159	4.26E-02	1.00E+00	-8.85E-01	6.30E-02	-3.17E-03	1.00E+00	7.41E-01	8.77E-02	2.09E-02	1.00E+00
ENSBTAG00000014166	-2.76E-01	6.87E-01	9.97E-01	4.48E-07	1.65E+00	1.09E-17	1.04E+00	1.55E-07	-1.97E-01	8.60E-01
ENSBTAG00000007895	2.55E-01	1.00E+00	1.14E+00	4.61E-04	1.26E+00	8.84E-05	9.00E-01	8.54E-03	-5.45E-01	5.03E-01
ENSBTAG00000042290	0.00E+00	1.00E+00	0.00E+00	1.00E+00	-5.13E-15	1.00E+00	0.00E+00	1.00E+00	0.00E+00	1.00E+00
ENSBTAG00000002288	5.36E-03	1.00E+00	-1.91E+00	4.69E-01	-1.91E+00	4.28E-01	1.12E+00	6.70E-01	-1.91E+00	8.00E-01
ENSBTAG00000002298	-4.40E-01	9.57E-01	-7.78E-01	2.36E-01	-7.66E-01	2.25E-01	-1.03E-01	1.00E+00	-7.16E-01	6.74E-01
ENSBTAG00000010349	6.38E+00	6.10E-14	2.99E+00	9.82E-05	1.11E+00	2.50E-01	3.83E+00	7.25E-07	3.95E-01	1.00E+00
ENSBTAG00000047637	-1.03E-01	1.00E+00	3.09E-01	2.99E-01	8.11E-01	4.35E-04	8.48E-01	2.71E-04	-7.94E-02	1.00E+00
ENSBTAG00000001321	4.03E+00	9.93E-24	2.13E+00	1.63E-08	9.43E-01	2.11E-02	5.45E-01	2.71E-01	1.78E-01	1.00E+00
ENSBTAG00000013675	-7.93E-01	1.00E+00	-7.23E-01	9.45E-01	-2.71E+00	1.99E-01	-2.71E+00	2.05E-01	-2.71E+00	6.01E-01
ENSBTAG00000039476	0.00E+00	1.00E+00	0.00E+00	1.00E+00	-5.13E-15	1.00E+00	0.00E+00	1.00E+00	0.00E+00	1.00E+00
ENSBTAG00000002085	1.92E+00	8.23E-01	0.00E+00	1.00E+00	-5.13E-15	1.00E+00	3.03E+00	1.50E-01	0.00E+00	1.00E+00
ENSBTAG00000002087	1.92E+00	8.23E-01	0.00E+00	1.00E+00	-5.13E-15	1.00E+00	1.76E+00	4.97E-01	0.00E+00	1.00E+00
ENSBTAG00000002088	-2.71E+00	5.76E-01	-7.23E-01	9.45E-01	-1.48E-01	1.00E+00	-1.78E-01	1.00E+00	-2.71E+00	6.01E-01
ENSBTAG00000039839	0.00E+00	1.00E+00	0.00E+00	1.00E+00	-5.13E-15	1.00E+00	0.00E+00	1.00E+00	1.91E+00	8.00E-01
ENSBTAG00000018012	1.91E+00	8.23E-01	2.79E+00	2.10E-01	-5.13E-15	1.00E+00	0.00E+00	1.00E+00	1.91E+00	8.00E-01
ENSBTAG00000019665	2.85E+00	5.78E-14	3.66E+00	1.20E-22	3.02E+00	1.26E-16	2.56E+00	1.78E-12	2.01E-01	1.00E+00
ENSBTAG00000019072	3.06E-03	1.00E+00	-4.53E-01	1.85E-01	-3.35E-01	3.78E-01	-5.02E-01	1.32E-01	-1.89E-01	1.00E+00
ENSBTAG00000029076	-4.96E-01	1.00E+00	-1.22E+00	6.14E-01	-6.50E-01	8.99E-01	-3.21E+00	9.96E-02	3.90E-01	1.00E+00
ENSBTAG00000019354	-5.34E-01	8.81E-01	-2.03E+00	4.05E-04	-1.70E+00	3.17E-03	-1.26E+00	3.92E-02	-2.44E-01	1.00E+00
ENSBTAG00000047185	0.00E+00	1.00E+00	0.00E+00	1.00E+00	-5.13E-15	1.00E+00	0.00E+00	1.00E+00	0.00E+00	1.00E+00
ENSBTAG00000047029	-1.13E-01	1.00E+00	3.96E-02	1.00E+00	3.24E-01	3.82E-01	4.19E-01	2.13E-01	-9.34E-02	1.00E+00
ENSBTAG00000003408	1.84E-01	1.00E+00	-1.83E-02	1.00E+00	-7.20E-01	4.39E-01	-6.69E-01	4.97E-01	9.22E-02	1.00E+00
ENSBTAG00000045514	0.00E+00	1.00E+00	0.00E+00	1.00E+00	-5.13E-15	1.00E+00	0.00E+00	1.00E+00	0.00E+00	1.00E+00
ENSBTAG00000045659	0.00E+00	1.00E+00	0.00E+00	1.00E+00	-5.13E-15	1.00E+00	0.00E+00	1.00E+00	0.00E+00	1.00E+00

ENSBTAG00000005895	-1.63E+00	5.78E-01	7.24E-01	5.56E-01	-6.28E-01	7.45E-01	-1.31E+00	3.34E-01	-2.43E+00	3.70E-01
ENSBTAG00000002866	6.38E-01	3.80E-02	2.19E+00	3.45E-25	1.87E+00	5.21E-19	1.34E+00	4.68E-10	-5.29E-01	2.39E-01
ENSBTAG00000000184	3.87E-01	7.48E-01	2.17E+00	4.88E-13	1.16E+00	1.37E-04	1.20E+00	1.07E-04	2.79E-01	8.97E-01
ENSBTAG00000009033	-2.12E+00	7.98E-04	-2.75E+00	2.21E-06	-4.38E+00	3.73E-11	-5.28E+00	8.49E-13	-1.75E+00	1.85E-02
ENSBTAG00000040093	1.18E-02	1.00E+00	7.78E-02	1.00E+00	6.45E-01	9.78E-01	-1.91E+00	4.39E-01	7.05E-03	1.00E+00
ENSBTAG00000017953	-1.29E+00	9.36E-01	-1.23E+00	6.12E-01	-1.52E-01	1.00E+00	-3.21E+00	9.92E-02	-4.95E-01	1.00E+00
ENSBTAG00000004364	-8.63E-01	4.08E-01	-3.15E+00	8.75E-10	-4.47E+00	1.11E-16	-2.85E+00	2.49E-08	-3.43E-01	1.00E+00
ENSBTAG00000018054	-1.28E+00	2.45E-02	-1.94E+00	6.13E-06	-2.85E+00	3.44E-11	-3.45E+00	4.62E-15	-8.09E-01	4.01E-01
ENSBTAG00000021120	-5.04E-01	1.00E+00	7.67E-01	7.45E-01	1.14E+00	4.28E-01	1.10E+00	4.61E-01	6.64E-01	1.00E+00
ENSBTAG00000002542	1.50E-01	1.00E+00	3.71E-01	1.70E-01	3.09E-01	2.83E-01	-3.16E-01	2.81E-01	-2.15E-01	8.72E-01
ENSBTAG00000002545	1.37E-02	1.00E+00	1.39E+00	5.18E-01	-1.24E-01	1.00E+00	-1.91E+00	4.40E-01	1.31E+00	8.99E-01
ENSBTAG00000008956	1.03E-01	1.00E+00	-3.21E-01	6.66E-01	-9.82E-01	2.98E-02	-5.21E-01	3.47E-01	-6.02E-01	6.20E-01
ENSBTAG00000021141	-1.84E-01	1.00E+00	-7.19E-01	4.49E-02	-1.13E+00	7.10E-04	-8.47E-01	1.58E-02	-8.24E-01	1.48E-01
ENSBTAG00000021140	-2.65E-01	1.00E+00	-1.23E-01	1.00E+00	-4.69E-01	2.98E-01	3.80E-01	4.54E-01	7.15E-01	3.25E-01
ENSBTAG00000020947	-4.08E-01	5.06E-01	-1.27E+00	4.05E-07	-8.35E-01	1.14E-03	-2.46E-01	5.16E-01	1.22E-01	1.00E+00
ENSBTAG00000013589	-2.39E-01	7.69E-01	-4.97E-01	1.57E-02	4.59E-02	1.00E+00	3.51E-01	1.15E-01	-3.51E-02	1.00E+00
ENSBTAG00000013580	1.05E-01	1.00E+00	7.06E-01	2.82E-03	8.74E-01	1.37E-04	6.47E-01	7.93E-03	3.20E-01	6.61E-01
ENSBTAG00000004824	1.15E+00	5.03E-01	1.14E+00	1.83E-01	2.06E-01	1.00E+00	1.43E+00	6.01E-02	6.25E-01	9.44E-01
ENSBTAG00000044483	0.00E+00	1.00E+00	0.00E+00	1.00E+00	-5.13E-15	1.00E+00	0.00E+00	1.00E+00	0.00E+00	1.00E+00
ENSBTAG00000004823	-5.78E-02	1.00E+00	3.09E-01	3.07E-01	5.11E-01	4.44E-02	4.02E-01	1.50E-01	-4.20E-01	4.49E-01
ENSBTAG00000003307	-8.48E-02	1.00E+00	7.12E-02	1.00E+00	4.03E-01	1.03E-01	6.62E-01	3.47E-03	4.37E-03	1.00E+00
ENSBTAG00000006482	4.88E-02	1.00E+00	1.12E+00	1.92E-09	1.50E+00	2.01E-16	1.21E+00	5.89E-11	-2.55E-01	7.22E-01
ENSBTAG00000042600	0.00E+00	1.00E+00	0.00E+00	1.00E+00	-5.13E-15	1.00E+00	0.00E+00	1.00E+00	0.00E+00	1.00E+00
ENSBTAG00000006481	5.46E-02	1.00E+00	1.63E+00	3.81E-14	1.99E+00	1.41E-20	1.75E+00	4.13E-16	2.28E-01	8.29E-01
ENSBTAG00000045256	0.00E+00	1.00E+00	0.00E+00	1.00E+00	-5.13E-15	1.00E+00	0.00E+00	1.00E+00	0.00E+00	1.00E+00
ENSBTAG00000011601	-3.36E-01	6.15E-01	-1.17E+00	8.65E-07	-7.06E-01	3.66E-03	-3.95E-01	1.61E-01	-2.82E-01	7.90E-01
ENSBTAG00000024000	-7.41E-01	7.27E-02	-3.17E+00	8.60E-27	-3.53E+00	2.51E-32	-2.92E+00	7.52E-24	-9.93E-02	1.00E+00
ENSBTAG00000047449	5.38E-01	8.23E-01	6.25E-01	3.58E-01	-1.34E-01	1.00E+00	-4.21E-01	6.39E-01	-3.73E-01	1.00E+00
ENSBTAG00000039981	-2.18E-01	1.00E+00	-4.77E-02	1.00E+00	7.29E-01	1.32E-02	1.14E+00	4.61E-05	-4.18E-02	1.00E+00
ENSBTAG00000047816	2.40E-01	1.00E+00	6.34E-01	4.42E-01	-2.52E-01	9.69E-01	-3.61E-01	8.06E-01	-9.59E-01	5.49E-01
ENSBTAG00000031828	5.30E-01	1.00E+00	-7.17E-01	9.45E-01	-1.48E-01	1.00E+00	-1.71E-01	1.00E+00	6.92E-03	1.00E+00
ENSBTAG00000021230	-3.88E+00	1.44E-01	-1.89E+00	2.58E-01	-3.88E+00	2.28E-02	-2.12E+00	1.79E-01	-1.17E+00	8.38E-01
ENSBTAG00000010691	-2.42E-02	1.00E+00	4.15E-01	4.64E-02	7.16E-01	1.45E-04	4.14E-01	5.10E-02	-1.70E-01	9.10E-01
ENSBTAG00000018291	-1.67E-01	1.00E+00	-1.34E+00	9.87E-10	-9.51E-01	1.45E-05	-4.72E-01	5.74E-02	9.15E-02	1.00E+00
ENSBTAG00000018289	-8.63E-02	1.00E+00	2.17E+00	4.91E-14	7.14E-01	2.37E-02	8.23E-01	8.62E-03	-4.89E-01	5.38E-01

ENSBTAG00000018286	1.38E-02	1.00E+00	3.31E-02	1.00E+00	-1.13E-02	1.00E+00	3.37E-01	2.04E-01	-2.50E-01	8.00E-01
ENSBTAG00000018285	3.42E-01	6.59E-01	-7.88E-02	1.00E+00	1.60E-01	7.80E-01	-1.67E-01	7.69E-01	-2.04E-01	9.60E-01
ENSBTAG00000023997	1.62E-01	1.00E+00	1.73E-01	6.41E-01	-1.47E-01	7.39E-01	-1.00E-01	9.43E-01	6.31E-02	1.00E+00
ENSBTAG00000038477	1.44E-01	1.00E+00	-7.07E-01	1.43E-03	-5.86E-01	1.01E-02	-1.87E-01	6.08E-01	8.25E-01	3.95E-03
ENSBTAG00000020873	-1.23E-01	1.00E+00	5.45E-01	2.15E-02	4.07E-01	1.10E-01	-5.22E-02	1.00E+00	5.55E-02	1.00E+00
ENSBTAG00000020828	-8.75E-01	8.23E-01	-2.08E+00	3.65E-02	-3.61E+00	9.40E-04	-3.61E+00	1.08E-03	-3.58E-01	1.00E+00
ENSBTAG00000005432	-3.42E-01	8.23E-01	-2.08E+00	9.02E-10	-1.07E+00	7.69E-04	-2.03E-01	7.61E-01	-3.85E-01	7.61E-01
ENSBTAG00000021935	-3.83E-01	2.64E-01	-1.22E+00	4.65E-11	-1.24E+00	1.36E-11	-1.27E+00	7.88E-12	6.40E-02	1.00E+00
ENSBTAG00000021934	-6.01E-01	2.27E-01	-1.24E+00	1.25E-05	-2.12E+00	3.95E-14	-2.53E+00	4.95E-19	-6.08E-02	1.00E+00
ENSBTAG00000038116	-1.76E-01	1.00E+00	-2.30E-01	6.50E-01	-2.49E-01	5.93E-01	1.08E-01	1.00E+00	4.58E-01	5.66E-01
ENSBTAG00000010111	-3.06E-01	8.23E-01	-1.35E+00	5.10E-07	-2.67E+00	5.48E-22	-1.64E+00	1.21E-09	-1.61E-02	1.00E+00
ENSBTAG00000047475	-2.23E-01	1.00E+00	-7.90E-01	4.51E-02	-2.21E+00	1.45E-08	-1.00E+00	8.22E-03	5.27E-02	1.00E+00
ENSBTAG00000031731	5.12E-02	1.00E+00	1.77E+00	1.03E-11	1.46E+00	2.16E-08	1.42E+00	7.17E-08	-2.35E-01	9.41E-01
ENSBTAG00000020630	2.00E-01	8.23E-01	6.32E-01	5.38E-04	8.26E-01	2.88E-06	6.62E-01	3.08E-04	-1.93E-02	1.00E+00
ENSBTAG00000043092	-1.91E+00	8.23E-01	-1.91E+00	4.69E-01	-1.91E+00	4.28E-01	-1.91E+00	4.39E-01	7.16E-03	1.00E+00
ENSBTAG00000005654	-9.78E-02	1.00E+00	-4.52E-01	1.02E-01	-4.25E-01	1.32E-01	-3.13E-01	3.34E-01	-2.84E-01	8.00E-01
ENSBTAG00000042444	0.00E+00	1.00E+00	0.00E+00	1.00E+00	-5.13E-15	1.00E+00	0.00E+00	1.00E+00	0.00E+00	1.00E+00
ENSBTAG00000006075	-3.07E-02	1.00E+00	-2.99E-01	4.43E-01	-3.30E-01	3.80E-01	-1.07E-01	1.00E+00	-8.63E-02	1.00E+00
ENSBTAG00000037167	0.00E+00	1.00E+00	0.00E+00	1.00E+00	-5.13E-15	1.00E+00	0.00E+00	1.00E+00	0.00E+00	1.00E+00
ENSBTAG00000043701	0.00E+00	1.00E+00	0.00E+00	1.00E+00	-5.13E-15	1.00E+00	0.00E+00	1.00E+00	0.00E+00	1.00E+00
ENSBTAG00000043685	0.00E+00	1.00E+00	0.00E+00	1.00E+00	-5.13E-15	1.00E+00	0.00E+00	1.00E+00	0.00E+00	1.00E+00
ENSBTAG00000042855	0.00E+00	1.00E+00	0.00E+00	1.00E+00	-5.13E-15	1.00E+00	0.00E+00	1.00E+00	0.00E+00	1.00E+00
ENSBTAG00000036889	0.00E+00	1.00E+00	0.00E+00	1.00E+00	-5.13E-15	1.00E+00	0.00E+00	1.00E+00	0.00E+00	1.00E+00
ENSBTAG00000043871	0.00E+00	1.00E+00	0.00E+00	1.00E+00	-5.13E-15	1.00E+00	0.00E+00	1.00E+00	0.00E+00	1.00E+00
ENSBTAG00000046487	-1.91E+00	8.23E-01	-1.91E+00	4.69E-01	-1.91E+00	4.28E-01	-1.91E+00	4.39E-01	-1.91E+00	8.00E-01
ENSBTAG00000039912	0.00E+00	1.00E+00	0.00E+00	1.00E+00	-5.13E-15	1.00E+00	0.00E+00	1.00E+00	0.00E+00	1.00E+00
ENSBTAG00000046697	0.00E+00	1.00E+00	0.00E+00	1.00E+00	-5.13E-15	1.00E+00	0.00E+00	1.00E+00	0.00E+00	1.00E+00
ENSBTAG00000031669	-7.53E-02	1.00E+00	4.43E-01	7.11E-01	-6.90E-02	1.00E+00	1.02E+00	1.56E-01	-6.43E-01	8.29E-01
ENSBTAG00000047167	0.00E+00	1.00E+00	0.00E+00	1.00E+00	-5.13E-15	1.00E+00	0.00E+00	1.00E+00	0.00E+00	1.00E+00
ENSBTAG00000010544	0.00E+00	1.00E+00	0.00E+00	1.00E+00	-5.13E-15	1.00E+00	0.00E+00	1.00E+00	0.00E+00	1.00E+00
ENSBTAG00000044723	0.00E+00	1.00E+00	0.00E+00	1.00E+00	-5.13E-15	1.00E+00	0.00E+00	1.00E+00	0.00E+00	1.00E+00
ENSBTAG00000044821	0.00E+00	1.00E+00	0.00E+00	1.00E+00	-5.13E-15	1.00E+00	0.00E+00	1.00E+00	0.00E+00	1.00E+00
ENSBTAG00000007135	2.71E+00	5.76E-01	6.96E+00	9.24E-11	8.52E+00	1.59E-22	8.76E+00	9.91E-25	4.70E+00	3.56E-02
ENSBTAG00000011314	1.34E+00	9.43E-01	5.19E+00	1.15E-06	7.24E+00	1.75E-13	7.74E+00	3.63E-15	4.90E+00	2.25E-04
ENSBTAG00000043050	0.00E+00	1.00E+00	0.00E+00	1.00E+00	-5.13E-15	1.00E+00	0.00E+00	1.00E+00	0.00E+00	1.00E+00

ENSBTAG00000000168	-3.59E+00	2.32E-01	-3.59E+00	6.68E-02	-5.31E-01	9.38E-01	3.61E-01	1.00E+00	-1.67E+00	7.79E-01
ENSBTAG00000029663	0.00E+00	1.00E+00	0.00E+00	1.00E+00	-5.13E-15	1.00E+00	0.00E+00	1.00E+00	0.00E+00	1.00E+00
ENSBTAG00000028628	0.00E+00	1.00E+00	0.00E+00	1.00E+00	-5.13E-15	1.00E+00	0.00E+00	1.00E+00	0.00E+00	1.00E+00
ENSBTAG00000000374	-1.91E+00	8.23E-01	-1.91E+00	4.69E-01	-1.91E+00	4.28E-01	-1.91E+00	4.39E-01	-1.91E+00	8.00E-01
ENSBTAG00000047149	5.36E-01	1.00E+00	-6.14E-01	9.84E-01	-1.41E+00	5.27E-01	-4.32E+00	2.67E-02	8.09E-01	1.00E+00
ENSBTAG00000006025	2.10E+00	2.66E-04	4.66E+00	1.58E-18	1.72E+00	7.68E-04	1.28E+00	1.87E-02	2.46E-01	1.00E+00
ENSBTAG00000006027	3.85E-02	1.00E+00	5.05E-01	3.84E-02	1.10E-01	9.20E-01	-1.36E-02	1.00E+00	3.39E-03	1.00E+00
ENSBTAG00000013254	-3.01E-01	9.44E-01	-2.35E-01	7.45E-01	-1.26E+00	2.63E-04	-8.59E-01	2.08E-02	-1.33E+00	3.17E-03
ENSBTAG00000039218	1.21E-01	1.00E+00	1.26E+00	6.98E-08	1.54E+00	1.69E-11	9.51E-01	7.73E-05	-1.35E-01	1.00E+00
ENSBTAG00000009290	-1.54E-02	1.00E+00	-6.31E-01	1.72E-02	-1.64E-01	7.46E-01	-4.46E-01	1.19E-01	-1.61E-01	1.00E+00
ENSBTAG00000004531	5.66E-01	3.00E-02	1.97E+00	1.22E-25	1.31E+00	2.98E-12	7.84E-01	6.00E-05	-3.44E-01	4.60E-01
ENSBTAG00000019867	-1.18E-01	1.00E+00	-8.36E-01	2.37E-03	-1.45E+00	3.45E-08	-8.67E-01	1.75E-03	-4.06E-01	6.01E-01
ENSBTAG00000018046	-5.65E-01	1.29E-01	-1.79E+00	4.65E-14	-4.06E-01	1.43E-01	-2.13E-02	1.00E+00	2.57E-01	8.00E-01
ENSBTAG00000000899	2.69E-01	8.23E-01	-1.05E+00	1.88E-05	-7.86E-01	1.46E-03	-5.87E-01	2.58E-02	1.99E-01	9.41E-01
ENSBTAG00000044173	2.00E-01	8.23E-01	-4.38E-01	3.22E-02	-9.72E-01	1.43E-07	-9.01E-01	1.60E-06	-1.77E-01	8.78E-01
ENSBTAG00000006760	1.92E+00	8.23E-01	0.00E+00	1.00E+00	-5.13E-15	1.00E+00	0.00E+00	1.00E+00	0.00E+00	1.00E+00
ENSBTAG00000023318	-2.32E-01	1.00E+00	-1.64E+00	1.43E-03	-1.12E+00	3.28E-02	4.02E-01	6.01E-01	-1.10E-01	1.00E+00
ENSBTAG00000005151	-8.40E-02	1.00E+00	-5.08E-02	1.00E+00	1.26E-01	1.00E+00	1.38E-01	1.00E+00	-2.90E-01	1.00E+00
ENSBTAG00000019295	1.21E-01	1.00E+00	1.44E-01	9.49E-01	-6.28E-02	1.00E+00	2.58E-01	6.38E-01	-4.21E-03	1.00E+00
ENSBTAG00000000111	3.86E-01	5.83E-01	5.17E-01	8.01E-02	7.60E-01	5.18E-03	7.90E-01	4.18E-03	-3.87E-01	6.05E-01
ENSBTAG00000000115	1.25E-01	1.00E+00	-2.62E-02	1.00E+00	-2.32E-02	1.00E+00	-1.20E-01	8.09E-01	-1.11E-01	1.00E+00
ENSBTAG00000020865	1.20E+00	4.66E-05	1.03E+00	1.30E-04	1.08E+00	4.32E-05	7.01E-01	1.48E-02	-5.03E-01	4.34E-01
ENSBTAG00000000156	-1.16E-02	1.00E+00	1.02E-01	1.00E+00	3.80E-01	5.04E-01	1.21E+00	1.48E-03	3.96E-01	8.33E-01
ENSBTAG00000009299	-3.09E-01	4.66E-01	-8.71E-02	9.43E-01	-2.41E-01	3.14E-01	-2.88E-01	2.07E-01	-4.67E-01	1.42E-01
ENSBTAG00000007793	-1.59E+00	8.44E-04	-3.19E+00	2.00E-12	-2.71E+00	1.71E-10	-7.76E-01	8.39E-02	3.94E-01	8.10E-01
ENSBTAG00000046117	5.39E-02	1.00E+00	-1.54E-01	7.83E-01	-6.79E-01	5.84E-03	-6.63E-01	8.58E-03	-5.59E-01	2.00E-01
ENSBTAG00000007763	0.00E+00	1.00E+00	0.00E+00	1.00E+00	-5.13E-15	1.00E+00	0.00E+00	1.00E+00	0.00E+00	1.00E+00
ENSBTAG00000016717	-4.32E-01	7.16E-01	1.82E-01	8.56E-01	4.04E-01	3.31E-01	5.86E-01	1.11E-01	1.14E-01	1.00E+00
ENSBTAG00000016720	3.71E-01	4.53E-01	1.01E+00	3.22E-06	6.91E-01	2.06E-03	5.46E-01	2.21E-02	-2.41E-02	1.00E+00
ENSBTAG00000009761	-4.67E-02	1.00E+00	7.20E-03	1.00E+00	9.91E-02	9.08E-01	1.45E-01	7.15E-01	-3.07E-01	5.98E-01
ENSBTAG00000019456	-1.86E-01	1.00E+00	-1.13E+00	1.61E-04	-4.96E-01	1.45E-01	-1.81E-01	8.04E-01	-3.61E-01	7.68E-01
ENSBTAG00000042829	0.00E+00	1.00E+00	0.00E+00	1.00E+00	-5.13E-15	1.00E+00	0.00E+00	1.00E+00	0.00E+00	1.00E+00
ENSBTAG00000043609	0.00E+00	1.00E+00	0.00E+00	1.00E+00	-5.13E-15	1.00E+00	0.00E+00	1.00E+00	0.00E+00	1.00E+00
ENSBTAG00000008448	-4.16E-01	7.41E-01	-3.24E-01	4.93E-01	-8.82E-02	1.00E+00	-2.19E-03	1.00E+00	-2.24E-01	1.00E+00
ENSBTAG00000046310	0.00E+00	1.00E+00	0.00E+00	1.00E+00	-5.13E-15	1.00E+00	0.00E+00	1.00E+00	0.00E+00	1.00E+00

ENSBTAG00000011727	-1.55E-01	1.00E+00	-1.42E+00	7.49E-12	-8.34E-01	7.01E-05	-4.67E-01	4.63E-02	-1.17E-01	1.00E+00
ENSBTAG00000004567	3.42E-01	7.56E-01	1.60E+00	6.65E-10	1.48E+00	1.09E-08	6.51E-01	2.63E-02	4.93E-01	4.34E-01
ENSBTAG00000010595	-2.78E-01	7.60E-01	-4.31E-01	8.83E-02	-1.42E-01	7.82E-01	-3.63E-01	1.81E-01	-1.38E-01	1.00E+00
ENSBTAG00000010604	2.54E-01	8.07E-01	1.36E+00	4.67E-11	1.56E+00	2.66E-14	1.25E+00	2.06E-09	-3.30E-01	6.01E-01
ENSBTAG00000010619	6.61E-01	1.73E-02	6.05E-01	6.70E-03	-8.90E-02	9.85E-01	8.62E-02	1.00E+00	2.58E-01	7.90E-01
ENSBTAG00000001408	-2.02E-01	1.00E+00	-4.58E-01	3.26E-01	-6.68E-01	9.76E-02	-7.42E-01	6.51E-02	-4.98E-01	6.51E-01
ENSBTAG00000009658	1.70E+00	4.20E-04	6.30E-01	2.17E-01	-1.21E+00	6.07E-03	-8.82E-01	6.42E-02	-1.06E-01	1.00E+00
ENSBTAG00000047772	-1.44E+00	1.20E-01	-3.12E+00	4.71E-05	-1.41E+00	2.24E-02	-8.81E-01	1.81E-01	-6.85E-02	1.00E+00
ENSBTAG00000018401	1.54E-01	1.00E+00	-1.23E+00	2.80E-05	-1.20E+00	3.63E-05	-1.34E+00	4.73E-06	-1.46E-02	1.00E+00
ENSBTAG00000020669	9.01E-01	7.30E-01	5.11E-01	7.47E-01	-9.52E-01	4.39E-01	-3.39E-01	9.83E-01	4.26E-01	1.00E+00
ENSBTAG00000023976	-4.97E-01	3.05E-01	6.66E-02	1.00E+00	-1.36E-02	1.00E+00	-2.81E-02	1.00E+00	-4.56E-01	4.45E-01
ENSBTAG00000000992	1.51E+00	8.06E-03	1.21E+00	1.17E-02	8.31E-01	1.13E-01	9.85E-01	5.41E-02	9.84E-02	1.00E+00
ENSBTAG00000038835	1.92E+00	8.23E-01	2.79E+00	2.10E-01	-5.13E-15	1.00E+00	0.00E+00	1.00E+00	1.93E+00	8.00E-01
ENSBTAG00000017199	-1.91E+00	8.23E-01	-1.91E+00	4.69E-01	-1.24E-01	1.00E+00	-1.47E-01	1.00E+00	7.05E-03	1.00E+00
ENSBTAG00000012949	1.92E+00	8.23E-01	0.00E+00	1.00E+00	-5.13E-15	1.00E+00	2.53E+00	2.71E-01	0.00E+00	1.00E+00
ENSBTAG00000007808	1.56E-01	1.00E+00	-9.29E-02	1.00E+00	-6.69E-01	9.18E-02	9.29E-02	1.00E+00	-9.97E-02	1.00E+00
ENSBTAG00000043661	0.00E+00	1.00E+00	0.00E+00	1.00E+00	-5.13E-15	1.00E+00	0.00E+00	1.00E+00	0.00E+00	1.00E+00
ENSBTAG00000017626	4.52E-01	8.23E-01	3.05E+00	1.89E-13	2.27E+00	3.30E-08	2.60E+00	3.67E-10	3.39E-01	9.65E-01
ENSBTAG00000000141	9.10E-02	1.00E+00	-3.87E-01	1.09E-01	-7.62E-01	3.18E-04	-5.78E-01	9.92E-03	-1.73E-01	9.49E-01
ENSBTAG00000047822	2.55E-01	1.00E+00	1.68E-01	1.00E+00	1.09E-01	1.00E+00	6.01E-01	2.47E-01	8.80E-01	2.83E-01
ENSBTAG00000009343	2.93E-01	9.21E-01	1.82E-01	8.53E-01	7.23E-01	3.10E-02	9.74E-01	2.42E-03	3.29E-01	8.09E-01
ENSBTAG00000001105	-9.31E-02	1.00E+00	-4.07E-01	8.52E-02	-7.47E-01	3.88E-04	-1.18E+00	8.45E-09	-4.27E-01	3.29E-01
ENSBTAG00000001108	2.87E-01	7.86E-01	3.01E-01	3.45E-01	1.00E+00	1.91E-05	9.73E-01	4.72E-05	5.15E-02	1.00E+00
ENSBTAG00000037746	4.42E-01	3.11E-01	7.52E-01	1.10E-03	4.05E-01	1.25E-01	-8.64E-02	1.00E+00	-2.11E-01	8.99E-01
ENSBTAG00000001042	1.14E+00	2.25E-02	3.15E+00	2.15E-16	1.85E+00	8.67E-07	1.51E+00	9.32E-05	6.29E-01	5.22E-01
ENSBTAG00000006497	-1.29E+00	9.39E-01	1.00E-01	1.00E+00	-6.54E-01	8.97E-01	-3.21E+00	1.00E-01	-1.28E+00	9.07E-01
ENSBTAG00000008985	9.77E-02	1.00E+00	9.39E-01	2.42E-07	9.70E-01	8.14E-08	6.43E-01	7.56E-04	-2.99E-01	5.56E-01
ENSBTAG00000016939	7.10E-01	9.92E-02	1.83E+00	1.86E-11	4.33E-01	2.17E-01	-7.44E-02	1.00E+00	1.54E-01	1.00E+00
ENSBTAG00000019182	-2.55E-01	9.17E-01	-4.82E-01	1.29E-01	-8.75E-01	1.79E-03	-1.20E+00	1.44E-05	-3.15E-01	8.00E-01
ENSBTAG00000002783	-3.23E-01	9.32E-01	-7.16E-01	7.38E-02	-1.53E+00	1.86E-05	-1.74E+00	1.48E-06	7.37E-01	3.13E-01
ENSBTAG00000001662	6.26E-02	1.00E+00	-3.17E-01	5.29E-01	-9.03E-02	1.00E+00	-4.08E-01	3.77E-01	1.48E-02	1.00E+00
ENSBTAG00000015865	-1.68E+00	7.76E-01	-1.61E+00	4.07E-01	3.68E-01	1.00E+00	-2.00E-01	1.00E+00	3.00E-01	1.00E+00
ENSBTAG00000021735	-5.07E-01	8.76E-01	-6.63E-01	3.36E-01	-1.93E+00	5.22E-04	-1.44E+00	1.20E-02	-6.97E-01	6.96E-01
ENSBTAG00000008946	-2.36E+00	6.38E-02	-2.78E+00	5.15E-03	-1.61E+00	6.03E-02	-8.18E-01	4.07E-01	-1.02E+00	6.67E-01
ENSBTAG00000005014	-6.38E-02	1.00E+00	4.08E-01	4.23E-01	1.06E+00	3.80E-03	1.11E+00	2.53E-03	2.44E-01	1.00E+00

ENSBTAG00000026111	-6.48E-01	6.85E-02	-9.05E-01	2.90E-04	-1.75E-01	7.10E-01	-3.92E-01	1.91E-01	-3.53E-01	6.24E-01
ENSBTAG00000023744	-6.75E-01	3.22E-02	-2.17E+00	2.98E-20	-1.25E+00	4.56E-08	-6.44E-01	9.30E-03	-3.44E-01	6.03E-01
ENSBTAG00000007379	-6.26E-01	9.17E-01	-2.25E+00	1.76E-03	-3.40E+00	3.04E-06	-4.46E+00	6.73E-09	-5.78E-03	1.00E+00
ENSBTAG00000007378	3.04E-01	1.00E+00	1.23E-01	1.00E+00	-8.64E-01	2.59E-01	-3.61E-01	8.04E-01	-9.82E-01	6.01E-01
ENSBTAG00000016934	7.98E-01	1.00E+00	6.92E-02	1.00E+00	-1.37E-01	1.00E+00	-1.91E+00	4.39E-01	4.42E-04	1.00E+00
ENSBTAG00000044021	-1.25E+00	3.49E-01	-1.72E+00	2.62E-02	-2.53E+00	1.23E-03	-1.81E+00	1.51E-02	-1.58E+00	2.33E-01
ENSBTAG00000018196	-5.38E-04	1.00E+00	1.92E+00	1.27E-24	1.66E+00	5.81E-19	7.71E-01	8.32E-05	-5.20E-01	1.11E-01
ENSBTAG00000046528	0.00E+00	1.00E+00	0.00E+00	1.00E+00	-5.13E-15	1.00E+00	0.00E+00	1.00E+00	0.00E+00	1.00E+00
ENSBTAG00000045956	0.00E+00	1.00E+00	0.00E+00	1.00E+00	-5.13E-15	1.00E+00	0.00E+00	1.00E+00	0.00E+00	1.00E+00
ENSBTAG00000048070	0.00E+00	1.00E+00	0.00E+00	1.00E+00	-5.13E-15	1.00E+00	0.00E+00	1.00E+00	0.00E+00	1.00E+00
ENSBTAG00000031347	4.75E-01	8.23E-01	2.30E+00	8.88E-08	3.68E-01	6.07E-01	-5.31E-01	3.96E-01	3.26E-01	1.00E+00
ENSBTAG00000012447	2.10E-01	8.23E-01	5.98E-01	2.26E-03	8.79E-01	2.68E-06	8.06E-01	2.52E-05	-3.19E-01	5.16E-01
ENSBTAG00000018669	4.89E-01	8.23E-01	3.89E-01	6.02E-01	4.35E-02	1.00E+00	-9.25E-01	1.20E-01	3.85E-01	9.29E-01
ENSBTAG00000023929	2.29E+00	5.69E-04	2.86E+00	8.10E-07	1.59E+00	7.39E-03	1.07E+00	1.03E-01	1.11E+00	3.68E-01
ENSBTAG00000031335	7.88E-04	1.00E+00	-1.09E-01	9.45E-01	-2.99E-01	3.28E-01	-1.08E-01	9.50E-01	-7.31E-02	1.00E+00
ENSBTAG00000031332	-2.18E-01	8.84E-01	-6.85E-01	3.26E-03	-5.03E-01	3.89E-02	-7.21E-01	2.03E-03	-5.07E-01	2.38E-01
ENSBTAG00000018680	1.78E-01	1.00E+00	1.50E+00	1.70E-09	2.10E+00	1.16E-17	1.16E+00	4.54E-06	-2.73E-02	1.00E+00
ENSBTAG00000003906	2.81E-01	8.85E-01	1.19E+00	2.75E-05	1.74E+00	2.98E-10	1.20E+00	2.50E-05	4.66E-01	5.52E-01
ENSBTAG00000003904	9.55E-02	1.00E+00	2.57E-01	3.93E-01	4.77E-01	4.35E-02	3.86E-01	1.33E-01	-1.53E-01	1.00E+00
ENSBTAG00000046049	-1.34E-01	1.00E+00	-1.26E+00	2.09E-02	-8.07E-01	1.54E-01	-6.28E-01	3.15E-01	5.87E-01	7.16E-01
ENSBTAG00000003902	-6.18E-02	1.00E+00	-3.47E-01	2.10E-01	3.11E-01	2.66E-01	3.41E-02	1.00E+00	2.44E-01	8.00E-01
ENSBTAG00000046280	2.70E+00	5.76E-01	0.00E+00	1.00E+00	1.78E+00	4.79E-01	1.76E+00	4.97E-01	1.91E+00	8.00E-01
ENSBTAG00000031302	1.89E+00	2.62E-01	2.77E+00	1.23E-03	4.46E+00	2.88E-11	3.94E+00	3.37E-08	2.62E+00	3.78E-02
ENSBTAG00000018164	-1.29E-01	1.00E+00	8.07E-01	5.29E-02	3.44E+00	6.60E-23	4.12E+00	2.98E-31	1.52E+00	9.94E-04
ENSBTAG00000018157	-1.68E-01	1.00E+00	-9.23E-01	1.87E-04	-6.05E-01	1.84E-02	-3.63E-01	2.27E-01	3.68E-01	5.91E-01
ENSBTAG00000018156	2.81E-01	9.70E-01	1.34E-01	1.00E+00	3.42E-01	4.70E-01	-7.74E-01	4.78E-02	2.23E-01	1.00E+00
ENSBTAG00000018153	-2.52E-02	1.00E+00	-4.03E-01	7.97E-02	4.26E-01	5.78E-02	1.01E+00	5.04E-07	8.57E-02	1.00E+00
ENSBTAG00000019522	-2.30E-01	8.23E-01	3.28E-01	1.97E-01	9.51E-01	3.45E-06	8.08E-01	1.30E-04	-2.56E-01	7.80E-01
ENSBTAG00000019518	-1.50E-02	1.00E+00	9.12E-01	5.35E-04	8.56E-01	1.19E-03	7.17E-01	9.72E-03	3.18E-02	1.00E+00
ENSBTAG00000019515	-1.77E-01	1.00E+00	-9.17E-01	3.23E-05	-1.11E+00	3.41E-07	-6.35E-01	6.54E-03	-1.29E-01	1.00E+00
ENSBTAG00000019510	-3.77E-02	1.00E+00	-5.40E-02	1.00E+00	4.13E-01	1.33E-01	4.32E-01	1.19E-01	5.78E-02	1.00E+00
ENSBTAG00000017283	-3.23E-01	4.80E-01	-2.60E-01	3.03E-01	-1.07E-01	8.60E-01	-1.84E-01	5.40E-01	1.27E-01	1.00E+00
ENSBTAG00000018269	-2.35E-01	9.80E-01	-8.28E-01	4.72E-03	-5.92E-01	5.79E-02	-5.68E-01	7.87E-02	2.40E-01	9.30E-01
ENSBTAG00000018268	-1.42E+00	7.60E-01	-1.34E+00	3.97E-01	-1.58E+00	2.64E-01	-4.13E+00	1.22E-02	-9.08E-01	9.24E-01
ENSBTAG00000018267	1.72E+00	5.90E-01	2.25E+00	8.46E-02	1.22E+00	4.76E-01	2.47E+00	5.38E-02	1.92E+00	5.32E-01

ENSBTAG00000018262	0.00E+00	1.00E+00	1.98E+00	4.43E-01	-5.13E-15	1.00E+00	0.00E+00	1.00E+00	0.00E+00	1.00E+00
ENSBTAG00000011381	3.04E+00	6.12E-02	2.48E+00	5.99E-02	1.42E+00	4.24E-01	4.36E+00	5.97E-05	1.86E+00	6.01E-01
ENSBTAG00000017894	-4.55E-02	1.00E+00	3.39E-01	2.77E-01	1.48E+00	5.91E-10	1.22E+00	5.55E-07	9.08E-02	1.00E+00
ENSBTAG00000031185	-1.15E-01	1.00E+00	-1.29E+00	3.78E-12	-1.99E+00	1.35E-26	-1.56E+00	4.28E-17	-7.41E-02	1.00E+00
ENSBTAG00000006832	-5.14E-01	4.47E-01	-4.38E-01	2.43E-01	9.47E-01	2.10E-03	1.14E+00	2.11E-04	-4.56E-01	6.01E-01
ENSBTAG00000014813	5.29E-01	1.00E+00	6.03E-01	9.45E-01	7.24E-01	8.24E-01	-9.38E-01	8.06E-01	-2.70E+00	6.01E-01
ENSBTAG00000047596	0.00E+00	1.00E+00	4.94E+00	1.04E-03	1.78E+00	4.79E-01	3.40E+00	8.36E-02	2.72E+00	6.01E-01
ENSBTAG00000007080	1.69E-01	1.00E+00	1.44E+00	7.17E-07	1.31E+00	6.32E-06	1.22E+00	3.91E-05	-3.73E-02	1.00E+00
ENSBTAG00000007077	-3.63E-01	7.42E-01	-1.19E+00	2.89E-05	-1.71E+00	1.05E-09	-1.67E+00	3.79E-09	3.42E-02	1.00E+00
ENSBTAG00000011328	-8.15E-01	1.18E-01	-1.83E+00	1.87E-07	-1.51E+00	1.02E-05	-9.01E-01	1.20E-02	5.61E-01	4.77E-01
ENSBTAG00000011325	-2.76E-02	1.00E+00	-7.29E-01	4.54E-03	-1.89E+00	6.25E-15	-2.37E+00	3.97E-22	2.46E-01	8.40E-01
ENSBTAG00000011324	-1.43E-01	1.00E+00	-1.36E-02	1.00E+00	-2.45E-01	4.74E-01	-2.55E-01	4.57E-01	-2.47E-01	8.02E-01
ENSBTAG00000013403	-1.74E-01	1.00E+00	-7.37E-01	2.37E-03	-1.00E+00	1.73E-05	-1.02E+00	1.44E-05	4.46E-01	3.64E-01
ENSBTAG00000017423	4.52E-01	2.86E-01	1.45E+00	5.77E-11	1.50E+00	1.14E-11	1.44E+00	9.36E-11	4.61E-01	3.38E-01
ENSBTAG00000008884	7.06E-02	1.00E+00	2.11E-02	1.00E+00	-7.63E-01	1.53E-03	-1.25E+00	1.01E-07	-2.13E-02	1.00E+00
ENSBTAG00000042923	0.00E+00	1.00E+00	0.00E+00	1.00E+00	-5.13E-15	1.00E+00	0.00E+00	1.00E+00	0.00E+00	1.00E+00
ENSBTAG00000018576	2.26E-02	1.00E+00	-1.62E+00	6.11E-05	-2.54E+00	1.50E-09	-3.00E+00	1.24E-11	3.30E-01	8.48E-01
ENSBTAG00000023938	-1.81E-02	1.00E+00	-8.26E-01	5.12E-02	-4.63E-01	3.27E-01	-9.40E-01	2.15E-02	-2.74E-01	9.96E-01
ENSBTAG00000018761	-6.45E-01	1.21E-01	-1.64E+00	1.11E-09	-3.11E-01	3.95E-01	4.70E-01	1.32E-01	7.29E-03	1.00E+00
ENSBTAG00000018193	-9.11E-01	6.56E-01	-1.10E+00	1.84E-01	-4.82E-01	6.51E-01	-8.86E-02	1.00E+00	-1.56E-01	1.00E+00
ENSBTAG00000005041	-1.30E+00	1.14E-01	-1.64E+00	5.27E-03	-2.31E+00	8.45E-05	-1.94E+00	7.38E-04	-8.64E-01	4.99E-01
ENSBTAG00000031150	-1.93E-01	1.00E+00	-7.56E-02	1.00E+00	-1.10E+00	4.22E-01	-1.12E+00	4.19E-01	-3.99E-01	1.00E+00
ENSBTAG00000037360	0.00E+00	1.00E+00	0.00E+00	1.00E+00	-5.13E-15	1.00E+00	0.00E+00	1.00E+00	0.00E+00	1.00E+00
ENSBTAG00000009650	-7.92E-01	8.23E-01	1.99E+00	1.03E-04	-3.53E-01	8.65E-01	-7.34E-01	4.54E-01	-1.13E+00	6.01E-01
ENSBTAG00000009649	5.71E-01	6.33E-01	4.05E+00	2.54E-31	2.98E+00	7.71E-18	1.32E+00	7.88E-04	1.26E+00	2.21E-02
ENSBTAG00000007837	5.99E-01	3.29E-01	2.38E+00	1.64E-15	1.86E+00	4.07E-10	1.31E+00	2.41E-05	3.83E-01	7.80E-01
ENSBTAG00000044553	0.00E+00	1.00E+00	0.00E+00	1.00E+00	-5.13E-15	1.00E+00	0.00E+00	1.00E+00	0.00E+00	1.00E+00
ENSBTAG00000017330	2.37E-01	1.00E+00	1.13E+00	7.77E-04	1.40E+00	1.94E-05	1.24E+00	2.37E-04	6.90E-02	1.00E+00
ENSBTAG00000005736	-3.01E-01	1.00E+00	1.59E+00	1.15E-02	3.31E-01	9.00E-01	-3.22E-01	9.50E-01	7.12E-01	8.00E-01
ENSBTAG00000010083	-1.01E-01	1.00E+00	-4.45E-01	2.30E-01	-9.87E-01	1.31E-03	-1.23E+00	5.40E-05	-2.94E-01	8.46E-01
ENSBTAG00000015038	-2.47E-01	8.54E-01	-1.05E+00	1.48E-05	-2.06E+00	5.26E-18	-2.11E+00	1.11E-18	1.18E-01	1.00E+00
ENSBTAG00000015037	-3.08E-01	1.00E+00	-7.58E-01	2.10E-01	-7.34E-02	1.00E+00	1.75E-01	9.97E-01	6.22E-01	6.10E-01
ENSBTAG00000000985	4.72E-02	1.00E+00	6.64E-01	2.20E-03	4.86E-01	3.37E-02	5.81E-01	9.98E-03	1.23E-01	1.00E+00
ENSBTAG00000019138	2.18E-01	1.00E+00	-5.47E-01	5.58E-01	-6.50E-01	4.28E-01	-4.80E-01	6.27E-01	5.20E-01	8.54E-01
ENSBTAG00000025335	1.05E-02	1.00E+00	-1.35E-01	1.00E+00	-5.79E-01	5.17E-01	-7.99E-02	1.00E+00	-5.84E-01	8.81E-01

ENSBTAG00000019983	-4.52E-02	1.00E+00	7.16E-01	2.98E-02	1.12E+00	2.53E-04	1.10E+00	4.19E-04	4.46E-01	6.15E-01
ENSBTAG00000021072	-1.23E-01	1.00E+00	-8.70E-01	8.42E-03	-1.12E+00	4.16E-04	-7.98E-01	1.95E-02	1.26E-01	1.00E+00
ENSBTAG00000038848	0.00E+00	1.00E+00	2.79E+00	2.10E-01	1.78E+00	4.79E-01	1.76E+00	4.97E-01	1.91E+00	8.00E-01
ENSBTAG00000021143	2.99E-02	1.00E+00	-2.65E-01	4.45E-01	-5.08E-01	6.15E-02	1.74E-01	7.22E-01	6.76E-02	1.00E+00
ENSBTAG00000007897	-3.24E-01	9.04E-01	-5.05E-01	2.38E-01	-5.77E-01	1.55E-01	-5.75E-01	1.65E-01	-1.56E-01	1.00E+00
ENSBTAG00000007888	3.36E-01	1.00E+00	-1.75E+00	2.65E-03	-3.58E-02	1.00E+00	6.00E-01	2.81E-01	6.67E-01	6.01E-01
ENSBTAG00000018239	9.53E-02	1.00E+00	-1.60E+00	1.00E-04	2.69E-01	6.72E-01	8.74E-01	1.86E-02	4.18E-01	8.00E-01
ENSBTAG00000018705	3.02E-01	1.00E+00	6.46E-01	5.09E-01	3.64E-01	8.61E-01	1.60E+00	1.00E-02	1.13E+00	4.34E-01
ENSBTAG00000015014	-3.08E-01	8.23E-01	-1.54E+00	1.92E-07	-9.29E-01	1.35E-03	-5.82E-01	6.73E-02	3.15E-01	8.00E-01
ENSBTAG00000015012	-3.98E-01	6.60E-01	-1.19E+00	1.15E-04	-1.31E+00	1.19E-05	-1.35E+00	9.15E-06	-6.42E-02	1.00E+00
ENSBTAG00000037300	0.00E+00	1.00E+00	0.00E+00	1.00E+00	-5.13E-15	1.00E+00	0.00E+00	1.00E+00	0.00E+00	1.00E+00
ENSBTAG00000047575	1.55E-01	1.00E+00	-2.88E-01	3.20E-01	-2.15E-01	4.96E-01	-1.12E-01	9.05E-01	1.93E-01	9.06E-01
ENSBTAG00000015007	1.74E-01	1.00E+00	1.65E-01	9.18E-01	1.35E-01	9.90E-01	8.24E-01	2.15E-02	5.65E-01	4.91E-01
ENSBTAG00000036858	-2.70E+00	5.76E-01	-2.70E+00	2.42E-01	-2.70E+00	1.99E-01	-2.70E+00	2.05E-01	1.87E-02	1.00E+00
ENSBTAG00000023172	1.31E-01	1.00E+00	-4.00E-01	9.18E-02	1.92E-01	5.45E-01	4.80E-01	3.61E-02	1.78E-01	9.27E-01
ENSBTAG00000000261	1.52E-01	1.00E+00	-8.78E-01	2.17E-01	-7.31E-01	2.97E-01	-1.78E+00	7.10E-03	1.45E-01	1.00E+00
ENSBTAG00000030864	-2.92E-01	8.70E-01	-1.66E+00	7.30E-08	-2.22E+00	7.07E-13	-2.60E+00	3.50E-16	-5.01E-02	1.00E+00
ENSBTAG00000003404	-5.13E-01	9.72E-01	-1.20E+00	1.03E-01	-1.22E+00	8.92E-02	-1.78E+00	1.21E-02	-9.68E-02	1.00E+00
ENSBTAG00000003405	1.39E-01	1.00E+00	1.00E+00	2.99E-03	1.30E+00	5.53E-05	7.92E-01	2.69E-02	5.85E-01	4.60E-01
ENSBTAG00000003407	9.68E-02	1.00E+00	1.47E+00	5.57E-07	1.43E+00	1.03E-06	1.02E+00	8.39E-04	-1.53E-01	1.00E+00
ENSBTAG00000003409	-1.07E-01	1.00E+00	1.32E+00	1.64E-01	2.19E+00	8.81E-03	1.29E+00	1.86E-01	-3.58E-01	1.00E+00
ENSBTAG00000016987	1.01E-01	1.00E+00	1.66E+00	1.59E-04	2.24E+00	2.18E-07	1.78E+00	5.72E-05	6.05E-02	1.00E+00
ENSBTAG00000002034	4.06E-01	9.20E-01	9.59E-01	4.57E-02	-1.39E-01	1.00E+00	-1.26E+00	8.96E-03	3.26E-01	1.00E+00
ENSBTAG00000002033	2.89E-01	8.23E-01	7.99E-01	3.10E-03	9.59E-01	2.57E-04	7.88E-01	4.10E-03	-1.12E-02	1.00E+00
ENSBTAG00000017255	-9.64E-02	1.00E+00	7.97E-01	6.07E-03	7.65E-01	8.68E-03	5.06E-01	1.25E-01	-2.34E-01	9.48E-01
ENSBTAG00000021969	-1.44E-01	1.00E+00	4.87E-01	3.48E-01	-1.67E-01	9.79E-01	-7.60E-01	1.12E-01	-3.85E-01	8.68E-01
ENSBTAG00000042219	0.00E+00	1.00E+00	0.00E+00	1.00E+00	-5.13E-15	1.00E+00	0.00E+00	1.00E+00	0.00E+00	1.00E+00
ENSBTAG00000004565	-7.38E-02	1.00E+00	1.63E-01	7.99E-01	3.10E-01	3.78E-01	2.72E-01	4.77E-01	-1.05E+00	1.57E-03
ENSBTAG00000030834	4.50E-01	1.00E+00	-2.23E-01	1.00E+00	7.60E-01	4.65E-01	7.74E-01	4.62E-01	8.88E-01	7.83E-01
ENSBTAG00000008505	-1.38E-01	1.00E+00	-1.03E+00	3.78E-04	-9.52E-01	1.12E-03	1.26E-03	1.00E+00	3.45E-01	7.87E-01
ENSBTAG00000031014	-6.33E-02	1.00E+00	-3.67E-01	3.10E-01	-1.20E-01	9.71E-01	7.03E-03	1.00E+00	3.73E-01	6.89E-01
ENSBTAG00000038224	9.82E-03	1.00E+00	6.06E-01	9.45E-01	-9.17E-01	8.09E-01	-1.65E-01	1.00E+00	-2.70E+00	6.01E-01
ENSBTAG00000015490	1.69E-01	1.00E+00	9.11E-01	1.54E-03	-5.60E-01	7.88E-02	-6.55E-01	3.67E-02	3.17E-01	8.00E-01
ENSBTAG00000043700	3.90E+00	1.42E-01	4.62E+00	3.85E-03	3.43E+00	7.44E-02	2.54E+00	2.71E-01	0.00E+00	1.00E+00
ENSBTAG00000046054	2.89E+00	7.40E-40	2.52E+00	7.12E-32	1.61E+00	2.36E-14	1.29E+00	1.41E-09	-1.17E-01	1.00E+00

ENSBTAG00000016270	4.16E+00	8.31E-02	5.41E+00	1.03E-04	4.16E+00	1.33E-02	2.53E+00	2.71E-01	3.59E+00	3.09E-01
ENSBTAG00000011155	3.29E-01	7.76E-01	5.30E-01	8.02E-02	6.57E-01	2.23E-02	6.52E-01	2.68E-02	5.56E-01	3.15E-01
ENSBTAG00000045434	0.00E+00	1.00E+00	0.00E+00	1.00E+00	-5.13E-15	1.00E+00	0.00E+00	1.00E+00	0.00E+00	1.00E+00
ENSBTAG00000011154	-6.44E-02	1.00E+00	-1.54E+00	1.35E-05	-3.36E+00	1.48E-20	-1.90E+00	8.13E-08	3.93E-01	8.00E-01
ENSBTAG00000020894	8.41E-03	1.00E+00	2.55E-02	1.00E+00	-3.54E-02	1.00E+00	1.26E-01	7.65E-01	-1.85E-01	8.41E-01
ENSBTAG00000020893	9.40E-01	9.99E-01	9.80E-01	6.16E-01	1.30E+00	4.23E-01	7.11E-01	8.18E-01	-3.62E-02	1.00E+00
ENSBTAG00000015587	3.41E-01	6.78E-01	1.04E+00	2.94E-05	1.23E+00	4.02E-07	9.51E-01	1.66E-04	3.61E-02	1.00E+00
ENSBTAG00000008113	-1.05E+00	7.38E-01	8.81E-02	1.00E+00	-1.80E-01	1.00E+00	-8.90E-01	4.24E-01	-2.59E-01	1.00E+00
ENSBTAG00000042118	0.00E+00	1.00E+00	0.00E+00	1.00E+00	-5.13E-15	1.00E+00	0.00E+00	1.00E+00	0.00E+00	1.00E+00
ENSBTAG00000005270	8.04E-01	1.00E+00	8.79E-01	8.52E-01	-1.31E-01	1.00E+00	6.21E-01	9.94E-01	-1.91E+00	8.00E-01
ENSBTAG00000042481	0.00E+00	1.00E+00	0.00E+00	1.00E+00	-5.13E-15	1.00E+00	0.00E+00	1.00E+00	0.00E+00	1.00E+00
ENSBTAG00000023928	-1.06E-01	1.00E+00	7.55E-01	4.38E-04	7.62E-01	3.50E-04	5.80E-01	1.06E-02	-1.13E-01	1.00E+00
ENSBTAG00000019212	-6.61E-01	9.04E-01	-4.75E-01	7.48E-01	4.77E-01	7.04E-01	6.82E-01	4.82E-01	2.21E-01	1.00E+00
ENSBTAG00000039113	0.00E+00	1.00E+00	0.00E+00	1.00E+00	-5.13E-15	1.00E+00	0.00E+00	1.00E+00	0.00E+00	1.00E+00
ENSBTAG00000020059	-6.75E-01	2.91E-01	-1.61E+00	8.25E-06	-1.38E+00	8.35E-05	-1.17E+00	1.16E-03	-1.48E-01	1.00E+00
ENSBTAG00000018256	1.95E-02	1.00E+00	-8.58E-01	7.54E-04	-7.13E-01	5.77E-03	-1.54E-01	8.07E-01	9.03E-03	1.00E+00
ENSBTAG00000017158	-2.90E-01	7.59E-01	-2.29E+00	3.57E-21	-3.05E+00	2.75E-34	-3.21E+00	8.00E-37	-3.70E-01	5.67E-01
ENSBTAG00000037812	2.71E+00	5.76E-01	2.79E+00	2.10E-01	1.78E+00	4.79E-01	3.41E+00	8.27E-02	0.00E+00	1.00E+00
ENSBTAG00000012779	1.79E-01	1.00E+00	-1.24E+00	1.67E-05	-1.57E+00	3.46E-08	-1.18E+00	4.88E-05	-2.58E-01	8.99E-01
ENSBTAG00000023372	1.37E-01	1.00E+00	-9.80E-01	3.84E-01	-1.23E+00	2.15E-01	2.33E-01	1.00E+00	6.56E-01	8.42E-01
ENSBTAG00000009906	-1.25E-01	1.00E+00	-6.58E-01	3.73E-03	7.97E-02	1.00E+00	2.80E-01	3.43E-01	-2.28E-01	8.07E-01
ENSBTAG00000014081	-6.58E-01	2.85E-01	-1.31E+00	8.62E-05	-7.25E-01	4.56E-02	-6.43E-01	9.32E-02	-2.62E-01	9.73E-01
ENSBTAG00000045350	0.00E+00	1.00E+00	0.00E+00	1.00E+00	-5.13E-15	1.00E+00	0.00E+00	1.00E+00	0.00E+00	1.00E+00
ENSBTAG00000045076	0.00E+00	1.00E+00	0.00E+00	1.00E+00	-5.13E-15	1.00E+00	0.00E+00	1.00E+00	0.00E+00	1.00E+00
ENSBTAG00000004948	-1.33E+00	1.56E-04	-2.73E+00	3.94E-17	-1.50E+00	1.02E-06	-1.51E+00	1.04E-06	-2.45E-01	9.66E-01
ENSBTAG00000019284	2.10E-01	9.87E-01	1.15E+00	4.73E-06	1.29E+00	1.91E-07	1.21E+00	1.50E-06	-1.35E-01	1.00E+00
ENSBTAG00000037009	0.00E+00	1.00E+00	0.00E+00	1.00E+00	1.78E+00	4.79E-01	0.00E+00	1.00E+00	0.00E+00	1.00E+00
ENSBTAG00000016045	5.48E-01	6.58E-01	1.96E+00	8.26E-07	1.85E+00	3.18E-06	1.28E+00	2.17E-03	-4.38E-01	8.00E-01
ENSBTAG00000044370	0.00E+00	1.00E+00	0.00E+00	1.00E+00	-5.13E-15	1.00E+00	0.00E+00	1.00E+00	0.00E+00	1.00E+00
ENSBTAG00000007689	1.05E+00	6.84E-02	-1.31E+00	1.42E-03	-5.13E-01	3.22E-01	-5.80E-02	1.00E+00	1.05E+00	1.14E-01
ENSBTAG00000003691	8.96E-01	1.00E+00	8.87E-02	1.00E+00	-2.70E+00	1.99E-01	3.28E-01	1.00E+00	-7.85E-01	1.00E+00
ENSBTAG00000015402	-8.52E-01	9.74E-01	2.88E-01	1.00E+00	2.61E+00	3.90E-03	9.59E-01	4.67E-01	-8.57E-01	9.37E-01
ENSBTAG00000048286	1.32E-01	1.00E+00	7.60E-01	9.99E-04	1.32E+00	7.66E-10	6.42E-01	7.38E-03	-1.52E-01	1.00E+00
ENSBTAG00000005847	2.17E-01	1.00E+00	1.08E+00	2.00E-04	7.20E-01	1.90E-02	1.33E+00	3.68E-06	2.42E-01	9.46E-01
ENSBTAG00000048065	0.00E+00	1.00E+00	0.00E+00	1.00E+00	-5.13E-15	1.00E+00	0.00E+00	1.00E+00	0.00E+00	1.00E+00

ENSBTAG00000020297	2.94E-01	1.00E+00	-4.01E-01	4.94E-01	-5.20E-02	1.00E+00	-1.64E-01	9.82E-01	-6.41E-01	5.82E-01
ENSBTAG00000001903	-1.07E+00	7.53E-01	-1.67E+00	1.29E-01	-1.92E+00	6.26E-02	-6.43E-01	6.30E-01	-4.30E-01	1.00E+00
ENSBTAG00000021283	0.00E+00	1.00E+00	1.98E+00	4.43E-01	-5.13E-15	1.00E+00	0.00E+00	1.00E+00	0.00E+00	1.00E+00
ENSBTAG00000001928	9.94E-01	5.53E-03	1.74E+00	1.26E-09	1.52E+00	9.97E-08	1.27E+00	1.31E-05	9.99E-01	1.34E-02
ENSBTAG00000001927	2.12E+00	1.89E-06	6.32E+00	3.91E-51	7.90E+00	2.49E-71	6.70E+00	3.12E-56	2.81E-01	1.00E+00
ENSBTAG00000002716	2.10E-01	8.27E-01	9.01E-01	4.24E-06	1.44E+00	4.00E-14	9.54E-01	1.17E-06	-3.87E-03	1.00E+00
ENSBTAG00000047431	-3.88E+00	1.42E-01	7.36E-01	6.34E-01	1.44E+00	1.12E-01	1.32E+00	1.76E-01	-6.60E-01	1.00E+00
ENSBTAG00000004256	2.33E-01	1.00E+00	9.18E-01	1.24E-02	2.02E+00	4.33E-09	2.11E+00	1.06E-09	-1.23E+00	1.21E-02
ENSBTAG00000004259	1.60E-01	1.00E+00	-3.52E-01	3.20E-01	-2.16E-01	6.40E-01	-2.71E-01	4.97E-01	-2.57E-01	8.65E-01
ENSBTAG00000008216	-5.07E-01	7.26E-01	-1.42E+00	5.99E-04	-9.89E-01	1.85E-02	-6.94E-01	1.35E-01	-8.12E-01	3.22E-01
ENSBTAG00000046218	-1.13E+00	5.63E-04	-3.47E+00	5.62E-32	-3.79E+00	3.10E-37	-3.05E+00	4.24E-26	-2.15E-01	9.94E-01
ENSBTAG00000007485	-9.05E-01	7.08E-01	7.34E-01	3.43E-01	2.82E+00	1.57E-08	2.47E+00	1.53E-06	3.98E-01	1.00E+00
ENSBTAG00000007543	2.24E-01	1.00E+00	2.09E-01	9.56E-01	8.20E-01	1.28E-01	5.77E-01	3.63E-01	1.57E-01	1.00E+00
ENSBTAG00000006236	0.00E+00	1.00E+00	0.00E+00	1.00E+00	-5.13E-15	1.00E+00	0.00E+00	1.00E+00	0.00E+00	1.00E+00
ENSBTAG00000002108	9.45E-02	1.00E+00	-4.15E-01	7.64E-02	-6.42E-01	2.67E-03	-8.39E-02	9.98E-01	-2.39E-02	1.00E+00
ENSBTAG00000001141	-5.88E-02	1.00E+00	1.00E+00	2.25E-06	7.51E-01	5.55E-04	7.03E-01	1.65E-03	4.45E-01	3.25E-01
ENSBTAG00000001140	-1.67E-01	1.00E+00	-7.53E-01	5.03E-02	-6.87E-01	8.08E-02	-7.56E-01	5.45E-02	-1.46E-01	1.00E+00
ENSBTAG00000019735	-1.88E-01	9.92E-01	3.82E-02	1.00E+00	3.66E-01	1.81E-01	2.88E-01	3.49E-01	-1.46E-01	1.00E+00
ENSBTAG00000013063	-1.21E-01	1.00E+00	-1.30E-01	7.84E-01	5.01E-01	1.65E-02	3.16E-01	1.95E-01	1.38E-01	1.00E+00
ENSBTAG00000002329	1.01E-01	1.00E+00	-1.09E+00	7.17E-03	-7.08E-01	1.10E-01	-4.75E-02	1.00E+00	1.45E-01	1.00E+00
ENSBTAG00000008160	-1.57E-02	1.00E+00	-9.83E-01	4.04E-01	-2.30E+00	1.29E-02	-3.00E+00	1.25E-03	4.51E-01	1.00E+00
ENSBTAG00000007963	8.97E-02	1.00E+00	-3.34E-01	2.86E-01	-3.33E-01	2.84E-01	5.58E-02	1.00E+00	2.80E-01	8.00E-01
ENSBTAG00000021187	-1.16E+00	2.46E-03	-2.30E+00	5.55E-13	-6.78E-01	5.36E-02	-6.39E-01	8.09E-02	-1.20E-01	1.00E+00
ENSBTAG00000047976	0.00E+00	1.00E+00	0.00E+00	1.00E+00	-5.13E-15	1.00E+00	0.00E+00	1.00E+00	0.00E+00	1.00E+00
ENSBTAG00000037793	0.00E+00	1.00E+00	0.00E+00	1.00E+00	-5.13E-15	1.00E+00	0.00E+00	1.00E+00	1.91E+00	8.00E-01
ENSBTAG00000047006	0.00E+00	1.00E+00	0.00E+00	1.00E+00	-5.13E-15	1.00E+00	0.00E+00	1.00E+00	0.00E+00	1.00E+00
ENSBTAG00000044064	8.91E-02	1.00E+00	-3.61E-01	7.12E-01	-6.08E-02	1.00E+00	-1.04E-01	1.00E+00	2.49E-01	1.00E+00
ENSBTAG00000016061	-9.12E-01	8.33E-01	-7.25E-01	6.24E-01	-5.39E-01	8.05E-01	-1.16E+00	3.22E-01	-9.67E-01	8.00E-01
ENSBTAG00000019979	-5.83E-01	8.87E-01	1.01E-01	1.00E+00	-1.93E-01	1.00E+00	-7.32E-01	3.82E-01	-1.50E-01	1.00E+00
ENSBTAG00000040462	0.00E+00	1.00E+00	0.00E+00	1.00E+00	-5.13E-15	1.00E+00	0.00E+00	1.00E+00	0.00E+00	1.00E+00
ENSBTAG00000037026	0.00E+00	1.00E+00	0.00E+00	1.00E+00	-5.13E-15	1.00E+00	0.00E+00	1.00E+00	0.00E+00	1.00E+00
ENSBTAG00000039201	0.00E+00	1.00E+00	1.98E+00	4.43E-01	1.78E+00	4.79E-01	1.76E+00	4.97E-01	0.00E+00	1.00E+00
ENSBTAG00000039186	1.05E-02	1.00E+00	6.04E-01	9.45E-01	3.60E-01	1.00E+00	-1.71E-01	1.00E+00	-2.70E+00	6.01E-01
ENSBTAG00000012090	-2.22E-01	9.72E-01	1.20E+00	1.21E-06	8.62E-01	7.27E-04	4.68E-01	1.18E-01	-3.88E-01	6.17E-01
ENSBTAG00000008232	-1.85E-01	1.00E+00	-8.20E-01	4.36E-03	-6.04E-01	4.61E-02	-2.10E-01	6.84E-01	4.49E-01	5.37E-01

ENSBTAG00000012827	-5.20E-01	8.04E-01	7.89E-03	1.00E+00	-2.28E-01	8.69E-01	-3.34E-01	6.63E-01	-6.66E-01	6.01E-01
ENSBTAG00000004295	-1.89E-01	1.00E+00	-5.90E-01	3.43E-02	-7.06E-01	8.56E-03	-4.11E-01	1.93E-01	-1.38E-01	1.00E+00
ENSBTAG00000004296	-6.16E-01	1.00E+00	-1.66E+00	1.27E-01	-1.00E+00	3.56E-01	-2.09E-01	1.00E+00	-2.60E-01	1.00E+00
ENSBTAG00000005525	-1.29E+00	3.49E-03	-1.53E+00	4.58E-05	-1.50E-01	9.58E-01	-6.40E-01	1.11E-01	1.05E-01	1.00E+00
ENSBTAG00000016367	6.85E-01	5.72E-02	1.31E+00	1.22E-07	9.87E-01	8.83E-05	1.10E+00	1.38E-05	3.31E-01	7.32E-01
ENSBTAG00000047226	3.66E-01	5.88E-01	9.48E-01	1.23E-04	6.07E-01	2.14E-02	6.38E-01	1.65E-02	-2.55E-01	8.33E-01
ENSBTAG00000018426	3.11E-01	7.31E-01	6.73E-01	7.12E-03	4.44E-01	1.06E-01	1.02E-01	9.84E-01	-2.61E-01	8.00E-01
ENSBTAG00000006716	-8.40E-01	5.03E-02	-2.66E+00	7.33E-17	-1.89E+00	5.78E-10	-5.97E-01	8.76E-02	-1.72E-01	1.00E+00
ENSBTAG00000047610	0.00E+00	1.00E+00	0.00E+00	1.00E+00	-5.13E-15	1.00E+00	0.00E+00	1.00E+00	0.00E+00	1.00E+00
ENSBTAG00000047693	0.00E+00	1.00E+00	0.00E+00	1.00E+00	-5.13E-15	1.00E+00	0.00E+00	1.00E+00	0.00E+00	1.00E+00
ENSBTAG00000046018	0.00E+00	1.00E+00	0.00E+00	1.00E+00	-5.13E-15	1.00E+00	0.00E+00	1.00E+00	0.00E+00	1.00E+00
ENSBTAG00000046137	0.00E+00	1.00E+00	0.00E+00	1.00E+00	-5.13E-15	1.00E+00	0.00E+00	1.00E+00	0.00E+00	1.00E+00
ENSBTAG00000038309	0.00E+00	1.00E+00	0.00E+00	1.00E+00	-5.13E-15	1.00E+00	0.00E+00	1.00E+00	0.00E+00	1.00E+00
ENSBTAG00000038796	0.00E+00	1.00E+00	0.00E+00	1.00E+00	-5.13E-15	1.00E+00	0.00E+00	1.00E+00	0.00E+00	1.00E+00
ENSBTAG00000037739	0.00E+00	1.00E+00	0.00E+00	1.00E+00	-5.13E-15	1.00E+00	0.00E+00	1.00E+00	0.00E+00	1.00E+00
ENSBTAG00000038278	1.92E+00	8.23E-01	0.00E+00	1.00E+00	-5.13E-15	1.00E+00	0.00E+00	1.00E+00	0.00E+00	1.00E+00
ENSBTAG00000039225	0.00E+00	1.00E+00	1.98E+00	4.43E-01	3.05E+00	1.36E-01	0.00E+00	1.00E+00	0.00E+00	1.00E+00
ENSBTAG00000038726	0.00E+00	1.00E+00	0.00E+00	1.00E+00	-5.13E-15	1.00E+00	0.00E+00	1.00E+00	0.00E+00	1.00E+00
ENSBTAG00000037542	0.00E+00	1.00E+00	0.00E+00	1.00E+00	1.78E+00	4.79E-01	0.00E+00	1.00E+00	0.00E+00	1.00E+00
ENSBTAG00000000726	0.00E+00	1.00E+00	0.00E+00	1.00E+00	-5.13E-15	1.00E+00	0.00E+00	1.00E+00	0.00E+00	1.00E+00
ENSBTAG00000046126	0.00E+00	1.00E+00	0.00E+00	1.00E+00	-5.13E-15	1.00E+00	0.00E+00	1.00E+00	0.00E+00	1.00E+00
ENSBTAG00000040047	0.00E+00	1.00E+00	0.00E+00	1.00E+00	-5.13E-15	1.00E+00	0.00E+00	1.00E+00	0.00E+00	1.00E+00
ENSBTAG00000037767	-1.91E+00	8.23E-01	-1.91E+00	4.69E-01	-1.91E+00	4.28E-01	-1.91E+00	4.39E-01	-1.91E+00	8.00E-01
ENSBTAG00000047728	0.00E+00	1.00E+00	0.00E+00	1.00E+00	-5.13E-15	1.00E+00	0.00E+00	1.00E+00	0.00E+00	1.00E+00
ENSBTAG00000047112	0.00E+00	1.00E+00	0.00E+00	1.00E+00	-5.13E-15	1.00E+00	0.00E+00	1.00E+00	0.00E+00	1.00E+00
ENSBTAG00000046536	0.00E+00	1.00E+00	0.00E+00	1.00E+00	-5.13E-15	1.00E+00	0.00E+00	1.00E+00	0.00E+00	1.00E+00
ENSBTAG00000045527	0.00E+00	1.00E+00	0.00E+00	1.00E+00	-5.13E-15	1.00E+00	0.00E+00	1.00E+00	0.00E+00	1.00E+00
ENSBTAG00000045545	0.00E+00	1.00E+00	0.00E+00	1.00E+00	-5.13E-15	1.00E+00	0.00E+00	1.00E+00	0.00E+00	1.00E+00
ENSBTAG00000020660	0.00E+00	1.00E+00	0.00E+00	1.00E+00	-5.13E-15	1.00E+00	1.77E+00	4.97E-01	0.00E+00	1.00E+00
ENSBTAG00000038551	0.00E+00	1.00E+00	0.00E+00	1.00E+00	-5.13E-15	1.00E+00	0.00E+00	1.00E+00	0.00E+00	1.00E+00
ENSBTAG00000046221	0.00E+00	1.00E+00	0.00E+00	1.00E+00	-5.13E-15	1.00E+00	0.00E+00	1.00E+00	0.00E+00	1.00E+00
ENSBTAG00000045528	0.00E+00	1.00E+00	0.00E+00	1.00E+00	-5.13E-15	1.00E+00	0.00E+00	1.00E+00	0.00E+00	1.00E+00
ENSBTAG00000001549	0.00E+00	1.00E+00	0.00E+00	1.00E+00	-5.13E-15	1.00E+00	0.00E+00	1.00E+00	0.00E+00	1.00E+00
ENSBTAG00000048218	0.00E+00	1.00E+00	0.00E+00	1.00E+00	-5.13E-15	1.00E+00	0.00E+00	1.00E+00	0.00E+00	1.00E+00
ENSBTAG00000038822	0.00E+00	1.00E+00	0.00E+00	1.00E+00	-5.13E-15	1.00E+00	0.00E+00	1.00E+00	0.00E+00	1.00E+00

ENSBTAG00000038941	0.00E+00	1.00E+00	0.00E+00	1.00E+00	-5.13E-15	1.00E+00	0.00E+00	1.00E+00	0.00E+00	1.00E+00
ENSBTAG00000037822	0.00E+00	1.00E+00	1.99E+00	4.43E-01	1.78E+00	4.79E-01	0.00E+00	1.00E+00	0.00E+00	1.00E+00
ENSBTAG00000038665	0.00E+00	1.00E+00	0.00E+00	1.00E+00	-5.13E-15	1.00E+00	0.00E+00	1.00E+00	0.00E+00	1.00E+00
ENSBTAG00000004667	0.00E+00	1.00E+00	0.00E+00	1.00E+00	-5.13E-15	1.00E+00	0.00E+00	1.00E+00	0.00E+00	1.00E+00
ENSBTAG00000030678	-2.62E-01	1.00E+00	-1.97E-01	9.17E-01	-1.56E-01	1.00E+00	-1.70E-03	1.00E+00	-1.24E+00	4.42E-02
ENSBTAG00000030677	0.00E+00	1.00E+00	0.00E+00	1.00E+00	-5.13E-15	1.00E+00	0.00E+00	1.00E+00	0.00E+00	1.00E+00
ENSBTAG00000038249	0.00E+00	1.00E+00	0.00E+00	1.00E+00	-5.13E-15	1.00E+00	0.00E+00	1.00E+00	0.00E+00	1.00E+00
ENSBTAG00000030676	-1.91E+00	8.23E-01	-1.91E+00	4.69E-01	-1.91E+00	4.28E-01	-1.91E+00	4.39E-01	-1.91E+00	8.00E-01
ENSBTAG00000030675	3.05E-01	8.04E-01	1.09E+00	1.83E-05	1.23E+00	7.85E-07	1.13E+00	9.10E-06	-4.08E-02	1.00E+00
ENSBTAG00000023867	2.07E-01	1.00E+00	2.23E-01	7.48E-01	6.51E-01	7.10E-02	7.80E-01	2.68E-02	1.04E-01	1.00E+00
ENSBTAG00000042329	0.00E+00	1.00E+00	0.00E+00	1.00E+00	-5.13E-15	1.00E+00	0.00E+00	1.00E+00	0.00E+00	1.00E+00
ENSBTAG00000039815	7.41E-01	2.26E-01	1.46E+00	2.51E-05	7.68E-01	4.43E-02	8.30E-01	3.03E-02	6.53E-01	4.29E-01
ENSBTAG00000038610	9.12E-02	1.00E+00	-4.68E-01	3.28E-01	-2.81E-01	6.52E-01	-4.12E-01	4.23E-01	3.97E-01	8.00E-01
ENSBTAG00000039172	1.12E-01	1.00E+00	2.87E-01	3.98E-01	4.27E-01	1.30E-01	6.33E-01	1.45E-02	2.07E-01	9.36E-01
ENSBTAG00000040296	0.00E+00	1.00E+00	0.00E+00	1.00E+00	-5.13E-15	1.00E+00	0.00E+00	1.00E+00	0.00E+00	1.00E+00
ENSBTAG00000024604	6.01E-02	1.00E+00	-4.15E-01	1.52E-01	-1.75E+00	3.66E-13	-2.09E+00	7.97E-18	1.52E-01	1.00E+00
ENSBTAG00000021921	4.13E-02	1.00E+00	-1.80E-01	6.97E-01	-4.09E-01	1.51E-01	1.54E-02	1.00E+00	4.04E-01	5.03E-01
ENSBTAG00000042085	0.00E+00	1.00E+00	0.00E+00	1.00E+00	-5.13E-15	1.00E+00	0.00E+00	1.00E+00	0.00E+00	1.00E+00
ENSBTAG00000014391	1.06E+00	7.28E-01	7.98E-01	5.45E-01	5.29E-01	8.06E-01	2.28E+00	1.86E-03	1.68E+00	2.66E-01
ENSBTAG00000003610	-7.13E-02	1.00E+00	-2.13E-01	5.25E-01	-3.53E-01	1.91E-01	-3.58E-01	1.92E-01	2.49E-01	8.00E-01
ENSBTAG00000044885	0.00E+00	1.00E+00	0.00E+00	1.00E+00	-5.13E-15	1.00E+00	0.00E+00	1.00E+00	0.00E+00	1.00E+00
ENSBTAG00000023860	0.00E+00	1.00E+00	0.00E+00	1.00E+00	-5.13E-15	1.00E+00	0.00E+00	1.00E+00	0.00E+00	1.00E+00
ENSBTAG00000010990	-2.93E-01	9.24E-01	-1.01E+00	2.49E-03	-8.29E-01	1.43E-02	-6.48E-01	7.51E-02	1.29E-01	1.00E+00
ENSBTAG00000019470	9.93E-02	1.00E+00	-2.80E-01	4.71E-01	5.25E-01	8.94E-02	5.27E-01	9.49E-02	-2.67E-01	8.47E-01
ENSBTAG00000003067	-1.35E-01	1.00E+00	-3.78E-01	2.83E-01	-7.79E-01	6.79E-03	-6.18E-01	4.53E-02	-3.09E-01	8.00E-01
ENSBTAG00000017576	-1.98E+00	5.76E-01	3.33E-01	1.00E+00	-2.11E+00	1.72E-01	-1.37E+00	4.08E-01	-2.98E-01	1.00E+00
ENSBTAG00000009017	1.57E-03	1.00E+00	2.59E-01	1.00E+00	-3.86E-01	9.78E-01	-9.44E-01	5.19E-01	-1.12E+00	8.00E-01
ENSBTAG00000040585	2.59E-01	1.00E+00	-6.99E-01	1.06E-01	-1.51E-01	9.87E-01	5.25E-01	2.59E-01	7.21E-01	3.56E-01
ENSBTAG00000029841	-5.15E-01	1.00E+00	-3.22E+00	1.25E-01	-3.22E+00	9.40E-02	-1.46E+00	4.92E-01	-3.22E+00	4.34E-01
ENSBTAG00000029896	0.00E+00	1.00E+00	0.00E+00	1.00E+00	-5.13E-15	1.00E+00	0.00E+00	1.00E+00	1.93E+00	8.00E-01
ENSBTAG00000004848	-6.21E-01	6.34E-01	-2.41E+00	7.62E-06	-1.42E+00	3.03E-03	-1.26E+00	1.00E-02	5.63E-01	6.55E-01
ENSBTAG00000044811	0.00E+00	1.00E+00	0.00E+00	1.00E+00	-5.13E-15	1.00E+00	0.00E+00	1.00E+00	0.00E+00	1.00E+00
ENSBTAG00000039223	-1.07E+00	7.56E-01	5.18E-01	6.93E-01	-8.04E-01	4.79E-01	-1.05E+00	3.44E-01	-6.46E-01	9.64E-01
ENSBTAG00000003205	1.27E-01	1.00E+00	4.77E-01	1.43E-01	2.08E-01	6.84E-01	-1.30E-01	9.43E-01	5.62E-02	1.00E+00
ENSBTAG00000015278	6.65E-02	1.00E+00	7.41E-01	1.05E-03	1.17E+00	6.37E-08	8.17E-01	3.01E-04	-2.96E-01	7.19E-01

ENSBTAG00000015286	5.10E-02	1.00E+00	7.99E-01	6.54E-04	5.72E-01	2.04E-02	1.72E-01	7.04E-01	2.02E-02	1.00E+00
ENSBTAG00000015553	4.83E-02	1.00E+00	-1.93E+00	2.59E-04	-6.98E-01	1.86E-01	-1.01E-01	1.00E+00	-4.23E-01	8.56E-01
ENSBTAG00000045071	0.00E+00	1.00E+00	0.00E+00	1.00E+00	-5.13E-15	1.00E+00	0.00E+00	1.00E+00	0.00E+00	1.00E+00
ENSBTAG00000008033	5.63E-02	1.00E+00	2.82E-01	2.65E-01	2.83E-01	2.59E-01	3.31E-01	1.74E-01	-3.20E-02	1.00E+00
ENSBTAG00000015098	-1.70E-01	1.00E+00	-9.11E-01	8.52E-03	-4.48E-01	2.80E-01	-1.85E-01	8.54E-01	-1.77E-01	1.00E+00
ENSBTAG00000007662	2.46E+00	2.40E-09	5.29E+00	8.93E-35	4.15E+00	3.05E-24	3.01E+00	2.05E-14	7.00E-01	4.34E-01
ENSBTAG00000011544	-9.33E-02	1.00E+00	1.04E-01	8.69E-01	5.19E-02	1.00E+00	1.71E-01	5.68E-01	1.49E-01	9.77E-01
ENSBTAG00000043246	0.00E+00	1.00E+00	0.00E+00	1.00E+00	-5.13E-15	1.00E+00	0.00E+00	1.00E+00	0.00E+00	1.00E+00
ENSBTAG00000048297	0.00E+00	1.00E+00	0.00E+00	1.00E+00	-5.13E-15	1.00E+00	0.00E+00	1.00E+00	0.00E+00	1.00E+00
ENSBTAG00000010271	2.24E-01	7.71E-01	7.89E-01	1.01E-05	8.19E-01	3.80E-06	3.71E-01	7.25E-02	1.92E-01	8.01E-01
ENSBTAG00000013314	2.25E-01	1.00E+00	1.96E-01	8.95E-01	-5.25E-02	1.00E+00	-6.88E-01	1.48E-01	-1.56E-01	1.00E+00
ENSBTAG00000044315	0.00E+00	1.00E+00	0.00E+00	1.00E+00	-5.13E-15	1.00E+00	0.00E+00	1.00E+00	0.00E+00	1.00E+00
ENSBTAG00000010228	-2.71E+00	5.76E-01	8.36E-02	1.00E+00	-1.53E-01	1.00E+00	-2.71E+00	2.05E-01	-7.80E-01	1.00E+00
ENSBTAG00000003438	3.91E-01	8.23E-01	2.09E+00	7.46E-09	2.06E+00	1.18E-08	1.45E+00	9.94E-05	7.40E-01	3.44E-01
ENSBTAG00000039343	4.18E-01	8.23E-01	5.89E-01	1.82E-01	-3.42E-01	5.51E-01	-4.55E-01	3.81E-01	6.09E-01	5.16E-01
ENSBTAG00000023843	-2.81E-02	1.00E+00	-1.32E-01	9.20E-01	-5.64E-01	5.69E-02	-8.60E-01	2.22E-03	7.50E-02	1.00E+00
ENSBTAG00000011810	0.00E+00	1.00E+00	0.00E+00	1.00E+00	-5.13E-15	1.00E+00	0.00E+00	1.00E+00	0.00E+00	1.00E+00
ENSBTAG00000006948	-8.63E-01	3.15E-01	1.38E-02	1.00E+00	6.01E-01	2.07E-01	-8.32E-03	1.00E+00	4.43E-01	8.00E-01
ENSBTAG00000043520	0.00E+00	1.00E+00	0.00E+00	1.00E+00	-5.13E-15	1.00E+00	0.00E+00	1.00E+00	0.00E+00	1.00E+00
ENSBTAG00000006957	3.80E-01	8.98E-01	1.99E+00	2.98E-07	2.58E+00	1.89E-11	2.60E+00	2.05E-11	2.82E-01	1.00E+00
ENSBTAG00000023885	5.65E-01	2.55E-01	1.61E+00	1.15E-09	1.51E+00	1.13E-08	7.71E-01	8.12E-03	1.17E-01	1.00E+00
ENSBTAG00000006963	3.26E-01	8.23E-01	1.48E+00	6.34E-08	1.70E+00	3.16E-10	7.31E-01	1.61E-02	-6.73E-01	2.36E-01
ENSBTAG00000043047	0.00E+00	1.00E+00	0.00E+00	1.00E+00	-5.13E-15	1.00E+00	0.00E+00	1.00E+00	0.00E+00	1.00E+00
ENSBTAG00000012844	2.05E-02	1.00E+00	-3.05E-01	3.71E-01	-2.75E-01	4.29E-01	5.56E-02	1.00E+00	1.08E-01	1.00E+00
ENSBTAG00000012847	-1.66E-01	1.00E+00	-1.25E+00	9.16E-05	-5.11E-01	1.59E-01	3.90E-01	3.29E-01	-8.16E-02	1.00E+00
ENSBTAG00000003525	1.71E-01	1.00E+00	8.80E-01	5.86E-05	8.86E-01	4.75E-05	2.93E-01	3.14E-01	-1.27E-01	1.00E+00
ENSBTAG00000023823	0.00E+00	1.00E+00	0.00E+00	1.00E+00	-5.13E-15	1.00E+00	0.00E+00	1.00E+00	0.00E+00	1.00E+00
ENSBTAG00000012735	-6.33E-02	1.00E+00	2.25E+00	1.81E-14	2.83E+00	6.71E-23	2.17E+00	1.26E-13	-3.01E-01	9.23E-01
ENSBTAG00000012736	2.01E-01	1.00E+00	9.98E-01	2.13E-03	1.81E+00	8.36E-10	2.02E+00	6.13E-12	-6.53E-01	4.34E-01
ENSBTAG00000013509	-1.69E-01	1.00E+00	8.49E-02	1.00E+00	-2.01E-02	1.00E+00	3.57E-01	9.43E-01	8.91E-01	7.80E-01
ENSBTAG00000013510	1.77E-01	1.00E+00	-1.42E+00	1.43E-04	4.85E-01	2.06E-01	1.29E+00	2.24E-05	7.99E-01	1.36E-01
ENSBTAG00000015375	9.97E-01	1.41E-04	1.43E+00	2.99E-10	9.73E-01	2.63E-05	2.63E-01	4.39E-01	-1.66E-01	1.00E+00
ENSBTAG00000047785	6.27E-01	9.08E-01	-1.28E+00	2.43E-01	-7.85E-01	4.87E-01	-6.88E-02	1.00E+00	2.47E-01	1.00E+00
ENSBTAG00000004695	-7.98E-02	1.00E+00	-6.47E-02	1.00E+00	3.25E-01	1.48E-01	1.39E-01	7.17E-01	1.52E-01	9.70E-01
ENSBTAG00000008994	-1.64E-01	1.00E+00	-1.47E+00	1.38E-10	-1.66E+00	3.04E-13	-7.89E-01	1.06E-03	-2.47E-01	8.00E-01

ENSBTAG00000046303	-2.61E-01	9.05E-01	-1.26E+00	4.70E-06	-1.13E+00	4.30E-05	-9.40E-01	1.00E-03	-4.45E-01	5.52E-01
ENSBTAG00000008997	-1.91E-01	1.00E+00	-1.04E+00	3.59E-04	-8.27E-01	5.35E-03	-5.00E-01	1.42E-01	-3.11E-01	8.00E-01
ENSBTAG00000006305	7.51E-02	1.00E+00	-1.34E+00	7.02E-04	-1.70E+00	1.17E-05	-2.14E+00	9.50E-08	5.79E-01	5.03E-01
ENSBTAG00000044129	-2.70E-01	7.50E-01	-1.37E+00	5.12E-11	-2.60E+00	3.48E-34	-2.24E+00	3.63E-26	5.90E-01	7.92E-02
ENSBTAG00000046548	-2.21E-01	9.52E-01	-9.17E-01	5.69E-04	2.29E-01	5.47E-01	2.32E-01	5.49E-01	-6.04E-01	2.05E-01
ENSBTAG00000003387	6.95E-01	1.00E+00	3.61E+00	1.73E-06	1.44E+00	2.28E-01	9.35E-01	5.72E-01	-1.29E+00	9.04E-01
ENSBTAG00000003388	1.84E-01	1.00E+00	6.85E-01	9.13E-03	9.56E-01	1.34E-04	9.94E-01	8.50E-05	-3.92E-02	1.00E+00
ENSBTAG00000023851	-6.79E-01	7.07E-01	-1.65E+00	2.31E-03	-5.21E-01	4.79E-01	5.55E-02	1.00E+00	7.82E-01	6.01E-01
ENSBTAG00000012156	-5.18E-01	4.37E-01	1.63E-01	8.71E-01	-2.15E-01	7.10E-01	-4.03E-01	3.07E-01	-6.74E-02	1.00E+00
ENSBTAG00000011912	-1.11E+00	9.88E-04	-2.03E+00	7.94E-13	-9.30E-01	1.53E-03	-7.83E-01	1.07E-02	-1.08E-01	1.00E+00
ENSBTAG00000016218	-9.13E-02	1.00E+00	2.02E-02	1.00E+00	5.28E-01	1.12E-01	7.62E-01	1.43E-02	-1.79E-01	1.00E+00
ENSBTAG00000014149	6.27E-01	8.23E-01	9.41E-01	1.64E-01	1.10E+00	8.67E-02	1.34E+00	3.38E-02	2.33E+00	1.93E-03
ENSBTAG00000014150	1.93E-02	1.00E+00	3.10E-01	4.50E-01	2.80E-01	5.09E-01	3.87E-01	3.07E-01	6.26E-01	2.75E-01
ENSBTAG00000012274	-2.23E-01	8.23E-01	-7.80E-01	2.43E-04	-3.10E-01	2.31E-01	-2.87E-01	2.94E-01	1.52E-01	1.00E+00
ENSBTAG00000011307	2.32E-01	1.00E+00	-1.00E+00	7.28E-03	-1.52E+00	2.58E-05	-1.50E+00	3.74E-05	-6.94E-02	1.00E+00
ENSBTAG00000029974	0.00E+00	1.00E+00	0.00E+00	1.00E+00	-5.13E-15	1.00E+00	0.00E+00	1.00E+00	0.00E+00	1.00E+00
ENSBTAG00000011317	3.46E-01	6.19E-01	1.06E+00	1.03E-05	1.15E+00	1.39E-06	1.04E+00	1.90E-05	2.56E-01	8.00E-01
ENSBTAG00000030608	1.94E-01	9.86E-01	5.31E-01	3.61E-02	3.71E-01	1.87E-01	1.04E-01	9.64E-01	4.64E-02	1.00E+00
ENSBTAG00000004663	-1.18E-01	1.00E+00	-4.54E-01	2.24E-01	4.36E-01	2.36E-01	8.13E-01	9.89E-03	2.56E-01	9.24E-01
ENSBTAG00000004664	-3.82E-01	7.67E-01	-8.15E-01	1.23E-02	-1.46E+00	2.00E-06	-1.47E+00	2.19E-06	-5.63E-02	1.00E+00
ENSBTAG00000015436	-3.97E-01	8.20E-01	-9.95E-01	5.11E-03	-2.15E+00	2.35E-10	-1.28E+00	2.65E-04	-1.20E-01	1.00E+00
ENSBTAG00000025612	-1.15E-02	1.00E+00	5.80E-01	2.13E-02	1.09E+00	1.99E-06	1.36E+00	3.34E-09	1.13E-02	1.00E+00
ENSBTAG00000030117	0.00E+00	1.00E+00	1.98E+00	4.43E-01	-5.13E-15	1.00E+00	0.00E+00	1.00E+00	1.91E+00	8.00E-01
ENSBTAG00000039842	0.00E+00	1.00E+00	0.00E+00	1.00E+00	-5.13E-15	1.00E+00	0.00E+00	1.00E+00	0.00E+00	1.00E+00
ENSBTAG00000044796	1.92E+00	8.23E-01	0.00E+00	1.00E+00	2.55E+00	2.55E-01	0.00E+00	1.00E+00	1.91E+00	8.00E-01
ENSBTAG00000038139	-1.48E-01	1.00E+00	-1.11E+00	4.83E-02	-1.30E+00	1.44E-02	-1.27E+00	1.86E-02	1.63E-01	1.00E+00
ENSBTAG00000030567	1.40E-01	1.00E+00	-3.87E-01	1.87E-01	4.34E-01	1.22E-01	5.37E-01	4.72E-02	3.59E-01	6.03E-01
ENSBTAG00000030566	-1.15E-01	1.00E+00	1.41E-01	8.91E-01	3.48E-01	3.16E-01	2.58E-01	5.24E-01	2.05E-01	9.91E-01
ENSBTAG00000015327	1.17E-01	1.00E+00	-9.98E-01	4.35E-04	-1.61E+00	5.27E-09	-6.13E-01	5.10E-02	-6.72E-03	1.00E+00
ENSBTAG00000015343	1.60E-01	1.00E+00	-4.72E-01	9.24E-02	-2.76E-01	4.19E-01	1.14E-01	9.41E-01	2.94E-01	7.79E-01
ENSBTAG00000020959	3.77E-01	4.30E-01	6.88E-01	2.00E-03	1.05E+00	7.32E-07	9.18E-01	2.40E-05	-2.23E-01	8.27E-01
ENSBTAG00000010195	-9.90E-02	1.00E+00	4.06E-01	2.47E-01	7.51E-01	1.01E-02	3.99E-01	2.68E-01	1.19E-01	1.00E+00
ENSBTAG00000030540	-1.51E-01	1.00E+00	-8.36E-01	9.79E-04	-4.34E-01	1.26E-01	-3.80E-02	1.00E+00	4.08E-01	5.00E-01
ENSBTAG00000010208	-3.73E-01	7.86E-01	-1.23E+00	9.27E-05	-4.51E-01	2.42E-01	-9.19E-02	1.00E+00	4.90E-01	5.67E-01
ENSBTAG00000043227	-1.16E+00	8.75E-01	-1.91E-01	1.00E+00	2.92E-01	1.00E+00	2.76E-01	1.00E+00	-2.76E-01	1.00E+00

ENSBTAG00000012480	4.26E-02	1.00E+00	-9.98E-01	2.94E-06	-5.47E-01	1.52E-02	-4.02E-01	1.05E-01	2.72E-01	7.37E-01
ENSBTAG00000012484	-1.97E-01	1.00E+00	-5.87E-01	7.83E-02	-1.25E+00	2.28E-05	-1.16E+00	1.30E-04	-1.72E-01	1.00E+00
ENSBTAG00000012485	-2.22E-01	9.70E-01	-5.35E-01	6.93E-02	-7.18E-01	9.41E-03	-6.81E-01	1.66E-02	-2.65E-01	8.33E-01
ENSBTAG00000036101	-3.49E-01	1.00E+00	-1.57E+00	2.75E-03	-2.97E+00	1.03E-08	-3.31E+00	3.66E-10	-5.40E-02	1.00E+00
ENSBTAG00000036099	0.00E+00	1.00E+00	0.00E+00	1.00E+00	-5.13E-15	1.00E+00	0.00E+00	1.00E+00	0.00E+00	1.00E+00
ENSBTAG00000013030	3.18E-01	8.84E-01	2.75E-01	6.13E-01	3.78E-01	4.05E-01	7.32E-01	4.43E-02	3.11E-01	8.69E-01
ENSBTAG00000038831	-6.62E-02	1.00E+00	-8.94E-01	3.58E-06	-1.58E+00	5.09E-17	-1.92E+00	6.47E-24	-1.01E-01	1.00E+00
ENSBTAG00000010601	-3.39E-01	6.76E-01	-1.15E+00	4.36E-06	-2.60E-01	4.68E-01	2.63E-01	4.71E-01	8.77E-02	1.00E+00
ENSBTAG00000010613	-1.54E-01	1.00E+00	-1.27E-01	8.20E-01	4.89E-01	2.80E-02	6.21E-01	4.13E-03	8.22E-02	1.00E+00
ENSBTAG00000010626	-1.51E-01	1.00E+00	-1.05E+00	3.14E-07	-1.80E+00	1.18E-18	-1.54E+00	4.07E-14	3.55E-01	4.94E-01
ENSBTAG00000010653	-5.09E-01	2.26E-01	-7.61E-01	2.11E-03	2.39E-01	4.84E-01	5.02E-01	6.33E-02	1.75E-01	1.00E+00
ENSBTAG00000017922	-9.50E-01	5.47E-05	-1.86E+00	5.00E-19	-1.98E+00	1.94E-21	-1.06E+00	4.64E-07	2.66E-01	7.63E-01
ENSBTAG00000001932	-6.45E-01	4.34E-02	-1.66E+00	1.74E-13	-1.51E+00	1.76E-11	-5.15E-01	4.52E-02	4.45E-01	3.84E-01
ENSBTAG00000001933	-2.17E-01	9.89E-01	-1.19E+00	6.57E-06	-7.08E-01	1.10E-02	-6.77E-04	1.00E+00	-1.54E-01	1.00E+00
ENSBTAG00000030521	5.40E-01	5.27E-01	1.32E+00	5.93E-05	9.20E-01	7.62E-03	7.21E-01	5.43E-02	-2.99E-02	1.00E+00
ENSBTAG00000019513	-8.91E-01	3.78E-01	-4.18E+00	3.51E-08	-2.62E+00	7.70E-06	-1.85E+00	8.27E-04	-2.75E-01	1.00E+00
ENSBTAG00000015437	-2.43E-01	1.00E+00	8.25E-01	9.09E-03	1.78E+00	1.05E-09	1.71E+00	6.46E-09	-1.47E-01	1.00E+00
ENSBTAG00000038183	-9.68E-01	5.78E-01	8.48E-01	2.01E-01	1.32E+00	1.58E-02	-1.24E-01	1.00E+00	-1.53E+00	2.73E-01
ENSBTAG00000008973	-1.65E-01	1.00E+00	4.00E-01	1.10E-01	7.45E-01	5.81E-04	4.71E-01	5.20E-02	-1.28E-02	1.00E+00
ENSBTAG00000002936	3.02E-01	1.00E+00	1.04E+00	2.10E-01	-1.57E-01	1.00E+00	5.97E-02	1.00E+00	-5.49E-02	1.00E+00
ENSBTAG00000019453	1.10E+00	3.91E-01	2.33E+00	2.23E-04	7.77E-01	3.35E-01	1.18E+00	9.84E-02	2.67E-01	1.00E+00
ENSBTAG00000002837	-2.05E-02	1.00E+00	6.14E-01	3.50E-02	1.07E+00	5.01E-05	9.33E-01	6.31E-04	5.19E-01	3.84E-01
ENSBTAG00000020305	1.66E-01	1.00E+00	4.40E-01	5.61E-02	8.27E-01	6.29E-05	7.51E-01	4.03E-04	2.12E-01	8.28E-01
ENSBTAG00000020307	1.23E-01	1.00E+00	2.63E-01	2.65E-01	4.21E-01	3.78E-02	4.75E-01	1.82E-02	-6.09E-02	1.00E+00
ENSBTAG00000020311	-1.94E-02	1.00E+00	-9.03E-01	4.72E-05	-9.50E-01	1.56E-05	-8.59E-01	1.33E-04	2.78E-01	7.59E-01
ENSBTAG00000047907	0.00E+00	1.00E+00	0.00E+00	1.00E+00	-5.13E-15	1.00E+00	0.00E+00	1.00E+00	0.00E+00	1.00E+00
ENSBTAG00000020313	-2.56E-01	7.50E-01	-2.34E+00	2.51E-31	-3.05E+00	6.11E-50	-2.14E+00	3.28E-27	-5.87E-03	1.00E+00
ENSBTAG00000004204	-8.07E-02	1.00E+00	1.99E-01	8.57E-01	2.65E-01	6.81E-01	1.10E+00	2.66E-03	7.35E-01	3.09E-01
ENSBTAG00000008726	2.65E-01	1.00E+00	9.66E-01	5.74E-02	1.58E+00	7.89E-04	1.44E+00	2.91E-03	6.37E-01	6.61E-01
ENSBTAG00000002319	-2.17E-01	1.00E+00	-1.71E+00	3.45E-09	-2.01E+00	2.33E-12	-1.65E+00	5.40E-09	-1.02E-01	1.00E+00
ENSBTAG00000020747	-2.14E-02	1.00E+00	-3.24E-01	2.77E-01	-9.17E-01	9.40E-05	-1.08E+00	4.10E-06	2.08E-02	1.00E+00
ENSBTAG00000013072	8.55E-02	1.00E+00	-1.10E-01	1.00E+00	5.07E-02	1.00E+00	-4.80E-02	1.00E+00	3.32E-01	8.94E-01
ENSBTAG00000043722	0.00E+00	1.00E+00	0.00E+00	1.00E+00	-5.13E-15	1.00E+00	0.00E+00	1.00E+00	0.00E+00	1.00E+00
ENSBTAG00000023797	1.51E-03	1.00E+00	4.00E-01	8.28E-01	5.39E-01	6.03E-01	-4.53E-01	8.04E-01	-1.51E+00	4.73E-01
ENSBTAG00000048071	-2.41E-01	8.25E-01	8.48E-01	1.53E-04	1.09E+00	4.70E-07	4.11E-01	1.19E-01	1.00E-01	1.00E+00

ENSBTAG00000017976	-5.17E-02	1.00E+00	1.77E-01	7.69E-01	3.62E-01	2.85E-01	5.45E-01	7.08E-02	3.55E-01	7.03E-01
ENSBTAG00000017980	0.00E+00	1.00E+00	0.00E+00	1.00E+00	-5.13E-15	1.00E+00	0.00E+00	1.00E+00	0.00E+00	1.00E+00
ENSBTAG00000017982	-1.24E+00	6.85E-01	-1.14E+00	3.57E-01	-1.78E+00	1.06E-01	4.55E-02	1.00E+00	-9.46E-01	8.00E-01
ENSBTAG00000017473	-5.25E-01	9.44E-01	-1.71E+00	2.27E-02	-1.63E+00	2.12E-02	-6.23E-01	4.67E-01	7.05E-01	6.95E-01
ENSBTAG00000046484	1.05E+00	1.45E-01	1.27E+00	7.79E-03	1.14E+00	1.86E-02	1.42E+00	2.82E-03	1.70E+00	5.55E-03
ENSBTAG00000021896	-1.09E-01	1.00E+00	2.95E-01	2.78E-01	8.71E-01	3.70E-05	6.92E-01	1.74E-03	2.31E-01	8.00E-01
ENSBTAG00000045249	0.00E+00	1.00E+00	0.00E+00	1.00E+00	-5.13E-15	1.00E+00	0.00E+00	1.00E+00	0.00E+00	1.00E+00
ENSBTAG00000004542	-2.80E-02	1.00E+00	-1.21E+00	1.53E-03	-1.88E-01	8.58E-01	-8.05E-01	3.98E-02	-3.70E-01	8.00E-01
ENSBTAG00000021205	-8.48E-02	1.00E+00	-9.31E-01	3.43E-02	-1.14E+00	6.88E-03	-8.40E-01	6.00E-02	1.44E-01	1.00E+00
ENSBTAG00000004400	1.38E-01	1.00E+00	4.52E-01	7.67E-02	8.34E-01	2.46E-04	5.51E-01	2.65E-02	4.90E-01	2.83E-01
ENSBTAG00000043373	0.00E+00	1.00E+00	0.00E+00	1.00E+00	-5.13E-15	1.00E+00	0.00E+00	1.00E+00	0.00E+00	1.00E+00
ENSBTAG00000042350	0.00E+00	1.00E+00	0.00E+00	1.00E+00	-5.13E-15	1.00E+00	0.00E+00	1.00E+00	0.00E+00	1.00E+00
ENSBTAG00000010805	-5.67E-02	1.00E+00	-5.96E-01	1.09E-02	-7.87E-01	4.42E-04	-8.92E-01	7.06E-05	1.55E-01	1.00E+00
ENSBTAG00000010810	-4.04E-01	3.95E-01	2.11E-01	5.37E-01	9.62E-01	1.17E-05	8.43E-01	1.91E-04	8.53E-02	1.00E+00
ENSBTAG00000020791	1.31E-01	1.00E+00	6.82E-02	1.00E+00	-1.58E-01	6.19E-01	-1.52E-01	6.54E-01	2.22E-01	8.00E-01
ENSBTAG00000000382	2.02E-01	8.82E-01	5.60E-01	9.27E-03	8.21E-01	4.97E-05	9.13E-01	6.72E-06	-1.60E-01	9.91E-01
ENSBTAG00000007235	-2.06E-01	1.00E+00	-4.91E-01	3.63E-01	-9.38E-01	3.20E-02	-1.63E+00	1.37E-04	-5.28E-01	7.13E-01
ENSBTAG00000024822	2.09E-01	9.66E-01	1.52E+00	2.09E-10	1.36E+00	1.26E-08	7.55E-01	3.32E-03	2.79E-01	8.00E-01
ENSBTAG00000018710	1.61E-01	1.00E+00	4.68E-01	6.20E-02	4.31E-01	9.25E-02	-3.14E-01	2.87E-01	2.02E-01	9.04E-01
ENSBTAG00000023697	0.00E+00	1.00E+00	0.00E+00	1.00E+00	-5.13E-15	1.00E+00	0.00E+00	1.00E+00	0.00E+00	1.00E+00
ENSBTAG00000019254	1.24E+00	3.82E-01	8.17E-01	4.36E-01	9.38E-02	1.00E+00	1.24E+00	1.21E-01	8.81E-01	7.77E-01
ENSBTAG00000023801	-3.21E+00	3.75E-01	-1.23E+00	6.11E-01	-6.56E-01	8.96E-01	-3.21E+00	9.91E-02	1.12E-02	1.00E+00
ENSBTAG00000003530	3.69E-02	1.00E+00	-4.10E-01	8.56E-02	-1.58E-01	6.84E-01	1.74E-01	6.35E-01	7.28E-02	1.00E+00
ENSBTAG00000004091	7.01E-01	4.58E-01	1.37E-01	1.00E+00	7.95E-01	9.07E-02	9.95E-01	2.75E-02	9.07E-01	2.68E-01
ENSBTAG00000004092	4.59E-02	1.00E+00	-4.98E-01	9.11E-01	-7.30E-01	6.96E-01	-4.47E-01	9.56E-01	3.58E-01	1.00E+00
ENSBTAG00000005189	-9.24E-01	9.51E-01	-4.13E+00	1.96E-02	-7.07E-01	7.19E-01	-4.28E-01	9.61E-01	-7.46E-03	1.00E+00
ENSBTAG00000005190	1.04E-01	1.00E+00	-2.03E-01	6.62E-01	-6.47E-02	1.00E+00	1.41E-01	8.74E-01	3.51E-01	6.80E-01
ENSBTAG00000012772	0.00E+00	1.00E+00	0.00E+00	1.00E+00	-5.13E-15	1.00E+00	0.00E+00	1.00E+00	0.00E+00	1.00E+00
ENSBTAG00000021373	0.00E+00	1.00E+00	0.00E+00	1.00E+00	-5.13E-15	1.00E+00	0.00E+00	1.00E+00	0.00E+00	1.00E+00
ENSBTAG00000007486	5.72E-01	1.00E+00	3.30E+00	8.53E-04	1.18E+00	4.31E-01	1.14E-01	1.00E+00	-3.59E+00	3.51E-01
ENSBTAG00000004194	2.83E+00	1.68E-10	3.37E+00	2.80E-15	2.07E+00	6.57E-07	1.39E+00	1.30E-03	-1.28E-01	1.00E+00
ENSBTAG00000030319	-1.19E-01	1.00E+00	7.86E-01	2.11E-03	1.13E+00	4.00E-06	1.08E+00	1.36E-05	-2.03E-01	9.69E-01
ENSBTAG00000000893	1.92E+00	8.23E-01	2.79E+00	2.10E-01	1.78E+00	4.79E-01	2.53E+00	2.71E-01	0.00E+00	1.00E+00
ENSBTAG00000048091	2.72E+00	5.76E-01	3.98E+00	2.57E-02	2.56E+00	2.54E-01	1.76E+00	4.97E-01	1.91E+00	8.00E-01
ENSBTAG00000014678	-3.57E+00	2.46E-01	6.52E-01	8.02E-01	-3.57E+00	4.98E-02	5.72E-01	8.57E-01	9.21E+00	1.47E-41

ENSBTAG00000020249	0.00E+00	1.00E+00	1.99E+00	4.43E-01	-5.13E-15	1.00E+00	0.00E+00	1.00E+00	2.71E+00	6.01E-01
ENSBTAG00000038972	0.00E+00	1.00E+00	2.79E+00	2.10E-01	-5.13E-15	1.00E+00	0.00E+00	1.00E+00	1.91E+00	8.00E-01
ENSBTAG00000006558	-1.29E+00	9.38E-01	-3.21E+00	1.26E-01	-6.50E-01	8.99E-01	-1.45E+00	4.97E-01	9.41E-01	9.05E-01
ENSBTAG00000018975	3.26E-01	9.45E-01	-6.99E-02	1.00E+00	-1.21E-01	1.00E+00	9.22E-02	1.00E+00	2.87E-01	9.90E-01
ENSBTAG00000006008	2.30E-01	7.97E-01	7.17E-01	1.70E-04	8.96E-01	1.26E-06	4.92E-01	1.66E-02	2.08E-01	8.00E-01
ENSBTAG00000039851	1.18E-02	1.00E+00	2.07E-02	1.00E+00	5.35E-01	2.91E-02	5.99E-01	1.43E-02	3.23E-01	6.43E-01
ENSBTAG00000020490	3.09E-01	8.23E-01	1.48E+00	6.01E-09	1.33E+00	1.78E-07	8.22E-01	2.77E-03	7.76E-02	1.00E+00
ENSBTAG00000046939	-3.80E-01	5.76E-01	-8.27E-01	1.28E-03	-8.19E-02	1.00E+00	4.44E-01	1.32E-01	7.39E-02	1.00E+00
ENSBTAG00000011124	2.71E+00	5.76E-01	1.99E+00	4.43E-01	2.56E+00	2.54E-01	3.04E+00	1.49E-01	4.13E+00	1.36E-01
ENSBTAG00000011129	-3.07E-01	6.32E-01	5.69E-01	1.22E-02	1.00E+00	1.95E-06	3.87E-01	1.29E-01	2.94E-01	6.84E-01
ENSBTAG00000023064	-2.82E-01	1.00E+00	-8.58E-01	4.06E-01	6.13E-01	4.79E-01	1.97E-01	1.00E+00	4.49E-01	1.00E+00
ENSBTAG00000018799	4.52E-01	5.76E-01	-4.34E-01	2.75E-01	-2.72E-01	5.77E-01	-2.77E-01	5.77E-01	4.45E-03	1.00E+00
ENSBTAG00000012880	-1.21E-01	1.00E+00	-1.62E-01	9.40E-01	-2.90E-01	6.04E-01	-4.59E-01	3.08E-01	-1.96E-01	1.00E+00
ENSBTAG00000006572	-4.29E-01	5.76E-01	-1.47E+00	2.50E-07	-3.85E-01	2.74E-01	-5.09E-01	1.21E-01	4.90E-02	1.00E+00
ENSBTAG00000000054	1.17E-01	1.00E+00	3.92E-01	1.21E-01	9.29E-01	1.55E-05	1.76E-01	6.50E-01	2.12E-01	8.55E-01
ENSBTAG00000001352	6.40E-02	1.00E+00	8.17E-01	1.33E-02	1.12E+00	3.89E-04	7.88E-01	2.01E-02	-4.43E-02	1.00E+00
ENSBTAG00000001353	-9.01E-02	1.00E+00	4.32E-02	1.00E+00	9.84E-02	9.68E-01	2.79E-02	1.00E+00	3.90E-02	1.00E+00
ENSBTAG00000001354	-1.97E-01	1.00E+00	1.53E-01	9.03E-01	-1.43E+00	6.36E-06	-1.77E+00	4.64E-08	-1.16E-01	1.00E+00
ENSBTAG00000023933	-1.68E-01	1.00E+00	5.05E-01	6.76E-02	3.25E-01	3.12E-01	5.52E-01	4.62E-02	5.74E-01	2.10E-01
ENSBTAG00000022799	-1.09E+00	3.55E-05	-1.16E+00	7.43E-07	-6.32E-01	1.14E-02	-1.09E+00	4.40E-06	2.74E-01	8.00E-01
ENSBTAG00000030278	-4.64E-02	1.00E+00	-8.45E-01	1.05E-01	9.17E-02	1.00E+00	-5.18E-01	4.23E-01	-3.01E-01	1.00E+00
ENSBTAG00000029979	-3.29E-01	1.00E+00	-5.80E-02	1.00E+00	1.72E+00	7.12E-03	3.23E-01	9.65E-01	4.20E-01	1.00E+00
ENSBTAG00000025161	-3.83E-01	3.75E-01	-1.74E+00	2.41E-17	-2.94E+00	7.65E-44	-2.00E+00	1.87E-22	-4.35E-02	1.00E+00
ENSBTAG00000021928	6.32E-01	9.44E-01	-1.90E+00	1.19E-01	-5.23E-01	7.83E-01	-1.66E+00	1.48E-01	2.95E-01	1.00E+00
ENSBTAG00000042930	-9.14E-01	2.09E-01	2.80E-01	7.30E-01	1.63E+00	1.39E-05	7.65E-01	8.49E-02	-1.28E-01	1.00E+00
ENSBTAG00000042482	-1.39E-01	1.00E+00	-1.67E-02	1.00E+00	1.23E+00	1.40E-03	5.25E-01	2.88E-01	5.49E-01	6.28E-01
ENSBTAG00000030281	-1.19E-01	1.00E+00	-9.09E-02	1.00E+00	-1.65E-01	8.89E-01	3.13E-02	1.00E+00	-7.53E-01	1.94E-01
ENSBTAG00000012525	1.07E-01	1.00E+00	6.05E-01	1.15E-01	3.31E-02	1.00E+00	-1.78E-01	8.94E-01	9.72E-03	1.00E+00
ENSBTAG00000042565	0.00E+00	1.00E+00	0.00E+00	1.00E+00	-5.13E-15	1.00E+00	0.00E+00	1.00E+00	0.00E+00	1.00E+00
ENSBTAG00000011554	3.20E-01	6.56E-01	1.98E+00	7.37E-20	2.49E+00	1.92E-30	2.04E+00	4.74E-21	-2.19E-02	1.00E+00
ENSBTAG00000011556	-1.63E-01	1.00E+00	3.12E-02	1.00E+00	1.06E+00	6.32E-07	1.07E+00	5.99E-07	1.97E-01	9.01E-01
ENSBTAG00000011559	1.68E-02	1.00E+00	3.68E-01	1.71E-01	2.94E-01	3.14E-01	4.47E-01	8.39E-02	-1.07E-01	1.00E+00
ENSBTAG00000043383	0.00E+00	1.00E+00	0.00E+00	1.00E+00	-5.13E-15	1.00E+00	0.00E+00	1.00E+00	0.00E+00	1.00E+00
ENSBTAG00000043101	0.00E+00	1.00E+00	0.00E+00	1.00E+00	-5.13E-15	1.00E+00	0.00E+00	1.00E+00	0.00E+00	1.00E+00
ENSBTAG00000043016	0.00E+00	1.00E+00	0.00E+00	1.00E+00	-5.13E-15	1.00E+00	0.00E+00	1.00E+00	0.00E+00	1.00E+00

ENSBTAG00000042381	0.00E+00	1.00E+00	0.00E+00	1.00E+00	-5.13E-15	1.00E+00	0.00E+00	1.00E+00	0.00E+00	1.00E+00
ENSBTAG00000043341	0.00E+00	1.00E+00	0.00E+00	1.00E+00	-5.13E-15	1.00E+00	0.00E+00	1.00E+00	0.00E+00	1.00E+00
ENSBTAG00000030384	2.94E-01	6.60E-01	9.69E-01	4.20E-06	6.73E-01	2.02E-03	4.78E-01	4.45E-02	2.09E-01	8.46E-01
ENSBTAG00000000046	0.00E+00	1.00E+00	0.00E+00	1.00E+00	-5.13E-15	1.00E+00	0.00E+00	1.00E+00	0.00E+00	1.00E+00
ENSBTAG00000004140	5.79E-01	7.71E-01	4.20E+00	7.48E-19	2.32E+00	4.02E-07	7.47E-01	1.92E-01	5.63E-01	7.92E-01
ENSBTAG00000019721	-9.78E-02	1.00E+00	1.00E+00	2.69E-06	1.67E+00	4.89E-16	1.21E+00	8.79E-09	-2.18E-01	8.47E-01
ENSBTAG00000015093	-1.77E-02	1.00E+00	-2.96E-01	4.69E-01	-9.59E-01	9.82E-04	-8.72E-01	3.67E-03	8.09E-02	1.00E+00
ENSBTAG00000015097	2.62E-01	8.24E-01	-8.28E-02	1.00E+00	4.83E-01	8.51E-02	8.73E-01	5.54E-04	-3.92E-02	1.00E+00
ENSBTAG00000001824	2.22E+00	5.51E-14	2.26E+00	1.85E-15	3.32E-01	4.60E-01	1.56E-01	9.23E-01	7.26E-02	1.00E+00
ENSBTAG00000013242	-3.50E-01	8.29E-01	-1.84E+00	5.92E-07	1.64E-01	9.01E-01	-2.59E-01	6.66E-01	1.53E-01	1.00E+00
ENSBTAG00000007492	1.56E-01	1.00E+00	-8.40E-01	3.18E-03	-4.06E-01	2.32E-01	-8.44E-01	3.49E-03	-1.27E-02	1.00E+00
ENSBTAG00000004507	7.82E-01	8.78E-01	-2.35E+00	1.03E-01	-6.12E-01	7.68E-01	-1.31E+00	3.41E-01	-1.83E-01	1.00E+00
ENSBTAG00000004508	1.64E+00	5.76E-01	1.27E+00	4.46E-01	3.38E-01	1.00E+00	-9.46E-01	8.04E-01	-5.10E-03	1.00E+00
ENSBTAG00000004510	-5.48E-01	2.28E-01	-1.38E+00	7.53E-08	-2.67E+00	7.37E-25	-3.11E+00	4.80E-32	-9.33E-02	1.00E+00
ENSBTAG00000004511	-1.68E-01	1.00E+00	-1.24E+00	2.70E-08	-6.45E-01	5.95E-03	-3.62E-01	1.92E-01	-1.52E-01	1.00E+00
ENSBTAG00000010582	-5.16E-01	1.45E-01	-1.29E+00	6.36E-09	-9.28E-02	9.79E-01	-5.62E-03	1.00E+00	4.37E-01	3.54E-01
ENSBTAG00000047031	-5.07E-02	1.00E+00	7.91E-01	5.40E-04	8.40E-01	2.05E-04	4.60E-01	7.54E-02	2.10E-01	8.92E-01
ENSBTAG00000045761	0.00E+00	1.00E+00	0.00E+00	1.00E+00	-5.13E-15	1.00E+00	0.00E+00	1.00E+00	0.00E+00	1.00E+00
ENSBTAG00000046751	0.00E+00	1.00E+00	0.00E+00	1.00E+00	-5.13E-15	1.00E+00	0.00E+00	1.00E+00	0.00E+00	1.00E+00
ENSBTAG00000047950	0.00E+00	1.00E+00	0.00E+00	1.00E+00	-5.13E-15	1.00E+00	0.00E+00	1.00E+00	0.00E+00	1.00E+00
ENSBTAG00000008308	-2.70E+00	5.76E-01	1.93E+00	1.22E-01	1.47E+00	3.00E-01	-9.39E-01	8.06E-01	1.88E-02	1.00E+00
ENSBTAG00000015929	0.00E+00	1.00E+00	1.98E+00	4.43E-01	1.78E+00	4.79E-01	0.00E+00	1.00E+00	0.00E+00	1.00E+00
ENSBTAG00000007882	-1.22E-01	1.00E+00	-1.46E-01	1.00E+00	-4.19E-01	6.49E-01	-2.70E-01	8.97E-01	1.85E-01	1.00E+00
ENSBTAG00000012148	-3.44E-02	1.00E+00	-1.77E-01	6.19E-01	2.37E-01	4.26E-01	3.82E-02	1.00E+00	-1.14E-02	1.00E+00
ENSBTAG00000012147	-2.19E-01	9.86E-01	-1.42E+00	1.17E-07	-5.98E-01	3.96E-02	-1.88E-01	7.33E-01	9.83E-02	1.00E+00
ENSBTAG00000012144	-2.19E-01	9.48E-01	1.43E-01	8.48E-01	1.02E+00	2.20E-05	6.81E-01	8.47E-03	-1.20E-01	1.00E+00
ENSBTAG00000038691	-3.23E-01	7.26E-01	8.30E-02	1.00E+00	9.28E-02	1.00E+00	-3.15E-01	3.49E-01	-1.84E-01	1.00E+00
ENSBTAG00000039138	-1.83E-01	1.00E+00	3.76E-01	1.73E-01	3.93E-01	1.47E-01	1.39E-01	8.23E-01	-2.87E-01	7.73E-01
ENSBTAG00000012126	1.10E-01	1.00E+00	-1.95E-01	6.85E-01	-1.41E-01	8.64E-01	-9.46E-01	3.49E-04	4.89E-01	3.93E-01
ENSBTAG00000030247	-1.27E-01	1.00E+00	-1.43E-01	7.67E-01	-1.09E-01	8.99E-01	-3.84E-01	1.32E-01	6.17E-02	1.00E+00
ENSBTAG00000030246	-1.59E-02	1.00E+00	-5.80E-01	2.44E-02	-1.37E+00	5.69E-09	-1.07E+00	8.22E-06	5.29E-01	2.58E-01
ENSBTAG00000012121	-6.36E-02	1.00E+00	-8.44E-01	1.36E-01	-1.53E+00	2.47E-03	-1.63E+00	1.48E-03	3.66E-01	9.86E-01
ENSBTAG00000023788	-1.60E-01	1.00E+00	-1.89E+00	3.00E-10	-6.22E-01	4.76E-02	-4.93E-01	1.49E-01	3.20E-01	8.00E-01
ENSBTAG00000046122	-1.35E+00	4.57E-01	-1.49E+00	1.23E-01	-8.06E-01	4.28E-01	-8.41E-01	4.21E-01	4.44E-01	1.00E+00
ENSBTAG00000023787	-1.42E+00	1.27E-03	-1.85E+00	1.60E-06	-7.36E-01	6.54E-02	-6.03E-01	1.60E-01	-6.01E-01	5.15E-01

ENSBTAG00000021861	-1.71E-01	1.00E+00	-4.44E-01	2.21E-01	-3.59E-01	3.67E-01	-3.79E-01	3.39E-01	1.71E-02	1.00E+00
ENSBTAG00000004095	-9.82E-03	1.00E+00	8.34E-01	5.97E-01	1.32E+00	2.20E-01	-5.73E-01	9.11E-01	7.51E-01	9.85E-01
ENSBTAG00000025274	8.33E-01	3.97E-04	2.55E+00	2.27E-36	1.70E+00	1.35E-17	8.49E-01	4.25E-05	-6.55E-01	3.02E-02
ENSBTAG00000046379	2.42E-02	1.00E+00	-3.58E+00	6.71E-02	-1.80E+00	2.98E-01	-5.44E-01	9.31E-01	7.74E-01	9.69E-01
ENSBTAG00000046418	1.11E-02	1.00E+00	-7.16E-01	9.45E-01	-2.70E+00	1.99E-01	-2.70E+00	2.05E-01	7.70E-03	1.00E+00
ENSBTAG00000045711	-1.42E+00	3.05E-01	-6.92E-01	4.69E-01	-5.76E+00	3.42E-06	-2.74E+00	1.76E-03	-4.28E-01	1.00E+00
ENSBTAG00000047357	-1.93E-01	1.00E+00	-2.15E+00	3.60E-06	-2.14E+00	1.37E-06	-1.97E+00	7.47E-06	3.48E-01	8.70E-01
ENSBTAG00000047061	4.67E-01	1.30E-01	1.63E+00	3.68E-18	1.69E+00	1.71E-19	7.32E-01	2.29E-04	5.03E-01	1.31E-01
ENSBTAG00000046215	4.39E-02	1.00E+00	-4.94E-01	5.07E-02	-5.80E-02	1.00E+00	1.54E-01	7.59E-01	-2.31E-01	8.30E-01
ENSBTAG00000047715	-1.02E+00	6.29E-02	-3.18E-01	6.17E-01	9.55E-01	1.76E-02	6.78E-01	1.30E-01	-9.40E-02	1.00E+00
ENSBTAG00000046415	-3.42E-01	6.08E-01	-8.83E-01	2.31E-04	-7.35E-01	2.55E-03	-6.53E-01	9.86E-03	-3.66E-01	5.98E-01
ENSBTAG00000046223	-6.19E-02	1.00E+00	1.73E-01	5.45E-01	2.61E-01	2.65E-01	-9.22E-02	9.22E-01	1.18E-01	1.00E+00
ENSBTAG00000046416	1.55E-01	1.00E+00	1.17E-01	9.49E-01	2.41E-01	5.24E-01	-5.32E-01	6.92E-02	-6.85E-02	1.00E+00
ENSBTAG00000047964	1.30E+00	9.32E-01	3.59E+00	9.25E-04	-1.31E-01	1.00E+00	-1.48E-01	1.00E+00	1.80E-02	1.00E+00
ENSBTAG00000045734	2.00E-01	1.00E+00	-6.57E-01	7.55E-01	-1.78E+00	1.69E-01	-1.31E+00	3.34E-01	8.58E-03	1.00E+00
ENSBTAG00000047202	-1.68E-01	1.00E+00	-1.68E+00	2.89E-06	-2.52E+00	1.18E-11	-1.70E+00	1.09E-06	2.23E-01	1.00E+00
ENSBTAG00000017851	-3.39E-01	7.27E-01	-1.15E+00	9.64E-06	-1.54E+00	2.13E-09	-6.97E-01	1.30E-02	5.90E-01	2.36E-01
ENSBTAG00000046775	5.70E-01	1.42E-01	1.63E+00	5.65E-12	1.17E+00	9.73E-07	1.06E+00	1.39E-05	7.21E-01	5.30E-02
ENSBTAG00000048054	-2.47E-01	8.81E-01	-1.51E+00	4.02E-09	-2.05E+00	1.51E-15	-1.38E+00	6.80E-08	5.85E-02	1.00E+00
ENSBTAG00000047807	1.09E+00	7.32E-01	5.52E-01	8.31E-01	1.35E+00	1.54E-01	6.42E-02	1.00E+00	1.45E+00	4.47E-01
ENSBTAG00000048125	-5.14E-01	8.74E-01	-1.24E+00	4.71E-02	-1.24E+00	3.68E-02	-7.12E-01	2.88E-01	-8.44E-02	1.00E+00
ENSBTAG00000046140	-1.04E+00	4.17E-01	-7.66E-01	3.13E-01	-1.03E+00	1.28E-01	-1.33E+00	4.50E-02	-3.76E-01	1.00E+00
ENSBTAG00000046516	-2.30E-01	1.00E+00	-1.66E+00	3.63E-02	-2.61E-01	9.60E-01	-2.63E-01	9.59E-01	-1.38E-01	1.00E+00
ENSBTAG00000047254	-2.89E-02	1.00E+00	-1.24E+00	6.15E-03	-1.00E+00	2.61E-02	-1.49E+00	8.29E-04	1.34E-01	1.00E+00
ENSBTAG00000015085	-8.06E-01	7.45E-01	-1.26E+00	9.90E-02	-1.34E+00	6.48E-02	-1.33E+00	7.47E-02	-7.23E-01	8.00E-01
ENSBTAG00000015081	0.00E+00	1.00E+00	0.00E+00	1.00E+00	-5.13E-15	1.00E+00	0.00E+00	1.00E+00	0.00E+00	1.00E+00
ENSBTAG00000030296	1.69E+00	7.72E-01	7.79E-02	1.00E+00	1.14E+00	6.48E-01	-1.47E-01	1.00E+00	1.31E+00	8.99E-01
ENSBTAG00000015074	-3.55E-01	9.67E-01	-4.08E-01	5.11E-01	-1.04E+00	2.10E-02	-1.15E+00	1.10E-02	-5.28E-01	7.59E-01
ENSBTAG00000039531	1.11E+00	1.07E-01	3.28E+00	3.69E-13	1.66E+00	2.50E-04	2.32E-01	9.08E-01	1.00E-01	1.00E+00
ENSBTAG00000010520	9.48E-02	1.00E+00	-3.82E-01	2.56E-01	-1.20E+00	6.44E-06	-1.68E+00	2.32E-10	9.26E-02	1.00E+00
ENSBTAG00000010515	2.41E-01	8.92E-01	1.66E-02	1.00E+00	-6.42E-01	1.61E-02	-1.05E+00	3.22E-05	4.62E-01	4.34E-01
ENSBTAG00000010497	5.28E-01	6.53E-01	1.23E+00	1.78E-03	-1.44E-01	1.00E+00	-4.40E-01	4.21E-01	3.02E-01	9.73E-01
ENSBTAG00000030302	-6.80E-02	1.00E+00	-3.21E-01	1.77E-01	-8.17E-01	3.28E-05	-8.08E-01	5.19E-05	-3.26E-01	5.32E-01
ENSBTAG00000021550	2.72E-01	9.74E-01	3.64E-01	4.29E-01	4.39E-01	2.80E-01	-1.06E-01	1.00E+00	2.63E-02	1.00E+00
ENSBTAG00000021549	-2.64E-01	8.23E-01	-8.94E-01	3.36E-04	-1.26E+00	2.23E-07	-1.23E+00	5.43E-07	-1.94E-01	9.76E-01

ENSBTAG00000039708	-4.23E-02	1.00E+00	2.45E+00	7.01E-12	4.29E-01	4.78E-01	2.23E-01	8.90E-01	-3.41E-01	1.00E+00
ENSBTAG00000039573	-1.81E-01	1.00E+00	-1.41E-01	8.42E-01	-1.48E-01	8.06E-01	-7.53E-02	1.00E+00	-1.56E-01	1.00E+00
ENSBTAG00000000933	-3.38E-01	8.55E-01	-4.28E-01	3.34E-01	-8.97E-03	1.00E+00	-9.21E-01	1.09E-02	5.63E-02	1.00E+00
ENSBTAG00000013889	-6.75E-02	1.00E+00	-1.04E+00	6.76E-04	1.12E-01	1.00E+00	6.41E-01	3.61E-02	1.25E-02	1.00E+00
ENSBTAG00000047297	-7.92E-01	1.00E+00	-2.71E+00	2.42E-01	-2.71E+00	1.99E-01	-2.71E+00	2.05E-01	-2.71E+00	6.01E-01
ENSBTAG00000017005	-2.70E+00	5.76E-01	-2.70E+00	2.42E-01	-9.17E-01	8.09E-01	-1.72E-01	1.00E+00	-2.70E+00	6.01E-01
ENSBTAG00000001206	1.92E+00	8.23E-01	0.00E+00	1.00E+00	1.78E+00	4.79E-01	1.76E+00	4.97E-01	0.00E+00	1.00E+00
ENSBTAG00000016148	1.68E+00	7.74E-01	1.39E+00	5.16E-01	-1.31E-01	1.00E+00	1.13E+00	6.66E-01	1.68E+00	7.78E-01
ENSBTAG00000016144	-1.91E+00	8.23E-01	-1.91E+00	4.69E-01	-1.91E+00	4.28E-01	-1.91E+00	4.39E-01	-1.91E+00	8.00E-01
ENSBTAG00000009599	-1.10E+00	1.00E+00	-1.34E+00	7.40E-01	-6.27E+00	1.52E-02	-4.55E+00	7.61E-02	-7.64E-01	1.00E+00
ENSBTAG00000038440	1.91E+00	8.23E-01	3.68E+00	5.07E-02	3.06E+00	1.36E-01	2.53E+00	2.71E-01	1.91E+00	8.00E-01
ENSBTAG00000005688	-3.38E-01	6.41E-01	2.64E-01	4.43E-01	5.62E-01	3.00E-02	6.97E-01	5.97E-03	-4.49E-01	4.31E-01
ENSBTAG00000023776	-4.51E-01	8.23E-01	-6.43E-01	1.95E-01	-1.63E+00	9.41E-05	-1.40E+00	1.08E-03	8.79E-02	1.00E+00
ENSBTAG00000030317	-3.32E-01	8.16E-01	-1.47E+00	4.16E-07	-5.69E-01	7.44E-02	5.64E-02	1.00E+00	1.50E-01	1.00E+00
ENSBTAG00000038584	-6.16E-01	1.00E+00	-3.09E-02	1.00E+00	3.62E-01	8.72E-01	6.46E-01	4.97E-01	-8.25E-01	8.24E-01
ENSBTAG00000048155	2.53E+00	4.87E-08	3.21E+00	1.31E-13	3.17E+00	1.95E-13	3.35E+00	1.44E-14	1.61E+00	7.28E-03
ENSBTAG00000047998	1.49E-01	1.00E+00	-1.30E-01	9.43E-01	-1.16E-01	9.79E-01	2.51E-01	5.61E-01	1.06E-01	1.00E+00
ENSBTAG00000022142	0.00E+00	1.00E+00	1.98E+00	4.43E-01	-5.13E-15	1.00E+00	0.00E+00	1.00E+00	1.91E+00	8.00E-01
ENSBTAG00000001730	6.06E-03	1.00E+00	7.64E-02	1.00E+00	6.45E-01	9.78E-01	-1.91E+00	4.39E-01	7.05E-03	1.00E+00
ENSBTAG00000001744	-1.34E-01	1.00E+00	5.48E-01	3.95E-02	8.44E-01	6.02E-04	1.06E+00	1.50E-05	6.50E-02	1.00E+00
ENSBTAG00000002042	-3.92E-01	5.03E-01	-4.22E-01	1.34E-01	1.75E-01	6.97E-01	3.05E-01	3.39E-01	6.57E-02	1.00E+00
ENSBTAG00000002044	-1.66E-01	9.90E-01	-9.91E-01	7.87E-07	-1.35E+00	9.58E-12	-1.76E+00	8.91E-19	-2.30E-01	8.00E-01
ENSBTAG00000019017	-2.11E-01	1.00E+00	-7.53E-02	1.00E+00	-5.50E-01	3.07E-01	-5.63E-01	3.04E-01	-1.28E-01	1.00E+00
ENSBTAG00000020575	2.10E-01	1.00E+00	-7.74E-01	2.80E-01	-2.16E+00	2.36E-04	-1.94E+00	1.18E-03	6.19E-01	8.00E-01
ENSBTAG00000016722	8.25E-02	1.00E+00	-8.00E-01	2.93E-02	-1.82E+00	7.60E-08	-1.82E+00	1.09E-07	4.96E-01	6.10E-01
ENSBTAG00000001076	5.44E-01	1.00E+00	9.30E-02	1.00E+00	-9.09E-01	8.24E-01	-9.36E-01	8.18E-01	3.23E+00	2.21E-02
ENSBTAG00000002046	-1.52E-01	1.00E+00	-5.37E-01	1.07E-01	-9.23E-01	2.02E-03	-4.94E-01	1.54E-01	-3.40E-01	8.00E-01
ENSBTAG00000002045	-7.70E-02	1.00E+00	-4.22E-01	5.60E-02	-2.75E-01	2.75E-01	-1.99E-02	1.00E+00	-1.12E-01	1.00E+00
ENSBTAG00000019018	-2.07E-01	1.00E+00	-2.50E-01	1.00E+00	-7.66E-01	4.12E-01	-9.37E-01	2.78E-01	-1.06E-01	1.00E+00
ENSBTAG00000011862	1.30E+00	9.32E-01	8.80E-01	8.52E-01	-1.91E+00	4.28E-01	-1.47E-01	1.00E+00	7.99E-01	1.00E+00
ENSBTAG00000035926	-1.35E-01	1.00E+00	1.41E-03	1.00E+00	-8.49E-02	1.00E+00	-1.74E-01	6.81E-01	-7.33E-01	2.68E-02
ENSBTAG00000043178	0.00E+00	1.00E+00	0.00E+00	1.00E+00	-5.13E-15	1.00E+00	0.00E+00	1.00E+00	0.00E+00	1.00E+00
ENSBTAG00000009900	1.03E+00	1.07E-01	6.01E-01	2.77E-01	4.20E-01	5.07E-01	6.98E-01	1.76E-01	4.91E-01	8.00E-01
ENSBTAG00000009308	1.39E-02	1.00E+00	-8.89E-02	9.75E-01	3.98E-01	8.35E-02	1.02E-01	9.18E-01	-2.69E-01	7.34E-01
ENSBTAG00000026861	0.00E+00	1.00E+00	0.00E+00	1.00E+00	-5.13E-15	1.00E+00	0.00E+00	1.00E+00	0.00E+00	1.00E+00

ENSBTAG00000012443	-3.58E-01	1.00E+00	-4.51E-01	5.72E-01	-1.42E+00	1.06E-02	-3.22E-02	1.00E+00	-6.51E-01	7.47E-01
ENSBTAG00000029683	0.00E+00	1.00E+00	1.98E+00	4.43E-01	2.55E+00	2.55E-01	0.00E+00	1.00E+00	0.00E+00	1.00E+00
ENSBTAG00000044554	0.00E+00	1.00E+00	0.00E+00	1.00E+00	-5.13E-15	1.00E+00	0.00E+00	1.00E+00	0.00E+00	1.00E+00
ENSBTAG00000010420	-6.24E-01	8.23E-01	2.14E-01	1.00E+00	-3.04E-01	8.50E-01	1.90E-01	1.00E+00	-1.31E-01	1.00E+00
ENSBTAG00000047201	0.00E+00	1.00E+00	0.00E+00	1.00E+00	-5.13E-15	1.00E+00	0.00E+00	1.00E+00	0.00E+00	1.00E+00
ENSBTAG00000045443	0.00E+00	1.00E+00	0.00E+00	1.00E+00	-5.13E-15	1.00E+00	1.76E+00	4.97E-01	0.00E+00	1.00E+00
ENSBTAG00000046667	0.00E+00	1.00E+00	0.00E+00	1.00E+00	-5.13E-15	1.00E+00	0.00E+00	1.00E+00	0.00E+00	1.00E+00
ENSBTAG00000045600	0.00E+00	1.00E+00	0.00E+00	1.00E+00	-5.13E-15	1.00E+00	0.00E+00	1.00E+00	0.00E+00	1.00E+00
ENSBTAG00000042569	0.00E+00	1.00E+00	0.00E+00	1.00E+00	-5.13E-15	1.00E+00	0.00E+00	1.00E+00	0.00E+00	1.00E+00
ENSBTAG00000025953	-1.03E+00	5.76E-01	9.87E-01	1.10E-01	4.75E-01	5.95E-01	7.68E-02	1.00E+00	-4.31E-01	1.00E+00
ENSBTAG00000036553	-1.91E+00	8.23E-01	-1.91E+00	4.69E-01	-1.91E+00	4.28E-01	-1.91E+00	4.39E-01	-1.91E+00	8.00E-01
ENSBTAG00000048161	2.72E+00	5.76E-01	3.68E+00	5.07E-02	2.56E+00	2.54E-01	0.00E+00	1.00E+00	0.00E+00	1.00E+00
ENSBTAG00000022779	8.49E-01	8.25E-01	2.83E+00	4.57E-06	2.94E+00	7.40E-07	2.13E+00	1.98E-03	7.17E-01	9.18E-01
ENSBTAG00000018179	1.95E+00	1.94E-01	1.72E+00	1.02E-01	1.92E+00	4.52E-02	1.27E+00	2.96E-01	1.88E+00	3.09E-01
ENSBTAG00000030110	0.00E+00	1.00E+00	0.00E+00	1.00E+00	-5.13E-15	1.00E+00	0.00E+00	1.00E+00	0.00E+00	1.00E+00
ENSBTAG00000025502	4.53E-02	1.00E+00	8.50E-01	7.18E-03	-1.81E-01	8.48E-01	-1.01E+00	4.87E-03	2.87E-01	8.97E-01
ENSBTAG00000002137	3.69E-01	4.03E-01	1.23E+00	1.03E-09	9.12E-01	8.38E-06	6.57E-01	2.41E-03	-1.44E-01	1.00E+00
ENSBTAG00000007356	3.44E-01	5.88E-01	1.51E+00	3.47E-11	1.85E+00	3.63E-16	1.72E+00	3.69E-14	2.07E-02	1.00E+00
ENSBTAG00000044514	1.92E+00	8.23E-01	3.30E+00	1.02E-01	2.55E+00	2.54E-01	0.00E+00	1.00E+00	0.00E+00	1.00E+00
ENSBTAG00000016637	3.65E-01	6.14E-01	8.36E-01	1.26E-03	2.97E-01	4.00E-01	6.01E-02	1.00E+00	-7.28E-02	1.00E+00
ENSBTAG00000008656	1.33E+00	2.10E-01	1.59E+00	1.57E-02	1.05E+00	1.47E-01	1.60E+00	1.74E-02	6.38E-01	8.08E-01
ENSBTAG00000039823	-8.80E-01	8.03E-02	-2.13E+00	2.08E-09	-1.52E+00	1.03E-05	-2.40E-01	7.25E-01	2.15E-01	1.00E+00
ENSBTAG00000045392	0.00E+00	1.00E+00	0.00E+00	1.00E+00	1.78E+00	4.79E-01	0.00E+00	1.00E+00	0.00E+00	1.00E+00
ENSBTAG00000012260	-1.43E-01	1.00E+00	-1.09E+00	2.08E-05	-8.26E-01	1.45E-03	-6.65E-01	1.48E-02	-2.72E-01	8.00E-01
ENSBTAG00000038251	-1.91E-01	1.00E+00	-9.63E-01	5.66E-04	3.72E-01	2.68E-01	1.61E-01	8.24E-01	-1.15E-02	1.00E+00
ENSBTAG00000034885	-1.52E-02	1.00E+00	-4.61E-01	4.64E-01	-3.24E-01	6.75E-01	4.61E-02	1.00E+00	-2.98E-01	1.00E+00
ENSBTAG00000001133	9.51E-03	1.00E+00	-4.80E-01	1.72E-01	-1.37E+00	2.69E-06	-1.32E+00	8.36E-06	1.07E-01	1.00E+00
ENSBTAG00000013879	2.50E-01	1.00E+00	8.46E-01	4.12E-02	8.71E-01	3.05E-02	8.62E-01	3.66E-02	-2.10E-01	1.00E+00
ENSBTAG00000014752	9.45E-02	1.00E+00	4.38E-02	1.00E+00	6.25E-01	1.06E-01	9.68E-01	6.84E-03	2.44E-01	1.00E+00
ENSBTAG00000008924	-1.41E+00	7.71E-01	-4.58E-01	9.54E-01	-1.09E+00	4.78E-01	6.30E-01	6.94E-01	-4.14E+00	1.39E-01
ENSBTAG00000003595	3.14E-03	1.00E+00	6.51E-02	1.00E+00	1.13E+00	6.61E-01	2.94E+00	2.01E-02	-1.92E+00	8.00E-01
ENSBTAG00000019054	-1.60E-01	1.00E+00	1.25E+00	1.74E-02	8.42E-02	1.00E+00	-9.19E-01	1.12E-01	-2.14E-01	1.00E+00
ENSBTAG00000034785	-7.94E-02	1.00E+00	-7.64E-01	3.71E-02	-5.61E-01	1.53E-01	-2.21E-01	7.65E-01	3.27E-01	8.34E-01
ENSBTAG00000046916	0.00E+00	1.00E+00	0.00E+00	1.00E+00	-5.13E-15	1.00E+00	0.00E+00	1.00E+00	0.00E+00	1.00E+00
ENSBTAG00000012434	1.33E+00	3.72E-01	7.51E-01	4.95E-01	-1.97E-01	1.00E+00	1.53E+00	5.47E-02	2.05E-01	1.00E+00

ENSBTAG00000034753	3.12E-01	1.00E+00	-1.60E+00	4.08E-01	-5.33E-01	9.37E-01	-5.51E-01	9.26E-01	5.50E-01	1.00E+00
ENSBTAG00000016691	-1.83E-01	1.00E+00	-1.02E+00	2.24E-03	-1.25E+00	1.36E-04	-7.61E-01	3.24E-02	4.44E-01	6.60E-01
ENSBTAG00000044602	0.00E+00	1.00E+00	0.00E+00	1.00E+00	-5.13E-15	1.00E+00	0.00E+00	1.00E+00	0.00E+00	1.00E+00
ENSBTAG00000047739	-4.92E-01	7.67E-01	-2.14E-01	8.78E-01	-7.78E-01	8.36E-02	-1.24E+00	3.21E-03	-1.12E+00	7.44E-02
ENSBTAG00000046664	1.40E-01	1.00E+00	-1.12E+00	2.62E-02	-4.01E-01	5.64E-01	1.46E-01	1.00E+00	6.41E-01	6.07E-01
ENSBTAG00000009415	4.71E-01	2.38E-01	1.80E+00	2.29E-17	1.46E+00	7.17E-12	5.96E-01	1.34E-02	-7.94E-02	1.00E+00
ENSBTAG00000009416	5.41E-02	1.00E+00	1.09E+00	9.21E-06	1.15E+00	2.29E-06	9.18E-01	2.85E-04	1.08E-01	1.00E+00
ENSBTAG00000045689	2.54E-02	1.00E+00	-1.82E-01	7.44E-01	-8.17E-02	1.00E+00	2.21E-01	6.22E-01	1.29E-01	1.00E+00
ENSBTAG00000034495	2.66E-01	8.23E-01	9.92E-01	9.29E-06	1.28E+00	3.36E-09	1.29E+00	4.40E-09	-1.71E-01	1.00E+00
ENSBTAG00000019416	0.00E+00	1.00E+00	0.00E+00	1.00E+00	-5.13E-15	1.00E+00	0.00E+00	1.00E+00	0.00E+00	1.00E+00
ENSBTAG00000044275	5.48E-01	1.00E+00	1.62E+00	4.45E-02	-1.77E-01	1.00E+00	7.56E-01	5.40E-01	-1.43E+00	7.58E-01
ENSBTAG00000010156	7.63E-01	3.53E-03	8.63E-01	6.19E-05	1.40E-01	7.80E-01	-3.37E-01	2.09E-01	-7.33E-01	1.63E-02
ENSBTAG00000043243	0.00E+00	1.00E+00	0.00E+00	1.00E+00	-5.13E-15	1.00E+00	0.00E+00	1.00E+00	0.00E+00	1.00E+00
ENSBTAG00000016529	-3.87E-01	1.00E+00	-1.47E+00	5.59E-02	-1.57E+00	3.83E-02	-5.58E-01	6.44E-01	8.23E-01	7.91E-01
ENSBTAG00000017907	5.41E-01	1.31E-01	1.68E+00	1.67E-14	1.34E+00	9.03E-10	1.15E+00	2.49E-07	4.13E-01	4.34E-01
ENSBTAG00000018580	-2.70E+00	5.76E-01	-7.15E-01	9.45E-01	3.56E-01	1.00E+00	-1.70E-01	1.00E+00	-2.70E+00	6.01E-01
ENSBTAG00000007067	2.79E-01	8.54E-01	3.03E+00	3.61E-28	1.76E+00	4.80E-11	7.36E-01	1.24E-02	5.23E-02	1.00E+00
ENSBTAG00000007073	2.60E-01	6.86E-01	3.98E-01	6.38E-02	1.45E-01	6.83E-01	3.26E-03	1.00E+00	2.23E-02	1.00E+00
ENSBTAG00000007079	5.52E-01	3.42E-01	1.10E+00	1.95E-04	6.77E-01	3.21E-02	4.47E-01	2.21E-01	-3.09E-01	8.01E-01
ENSBTAG00000046444	0.00E+00	1.00E+00	0.00E+00	1.00E+00	-5.13E-15	1.00E+00	0.00E+00	1.00E+00	0.00E+00	1.00E+00
ENSBTAG00000015994	7.29E-01	8.23E-01	7.85E-01	3.84E-01	3.51E-01	8.82E-01	3.17E-01	9.38E-01	4.09E-02	1.00E+00
ENSBTAG00000045156	0.00E+00	1.00E+00	0.00E+00	1.00E+00	-5.13E-15	1.00E+00	0.00E+00	1.00E+00	0.00E+00	1.00E+00
ENSBTAG00000013371	1.97E+00	2.99E-03	1.63E+00	4.17E-03	1.14E+00	5.94E-02	2.45E+00	1.07E-05	-2.86E-01	1.00E+00
ENSBTAG00000002959	3.28E-01	1.00E+00	1.48E+00	3.66E-04	-6.17E-01	2.21E-01	-1.14E+00	1.00E-02	-4.15E-01	8.29E-01
ENSBTAG00000003415	-2.93E-01	8.23E-01	-1.42E+00	2.35E-08	-2.85E+00	4.16E-28	-2.20E+00	7.14E-18	-1.73E-01	1.00E+00
ENSBTAG00000013498	-4.28E-01	1.00E+00	-1.78E-02	1.00E+00	7.37E-02	1.00E+00	1.11E+00	1.51E-01	3.96E-02	1.00E+00
ENSBTAG00000006759	2.78E-01	7.48E-01	2.74E+00	2.60E-38	2.29E+00	4.92E-28	1.55E+00	1.32E-13	-3.74E-01	5.03E-01
ENSBTAG00000019501	1.76E-01	1.00E+00	1.10E+00	7.10E-05	1.26E+00	3.40E-06	8.45E-01	3.45E-03	1.91E-01	1.00E+00
ENSBTAG00000019502	1.70E-01	9.59E-01	4.54E-01	3.31E-02	4.70E-01	2.56E-02	-8.67E-02	9.69E-01	-8.44E-02	1.00E+00
ENSBTAG00000024964	7.57E-02	1.00E+00	-2.80E-01	7.23E-01	4.37E-01	4.23E-01	1.25E+00	1.83E-03	-6.64E-01	5.08E-01
ENSBTAG00000003109	-1.40E-01	1.00E+00	-7.14E-01	1.67E-03	-1.32E+00	9.17E-10	-1.59E+00	2.23E-13	-5.97E-01	9.85E-02
ENSBTAG00000003560	-7.22E-02	1.00E+00	9.75E-02	1.00E+00	6.78E-02	1.00E+00	1.88E-01	7.39E-01	-2.89E-01	8.00E-01
ENSBTAG00000006640	-4.85E-01	5.40E-01	1.73E-01	8.54E-01	-3.88E-01	3.37E-01	-5.78E-01	1.06E-01	-4.89E-01	5.64E-01
ENSBTAG00000006645	0.00E+00	1.00E+00	0.00E+00	1.00E+00	-5.13E-15	1.00E+00	0.00E+00	1.00E+00	0.00E+00	1.00E+00
ENSBTAG00000006647	-3.20E-02	1.00E+00	4.82E-02	1.00E+00	-1.52E-01	9.52E-01	-1.02E+00	3.71E-03	-4.63E-01	6.65E-01

ENSBTAG00000017508	-1.44E-01	1.00E+00	-1.68E+00	2.37E-05	-6.54E-01	1.43E-01	-1.17E-01	1.00E+00	-4.47E-01	8.00E-01
ENSBTAG00000020681	-1.97E-01	1.00E+00	-4.11E-01	1.98E-01	-2.26E-01	5.94E-01	3.06E-01	4.11E-01	4.72E-01	4.36E-01
ENSBTAG00000010475	1.91E+00	8.23E-01	0.00E+00	1.00E+00	3.06E+00	1.36E-01	3.03E+00	1.50E-01	1.93E+00	8.00E-01
ENSBTAG00000002271	-4.90E-01	4.88E-01	-1.12E+00	2.50E-04	-1.01E+00	8.91E-04	-3.90E-01	3.22E-01	-1.98E-01	1.00E+00
ENSBTAG00000034222	2.23E-01	1.00E+00	1.08E+00	1.94E-04	1.45E+00	1.65E-07	1.46E+00	1.67E-07	-1.08E-01	1.00E+00
ENSBTAG00000029179	0.00E+00	1.00E+00	0.00E+00	1.00E+00	-5.13E-15	1.00E+00	0.00E+00	1.00E+00	0.00E+00	1.00E+00
ENSBTAG00000007329	4.21E-01	7.16E-01	7.15E-01	4.11E-02	7.20E-01	3.83E-02	7.11E-01	4.63E-02	3.85E-01	7.93E-01
ENSBTAG00000013159	3.48E-01	4.19E-01	5.87E-01	4.44E-03	3.30E-01	1.64E-01	-1.93E-01	5.26E-01	-1.13E-01	1.00E+00
ENSBTAG00000034269	-2.99E-01	8.31E-01	-1.43E+00	8.92E-07	-7.54E-01	1.34E-02	-2.68E-01	5.47E-01	2.49E-01	9.15E-01
ENSBTAG00000016998	-1.05E+00	4.54E-01	-4.82E-01	6.36E-01	5.48E-01	4.66E-01	2.13E-01	1.00E+00	-1.29E+00	3.47E-01
ENSBTAG00000018410	-3.71E-02	1.00E+00	-6.21E-01	2.67E-02	-1.59E+00	4.36E-10	-2.02E+00	4.82E-15	-5.09E-01	3.71E-01
ENSBTAG00000004850	4.16E-02	1.00E+00	-7.00E-01	5.01E-02	-5.59E-02	1.00E+00	6.66E-01	6.26E-02	1.65E-01	1.00E+00
ENSBTAG00000047343	-3.33E-01	8.10E-01	-4.54E-01	1.87E-01	-9.72E-01	8.68E-04	-1.04E+00	4.50E-04	-9.56E-01	2.10E-02
ENSBTAG00000018829	-5.07E-01	5.81E-01	-2.11E+00	2.04E-09	-5.33E-01	1.99E-01	-4.06E-01	4.00E-01	-1.92E-01	1.00E+00
ENSBTAG00000046214	1.71E-01	1.00E+00	-2.71E+00	3.69E-02	-1.64E+00	1.48E-01	-1.28E+00	2.77E-01	-7.99E-01	9.00E-01
ENSBTAG00000008173	-7.48E-01	8.91E-03	-1.45E+00	2.21E-10	-3.30E-01	2.36E-01	-9.08E-02	9.96E-01	-1.82E-01	9.63E-01
ENSBTAG00000008176	-8.02E-01	5.80E-01	-2.17E+00	1.17E-03	-1.30E+00	3.17E-02	-8.10E-01	2.21E-01	-6.25E-01	8.00E-01
ENSBTAG00000036389	-1.91E+00	8.23E-01	7.78E-02	1.00E+00	-1.30E-01	1.00E+00	-1.91E+00	4.39E-01	7.05E-03	1.00E+00
ENSBTAG00000029867	-2.71E+00	5.76E-01	-7.23E-01	9.45E-01	-2.71E+00	1.99E-01	-2.71E+00	2.05E-01	1.16E-02	1.00E+00
ENSBTAG00000020149	1.78E-01	1.00E+00	1.13E+00	1.67E-07	8.82E-01	5.65E-05	6.01E-01	1.09E-02	-3.59E-01	5.69E-01
ENSBTAG00000007457	3.38E-02	1.00E+00	4.23E-01	8.13E-01	-5.51E-01	6.98E-01	-1.15E+00	2.71E-01	-1.48E+00	5.09E-01
ENSBTAG00000046408	-6.70E-01	7.69E-01	-1.41E+00	3.17E-02	1.03E-01	1.00E+00	-8.01E-01	2.34E-01	-1.97E-01	1.00E+00
ENSBTAG00000005284	1.82E-01	1.00E+00	-2.74E-01	6.59E-01	-3.77E-01	4.39E-01	-6.63E-01	1.03E-01	-2.00E-01	1.00E+00
ENSBTAG00000002970	3.52E-01	3.95E-01	3.82E-01	8.92E-02	4.08E-01	6.38E-02	3.21E-01	1.85E-01	-2.25E-01	8.00E-01
ENSBTAG00000008053	7.33E-02	1.00E+00	-1.23E+00	2.83E-08	-1.21E+00	2.68E-08	-6.14E-01	8.88E-03	-4.94E-02	1.00E+00
ENSBTAG00000016109	-3.09E-01	5.24E-01	-1.24E+00	3.98E-11	-2.40E+00	2.04E-36	-3.22E+00	9.64E-60	-6.13E-01	2.90E-02
ENSBTAG00000019863	5.18E-01	1.00E+00	9.82E-01	6.50E-01	-9.22E-01	8.08E-01	3.26E-01	1.00E+00	1.94E-02	1.00E+00
ENSBTAG00000010353	5.07E-01	2.09E-01	-7.49E-01	1.99E-03	-1.05E+00	7.48E-06	-3.24E-01	2.92E-01	8.58E-01	6.93E-03
ENSBTAG00000010337	6.56E-02	1.00E+00	1.09E+00	4.29E-08	1.21E+00	9.35E-10	1.05E+00	1.97E-07	-1.05E-01	1.00E+00
ENSBTAG00000019134	8.19E-01	9.17E-01	7.46E-01	6.24E-01	-8.23E-01	6.74E-01	-8.42E-01	6.70E-01	1.22E+00	6.12E-01
ENSBTAG00000020612	1.68E-01	1.00E+00	-1.35E+00	2.53E-05	-9.74E-01	2.62E-03	-8.54E-01	1.10E-02	-1.18E-02	1.00E+00
ENSBTAG00000021162	1.53E-01	1.00E+00	-9.32E-01	1.25E-02	-4.89E-01	2.50E-01	-7.14E-01	6.73E-02	-5.78E-01	5.12E-01
ENSBTAG00000004307	-4.18E-01	3.88E-01	-6.07E-01	1.48E-02	-1.69E-01	7.04E-01	3.31E-01	2.60E-01	-4.21E-01	4.41E-01
ENSBTAG00000020374	-2.05E+00	7.52E-08	-2.46E+00	2.42E-11	-1.37E+00	6.04E-05	-1.55E+00	7.35E-06	-1.13E+00	2.10E-02
ENSBTAG00000042987	0.00E+00	1.00E+00	0.00E+00	1.00E+00	-5.13E-15	1.00E+00	0.00E+00	1.00E+00	0.00E+00	1.00E+00

ENSBTAG00000011654	-6.85E-01	7.22E-01	-9.55E-01	1.27E-01	-6.63E-01	3.30E-01	-8.05E-01	2.14E-01	-3.16E-01	1.00E+00
ENSBTAG00000011647	-7.01E-01	2.03E-01	-1.34E+00	3.43E-05	-1.42E+00	1.11E-05	-7.54E-01	3.47E-02	2.09E-01	1.00E+00
ENSBTAG00000015522	1.74E-01	1.00E+00	1.12E+00	2.35E-07	1.45E+00	8.13E-12	1.05E+00	1.52E-06	-2.49E-01	8.00E-01
ENSBTAG00000042372	0.00E+00	1.00E+00	0.00E+00	1.00E+00	-5.13E-15	1.00E+00	0.00E+00	1.00E+00	0.00E+00	1.00E+00
ENSBTAG00000044105	3.47E-01	8.81E-01	7.60E-01	5.11E-02	1.67E+00	1.59E-06	1.03E+00	5.52E-03	3.33E-01	8.81E-01
ENSBTAG00000016635	1.67E-01	1.00E+00	6.30E-02	1.00E+00	5.04E-02	1.00E+00	4.22E-01	8.23E-02	-3.41E-01	5.63E-01
ENSBTAG00000034033	-4.07E-01	7.63E-01	-6.23E-01	9.73E-02	-9.28E-01	6.89E-03	-7.92E-01	2.71E-02	-3.41E-01	8.05E-01
ENSBTAG00000042473	0.00E+00	1.00E+00	0.00E+00	1.00E+00	-5.13E-15	1.00E+00	0.00E+00	1.00E+00	0.00E+00	1.00E+00
ENSBTAG00000010951	-1.15E-01	1.00E+00	-9.77E-01	1.29E-02	7.70E-02	1.00E+00	3.90E-01	4.65E-01	7.17E-01	3.68E-01
ENSBTAG00000010949	1.54E-01	1.00E+00	1.15E+00	1.91E-04	1.19E+00	1.01E-04	6.54E-01	5.97E-02	1.93E-01	1.00E+00
ENSBTAG00000018232	1.32E+00	9.29E-01	1.39E+00	5.17E-01	-1.91E+00	4.28E-01	-1.47E-01	1.00E+00	6.29E-03	1.00E+00
ENSBTAG00000017032	2.18E-01	1.00E+00	4.51E-01	7.25E-01	-2.78E-01	9.85E-01	-1.91E-01	1.00E+00	-4.36E-01	1.00E+00
ENSBTAG00000042454	0.00E+00	1.00E+00	0.00E+00	1.00E+00	-5.13E-15	1.00E+00	0.00E+00	1.00E+00	0.00E+00	1.00E+00
ENSBTAG00000009405	-8.54E-01	8.23E-01	1.94E+00	8.75E-04	1.46E+00	1.88E-02	1.50E+00	1.66E-02	-5.75E-01	9.69E-01
ENSBTAG00000012409	2.57E-01	1.00E+00	-3.43E-01	5.04E-01	1.59E-02	1.00E+00	6.79E-01	9.22E-02	-2.43E-01	1.00E+00
ENSBTAG00000011860	1.75E-01	1.00E+00	-5.82E-01	1.64E-02	-9.37E-01	3.70E-05	-1.08E+00	2.05E-06	1.35E-01	1.00E+00
ENSBTAG00000011855	5.59E-02	1.00E+00	6.84E-01	4.00E-03	1.38E+00	4.81E-10	8.68E-01	1.92E-04	-4.24E-01	4.37E-01
ENSBTAG00000011850	3.87E-01	5.79E-01	8.83E-01	8.62E-04	1.05E+00	5.17E-05	9.99E-01	1.62E-04	-8.90E-02	1.00E+00
ENSBTAG00000007589	-2.17E-01	1.00E+00	-1.43E+00	2.95E-02	2.09E-01	1.00E+00	-1.23E+00	7.44E-02	6.67E-01	8.00E-01
ENSBTAG00000005351	6.12E-02	1.00E+00	6.58E-01	2.80E-03	8.23E-01	1.09E-04	9.06E-01	2.21E-05	4.02E-02	1.00E+00
ENSBTAG00000046386	1.30E+00	9.32E-01	3.16E+00	7.49E-03	-1.30E-01	1.00E+00	-1.47E-01	1.00E+00	-1.91E+00	8.00E-01
ENSBTAG00000007284	2.25E+00	4.08E-01	-1.91E+00	4.69E-01	-1.91E+00	4.28E-01	-1.48E-01	1.00E+00	8.24E-01	1.00E+00
ENSBTAG00000008040	-5.94E-04	1.00E+00	9.33E-01	2.84E-04	5.56E-01	4.66E-02	5.31E-01	6.69E-02	-1.60E-01	1.00E+00
ENSBTAG00000018955	-2.48E-01	1.00E+00	-3.18E-01	7.60E-01	-2.15E-01	9.50E-01	-4.16E-03	1.00E+00	2.22E-03	1.00E+00
ENSBTAG00000035493	0.00E+00	1.00E+00	0.00E+00	1.00E+00	-5.13E-15	1.00E+00	0.00E+00	1.00E+00	0.00E+00	1.00E+00
ENSBTAG00000038495	1.58E-02	1.00E+00	-2.41E-01	8.52E-01	-2.02E-01	9.22E-01	-2.68E-01	7.88E-01	-4.46E-01	8.00E-01
ENSBTAG00000022991	-7.86E-02	1.00E+00	-1.77E-01	8.48E-01	-6.76E-01	4.58E-02	-4.64E-01	2.25E-01	-2.14E-01	1.00E+00
ENSBTAG00000034069	0.00E+00	1.00E+00	0.00E+00	1.00E+00	-5.13E-15	1.00E+00	0.00E+00	1.00E+00	0.00E+00	1.00E+00
ENSBTAG00000019268	0.00E+00	1.00E+00	0.00E+00	1.00E+00	-5.13E-15	1.00E+00	0.00E+00	1.00E+00	0.00E+00	1.00E+00
ENSBTAG00000010787	-2.45E-01	8.50E-01	-4.06E-01	1.55E-01	2.28E-01	5.14E-01	2.94E-01	3.66E-01	-5.08E-01	3.08E-01
ENSBTAG00000021815	-1.45E-01	1.00E+00	-1.31E+00	1.33E-05	-2.10E+00	2.25E-12	-1.36E+00	6.99E-06	5.58E-01	4.15E-01
ENSBTAG00000013984	-1.58E+00	1.98E-02	-2.06E+00	1.63E-04	-2.00E+00	1.45E-04	-2.62E+00	2.25E-06	-1.01E+00	3.34E-01
ENSBTAG00000011597	4.27E-03	1.00E+00	-1.97E-01	6.64E-01	1.91E-01	6.72E-01	7.08E-01	6.79E-03	-1.60E-01	1.00E+00
ENSBTAG00000023279	2.48E-01	7.76E-01	1.23E+00	2.64E-10	1.04E+00	9.79E-08	7.76E-01	1.40E-04	-2.63E-01	7.45E-01
ENSBTAG00000000993	-2.04E-01	1.00E+00	-8.78E-01	2.16E-03	-2.35E+00	1.45E-17	-2.91E+00	4.95E-25	-9.79E-02	1.00E+00

ENSBTAG00000000988	-1.09E-01	1.00E+00	-6.95E-01	6.43E-02	-2.03E-01	8.06E-01	1.49E-01	9.55E-01	-3.75E-01	8.00E-01
ENSBTAG00000026384	3.43E-01	9.91E-01	-1.46E+00	1.90E-03	-1.79E+00	9.47E-05	-9.56E-01	5.10E-02	1.49E-01	1.00E+00
ENSBTAG00000006771	1.42E-01	1.00E+00	-1.60E+00	1.59E-13	-1.83E+00	1.90E-17	-1.44E+00	2.79E-11	-5.53E-02	1.00E+00
ENSBTAG00000015132	-1.63E+00	5.89E-01	-2.36E+00	1.02E-01	-9.21E-01	5.27E-01	-2.93E-02	1.00E+00	-1.62E+00	6.15E-01
ENSBTAG00000044236	-8.36E-01	5.24E-01	-1.86E+00	2.86E-03	-3.22E+00	2.51E-06	-1.43E+00	1.49E-02	-1.56E+00	9.18E-02
ENSBTAG00000033412	-4.98E-01	4.20E-01	-1.63E+00	6.58E-08	-1.57E+00	8.98E-08	-1.14E+00	1.10E-04	-5.05E-01	4.64E-01
ENSBTAG00000005012	2.93E+00	9.86E-05	5.55E+00	2.74E-14	2.11E+00	1.62E-03	2.36E+00	4.86E-04	-4.54E-02	1.00E+00
ENSBTAG00000016052	-7.86E-01	1.00E+00	-7.23E-01	9.45E-01	-1.49E-01	1.00E+00	3.19E-01	1.00E+00	-2.71E+00	6.01E-01
ENSBTAG00000033386	-8.70E-01	1.00E+00	9.53E-02	1.00E+00	-1.80E+00	2.96E-01	-1.88E-01	1.00E+00	-3.59E+00	3.09E-01
ENSBTAG00000008271	-1.20E+00	7.83E-01	7.88E-01	5.07E-01	-1.10E+00	4.01E-01	-6.97E-02	1.00E+00	-2.87E+00	2.12E-01
ENSBTAG00000013201	2.79E+00	4.68E-05	3.68E+00	4.86E-11	4.90E+00	1.13E-21	5.40E+00	8.58E-27	3.10E+00	7.13E-06
ENSBTAG00000001178	1.02E+00	1.72E-03	1.99E+00	6.27E-14	1.81E+00	6.73E-12	1.97E+00	1.22E-13	-6.13E-02	1.00E+00
ENSBTAG00000018103	-4.63E-02	1.00E+00	-3.80E-01	1.30E-01	3.24E-03	1.00E+00	-6.64E-02	1.00E+00	-4.77E-01	2.57E-01
ENSBTAG00000009340	1.32E-01	1.00E+00	-1.69E+00	9.84E-05	-8.87E-01	4.92E-02	-3.79E-01	5.46E-01	6.23E-01	5.85E-01
ENSBTAG00000045239	0.00E+00	1.00E+00	0.00E+00	1.00E+00	-5.13E-15	1.00E+00	0.00E+00	1.00E+00	0.00E+00	1.00E+00
ENSBTAG00000012170	9.07E-02	1.00E+00	6.16E-01	4.88E-03	2.31E-01	4.35E-01	-9.18E-02	9.74E-01	-4.39E-01	3.15E-01
ENSBTAG00000018577	7.12E-01	1.96E-01	3.47E+00	8.56E-29	3.19E+00	5.05E-25	2.70E+00	1.66E-18	-6.68E-01	3.92E-01
ENSBTAG00000001094	4.37E-01	1.00E+00	3.45E+00	2.00E-11	1.40E+00	1.12E-02	8.73E-02	1.00E+00	-6.09E-01	8.00E-01
ENSBTAG00000008686	-5.15E-01	8.23E-01	-2.44E+00	1.40E-06	-2.66E+00	1.28E-07	-1.92E+00	1.65E-04	5.38E-01	8.00E-01
ENSBTAG00000014024	7.43E-02	1.00E+00	4.66E-01	3.31E-02	3.46E-01	1.44E-01	4.08E-02	1.00E+00	-6.68E-01	2.08E-02
ENSBTAG00000016915	1.10E+00	1.81E-02	1.66E+00	2.91E-06	1.04E+00	4.87E-03	1.05E+00	5.56E-03	-1.09E-01	1.00E+00
ENSBTAG00000044883	0.00E+00	1.00E+00	0.00E+00	1.00E+00	-5.13E-15	1.00E+00	0.00E+00	1.00E+00	0.00E+00	1.00E+00
ENSBTAG00000009912	9.09E-02	1.00E+00	6.97E-01	6.17E-02	1.66E+00	6.09E-07	6.52E-01	9.33E-02	1.54E-01	1.00E+00
ENSBTAG00000018572	2.07E-01	1.00E+00	-7.86E-01	1.89E-01	6.29E-04	1.00E+00	-3.00E-01	7.90E-01	-3.28E-01	1.00E+00
ENSBTAG00000013819	3.57E-01	4.17E-01	-1.03E+00	3.43E-07	-1.94E+00	3.18E-22	-7.52E-01	3.24E-04	3.54E-01	4.80E-01
ENSBTAG00000001819	2.20E-01	1.00E+00	-2.14E+00	1.65E-01	-1.56E+00	2.72E-01	-2.36E+00	1.06E-01	-2.27E-01	1.00E+00
ENSBTAG00000010690	-6.33E-01	1.00E+00	-4.53E+00	5.96E-03	-1.97E+00	1.05E-01	-4.53E+00	3.24E-03	-9.44E-01	8.37E-01
ENSBTAG00000009166	-1.91E+00	8.23E-01	-1.91E+00	4.69E-01	-1.91E+00	4.28E-01	1.12E+00	6.68E-01	6.49E-03	1.00E+00
ENSBTAG00000008642	-2.18E-02	1.00E+00	1.51E-01	6.80E-01	1.33E-01	7.57E-01	-2.17E-02	1.00E+00	-1.56E-01	9.80E-01
ENSBTAG00000015614	-3.75E-01	5.99E-01	1.09E+00	1.07E-05	7.19E-01	5.39E-03	9.12E-01	3.20E-04	2.17E-01	9.26E-01
ENSBTAG00000020530	-6.73E-02	1.00E+00	-6.78E-01	2.70E-03	-3.50E-01	1.81E-01	-2.09E-01	5.29E-01	8.55E-03	1.00E+00
ENSBTAG00000012600	-2.45E-01	8.23E-01	-5.19E-01	3.26E-02	1.29E-01	8.27E-01	1.49E-01	7.60E-01	-2.42E-02	1.00E+00
ENSBTAG00000005244	1.54E+00	7.22E-02	-2.55E+00	1.11E-04	-1.03E+00	1.48E-01	1.54E+00	1.54E-02	2.44E+00	1.42E-03
ENSBTAG00000001648	-3.91E-02	1.00E+00	6.07E-02	1.00E+00	-1.48E-01	8.43E-01	-3.89E-01	2.34E-01	-4.20E-01	5.49E-01
ENSBTAG00000042408	0.00E+00	1.00E+00	0.00E+00	1.00E+00	-5.13E-15	1.00E+00	0.00E+00	1.00E+00	0.00E+00	1.00E+00

ENSBTAG00000042100	0.00E+00	1.00E+00	0.00E+00	1.00E+00	-5.13E-15	1.00E+00	0.00E+00	1.00E+00	0.00E+00	1.00E+00
ENSBTAG00000017179	3.99E-01	3.76E-01	-6.02E-01	1.02E-02	2.99E-01	2.88E-01	6.76E-01	3.34E-03	2.22E-01	8.33E-01
ENSBTAG00000010769	-1.29E+00	9.36E-01	-1.22E+00	6.15E-01	-3.21E+00	9.50E-02	-3.21E+00	9.97E-02	6.69E-03	1.00E+00
ENSBTAG00000045188	0.00E+00	1.00E+00	0.00E+00	1.00E+00	-5.13E-15	1.00E+00	0.00E+00	1.00E+00	0.00E+00	1.00E+00
ENSBTAG00000014619	-5.42E-01	1.46E-01	-2.94E+00	7.85E-34	-3.11E+00	9.26E-38	-1.96E+00	2.66E-17	4.63E-01	3.53E-01
ENSBTAG00000016737	3.11E-03	1.00E+00	6.03E-01	1.97E-02	1.08E+00	4.96E-06	8.84E-01	3.07E-04	-7.42E-02	1.00E+00
ENSBTAG00000005318	-2.45E-01	8.23E-01	-1.41E+00	2.94E-10	-3.64E-01	1.63E-01	3.72E-01	1.53E-01	2.92E-01	6.96E-01
ENSBTAG00000025428	-1.82E-01	1.00E+00	-3.56E-01	1.00E+00	-6.29E-01	7.50E-01	-2.58E+00	6.11E-02	-1.13E+00	8.00E-01
ENSBTAG00000018214	-5.85E-01	6.36E-01	-3.53E-01	6.02E-01	-6.42E-01	1.99E-01	1.70E-02	1.00E+00	-2.05E+00	6.18E-04
ENSBTAG00000044965	0.00E+00	1.00E+00	0.00E+00	1.00E+00	-5.13E-15	1.00E+00	0.00E+00	1.00E+00	0.00E+00	1.00E+00
ENSBTAG00000016116	1.13E+00	1.45E-01	1.36E+00	7.31E-03	4.68E-01	5.13E-01	7.70E-01	1.99E-01	-2.09E-02	1.00E+00
ENSBTAG00000019529	-1.23E+00	4.85E-03	-3.42E+00	3.21E-18	-3.88E+00	2.23E-22	-3.09E+00	2.75E-16	1.18E+00	1.33E-02
ENSBTAG00000042230	0.00E+00	1.00E+00	0.00E+00	1.00E+00	-5.13E-15	1.00E+00	0.00E+00	1.00E+00	0.00E+00	1.00E+00
ENSBTAG00000015225	-9.55E-02	1.00E+00	1.64E-01	6.14E-01	4.08E-01	5.63E-02	6.20E-01	1.99E-03	-2.40E-01	7.78E-01
ENSBTAG00000003116	-1.14E-01	1.00E+00	-2.00E-01	4.59E-01	1.88E-01	4.82E-01	1.91E-01	4.97E-01	-3.59E-02	1.00E+00
ENSBTAG00000019545	-2.32E-01	9.79E-01	-1.60E+00	1.17E-08	-1.58E+00	1.21E-08	-2.72E-01	5.15E-01	6.07E-01	2.64E-01
ENSBTAG00000001365	-8.69E-01	3.47E-01	-1.86E+00	2.39E-04	-1.84E+00	1.70E-04	-1.09E+00	3.22E-02	-9.97E-02	1.00E+00
ENSBTAG00000008888	1.48E-01	1.00E+00	1.47E-01	7.43E-01	4.03E-01	9.28E-02	1.27E-01	8.27E-01	1.67E-01	9.79E-01
ENSBTAG00000018300	6.32E-01	4.38E-01	-3.60E-01	5.39E-01	-2.45E-01	7.75E-01	-3.67E-01	5.25E-01	5.05E-01	6.81E-01
ENSBTAG00000001867	4.91E-01	5.02E-01	-2.36E-01	6.86E-01	2.02E+00	8.63E-13	2.02E+00	1.29E-12	3.32E-01	8.00E-01
ENSBTAG00000001865	-3.57E-01	1.00E+00	-2.79E-01	1.00E+00	3.96E-01	1.00E+00	-1.84E-01	1.00E+00	-3.44E-01	1.00E+00
ENSBTAG00000048237	-2.75E-03	1.00E+00	-1.90E+00	2.58E-01	-4.72E-01	9.53E-01	-1.99E-01	1.00E+00	-3.88E+00	2.09E-01
ENSBTAG00000015943	1.36E-01	1.00E+00	-1.67E-01	6.04E-01	-2.03E-02	1.00E+00	1.03E-01	8.90E-01	-3.58E-01	4.34E-01
ENSBTAG00000006177	1.82E-01	1.00E+00	-1.73E-01	8.52E-01	3.84E-01	3.27E-01	8.18E-01	1.02E-02	2.41E-01	9.68E-01
ENSBTAG00000003315	-1.30E-01	1.00E+00	-4.32E-01	1.43E-01	-2.96E-01	3.90E-01	-4.40E-01	1.43E-01	-2.55E-01	8.27E-01
ENSBTAG00000003314	-6.56E-03	1.00E+00	1.08E+00	4.50E-03	9.09E-01	1.93E-02	-4.06E-01	4.85E-01	-8.41E-01	3.26E-01
ENSBTAG00000018631	5.11E-02	1.00E+00	-6.61E-02	1.00E+00	1.90E-01	5.44E-01	2.61E-01	3.49E-01	-3.31E-01	5.58E-01
ENSBTAG00000014406	-1.33E-01	1.00E+00	-3.47E-01	3.57E-01	-2.77E-01	4.90E-01	-7.44E-02	1.00E+00	3.65E-02	1.00E+00
ENSBTAG00000003710	-1.14E-02	1.00E+00	1.23E-01	7.91E-01	5.95E-01	2.25E-03	5.87E-01	3.19E-03	-2.24E-01	8.00E-01
ENSBTAG00000008296	3.90E-01	6.35E-01	1.56E+00	1.01E-08	1.51E+00	2.71E-08	5.63E-01	7.60E-02	2.39E-01	9.23E-01
ENSBTAG00000025402	2.01E-02	1.00E+00	-1.36E+00	1.20E-06	-8.75E-01	1.88E-03	-1.67E+00	2.53E-09	2.72E-01	8.27E-01
ENSBTAG00000014286	-4.19E-02	1.00E+00	-1.17E+00	6.27E-07	-8.59E-01	2.66E-04	-8.91E-01	1.85E-04	-8.10E-02	1.00E+00
ENSBTAG00000011726	-1.09E-01	1.00E+00	-1.02E+00	4.08E-06	-1.91E+00	2.52E-18	-2.11E+00	1.27E-21	-4.57E-02	1.00E+00
ENSBTAG00000038662	-1.75E+00	2.45E-01	-8.30E-01	4.46E-01	-3.85E+00	4.26E-04	-1.47E+00	1.14E-01	-6.75E-02	1.00E+00
ENSBTAG00000017425	-5.34E-01	8.85E-01	-1.64E+00	1.58E-02	-1.30E+00	4.23E-02	-5.39E-01	4.97E-01	-8.05E-01	6.45E-01

ENSBTAG00000020400	0.00E+00	1.00E+00	2.79E+00	2.10E-01	2.55E+00	2.54E-01	0.00E+00	1.00E+00	1.93E+00	8.00E-01
ENSBTAG00000020403	3.62E-02	1.00E+00	-8.81E-01	3.70E-05	-4.28E-01	7.39E-02	-3.31E-01	2.13E-01	-6.93E-03	1.00E+00
ENSBTAG00000025383	1.09E-01	1.00E+00	2.51E-02	1.00E+00	-1.73E-02	1.00E+00	7.48E-02	1.00E+00	-3.30E-01	7.93E-01
ENSBTAG00000007167	1.54E-01	1.00E+00	-2.61E-01	3.50E-01	2.07E-01	4.87E-01	-1.95E-01	5.48E-01	1.99E-02	1.00E+00
ENSBTAG00000003934	-9.94E-02	1.00E+00	-1.01E+00	2.56E-03	9.78E-01	3.42E-03	1.04E+00	2.06E-03	-1.05E+00	2.54E-02
ENSBTAG00000003922	5.28E-01	2.94E-01	1.56E+00	2.60E-09	1.37E+00	1.56E-07	8.47E-01	2.39E-03	2.35E-01	9.15E-01
ENSBTAG00000025400	7.11E-02	1.00E+00	-1.22E+00	1.23E-05	2.39E-01	5.84E-01	4.52E-01	1.74E-01	2.68E-01	8.48E-01
ENSBTAG00000045099	0.00E+00	1.00E+00	0.00E+00	1.00E+00	-5.13E-15	1.00E+00	0.00E+00	1.00E+00	0.00E+00	1.00E+00
ENSBTAG00000017048	2.22E-02	1.00E+00	-1.32E+00	2.56E-04	8.62E-01	6.97E-03	1.09E+00	4.49E-04	4.46E-01	6.45E-01
ENSBTAG00000000278	-3.81E-01	1.00E+00	4.22E-01	8.96E-01	-3.25E-01	1.00E+00	1.22E+00	1.51E-01	-1.80E+00	5.25E-01
ENSBTAG00000014486	1.19E+00	8.57E-01	1.52E+00	2.99E-01	7.11E-01	8.33E-01	-1.72E-01	1.00E+00	1.20E+00	8.27E-01
ENSBTAG00000014274	0.00E+00	1.00E+00	0.00E+00	1.00E+00	-5.13E-15	1.00E+00	3.40E+00	8.35E-02	0.00E+00	1.00E+00
ENSBTAG00000045045	0.00E+00	1.00E+00	0.00E+00	1.00E+00	-5.13E-15	1.00E+00	0.00E+00	1.00E+00	0.00E+00	1.00E+00
ENSBTAG00000032984	0.00E+00	1.00E+00	0.00E+00	1.00E+00	-5.13E-15	1.00E+00	0.00E+00	1.00E+00	0.00E+00	1.00E+00
ENSBTAG00000047831	0.00E+00	1.00E+00	0.00E+00	1.00E+00	-5.13E-15	1.00E+00	0.00E+00	1.00E+00	0.00E+00	1.00E+00
ENSBTAG00000045687	0.00E+00	1.00E+00	0.00E+00	1.00E+00	-5.13E-15	1.00E+00	0.00E+00	1.00E+00	0.00E+00	1.00E+00
ENSBTAG00000019340	-4.98E-01	9.62E-01	1.09E+00	5.24E-02	-4.13E-01	7.02E-01	8.91E-01	1.40E-01	-1.11E+00	4.51E-01
ENSBTAG00000032979	-1.95E-01	1.00E+00	1.44E+00	8.24E-04	9.98E-01	3.36E-02	4.46E-01	5.17E-01	1.57E-01	1.00E+00
ENSBTAG00000008647	2.39E-01	1.00E+00	-2.96E-02	1.00E+00	-4.81E-01	7.70E-01	8.44E-01	3.69E-01	-1.27E+00	5.72E-01
ENSBTAG00000046715	0.00E+00	1.00E+00	0.00E+00	1.00E+00	-5.13E-15	1.00E+00	0.00E+00	1.00E+00	0.00E+00	1.00E+00
ENSBTAG00000046942	0.00E+00	1.00E+00	0.00E+00	1.00E+00	-5.13E-15	1.00E+00	0.00E+00	1.00E+00	0.00E+00	1.00E+00
ENSBTAG00000042849	0.00E+00	1.00E+00	0.00E+00	1.00E+00	-5.13E-15	1.00E+00	0.00E+00	1.00E+00	0.00E+00	1.00E+00
ENSBTAG00000043985	6.16E-01	1.00E+00	5.89E-01	7.18E-01	-3.84E-01	9.77E-01	-1.88E-01	1.00E+00	1.89E-01	1.00E+00
ENSBTAG00000047390	0.00E+00	1.00E+00	0.00E+00	1.00E+00	-5.13E-15	1.00E+00	0.00E+00	1.00E+00	0.00E+00	1.00E+00
ENSBTAG00000025028	-1.90E-01	1.00E+00	5.45E-01	7.88E-02	8.24E-01	3.04E-03	8.12E-01	4.19E-03	-1.71E-01	1.00E+00
ENSBTAG00000019886	-6.47E-01	2.29E-01	-1.39E+00	6.75E-06	-9.94E-01	1.58E-03	-1.08E+00	6.89E-04	-1.33E-01	1.00E+00
ENSBTAG00000019889	-1.89E-01	1.00E+00	9.09E-01	1.38E-04	1.03E+00	1.06E-05	6.48E-01	1.08E-02	-6.04E-02	1.00E+00
ENSBTAG00000019895	2.32E-01	8.85E-01	1.23E+00	7.44E-08	1.24E+00	4.95E-08	6.59E-01	8.41E-03	-2.51E-01	8.12E-01
ENSBTAG00000002129	-1.94E-01	1.00E+00	-3.12E-02	1.00E+00	2.38E-01	9.31E-01	3.01E-01	8.23E-01	-6.25E-01	7.79E-01
ENSBTAG00000042943	0.00E+00	1.00E+00	0.00E+00	1.00E+00	-5.13E-15	1.00E+00	0.00E+00	1.00E+00	0.00E+00	1.00E+00
ENSBTAG00000044007	4.23E-02	1.00E+00	-1.29E+00	4.85E-06	-2.44E+00	1.70E-17	-1.07E+00	1.80E-04	1.46E-01	1.00E+00
ENSBTAG00000042618	0.00E+00	1.00E+00	0.00E+00	1.00E+00	-5.13E-15	1.00E+00	0.00E+00	1.00E+00	0.00E+00	1.00E+00
ENSBTAG00000005760	9.46E-01	2.11E-02	1.36E+00	1.01E-05	1.56E+00	2.28E-07	1.48E+00	1.35E-06	-1.64E-01	1.00E+00
ENSBTAG00000008024	-1.63E-01	1.00E+00	8.95E-01	1.74E-04	1.12E+00	1.47E-06	9.60E-01	6.03E-05	-4.33E-01	4.42E-01
ENSBTAG00000010680	1.12E-02	1.00E+00	-1.91E+00	4.69E-01	-1.91E+00	4.28E-01	-1.47E-01	1.00E+00	-1.91E+00	8.00E-01

ENSBTAG00000010693	1.75E-01	1.00E+00	-1.89E+00	1.18E-09	-2.42E+00	6.61E-15	-6.58E-01	6.21E-02	3.00E-02	1.00E+00
ENSBTAG00000045253	0.00E+00	1.00E+00	0.00E+00	1.00E+00	-5.13E-15	1.00E+00	0.00E+00	1.00E+00	0.00E+00	1.00E+00
ENSBTAG00000032878	0.00E+00	1.00E+00	0.00E+00	1.00E+00	-5.13E-15	1.00E+00	0.00E+00	1.00E+00	0.00E+00	1.00E+00
ENSBTAG00000042089	0.00E+00	1.00E+00	0.00E+00	1.00E+00	-5.13E-15	1.00E+00	0.00E+00	1.00E+00	0.00E+00	1.00E+00
ENSBTAG00000001414	3.33E-01	9.63E-01	1.21E+00	1.95E-03	1.51E+00	6.39E-05	1.28E+00	1.03E-03	-2.15E-02	1.00E+00
ENSBTAG00000046640	5.70E-01	1.00E+00	1.27E+00	1.73E-01	1.42E+00	9.07E-02	6.50E-01	6.61E-01	-2.45E-01	1.00E+00
ENSBTAG00000044957	0.00E+00	1.00E+00	0.00E+00	1.00E+00	-5.13E-15	1.00E+00	0.00E+00	1.00E+00	0.00E+00	1.00E+00
ENSBTAG00000008259	4.92E+00	4.36E-39	4.07E+00	1.12E-28	1.66E+00	1.34E-05	1.47E+00	1.89E-04	6.73E-01	5.26E-01
ENSBTAG00000018846	-1.31E-01	1.00E+00	-6.93E-01	5.43E-04	-8.36E-01	1.94E-05	-4.40E-01	4.60E-02	-2.78E-01	6.48E-01
ENSBTAG00000018848	-4.51E-01	2.29E-01	-1.35E+00	2.02E-10	-9.07E-01	2.08E-05	-5.49E-01	1.77E-02	-1.70E-01	9.70E-01
ENSBTAG00000018851	-5.22E-02	1.00E+00	-6.73E-01	3.20E-03	-6.29E-01	6.23E-03	-5.97E-01	1.17E-02	5.57E-02	1.00E+00
ENSBTAG00000032821	1.35E-02	1.00E+00	-9.83E-01	1.04E-01	-1.01E+00	8.82E-02	-7.91E-01	2.24E-01	1.20E-01	1.00E+00
ENSBTAG00000043371	0.00E+00	1.00E+00	0.00E+00	1.00E+00	-5.13E-15	1.00E+00	0.00E+00	1.00E+00	0.00E+00	1.00E+00
ENSBTAG00000003511	6.52E-01	5.53E-01	-3.26E-01	7.11E-01	1.34E+00	1.00E-03	1.34E+00	1.27E-03	5.27E-01	7.63E-01
ENSBTAG00000044565	0.00E+00	1.00E+00	0.00E+00	1.00E+00	-5.13E-15	1.00E+00	0.00E+00	1.00E+00	0.00E+00	1.00E+00
ENSBTAG00000005299	1.31E-01	1.00E+00	-4.08E-01	1.27E-01	-1.51E-01	7.66E-01	8.76E-01	1.20E-04	-4.08E-01	4.57E-01
ENSBTAG00000000869	-2.92E-01	1.00E+00	4.23E-01	6.16E-01	7.20E-01	2.48E-01	2.91E-01	8.46E-01	-6.33E-01	7.82E-01
ENSBTAG00000043485	0.00E+00	1.00E+00	0.00E+00	1.00E+00	-5.13E-15	1.00E+00	0.00E+00	1.00E+00	0.00E+00	1.00E+00
ENSBTAG00000043303	0.00E+00	1.00E+00	0.00E+00	1.00E+00	-5.13E-15	1.00E+00	0.00E+00	1.00E+00	0.00E+00	1.00E+00
ENSBTAG00000042561	0.00E+00	1.00E+00	0.00E+00	1.00E+00	-5.13E-15	1.00E+00	0.00E+00	1.00E+00	0.00E+00	1.00E+00
ENSBTAG00000017573	-5.92E-02	1.00E+00	1.56E-01	6.78E-01	3.44E-01	1.51E-01	2.66E-01	3.26E-01	6.75E-02	1.00E+00
ENSBTAG00000014227	9.26E-01	2.57E-03	2.93E+00	2.46E-30	2.50E+00	3.18E-23	1.59E+00	2.73E-10	-1.13E-01	1.00E+00
ENSBTAG00000032727	1.92E+00	8.23E-01	2.79E+00	2.10E-01	-5.13E-15	1.00E+00	0.00E+00	1.00E+00	0.00E+00	1.00E+00
ENSBTAG00000001774	4.98E-01	3.71E-01	7.53E-01	7.80E-03	3.87E-01	2.54E-01	-6.21E-02	1.00E+00	-3.93E-01	6.26E-01
ENSBTAG00000045289	0.00E+00	1.00E+00	0.00E+00	1.00E+00	-5.13E-15	1.00E+00	0.00E+00	1.00E+00	0.00E+00	1.00E+00
ENSBTAG00000020684	-1.56E-01	1.00E+00	-3.48E-01	5.35E-01	1.50E-01	9.92E-01	4.66E-01	3.51E-01	-2.33E-01	1.00E+00
ENSBTAG00000039497	0.00E+00	1.00E+00	0.00E+00	1.00E+00	-5.13E-15	1.00E+00	0.00E+00	1.00E+00	0.00E+00	1.00E+00
ENSBTAG00000045950	0.00E+00	1.00E+00	0.00E+00	1.00E+00	-5.13E-15	1.00E+00	0.00E+00	1.00E+00	0.00E+00	1.00E+00
ENSBTAG00000047391	0.00E+00	1.00E+00	1.98E+00	4.43E-01	-5.13E-15	1.00E+00	1.76E+00	4.97E-01	0.00E+00	1.00E+00
ENSBTAG00000045992	0.00E+00	1.00E+00	0.00E+00	1.00E+00	-5.13E-15	1.00E+00	0.00E+00	1.00E+00	0.00E+00	1.00E+00
ENSBTAG00000021503	2.62E-01	1.00E+00	9.85E-02	1.00E+00	7.20E-01	1.43E-01	8.41E-01	7.65E-02	-3.38E-01	1.00E+00
ENSBTAG00000038168	0.00E+00	1.00E+00	1.99E+00	4.43E-01	1.78E+00	4.79E-01	0.00E+00	1.00E+00	0.00E+00	1.00E+00
ENSBTAG00000042992	0.00E+00	1.00E+00	0.00E+00	1.00E+00	-5.13E-15	1.00E+00	0.00E+00	1.00E+00	0.00E+00	1.00E+00
ENSBTAG00000004388	1.19E+00	8.56E-01	1.28E+00	4.43E-01	-2.70E+00	1.99E-01	-2.70E+00	2.05E-01	2.01E+00	4.01E-01
ENSBTAG00000032711	2.32E-01	1.00E+00	3.09E-02	1.00E+00	2.63E-01	7.19E-01	7.95E-01	4.72E-02	1.57E-01	1.00E+00

ENSBTAG00000010129	8.04E-01	1.00E+00	-1.91E+00	4.69E-01	-1.91E+00	4.28E-01	-1.91E+00	4.39E-01	-1.91E+00	8.00E-01
ENSBTAG00000047066	0.00E+00	1.00E+00	0.00E+00	1.00E+00	-5.13E-15	1.00E+00	0.00E+00	1.00E+00	0.00E+00	1.00E+00
ENSBTAG00000029951	0.00E+00	1.00E+00	3.30E+00	1.02E-01	2.55E+00	2.54E-01	0.00E+00	1.00E+00	0.00E+00	1.00E+00
ENSBTAG00000029847	0.00E+00	1.00E+00	0.00E+00	1.00E+00	3.95E+00	2.31E-02	1.76E+00	4.97E-01	0.00E+00	1.00E+00
ENSBTAG00000029764	0.00E+00	1.00E+00	0.00E+00	1.00E+00	3.42E+00	7.39E-02	1.77E+00	4.97E-01	0.00E+00	1.00E+00
ENSBTAG00000029860	0.00E+00	1.00E+00	0.00E+00	1.00E+00	1.78E+00	4.79E-01	1.76E+00	4.97E-01	0.00E+00	1.00E+00
ENSBTAG00000029948	3.59E+00	2.30E-01	3.30E+00	1.02E-01	5.48E+00	3.61E-05	3.03E+00	1.50E-01	1.91E+00	8.00E-01
ENSBTAG00000029802	-7.78E-01	7.52E-01	1.15E+00	4.01E-02	2.07E+00	1.32E-05	-7.73E-02	1.00E+00	-9.64E-01	6.01E-01
ENSBTAG00000008708	-2.80E-01	1.00E+00	1.90E-01	9.85E-01	-4.50E-01	5.01E-01	1.69E-01	1.00E+00	-9.36E-01	3.69E-01
ENSBTAG00000043931	0.00E+00	1.00E+00	0.00E+00	1.00E+00	-5.13E-15	1.00E+00	0.00E+00	1.00E+00	0.00E+00	1.00E+00
ENSBTAG00000025307	-6.19E-04	1.00E+00	-1.91E+00	4.69E-01	-1.91E+00	4.28E-01	-1.91E+00	4.39E-01	3.84E-04	1.00E+00
ENSBTAG00000040347	2.69E+00	4.41E-05	4.91E+00	7.42E-15	2.93E+00	6.93E-07	1.83E+00	2.48E-03	-4.09E-01	1.00E+00
ENSBTAG00000045195	0.00E+00	1.00E+00	0.00E+00	1.00E+00	-5.13E-15	1.00E+00	0.00E+00	1.00E+00	0.00E+00	1.00E+00
ENSBTAG00000045317	0.00E+00	1.00E+00	0.00E+00	1.00E+00	-5.13E-15	1.00E+00	0.00E+00	1.00E+00	0.00E+00	1.00E+00
ENSBTAG00000002300	-1.29E+00	9.36E-01	8.78E-02	1.00E+00	2.15E-01	1.00E+00	-1.88E-01	1.00E+00	-5.07E-01	1.00E+00
ENSBTAG00000047008	8.03E-01	2.26E-01	3.29E+00	2.58E-17	2.67E+00	2.18E-12	2.04E+00	9.76E-08	3.27E-01	9.23E-01
ENSBTAG00000047005	0.00E+00	1.00E+00	0.00E+00	1.00E+00	-5.13E-15	1.00E+00	0.00E+00	1.00E+00	0.00E+00	1.00E+00
ENSBTAG00000005309	-4.92E-01	2.18E-01	-2.19E+00	1.06E-21	-1.96E+00	3.22E-18	-1.63E+00	5.03E-13	-3.05E-01	7.10E-01
ENSBTAG00000005310	-3.42E-01	8.37E-01	-8.82E-01	1.57E-02	-2.39E-01	6.96E-01	3.63E-01	4.23E-01	-3.42E-01	8.16E-01
ENSBTAG00000036625	-6.19E-04	1.00E+00	-1.91E+00	4.69E-01	-1.91E+00	4.28E-01	6.14E-01	9.97E-01	-1.91E+00	8.00E-01
ENSBTAG00000022484	0.00E+00	1.00E+00	0.00E+00	1.00E+00	-5.13E-15	1.00E+00	1.76E+00	4.97E-01	0.00E+00	1.00E+00
ENSBTAG00000032603	7.86E-01	6.66E-01	1.40E+00	2.10E-02	1.12E+00	8.07E-02	1.53E+00	1.15E-02	-1.63E-01	1.00E+00
ENSBTAG00000047383	-7.90E-02	1.00E+00	1.42E-01	1.00E+00	5.82E-02	1.00E+00	-6.16E-01	5.32E-01	-2.97E-01	1.00E+00
ENSBTAG00000045751	9.53E-01	1.00E+00	1.12E-01	1.00E+00	-2.46E-01	1.00E+00	-3.42E-01	1.00E+00	3.81E-01	1.00E+00
ENSBTAG00000047181	-4.29E-01	1.00E+00	4.36E-01	8.10E-01	4.95E-01	6.98E-01	-3.42E-01	9.55E-01	-8.21E-01	8.38E-01
ENSBTAG00000046041	1.37E-01	1.00E+00	5.41E-01	4.43E-01	1.94E-01	1.00E+00	-3.64E-01	7.29E-01	-4.71E-01	9.01E-01
ENSBTAG00000047764	1.48E-01	1.00E+00	-4.62E-01	9.74E-01	-4.31E-01	9.95E-01	-2.58E-01	1.00E+00	-1.84E-01	1.00E+00
ENSBTAG00000047360	0.00E+00	1.00E+00	0.00E+00	1.00E+00	-5.13E-15	1.00E+00	0.00E+00	1.00E+00	1.91E+00	8.00E-01
ENSBTAG00000023309	-1.23E-01	1.00E+00	-4.26E-02	1.00E+00	-6.43E-01	2.58E-01	1.35E-02	1.00E+00	-8.06E-01	4.90E-01
ENSBTAG00000026070	-2.58E-01	1.00E+00	5.62E-01	4.25E-01	-1.97E-02	1.00E+00	-4.61E-01	5.95E-01	-9.74E-01	4.90E-01
ENSBTAG00000039714	8.78E-01	7.94E-01	1.00E+00	2.88E-01	8.07E-01	4.31E-01	1.08E-01	1.00E+00	1.86E-01	1.00E+00
ENSBTAG00000003568	-1.70E-01	1.00E+00	-6.83E-01	6.34E-01	-8.80E-02	1.00E+00	1.08E+00	1.90E-01	-1.54E-01	1.00E+00
ENSBTAG00000003569	2.16E-02	1.00E+00	-2.58E-01	7.02E-01	-6.15E-01	1.33E-01	-4.06E-01	4.12E-01	-2.61E-01	1.00E+00
ENSBTAG00000012065	6.61E-01	4.79E-02	2.82E+00	1.76E-32	2.83E+00	4.72E-33	2.56E+00	1.49E-27	4.97E-01	3.11E-01
ENSBTAG00000004401	1.35E-01	1.00E+00	2.41E+00	1.89E-05	5.61E-01	4.94E-01	-7.23E-02	1.00E+00	1.48E-01	1.00E+00

ENSBTAG00000047881	-7.87E-01	1.00E+00	-7.23E-01	9.45E-01	-9.24E-01	8.08E-01	3.25E-01	1.00E+00	-2.71E+00	6.01E-01
ENSBTAG00000039065	0.00E+00	1.00E+00	0.00E+00	1.00E+00	1.78E+00	4.79E-01	0.00E+00	1.00E+00	0.00E+00	1.00E+00
ENSBTAG00000045566	-2.96E-01	8.69E-01	-4.82E-01	1.83E-01	-2.84E-01	5.16E-01	-4.56E-01	2.17E-01	-1.24E+00	1.96E-03
ENSBTAG00000021345	-2.65E-01	1.00E+00	-3.99E+00	3.25E-03	-4.21E+00	1.35E-03	-2.61E+00	2.89E-02	4.02E-01	1.00E+00
ENSBTAG00000018313	9.49E-02	1.00E+00	2.05E-01	5.70E-01	-3.24E-01	2.60E-01	-2.68E-01	4.11E-01	1.81E-01	9.75E-01
ENSBTAG00000020412	2.75E-01	1.00E+00	-3.90E-02	1.00E+00	1.53E-01	1.00E+00	7.98E-01	7.11E-02	-6.58E-01	6.11E-01
ENSBTAG00000047456	0.00E+00	1.00E+00	0.00E+00	1.00E+00	-5.13E-15	1.00E+00	0.00E+00	1.00E+00	0.00E+00	1.00E+00
ENSBTAG00000045512	0.00E+00	1.00E+00	0.00E+00	1.00E+00	-5.13E-15	1.00E+00	0.00E+00	1.00E+00	0.00E+00	1.00E+00
ENSBTAG00000046479	0.00E+00	1.00E+00	0.00E+00	1.00E+00	-5.13E-15	1.00E+00	0.00E+00	1.00E+00	0.00E+00	1.00E+00
ENSBTAG00000039374	-7.47E-02	1.00E+00	4.15E-01	6.73E-02	7.04E-01	5.88E-04	5.83E-01	6.59E-03	-1.83E-01	9.02E-01
ENSBTAG00000000146	-1.66E-01	1.00E+00	-1.16E+00	2.45E-08	-2.34E+00	3.39E-29	-2.43E+00	4.49E-31	2.34E-01	8.00E-01
ENSBTAG00000045262	0.00E+00	1.00E+00	0.00E+00	1.00E+00	-5.13E-15	1.00E+00	0.00E+00	1.00E+00	0.00E+00	1.00E+00
ENSBTAG00000032234	1.56E-01	1.00E+00	1.00E-01	9.85E-01	3.29E-01	2.65E-01	1.12E-01	9.40E-01	7.80E-02	1.00E+00
ENSBTAG00000004440	-6.43E-01	5.76E-01	-1.81E+00	7.62E-05	-2.10E+00	3.54E-06	-2.58E+00	2.97E-08	-7.40E-01	4.90E-01
ENSBTAG00000010395	-1.11E-01	1.00E+00	1.09E+00	1.49E-01	2.73E+00	3.94E-05	1.94E+00	4.60E-03	5.54E-02	1.00E+00
ENSBTAG00000005682	9.41E-02	1.00E+00	-4.31E-01	1.26E-01	-6.02E-01	2.04E-02	-3.12E-01	3.37E-01	-6.76E-02	1.00E+00
ENSBTAG00000002240	0.00E+00	1.00E+00	0.00E+00	1.00E+00	-5.13E-15	1.00E+00	0.00E+00	1.00E+00	0.00E+00	1.00E+00
ENSBTAG00000032084	0.00E+00	1.00E+00	0.00E+00	1.00E+00	-5.13E-15	1.00E+00	0.00E+00	1.00E+00	0.00E+00	1.00E+00
ENSBTAG00000038268	-4.96E-01	1.00E+00	-1.22E+00	6.15E-01	-6.54E-01	8.97E-01	-3.21E+00	9.97E-02	7.51E-03	1.00E+00
ENSBTAG00000001589	2.60E-01	8.10E-01	4.65E-01	5.67E-02	4.50E-01	6.77E-02	4.61E-01	6.55E-02	1.47E-01	1.00E+00
ENSBTAG00000004165	-2.92E-02	1.00E+00	-7.48E-01	3.83E-03	-1.17E+00	2.25E-06	-1.22E+00	1.05E-06	-1.42E-01	1.00E+00
ENSBTAG00000046271	0.00E+00	1.00E+00	2.79E+00	2.10E-01	-5.13E-15	1.00E+00	0.00E+00	1.00E+00	1.93E+00	8.00E-01
ENSBTAG00000025246	1.38E-02	1.00E+00	-1.91E+00	4.69E-01	-1.91E+00	4.28E-01	1.50E+00	4.50E-01	1.68E+00	7.79E-01
ENSBTAG00000010672	1.66E-02	1.00E+00	-4.24E-01	1.38E-01	-1.28E+00	9.51E-08	-1.60E+00	2.85E-11	2.88E-01	8.00E-01
ENSBTAG00000043343	0.00E+00	1.00E+00	0.00E+00	1.00E+00	-5.13E-15	1.00E+00	0.00E+00	1.00E+00	0.00E+00	1.00E+00
ENSBTAG00000046552	-2.02E-01	9.89E-01	-1.04E+00	2.96E-05	-1.57E+00	9.65E-11	-1.36E+00	3.26E-08	1.56E-01	1.00E+00
ENSBTAG00000007066	1.46E-01	1.00E+00	-7.19E-01	3.65E-02	1.19E+00	1.17E-04	1.52E+00	8.20E-07	-1.74E-01	1.00E+00
ENSBTAG00000045721	-8.80E-04	1.00E+00	-4.29E-01	1.00E+00	1.30E+00	2.98E-01	1.67E+00	1.24E-01	3.79E-01	1.00E+00
ENSBTAG00000037786	-2.79E-01	1.00E+00	-2.96E-01	9.58E-01	-9.88E-01	2.54E-01	-6.50E-02	1.00E+00	-1.18E+00	5.46E-01
ENSBTAG00000044027	2.96E-01	1.00E+00	-6.73E-01	6.19E-01	-4.40E-01	8.29E-01	-1.25E-01	1.00E+00	1.33E+00	2.98E-01
ENSBTAG00000045920	0.00E+00	1.00E+00	0.00E+00	1.00E+00	-5.13E-15	1.00E+00	0.00E+00	1.00E+00	0.00E+00	1.00E+00
ENSBTAG00000020389	2.50E-01	7.31E-01	-4.41E-01	3.67E-02	-5.39E-01	7.75E-03	-6.03E-02	1.00E+00	-1.70E-02	1.00E+00
ENSBTAG00000020393	2.53E+00	2.45E-02	1.41E+00	2.58E-01	-1.52E-01	1.00E+00	1.94E-01	1.00E+00	1.91E+00	3.17E-01
ENSBTAG00000017515	3.40E-01	1.00E+00	2.27E+00	1.68E-08	1.58E+00	1.28E-04	-2.08E-01	9.41E-01	-3.33E-01	1.00E+00
ENSBTAG00000025221	-2.50E-01	9.93E-01	5.85E-01	8.68E-02	4.89E-01	1.75E-01	3.31E-01	4.48E-01	-3.54E-01	8.00E-01

ENSBTAG00000008880	2.49E-01	1.00E+00	1.10E+00	3.94E-04	1.75E+00	5.29E-09	1.82E+00	1.94E-09	3.06E-01	8.44E-01
ENSBTAG00000014043	-2.05E-02	1.00E+00	3.26E-01	1.10E-01	5.47E-01	2.53E-03	1.48E-01	6.27E-01	2.07E-02	1.00E+00
ENSBTAG00000001344	1.69E+00	7.71E-01	2.07E+00	1.95E-01	2.26E+00	1.13E-01	2.42E+00	8.34E-02	1.32E+00	8.97E-01
ENSBTAG00000005925	1.10E+00	5.65E-01	1.75E+00	1.36E-02	1.09E+00	1.99E-01	1.29E+00	1.05E-01	7.06E-01	8.68E-01
ENSBTAG00000045408	0.00E+00	1.00E+00	0.00E+00	1.00E+00	-5.13E-15	1.00E+00	0.00E+00	1.00E+00	0.00E+00	1.00E+00
ENSBTAG00000046889	0.00E+00	1.00E+00	0.00E+00	1.00E+00	-5.13E-15	1.00E+00	0.00E+00	1.00E+00	0.00E+00	1.00E+00
ENSBTAG00000017538	0.00E+00	1.00E+00	0.00E+00	1.00E+00	-5.13E-15	1.00E+00	0.00E+00	1.00E+00	0.00E+00	1.00E+00
ENSBTAG00000016991	2.92E-01	9.80E-01	6.71E-02	1.00E+00	1.07E-01	1.00E+00	-2.39E-01	7.66E-01	-3.30E-01	8.99E-01
ENSBTAG00000015124	-1.59E-01	1.00E+00	-3.42E-01	1.91E-01	-1.11E-01	8.97E-01	-7.16E-01	1.38E-03	-2.13E-01	8.44E-01
ENSBTAG00000025220	2.82E-01	9.48E-01	-1.72E-01	8.92E-01	-3.46E-01	4.60E-01	1.59E-01	9.22E-01	-4.93E-01	6.03E-01
ENSBTAG00000032038	0.00E+00	1.00E+00	0.00E+00	1.00E+00	-5.13E-15	1.00E+00	0.00E+00	1.00E+00	0.00E+00	1.00E+00
ENSBTAG00000015868	4.58E-01	2.11E-01	1.56E+00	1.81E-14	1.06E+00	2.26E-07	6.44E-01	3.69E-03	9.12E-02	1.00E+00
ENSBTAG00000002432	3.03E-01	7.34E-01	1.22E+00	4.87E-08	1.27E+00	8.12E-09	1.05E+00	4.39E-06	3.08E-01	7.21E-01
ENSBTAG00000045907	1.34E+00	6.82E-02	2.64E+00	3.15E-09	1.42E+00	6.18E-03	5.77E-01	4.72E-01	2.47E-04	1.00E+00
ENSBTAG00000011563	1.88E+00	8.98E-07	2.46E+00	4.06E-13	9.36E-01	2.17E-02	-6.62E-01	2.37E-01	-1.22E+00	1.52E-01
ENSBTAG00000018237	1.75E-01	1.00E+00	5.58E-01	4.74E-01	-2.93E-01	8.99E-01	8.36E-01	1.92E-01	2.08E-01	1.00E+00
ENSBTAG00000012019	2.66E+00	1.22E-07	3.37E+00	1.32E-12	4.67E+00	3.58E-21	3.93E+00	4.15E-16	1.93E+00	1.15E-03
ENSBTAG00000043755	0.00E+00	1.00E+00	0.00E+00	1.00E+00	-5.13E-15	1.00E+00	0.00E+00	1.00E+00	0.00E+00	1.00E+00
ENSBTAG00000045170	0.00E+00	1.00E+00	0.00E+00	1.00E+00	-5.13E-15	1.00E+00	0.00E+00	1.00E+00	0.00E+00	1.00E+00
ENSBTAG00000012849	3.08E-01	6.54E-01	6.78E-01	3.04E-03	4.50E-01	7.02E-02	6.29E-01	7.58E-03	7.25E-02	1.00E+00
ENSBTAG00000025210	7.74E-02	1.00E+00	4.94E-01	1.13E-01	1.70E-01	7.94E-01	2.32E-01	6.08E-01	1.24E-01	1.00E+00
ENSBTAG00000031962	1.20E+00	5.63E-05	3.10E+00	1.91E-30	1.84E+00	2.37E-12	5.82E-01	5.57E-02	2.50E-01	8.94E-01
ENSBTAG00000011466	-1.89E-01	9.32E-01	-1.55E-01	6.98E-01	-5.02E-01	2.50E-02	-4.72E-01	4.19E-02	1.75E-01	9.39E-01
ENSBTAG00000011471	-9.92E-02	1.00E+00	-1.01E+00	4.86E-05	-1.04E+00	2.45E-05	-1.05E+00	2.57E-05	2.20E-01	9.07E-01
ENSBTAG00000004041	2.88E-01	7.76E-01	-2.60E-02	1.00E+00	1.06E-01	9.56E-01	-5.10E-01	5.46E-02	-2.48E-01	8.02E-01
ENSBTAG00000025211	-4.46E-01	4.55E-01	-9.31E-01	6.21E-04	-2.95E-01	4.27E-01	2.73E-02	1.00E+00	1.33E-02	1.00E+00
ENSBTAG00000020726	-2.13E-01	8.23E-01	-6.80E-01	9.30E-04	-5.92E-01	4.40E-03	-6.02E-01	4.40E-03	3.31E-01	5.24E-01
ENSBTAG00000007298	-1.66E+00	7.81E-01	-3.58E+00	6.71E-02	-5.22E-01	9.45E-01	-1.82E-01	1.00E+00	-8.75E-01	1.00E+00
ENSBTAG00000047887	0.00E+00	1.00E+00	0.00E+00	1.00E+00	-5.13E-15	1.00E+00	0.00E+00	1.00E+00	0.00E+00	1.00E+00
ENSBTAG00000038272	1.92E+00	8.23E-01	1.98E+00	4.43E-01	3.06E+00	1.36E-01	2.53E+00	2.71E-01	0.00E+00	1.00E+00
ENSBTAG00000044184	-1.47E-01	1.00E+00	2.37E-01	4.43E-01	5.70E-01	1.15E-02	1.98E-01	5.59E-01	-2.32E-02	1.00E+00
ENSBTAG00000000446	-2.22E-01	1.00E+00	-1.84E-01	7.95E-01	1.94E-01	7.55E-01	-5.95E-01	7.27E-02	4.56E-02	1.00E+00
ENSBTAG00000017289	-9.01E-02	1.00E+00	-7.67E-01	1.46E-02	-9.89E-01	1.04E-03	-9.09E-01	3.44E-03	2.73E-01	8.86E-01
ENSBTAG00000007411	-2.72E-02	1.00E+00	-4.95E-01	1.53E-01	-3.69E-01	3.41E-01	-6.39E-01	5.23E-02	3.15E-01	8.00E-01
ENSBTAG00000016385	-1.15E-01	1.00E+00	-6.66E-01	1.70E-03	-1.38E+00	6.03E-12	-1.75E+00	4.57E-18	1.19E-01	1.00E+00

ENSBTAG00000019440	1.10E-01	1.00E+00	-1.65E-01	6.99E-01	-5.31E-02	1.00E+00	-4.90E-01	4.93E-02	3.62E-01	5.43E-01
ENSBTAG00000021725	3.98E-04	1.00E+00	-3.32E-01	2.09E-01	-6.52E-01	3.35E-03	-1.09E+00	3.61E-07	2.80E-01	7.27E-01
ENSBTAG00000020093	-3.31E-02	1.00E+00	2.86E-01	2.57E-01	5.22E-01	1.37E-02	4.33E-01	5.56E-02	1.43E-01	1.00E+00
ENSBTAG00000010242	-9.76E-02	1.00E+00	-3.06E-01	2.95E-01	-8.97E-01	7.47E-05	-1.13E+00	5.82E-07	-2.58E-01	8.00E-01
ENSBTAG00000010246	-1.37E-01	1.00E+00	-1.72E+00	7.02E-07	-1.75E+00	3.84E-07	-1.27E+00	3.59E-04	3.71E-01	8.00E-01
ENSBTAG00000045250	0.00E+00	1.00E+00	0.00E+00	1.00E+00	1.78E+00	4.79E-01	0.00E+00	1.00E+00	1.93E+00	8.00E-01
ENSBTAG00000020928	2.41E-01	1.00E+00	-4.58E-01	7.92E-01	-9.20E-01	3.37E-01	-1.22E+00	1.76E-01	9.52E-01	6.57E-01
ENSBTAG00000018671	-2.54E-01	1.00E+00	8.27E-01	1.18E-02	2.22E-01	7.19E-01	-9.26E-01	5.86E-03	-4.45E-02	1.00E+00
ENSBTAG00000015704	-6.01E-02	1.00E+00	-6.72E-01	2.13E-03	-2.86E-02	1.00E+00	-2.19E-02	1.00E+00	1.47E-01	1.00E+00
ENSBTAG00000019645	-6.08E-02	1.00E+00	-8.59E-01	2.24E-04	-6.94E-01	3.42E-03	-2.64E-01	4.23E-01	3.49E-01	6.01E-01
ENSBTAG00000019961	-3.66E-01	1.00E+00	-1.79E+00	5.56E-02	-7.97E-01	4.26E-01	-1.78E+00	4.63E-02	-6.31E-01	9.08E-01
ENSBTAG00000019963	3.95E-01	1.00E+00	-4.20E-01	1.00E+00	-6.49E-01	8.99E-01	4.98E-01	9.55E-01	-3.21E+00	4.34E-01
ENSBTAG00000019965	-2.14E+00	2.39E-01	-2.86E+00	2.22E-02	-2.30E+00	3.79E-02	-9.08E-01	4.52E-01	-1.63E+00	4.94E-01
ENSBTAG00000019964	-4.78E-01	5.80E-01	-1.00E+00	2.87E-03	-7.64E-01	2.89E-02	-8.35E-01	1.74E-02	-5.04E-01	5.90E-01
ENSBTAG00000025192	-2.93E-01	1.00E+00	-1.10E+00	5.46E-01	2.79E-01	1.00E+00	-8.60E-01	6.61E-01	4.46E-01	1.00E+00
ENSBTAG00000019967	-4.90E-02	1.00E+00	-4.86E-01	3.96E-02	-4.04E-01	1.01E-01	-6.82E-01	2.51E-03	-1.26E-01	1.00E+00
ENSBTAG00000000059	0.00E+00	1.00E+00	1.98E+00	4.43E-01	-5.13E-15	1.00E+00	0.00E+00	1.00E+00	0.00E+00	1.00E+00
ENSBTAG00000019971	1.07E-01	1.00E+00	9.91E-01	9.45E-08	1.32E+00	4.01E-13	8.60E-01	5.40E-06	-6.83E-02	1.00E+00
ENSBTAG00000044304	0.00E+00	1.00E+00	0.00E+00	1.00E+00	-5.13E-15	1.00E+00	0.00E+00	1.00E+00	0.00E+00	1.00E+00
ENSBTAG00000020858	2.35E-01	9.71E-01	1.51E+00	5.04E-09	7.56E-01	7.07E-03	8.91E-01	1.30E-03	2.59E-01	8.89E-01
ENSBTAG00000020861	-3.14E-02	1.00E+00	1.11E+00	1.00E-05	1.37E+00	2.71E-08	1.10E+00	1.32E-05	-8.66E-03	1.00E+00
ENSBTAG00000038732	0.00E+00	1.00E+00	2.79E+00	2.10E-01	-5.13E-15	1.00E+00	0.00E+00	1.00E+00	0.00E+00	1.00E+00
ENSBTAG00000023187	0.00E+00	1.00E+00	0.00E+00	1.00E+00	1.78E+00	4.79E-01	1.76E+00	4.97E-01	1.93E+00	8.00E-01
ENSBTAG00000024141	0.00E+00	1.00E+00	1.99E+00	4.43E-01	-5.13E-15	1.00E+00	0.00E+00	1.00E+00	1.91E+00	8.00E-01
ENSBTAG00000008177	0.00E+00	1.00E+00	0.00E+00	1.00E+00	-5.13E-15	1.00E+00	0.00E+00	1.00E+00	0.00E+00	1.00E+00
ENSBTAG00000024139	0.00E+00	1.00E+00	0.00E+00	1.00E+00	-5.13E-15	1.00E+00	0.00E+00	1.00E+00	0.00E+00	1.00E+00
ENSBTAG00000024891	1.92E+00	8.23E-01	0.00E+00	1.00E+00	-5.13E-15	1.00E+00	0.00E+00	1.00E+00	0.00E+00	1.00E+00
ENSBTAG00000025720	0.00E+00	1.00E+00	0.00E+00	1.00E+00	1.78E+00	4.79E-01	0.00E+00	1.00E+00	0.00E+00	1.00E+00
ENSBTAG00000035323	5.13E-02	1.00E+00	-1.79E-02	1.00E+00	2.94E-01	3.31E-01	7.48E-01	1.68E-03	7.77E-02	1.00E+00
ENSBTAG00000008338	1.48E-01	1.00E+00	-7.54E-01	4.48E-02	-1.38E+00	6.43E-05	-1.08E+00	2.76E-03	-1.62E-01	1.00E+00
ENSBTAG00000044845	0.00E+00	1.00E+00	0.00E+00	1.00E+00	-5.13E-15	1.00E+00	0.00E+00	1.00E+00	0.00E+00	1.00E+00
ENSBTAG00000013116	2.13E-01	1.00E+00	-4.12E-01	2.53E-01	-1.28E+00	9.97E-06	-4.79E-01	1.68E-01	-2.77E-01	8.58E-01
ENSBTAG00000007977	-7.79E-01	1.00E+00	-2.70E+00	2.42E-01	-2.70E+00	1.99E-01	-2.70E+00	2.05E-01	5.15E-01	1.00E+00
ENSBTAG00000017679	1.69E+00	7.72E-01	1.39E+00	5.19E-01	1.13E+00	6.52E-01	1.12E+00	6.71E-01	1.30E+00	9.00E-01
ENSBTAG00000046074	0.00E+00	1.00E+00	1.98E+00	4.43E-01	-5.13E-15	1.00E+00	0.00E+00	1.00E+00	0.00E+00	1.00E+00

ENSBTAG00000045541	0.00E+00	1.00E+00	0.00E+00	1.00E+00	-5.13E-15	1.00E+00	0.00E+00	1.00E+00	0.00E+00	1.00E+00
ENSBTAG00000035199	8.25E-02	1.00E+00	2.30E-01	7.22E-01	2.32E-01	7.06E-01	3.90E-01	3.73E-01	-1.71E-01	1.00E+00
ENSBTAG00000019726	1.69E-02	1.00E+00	1.02E+00	5.15E-01	-1.51E-01	1.00E+00	4.93E-01	9.59E-01	2.98E-02	1.00E+00
ENSBTAG00000008323	4.08E-01	1.00E+00	9.36E-02	1.00E+00	-4.08E-02	1.00E+00	4.00E-01	8.80E-01	3.97E-01	1.00E+00
ENSBTAG00000034987	2.61E-02	1.00E+00	3.88E-01	1.98E-01	-2.00E-02	1.00E+00	-8.00E-01	2.38E-03	-1.98E-01	9.74E-01
ENSBTAG00000043163	-2.70E+00	5.76E-01	-7.15E-01	9.45E-01	2.62E+00	7.93E-03	1.45E+00	3.25E-01	9.31E-01	9.98E-01
ENSBTAG00000034991	4.27E-01	8.23E-01	-3.79E-01	5.34E-01	1.76E-01	9.58E-01	4.78E-02	1.00E+00	2.64E-01	1.00E+00
ENSBTAG00000012817	1.63E+00	2.11E-06	2.42E+00	1.92E-14	1.52E+00	1.35E-06	4.99E-01	2.05E-01	-3.95E-03	1.00E+00
ENSBTAG00000047779	0.00E+00	1.00E+00	0.00E+00	1.00E+00	-5.13E-15	1.00E+00	0.00E+00	1.00E+00	0.00E+00	1.00E+00
ENSBTAG00000019304	-5.06E-02	1.00E+00	-3.33E+00	3.46E-09	-2.77E-01	7.16E-01	6.96E-01	9.57E-02	5.38E-01	6.18E-01
ENSBTAG00000044447	0.00E+00	1.00E+00	0.00E+00	1.00E+00	-5.13E-15	1.00E+00	0.00E+00	1.00E+00	0.00E+00	1.00E+00
ENSBTAG00000045631	0.00E+00	1.00E+00	1.98E+00	4.43E-01	-5.13E-15	1.00E+00	0.00E+00	1.00E+00	0.00E+00	1.00E+00
ENSBTAG00000014252	-1.91E-01	1.00E+00	-1.33E+00	2.22E-04	-2.49E+00	1.05E-11	-1.45E+00	4.83E-05	6.87E-02	1.00E+00
ENSBTAG00000045450	0.00E+00	1.00E+00	0.00E+00	1.00E+00	-5.13E-15	1.00E+00	0.00E+00	1.00E+00	0.00E+00	1.00E+00
ENSBTAG00000017188	-4.64E-01	7.65E-01	-3.64E-01	5.11E-01	-2.02E-01	8.60E-01	-5.53E-01	2.37E-01	-1.34E-01	1.00E+00
ENSBTAG00000021614	5.71E-02	1.00E+00	6.44E-01	1.10E-02	1.02E+00	1.33E-05	4.60E-01	9.94E-02	-4.56E-01	4.34E-01
ENSBTAG00000007130	3.70E-01	3.88E-01	1.98E+00	4.45E-24	1.66E+00	1.48E-17	1.11E+00	2.84E-08	-3.64E-01	4.81E-01
ENSBTAG00000004302	-3.83E-01	8.23E-01	7.01E-01	5.68E-02	8.30E-01	1.84E-02	6.84E-01	6.96E-02	-3.12E-01	8.94E-01
ENSBTAG00000020120	9.78E-04	1.00E+00	-7.89E-01	5.18E-01	-2.01E+00	3.78E-02	-5.58E-01	7.29E-01	-1.16E+00	6.78E-01
ENSBTAG00000003319	5.45E-01	9.66E-01	-1.42E-02	1.00E+00	2.49E+00	9.63E-05	8.86E-01	2.81E-01	-8.59E-01	7.62E-01
ENSBTAG00000034441	-3.01E-01	1.00E+00	1.86E-01	1.00E+00	-4.67E-02	1.00E+00	2.02E-01	1.00E+00	9.21E-01	5.22E-01
ENSBTAG00000000939	1.08E-01	1.00E+00	6.68E-01	3.07E-03	-3.21E-01	2.41E-01	1.17E-01	8.87E-01	3.25E-01	6.03E-01
ENSBTAG00000032609	8.11E-01	3.91E-03	2.31E+00	1.78E-25	2.22E+00	6.49E-24	1.89E+00	1.52E-17	6.57E-02	1.00E+00
ENSBTAG00000027506	4.71E-01	1.44E-01	1.58E+00	1.88E-16	1.33E+00	4.67E-12	6.52E-01	1.76E-03	-2.21E-01	8.00E-01
ENSBTAG00000000516	-3.21E+00	3.75E-01	-3.21E+00	1.25E-01	-6.57E-01	8.96E-01	-3.21E+00	9.91E-02	-3.21E+00	4.34E-01
ENSBTAG00000000686	-8.66E-02	1.00E+00	-3.00E-02	1.00E+00	1.70E+00	3.16E-08	1.13E+00	4.95E-04	-4.17E-01	7.71E-01
ENSBTAG00000023169	-1.21E-01	1.00E+00	-1.07E+00	3.63E-02	-3.20E-01	7.28E-01	-6.29E-01	2.94E-01	-1.80E-02	1.00E+00
ENSBTAG00000004354	2.59E-01	1.00E+00	-1.01E+00	2.38E-01	-9.26E-01	2.84E-01	-1.57E+00	4.34E-02	-8.95E-01	7.17E-01
ENSBTAG00000022922	-5.08E-01	7.75E-01	-7.27E-01	1.47E-01	-5.60E-02	1.00E+00	7.26E-02	1.00E+00	-1.91E-01	1.00E+00
ENSBTAG00000040046	4.85E-02	1.00E+00	1.78E-01	9.50E-01	-5.72E-01	2.60E-01	-4.48E-01	4.39E-01	3.04E-01	9.76E-01
ENSBTAG00000040490	5.31E-01	6.62E-01	6.66E-01	1.37E-01	3.04E-01	6.55E-01	3.14E-01	6.45E-01	5.74E-01	6.01E-01
ENSBTAG00000008650	3.08E-02	1.00E+00	-1.17E+00	2.63E-03	-1.03E+00	6.13E-03	-3.43E-01	4.97E-01	1.68E-01	1.00E+00
ENSBTAG00000045070	0.00E+00	1.00E+00	0.00E+00	1.00E+00	-5.13E-15	1.00E+00	0.00E+00	1.00E+00	0.00E+00	1.00E+00
ENSBTAG00000019511	9.21E-02	1.00E+00	2.20E-01	5.62E-01	5.11E-01	5.54E-02	5.78E-01	2.91E-02	2.70E-03	1.00E+00
ENSBTAG00000019509	-2.61E-01	9.28E-01	-7.83E-01	8.74E-03	2.99E-01	4.62E-01	3.01E-01	4.72E-01	-2.20E-01	9.87E-01

ENSBTAG00000019508	-1.37E-01	1.00E+00	1.04E-01	9.68E-01	1.04E+00	7.58E-06	4.32E-01	1.21E-01	1.42E-01	1.00E+00
ENSBTAG00000000291	2.42E-02	1.00E+00	-4.35E-01	1.38E-01	-4.79E-01	9.35E-02	-2.31E-01	5.58E-01	8.08E-01	2.43E-02
ENSBTAG00000000288	-2.37E-01	8.60E-01	-5.11E-01	4.88E-02	-5.43E-01	3.36E-02	-4.48E-01	1.03E-01	4.05E-01	4.87E-01
ENSBTAG00000044851	0.00E+00	1.00E+00	0.00E+00	1.00E+00	-5.13E-15	1.00E+00	0.00E+00	1.00E+00	0.00E+00	1.00E+00
ENSBTAG00000039571	-9.84E-01	4.21E-03	-2.90E+00	2.59E-24	-2.21E+00	1.70E-15	-6.24E-01	4.77E-02	2.07E-01	1.00E+00
ENSBTAG00000012377	1.80E-01	1.00E+00	-9.48E-01	1.31E-04	-1.32E+00	4.46E-08	-1.24E+00	4.48E-07	3.95E-01	5.57E-01
ENSBTAG00000019488	2.57E-01	9.04E-01	1.34E+00	2.60E-07	1.38E+00	8.71E-08	1.12E+00	2.39E-05	2.80E-01	8.17E-01
ENSBTAG00000008937	2.34E-01	1.00E+00	-8.70E-02	1.00E+00	3.58E-01	3.89E-01	1.07E-01	1.00E+00	-2.16E-01	1.00E+00
ENSBTAG00000045036	0.00E+00	1.00E+00	0.00E+00	1.00E+00	-5.13E-15	1.00E+00	0.00E+00	1.00E+00	0.00E+00	1.00E+00
ENSBTAG00000045874	0.00E+00	1.00E+00	0.00E+00	1.00E+00	-5.13E-15	1.00E+00	0.00E+00	1.00E+00	0.00E+00	1.00E+00
ENSBTAG00000048047	0.00E+00	1.00E+00	0.00E+00	1.00E+00	-5.13E-15	1.00E+00	0.00E+00	1.00E+00	0.00E+00	1.00E+00
ENSBTAG00000017243	3.43E-02	1.00E+00	6.00E-01	3.53E-01	2.40E+00	6.45E-08	2.46E-01	9.07E-01	-6.07E-01	8.00E-01
ENSBTAG00000002836	4.04E-02	1.00E+00	8.01E-01	2.60E-04	1.12E+00	1.07E-07	7.12E-01	1.55E-03	3.85E-01	4.73E-01
ENSBTAG00000013930	-3.88E-02	1.00E+00	-1.33E-01	9.38E-01	-2.42E-01	5.86E-01	-9.65E-02	1.00E+00	-3.47E-01	7.67E-01
ENSBTAG00000013925	3.88E-01	3.39E-01	9.12E-01	5.05E-06	4.74E-01	3.12E-02	1.98E-01	5.26E-01	3.35E-01	5.43E-01
ENSBTAG00000014075	3.94E-01	2.64E-01	8.85E-01	3.98E-06	1.08E+00	1.29E-08	1.03E+00	8.84E-08	9.25E-01	5.84E-05
ENSBTAG00000037516	1.65E-01	1.00E+00	8.13E-01	9.86E-04	9.23E-01	1.44E-04	7.11E-01	5.49E-03	-2.89E-01	7.98E-01
ENSBTAG00000013004	-9.77E-01	4.59E-01	-9.46E-01	1.79E-01	-4.25E-01	6.55E-01	-4.88E-01	5.77E-01	-3.90E-01	1.00E+00
ENSBTAG00000005915	1.03E-01	1.00E+00	4.78E-01	9.95E-02	4.39E-01	1.37E-01	-4.52E-01	1.42E-01	3.63E-01	6.31E-01
ENSBTAG00000042724	0.00E+00	1.00E+00	0.00E+00	1.00E+00	-5.13E-15	1.00E+00	0.00E+00	1.00E+00	0.00E+00	1.00E+00
ENSBTAG00000044988	0.00E+00	1.00E+00	0.00E+00	1.00E+00	-5.13E-15	1.00E+00	0.00E+00	1.00E+00	0.00E+00	1.00E+00
ENSBTAG00000010664	1.81E-02	1.00E+00	7.79E-02	1.00E+00	-1.55E-01	8.38E-01	-5.03E-01	1.19E-01	-3.56E-01	7.38E-01
ENSBTAG00000008401	3.36E+00	1.96E-07	2.12E+00	3.35E-04	1.32E+00	3.49E-02	1.34E+00	3.53E-02	-1.33E-02	1.00E+00
ENSBTAG00000021951	1.12E-01	1.00E+00	1.22E+00	4.16E-08	8.83E-01	9.65E-05	2.34E-01	4.97E-01	-4.82E-01	3.15E-01
ENSBTAG00000020892	1.02E+00	9.56E-02	1.06E+00	1.40E-02	3.73E-01	5.79E-01	1.02E+00	1.93E-02	1.04E+00	1.36E-01
ENSBTAG00000006078	6.70E-01	8.92E-01	3.88E+00	1.78E-07	3.74E+00	4.02E-07	1.93E+00	1.10E-02	-3.14E-01	1.00E+00
ENSBTAG00000004147	-1.31E-02	1.00E+00	1.36E-01	7.41E-01	5.43E-01	7.17E-03	3.49E-01	1.29E-01	3.26E-01	5.09E-01
ENSBTAG00000036222	-1.14E-01	1.00E+00	-1.54E-01	1.00E+00	-1.23E+00	1.27E-01	-3.49E-01	8.80E-01	6.95E-02	1.00E+00
ENSBTAG00000017012	-1.63E-03	1.00E+00	-7.27E-01	9.45E-01	-9.35E-01	8.05E-01	3.20E-01	1.00E+00	1.44E+00	7.57E-01
ENSBTAG00000022931	-7.12E-01	1.00E+00	-1.98E+00	4.51E-01	-2.51E+00	2.98E-01	-2.59E+00	2.91E-01	-8.91E-01	1.00E+00
ENSBTAG00000034145	2.95E-01	1.00E+00	-7.98E-01	7.84E-01	-1.81E+00	2.94E-01	-1.05E+00	5.98E-01	-1.66E+00	7.82E-01
ENSBTAG00000008288	-3.40E-01	5.55E-01	-1.66E+00	3.25E-14	-1.05E+00	1.04E-06	-2.90E-01	3.11E-01	-1.92E-01	9.10E-01
ENSBTAG00000016445	1.68E-01	1.00E+00	3.77E-01	1.49E-01	2.19E-01	4.94E-01	4.32E-01	9.19E-02	3.26E-01	6.09E-01
ENSBTAG00000017271	-2.76E-01	1.00E+00	-1.55E+00	2.82E-03	-2.06E+00	5.15E-05	-1.67E+00	1.15E-03	-2.31E-01	1.00E+00
ENSBTAG00000017278	-6.32E-01	2.30E-01	-2.96E+00	5.95E-22	-2.41E+00	9.66E-16	-1.07E+00	5.83E-04	4.10E-01	6.77E-01

ENSBTAG00000005136	2.11E-01	1.00E+00	8.09E-01	3.98E-03	6.35E-01	3.02E-02	5.14E-01	1.05E-01	2.88E-01	8.00E-01
ENSBTAG00000006529	3.31E-01	8.23E-01	1.64E+00	2.29E-08	2.01E+00	4.44E-12	1.75E+00	2.38E-09	1.11E-01	1.00E+00
ENSBTAG00000012526	-1.85E-02	1.00E+00	-3.67E-01	2.54E-01	5.34E-01	5.21E-02	1.23E-01	9.26E-01	-3.69E-01	6.27E-01
ENSBTAG00000005492	1.51E-01	1.00E+00	6.37E-01	7.17E-04	5.05E-01	9.34E-03	4.50E-01	2.67E-02	3.32E-01	4.52E-01
ENSBTAG00000016060	1.72E+00	2.13E-03	4.70E+00	1.18E-20	4.46E+00	3.12E-19	2.83E+00	2.68E-09	1.02E-01	1.00E+00
ENSBTAG00000007133	2.14E-01	8.23E-01	3.54E-01	1.21E-01	6.11E-01	2.30E-03	4.72E-01	2.81E-02	-3.54E-02	1.00E+00
ENSBTAG00000014991	6.46E-02	1.00E+00	2.45E-01	2.69E-01	-2.57E-01	2.38E-01	-3.60E-01	7.25E-02	1.47E-01	9.40E-01
ENSBTAG00000019866	2.44E-01	8.96E-01	-4.88E-01	9.76E-02	9.95E-02	1.00E+00	6.41E-01	2.02E-02	3.21E-01	7.63E-01
ENSBTAG00000015910	1.94E-01	8.27E-01	5.75E-01	2.39E-03	6.09E-01	1.15E-03	7.11E-01	1.38E-04	-6.42E-02	1.00E+00
ENSBTAG00000002675	-2.97E-01	1.00E+00	-1.57E+00	5.32E-02	-2.38E+00	3.25E-03	-1.48E+00	5.73E-02	-9.82E-02	1.00E+00
ENSBTAG00000043964	1.16E+00	3.74E-02	2.12E+00	6.55E-08	1.46E+00	3.14E-04	1.36E+00	1.11E-03	4.15E-02	1.00E+00
ENSBTAG00000047430	0.00E+00	1.00E+00	0.00E+00	1.00E+00	-5.13E-15	1.00E+00	0.00E+00	1.00E+00	0.00E+00	1.00E+00
ENSBTAG00000037969	-1.91E+00	8.23E-01	2.07E+00	1.95E-01	1.82E+00	2.76E-01	1.49E+00	4.55E-01	8.00E-01	1.00E+00
ENSBTAG00000023216	-2.28E-01	1.00E+00	-1.40E-01	1.00E+00	-7.84E-01	4.04E-01	1.02E+00	1.28E-01	-5.07E-01	9.98E-01
ENSBTAG00000044751	0.00E+00	1.00E+00	0.00E+00	1.00E+00	-5.13E-15	1.00E+00	0.00E+00	1.00E+00	0.00E+00	1.00E+00
ENSBTAG00000045189	0.00E+00	1.00E+00	0.00E+00	1.00E+00	-5.13E-15	1.00E+00	0.00E+00	1.00E+00	0.00E+00	1.00E+00
ENSBTAG00000009475	7.67E-02	1.00E+00	-1.05E-01	1.00E+00	-1.38E-01	9.48E-01	2.94E-01	5.07E-01	-3.49E-01	8.00E-01
ENSBTAG00000013339	3.67E-01	7.85E-01	6.75E-01	4.21E-02	-1.85E+00	3.59E-09	-2.51E+00	8.72E-15	8.55E-01	7.31E-02
ENSBTAG00000003937	2.42E-02	1.00E+00	1.39E-01	8.14E-01	-3.12E-03	1.00E+00	7.45E-02	1.00E+00	-2.73E-01	7.86E-01
ENSBTAG00000044047	1.12E-02	1.00E+00	7.51E-02	1.00E+00	1.14E+00	6.46E-01	6.22E-01	9.94E-01	7.99E-01	1.00E+00
ENSBTAG00000044074	-1.75E-01	1.00E+00	-1.99E+00	4.20E-18	-9.75E-01	2.30E-05	-5.30E-01	3.90E-02	-9.50E-02	1.00E+00
ENSBTAG00000033315	1.03E-01	1.00E+00	2.59E-01	4.46E-01	5.32E-01	4.21E-02	5.92E-01	2.34E-02	-9.80E-02	1.00E+00
ENSBTAG00000044566	0.00E+00	1.00E+00	0.00E+00	1.00E+00	-5.13E-15	1.00E+00	0.00E+00	1.00E+00	0.00E+00	1.00E+00
ENSBTAG00000025968	-3.20E-02	1.00E+00	1.66E-01	8.50E-01	2.12E-02	1.00E+00	9.14E-02	1.00E+00	-4.31E-01	6.06E-01
ENSBTAG00000015584	9.70E-02	1.00E+00	2.13E-01	6.47E-01	1.25E+00	8.03E-07	1.56E+00	6.22E-10	7.58E-02	1.00E+00
ENSBTAG00000033291	-1.19E+00	2.33E-01	-2.25E+00	5.14E-04	-8.29E-01	2.16E-01	-8.77E-01	1.94E-01	-8.98E-01	5.58E-01
ENSBTAG00000003589	0.00E+00	1.00E+00	0.00E+00	1.00E+00	-5.13E-15	1.00E+00	0.00E+00	1.00E+00	0.00E+00	1.00E+00
ENSBTAG00000018033	9.02E-02	1.00E+00	-3.93E-01	1.88E-01	-1.67E-01	7.47E-01	2.39E-01	5.13E-01	-3.57E-02	1.00E+00
ENSBTAG00000044698	0.00E+00	1.00E+00	0.00E+00	1.00E+00	-5.13E-15	1.00E+00	0.00E+00	1.00E+00	0.00E+00	1.00E+00
ENSBTAG00000042808	0.00E+00	1.00E+00	0.00E+00	1.00E+00	-5.13E-15	1.00E+00	0.00E+00	1.00E+00	0.00E+00	1.00E+00
ENSBTAG00000043527	0.00E+00	1.00E+00	0.00E+00	1.00E+00	-5.13E-15	1.00E+00	0.00E+00	1.00E+00	0.00E+00	1.00E+00
ENSBTAG00000020929	3.97E-02	1.00E+00	-5.71E-01	5.09E-01	-1.63E+00	1.49E-02	-1.25E+00	6.98E-02	-7.91E-01	7.04E-01
ENSBTAG00000044112	-1.85E-01	1.00E+00	-8.26E-01	3.21E-03	-1.65E+00	6.59E-10	-1.98E+00	1.94E-13	1.73E-01	1.00E+00
ENSBTAG00000047315	5.15E-03	1.00E+00	3.05E-01	1.00E+00	-1.08E+00	4.76E-01	2.04E-01	1.00E+00	3.93E-01	1.00E+00
ENSBTAG00000044137	1.21E+00	6.50E-01	1.72E+00	6.36E-02	6.25E-01	7.46E-01	1.56E+00	1.02E-01	1.68E+00	3.32E-01

ENSBTAG00000047752	-6.19E-01	6.14E-01	-6.81E-01	1.96E-01	1.28E-01	1.00E+00	6.48E-01	2.05E-01	-1.12E-01	1.00E+00
ENSBTAG00000044362	0.00E+00	1.00E+00	0.00E+00	1.00E+00	-5.13E-15	1.00E+00	0.00E+00	1.00E+00	0.00E+00	1.00E+00
ENSBTAG00000043235	0.00E+00	1.00E+00	0.00E+00	1.00E+00	-5.13E-15	1.00E+00	0.00E+00	1.00E+00	0.00E+00	1.00E+00
ENSBTAG00000040232	3.90E+00	1.40E-01	0.00E+00	1.00E+00	1.78E+00	4.79E-01	1.76E+00	4.97E-01	0.00E+00	1.00E+00
ENSBTAG00000018395	4.45E-01	4.05E-01	9.70E-01	1.23E-04	-2.09E-01	6.23E-01	6.31E-02	1.00E+00	1.41E-01	1.00E+00
ENSBTAG00000021857	8.10E-02	1.00E+00	-2.54E-01	3.62E-01	-2.98E-01	2.45E-01	4.69E-01	3.84E-02	1.59E-01	9.85E-01
ENSBTAG00000044011	1.44E+00	7.50E-01	-2.70E+00	2.42E-01	1.26E+00	4.28E-01	-1.70E-01	1.00E+00	7.72E-03	1.00E+00
ENSBTAG00000015569	6.36E-01	8.09E-01	2.90E-01	8.97E-01	3.02E-04	1.00E+00	6.76E-01	3.46E-01	1.15E-01	1.00E+00
ENSBTAG00000042232	0.00E+00	1.00E+00	0.00E+00	1.00E+00	-5.13E-15	1.00E+00	0.00E+00	1.00E+00	0.00E+00	1.00E+00
ENSBTAG00000009117	3.57E-01	9.60E-01	-8.59E-01	9.99E-02	5.28E-01	3.21E-01	6.24E-01	2.18E-01	4.85E-01	8.00E-01
ENSBTAG00000023920	-3.76E-01	1.00E+00	1.04E-01	1.00E+00	-7.59E-01	4.74E-01	8.92E-01	2.83E-01	-1.01E+00	7.13E-01
ENSBTAG00000002123	1.51E+00	4.60E-01	1.59E+00	1.25E-01	1.22E+00	3.01E-01	2.13E+00	1.51E-02	3.04E-01	1.00E+00
ENSBTAG00000008056	-6.60E-01	1.00E+00	-1.09E+00	5.55E-01	-1.34E+00	4.16E-01	5.91E-01	7.77E-01	-1.97E+00	6.03E-01
ENSBTAG00000003640	-7.81E-01	1.00E+00	-2.70E+00	2.42E-01	-2.70E+00	1.99E-01	-2.70E+00	2.05E-01	-2.70E+00	6.01E-01
ENSBTAG00000015072	-5.53E-01	3.83E-01	1.06E-01	1.00E+00	4.66E-01	1.86E-01	8.52E-01	5.17E-03	-1.00E-01	1.00E+00
ENSBTAG00000047440	0.00E+00	1.00E+00	0.00E+00	1.00E+00	-5.13E-15	1.00E+00	0.00E+00	1.00E+00	0.00E+00	1.00E+00
ENSBTAG00000044876	0.00E+00	1.00E+00	0.00E+00	1.00E+00	-5.13E-15	1.00E+00	0.00E+00	1.00E+00	0.00E+00	1.00E+00
ENSBTAG00000042704	0.00E+00	1.00E+00	0.00E+00	1.00E+00	-5.13E-15	1.00E+00	1.76E+00	4.97E-01	0.00E+00	1.00E+00
ENSBTAG00000016401	6.82E-01	5.60E-02	1.44E+00	4.90E-09	1.27E+00	2.90E-07	1.01E+00	7.27E-05	-3.54E-01	6.51E-01
ENSBTAG00000016406	-2.25E-01	1.00E+00	-9.04E-02	1.00E+00	-6.17E-02	1.00E+00	3.00E-01	8.08E-01	6.16E-01	7.43E-01
ENSBTAG00000047869	-3.29E-01	1.00E+00	-6.32E-01	2.43E-01	-1.11E+00	1.92E-02	-1.63E+00	6.64E-04	-1.45E+00	3.75E-02
ENSBTAG00000007700	8.88E-02	1.00E+00	-2.14E-01	4.87E-01	-8.82E-01	2.85E-05	-1.28E+00	7.15E-10	-1.09E+00	7.13E-06
ENSBTAG00000008821	-2.05E-01	1.00E+00	-9.91E-01	2.95E-03	-2.41E-01	6.81E-01	5.23E-01	1.78E-01	3.89E-01	7.79E-01
ENSBTAG00000020800	2.47E-01	1.00E+00	2.28E+00	8.38E-14	6.77E-01	4.88E-02	6.01E-01	9.80E-02	3.44E-01	8.00E-01
ENSBTAG00000017244	1.95E-01	1.00E+00	1.15E+00	1.18E-06	9.14E-01	1.37E-04	4.26E-01	1.38E-01	5.94E-02	1.00E+00
ENSBTAG00000008279	3.33E-01	5.86E-01	6.40E-01	6.60E-03	2.42E-01	4.52E-01	-2.11E-01	5.54E-01	3.37E-01	6.01E-01
ENSBTAG00000010023	9.21E-02	1.00E+00	7.13E-01	6.94E-03	-2.82E-01	4.28E-01	-6.60E-02	1.00E+00	4.12E-01	5.45E-01
ENSBTAG00000016450	-5.07E-01	1.00E+00	-7.01E-01	4.92E-01	-2.06E+00	1.69E-02	-1.54E+00	7.21E-02	2.77E-02	1.00E+00
ENSBTAG00000016451	8.89E-02	1.00E+00	6.34E-01	4.06E-03	1.72E-01	6.41E-01	5.16E-02	1.00E+00	-3.85E-01	4.46E-01
ENSBTAG00000045609	0.00E+00	1.00E+00	0.00E+00	1.00E+00	-5.13E-15	1.00E+00	0.00E+00	1.00E+00	0.00E+00	1.00E+00
ENSBTAG00000042878	0.00E+00	1.00E+00	0.00E+00	1.00E+00	-5.13E-15	1.00E+00	0.00E+00	1.00E+00	0.00E+00	1.00E+00
ENSBTAG00000010210	3.43E-01	7.27E-01	1.13E+00	6.81E-06	9.61E-01	1.52E-04	8.97E-01	5.78E-04	4.63E-01	4.64E-01
ENSBTAG00000010220	2.31E-01	9.71E-01	6.13E-01	3.79E-02	1.02E+00	1.29E-04	8.96E-01	1.16E-03	8.77E-02	1.00E+00
ENSBTAG00000002856	1.26E-02	1.00E+00	-1.91E+00	4.69E-01	-1.91E+00	4.28E-01	-1.91E+00	4.39E-01	-1.91E+00	8.00E-01
ENSBTAG00000047454	1.31E+00	9.30E-01	-1.91E+00	4.69E-01	1.51E+00	4.28E-01	6.28E-01	9.94E-01	7.16E-03	1.00E+00

ENSBTAG00000002858	-5.64E-01	1.00E+00	-1.99E+00	4.92E-02	-1.33E+00	1.60E-01	-1.72E-01	1.00E+00	-3.03E-01	1.00E+00
ENSBTAG00000007702	-2.79E-01	9.25E-01	7.11E-01	2.33E-02	1.56E+00	2.26E-08	1.43E+00	4.58E-07	-1.85E-01	1.00E+00
ENSBTAG00000007703	-3.28E-01	8.70E-01	-2.71E+00	5.44E-16	-1.37E+00	3.63E-05	-5.34E-01	1.87E-01	-1.68E-01	1.00E+00
ENSBTAG00000008787	-1.84E-01	1.00E+00	-1.09E+00	6.99E-04	-1.42E+00	7.17E-06	-8.29E-01	1.46E-02	2.14E-01	1.00E+00
ENSBTAG00000044414	0.00E+00	1.00E+00	0.00E+00	1.00E+00	-5.13E-15	1.00E+00	0.00E+00	1.00E+00	0.00E+00	1.00E+00
ENSBTAG00000007602	8.78E-01	6.36E-01	1.06E+00	1.47E-01	1.22E+00	6.84E-02	1.55E+00	1.37E-02	1.30E+00	2.84E-01
ENSBTAG00000010387	-1.09E-01	1.00E+00	-6.20E-01	4.32E-03	-8.50E-01	4.58E-05	-1.02E+00	1.02E-06	-4.23E-01	3.41E-01
ENSBTAG00000016676	-6.31E-01	4.09E-01	-2.31E+00	9.92E-09	-2.19E+00	1.50E-08	-1.87E+00	1.04E-06	-3.23E-01	8.98E-01
ENSBTAG00000016677	4.17E-01	1.00E+00	-1.34E+00	3.97E-01	-1.57E+00	2.64E-01	-2.37E+00	1.02E-01	4.08E-01	1.00E+00
ENSBTAG00000027446	-3.82E-02	1.00E+00	-5.96E-01	2.12E-02	-9.46E-01	9.19E-05	-5.77E-01	2.92E-02	-1.03E-01	1.00E+00
ENSBTAG00000005072	-4.96E-01	9.71E-01	-4.85E-01	6.30E-01	-5.87E-01	4.83E-01	-4.11E-01	7.32E-01	-8.15E-01	6.82E-01
ENSBTAG00000020546	3.27E-02	1.00E+00	-1.02E+00	4.04E-04	-4.14E-01	2.20E-01	9.04E-02	1.00E+00	-1.73E-01	1.00E+00
ENSBTAG00000018463	1.67E-01	1.00E+00	-6.68E-01	2.04E-02	-4.77E-01	1.28E-01	-9.74E-02	1.00E+00	-1.04E-01	1.00E+00
ENSBTAG00000011366	-2.29E-01	1.00E+00	-2.57E-02	1.00E+00	-7.37E-01	5.37E-01	9.01E-02	1.00E+00	1.98E-01	1.00E+00
ENSBTAG00000033089	-2.35E-01	9.44E-01	-1.11E+00	3.84E-05	-1.09E+00	5.04E-05	-8.51E-01	2.31E-03	-4.76E-01	4.52E-01
ENSBTAG00000002658	6.63E-01	8.50E-02	2.07E+00	5.61E-17	1.53E+00	7.54E-10	1.89E+00	2.12E-14	-8.63E-03	1.00E+00
ENSBTAG00000042890	0.00E+00	1.00E+00	0.00E+00	1.00E+00	-5.13E-15	1.00E+00	0.00E+00	1.00E+00	0.00E+00	1.00E+00
ENSBTAG00000018981	-2.95E-01	1.00E+00	1.33E+00	1.10E-02	2.65E+00	1.15E-08	1.01E+00	7.59E-02	-2.51E-02	1.00E+00
ENSBTAG00000038048	-1.10E-01	1.00E+00	2.98E-01	2.43E-01	-4.16E-02	1.00E+00	-3.48E-01	1.60E-01	-3.88E-01	4.17E-01
ENSBTAG00000015525	4.10E-02	1.00E+00	-7.92E-01	3.97E-01	-1.31E+00	9.63E-02	-8.07E-01	3.64E-01	-1.10E+00	5.79E-01
ENSBTAG00000022000	-1.68E-02	1.00E+00	-1.67E+00	2.35E-12	-1.56E+00	2.65E-11	-1.66E+00	2.22E-12	-3.06E-01	7.32E-01
ENSBTAG00000022003	1.94E-02	1.00E+00	-1.09E+00	1.56E-04	-1.16E+00	4.48E-05	-1.17E+00	4.63E-05	-2.48E-02	1.00E+00
ENSBTAG00000001552	3.94E-01	5.04E-01	1.13E+00	1.69E-06	9.55E-01	6.59E-05	7.35E-01	3.60E-03	4.96E-01	3.26E-01
ENSBTAG00000043839	0.00E+00	1.00E+00	0.00E+00	1.00E+00	-5.13E-15	1.00E+00	0.00E+00	1.00E+00	0.00E+00	1.00E+00
ENSBTAG00000002280	1.72E-01	1.00E+00	9.74E-01	5.62E-03	1.01E+00	4.04E-03	1.25E+00	3.19E-04	-4.71E-01	6.61E-01
ENSBTAG00000044257	0.00E+00	1.00E+00	0.00E+00	1.00E+00	-5.13E-15	1.00E+00	0.00E+00	1.00E+00	0.00E+00	1.00E+00
ENSBTAG00000012677	-7.98E-02	1.00E+00	-1.11E+00	2.56E-05	-6.71E-01	1.60E-02	2.31E-01	5.85E-01	-5.81E-02	1.00E+00
ENSBTAG00000020053	1.03E-01	1.00E+00	1.53E+00	4.66E-12	1.11E+00	7.53E-07	3.99E-01	1.49E-01	-2.60E-01	8.00E-01
ENSBTAG00000044537	0.00E+00	1.00E+00	0.00E+00	1.00E+00	-5.13E-15	1.00E+00	0.00E+00	1.00E+00	0.00E+00	1.00E+00
ENSBTAG00000016294	3.51E-01	7.69E-01	-1.35E+00	1.16E-05	-9.60E-01	1.56E-03	-7.30E-01	2.25E-02	-9.86E-02	1.00E+00
ENSBTAG00000027444	-4.92E-02	1.00E+00	-2.09E+00	1.28E-09	-2.11E+00	6.53E-10	-1.48E+00	1.97E-05	3.95E-01	8.00E-01
ENSBTAG00000046981	0.00E+00	1.00E+00	0.00E+00	1.00E+00	-5.13E-15	1.00E+00	0.00E+00	1.00E+00	0.00E+00	1.00E+00
ENSBTAG00000046352	0.00E+00	1.00E+00	0.00E+00	1.00E+00	-5.13E-15	1.00E+00	0.00E+00	1.00E+00	0.00E+00	1.00E+00
ENSBTAG00000001204	2.10E-02	1.00E+00	-1.47E-01	8.38E-01	-3.63E-02	1.00E+00	2.81E-01	4.38E-01	2.17E-01	9.19E-01
ENSBTAG00000004387	3.59E-01	4.98E-01	1.63E+00	9.12E-15	1.43E+00	9.66E-12	6.59E-01	4.05E-03	-1.92E-01	9.22E-01

ENSBTAG00000011600	3.34E+00	3.90E-32	3.23E+00	3.82E-31	2.19E+00	4.47E-16	6.57E-01	3.01E-02	-3.18E-01	8.00E-01
ENSBTAG00000010330	-4.55E-01	1.00E+00	-2.08E-01	1.00E+00	-2.28E+00	4.54E-02	-1.44E+00	2.07E-01	1.57E-01	1.00E+00
ENSBTAG00000003045	-1.20E+00	4.72E-03	2.36E-01	7.41E-01	1.64E+00	1.05E-06	-4.48E-02	1.00E+00	-6.35E-01	4.31E-01
ENSBTAG00000018263	7.81E-02	1.00E+00	6.45E-01	1.09E-03	4.65E-01	2.56E-02	4.56E-01	3.30E-02	1.94E-01	8.38E-01
ENSBTAG00000017354	1.80E-01	1.00E+00	-2.69E+00	3.81E-21	-4.59E+00	7.23E-50	-5.03E+00	6.65E-57	-1.09E-02	1.00E+00
ENSBTAG00000000654	3.30E-01	1.00E+00	-9.92E-01	1.21E-01	-3.14E+00	9.62E-08	-3.57E+00	4.47E-09	-7.32E-01	7.03E-01
ENSBTAG00000007678	-4.28E-01	1.00E+00	-1.91E-01	1.00E+00	-1.03E+00	3.40E-01	-1.29E+00	2.05E-01	-8.67E-01	8.00E-01
ENSBTAG00000009871	6.89E-02	1.00E+00	4.48E-01	5.52E-02	3.08E-01	2.43E-01	1.97E-01	5.51E-01	-3.42E-01	5.55E-01
ENSBTAG00000048079	0.00E+00	1.00E+00	0.00E+00	1.00E+00	1.78E+00	4.79E-01	0.00E+00	1.00E+00	0.00E+00	1.00E+00
ENSBTAG00000008687	5.08E-01	8.24E-01	1.58E+00	6.62E-04	1.16E+00	1.81E-02	1.83E+00	5.87E-05	2.61E-01	1.00E+00
ENSBTAG00000006043	-1.70E-01	1.00E+00	-9.47E-01	3.93E-01	-9.98E-01	3.49E-01	-9.21E-01	4.23E-01	7.22E-02	1.00E+00
ENSBTAG00000021867	4.10E-01	1.00E+00	-1.39E+00	3.85E-02	7.56E-01	2.23E-01	1.32E+00	1.25E-02	5.32E-01	8.15E-01
ENSBTAG00000015434	-6.18E-02	1.00E+00	-4.07E-01	6.95E-02	-8.19E-01	3.94E-05	-5.73E-01	6.92E-03	1.88E-01	8.77E-01
ENSBTAG00000015438	2.82E-01	8.23E-01	1.27E+00	7.60E-07	1.34E+00	1.56E-07	1.38E+00	9.58E-08	3.67E-01	6.47E-01
ENSBTAG00000004474	2.09E+00	1.14E-02	1.71E+00	1.24E-02	1.15E+00	1.20E-01	6.67E-01	4.84E-01	9.17E-01	6.30E-01
ENSBTAG00000003422	-2.67E-03	1.00E+00	-1.80E-01	6.68E-01	1.35E-01	8.32E-01	1.10E-01	9.37E-01	-1.70E-01	1.00E+00
ENSBTAG00000042662	0.00E+00	1.00E+00	0.00E+00	1.00E+00	-5.13E-15	1.00E+00	0.00E+00	1.00E+00	0.00E+00	1.00E+00
ENSBTAG00000003425	-6.53E-02	1.00E+00	-1.04E+00	1.09E-03	-9.72E-02	1.00E+00	-3.05E-01	4.97E-01	-2.69E-01	9.11E-01
ENSBTAG00000008410	-7.85E-01	8.23E-01	2.81E-01	9.51E-01	-5.70E-01	5.96E-01	-1.54E+00	7.50E-02	3.23E-01	1.00E+00
ENSBTAG00000018978	4.02E-01	3.69E-01	1.73E+00	6.26E-17	1.81E+00	1.11E-18	1.12E+00	1.04E-07	2.35E-01	8.00E-01
ENSBTAG00000004620	-2.03E-01	9.42E-01	3.13E-01	2.74E-01	7.90E-01	4.67E-04	5.05E-01	4.39E-02	3.04E-01	6.96E-01
ENSBTAG00000004622	4.68E-01	8.23E-01	-2.86E-01	8.15E-01	-7.43E-01	1.99E-01	8.46E-01	6.89E-02	1.07E+00	1.14E-01
ENSBTAG00000008825	4.13E-02	1.00E+00	2.26E-01	4.51E-01	4.16E-01	7.57E-02	-8.24E-02	1.00E+00	-1.33E-01	1.00E+00
ENSBTAG00000008826	-1.79E-01	1.00E+00	-8.90E-01	5.69E-03	-8.68E-01	6.85E-03	-3.38E-01	4.42E-01	-3.89E-01	7.54E-01
ENSBTAG00000018295	1.45E+00	2.68E-07	3.93E+00	5.13E-46	3.63E+00	1.27E-40	3.03E+00	6.92E-30	1.72E-01	1.00E+00
ENSBTAG00000008964	6.89E-02	1.00E+00	-5.37E-01	5.39E-02	-7.41E-01	4.48E-03	-5.42E-01	5.48E-02	-2.19E-01	9.15E-01
ENSBTAG00000012276	0.00E+00	1.00E+00	0.00E+00	1.00E+00	-5.13E-15	1.00E+00	0.00E+00	1.00E+00	0.00E+00	1.00E+00
ENSBTAG00000032827	-1.67E+00	7.80E-01	-2.80E-01	1.00E+00	-5.32E-01	9.38E-01	-5.48E-01	9.29E-01	-3.58E+00	3.10E-01
ENSBTAG00000005202	-1.13E+00	8.23E-01	-3.64E-01	1.00E+00	-9.10E-01	5.29E-01	-2.58E+00	6.07E-02	9.09E-04	1.00E+00
ENSBTAG00000011882	4.03E-01	8.39E-01	-2.59E-01	8.02E-01	-4.68E-01	4.17E-01	-5.69E-01	2.90E-01	-4.54E-01	8.00E-01
ENSBTAG00000032893	-3.57E-01	9.66E-01	-5.13E-01	3.77E-01	-3.13E-01	6.82E-01	4.70E-01	4.14E-01	-5.45E-02	1.00E+00
ENSBTAG00000020382	5.91E-02	1.00E+00	-1.26E+00	6.65E-04	3.13E-01	5.78E-01	5.91E-01	1.71E-01	-2.64E-01	1.00E+00
ENSBTAG00000045220	0.00E+00	1.00E+00	0.00E+00	1.00E+00	-5.13E-15	1.00E+00	0.00E+00	1.00E+00	0.00E+00	1.00E+00
ENSBTAG00000001144	2.07E-02	1.00E+00	4.61E-01	3.69E-02	7.26E-01	3.67E-04	4.86E-01	2.88E-02	-3.01E-01	6.07E-01
ENSBTAG00000008472	2.23E-01	8.94E-01	1.11E+00	1.00E-06	9.51E-01	3.24E-05	4.44E-01	9.87E-02	1.63E-01	1.00E+00

ENSBTAG00000009291	1.40E-01	1.00E+00	-3.82E-01	7.44E-01	-1.37E-01	1.00E+00	5.17E-01	4.70E-01	1.47E-01	1.00E+00
ENSBTAG00000045526	-7.80E-01	1.00E+00	-2.70E+00	2.42E-01	-2.70E+00	1.99E-01	-2.70E+00	2.05E-01	8.99E-01	1.00E+00
ENSBTAG00000014178	1.51E-01	1.00E+00	-3.66E-01	4.16E-01	-4.81E-01	2.20E-01	-1.16E-02	1.00E+00	3.84E-01	7.82E-01
ENSBTAG00000042976	0.00E+00	1.00E+00	0.00E+00	1.00E+00	-5.13E-15	1.00E+00	0.00E+00	1.00E+00	0.00E+00	1.00E+00
ENSBTAG00000020988	2.87E-01	9.30E-01	-3.06E-02	1.00E+00	1.94E-01	8.05E-01	3.82E-01	3.74E-01	1.74E-01	1.00E+00
ENSBTAG00000011304	1.73E-01	1.00E+00	2.33E+00	3.97E-13	2.03E+00	1.84E-10	1.10E+00	1.00E-03	-1.83E-01	1.00E+00
ENSBTAG00000011312	-1.91E+00	8.23E-01	-1.91E+00	4.69E-01	-1.24E-01	1.00E+00	-1.91E+00	4.39E-01	1.84E-02	1.00E+00
ENSBTAG00000010277	-1.91E+00	8.23E-01	7.51E-02	1.00E+00	6.49E-01	9.78E-01	-1.91E+00	4.39E-01	-1.91E+00	8.00E-01
ENSBTAG00000035643	1.32E-01	1.00E+00	-2.43E-01	8.51E-01	4.40E-01	4.28E-01	3.91E-01	5.04E-01	-8.98E-01	3.42E-01
ENSBTAG00000006742	7.97E-01	1.00E+00	1.40E+00	5.16E-01	-1.30E-01	1.00E+00	-1.91E+00	4.39E-01	8.22E-01	1.00E+00
ENSBTAG00000012407	6.60E-01	9.80E-02	2.18E-01	6.32E-01	1.02E-01	1.00E+00	4.64E-01	1.43E-01	4.95E-01	4.24E-01
ENSBTAG00000045231	0.00E+00	1.00E+00	1.98E+00	4.43E-01	-5.13E-15	1.00E+00	0.00E+00	1.00E+00	0.00E+00	1.00E+00
ENSBTAG00000002758	4.42E-01	9.59E-01	8.78E-01	1.45E-01	1.97E+00	1.52E-04	2.55E+00	1.09E-06	-4.38E-01	9.37E-01
ENSBTAG00000004207	-1.06E-01	1.00E+00	-9.90E-01	8.09E-04	-6.35E-03	1.00E+00	1.98E-01	7.52E-01	-3.13E-01	8.00E-01
ENSBTAG00000004993	-4.57E-01	6.32E-01	1.03E-01	1.00E+00	3.10E-01	4.83E-01	1.33E-01	9.78E-01	-2.80E-01	9.18E-01
ENSBTAG00000008908	3.83E-01	4.36E-01	1.13E+00	1.23E-07	8.34E-01	1.41E-04	4.69E-01	6.12E-02	4.87E-01	2.66E-01
ENSBTAG00000032704	5.27E-02	1.00E+00	4.07E-01	1.48E-01	7.42E-01	2.27E-03	2.08E-01	6.01E-01	-7.57E-02	1.00E+00
ENSBTAG00000032701	2.72E+00	5.76E-01	0.00E+00	1.00E+00	-5.13E-15	1.00E+00	0.00E+00	1.00E+00	0.00E+00	1.00E+00
ENSBTAG00000013415	1.43E-01	1.00E+00	-8.70E-01	4.92E-01	-1.42E+00	1.89E-01	-1.81E+00	9.93E-02	2.42E-02	1.00E+00
ENSBTAG00000013416	-2.09E+00	2.34E-01	-3.35E+00	7.27E-03	-1.92E+00	7.04E-02	-1.20E+00	2.86E-01	-1.00E+00	8.00E-01
ENSBTAG00000027420	2.71E+00	5.76E-01	1.98E+00	4.43E-01	-5.13E-15	1.00E+00	0.00E+00	1.00E+00	1.91E+00	8.00E-01
ENSBTAG00000032694	1.92E+00	8.23E-01	0.00E+00	1.00E+00	-5.13E-15	1.00E+00	2.53E+00	2.71E-01	0.00E+00	1.00E+00
ENSBTAG00000015949	5.34E-03	1.00E+00	-1.91E+00	4.69E-01	-1.26E-01	1.00E+00	-1.91E+00	4.39E-01	-1.91E+00	8.00E-01
ENSBTAG00000047137	2.71E+00	5.76E-01	0.00E+00	1.00E+00	2.56E+00	2.54E-01	1.76E+00	4.97E-01	0.00E+00	1.00E+00
ENSBTAG00000032687	0.00E+00	1.00E+00	0.00E+00	1.00E+00	-5.13E-15	1.00E+00	0.00E+00	1.00E+00	0.00E+00	1.00E+00
ENSBTAG00000000598	-1.88E-02	1.00E+00	-4.15E-01	3.18E-01	-1.00E+00	2.09E-03	-1.15E+00	4.45E-04	-2.42E-01	9.93E-01
ENSBTAG00000040147	1.60E-01	1.00E+00	2.35E-01	1.00E+00	-3.48E-01	9.69E-01	3.03E-01	9.91E-01	1.05E+00	5.98E-01
ENSBTAG00000004273	8.82E-02	1.00E+00	-1.29E-01	1.00E+00	-8.26E-01	3.96E-02	-1.14E+00	3.60E-03	4.08E-01	8.00E-01
ENSBTAG00000004278	-4.73E-01	3.51E-01	-1.14E+00	5.96E-06	-1.57E+00	2.32E-10	-1.37E+00	4.26E-08	4.66E-01	4.33E-01
ENSBTAG00000009881	-9.74E-02	1.00E+00	-5.53E-01	6.73E-02	-4.03E-01	2.19E-01	2.26E-01	6.09E-01	8.76E-02	1.00E+00
ENSBTAG00000004281	0.00E+00	1.00E+00	0.00E+00	1.00E+00	-5.13E-15	1.00E+00	0.00E+00	1.00E+00	0.00E+00	1.00E+00
ENSBTAG00000003335	1.92E+00	8.23E-01	3.30E+00	1.02E-01	1.78E+00	4.79E-01	2.53E+00	2.71E-01	2.72E+00	6.01E-01
ENSBTAG00000004625	-2.37E-01	1.00E+00	-5.36E-01	1.24E-01	-7.16E-01	2.60E-02	-8.82E-01	5.43E-03	-1.85E-01	1.00E+00
ENSBTAG00000001420	-1.81E-01	9.47E-01	-5.63E-01	9.59E-03	-3.54E-01	1.45E-01	-2.91E-01	2.72E-01	-9.72E-02	1.00E+00
ENSBTAG00000039071	-6.95E-01	4.57E-01	-7.26E-01	1.30E-01	-1.47E+00	5.09E-04	-1.90E+00	7.64E-06	1.41E-01	1.00E+00

ENSBTAG00000035572	3.60E-01	6.35E-01	8.35E-01	1.21E-03	7.48E-01	4.05E-03	7.08E-01	8.29E-03	8.16E-02	1.00E+00
ENSBTAG00000002446	0.00E+00	1.00E+00	0.00E+00	1.00E+00	-5.13E-15	1.00E+00	0.00E+00	1.00E+00	0.00E+00	1.00E+00
ENSBTAG00000005316	-5.57E-02	1.00E+00	-2.14E-01	4.22E-01	-2.03E-01	4.48E-01	-1.86E-02	1.00E+00	-1.47E-01	9.83E-01
ENSBTAG00000019197	2.90E-01	8.09E-01	1.44E+00	1.78E-09	1.33E+00	2.70E-08	9.19E-01	2.40E-04	2.48E-01	8.33E-01
ENSBTAG00000045310	-7.79E-01	1.00E+00	-2.70E+00	2.42E-01	3.56E-01	1.00E+00	-2.70E+00	2.05E-01	-7.86E-01	1.00E+00
ENSBTAG00000018805	-1.12E+00	5.83E-02	-3.16E+00	4.02E-13	-1.39E+00	1.01E-03	-6.26E-01	2.25E-01	-5.16E-02	1.00E+00
ENSBTAG00000027416	0.00E+00	1.00E+00	0.00E+00	1.00E+00	-5.13E-15	1.00E+00	0.00E+00	1.00E+00	0.00E+00	1.00E+00
ENSBTAG00000013854	0.00E+00	1.00E+00	0.00E+00	1.00E+00	-5.13E-15	1.00E+00	0.00E+00	1.00E+00	1.93E+00	8.00E-01
ENSBTAG00000007648	-7.36E-01	4.90E-02	-8.89E-01	1.09E-03	-5.84E-01	4.55E-02	-1.74E-01	7.81E-01	-4.88E-02	1.00E+00
ENSBTAG00000035696	-2.70E+00	5.76E-01	8.94E-02	1.00E+00	-9.22E-01	8.08E-01	-2.70E+00	2.05E-01	1.80E-02	1.00E+00
ENSBTAG00000017740	-1.25E+00	8.10E-04	-4.01E+00	2.14E-27	-5.04E+00	3.14E-37	-4.78E+00	2.98E-35	-6.83E-02	1.00E+00
ENSBTAG00000037509	-2.30E-01	8.99E-01	-1.27E+00	9.67E-08	-2.85E+00	4.12E-32	-4.13E+00	7.75E-59	1.67E-01	1.00E+00
ENSBTAG00000039722	-1.11E-01	1.00E+00	-1.05E+00	1.13E-05	-3.15E+00	3.70E-37	-4.40E+00	1.32E-60	-4.92E-01	3.19E-01
ENSBTAG00000039643	-5.34E-01	8.06E-01	-1.52E+00	1.09E-03	-2.85E+00	7.07E-10	-3.89E+00	4.90E-16	-3.18E-01	1.00E+00
ENSBTAG00000017938	3.81E-01	8.57E-01	-1.67E+00	2.18E-04	-3.03E+00	6.03E-10	-3.06E+00	5.31E-10	6.05E-02	1.00E+00
ENSBTAG00000027419	-2.32E-02	1.00E+00	-2.02E+00	1.36E-03	-4.40E+00	2.84E-11	-5.65E+00	9.21E-16	5.55E-01	8.90E-01
ENSBTAG00000022571	-5.51E-01	1.96E-01	-1.71E+00	4.49E-12	-3.62E+00	1.51E-44	-5.11E+00	7.35E-76	-5.12E-01	3.32E-01
ENSBTAG00000022564	-2.15E-01	1.00E+00	-1.26E+00	3.05E-05	-3.46E+00	5.53E-29	-4.70E+00	7.25E-47	-7.41E-02	1.00E+00
ENSBTAG00000039289	0.00E+00	1.00E+00	0.00E+00	1.00E+00	-5.13E-15	1.00E+00	0.00E+00	1.00E+00	0.00E+00	1.00E+00
ENSBTAG00000021368	0.00E+00	1.00E+00	0.00E+00	1.00E+00	-5.13E-15	1.00E+00	0.00E+00	1.00E+00	0.00E+00	1.00E+00
ENSBTAG00000022570	0.00E+00	1.00E+00	0.00E+00	1.00E+00	-5.13E-15	1.00E+00	0.00E+00	1.00E+00	0.00E+00	1.00E+00
ENSBTAG00000037453	-8.05E-01	1.49E-01	-2.27E+00	3.34E-09	-4.21E+00	6.28E-21	-3.91E+00	2.01E-19	-3.37E-01	8.40E-01
ENSBTAG00000038260	-2.51E-01	1.00E+00	-1.32E+00	3.20E-03	-1.21E+00	5.70E-03	-1.40E+00	1.51E-03	5.16E-01	7.23E-01
ENSBTAG00000017191	-1.35E-01	1.00E+00	9.83E-02	9.77E-01	2.13E-01	5.40E-01	-2.84E-02	1.00E+00	-2.53E-01	8.00E-01
ENSBTAG00000015188	1.43E+00	6.66E-02	2.56E+00	3.41E-06	1.57E+00	5.09E-03	7.82E-01	2.52E-01	-2.49E-01	1.00E+00
ENSBTAG00000046128	0.00E+00	1.00E+00	0.00E+00	1.00E+00	-5.13E-15	1.00E+00	0.00E+00	1.00E+00	0.00E+00	1.00E+00
ENSBTAG00000002685	1.81E-01	9.87E-01	9.84E-01	5.44E-06	9.71E-01	6.94E-06	5.82E-01	1.34E-02	-8.44E-02	1.00E+00
ENSBTAG00000002683	1.84E-01	1.00E+00	-2.23E-01	5.92E-01	-3.78E-02	1.00E+00	-2.44E-01	5.32E-01	-5.06E-01	3.72E-01
ENSBTAG00000044982	0.00E+00	1.00E+00	0.00E+00	1.00E+00	-5.13E-15	1.00E+00	0.00E+00	1.00E+00	0.00E+00	1.00E+00
ENSBTAG00000014194	3.26E-01	8.94E-01	-1.92E+00	7.68E-08	-2.37E+00	3.29E-11	-2.04E+00	8.93E-09	-3.83E-01	8.00E-01
ENSBTAG00000039356	0.00E+00	1.00E+00	0.00E+00	1.00E+00	-5.13E-15	1.00E+00	0.00E+00	1.00E+00	0.00E+00	1.00E+00
ENSBTAG00000037833	0.00E+00	1.00E+00	0.00E+00	1.00E+00	-5.13E-15	1.00E+00	0.00E+00	1.00E+00	0.00E+00	1.00E+00
ENSBTAG00000000683	-4.98E-01	9.88E-02	4.52E-01	3.66E-02	2.25E-01	4.21E-01	-3.28E-01	1.77E-01	3.95E-01	3.54E-01
ENSBTAG00000004075	-5.42E-01	5.76E-01	-7.93E-01	4.77E-02	-1.28E+00	5.45E-04	-1.49E+00	6.03E-05	-4.14E-01	8.00E-01
ENSBTAG00000014917	2.82E-01	8.18E-01	1.79E+00	3.25E-14	1.90E+00	5.21E-16	1.20E+00	5.35E-07	-4.45E-01	4.34E-01

ENSBTAG00000045453	0.00E+00	1.00E+00	0.00E+00	1.00E+00	-5.13E-15	1.00E+00	0.00E+00	1.00E+00	0.00E+00	1.00E+00
ENSBTAG00000013899	-1.10E-01	1.00E+00	6.20E-01	1.24E-02	7.51E-01	1.72E-03	4.23E-01	1.28E-01	2.42E-01	8.19E-01
ENSBTAG00000006531	6.34E-02	1.00E+00	-1.84E+00	1.55E-10	-1.72E+00	1.47E-09	-3.23E-01	4.33E-01	5.35E-01	4.10E-01
ENSBTAG00000043211	0.00E+00	1.00E+00	0.00E+00	1.00E+00	-5.13E-15	1.00E+00	0.00E+00	1.00E+00	0.00E+00	1.00E+00
ENSBTAG00000043795	0.00E+00	1.00E+00	0.00E+00	1.00E+00	-5.13E-15	1.00E+00	0.00E+00	1.00E+00	0.00E+00	1.00E+00
ENSBTAG00000002578	-2.55E-02	1.00E+00	-6.60E-01	3.13E-04	-1.53E-01	6.05E-01	1.61E-01	5.79E-01	-1.07E-01	1.00E+00
ENSBTAG00000027937	4.60E-01	3.04E-01	1.72E+00	5.54E-14	1.27E+00	2.82E-08	9.05E-01	1.46E-04	-1.40E-01	1.00E+00
ENSBTAG00000011010	6.84E-01	1.00E+00	-5.64E-01	9.20E-01	-1.32E-01	1.00E+00	-1.33E+00	4.42E-01	-1.96E+00	6.25E-01
ENSBTAG00000017346	7.61E-02	1.00E+00	-3.35E-01	5.27E-01	-3.09E-01	5.68E-01	-1.33E-01	1.00E+00	-2.76E-01	9.77E-01
ENSBTAG00000042488	0.00E+00	1.00E+00	0.00E+00	1.00E+00	-5.13E-15	1.00E+00	0.00E+00	1.00E+00	0.00E+00	1.00E+00
ENSBTAG00000032366	5.07E-01	5.76E-01	9.97E-01	2.98E-03	1.23E+00	1.46E-04	1.52E+00	2.04E-06	5.18E-01	5.85E-01
ENSBTAG00000006063	-4.57E-02	1.00E+00	4.66E-01	2.32E-02	8.39E-01	8.11E-06	6.54E-01	8.80E-04	-6.82E-02	1.00E+00
ENSBTAG00000006065	-2.66E-01	8.23E-01	-4.20E-01	1.29E-01	1.97E-01	6.14E-01	3.11E-01	3.19E-01	-4.98E-01	3.09E-01
ENSBTAG00000006066	-1.61E-01	1.00E+00	-1.22E+00	1.87E-09	-1.12E+00	3.04E-08	-3.06E-01	2.43E-01	-8.21E-02	1.00E+00
ENSBTAG00000042644	0.00E+00	1.00E+00	0.00E+00	1.00E+00	-5.13E-15	1.00E+00	0.00E+00	1.00E+00	0.00E+00	1.00E+00
ENSBTAG00000020668	4.88E-02	1.00E+00	-2.76E-01	2.01E-01	-6.55E-01	2.29E-04	-7.53E-01	2.32E-05	-2.55E-01	6.27E-01
ENSBTAG00000015872	2.71E+00	5.76E-01	0.00E+00	1.00E+00	-5.13E-15	1.00E+00	3.40E+00	8.29E-02	0.00E+00	1.00E+00
ENSBTAG00000008293	-4.17E-01	7.32E-01	1.40E+00	1.33E-05	1.14E+00	4.61E-04	3.10E-01	5.28E-01	-7.39E-01	2.28E-01
ENSBTAG00000014048	4.81E-01	4.26E-01	1.15E+00	1.57E-05	9.49E-01	4.61E-04	4.25E-01	2.15E-01	2.54E-01	9.00E-01
ENSBTAG00000011782	-5.19E-01	1.00E+00	2.09E+00	4.55E-05	2.25E+00	5.42E-06	6.97E-01	3.84E-01	-8.01E-01	8.00E-01
ENSBTAG00000001314	3.44E-01	8.08E-01	1.21E+00	2.28E-05	2.47E+00	3.75E-19	2.45E+00	8.34E-19	5.22E-02	1.00E+00
ENSBTAG00000014623	2.84E-01	9.18E-01	-1.02E+00	2.77E-03	-6.65E-01	5.86E-02	-7.46E-01	3.40E-02	7.50E-01	1.51E-01
ENSBTAG00000000065	-4.18E-01	2.88E-01	-9.80E-01	2.63E-06	-4.90E-01	3.16E-02	-6.50E-01	3.23E-03	-3.66E-01	4.77E-01
ENSBTAG00000000066	-1.02E+00	9.70E-01	-1.36E+00	4.43E-01	-2.19E+00	1.50E-01	-3.06E+00	4.81E-02	-7.03E-01	1.00E+00
ENSBTAG00000020465	7.70E-03	1.00E+00	8.09E-01	1.23E-01	-9.53E-02	1.00E+00	-2.45E-01	8.81E-01	-1.58E-01	1.00E+00
ENSBTAG00000046562	0.00E+00	1.00E+00	0.00E+00	1.00E+00	-5.13E-15	1.00E+00	0.00E+00	1.00E+00	0.00E+00	1.00E+00
ENSBTAG00000005111	7.17E-01	4.49E-01	1.50E+00	4.35E-04	6.46E-01	2.07E-01	9.92E-01	3.22E-02	-8.19E-01	4.23E-01
ENSBTAG00000048291	1.15E-01	1.00E+00	1.91E+00	8.94E-09	2.00E+00	1.19E-09	1.05E+00	3.81E-03	2.62E-01	1.00E+00
ENSBTAG00000045753	2.15E+00	2.32E-01	3.13E+00	6.22E-04	2.09E+00	6.42E-02	2.19E+00	5.00E-02	1.19E+00	8.26E-01
ENSBTAG00000046276	3.43E-01	9.27E-01	2.02E+00	1.83E-09	1.71E+00	3.73E-07	1.31E+00	2.41E-04	4.35E-01	8.00E-01
ENSBTAG00000046776	0.00E+00	1.00E+00	0.00E+00	1.00E+00	-5.13E-15	1.00E+00	0.00E+00	1.00E+00	0.00E+00	1.00E+00
ENSBTAG00000047638	0.00E+00	1.00E+00	0.00E+00	1.00E+00	-5.13E-15	1.00E+00	0.00E+00	1.00E+00	0.00E+00	1.00E+00
ENSBTAG00000047320	0.00E+00	1.00E+00	0.00E+00	1.00E+00	-5.13E-15	1.00E+00	0.00E+00	1.00E+00	0.00E+00	1.00E+00
ENSBTAG00000047962	0.00E+00	1.00E+00	0.00E+00	1.00E+00	-5.13E-15	1.00E+00	0.00E+00	1.00E+00	0.00E+00	1.00E+00
ENSBTAG00000048089	0.00E+00	1.00E+00	0.00E+00	1.00E+00	-5.13E-15	1.00E+00	0.00E+00	1.00E+00	0.00E+00	1.00E+00

ENSBTAG00000047653	0.00E+00	1.00E+00	0.00E+00	1.00E+00	-5.13E-15	1.00E+00	0.00E+00	1.00E+00	0.00E+00	1.00E+00
ENSBTAG00000047102	0.00E+00	1.00E+00	0.00E+00	1.00E+00	-5.13E-15	1.00E+00	0.00E+00	1.00E+00	0.00E+00	1.00E+00
ENSBTAG00000046716	0.00E+00	1.00E+00	0.00E+00	1.00E+00	-5.13E-15	1.00E+00	0.00E+00	1.00E+00	0.00E+00	1.00E+00
ENSBTAG00000045555	0.00E+00	1.00E+00	0.00E+00	1.00E+00	-5.13E-15	1.00E+00	0.00E+00	1.00E+00	0.00E+00	1.00E+00
ENSBTAG00000046631	5.15E-01	1.00E+00	-7.21E-01	9.45E-01	-9.29E-01	8.06E-01	-2.71E+00	2.05E-01	-2.71E+00	6.01E-01
ENSBTAG00000007199	2.91E+00	1.02E-01	1.40E+00	5.18E-01	-1.91E+00	4.29E-01	2.59E+00	5.67E-02	8.13E-01	1.00E+00
ENSBTAG00000019811	-1.09E-01	1.00E+00	-1.18E+00	2.89E-07	-3.35E+00	1.41E-44	-4.64E+00	3.17E-74	-6.95E-02	1.00E+00
ENSBTAG00000032156	5.34E-03	1.00E+00	-1.91E+00	4.69E-01	6.44E-01	9.78E-01	-1.91E+00	4.39E-01	7.99E-01	1.00E+00
ENSBTAG00000019504	-2.45E-01	1.00E+00	-1.26E+00	2.97E-01	3.47E-03	1.00E+00	7.30E-01	4.97E-01	-7.73E-01	9.07E-01
ENSBTAG00000001112	4.62E-02	1.00E+00	-3.81E-01	5.03E-01	5.83E-02	1.00E+00	3.28E-01	5.65E-01	6.72E-01	4.16E-01
ENSBTAG00000007922	1.11E+00	2.66E-01	2.48E+00	1.74E-06	2.20E+00	2.52E-05	2.37E+00	6.02E-06	1.10E+00	3.47E-01
ENSBTAG00000007920	-1.46E-01	1.00E+00	-4.05E-01	7.50E-02	-3.29E-01	1.70E-01	-1.38E-01	7.49E-01	-1.41E-01	1.00E+00
ENSBTAG00000029793	0.00E+00	1.00E+00	0.00E+00	1.00E+00	-5.13E-15	1.00E+00	0.00E+00	1.00E+00	0.00E+00	1.00E+00
ENSBTAG00000013545	1.03E+00	2.57E-03	2.87E+00	3.79E-25	2.42E+00	9.05E-19	2.15E+00	5.76E-15	4.92E-01	5.00E-01
ENSBTAG00000008439	-3.07E-01	7.85E-01	-4.94E-01	8.94E-02	-3.26E-01	3.26E-01	-9.89E-02	1.00E+00	-1.53E-01	1.00E+00
ENSBTAG00000008436	9.64E-02	1.00E+00	-8.46E-01	3.07E-02	-9.48E-01	1.20E-02	-1.01E+00	8.04E-03	-1.77E-01	1.00E+00
ENSBTAG00000045784	-2.13E-01	1.00E+00	-9.61E-01	1.43E-02	6.39E-01	1.31E-01	1.15E+00	2.32E-03	-2.90E-02	1.00E+00
ENSBTAG00000006578	2.21E-01	1.00E+00	3.04E+00	8.25E-08	1.89E+00	1.50E-03	3.33E-01	8.88E-01	9.95E-02	1.00E+00
ENSBTAG00000018735	-3.26E-02	1.00E+00	1.20E-02	1.00E+00	2.77E-01	3.41E-01	1.59E-01	7.20E-01	-1.17E-01	1.00E+00
ENSBTAG00000018732	-8.95E-01	7.45E-02	-1.05E+00	3.20E-03	-1.22E+00	4.53E-04	-9.13E-01	1.28E-02	-4.71E-01	6.67E-01
ENSBTAG00000027412	-9.54E-02	1.00E+00	-6.35E-01	2.03E-03	-1.24E+00	1.67E-10	-1.46E+00	8.48E-14	-2.83E-01	6.47E-01
ENSBTAG00000013167	-7.29E-01	1.69E-01	-1.54E+00	2.29E-06	-2.76E+00	7.60E-17	-3.22E+00	2.97E-21	-7.41E-02	1.00E+00
ENSBTAG00000013163	-1.31E+00	1.30E-01	-1.23E+00	4.14E-02	-2.45E+00	2.92E-05	-2.96E+00	1.65E-06	-9.49E-01	4.64E-01
ENSBTAG00000013160	-6.37E-01	1.00E+00	2.04E-01	1.00E+00	-1.25E+00	2.34E-01	-1.58E+00	1.35E-01	3.48E-01	1.00E+00
ENSBTAG00000003848	-2.95E-01	8.23E-01	-1.35E+00	2.43E-07	-1.32E+00	4.24E-07	-1.10E+00	3.69E-05	2.06E-01	9.82E-01
ENSBTAG00000003494	2.40E-01	1.00E+00	-1.66E+00	6.04E-07	-1.88E+00	1.05E-08	-6.20E-01	9.72E-02	-1.26E-01	1.00E+00
ENSBTAG00000027409	4.37E-01	1.00E+00	1.27E+00	4.18E-02	1.81E+00	7.13E-04	-1.22E-01	1.00E+00	-1.83E-01	1.00E+00
ENSBTAG00000013776	-1.86E-02	1.00E+00	-1.12E-01	9.12E-01	2.43E-01	4.31E-01	3.12E-01	2.72E-01	-2.28E-01	8.22E-01
ENSBTAG00000013753	-6.61E-02	1.00E+00	-5.51E-01	4.18E-03	-9.34E-01	2.78E-07	-1.12E+00	7.50E-10	-1.72E-01	8.90E-01
ENSBTAG00000020454	-1.77E-01	1.00E+00	-1.38E+00	8.68E-09	-2.10E+00	2.33E-18	-1.27E+00	1.24E-07	1.98E-01	9.52E-01
ENSBTAG00000015379	1.11E-01	1.00E+00	7.08E-01	6.31E-05	7.94E-01	5.21E-06	5.07E-01	6.97E-03	-2.34E-02	1.00E+00
ENSBTAG00000008027	2.71E+00	5.76E-01	1.98E+00	4.43E-01	1.78E+00	4.79E-01	1.77E+00	4.97E-01	0.00E+00	1.00E+00
ENSBTAG00000008026	3.21E+00	3.75E-01	4.23E+00	1.32E-02	2.56E+00	2.54E-01	3.41E+00	8.27E-02	1.91E+00	8.00E-01
ENSBTAG00000000825	-2.39E-01	1.00E+00	-5.94E-01	1.89E-01	-7.25E-01	8.76E-02	-7.03E-01	1.07E-01	-2.68E-01	1.00E+00
ENSBTAG00000011425	1.32E-01	1.00E+00	-6.98E-01	2.94E-04	-6.92E-01	3.02E-04	-6.00E-01	2.51E-03	2.58E-01	6.83E-01

ENSBTAG00000001320	-1.37E-01	1.00E+00	7.32E-01	8.53E-05	6.65E-01	3.90E-04	-1.76E-02	1.00E+00	-1.45E-02	1.00E+00
ENSBTAG00000003863	-1.23E-01	1.00E+00	-5.75E-02	1.00E+00	1.02E-01	1.00E+00	-7.40E-01	7.79E-03	3.42E-01	7.13E-01
ENSBTAG00000021816	0.00E+00	1.00E+00	1.98E+00	4.43E-01	1.78E+00	4.79E-01	0.00E+00	1.00E+00	0.00E+00	1.00E+00
ENSBTAG00000021817	0.00E+00	1.00E+00	0.00E+00	1.00E+00	2.55E+00	2.54E-01	0.00E+00	1.00E+00	1.91E+00	8.00E-01
ENSBTAG00000011458	1.55E-01	1.00E+00	-1.40E-01	9.45E-01	-1.35E+00	7.02E-06	-2.46E+00	9.40E-16	3.05E-02	1.00E+00
ENSBTAG00000037757	5.42E-01	5.03E-01	6.12E-01	1.11E-01	3.83E-01	4.11E-01	-4.20E-01	3.92E-01	-1.57E-01	1.00E+00
ENSBTAG00000018813	-6.52E-02	1.00E+00	-3.41E-01	2.28E-01	-4.77E-01	6.05E-02	-4.01E-01	1.42E-01	-1.43E-01	1.00E+00
ENSBTAG00000018812	-1.42E-02	1.00E+00	1.12E+00	1.56E-07	2.04E+00	2.75E-22	1.16E+00	6.99E-08	-2.95E-01	6.97E-01
ENSBTAG00000042198	0.00E+00	1.00E+00	0.00E+00	1.00E+00	-5.13E-15	1.00E+00	0.00E+00	1.00E+00	0.00E+00	1.00E+00
ENSBTAG00000042607	0.00E+00	1.00E+00	0.00E+00	1.00E+00	-5.13E-15	1.00E+00	0.00E+00	1.00E+00	0.00E+00	1.00E+00
ENSBTAG00000042631	0.00E+00	1.00E+00	0.00E+00	1.00E+00	-5.13E-15	1.00E+00	0.00E+00	1.00E+00	0.00E+00	1.00E+00
ENSBTAG00000043077	0.00E+00	1.00E+00	0.00E+00	1.00E+00	-5.13E-15	1.00E+00	0.00E+00	1.00E+00	0.00E+00	1.00E+00
ENSBTAG00000043306	0.00E+00	1.00E+00	0.00E+00	1.00E+00	-5.13E-15	1.00E+00	0.00E+00	1.00E+00	0.00E+00	1.00E+00
ENSBTAG00000018811	-1.03E+00	2.15E-01	-1.89E-01	9.78E-01	2.52E+00	6.89E-13	5.75E-01	2.69E-01	-1.17E-01	1.00E+00
ENSBTAG00000001038	3.06E-01	6.71E-01	1.36E+00	4.61E-10	1.37E+00	3.09E-10	1.16E+00	1.41E-07	-8.67E-02	1.00E+00
ENSBTAG00000044240	0.00E+00	1.00E+00	0.00E+00	1.00E+00	-5.13E-15	1.00E+00	0.00E+00	1.00E+00	0.00E+00	1.00E+00
ENSBTAG00000037464	-2.71E+00	5.76E-01	-2.71E+00	2.42E-01	1.00E+00	6.00E-01	-1.85E-01	1.00E+00	-7.98E-01	1.00E+00
ENSBTAG00000001785	1.11E+00	2.59E-01	3.49E+00	7.87E-11	4.43E+00	4.75E-16	5.36E+00	2.11E-21	2.79E+00	7.34E-06
ENSBTAG00000022534	0.00E+00	1.00E+00	0.00E+00	1.00E+00	-5.13E-15	1.00E+00	1.77E+00	4.97E-01	3.60E+00	3.09E-01
ENSBTAG00000042234	2.91E-01	1.00E+00	1.99E+00	2.85E-02	-1.05E+00	5.95E-01	-5.72E-01	9.17E-01	-8.76E-01	1.00E+00
ENSBTAG00000003561	1.01E-01	1.00E+00	1.31E+00	9.77E-07	1.58E+00	1.14E-09	9.26E-01	9.98E-04	-1.34E-01	1.00E+00
ENSBTAG00000045520	-4.76E-02	1.00E+00	-2.34E+00	6.57E-03	-4.37E+00	1.03E-06	-4.75E+00	2.74E-07	-5.77E-02	1.00E+00
ENSBTAG00000007213	2.44E-01	7.58E-01	4.63E-01	2.68E-02	-2.36E-01	3.69E-01	-8.68E-01	8.05E-06	-4.97E-02	1.00E+00
ENSBTAG00000048087	-7.87E-01	1.00E+00	-7.17E-01	9.45E-01	-9.22E-01	8.08E-01	-9.40E-01	8.06E-01	8.98E-01	1.00E+00
ENSBTAG00000039016	5.35E-01	7.98E-01	8.65E-01	8.13E-02	8.60E-01	7.59E-02	7.20E-01	1.75E-01	9.06E-01	3.11E-01
ENSBTAG00000039520	2.02E-01	1.00E+00	7.66E-01	2.03E-01	5.50E-01	4.28E-01	2.24E-01	9.56E-01	2.17E-01	1.00E+00
ENSBTAG00000011638	-1.10E+00	8.23E-01	-4.33E+00	1.12E-02	-1.79E+00	1.72E-01	-6.26E-01	7.61E-01	-1.63E+00	6.11E-01
ENSBTAG00000027221	-3.65E-01	9.60E-01	-4.39E-01	4.80E-01	-4.86E-01	4.09E-01	-2.25E-01	8.75E-01	-2.25E-01	1.00E+00
ENSBTAG00000019984	1.36E-01	1.00E+00	-1.40E-01	9.60E-01	5.84E-01	1.03E-01	6.90E-01	4.93E-02	-3.76E-02	1.00E+00
ENSBTAG00000031715	-1.33E-01	1.00E+00	-1.50E+00	1.35E-12	-6.37E-01	4.10E-03	-3.25E-01	2.26E-01	2.03E-01	8.74E-01
ENSBTAG00000006352	-9.07E-01	9.61E-01	-1.55E-01	1.00E+00	4.15E-02	1.00E+00	3.62E-01	9.83E-01	1.25E-02	1.00E+00
ENSBTAG00000047704	0.00E+00	1.00E+00	1.99E+00	4.43E-01	-5.13E-15	1.00E+00	0.00E+00	1.00E+00	0.00E+00	1.00E+00
ENSBTAG00000045701	1.25E-02	1.00E+00	-2.71E+00	2.42E-01	-9.24E-01	8.08E-01	-9.40E-01	8.06E-01	-2.71E+00	6.01E-01
ENSBTAG00000021423	-1.31E+00	9.32E-01	-1.24E+00	6.09E-01	-3.22E+00	9.40E-02	-3.22E+00	9.87E-02	5.71E-03	1.00E+00
ENSBTAG00000021837	-5.28E-01	3.24E-01	-4.22E-01	2.10E-01	4.15E-01	1.97E-01	1.69E-01	7.99E-01	-1.80E-02	1.00E+00

ENSBTAG00000021836	-5.35E-01	4.19E-01	-1.42E+00	1.13E-05	-1.25E+00	7.68E-05	-1.38E+00	1.57E-05	-4.53E-01	6.04E-01
ENSBTAG00000011662	-2.07E+00	1.24E-03	-2.20E+00	6.54E-05	-7.80E-01	2.25E-01	-1.35E+00	1.90E-02	-2.77E-01	1.00E+00
ENSBTAG00000011656	-9.89E-03	1.00E+00	3.66E-01	9.64E-02	4.11E-01	5.36E-02	-1.65E-04	1.00E+00	2.45E-02	1.00E+00
ENSBTAG00000043826	0.00E+00	1.00E+00	0.00E+00	1.00E+00	-5.13E-15	1.00E+00	0.00E+00	1.00E+00	0.00E+00	1.00E+00
ENSBTAG00000011655	-1.59E+00	1.77E-04	-3.47E+00	2.75E-14	-3.06E+00	1.49E-13	-2.08E+00	4.29E-08	1.30E-04	1.00E+00
ENSBTAG00000011646	-6.87E-01	3.63E-01	-3.91E+00	1.93E-22	-1.71E+00	4.65E-06	-7.74E-01	6.66E-02	1.92E-01	1.00E+00
ENSBTAG00000015798	-3.11E-01	6.21E-01	-2.04E-01	5.39E-01	2.74E-01	3.40E-01	-2.38E-01	4.52E-01	2.10E-02	1.00E+00
ENSBTAG00000043697	0.00E+00	1.00E+00	3.68E+00	5.07E-02	2.55E+00	2.54E-01	2.54E+00	2.71E-01	1.91E+00	8.00E-01
ENSBTAG00000015796	-5.80E-01	1.15E-01	-9.18E-01	1.42E-04	-4.43E-01	1.03E-01	9.00E-02	1.00E+00	3.47E-01	6.07E-01
ENSBTAG00000021232	-1.87E-01	8.23E-01	3.99E-02	1.00E+00	2.32E-01	2.89E-01	9.89E-02	8.49E-01	-4.04E-02	1.00E+00
ENSBTAG00000031906	-3.52E-01	9.32E-01	5.65E-01	1.94E-01	4.39E-01	3.54E-01	6.71E-01	9.54E-02	5.40E-01	6.01E-01
ENSBTAG00000016667	1.48E-01	1.00E+00	-1.39E+00	1.05E-04	-7.62E-02	1.00E+00	-4.82E-01	2.60E-01	-2.02E-01	1.00E+00
ENSBTAG00000016664	-5.29E-01	5.91E-01	8.10E-02	1.00E+00	-2.45E+00	1.58E-11	-2.88E+00	5.52E-15	-1.78E-01	1.00E+00
ENSBTAG00000007501	-3.84E-02	1.00E+00	4.06E+00	3.44E-23	1.24E+00	2.38E-03	2.69E-01	7.65E-01	3.45E-02	1.00E+00
ENSBTAG00000007498	-2.22E-01	8.96E-01	1.54E+00	6.66E-12	1.06E+00	2.97E-06	2.92E-01	3.49E-01	-1.96E-01	9.34E-01
ENSBTAG00000007497	4.33E-02	1.00E+00	9.68E-01	9.83E-07	6.97E-01	6.12E-04	9.59E-01	1.51E-06	5.68E-02	1.00E+00
ENSBTAG00000027407	1.88E+00	2.13E-04	7.11E+00	1.17E-52	8.34E+00	1.67E-66	8.11E+00	6.43E-64	1.82E-01	1.00E+00
ENSBTAG00000047150	-2.08E-01	9.36E-01	-1.05E+00	8.40E-06	-8.07E-01	6.32E-04	-9.00E-01	1.50E-04	3.93E-01	4.90E-01
ENSBTAG00000021096	0.00E+00	1.00E+00	0.00E+00	1.00E+00	-5.13E-15	1.00E+00	0.00E+00	1.00E+00	0.00E+00	1.00E+00
ENSBTAG00000038974	-4.00E-01	1.00E+00	-2.40E+00	2.16E-03	-8.58E-01	2.40E-01	-1.59E+00	2.33E-02	-2.72E-01	1.00E+00
ENSBTAG00000014769	-4.41E-03	1.00E+00	4.84E-01	2.20E-02	7.26E-01	2.27E-04	2.06E-01	4.85E-01	1.06E-01	1.00E+00
ENSBTAG00000027405	-1.91E+00	8.23E-01	-1.91E+00	4.69E-01	-1.25E-01	1.00E+00	6.22E-01	9.94E-01	6.33E-03	1.00E+00
ENSBTAG00000001985	-4.58E-01	1.00E+00	-4.07E+00	8.14E-05	-1.53E+00	4.70E-02	-2.12E+00	7.90E-03	-6.79E-02	1.00E+00
ENSBTAG00000044091	-1.31E+00	2.02E-01	-4.31E+00	3.75E-06	-3.23E+00	1.93E-05	-2.36E+00	6.26E-04	4.22E-01	9.20E-01
ENSBTAG00000031800	-2.19E-01	8.69E-01	-6.14E-01	7.74E-03	-1.31E+00	9.99E-10	-1.45E+00	2.31E-11	-3.42E-01	5.93E-01
ENSBTAG00000021685	4.97E-01	1.00E+00	1.93E+00	1.36E-01	-1.67E-01	1.00E+00	1.23E+00	4.39E-01	1.76E+00	5.66E-01
ENSBTAG00000003030	-2.33E-01	1.00E+00	-8.26E-01	6.64E-01	-4.10E-01	9.73E-01	-4.26E-01	9.60E-01	-9.15E-01	9.19E-01
ENSBTAG00000031764	-7.97E-02	1.00E+00	4.18E-02	1.00E+00	-1.19E-01	8.55E-01	-2.01E-02	1.00E+00	1.39E-01	1.00E+00
ENSBTAG00000017198	-4.96E-01	1.00E+00	4.67E-01	9.94E-01	-6.61E-01	8.93E-01	-3.21E+00	9.90E-02	1.03E-03	1.00E+00
ENSBTAG00000004582	3.14E-01	1.00E+00	1.05E+00	8.35E-02	6.88E-01	3.37E-01	-3.74E-01	8.21E-01	3.63E-01	1.00E+00
ENSBTAG00000004916	6.32E-02	1.00E+00	9.25E-01	6.71E-06	8.01E-01	1.12E-04	5.17E-01	2.23E-02	1.05E-02	1.00E+00
ENSBTAG00000004911	1.25E-02	1.00E+00	1.39E+00	5.16E-01	6.45E-01	9.78E-01	-1.91E+00	4.39E-01	-1.91E+00	8.00E-01
ENSBTAG00000010383	-2.80E-03	1.00E+00	1.11E+00	1.24E-09	6.51E-01	6.19E-04	2.62E-02	1.00E+00	-1.59E-01	9.46E-01
ENSBTAG00000029962	0.00E+00	1.00E+00	0.00E+00	1.00E+00	-5.13E-15	1.00E+00	0.00E+00	1.00E+00	0.00E+00	1.00E+00
ENSBTAG00000044990	0.00E+00	1.00E+00	0.00E+00	1.00E+00	-5.13E-15	1.00E+00	0.00E+00	1.00E+00	0.00E+00	1.00E+00

ENSBTAG00000010873	-1.71E-01	1.00E+00	-1.21E+00	3.89E-01	-4.51E+00	3.27E-03	3.91E-01	9.18E-01	8.24E-01	8.00E-01
ENSBTAG00000001829	4.42E-01	9.37E-01	2.72E+00	7.11E-08	2.24E+00	8.22E-06	2.23E+00	1.16E-05	4.79E-01	8.54E-01
ENSBTAG00000001827	-3.02E-01	6.56E-01	-4.95E-01	3.75E-02	-3.29E-01	2.18E-01	-2.57E-01	4.05E-01	-4.82E-02	1.00E+00
ENSBTAG00000006002	-3.03E-01	6.21E-01	3.27E-01	2.05E-01	-9.36E-02	9.65E-01	-5.24E-01	2.37E-02	9.83E-02	1.00E+00
ENSBTAG00000046520	-4.62E-01	4.77E-01	-2.80E-01	4.85E-01	4.34E-01	1.72E-01	-2.40E-01	5.92E-01	1.89E-01	1.00E+00
ENSBTAG00000015581	5.38E-01	1.00E+00	1.94E+00	7.90E-04	1.20E+00	7.72E-02	1.46E-01	1.00E+00	8.00E-01	7.76E-01
ENSBTAG00000031718	-1.12E-01	1.00E+00	1.02E+00	3.95E-06	1.28E+00	3.59E-09	9.91E-01	8.60E-06	-4.70E-03	1.00E+00
ENSBTAG00000031716	-8.01E-01	9.35E-02	-1.52E+00	6.51E-06	-2.24E-01	6.96E-01	-1.36E-01	9.63E-01	8.60E-02	1.00E+00
ENSBTAG00000021727	-8.75E-01	1.00E+00	8.45E-01	5.86E-01	3.73E-01	1.00E+00	7.32E-01	6.71E-01	3.01E-01	1.00E+00
ENSBTAG00000016388	5.98E+00	2.71E-55	6.42E+00	6.30E-62	6.01E+00	9.37E-57	4.20E+00	5.25E-33	-9.11E-04	1.00E+00
ENSBTAG00000036363	0.00E+00	1.00E+00	0.00E+00	1.00E+00	-5.13E-15	1.00E+00	0.00E+00	1.00E+00	0.00E+00	1.00E+00
ENSBTAG00000029790	0.00E+00	1.00E+00	0.00E+00	1.00E+00	-5.13E-15	1.00E+00	0.00E+00	1.00E+00	0.00E+00	1.00E+00
ENSBTAG00000047944	-1.36E+00	1.37E-02	-3.32E+00	1.77E-11	-1.18E+00	7.37E-03	-2.16E+00	1.19E-06	-9.31E-01	2.60E-01
ENSBTAG00000003163	6.49E-01	9.96E-01	9.16E-02	1.00E+00	-2.36E-03	1.00E+00	1.49E-01	1.00E+00	6.44E-01	9.65E-01
ENSBTAG00000020797	-7.83E-02	1.00E+00	-3.97E-02	1.00E+00	1.03E+00	6.11E-04	5.22E-01	1.43E-01	-1.39E-01	1.00E+00
ENSBTAG00000020795	7.81E-02	1.00E+00	3.67E-01	2.77E-01	3.92E-01	2.32E-01	7.91E-01	4.62E-03	2.23E-01	9.40E-01
ENSBTAG00000045225	2.70E+00	5.76E-01	1.98E+00	4.43E-01	-5.13E-15	1.00E+00	1.77E+00	4.97E-01	0.00E+00	1.00E+00
ENSBTAG00000003061	-5.67E-01	9.43E-02	-1.17E+00	1.43E-07	-8.17E-01	3.22E-04	-4.19E-01	1.12E-01	-7.55E-02	1.00E+00
ENSBTAG00000003058	-4.64E-01	2.30E-01	-5.25E-01	2.71E-02	-3.06E-02	1.00E+00	-9.01E-03	1.00E+00	-6.13E-01	8.02E-02
ENSBTAG00000017402	-1.56E-02	1.00E+00	8.22E-02	1.00E+00	3.49E-01	2.37E-01	6.10E-01	1.73E-02	4.13E-01	4.77E-01
ENSBTAG00000005127	-1.85E-01	1.00E+00	-6.20E-01	2.04E-02	-6.63E-01	1.18E-02	-5.10E-01	7.40E-02	-2.85E-01	8.00E-01
ENSBTAG00000021558	-1.99E-01	8.45E-01	3.86E-02	1.00E+00	5.30E-01	7.93E-03	3.64E-01	1.03E-01	5.83E-02	1.00E+00
ENSBTAG00000031625	1.14E-02	1.00E+00	-1.91E+00	4.69E-01	-1.91E+00	4.28E-01	2.24E+00	1.30E-01	1.83E-02	1.00E+00
ENSBTAG00000014442	-2.25E-01	8.81E-01	-7.47E-01	1.69E-03	-5.24E-02	1.00E+00	5.91E-02	1.00E+00	1.44E-01	1.00E+00
ENSBTAG00000014438	-2.55E-01	8.84E-01	-7.47E-01	7.04E-03	4.51E-02	1.00E+00	7.45E-02	1.00E+00	-3.03E-01	8.00E-01
ENSBTAG00000047991	1.47E-01	1.00E+00	1.65E-01	8.48E-01	9.20E-01	1.38E-03	6.68E-01	3.19E-02	3.18E-01	8.00E-01
ENSBTAG00000015058	3.58E-02	1.00E+00	-3.39E-01	6.50E-01	-1.02E+00	3.13E-02	-1.06E+00	2.83E-02	-4.75E-01	8.00E-01
ENSBTAG00000020261	-2.70E+00	5.76E-01	8.75E-02	1.00E+00	-9.18E-01	8.09E-01	7.05E-01	8.49E-01	7.09E-03	1.00E+00
ENSBTAG00000045509	4.91E-01	6.26E-01	5.63E-01	1.75E-01	1.15E-01	1.00E+00	-6.93E-01	1.04E-01	3.75E-01	8.00E-01
ENSBTAG00000027397	5.35E-01	8.23E-01	1.30E+00	3.79E-03	1.43E+00	7.66E-04	1.72E+00	3.32E-05	3.08E-01	1.00E+00
ENSBTAG00000003608	2.96E-01	1.00E+00	6.87E-01	2.29E-01	1.03E+00	3.74E-02	4.32E-01	5.47E-01	-4.42E-01	8.98E-01
ENSBTAG00000015080	-4.80E-01	1.00E+00	-9.77E-01	3.76E-01	-6.54E-02	1.00E+00	1.69E-01	1.00E+00	-1.31E+00	5.85E-01
ENSBTAG00000012109	-1.97E+00	3.57E-01	-1.38E+00	2.68E-01	-1.26E+00	2.79E-01	-1.68E-01	1.00E+00	-1.07E+00	8.00E-01
ENSBTAG00000011940	5.17E-01	8.23E-01	-1.69E-01	1.00E+00	-9.85E-02	1.00E+00	-6.76E-01	3.28E-01	-1.62E-01	1.00E+00
ENSBTAG00000004402	4.67E-01	1.35E-01	1.45E+00	1.79E-14	1.39E+00	1.23E-13	8.08E-01	4.15E-05	-2.66E-01	7.08E-01

ENSBTAG00000039208	-2.03E-01	1.00E+00	-4.86E-01	1.86E-01	-4.11E-01	2.87E-01	-6.84E-01	4.36E-02	-3.37E-01	8.00E-01
ENSBTAG00000018785	8.02E-01	1.75E-01	1.94E+00	1.77E-08	8.86E-01	1.96E-02	4.04E-01	4.39E-01	1.78E-01	1.00E+00
ENSBTAG00000018784	-1.20E-01	1.00E+00	-2.82E-01	3.04E-01	-7.63E-01	3.49E-04	-7.22E-01	9.52E-04	1.43E-01	1.00E+00
ENSBTAG00000018783	-2.40E-01	8.23E-01	-1.33E+00	1.23E-10	-9.05E-01	1.54E-05	-6.77E-01	2.08E-03	-1.06E-01	1.00E+00
ENSBTAG00000017475	8.20E-04	1.00E+00	-1.44E-01	9.07E-01	-3.96E-01	2.64E-01	-4.44E-01	2.05E-01	1.40E-01	1.00E+00
ENSBTAG00000047223	0.00E+00	1.00E+00	0.00E+00	1.00E+00	-5.13E-15	1.00E+00	0.00E+00	1.00E+00	0.00E+00	1.00E+00
ENSBTAG00000037319	-3.89E+00	1.42E-01	-3.89E+00	3.54E-02	-3.89E+00	2.21E-02	-2.13E+00	1.75E-01	-1.98E+00	6.01E-01
ENSBTAG00000016724	-4.27E-01	5.76E-01	-1.27E+00	6.85E-06	-5.06E-01	1.16E-01	7.60E-02	1.00E+00	-3.43E-02	1.00E+00
ENSBTAG00000003872	3.04E-02	1.00E+00	3.02E-01	3.54E-01	5.45E-01	3.76E-02	1.59E-01	7.80E-01	3.56E-02	1.00E+00
ENSBTAG00000038838	0.00E+00	1.00E+00	0.00E+00	1.00E+00	-5.13E-15	1.00E+00	0.00E+00	1.00E+00	0.00E+00	1.00E+00
ENSBTAG00000020397	-1.29E+00	9.38E-01	7.77E-01	7.28E-01	-6.54E-01	8.97E-01	-1.44E+00	4.97E-01	-1.29E+00	9.02E-01
ENSBTAG00000017424	2.89E-03	1.00E+00	1.19E-02	1.00E+00	-5.75E-02	1.00E+00	7.18E-02	1.00E+00	-6.40E-02	1.00E+00
ENSBTAG00000018053	2.88E-01	7.51E-01	1.74E-01	6.81E-01	3.15E-01	2.68E-01	5.68E-01	1.96E-02	2.82E-01	7.71E-01
ENSBTAG00000013574	0.00E+00	1.00E+00	1.99E+00	4.43E-01	-5.13E-15	1.00E+00	3.68E+00	4.84E-02	0.00E+00	1.00E+00
ENSBTAG00000016926	0.00E+00	1.00E+00	2.79E+00	2.10E-01	1.78E+00	4.79E-01	0.00E+00	1.00E+00	0.00E+00	1.00E+00
ENSBTAG00000031524	3.99E-01	8.67E-01	-6.56E-01	1.77E-01	1.53E-02	1.00E+00	8.86E-01	4.70E-02	-2.87E-01	1.00E+00
ENSBTAG00000012406	-2.28E-01	1.00E+00	-5.29E-01	3.03E-01	-7.55E-01	9.57E-02	-1.46E+00	3.79E-04	3.44E-01	9.20E-01
ENSBTAG00000001936	-1.63E-01	1.00E+00	-3.80E+00	1.70E-32	-3.84E+00	2.88E-33	-3.59E+00	1.13E-29	1.20E-01	1.00E+00
ENSBTAG00000013079	-1.17E+00	8.71E-01	-1.10E+00	5.46E-01	-1.33E+00	4.13E-01	4.40E-01	9.28E-01	8.24E-01	8.85E-01
ENSBTAG00000008105	-4.41E-01	7.84E-01	3.07E-01	5.80E-01	1.87E+00	1.63E-09	1.78E+00	1.51E-08	-2.75E-01	9.95E-01
ENSBTAG00000010934	-8.61E-02	1.00E+00	1.09E+00	6.56E-09	1.28E+00	3.23E-12	8.68E-01	5.70E-06	-1.64E-01	9.42E-01
ENSBTAG00000044210	-2.70E+00	5.77E-01	6.02E-01	9.45E-01	-9.22E-01	8.09E-01	1.93E+00	1.17E-01	-7.71E-01	1.00E+00
ENSBTAG00000047773	0.00E+00	1.00E+00	0.00E+00	1.00E+00	-5.13E-15	1.00E+00	0.00E+00	1.00E+00	0.00E+00	1.00E+00
ENSBTAG00000015362	3.87E-01	1.00E+00	-1.35E+00	3.98E-01	-4.34E-01	9.57E-01	1.22E+00	2.05E-01	3.95E-01	1.00E+00
ENSBTAG00000044456	0.00E+00	1.00E+00	0.00E+00	1.00E+00	-5.13E-15	1.00E+00	0.00E+00	1.00E+00	0.00E+00	1.00E+00
ENSBTAG00000037492	0.00E+00	1.00E+00	0.00E+00	1.00E+00	-5.13E-15	1.00E+00	0.00E+00	1.00E+00	0.00E+00	1.00E+00
ENSBTAG00000002065	2.70E+00	5.76E-01	3.68E+00	5.08E-02	-5.13E-15	1.00E+00	3.70E+00	4.67E-02	3.22E+00	4.34E-01
ENSBTAG00000024608	-5.34E-02	1.00E+00	-1.39E-02	1.00E+00	-1.40E-01	8.49E-01	1.49E-01	8.26E-01	-5.95E-01	1.84E-01
ENSBTAG00000031462	-3.78E-01	1.00E+00	8.38E-01	5.95E-01	1.43E+00	1.64E-01	5.58E-01	8.49E-01	-1.44E-03	1.00E+00
ENSBTAG00000027390	-5.16E-02	1.00E+00	2.77E-01	2.69E-01	4.06E-01	6.44E-02	1.58E-01	6.57E-01	-1.32E-01	1.00E+00
ENSBTAG00000006243	0.00E+00	1.00E+00	0.00E+00	1.00E+00	-5.13E-15	1.00E+00	0.00E+00	1.00E+00	0.00E+00	1.00E+00
ENSBTAG00000014555	1.10E+00	5.02E-01	2.40E-01	1.00E+00	2.01E-01	1.00E+00	1.18E+00	1.32E-01	2.64E-01	1.00E+00
ENSBTAG00000037205	0.00E+00	1.00E+00	0.00E+00	1.00E+00	-5.13E-15	1.00E+00	0.00E+00	1.00E+00	0.00E+00	1.00E+00
ENSBTAG00000006639	-2.60E-01	8.23E-01	5.93E-01	1.29E-02	3.28E-01	2.44E-01	1.84E-01	6.46E-01	1.29E-01	1.00E+00
ENSBTAG00000013009	6.05E-02	1.00E+00	-1.07E+00	7.54E-03	-7.02E-01	9.21E-02	-3.31E-01	5.57E-01	6.59E-01	4.03E-01

ENSBTAG00000013007	1.01E-02	1.00E+00	-7.31E-01	5.52E-04	-1.70E+00	4.69E-17	-2.21E+00	3.43E-27	-4.45E-01	2.98E-01
ENSBTAG00000004712	1.92E+00	8.23E-01	0.00E+00	1.00E+00	2.55E+00	2.54E-01	0.00E+00	1.00E+00	1.91E+00	8.00E-01
ENSBTAG00000033397	0.00E+00	1.00E+00	0.00E+00	1.00E+00	-5.13E-15	1.00E+00	1.76E+00	4.97E-01	0.00E+00	1.00E+00
ENSBTAG00000006539	5.68E-01	9.42E-01	-2.30E+00	3.21E-02	3.15E-01	9.22E-01	1.23E+00	5.92E-02	1.44E+00	1.49E-01
ENSBTAG00000006533	-6.32E-02	1.00E+00	-9.38E-02	9.81E-01	7.23E-02	1.00E+00	6.13E-02	1.00E+00	-4.19E-01	4.12E-01
ENSBTAG00000008303	-5.77E-02	1.00E+00	-4.02E-01	1.17E-01	-3.69E-01	1.61E-01	-1.32E-01	8.32E-01	-2.52E-01	8.00E-01
ENSBTAG00000021321	5.13E-01	1.00E+00	-1.17E+00	4.69E-01	-2.21E+00	9.58E-02	-7.61E-01	7.56E-01	8.70E-01	9.78E-01
ENSBTAG00000031278	9.17E-01	6.21E-01	1.83E+00	5.77E-03	2.66E+00	2.55E-05	1.56E+00	2.49E-02	1.99E+00	3.26E-02
ENSBTAG00000001376	8.06E-01	1.65E-01	1.54E+00	1.49E-05	2.30E+00	7.08E-11	2.23E+00	3.60E-10	1.33E+00	5.47E-03
ENSBTAG00000018418	8.10E-01	1.00E+00	5.75E+00	1.21E-14	7.93E+00	8.07E-41	7.83E+00	3.94E-39	1.70E+00	7.69E-01
ENSBTAG00000018417	-1.14E-01	1.00E+00	2.06E-01	8.51E-01	8.96E-01	1.88E-02	8.41E-01	3.35E-02	-3.65E-01	8.24E-01
ENSBTAG00000021885	-7.92E-01	1.00E+00	7.55E-02	1.00E+00	-9.30E-01	8.06E-01	-2.71E+00	2.05E-01	-7.98E-01	1.00E+00
ENSBTAG00000021811	-2.39E-01	1.00E+00	-8.48E-01	2.05E-01	-6.70E-01	3.18E-01	-2.57E-01	8.94E-01	-1.64E-01	1.00E+00
ENSBTAG00000008638	-1.96E-01	1.00E+00	-6.01E-01	1.61E-01	-2.64E-01	6.75E-01	-8.85E-01	2.43E-02	-3.29E-01	8.85E-01
ENSBTAG00000003977	-1.30E+00	2.01E-01	-2.55E+00	4.21E-04	-1.49E+00	1.91E-02	-1.64E+00	1.10E-02	-1.20E-01	1.00E+00
ENSBTAG00000010913	-6.81E-01	9.74E-02	9.48E-01	4.16E-04	2.26E+00	1.58E-18	1.70E+00	5.13E-11	-4.62E-01	4.99E-01
ENSBTAG00000005750	-3.58E+00	2.33E-01	-7.94E-01	7.87E-01	-1.80E+00	2.98E-01	-3.58E+00	4.94E-02	2.72E-02	1.00E+00
ENSBTAG00000012341	1.10E-01	1.00E+00	6.63E-01	8.13E-04	8.31E-01	1.50E-05	7.52E-01	1.34E-04	1.32E-01	1.00E+00
ENSBTAG00000013330	-3.32E-02	1.00E+00	3.11E-01	1.88E-01	2.36E-01	3.71E-01	4.11E-01	6.16E-02	-5.07E-02	1.00E+00
ENSBTAG00000017002	-5.12E-02	1.00E+00	1.42E+00	3.74E-13	9.52E-01	1.64E-06	1.54E-01	6.89E-01	4.11E-02	1.00E+00
ENSBTAG00000017007	-5.57E-01	9.05E-01	2.93E+00	1.87E-06	1.16E+00	8.23E-02	-8.20E-02	1.00E+00	-4.14E-01	1.00E+00
ENSBTAG00000027387	-1.18E+00	8.67E-01	-1.91E+00	2.51E-01	-3.89E+00	2.21E-02	-2.13E+00	1.75E-01	-1.98E+00	6.01E-01
ENSBTAG00000027386	-1.84E+00	2.10E-03	-3.30E+00	3.38E-08	-2.11E+00	2.60E-05	-2.05E+00	4.66E-05	-4.43E-01	8.29E-01
ENSBTAG00000002116	-3.43E-01	5.76E-01	-2.01E-01	5.68E-01	1.77E-02	1.00E+00	1.40E-02	1.00E+00	-2.74E-01	7.78E-01
ENSBTAG00000002115	-9.52E-01	8.06E-01	-1.11E+00	3.05E-01	-1.12E+00	2.63E-01	-1.69E+00	8.97E-02	-3.92E-01	1.00E+00
ENSBTAG00000048288	0.00E+00	1.00E+00	0.00E+00	1.00E+00	1.78E+00	4.79E-01	0.00E+00	1.00E+00	0.00E+00	1.00E+00
ENSBTAG00000048009	0.00E+00	1.00E+00	0.00E+00	1.00E+00	-5.13E-15	1.00E+00	0.00E+00	1.00E+00	0.00E+00	1.00E+00
ENSBTAG00000003364	0.00E+00	1.00E+00	0.00E+00	1.00E+00	1.78E+00	4.79E-01	2.53E+00	2.71E-01	1.91E+00	8.00E-01
ENSBTAG00000027384	0.00E+00	1.00E+00	0.00E+00	1.00E+00	-5.13E-15	1.00E+00	1.77E+00	4.97E-01	0.00E+00	1.00E+00
ENSBTAG00000027383	-1.91E+00	8.23E-01	-1.91E+00	4.69E-01	-1.91E+00	4.28E-01	-1.91E+00	4.39E-01	-1.91E+00	8.00E-01
ENSBTAG00000020555	4.56E-03	1.00E+00	9.02E-02	1.00E+00	-1.48E-01	1.00E+00	6.93E-01	8.56E-01	-2.70E+00	6.01E-01
ENSBTAG00000031254	-8.62E-01	1.00E+00	-7.92E-01	7.89E-01	-3.58E+00	4.62E-02	-1.81E+00	3.05E-01	1.71E+00	3.47E-01
ENSBTAG00000014176	5.92E-01	7.85E-01	-4.74E+00	1.50E-10	-1.07E+00	6.46E-02	-9.10E-01	1.38E-01	2.25E-01	1.00E+00
ENSBTAG00000008840	4.60E-01	4.13E-01	1.65E+00	1.56E-10	1.74E+00	1.00E-11	1.71E+00	3.98E-11	2.24E-01	9.31E-01
ENSBTAG00000016169	-2.35E-01	1.00E+00	3.31E-01	6.74E-01	4.34E-02	1.00E+00	2.46E-01	8.54E-01	-9.62E-02	1.00E+00

ENSBTAG00000016171	-7.38E-01	1.00E+00	1.08E+00	2.97E-01	-6.04E-01	7.91E-01	4.22E-01	9.12E-01	-1.09E+00	8.15E-01
ENSBTAG00000006526	-7.03E-02	1.00E+00	1.02E-01	9.68E-01	-3.35E-01	2.53E-01	1.08E-01	9.49E-01	1.39E-01	1.00E+00
ENSBTAG00000018775	-3.23E-01	9.56E-01	-8.55E-01	3.78E-02	-1.59E+00	3.24E-05	-9.33E-02	1.00E+00	2.39E-02	1.00E+00
ENSBTAG00000014930	-1.16E-01	1.00E+00	8.51E-01	4.33E-01	-6.96E-01	6.34E-01	-4.28E-01	9.02E-01	-7.17E-01	9.58E-01
ENSBTAG00000009206	-2.87E-01	1.00E+00	8.35E-01	4.16E-01	9.79E-01	2.97E-01	-3.60E-01	9.22E-01	-5.42E-01	1.00E+00
ENSBTAG00000031214	-2.39E-01	1.00E+00	1.12E+00	3.27E-01	1.21E+00	2.73E-01	8.96E-02	1.00E+00	-4.49E-01	1.00E+00
ENSBTAG00000009005	1.96E-04	1.00E+00	-4.27E-01	1.17E-01	-1.26E+00	8.61E-08	-1.37E+00	7.18E-09	5.87E-02	1.00E+00
ENSBTAG00000005937	-1.10E-01	1.00E+00	3.31E-01	3.68E-01	-7.22E-01	1.50E-02	-1.79E+00	9.00E-10	3.44E-01	7.54E-01
ENSBTAG00000009743	-3.84E-01	4.02E-01	2.66E-01	3.60E-01	6.33E-01	4.27E-03	6.45E-01	4.17E-03	-1.76E-01	9.61E-01
ENSBTAG00000016348	2.42E-02	1.00E+00	9.57E-01	1.70E-01	1.03E-01	1.00E+00	-3.07E-01	9.29E-01	-1.14E+00	5.58E-01
ENSBTAG00000031453	6.80E-01	3.88E-01	1.18E+00	2.54E-03	7.22E-01	9.40E-02	7.44E-02	1.00E+00	-6.13E-03	1.00E+00
ENSBTAG00000007932	1.96E+00	1.10E-16	2.36E+00	6.89E-25	1.50E+00	5.87E-11	6.12E-01	1.69E-02	-6.52E-02	1.00E+00
ENSBTAG00000001015	4.55E-02	1.00E+00	2.99E-01	2.50E-01	3.21E-01	2.03E-01	3.17E-01	2.24E-01	5.63E-01	9.47E-02
ENSBTAG00000018334	2.15E-01	1.00E+00	1.13E+00	2.91E-03	1.23E+00	9.89E-04	8.70E-01	3.30E-02	3.14E-01	9.48E-01
ENSBTAG00000016231	-6.48E-02	1.00E+00	-8.73E-01	2.67E-04	-3.20E-01	2.90E-01	-1.20E-01	9.09E-01	5.28E-01	2.48E-01
ENSBTAG00000003215	-3.82E-01	5.18E-01	-2.34E+00	1.28E-22	-1.90E+00	3.82E-16	-8.43E-01	5.59E-04	3.15E-01	7.06E-01
ENSBTAG00000031402	7.15E-01	2.05E-02	1.46E+00	2.09E-10	9.31E-01	7.06E-05	3.98E-01	1.60E-01	7.49E-01	2.78E-02
ENSBTAG00000008348	1.38E-01	1.00E+00	1.99E+00	3.39E-10	8.31E-01	1.81E-02	-2.22E-02	1.00E+00	4.06E-01	7.91E-01
ENSBTAG00000016427	-9.40E-02	1.00E+00	-4.28E-01	6.46E-02	-7.09E-01	7.58E-04	-8.13E-01	1.13E-04	3.29E-02	1.00E+00
ENSBTAG00000003901	-3.31E-02	1.00E+00	5.63E-02	1.00E+00	-5.20E-01	4.75E-02	-1.75E-01	7.12E-01	1.05E-01	1.00E+00
ENSBTAG00000003908	4.87E-02	1.00E+00	6.52E-01	1.94E-03	7.91E-01	1.06E-04	7.59E-01	2.67E-04	-1.06E-01	1.00E+00
ENSBTAG00000013056	0.00E+00	1.00E+00	3.98E+00	2.57E-02	3.43E+00	7.35E-02	3.03E+00	1.50E-01	0.00E+00	1.00E+00
ENSBTAG00000042086	0.00E+00	1.00E+00	0.00E+00	1.00E+00	-5.13E-15	1.00E+00	0.00E+00	1.00E+00	0.00E+00	1.00E+00
ENSBTAG00000019200	-1.91E+00	8.23E-01	1.39E+00	5.16E-01	6.44E-01	9.78E-01	-1.47E-01	1.00E+00	-1.91E+00	8.00E-01
ENSBTAG00000010112	-1.91E+00	8.23E-01	7.58E-02	1.00E+00	-1.30E-01	1.00E+00	6.28E-01	9.94E-01	7.05E-03	1.00E+00
ENSBTAG00000038687	-1.91E+00	8.23E-01	2.88E+00	2.21E-02	-1.91E+00	4.28E-01	-1.91E+00	4.39E-01	6.51E-03	1.00E+00
ENSBTAG00000038412	-4.17E-04	1.00E+00	-2.92E-01	1.00E+00	7.54E-01	6.48E-01	-2.01E-01	1.00E+00	2.99E-01	1.00E+00
ENSBTAG00000038453	0.00E+00	1.00E+00	1.99E+00	4.43E-01	1.78E+00	4.79E-01	4.32E+00	9.50E-03	1.91E+00	8.00E-01
ENSBTAG00000009144	4.84E-03	1.00E+00	-1.91E+00	4.69E-01	-1.91E+00	4.28E-01	2.23E+00	1.34E-01	1.30E+00	9.00E-01
ENSBTAG00000031376	-3.21E+00	3.75E-01	9.99E-02	1.00E+00	-1.42E+00	4.82E-01	-1.45E+00	4.97E-01	1.17E+00	8.00E-01
ENSBTAG00000031375	0.00E+00	1.00E+00	0.00E+00	1.00E+00	1.78E+00	4.79E-01	2.53E+00	2.71E-01	1.91E+00	8.00E-01
ENSBTAG00000011704	2.71E-01	1.00E+00	5.31E-01	2.55E-01	1.56E-01	9.81E-01	4.45E-01	3.94E-01	-1.66E-01	1.00E+00
ENSBTAG00000019752	-1.91E+00	8.23E-01	1.39E+00	5.17E-01	-1.25E-01	1.00E+00	-1.91E+00	4.39E-01	-1.91E+00	8.00E-01
ENSBTAG00000031373	-1.91E+00	8.23E-01	7.58E-02	1.00E+00	-1.91E+00	4.28E-01	6.21E-01	9.94E-01	1.85E-02	1.00E+00
ENSBTAG00000006802	4.54E+00	2.75E-02	0.00E+00	1.00E+00	1.78E+00	4.79E-01	2.53E+00	2.71E-01	1.91E+00	8.00E-01

ENSBTAG00000017098	2.70E+00	5.76E-01	1.99E+00	4.43E-01	2.56E+00	2.54E-01	3.40E+00	8.35E-02	1.91E+00	8.00E-01
ENSBTAG00000015724	1.64E+00	3.45E-01	1.34E+00	2.40E-01	1.32E+00	2.25E-01	1.86E+00	4.35E-02	-8.89E-01	1.00E+00
ENSBTAG00000031361	6.25E-01	1.00E+00	1.84E+00	1.55E-01	1.57E+00	2.51E-01	-1.74E-01	1.00E+00	1.54E+00	6.61E-01
ENSBTAG00000031354	1.53E+00	3.78E-01	1.66E+00	8.34E-02	2.32E+00	1.08E-02	2.32E+00	1.22E-02	3.41E+00	4.09E-03
ENSBTAG00000018535	1.19E-01	1.00E+00	5.89E-01	1.92E-02	1.05E+00	5.00E-06	7.32E-01	2.81E-03	-1.42E-01	1.00E+00
ENSBTAG00000000512	5.01E-01	7.67E-01	1.44E+00	1.68E-04	1.62E+00	1.17E-05	1.81E+00	7.86E-07	4.02E-01	8.43E-01
ENSBTAG00000014869	1.31E+00	5.53E-01	1.02E+00	4.03E-01	6.80E-02	1.00E+00	3.27E-02	1.00E+00	-1.19E+00	8.34E-01
ENSBTAG00000010131	1.25E+00	1.74E-02	2.01E+00	6.04E-07	1.78E+00	1.02E-05	1.97E+00	1.27E-06	9.11E-01	2.42E-01
ENSBTAG00000003946	-2.23E-01	1.00E+00	6.62E-01	3.19E-01	-1.51E+00	5.75E-03	-2.20E+00	5.73E-05	-1.44E-01	1.00E+00
ENSBTAG00000003962	0.00E+00	1.00E+00	0.00E+00	1.00E+00	-5.13E-15	1.00E+00	0.00E+00	1.00E+00	0.00E+00	1.00E+00
ENSBTAG00000047885	0.00E+00	1.00E+00	0.00E+00	1.00E+00	-5.13E-15	1.00E+00	0.00E+00	1.00E+00	0.00E+00	1.00E+00
ENSBTAG00000003971	-4.93E-01	6.21E-01	-2.16E+00	4.71E-08	-2.44E+00	3.36E-10	-2.34E+00	1.67E-09	-1.46E-01	1.00E+00
ENSBTAG00000038738	-2.06E-01	1.00E+00	-1.78E-01	1.00E+00	-7.08E-01	2.05E-01	-1.42E-01	1.00E+00	1.29E-01	1.00E+00
ENSBTAG00000039313	-1.05E-01	1.00E+00	2.91E-01	3.81E-01	9.62E-01	4.59E-05	8.87E-01	2.44E-04	1.34E-01	1.00E+00
ENSBTAG00000013387	-1.28E-01	1.00E+00	1.65E-01	6.43E-01	6.31E-01	2.35E-03	6.97E-01	8.28E-04	-1.04E-01	1.00E+00
ENSBTAG00000016524	-4.97E-02	1.00E+00	3.79E-01	8.02E-02	1.46E-01	6.75E-01	-4.70E-02	1.00E+00	-1.48E-02	1.00E+00
ENSBTAG00000005969	3.04E-01	5.14E-01	2.16E+00	3.08E-32	2.08E+00	1.80E-30	1.48E+00	3.02E-16	-4.47E-01	2.00E-01
ENSBTAG00000042501	0.00E+00	1.00E+00	0.00E+00	1.00E+00	-5.13E-15	1.00E+00	0.00E+00	1.00E+00	0.00E+00	1.00E+00
ENSBTAG00000034077	-5.35E-01	8.87E-01	-5.60E-01	4.95E-01	-3.29E-01	8.04E-01	-9.47E-01	1.66E-01	1.08E+00	2.28E-01
ENSBTAG00000018101	-3.98E-02	1.00E+00	-4.20E-01	3.92E-02	-1.25E+00	3.89E-12	-1.46E+00	6.12E-16	-1.50E-01	9.66E-01
ENSBTAG00000000308	-1.46E-01	1.00E+00	-1.07E+00	1.35E-07	-1.07E+00	1.03E-07	-8.84E-01	1.78E-05	-4.03E-01	3.68E-01
ENSBTAG00000046623	0.00E+00	1.00E+00	0.00E+00	1.00E+00	-5.13E-15	1.00E+00	0.00E+00	1.00E+00	0.00E+00	1.00E+00
ENSBTAG00000006134	-9.86E-02	1.00E+00	-5.92E-01	7.52E-03	-7.01E-01	1.12E-03	-4.61E-01	5.17E-02	-1.72E-01	9.56E-01
ENSBTAG00000006135	-4.41E-01	6.54E-01	-7.29E-01	3.51E-02	-9.26E-01	5.11E-03	-1.08E+00	1.10E-03	-7.99E-01	1.49E-01
ENSBTAG00000011620	-2.20E-01	9.42E-01	-3.14E-01	3.42E-01	2.11E-01	5.91E-01	1.80E-01	7.07E-01	-2.77E-01	8.00E-01
ENSBTAG00000006725	0.00E+00	1.00E+00	0.00E+00	1.00E+00	-5.13E-15	1.00E+00	0.00E+00	1.00E+00	0.00E+00	1.00E+00
ENSBTAG00000005504	1.45E+00	4.26E-05	5.86E-01	1.30E-01	1.42E+00	6.53E-06	1.39E+00	1.44E-05	5.74E-01	4.83E-01
ENSBTAG00000007930	-3.89E-02	1.00E+00	-4.37E-01	3.62E-02	-1.99E-01	4.72E-01	-2.14E-01	4.39E-01	-1.95E-01	8.27E-01
ENSBTAG00000013301	3.47E-01	1.00E+00	4.95E-03	1.00E+00	-8.47E-01	1.24E-01	-2.79E-01	8.06E-01	-1.18E-01	1.00E+00
ENSBTAG00000013303	-8.45E-01	3.75E-01	-2.01E+00	2.17E-05	-4.13E+00	7.88E-17	-3.57E+00	2.56E-13	-1.04E+00	2.44E-01
ENSBTAG00000003504	-6.84E-02	1.00E+00	-4.96E-01	1.39E-01	-4.47E-01	1.99E-01	-1.72E-01	8.25E-01	-1.81E-01	1.00E+00
ENSBTAG00000003512	-1.26E-01	1.00E+00	-1.21E+00	6.05E-02	-2.61E+00	4.88E-05	-1.48E+00	1.79E-02	4.87E-01	8.81E-01
ENSBTAG00000029949	0.00E+00	1.00E+00	0.00E+00	1.00E+00	-5.13E-15	1.00E+00	0.00E+00	1.00E+00	0.00E+00	1.00E+00
ENSBTAG00000002236	2.96E-01	6.86E-01	1.05E+00	1.13E-06	5.38E-01	2.18E-02	4.67E-01	5.96E-02	5.18E-01	1.99E-01
ENSBTAG00000003815	4.44E-01	3.08E-01	7.70E-01	8.54E-04	5.14E-01	3.76E-02	6.20E-01	1.06E-02	3.34E-01	6.11E-01

ENSBTAG00000008291	7.82E-01	3.72E-01	2.44E+00	8.51E-09	2.29E+00	5.66E-08	2.56E+00	1.79E-09	-3.66E-01	9.35E-01
ENSBTAG00000010024	5.86E-01	1.00E+00	3.06E+00	3.22E-06	2.46E+00	2.57E-04	2.39E+00	5.15E-04	1.73E-01	1.00E+00
ENSBTAG00000011263	2.55E-01	8.23E-01	9.38E-01	1.98E-05	7.24E-01	1.31E-03	6.97E-01	2.50E-03	-2.47E-01	8.00E-01
ENSBTAG00000006278	1.54E+00	5.91E-01	3.82E+00	7.31E-06	2.14E+00	4.34E-02	1.28E+00	3.81E-01	2.22E+00	2.28E-01
ENSBTAG00000030990	-1.75E-02	1.00E+00	9.54E-02	9.68E-01	2.26E-01	4.73E-01	4.44E-01	7.30E-02	9.07E-03	1.00E+00
ENSBTAG00000004429	2.72E+00	5.76E-01	0.00E+00	1.00E+00	1.78E+00	4.79E-01	1.77E+00	4.97E-01	0.00E+00	1.00E+00
ENSBTAG00000006021	-2.60E-01	8.23E-01	-2.00E+00	2.25E-17	-2.30E+00	2.31E-22	-1.54E+00	4.07E-11	3.09E-01	7.14E-01
ENSBTAG00000030976	0.00E+00	1.00E+00	1.98E+00	4.43E-01	1.78E+00	4.79E-01	0.00E+00	1.00E+00	0.00E+00	1.00E+00
ENSBTAG00000006670	-2.90E-02	1.00E+00	-1.24E-01	7.55E-01	-4.08E-01	4.08E-02	-6.41E-01	6.63E-04	1.15E-01	1.00E+00
ENSBTAG00000006949	-1.25E-01	1.00E+00	-4.48E-01	8.93E-01	-1.62E+00	1.57E-01	-7.33E-01	6.05E-01	1.09E+00	5.69E-01
ENSBTAG00000006955	1.47E-01	1.00E+00	4.33E-01	5.63E-02	8.64E-02	9.79E-01	-2.58E-01	3.58E-01	2.68E-01	7.27E-01
ENSBTAG00000044015	1.92E+00	8.23E-01	0.00E+00	1.00E+00	-5.13E-15	1.00E+00	1.77E+00	4.97E-01	1.91E+00	8.00E-01
ENSBTAG00000006962	-2.22E-01	8.23E-01	-5.47E-01	9.98E-03	-6.94E-01	6.78E-04	-7.70E-01	1.75E-04	1.05E-01	1.00E+00
ENSBTAG00000027361	5.54E-02	1.00E+00	-8.34E-02	1.00E+00	-4.36E-01	1.89E-01	-4.07E-01	2.43E-01	1.88E-01	1.00E+00
ENSBTAG00000003949	-3.70E-02	1.00E+00	5.18E-01	8.38E-03	3.99E-01	5.40E-02	2.31E-02	1.00E+00	-1.52E-01	9.62E-01
ENSBTAG00000005571	4.20E-01	3.82E-01	9.81E-01	2.18E-05	3.89E-03	1.00E+00	6.47E-01	8.92E-03	1.64E-01	1.00E+00
ENSBTAG00000028669	0.00E+00	1.00E+00	0.00E+00	1.00E+00	-5.13E-15	1.00E+00	0.00E+00	1.00E+00	0.00E+00	1.00E+00
ENSBTAG00000005573	2.06E-01	1.00E+00	1.21E+00	4.58E-06	1.09E+00	3.64E-05	5.99E-01	4.36E-02	-4.52E-02	1.00E+00
ENSBTAG00000002822	-5.64E-01	8.05E-01	4.23E-01	5.13E-01	3.44E-01	6.42E-01	-9.88E-01	7.58E-02	-5.78E-01	7.82E-01
ENSBTAG00000001640	2.75E-02	1.00E+00	-7.29E-01	4.83E-02	-7.08E-01	5.08E-02	-6.14E-02	1.00E+00	6.01E-01	3.96E-01
ENSBTAG00000001645	-2.17E-01	9.19E-01	4.79E-01	6.17E-02	1.00E+00	9.01E-06	8.39E-01	3.32E-04	-3.00E-01	7.44E-01
ENSBTAG00000001741	-1.09E-01	1.00E+00	-4.82E-01	8.12E-02	1.82E-01	6.87E-01	1.33E-02	1.00E+00	-3.03E-01	7.71E-01
ENSBTAG00000011473	-4.61E-01	4.55E-01	-9.37E-01	7.98E-04	-1.46E+00	6.24E-08	-1.22E+00	1.01E-05	-4.70E-01	4.87E-01
ENSBTAG00000005286	1.18E+00	6.53E-04	9.69E-01	1.79E-03	4.82E-01	1.92E-01	3.64E-01	4.00E-01	2.75E-01	9.07E-01
ENSBTAG00000021805	4.71E-01	3.75E-01	1.38E+00	5.88E-08	1.41E+00	2.65E-08	1.15E+00	9.34E-06	3.50E-01	6.85E-01
ENSBTAG00000040607	5.87E-01	5.76E-01	2.16E+00	3.19E-08	5.63E-01	2.51E-01	-5.28E-01	3.12E-01	4.08E-01	8.08E-01
ENSBTAG00000019621	-1.23E-02	1.00E+00	4.17E-01	1.15E-01	9.45E-01	3.18E-05	1.13E-01	9.22E-01	-1.20E-01	1.00E+00
ENSBTAG00000022005	0.00E+00	1.00E+00	0.00E+00	1.00E+00	-5.13E-15	1.00E+00	0.00E+00	1.00E+00	0.00E+00	1.00E+00
ENSBTAG00000022007	0.00E+00	1.00E+00	0.00E+00	1.00E+00	-5.13E-15	1.00E+00	0.00E+00	1.00E+00	0.00E+00	1.00E+00
ENSBTAG00000046344	0.00E+00	1.00E+00	0.00E+00	1.00E+00	-5.13E-15	1.00E+00	0.00E+00	1.00E+00	0.00E+00	1.00E+00
ENSBTAG00000011541	-7.28E-02	1.00E+00	-6.82E-01	1.78E-02	-2.48E-01	5.38E-01	-2.50E-01	5.46E-01	-1.14E-01	1.00E+00
ENSBTAG00000013915	0.00E+00	1.00E+00	0.00E+00	1.00E+00	-5.13E-15	1.00E+00	0.00E+00	1.00E+00	0.00E+00	1.00E+00
ENSBTAG00000021669	0.00E+00	1.00E+00	0.00E+00	1.00E+00	-5.13E-15	1.00E+00	0.00E+00	1.00E+00	0.00E+00	1.00E+00
ENSBTAG00000013737	-2.31E-01	1.00E+00	-1.43E-01	1.00E+00	-6.95E-01	7.29E-01	-2.36E+00	1.05E-01	2.57E-01	1.00E+00
ENSBTAG00000014648	2.36E-01	8.29E-01	7.18E-01	1.91E-03	7.13E-01	2.03E-03	8.49E-01	2.18E-04	1.28E+00	3.45E-07

ENSBTAG00000042176	0.00E+00	1.00E+00	0.00E+00	1.00E+00	-5.13E-15	1.00E+00	0.00E+00	1.00E+00	0.00E+00	1.00E+00
ENSBTAG00000012710	-1.29E+00	9.38E-01	1.23E+00	3.81E-01	9.66E-01	5.29E-01	-1.76E-01	1.00E+00	-5.00E-01	1.00E+00
ENSBTAG00000022562	1.37E+00	1.20E-01	1.12E+00	8.83E-02	1.92E-01	1.00E+00	1.09E+00	9.78E-02	-4.54E-01	1.00E+00
ENSBTAG00000000528	-1.46E-01	1.00E+00	1.21E-01	8.02E-01	2.78E-01	2.46E-01	2.74E-01	2.70E-01	-3.85E-02	1.00E+00
ENSBTAG00000008938	5.15E-01	7.42E-01	5.87E-01	2.42E-01	1.31E-01	1.00E+00	6.86E-01	1.48E-01	1.76E-01	1.00E+00
ENSBTAG00000045928	-4.19E-01	9.82E-01	-2.94E-01	8.46E-01	-1.91E-01	1.00E+00	-1.13E-01	1.00E+00	3.08E-01	1.00E+00
ENSBTAG00000003209	1.13E-01	1.00E+00	2.51E-03	1.00E+00	8.31E-02	1.00E+00	2.81E-01	4.97E-01	6.33E-01	2.16E-01
ENSBTAG00000003212	0.00E+00	1.00E+00	0.00E+00	1.00E+00	-5.13E-15	1.00E+00	0.00E+00	1.00E+00	0.00E+00	1.00E+00
ENSBTAG00000007658	-2.12E-01	8.27E-01	-5.53E-01	9.38E-03	-9.02E-01	6.62E-06	-1.18E+00	2.85E-09	4.86E-02	1.00E+00
ENSBTAG00000004750	-2.70E-01	1.00E+00	-4.29E-01	6.62E-01	-1.08E+00	9.65E-02	-1.02E+00	1.26E-01	-2.49E-01	1.00E+00
ENSBTAG00000004100	-1.23E-02	1.00E+00	-3.46E-01	2.22E-01	4.67E-01	6.60E-02	2.40E-01	4.81E-01	7.51E-02	1.00E+00
ENSBTAG00000005086	1.52E-01	1.00E+00	8.41E-01	5.70E-05	9.03E-01	1.25E-05	6.73E-01	1.98E-03	5.92E-02	1.00E+00
ENSBTAG00000016208	6.69E-01	1.00E+00	1.67E+00	1.03E-01	1.66E+00	1.05E-01	1.70E+00	1.04E-01	1.33E-01	1.00E+00
ENSBTAG00000042527	0.00E+00	1.00E+00	0.00E+00	1.00E+00	-5.13E-15	1.00E+00	0.00E+00	1.00E+00	0.00E+00	1.00E+00
ENSBTAG00000019378	1.65E+00	5.70E-02	3.03E+00	7.87E-09	2.25E+00	6.60E-05	7.53E-01	4.36E-01	1.23E+00	3.96E-01
ENSBTAG00000014046	-3.31E-01	1.00E+00	1.37E+00	6.82E-03	1.02E+00	6.04E-02	8.10E-02	1.00E+00	-5.83E-01	8.00E-01
ENSBTAG00000016864	3.69E+00	1.37E-21	4.73E+00	3.36E-33	5.25E+00	5.09E-39	5.33E+00	9.35E-40	2.56E+00	1.21E-10
ENSBTAG00000047302	8.02E-01	1.00E+00	1.77E+00	3.26E-01	1.51E+00	4.29E-01	1.79E+00	3.01E-01	2.22E+00	4.75E-01
ENSBTAG00000042744	1.77E+00	1.49E-01	2.38E+00	1.10E-03	2.46E+00	4.31E-04	1.11E+00	3.00E-01	-6.76E-01	1.00E+00
ENSBTAG00000042652	2.80E+00	1.38E-01	3.16E+00	8.22E-03	4.01E+00	5.55E-05	1.50E+00	4.53E-01	7.99E-01	1.00E+00
ENSBTAG00000043412	4.34E+00	5.06E-02	5.19E+00	3.23E-04	6.02E+00	9.79E-07	3.69E+00	4.80E-02	2.71E+00	6.01E-01
ENSBTAG00000042678	-3.11E-01	1.00E+00	4.41E-01	6.55E-01	3.42E-01	7.96E-01	-8.37E-01	3.16E-01	-6.34E-01	8.34E-01
ENSBTAG00000042322	2.55E-01	1.00E+00	4.65E-01	7.85E-01	8.31E-02	1.00E+00	-9.02E-01	4.55E-01	-1.45E-01	1.00E+00
ENSBTAG00000003040	1.40E-01	1.00E+00	1.02E+00	9.16E-08	1.08E+00	1.31E-08	8.05E-01	4.06E-05	-1.72E-01	9.23E-01
ENSBTAG00000031188	7.96E-01	1.00E+00	2.53E+00	6.74E-02	1.14E+00	6.49E-01	1.13E+00	6.68E-01	1.32E+00	8.97E-01
ENSBTAG00000047894	9.80E-03	1.00E+00	7.94E-02	1.00E+00	3.49E-01	1.00E+00	2.28E+00	4.11E-02	-2.71E+00	6.01E-01
ENSBTAG00000048262	-3.64E-02	1.00E+00	1.12E-01	9.45E-01	7.73E-01	1.22E-03	5.31E-01	4.39E-02	1.08E-01	1.00E+00
ENSBTAG00000000653	3.01E-01	7.57E-01	-2.39E-02	1.00E+00	-2.57E-01	4.59E-01	-4.77E-01	9.11E-02	-5.72E-01	2.13E-01
ENSBTAG00000000660	-3.52E-01	1.00E+00	1.43E+00	4.85E-02	1.38E-01	1.00E+00	-6.41E-01	5.71E-01	-9.62E-01	7.23E-01
ENSBTAG00000044690	0.00E+00	1.00E+00	0.00E+00	1.00E+00	-5.13E-15	1.00E+00	0.00E+00	1.00E+00	0.00E+00	1.00E+00
ENSBTAG00000004039	-1.09E-01	1.00E+00	8.28E-02	1.00E+00	7.45E-01	9.07E-03	3.70E-01	3.03E-01	-8.69E-02	1.00E+00
ENSBTAG00000045562	0.00E+00	1.00E+00	0.00E+00	1.00E+00	-5.13E-15	1.00E+00	0.00E+00	1.00E+00	0.00E+00	1.00E+00
ENSBTAG00000046646	0.00E+00	1.00E+00	0.00E+00	1.00E+00	-5.13E-15	1.00E+00	0.00E+00	1.00E+00	0.00E+00	1.00E+00
ENSBTAG00000042334	0.00E+00	1.00E+00	0.00E+00	1.00E+00	-5.13E-15	1.00E+00	0.00E+00	1.00E+00	0.00E+00	1.00E+00
ENSBTAG00000039199	0.00E+00	1.00E+00	0.00E+00	1.00E+00	-5.13E-15	1.00E+00	0.00E+00	1.00E+00	1.93E+00	8.00E-01

ENSBTAG00000003396	-2.14E+00	1.36E-11	-2.40E+00	3.50E-15	-2.89E+00	4.95E-21	-2.57E+00	3.20E-17	3.84E-01	7.45E-01
ENSBTAG00000007960	1.71E+00	2.03E-06	3.73E+00	3.92E-27	3.25E+00	7.52E-22	2.53E+00	3.43E-14	-1.81E-02	1.00E+00
ENSBTAG00000043116	0.00E+00	1.00E+00	0.00E+00	1.00E+00	-5.13E-15	1.00E+00	0.00E+00	1.00E+00	0.00E+00	1.00E+00
ENSBTAG00000042521	0.00E+00	1.00E+00	0.00E+00	1.00E+00	-5.13E-15	1.00E+00	0.00E+00	1.00E+00	0.00E+00	1.00E+00
ENSBTAG00000043708	4.24E+00	6.48E-05	5.12E+00	6.84E-10	3.98E+00	4.14E-05	3.21E+00	5.08E-03	8.20E-01	1.00E+00
ENSBTAG00000046785	1.92E+00	8.23E-01	2.79E+00	2.10E-01	1.78E+00	4.79E-01	2.53E+00	2.71E-01	1.91E+00	8.00E-01
ENSBTAG00000017584	-1.12E-01	1.00E+00	1.52E-01	8.65E-01	-2.34E-01	6.02E-01	1.04E-01	1.00E+00	-8.09E-02	1.00E+00
ENSBTAG00000017594	1.39E+00	3.21E-04	3.13E+00	2.69E-20	1.70E+00	3.01E-07	1.85E+00	3.68E-08	9.74E-01	6.35E-02
ENSBTAG00000045671	1.32E+00	9.28E-01	2.07E+00	1.95E-01	1.15E+00	6.41E-01	6.23E-01	9.94E-01	1.32E+00	8.97E-01
ENSBTAG00000009165	-3.88E-02	1.00E+00	-1.05E+00	4.81E-04	-1.71E+00	4.32E-09	-1.03E+00	6.66E-04	3.35E-01	8.00E-01
ENSBTAG00000016279	-1.67E-01	1.00E+00	-2.54E+00	6.09E-02	-5.56E-01	7.83E-01	-1.99E+00	1.07E-01	-1.70E-01	1.00E+00
ENSBTAG00000005733	3.35E-01	6.21E-01	-4.07E-01	1.53E-01	-2.76E-01	4.06E-01	1.57E-01	7.69E-01	5.21E-01	2.52E-01
ENSBTAG00000030090	0.00E+00	1.00E+00	0.00E+00	1.00E+00	-5.13E-15	1.00E+00	0.00E+00	1.00E+00	0.00E+00	1.00E+00
ENSBTAG00000048055	0.00E+00	1.00E+00	0.00E+00	1.00E+00	-5.13E-15	1.00E+00	0.00E+00	1.00E+00	0.00E+00	1.00E+00
ENSBTAG00000046673	0.00E+00	1.00E+00	0.00E+00	1.00E+00	-5.13E-15	1.00E+00	0.00E+00	1.00E+00	0.00E+00	1.00E+00
ENSBTAG00000000309	-9.07E-01	7.44E-01	2.83E-01	9.66E-01	-8.41E-01	3.66E-01	-4.22E-02	1.00E+00	-6.30E-01	8.98E-01
ENSBTAG00000042629	0.00E+00	1.00E+00	0.00E+00	1.00E+00	-5.13E-15	1.00E+00	0.00E+00	1.00E+00	0.00E+00	1.00E+00
ENSBTAG00000045300	0.00E+00	1.00E+00	0.00E+00	1.00E+00	-5.13E-15	1.00E+00	0.00E+00	1.00E+00	0.00E+00	1.00E+00
ENSBTAG00000008527	2.52E-01	7.32E-01	4.70E-01	2.51E-02	8.08E-01	2.96E-05	1.33E-01	7.59E-01	-8.71E-02	1.00E+00
ENSBTAG00000047678	0.00E+00	1.00E+00	0.00E+00	1.00E+00	-5.13E-15	1.00E+00	0.00E+00	1.00E+00	0.00E+00	1.00E+00
ENSBTAG00000021033	7.63E-02	1.00E+00	-1.46E+00	2.96E-07	-2.67E+00	2.46E-20	-1.98E+00	4.08E-12	3.78E-01	6.98E-01
ENSBTAG00000019606	-2.88E-01	8.97E-01	2.70E-01	5.62E-01	-1.71E-01	8.43E-01	-8.28E-01	1.11E-02	6.76E-02	1.00E+00
ENSBTAG00000007783	-3.78E-01	6.87E-01	-8.59E-01	4.31E-03	-1.01E+00	5.09E-04	-1.54E+00	1.66E-07	9.90E-02	1.00E+00
ENSBTAG00000011233	0.00E+00	1.00E+00	0.00E+00	1.00E+00	-5.13E-15	1.00E+00	0.00E+00	1.00E+00	0.00E+00	1.00E+00
ENSBTAG00000016741	-3.94E-01	1.00E+00	-5.20E-01	7.50E-01	3.09E-01	9.79E-01	6.24E-01	6.12E-01	6.16E-01	9.39E-01
ENSBTAG00000023002	-6.87E-02	1.00E+00	2.41E+00	2.45E-05	7.94E-01	3.37E-01	5.10E-01	6.59E-01	-2.94E-02	1.00E+00
ENSBTAG00000016873	8.82E-01	4.36E-03	2.25E+00	1.31E-19	2.01E+00	3.18E-16	1.15E+00	5.70E-06	-9.64E-02	1.00E+00
ENSBTAG00000002593	5.48E-01	1.00E+00	4.49E-01	7.88E-01	-4.37E-01	8.37E-01	3.46E-02	1.00E+00	-4.31E-01	1.00E+00
ENSBTAG00000020030	-4.37E-01	8.34E-01	-1.48E+00	6.51E-04	-2.13E-01	9.01E-01	6.40E-01	2.24E-01	2.33E-02	1.00E+00
ENSBTAG00000013220	1.63E-01	1.00E+00	-3.78E-01	7.75E-01	-1.42E+00	2.98E-02	-6.61E-01	4.24E-01	1.14E+00	3.09E-01
ENSBTAG00000012116	-6.77E-01	1.66E-01	-1.59E+00	7.74E-08	-1.96E+00	3.13E-11	-1.23E-01	9.94E-01	6.74E-01	2.39E-01
ENSBTAG00000042599	-1.15E-01	1.00E+00	-7.53E-01	5.31E-01	-2.14E+00	3.12E-02	-1.79E+00	7.20E-02	2.17E-01	1.00E+00
ENSBTAG00000018873	1.46E+00	5.22E-09	3.13E+00	3.28E-40	3.01E+00	8.78E-38	3.03E+00	3.23E-38	3.04E-01	7.79E-01
ENSBTAG00000002187	1.34E-01	1.00E+00	2.97E-01	4.11E-01	4.90E-01	9.44E-02	5.30E-01	7.04E-02	-5.39E-02	1.00E+00
ENSBTAG00000005275	-1.19E-02	1.00E+00	-8.72E-01	6.80E-03	-2.13E-01	7.22E-01	6.19E-01	6.69E-02	-5.48E-01	4.45E-01

ENSBTAG00000042131	0.00E+00	1.00E+00	0.00E+00	1.00E+00	-5.13E-15	1.00E+00	0.00E+00	1.00E+00	0.00E+00	1.00E+00
ENSBTAG00000043264	0.00E+00	1.00E+00	0.00E+00	1.00E+00	-5.13E-15	1.00E+00	0.00E+00	1.00E+00	0.00E+00	1.00E+00
ENSBTAG00000005280	6.00E-01	2.32E-01	6.06E-01	5.56E-02	1.04E+00	3.02E-04	4.70E-01	1.78E-01	-5.26E-01	4.34E-01
ENSBTAG00000044688	0.00E+00	1.00E+00	0.00E+00	1.00E+00	-5.13E-15	1.00E+00	0.00E+00	1.00E+00	0.00E+00	1.00E+00
ENSBTAG00000006037	3.64E-01	1.00E+00	-5.91E-01	4.12E-01	-6.27E-01	3.42E-01	-5.27E-02	1.00E+00	2.56E-01	1.00E+00
ENSBTAG00000003380	5.17E-01	1.00E+00	-2.71E+00	2.42E-01	-2.71E+00	1.99E-01	-2.71E+00	2.05E-01	-2.71E+00	6.01E-01
ENSBTAG00000003382	-3.21E+00	3.75E-01	9.06E-02	1.00E+00	-1.43E+00	4.79E-01	-3.21E+00	9.91E-02	-3.21E+00	4.34E-01
ENSBTAG00000016846	-2.83E-03	1.00E+00	3.26E-01	1.45E-01	5.08E-01	1.13E-02	4.47E-01	3.33E-02	-2.26E-01	8.00E-01
ENSBTAG00000016849	1.46E-01	1.00E+00	3.24E+00	1.24E-08	1.17E+00	1.36E-01	1.35E+00	7.23E-02	6.01E-01	9.54E-01
ENSBTAG00000003256	5.83E-01	1.23E-01	2.36E+00	9.98E-25	9.75E-01	4.16E-05	7.21E-01	4.34E-03	-1.56E-01	1.00E+00
ENSBTAG00000003257	3.23E-02	1.00E+00	7.92E-01	8.02E-03	8.45E-01	3.93E-03	8.29E-01	5.73E-03	3.31E-02	1.00E+00
ENSBTAG00000008870	-6.54E-01	1.00E+00	-2.04E-01	1.00E+00	-1.69E-01	1.00E+00	-8.56E-01	6.62E-01	-1.17E+00	8.38E-01
ENSBTAG00000030624	1.92E+00	8.23E-01	1.98E+00	4.43E-01	1.78E+00	4.79E-01	3.39E+00	8.39E-02	0.00E+00	1.00E+00
ENSBTAG00000020813	-2.71E+00	5.76E-01	-2.71E+00	2.42E-01	-2.71E+00	1.99E-01	-2.71E+00	2.05E-01	-2.71E+00	6.01E-01
ENSBTAG00000046375	0.00E+00	1.00E+00	0.00E+00	1.00E+00	-5.13E-15	1.00E+00	1.76E+00	4.97E-01	0.00E+00	1.00E+00
ENSBTAG00000039446	1.83E-02	1.00E+00	-2.70E+00	2.42E-01	-9.16E-01	8.09E-01	-2.70E+00	2.05E-01	-2.70E+00	6.01E-01
ENSBTAG00000039028	-4.49E+00	1.32E-01	-1.20E+00	6.09E-01	-4.49E+00	2.08E-02	-2.74E+00	1.44E-01	1.51E-01	1.00E+00
ENSBTAG00000014329	-1.91E+00	8.23E-01	-1.91E+00	4.69E-01	-1.91E+00	4.28E-01	-1.91E+00	4.39E-01	-1.91E+00	8.00E-01
ENSBTAG00000004148	1.92E+00	8.23E-01	3.98E+00	2.76E-02	4.93E+00	1.00E-03	5.84E+00	8.75E-06	0.00E+00	1.00E+00
ENSBTAG00000037925	2.99E-03	1.00E+00	-4.33E-03	1.00E+00	-6.13E-01	2.15E-01	-4.69E-01	4.10E-01	-8.94E-01	2.83E-01
ENSBTAG00000011279	-7.80E-01	1.00E+00	8.74E-02	1.00E+00	-1.48E-01	1.00E+00	1.78E+00	1.62E-01	-7.86E-01	1.00E+00
ENSBTAG00000011283	6.27E-01	9.96E-01	3.82E+00	2.75E-07	1.94E+00	1.36E-02	1.21E+00	1.89E-01	-1.20E+00	6.88E-01
ENSBTAG00000015127	1.75E+00	6.35E-06	2.52E+00	1.31E-12	1.82E+00	2.52E-07	1.54E+00	1.89E-05	6.70E-01	4.10E-01
ENSBTAG00000005181	3.78E-01	3.85E-01	1.04E+00	3.99E-07	9.39E-01	4.77E-06	1.00E+00	1.15E-06	6.32E-02	1.00E+00
ENSBTAG00000005184	5.72E-01	8.50E-01	-1.58E-01	1.00E+00	7.20E-01	3.00E-01	7.49E-01	2.83E-01	8.35E-01	6.01E-01
ENSBTAG00000009818	-2.67E-01	1.00E+00	-9.93E-01	4.10E-01	6.71E-01	4.79E-01	-2.09E-01	1.00E+00	1.40E-01	1.00E+00
ENSBTAG00000005186	1.43E-01	1.00E+00	-1.94E-01	8.05E-01	-2.58E-02	1.00E+00	1.65E-01	8.70E-01	-2.85E-01	8.96E-01
ENSBTAG00000016122	8.28E-03	1.00E+00	-4.10E-02	1.00E+00	1.54E-01	6.55E-01	2.57E-01	3.25E-01	3.84E-01	3.71E-01
ENSBTAG00000016127	-4.29E-01	7.97E-01	2.73E-02	1.00E+00	-4.66E-02	1.00E+00	-9.48E-01	1.70E-02	-1.09E-01	1.00E+00
ENSBTAG00000037942	7.47E-02	1.00E+00	3.99E-01	1.51E-01	5.12E-01	4.62E-02	5.08E-01	5.38E-02	2.97E-01	7.62E-01
ENSBTAG00000046614	8.02E-01	1.00E+00	1.76E+00	3.30E-01	-1.32E-01	1.00E+00	-1.53E-01	1.00E+00	1.67E+00	7.79E-01
ENSBTAG00000014345	0.00E+00	1.00E+00	0.00E+00	1.00E+00	-5.13E-15	1.00E+00	0.00E+00	1.00E+00	0.00E+00	1.00E+00
ENSBTAG00000046253	0.00E+00	1.00E+00	0.00E+00	1.00E+00	-5.13E-15	1.00E+00	0.00E+00	1.00E+00	0.00E+00	1.00E+00
ENSBTAG00000039693	0.00E+00	1.00E+00	1.98E+00	4.43E-01	2.55E+00	2.55E-01	2.53E+00	2.71E-01	3.22E+00	4.34E-01
ENSBTAG00000018122	0.00E+00	1.00E+00	1.98E+00	4.43E-01	-5.13E-15	1.00E+00	1.76E+00	4.97E-01	0.00E+00	1.00E+00

ENSBTAG00000046704	0.00E+00	1.00E+00	0.00E+00	1.00E+00	-5.13E-15	1.00E+00	0.00E+00	1.00E+00	0.00E+00	1.00E+00
ENSBTAG00000038966	5.13E-03	1.00E+00	8.73E-01	8.53E-01	-1.91E+00	4.28E-01	-1.91E+00	4.39E-01	7.93E-01	1.00E+00
ENSBTAG00000013950	-1.91E+00	8.23E-01	-1.91E+00	4.69E-01	-1.91E+00	4.28E-01	-1.91E+00	4.39E-01	-1.91E+00	8.00E-01
ENSBTAG00000006096	2.70E+00	5.76E-01	1.98E+00	4.43E-01	-5.13E-15	1.00E+00	0.00E+00	1.00E+00	0.00E+00	1.00E+00
ENSBTAG00000017328	-4.57E-02	1.00E+00	-7.17E-02	1.00E+00	-5.03E-01	4.17E-01	-2.76E-02	1.00E+00	-2.94E-01	1.00E+00
ENSBTAG00000045630	-8.36E-01	8.48E-01	1.07E+00	1.56E-01	-1.02E+00	3.46E-01	-4.74E-01	8.03E-01	8.49E-01	7.23E-01
ENSBTAG00000026119	0.00E+00	1.00E+00	0.00E+00	1.00E+00	-5.13E-15	1.00E+00	0.00E+00	1.00E+00	0.00E+00	1.00E+00
ENSBTAG00000013928	1.25E-01	1.00E+00	-1.32E-01	1.00E+00	-8.69E-01	2.38E-02	-1.39E+00	1.75E-04	-2.95E-01	9.46E-01
ENSBTAG00000044400	0.00E+00	1.00E+00	0.00E+00	1.00E+00	-5.13E-15	1.00E+00	0.00E+00	1.00E+00	0.00E+00	1.00E+00
ENSBTAG00000045129	0.00E+00	1.00E+00	0.00E+00	1.00E+00	-5.13E-15	1.00E+00	0.00E+00	1.00E+00	0.00E+00	1.00E+00
ENSBTAG00000001495	1.92E+00	8.23E-01	0.00E+00	1.00E+00	-5.13E-15	1.00E+00	0.00E+00	1.00E+00	0.00E+00	1.00E+00
ENSBTAG00000039490	2.70E+00	5.76E-01	3.30E+00	1.02E-01	-5.13E-15	1.00E+00	0.00E+00	1.00E+00	0.00E+00	1.00E+00
ENSBTAG00000030802	1.92E+00	8.23E-01	1.99E+00	4.43E-01	2.55E+00	2.54E-01	0.00E+00	1.00E+00	0.00E+00	1.00E+00
ENSBTAG00000017587	0.00E+00	1.00E+00	0.00E+00	1.00E+00	-5.13E-15	1.00E+00	0.00E+00	1.00E+00	0.00E+00	1.00E+00
ENSBTAG00000030796	0.00E+00	1.00E+00	1.98E+00	4.43E-01	-5.13E-15	1.00E+00	0.00E+00	1.00E+00	0.00E+00	1.00E+00
ENSBTAG00000030798	0.00E+00	1.00E+00	1.98E+00	4.43E-01	1.78E+00	4.79E-01	3.03E+00	1.50E-01	0.00E+00	1.00E+00
ENSBTAG00000030800	1.92E+00	8.23E-01	0.00E+00	1.00E+00	-5.13E-15	1.00E+00	0.00E+00	1.00E+00	0.00E+00	1.00E+00
ENSBTAG00000017568	0.00E+00	1.00E+00	1.98E+00	4.43E-01	-5.13E-15	1.00E+00	0.00E+00	1.00E+00	0.00E+00	1.00E+00
ENSBTAG00000009626	1.92E+00	8.23E-01	1.99E+00	4.43E-01	-5.13E-15	1.00E+00	2.53E+00	2.71E-01	0.00E+00	1.00E+00
ENSBTAG00000016742	1.59E-01	1.00E+00	-1.10E+00	5.24E-03	-1.21E+00	1.60E-03	-1.76E+00	6.10E-06	8.63E-02	1.00E+00
ENSBTAG00000016743	9.34E-02	1.00E+00	4.81E-01	3.22E-02	3.21E-01	2.01E-01	-1.13E-01	8.81E-01	1.73E-01	9.47E-01
ENSBTAG00000043272	0.00E+00	1.00E+00	0.00E+00	1.00E+00	-5.13E-15	1.00E+00	0.00E+00	1.00E+00	0.00E+00	1.00E+00
ENSBTAG00000016746	-2.44E-01	1.00E+00	1.51E-01	1.00E+00	-1.15E+00	1.09E-02	-4.25E-01	4.90E-01	-1.17E+00	1.05E-01
ENSBTAG00000046725	-2.00E+00	2.28E-01	6.76E-01	6.79E-01	-3.83E+00	3.55E-04	-1.63E+00	1.40E-01	-7.09E-01	9.77E-01
ENSBTAG00000020371	-7.00E-01	1.33E-01	-1.91E+00	8.06E-11	-2.48E+00	4.13E-17	-2.12E+00	5.48E-13	1.73E-01	1.00E+00
ENSBTAG00000037709	-2.17E-01	1.00E+00	-4.15E-01	5.16E-01	-1.12E+00	1.71E-02	-7.22E-02	1.00E+00	-1.97E-01	1.00E+00
ENSBTAG00000000958	-2.82E+00	4.08E-13	-5.31E+00	4.05E-28	-4.42E+00	1.32E-25	-3.57E+00	3.88E-20	7.10E-02	1.00E+00
ENSBTAG00000000959	9.53E-01	1.18E-02	-9.21E-01	8.40E-03	-9.61E-01	4.55E-03	-4.51E-01	2.69E-01	5.84E-01	3.88E-01
ENSBTAG00000000960	7.83E-01	4.61E-01	-1.04E+00	1.26E-01	-9.28E-01	1.56E-01	-1.43E+00	2.59E-02	-2.00E-01	1.00E+00
ENSBTAG00000000961	1.69E+00	1.55E-05	1.03E+00	7.46E-03	4.10E-02	1.00E+00	-2.16E-01	8.66E-01	4.60E-01	7.79E-01
ENSBTAG00000027337	1.59E-01	1.00E+00	8.74E-02	9.78E-01	-4.78E-01	3.08E-02	-7.28E-01	5.31E-04	-2.62E-02	1.00E+00
ENSBTAG00000000559	1.65E+00	5.96E-08	2.25E+00	1.87E-15	1.76E+00	4.06E-10	1.25E+00	1.71E-05	-2.49E-01	9.29E-01
ENSBTAG00000010126	-2.53E-01	8.00E-01	-7.85E-01	2.63E-04	-7.28E-01	7.45E-04	-8.42E-01	9.37E-05	3.14E-01	6.01E-01
ENSBTAG00000010130	-7.15E-02	1.00E+00	7.07E-01	9.40E-04	6.85E-01	1.34E-03	4.39E-01	6.65E-02	3.13E-01	6.03E-01
ENSBTAG00000020676	2.56E+00	4.19E-04	2.80E+00	5.72E-06	3.86E+00	1.36E-12	3.78E+00	6.58E-12	8.64E-01	8.00E-01

ENSBTAG00000014179	1.44E+00	7.44E-01	1.28E+00	4.43E-01	1.02E+00	5.85E-01	1.24E+00	4.50E-01	1.84E+00	4.90E-01
ENSBTAG00000014186	3.00E-01	6.28E-01	1.02E+00	6.75E-07	1.10E+00	6.19E-08	6.19E-01	5.10E-03	4.69E-02	1.00E+00
ENSBTAG00000020736	4.21E+00	1.86E-15	3.96E+00	5.99E-15	2.02E+00	4.11E-05	1.51E+00	3.28E-03	-2.93E-01	1.00E+00
ENSBTAG00000043459	0.00E+00	1.00E+00	0.00E+00	1.00E+00	-5.13E-15	1.00E+00	0.00E+00	1.00E+00	0.00E+00	1.00E+00
ENSBTAG00000005672	7.08E-01	9.29E-01	5.44E-01	7.52E-01	5.07E-01	7.70E-01	2.32E-01	1.00E+00	4.64E-02	1.00E+00
ENSBTAG00000003635	-1.97E-01	9.71E-01	-4.36E-01	1.03E-01	-1.50E-01	7.78E-01	-2.12E-01	5.66E-01	-1.70E-02	1.00E+00
ENSBTAG00000003639	1.30E-01	1.00E+00	1.24E-01	8.12E-01	1.07E-01	8.78E-01	-5.83E-02	1.00E+00	2.08E-01	8.08E-01
ENSBTAG00000027328	0.00E+00	1.00E+00	0.00E+00	1.00E+00	2.56E+00	2.54E-01	0.00E+00	1.00E+00	1.93E+00	8.00E-01
ENSBTAG00000009675	-1.89E-01	1.00E+00	-1.56E+00	2.78E-01	-1.29E+00	3.45E-01	6.39E-01	6.54E-01	-1.10E+00	8.00E-01
ENSBTAG00000047336	-4.95E-01	1.00E+00	7.64E-01	7.36E-01	-1.58E-01	1.00E+00	-1.80E-01	1.00E+00	3.95E-01	1.00E+00
ENSBTAG00000027327	3.26E+00	3.40E-02	2.88E+00	2.63E-02	1.81E+00	2.92E-01	2.59E+00	5.87E-02	2.36E-02	1.00E+00
ENSBTAG00000000873	-5.18E-01	1.83E-01	-5.72E-01	2.22E-02	-9.25E-01	7.45E-05	-1.31E+00	1.29E-08	-1.10E+00	8.48E-05
ENSBTAG00000027326	-3.14E-01	6.83E-01	7.05E-02	1.00E+00	3.41E-01	2.32E-01	-3.60E-02	1.00E+00	-1.86E-01	9.69E-01
ENSBTAG00000002942	2.46E-02	1.00E+00	-2.45E-01	9.56E-01	-8.16E-01	2.28E-01	-3.53E-01	7.89E-01	6.48E-01	8.00E-01
ENSBTAG00000042101	0.00E+00	1.00E+00	0.00E+00	1.00E+00	-5.13E-15	1.00E+00	0.00E+00	1.00E+00	0.00E+00	1.00E+00
ENSBTAG00000013336	-3.51E-01	1.00E+00	-2.07E-01	1.00E+00	-5.55E-01	6.53E-01	7.95E-01	3.77E-01	-1.39E-02	1.00E+00
ENSBTAG00000013114	1.73E-01	1.00E+00	-3.35E-01	3.35E-01	1.25E-01	9.31E-01	7.21E-01	9.85E-03	-3.43E-02	1.00E+00
ENSBTAG00000044512	0.00E+00	1.00E+00	0.00E+00	1.00E+00	-5.13E-15	1.00E+00	0.00E+00	1.00E+00	0.00E+00	1.00E+00
ENSBTAG00000042533	0.00E+00	1.00E+00	0.00E+00	1.00E+00	-5.13E-15	1.00E+00	1.76E+00	4.97E-01	0.00E+00	1.00E+00
ENSBTAG00000007489	1.97E-01	1.00E+00	-1.70E-01	7.81E-01	-2.36E-04	1.00E+00	4.97E-01	9.33E-02	2.94E-01	8.00E-01
ENSBTAG00000007490	-2.18E-01	1.00E+00	-9.26E-01	6.32E-03	-8.36E-01	1.21E-02	-7.04E-01	4.37E-02	2.87E-01	8.65E-01
ENSBTAG00000043724	0.00E+00	1.00E+00	0.00E+00	1.00E+00	-5.13E-15	1.00E+00	0.00E+00	1.00E+00	0.00E+00	1.00E+00
ENSBTAG00000015609	-1.02E-01	1.00E+00	-6.28E-01	6.48E-03	-4.77E-01	4.82E-02	-8.46E-02	1.00E+00	-6.28E-02	1.00E+00
ENSBTAG00000007120	1.07E-01	1.00E+00	2.50E-01	6.14E-01	6.73E-01	3.65E-02	9.98E-01	1.10E-03	6.76E-01	2.27E-01
ENSBTAG00000005957	-1.55E-01	1.00E+00	2.52E-02	1.00E+00	5.43E-01	1.09E-02	7.54E-01	2.59E-04	-4.16E-01	3.32E-01
ENSBTAG00000000241	-1.79E-01	8.32E-01	2.72E-01	1.90E-01	-9.04E-02	8.87E-01	-3.33E-01	9.47E-02	5.36E-02	1.00E+00
ENSBTAG00000006671	3.05E-01	4.92E-01	2.34E+00	2.07E-39	2.43E+00	2.08E-42	1.65E+00	9.99E-21	-1.20E-01	1.00E+00
ENSBTAG00000020166	-2.38E-01	1.00E+00	1.40E+00	4.83E-02	6.33E-01	4.88E-01	-1.62E-01	1.00E+00	-4.99E-01	9.79E-01
ENSBTAG00000042099	1.07E+00	3.78E-01	2.08E+00	1.22E-04	2.64E+00	1.17E-07	2.10E+00	9.39E-05	6.35E-01	8.23E-01
ENSBTAG00000043036	8.18E-01	4.00E-01	1.83E+00	1.64E-05	2.39E+00	2.44E-09	1.51E+00	6.22E-04	-2.76E-01	1.00E+00
ENSBTAG00000043350	1.33E+00	7.49E-02	1.98E+00	5.63E-05	2.50E+00	7.07E-08	1.68E+00	9.28E-04	5.36E-01	8.46E-01
ENSBTAG00000027320	-1.62E-01	1.00E+00	-5.10E-01	2.88E-01	-1.23E+00	1.69E-03	-1.22E+00	2.27E-03	-5.20E-01	6.59E-01
ENSBTAG00000017537	5.87E-03	1.00E+00	-5.98E-01	1.14E-01	-2.68E-01	6.20E-01	2.83E-01	5.87E-01	1.89E-01	1.00E+00
ENSBTAG00000012626	-1.78E-01	1.00E+00	4.37E-01	3.81E-01	5.70E-02	1.00E+00	2.83E-01	6.72E-01	7.98E-02	1.00E+00
ENSBTAG00000008969	1.80E-01	1.00E+00	3.09E-01	5.07E-01	4.66E-01	2.30E-01	1.70E-01	8.81E-01	8.96E-02	1.00E+00

ENSBTAG00000018063	-4.39E-02	1.00E+00	1.26E+00	2.34E-08	1.17E+00	2.25E-07	5.95E-01	1.82E-02	1.55E-01	1.00E+00
ENSBTAG00000027317	-3.62E-01	5.76E-01	1.37E-01	8.48E-01	5.98E-01	1.68E-02	4.43E-01	1.07E-01	-2.87E-01	7.81E-01
ENSBTAG00000044591	0.00E+00	1.00E+00	0.00E+00	1.00E+00	-5.13E-15	1.00E+00	0.00E+00	1.00E+00	0.00E+00	1.00E+00
ENSBTAG00000014554	4.55E-01	8.23E-01	1.61E+00	2.88E-05	1.64E+00	1.59E-05	1.34E+00	7.10E-04	-2.69E-01	1.00E+00
ENSBTAG00000027316	-2.29E-02	1.00E+00	-1.31E-01	7.15E-01	-3.25E-01	1.19E-01	-6.62E-01	3.17E-04	3.44E-02	1.00E+00
ENSBTAG00000010135	2.64E-02	1.00E+00	-1.55E-02	1.00E+00	-8.66E-02	1.00E+00	-2.10E-01	6.55E-01	-1.55E-01	1.00E+00
ENSBTAG00000006616	8.41E-01	2.82E-02	2.34E+00	6.81E-17	1.57E+00	1.96E-08	1.07E+00	2.25E-04	3.13E-01	8.00E-01
ENSBTAG00000012297	4.13E-01	5.02E-01	1.56E+00	2.29E-10	9.73E-01	1.17E-04	5.92E-01	3.41E-02	2.15E-01	9.37E-01
ENSBTAG00000021601	5.06E-01	8.23E-01	1.76E+00	1.59E-04	1.18E+00	1.57E-02	1.52E+00	1.44E-03	6.47E-01	6.47E-01
ENSBTAG00000038929	-2.78E-02	1.00E+00	-1.26E-01	1.00E+00	3.67E-02	1.00E+00	2.09E-01	8.23E-01	6.28E-02	1.00E+00
ENSBTAG00000000340	2.84E-01	6.76E-01	1.10E+00	5.66E-08	8.15E-01	7.97E-05	4.84E-01	3.52E-02	3.45E-01	5.25E-01
ENSBTAG00000007721	2.30E-01	7.43E-01	9.10E-01	2.15E-07	9.07E-01	2.04E-07	6.45E-01	4.32E-04	-2.21E-01	7.78E-01
ENSBTAG00000007722	3.15E-01	7.32E-01	1.82E+00	2.99E-15	1.66E+00	6.25E-13	1.22E+00	2.28E-07	1.34E-01	1.00E+00
ENSBTAG00000043709	0.00E+00	1.00E+00	0.00E+00	1.00E+00	-5.13E-15	1.00E+00	0.00E+00	1.00E+00	0.00E+00	1.00E+00
ENSBTAG00000003398	-8.97E-01	6.87E-01	-1.13E+00	1.79E-01	-6.20E-01	4.92E-01	-5.57E-01	5.74E-01	-1.39E+00	3.78E-01
ENSBTAG00000018270	1.24E-01	1.00E+00	2.25E-01	8.32E-01	-4.62E-01	3.83E-01	4.65E-02	1.00E+00	-6.27E-01	5.81E-01
ENSBTAG00000007962	1.56E-01	1.00E+00	2.49E-01	7.08E-01	5.62E-01	1.63E-01	8.19E-01	2.76E-02	3.33E-01	8.46E-01
ENSBTAG00000003101	4.14E-02	1.00E+00	-1.16E+00	4.09E-03	-2.09E+00	1.06E-07	-1.89E+00	1.88E-06	1.37E-01	1.00E+00
ENSBTAG00000030564	1.91E+00	8.23E-01	1.98E+00	4.43E-01	-5.13E-15	1.00E+00	0.00E+00	1.00E+00	0.00E+00	1.00E+00
ENSBTAG00000047987	0.00E+00	1.00E+00	0.00E+00	1.00E+00	-5.13E-15	1.00E+00	0.00E+00	1.00E+00	0.00E+00	1.00E+00
ENSBTAG00000001512	-1.39E-01	1.00E+00	-3.47E-01	2.54E-01	6.76E-01	5.70E-03	5.26E-01	4.63E-02	1.11E-01	1.00E+00
ENSBTAG00000007917	1.09E-01	1.00E+00	-5.57E-01	1.53E-01	-1.38E+00	4.29E-05	-1.46E+00	1.77E-05	7.89E-01	1.53E-01
ENSBTAG00000042191	0.00E+00	1.00E+00	0.00E+00	1.00E+00	-5.13E-15	1.00E+00	0.00E+00	1.00E+00	0.00E+00	1.00E+00
ENSBTAG00000042320	0.00E+00	1.00E+00	0.00E+00	1.00E+00	-5.13E-15	1.00E+00	0.00E+00	1.00E+00	0.00E+00	1.00E+00
ENSBTAG00000030556	2.33E-01	9.50E-01	1.41E+00	2.80E-08	8.26E-01	1.99E-03	6.34E-01	2.72E-02	2.06E-01	9.90E-01
ENSBTAG00000000835	1.75E-01	1.00E+00	-2.32E+00	6.44E-16	-3.76E+00	4.13E-35	-4.06E+00	4.52E-39	2.79E-01	8.44E-01
ENSBTAG00000002765	-1.13E+00	8.23E-01	-1.55E+00	2.68E-01	-3.82E-01	9.77E-01	-1.64E-02	1.00E+00	-2.43E+00	3.72E-01
ENSBTAG00000008812	2.37E-01	9.12E-01	3.14E-01	3.55E-01	5.98E-01	2.60E-02	1.79E-01	7.32E-01	-2.61E-01	8.24E-01
ENSBTAG00000047975	2.91E-01	1.00E+00	1.31E+00	5.76E-02	1.15E+00	1.06E-01	1.31E+00	5.75E-02	1.17E-01	1.00E+00
ENSBTAG00000045236	0.00E+00	1.00E+00	1.98E+00	4.43E-01	-5.13E-15	1.00E+00	0.00E+00	1.00E+00	0.00E+00	1.00E+00
ENSBTAG00000017524	-2.41E+00	3.16E-01	-4.33E+00	1.13E-02	-2.55E+00	5.99E-02	-1.80E+00	1.76E-01	-2.40E+00	3.88E-01
ENSBTAG00000038057	1.92E+00	8.23E-01	0.00E+00	1.00E+00	-5.13E-15	1.00E+00	0.00E+00	1.00E+00	0.00E+00	1.00E+00
ENSBTAG00000035883	0.00E+00	1.00E+00	0.00E+00	1.00E+00	1.78E+00	4.79E-01	0.00E+00	1.00E+00	0.00E+00	1.00E+00
ENSBTAG00000046736	0.00E+00	1.00E+00	0.00E+00	1.00E+00	-5.13E-15	1.00E+00	0.00E+00	1.00E+00	0.00E+00	1.00E+00
ENSBTAG00000046737	1.83E-02	1.00E+00	-2.70E+00	2.42E-01	-2.70E+00	1.99E-01	-2.70E+00	2.05E-01	-2.70E+00	6.01E-01

ENSBTAG00000036045	0.00E+00	1.00E+00	0.00E+00	1.00E+00	-5.13E-15	1.00E+00	0.00E+00	1.00E+00	0.00E+00	1.00E+00
ENSBTAG00000046868	2.71E+00	5.76E-01	0.00E+00	1.00E+00	-5.13E-15	1.00E+00	0.00E+00	1.00E+00	0.00E+00	1.00E+00
ENSBTAG00000048266	-2.71E+00	5.76E-01	-2.71E+00	2.42E-01	-2.71E+00	1.99E-01	-2.71E+00	2.05E-01	1.15E-02	1.00E+00
ENSBTAG00000046827	0.00E+00	1.00E+00	0.00E+00	1.00E+00	-5.13E-15	1.00E+00	0.00E+00	1.00E+00	0.00E+00	1.00E+00
ENSBTAG00000046822	0.00E+00	1.00E+00	0.00E+00	1.00E+00	-5.13E-15	1.00E+00	0.00E+00	1.00E+00	0.00E+00	1.00E+00
ENSBTAG00000045988	0.00E+00	1.00E+00	0.00E+00	1.00E+00	1.78E+00	4.79E-01	0.00E+00	1.00E+00	0.00E+00	1.00E+00
ENSBTAG00000034261	4.59E-01	5.57E-01	1.72E+00	2.78E-10	1.22E+00	1.20E-05	1.25E+00	9.33E-06	6.03E-01	3.18E-01
ENSBTAG00000047283	-7.87E-01	1.00E+00	8.27E-02	1.00E+00	-1.53E-01	1.00E+00	-2.71E+00	2.05E-01	-2.71E+00	6.01E-01
ENSBTAG00000047792	-1.91E+00	8.23E-01	-1.91E+00	4.69E-01	-1.91E+00	4.28E-01	-1.91E+00	4.39E-01	-1.91E+00	8.00E-01
ENSBTAG00000047897	0.00E+00	1.00E+00	2.79E+00	2.10E-01	-5.13E-15	1.00E+00	0.00E+00	1.00E+00	1.91E+00	8.00E-01
ENSBTAG00000045780	-1.91E+00	8.23E-01	-1.91E+00	4.69E-01	-1.91E+00	4.28E-01	-1.91E+00	4.39E-01	-1.91E+00	8.00E-01
ENSBTAG00000044546	0.00E+00	1.00E+00	0.00E+00	1.00E+00	-5.13E-15	1.00E+00	0.00E+00	1.00E+00	0.00E+00	1.00E+00
ENSBTAG00000046592	0.00E+00	1.00E+00	0.00E+00	1.00E+00	-5.13E-15	1.00E+00	0.00E+00	1.00E+00	0.00E+00	1.00E+00
ENSBTAG00000046719	2.72E+00	5.76E-01	0.00E+00	1.00E+00	1.78E+00	4.79E-01	0.00E+00	1.00E+00	0.00E+00	1.00E+00
ENSBTAG00000048242	0.00E+00	1.00E+00	0.00E+00	1.00E+00	-5.13E-15	1.00E+00	0.00E+00	1.00E+00	0.00E+00	1.00E+00
ENSBTAG00000047387	2.71E+00	5.76E-01	0.00E+00	1.00E+00	-5.13E-15	1.00E+00	0.00E+00	1.00E+00	0.00E+00	1.00E+00
ENSBTAG00000047800	-3.21E+00	3.75E-01	-3.21E+00	1.26E-01	-3.21E+00	9.49E-02	-3.21E+00	9.96E-02	-3.21E+00	4.34E-01
ENSBTAG00000046241	0.00E+00	1.00E+00	0.00E+00	1.00E+00	-5.13E-15	1.00E+00	1.76E+00	4.97E-01	1.93E+00	8.00E-01
ENSBTAG00000045707	0.00E+00	1.00E+00	0.00E+00	1.00E+00	-5.13E-15	1.00E+00	0.00E+00	1.00E+00	0.00E+00	1.00E+00
ENSBTAG00000045524	0.00E+00	1.00E+00	0.00E+00	1.00E+00	-5.13E-15	1.00E+00	0.00E+00	1.00E+00	0.00E+00	1.00E+00
ENSBTAG00000045815	2.72E+00	5.76E-01	1.98E+00	4.43E-01	-5.13E-15	1.00E+00	0.00E+00	1.00E+00	2.73E+00	6.01E-01
ENSBTAG00000047065	-2.71E+00	5.76E-01	-2.71E+00	2.42E-01	-2.71E+00	1.99E-01	-2.71E+00	2.05E-01	-2.71E+00	6.01E-01
ENSBTAG00000042158	0.00E+00	1.00E+00	0.00E+00	1.00E+00	-5.13E-15	1.00E+00	0.00E+00	1.00E+00	0.00E+00	1.00E+00
ENSBTAG00000034176	2.04E-01	1.00E+00	-9.08E-02	1.00E+00	-8.24E-01	5.53E-01	1.57E+00	1.10E-01	2.83E+00	1.96E-02
ENSBTAG00000045763	0.00E+00	1.00E+00	0.00E+00	1.00E+00	-5.13E-15	1.00E+00	1.76E+00	4.97E-01	3.21E+00	4.34E-01
ENSBTAG00000045557	2.70E+00	5.76E-01	0.00E+00	1.00E+00	-5.13E-15	1.00E+00	1.76E+00	4.97E-01	0.00E+00	1.00E+00
ENSBTAG00000047553	0.00E+00	1.00E+00	0.00E+00	1.00E+00	-5.13E-15	1.00E+00	0.00E+00	1.00E+00	0.00E+00	1.00E+00
ENSBTAG00000045654	0.00E+00	1.00E+00	0.00E+00	1.00E+00	-5.13E-15	1.00E+00	0.00E+00	1.00E+00	0.00E+00	1.00E+00
ENSBTAG00000036098	0.00E+00	1.00E+00	0.00E+00	1.00E+00	-5.13E-15	1.00E+00	0.00E+00	1.00E+00	0.00E+00	1.00E+00
ENSBTAG00000048001	0.00E+00	1.00E+00	0.00E+00	1.00E+00	-5.13E-15	1.00E+00	0.00E+00	1.00E+00	0.00E+00	1.00E+00
ENSBTAG00000045579	-1.75E-01	1.00E+00	-6.37E-02	1.00E+00	-6.84E-02	1.00E+00	-4.14E-01	5.16E-01	-1.14E-02	1.00E+00
ENSBTAG00000003273	0.00E+00	1.00E+00	0.00E+00	1.00E+00	-5.13E-15	1.00E+00	0.00E+00	1.00E+00	0.00E+00	1.00E+00
ENSBTAG00000006597	0.00E+00	1.00E+00	0.00E+00	1.00E+00	-5.13E-15	1.00E+00	0.00E+00	1.00E+00	0.00E+00	1.00E+00
ENSBTAG00000046932	0.00E+00	1.00E+00	1.99E+00	4.43E-01	-5.13E-15	1.00E+00	0.00E+00	1.00E+00	0.00E+00	1.00E+00
ENSBTAG00000046435	0.00E+00	1.00E+00	0.00E+00	1.00E+00	-5.13E-15	1.00E+00	0.00E+00	1.00E+00	0.00E+00	1.00E+00

ENSBTAG00000047447	0.00E+00	1.00E+00	0.00E+00	1.00E+00	-5.13E-15	1.00E+00	0.00E+00	1.00E+00	0.00E+00	1.00E+00
ENSBTAG00000016203	-2.07E-02	1.00E+00	1.70E+00	5.55E-13	1.30E+00	5.32E-08	7.52E-01	4.46E-03	3.27E-01	7.65E-01
ENSBTAG00000000879	-2.19E-01	1.00E+00	5.84E-01	4.83E-02	1.29E+00	7.79E-07	1.29E+00	9.88E-07	-2.71E-01	8.41E-01
ENSBTAG00000012353	1.92E-01	9.27E-01	6.98E-02	1.00E+00	3.66E-02	1.00E+00	-3.16E-01	2.30E-01	1.65E-01	9.75E-01
ENSBTAG00000026327	-6.60E-02	1.00E+00	2.22E-01	5.22E-01	2.78E-01	3.80E-01	4.00E-01	1.50E-01	-1.36E-01	1.00E+00
ENSBTAG00000018456	-5.59E-02	1.00E+00	7.53E-01	5.81E-04	1.01E+00	1.58E-06	3.86E-01	1.34E-01	2.29E-01	8.06E-01
ENSBTAG00000018455	-3.73E-01	4.87E-01	-6.90E-01	3.40E-03	-4.57E-01	7.09E-02	-4.98E-01	4.95E-02	-2.86E-01	7.58E-01
ENSBTAG00000007186	2.54E-03	1.00E+00	-1.04E+00	9.34E-05	-4.33E-01	1.51E-01	-9.24E-02	1.00E+00	1.50E-01	1.00E+00
ENSBTAG00000045478	0.00E+00	1.00E+00	0.00E+00	1.00E+00	-5.13E-15	1.00E+00	0.00E+00	1.00E+00	0.00E+00	1.00E+00
ENSBTAG00000046031	-1.07E-01	1.00E+00	-8.38E-01	6.69E-03	-3.56E-01	3.67E-01	2.94E-01	4.97E-01	-1.64E-01	1.00E+00
ENSBTAG00000004970	-2.35E-01	9.44E-01	-9.58E-03	1.00E+00	-8.32E-02	1.00E+00	-3.00E-01	4.39E-01	-2.80E-01	8.02E-01
ENSBTAG00000004969	-2.60E-01	8.23E-01	-1.02E-02	1.00E+00	5.59E-01	1.71E-02	5.47E-01	2.31E-02	-4.84E-02	1.00E+00
ENSBTAG00000010276	2.99E-02	1.00E+00	1.01E+00	2.08E-02	8.97E-01	4.47E-02	2.57E-01	8.21E-01	-1.33E-01	1.00E+00
ENSBTAG00000007838	-1.83E-01	1.00E+00	-7.53E-02	1.00E+00	-7.54E-01	1.79E-03	-9.85E-01	3.92E-05	1.04E-01	1.00E+00
ENSBTAG00000007835	-4.76E-01	7.71E-01	-1.08E+00	1.20E-02	-9.59E-01	2.33E-02	-1.42E+00	7.10E-04	2.07E-02	1.00E+00
ENSBTAG00000007834	-3.10E-01	8.23E-01	-1.87E+00	1.08E-10	-1.65E+00	3.81E-09	-9.36E-01	1.09E-03	3.34E-02	1.00E+00
ENSBTAG00000004761	-1.91E+00	8.23E-01	8.80E-01	8.52E-01	-1.91E+00	4.28E-01	1.12E+00	6.70E-01	-1.91E+00	8.00E-01
ENSBTAG00000007753	-1.82E-01	1.00E+00	-1.26E+00	1.60E-04	-1.42E+00	1.22E-05	-1.22E+00	2.19E-04	1.93E-01	1.00E+00
ENSBTAG00000035254	-4.66E-02	1.00E+00	1.71E-01	6.24E-01	3.95E-01	9.15E-02	1.49E-02	1.00E+00	3.18E-01	5.98E-01
ENSBTAG00000007749	1.98E-01	1.00E+00	7.36E-02	1.00E+00	3.57E-02	1.00E+00	-3.47E-01	5.02E-01	4.31E-01	7.59E-01
ENSBTAG00000026320	-1.58E-01	1.00E+00	-6.74E-01	3.40E-03	-1.31E+00	1.47E-09	-1.38E+00	3.38E-10	-2.55E-01	8.00E-01
ENSBTAG00000046026	-5.13E-01	5.29E-01	-4.53E-01	2.63E-01	-1.25E+00	1.24E-04	-1.22E+00	2.16E-04	-1.70E-01	1.00E+00
ENSBTAG00000008355	-1.23E-01	1.00E+00	-2.27E-01	4.20E-01	-3.90E-02	1.00E+00	-2.11E-01	4.72E-01	1.98E-01	8.38E-01
ENSBTAG00000011064	1.44E-01	1.00E+00	8.87E-01	6.61E-05	1.15E+00	1.27E-07	5.41E-01	2.67E-02	5.85E-02	1.00E+00
ENSBTAG00000000857	8.29E-02	1.00E+00	4.39E-01	1.33E-01	3.78E-01	2.19E-01	6.29E-02	1.00E+00	-1.04E-02	1.00E+00
ENSBTAG00000000856	3.90E-01	8.82E-01	1.04E+00	1.40E-02	1.97E-01	9.15E-01	-9.57E-01	3.21E-02	4.17E-01	8.06E-01
ENSBTAG00000035158	3.49E-01	8.78E-01	3.25E-01	5.56E-01	-6.63E-01	1.07E-01	-1.42E+00	1.24E-04	5.27E-01	6.01E-01
ENSBTAG00000046208	-1.91E+00	8.23E-01	-1.91E+00	4.69E-01	-1.91E+00	4.28E-01	1.48E+00	4.57E-01	-1.91E+00	8.00E-01
ENSBTAG00000026356	-2.63E-02	1.00E+00	-3.54E-01	4.98E-01	-1.12E-01	1.00E+00	-9.94E-02	1.00E+00	2.64E-01	1.00E+00
ENSBTAG00000020751	-1.82E-02	1.00E+00	7.66E-01	1.61E-04	1.05E+00	8.99E-08	7.99E-01	9.24E-05	4.55E-02	1.00E+00
ENSBTAG00000009811	1.05E-01	1.00E+00	1.37E+00	2.27E-09	1.71E+00	3.49E-14	1.39E+00	1.53E-09	-9.06E-02	1.00E+00
ENSBTAG00000009816	0.00E+00	1.00E+00	0.00E+00	1.00E+00	-5.13E-15	1.00E+00	0.00E+00	1.00E+00	0.00E+00	1.00E+00
ENSBTAG00000014458	-4.84E-02	1.00E+00	-1.87E-01	5.57E-01	-8.61E-01	2.19E-05	-1.22E+00	1.21E-09	5.99E-02	1.00E+00
ENSBTAG00000044406	0.00E+00	1.00E+00	0.00E+00	1.00E+00	-5.13E-15	1.00E+00	0.00E+00	1.00E+00	0.00E+00	1.00E+00
ENSBTAG00000039978	7.37E-01	3.59E-01	3.97E+00	2.08E-28	2.67E+00	7.74E-14	7.61E-01	9.37E-02	2.16E-01	1.00E+00

ENSBTAG00000039705	-1.40E-01	1.00E+00	8.91E-01	5.88E-04	1.66E+00	2.09E-11	1.69E+00	1.28E-11	-6.06E-02	1.00E+00
ENSBTAG00000000658	1.04E-01	1.00E+00	1.54E-01	7.23E-01	-3.00E-01	2.73E-01	-1.33E+00	6.16E-10	7.66E-02	1.00E+00
ENSBTAG00000012242	-3.53E-01	4.68E-01	-8.37E-01	7.96E-05	-4.09E-01	8.81E-02	-1.32E-01	8.07E-01	2.34E-01	8.00E-01
ENSBTAG00000012235	-1.97E-01	9.09E-01	6.65E-01	1.64E-03	1.13E+00	1.41E-08	2.76E-01	3.13E-01	-2.92E-02	1.00E+00
ENSBTAG00000012232	-2.53E-01	9.06E-01	-1.95E-01	7.10E-01	2.30E-01	5.95E-01	6.26E-01	3.40E-02	-2.21E-01	9.50E-01
ENSBTAG00000014610	-3.49E-02	1.00E+00	-2.50E-01	3.22E-01	-7.96E-01	3.07E-05	-9.82E-01	2.45E-07	1.00E-01	1.00E+00
ENSBTAG00000014607	-2.01E-01	1.00E+00	1.15E-01	1.00E+00	7.21E-01	3.63E-02	7.73E-01	2.57E-02	-2.93E-01	8.97E-01
ENSBTAG00000017281	-2.41E-01	1.00E+00	-9.18E-01	3.50E-03	-1.35E+00	8.53E-06	-6.62E-01	5.21E-02	1.96E-01	1.00E+00
ENSBTAG00000015040	-1.88E-01	1.00E+00	9.81E-02	1.00E+00	-1.28E+00	3.41E-01	-1.31E+00	3.37E-01	1.00E+00	7.26E-01
ENSBTAG00000026350	-1.37E-01	1.00E+00	1.81E+00	3.00E-03	-1.60E-01	1.00E+00	-5.95E-01	6.14E-01	3.63E-01	1.00E+00
ENSBTAG00000000312	-1.30E-02	1.00E+00	-2.60E-01	4.41E-01	-1.80E-02	1.00E+00	2.34E-01	4.98E-01	-6.44E-02	1.00E+00
ENSBTAG00000009677	-1.01E-01	1.00E+00	-1.37E-01	9.52E-01	-3.74E-01	3.56E-01	-6.38E-01	6.36E-02	1.76E-01	1.00E+00
ENSBTAG00000011922	-4.19E-01	8.23E-01	-2.06E+00	1.93E-07	-1.89E+00	1.76E-06	-4.39E-01	4.39E-01	4.88E-01	7.68E-01
ENSBTAG00000044263	0.00E+00	1.00E+00	0.00E+00	1.00E+00	-5.13E-15	1.00E+00	0.00E+00	1.00E+00	0.00E+00	1.00E+00
ENSBTAG00000045727	-6.57E-01	6.39E-01	-3.60E+00	3.90E-13	-2.37E+00	7.09E-07	-6.14E-01	3.26E-01	1.97E-01	1.00E+00
ENSBTAG00000008079	-3.27E-01	7.45E-01	-1.44E+00	1.92E-08	-1.29E+00	3.97E-07	-5.68E-01	4.82E-02	2.51E-01	8.46E-01
ENSBTAG00000037493	1.87E-01	9.31E-01	5.11E-01	1.94E-02	6.51E-01	1.86E-03	4.97E-01	2.68E-02	4.68E-02	1.00E+00
ENSBTAG00000008421	-1.17E-01	1.00E+00	1.01E+00	3.53E-07	1.13E-01	8.60E-01	4.26E-03	1.00E+00	2.52E-01	7.71E-01
ENSBTAG00000038682	-4.23E-01	5.44E-01	-8.69E-01	1.69E-03	-1.30E-01	9.23E-01	2.48E-01	5.53E-01	6.92E-02	1.00E+00
ENSBTAG00000019864	-1.58E-01	1.00E+00	-7.14E-01	9.89E-02	-1.03E+00	9.39E-03	-1.45E+00	2.25E-04	3.76E-01	8.19E-01
ENSBTAG00000040360	-3.75E-01	1.00E+00	8.20E-01	2.92E-01	1.01E+00	1.40E-01	-3.57E-01	8.84E-01	3.03E-01	1.00E+00
ENSBTAG00000045791	-7.04E-02	1.00E+00	1.68E+00	1.72E-06	1.16E+00	1.46E-03	5.91E-01	1.81E-01	1.98E-01	1.00E+00
ENSBTAG00000046866	-2.89E-01	1.00E+00	-2.60E+00	2.59E-04	-5.96E-01	4.27E-01	4.05E-01	6.26E-01	8.11E-01	4.99E-01
ENSBTAG00000034691	-1.43E-01	1.00E+00	1.45E-01	8.22E-01	6.61E-01	8.14E-03	6.52E-01	1.07E-02	9.43E-02	1.00E+00
ENSBTAG00000016810	-4.58E-02	1.00E+00	6.98E-01	1.29E-03	9.98E-03	1.00E+00	1.77E-01	6.32E-01	-1.18E-01	1.00E+00
ENSBTAG00000048160	-2.56E-01	9.36E-01	4.79E-02	1.00E+00	4.64E-01	1.49E-01	7.14E-01	1.39E-02	3.50E-01	7.56E-01
ENSBTAG00000014643	3.74E-02	1.00E+00	6.39E-01	3.93E-03	7.87E-01	2.56E-04	6.35E-01	4.90E-03	-6.07E-01	7.20E-02
ENSBTAG00000014642	-3.02E-01	6.37E-01	-1.25E+00	3.13E-09	-2.08E+00	4.12E-23	-2.41E+00	6.92E-30	-1.97E-01	8.97E-01
ENSBTAG00000034613	1.50E-01	1.00E+00	1.13E-02	1.00E+00	-1.17E+00	2.22E-03	-2.03E+00	1.62E-07	1.31E-01	1.00E+00
ENSBTAG00000021474	-4.28E-01	3.93E-01	-8.64E-01	4.33E-04	-2.99E-01	3.46E-01	-5.24E-01	5.15E-02	1.75E-01	1.00E+00
ENSBTAG00000021472	-1.23E-01	1.00E+00	-2.82E-01	4.40E-01	2.78E-01	4.28E-01	2.49E-01	5.00E-01	1.87E-01	1.00E+00
ENSBTAG00000026344	1.32E+00	9.28E-01	1.77E+00	3.26E-01	6.40E-01	9.79E-01	-1.91E+00	4.39E-01	1.32E+00	8.97E-01
ENSBTAG00000002104	-3.15E-01	1.00E+00	-1.04E+00	4.22E-02	-5.07E-01	3.98E-01	-5.85E-01	3.07E-01	-3.84E-01	9.12E-01
ENSBTAG00000003606	-3.60E-01	6.06E-01	-1.39E+00	1.84E-08	-1.15E+00	2.94E-06	-1.37E+00	3.35E-08	-4.09E-01	5.33E-01
ENSBTAG00000011939	-5.04E-04	1.00E+00	8.74E-01	8.53E-01	6.44E-01	9.78E-01	-1.53E-01	1.00E+00	5.96E-04	1.00E+00

ENSBTAG00000017040	-6.77E-01	8.53E-01	-1.15E+00	1.43E-01	-2.23E+00	1.51E-03	-2.68E+00	1.74E-04	-9.65E-01	6.22E-01
ENSBTAG00000004596	2.72E+00	5.76E-01	0.00E+00	1.00E+00	2.56E+00	2.54E-01	0.00E+00	1.00E+00	0.00E+00	1.00E+00
ENSBTAG00000004595	8.05E-01	1.00E+00	1.78E+00	3.26E-01	-1.91E+00	4.28E-01	1.78E+00	3.07E-01	8.12E-01	1.00E+00
ENSBTAG00000000158	-5.35E-01	7.86E-01	-1.65E+00	1.58E-03	-4.56E-01	4.66E-01	-1.57E+00	1.78E-03	-5.84E-01	7.29E-01
ENSBTAG00000037824	-2.70E+00	5.76E-01	-2.70E+00	2.42E-01	-9.22E-01	8.08E-01	-2.70E+00	2.05E-01	-2.70E+00	6.01E-01
ENSBTAG00000034498	1.45E+00	7.49E-01	1.28E+00	4.44E-01	-2.70E+00	1.99E-01	6.95E-01	8.56E-01	1.82E-02	1.00E+00
ENSBTAG00000005762	-2.62E-01	1.00E+00	-2.00E+00	1.37E-03	-2.40E+00	9.57E-05	-9.55E-01	1.37E-01	-3.81E-01	1.00E+00
ENSBTAG00000016210	7.97E-01	1.00E+00	1.78E+00	3.25E-01	-1.30E-01	1.00E+00	-1.91E+00	4.39E-01	7.14E-03	1.00E+00
ENSBTAG00000016209	-1.91E+00	8.23E-01	8.83E-01	8.52E-01	-1.25E-01	1.00E+00	6.27E-01	9.94E-01	1.69E+00	7.74E-01
ENSBTAG00000005063	-5.88E-01	5.10E-01	-1.24E+00	9.50E-04	-1.65E+00	7.84E-06	-2.24E+00	2.07E-09	-5.05E-01	6.61E-01
ENSBTAG00000047022	-1.08E-01	1.00E+00	-1.45E+00	2.22E-04	-2.04E+00	1.38E-07	-2.46E+00	6.51E-10	2.07E-01	1.00E+00
ENSBTAG00000021639	-6.45E-01	1.00E+00	2.13E+00	7.85E-03	7.94E-01	4.96E-01	1.47E-01	1.00E+00	-2.34E+00	3.15E-01
ENSBTAG00000026340	6.19E-01	8.81E-01	-1.14E-02	1.00E+00	-1.03E+00	2.94E-01	-7.08E-01	5.21E-01	2.20E-01	1.00E+00
ENSBTAG00000006385	-6.86E-01	7.67E-01	-2.46E-01	9.45E-01	-4.60E-01	5.79E-01	-7.50E-01	2.83E-01	-7.91E-01	6.44E-01
ENSBTAG00000009974	1.85E-01	9.44E-01	-7.38E-01	6.46E-04	-1.03E+00	9.30E-07	-6.54E-01	3.13E-03	3.95E-01	4.14E-01
ENSBTAG00000046166	7.51E-01	8.23E-01	-7.22E-01	5.17E-01	-1.12E+00	2.31E-01	-1.88E+00	3.63E-02	1.24E+00	4.07E-01
ENSBTAG00000046467	-6.69E-01	3.46E-01	-2.85E+00	1.34E-14	-3.22E+00	5.68E-18	-1.97E+00	4.31E-08	-5.25E-01	6.03E-01
ENSBTAG00000015985	8.04E-01	1.00E+00	-1.91E+00	4.69E-01	-1.31E-01	1.00E+00	-1.47E-01	1.00E+00	7.99E-01	1.00E+00
ENSBTAG00000008597	6.91E-01	2.89E-01	4.16E-01	4.03E-01	2.93E-01	6.21E-01	6.27E-01	1.27E-01	5.40E-01	6.01E-01
ENSBTAG00000006132	1.49E-01	1.00E+00	-4.89E-01	3.19E-01	-1.31E-02	1.00E+00	6.64E-01	1.19E-01	5.37E-01	6.05E-01
ENSBTAG00000009578	1.35E-01	1.00E+00	6.61E-01	4.10E-04	7.27E-01	8.18E-05	5.73E-01	3.08E-03	1.36E-01	1.00E+00
ENSBTAG00000029865	0.00E+00	1.00E+00	0.00E+00	1.00E+00	-5.13E-15	1.00E+00	0.00E+00	1.00E+00	0.00E+00	1.00E+00
ENSBTAG00000001579	4.60E-01	3.22E-01	1.88E+00	1.40E-16	1.64E+00	4.12E-13	1.46E+00	2.25E-10	4.99E-01	3.10E-01
ENSBTAG00000020226	-2.68E-01	8.23E-01	2.39E-02	1.00E+00	2.92E-01	3.43E-01	5.58E-02	1.00E+00	-8.57E-02	1.00E+00
ENSBTAG00000013955	-1.94E-01	1.00E+00	-8.82E-01	4.81E-04	-1.39E+00	1.18E-08	-8.24E-01	1.35E-03	-1.05E-01	1.00E+00
ENSBTAG00000045371	0.00E+00	1.00E+00	0.00E+00	1.00E+00	-5.13E-15	1.00E+00	0.00E+00	1.00E+00	0.00E+00	1.00E+00
ENSBTAG00000047488	3.67E-01	1.00E+00	-3.79E-01	8.31E-01	-4.93E-02	1.00E+00	2.08E-02	1.00E+00	-9.87E-02	1.00E+00
ENSBTAG00000015374	-4.97E-02	1.00E+00	-1.42E-01	1.00E+00	-3.98E-01	4.41E-01	1.04E-01	1.00E+00	5.26E-01	6.01E-01
ENSBTAG00000042332	0.00E+00	1.00E+00	0.00E+00	1.00E+00	-5.13E-15	1.00E+00	0.00E+00	1.00E+00	0.00E+00	1.00E+00
ENSBTAG00000018218	-7.91E-01	8.46E-01	-5.03E-01	7.69E-01	-3.50E-01	9.31E-01	2.43E-01	1.00E+00	-8.45E-01	8.00E-01
ENSBTAG00000047010	0.00E+00	1.00E+00	0.00E+00	1.00E+00	-5.13E-15	1.00E+00	0.00E+00	1.00E+00	0.00E+00	1.00E+00
ENSBTAG00000048078	-2.71E+00	5.76E-01	-2.71E+00	2.42E-01	-1.55E-01	1.00E+00	1.77E+00	1.74E-01	-7.94E-01	1.00E+00
ENSBTAG00000045100	0.00E+00	1.00E+00	0.00E+00	1.00E+00	-5.13E-15	1.00E+00	0.00E+00	1.00E+00	0.00E+00	1.00E+00
ENSBTAG00000002181	-5.62E-01	2.61E-01	-1.93E-02	1.00E+00	-2.11E-01	6.61E-01	2.59E-01	5.22E-01	-6.83E-01	1.67E-01
ENSBTAG00000029987	0.00E+00	1.00E+00	0.00E+00	1.00E+00	-5.13E-15	1.00E+00	0.00E+00	1.00E+00	0.00E+00	1.00E+00

ENSBTAG00000029972	-1.91E+00	8.23E-01	7.71E-02	1.00E+00	-1.91E+00	4.28E-01	6.20E-01	9.94E-01	7.99E-01	1.00E+00
ENSBTAG00000017731	2.23E-02	1.00E+00	3.66E-01	3.81E-01	6.13E-01	6.51E-02	4.17E-01	2.83E-01	5.84E-01	3.83E-01
ENSBTAG00000001156	-9.21E-03	1.00E+00	-8.09E-01	1.14E-02	-4.80E-01	1.90E-01	-5.23E-01	1.50E-01	2.54E-01	9.38E-01
ENSBTAG00000000711	1.93E-01	1.00E+00	2.54E-01	9.83E-01	4.34E-01	7.10E-01	1.65E+00	1.18E-02	-4.31E-02	1.00E+00
ENSBTAG00000000707	2.94E+00	7.39E-03	1.43E+00	2.10E-01	8.78E-01	5.44E-01	1.61E+00	1.38E-01	-6.83E-01	1.00E+00
ENSBTAG00000007823	1.23E-01	1.00E+00	2.10E-01	9.25E-01	-5.04E-01	4.17E-01	2.03E-01	9.30E-01	-9.01E-01	3.85E-01
ENSBTAG00000007828	0.00E+00	1.00E+00	0.00E+00	1.00E+00	-5.13E-15	1.00E+00	0.00E+00	1.00E+00	0.00E+00	1.00E+00
ENSBTAG00000006869	4.33E-01	3.63E-01	9.38E-01	5.32E-05	1.10E+00	1.41E-06	8.94E-01	1.48E-04	-8.62E-03	1.00E+00
ENSBTAG00000017138	5.61E-01	7.40E-01	-6.11E-01	3.25E-01	-8.96E-01	9.51E-02	-4.86E-01	4.73E-01	-6.62E-01	6.50E-01
ENSBTAG00000010548	-6.47E-01	8.85E-01	-3.01E+00	4.74E-05	-2.14E+00	2.92E-03	-2.41E+00	9.45E-04	-4.34E-01	1.00E+00
ENSBTAG00000020667	2.43E-02	1.00E+00	4.84E-01	6.57E-01	7.22E-02	1.00E+00	6.68E-01	4.23E-01	-9.55E-01	7.22E-01
ENSBTAG00000034196	0.00E+00	1.00E+00	1.98E+00	4.43E-01	3.05E+00	1.36E-01	0.00E+00	1.00E+00	1.91E+00	8.00E-01
ENSBTAG00000007736	3.76E-01	1.00E+00	7.52E-02	1.00E+00	-1.77E-01	1.00E+00	7.09E-01	7.64E-01	-3.23E+00	4.34E-01
ENSBTAG00000047834	-8.71E-02	1.00E+00	-3.58E-01	3.14E-01	5.80E-01	4.63E-02	7.68E-01	6.04E-03	7.85E-02	1.00E+00
ENSBTAG00000007731	-3.22E-01	9.44E-01	-3.51E-02	1.00E+00	3.29E-01	5.12E-01	8.84E-01	1.19E-02	-5.92E-01	5.77E-01
ENSBTAG00000003838	1.92E+00	8.23E-01	0.00E+00	1.00E+00	-5.13E-15	1.00E+00	0.00E+00	1.00E+00	1.91E+00	8.00E-01
ENSBTAG00000014600	-1.19E+00	5.12E-01	-7.12E-01	4.90E-01	-9.61E-01	2.88E-01	3.93E-01	8.17E-01	-1.33E+00	4.52E-01
ENSBTAG00000003185	2.19E-01	9.43E-01	1.96E+00	1.26E-16	1.76E+00	8.45E-14	7.69E-01	2.65E-03	-7.58E-02	1.00E+00
ENSBTAG00000036942	0.00E+00	1.00E+00	2.79E+00	2.10E-01	1.78E+00	4.79E-01	0.00E+00	1.00E+00	0.00E+00	1.00E+00
ENSBTAG00000020801	2.69E-01	7.76E-01	1.18E+00	3.39E-08	1.18E+00	2.94E-08	7.53E-01	8.09E-04	-3.74E-01	5.10E-01
ENSBTAG00000017478	8.11E-01	1.00E+00	1.40E+00	5.16E-01	-1.25E-01	1.00E+00	-1.42E-01	1.00E+00	6.33E-03	1.00E+00
ENSBTAG00000047656	0.00E+00	1.00E+00	0.00E+00	1.00E+00	-5.13E-15	1.00E+00	0.00E+00	1.00E+00	0.00E+00	1.00E+00
ENSBTAG00000045200	0.00E+00	1.00E+00	0.00E+00	1.00E+00	-5.13E-15	1.00E+00	0.00E+00	1.00E+00	0.00E+00	1.00E+00
ENSBTAG00000037897	0.00E+00	1.00E+00	0.00E+00	1.00E+00	-5.13E-15	1.00E+00	2.53E+00	2.71E-01	0.00E+00	1.00E+00
ENSBTAG00000008409	4.76E+00	5.28E-19	4.49E+00	2.41E-18	3.35E+00	9.99E-12	2.12E+00	1.60E-05	-1.03E+00	3.04E-01
ENSBTAG00000045398	0.00E+00	1.00E+00	0.00E+00	1.00E+00	-5.13E-15	1.00E+00	0.00E+00	1.00E+00	0.00E+00	1.00E+00
ENSBTAG00000026172	-7.02E-01	3.69E-02	-1.61E-02	1.00E+00	-1.44E+00	2.70E-09	-1.25E+00	3.12E-07	7.45E-02	1.00E+00
ENSBTAG00000044438	0.00E+00	1.00E+00	0.00E+00	1.00E+00	-5.13E-15	1.00E+00	0.00E+00	1.00E+00	0.00E+00	1.00E+00
ENSBTAG00000044459	0.00E+00	1.00E+00	0.00E+00	1.00E+00	-5.13E-15	1.00E+00	0.00E+00	1.00E+00	0.00E+00	1.00E+00
ENSBTAG00000042082	0.00E+00	1.00E+00	0.00E+00	1.00E+00	-5.13E-15	1.00E+00	0.00E+00	1.00E+00	0.00E+00	1.00E+00
ENSBTAG00000023179	2.46E+00	9.88E-15	4.03E+00	5.87E-36	2.98E+00	5.40E-22	2.54E+00	1.66E-16	-1.89E-01	1.00E+00
ENSBTAG00000009394	3.74E-01	6.72E-01	2.51E+00	5.67E-21	1.52E+00	1.19E-08	7.66E-01	8.28E-03	-1.24E-01	1.00E+00
ENSBTAG00000010275	1.76E-01	1.00E+00	2.14E-01	5.41E-01	-3.51E-02	1.00E+00	-2.52E-01	4.51E-01	1.08E-01	1.00E+00
ENSBTAG00000005498	-2.15E-01	1.00E+00	-7.70E-01	1.40E-01	-1.18E+00	1.30E-02	-1.23E+00	1.01E-02	-5.99E-01	6.96E-01
ENSBTAG00000009072	-1.03E-01	1.00E+00	-1.51E+00	1.04E-08	-1.19E+00	4.76E-06	-1.00E+00	1.71E-04	9.55E-02	1.00E+00

ENSBTAG00000029569	0.00E+00	1.00E+00	0.00E+00	1.00E+00	-5.13E-15	1.00E+00	0.00E+00	1.00E+00	0.00E+00	1.00E+00
ENSBTAG00000020407	-6.44E-03	1.00E+00	-2.38E+00	4.82E-21	-2.39E+00	1.85E-21	-2.59E+00	1.83E-24	1.64E-01	1.00E+00
ENSBTAG00000020405	3.64E-02	1.00E+00	1.32E-01	9.06E-01	4.98E-02	1.00E+00	1.02E-01	1.00E+00	-3.12E-01	7.90E-01
ENSBTAG00000033446	3.85E-02	1.00E+00	9.00E-02	1.00E+00	-2.55E-01	4.25E-01	-5.78E-01	1.83E-02	-4.35E-01	3.94E-01
ENSBTAG00000020399	-2.25E-01	8.28E-01	-4.85E-01	3.89E-02	3.00E-01	2.61E-01	4.45E-01	6.55E-02	-2.29E-01	8.00E-01
ENSBTAG00000020398	-7.57E-01	1.49E-02	6.26E-01	1.00E-02	9.93E-01	1.09E-05	3.98E-01	1.52E-01	-1.64E-01	1.00E+00
ENSBTAG00000010813	-3.42E-01	9.80E-01	2.35E-01	8.49E-01	7.81E-02	1.00E+00	2.66E-01	7.76E-01	8.84E-02	1.00E+00
ENSBTAG00000022114	-9.51E-02	1.00E+00	-5.40E-01	5.45E-02	-2.36E-02	1.00E+00	1.53E-02	1.00E+00	-1.97E-01	9.80E-01
ENSBTAG00000013537	-1.37E-02	1.00E+00	-3.39E-01	5.41E-01	-9.89E-01	1.06E-02	-1.12E+00	3.92E-03	-1.02E-01	1.00E+00
ENSBTAG00000005025	1.22E-01	1.00E+00	1.32E+00	3.91E-07	1.43E+00	2.40E-08	1.39E+00	9.01E-08	1.47E-01	1.00E+00
ENSBTAG00000047212	2.23E-01	1.00E+00	-7.05E-01	2.74E-01	-9.36E-01	1.08E-01	-1.78E+00	1.18E-03	-2.16E-01	1.00E+00
ENSBTAG00000005585	-2.34E-01	1.00E+00	-7.93E-01	9.09E-02	-1.65E+00	7.86E-05	-2.29E+00	5.29E-08	-2.11E-01	1.00E+00
ENSBTAG00000045178	0.00E+00	1.00E+00	0.00E+00	1.00E+00	-5.13E-15	1.00E+00	0.00E+00	1.00E+00	0.00E+00	1.00E+00
ENSBTAG00000016754	-1.82E+00	4.54E-01	-1.73E+00	1.85E-01	-2.74E+00	3.24E-02	-1.99E+00	1.06E-01	-1.64E-01	1.00E+00
ENSBTAG00000044213	0.00E+00	1.00E+00	0.00E+00	1.00E+00	-5.13E-15	1.00E+00	0.00E+00	1.00E+00	0.00E+00	1.00E+00
ENSBTAG00000016194	-3.09E-01	1.00E+00	-7.28E-01	5.46E-01	9.17E-01	3.56E-01	1.97E+00	1.45E-02	1.38E+00	4.31E-01
ENSBTAG00000009916	-8.90E-02	1.00E+00	-4.19E-01	1.23E-01	3.04E-03	1.00E+00	-2.96E-02	1.00E+00	-2.19E-01	8.85E-01
ENSBTAG00000002002	-6.76E-01	3.91E-01	-1.46E+00	1.58E-04	-6.23E-01	1.67E-01	-1.40E+00	3.52E-04	-5.43E-01	6.28E-01
ENSBTAG00000015334	-5.04E-02	1.00E+00	-2.64E-01	3.74E-01	-6.41E-01	4.43E-03	-8.37E-01	1.52E-04	-3.52E-01	5.49E-01
ENSBTAG00000015319	-1.47E-01	1.00E+00	-1.64E-01	7.60E-01	2.12E-01	5.76E-01	-2.53E-01	4.83E-01	-1.57E-02	1.00E+00
ENSBTAG00000005877	9.57E-01	8.23E-01	1.40E+00	1.48E-01	6.16E-01	7.31E-01	-2.04E-01	1.00E+00	-3.08E-01	1.00E+00
ENSBTAG00000021156	-1.17E-01	1.00E+00	-7.17E-01	1.57E-02	-3.90E-01	2.64E-01	-7.18E-01	1.72E-02	-6.47E-01	2.11E-01
ENSBTAG00000020693	2.08E-01	1.00E+00	1.37E+00	5.10E-07	1.46E+00	7.21E-08	1.40E+00	3.77E-07	2.19E-01	9.73E-01
ENSBTAG00000026309	-1.88E-01	1.00E+00	-5.50E-01	1.61E-01	8.43E-02	1.00E+00	-2.24E-01	7.54E-01	2.67E-01	9.52E-01
ENSBTAG00000043938	0.00E+00	1.00E+00	0.00E+00	1.00E+00	-5.13E-15	1.00E+00	0.00E+00	1.00E+00	0.00E+00	1.00E+00
ENSBTAG00000044730	0.00E+00	1.00E+00	0.00E+00	1.00E+00	-5.13E-15	1.00E+00	0.00E+00	1.00E+00	0.00E+00	1.00E+00
ENSBTAG00000019892	2.64E+00	1.48E-01	1.26E+00	4.54E-01	2.15E+00	9.96E-02	4.38E+00	1.91E-04	-2.01E+00	7.29E-01
ENSBTAG00000009474	1.92E+00	8.23E-01	0.00E+00	1.00E+00	-5.13E-15	1.00E+00	0.00E+00	1.00E+00	0.00E+00	1.00E+00
ENSBTAG00000040351	7.85E-01	8.65E-01	3.46E+00	1.06E-07	3.51E+00	4.91E-08	2.60E+00	1.72E-04	2.03E+00	6.81E-02
ENSBTAG00000046307	2.66E+00	9.62E-13	2.46E+00	4.89E-12	5.25E-01	2.31E-01	6.79E-01	9.98E-02	-3.19E-01	9.02E-01
ENSBTAG00000044106	1.61E+00	2.38E-03	3.98E+00	6.29E-18	3.61E+00	1.66E-15	2.58E+00	7.12E-09	6.01E-01	6.70E-01
ENSBTAG00000017016	-1.54E-01	1.00E+00	-1.78E-01	8.39E-01	3.88E-02	1.00E+00	-5.14E-01	1.60E-01	-5.90E-01	4.05E-01
ENSBTAG00000017019	3.34E-01	7.02E-01	3.54E-01	2.64E-01	1.88E-01	6.86E-01	1.74E-01	7.49E-01	5.36E-01	2.95E-01
ENSBTAG00000046325	-1.32E-01	1.00E+00	-4.91E-01	2.88E-01	-3.07E-01	5.88E-01	-2.29E-01	7.86E-01	1.48E-01	1.00E+00
ENSBTAG00000023218	2.92E-01	5.76E-01	1.17E+00	2.42E-10	1.07E+00	6.59E-09	7.08E-01	2.71E-04	-2.30E-01	8.00E-01

ENSBTAG00000008693	1.92E+00	8.23E-01	0.00E+00	1.00E+00	3.06E+00	1.36E-01	0.00E+00	1.00E+00	0.00E+00	1.00E+00
ENSBTAG00000013227	-7.05E-01	4.77E-01	-1.99E+00	4.89E-06	-8.46E-01	7.68E-02	-1.35E-01	1.00E+00	-2.59E-01	1.00E+00
ENSBTAG00000038286	-6.36E-01	7.02E-01	-4.25E+00	3.00E-16	-6.17E+00	1.45E-27	-3.15E+00	3.33E-10	-3.67E-02	1.00E+00
ENSBTAG00000027760	3.00E-01	8.23E-01	1.95E-01	7.11E-01	6.35E-01	2.13E-02	-3.21E-02	1.00E+00	-2.34E-01	9.31E-01
ENSBTAG00000002448	-6.95E-01	7.45E-01	5.24E-02	1.00E+00	8.57E-02	1.00E+00	-5.93E-03	1.00E+00	1.77E-02	1.00E+00
ENSBTAG00000044404	0.00E+00	1.00E+00	0.00E+00	1.00E+00	-5.13E-15	1.00E+00	0.00E+00	1.00E+00	0.00E+00	1.00E+00
ENSBTAG00000044616	0.00E+00	1.00E+00	0.00E+00	1.00E+00	-5.13E-15	1.00E+00	0.00E+00	1.00E+00	0.00E+00	1.00E+00
ENSBTAG00000043686	0.00E+00	1.00E+00	0.00E+00	1.00E+00	-5.13E-15	1.00E+00	0.00E+00	1.00E+00	0.00E+00	1.00E+00
ENSBTAG00000017492	7.29E-02	1.00E+00	-5.08E-01	1.91E-02	-1.09E+00	4.01E-08	-1.15E+00	9.79E-09	-8.95E-02	1.00E+00
ENSBTAG00000005560	1.53E-01	1.00E+00	-6.35E-02	1.00E+00	-2.46E-02	1.00E+00	-2.01E-01	1.00E+00	-7.85E-01	9.02E-01
ENSBTAG00000026306	1.89E-01	1.00E+00	8.14E-01	7.55E-04	1.24E+00	5.28E-08	1.04E+00	9.37E-06	1.17E-01	1.00E+00
ENSBTAG00000000878	-2.22E-01	7.86E-01	6.97E-02	1.00E+00	3.36E-01	1.12E-01	7.63E-02	9.94E-01	2.64E-01	6.29E-01
ENSBTAG00000042577	0.00E+00	1.00E+00	0.00E+00	1.00E+00	-5.13E-15	1.00E+00	0.00E+00	1.00E+00	0.00E+00	1.00E+00
ENSBTAG00000016159	0.00E+00	1.00E+00	0.00E+00	1.00E+00	1.78E+00	4.79E-01	0.00E+00	1.00E+00	0.00E+00	1.00E+00
ENSBTAG00000000914	-5.01E-01	1.00E+00	-4.25E-01	1.00E+00	-1.63E-01	1.00E+00	-6.84E-01	8.84E-01	-1.30E+00	9.00E-01
ENSBTAG00000003450	-1.61E-01	1.00E+00	1.36E-01	8.35E-01	-1.62E-01	7.22E-01	-1.84E-01	6.54E-01	5.85E-03	1.00E+00
ENSBTAG00000003454	6.03E-01	6.85E-01	5.46E-01	3.75E-01	-6.15E-01	3.31E-01	-1.57E-01	1.00E+00	-5.06E-01	8.08E-01
ENSBTAG00000003460	9.10E-02	1.00E+00	1.32E+00	2.06E-07	1.28E+00	4.43E-07	8.57E-01	1.35E-03	6.99E-02	1.00E+00
ENSBTAG00000004243	-3.99E-01	3.72E-01	-7.32E-01	9.14E-04	-1.05E+00	7.93E-07	-9.67E-01	8.26E-06	-8.06E-01	5.33E-03
ENSBTAG00000001174	-9.72E-02	1.00E+00	1.63E+00	4.26E-11	1.95E+00	2.27E-15	1.66E+00	2.60E-11	-7.43E-01	6.48E-02
ENSBTAG00000033627	0.00E+00	1.00E+00	0.00E+00	1.00E+00	1.78E+00	4.79E-01	0.00E+00	1.00E+00	0.00E+00	1.00E+00
ENSBTAG00000022539	2.35E-01	9.19E-01	1.94E+00	4.94E-16	1.65E+00	4.49E-12	7.19E-01	6.39E-03	-3.19E-02	1.00E+00
ENSBTAG00000005748	6.17E-01	8.23E-01	1.58E+00	6.93E-03	1.93E+00	6.06E-04	2.17E+00	1.09E-04	-9.72E-02	1.00E+00
ENSBTAG00000011203	1.30E+00	9.32E-01	-1.91E+00	4.69E-01	6.39E-01	9.79E-01	-1.48E-01	1.00E+00	-1.91E+00	8.00E-01
ENSBTAG00000047303	1.12E+00	7.80E-01	3.91E-01	1.00E+00	7.70E-01	6.28E-01	1.61E+00	9.70E-02	3.16E-01	1.00E+00
ENSBTAG00000044050	6.70E-01	8.23E-01	9.78E-02	1.00E+00	6.51E-02	1.00E+00	8.19E-01	3.04E-01	3.11E-01	1.00E+00
ENSBTAG00000005893	1.10E-01	1.00E+00	3.08E-02	1.00E+00	8.39E-01	6.32E-05	8.41E-01	7.79E-05	2.64E-01	7.72E-01
ENSBTAG00000005898	4.14E-01	4.32E-01	1.71E+00	1.17E-13	1.67E+00	3.00E-13	9.40E-01	9.39E-05	6.83E-03	1.00E+00
ENSBTAG00000020034	4.42E-01	1.93E-01	9.31E-01	2.40E-06	1.10E+00	1.54E-08	9.44E-01	2.03E-06	1.63E-01	9.66E-01
ENSBTAG00000019147	5.89E-03	1.00E+00	3.34E-02	1.00E+00	3.00E-02	1.00E+00	2.44E-01	4.39E-01	1.43E-02	1.00E+00
ENSBTAG00000045097	0.00E+00	1.00E+00	0.00E+00	1.00E+00	-5.13E-15	1.00E+00	0.00E+00	1.00E+00	0.00E+00	1.00E+00
ENSBTAG00000028889	0.00E+00	1.00E+00	0.00E+00	1.00E+00	-5.13E-15	1.00E+00	0.00E+00	1.00E+00	0.00E+00	1.00E+00
ENSBTAG00000019145	-1.91E+00	8.23E-01	-1.91E+00	4.69E-01	-1.91E+00	4.28E-01	-1.91E+00	4.39E-01	-1.91E+00	8.00E-01
ENSBTAG00000004022	7.10E-01	8.24E-01	-2.26E+00	2.66E-02	-1.23E+00	2.06E-01	-4.96E-02	1.00E+00	4.30E-01	1.00E+00
ENSBTAG00000033284	-2.96E-02	1.00E+00	2.67E-01	4.43E-01	3.71E-01	2.17E-01	-1.28E-01	8.99E-01	4.99E-03	1.00E+00

ENSBTAG00000039031	0.00E+00	1.00E+00	0.00E+00	1.00E+00	-5.13E-15	1.00E+00	0.00E+00	1.00E+00	0.00E+00	1.00E+00
ENSBTAG00000018570	7.21E-01	9.23E-01	9.24E-02	1.00E+00	-1.10E+00	4.15E-01	-1.13E+00	4.05E-01	-3.89E-01	1.00E+00
ENSBTAG00000040321	4.75E-01	1.00E+00	3.45E-01	1.00E+00	-2.11E+00	1.73E-01	-8.52E-01	6.65E-01	-2.95E-01	1.00E+00
ENSBTAG00000004924	8.03E-01	1.00E+00	-1.91E+00	4.69E-01	1.14E+00	6.46E-01	6.21E-01	9.94E-01	-1.91E+00	8.00E-01
ENSBTAG00000043923	0.00E+00	1.00E+00	0.00E+00	1.00E+00	-5.13E-15	1.00E+00	2.53E+00	2.71E-01	0.00E+00	1.00E+00
ENSBTAG00000015637	5.38E-02	1.00E+00	5.85E-01	7.45E-02	1.15E+00	7.98E-05	1.47E+00	3.56E-07	4.14E-01	6.45E-01
ENSBTAG00000011933	-4.58E-02	1.00E+00	-3.06E-01	6.97E-01	1.09E+00	5.46E-03	9.67E-01	1.81E-02	5.82E-01	6.01E-01
ENSBTAG00000047136	-1.38E-01	1.00E+00	-1.30E-01	8.39E-01	-4.20E-01	9.41E-02	-1.05E-01	9.37E-01	-4.35E-01	3.66E-01
ENSBTAG00000009138	-4.09E-01	7.64E-01	-6.46E-01	8.59E-02	1.63E-01	8.99E-01	1.09E+00	8.00E-04	3.88E-01	7.82E-01
ENSBTAG00000005287	-2.79E+00	4.57E-21	-5.90E+00	4.23E-70	-7.20E+00	6.38E-91	-5.24E+00	3.78E-60	2.83E-01	8.42E-01
ENSBTAG00000033622	-7.87E-01	1.00E+00	8.86E-02	1.00E+00	-2.70E+00	1.99E-01	-2.70E+00	2.05E-01	7.02E-03	1.00E+00
ENSBTAG00000019910	6.50E-01	3.01E-02	1.66E+00	1.86E-14	1.25E+00	6.84E-09	1.01E+00	5.38E-06	4.95E-02	1.00E+00
ENSBTAG00000008958	3.06E-02	1.00E+00	9.88E-02	9.75E-01	1.24E+00	1.11E-08	1.21E+00	3.44E-08	2.04E-01	9.06E-01
ENSBTAG00000004954	5.29E-01	9.43E-01	3.73E-01	8.08E-01	4.60E-01	6.66E-01	-1.11E-01	1.00E+00	-3.36E-01	1.00E+00
ENSBTAG00000017529	-1.84E-01	1.00E+00	-1.64E+00	6.88E-02	-2.26E+00	8.95E-03	-8.74E-01	4.39E-01	-6.10E-01	9.85E-01
ENSBTAG00000000948	2.11E-01	9.04E-01	4.53E-01	6.76E-02	5.33E-01	2.52E-02	5.42E-01	2.57E-02	-1.52E-01	1.00E+00
ENSBTAG00000021841	1.12E-01	1.00E+00	3.11E-01	4.40E-01	1.06E+00	1.30E-04	1.21E+00	1.26E-05	-8.71E-02	1.00E+00
ENSBTAG00000045005	0.00E+00	1.00E+00	0.00E+00	1.00E+00	-5.13E-15	1.00E+00	0.00E+00	1.00E+00	0.00E+00	1.00E+00
ENSBTAG00000043978	0.00E+00	1.00E+00	0.00E+00	1.00E+00	-5.13E-15	1.00E+00	0.00E+00	1.00E+00	0.00E+00	1.00E+00
ENSBTAG00000026283	8.74E-02	1.00E+00	1.54E-01	8.56E-01	-3.40E-02	1.00E+00	1.63E-01	8.31E-01	5.06E-01	4.31E-01
ENSBTAG00000044415	0.00E+00	1.00E+00	0.00E+00	1.00E+00	-5.13E-15	1.00E+00	0.00E+00	1.00E+00	0.00E+00	1.00E+00
ENSBTAG00000014045	0.00E+00	1.00E+00	0.00E+00	1.00E+00	-5.13E-15	1.00E+00	0.00E+00	1.00E+00	0.00E+00	1.00E+00
ENSBTAG00000045031	0.00E+00	1.00E+00	0.00E+00	1.00E+00	-5.13E-15	1.00E+00	0.00E+00	1.00E+00	0.00E+00	1.00E+00
ENSBTAG00000007534	-4.12E-01	4.98E-01	-1.29E+00	2.58E-07	-1.97E+00	2.17E-15	-2.11E+00	3.56E-17	1.75E-01	1.00E+00
ENSBTAG00000022471	-1.75E-01	1.00E+00	-2.35E+00	1.39E-10	-2.78E+00	4.33E-14	-1.85E+00	4.44E-07	3.14E-01	9.20E-01
ENSBTAG00000018557	1.10E-01	1.00E+00	4.27E-01	2.88E-02	5.70E-01	2.03E-03	2.62E-01	2.59E-01	-8.93E-02	1.00E+00
ENSBTAG00000046964	0.00E+00	1.00E+00	0.00E+00	1.00E+00	-5.13E-15	1.00E+00	0.00E+00	1.00E+00	0.00E+00	1.00E+00
ENSBTAG00000036421	0.00E+00	1.00E+00	0.00E+00	1.00E+00	-5.13E-15	1.00E+00	0.00E+00	1.00E+00	0.00E+00	1.00E+00
ENSBTAG00000033173	-5.02E-01	1.00E+00	-1.17E+00	3.33E-01	-4.92E-01	8.02E-01	-5.21E-01	7.80E-01	-2.14E+00	3.15E-01
ENSBTAG00000046203	0.00E+00	1.00E+00	0.00E+00	1.00E+00	-5.13E-15	1.00E+00	0.00E+00	1.00E+00	0.00E+00	1.00E+00
ENSBTAG00000001299	4.21E-01	5.67E-01	8.00E-01	4.52E-03	3.65E-01	2.96E-01	5.80E-01	5.85E-02	9.72E-02	1.00E+00
ENSBTAG00000044542	0.00E+00	1.00E+00	0.00E+00	1.00E+00	-5.13E-15	1.00E+00	0.00E+00	1.00E+00	0.00E+00	1.00E+00
ENSBTAG00000044711	0.00E+00	1.00E+00	0.00E+00	1.00E+00	-5.13E-15	1.00E+00	0.00E+00	1.00E+00	0.00E+00	1.00E+00
ENSBTAG00000042153	0.00E+00	1.00E+00	0.00E+00	1.00E+00	-5.13E-15	1.00E+00	0.00E+00	1.00E+00	0.00E+00	1.00E+00
ENSBTAG00000015901	2.52E-01	7.44E-01	1.29E+00	9.40E-12	1.14E+00	1.90E-09	8.59E-01	1.10E-05	-1.45E-01	1.00E+00

ENSBTAG00000008629	-6.20E-02	1.00E+00	5.99E-01	1.61E-03	1.03E+00	8.72E-09	9.09E-01	6.60E-07	-1.49E-01	9.73E-01
ENSBTAG00000011614	2.74E-02	1.00E+00	-5.30E-01	2.21E-01	2.49E-02	1.00E+00	-4.71E-02	1.00E+00	-1.28E-01	1.00E+00
ENSBTAG00000042250	0.00E+00	1.00E+00	0.00E+00	1.00E+00	-5.13E-15	1.00E+00	0.00E+00	1.00E+00	0.00E+00	1.00E+00
ENSBTAG00000021821	0.00E+00	1.00E+00	0.00E+00	1.00E+00	-5.13E-15	1.00E+00	0.00E+00	1.00E+00	0.00E+00	1.00E+00
ENSBTAG00000015229	3.44E-01	1.00E+00	5.26E-01	8.52E-01	3.94E-01	9.70E-01	1.16E+00	2.98E-01	-6.63E-01	1.00E+00
ENSBTAG00000021009	-5.02E-01	1.00E+00	4.66E-01	9.95E-01	-3.21E+00	9.44E-02	1.12E+00	4.39E-01	6.69E-01	1.00E+00
ENSBTAG00000033128	1.91E+00	8.23E-01	0.00E+00	1.00E+00	-5.13E-15	1.00E+00	0.00E+00	1.00E+00	0.00E+00	1.00E+00
ENSBTAG00000040021	0.00E+00	1.00E+00	0.00E+00	1.00E+00	-5.13E-15	1.00E+00	1.76E+00	4.97E-01	0.00E+00	1.00E+00
ENSBTAG00000016716	8.25E-01	6.14E-04	2.39E+00	1.02E-32	2.51E+00	4.65E-36	1.81E+00	1.48E-19	-6.20E-01	6.53E-02
ENSBTAG00000004476	2.41E-02	1.00E+00	-7.26E-01	1.26E-03	-1.79E+00	6.94E-17	-2.64E+00	6.06E-33	1.60E-01	1.00E+00
ENSBTAG00000002192	-6.28E-01	9.61E-01	-5.41E-01	7.02E-01	-1.15E+00	2.20E-01	-3.36E-01	9.24E-01	-1.43E+00	4.71E-01
ENSBTAG00000042834	0.00E+00	1.00E+00	0.00E+00	1.00E+00	-5.13E-15	1.00E+00	0.00E+00	1.00E+00	0.00E+00	1.00E+00
ENSBTAG00000011981	1.73E-01	1.00E+00	-9.04E-01	2.35E-02	-1.50E+00	7.84E-05	-1.06E+00	6.41E-03	-8.39E-01	2.27E-01
ENSBTAG00000006695	5.46E-02	1.00E+00	1.31E+00	4.20E-05	1.43E+00	6.40E-06	1.27E+00	9.32E-05	3.35E-01	8.06E-01
ENSBTAG00000013284	-3.22E-01	6.19E-01	-3.61E-01	1.87E-01	2.09E-01	5.28E-01	4.07E-01	1.15E-01	-5.08E-01	2.49E-01
ENSBTAG00000000620	-1.66E-01	9.86E-01	-5.38E-01	1.12E-02	-9.62E-01	1.20E-06	-8.79E-01	1.31E-05	9.80E-02	1.00E+00
ENSBTAG00000043234	9.48E-01	6.00E-01	1.05E+00	1.81E-01	5.32E-01	6.49E-01	7.66E-02	1.00E+00	-4.36E-01	1.00E+00
ENSBTAG00000011205	2.44E+00	2.92E-01	2.72E+00	3.76E-02	3.57E+00	6.39E-04	3.77E+00	2.26E-04	1.31E+00	8.98E-01
ENSBTAG00000028700	0.00E+00	1.00E+00	0.00E+00	1.00E+00	-5.13E-15	1.00E+00	0.00E+00	1.00E+00	0.00E+00	1.00E+00
ENSBTAG00000002784	4.19E-02	1.00E+00	1.04E-01	9.18E-01	1.04E-02	1.00E+00	4.69E-02	1.00E+00	-3.09E-01	6.07E-01
ENSBTAG00000014689	2.46E-01	8.23E-01	-8.37E-01	2.04E-04	-2.45E-01	4.28E-01	-1.34E-01	8.24E-01	2.70E-01	7.79E-01
ENSBTAG00000014691	8.53E-02	1.00E+00	7.00E-02	1.00E+00	2.06E-01	4.40E-01	4.31E-01	4.10E-02	-1.28E-01	1.00E+00
ENSBTAG00000044080	1.84E+00	8.83E-02	2.55E+00	2.74E-04	2.22E+00	1.87E-03	9.65E-01	3.49E-01	-1.09E+00	8.00E-01
ENSBTAG00000022169	6.39E-02	1.00E+00	-9.80E-01	2.90E-02	-8.86E-01	4.65E-02	-3.61E-01	5.60E-01	-2.76E-01	1.00E+00
ENSBTAG00000022588	7.69E-01	8.23E-01	7.37E-01	4.46E-01	-1.60E-01	1.00E+00	8.75E-01	3.22E-01	-3.23E-01	1.00E+00
ENSBTAG00000004720	-8.63E-01	8.23E-01	1.93E-01	1.00E+00	1.59E-01	1.00E+00	4.55E-02	1.00E+00	-1.04E+00	7.19E-01
ENSBTAG00000037399	-7.78E-01	1.00E+00	8.82E-02	1.00E+00	3.56E-01	1.00E+00	3.28E-01	1.00E+00	1.89E-02	1.00E+00
ENSBTAG00000032881	3.90E-01	9.24E-01	5.44E-01	3.23E-01	2.44E-02	1.00E+00	5.78E-01	2.75E-01	-6.91E-02	1.00E+00
ENSBTAG00000044870	0.00E+00	1.00E+00	0.00E+00	1.00E+00	-5.13E-15	1.00E+00	0.00E+00	1.00E+00	0.00E+00	1.00E+00
ENSBTAG00000000060	3.22E+00	3.75E-01	3.30E+00	1.03E-01	2.55E+00	2.55E-01	4.32E+00	9.46E-03	1.93E+00	8.00E-01
ENSBTAG00000020312	6.81E-02	1.00E+00	2.14E-01	8.81E-01	4.88E-01	3.62E-01	8.76E-01	5.01E-02	5.98E-01	6.01E-01
ENSBTAG00000046617	1.92E+00	8.23E-01	0.00E+00	1.00E+00	-5.13E-15	1.00E+00	0.00E+00	1.00E+00	0.00E+00	1.00E+00
ENSBTAG00000042529	0.00E+00	1.00E+00	0.00E+00	1.00E+00	-5.13E-15	1.00E+00	0.00E+00	1.00E+00	0.00E+00	1.00E+00
ENSBTAG00000007562	5.91E-01	2.06E-01	1.34E+00	7.35E-07	1.61E+00	1.82E-09	1.87E+00	3.36E-12	7.13E-01	1.19E-01
ENSBTAG00000001808	-5.11E-02	1.00E+00	-7.74E-01	1.59E-03	-1.56E+00	2.54E-11	-1.25E+00	1.40E-07	-4.55E-01	4.04E-01

ENSBTAG00000048138	-2.51E-01	1.00E+00	-2.64E+00	1.51E-07	-3.12E+00	6.74E-10	-3.05E+00	1.52E-09	-8.84E-01	3.32E-01
ENSBTAG00000043212	0.00E+00	1.00E+00	0.00E+00	1.00E+00	-5.13E-15	1.00E+00	0.00E+00	1.00E+00	0.00E+00	1.00E+00
ENSBTAG00000011298	-4.00E-01	8.90E-01	-6.69E-01	1.94E-01	-7.57E-01	1.18E-01	-5.05E-01	3.72E-01	-2.83E-01	1.00E+00
ENSBTAG00000005404	2.13E+00	4.35E-02	4.73E+00	4.63E-10	4.38E+00	5.57E-09	2.93E+00	1.33E-04	-2.41E-01	1.00E+00
ENSBTAG00000002062	5.70E-01	2.61E-01	2.48E+00	1.94E-19	2.29E+00	4.59E-17	1.74E+00	2.32E-10	-3.38E-01	7.96E-01
ENSBTAG00000048040	0.00E+00	1.00E+00	0.00E+00	1.00E+00	-5.13E-15	1.00E+00	0.00E+00	1.00E+00	0.00E+00	1.00E+00
ENSBTAG00000040496	4.61E-01	9.59E-01	-2.51E-01	9.61E-01	-3.75E-01	7.54E-01	7.01E-01	3.09E-01	-5.55E-04	1.00E+00
ENSBTAG00000046847	0.00E+00	1.00E+00	0.00E+00	1.00E+00	-5.13E-15	1.00E+00	0.00E+00	1.00E+00	0.00E+00	1.00E+00
ENSBTAG00000043614	0.00E+00	1.00E+00	0.00E+00	1.00E+00	-5.13E-15	1.00E+00	0.00E+00	1.00E+00	0.00E+00	1.00E+00
ENSBTAG00000032982	3.06E-02	1.00E+00	-3.19E-01	2.31E-01	4.39E-01	6.16E-02	4.10E-01	9.46E-02	-1.76E-01	9.54E-01
ENSBTAG00000017249	-5.73E-02	1.00E+00	-8.93E-01	4.43E-01	-4.88E-01	8.02E-01	7.15E-02	1.00E+00	-3.91E-01	1.00E+00
ENSBTAG00000037470	-5.77E-02	1.00E+00	-6.28E-01	5.11E-02	-9.67E-01	1.19E-03	-7.02E-01	2.77E-02	-1.05E+00	8.93E-03
ENSBTAG00000020139	-2.79E-02	1.00E+00	3.75E-01	2.67E-01	6.37E-01	2.65E-02	6.81E-01	1.85E-02	-3.38E-01	7.60E-01
ENSBTAG00000020143	-4.81E-01	7.64E-01	-8.35E-01	5.01E-02	5.21E-01	2.89E-01	6.01E-02	1.00E+00	2.10E-01	1.00E+00
ENSBTAG00000047607	0.00E+00	1.00E+00	0.00E+00	1.00E+00	-5.13E-15	1.00E+00	0.00E+00	1.00E+00	0.00E+00	1.00E+00
ENSBTAG00000002076	-4.89E-02	1.00E+00	-2.43E-01	6.74E-01	-1.17E+00	3.43E-04	-2.67E-02	1.00E+00	-1.26E-02	1.00E+00
ENSBTAG00000045269	0.00E+00	1.00E+00	0.00E+00	1.00E+00	-5.13E-15	1.00E+00	0.00E+00	1.00E+00	0.00E+00	1.00E+00
ENSBTAG00000045113	-1.29E+00	9.36E-01	9.26E-02	1.00E+00	-3.21E+00	9.50E-02	-1.44E+00	4.97E-01	-1.29E+00	9.02E-01
ENSBTAG00000008260	3.12E-01	6.22E-01	8.87E-01	4.11E-05	9.07E-01	2.31E-05	7.36E-01	1.00E-03	-1.34E-01	1.00E+00
ENSBTAG00000002196	1.11E-01	1.00E+00	1.23E+00	5.60E-06	1.36E+00	4.24E-07	9.25E-01	1.03E-03	-2.89E-01	8.02E-01
ENSBTAG00000012920	-2.81E-01	1.00E+00	2.48E-02	1.00E+00	2.82E-01	6.83E-01	1.47E-01	1.00E+00	-3.86E-01	8.19E-01
ENSBTAG00000008864	7.44E-03	1.00E+00	-2.62E-01	5.99E-01	-5.86E-01	9.05E-02	-5.33E-01	1.40E-01	-3.91E-01	7.44E-01
ENSBTAG00000042485	0.00E+00	1.00E+00	0.00E+00	1.00E+00	-5.13E-15	1.00E+00	0.00E+00	1.00E+00	0.00E+00	1.00E+00
ENSBTAG00000032944	-6.97E-01	9.82E-01	-8.74E-01	4.91E-01	-2.30E+00	3.86E-02	-2.32E+00	4.03E-02	-2.32E-02	1.00E+00
ENSBTAG00000005559	4.61E-03	1.00E+00	-2.70E+00	2.42E-01	1.01E+00	5.96E-01	-1.65E-01	1.00E+00	6.81E-03	1.00E+00
ENSBTAG00000008842	-1.37E-01	1.00E+00	-3.97E-02	1.00E+00	1.52E+00	1.77E-05	1.26E+00	6.78E-04	-4.34E-01	8.00E-01
ENSBTAG00000012608	6.54E-01	6.94E-01	6.81E-01	2.77E-01	1.06E+00	3.60E-02	1.40E+00	2.86E-03	7.24E-02	1.00E+00
ENSBTAG00000019587	5.68E-01	1.00E+00	1.25E+00	1.46E-01	5.17E-01	7.45E-01	1.75E+00	2.17E-02	-1.09E-01	1.00E+00
ENSBTAG00000004411	-6.62E-03	1.00E+00	8.33E-01	6.07E-01	1.25E-01	1.00E+00	-2.05E-01	1.00E+00	1.08E+00	8.00E-01
ENSBTAG00000047574	-5.04E-01	1.00E+00	1.16E-01	1.00E+00	9.31E-01	1.77E-01	9.66E-01	1.63E-01	-9.60E-01	7.26E-01
ENSBTAG00000008280	1.12E+00	1.30E-04	2.13E+00	8.78E-17	1.21E-02	1.00E+00	-7.14E-02	1.00E+00	5.48E-01	3.09E-01
ENSBTAG00000042995	0.00E+00	1.00E+00	0.00E+00	1.00E+00	-5.13E-15	1.00E+00	0.00E+00	1.00E+00	0.00E+00	1.00E+00
ENSBTAG00000033268	1.63E-01	1.00E+00	-1.55E+00	1.53E-04	-2.78E+00	2.29E-11	-2.23E+00	4.94E-08	1.75E-01	1.00E+00
ENSBTAG00000047355	1.91E+00	8.23E-01	0.00E+00	1.00E+00	-5.13E-15	1.00E+00	1.76E+00	4.97E-01	0.00E+00	1.00E+00
ENSBTAG00000003885	5.98E-01	6.11E-02	8.97E-01	5.56E-05	2.84E-01	3.37E-01	3.29E-01	2.47E-01	2.16E-01	8.61E-01

ENSBTAG00000032831	6.39E-02	1.00E+00	2.16E-01	6.71E-01	3.19E-01	4.09E-01	1.83E-01	7.77E-01	-5.35E-01	4.20E-01
ENSBTAG00000039499	-5.12E-01	9.43E-01	7.81E-01	1.99E-01	6.56E-01	3.01E-01	7.03E-01	2.64E-01	-1.32E-01	1.00E+00
ENSBTAG00000047079	0.00E+00	1.00E+00	0.00E+00	1.00E+00	-5.13E-15	1.00E+00	0.00E+00	1.00E+00	0.00E+00	1.00E+00
ENSBTAG00000017169	2.55E-02	1.00E+00	-4.64E-01	2.95E-01	-3.60E-01	4.54E-01	-5.41E-01	1.99E-01	7.39E-02	1.00E+00
ENSBTAG00000045131	0.00E+00	1.00E+00	0.00E+00	1.00E+00	-5.13E-15	1.00E+00	0.00E+00	1.00E+00	0.00E+00	1.00E+00
ENSBTAG00000009539	-1.30E-01	1.00E+00	-1.06E+00	1.36E-02	-2.08E+00	3.80E-07	-1.23E+00	4.11E-03	-1.95E-01	1.00E+00
ENSBTAG00000008150	1.72E-01	1.00E+00	-1.88E+00	9.52E-04	-7.01E-01	3.11E-01	9.86E-01	1.11E-01	2.93E-01	1.00E+00
ENSBTAG00000018902	-1.78E-01	1.00E+00	-7.48E-02	1.00E+00	4.36E-01	2.20E-01	5.08E-01	1.39E-01	-1.08E-02	1.00E+00
ENSBTAG00000016284	2.25E+00	2.41E-07	3.55E+00	1.49E-17	1.32E+00	1.38E-03	4.40E-01	4.53E-01	5.35E-01	7.09E-01
ENSBTAG00000042141	0.00E+00	1.00E+00	0.00E+00	1.00E+00	-5.13E-15	1.00E+00	0.00E+00	1.00E+00	0.00E+00	1.00E+00
ENSBTAG00000020099	2.31E-01	1.00E+00	-6.50E-01	3.54E-01	-2.40E+00	7.70E-05	-2.04E+00	6.71E-04	2.68E-01	1.00E+00
ENSBTAG00000032812	-7.12E-02	1.00E+00	-1.55E-01	1.00E+00	-3.33E-01	8.42E-01	-2.28E-01	1.00E+00	-1.33E-01	1.00E+00
ENSBTAG00000015717	2.81E-01	1.00E+00	8.09E-02	1.00E+00	1.38E+00	2.55E-04	1.39E+00	2.71E-04	3.37E-01	9.62E-01
ENSBTAG00000001302	2.94E-02	1.00E+00	-1.50E-01	7.70E-01	-1.26E-01	8.55E-01	-2.34E-01	4.88E-01	-1.16E-01	1.00E+00
ENSBTAG00000007335	8.75E-02	1.00E+00	7.09E-01	1.97E-04	2.59E-01	2.86E-01	4.27E-02	1.00E+00	-3.01E-01	5.79E-01
ENSBTAG00000003339	3.02E-01	8.95E-01	-2.98E-01	5.53E-01	1.36E+00	6.65E-06	1.09E+00	5.24E-04	-9.87E-02	1.00E+00
ENSBTAG00000021743	3.88E-01	9.32E-01	-1.40E+00	4.84E-03	-3.24E+00	1.53E-09	-2.12E+00	1.94E-05	3.55E-01	9.49E-01
ENSBTAG00000014026	8.09E-01	1.95E-01	5.74E-01	2.18E-01	-2.97E-01	6.94E-01	-5.17E-02	1.00E+00	1.17E+00	2.28E-02
ENSBTAG00000047330	2.39E-01	1.00E+00	-4.13E-01	5.56E-01	5.30E-02	1.00E+00	2.10E-01	9.10E-01	1.89E-01	1.00E+00
ENSBTAG00000000071	0.00E+00	1.00E+00	0.00E+00	1.00E+00	-5.13E-15	1.00E+00	0.00E+00	1.00E+00	0.00E+00	1.00E+00
ENSBTAG00000039718	0.00E+00	1.00E+00	0.00E+00	1.00E+00	-5.13E-15	1.00E+00	0.00E+00	1.00E+00	0.00E+00	1.00E+00
ENSBTAG00000037526	-5.26E-01	1.00E+00	-6.04E-01	8.50E-01	-2.99E+00	1.92E-02	-1.84E+00	1.65E-01	4.13E-01	1.00E+00
ENSBTAG00000047661	1.92E+00	8.23E-01	1.99E+00	4.43E-01	-5.13E-15	1.00E+00	3.03E+00	1.50E-01	0.00E+00	1.00E+00
ENSBTAG00000002080	-4.82E-01	8.81E-01	-8.07E-02	1.00E+00	-1.17E+00	3.41E-02	-1.35E+00	1.51E-02	-4.61E-01	8.82E-01
ENSBTAG00000011779	-2.72E-01	9.91E-01	-2.39E+00	6.76E-13	-2.90E+00	6.14E-18	-1.19E+00	4.93E-04	-1.91E-01	1.00E+00
ENSBTAG00000017343	1.80E-02	1.00E+00	-1.30E-02	1.00E+00	-2.88E-01	5.35E-01	-2.11E-02	1.00E+00	1.11E-01	1.00E+00
ENSBTAG00000007423	1.43E-01	1.00E+00	-1.29E+00	2.37E-03	7.97E-02	1.00E+00	3.63E-01	5.42E-01	-1.42E+00	1.34E-02
ENSBTAG00000042620	0.00E+00	1.00E+00	0.00E+00	1.00E+00	-5.13E-15	1.00E+00	0.00E+00	1.00E+00	0.00E+00	1.00E+00
ENSBTAG00000043996	-1.97E-03	1.00E+00	4.97E-01	2.54E-01	-3.78E-01	4.65E-01	-5.50E-01	2.25E-01	-5.01E-01	6.79E-01
ENSBTAG00000044458	0.00E+00	1.00E+00	0.00E+00	1.00E+00	-5.13E-15	1.00E+00	0.00E+00	1.00E+00	0.00E+00	1.00E+00
ENSBTAG00000006209	1.21E-01	1.00E+00	1.17E+00	1.45E-09	9.17E-01	2.39E-06	2.80E-02	1.00E+00	2.84E-01	6.29E-01
ENSBTAG00000026254	-3.77E-03	1.00E+00	-1.05E-01	9.45E-01	-3.83E-02	1.00E+00	-2.60E-01	4.23E-01	-7.84E-02	1.00E+00
ENSBTAG00000045207	0.00E+00	1.00E+00	0.00E+00	1.00E+00	-5.13E-15	1.00E+00	0.00E+00	1.00E+00	0.00E+00	1.00E+00
ENSBTAG00000032738	5.11E-01	1.00E+00	9.74E-01	6.53E-01	-1.48E-01	1.00E+00	-9.44E-01	8.05E-01	-2.71E+00	6.01E-01
ENSBTAG00000007303	6.30E-02	1.00E+00	2.20E-01	4.43E-01	1.64E-01	6.24E-01	3.20E-01	1.89E-01	-2.40E-02	1.00E+00

ENSBTAG00000040422	1.81E-01	1.00E+00	6.68E-01	4.33E-03	7.32E-01	1.39E-03	1.46E-01	7.93E-01	-2.60E-01	8.00E-01
ENSBTAG00000014032	4.74E-02	1.00E+00	6.87E-01	8.08E-05	7.34E-01	2.15E-05	4.60E-01	1.43E-02	-4.97E-01	7.20E-02
ENSBTAG00000043498	0.00E+00	1.00E+00	0.00E+00	1.00E+00	-5.13E-15	1.00E+00	0.00E+00	1.00E+00	0.00E+00	1.00E+00
ENSBTAG00000045108	0.00E+00	1.00E+00	0.00E+00	1.00E+00	-5.13E-15	1.00E+00	0.00E+00	1.00E+00	0.00E+00	1.00E+00
ENSBTAG00000039631	0.00E+00	1.00E+00	0.00E+00	1.00E+00	-5.13E-15	1.00E+00	0.00E+00	1.00E+00	0.00E+00	1.00E+00
ENSBTAG00000026157	0.00E+00	1.00E+00	0.00E+00	1.00E+00	-5.13E-15	1.00E+00	0.00E+00	1.00E+00	0.00E+00	1.00E+00
ENSBTAG00000017694	2.21E-01	1.00E+00	2.42E-01	7.61E-01	5.65E-01	1.72E-01	3.52E-01	4.97E-01	-5.46E-01	6.32E-01
ENSBTAG00000042355	0.00E+00	1.00E+00	0.00E+00	1.00E+00	-5.13E-15	1.00E+00	2.53E+00	2.71E-01	0.00E+00	1.00E+00
ENSBTAG00000044733	0.00E+00	1.00E+00	0.00E+00	1.00E+00	-5.13E-15	1.00E+00	0.00E+00	1.00E+00	0.00E+00	1.00E+00
ENSBTAG00000039068	0.00E+00	1.00E+00	0.00E+00	1.00E+00	-5.13E-15	1.00E+00	0.00E+00	1.00E+00	0.00E+00	1.00E+00
ENSBTAG00000048026	0.00E+00	1.00E+00	0.00E+00	1.00E+00	-5.13E-15	1.00E+00	0.00E+00	1.00E+00	0.00E+00	1.00E+00
ENSBTAG00000038281	-1.20E-01	1.00E+00	-3.62E-01	3.23E-01	-7.29E-01	1.36E-02	-2.71E-01	5.20E-01	2.66E-02	1.00E+00
ENSBTAG00000045061	0.00E+00	1.00E+00	0.00E+00	1.00E+00	-5.13E-15	1.00E+00	0.00E+00	1.00E+00	0.00E+00	1.00E+00
ENSBTAG00000042375	0.00E+00	1.00E+00	0.00E+00	1.00E+00	-5.13E-15	1.00E+00	0.00E+00	1.00E+00	0.00E+00	1.00E+00
ENSBTAG00000043367	0.00E+00	1.00E+00	0.00E+00	1.00E+00	-5.13E-15	1.00E+00	1.76E+00	4.97E-01	0.00E+00	1.00E+00
ENSBTAG00000008512	0.00E+00	1.00E+00	0.00E+00	1.00E+00	-5.13E-15	1.00E+00	2.53E+00	2.71E-01	1.91E+00	8.00E-01
ENSBTAG00000044217	0.00E+00	1.00E+00	0.00E+00	1.00E+00	-5.13E-15	1.00E+00	0.00E+00	1.00E+00	0.00E+00	1.00E+00
ENSBTAG00000021747	0.00E+00	1.00E+00	0.00E+00	1.00E+00	-5.13E-15	1.00E+00	0.00E+00	1.00E+00	1.93E+00	8.00E-01
ENSBTAG00000032548	3.81E-01	4.33E-01	-2.79E+00	2.19E-35	-4.27E+00	4.50E-70	-1.65E+00	2.56E-14	4.69E-01	3.00E-01
ENSBTAG00000044232	0.00E+00	1.00E+00	0.00E+00	1.00E+00	-5.13E-15	1.00E+00	0.00E+00	1.00E+00	0.00E+00	1.00E+00
ENSBTAG00000037571	-5.67E-02	1.00E+00	-3.15E-01	1.92E-01	-5.65E-01	6.47E-03	-5.83E-01	5.59E-03	-4.44E-01	2.52E-01
ENSBTAG00000026247	-1.87E+00	6.78E-02	-1.02E+00	1.91E-01	-2.79E+00	3.34E-04	-1.43E+00	4.97E-02	-7.38E-01	7.90E-01
ENSBTAG00000007387	2.05E-01	8.23E-01	4.86E-01	1.17E-02	3.55E-01	8.66E-02	2.21E-01	3.88E-01	-8.09E-02	1.00E+00
ENSBTAG00000007386	-7.18E-02	1.00E+00	9.22E-01	2.77E-05	1.53E+00	4.87E-13	1.61E+00	3.51E-14	-3.28E-01	6.22E-01
ENSBTAG00000019997	2.71E+00	5.76E-01	3.30E+00	1.02E-01	3.05E+00	1.37E-01	2.53E+00	2.71E-01	2.71E+00	6.01E-01
ENSBTAG00000039141	0.00E+00	1.00E+00	0.00E+00	1.00E+00	2.56E+00	2.54E-01	2.54E+00	2.71E-01	1.91E+00	8.00E-01
ENSBTAG00000010006	-7.28E-02	1.00E+00	-2.70E-01	3.00E-01	-3.40E-01	1.53E-01	-1.08E-01	8.87E-01	-4.26E-01	3.07E-01
ENSBTAG00000006702	-5.77E-02	1.00E+00	9.29E-02	8.88E-01	-2.06E-02	1.00E+00	-1.16E-01	7.75E-01	-8.70E-01	1.99E-05
ENSBTAG00000013598	6.10E-01	8.23E-01	1.33E-01	1.00E+00	2.83E-01	8.92E-01	1.11E+00	5.52E-02	-4.53E-02	1.00E+00
ENSBTAG00000029159	0.00E+00	1.00E+00	0.00E+00	1.00E+00	-5.13E-15	1.00E+00	0.00E+00	1.00E+00	0.00E+00	1.00E+00
ENSBTAG00000006213	-3.43E-01	8.23E-01	-6.46E-01	7.23E-02	-1.37E+00	2.94E-05	-7.26E-01	3.84E-02	-1.18E-01	1.00E+00
ENSBTAG00000008257	1.02E-01	1.00E+00	1.13E+00	8.20E-07	1.90E+00	2.01E-18	1.84E+00	3.85E-17	-1.91E-01	9.97E-01
ENSBTAG00000014051	-4.34E-01	8.23E-01	-1.65E+00	2.48E-04	-3.13E-01	6.41E-01	-8.43E-02	1.00E+00	-1.23E+00	6.37E-02
ENSBTAG00000018352	1.34E-02	1.00E+00	8.81E-01	8.52E-01	-1.91E+00	4.28E-01	1.49E+00	4.54E-01	-1.91E+00	8.00E-01
ENSBTAG00000008285	3.71E-02	1.00E+00	-1.41E+00	6.07E-09	-8.69E-01	4.00E-04	1.66E-01	7.51E-01	-3.50E-02	1.00E+00

ENSBTAG00000001649	-1.19E-01	1.00E+00	-3.22E-01	5.60E-01	-2.30E-01	7.66E-01	-2.77E-01	6.61E-01	-5.14E-01	6.24E-01
ENSBTAG00000038276	0.00E+00	1.00E+00	0.00E+00	1.00E+00	-5.13E-15	1.00E+00	0.00E+00	1.00E+00	0.00E+00	1.00E+00
ENSBTAG00000042347	0.00E+00	1.00E+00	0.00E+00	1.00E+00	-5.13E-15	1.00E+00	0.00E+00	1.00E+00	0.00E+00	1.00E+00
ENSBTAG00000011516	-4.81E-01	1.00E+00	5.59E-01	5.36E-01	5.34E-01	5.45E-01	7.50E-02	1.00E+00	-4.36E-01	1.00E+00
ENSBTAG00000021240	3.03E-02	1.00E+00	-7.68E-01	2.47E-02	-9.30E-01	4.75E-03	-1.63E+00	3.79E-07	4.33E-01	6.68E-01
ENSBTAG00000021887	0.00E+00	1.00E+00	0.00E+00	1.00E+00	-5.13E-15	1.00E+00	0.00E+00	1.00E+00	0.00E+00	1.00E+00
ENSBTAG00000046422	0.00E+00	1.00E+00	0.00E+00	1.00E+00	-5.13E-15	1.00E+00	0.00E+00	1.00E+00	0.00E+00	1.00E+00
ENSBTAG00000003827	5.15E-01	1.00E+00	-2.45E-01	1.00E+00	1.19E-01	1.00E+00	9.03E-01	2.48E-01	-3.62E-01	1.00E+00
ENSBTAG00000021449	-4.39E-02	1.00E+00	1.01E+00	5.05E-05	1.44E+00	2.21E-09	1.22E+00	7.80E-07	3.21E-02	1.00E+00
ENSBTAG00000033902	-8.02E-02	1.00E+00	6.80E-01	8.76E-03	1.38E+00	7.07E-09	6.61E-01	1.25E-02	-8.29E-03	1.00E+00
ENSBTAG00000032591	1.44E+00	7.50E-01	9.01E-02	1.00E+00	-9.22E-01	8.08E-01	1.79E+00	1.58E-01	7.59E-03	1.00E+00
ENSBTAG00000010529	-8.60E-02	1.00E+00	-4.67E-01	1.48E-01	1.09E+00	2.94E-05	1.17E+00	7.60E-06	-5.50E-02	1.00E+00
ENSBTAG00000042499	0.00E+00	1.00E+00	1.98E+00	4.43E-01	1.78E+00	4.79E-01	0.00E+00	1.00E+00	0.00E+00	1.00E+00
ENSBTAG00000026242	-1.40E+00	4.24E-01	3.86E-01	8.21E-01	-3.73E-01	8.61E-01	1.65E-01	1.00E+00	1.16E-01	1.00E+00
ENSBTAG00000013513	2.78E-01	7.74E-01	4.11E-01	1.15E-01	4.88E-01	4.87E-02	3.92E-01	1.48E-01	6.90E-02	1.00E+00
ENSBTAG00000007943	1.06E+00	9.82E-07	7.99E-01	8.58E-05	1.11E+00	1.90E-08	1.02E+00	3.65E-07	-1.79E-01	9.15E-01
ENSBTAG00000014396	1.78E+00	4.42E-08	8.35E-01	9.93E-03	-2.56E-01	6.20E-01	2.28E-01	7.04E-01	6.81E-01	2.58E-01
ENSBTAG00000003445	8.05E-01	1.00E+00	-1.91E+00	4.69E-01	1.50E+00	4.32E-01	-1.54E-01	1.00E+00	7.93E-01	1.00E+00
ENSBTAG00000021209	-5.99E-03	1.00E+00	8.14E-02	9.69E-01	3.86E-01	6.14E-02	6.27E-01	1.05E-03	-2.97E-01	5.69E-01
ENSBTAG00000021208	-2.02E-01	9.37E-01	-1.15E+00	3.90E-07	-3.79E-01	1.54E-01	-4.89E-01	5.31E-02	-1.63E-01	1.00E+00
ENSBTAG00000043335	0.00E+00	1.00E+00	0.00E+00	1.00E+00	-5.13E-15	1.00E+00	0.00E+00	1.00E+00	0.00E+00	1.00E+00
ENSBTAG00000026963	-6.57E-01	3.85E-01	-2.56E-01	7.25E-01	-1.02E+00	8.73E-03	-5.45E-01	2.24E-01	-8.25E-01	2.52E-01
ENSBTAG00000004518	-5.90E-02	1.00E+00	1.37E+00	1.60E-02	4.14E-01	6.90E-01	-1.84E-01	1.00E+00	2.47E-01	1.00E+00
ENSBTAG00000044766	0.00E+00	1.00E+00	0.00E+00	1.00E+00	-5.13E-15	1.00E+00	0.00E+00	1.00E+00	0.00E+00	1.00E+00
ENSBTAG00000045224	0.00E+00	1.00E+00	0.00E+00	1.00E+00	-5.13E-15	1.00E+00	0.00E+00	1.00E+00	0.00E+00	1.00E+00
ENSBTAG00000043313	0.00E+00	1.00E+00	0.00E+00	1.00E+00	-5.13E-15	1.00E+00	0.00E+00	1.00E+00	0.00E+00	1.00E+00
ENSBTAG00000008054	8.45E-03	1.00E+00	5.35E-01	1.24E-02	6.75E-01	1.04E-03	5.41E-01	1.28E-02	-5.07E-01	1.56E-01
ENSBTAG00000000236	5.90E-01	2.21E-01	4.41E-01	1.93E-01	8.39E-01	2.75E-03	6.80E-01	2.26E-02	-4.89E-02	1.00E+00
ENSBTAG00000040590	1.09E+00	8.09E-01	-3.01E-01	1.00E+00	1.05E+00	4.04E-01	2.31E+00	4.22E-03	2.88E-01	1.00E+00
ENSBTAG00000046358	1.39E-02	1.00E+00	4.35E-01	1.11E-02	6.66E-01	3.83E-05	5.05E-01	3.06E-03	-2.95E-01	4.36E-01
ENSBTAG00000011271	1.31E-02	1.00E+00	1.42E+00	1.47E-01	-4.54E-01	9.67E-01	1.30E+00	1.93E-01	2.54E-01	1.00E+00
ENSBTAG00000001254	1.22E-01	1.00E+00	8.38E-01	2.33E-05	1.14E+00	3.24E-09	5.59E-01	8.29E-03	-3.22E-01	5.62E-01
ENSBTAG00000017833	3.75E-01	7.37E-01	1.48E+00	2.81E-07	8.40E-01	5.42E-03	3.69E-01	3.49E-01	-4.71E-02	1.00E+00
ENSBTAG00000032544	1.75E+00	1.15E-04	5.43E+00	2.07E-40	5.24E+00	1.97E-38	3.39E+00	3.07E-18	-1.66E-01	1.00E+00
ENSBTAG00000019795	-3.47E-03	1.00E+00	1.77E+00	3.31E-01	2.42E+00	8.09E-02	-1.55E-01	1.00E+00	-2.07E-03	1.00E+00

ENSBTAG00000019793	-6.53E-01	9.02E-01	-5.65E-01	6.32E-01	-8.30E-01	3.70E-01	9.82E-02	1.00E+00	-3.87E-01	1.00E+00
ENSBTAG00000014130	-1.38E-02	1.00E+00	1.06E-02	1.00E+00	-5.69E-01	7.22E-02	-5.06E-01	1.31E-01	-1.50E-01	1.00E+00
ENSBTAG00000042171	0.00E+00	1.00E+00	0.00E+00	1.00E+00	1.78E+00	4.79E-01	2.53E+00	2.71E-01	0.00E+00	1.00E+00
ENSBTAG00000037291	0.00E+00	1.00E+00	0.00E+00	1.00E+00	-5.13E-15	1.00E+00	0.00E+00	1.00E+00	0.00E+00	1.00E+00
ENSBTAG00000030020	0.00E+00	1.00E+00	0.00E+00	1.00E+00	-5.13E-15	1.00E+00	0.00E+00	1.00E+00	0.00E+00	1.00E+00
ENSBTAG00000012357	4.19E-01	5.55E-01	6.00E-01	3.92E-02	6.16E-01	3.15E-02	4.10E-01	2.18E-01	-5.43E-01	3.82E-01
ENSBTAG00000047534	-2.14E-01	1.00E+00	-1.47E-01	1.00E+00	6.64E-01	1.36E-01	7.33E-01	9.51E-02	-6.05E-01	6.72E-01
ENSBTAG00000026233	3.79E-01	8.23E-01	6.01E-01	1.26E-01	8.82E-01	1.03E-02	1.41E+00	1.05E-05	-1.39E-01	1.00E+00
ENSBTAG00000005855	-2.70E+00	5.76E-01	9.08E-02	1.00E+00	-2.70E+00	1.99E-01	-2.70E+00	2.05E-01	-7.86E-01	1.00E+00
ENSBTAG00000013213	-1.69E+00	7.72E-01	-8.19E-01	7.71E-01	-1.05E+00	5.90E-01	-1.08E+00	5.85E-01	-3.61E+00	3.09E-01
ENSBTAG00000026234	3.97E-01	3.89E-01	1.36E+00	1.59E-10	9.73E-01	6.90E-06	7.78E-01	5.53E-04	-1.06E-01	1.00E+00
ENSBTAG00000007768	8.08E-01	1.00E+00	-1.91E+00	4.69E-01	-1.31E-01	1.00E+00	-1.91E+00	4.39E-01	-1.91E+00	8.00E-01
ENSBTAG00000046739	-3.22E+00	3.75E-01	-4.29E-01	1.00E+00	-6.62E-01	8.93E-01	1.74E-01	1.00E+00	9.06E-01	9.25E-01
ENSBTAG00000018206	1.98E-01	1.00E+00	8.43E-01	8.32E-02	1.94E+00	5.98E-06	1.94E+00	8.13E-06	6.04E-02	1.00E+00
ENSBTAG00000012596	2.29E-01	9.94E-01	1.84E+00	4.20E-12	2.38E+00	1.46E-19	1.58E+00	3.76E-09	-2.51E-01	9.11E-01
ENSBTAG00000012595	-1.01E-01	1.00E+00	-1.17E-01	9.90E-01	-5.70E-01	7.27E-02	-7.33E-01	1.65E-02	-8.44E-01	5.19E-02
ENSBTAG00000018493	1.18E-01	1.00E+00	-3.06E-01	7.98E-01	-5.85E-01	3.72E-01	-1.05E+00	6.24E-02	-3.57E-01	1.00E+00
ENSBTAG00000016278	1.76E-02	1.00E+00	3.91E-01	2.17E-01	3.22E-01	3.49E-01	1.44E-01	8.64E-01	-3.47E-01	6.99E-01
ENSBTAG00000042293	0.00E+00	1.00E+00	0.00E+00	1.00E+00	-5.13E-15	1.00E+00	0.00E+00	1.00E+00	0.00E+00	1.00E+00
ENSBTAG00000017165	2.71E-01	1.00E+00	2.13E+00	2.06E-05	9.83E-01	8.02E-02	4.43E-01	5.86E-01	6.36E-01	7.53E-01
ENSBTAG00000036902	0.00E+00	1.00E+00	0.00E+00	1.00E+00	-5.13E-15	1.00E+00	0.00E+00	1.00E+00	0.00E+00	1.00E+00
ENSBTAG00000043410	0.00E+00	1.00E+00	0.00E+00	1.00E+00	1.78E+00	4.79E-01	0.00E+00	1.00E+00	0.00E+00	1.00E+00
ENSBTAG00000016959	4.54E-01	2.54E-01	2.82E-01	3.38E-01	-2.30E-01	4.76E-01	-4.44E-01	8.23E-02	1.39E-01	1.00E+00
ENSBTAG00000003098	2.84E-02	1.00E+00	6.22E-01	3.75E-02	1.23E+00	6.22E-06	1.15E+00	3.59E-05	-2.62E-03	1.00E+00
ENSBTAG00000032432	2.85E-02	1.00E+00	-3.33E-02	1.00E+00	-2.80E-01	4.83E-01	-2.36E-01	6.15E-01	1.65E-03	1.00E+00
ENSBTAG00000011290	1.69E-01	1.00E+00	5.74E-01	6.07E-02	3.66E-01	3.03E-01	3.85E-01	2.80E-01	-4.71E-01	4.99E-01
ENSBTAG00000011572	1.51E-01	1.00E+00	8.82E-03	1.00E+00	2.17E+00	2.25E-13	1.73E+00	1.62E-08	3.42E-01	8.38E-01
ENSBTAG00000046135	1.18E-01	1.00E+00	3.44E-01	1.98E-01	5.22E-02	1.00E+00	-4.33E-01	8.81E-02	-1.22E-01	1.00E+00
ENSBTAG00000011908	2.72E-01	8.23E-01	-9.20E-02	1.00E+00	-6.44E-01	1.36E-02	-1.21E+00	9.66E-07	9.94E-01	1.39E-03
ENSBTAG00000030067	0.00E+00	1.00E+00	0.00E+00	1.00E+00	-5.13E-15	1.00E+00	0.00E+00	1.00E+00	0.00E+00	1.00E+00
ENSBTAG00000047205	0.00E+00	1.00E+00	0.00E+00	1.00E+00	-5.13E-15	1.00E+00	0.00E+00	1.00E+00	0.00E+00	1.00E+00
ENSBTAG00000014357	3.78E-01	6.07E-01	-2.93E-01	4.32E-01	-4.78E-01	1.13E-01	-2.97E-02	1.00E+00	8.57E-01	2.18E-02
ENSBTAG00000013901	1.43E-01	1.00E+00	-6.57E-02	1.00E+00	-2.79E-01	2.38E-01	-2.20E-02	1.00E+00	3.16E-01	5.19E-01
ENSBTAG00000001522	2.92E-01	7.20E-01	1.21E+00	2.15E-08	1.50E+00	2.03E-12	8.43E-01	1.81E-04	-1.05E-01	1.00E+00
ENSBTAG00000001521	1.69E-01	1.00E+00	4.02E-01	2.83E-01	7.17E-01	2.22E-02	1.14E+00	1.37E-04	7.79E-02	1.00E+00

ENSBTAG00000007687	1.70E+00	7.75E-01	2.32E+00	1.19E-01	6.38E-01	9.81E-01	2.97E+00	1.65E-02	7.98E-01	1.00E+00
ENSBTAG00000044846	0.00E+00	1.00E+00	0.00E+00	1.00E+00	-5.13E-15	1.00E+00	0.00E+00	1.00E+00	0.00E+00	1.00E+00
ENSBTAG00000043970	-3.49E-01	8.27E-01	-2.19E+00	1.21E-09	-4.31E-01	3.19E-01	3.91E-02	1.00E+00	2.67E-01	9.56E-01
ENSBTAG00000019581	1.14E-01	1.00E+00	-5.06E-01	3.83E-02	6.70E-02	1.00E+00	-8.31E-02	1.00E+00	-3.92E-01	4.70E-01
ENSBTAG00000007570	3.10E-01	7.43E-01	1.21E+00	2.14E-07	1.24E+00	8.59E-08	1.19E+00	4.46E-07	-1.06E-02	1.00E+00
ENSBTAG00000042492	0.00E+00	1.00E+00	0.00E+00	1.00E+00	-5.13E-15	1.00E+00	0.00E+00	1.00E+00	0.00E+00	1.00E+00
ENSBTAG00000026232	-1.61E+00	2.53E-03	-1.36E+00	2.86E-03	-1.87E+00	2.41E-05	-8.23E-01	1.06E-01	3.91E-02	1.00E+00
ENSBTAG00000004906	-2.57E-01	9.30E-01	-8.14E-03	1.00E+00	-4.81E-01	1.47E-01	-1.24E+00	1.86E-05	-5.41E-01	4.04E-01
ENSBTAG00000004902	-2.97E-01	6.68E-01	-5.94E-01	9.19E-03	-2.56E-01	3.88E-01	-2.66E-01	3.74E-01	3.06E-01	6.40E-01
ENSBTAG00000013621	2.71E-01	8.81E-01	6.29E-01	3.46E-02	9.62E-01	4.28E-04	1.15E+00	2.33E-05	3.75E-02	1.00E+00
ENSBTAG00000021838	-3.88E-01	1.00E+00	-7.29E-01	4.67E-01	-4.40E-01	7.69E-01	-5.25E-03	1.00E+00	-5.24E-01	9.91E-01
ENSBTAG00000003789	-6.26E-02	1.00E+00	6.93E-01	3.30E-04	7.24E-01	1.52E-04	6.43E-01	1.11E-03	-7.34E-02	1.00E+00
ENSBTAG00000000632	-4.39E-01	4.62E-01	-2.15E+00	4.84E-15	-1.24E+00	2.13E-06	-8.78E-01	1.24E-03	-6.04E-02	1.00E+00
ENSBTAG00000024984	0.00E+00	1.00E+00	0.00E+00	1.00E+00	-5.13E-15	1.00E+00	0.00E+00	1.00E+00	0.00E+00	1.00E+00
ENSBTAG00000007596	2.26E+00	1.05E-05	2.64E-01	8.69E-01	1.02E+00	4.77E-02	9.36E-01	8.31E-02	1.71E-02	1.00E+00
ENSBTAG00000021964	1.48E-01	1.00E+00	-3.97E-01	5.14E-01	-3.90E-01	5.20E-01	2.61E-01	7.93E-01	5.11E-01	7.63E-01
ENSBTAG00000000199	2.12E-01	1.00E+00	-6.04E-01	5.40E-02	7.46E-01	8.69E-03	1.10E+00	5.31E-05	1.08E-02	1.00E+00
ENSBTAG00000045865	0.00E+00	1.00E+00	0.00E+00	1.00E+00	-5.13E-15	1.00E+00	0.00E+00	1.00E+00	0.00E+00	1.00E+00
ENSBTAG00000044190	6.57E-02	1.00E+00	3.49E-01	6.05E-01	1.13E+00	7.02E-03	1.10E+00	1.03E-02	5.96E-03	1.00E+00
ENSBTAG00000026231	0.00E+00	1.00E+00	0.00E+00	1.00E+00	-5.13E-15	1.00E+00	0.00E+00	1.00E+00	0.00E+00	1.00E+00
ENSBTAG00000013408	3.30E-01	7.16E-01	1.24E+00	4.59E-07	1.18E+00	1.47E-06	7.25E-01	6.28E-03	1.85E-01	1.00E+00
ENSBTAG00000013405	-1.34E-01	1.00E+00	-2.11E-02	1.00E+00	-2.03E-01	6.60E-01	-4.77E-01	1.21E-01	-1.30E-01	1.00E+00
ENSBTAG00000047532	3.02E+00	3.99E-19	2.22E-01	7.61E-01	1.04E-01	1.00E+00	7.66E-01	3.45E-02	6.84E-01	3.11E-01
ENSBTAG00000017339	-4.11E-01	8.23E-01	-9.90E-01	1.36E-02	-6.37E-01	1.32E-01	2.79E-02	1.00E+00	-5.03E-01	6.68E-01
ENSBTAG00000032301	-2.84E-01	1.00E+00	-5.26E-01	4.04E-01	-7.23E-01	1.84E-01	-1.66E+00	6.07E-04	5.55E-01	7.58E-01
ENSBTAG00000006004	3.61E-01	7.67E-01	5.25E-01	1.16E-01	3.18E-01	4.31E-01	-4.09E-01	2.90E-01	4.19E-01	6.25E-01
ENSBTAG00000003481	2.46E-01	8.67E-01	9.33E-01	1.26E-04	1.21E+00	2.56E-07	1.28E+00	7.43E-08	2.06E-01	9.43E-01
ENSBTAG00000039968	2.25E-01	1.00E+00	-9.14E-02	1.00E+00	3.84E-01	3.29E-01	6.29E-02	1.00E+00	-3.37E-01	8.00E-01
ENSBTAG00000032281	-2.53E-01	1.00E+00	4.73E-01	4.00E-01	1.68E+00	1.72E-05	1.79E+00	5.15E-06	-1.66E+00	6.37E-03
ENSBTAG00000011268	1.54E-01	1.00E+00	3.22E-01	4.49E-01	1.15E+00	5.12E-05	1.31E+00	3.36E-06	3.40E-01	8.00E-01
ENSBTAG00000015924	9.33E-01	9.43E-01	-3.21E+00	1.26E-01	-1.43E+00	4.79E-01	-3.21E+00	9.97E-02	9.19E-01	9.17E-01
ENSBTAG00000019663	-1.92E-02	1.00E+00	-2.89E-01	3.90E-01	-7.61E-01	2.40E-03	-1.00E+00	4.82E-05	-1.67E-01	1.00E+00
ENSBTAG00000013225	3.59E-01	5.79E-01	1.63E+00	5.21E-12	1.11E+00	3.28E-06	5.22E-01	5.59E-02	-3.71E-01	6.01E-01
ENSBTAG00000013678	-2.42E-01	9.43E-01	-5.28E-01	9.06E-02	5.74E-03	1.00E+00	4.98E-01	1.17E-01	4.61E-02	1.00E+00
ENSBTAG00000015271	7.29E-01	4.27E-02	1.56E+00	8.02E-10	1.22E+00	1.61E-06	7.88E-01	3.84E-03	-1.96E-01	1.00E+00

ENSBTAG00000006877	-1.36E-01	1.00E+00	-2.00E-01	1.00E+00	-4.01E-01	8.65E-01	1.28E+00	1.14E-01	1.77E-01	1.00E+00
ENSBTAG00000046836	0.00E+00	1.00E+00	0.00E+00	1.00E+00	-5.13E-15	1.00E+00	0.00E+00	1.00E+00	0.00E+00	1.00E+00
ENSBTAG00000007302	9.51E-01	1.00E+00	-4.30E-01	1.00E+00	-1.89E+00	5.84E-01	-2.30E+00	4.84E-01	1.08E+00	1.00E+00
ENSBTAG00000048317	2.71E+00	5.76E-01	0.00E+00	1.00E+00	1.78E+00	4.79E-01	1.76E+00	4.97E-01	0.00E+00	1.00E+00
ENSBTAG00000036304	-5.72E-01	1.00E+00	-7.18E-01	6.42E-01	-7.38E-01	5.89E-01	7.07E-02	1.00E+00	-1.41E-01	1.00E+00
ENSBTAG00000015536	3.69E-03	1.00E+00	-3.73E-01	3.60E-01	3.28E-01	4.34E-01	5.77E-01	9.63E-02	4.13E-01	6.68E-01
ENSBTAG00000015734	1.05E-01	1.00E+00	1.98E-01	4.82E-01	-2.15E-01	4.31E-01	-4.43E-01	4.23E-02	-6.55E-02	1.00E+00
ENSBTAG00000015720	2.76E-01	7.70E-01	1.53E+00	1.65E-12	9.72E-01	1.04E-05	2.89E-01	3.34E-01	4.55E-02	1.00E+00
ENSBTAG00000032233	0.00E+00	1.00E+00	0.00E+00	1.00E+00	-5.13E-15	1.00E+00	0.00E+00	1.00E+00	0.00E+00	1.00E+00
ENSBTAG00000023530	0.00E+00	1.00E+00	0.00E+00	1.00E+00	-5.13E-15	1.00E+00	0.00E+00	1.00E+00	0.00E+00	1.00E+00
ENSBTAG00000021092	3.81E-01	1.00E+00	2.67E-02	1.00E+00	-1.37E+00	2.54E-01	-1.08E+00	4.28E-01	1.52E+00	5.50E-01
ENSBTAG00000043511	0.00E+00	1.00E+00	0.00E+00	1.00E+00	-5.13E-15	1.00E+00	0.00E+00	1.00E+00	0.00E+00	1.00E+00
ENSBTAG00000002851	-2.71E+00	5.76E-01	-2.71E+00	2.42E-01	-9.29E-01	8.06E-01	-2.71E+00	2.05E-01	-7.87E-01	1.00E+00
ENSBTAG00000046121	-1.50E+00	5.42E-02	-2.83E+00	1.53E-05	-1.94E+00	6.92E-04	-1.01E+00	8.53E-02	-3.19E-01	1.00E+00
ENSBTAG00000017733	1.57E-01	1.00E+00	-6.70E-01	2.90E-01	-3.89E-01	6.43E-01	5.34E-01	4.39E-01	2.57E-01	1.00E+00
ENSBTAG00000015214	-4.94E-01	5.76E-01	-1.59E+00	1.55E-06	-3.78E+00	9.21E-28	-5.25E+00	8.18E-46	-7.23E-01	2.75E-01
ENSBTAG00000032236	-2.70E+00	5.76E-01	-2.70E+00	2.42E-01	-2.70E+00	1.99E-01	-9.41E-01	8.06E-01	-2.70E+00	6.01E-01
ENSBTAG00000036116	0.00E+00	1.00E+00	0.00E+00	1.00E+00	3.42E+00	7.40E-02	2.54E+00	2.71E-01	0.00E+00	1.00E+00
ENSBTAG00000001000	-1.35E-01	1.00E+00	-1.18E+00	5.40E-03	-2.25E+00	4.00E-08	-2.68E+00	1.51E-10	-2.06E-01	1.00E+00
ENSBTAG00000032227	1.75E-01	1.00E+00	1.24E-01	1.00E+00	5.65E-01	1.33E-01	2.67E-02	1.00E+00	-3.27E-01	8.51E-01
ENSBTAG00000009378	1.15E-01	1.00E+00	8.85E-01	4.14E-03	1.05E+00	4.41E-04	2.96E-01	5.08E-01	-5.06E-01	5.32E-01
ENSBTAG00000003338	1.59E-01	1.00E+00	8.84E-02	1.00E+00	1.88E-01	6.19E-01	5.15E-02	1.00E+00	-3.09E-01	6.84E-01
ENSBTAG00000040461	-1.91E+00	8.23E-01	1.39E+00	5.17E-01	1.52E+00	4.28E-01	-1.47E-01	1.00E+00	1.76E-02	1.00E+00
ENSBTAG00000038798	2.71E+00	5.76E-01	0.00E+00	1.00E+00	3.05E+00	1.36E-01	0.00E+00	1.00E+00	0.00E+00	1.00E+00
ENSBTAG00000016859	0.00E+00	1.00E+00	0.00E+00	1.00E+00	-5.13E-15	1.00E+00	0.00E+00	1.00E+00	0.00E+00	1.00E+00
ENSBTAG00000017717	-1.80E-01	1.00E+00	-9.61E-01	8.67E-03	-2.20E+00	2.40E-09	-2.22E+00	2.43E-09	-8.39E-01	1.74E-01
ENSBTAG00000046821	3.57E-02	1.00E+00	6.80E-01	5.92E-03	1.77E-01	6.95E-01	5.01E-02	1.00E+00	-2.40E-01	8.42E-01
ENSBTAG00000043332	0.00E+00	1.00E+00	0.00E+00	1.00E+00	-5.13E-15	1.00E+00	0.00E+00	1.00E+00	0.00E+00	1.00E+00
ENSBTAG00000019639	7.87E-01	4.52E-03	2.29E+00	1.75E-25	1.86E+00	1.07E-17	9.97E-01	1.03E-05	-4.48E-01	3.88E-01
ENSBTAG00000004792	-7.47E-01	7.76E-01	7.05E-01	2.86E-01	3.39E-01	7.76E-01	-2.74E-01	9.27E-01	-7.36E-01	7.90E-01
ENSBTAG00000043940	0.00E+00	1.00E+00	0.00E+00	1.00E+00	-5.13E-15	1.00E+00	0.00E+00	1.00E+00	0.00E+00	1.00E+00
ENSBTAG00000003588	1.04E-01	1.00E+00	-2.89E-02	1.00E+00	5.77E-01	2.14E-02	4.21E-01	1.31E-01	-1.96E-01	9.48E-01
ENSBTAG00000020523	2.38E-01	9.43E-01	-9.10E-02	1.00E+00	-9.27E-01	9.11E-04	-1.14E+00	4.15E-05	-2.06E-01	9.97E-01
ENSBTAG00000011709	1.21E+00	7.83E-04	1.81E-01	8.43E-01	-5.27E-01	1.48E-01	-8.15E-01	1.45E-02	7.01E-03	1.00E+00
ENSBTAG00000013165	1.19E-01	1.00E+00	-4.96E-01	9.93E-02	-5.79E-01	4.45E-02	-1.04E+00	1.02E-04	2.03E-01	9.88E-01

ENSBTAG00000015333	4.20E-01	3.45E-01	1.87E+00	2.75E-18	1.74E+00	4.26E-16	1.05E+00	1.68E-06	1.91E-01	9.28E-01
ENSBTAG00000015338	-1.49E-01	1.00E+00	6.39E-02	1.00E+00	4.41E-01	2.19E-01	-6.94E-02	1.00E+00	-1.75E-01	1.00E+00
ENSBTAG00000015341	-2.98E-01	1.00E+00	-7.25E-01	1.06E-01	9.32E-01	2.43E-02	9.47E-01	2.47E-02	3.82E-02	1.00E+00
ENSBTAG00000013369	-1.41E-01	1.00E+00	-4.61E-01	9.67E-02	-1.48E+00	5.73E-10	-1.29E+00	9.41E-08	-1.54E-01	1.00E+00
ENSBTAG00000042604	0.00E+00	1.00E+00	0.00E+00	1.00E+00	-5.13E-15	1.00E+00	0.00E+00	1.00E+00	0.00E+00	1.00E+00
ENSBTAG00000006767	8.30E-02	1.00E+00	-4.09E-02	1.00E+00	-1.72E-01	6.23E-01	-1.77E-01	6.15E-01	-2.42E-01	8.00E-01
ENSBTAG00000006770	2.00E-01	1.00E+00	1.93E-01	1.00E+00	6.68E-01	5.75E-01	2.65E-01	1.00E+00	2.89E-01	1.00E+00
ENSBTAG00000044126	3.74E-01	5.15E-01	-6.04E-01	1.44E-02	-2.07E+00	1.51E-19	-2.34E+00	4.80E-24	6.53E-01	7.36E-02
ENSBTAG00000043120	0.00E+00	1.00E+00	0.00E+00	1.00E+00	-5.13E-15	1.00E+00	0.00E+00	1.00E+00	0.00E+00	1.00E+00
ENSBTAG00000046228	0.00E+00	1.00E+00	0.00E+00	1.00E+00	-5.13E-15	1.00E+00	0.00E+00	1.00E+00	0.00E+00	1.00E+00
ENSBTAG00000040016	0.00E+00	1.00E+00	0.00E+00	1.00E+00	-5.13E-15	1.00E+00	0.00E+00	1.00E+00	0.00E+00	1.00E+00
ENSBTAG00000038319	0.00E+00	1.00E+00	0.00E+00	1.00E+00	-5.13E-15	1.00E+00	0.00E+00	1.00E+00	0.00E+00	1.00E+00
ENSBTAG00000035803	0.00E+00	1.00E+00	0.00E+00	1.00E+00	-5.13E-15	1.00E+00	0.00E+00	1.00E+00	0.00E+00	1.00E+00
ENSBTAG00000045558	0.00E+00	1.00E+00	0.00E+00	1.00E+00	-5.13E-15	1.00E+00	1.76E+00	4.97E-01	0.00E+00	1.00E+00
ENSBTAG00000040008	0.00E+00	1.00E+00	0.00E+00	1.00E+00	-5.13E-15	1.00E+00	0.00E+00	1.00E+00	0.00E+00	1.00E+00
ENSBTAG00000048221	0.00E+00	1.00E+00	0.00E+00	1.00E+00	-5.13E-15	1.00E+00	1.76E+00	4.97E-01	0.00E+00	1.00E+00
ENSBTAG00000010486	0.00E+00	1.00E+00	0.00E+00	1.00E+00	-5.13E-15	1.00E+00	0.00E+00	1.00E+00	0.00E+00	1.00E+00
ENSBTAG00000045790	0.00E+00	1.00E+00	0.00E+00	1.00E+00	-5.13E-15	1.00E+00	0.00E+00	1.00E+00	0.00E+00	1.00E+00
ENSBTAG00000046028	0.00E+00	1.00E+00	0.00E+00	1.00E+00	-5.13E-15	1.00E+00	0.00E+00	1.00E+00	0.00E+00	1.00E+00
ENSBTAG00000047552	0.00E+00	1.00E+00	0.00E+00	1.00E+00	-5.13E-15	1.00E+00	0.00E+00	1.00E+00	0.00E+00	1.00E+00
ENSBTAG00000003930	-1.91E+00	8.23E-01	-1.91E+00	4.69E-01	-1.91E+00	4.28E-01	-1.91E+00	4.39E-01	-1.91E+00	8.00E-01
ENSBTAG00000016807	0.00E+00	1.00E+00	0.00E+00	1.00E+00	-5.13E-15	1.00E+00	0.00E+00	1.00E+00	0.00E+00	1.00E+00
ENSBTAG00000001515	0.00E+00	1.00E+00	0.00E+00	1.00E+00	-5.13E-15	1.00E+00	0.00E+00	1.00E+00	0.00E+00	1.00E+00
ENSBTAG00000013156	0.00E+00	1.00E+00	0.00E+00	1.00E+00	-5.13E-15	1.00E+00	0.00E+00	1.00E+00	0.00E+00	1.00E+00
ENSBTAG00000003023	0.00E+00	1.00E+00	0.00E+00	1.00E+00	-5.13E-15	1.00E+00	0.00E+00	1.00E+00	0.00E+00	1.00E+00
ENSBTAG00000047007	0.00E+00	1.00E+00	0.00E+00	1.00E+00	-5.13E-15	1.00E+00	0.00E+00	1.00E+00	0.00E+00	1.00E+00
ENSBTAG00000039136	0.00E+00	1.00E+00	0.00E+00	1.00E+00	-5.13E-15	1.00E+00	0.00E+00	1.00E+00	0.00E+00	1.00E+00
ENSBTAG00000036025	1.92E+00	8.23E-01	0.00E+00	1.00E+00	-5.13E-15	1.00E+00	0.00E+00	1.00E+00	0.00E+00	1.00E+00
ENSBTAG00000046387	0.00E+00	1.00E+00	0.00E+00	1.00E+00	-5.13E-15	1.00E+00	0.00E+00	1.00E+00	0.00E+00	1.00E+00
ENSBTAG00000008923	7.45E-01	4.76E-01	1.83E+00	1.75E-05	3.52E-02	1.00E+00	-8.42E-01	1.56E-01	6.70E-01	6.03E-01
ENSBTAG00000008925	2.41E-01	9.78E-01	1.08E+00	1.60E-04	1.23E+00	1.17E-05	8.42E-01	4.99E-03	3.18E-02	1.00E+00
ENSBTAG00000009176	3.88E-03	1.00E+00	1.25E+00	7.35E-06	6.08E-01	4.99E-02	8.01E-02	1.00E+00	-7.87E-02	1.00E+00
ENSBTAG00000012279	9.66E-03	1.00E+00	6.19E-01	3.60E-02	7.82E-01	5.16E-03	6.18E-01	3.93E-02	-1.36E-01	1.00E+00
ENSBTAG00000005392	2.21E-02	1.00E+00	-6.44E-01	2.25E-02	-3.24E-01	3.45E-01	-5.21E-01	8.10E-02	-3.25E-01	7.68E-01
ENSBTAG00000047187	0.00E+00	1.00E+00	0.00E+00	1.00E+00	-5.13E-15	1.00E+00	0.00E+00	1.00E+00	0.00E+00	1.00E+00

ENSBTAG00000023989	1.26E-01	1.00E+00	1.52E-01	7.74E-01	9.25E-01	3.18E-05	7.61E-01	1.04E-03	1.06E-01	1.00E+00
ENSBTAG00000006176	1.70E+00	7.71E-01	2.32E+00	1.16E-01	1.80E+00	2.86E-01	3.08E+00	9.85E-03	1.32E+00	8.97E-01
ENSBTAG00000046094	0.00E+00	1.00E+00	0.00E+00	1.00E+00	-5.13E-15	1.00E+00	0.00E+00	1.00E+00	0.00E+00	1.00E+00
ENSBTAG00000036451	0.00E+00	1.00E+00	0.00E+00	1.00E+00	-5.13E-15	1.00E+00	0.00E+00	1.00E+00	0.00E+00	1.00E+00
ENSBTAG00000024639	1.77E-01	1.00E+00	-8.36E-01	3.78E-02	-5.75E-01	1.79E-01	-6.84E-03	1.00E+00	1.93E-01	1.00E+00
ENSBTAG00000020884	8.04E-01	2.45E-02	2.47E+00	1.42E-20	1.94E+00	1.61E-13	8.46E-01	2.56E-03	-3.63E-01	6.95E-01
ENSBTAG00000006093	0.00E+00	1.00E+00	0.00E+00	1.00E+00	-5.13E-15	1.00E+00	0.00E+00	1.00E+00	0.00E+00	1.00E+00
ENSBTAG00000034827	2.09E-01	1.00E+00	7.08E-02	1.00E+00	-4.36E-01	5.66E-01	-6.07E-01	3.65E-01	-2.62E-02	1.00E+00
ENSBTAG00000002887	0.00E+00	1.00E+00	0.00E+00	1.00E+00	-5.13E-15	1.00E+00	0.00E+00	1.00E+00	0.00E+00	1.00E+00
ENSBTAG00000047461	2.72E-02	1.00E+00	-1.14E+00	6.29E-03	-2.24E+00	3.13E-08	-2.12E+00	2.11E-07	-7.05E-01	4.45E-01
ENSBTAG00000019309	5.74E-01	8.89E-01	-1.89E-01	1.00E+00	4.09E-01	7.41E-01	2.10E+00	3.67E-05	7.63E-01	7.24E-01
ENSBTAG00000003087	-1.32E-01	1.00E+00	6.40E-01	3.13E-03	8.50E-01	4.36E-05	1.26E-01	8.29E-01	-3.57E-01	5.14E-01
ENSBTAG00000015059	-1.29E+00	9.36E-01	7.73E-01	7.31E-01	-6.55E-01	8.97E-01	-6.77E-01	8.89E-01	-1.29E+00	9.02E-01
ENSBTAG00000044985	0.00E+00	1.00E+00	0.00E+00	1.00E+00	-5.13E-15	1.00E+00	0.00E+00	1.00E+00	0.00E+00	1.00E+00
ENSBTAG00000037768	2.18E+00	2.34E-01	8.18E-01	6.73E-01	8.68E-01	6.21E-01	1.26E+00	3.86E-01	-2.08E+00	5.31E-01
ENSBTAG00000048029	1.31E-02	1.00E+00	8.84E-01	8.52E-01	-1.30E-01	1.00E+00	2.85E+00	2.45E-02	2.19E-02	1.00E+00
ENSBTAG00000015818	-2.70E+00	5.76E-01	-2.70E+00	2.42E-01	-2.70E+00	1.99E-01	1.23E+00	4.57E-01	-2.70E+00	6.01E-01
ENSBTAG00000020007	3.07E+00	5.65E-02	8.84E-01	8.52E-01	1.15E+00	6.43E-01	1.13E+00	6.66E-01	1.69E+00	7.74E-01
ENSBTAG00000045534	0.00E+00	1.00E+00	0.00E+00	1.00E+00	-5.13E-15	1.00E+00	0.00E+00	1.00E+00	0.00E+00	1.00E+00
ENSBTAG00000046732	-1.92E+00	8.23E-01	2.07E+00	2.02E-01	3.60E+00	8.07E-04	2.02E+00	2.05E-01	-1.92E+00	8.00E-01
ENSBTAG00000040567	-2.71E+00	5.76E-01	9.72E-01	6.61E-01	1.26E+00	4.30E-01	1.43E+00	3.40E-01	-2.71E+00	6.01E-01
ENSBTAG00000014251	-1.19E+00	6.14E-01	-8.57E-01	4.51E-01	-5.38E-02	1.00E+00	2.10E-02	1.00E+00	-5.55E-01	1.00E+00
ENSBTAG00000004594	-6.09E-01	9.62E-01	3.84E-02	1.00E+00	5.35E-01	6.79E-01	3.88E-01	8.73E-01	-1.05E+00	6.13E-01
ENSBTAG00000046567	0.00E+00	1.00E+00	0.00E+00	1.00E+00	-5.13E-15	1.00E+00	0.00E+00	1.00E+00	0.00E+00	1.00E+00
ENSBTAG00000035735	9.15E-01	1.95E-02	6.93E-01	3.38E-02	3.78E-01	3.43E-01	7.40E-01	2.32E-02	2.70E-02	1.00E+00
ENSBTAG00000024918	3.98E+00	3.57E-08	5.50E+00	1.36E-14	3.43E+00	2.14E-07	2.46E+00	2.10E-04	-7.19E-01	8.00E-01
ENSBTAG00000047406	2.04E-02	1.00E+00	1.28E-01	8.19E-01	1.76E-01	6.12E-01	1.65E-01	6.67E-01	-3.49E-01	5.28E-01
ENSBTAG00000039307	0.00E+00	1.00E+00	0.00E+00	1.00E+00	-5.13E-15	1.00E+00	0.00E+00	1.00E+00	0.00E+00	1.00E+00
ENSBTAG00000015309	-1.70E+00	1.14E-01	-6.04E+00	1.91E-06	-1.38E+00	5.52E-02	-5.97E-01	4.97E-01	-3.11E-01	1.00E+00
ENSBTAG00000043879	0.00E+00	1.00E+00	0.00E+00	1.00E+00	-5.13E-15	1.00E+00	0.00E+00	1.00E+00	0.00E+00	1.00E+00
ENSBTAG00000020032	-4.58E-01	1.00E+00	-6.32E-01	5.49E-01	-1.14E+00	1.74E-01	1.66E-01	1.00E+00	-9.82E-01	6.56E-01
ENSBTAG00000005308	6.06E-03	1.00E+00	-7.23E-01	9.45E-01	-2.71E+00	1.99E-01	-9.46E-01	8.04E-01	-7.91E-01	1.00E+00
ENSBTAG00000011227	7.13E-01	8.23E-01	1.50E+00	2.33E-02	1.06E+00	1.54E-01	5.57E-01	6.14E-01	7.10E-01	8.00E-01
ENSBTAG00000024648	1.12E+00	5.26E-01	6.67E-01	5.69E-01	1.88E-01	1.00E+00	5.93E-01	6.39E-01	5.83E-01	9.80E-01
ENSBTAG00000004423	3.33E-01	8.23E-01	-4.33E-01	2.92E-01	-1.48E+00	4.45E-06	-8.12E-01	1.98E-02	3.96E-01	7.63E-01

ENSBTAG00000047425	-4.23E-02	1.00E+00	1.50E-01	7.56E-01	1.71E-01	6.65E-01	2.12E-01	5.30E-01	-4.19E-01	4.13E-01
ENSBTAG00000020466	7.30E-01	2.85E-01	1.50E+00	4.26E-05	1.56E+00	1.76E-05	1.55E+00	2.55E-05	5.63E-01	6.01E-01
ENSBTAG00000034724	0.00E+00	1.00E+00	0.00E+00	1.00E+00	-5.13E-15	1.00E+00	0.00E+00	1.00E+00	0.00E+00	1.00E+00
ENSBTAG00000009511	0.00E+00	1.00E+00	0.00E+00	1.00E+00	-5.13E-15	1.00E+00	0.00E+00	1.00E+00	0.00E+00	1.00E+00
ENSBTAG00000037488	-3.70E-02	1.00E+00	-4.12E-04	1.00E+00	3.30E-01	3.46E-01	-4.63E-01	1.57E-01	3.26E-01	7.77E-01
ENSBTAG00000006581	2.42E-01	8.69E-01	6.46E-02	1.00E+00	4.63E-01	9.39E-02	-1.62E-01	7.73E-01	-3.10E-01	7.58E-01
ENSBTAG00000045881	1.92E+00	8.23E-01	3.98E+00	2.55E-02	3.96E+00	2.24E-02	3.03E+00	1.49E-01	1.91E+00	8.00E-01
ENSBTAG00000037962	7.03E-01	3.59E-01	8.62E-01	3.52E-02	2.14E-01	8.56E-01	5.07E-01	3.09E-01	3.58E-01	8.89E-01
ENSBTAG00000016557	8.30E-02	1.00E+00	2.71E-01	3.63E-01	1.06E+00	6.22E-07	9.85E-01	5.26E-06	4.22E-02	1.00E+00
ENSBTAG00000016547	6.22E-02	1.00E+00	5.79E-01	3.99E-02	7.31E-01	5.82E-03	7.20E-01	7.80E-03	-1.04E-01	1.00E+00
ENSBTAG00000020748	-2.28E-01	9.72E-01	-2.46E+00	1.96E-18	-1.35E+00	5.44E-07	-1.19E+00	1.43E-05	1.75E-02	1.00E+00
ENSBTAG00000004034	7.28E-02	1.00E+00	-7.35E-01	9.32E-02	-2.36E+00	1.62E-08	-1.43E+00	3.84E-04	8.50E-01	1.88E-01
ENSBTAG00000024803	-3.09E-02	1.00E+00	9.84E-02	1.00E+00	1.02E+00	6.91E-02	1.11E+00	4.85E-02	-3.10E-01	1.00E+00
ENSBTAG00000047573	0.00E+00	1.00E+00	0.00E+00	1.00E+00	-5.13E-15	1.00E+00	0.00E+00	1.00E+00	0.00E+00	1.00E+00
ENSBTAG00000001695	1.27E+00	6.38E-02	7.87E-01	2.07E-01	6.27E-01	3.51E-01	1.11E+00	3.53E-02	8.53E-01	4.99E-01
ENSBTAG00000001704	2.70E+00	5.76E-01	0.00E+00	1.00E+00	-5.13E-15	1.00E+00	0.00E+00	1.00E+00	0.00E+00	1.00E+00
ENSBTAG00000007913	1.05E-03	1.00E+00	3.94E-02	1.00E+00	2.21E-01	3.57E-01	1.60E-01	5.81E-01	-2.93E-01	5.46E-01
ENSBTAG00000018945	-1.24E-01	1.00E+00	-2.67E-01	4.89E-01	-1.94E-01	7.04E-01	2.52E-01	5.34E-01	-2.96E-02	1.00E+00
ENSBTAG00000014910	4.70E-01	1.00E+00	3.50E-01	1.00E+00	-8.23E-01	6.74E-01	2.71E-01	1.00E+00	1.10E+00	7.32E-01
ENSBTAG00000038062	-1.11E-01	1.00E+00	-9.83E-01	1.85E-02	-7.73E-01	7.23E-02	-5.90E-02	1.00E+00	7.85E-01	3.07E-01
ENSBTAG00000040449	-1.67E+00	7.80E-01	-2.76E-01	1.00E+00	-1.02E+00	6.06E-01	-1.05E+00	5.99E-01	-3.58E+00	3.09E-01
ENSBTAG00000018629	5.45E-02	1.00E+00	-1.80E-02	1.00E+00	4.87E-01	7.45E-02	3.87E-01	2.00E-01	-4.45E-01	4.43E-01
ENSBTAG00000044144	1.52E-01	1.00E+00	4.20E-01	8.68E-01	-6.85E-01	5.94E-01	1.11E+00	2.33E-01	-5.98E-01	1.00E+00
ENSBTAG00000010642	-1.04E-02	1.00E+00	-1.54E-01	7.83E-01	-1.47E-01	8.02E-01	-3.34E-01	2.74E-01	-1.40E-01	1.00E+00
ENSBTAG00000042301	0.00E+00	1.00E+00	0.00E+00	1.00E+00	-5.13E-15	1.00E+00	0.00E+00	1.00E+00	0.00E+00	1.00E+00
ENSBTAG00000002691	1.11E+00	7.18E-01	1.02E-01	1.00E+00	-1.33E+00	4.16E-01	8.83E-01	4.89E-01	2.60E-01	1.00E+00
ENSBTAG00000042844	0.00E+00	1.00E+00	0.00E+00	1.00E+00	-5.13E-15	1.00E+00	0.00E+00	1.00E+00	0.00E+00	1.00E+00
ENSBTAG00000003661	-4.09E-01	9.82E-01	-2.62E-01	8.85E-01	1.19E+00	1.88E-02	4.66E-01	5.18E-01	-2.18E-02	1.00E+00
ENSBTAG00000018246	-1.24E+00	1.54E-01	5.61E-01	3.41E-01	3.48E-01	6.53E-01	9.00E-02	1.00E+00	-5.17E-01	8.07E-01
ENSBTAG00000012882	-2.95E-02	1.00E+00	-7.96E-01	8.51E-03	-9.48E-02	1.00E+00	6.76E-01	2.88E-02	1.84E-01	1.00E+00
ENSBTAG00000042968	0.00E+00	1.00E+00	0.00E+00	1.00E+00	-5.13E-15	1.00E+00	1.76E+00	4.97E-01	0.00E+00	1.00E+00
ENSBTAG00000012885	-2.40E-01	8.40E-01	-7.51E-01	1.60E-03	-1.55E+00	8.34E-12	-2.01E+00	9.97E-19	-1.90E-01	9.57E-01
ENSBTAG00000010286	-2.65E-02	1.00E+00	-5.46E-01	4.66E-02	-4.01E-01	1.75E-01	-1.81E-01	7.07E-01	1.42E-01	1.00E+00
ENSBTAG00000003111	2.38E-01	9.04E-01	6.76E-01	1.02E-02	1.15E+00	2.57E-06	9.53E-01	1.72E-04	2.14E-01	9.34E-01
ENSBTAG00000036744	0.00E+00	1.00E+00	0.00E+00	1.00E+00	-5.13E-15	1.00E+00	0.00E+00	1.00E+00	0.00E+00	1.00E+00

ENSBTAG00000008709	8.03E-03	1.00E+00	-1.48E+00	1.01E-07	-1.15E+00	2.25E-05	-6.68E-01	2.11E-02	2.20E-01	9.37E-01
ENSBTAG00000008253	-3.73E-01	8.95E-01	-2.41E+00	1.46E-09	-1.32E+00	9.55E-04	6.83E-01	1.41E-01	1.62E-01	1.00E+00
ENSBTAG00000002382	1.03E-01	1.00E+00	1.76E+00	2.72E-13	2.57E+00	7.86E-27	2.37E+00	5.37E-23	-5.52E-01	3.09E-01
ENSBTAG00000047738	0.00E+00	1.00E+00	0.00E+00	1.00E+00	-5.13E-15	1.00E+00	0.00E+00	1.00E+00	0.00E+00	1.00E+00
ENSBTAG00000004130	0.00E+00	1.00E+00	0.00E+00	1.00E+00	-5.13E-15	1.00E+00	0.00E+00	1.00E+00	0.00E+00	1.00E+00
ENSBTAG00000046145	0.00E+00	1.00E+00	0.00E+00	1.00E+00	-5.13E-15	1.00E+00	0.00E+00	1.00E+00	0.00E+00	1.00E+00
ENSBTAG00000046342	6.62E-03	1.00E+00	6.91E-02	1.00E+00	-1.32E-01	1.00E+00	-1.91E+00	4.39E-01	-1.91E+00	8.00E-01
ENSBTAG00000008732	5.54E-01	3.47E-01	3.83E-01	3.27E-01	5.83E-01	7.67E-02	3.15E-01	4.67E-01	7.67E-02	1.00E+00
ENSBTAG00000047384	0.00E+00	1.00E+00	0.00E+00	1.00E+00	-5.13E-15	1.00E+00	0.00E+00	1.00E+00	0.00E+00	1.00E+00
ENSBTAG00000004672	-1.14E-01	1.00E+00	-5.47E-01	1.45E-02	-6.28E-01	3.97E-03	-2.98E-01	2.72E-01	-1.08E-01	1.00E+00
ENSBTAG00000011793	-2.14E-01	9.57E-01	-7.25E-01	4.79E-03	-1.44E+00	2.75E-09	-1.50E+00	9.10E-10	-4.67E-01	4.15E-01
ENSBTAG00000000333	-1.03E+00	4.80E-02	-3.50E+00	5.01E-13	-2.33E+00	9.19E-09	-1.11E+00	4.39E-03	-1.04E+00	8.28E-02
ENSBTAG00000043096	0.00E+00	1.00E+00	0.00E+00	1.00E+00	-5.13E-15	1.00E+00	0.00E+00	1.00E+00	0.00E+00	1.00E+00
ENSBTAG00000044177	3.37E-02	1.00E+00	5.53E-01	8.07E-01	4.18E+00	1.00E-07	4.25E+00	8.20E-08	1.12E-01	1.00E+00
ENSBTAG00000006282	7.99E-01	3.10E-01	-3.99E-01	5.75E-01	3.31E-03	1.00E+00	-4.73E-02	1.00E+00	3.15E-01	1.00E+00
ENSBTAG00000034346	-1.03E-01	1.00E+00	3.07E-01	9.69E-01	1.22E-01	1.00E+00	1.84E-01	1.00E+00	-4.99E-01	1.00E+00
ENSBTAG00000029856	-1.91E+00	8.23E-01	-1.91E+00	4.69E-01	-1.91E+00	4.28E-01	-1.91E+00	4.39E-01	-1.91E+00	8.00E-01
ENSBTAG00000029991	0.00E+00	1.00E+00	0.00E+00	1.00E+00	-5.13E-15	1.00E+00	0.00E+00	1.00E+00	0.00E+00	1.00E+00
ENSBTAG00000034338	-1.67E+00	7.76E-01	-3.59E+00	6.66E-02	-3.59E+00	4.53E-02	-1.83E+00	2.99E-01	-3.59E+00	3.09E-01
ENSBTAG00000017901	-3.11E-01	8.93E-01	-1.88E+00	3.69E-08	-2.45E-01	6.75E-01	-9.06E-01	9.46E-03	-6.15E-01	4.15E-01
ENSBTAG00000020272	2.42E-01	8.54E-01	5.61E-02	1.00E+00	4.20E-01	1.28E-01	1.04E+00	1.02E-05	2.46E-02	1.00E+00
ENSBTAG00000020277	-7.93E-02	1.00E+00	-4.60E-01	2.24E-02	-2.07E-01	4.29E-01	5.71E-02	1.00E+00	-3.94E-01	3.09E-01
ENSBTAG00000020279	-3.00E-01	7.83E-01	-1.42E+00	1.51E-08	-1.58E+00	1.56E-10	-7.74E-01	3.05E-03	2.60E-02	1.00E+00
ENSBTAG00000004949	1.32E-01	1.00E+00	1.01E+00	4.39E-03	1.66E+00	2.59E-07	1.53E+00	3.88E-06	5.99E-02	1.00E+00
ENSBTAG00000004951	-2.40E-01	1.00E+00	-1.55E+00	1.09E-02	-2.31E+00	1.38E-04	-1.51E+00	1.05E-02	5.05E-01	8.00E-01
ENSBTAG00000045821	0.00E+00	1.00E+00	0.00E+00	1.00E+00	-5.13E-15	1.00E+00	0.00E+00	1.00E+00	0.00E+00	1.00E+00
ENSBTAG00000000434	6.52E-01	9.31E-01	2.82E+00	2.04E-04	2.26E+00	3.09E-03	2.17E+00	5.49E-03	1.05E+00	6.17E-01
ENSBTAG00000000440	-2.11E-01	1.00E+00	-7.70E-01	5.94E-02	-1.92E+00	5.24E-07	-2.97E-01	6.23E-01	-9.54E-02	1.00E+00
ENSBTAG00000008661	-3.69E-01	8.23E-01	-1.54E+00	7.24E-06	-8.67E-01	1.57E-02	8.64E-01	1.66E-02	-2.35E-02	1.00E+00
ENSBTAG00000008665	6.66E-01	8.04E-01	3.82E-01	7.58E-01	7.25E-01	3.23E-01	1.66E+00	5.20E-03	2.92E-02	1.00E+00
ENSBTAG00000010709	2.36E-02	1.00E+00	-3.84E-01	4.77E-01	8.83E-01	2.81E-02	1.88E+00	4.93E-07	2.89E-01	9.88E-01
ENSBTAG00000005156	-3.64E-01	1.00E+00	-2.75E-01	8.84E-01	-8.05E-01	2.13E-01	9.41E-02	1.00E+00	-1.45E-01	1.00E+00
ENSBTAG00000001999	3.03E-01	6.08E-01	1.44E+00	2.14E-13	1.72E+00	4.27E-19	1.38E+00	2.03E-12	1.55E-01	1.00E+00
ENSBTAG00000002000	1.57E-02	1.00E+00	7.24E-01	1.36E-03	7.18E-01	1.43E-03	8.03E-02	1.00E+00	-2.27E-01	8.29E-01
ENSBTAG00000016266	-8.31E-02	1.00E+00	-3.44E-01	2.48E-01	-3.76E-01	1.93E-01	-2.30E-01	5.27E-01	-3.33E-02	1.00E+00

ENSBTAG00000000277	2.57E-01	9.50E-01	-1.08E+00	3.05E-04	-1.69E+00	6.64E-09	-2.19E+00	1.20E-13	-1.11E+00	5.27E-03
ENSBTAG00000016548	-1.20E+00	2.00E-02	-1.91E+00	1.74E-06	-1.93E+00	1.11E-06	-2.41E+00	2.46E-09	-2.17E+00	2.90E-06
ENSBTAG00000034193	-4.75E-01	3.68E-01	-1.02E+00	8.02E-05	-2.04E+00	1.28E-15	-2.70E+00	7.97E-25	2.21E-01	9.24E-01
ENSBTAG00000003770	-3.48E-01	7.06E-01	-1.05E+00	9.37E-05	-7.99E-01	3.37E-03	-7.89E-01	4.54E-03	-2.34E-01	9.07E-01
ENSBTAG00000015810	3.04E-01	9.79E-01	-1.44E+00	1.23E-03	-1.06E+00	1.32E-02	-2.34E+00	3.35E-07	-3.33E-01	9.23E-01
ENSBTAG00000005710	7.88E-01	8.23E-01	1.54E-01	1.00E+00	-1.11E+00	2.68E-01	-9.88E-01	3.51E-01	9.68E-01	6.40E-01
ENSBTAG00000010008	4.43E-02	1.00E+00	-1.49E+00	2.28E-04	-1.71E+00	1.54E-05	-1.63E+00	4.79E-05	-5.76E-01	6.01E-01
ENSBTAG00000010855	1.92E+00	8.23E-01	0.00E+00	1.00E+00	1.78E+00	4.79E-01	3.04E+00	1.49E-01	1.91E+00	8.00E-01
ENSBTAG00000010860	1.74E-03	1.00E+00	-1.11E+00	5.40E-01	-1.70E-01	1.00E+00	-2.13E+00	1.75E-01	-3.89E+00	2.05E-01
ENSBTAG00000017650	2.47E+00	2.87E-01	1.77E+00	3.28E-01	-1.24E-01	1.00E+00	6.23E-01	9.94E-01	1.68E+00	7.79E-01
ENSBTAG00000020066	-2.84E-01	7.53E-01	-2.62E-01	4.05E-01	2.64E-01	3.90E-01	4.82E-01	5.43E-02	-2.56E-01	8.00E-01
ENSBTAG00000039149	0.00E+00	1.00E+00	0.00E+00	1.00E+00	-5.13E-15	1.00E+00	0.00E+00	1.00E+00	0.00E+00	1.00E+00
ENSBTAG00000002323	-2.48E-01	9.17E-01	-1.95E+00	1.81E-13	-1.79E+00	8.43E-12	-1.10E+00	4.61E-05	-3.62E-01	6.83E-01
ENSBTAG00000031330	9.39E-01	9.44E-01	-4.34E-01	1.00E+00	-6.63E-01	8.94E-01	1.54E+00	1.89E-01	-1.31E+00	9.00E-01
ENSBTAG00000044464	0.00E+00	1.00E+00	0.00E+00	1.00E+00	-5.13E-15	1.00E+00	0.00E+00	1.00E+00	0.00E+00	1.00E+00
ENSBTAG00000010791	2.72E+00	5.76E-01	1.98E+00	4.43E-01	1.78E+00	4.79E-01	3.41E+00	8.26E-02	3.21E+00	4.34E-01
ENSBTAG00000011266	2.07E+00	2.50E-10	2.38E+00	1.89E-14	1.90E+00	8.36E-10	1.93E+00	5.83E-10	1.64E+00	6.60E-06
ENSBTAG00000046254	0.00E+00	1.00E+00	0.00E+00	1.00E+00	-5.13E-15	1.00E+00	0.00E+00	1.00E+00	0.00E+00	1.00E+00
ENSBTAG00000008564	7.72E-01	6.80E-01	-1.30E-01	1.00E+00	-9.98E-01	1.36E-01	4.05E-01	7.30E-01	6.21E-01	8.00E-01
ENSBTAG00000008220	-2.47E-01	9.24E-01	-1.10E+00	5.17E-05	-1.46E+00	4.69E-08	-1.26E+00	3.70E-06	-2.47E-02	1.00E+00
ENSBTAG00000008219	6.56E-02	1.00E+00	9.19E-01	9.07E-06	1.16E+00	8.90E-09	9.74E-01	2.68E-06	-4.12E-01	3.89E-01
ENSBTAG00000005843	1.82E-01	1.00E+00	8.31E-01	8.17E-03	1.86E+00	2.73E-10	2.34E+00	3.40E-15	-9.45E-02	1.00E+00
ENSBTAG00000005945	3.92E-01	1.00E+00	1.23E+00	4.98E-02	1.14E-01	1.00E+00	-8.07E-01	4.38E-01	2.18E-01	1.00E+00
ENSBTAG00000031362	-1.91E+00	8.23E-01	7.70E-02	1.00E+00	-1.91E+00	4.28E-01	1.12E+00	6.68E-01	-1.91E+00	8.00E-01
ENSBTAG00000044938	0.00E+00	1.00E+00	0.00E+00	1.00E+00	-5.13E-15	1.00E+00	0.00E+00	1.00E+00	0.00E+00	1.00E+00
ENSBTAG00000000977	1.13E-01	1.00E+00	-2.87E-01	3.00E-01	-7.54E-01	4.88E-04	-1.62E-01	6.90E-01	5.50E-01	1.27E-01
ENSBTAG00000045283	0.00E+00	1.00E+00	0.00E+00	1.00E+00	-5.13E-15	1.00E+00	0.00E+00	1.00E+00	0.00E+00	1.00E+00
ENSBTAG00000037657	8.52E-01	8.39E-01	1.55E+00	6.70E-02	2.19E+00	1.83E-03	8.78E-01	4.50E-01	-4.12E+00	1.39E-01
ENSBTAG00000016855	1.21E-01	1.00E+00	1.06E+00	2.57E-07	1.56E+00	7.05E-15	3.38E-01	1.95E-01	4.50E-02	1.00E+00
ENSBTAG00000002650	-8.05E-02	1.00E+00	6.01E-01	2.71E-02	9.76E-01	1.12E-04	3.30E-01	3.29E-01	2.58E-01	8.24E-01
ENSBTAG00000019764	-1.30E-01	1.00E+00	-1.31E+00	2.09E-08	-2.64E+00	4.33E-29	-3.19E+00	8.25E-40	3.24E-01	6.84E-01
ENSBTAG00000039673	3.61E-02	1.00E+00	-4.15E-01	8.46E-01	-2.55E-01	1.00E+00	-9.32E-01	3.38E-01	-1.88E-01	1.00E+00
ENSBTAG00000019770	-3.31E-02	1.00E+00	-7.02E-01	1.03E-01	-1.75E+00	3.39E-06	-1.51E+00	9.01E-05	-1.53E-01	1.00E+00
ENSBTAG00000012398	-1.61E-01	1.00E+00	-5.10E-01	8.50E-02	-1.34E+00	2.01E-07	-1.71E+00	3.26E-11	-1.18E+00	2.18E-04
ENSBTAG00000002258	-5.90E-02	1.00E+00	-6.34E-01	1.25E-01	-1.23E+00	7.20E-04	-1.40E+00	1.23E-04	1.67E-02	1.00E+00

ENSBTAG00000015119	-9.48E-02	1.00E+00	-5.11E-01	5.03E-03	-4.86E-01	7.85E-03	-3.09E-01	1.39E-01	4.44E-01	1.36E-01
ENSBTAG00000005627	-1.73E-01	1.00E+00	-2.17E-01	5.69E-01	5.97E-01	1.77E-02	9.41E-01	7.59E-05	2.40E-01	8.33E-01
ENSBTAG00000007192	2.38E-01	8.26E-01	-3.58E-01	2.03E-01	4.56E-01	6.96E-02	9.49E-01	2.19E-05	3.77E-01	5.16E-01
ENSBTAG00000007196	2.26E-01	1.00E+00	-2.41E-01	8.70E-01	-8.04E-01	1.13E-01	-4.15E-01	5.40E-01	-1.73E-01	1.00E+00
ENSBTAG00000003052	-4.10E-01	3.98E-01	-7.06E-01	3.07E-03	-5.60E-01	2.34E-02	-1.65E-01	7.27E-01	8.18E-02	1.00E+00
ENSBTAG00000019358	-3.46E-02	1.00E+00	-1.32E+00	4.01E-07	-8.39E-01	1.39E-03	-2.83E-01	4.39E-01	2.17E-01	9.24E-01
ENSBTAG00000043426	0.00E+00	1.00E+00	0.00E+00	1.00E+00	-5.13E-15	1.00E+00	0.00E+00	1.00E+00	0.00E+00	1.00E+00
ENSBTAG00000019365	-2.76E-01	1.00E+00	-1.35E+00	2.21E-04	-1.05E+00	4.70E-03	-4.18E-01	4.08E-01	5.32E-01	6.01E-01
ENSBTAG00000014137	1.54E-01	1.00E+00	-7.19E-01	1.18E-02	-2.20E-02	1.00E+00	1.46E-01	8.60E-01	7.14E-01	7.86E-02
ENSBTAG00000016692	1.07E+00	6.24E-01	3.35E-01	9.90E-01	7.36E-02	1.00E+00	1.33E-01	1.00E+00	5.84E-01	1.00E+00
ENSBTAG00000039510	0.00E+00	1.00E+00	0.00E+00	1.00E+00	-5.13E-15	1.00E+00	0.00E+00	1.00E+00	0.00E+00	1.00E+00
ENSBTAG00000009828	-4.73E-01	1.00E+00	-1.18E+00	3.88E-01	-9.52E-01	5.06E-01	-3.59E+00	2.13E-03	-1.46E+00	6.15E-01
ENSBTAG00000014354	-5.27E-01	4.07E-01	-1.84E+00	9.55E-10	-2.22E+00	1.52E-13	-1.54E+00	3.06E-07	-2.26E-01	9.98E-01
ENSBTAG00000047932	0.00E+00	1.00E+00	0.00E+00	1.00E+00	-5.13E-15	1.00E+00	0.00E+00	1.00E+00	0.00E+00	1.00E+00
ENSBTAG00000008342	-9.29E-01	8.75E-01	-2.55E+00	5.97E-02	-1.48E+00	2.23E-01	-1.13E+00	3.86E-01	-3.92E-01	1.00E+00
ENSBTAG00000005215	-2.89E-01	9.70E-01	-4.48E-01	3.20E-01	-1.56E-01	9.46E-01	-1.27E-02	1.00E+00	-1.69E-02	1.00E+00
ENSBTAG00000042236	0.00E+00	1.00E+00	0.00E+00	1.00E+00	-5.13E-15	1.00E+00	0.00E+00	1.00E+00	0.00E+00	1.00E+00
ENSBTAG00000021518	-1.34E-01	1.00E+00	-4.30E+00	2.60E-08	-3.46E+00	4.68E-07	-4.58E+00	2.37E-09	-1.17E+00	3.67E-01
ENSBTAG00000039340	-1.01E+00	8.12E-01	-1.47E+00	1.91E-01	-3.37E+00	4.18E-03	-2.61E+00	1.87E-02	-8.07E-01	8.60E-01
ENSBTAG00000033529	5.15E-01	1.00E+00	5.89E-01	9.49E-01	1.46E+00	3.04E-01	6.88E-01	8.58E-01	2.40E+00	1.79E-01
ENSBTAG00000023283	6.61E-01	3.63E-01	1.85E+00	1.01E-07	7.49E-01	5.70E-02	-4.25E-01	4.17E-01	6.64E-01	4.30E-01
ENSBTAG00000001233	5.79E-01	3.06E-01	1.85E+00	2.12E-10	1.70E+00	4.89E-09	6.73E-01	4.12E-02	-1.13E-01	1.00E+00
ENSBTAG00000033510	-9.89E-01	2.11E-01	-1.04E+00	3.71E-02	-1.26E+00	9.33E-03	-2.10E+00	9.04E-06	-3.05E-01	1.00E+00
ENSBTAG00000015710	-1.93E-01	1.00E+00	-6.99E-01	9.67E-02	-8.50E-01	3.35E-02	-1.10E+00	4.94E-03	-8.91E-01	1.84E-01
ENSBTAG00000006452	-2.90E-01	9.36E-01	-8.95E-01	9.93E-03	-1.18E+00	4.13E-04	-8.51E-01	1.55E-02	-8.77E-01	1.08E-01
ENSBTAG00000006453	-3.20E-01	9.45E-01	-1.01E+00	1.06E-02	-1.13E+00	3.09E-03	-1.13E+00	3.91E-03	-7.85E-01	3.07E-01
ENSBTAG00000019725	2.03E-01	1.00E+00	8.06E-01	3.20E-02	8.00E-01	3.33E-02	1.01E+00	5.87E-03	6.01E-01	4.80E-01
ENSBTAG00000047070	0.00E+00	1.00E+00	0.00E+00	1.00E+00	-5.13E-15	1.00E+00	0.00E+00	1.00E+00	0.00E+00	1.00E+00
ENSBTAG00000007332	-1.37E-01	1.00E+00	1.42E-02	1.00E+00	-4.18E-02	1.00E+00	-2.96E-01	3.08E-01	-1.95E-01	9.11E-01
ENSBTAG00000018093	3.11E-01	9.27E-01	-6.10E-02	1.00E+00	-2.04E-01	8.15E-01	-1.36E-01	1.00E+00	4.66E-01	6.68E-01
ENSBTAG00000042378	0.00E+00	1.00E+00	0.00E+00	1.00E+00	-5.13E-15	1.00E+00	0.00E+00	1.00E+00	0.00E+00	1.00E+00
ENSBTAG00000014899	-5.20E-02	1.00E+00	-9.64E-01	2.22E-05	-2.07E+00	2.18E-20	-2.38E+00	5.92E-26	1.35E-01	1.00E+00
ENSBTAG00000012940	-3.94E-01	8.23E-01	-3.14E+00	3.88E-18	-4.99E+00	3.40E-37	-3.14E+00	4.47E-18	9.51E-01	9.77E-02
ENSBTAG00000012941	6.62E-02	1.00E+00	-5.47E-01	1.02E-01	-2.93E-01	4.79E-01	5.66E-01	8.61E-02	5.32E-01	4.32E-01
ENSBTAG00000006690	3.60E-01	4.59E-01	1.37E+00	3.31E-11	1.49E+00	4.56E-13	1.43E+00	6.20E-12	1.67E-01	9.83E-01

ENSBTAG00000016197	1.26E-01	1.00E+00	-1.23E+00	3.14E-05	-1.85E+00	1.69E-10	-6.09E-01	6.91E-02	9.14E-02	1.00E+00
ENSBTAG00000016202	-9.29E-02	1.00E+00	-2.75E-01	3.47E-01	-9.21E-01	2.24E-05	-1.15E+00	1.07E-07	-4.52E-02	1.00E+00
ENSBTAG00000009067	2.13E-01	8.23E-01	4.51E-01	2.12E-02	4.40E-01	2.50E-02	4.30E-01	3.35E-02	-9.81E-02	1.00E+00
ENSBTAG00000043660	0.00E+00	1.00E+00	0.00E+00	1.00E+00	-5.13E-15	1.00E+00	0.00E+00	1.00E+00	0.00E+00	1.00E+00
ENSBTAG00000046320	4.87E-01	1.00E+00	-6.92E-01	4.84E-01	-1.26E+00	1.13E-01	-1.08E+00	2.05E-01	-2.39E+00	1.84E-02
ENSBTAG00000005138	-7.36E-01	1.45E-01	-3.10E+00	2.14E-21	-1.98E+00	3.03E-10	1.64E-01	8.97E-01	3.79E-01	7.90E-01
ENSBTAG00000012750	-1.30E+00	9.36E-01	-3.22E+00	1.25E-01	-3.22E+00	9.40E-02	-1.46E+00	4.92E-01	5.49E-03	1.00E+00
ENSBTAG00000014606	-3.90E+00	1.41E-01	-1.11E+00	5.39E-01	-1.34E+00	4.08E-01	-8.64E-01	6.57E-01	-1.98E+00	6.01E-01
ENSBTAG00000017932	-6.06E-01	1.23E-01	-1.49E+00	6.80E-09	-6.00E-01	2.55E-02	-2.42E-01	5.15E-01	1.57E-01	1.00E+00
ENSBTAG00000027772	3.92E-02	1.00E+00	2.81E-01	5.01E-01	3.15E-01	4.28E-01	1.40E-01	9.22E-01	-8.37E-01	6.45E-02
ENSBTAG00000008075	-2.61E-02	1.00E+00	-1.77E-01	6.26E-01	-5.95E-01	7.64E-03	-6.42E-01	4.29E-03	-9.16E-02	1.00E+00
ENSBTAG00000008077	-4.35E-01	3.80E-01	-1.04E+00	1.63E-05	-2.10E+00	6.53E-19	-2.79E+00	9.73E-31	7.79E-02	1.00E+00
ENSBTAG00000010322	2.54E+00	2.82E-12	5.63E+00	4.58E-47	5.08E+00	1.11E-40	4.03E+00	1.81E-28	8.91E-01	1.34E-01
ENSBTAG00000005357	-2.38E-01	9.48E-01	6.98E-01	1.48E-02	-4.63E-01	1.48E-01	-7.72E-01	7.59E-03	8.36E-02	1.00E+00
ENSBTAG00000005364	-3.43E-01	7.56E-01	-7.92E-02	1.00E+00	-7.33E-01	1.05E-02	-5.88E-01	5.53E-02	-4.52E-01	5.20E-01
ENSBTAG00000038047	2.54E-01	1.00E+00	2.06E+00	3.17E-11	1.58E+00	3.45E-07	1.11E+00	7.11E-04	-1.41E-02	1.00E+00
ENSBTAG00000005371	6.28E-01	1.16E-01	1.40E+00	3.64E-08	1.52E+00	1.54E-09	1.33E+00	2.14E-07	5.42E-01	3.09E-01
ENSBTAG00000015013	1.78E-01	1.00E+00	8.36E-01	2.27E-03	6.78E-01	1.65E-02	3.89E-01	2.55E-01	3.30E-01	7.76E-01
ENSBTAG00000015015	-4.40E-01	3.44E-01	-3.66E-01	1.96E-01	-7.83E-02	1.00E+00	-3.11E-01	3.07E-01	1.13E-01	1.00E+00
ENSBTAG00000011800	2.38E-01	1.00E+00	-2.17E-01	1.00E+00	-2.11E+00	1.74E-01	-1.37E+00	4.10E-01	6.53E-01	1.00E+00
ENSBTAG00000040569	-4.87E-01	5.03E-01	-1.85E+00	1.13E-09	-1.81E+00	1.44E-09	-1.54E+00	3.58E-07	7.35E-02	1.00E+00
ENSBTAG00000012507	9.74E-01	5.76E-01	7.16E-01	4.50E-01	2.57E-01	1.00E+00	-2.02E-01	1.00E+00	8.30E-01	7.45E-01
ENSBTAG00000017794	1.10E-02	1.00E+00	-4.26E-01	1.00E+00	2.09E-01	1.00E+00	-1.88E-01	1.00E+00	9.24E-01	9.13E-01
ENSBTAG00000006817	-7.74E-02	1.00E+00	-1.77E-02	1.00E+00	4.40E-01	2.72E-01	4.79E-01	2.26E-01	1.84E-01	1.00E+00
ENSBTAG00000006835	6.63E-01	5.15E-01	-5.98E-01	2.86E-01	-1.63E-02	1.00E+00	1.85E-02	1.00E+00	1.14E-01	1.00E+00
ENSBTAG00000012562	-4.62E-01	4.71E-01	-5.86E-01	5.69E-02	-6.22E-01	3.96E-02	-4.14E-01	2.36E-01	3.68E-01	7.01E-01
ENSBTAG00000027766	3.68E-01	8.28E-01	6.22E-01	1.26E-01	-4.16E-02	1.00E+00	-5.91E-02	1.00E+00	-7.02E-01	3.82E-01
ENSBTAG00000009748	1.92E+00	8.23E-01	0.00E+00	1.00E+00	2.55E+00	2.54E-01	2.53E+00	2.71E-01	1.93E+00	8.00E-01
ENSBTAG00000009749	-1.84E+00	2.66E-04	-3.24E+00	5.13E-11	-1.95E+00	7.81E-06	-1.15E+00	9.63E-03	1.34E+00	1.44E-02
ENSBTAG00000019627	4.31E-01	8.23E-01	1.13E-01	1.00E+00	-6.64E-01	1.92E-01	-5.01E-01	3.82E-01	7.57E-01	3.44E-01
ENSBTAG00000027759	1.12E-02	1.00E+00	-1.91E+00	4.69E-01	-1.91E+00	4.28E-01	6.20E-01	9.94E-01	-1.91E+00	8.00E-01
ENSBTAG00000015939	6.49E-01	8.23E-01	1.44E-01	1.00E+00	1.21E+00	4.62E-02	2.46E+00	6.06E-06	4.58E-01	9.69E-01
ENSBTAG00000048149	0.00E+00	1.00E+00	0.00E+00	1.00E+00	-5.13E-15	1.00E+00	0.00E+00	1.00E+00	0.00E+00	1.00E+00
ENSBTAG00000009419	1.70E-02	1.00E+00	9.82E-01	6.49E-01	1.02E+00	5.86E-01	1.24E+00	4.55E-01	1.19E+00	8.27E-01
ENSBTAG00000021338	-9.35E-02	1.00E+00	-1.68E+00	4.12E-11	-1.17E+00	4.56E-06	1.21E-01	9.41E-01	5.89E-01	2.19E-01

ENSBTAG00000021340	-6.90E-01	7.91E-01	-1.95E+00	7.17E-03	-6.18E+00	5.55E-08	-2.24E+00	1.61E-03	-8.90E-02	1.00E+00
ENSBTAG00000021342	3.25E-01	9.32E-01	-8.91E-01	3.38E-02	8.36E-02	1.00E+00	2.85E-03	1.00E+00	-4.31E-01	8.00E-01
ENSBTAG00000021343	2.51E-01	8.85E-01	6.77E-01	1.30E-02	6.71E-01	1.37E-02	6.29E-01	2.57E-02	3.54E-01	6.76E-01
ENSBTAG00000017146	2.65E-02	1.00E+00	-2.59E-01	8.04E-01	-2.24E+00	2.86E-06	-1.97E+00	3.28E-05	4.12E-01	8.02E-01
ENSBTAG00000044429	0.00E+00	1.00E+00	0.00E+00	1.00E+00	-5.13E-15	1.00E+00	0.00E+00	1.00E+00	0.00E+00	1.00E+00
ENSBTAG00000040051	-1.57E-01	1.00E+00	-8.56E-02	1.00E+00	-2.73E-01	4.89E-01	4.41E-02	1.00E+00	-5.01E-01	4.36E-01
ENSBTAG00000018213	-2.80E-01	1.00E+00	-2.02E+00	9.61E-08	-2.97E+00	1.19E-14	-2.67E+00	3.46E-12	1.30E-02	1.00E+00
ENSBTAG00000004456	-3.98E-01	8.57E-01	-3.12E+00	2.68E-11	-4.29E+00	3.00E-17	-3.85E+00	2.46E-15	6.34E-01	5.20E-01
ENSBTAG00000019246	-2.59E-01	9.05E-01	-7.28E-01	1.20E-02	-1.89E+00	2.38E-12	-2.18E+00	1.08E-15	-4.26E-01	5.93E-01
ENSBTAG00000014611	2.70E-01	8.65E-01	-2.88E-01	4.55E-01	-7.12E-01	1.26E-02	-6.07E-01	4.43E-02	9.47E-01	1.10E-02
ENSBTAG00000029925	7.74E-01	1.00E+00	1.05E+00	4.54E-01	1.29E+00	2.62E-01	-1.74E-01	1.00E+00	-3.50E-01	1.00E+00
ENSBTAG00000029881	-1.97E+00	3.60E-01	-4.51E-01	8.95E-01	-9.77E-01	4.32E-01	-4.68E+00	1.85E-03	-1.98E+00	4.29E-01
ENSBTAG00000036369	7.96E-01	1.00E+00	8.83E-01	8.52E-01	6.39E-01	9.79E-01	-1.42E-01	1.00E+00	6.30E-03	1.00E+00
ENSBTAG00000000842	1.78E+00	2.95E-08	7.52E-01	3.28E-02	1.09E+00	6.21E-04	6.26E-01	9.48E-02	1.78E-02	1.00E+00
ENSBTAG00000005193	6.79E-01	4.74E-01	2.28E-01	8.78E-01	-2.12E-01	9.11E-01	7.52E-02	1.00E+00	-2.28E-01	1.00E+00
ENSBTAG00000013989	-1.43E+00	7.71E-01	-4.70E-01	9.50E-01	2.65E-02	1.00E+00	1.58E+00	7.10E-02	-5.37E-01	1.00E+00
ENSBTAG00000013994	5.35E-03	1.00E+00	1.40E+00	5.16E-01	1.15E+00	6.44E-01	-1.91E+00	4.39E-01	6.47E-03	1.00E+00
ENSBTAG00000013162	1.00E+00	1.37E-01	3.44E+00	6.09E-15	7.34E-01	1.40E-01	6.16E-01	2.56E-01	-3.79E-01	9.03E-01
ENSBTAG00000043480	0.00E+00	1.00E+00	0.00E+00	1.00E+00	-5.13E-15	1.00E+00	0.00E+00	1.00E+00	0.00E+00	1.00E+00
ENSBTAG00000042134	0.00E+00	1.00E+00	0.00E+00	1.00E+00	-5.13E-15	1.00E+00	0.00E+00	1.00E+00	0.00E+00	1.00E+00
ENSBTAG00000043462	0.00E+00	1.00E+00	0.00E+00	1.00E+00	-5.13E-15	1.00E+00	0.00E+00	1.00E+00	0.00E+00	1.00E+00
ENSBTAG00000020046	-1.02E-01	1.00E+00	-4.96E-01	2.70E-01	-4.33E-01	3.48E-01	-1.27E-02	1.00E+00	-8.70E-02	1.00E+00
ENSBTAG00000038401	0.00E+00	1.00E+00	0.00E+00	1.00E+00	-5.13E-15	1.00E+00	0.00E+00	1.00E+00	0.00E+00	1.00E+00
ENSBTAG00000001410	9.42E-02	1.00E+00	-2.30E-01	7.47E-01	-2.15E-01	7.79E-01	-1.78E-01	8.85E-01	4.35E-01	7.15E-01
ENSBTAG00000044589	0.00E+00	1.00E+00	0.00E+00	1.00E+00	-5.13E-15	1.00E+00	0.00E+00	1.00E+00	0.00E+00	1.00E+00
ENSBTAG00000016768	-1.96E+00	5.78E-01	1.31E+00	1.99E-01	-3.88E+00	2.28E-02	5.68E-02	1.00E+00	-2.69E-01	1.00E+00
ENSBTAG00000016684	-4.44E-01	5.20E-01	1.59E-01	8.35E-01	5.09E-01	8.39E-02	-3.87E-01	2.71E-01	-2.28E-01	9.45E-01
ENSBTAG00000008804	-4.46E-01	7.03E-01	-1.85E+00	2.39E-07	-1.28E+00	2.19E-04	-1.12E+00	1.65E-03	9.26E-01	6.98E-02
ENSBTAG00000005343	2.89E-01	1.00E+00	-3.31E-01	7.97E-01	-1.02E+00	8.82E-02	-2.63E-01	9.13E-01	1.30E+00	1.57E-01
ENSBTAG00000005340	-6.59E-02	1.00E+00	-1.70E-01	9.29E-01	-3.81E-01	4.37E-01	-1.42E-01	9.94E-01	1.41E-01	1.00E+00
ENSBTAG00000005424	-7.73E-01	1.00E+00	4.26E-01	9.03E-01	-6.41E-01	7.36E-01	-3.83E-02	1.00E+00	-4.56E-01	1.00E+00
ENSBTAG00000002216	-2.71E+00	5.76E-01	-2.71E+00	2.42E-01	-2.71E+00	1.99E-01	-2.71E+00	2.05E-01	-2.71E+00	6.01E-01
ENSBTAG00000000987	-7.24E-01	5.55E-01	-3.45E+00	4.74E-09	-2.04E+00	4.58E-05	-1.46E+00	3.76E-03	-4.35E-02	1.00E+00
ENSBTAG00000000986	-3.37E-01	1.00E+00	-2.00E+00	1.58E-01	-2.79E+00	3.70E-02	-1.32E+00	4.37E-01	-2.98E-02	1.00E+00
ENSBTAG00000000983	-5.53E-01	8.23E-01	-1.38E+00	1.80E-02	-2.18E+00	1.32E-04	-2.19E+00	1.61E-04	-2.46E-01	1.00E+00

ENSBTAG00000016170	-5.43E-01	1.00E+00	1.96E-01	1.00E+00	-1.04E+00	2.51E-01	-1.11E-01	1.00E+00	1.90E-01	1.00E+00
ENSBTAG00000017468	1.07E+00	4.96E-02	1.86E+00	1.87E-06	2.23E+00	9.06E-09	2.26E+00	7.45E-09	1.35E+00	1.28E-02
ENSBTAG00000015244	0.00E+00	1.00E+00	3.98E+00	2.56E-02	3.43E+00	7.33E-02	2.53E+00	2.71E-01	0.00E+00	1.00E+00
ENSBTAG00000032859	-1.23E-02	1.00E+00	-4.79E-01	1.13E-01	-1.91E+00	2.20E-13	-2.74E+00	1.58E-24	2.47E-02	1.00E+00
ENSBTAG00000004901	4.43E-01	8.23E-01	2.33E+00	8.70E-10	1.13E+00	4.74E-03	1.18E+00	3.56E-03	-1.61E-01	1.00E+00
ENSBTAG00000040308	7.68E-01	3.01E-02	1.42E+00	1.51E-08	1.25E+00	6.61E-07	6.97E-01	1.30E-02	-7.41E-01	1.32E-01
ENSBTAG00000043325	0.00E+00	1.00E+00	0.00E+00	1.00E+00	-5.13E-15	1.00E+00	0.00E+00	1.00E+00	0.00E+00	1.00E+00
ENSBTAG00000042684	0.00E+00	1.00E+00	0.00E+00	1.00E+00	-5.13E-15	1.00E+00	0.00E+00	1.00E+00	0.00E+00	1.00E+00
ENSBTAG00000043276	0.00E+00	1.00E+00	0.00E+00	1.00E+00	-5.13E-15	1.00E+00	0.00E+00	1.00E+00	0.00E+00	1.00E+00
ENSBTAG00000006974	-1.89E-01	1.00E+00	1.87E-01	9.14E-01	1.39E+00	2.08E-04	2.06E-01	8.69E-01	2.09E-01	1.00E+00
ENSBTAG00000006755	2.00E-02	1.00E+00	1.77E-01	5.25E-01	2.35E-01	3.38E-01	2.87E-01	2.14E-01	-2.56E-01	6.72E-01
ENSBTAG00000044185	-4.24E-03	1.00E+00	-1.55E+00	2.37E-07	-2.05E+00	9.01E-12	-1.28E+00	1.55E-05	-2.55E-01	9.04E-01
ENSBTAG00000048131	0.00E+00	1.00E+00	0.00E+00	1.00E+00	-5.13E-15	1.00E+00	0.00E+00	1.00E+00	0.00E+00	1.00E+00
ENSBTAG00000032782	-1.02E-01	1.00E+00	-2.81E-01	1.00E+00	1.15E-01	1.00E+00	-3.21E-01	9.52E-01	9.84E-01	5.56E-01
ENSBTAG00000047380	0.00E+00	1.00E+00	1.99E+00	4.43E-01	3.43E+00	7.38E-02	3.39E+00	8.42E-02	1.93E+00	8.00E-01
ENSBTAG00000010089	1.92E+00	8.23E-01	0.00E+00	1.00E+00	1.78E+00	4.79E-01	0.00E+00	1.00E+00	2.71E+00	6.01E-01
ENSBTAG00000001516	1.92E+00	8.23E-01	0.00E+00	1.00E+00	3.05E+00	1.36E-01	1.76E+00	4.97E-01	1.91E+00	8.00E-01
ENSBTAG00000045058	0.00E+00	1.00E+00	0.00E+00	1.00E+00	-5.13E-15	1.00E+00	0.00E+00	1.00E+00	0.00E+00	1.00E+00
ENSBTAG00000010419	-3.56E-01	5.79E-01	-9.39E-01	1.28E-04	-1.07E+00	8.50E-06	-7.27E-01	4.12E-03	-2.21E-01	8.94E-01
ENSBTAG00000005218	3.07E-02	1.00E+00	-4.38E-01	1.85E-01	-8.02E-02	1.00E+00	-8.25E-01	4.21E-03	-4.97E-02	1.00E+00
ENSBTAG00000006564	4.35E-02	1.00E+00	7.12E-01	8.53E-04	8.67E-01	3.23E-05	7.25E-01	8.02E-04	-5.91E-01	7.51E-02
ENSBTAG00000006556	3.09E-01	6.18E-01	1.43E+00	8.26E-12	1.38E+00	3.81E-11	1.13E+00	1.06E-07	-7.77E-02	1.00E+00
ENSBTAG00000009359	-6.02E-03	1.00E+00	3.92E-02	1.00E+00	-3.34E-01	2.50E-01	-7.08E-01	3.59E-03	-2.80E-01	7.84E-01
ENSBTAG00000011905	5.67E-02	1.00E+00	-1.53E-01	8.98E-01	6.57E-01	4.01E-02	-9.98E-03	1.00E+00	-8.32E-02	1.00E+00
ENSBTAG00000009150	2.04E-01	1.00E+00	7.16E-01	2.68E-02	1.04E+00	5.69E-04	7.83E-01	1.51E-02	2.20E-01	1.00E+00
ENSBTAG00000045263	0.00E+00	1.00E+00	0.00E+00	1.00E+00	-5.13E-15	1.00E+00	0.00E+00	1.00E+00	0.00E+00	1.00E+00
ENSBTAG00000009061	5.02E-01	3.53E-01	4.24E-01	1.95E-01	2.69E-01	4.85E-01	2.01E-01	6.99E-01	-2.04E-01	1.00E+00
ENSBTAG00000045299	0.00E+00	1.00E+00	0.00E+00	1.00E+00	-5.13E-15	1.00E+00	0.00E+00	1.00E+00	0.00E+00	1.00E+00
ENSBTAG00000042449	0.00E+00	1.00E+00	0.00E+00	1.00E+00	-5.13E-15	1.00E+00	0.00E+00	1.00E+00	0.00E+00	1.00E+00
ENSBTAG00000019080	1.91E+00	8.23E-01	0.00E+00	1.00E+00	-5.13E-15	1.00E+00	0.00E+00	1.00E+00	1.91E+00	8.00E-01
ENSBTAG00000013033	-1.12E-01	1.00E+00	-1.18E+00	2.84E-07	6.50E-01	3.58E-03	6.03E-01	8.86E-03	-3.05E-01	6.85E-01
ENSBTAG00000013029	-1.82E-01	1.00E+00	7.85E-02	1.00E+00	7.97E-01	1.22E-02	4.49E-01	2.40E-01	6.46E-01	2.94E-01
ENSBTAG00000045007	0.00E+00	1.00E+00	0.00E+00	1.00E+00	-5.13E-15	1.00E+00	0.00E+00	1.00E+00	0.00E+00	1.00E+00
ENSBTAG00000011914	-1.24E+00	6.75E-01	-2.04E+00	8.25E-02	-1.78E+00	1.00E-01	-2.05E-01	1.00E+00	-4.63E-01	1.00E+00
ENSBTAG00000032657	3.41E-01	8.75E-01	2.96E-01	6.07E-01	2.75E-01	6.46E-01	8.41E-01	2.26E-02	1.41E-01	1.00E+00

ENSBTAG00000044661	0.00E+00	1.00E+00	0.00E+00	1.00E+00	-5.13E-15	1.00E+00	0.00E+00	1.00E+00	0.00E+00	1.00E+00
ENSBTAG00000000700	8.00E-04	1.00E+00	-2.24E-01	6.17E-01	-2.71E-01	4.79E-01	1.91E-01	7.22E-01	5.74E-01	2.75E-01
ENSBTAG00000047961	0.00E+00	1.00E+00	0.00E+00	1.00E+00	-5.13E-15	1.00E+00	0.00E+00	1.00E+00	0.00E+00	1.00E+00
ENSBTAG00000008193	8.11E-01	7.18E-01	3.50E+00	1.33E-12	3.54E+00	3.85E-13	2.37E+00	9.37E-06	1.81E+00	2.75E-02
ENSBTAG00000014855	2.18E+00	3.61E-15	4.90E+00	1.03E-65	4.92E+00	2.31E-66	4.63E+00	1.92E-60	2.08E+00	3.39E-13
ENSBTAG00000004275	-1.21E-01	1.00E+00	4.97E-01	1.43E-01	-4.75E-02	1.00E+00	1.09E-01	1.00E+00	-4.28E-01	6.51E-01
ENSBTAG00000010919	6.10E-02	1.00E+00	-4.70E-01	2.45E-02	-4.20E-02	1.00E+00	5.74E-02	1.00E+00	-4.08E-02	1.00E+00
ENSBTAG00000047242	-1.61E+00	3.47E-01	-1.91E+00	5.87E-02	-2.66E+00	8.05E-03	-2.67E+00	8.84E-03	-2.49E+00	1.36E-01
ENSBTAG00000045396	0.00E+00	1.00E+00	0.00E+00	1.00E+00	-5.13E-15	1.00E+00	0.00E+00	1.00E+00	0.00E+00	1.00E+00
ENSBTAG00000002914	-2.56E-01	9.61E-01	-1.06E+00	7.76E-04	-1.14E+00	2.11E-04	-8.74E-01	6.44E-03	-4.07E-01	6.89E-01
ENSBTAG00000046636	-4.32E-01	8.92E-01	-5.73E-01	3.53E-01	-3.23E-01	6.93E-01	-1.72E-02	1.00E+00	4.44E-02	1.00E+00
ENSBTAG00000027716	0.00E+00	1.00E+00	0.00E+00	1.00E+00	-5.13E-15	1.00E+00	0.00E+00	1.00E+00	0.00E+00	1.00E+00
ENSBTAG00000010568	-8.09E-02	1.00E+00	-1.31E+00	4.83E-06	-2.68E-01	5.27E-01	-6.21E-01	5.15E-02	-9.99E-02	1.00E+00
ENSBTAG00000020308	3.14E-01	3.75E-01	9.72E-01	7.30E-09	1.24E+00	9.98E-14	1.20E+00	6.55E-13	1.45E-02	1.00E+00
ENSBTAG00000044741	0.00E+00	1.00E+00	0.00E+00	1.00E+00	-5.13E-15	1.00E+00	0.00E+00	1.00E+00	0.00E+00	1.00E+00
ENSBTAG00000015566	1.95E-01	1.00E+00	9.58E-01	1.53E-02	9.91E-01	1.16E-02	9.20E-01	2.37E-02	1.99E-01	1.00E+00
ENSBTAG00000038174	-7.26E-02	1.00E+00	-6.72E-01	3.58E-01	-2.96E-01	8.28E-01	-1.81E-01	1.00E+00	1.35E-01	1.00E+00
ENSBTAG00000007129	3.42E-01	8.23E-01	4.10E-01	3.21E-01	7.11E-01	3.29E-02	1.13E+00	2.66E-04	1.32E-01	1.00E+00
ENSBTAG00000000802	1.44E-01	1.00E+00	2.41E-01	4.86E-01	-6.94E-02	1.00E+00	5.27E-02	1.00E+00	-6.31E-01	1.17E-01
ENSBTAG00000015824	1.28E-01	1.00E+00	-1.99E-01	7.52E-01	6.64E-01	2.83E-02	8.19E-01	5.57E-03	5.23E-01	4.34E-01
ENSBTAG00000015821	8.33E-01	4.54E-01	1.10E+00	3.95E-02	1.01E+00	6.16E-02	2.29E+00	1.65E-06	5.66E-01	8.00E-01
ENSBTAG00000021048	1.23E+00	2.34E-01	7.65E-01	3.17E-01	-4.75E-01	6.34E-01	3.72E-01	7.97E-01	-5.22E-01	9.05E-01
ENSBTAG00000043981	9.63E-02	1.00E+00	-8.08E-01	2.37E-04	-1.35E+00	2.25E-10	-7.30E-01	1.16E-03	-4.29E-02	1.00E+00
ENSBTAG00000001987	-2.24E-01	9.60E-01	-1.75E+00	8.15E-12	-1.89E+00	1.62E-13	-1.61E+00	3.73E-10	3.34E-02	1.00E+00
ENSBTAG00000045098	0.00E+00	1.00E+00	0.00E+00	1.00E+00	-5.13E-15	1.00E+00	0.00E+00	1.00E+00	0.00E+00	1.00E+00
ENSBTAG00000000426	-6.92E-01	8.23E-01	-4.86E-01	6.66E-01	8.26E-02	1.00E+00	6.31E-01	4.01E-01	-2.12E-01	1.00E+00
ENSBTAG00000005278	-1.88E-01	1.00E+00	-2.32E+00	2.79E-05	-2.04E+00	2.23E-04	-2.38E-01	9.63E-01	-1.90E-01	1.00E+00
ENSBTAG00000043738	0.00E+00	1.00E+00	0.00E+00	1.00E+00	-5.13E-15	1.00E+00	3.40E+00	8.29E-02	0.00E+00	1.00E+00
ENSBTAG00000043540	0.00E+00	1.00E+00	0.00E+00	1.00E+00	-5.13E-15	1.00E+00	0.00E+00	1.00E+00	0.00E+00	1.00E+00
ENSBTAG00000016074	2.62E-01	8.23E-01	5.76E-01	2.05E-02	5.47E-01	2.89E-02	4.13E-01	1.36E-01	-1.08E-01	1.00E+00
ENSBTAG00000045658	0.00E+00	1.00E+00	0.00E+00	1.00E+00	-5.13E-15	1.00E+00	0.00E+00	1.00E+00	0.00E+00	1.00E+00
ENSBTAG00000048023	0.00E+00	1.00E+00	0.00E+00	1.00E+00	-5.13E-15	1.00E+00	0.00E+00	1.00E+00	0.00E+00	1.00E+00
ENSBTAG00000027722	2.24E-01	1.00E+00	1.20E+00	1.43E-04	1.52E+00	9.60E-07	1.44E+00	5.02E-06	-4.57E-01	6.20E-01
ENSBTAG00000043305	0.00E+00	1.00E+00	0.00E+00	1.00E+00	-5.13E-15	1.00E+00	0.00E+00	1.00E+00	0.00E+00	1.00E+00
ENSBTAG00000027723	-7.80E-01	1.00E+00	2.23E+00	4.69E-02	2.54E+00	1.15E-02	3.38E-01	1.00E+00	1.79E-02	1.00E+00

ENSBTAG00000001607	5.40E-01	2.81E-01	1.92E+00	4.00E-13	1.15E+00	1.78E-05	1.20E+00	8.26E-06	-5.17E-01	4.10E-01
ENSBTAG00000042184	0.00E+00	1.00E+00	0.00E+00	1.00E+00	1.78E+00	4.79E-01	0.00E+00	1.00E+00	0.00E+00	1.00E+00
ENSBTAG00000038968	0.00E+00	1.00E+00	1.98E+00	4.43E-01	-5.13E-15	1.00E+00	0.00E+00	1.00E+00	0.00E+00	1.00E+00
ENSBTAG00000006322	5.15E-01	1.99E-01	1.62E+00	1.14E-12	1.05E+00	6.37E-06	7.91E-01	1.12E-03	2.12E-01	9.04E-01
ENSBTAG00000020573	4.92E-01	8.03E-01	1.23E+00	3.10E-03	6.79E-01	1.58E-01	3.71E-01	5.75E-01	3.87E-01	8.88E-01
ENSBTAG00000013366	4.31E-01	1.00E+00	9.52E-01	2.30E-01	1.46E+00	2.12E-02	1.01E+00	1.85E-01	-1.10E-01	1.00E+00
ENSBTAG00000038873	0.00E+00	1.00E+00	0.00E+00	1.00E+00	-5.13E-15	1.00E+00	0.00E+00	1.00E+00	0.00E+00	1.00E+00
ENSBTAG00000008310	-4.20E-02	1.00E+00	1.97E-02	1.00E+00	2.72E-01	3.03E-01	2.36E-01	4.25E-01	-2.06E-01	8.34E-01
ENSBTAG00000004771	-1.77E-01	1.00E+00	-1.44E+00	6.64E-08	-9.80E-01	2.44E-04	6.22E-02	1.00E+00	2.60E-01	8.40E-01
ENSBTAG00000032339	1.92E+00	8.23E-01	0.00E+00	1.00E+00	-5.13E-15	1.00E+00	0.00E+00	1.00E+00	0.00E+00	1.00E+00
ENSBTAG00000001917	1.43E+00	7.57E-01	6.03E-01	9.45E-01	-9.16E-01	8.10E-01	1.45E+00	3.29E-01	-7.72E-01	1.00E+00
ENSBTAG00000001922	-2.53E-02	1.00E+00	4.08E-01	1.16E-01	3.44E-01	2.08E-01	2.20E-01	5.17E-01	-4.02E-01	4.68E-01
ENSBTAG00000005356	-1.65E-01	1.00E+00	-3.99E-01	2.67E-01	-1.23E+00	1.52E-05	-1.45E+00	3.56E-07	4.34E-02	1.00E+00
ENSBTAG00000005349	9.04E-02	1.00E+00	4.65E-01	9.33E-02	5.04E-01	6.28E-02	2.28E-01	5.48E-01	-4.01E-01	5.37E-01
ENSBTAG00000042335	0.00E+00	1.00E+00	0.00E+00	1.00E+00	-5.13E-15	1.00E+00	0.00E+00	1.00E+00	0.00E+00	1.00E+00
ENSBTAG00000042354	0.00E+00	1.00E+00	0.00E+00	1.00E+00	-5.13E-15	1.00E+00	0.00E+00	1.00E+00	0.00E+00	1.00E+00
ENSBTAG00000020890	2.53E-01	1.00E+00	-1.00E+00	1.70E-01	-1.37E+00	4.07E-02	-4.45E-01	6.43E-01	1.18E+00	1.51E-01
ENSBTAG00000011910	-4.98E-01	9.59E-01	-2.61E-01	9.46E-01	-7.60E-01	3.15E-01	3.71E-02	1.00E+00	-5.79E-01	8.41E-01
ENSBTAG00000016320	8.85E-01	7.16E-01	7.77E-01	4.18E-01	1.44E+00	5.09E-02	8.17E-01	3.85E-01	-3.13E-01	1.00E+00
ENSBTAG00000004863	1.86E-01	1.00E+00	-6.15E-01	5.49E-02	-4.67E-01	1.73E-01	-2.95E-01	4.92E-01	3.13E-02	1.00E+00
ENSBTAG00000004862	2.76E-01	1.00E+00	-8.62E-01	1.33E-01	8.38E-01	5.77E-02	3.04E-01	7.17E-01	7.56E-01	4.32E-01
ENSBTAG00000004861	-6.67E-02	1.00E+00	3.16E-01	2.00E-01	2.81E-01	2.72E-01	2.60E-01	3.39E-01	-3.78E-01	4.28E-01
ENSBTAG00000039405	0.00E+00	1.00E+00	0.00E+00	1.00E+00	-5.13E-15	1.00E+00	1.76E+00	4.97E-01	0.00E+00	1.00E+00
ENSBTAG00000048045	0.00E+00	1.00E+00	0.00E+00	1.00E+00	-5.13E-15	1.00E+00	0.00E+00	1.00E+00	0.00E+00	1.00E+00
ENSBTAG00000040252	0.00E+00	1.00E+00	0.00E+00	1.00E+00	-5.13E-15	1.00E+00	0.00E+00	1.00E+00	0.00E+00	1.00E+00
ENSBTAG00000038641	0.00E+00	1.00E+00	0.00E+00	1.00E+00	-5.13E-15	1.00E+00	0.00E+00	1.00E+00	0.00E+00	1.00E+00
ENSBTAG00000046894	0.00E+00	1.00E+00	0.00E+00	1.00E+00	-5.13E-15	1.00E+00	0.00E+00	1.00E+00	0.00E+00	1.00E+00
ENSBTAG00000021150	-1.91E+00	8.23E-01	-1.91E+00	4.69E-01	-1.37E-01	1.00E+00	-1.91E+00	4.39E-01	-1.91E+00	8.00E-01
ENSBTAG00000019768	0.00E+00	1.00E+00	0.00E+00	1.00E+00	-5.13E-15	1.00E+00	1.76E+00	4.97E-01	0.00E+00	1.00E+00
ENSBTAG00000040285	-1.91E+00	8.23E-01	-1.91E+00	4.69E-01	-1.91E+00	4.28E-01	-1.91E+00	4.39E-01	8.15E-01	1.00E+00
ENSBTAG00000048176	0.00E+00	1.00E+00	0.00E+00	1.00E+00	-5.13E-15	1.00E+00	0.00E+00	1.00E+00	0.00E+00	1.00E+00
ENSBTAG00000045639	-1.91E+00	8.23E-01	-1.91E+00	4.69E-01	-1.91E+00	4.28E-01	-1.41E-01	1.00E+00	7.05E-03	1.00E+00
ENSBTAG00000037384	0.00E+00	1.00E+00	0.00E+00	1.00E+00	-5.13E-15	1.00E+00	0.00E+00	1.00E+00	0.00E+00	1.00E+00
ENSBTAG00000026826	1.91E+00	8.23E-01	0.00E+00	1.00E+00	-5.13E-15	1.00E+00	1.76E+00	4.97E-01	1.91E+00	8.00E-01
ENSBTAG00000038317	6.04E-03	1.00E+00	-1.91E+00	4.69E-01	-1.91E+00	4.28E-01	-1.91E+00	4.39E-01	-1.91E+00	8.00E-01

ENSBTAG00000038245	0.00E+00	1.00E+00	0.00E+00	1.00E+00	-5.13E-15	1.00E+00	0.00E+00	1.00E+00	3.22E+00	4.34E-01
ENSBTAG00000047850	1.92E+00	8.23E-01	1.98E+00	4.43E-01	-5.13E-15	1.00E+00	0.00E+00	1.00E+00	3.88E+00	2.05E-01
ENSBTAG00000038598	-4.83E-01	9.32E-01	-2.55E+00	2.85E-05	-4.79E+00	3.07E-11	-5.71E+00	7.48E-13	1.05E-01	1.00E+00
ENSBTAG00000037921	-1.62E-01	1.00E+00	-2.45E+00	8.46E-06	-4.63E+00	2.60E-13	-4.48E+00	8.66E-13	-3.24E-02	1.00E+00
ENSBTAG00000043396	-1.52E+00	3.33E-01	-2.62E+00	8.87E-03	-5.41E+00	3.15E-05	-5.41E+00	3.58E-05	-2.70E+00	7.05E-02
ENSBTAG00000016142	2.13E-01	1.00E+00	-1.68E+00	1.37E-05	-2.82E+00	9.28E-13	-2.94E+00	1.90E-13	-2.34E-01	1.00E+00
ENSBTAG00000016140	-1.50E-01	1.00E+00	-2.30E+00	3.32E-17	-3.04E+00	1.05E-27	-2.11E+00	6.59E-15	-1.69E-01	1.00E+00
ENSBTAG00000021483	-1.13E-01	1.00E+00	1.02E-01	1.00E+00	3.40E-01	3.56E-01	4.70E-01	1.53E-01	-1.05E-01	1.00E+00
ENSBTAG00000032079	8.78E-01	6.65E-01	-4.57E-01	8.34E-01	-4.37E-02	1.00E+00	1.15E+00	1.06E-01	-7.43E-01	8.77E-01
ENSBTAG00000046099	0.00E+00	1.00E+00	0.00E+00	1.00E+00	-5.13E-15	1.00E+00	0.00E+00	1.00E+00	0.00E+00	1.00E+00
ENSBTAG00000047652	0.00E+00	1.00E+00	1.99E+00	4.43E-01	1.78E+00	4.79E-01	0.00E+00	1.00E+00	0.00E+00	1.00E+00
ENSBTAG00000002289	-7.87E-01	1.00E+00	6.03E-01	9.45E-01	7.23E-01	8.24E-01	-9.41E-01	8.06E-01	-2.70E+00	6.01E-01
ENSBTAG00000020289	5.23E-01	1.00E+00	-3.56E+00	2.01E-03	-2.58E-01	1.00E+00	7.55E-01	4.23E-01	1.09E+00	5.09E-01
ENSBTAG00000003638	3.90E-01	1.00E+00	-1.91E+00	6.97E-02	-1.51E+00	1.24E-01	3.41E-01	9.13E-01	9.14E-01	6.47E-01
ENSBTAG00000038518	0.00E+00	1.00E+00	1.98E+00	4.43E-01	-5.13E-15	1.00E+00	0.00E+00	1.00E+00	0.00E+00	1.00E+00
ENSBTAG00000018490	0.00E+00	1.00E+00	0.00E+00	1.00E+00	-5.13E-15	1.00E+00	0.00E+00	1.00E+00	0.00E+00	1.00E+00
ENSBTAG00000032056	1.19E+00	8.63E-01	-7.16E-01	9.45E-01	-2.70E+00	1.99E-01	1.23E+00	4.64E-01	6.50E-03	1.00E+00
ENSBTAG00000026441	0.00E+00	1.00E+00	0.00E+00	1.00E+00	-5.13E-15	1.00E+00	0.00E+00	1.00E+00	0.00E+00	1.00E+00
ENSBTAG00000047104	0.00E+00	1.00E+00	0.00E+00	1.00E+00	-5.13E-15	1.00E+00	0.00E+00	1.00E+00	0.00E+00	1.00E+00
ENSBTAG00000045599	0.00E+00	1.00E+00	0.00E+00	1.00E+00	-5.13E-15	1.00E+00	0.00E+00	1.00E+00	0.00E+00	1.00E+00
ENSBTAG00000043138	0.00E+00	1.00E+00	0.00E+00	1.00E+00	-5.13E-15	1.00E+00	0.00E+00	1.00E+00	0.00E+00	1.00E+00
ENSBTAG00000032052	0.00E+00	1.00E+00	0.00E+00	1.00E+00	-5.13E-15	1.00E+00	0.00E+00	1.00E+00	0.00E+00	1.00E+00
ENSBTAG00000019144	0.00E+00	1.00E+00	0.00E+00	1.00E+00	-5.13E-15	1.00E+00	0.00E+00	1.00E+00	0.00E+00	1.00E+00
ENSBTAG00000032050	0.00E+00	1.00E+00	0.00E+00	1.00E+00	-5.13E-15	1.00E+00	0.00E+00	1.00E+00	0.00E+00	1.00E+00
ENSBTAG00000037603	0.00E+00	1.00E+00	0.00E+00	1.00E+00	1.78E+00	4.79E-01	0.00E+00	1.00E+00	1.91E+00	8.00E-01
ENSBTAG00000038031	0.00E+00	1.00E+00	0.00E+00	1.00E+00	-5.13E-15	1.00E+00	2.53E+00	2.71E-01	0.00E+00	1.00E+00
ENSBTAG00000036031	0.00E+00	1.00E+00	0.00E+00	1.00E+00	-5.13E-15	1.00E+00	0.00E+00	1.00E+00	0.00E+00	1.00E+00
ENSBTAG00000042988	0.00E+00	1.00E+00	0.00E+00	1.00E+00	-5.13E-15	1.00E+00	0.00E+00	1.00E+00	0.00E+00	1.00E+00
ENSBTAG00000035675	0.00E+00	1.00E+00	0.00E+00	1.00E+00	-5.13E-15	1.00E+00	1.76E+00	4.97E-01	0.00E+00	1.00E+00
ENSBTAG00000015791	0.00E+00	1.00E+00	0.00E+00	1.00E+00	-5.13E-15	1.00E+00	0.00E+00	1.00E+00	0.00E+00	1.00E+00
ENSBTAG00000047025	0.00E+00	1.00E+00	0.00E+00	1.00E+00	-5.13E-15	1.00E+00	0.00E+00	1.00E+00	0.00E+00	1.00E+00
ENSBTAG00000021147	0.00E+00	1.00E+00	0.00E+00	1.00E+00	-5.13E-15	1.00E+00	0.00E+00	1.00E+00	1.91E+00	8.00E-01
ENSBTAG00000046196	0.00E+00	1.00E+00	0.00E+00	1.00E+00	-5.13E-15	1.00E+00	0.00E+00	1.00E+00	1.91E+00	8.00E-01
ENSBTAG00000004443	4.61E-01	1.00E+00	3.09E+00	1.30E-06	1.57E+00	2.11E-02	3.59E-01	8.70E-01	1.32E-01	1.00E+00
ENSBTAG00000037937	5.18E-01	8.92E-01	2.80E+00	4.74E-07	2.23E+00	5.67E-05	5.58E-01	4.91E-01	1.56E-01	1.00E+00

ENSBTAG00000037991	5.50E-01	2.30E-01	1.94E+00	6.28E-15	2.08E+00	4.67E-17	1.80E+00	6.04E-13	-2.85E-01	8.00E-01
ENSBTAG00000024781	2.64E-01	7.39E-01	1.15E+00	7.38E-09	1.46E+00	1.38E-13	1.40E+00	1.87E-12	-9.81E-02	1.00E+00
ENSBTAG00000015405	-8.26E-02	1.00E+00	-1.26E+00	8.73E-06	-8.29E-01	3.76E-03	-9.07E-01	1.63E-03	1.80E-02	1.00E+00
ENSBTAG00000015403	-5.48E-02	1.00E+00	-3.74E-01	2.82E-01	-7.43E-01	9.42E-03	-5.26E-01	9.65E-02	4.72E-01	4.76E-01
ENSBTAG00000000694	-1.74E-02	1.00E+00	-1.63E-01	7.36E-01	-8.16E-02	1.00E+00	1.41E-01	8.20E-01	-5.70E-02	1.00E+00
ENSBTAG00000000696	5.24E-02	1.00E+00	-5.74E-02	1.00E+00	-3.18E-01	2.16E-01	5.45E-03	1.00E+00	-2.04E-01	8.54E-01
ENSBTAG00000000697	7.02E-01	1.49E-01	2.15E+00	2.48E-14	2.51E+00	2.98E-19	1.74E+00	1.33E-09	2.72E-01	9.14E-01
ENSBTAG00000009255	4.54E+00	2.76E-02	4.44E+00	6.76E-03	4.52E+00	4.04E-03	3.69E+00	4.70E-02	3.91E+00	2.00E-01
ENSBTAG00000015655	-4.71E-02	1.00E+00	1.97E+00	4.39E-13	1.65E+00	1.34E-09	1.40E+00	4.70E-07	1.82E-01	1.00E+00
ENSBTAG00000003567	0.00E+00	1.00E+00	0.00E+00	1.00E+00	-5.13E-15	1.00E+00	0.00E+00	1.00E+00	0.00E+00	1.00E+00
ENSBTAG00000003566	0.00E+00	1.00E+00	0.00E+00	1.00E+00	-5.13E-15	1.00E+00	0.00E+00	1.00E+00	0.00E+00	1.00E+00
ENSBTAG00000003565	0.00E+00	1.00E+00	0.00E+00	1.00E+00	-5.13E-15	1.00E+00	0.00E+00	1.00E+00	0.00E+00	1.00E+00
ENSBTAG00000003564	0.00E+00	1.00E+00	0.00E+00	1.00E+00	-5.13E-15	1.00E+00	0.00E+00	1.00E+00	0.00E+00	1.00E+00
ENSBTAG00000015628	-6.40E-02	1.00E+00	-4.34E-01	5.13E-02	-5.23E-01	1.42E-02	-1.59E-01	6.63E-01	9.07E-01	1.72E-04
ENSBTAG00000044799	1.91E+00	8.23E-01	2.79E+00	2.10E-01	-5.13E-15	1.00E+00	0.00E+00	1.00E+00	1.91E+00	8.00E-01
ENSBTAG00000009440	0.00E+00	1.00E+00	0.00E+00	1.00E+00	-5.13E-15	1.00E+00	1.76E+00	4.97E-01	0.00E+00	1.00E+00
ENSBTAG00000019754	-3.14E-01	9.38E-01	-6.09E-01	1.50E-01	-6.12E-01	1.38E-01	2.98E-02	1.00E+00	8.42E-04	1.00E+00
ENSBTAG00000005236	5.13E-03	1.00E+00	2.71E+00	3.94E-02	-1.25E-01	1.00E+00	-1.48E-01	1.00E+00	8.10E-01	1.00E+00
ENSBTAG00000005228	0.00E+00	1.00E+00	1.98E+00	4.43E-01	2.55E+00	2.54E-01	0.00E+00	1.00E+00	1.93E+00	8.00E-01
ENSBTAG00000047176	0.00E+00	1.00E+00	0.00E+00	1.00E+00	-5.13E-15	1.00E+00	0.00E+00	1.00E+00	0.00E+00	1.00E+00
ENSBTAG00000020515	7.93E-01	5.23E-03	1.75E+00	3.10E-15	1.77E+00	7.85E-16	1.39E+00	5.08E-10	4.66E-01	3.49E-01
ENSBTAG00000027642	5.59E-01	3.47E-01	9.30E-01	2.08E-03	9.30E-01	1.87E-03	3.66E-01	3.74E-01	2.39E-01	9.73E-01
ENSBTAG00000008892	0.00E+00	1.00E+00	1.99E+00	4.43E-01	1.78E+00	4.79E-01	0.00E+00	1.00E+00	0.00E+00	1.00E+00
ENSBTAG00000021590	0.00E+00	1.00E+00	0.00E+00	1.00E+00	-5.13E-15	1.00E+00	0.00E+00	1.00E+00	0.00E+00	1.00E+00
ENSBTAG00000038977	0.00E+00	1.00E+00	0.00E+00	1.00E+00	-5.13E-15	1.00E+00	0.00E+00	1.00E+00	0.00E+00	1.00E+00
ENSBTAG00000027443	0.00E+00	1.00E+00	0.00E+00	1.00E+00	-5.13E-15	1.00E+00	0.00E+00	1.00E+00	0.00E+00	1.00E+00
ENSBTAG00000000368	2.72E+00	5.76E-01	0.00E+00	1.00E+00	-5.13E-15	1.00E+00	0.00E+00	1.00E+00	0.00E+00	1.00E+00
ENSBTAG00000032276	0.00E+00	1.00E+00	1.98E+00	4.43E-01	-5.13E-15	1.00E+00	0.00E+00	1.00E+00	0.00E+00	1.00E+00
ENSBTAG00000048223	0.00E+00	1.00E+00	0.00E+00	1.00E+00	-5.13E-15	1.00E+00	0.00E+00	1.00E+00	0.00E+00	1.00E+00
ENSBTAG00000047246	0.00E+00	1.00E+00	0.00E+00	1.00E+00	-5.13E-15	1.00E+00	0.00E+00	1.00E+00	0.00E+00	1.00E+00
ENSBTAG00000032280	0.00E+00	1.00E+00	1.98E+00	4.43E-01	-5.13E-15	1.00E+00	0.00E+00	1.00E+00	0.00E+00	1.00E+00
ENSBTAG00000023125	0.00E+00	1.00E+00	0.00E+00	1.00E+00	-5.13E-15	1.00E+00	0.00E+00	1.00E+00	0.00E+00	1.00E+00
ENSBTAG00000000351	0.00E+00	1.00E+00	0.00E+00	1.00E+00	-5.13E-15	1.00E+00	0.00E+00	1.00E+00	0.00E+00	1.00E+00
ENSBTAG00000032274	0.00E+00	1.00E+00	0.00E+00	1.00E+00	-5.13E-15	1.00E+00	0.00E+00	1.00E+00	0.00E+00	1.00E+00
ENSBTAG00000037505	0.00E+00	1.00E+00	0.00E+00	1.00E+00	-5.13E-15	1.00E+00	0.00E+00	1.00E+00	0.00E+00	1.00E+00

ENSBTAG00000000118	0.00E+00	1.00E+00	0.00E+00	1.00E+00	-5.13E-15	1.00E+00	0.00E+00	1.00E+00	0.00E+00	1.00E+00
ENSBTAG00000038452	0.00E+00	1.00E+00	0.00E+00	1.00E+00	-5.13E-15	1.00E+00	0.00E+00	1.00E+00	0.00E+00	1.00E+00
ENSBTAG00000020952	0.00E+00	1.00E+00	0.00E+00	1.00E+00	-5.13E-15	1.00E+00	0.00E+00	1.00E+00	0.00E+00	1.00E+00
ENSBTAG00000027949	0.00E+00	1.00E+00	0.00E+00	1.00E+00	-5.13E-15	1.00E+00	0.00E+00	1.00E+00	0.00E+00	1.00E+00
ENSBTAG00000040480	0.00E+00	1.00E+00	0.00E+00	1.00E+00	-5.13E-15	1.00E+00	0.00E+00	1.00E+00	0.00E+00	1.00E+00
ENSBTAG00000036326	0.00E+00	1.00E+00	0.00E+00	1.00E+00	-5.13E-15	1.00E+00	0.00E+00	1.00E+00	0.00E+00	1.00E+00
ENSBTAG00000043009	0.00E+00	1.00E+00	0.00E+00	1.00E+00	-5.13E-15	1.00E+00	0.00E+00	1.00E+00	0.00E+00	1.00E+00
ENSBTAG00000036328	0.00E+00	1.00E+00	0.00E+00	1.00E+00	-5.13E-15	1.00E+00	0.00E+00	1.00E+00	0.00E+00	1.00E+00
ENSBTAG00000009044	0.00E+00	1.00E+00	0.00E+00	1.00E+00	-5.13E-15	1.00E+00	0.00E+00	1.00E+00	0.00E+00	1.00E+00
ENSBTAG00000027430	0.00E+00	1.00E+00	0.00E+00	1.00E+00	-5.13E-15	1.00E+00	2.53E+00	2.71E-01	0.00E+00	1.00E+00
ENSBTAG00000032262	0.00E+00	1.00E+00	1.98E+00	4.43E-01	-5.13E-15	1.00E+00	0.00E+00	1.00E+00	0.00E+00	1.00E+00
ENSBTAG00000032259	0.00E+00	1.00E+00	0.00E+00	1.00E+00	1.78E+00	4.79E-01	2.54E+00	2.71E-01	0.00E+00	1.00E+00
ENSBTAG00000032256	0.00E+00	1.00E+00	0.00E+00	1.00E+00	1.78E+00	4.79E-01	0.00E+00	1.00E+00	0.00E+00	1.00E+00
ENSBTAG00000032255	0.00E+00	1.00E+00	0.00E+00	1.00E+00	-5.13E-15	1.00E+00	0.00E+00	1.00E+00	0.00E+00	1.00E+00
ENSBTAG00000023912	0.00E+00	1.00E+00	0.00E+00	1.00E+00	-5.13E-15	1.00E+00	0.00E+00	1.00E+00	0.00E+00	1.00E+00
ENSBTAG00000026929	0.00E+00	1.00E+00	0.00E+00	1.00E+00	-5.13E-15	1.00E+00	1.76E+00	4.97E-01	0.00E+00	1.00E+00
ENSBTAG00000027889	-1.91E+00	8.23E-01	-1.91E+00	4.69E-01	-1.91E+00	4.28E-01	-1.91E+00	4.39E-01	-1.91E+00	8.00E-01
ENSBTAG00000026924	0.00E+00	1.00E+00	0.00E+00	1.00E+00	-5.13E-15	1.00E+00	0.00E+00	1.00E+00	0.00E+00	1.00E+00
ENSBTAG00000039029	-1.91E+00	8.23E-01	-1.91E+00	4.69E-01	-1.91E+00	4.28E-01	-1.91E+00	4.39E-01	-1.91E+00	8.00E-01
ENSBTAG00000046618	0.00E+00	1.00E+00	0.00E+00	1.00E+00	-5.13E-15	1.00E+00	0.00E+00	1.00E+00	0.00E+00	1.00E+00
ENSBTAG00000026922	-1.91E+00	8.23E-01	-1.91E+00	4.69E-01	-1.91E+00	4.28E-01	-1.91E+00	4.39E-01	-1.91E+00	8.00E-01
ENSBTAG00000016119	0.00E+00	1.00E+00	0.00E+00	1.00E+00	-5.13E-15	1.00E+00	0.00E+00	1.00E+00	0.00E+00	1.00E+00
ENSBTAG00000006815	2.67E-01	9.17E-01	2.17E-01	6.85E-01	7.00E-01	2.18E-02	1.32E+00	3.31E-06	-3.82E-01	7.15E-01
ENSBTAG00000046764	-1.91E+00	8.23E-01	-1.91E+00	4.69E-01	-1.91E+00	4.28E-01	-1.91E+00	4.39E-01	-1.91E+00	8.00E-01
ENSBTAG00000047088	3.66E-01	7.09E-01	8.69E-01	2.27E-03	-2.98E-02	1.00E+00	-5.25E-01	1.07E-01	4.95E-01	4.49E-01
ENSBTAG00000005609	5.17E-01	1.00E+00	-7.22E-01	9.45E-01	-1.48E-01	1.00E+00	-9.40E-01	8.06E-01	1.45E-03	1.00E+00
ENSBTAG00000000953	0.00E+00	1.00E+00	0.00E+00	1.00E+00	-5.13E-15	1.00E+00	0.00E+00	1.00E+00	0.00E+00	1.00E+00
ENSBTAG00000002043	0.00E+00	1.00E+00	0.00E+00	1.00E+00	-5.13E-15	1.00E+00	0.00E+00	1.00E+00	0.00E+00	1.00E+00
ENSBTAG00000038536	-3.67E-01	5.10E-01	5.44E-01	2.43E-02	4.09E-01	1.15E-01	-1.47E-01	7.86E-01	8.32E-02	1.00E+00
ENSBTAG00000005251	-1.64E-02	1.00E+00	3.07E-01	5.73E-01	1.65E-02	1.00E+00	-6.01E-01	1.60E-01	6.55E-03	1.00E+00
ENSBTAG00000037465	-1.82E-02	1.00E+00	-8.20E-01	7.75E-04	-7.90E-01	1.20E-03	-5.81E-01	2.65E-02	1.35E-01	1.00E+00
ENSBTAG00000046541	0.00E+00	1.00E+00	0.00E+00	1.00E+00	1.78E+00	4.79E-01	1.76E+00	4.97E-01	1.93E+00	8.00E-01
ENSBTAG00000048214	0.00E+00	1.00E+00	0.00E+00	1.00E+00	-5.13E-15	1.00E+00	0.00E+00	1.00E+00	0.00E+00	1.00E+00
ENSBTAG00000046741	0.00E+00	1.00E+00	0.00E+00	1.00E+00	-5.13E-15	1.00E+00	0.00E+00	1.00E+00	0.00E+00	1.00E+00
ENSBTAG00000046489	-3.53E-01	1.00E+00	-3.58E+00	6.74E-02	-3.58E+00	4.60E-02	-3.58E+00	4.97E-02	-3.58E+00	3.10E-01

ENSBTAG00000048166	0.00E+00	1.00E+00	1.98E+00	4.43E-01	-5.13E-15	1.00E+00	0.00E+00	1.00E+00	0.00E+00	1.00E+00
ENSBTAG00000046762	0.00E+00	1.00E+00	0.00E+00	1.00E+00	-5.13E-15	1.00E+00	0.00E+00	1.00E+00	1.93E+00	8.00E-01
ENSBTAG00000005671	0.00E+00	1.00E+00	0.00E+00	1.00E+00	-5.13E-15	1.00E+00	0.00E+00	1.00E+00	0.00E+00	1.00E+00
ENSBTAG00000025266	0.00E+00	1.00E+00	0.00E+00	1.00E+00	-5.13E-15	1.00E+00	0.00E+00	1.00E+00	0.00E+00	1.00E+00
ENSBTAG00000017647	0.00E+00	1.00E+00	0.00E+00	1.00E+00	-5.13E-15	1.00E+00	1.76E+00	4.97E-01	0.00E+00	1.00E+00
ENSBTAG00000032224	0.00E+00	1.00E+00	0.00E+00	1.00E+00	-5.13E-15	1.00E+00	0.00E+00	1.00E+00	0.00E+00	1.00E+00
ENSBTAG00000046441	0.00E+00	1.00E+00	0.00E+00	1.00E+00	-5.13E-15	1.00E+00	0.00E+00	1.00E+00	0.00E+00	1.00E+00
ENSBTAG00000047232	0.00E+00	1.00E+00	0.00E+00	1.00E+00	-5.13E-15	1.00E+00	0.00E+00	1.00E+00	0.00E+00	1.00E+00
ENSBTAG00000038637	1.92E+00	8.23E-01	0.00E+00	1.00E+00	-5.13E-15	1.00E+00	0.00E+00	1.00E+00	0.00E+00	1.00E+00
ENSBTAG00000046825	0.00E+00	1.00E+00	0.00E+00	1.00E+00	-5.13E-15	1.00E+00	0.00E+00	1.00E+00	0.00E+00	1.00E+00
ENSBTAG00000039214	0.00E+00	1.00E+00	0.00E+00	1.00E+00	-5.13E-15	1.00E+00	0.00E+00	1.00E+00	0.00E+00	1.00E+00
ENSBTAG00000018182	0.00E+00	1.00E+00	0.00E+00	1.00E+00	-5.13E-15	1.00E+00	0.00E+00	1.00E+00	0.00E+00	1.00E+00
ENSBTAG00000027651	-1.91E+00	8.23E-01	-1.91E+00	4.69E-01	-1.91E+00	4.28E-01	-1.91E+00	4.39E-01	-1.91E+00	8.00E-01
ENSBTAG00000027653	0.00E+00	1.00E+00	0.00E+00	1.00E+00	1.78E+00	4.79E-01	0.00E+00	1.00E+00	0.00E+00	1.00E+00
ENSBTAG00000003582	0.00E+00	1.00E+00	0.00E+00	1.00E+00	-5.13E-15	1.00E+00	0.00E+00	1.00E+00	0.00E+00	1.00E+00
ENSBTAG00000039088	1.92E+00	8.23E-01	0.00E+00	1.00E+00	-5.13E-15	1.00E+00	1.76E+00	4.97E-01	0.00E+00	1.00E+00
ENSBTAG00000037992	0.00E+00	1.00E+00	0.00E+00	1.00E+00	-5.13E-15	1.00E+00	0.00E+00	1.00E+00	1.93E+00	8.00E-01
ENSBTAG00000019544	0.00E+00	1.00E+00	0.00E+00	1.00E+00	-5.13E-15	1.00E+00	0.00E+00	1.00E+00	0.00E+00	1.00E+00
ENSBTAG00000019549	0.00E+00	1.00E+00	0.00E+00	1.00E+00	-5.13E-15	1.00E+00	0.00E+00	1.00E+00	0.00E+00	1.00E+00
ENSBTAG00000044742	0.00E+00	1.00E+00	0.00E+00	1.00E+00	-5.13E-15	1.00E+00	0.00E+00	1.00E+00	0.00E+00	1.00E+00
ENSBTAG00000037815	-1.91E+00	8.23E-01	-1.91E+00	4.69E-01	-1.91E+00	4.28E-01	-1.91E+00	4.39E-01	-1.91E+00	8.00E-01
ENSBTAG00000039178	0.00E+00	1.00E+00	0.00E+00	1.00E+00	2.56E+00	2.54E-01	1.76E+00	4.97E-01	0.00E+00	1.00E+00
ENSBTAG00000038748	2.48E-01	1.00E+00	3.44E-01	8.38E-01	3.96E-01	7.45E-01	4.73E-01	6.40E-01	2.82E-01	1.00E+00
ENSBTAG00000039056	0.00E+00	1.00E+00	0.00E+00	1.00E+00	-5.13E-15	1.00E+00	0.00E+00	1.00E+00	0.00E+00	1.00E+00
ENSBTAG00000037751	0.00E+00	1.00E+00	0.00E+00	1.00E+00	-5.13E-15	1.00E+00	0.00E+00	1.00E+00	0.00E+00	1.00E+00
ENSBTAG00000037644	-2.51E-02	1.00E+00	-8.37E-01	5.88E-01	9.54E-02	1.00E+00	-1.12E+00	4.04E-01	2.68E-01	1.00E+00
ENSBTAG00000040185	0.00E+00	1.00E+00	0.00E+00	1.00E+00	1.78E+00	4.79E-01	0.00E+00	1.00E+00	0.00E+00	1.00E+00
ENSBTAG00000020673	0.00E+00	1.00E+00	0.00E+00	1.00E+00	1.78E+00	4.79E-01	3.03E+00	1.50E-01	0.00E+00	1.00E+00
ENSBTAG00000047477	-1.91E+00	8.23E-01	-1.91E+00	4.69E-01	-1.91E+00	4.28E-01	-1.91E+00	4.39E-01	-1.91E+00	8.00E-01
ENSBTAG00000046378	0.00E+00	1.00E+00	1.99E+00	4.43E-01	-5.13E-15	1.00E+00	1.76E+00	4.97E-01	0.00E+00	1.00E+00
ENSBTAG00000048301	0.00E+00	1.00E+00	1.98E+00	4.43E-01	-5.13E-15	1.00E+00	0.00E+00	1.00E+00	0.00E+00	1.00E+00
ENSBTAG00000046983	0.00E+00	1.00E+00	0.00E+00	1.00E+00	-5.13E-15	1.00E+00	0.00E+00	1.00E+00	0.00E+00	1.00E+00
ENSBTAG00000048003	-1.91E+00	8.23E-01	-1.91E+00	4.69E-01	-1.91E+00	4.28E-01	-1.91E+00	4.39E-01	-1.91E+00	8.00E-01
ENSBTAG00000048300	0.00E+00	1.00E+00	0.00E+00	1.00E+00	-5.13E-15	1.00E+00	0.00E+00	1.00E+00	0.00E+00	1.00E+00
ENSBTAG00000038679	0.00E+00	1.00E+00	0.00E+00	1.00E+00	-5.13E-15	1.00E+00	0.00E+00	1.00E+00	0.00E+00	1.00E+00

ENSBTAG00000039773	0.00E+00	1.00E+00	1.98E+00	4.43E-01	-5.13E-15	1.00E+00	2.53E+00	2.71E-01	0.00E+00	1.00E+00
ENSBTAG00000048310	0.00E+00	1.00E+00	0.00E+00	1.00E+00	-5.13E-15	1.00E+00	0.00E+00	1.00E+00	0.00E+00	1.00E+00
ENSBTAG00000047356	-3.21E+00	3.75E-01	-1.23E+00	6.11E-01	-1.43E+00	4.79E-01	-1.45E+00	4.94E-01	-1.30E+00	9.00E-01
ENSBTAG00000047847	-2.70E+00	5.76E-01	-2.70E+00	2.42E-01	-2.70E+00	1.99E-01	-9.39E-01	8.06E-01	-2.70E+00	6.01E-01
ENSBTAG00000046829	1.92E+00	8.23E-01	0.00E+00	1.00E+00	-5.13E-15	1.00E+00	0.00E+00	1.00E+00	0.00E+00	1.00E+00
ENSBTAG00000040017	0.00E+00	1.00E+00	0.00E+00	1.00E+00	-5.13E-15	1.00E+00	0.00E+00	1.00E+00	0.00E+00	1.00E+00
ENSBTAG00000045959	0.00E+00	1.00E+00	0.00E+00	1.00E+00	-5.13E-15	1.00E+00	0.00E+00	1.00E+00	0.00E+00	1.00E+00
ENSBTAG00000046921	0.00E+00	1.00E+00	0.00E+00	1.00E+00	-5.13E-15	1.00E+00	0.00E+00	1.00E+00	0.00E+00	1.00E+00
ENSBTAG00000039054	0.00E+00	1.00E+00	0.00E+00	1.00E+00	-5.13E-15	1.00E+00	0.00E+00	1.00E+00	0.00E+00	1.00E+00
ENSBTAG00000038578	0.00E+00	1.00E+00	0.00E+00	1.00E+00	-5.13E-15	1.00E+00	0.00E+00	1.00E+00	0.00E+00	1.00E+00
ENSBTAG00000038899	0.00E+00	1.00E+00	0.00E+00	1.00E+00	-5.13E-15	1.00E+00	0.00E+00	1.00E+00	0.00E+00	1.00E+00
ENSBTAG00000047662	0.00E+00	1.00E+00	0.00E+00	1.00E+00	-5.13E-15	1.00E+00	0.00E+00	1.00E+00	0.00E+00	1.00E+00
ENSBTAG00000040236	0.00E+00	1.00E+00	0.00E+00	1.00E+00	-5.13E-15	1.00E+00	0.00E+00	1.00E+00	0.00E+00	1.00E+00
ENSBTAG00000047178	0.00E+00	1.00E+00	0.00E+00	1.00E+00	-5.13E-15	1.00E+00	0.00E+00	1.00E+00	0.00E+00	1.00E+00
ENSBTAG00000037398	0.00E+00	1.00E+00	0.00E+00	1.00E+00	-5.13E-15	1.00E+00	0.00E+00	1.00E+00	0.00E+00	1.00E+00
ENSBTAG00000039253	0.00E+00	1.00E+00	0.00E+00	1.00E+00	-5.13E-15	1.00E+00	0.00E+00	1.00E+00	0.00E+00	1.00E+00
ENSBTAG00000048220	0.00E+00	1.00E+00	0.00E+00	1.00E+00	-5.13E-15	1.00E+00	0.00E+00	1.00E+00	0.00E+00	1.00E+00
ENSBTAG00000039651	0.00E+00	1.00E+00	0.00E+00	1.00E+00	-5.13E-15	1.00E+00	0.00E+00	1.00E+00	0.00E+00	1.00E+00
ENSBTAG00000038727	0.00E+00	1.00E+00	0.00E+00	1.00E+00	-5.13E-15	1.00E+00	0.00E+00	1.00E+00	0.00E+00	1.00E+00
ENSBTAG00000014853	0.00E+00	1.00E+00	0.00E+00	1.00E+00	-5.13E-15	1.00E+00	0.00E+00	1.00E+00	0.00E+00	1.00E+00
ENSBTAG00000042309	0.00E+00	1.00E+00	0.00E+00	1.00E+00	-5.13E-15	1.00E+00	0.00E+00	1.00E+00	0.00E+00	1.00E+00
ENSBTAG00000038824	0.00E+00	1.00E+00	1.98E+00	4.43E-01	-5.13E-15	1.00E+00	0.00E+00	1.00E+00	0.00E+00	1.00E+00
ENSBTAG00000000763	0.00E+00	1.00E+00	0.00E+00	1.00E+00	-5.13E-15	1.00E+00	0.00E+00	1.00E+00	0.00E+00	1.00E+00
ENSBTAG00000040526	0.00E+00	1.00E+00	0.00E+00	1.00E+00	-5.13E-15	1.00E+00	0.00E+00	1.00E+00	0.00E+00	1.00E+00
ENSBTAG00000038161	0.00E+00	1.00E+00	0.00E+00	1.00E+00	-5.13E-15	1.00E+00	0.00E+00	1.00E+00	0.00E+00	1.00E+00
ENSBTAG00000045612	0.00E+00	1.00E+00	0.00E+00	1.00E+00	-5.13E-15	1.00E+00	0.00E+00	1.00E+00	0.00E+00	1.00E+00
ENSBTAG00000046913	0.00E+00	1.00E+00	0.00E+00	1.00E+00	-5.13E-15	1.00E+00	0.00E+00	1.00E+00	0.00E+00	1.00E+00
ENSBTAG00000048307	0.00E+00	1.00E+00	0.00E+00	1.00E+00	-5.13E-15	1.00E+00	0.00E+00	1.00E+00	0.00E+00	1.00E+00
ENSBTAG00000040408	0.00E+00	1.00E+00	0.00E+00	1.00E+00	1.78E+00	4.79E-01	0.00E+00	1.00E+00	0.00E+00	1.00E+00
ENSBTAG00000040139	0.00E+00	1.00E+00	0.00E+00	1.00E+00	-5.13E-15	1.00E+00	0.00E+00	1.00E+00	0.00E+00	1.00E+00
ENSBTAG00000045872	0.00E+00	1.00E+00	0.00E+00	1.00E+00	-5.13E-15	1.00E+00	0.00E+00	1.00E+00	0.00E+00	1.00E+00
ENSBTAG00000035006	0.00E+00	1.00E+00	0.00E+00	1.00E+00	-5.13E-15	1.00E+00	0.00E+00	1.00E+00	0.00E+00	1.00E+00
ENSBTAG00000035008	0.00E+00	1.00E+00	0.00E+00	1.00E+00	-5.13E-15	1.00E+00	0.00E+00	1.00E+00	0.00E+00	1.00E+00
ENSBTAG00000008312	1.91E+00	8.23E-01	0.00E+00	1.00E+00	-5.13E-15	1.00E+00	0.00E+00	1.00E+00	0.00E+00	1.00E+00
ENSBTAG00000045488	0.00E+00	1.00E+00	0.00E+00	1.00E+00	-5.13E-15	1.00E+00	0.00E+00	1.00E+00	0.00E+00	1.00E+00

ENSBTAG00000047960	0.00E+00	1.00E+00	0.00E+00	1.00E+00	-5.13E-15	1.00E+00	0.00E+00	1.00E+00	0.00E+00	1.00E+00
ENSBTAG00000035027	0.00E+00	1.00E+00	0.00E+00	1.00E+00	-5.13E-15	1.00E+00	0.00E+00	1.00E+00	0.00E+00	1.00E+00
ENSBTAG00000046624	0.00E+00	1.00E+00	0.00E+00	1.00E+00	-5.13E-15	1.00E+00	0.00E+00	1.00E+00	1.91E+00	8.00E-01
ENSBTAG00000039298	0.00E+00	1.00E+00	0.00E+00	1.00E+00	-5.13E-15	1.00E+00	0.00E+00	1.00E+00	0.00E+00	1.00E+00
ENSBTAG00000037875	0.00E+00	1.00E+00	0.00E+00	1.00E+00	1.78E+00	4.79E-01	0.00E+00	1.00E+00	0.00E+00	1.00E+00
ENSBTAG00000047945	1.92E+00	8.23E-01	0.00E+00	1.00E+00	-5.13E-15	1.00E+00	1.76E+00	4.97E-01	0.00E+00	1.00E+00
ENSBTAG00000039735	0.00E+00	1.00E+00	0.00E+00	1.00E+00	-5.13E-15	1.00E+00	1.76E+00	4.97E-01	0.00E+00	1.00E+00
ENSBTAG00000039330	0.00E+00	1.00E+00	0.00E+00	1.00E+00	-5.13E-15	1.00E+00	0.00E+00	1.00E+00	0.00E+00	1.00E+00
ENSBTAG00000040339	0.00E+00	1.00E+00	1.98E+00	4.43E-01	-5.13E-15	1.00E+00	0.00E+00	1.00E+00	0.00E+00	1.00E+00
ENSBTAG00000004765	0.00E+00	1.00E+00	0.00E+00	1.00E+00	-5.13E-15	1.00E+00	0.00E+00	1.00E+00	0.00E+00	1.00E+00
ENSBTAG00000038636	0.00E+00	1.00E+00	0.00E+00	1.00E+00	-5.13E-15	1.00E+00	0.00E+00	1.00E+00	0.00E+00	1.00E+00
ENSBTAG00000037886	0.00E+00	1.00E+00	0.00E+00	1.00E+00	-5.13E-15	1.00E+00	0.00E+00	1.00E+00	0.00E+00	1.00E+00
ENSBTAG00000047021	1.91E+00	8.23E-01	0.00E+00	1.00E+00	-5.13E-15	1.00E+00	1.76E+00	4.97E-01	0.00E+00	1.00E+00
ENSBTAG00000017934	0.00E+00	1.00E+00	0.00E+00	1.00E+00	-5.13E-15	1.00E+00	0.00E+00	1.00E+00	0.00E+00	1.00E+00
ENSBTAG00000040478	0.00E+00	1.00E+00	0.00E+00	1.00E+00	-5.13E-15	1.00E+00	0.00E+00	1.00E+00	0.00E+00	1.00E+00
ENSBTAG00000045850	0.00E+00	1.00E+00	0.00E+00	1.00E+00	-5.13E-15	1.00E+00	0.00E+00	1.00E+00	0.00E+00	1.00E+00
ENSBTAG00000020033	0.00E+00	1.00E+00	0.00E+00	1.00E+00	-5.13E-15	1.00E+00	0.00E+00	1.00E+00	0.00E+00	1.00E+00
ENSBTAG00000047489	0.00E+00	1.00E+00	1.98E+00	4.43E-01	-5.13E-15	1.00E+00	0.00E+00	1.00E+00	0.00E+00	1.00E+00
ENSBTAG00000022368	0.00E+00	1.00E+00	0.00E+00	1.00E+00	-5.13E-15	1.00E+00	0.00E+00	1.00E+00	0.00E+00	1.00E+00
ENSBTAG00000038075	-3.60E+00	2.31E-01	-3.60E+00	6.58E-02	-3.60E+00	4.48E-02	-3.60E+00	4.84E-02	-1.67E+00	7.79E-01
ENSBTAG00000038579	0.00E+00	1.00E+00	0.00E+00	1.00E+00	-5.13E-15	1.00E+00	0.00E+00	1.00E+00	0.00E+00	1.00E+00
ENSBTAG00000040119	0.00E+00	1.00E+00	0.00E+00	1.00E+00	-5.13E-15	1.00E+00	0.00E+00	1.00E+00	0.00E+00	1.00E+00
ENSBTAG00000038787	0.00E+00	1.00E+00	0.00E+00	1.00E+00	-5.13E-15	1.00E+00	0.00E+00	1.00E+00	1.91E+00	8.00E-01
ENSBTAG00000039503	0.00E+00	1.00E+00	0.00E+00	1.00E+00	-5.13E-15	1.00E+00	3.03E+00	1.50E-01	1.91E+00	8.00E-01
ENSBTAG00000039654	0.00E+00	1.00E+00	0.00E+00	1.00E+00	-5.13E-15	1.00E+00	0.00E+00	1.00E+00	0.00E+00	1.00E+00
ENSBTAG00000046627	0.00E+00	1.00E+00	0.00E+00	1.00E+00	-5.13E-15	1.00E+00	0.00E+00	1.00E+00	0.00E+00	1.00E+00
ENSBTAG00000039134	0.00E+00	1.00E+00	0.00E+00	1.00E+00	-5.13E-15	1.00E+00	0.00E+00	1.00E+00	0.00E+00	1.00E+00
ENSBTAG00000046816	0.00E+00	1.00E+00	0.00E+00	1.00E+00	-5.13E-15	1.00E+00	0.00E+00	1.00E+00	0.00E+00	1.00E+00
ENSBTAG00000037474	0.00E+00	1.00E+00	0.00E+00	1.00E+00	-5.13E-15	1.00E+00	1.76E+00	4.97E-01	0.00E+00	1.00E+00
ENSBTAG00000047714	0.00E+00	1.00E+00	0.00E+00	1.00E+00	-5.13E-15	1.00E+00	0.00E+00	1.00E+00	0.00E+00	1.00E+00
ENSBTAG00000047500	-1.91E+00	8.23E-01	-1.91E+00	4.69E-01	-1.91E+00	4.28E-01	-1.91E+00	4.39E-01	-1.91E+00	8.00E-01
ENSBTAG00000038170	0.00E+00	1.00E+00	1.98E+00	4.43E-01	-5.13E-15	1.00E+00	0.00E+00	1.00E+00	0.00E+00	1.00E+00
ENSBTAG00000046853	0.00E+00	1.00E+00	0.00E+00	1.00E+00	-5.13E-15	1.00E+00	0.00E+00	1.00E+00	0.00E+00	1.00E+00
ENSBTAG00000047498	0.00E+00	1.00E+00	0.00E+00	1.00E+00	-5.13E-15	1.00E+00	0.00E+00	1.00E+00	0.00E+00	1.00E+00
ENSBTAG00000043761	0.00E+00	1.00E+00	0.00E+00	1.00E+00	-5.13E-15	1.00E+00	0.00E+00	1.00E+00	0.00E+00	1.00E+00

ENSBTAG00000047630	0.00E+00	1.00E+00	0.00E+00	1.00E+00	-5.13E-15	1.00E+00	0.00E+00	1.00E+00	0.00E+00	1.00E+00
ENSBTAG00000031943	0.00E+00	1.00E+00	0.00E+00	1.00E+00	1.78E+00	4.79E-01	0.00E+00	1.00E+00	0.00E+00	1.00E+00
ENSBTAG00000031937	0.00E+00	1.00E+00	0.00E+00	1.00E+00	-5.13E-15	1.00E+00	0.00E+00	1.00E+00	0.00E+00	1.00E+00
ENSBTAG00000008236	0.00E+00	1.00E+00	0.00E+00	1.00E+00	-5.13E-15	1.00E+00	0.00E+00	1.00E+00	0.00E+00	1.00E+00
ENSBTAG00000034711	0.00E+00	1.00E+00	0.00E+00	1.00E+00	-5.13E-15	1.00E+00	0.00E+00	1.00E+00	0.00E+00	1.00E+00
ENSBTAG00000027637	0.00E+00	1.00E+00	0.00E+00	1.00E+00	-5.13E-15	1.00E+00	0.00E+00	1.00E+00	0.00E+00	1.00E+00
ENSBTAG00000000233	1.92E+00	8.23E-01	0.00E+00	1.00E+00	-5.13E-15	1.00E+00	1.76E+00	4.97E-01	1.91E+00	8.00E-01
ENSBTAG00000000232	0.00E+00	1.00E+00	0.00E+00	1.00E+00	-5.13E-15	1.00E+00	1.77E+00	4.97E-01	0.00E+00	1.00E+00
ENSBTAG00000011332	-7.06E-01	3.75E-01	-3.58E+00	2.73E-17	-1.97E+00	4.26E-07	1.47E-01	1.00E+00	4.79E-01	7.62E-01
ENSBTAG00000048052	-3.21E+00	3.75E-01	-3.21E+00	1.26E-01	-3.21E+00	9.50E-02	7.28E-01	7.48E-01	-5.00E-01	1.00E+00
ENSBTAG00000047556	0.00E+00	1.00E+00	0.00E+00	1.00E+00	-5.13E-15	1.00E+00	0.00E+00	1.00E+00	1.91E+00	8.00E-01
ENSBTAG00000047803	0.00E+00	1.00E+00	0.00E+00	1.00E+00	-5.13E-15	1.00E+00	0.00E+00	1.00E+00	0.00E+00	1.00E+00
ENSBTAG00000039185	0.00E+00	1.00E+00	0.00E+00	1.00E+00	-5.13E-15	1.00E+00	0.00E+00	1.00E+00	0.00E+00	1.00E+00
ENSBTAG00000005060	0.00E+00	1.00E+00	0.00E+00	1.00E+00	-5.13E-15	1.00E+00	0.00E+00	1.00E+00	0.00E+00	1.00E+00
ENSBTAG00000039467	0.00E+00	1.00E+00	0.00E+00	1.00E+00	-5.13E-15	1.00E+00	0.00E+00	1.00E+00	0.00E+00	1.00E+00
ENSBTAG00000031923	0.00E+00	1.00E+00	0.00E+00	1.00E+00	-5.13E-15	1.00E+00	0.00E+00	1.00E+00	0.00E+00	1.00E+00
ENSBTAG00000031924	0.00E+00	1.00E+00	0.00E+00	1.00E+00	-5.13E-15	1.00E+00	0.00E+00	1.00E+00	0.00E+00	1.00E+00
ENSBTAG00000013683	0.00E+00	1.00E+00	0.00E+00	1.00E+00	-5.13E-15	1.00E+00	0.00E+00	1.00E+00	0.00E+00	1.00E+00
ENSBTAG00000040075	-1.91E+00	8.23E-01	-1.91E+00	4.69E-01	-1.91E+00	4.28E-01	-1.91E+00	4.39E-01	-1.91E+00	8.00E-01
ENSBTAG00000013900	2.06E-01	1.00E+00	7.98E-01	2.93E-02	5.83E-01	1.42E-01	-1.36E-01	1.00E+00	3.46E-01	8.24E-01
ENSBTAG00000012325	-2.70E+00	5.76E-01	-2.70E+00	2.42E-01	-2.70E+00	1.99E-01	-2.70E+00	2.05E-01	-2.70E+00	6.01E-01
ENSBTAG00000015357	0.00E+00	1.00E+00	0.00E+00	1.00E+00	-5.13E-15	1.00E+00	0.00E+00	1.00E+00	0.00E+00	1.00E+00
ENSBTAG00000031915	0.00E+00	1.00E+00	0.00E+00	1.00E+00	-5.13E-15	1.00E+00	0.00E+00	1.00E+00	0.00E+00	1.00E+00
ENSBTAG00000047860	0.00E+00	1.00E+00	0.00E+00	1.00E+00	-5.13E-15	1.00E+00	0.00E+00	1.00E+00	0.00E+00	1.00E+00
ENSBTAG00000031907	0.00E+00	1.00E+00	0.00E+00	1.00E+00	-5.13E-15	1.00E+00	0.00E+00	1.00E+00	1.91E+00	8.00E-01
ENSBTAG00000045696	0.00E+00	1.00E+00	0.00E+00	1.00E+00	-5.13E-15	1.00E+00	0.00E+00	1.00E+00	0.00E+00	1.00E+00
ENSBTAG00000040380	-1.79E-04	1.00E+00	-1.23E-02	1.00E+00	-6.47E-01	1.22E-03	-5.97E-01	3.74E-03	-1.81E-02	1.00E+00
ENSBTAG00000045556	0.00E+00	1.00E+00	0.00E+00	1.00E+00	-5.13E-15	1.00E+00	0.00E+00	1.00E+00	0.00E+00	1.00E+00
ENSBTAG00000031903	0.00E+00	1.00E+00	0.00E+00	1.00E+00	-5.13E-15	1.00E+00	0.00E+00	1.00E+00	0.00E+00	1.00E+00
ENSBTAG00000035686	0.00E+00	1.00E+00	1.98E+00	4.43E-01	-5.13E-15	1.00E+00	0.00E+00	1.00E+00	0.00E+00	1.00E+00
ENSBTAG00000005070	0.00E+00	1.00E+00	0.00E+00	1.00E+00	-5.13E-15	1.00E+00	0.00E+00	1.00E+00	0.00E+00	1.00E+00
ENSBTAG00000013111	-1.75E-01	9.23E-01	-1.72E+00	2.27E-20	-1.77E+00	8.56E-22	-6.98E-01	2.96E-04	-3.29E-02	1.00E+00
ENSBTAG00000013109	6.27E-02	1.00E+00	-9.72E-01	1.44E-05	-2.04E+00	2.23E-20	-1.33E+00	1.93E-09	7.34E-01	2.18E-02
ENSBTAG00000000347	-6.01E-02	1.00E+00	1.21E-01	8.39E-01	3.64E-01	1.27E-01	3.67E-01	1.32E-01	-9.03E-02	1.00E+00
ENSBTAG00000008433	3.45E-01	5.98E-01	-9.31E-02	1.00E+00	-6.22E-01	1.35E-02	-3.11E-01	3.26E-01	3.58E-01	6.01E-01

ENSBTAG00000008432	3.10E-01	7.21E-01	1.60E+00	5.16E-12	5.95E-01	1.89E-02	3.00E-01	3.54E-01	-4.83E-03	1.00E+00
ENSBTAG00000006345	9.89E-01	5.08E-01	2.13E+00	8.29E-05	1.34E+00	3.14E-02	1.51E-02	1.00E+00	1.38E-02	1.00E+00
ENSBTAG00000044059	0.00E+00	1.00E+00	0.00E+00	1.00E+00	-5.13E-15	1.00E+00	0.00E+00	1.00E+00	0.00E+00	1.00E+00
ENSBTAG00000013997	0.00E+00	1.00E+00	0.00E+00	1.00E+00	-5.13E-15	1.00E+00	0.00E+00	1.00E+00	1.91E+00	8.00E-01
ENSBTAG00000023648	1.24E+00	2.65E-01	5.55E-01	6.22E-01	1.31E+00	4.56E-02	1.54E+00	1.50E-02	1.35E+00	2.56E-01
ENSBTAG00000037826	0.00E+00	1.00E+00	0.00E+00	1.00E+00	3.05E+00	1.37E-01	1.77E+00	4.97E-01	3.88E+00	2.06E-01
ENSBTAG00000026796	-5.09E-01	1.00E+00	-4.33E-01	1.00E+00	-1.60E-01	1.00E+00	4.64E-01	9.83E-01	4.21E-03	1.00E+00
ENSBTAG00000011549	-1.74E-01	1.00E+00	2.50E-01	6.47E-01	8.61E-02	1.00E+00	-1.86E-01	8.36E-01	2.38E-01	9.93E-01
ENSBTAG00000044509	0.00E+00	1.00E+00	0.00E+00	1.00E+00	-5.13E-15	1.00E+00	0.00E+00	1.00E+00	0.00E+00	1.00E+00
ENSBTAG00000014167	1.78E-01	9.32E-01	9.10E-01	1.64E-06	1.24E+00	2.29E-11	8.91E-01	3.20E-06	3.67E-02	1.00E+00
ENSBTAG00000027676	1.30E+00	2.07E-04	3.66E+00	6.77E-33	3.43E+00	1.33E-29	3.35E+00	3.06E-28	-4.73E-02	1.00E+00
ENSBTAG00000018449	-2.74E-01	6.46E-01	-1.66E+00	4.45E-18	-1.38E+00	3.84E-13	-1.02E+00	1.40E-07	4.68E-01	1.92E-01
ENSBTAG00000017964	-1.49E-01	1.00E+00	-2.45E-01	3.72E-01	-7.49E-01	2.27E-04	-1.12E+00	3.11E-08	1.49E-01	1.00E+00
ENSBTAG00000003001	-1.04E-01	1.00E+00	-2.90E-01	2.68E-01	3.91E-01	9.45E-02	9.29E-01	5.07E-06	-3.28E-01	5.74E-01
ENSBTAG00000003000	-3.33E-01	1.00E+00	-1.91E+00	2.84E-03	-1.95E+00	1.39E-03	-2.89E+00	1.74E-05	4.40E-01	9.00E-01
ENSBTAG00000002999	-5.46E-02	1.00E+00	-4.83E-01	1.26E-01	-3.18E-01	3.86E-01	-4.82E-01	1.31E-01	4.96E-02	1.00E+00
ENSBTAG00000038532	1.24E+00	1.49E-02	1.02E+00	1.49E-02	6.31E-01	1.81E-01	1.02E+00	1.57E-02	1.34E+00	1.58E-02
ENSBTAG00000021007	1.06E+00	2.21E-02	9.53E-01	9.74E-03	4.99E-01	2.58E-01	3.83E-03	1.00E+00	5.10E-01	6.24E-01
ENSBTAG00000027684	-4.78E-01	6.07E-01	-1.22E+00	3.29E-04	-1.81E+00	5.43E-08	-1.62E+00	1.47E-06	-5.05E-01	6.01E-01
ENSBTAG00000019167	-2.06E-01	1.00E+00	-1.56E+00	6.89E-09	-5.51E-01	7.11E-02	3.25E-01	3.94E-01	-7.41E-02	1.00E+00
ENSBTAG00000019168	-3.38E+00	1.63E-02	-2.00E+00	4.30E-02	-1.13E+00	2.28E-01	-9.61E-01	3.31E-01	-9.67E-01	7.56E-01
ENSBTAG00000001280	2.33E-01	9.28E-01	9.33E-01	1.88E-04	1.43E+00	1.85E-09	1.64E+00	5.76E-12	4.85E-01	3.87E-01
ENSBTAG00000008419	3.08E-01	7.97E-01	-3.56E-01	2.80E-01	-5.84E-01	3.83E-02	-6.56E-01	1.97E-02	-2.54E-01	8.46E-01
ENSBTAG00000029859	0.00E+00	1.00E+00	0.00E+00	1.00E+00	-5.13E-15	1.00E+00	0.00E+00	1.00E+00	0.00E+00	1.00E+00
ENSBTAG00000002051	2.40E-04	1.00E+00	-9.37E-01	2.21E-05	-1.01E-01	9.50E-01	-4.87E-02	1.00E+00	-1.38E-02	1.00E+00
ENSBTAG00000015182	-2.51E-01	8.23E-01	-9.92E-01	1.25E-05	-1.97E+00	8.16E-19	-1.70E+00	2.76E-14	-2.15E-01	8.78E-01
ENSBTAG00000044515	0.00E+00	1.00E+00	0.00E+00	1.00E+00	-5.13E-15	1.00E+00	0.00E+00	1.00E+00	0.00E+00	1.00E+00
ENSBTAG00000019059	5.94E-01	2.30E-01	1.20E+00	1.53E-05	7.03E-01	1.86E-02	5.42E-01	9.79E-02	5.18E-01	4.34E-01
ENSBTAG00000019062	-1.09E-01	1.00E+00	-2.03E+00	1.79E-14	-1.38E+00	1.28E-07	-1.07E+00	6.70E-05	-7.68E-02	1.00E+00
ENSBTAG00000039050	-6.40E-01	4.40E-01	-2.00E-01	8.82E-01	1.49E+00	6.95E-05	1.76E+00	2.65E-06	3.84E-01	8.15E-01
ENSBTAG00000038737	-2.00E-01	1.00E+00	-2.65E-01	8.53E-01	-1.58E+00	1.87E-03	-1.91E+00	2.40E-04	3.24E-01	1.00E+00
ENSBTAG00000012505	4.64E-01	6.68E-01	-1.85E+00	2.89E-07	1.18E-01	1.00E+00	1.81E-01	8.87E-01	1.91E-01	1.00E+00
ENSBTAG00000016494	1.71E+00	4.79E-07	1.42E+00	1.17E-05	6.81E-01	6.81E-02	5.37E-01	1.96E-01	7.54E-02	1.00E+00
ENSBTAG00000016497	-6.91E-02	1.00E+00	-1.33E+00	2.20E-04	-1.35E+00	1.34E-04	-1.39E-01	9.94E-01	6.07E-01	4.34E-01
ENSBTAG00000042782	0.00E+00	1.00E+00	0.00E+00	1.00E+00	-5.13E-15	1.00E+00	0.00E+00	1.00E+00	0.00E+00	1.00E+00

ENSBTAG00000009830	-2.77E-02	1.00E+00	-7.79E-01	1.84E-02	-7.89E-01	1.48E-02	-4.65E-01	2.14E-01	4.60E-01	5.81E-01
ENSBTAG00000000025	1.15E-01	1.00E+00	8.26E-01	2.21E-05	6.02E-01	2.79E-03	1.47E-01	6.98E-01	-1.46E-01	1.00E+00
ENSBTAG00000042173	-2.71E+00	5.76E-01	8.15E-02	1.00E+00	-9.24E-01	8.08E-01	-1.67E-01	1.00E+00	-2.71E+00	6.01E-01
ENSBTAG00000008347	4.18E-02	1.00E+00	-1.38E-01	9.29E-01	-2.27E-01	6.42E-01	1.34E-01	9.36E-01	-4.44E-01	5.89E-01
ENSBTAG00000003679	-2.56E-01	8.23E-01	-7.83E-01	5.08E-04	-9.93E-01	6.00E-06	-7.29E-01	1.49E-03	-2.35E-01	8.00E-01
ENSBTAG00000003682	-1.05E+00	4.38E-01	-1.47E-01	1.00E+00	-6.45E-01	4.17E-01	-1.80E+00	1.02E-02	-9.24E-01	6.01E-01
ENSBTAG00000003692	-4.34E-01	4.42E-01	-1.10E+00	1.48E-05	-6.15E-01	2.39E-02	-3.16E-01	3.62E-01	-2.28E-01	9.01E-01
ENSBTAG00000005259	-2.21E+00	4.32E-01	4.95E-01	8.59E-01	-1.07E+00	4.79E-01	-1.09E+00	4.91E-01	-4.13E+00	1.39E-01
ENSBTAG00000002907	2.48E-01	8.23E-01	1.14E+00	2.96E-08	1.20E+00	4.28E-09	8.62E-01	5.04E-05	9.77E-02	1.00E+00
ENSBTAG00000027612	1.64E-01	1.00E+00	1.16E+00	8.93E-09	1.29E+00	1.19E-10	1.00E+00	1.03E-06	1.76E-02	1.00E+00
ENSBTAG00000006579	-3.27E-03	1.00E+00	-2.86E-01	9.95E-01	8.60E-02	1.00E+00	2.91E-01	9.88E-01	6.76E-01	8.50E-01
ENSBTAG00000027610	-5.24E-02	1.00E+00	1.11E-01	8.82E-01	7.57E-02	1.00E+00	2.52E-01	3.81E-01	-5.60E-01	9.77E-02
ENSBTAG00000000770	1.57E-01	1.00E+00	-5.61E-01	3.53E-01	-1.04E+00	3.66E-02	-8.45E-02	1.00E+00	-3.33E-01	9.93E-01
ENSBTAG00000042312	0.00E+00	1.00E+00	0.00E+00	1.00E+00	-5.13E-15	1.00E+00	0.00E+00	1.00E+00	0.00E+00	1.00E+00
ENSBTAG00000011873	-1.15E+00	8.86E-01	3.56E-01	1.00E+00	3.17E-01	1.00E+00	4.51E-01	9.26E-01	3.05E-01	1.00E+00
ENSBTAG00000008227	-1.32E+00	2.74E-01	-1.78E+00	1.95E-02	-6.78E-01	4.13E-01	-1.38E+00	5.55E-02	-3.50E-01	1.00E+00
ENSBTAG00000016869	1.50E-01	1.00E+00	3.96E-01	1.89E-01	5.56E-01	3.78E-02	3.66E-01	2.43E-01	4.58E-02	1.00E+00
ENSBTAG00000021306	-3.84E-01	1.00E+00	-3.04E-01	9.93E-01	5.42E-01	6.02E-01	-5.88E-01	6.57E-01	-5.61E-01	1.00E+00
ENSBTAG00000000727	-9.27E-02	1.00E+00	-9.77E-01	3.71E-05	-8.38E-01	3.72E-04	-6.17E-01	1.32E-02	4.54E-01	3.49E-01
ENSBTAG00000047393	0.00E+00	1.00E+00	0.00E+00	1.00E+00	-5.13E-15	1.00E+00	0.00E+00	1.00E+00	0.00E+00	1.00E+00
ENSBTAG00000042319	0.00E+00	1.00E+00	0.00E+00	1.00E+00	-5.13E-15	1.00E+00	0.00E+00	1.00E+00	0.00E+00	1.00E+00
ENSBTAG00000006610	1.89E-02	1.00E+00	-8.88E-01	2.05E-02	-2.89E-01	5.97E-01	-6.16E-03	1.00E+00	8.83E-02	1.00E+00
ENSBTAG00000006644	4.51E-01	3.33E-01	1.24E+00	9.11E-08	1.25E+00	5.38E-08	1.26E+00	7.02E-08	9.22E-02	1.00E+00
ENSBTAG00000025931	7.25E-03	1.00E+00	1.61E-01	9.19E-01	1.89E-01	8.19E-01	5.24E-01	1.61E-01	2.98E-01	8.82E-01
ENSBTAG00000048088	0.00E+00	1.00E+00	1.98E+00	4.43E-01	-5.13E-15	1.00E+00	1.76E+00	4.97E-01	0.00E+00	1.00E+00
ENSBTAG00000047344	0.00E+00	1.00E+00	0.00E+00	1.00E+00	1.78E+00	4.79E-01	2.53E+00	2.71E-01	1.91E+00	8.00E-01
ENSBTAG00000015596	-4.36E-01	8.23E-01	-2.82E+00	1.85E-12	-4.52E+00	1.97E-26	-4.54E+00	1.62E-26	2.06E-01	1.00E+00
ENSBTAG00000019622	-1.36E+00	5.58E-01	-2.45E+00	2.72E-02	-1.51E+00	1.34E-01	-1.53E-01	1.00E+00	-9.16E-01	8.00E-01
ENSBTAG00000020485	2.01E-01	9.40E-01	5.01E-01	4.05E-02	-2.24E-01	4.95E-01	5.63E-02	1.00E+00	2.19E-03	1.00E+00
ENSBTAG00000029833	0.00E+00	1.00E+00	0.00E+00	1.00E+00	-5.13E-15	1.00E+00	0.00E+00	1.00E+00	0.00E+00	1.00E+00
ENSBTAG00000005620	3.73E-02	1.00E+00	2.07E-01	4.86E-01	-4.05E-02	1.00E+00	1.82E-02	1.00E+00	-1.08E-01	1.00E+00
ENSBTAG00000042649	0.00E+00	1.00E+00	0.00E+00	1.00E+00	-5.13E-15	1.00E+00	0.00E+00	1.00E+00	0.00E+00	1.00E+00
ENSBTAG00000042916	0.00E+00	1.00E+00	0.00E+00	1.00E+00	-5.13E-15	1.00E+00	0.00E+00	1.00E+00	0.00E+00	1.00E+00
ENSBTAG00000015087	7.88E-01	7.65E-01	1.17E+00	9.07E-02	-1.86E-01	1.00E+00	8.92E-02	1.00E+00	2.05E-01	1.00E+00
ENSBTAG00000031777	2.88E-01	1.00E+00	-8.39E-01	5.44E-02	-1.19E+00	3.72E-03	-1.08E+00	1.02E-02	-1.35E-01	1.00E+00

ENSBTAG00000001027	1.42E+00	7.12E-03	2.70E+00	2.75E-10	1.55E+00	2.88E-04	1.30E+00	3.45E-03	-4.41E-01	8.08E-01
ENSBTAG00000001028	-4.73E-01	1.00E+00	-6.05E-01	7.50E-01	-6.39E-01	6.86E-01	6.06E-01	6.38E-01	-1.25E+00	7.17E-01
ENSBTAG00000017443	-1.91E+00	8.23E-01	7.51E-02	1.00E+00	-1.25E-01	1.00E+00	-1.91E+00	4.39E-01	-1.91E+00	8.00E-01
ENSBTAG00000047111	2.72E+00	5.76E-01	0.00E+00	1.00E+00	1.78E+00	4.79E-01	1.76E+00	4.97E-01	0.00E+00	1.00E+00
ENSBTAG00000015091	-1.91E+00	8.23E-01	-1.91E+00	4.69E-01	-1.91E+00	4.28E-01	-1.54E-01	1.00E+00	-1.91E+00	8.00E-01
ENSBTAG00000046149	0.00E+00	1.00E+00	0.00E+00	1.00E+00	-5.13E-15	1.00E+00	0.00E+00	1.00E+00	0.00E+00	1.00E+00
ENSBTAG00000000628	3.00E-01	1.00E+00	-4.72E-01	4.34E-01	3.44E-01	5.84E-01	1.42E+00	2.08E-04	2.17E-01	1.00E+00
ENSBTAG00000037483	0.00E+00	1.00E+00	0.00E+00	1.00E+00	-5.13E-15	1.00E+00	0.00E+00	1.00E+00	0.00E+00	1.00E+00
ENSBTAG00000001154	-2.20E-01	1.00E+00	-1.73E+00	1.18E-07	-2.05E+00	2.58E-10	-1.52E+00	3.73E-06	-2.69E-01	9.48E-01
ENSBTAG00000016355	-2.11E-03	1.00E+00	-5.79E-01	2.37E-02	-2.73E-01	4.06E-01	-4.24E-01	1.30E-01	2.25E-02	1.00E+00
ENSBTAG00000010820	3.06E-01	1.00E+00	2.03E+00	5.72E-06	2.05E+00	3.50E-06	1.08E+00	3.45E-02	3.56E-01	1.00E+00
ENSBTAG00000031609	-1.11E-01	1.00E+00	-3.05E-01	4.54E-01	3.82E-01	2.85E-01	3.61E-01	3.38E-01	2.99E-02	1.00E+00
ENSBTAG00000010826	3.01E-01	7.49E-01	6.69E-01	6.45E-03	8.68E-01	2.20E-04	7.59E-01	1.84E-03	1.44E-01	1.00E+00
ENSBTAG00000001301	1.70E+00	4.57E-08	2.15E+00	1.09E-13	1.54E+00	1.11E-07	1.55E+00	1.19E-07	-2.16E-01	1.00E+00
ENSBTAG00000027438	5.25E-01	1.00E+00	9.70E-01	6.60E-01	3.49E-01	1.00E+00	1.22E+00	4.69E-01	5.18E-01	1.00E+00
ENSBTAG00000001537	-1.00E+00	3.33E-01	-4.84E+00	8.19E-17	-5.40E+00	7.42E-20	-3.05E+00	2.72E-08	-4.50E-01	9.24E-01
ENSBTAG00000017218	4.26E-02	1.00E+00	1.91E-01	7.07E-01	1.78E-01	7.43E-01	1.57E-01	8.22E-01	-1.25E-01	1.00E+00
ENSBTAG00000008557	0.00E+00	1.00E+00	0.00E+00	1.00E+00	-5.13E-15	1.00E+00	0.00E+00	1.00E+00	0.00E+00	1.00E+00
ENSBTAG00000047129	1.65E+00	3.25E-01	1.49E+00	1.57E-01	1.21E+00	2.84E-01	7.50E-01	6.61E-01	1.63E+00	4.09E-01
ENSBTAG00000005034	-2.98E-01	6.57E-01	-1.97E+00	2.17E-20	-2.16E+00	3.69E-24	-1.52E+00	6.04E-13	-8.75E-02	1.00E+00
ENSBTAG00000003955	3.65E-02	1.00E+00	5.55E-03	1.00E+00	3.01E-01	3.44E-01	-2.32E-01	5.33E-01	-7.10E-02	1.00E+00
ENSBTAG00000000886	1.37E-01	1.00E+00	-1.02E+00	4.43E-01	-1.28E+00	2.79E-01	3.90E-01	8.94E-01	-8.01E-01	9.06E-01
ENSBTAG00000047903	0.00E+00	1.00E+00	0.00E+00	1.00E+00	1.78E+00	4.79E-01	0.00E+00	1.00E+00	1.93E+00	8.00E-01
ENSBTAG00000021855	4.62E-01	8.23E-01	-2.95E-01	7.72E-01	-8.61E-01	1.01E-01	-7.63E-02	1.00E+00	-3.53E-01	9.73E-01
ENSBTAG00000027126	0.00E+00	1.00E+00	0.00E+00	1.00E+00	-5.13E-15	1.00E+00	0.00E+00	1.00E+00	0.00E+00	1.00E+00
ENSBTAG00000021858	4.48E-01	8.68E-01	3.76E-01	6.17E-01	-3.53E-01	6.61E-01	-5.52E-01	3.86E-01	1.04E+00	2.09E-01
ENSBTAG00000006676	-1.47E+00	2.47E-02	-2.69E+00	1.02E-07	-3.25E+00	1.90E-10	-2.79E+00	3.71E-08	-4.83E-01	8.34E-01
ENSBTAG00000014847	-3.74E-01	8.23E-01	-2.04E+00	4.92E-10	-4.56E+00	8.04E-38	-4.25E+00	8.31E-34	-7.03E-02	1.00E+00
ENSBTAG00000043814	0.00E+00	1.00E+00	0.00E+00	1.00E+00	-5.13E-15	1.00E+00	0.00E+00	1.00E+00	0.00E+00	1.00E+00
ENSBTAG00000021346	2.16E-01	1.00E+00	-1.09E+00	1.28E-03	-5.28E-01	1.59E-01	-8.24E-01	1.75E-02	-3.30E-01	8.06E-01
ENSBTAG00000002606	8.96E-01	7.45E-02	9.16E-01	1.15E-02	2.71E+00	2.67E-15	2.43E+00	1.41E-12	7.56E-01	2.64E-01
ENSBTAG00000001460	4.00E-01	4.32E-01	8.54E-01	2.46E-04	9.15E-01	7.34E-05	6.10E-01	1.42E-02	-3.52E-01	6.01E-01
ENSBTAG00000008134	4.22E-01	1.00E+00	1.76E+00	4.14E-01	1.21E+00	6.44E-01	1.65E+00	4.56E-01	-4.40E-01	1.00E+00
ENSBTAG00000002117	4.16E-01	9.98E-01	1.19E+00	2.11E-02	1.34E-01	1.00E+00	9.13E-01	1.04E-01	3.61E-01	1.00E+00
ENSBTAG00000003361	1.28E-01	1.00E+00	1.24E-01	9.45E-01	7.13E-02	1.00E+00	-7.47E-02	1.00E+00	-1.73E-02	1.00E+00

ENSBTAG00000044677	0.00E+00	1.00E+00	0.00E+00	1.00E+00	-5.13E-15	1.00E+00	0.00E+00	1.00E+00	0.00E+00	1.00E+00
ENSBTAG00000045944	0.00E+00	1.00E+00	0.00E+00	1.00E+00	-5.13E-15	1.00E+00	0.00E+00	1.00E+00	0.00E+00	1.00E+00
ENSBTAG00000020793	-5.29E-01	9.43E-01	-2.57E+00	1.37E-04	-3.29E+00	1.69E-06	-6.57E+00	2.29E-12	-6.77E-01	8.00E-01
ENSBTAG00000039236	0.00E+00	1.00E+00	0.00E+00	1.00E+00	-5.13E-15	1.00E+00	0.00E+00	1.00E+00	0.00E+00	1.00E+00
ENSBTAG00000010889	0.00E+00	1.00E+00	0.00E+00	1.00E+00	-5.13E-15	1.00E+00	0.00E+00	1.00E+00	0.00E+00	1.00E+00
ENSBTAG00000012417	-9.62E-02	1.00E+00	1.23E+00	6.39E-08	3.85E-01	1.64E-01	9.59E-02	9.94E-01	-6.01E-01	1.36E-01
ENSBTAG00000020956	2.01E-01	1.00E+00	3.70E-01	8.85E-01	-3.97E-01	8.72E-01	8.92E-01	2.85E-01	-1.43E+00	5.14E-01
ENSBTAG00000048290	1.31E-02	1.00E+00	8.81E-01	8.52E-01	6.45E-01	9.78E-01	1.49E+00	4.54E-01	1.69E+00	7.74E-01
ENSBTAG00000006060	-7.91E-01	9.32E-01	-6.34E-02	1.00E+00	-4.69E+00	1.40E-03	-5.38E-01	7.75E-01	6.27E-01	9.12E-01
ENSBTAG00000045878	0.00E+00	1.00E+00	0.00E+00	1.00E+00	-5.13E-15	1.00E+00	1.77E+00	4.97E-01	0.00E+00	1.00E+00
ENSBTAG00000000954	8.70E-01	6.44E-01	5.04E-01	6.79E-01	-7.27E-01	5.11E-01	-5.94E-01	6.49E-01	7.34E-01	8.00E-01
ENSBTAG00000032200	5.60E-02	1.00E+00	-2.84E-01	3.37E-01	-5.12E-01	3.44E-02	-1.03E+00	3.89E-06	-3.55E-01	5.67E-01
ENSBTAG00000000475	-1.59E-01	1.00E+00	-1.24E-01	9.03E-01	-3.61E-01	2.27E-01	-2.74E-01	4.37E-01	-2.86E-01	8.00E-01
ENSBTAG00000023831	1.45E-01	1.00E+00	3.15E-01	2.48E-01	2.45E-01	4.28E-01	-6.06E-02	1.00E+00	-4.30E-01	3.89E-01
ENSBTAG00000004561	9.66E-01	7.86E-01	1.07E+00	3.04E-01	8.97E-01	4.21E-01	6.37E-01	6.70E-01	4.09E-01	1.00E+00
ENSBTAG00000047362	-1.46E-01	9.71E-01	-4.47E-01	1.47E-02	-1.10E+00	4.01E-11	-1.40E+00	6.79E-17	-4.89E-01	7.20E-02
ENSBTAG00000047268	4.74E-01	9.50E-01	1.65E-01	1.00E+00	-1.15E+00	1.20E-01	9.13E-02	1.00E+00	-1.42E+00	3.11E-01
ENSBTAG00000005338	1.77E-01	9.94E-01	9.58E-01	5.22E-06	8.53E-01	5.36E-05	1.60E-01	7.04E-01	-4.43E-01	3.46E-01
ENSBTAG00000032151	-7.58E-03	1.00E+00	1.11E+00	2.34E-04	1.29E+00	1.38E-05	1.08E+00	4.21E-04	-3.15E-01	8.02E-01
ENSBTAG00000020731	2.35E+00	2.30E-06	1.96E+00	1.93E-05	1.03E+00	3.90E-02	1.22E+00	1.23E-02	9.17E-01	3.46E-01
ENSBTAG00000014399	6.77E-01	6.53E-02	1.80E+00	4.05E-13	1.60E+00	1.05E-10	1.44E+00	7.93E-09	1.34E-01	1.00E+00
ENSBTAG00000021809	7.38E-01	5.18E-02	-1.09E+00	6.37E-05	-6.11E-01	3.79E-02	-7.64E-01	7.85E-03	-2.52E-01	8.85E-01
ENSBTAG00000008613	1.64E+00	5.15E-07	4.28E-01	2.71E-01	9.01E-02	1.00E+00	9.49E-02	1.00E+00	4.31E-01	6.51E-01
ENSBTAG00000044774	0.00E+00	1.00E+00	0.00E+00	1.00E+00	-5.13E-15	1.00E+00	0.00E+00	1.00E+00	0.00E+00	1.00E+00
ENSBTAG00000008617	7.69E-01	9.42E-03	8.93E-01	1.56E-04	5.25E-01	4.10E-02	-3.17E-01	3.09E-01	-3.89E-01	5.36E-01
ENSBTAG00000042111	0.00E+00	1.00E+00	0.00E+00	1.00E+00	-5.13E-15	1.00E+00	0.00E+00	1.00E+00	0.00E+00	1.00E+00
ENSBTAG00000014884	1.40E+00	1.21E-04	2.38E+00	9.03E-14	2.08E+00	6.11E-11	2.03E+00	2.38E-10	6.99E-01	2.83E-01
ENSBTAG00000043706	8.04E-01	2.72E-02	2.28E+00	7.11E-20	1.75E+00	3.43E-12	1.49E+00	6.52E-09	1.37E-01	1.00E+00
ENSBTAG00000027578	1.92E+00	8.23E-01	0.00E+00	1.00E+00	-5.13E-15	1.00E+00	0.00E+00	1.00E+00	0.00E+00	1.00E+00
ENSBTAG00000012846	2.85E-01	1.00E+00	5.52E-01	5.65E-01	5.37E-01	5.74E-01	2.14E-01	1.00E+00	-1.13E-02	1.00E+00
ENSBTAG00000027577	1.18E+00	2.68E-01	1.55E+00	9.54E-03	1.39E+00	2.20E-02	7.36E-01	3.67E-01	1.15E+00	3.68E-01
ENSBTAG00000044787	1.92E+00	8.23E-01	3.68E+00	5.09E-02	2.55E+00	2.54E-01	1.76E+00	4.97E-01	0.00E+00	1.00E+00
ENSBTAG00000002302	5.84E-02	1.00E+00	-3.79E-01	1.84E-01	-8.50E-01	3.74E-04	-7.37E-01	2.94E-03	5.89E-01	1.53E-01
ENSBTAG00000010071	-1.54E-01	9.71E-01	-6.91E-01	1.59E-04	-1.23E+00	3.65E-12	-9.56E-01	1.06E-07	-1.47E-01	9.63E-01
ENSBTAG00000006951	-7.95E-01	7.53E-03	-1.78E+00	7.53E-14	-3.74E-01	1.93E-01	-6.31E-01	1.34E-02	-3.76E-01	5.70E-01

ENSBTAG00000016744	-1.25E-01	1.00E+00	-7.29E-02	1.00E+00	6.34E-01	1.78E-03	5.24E-01	1.42E-02	3.96E-02	1.00E+00
ENSBTAG00000016747	-5.40E-02	1.00E+00	1.52E+00	2.74E-11	1.96E+00	6.07E-18	1.62E+00	1.42E-12	8.57E-02	1.00E+00
ENSBTAG00000005923	1.90E-01	1.00E+00	1.13E+00	1.68E-02	6.01E-01	2.86E-01	-5.20E-01	4.09E-01	2.06E-01	1.00E+00
ENSBTAG00000045400	0.00E+00	1.00E+00	0.00E+00	1.00E+00	-5.13E-15	1.00E+00	0.00E+00	1.00E+00	0.00E+00	1.00E+00
ENSBTAG00000020980	-3.10E-01	8.23E-01	-1.12E+00	3.64E-05	-2.66E+00	6.01E-23	-3.99E+00	5.68E-45	-2.79E-01	8.16E-01
ENSBTAG00000012460	-4.36E-01	7.86E-01	-2.43E+00	2.70E-08	-2.71E+00	2.82E-10	-2.89E+00	4.60E-11	1.65E-01	1.00E+00
ENSBTAG00000017150	1.80E-02	1.00E+00	1.45E+00	2.98E-02	2.13E+00	7.19E-04	2.61E+00	3.75E-05	-1.35E-01	1.00E+00
ENSBTAG00000018257	-3.17E-01	6.28E-01	-4.42E-01	8.23E-02	8.84E-01	5.45E-05	9.18E-01	3.30E-05	-4.83E-01	2.90E-01
ENSBTAG00000018261	-9.43E-03	1.00E+00	-1.22E-02	1.00E+00	5.74E-01	1.73E-01	3.88E-01	4.46E-01	-2.61E-01	1.00E+00
ENSBTAG00000044322	0.00E+00	1.00E+00	0.00E+00	1.00E+00	-5.13E-15	1.00E+00	0.00E+00	1.00E+00	0.00E+00	1.00E+00
ENSBTAG00000011578	1.44E+00	1.87E-08	2.21E+00	2.00E-20	1.31E+00	4.55E-08	8.25E-01	1.16E-03	-2.16E-01	9.24E-01
ENSBTAG00000012628	2.12E+00	9.64E-07	4.15E+00	3.46E-23	4.21E+00	7.53E-24	4.40E+00	1.88E-25	-5.63E-01	6.64E-01
ENSBTAG00000012630	3.39E-01	1.00E+00	-7.58E-01	3.48E-01	3.38E-01	8.25E-01	-3.56E-01	8.18E-01	4.21E-01	1.00E+00
ENSBTAG00000047083	-1.97E-02	1.00E+00	5.96E-01	3.67E-01	5.94E-01	3.62E-01	2.84E-01	8.45E-01	1.18E+00	1.70E-01
ENSBTAG00000037389	4.31E-01	3.18E-01	5.69E-02	1.00E+00	-1.46E-01	7.74E-01	-6.05E-01	1.28E-02	4.55E-01	3.29E-01
ENSBTAG00000044158	6.37E-01	4.32E-01	1.08E+00	4.80E-03	3.08E-01	6.18E-01	-3.49E-01	5.53E-01	5.85E-01	5.73E-01
ENSBTAG00000019045	-1.52E-01	1.00E+00	-8.22E-01	6.88E-04	-1.05E+00	8.08E-06	-7.44E-01	2.67E-03	-1.50E-01	1.00E+00
ENSBTAG00000021029	6.85E-05	1.00E+00	-3.45E-02	1.00E+00	6.92E-02	1.00E+00	1.79E-01	9.41E-01	3.29E-01	9.22E-01
ENSBTAG00000036009	7.74E-01	4.24E-03	1.60E+00	7.17E-14	9.04E-01	4.01E-05	1.55E-01	7.48E-01	1.63E-02	1.00E+00
ENSBTAG00000040293	0.00E+00	1.00E+00	0.00E+00	1.00E+00	-5.13E-15	1.00E+00	0.00E+00	1.00E+00	0.00E+00	1.00E+00
ENSBTAG00000031309	-7.80E-01	1.00E+00	-2.70E+00	2.42E-01	-1.53E-01	1.00E+00	1.24E+00	4.52E-01	1.73E-02	1.00E+00
ENSBTAG00000021611	2.17E-01	9.36E-01	-6.11E-01	2.16E-02	-2.71E-01	4.28E-01	-9.11E-02	1.00E+00	2.48E-01	8.18E-01
ENSBTAG00000044849	0.00E+00	1.00E+00	0.00E+00	1.00E+00	-5.13E-15	1.00E+00	0.00E+00	1.00E+00	0.00E+00	1.00E+00
ENSBTAG00000001623	1.31E+00	9.31E-01	7.64E-02	1.00E+00	6.51E-01	9.78E-01	6.27E-01	9.94E-01	1.68E+00	7.78E-01
ENSBTAG00000042407	0.00E+00	1.00E+00	0.00E+00	1.00E+00	-5.13E-15	1.00E+00	0.00E+00	1.00E+00	0.00E+00	1.00E+00
ENSBTAG00000029461	0.00E+00	1.00E+00	0.00E+00	1.00E+00	-5.13E-15	1.00E+00	0.00E+00	1.00E+00	0.00E+00	1.00E+00
ENSBTAG00000007324	2.72E+00	5.76E-01	3.30E+00	1.03E-01	3.73E+00	4.00E-02	3.04E+00	1.49E-01	0.00E+00	1.00E+00
ENSBTAG00000037580	-9.73E-01	5.24E-01	-2.40E+00	1.51E-03	-1.59E+00	1.92E-02	-4.15E-01	7.04E-01	-4.30E-01	1.00E+00
ENSBTAG00000000487	0.00E+00	1.00E+00	0.00E+00	1.00E+00	-5.13E-15	1.00E+00	0.00E+00	1.00E+00	0.00E+00	1.00E+00
ENSBTAG00000014474	2.63E-01	8.87E-01	6.38E-01	2.91E-02	8.33E-01	2.70E-03	9.32E-01	8.23E-04	2.49E-01	8.97E-01
ENSBTAG00000031299	1.79E-01	9.09E-01	2.61E-01	2.83E-01	2.84E-01	2.26E-01	5.86E-02	1.00E+00	3.20E-01	5.08E-01
ENSBTAG00000046968	0.00E+00	1.00E+00	0.00E+00	1.00E+00	-5.13E-15	1.00E+00	0.00E+00	1.00E+00	0.00E+00	1.00E+00
ENSBTAG00000030083	1.92E+00	8.23E-01	0.00E+00	1.00E+00	-5.13E-15	1.00E+00	0.00E+00	1.00E+00	0.00E+00	1.00E+00
ENSBTAG00000029939	0.00E+00	1.00E+00	0.00E+00	1.00E+00	-5.13E-15	1.00E+00	0.00E+00	1.00E+00	0.00E+00	1.00E+00
ENSBTAG00000000087	-1.45E-01	1.00E+00	-9.72E-02	1.00E+00	-4.01E-01	4.48E-01	-4.90E-01	3.24E-01	-1.40E-01	1.00E+00

ENSBTAG00000043088	0.00E+00	1.00E+00	0.00E+00	1.00E+00	-5.13E-15	1.00E+00	0.00E+00	1.00E+00	0.00E+00	1.00E+00
ENSBTAG00000009518	1.40E-01	1.00E+00	6.34E-01	2.79E-03	5.50E-01	1.10E-02	3.82E-01	1.14E-01	2.14E-02	1.00E+00
ENSBTAG00000045822	3.28E+00	3.65E-17	3.75E+00	1.54E-22	2.77E+00	9.45E-14	3.23E+00	1.16E-17	4.79E-01	7.46E-01
ENSBTAG00000012053	1.11E+00	7.42E-01	-5.93E-01	8.82E-01	-8.55E-01	6.58E-01	-1.37E+00	4.11E-01	2.69E+00	4.37E-03
ENSBTAG00000008465	5.31E-02	1.00E+00	-3.84E-01	1.35E-01	-7.05E-01	1.75E-03	-7.02E-01	2.23E-03	-8.72E-02	1.00E+00
ENSBTAG00000019644	-4.68E-02	1.00E+00	-3.65E-01	2.11E-01	-1.56E-01	7.70E-01	9.51E-02	1.00E+00	5.80E-01	1.70E-01
ENSBTAG00000027563	0.00E+00	1.00E+00	0.00E+00	1.00E+00	-5.13E-15	1.00E+00	0.00E+00	1.00E+00	0.00E+00	1.00E+00
ENSBTAG00000031252	1.39E-01	1.00E+00	-1.17E-01	9.77E-01	-9.73E-01	4.31E-04	-1.08E+00	1.10E-04	3.22E-02	1.00E+00
ENSBTAG00000043122	0.00E+00	1.00E+00	0.00E+00	1.00E+00	-5.13E-15	1.00E+00	0.00E+00	1.00E+00	0.00E+00	1.00E+00
ENSBTAG00000021484	-7.47E-01	6.70E-01	2.04E-01	9.90E-01	-1.10E+00	7.99E-02	2.54E-01	8.84E-01	-2.08E-01	1.00E+00
ENSBTAG00000017228	9.44E-01	7.30E-02	-4.94E-01	3.57E-01	-1.83E-01	9.29E-01	-2.80E-01	7.12E-01	-1.53E-01	1.00E+00
ENSBTAG00000006443	3.15E-01	8.99E-01	1.25E-02	1.00E+00	7.21E-01	4.07E-02	6.13E-01	1.05E-01	3.50E-01	8.00E-01
ENSBTAG00000031210	0.00E+00	1.00E+00	0.00E+00	1.00E+00	-5.13E-15	1.00E+00	0.00E+00	1.00E+00	0.00E+00	1.00E+00
ENSBTAG00000001703	1.98E+00	5.78E-01	8.80E-01	8.54E-01	-1.23E-01	1.00E+00	2.40E+00	9.35E-02	1.67E+00	7.86E-01
ENSBTAG00000003721	3.72E-01	8.90E-01	-1.81E+00	1.12E-04	-1.14E+00	1.07E-02	-1.34E+00	2.97E-03	-4.90E-03	1.00E+00
ENSBTAG00000003199	-5.04E-01	5.76E-01	-1.56E+00	1.33E-05	-7.59E-01	3.92E-02	-2.93E-01	5.88E-01	2.94E-01	9.02E-01
ENSBTAG00000021223	-1.78E+00	8.25E-11	-4.68E+00	7.75E-57	-3.52E+00	8.12E-38	-3.05E+00	6.10E-30	-4.02E-01	6.01E-01
ENSBTAG00000000317	2.33E-01	1.00E+00	2.09E+00	2.74E-06	-2.86E-01	8.53E-01	-1.53E-01	1.00E+00	1.12E+00	1.85E-01
ENSBTAG00000027562	9.27E-01	9.43E-01	4.98E+00	1.41E-16	1.92E+00	4.96E-02	7.37E-01	7.38E-01	1.32E+00	7.00E-01
ENSBTAG00000003126	-4.01E-01	2.91E-01	-2.67E-01	3.14E-01	2.08E-01	4.78E-01	3.78E-02	1.00E+00	6.00E-02	1.00E+00
ENSBTAG00000009607	-5.31E-01	2.70E-01	-2.92E+00	1.22E-25	-3.63E+00	1.41E-36	-2.30E+00	8.33E-18	-9.04E-03	1.00E+00
ENSBTAG00000009611	1.33E-02	1.00E+00	-9.72E-01	1.16E-04	-1.11E+00	6.75E-06	-6.71E-01	1.09E-02	1.94E-01	9.70E-01
ENSBTAG00000047243	0.00E+00	1.00E+00	0.00E+00	1.00E+00	-5.13E-15	1.00E+00	0.00E+00	1.00E+00	0.00E+00	1.00E+00
ENSBTAG00000006143	1.55E-01	1.00E+00	4.76E-01	3.19E-01	2.01E-01	8.61E-01	2.96E-01	6.44E-01	8.23E-02	1.00E+00
ENSBTAG00000013636	-4.29E-01	1.80E-01	-1.36E+00	4.68E-13	-8.71E-01	4.64E-06	-4.01E-01	6.68E-02	-1.33E-01	1.00E+00
ENSBTAG00000007740	-5.50E-01	6.19E-01	-9.34E-01	3.11E-02	-9.09E-01	3.12E-02	-7.13E-01	1.12E-01	-5.18E-01	6.95E-01
ENSBTAG00000033699	1.91E+00	8.23E-01	0.00E+00	1.00E+00	1.78E+00	4.79E-01	0.00E+00	1.00E+00	0.00E+00	1.00E+00
ENSBTAG00000004282	-7.36E-02	1.00E+00	5.86E-01	3.02E-03	5.94E-01	2.47E-03	3.95E-01	7.06E-02	1.34E-01	1.00E+00
ENSBTAG00000004639	-3.43E-02	1.00E+00	7.34E-02	1.00E+00	-2.68E-01	4.97E-01	-5.24E-01	9.97E-02	3.58E-01	7.11E-01
ENSBTAG00000017325	9.68E-02	1.00E+00	1.35E+00	9.39E-06	8.94E-01	4.69E-03	1.03E-01	1.00E+00	6.46E-01	3.05E-01
ENSBTAG00000016501	3.92E-02	1.00E+00	-7.58E-01	1.02E-03	-1.03E+00	3.60E-06	-6.53E-01	6.32E-03	3.89E-01	4.82E-01
ENSBTAG00000027557	-2.00E-02	1.00E+00	1.41E+00	4.48E-13	1.02E+00	1.90E-07	6.43E-01	2.20E-03	5.13E-02	1.00E+00
ENSBTAG00000007148	1.32E-01	1.00E+00	3.74E-02	1.00E+00	-2.23E-01	3.90E-01	-4.31E-01	3.94E-02	4.97E-01	1.14E-01
ENSBTAG00000004448	3.51E-01	6.32E-01	1.89E+00	3.93E-15	8.62E-01	5.86E-04	4.36E-01	1.41E-01	1.93E-01	9.87E-01
ENSBTAG00000043098	0.00E+00	1.00E+00	0.00E+00	1.00E+00	-5.13E-15	1.00E+00	0.00E+00	1.00E+00	0.00E+00	1.00E+00

ENSBTAG00000045369	0.00E+00	1.00E+00	0.00E+00	1.00E+00	-5.13E-15	1.00E+00	0.00E+00	1.00E+00	0.00E+00	1.00E+00
ENSBTAG00000008429	1.45E-01	1.00E+00	-4.85E-01	1.02E-01	3.74E-01	2.44E-01	7.22E-01	8.27E-03	3.72E-01	6.25E-01
ENSBTAG00000047298	0.00E+00	1.00E+00	0.00E+00	1.00E+00	-5.13E-15	1.00E+00	1.76E+00	4.97E-01	0.00E+00	1.00E+00
ENSBTAG00000012546	0.00E+00	1.00E+00	0.00E+00	1.00E+00	-5.13E-15	1.00E+00	0.00E+00	1.00E+00	0.00E+00	1.00E+00
ENSBTAG00000038160	0.00E+00	1.00E+00	0.00E+00	1.00E+00	-5.13E-15	1.00E+00	0.00E+00	1.00E+00	0.00E+00	1.00E+00
ENSBTAG00000016082	-1.91E+00	8.23E-01	-1.91E+00	4.69E-01	-1.91E+00	4.28E-01	-1.91E+00	4.39E-01	-1.91E+00	8.00E-01
ENSBTAG00000046354	0.00E+00	1.00E+00	0.00E+00	1.00E+00	-5.13E-15	1.00E+00	0.00E+00	1.00E+00	0.00E+00	1.00E+00
ENSBTAG00000009573	6.47E-01	4.33E-01	2.26E+00	1.02E-09	3.38E-01	5.65E-01	-3.90E-01	4.97E-01	5.02E-01	7.03E-01
ENSBTAG00000044593	0.00E+00	1.00E+00	0.00E+00	1.00E+00	-5.13E-15	1.00E+00	0.00E+00	1.00E+00	0.00E+00	1.00E+00
ENSBTAG00000009574	4.25E-02	1.00E+00	5.37E-01	8.23E-03	7.78E-01	5.48E-05	6.45E-01	1.29E-03	-5.12E-02	1.00E+00
ENSBTAG00000009576	-5.75E-01	4.03E-01	-2.16E+00	2.34E-11	-2.83E+00	5.32E-18	-1.10E+00	1.12E-03	-1.16E-01	1.00E+00
ENSBTAG00000020999	-3.45E-01	4.15E-01	-1.79E+00	1.36E-20	-2.11E+00	8.17E-28	-2.10E+00	1.68E-27	1.38E-01	1.00E+00
ENSBTAG00000021002	-3.01E-01	8.58E-01	-1.55E+00	1.88E-07	-1.84E+00	4.89E-10	-1.41E+00	2.79E-06	4.21E-01	6.44E-01
ENSBTAG00000010681	-3.72E-01	5.80E-01	-1.08E+00	1.61E-05	-1.56E+00	2.30E-10	-1.51E+00	1.21E-09	-3.38E-01	6.97E-01
ENSBTAG00000021700	-3.59E-01	5.52E-01	-2.82E+00	2.33E-33	-2.91E+00	1.03E-35	-1.45E+00	1.30E-10	2.44E-01	8.00E-01
ENSBTAG00000021707	-1.49E+00	4.33E-01	-6.49E-01	6.20E-01	-5.76E-01	6.58E-01	-1.15E+00	2.60E-01	-1.15E-01	1.00E+00
ENSBTAG00000021709	4.24E-01	3.44E-01	9.23E-01	3.27E-05	6.46E-01	5.25E-03	1.28E-01	8.56E-01	-4.75E-02	1.00E+00
ENSBTAG00000019436	9.47E-02	1.00E+00	-2.03E-01	5.12E-01	1.39E-01	7.54E-01	6.90E-01	1.08E-03	3.12E-01	6.01E-01
ENSBTAG00000021744	-1.45E-01	1.00E+00	-1.15E-01	8.76E-01	-1.15E-01	8.72E-01	-8.70E-02	9.94E-01	-8.57E-02	1.00E+00
ENSBTAG00000021745	-4.98E-01	6.66E-01	-3.01E+00	5.74E-11	-3.96E+00	6.07E-16	-3.83E+00	2.17E-15	5.27E-01	5.98E-01
ENSBTAG00000002520	3.30E-01	4.80E-01	1.02E-01	9.06E-01	2.31E-02	1.00E+00	3.10E-01	2.10E-01	3.60E-01	4.36E-01
ENSBTAG00000018479	-1.45E-01	1.00E+00	-3.84E-01	4.76E-01	2.06E-01	8.61E-01	4.47E-01	3.95E-01	-2.47E-01	1.00E+00
ENSBTAG00000018482	-5.60E-01	1.40E-01	-3.24E-01	2.88E-01	3.68E-01	1.88E-01	5.40E-01	3.39E-02	5.54E-02	1.00E+00
ENSBTAG00000018483	-2.23E-01	9.40E-01	-3.72E-01	2.30E-01	-1.91E-01	6.75E-01	-1.83E-01	7.12E-01	-2.11E-01	9.37E-01
ENSBTAG00000018742	-8.26E-02	1.00E+00	-9.13E-01	1.57E-04	-1.29E+00	4.08E-08	-9.79E-01	5.51E-05	-5.56E-02	1.00E+00
ENSBTAG00000020911	2.28E-01	9.66E-01	5.96E-01	4.11E-02	4.65E-01	1.36E-01	-2.76E-01	4.94E-01	2.22E-01	9.46E-01
ENSBTAG00000021125	1.29E-01	1.00E+00	1.31E+00	5.72E-07	1.89E+00	2.01E-13	1.59E+00	9.87E-10	1.44E-01	1.00E+00
ENSBTAG00000024715	9.43E-02	1.00E+00	2.59E-02	1.00E+00	5.10E-01	1.28E-01	6.16E-01	5.79E-02	2.50E-01	9.23E-01
ENSBTAG00000005914	0.00E+00	1.00E+00	0.00E+00	1.00E+00	-5.13E-15	1.00E+00	0.00E+00	1.00E+00	0.00E+00	1.00E+00
ENSBTAG00000000926	-7.87E-01	1.00E+00	6.03E-01	9.45E-01	-2.70E+00	1.99E-01	-2.70E+00	2.05E-01	7.15E-03	1.00E+00
ENSBTAG00000031025	1.19E-02	1.00E+00	7.64E-02	1.00E+00	-1.30E-01	1.00E+00	6.21E-01	9.94E-01	-1.91E+00	8.00E-01
ENSBTAG00000031026	-1.91E+00	8.23E-01	-1.91E+00	4.69E-01	-1.32E-01	1.00E+00	-1.54E-01	1.00E+00	-1.91E+00	8.00E-01
ENSBTAG00000031119	-1.20E+00	2.30E-01	1.30E+00	3.93E-03	-3.22E-03	1.00E+00	-2.86E-01	8.49E-01	-5.31E-01	8.30E-01
ENSBTAG00000031029	6.60E-01	9.37E-01	1.36E+00	1.11E-01	4.19E-01	8.45E-01	1.12E-01	1.00E+00	2.77E-01	1.00E+00
ENSBTAG00000031028	8.54E-01	5.76E-01	1.70E+00	1.99E-03	9.09E-01	1.65E-01	-4.07E-01	7.63E-01	6.24E-01	8.00E-01

ENSBTAG00000031027	2.59E-01	1.00E+00	2.15E+00	5.42E-06	8.44E-01	1.63E-01	-4.83E-01	6.16E-01	6.73E-01	7.38E-01
ENSBTAG00000024788	1.16E-01	1.00E+00	5.77E-01	5.04E-01	-3.77E-01	8.18E-01	-1.47E+00	7.10E-02	-3.10E-01	1.00E+00
ENSBTAG00000031030	1.21E-01	1.00E+00	2.45E-01	9.20E-01	-4.71E-01	5.41E-01	-6.88E-01	3.11E-01	3.56E-01	1.00E+00
ENSBTAG00000012549	1.18E+00	8.65E-01	2.36E+00	3.09E-02	3.49E-01	1.00E+00	-2.71E+00	2.05E-01	1.66E+00	6.01E-01
ENSBTAG00000008379	-2.92E+00	9.02E-02	-4.01E-01	9.21E-01	-2.29E+00	3.79E-02	-3.08E+00	1.08E-02	-9.34E-01	8.00E-01
ENSBTAG00000048031	-1.81E-01	1.00E+00	9.73E-01	3.03E-01	-7.96E-01	5.74E-01	-1.50E+00	2.27E-01	3.16E-01	1.00E+00
ENSBTAG00000031032	1.14E-01	1.00E+00	-1.38E-01	1.00E+00	-2.10E+00	1.19E-02	-1.88E+00	2.50E-02	-8.46E-01	7.79E-01
ENSBTAG00000016090	-1.29E+00	9.39E-01	4.75E-01	9.90E-01	-1.43E+00	4.79E-01	-1.45E+00	4.97E-01	3.80E-01	1.00E+00
ENSBTAG00000030909	-3.86E-01	1.00E+00	-1.24E+00	7.17E-02	-1.63E+00	1.33E-02	-3.90E+00	2.75E-06	-4.17E-01	1.00E+00
ENSBTAG00000039821	1.57E-01	1.00E+00	-9.93E-01	4.54E-01	-9.51E-01	4.44E-01	-2.14E+00	6.99E-02	7.53E-01	8.06E-01
ENSBTAG00000047026	8.42E-01	7.41E-01	2.86E-01	9.87E-01	-1.18E+00	1.99E-01	-2.30E+00	1.12E-02	3.89E-01	1.00E+00
ENSBTAG00000048037	3.15E-01	1.00E+00	-4.51E-01	8.05E-01	-1.64E+00	5.37E-02	-2.91E+00	9.92E-04	2.50E-01	1.00E+00
ENSBTAG00000035489	5.82E-01	1.00E+00	-4.28E-01	9.77E-01	-5.12E-01	8.98E-01	-2.16E+00	7.51E-02	-8.80E-02	1.00E+00
ENSBTAG00000043042	0.00E+00	1.00E+00	1.98E+00	4.43E-01	-5.13E-15	1.00E+00	0.00E+00	1.00E+00	1.93E+00	8.00E-01
ENSBTAG00000035487	-2.70E+00	5.76E-01	-7.16E-01	9.45E-01	-2.70E+00	1.99E-01	-2.70E+00	2.05E-01	-7.74E-01	1.00E+00
ENSBTAG00000027275	-1.07E+00	9.09E-01	-3.48E-01	1.00E+00	-1.25E+00	4.28E-01	-4.32E+00	1.13E-02	1.40E+00	5.86E-01
ENSBTAG00000037666	1.91E+00	8.23E-01	0.00E+00	1.00E+00	-5.13E-15	1.00E+00	0.00E+00	1.00E+00	0.00E+00	1.00E+00
ENSBTAG00000046923	0.00E+00	1.00E+00	0.00E+00	1.00E+00	-5.13E-15	1.00E+00	0.00E+00	1.00E+00	0.00E+00	1.00E+00
ENSBTAG00000010005	0.00E+00	1.00E+00	0.00E+00	1.00E+00	-5.13E-15	1.00E+00	0.00E+00	1.00E+00	0.00E+00	1.00E+00
ENSBTAG00000011521	1.91E+00	8.23E-01	0.00E+00	1.00E+00	-5.13E-15	1.00E+00	0.00E+00	1.00E+00	1.91E+00	8.00E-01
ENSBTAG00000035485	0.00E+00	1.00E+00	0.00E+00	1.00E+00	-5.13E-15	1.00E+00	0.00E+00	1.00E+00	0.00E+00	1.00E+00
ENSBTAG00000040425	0.00E+00	1.00E+00	0.00E+00	1.00E+00	-5.13E-15	1.00E+00	0.00E+00	1.00E+00	0.00E+00	1.00E+00
ENSBTAG00000039272	0.00E+00	1.00E+00	0.00E+00	1.00E+00	-5.13E-15	1.00E+00	0.00E+00	1.00E+00	0.00E+00	1.00E+00
ENSBTAG00000040341	-1.91E+00	8.23E-01	7.51E-02	1.00E+00	-1.91E+00	4.28E-01	-1.91E+00	4.39E-01	-1.91E+00	8.00E-01
ENSBTAG00000005624	0.00E+00	1.00E+00	0.00E+00	1.00E+00	-5.13E-15	1.00E+00	0.00E+00	1.00E+00	0.00E+00	1.00E+00
ENSBTAG00000007944	0.00E+00	1.00E+00	0.00E+00	1.00E+00	-5.13E-15	1.00E+00	0.00E+00	1.00E+00	0.00E+00	1.00E+00
ENSBTAG00000018890	0.00E+00	1.00E+00	0.00E+00	1.00E+00	-5.13E-15	1.00E+00	0.00E+00	1.00E+00	0.00E+00	1.00E+00
ENSBTAG00000020746	0.00E+00	1.00E+00	0.00E+00	1.00E+00	-5.13E-15	1.00E+00	0.00E+00	1.00E+00	0.00E+00	1.00E+00
ENSBTAG00000015876	0.00E+00	1.00E+00	0.00E+00	1.00E+00	-5.13E-15	1.00E+00	0.00E+00	1.00E+00	0.00E+00	1.00E+00
ENSBTAG00000005087	0.00E+00	1.00E+00	0.00E+00	1.00E+00	-5.13E-15	1.00E+00	0.00E+00	1.00E+00	0.00E+00	1.00E+00
ENSBTAG00000003056	0.00E+00	1.00E+00	0.00E+00	1.00E+00	-5.13E-15	1.00E+00	0.00E+00	1.00E+00	0.00E+00	1.00E+00
ENSBTAG00000014594	0.00E+00	1.00E+00	0.00E+00	1.00E+00	-5.13E-15	1.00E+00	0.00E+00	1.00E+00	0.00E+00	1.00E+00
ENSBTAG00000046471	0.00E+00	1.00E+00	0.00E+00	1.00E+00	-5.13E-15	1.00E+00	0.00E+00	1.00E+00	0.00E+00	1.00E+00
ENSBTAG00000023511	0.00E+00	1.00E+00	0.00E+00	1.00E+00	-5.13E-15	1.00E+00	0.00E+00	1.00E+00	0.00E+00	1.00E+00
ENSBTAG00000048141	0.00E+00	1.00E+00	0.00E+00	1.00E+00	1.78E+00	4.79E-01	0.00E+00	1.00E+00	0.00E+00	1.00E+00

ENSBTAG00000005874	0.00E+00	1.00E+00	0.00E+00	1.00E+00	-5.13E-15	1.00E+00	0.00E+00	1.00E+00	0.00E+00	1.00E+00
ENSBTAG00000046251	0.00E+00	1.00E+00	0.00E+00	1.00E+00	-5.13E-15	1.00E+00	0.00E+00	1.00E+00	0.00E+00	1.00E+00
ENSBTAG00000039331	0.00E+00	1.00E+00	0.00E+00	1.00E+00	-5.13E-15	1.00E+00	0.00E+00	1.00E+00	0.00E+00	1.00E+00
ENSBTAG00000030920	3.89E-01	1.00E+00	7.70E-01	7.36E-01	-6.48E-01	9.01E-01	1.11E+00	4.44E-01	6.98E-01	1.00E+00
ENSBTAG00000001383	0.00E+00	1.00E+00	0.00E+00	1.00E+00	-5.13E-15	1.00E+00	3.03E+00	1.50E-01	0.00E+00	1.00E+00
ENSBTAG00000030925	0.00E+00	1.00E+00	0.00E+00	1.00E+00	-5.13E-15	1.00E+00	0.00E+00	1.00E+00	0.00E+00	1.00E+00
ENSBTAG00000046527	0.00E+00	1.00E+00	0.00E+00	1.00E+00	-5.13E-15	1.00E+00	0.00E+00	1.00E+00	0.00E+00	1.00E+00
ENSBTAG00000046285	0.00E+00	1.00E+00	0.00E+00	1.00E+00	-5.13E-15	1.00E+00	0.00E+00	1.00E+00	0.00E+00	1.00E+00
ENSBTAG00000001291	0.00E+00	1.00E+00	0.00E+00	1.00E+00	1.78E+00	4.79E-01	0.00E+00	1.00E+00	0.00E+00	1.00E+00
ENSBTAG00000014309	0.00E+00	1.00E+00	0.00E+00	1.00E+00	-5.13E-15	1.00E+00	0.00E+00	1.00E+00	1.91E+00	8.00E-01
ENSBTAG00000037878	-1.91E+00	8.23E-01	-1.91E+00	4.69E-01	-1.91E+00	4.28E-01	-1.91E+00	4.39E-01	-1.91E+00	8.00E-01
ENSBTAG00000046278	0.00E+00	1.00E+00	0.00E+00	1.00E+00	-5.13E-15	1.00E+00	0.00E+00	1.00E+00	0.00E+00	1.00E+00
ENSBTAG00000045709	0.00E+00	1.00E+00	0.00E+00	1.00E+00	-5.13E-15	1.00E+00	0.00E+00	1.00E+00	0.00E+00	1.00E+00
ENSBTAG00000035988	0.00E+00	1.00E+00	0.00E+00	1.00E+00	-5.13E-15	1.00E+00	0.00E+00	1.00E+00	0.00E+00	1.00E+00
ENSBTAG00000035986	0.00E+00	1.00E+00	0.00E+00	1.00E+00	-5.13E-15	1.00E+00	0.00E+00	1.00E+00	0.00E+00	1.00E+00
ENSBTAG00000035985	0.00E+00	1.00E+00	0.00E+00	1.00E+00	-5.13E-15	1.00E+00	0.00E+00	1.00E+00	0.00E+00	1.00E+00
ENSBTAG00000046710	0.00E+00	1.00E+00	0.00E+00	1.00E+00	-5.13E-15	1.00E+00	0.00E+00	1.00E+00	0.00E+00	1.00E+00
ENSBTAG00000046274	0.00E+00	1.00E+00	0.00E+00	1.00E+00	-5.13E-15	1.00E+00	0.00E+00	1.00E+00	0.00E+00	1.00E+00
ENSBTAG00000035059	1.92E+00	8.23E-01	0.00E+00	1.00E+00	1.78E+00	4.79E-01	0.00E+00	1.00E+00	0.00E+00	1.00E+00
ENSBTAG00000022858	0.00E+00	1.00E+00	0.00E+00	1.00E+00	-5.13E-15	1.00E+00	0.00E+00	1.00E+00	0.00E+00	1.00E+00
ENSBTAG00000023310	-1.91E+00	8.23E-01	-1.91E+00	4.69E-01	-1.91E+00	4.28E-01	-1.91E+00	4.39E-01	-1.91E+00	8.00E-01
ENSBTAG00000024423	0.00E+00	1.00E+00	0.00E+00	1.00E+00	-5.13E-15	1.00E+00	0.00E+00	1.00E+00	0.00E+00	1.00E+00
ENSBTAG00000046845	0.00E+00	1.00E+00	0.00E+00	1.00E+00	-5.13E-15	1.00E+00	0.00E+00	1.00E+00	0.00E+00	1.00E+00
ENSBTAG00000035990	0.00E+00	1.00E+00	0.00E+00	1.00E+00	-5.13E-15	1.00E+00	0.00E+00	1.00E+00	0.00E+00	1.00E+00
ENSBTAG00000039348	0.00E+00	1.00E+00	0.00E+00	1.00E+00	-5.13E-15	1.00E+00	0.00E+00	1.00E+00	0.00E+00	1.00E+00
ENSBTAG00000038049	0.00E+00	1.00E+00	1.99E+00	4.43E-01	-5.13E-15	1.00E+00	0.00E+00	1.00E+00	0.00E+00	1.00E+00
ENSBTAG00000014513	0.00E+00	1.00E+00	0.00E+00	1.00E+00	-5.13E-15	1.00E+00	0.00E+00	1.00E+00	0.00E+00	1.00E+00
ENSBTAG00000035989	0.00E+00	1.00E+00	0.00E+00	1.00E+00	-5.13E-15	1.00E+00	0.00E+00	1.00E+00	0.00E+00	1.00E+00
ENSBTAG00000045723	0.00E+00	1.00E+00	0.00E+00	1.00E+00	-5.13E-15	1.00E+00	0.00E+00	1.00E+00	0.00E+00	1.00E+00
ENSBTAG00000046167	0.00E+00	1.00E+00	0.00E+00	1.00E+00	-5.13E-15	1.00E+00	0.00E+00	1.00E+00	0.00E+00	1.00E+00
ENSBTAG00000035031	0.00E+00	1.00E+00	0.00E+00	1.00E+00	-5.13E-15	1.00E+00	0.00E+00	1.00E+00	0.00E+00	1.00E+00
ENSBTAG00000035029	0.00E+00	1.00E+00	0.00E+00	1.00E+00	-5.13E-15	1.00E+00	0.00E+00	1.00E+00	0.00E+00	1.00E+00
ENSBTAG00000047721	0.00E+00	1.00E+00	0.00E+00	1.00E+00	-5.13E-15	1.00E+00	0.00E+00	1.00E+00	0.00E+00	1.00E+00
ENSBTAG00000046801	1.91E+00	8.23E-01	0.00E+00	1.00E+00	-5.13E-15	1.00E+00	0.00E+00	1.00E+00	0.00E+00	1.00E+00
ENSBTAG00000038479	0.00E+00	1.00E+00	0.00E+00	1.00E+00	-5.13E-15	1.00E+00	0.00E+00	1.00E+00	0.00E+00	1.00E+00

ENSBTAG00000039110	0.00E+00	1.00E+00	1.99E+00	4.43E-01	-5.13E-15	1.00E+00	0.00E+00	1.00E+00	0.00E+00	1.00E+00
ENSBTAG00000045739	0.00E+00	1.00E+00	0.00E+00	1.00E+00	-5.13E-15	1.00E+00	0.00E+00	1.00E+00	0.00E+00	1.00E+00
ENSBTAG00000039182	0.00E+00	1.00E+00	0.00E+00	1.00E+00	-5.13E-15	1.00E+00	0.00E+00	1.00E+00	0.00E+00	1.00E+00
ENSBTAG00000040330	0.00E+00	1.00E+00	0.00E+00	1.00E+00	-5.13E-15	1.00E+00	0.00E+00	1.00E+00	0.00E+00	1.00E+00
ENSBTAG00000016146	1.91E+00	8.23E-01	0.00E+00	1.00E+00	-5.13E-15	1.00E+00	0.00E+00	1.00E+00	0.00E+00	1.00E+00
ENSBTAG00000023542	0.00E+00	1.00E+00	0.00E+00	1.00E+00	-5.13E-15	1.00E+00	0.00E+00	1.00E+00	1.91E+00	8.00E-01
ENSBTAG00000046187	1.91E+00	8.23E-01	0.00E+00	1.00E+00	-5.13E-15	1.00E+00	0.00E+00	1.00E+00	0.00E+00	1.00E+00
ENSBTAG00000006421	0.00E+00	1.00E+00	0.00E+00	1.00E+00	-5.13E-15	1.00E+00	1.76E+00	4.97E-01	0.00E+00	1.00E+00
ENSBTAG00000036130	0.00E+00	1.00E+00	0.00E+00	1.00E+00	-5.13E-15	1.00E+00	0.00E+00	1.00E+00	0.00E+00	1.00E+00
ENSBTAG00000039801	0.00E+00	1.00E+00	0.00E+00	1.00E+00	-5.13E-15	1.00E+00	0.00E+00	1.00E+00	0.00E+00	1.00E+00
ENSBTAG00000036131	0.00E+00	1.00E+00	0.00E+00	1.00E+00	-5.13E-15	1.00E+00	0.00E+00	1.00E+00	0.00E+00	1.00E+00
ENSBTAG00000040463	-1.91E+00	8.23E-01	-1.91E+00	4.69E-01	-1.91E+00	4.28E-01	-1.91E+00	4.39E-01	-1.91E+00	8.00E-01
ENSBTAG00000037928	0.00E+00	1.00E+00	1.98E+00	4.43E-01	-5.13E-15	1.00E+00	0.00E+00	1.00E+00	0.00E+00	1.00E+00
ENSBTAG00000038539	0.00E+00	1.00E+00	0.00E+00	1.00E+00	1.78E+00	4.79E-01	0.00E+00	1.00E+00	0.00E+00	1.00E+00
ENSBTAG00000031017	1.21E-02	1.00E+00	8.83E-01	8.52E-01	-1.25E-01	1.00E+00	2.72E+00	3.49E-02	1.31E+00	8.99E-01
ENSBTAG00000023202	0.00E+00	1.00E+00	0.00E+00	1.00E+00	-5.13E-15	1.00E+00	1.76E+00	4.97E-01	1.91E+00	8.00E-01
ENSBTAG00000024952	0.00E+00	1.00E+00	0.00E+00	1.00E+00	-5.13E-15	1.00E+00	0.00E+00	1.00E+00	0.00E+00	1.00E+00
ENSBTAG00000039448	0.00E+00	1.00E+00	0.00E+00	1.00E+00	-5.13E-15	1.00E+00	0.00E+00	1.00E+00	0.00E+00	1.00E+00
ENSBTAG00000004206	-4.17E-01	1.00E+00	-2.97E+00	1.55E-02	-2.41E+00	2.58E-02	-3.20E+00	7.07E-03	2.98E-01	1.00E+00
ENSBTAG00000027516	-7.22E-02	1.00E+00	-8.00E-01	2.99E-02	-1.24E+00	3.43E-04	-1.70E+00	7.75E-07	-5.00E-01	6.09E-01
ENSBTAG00000016469	3.97E-01	4.17E-01	-5.35E-01	2.95E-02	-1.06E+00	2.27E-06	-3.71E-01	1.83E-01	1.87E-01	9.51E-01
ENSBTAG00000000375	-3.81E-02	1.00E+00	4.94E-01	2.26E-02	8.36E-01	3.01E-05	6.96E-01	8.13E-04	-3.71E-02	1.00E+00
ENSBTAG00000000376	-3.57E-01	1.00E+00	-1.23E+00	1.44E-01	-5.43E-01	5.92E-01	-2.10E-01	1.00E+00	-6.73E-01	8.22E-01
ENSBTAG00000020285	0.00E+00	1.00E+00	1.98E+00	4.43E-01	-5.13E-15	1.00E+00	0.00E+00	1.00E+00	0.00E+00	1.00E+00
ENSBTAG00000002007	1.69E+00	7.71E-01	8.84E-01	8.52E-01	-1.25E-01	1.00E+00	-1.41E-01	1.00E+00	7.99E-01	1.00E+00
ENSBTAG00000040320	0.00E+00	1.00E+00	0.00E+00	1.00E+00	-5.13E-15	1.00E+00	0.00E+00	1.00E+00	0.00E+00	1.00E+00
ENSBTAG00000010060	6.07E-01	5.76E-01	1.68E+00	1.27E-05	1.66E+00	1.38E-05	1.62E+00	3.11E-05	8.50E-02	1.00E+00
ENSBTAG00000012621	-6.13E-01	8.88E-01	-1.85E+00	2.32E-02	-6.94E-01	4.28E-01	-1.02E-01	1.00E+00	-8.64E-01	7.13E-01
ENSBTAG00000024701	-3.69E-02	1.00E+00	7.17E-01	4.87E-03	1.07E+00	9.82E-06	9.85E-01	6.62E-05	-3.74E-01	6.01E-01
ENSBTAG00000027524	2.11E-01	1.00E+00	9.69E-01	2.23E-02	-6.50E-01	2.36E-01	-2.64E-01	8.10E-01	3.88E-02	1.00E+00
ENSBTAG00000012989	7.37E-01	1.10E-01	5.15E-01	1.45E-01	4.59E-01	2.11E-01	8.54E-01	7.53E-03	6.64E-01	2.60E-01
ENSBTAG00000016267	1.43E-01	1.00E+00	1.99E-01	4.69E-01	-2.64E-01	2.82E-01	-3.80E-01	8.74E-02	3.26E-01	5.03E-01
ENSBTAG00000030122	0.00E+00	1.00E+00	0.00E+00	1.00E+00	-5.13E-15	1.00E+00	0.00E+00	1.00E+00	0.00E+00	1.00E+00
ENSBTAG00000006493	2.09E-01	8.90E-01	1.14E+00	4.96E-08	1.12E+00	6.60E-08	5.55E-01	1.65E-02	8.78E-04	1.00E+00
ENSBTAG00000002411	-2.30E-01	9.37E-01	-3.60E-01	2.67E-01	-5.07E-01	8.05E-02	-3.40E-01	3.19E-01	2.97E-01	8.00E-01

ENSBTAG00000002381	-5.51E-02	1.00E+00	7.01E-01	6.80E-04	7.10E-01	5.36E-04	6.28E-01	3.14E-03	5.75E-02	1.00E+00
ENSBTAG00000002389	1.33E-01	1.00E+00	1.03E+00	7.48E-06	5.93E-01	1.60E-02	1.73E-01	7.06E-01	-3.11E-01	7.21E-01
ENSBTAG00000002404	-1.02E-01	1.00E+00	6.66E-02	1.00E+00	2.75E-01	3.35E-01	4.58E-01	6.10E-02	-2.38E-01	8.00E-01
ENSBTAG00000047968	5.17E-01	1.00E+00	9.72E-01	6.56E-01	1.65E+00	2.09E-01	3.36E-01	1.00E+00	-2.71E+00	6.01E-01
ENSBTAG00000047396	0.00E+00	1.00E+00	0.00E+00	1.00E+00	-5.13E-15	1.00E+00	0.00E+00	1.00E+00	0.00E+00	1.00E+00
ENSBTAG00000007971	1.92E+00	8.23E-01	1.98E+00	4.43E-01	-5.13E-15	1.00E+00	0.00E+00	1.00E+00	1.93E+00	8.00E-01
ENSBTAG00000039893	-3.21E+00	3.75E-01	-1.22E+00	6.15E-01	-3.21E+00	9.50E-02	-3.21E+00	9.97E-02	-3.21E+00	4.34E-01
ENSBTAG00000038267	1.92E+00	8.23E-01	0.00E+00	1.00E+00	-5.13E-15	1.00E+00	0.00E+00	1.00E+00	3.25E+00	4.34E-01
ENSBTAG00000030908	1.99E-02	1.00E+00	-7.16E-01	9.45E-01	-9.16E-01	8.10E-01	-9.39E-01	8.06E-01	1.89E-02	1.00E+00
ENSBTAG00000040150	0.00E+00	1.00E+00	0.00E+00	1.00E+00	-5.13E-15	1.00E+00	0.00E+00	1.00E+00	0.00E+00	1.00E+00
ENSBTAG00000021375	0.00E+00	1.00E+00	1.99E+00	4.43E-01	-5.13E-15	1.00E+00	0.00E+00	1.00E+00	0.00E+00	1.00E+00
ENSBTAG00000040197	0.00E+00	1.00E+00	1.98E+00	4.43E-01	-5.13E-15	1.00E+00	0.00E+00	1.00E+00	0.00E+00	1.00E+00
ENSBTAG00000039306	0.00E+00	1.00E+00	0.00E+00	1.00E+00	-5.13E-15	1.00E+00	0.00E+00	1.00E+00	0.00E+00	1.00E+00
ENSBTAG00000039917	0.00E+00	1.00E+00	0.00E+00	1.00E+00	-5.13E-15	1.00E+00	0.00E+00	1.00E+00	0.00E+00	1.00E+00
ENSBTAG00000037853	-2.70E+00	5.76E-01	-2.70E+00	2.42E-01	-2.70E+00	1.99E-01	-2.70E+00	2.05E-01	-2.70E+00	6.01E-01
ENSBTAG00000014572	-1.91E+00	8.23E-01	-1.91E+00	4.69E-01	-1.91E+00	4.28E-01	-1.91E+00	4.39E-01	1.77E-02	1.00E+00
ENSBTAG00000021233	0.00E+00	1.00E+00	0.00E+00	1.00E+00	-5.13E-15	1.00E+00	0.00E+00	1.00E+00	0.00E+00	1.00E+00
ENSBTAG00000039592	0.00E+00	1.00E+00	0.00E+00	1.00E+00	-5.13E-15	1.00E+00	0.00E+00	1.00E+00	0.00E+00	1.00E+00
ENSBTAG00000039300	0.00E+00	1.00E+00	0.00E+00	1.00E+00	-5.13E-15	1.00E+00	0.00E+00	1.00E+00	0.00E+00	1.00E+00
ENSBTAG00000013503	0.00E+00	1.00E+00	0.00E+00	1.00E+00	-5.13E-15	1.00E+00	0.00E+00	1.00E+00	0.00E+00	1.00E+00
ENSBTAG00000027942	0.00E+00	1.00E+00	0.00E+00	1.00E+00	-5.13E-15	1.00E+00	0.00E+00	1.00E+00	0.00E+00	1.00E+00
ENSBTAG00000036042	0.00E+00	1.00E+00	0.00E+00	1.00E+00	-5.13E-15	1.00E+00	0.00E+00	1.00E+00	0.00E+00	1.00E+00
ENSBTAG00000047464	0.00E+00	1.00E+00	0.00E+00	1.00E+00	-5.13E-15	1.00E+00	0.00E+00	1.00E+00	0.00E+00	1.00E+00
ENSBTAG00000046380	0.00E+00	1.00E+00	0.00E+00	1.00E+00	-5.13E-15	1.00E+00	0.00E+00	1.00E+00	0.00E+00	1.00E+00
ENSBTAG00000046460	0.00E+00	1.00E+00	0.00E+00	1.00E+00	-5.13E-15	1.00E+00	0.00E+00	1.00E+00	0.00E+00	1.00E+00
ENSBTAG00000036260	-4.69E-01	3.46E-01	-7.06E-01	7.10E-03	-6.38E-01	1.55E-02	-4.66E-01	1.06E-01	-4.04E-01	5.46E-01
ENSBTAG00000024657	-5.42E-02	1.00E+00	3.37E-01	1.85E-01	6.77E-01	1.51E-03	1.09E+00	1.19E-07	3.87E-01	4.34E-01
ENSBTAG00000003624	2.63E+00	2.08E-01	2.87E+00	2.45E-02	2.45E+00	7.35E-02	2.24E+00	1.32E-01	2.97E+00	1.36E-01
ENSBTAG00000045939	2.44E+00	2.97E-01	1.39E+00	5.17E-01	1.52E+00	4.28E-01	1.49E+00	4.56E-01	2.44E+00	3.68E-01
ENSBTAG00000038969	6.78E-01	1.00E+00	-2.13E+00	1.03E-01	-3.15E+00	1.62E-02	-4.92E+00	1.62E-03	-1.40E-01	1.00E+00
ENSBTAG00000038058	-6.25E-01	8.84E-01	-2.14E+00	1.57E-03	-4.12E+00	3.24E-09	-4.96E+00	7.21E-12	-3.13E-01	1.00E+00
ENSBTAG00000038323	-1.70E-01	1.00E+00	-1.31E+00	1.71E-06	-2.73E+00	4.51E-23	-3.24E+00	1.01E-30	-2.10E-02	1.00E+00
ENSBTAG00000012540	-4.06E-01	8.49E-01	-2.54E+00	2.84E-10	-4.97E+00	7.67E-30	-4.80E+00	2.89E-28	-4.61E-02	1.00E+00
ENSBTAG00000030847	2.81E-01	1.00E+00	-7.25E-01	6.38E-01	-2.15E+00	6.36E-02	-7.65E-01	5.78E-01	5.17E-01	1.00E+00
ENSBTAG00000036016	1.11E-02	1.00E+00	-2.04E-01	7.83E-01	6.96E-01	3.74E-02	9.75E-01	2.27E-03	-4.71E-02	1.00E+00

ENSBTAG00000002450	3.75E-01	1.00E+00	1.52E+00	6.18E-03	2.07E+00	3.77E-05	1.38E+00	1.52E-02	1.13E+00	3.25E-01
ENSBTAG00000004046	2.37E-03	1.00E+00	4.92E-01	4.35E-01	8.93E-01	5.38E-02	-5.02E-02	1.00E+00	5.73E-01	7.17E-01
ENSBTAG00000030845	1.68E-01	1.00E+00	-1.24E-01	1.00E+00	-5.12E-02	1.00E+00	-4.29E-01	3.80E-01	-3.44E-01	8.50E-01
ENSBTAG00000039760	0.00E+00	1.00E+00	0.00E+00	1.00E+00	-5.13E-15	1.00E+00	1.77E+00	4.97E-01	0.00E+00	1.00E+00
ENSBTAG00000005255	0.00E+00	1.00E+00	0.00E+00	1.00E+00	-5.13E-15	1.00E+00	0.00E+00	1.00E+00	0.00E+00	1.00E+00
ENSBTAG00000046811	0.00E+00	1.00E+00	0.00E+00	1.00E+00	-5.13E-15	1.00E+00	0.00E+00	1.00E+00	0.00E+00	1.00E+00
ENSBTAG00000040023	0.00E+00	1.00E+00	0.00E+00	1.00E+00	-5.13E-15	1.00E+00	0.00E+00	1.00E+00	0.00E+00	1.00E+00
ENSBTAG00000037538	0.00E+00	1.00E+00	0.00E+00	1.00E+00	-5.13E-15	1.00E+00	0.00E+00	1.00E+00	0.00E+00	1.00E+00
ENSBTAG00000046973	0.00E+00	1.00E+00	0.00E+00	1.00E+00	-5.13E-15	1.00E+00	0.00E+00	1.00E+00	0.00E+00	1.00E+00
ENSBTAG00000027490	-1.91E+00	8.23E-01	-1.91E+00	4.69E-01	-1.91E+00	4.28E-01	-1.91E+00	4.39E-01	-1.91E+00	8.00E-01
ENSBTAG00000027489	0.00E+00	1.00E+00	0.00E+00	1.00E+00	-5.13E-15	1.00E+00	0.00E+00	1.00E+00	1.91E+00	8.00E-01
ENSBTAG00000001432	-6.52E-02	1.00E+00	-2.28E-01	5.89E-01	-4.07E-01	1.97E-01	-2.24E-01	6.01E-01	-2.53E-01	8.53E-01
ENSBTAG00000033519	0.00E+00	1.00E+00	1.98E+00	4.43E-01	-5.13E-15	1.00E+00	0.00E+00	1.00E+00	0.00E+00	1.00E+00
ENSBTAG00000002679	0.00E+00	1.00E+00	0.00E+00	1.00E+00	-5.13E-15	1.00E+00	0.00E+00	1.00E+00	0.00E+00	1.00E+00
ENSBTAG00000002071	1.92E+00	8.23E-01	0.00E+00	1.00E+00	-5.13E-15	1.00E+00	0.00E+00	1.00E+00	0.00E+00	1.00E+00
ENSBTAG00000018259	0.00E+00	1.00E+00	0.00E+00	1.00E+00	-5.13E-15	1.00E+00	0.00E+00	1.00E+00	0.00E+00	1.00E+00
ENSBTAG00000033509	0.00E+00	1.00E+00	2.79E+00	2.10E-01	-5.13E-15	1.00E+00	1.76E+00	4.97E-01	0.00E+00	1.00E+00
ENSBTAG00000033506	-1.91E+00	8.23E-01	-1.91E+00	4.69E-01	-1.91E+00	4.28E-01	-1.91E+00	4.39E-01	3.84E-04	1.00E+00
ENSBTAG00000020332	3.57E-01	6.65E-01	1.73E+00	1.03E-11	1.31E+00	2.69E-07	1.12E+00	1.75E-05	4.52E-01	4.76E-01
ENSBTAG00000005913	2.18E-01	8.75E-01	9.53E-01	1.34E-05	7.39E-01	9.72E-04	3.52E-01	1.97E-01	2.77E-01	7.66E-01
ENSBTAG00000047472	0.00E+00	1.00E+00	0.00E+00	1.00E+00	-5.13E-15	1.00E+00	0.00E+00	1.00E+00	0.00E+00	1.00E+00
ENSBTAG00000048032	-1.91E+00	8.23E-01	-1.91E+00	4.69E-01	-1.91E+00	4.28E-01	-1.48E-01	1.00E+00	-1.91E+00	8.00E-01
ENSBTAG00000012820	-2.98E-01	8.62E-01	-9.48E-01	2.89E-03	-5.75E-01	8.81E-02	-6.57E-01	4.91E-02	2.82E-02	1.00E+00
ENSBTAG00000021188	-9.90E-02	1.00E+00	6.27E-01	4.72E-02	9.50E-01	1.18E-03	9.72E-01	1.08E-03	-4.88E-02	1.00E+00
ENSBTAG00000012057	-2.34E-01	1.00E+00	-4.80E-01	1.00E+00	-6.59E-01	1.00E+00	-1.60E+00	6.37E-01	-4.98E-01	1.00E+00
ENSBTAG00000020580	1.50E+00	2.91E-01	2.53E+00	1.29E-03	3.00E+00	1.19E-04	3.60E+00	5.94E-06	1.05E+00	6.64E-01
ENSBTAG00000027348	5.03E-01	1.00E+00	4.32E-01	1.00E+00	5.49E-01	9.88E-01	-1.02E+00	6.95E-01	2.51E-01	1.00E+00
ENSBTAG00000020095	1.13E+00	6.97E-01	5.43E+00	2.44E-13	6.01E+00	6.64E-16	6.42E+00	1.51E-17	1.61E+00	3.77E-01
ENSBTAG00000020096	-2.71E+00	5.76E-01	5.93E-01	9.50E-01	1.00E+00	6.05E-01	1.25E+00	4.52E-01	-1.46E-03	1.00E+00
ENSBTAG00000038650	-6.11E-01	5.63E-01	-1.63E+00	7.44E-05	2.86E-01	6.92E-01	2.12E-01	8.72E-01	1.16E-01	1.00E+00
ENSBTAG00000004581	-1.51E-02	1.00E+00	-6.03E-01	7.33E-01	-6.79E-01	6.27E-01	-8.89E-01	4.83E-01	-1.22E-01	1.00E+00
ENSBTAG00000004583	-1.48E+00	4.67E-01	-6.43E-01	6.46E-01	1.44E-01	1.00E+00	-7.56E-01	5.17E-01	5.10E-01	1.00E+00
ENSBTAG00000004580	3.19E-01	9.95E-01	-5.03E-01	3.56E-01	-4.22E-01	4.61E-01	-4.14E-01	4.89E-01	7.79E-02	1.00E+00
ENSBTAG00000030898	-2.19E+00	5.76E-01	-2.79E-01	1.00E+00	-1.02E+00	6.73E-01	-6.24E-01	9.38E-01	-3.98E-01	1.00E+00
ENSBTAG00000002260	-1.47E-01	1.00E+00	-6.46E-01	1.78E-02	-9.39E-01	2.66E-04	-1.37E+00	7.84E-08	4.04E-01	5.62E-01

ENSBTAG00000046088	0.00E+00	1.00E+00	0.00E+00	1.00E+00	-5.13E-15	1.00E+00	0.00E+00	1.00E+00	0.00E+00	1.00E+00
ENSBTAG00000014838	-6.14E-02	1.00E+00	4.62E-02	1.00E+00	8.76E-03	1.00E+00	4.60E-02	1.00E+00	1.57E-01	1.00E+00
ENSBTAG00000012936	-2.00E-01	8.66E-01	-1.06E+00	7.74E-08	-1.23E+00	2.30E-10	-1.25E+00	1.77E-10	-1.12E-01	1.00E+00
ENSBTAG00000012937	-2.99E-01	4.66E-01	-1.18E+00	9.27E-12	-1.93E+00	5.81E-29	-2.13E+00	2.54E-34	-2.05E-01	8.00E-01
ENSBTAG00000012229	-6.61E-01	1.00E+00	-1.90E+00	2.53E-01	-1.64E-01	1.00E+00	-1.35E+00	4.12E-01	-1.18E+00	8.33E-01
ENSBTAG00000038756	-1.60E-01	1.00E+00	-5.03E-01	6.00E-01	-5.11E-01	5.77E-01	-8.06E-01	2.90E-01	6.21E-01	8.00E-01
ENSBTAG00000018390	4.14E-01	8.76E-01	1.56E-02	1.00E+00	-2.77E-01	7.73E-01	-4.69E-01	4.50E-01	3.49E-01	9.47E-01
ENSBTAG00000027477	1.43E+00	1.33E-01	5.04E-01	6.96E-01	6.91E-01	4.45E-01	5.99E-01	5.54E-01	9.17E-01	6.25E-01
ENSBTAG00000048316	-7.81E-01	1.00E+00	9.13E-02	1.00E+00	-2.70E+00	1.99E-01	9.94E-01	6.17E-01	-7.85E-01	1.00E+00
ENSBTAG00000035659	0.00E+00	1.00E+00	1.98E+00	4.43E-01	1.78E+00	4.79E-01	0.00E+00	1.00E+00	0.00E+00	1.00E+00
ENSBTAG00000015517	0.00E+00	1.00E+00	0.00E+00	1.00E+00	-5.13E-15	1.00E+00	0.00E+00	1.00E+00	0.00E+00	1.00E+00
ENSBTAG00000039944	0.00E+00	1.00E+00	0.00E+00	1.00E+00	-5.13E-15	1.00E+00	0.00E+00	1.00E+00	0.00E+00	1.00E+00
ENSBTAG00000047603	0.00E+00	1.00E+00	0.00E+00	1.00E+00	-5.13E-15	1.00E+00	0.00E+00	1.00E+00	0.00E+00	1.00E+00
ENSBTAG00000046445	0.00E+00	1.00E+00	0.00E+00	1.00E+00	-5.13E-15	1.00E+00	0.00E+00	1.00E+00	0.00E+00	1.00E+00
ENSBTAG00000002874	3.11E-01	6.91E-01	1.63E+00	3.56E-13	1.57E+00	2.24E-12	1.20E+00	1.51E-07	-2.35E-02	1.00E+00
ENSBTAG00000015885	-2.95E-01	8.98E-01	-1.68E+00	2.51E-07	-1.52E+00	1.69E-06	-1.43E+00	8.18E-06	2.66E-01	9.17E-01
ENSBTAG00000006030	2.24E+00	4.19E-01	6.85E-02	1.00E+00	6.44E-01	9.79E-01	2.23E+00	1.34E-01	1.28E-02	1.00E+00
ENSBTAG00000044402	0.00E+00	1.00E+00	0.00E+00	1.00E+00	-5.13E-15	1.00E+00	0.00E+00	1.00E+00	0.00E+00	1.00E+00
ENSBTAG00000011461	3.07E-01	1.00E+00	8.57E-01	5.77E-01	5.91E-01	8.08E-01	-5.53E-01	9.24E-01	5.47E-01	1.00E+00
ENSBTAG00000011465	1.93E+00	4.02E-03	3.15E+00	3.17E-11	2.36E+00	3.69E-06	2.27E+00	1.45E-05	2.66E+00	4.72E-06
ENSBTAG00000018223	1.40E+00	4.03E-02	2.62E+00	1.34E-08	2.83E+00	3.94E-10	2.84E+00	4.46E-10	3.25E+00	2.57E-11
ENSBTAG00000015711	3.48E+00	1.72E-57	3.15E+00	4.62E-49	2.72E+00	3.74E-38	2.91E+00	5.02E-43	2.50E-01	8.00E-01
ENSBTAG00000014912	-5.93E-02	1.00E+00	-9.58E-01	6.49E-03	-8.80E-01	1.29E-02	3.41E-01	4.91E-01	-1.65E-01	1.00E+00
ENSBTAG00000017834	-3.40E-01	1.00E+00	-7.80E-01	1.16E-01	-1.84E+00	2.78E-05	-1.61E+00	3.24E-04	-5.77E-01	6.93E-01
ENSBTAG00000013764	7.43E-01	1.00E+00	1.02E+00	4.45E-01	-5.45E-01	9.28E-01	-1.98E-01	1.00E+00	7.45E-01	9.91E-01
ENSBTAG00000043823	0.00E+00	1.00E+00	0.00E+00	1.00E+00	-5.13E-15	1.00E+00	0.00E+00	1.00E+00	0.00E+00	1.00E+00
ENSBTAG00000014059	3.00E-01	1.00E+00	1.06E+00	3.12E-02	-7.41E-02	1.00E+00	6.55E-01	2.69E-01	2.05E-01	1.00E+00
ENSBTAG00000012349	1.87E-01	1.00E+00	5.04E-01	3.99E-01	-8.04E-03	1.00E+00	-3.02E-01	7.56E-01	-2.74E-01	1.00E+00
ENSBTAG00000045702	8.46E-01	2.59E-01	1.93E+00	3.70E-06	-1.73E-01	9.84E-01	3.04E-01	7.13E-01	5.56E-01	7.02E-01
ENSBTAG00000017352	2.98E-01	7.47E-01	1.06E+00	4.31E-06	3.44E-01	2.35E-01	-1.29E-01	8.71E-01	1.57E-01	1.00E+00
ENSBTAG00000042785	0.00E+00	1.00E+00	0.00E+00	1.00E+00	-5.13E-15	1.00E+00	0.00E+00	1.00E+00	0.00E+00	1.00E+00
ENSBTAG00000008132	-3.84E-01	5.13E-01	-5.97E-01	2.01E-02	-7.43E-01	2.45E-03	-7.45E-01	2.87E-03	2.17E-02	1.00E+00
ENSBTAG00000018509	-2.28E-01	9.33E-01	-8.15E-01	1.62E-03	-1.72E+00	2.99E-12	-2.06E+00	1.24E-16	4.07E-01	5.50E-01
ENSBTAG00000021333	5.44E-01	6.59E-01	-2.32E+00	7.50E-08	-4.21E+00	2.87E-18	-4.14E+00	8.93E-18	1.07E+00	9.47E-02
ENSBTAG00000021200	-8.29E-01	8.04E-01	-1.06E+00	2.45E-01	-2.62E+00	3.20E-03	-1.99E+00	1.98E-02	-3.73E-01	1.00E+00

ENSBTAG00000010177	-1.25E-01	1.00E+00	-1.12E+00	1.88E-03	-1.43E+00	4.80E-05	-6.22E-01	1.33E-01	2.08E-01	1.00E+00
ENSBTAG00000004641	-5.97E-02	1.00E+00	-1.42E+00	2.04E-06	-2.47E+00	1.41E-16	-2.55E+00	2.83E-17	4.37E-02	1.00E+00
ENSBTAG00000013074	1.88E-01	1.00E+00	1.75E+00	1.90E-13	1.24E+00	2.18E-07	7.40E-01	3.85E-03	4.30E-01	4.64E-01
ENSBTAG00000024633	-5.54E-01	2.23E-01	-3.23E-01	3.55E-01	-8.31E-02	1.00E+00	-3.33E-01	3.35E-01	-7.65E-02	1.00E+00
ENSBTAG00000042752	0.00E+00	1.00E+00	0.00E+00	1.00E+00	1.78E+00	4.79E-01	0.00E+00	1.00E+00	0.00E+00	1.00E+00
ENSBTAG00000006255	6.77E-01	2.62E-01	1.80E+00	5.14E-08	9.98E-01	3.78E-03	-4.50E-02	1.00E+00	-7.92E-01	2.01E-01
ENSBTAG00000026068	-9.12E-01	8.90E-01	-4.51E+00	6.24E-03	-1.96E+00	1.10E-01	-1.12E+00	3.99E-01	-1.30E+00	7.31E-01
ENSBTAG00000034611	-8.51E-02	1.00E+00	-1.16E+00	2.31E-01	-1.66E+00	6.20E-02	-1.99E+00	2.97E-02	-1.48E+00	4.34E-01
ENSBTAG00000009111	-3.86E-01	9.27E-01	-1.67E+00	2.28E-04	-8.34E-01	8.13E-02	-1.00E+00	3.29E-02	-4.11E-01	8.49E-01
ENSBTAG00000044886	0.00E+00	1.00E+00	0.00E+00	1.00E+00	-5.13E-15	1.00E+00	0.00E+00	1.00E+00	0.00E+00	1.00E+00
ENSBTAG00000045895	4.23E-01	4.76E-01	-1.19E-01	9.45E-01	-7.08E-01	8.90E-03	-6.90E-01	1.28E-02	7.11E-01	7.68E-02
ENSBTAG00000014645	9.44E-01	9.37E-01	1.02E+00	5.14E-01	-1.47E-01	1.00E+00	-6.78E-01	8.87E-01	3.81E-01	1.00E+00
ENSBTAG00000015945	-3.61E-01	9.32E-01	-5.32E-02	1.00E+00	1.67E-01	9.64E-01	-1.82E-01	9.41E-01	1.03E-02	1.00E+00
ENSBTAG00000015946	-6.46E-02	1.00E+00	6.58E-01	6.40E-03	8.24E-01	4.02E-04	5.93E-01	1.79E-02	1.02E-01	1.00E+00
ENSBTAG00000016618	-4.39E-02	1.00E+00	-3.83E-01	1.50E-01	2.05E-01	5.42E-01	-6.03E-02	1.00E+00	-1.79E-01	9.69E-01
ENSBTAG00000002361	-1.48E+00	1.40E-01	-5.91E-01	4.69E-01	-9.50E-01	1.60E-01	-1.30E+00	5.14E-02	-2.97E-01	1.00E+00
ENSBTAG00000018774	2.93E+00	3.60E-24	2.70E+00	9.20E-22	2.22E+00	1.34E-15	1.93E+00	5.87E-12	3.54E-01	7.76E-01
ENSBTAG00000047727	0.00E+00	1.00E+00	0.00E+00	1.00E+00	-5.13E-15	1.00E+00	0.00E+00	1.00E+00	0.00E+00	1.00E+00
ENSBTAG00000015197	-1.43E+00	7.57E-01	-1.36E+00	3.90E-01	-2.36E+00	9.77E-02	-7.45E-01	6.95E-01	-2.43E-01	1.00E+00
ENSBTAG00000034632	-1.91E+00	8.23E-01	7.64E-02	1.00E+00	-1.24E-01	1.00E+00	-1.41E-01	1.00E+00	-1.91E+00	8.00E-01
ENSBTAG00000029815	0.00E+00	1.00E+00	0.00E+00	1.00E+00	-5.13E-15	1.00E+00	0.00E+00	1.00E+00	0.00E+00	1.00E+00
ENSBTAG00000012673	-2.05E-01	1.00E+00	-1.67E+00	4.79E-06	-3.43E+00	2.67E-19	-2.47E+00	2.24E-11	2.96E-01	9.49E-01
ENSBTAG00000004952	7.07E-02	1.00E+00	7.20E-01	4.33E-01	1.75E-01	1.00E+00	-6.31E-01	5.45E-01	3.32E-02	1.00E+00
ENSBTAG00000004953	2.95E-02	1.00E+00	1.90E+00	8.87E-09	1.65E+00	6.00E-07	1.58E+00	2.37E-06	4.65E-02	1.00E+00
ENSBTAG00000007693	2.70E+00	1.30E-02	4.39E+00	9.24E-07	3.04E+00	4.74E-04	1.10E+00	3.10E-01	3.65E-01	1.00E+00
ENSBTAG00000008001	-6.85E-02	1.00E+00	-5.42E-01	1.17E-02	-9.00E-01	8.03E-06	-1.02E+00	5.18E-07	3.65E-02	1.00E+00
ENSBTAG00000003232	9.91E-02	1.00E+00	5.15E-01	1.24E-01	1.05E+00	2.53E-04	8.48E-01	4.90E-03	-2.10E-01	1.00E+00
ENSBTAG00000005379	1.20E+00	2.51E-02	2.82E+00	2.26E-13	2.67E+00	2.94E-12	2.43E+00	3.27E-10	1.28E+00	2.80E-02
ENSBTAG00000008734	7.52E-02	1.00E+00	-4.52E-01	1.45E-01	-9.14E-01	6.50E-04	-9.83E-01	2.78E-04	4.76E-01	4.38E-01
ENSBTAG00000001382	-3.51E-01	8.53E-01	-1.07E+00	4.52E-03	-9.41E-01	1.11E-02	-2.13E+00	1.53E-08	5.63E-01	5.02E-01
ENSBTAG00000012774	2.72E-01	1.00E+00	1.38E-01	1.00E+00	9.03E-01	1.18E-01	1.02E+00	7.19E-02	-6.26E-02	1.00E+00
ENSBTAG00000008513	-2.47E-01	1.00E+00	-8.57E-01	1.12E-01	5.27E-01	3.07E-01	2.00E-01	9.27E-01	1.86E-02	1.00E+00
ENSBTAG00000017301	5.07E-03	1.00E+00	6.95E-02	1.00E+00	1.15E+00	6.45E-01	-1.91E+00	4.39E-01	-1.91E+00	8.00E-01
ENSBTAG00000020227	9.41E-03	1.00E+00	-2.28E+00	1.19E-02	-6.70E-01	4.79E-01	-9.48E-01	2.73E-01	-7.31E-01	8.00E-01
ENSBTAG00000001483	1.73E-01	1.00E+00	-1.03E+00	5.45E-04	-1.86E+00	1.23E-10	-1.58E+00	5.69E-08	3.77E-01	7.26E-01

ENSBTAG00000010408	8.91E-01	1.65E-02	3.29E-01	4.24E-01	-6.00E-01	7.12E-02	-8.44E-01	7.97E-03	-3.08E-02	1.00E+00
ENSBTAG00000010427	9.00E-01	4.87E-02	-6.00E-02	1.00E+00	-1.75E+00	8.40E-08	-1.01E+00	3.37E-03	-1.03E-01	1.00E+00
ENSBTAG00000010432	9.75E-02	1.00E+00	4.42E-01	1.25E-01	9.38E-01	1.53E-04	4.54E-01	1.20E-01	-1.78E-01	1.00E+00
ENSBTAG00000013294	3.05E+00	2.35E-08	2.36E+00	3.75E-06	2.75E+00	5.67E-08	2.27E+00	1.03E-05	1.37E-01	1.00E+00
ENSBTAG00000038107	1.38E-01	1.00E+00	-2.91E-01	4.02E-01	-2.83E-01	4.18E-01	2.51E-01	4.97E-01	1.57E-01	1.00E+00
ENSBTAG00000006685	2.98E+00	7.19E-12	3.47E+00	8.00E-17	9.17E-01	6.27E-02	3.90E-01	6.07E-01	-1.11E+00	2.85E-01
ENSBTAG00000006692	1.91E+00	8.23E-01	0.00E+00	1.00E+00	-5.13E-15	1.00E+00	0.00E+00	1.00E+00	1.93E+00	8.00E-01
ENSBTAG00000002757	0.00E+00	1.00E+00	0.00E+00	1.00E+00	-5.13E-15	1.00E+00	0.00E+00	1.00E+00	0.00E+00	1.00E+00
ENSBTAG00000019798	4.34E-01	6.22E-01	-1.67E-01	8.73E-01	-4.51E-01	2.32E-01	-2.28E-01	7.00E-01	8.27E-01	9.53E-02
ENSBTAG00000019799	1.52E+00	4.27E-05	2.49E+00	2.97E-15	1.52E+00	4.14E-06	6.79E-01	9.34E-02	-2.33E-01	1.00E+00
ENSBTAG00000019796	-6.33E-01	7.73E-01	-1.76E+00	1.20E-03	-2.25E+00	2.67E-05	-1.40E+00	1.29E-02	-8.79E-01	5.00E-01
ENSBTAG00000007851	-6.02E-01	6.88E-01	-1.17E+00	2.35E-02	-1.58E+00	1.46E-03	-2.57E+00	2.64E-06	-1.52E+00	4.41E-02
ENSBTAG00000046946	0.00E+00	1.00E+00	0.00E+00	1.00E+00	-5.13E-15	1.00E+00	0.00E+00	1.00E+00	0.00E+00	1.00E+00
ENSBTAG00000018143	-3.71E-01	1.00E+00	-1.46E+00	2.92E-02	-2.22E+00	8.38E-04	-1.50E+00	2.07E-02	-2.75E-01	1.00E+00
ENSBTAG00000020273	4.38E-01	9.28E-01	1.94E+00	3.54E-05	1.42E+00	3.24E-03	2.00E+00	2.29E-05	5.56E-01	8.00E-01
ENSBTAG00000002126	-1.09E+00	7.35E-06	-4.12E+00	5.97E-66	-3.37E+00	1.24E-48	-1.94E+00	1.17E-18	-8.08E-01	8.00E-03
ENSBTAG00000017714	4.30E-01	2.00E-01	-1.72E-01	5.92E-01	-1.10E+00	1.18E-08	-1.87E+00	3.24E-22	-6.59E-03	1.00E+00
ENSBTAG00000009876	9.42E-01	1.93E-05	3.76E-01	1.03E-01	-2.17E-01	4.48E-01	-7.44E-01	3.05E-04	5.94E-01	5.57E-02
ENSBTAG00000039196	5.31E-01	3.39E-01	-4.56E-01	1.77E-01	-1.21E+00	1.74E-05	-1.70E+00	1.26E-09	-5.34E-03	1.00E+00
ENSBTAG00000047621	1.08E+00	5.78E-06	1.02E+00	2.89E-06	5.91E-01	1.09E-02	3.85E-01	1.45E-01	7.86E-01	9.18E-03
ENSBTAG00000006984	1.31E+00	2.04E-03	2.29E+00	4.34E-11	2.38E+00	5.28E-12	2.31E+00	3.35E-11	2.37E-01	1.00E+00
ENSBTAG00000045783	-5.78E-02	1.00E+00	9.96E-02	1.00E+00	5.78E-03	1.00E+00	1.24E-01	9.16E-01	-4.22E-01	4.99E-01
ENSBTAG00000038496	-7.86E-01	2.72E-01	-1.36E+00	6.83E-04	-1.91E+00	1.12E-06	-2.19E+00	3.34E-08	-8.76E-01	2.48E-01
ENSBTAG00000023177	8.02E-01	7.26E-02	5.81E-01	9.68E-02	-4.09E-02	1.00E+00	-1.45E-01	9.42E-01	1.07E+00	1.15E-02
ENSBTAG00000024647	8.59E-01	9.58E-04	8.20E-01	2.66E-04	5.22E-02	1.00E+00	-5.49E-03	1.00E+00	1.06E+00	6.41E-05
ENSBTAG00000047228	0.00E+00	1.00E+00	0.00E+00	1.00E+00	-5.13E-15	1.00E+00	0.00E+00	1.00E+00	0.00E+00	1.00E+00
ENSBTAG00000040409	0.00E+00	1.00E+00	0.00E+00	1.00E+00	-5.13E-15	1.00E+00	0.00E+00	1.00E+00	0.00E+00	1.00E+00
ENSBTAG00000039995	7.13E-01	4.79E-02	5.74E-01	4.11E-02	1.13E-01	9.67E-01	-1.68E-01	7.83E-01	8.55E-01	1.94E-02
ENSBTAG00000047966	-1.41E-01	1.00E+00	-9.38E-01	9.39E-04	-1.73E+00	2.70E-10	-1.29E+00	3.48E-06	9.95E-02	1.00E+00
ENSBTAG00000016729	1.92E+00	4.26E-03	4.16E+00	5.11E-13	2.62E+00	2.14E-06	1.10E+00	7.61E-02	1.36E+00	1.53E-01
ENSBTAG00000044933	0.00E+00	1.00E+00	0.00E+00	1.00E+00	-5.13E-15	1.00E+00	0.00E+00	1.00E+00	0.00E+00	1.00E+00
ENSBTAG00000025113	0.00E+00	1.00E+00	0.00E+00	1.00E+00	-5.13E-15	1.00E+00	0.00E+00	1.00E+00	0.00E+00	1.00E+00
ENSBTAG00000020953	1.59E-01	1.00E+00	-4.37E-01	5.78E-02	-1.01E+00	7.55E-07	-5.05E-01	2.57E-02	4.93E-02	1.00E+00
ENSBTAG00000044235	0.00E+00	1.00E+00	0.00E+00	1.00E+00	-5.13E-15	1.00E+00	0.00E+00	1.00E+00	0.00E+00	1.00E+00
ENSBTAG00000014862	-1.67E-01	1.00E+00	-5.47E-01	1.58E-02	1.24E-01	8.32E-01	4.38E-01	7.07E-02	-2.51E-01	8.00E-01

ENSBTAG00000043874	0.00E+00	1.00E+00	0.00E+00	1.00E+00	-5.13E-15	1.00E+00	0.00E+00	1.00E+00	0.00E+00	1.00E+00
ENSBTAG00000012765	-1.35E-01	1.00E+00	-3.79E-01	4.69E-01	5.96E-02	1.00E+00	-6.73E-02	1.00E+00	-3.70E-02	1.00E+00
ENSBTAG00000016703	1.16E-01	1.00E+00	9.77E-01	2.11E-05	1.03E+00	5.44E-06	9.86E-01	2.08E-05	1.91E-01	9.54E-01
ENSBTAG00000047703	0.00E+00	1.00E+00	0.00E+00	1.00E+00	-5.13E-15	1.00E+00	0.00E+00	1.00E+00	0.00E+00	1.00E+00
ENSBTAG00000046060	0.00E+00	1.00E+00	0.00E+00	1.00E+00	-5.13E-15	1.00E+00	0.00E+00	1.00E+00	0.00E+00	1.00E+00
ENSBTAG00000019859	0.00E+00	1.00E+00	0.00E+00	1.00E+00	-5.13E-15	1.00E+00	0.00E+00	1.00E+00	0.00E+00	1.00E+00
ENSBTAG00000015972	-9.62E-02	1.00E+00	-3.18E-01	1.58E-01	-2.90E-01	2.11E-01	-1.17E-01	8.12E-01	-5.93E-01	3.43E-02
ENSBTAG00000013812	2.95E-01	8.23E-01	1.04E+00	1.34E-04	6.51E-01	2.69E-02	8.00E-01	5.43E-03	2.81E-01	8.29E-01
ENSBTAG00000013620	1.04E-01	1.00E+00	9.44E-01	3.26E-08	1.06E+00	3.05E-10	6.90E-01	9.66E-05	-2.95E-01	5.22E-01
ENSBTAG00000044446	0.00E+00	1.00E+00	0.00E+00	1.00E+00	-5.13E-15	1.00E+00	0.00E+00	1.00E+00	0.00E+00	1.00E+00
ENSBTAG00000034366	1.36E+00	3.68E-03	1.28E+00	1.20E-03	1.12E+00	5.20E-03	1.34E+00	7.70E-04	-4.70E-01	8.00E-01
ENSBTAG00000013849	2.71E+00	5.76E-01	4.23E+00	1.39E-02	3.42E+00	7.65E-02	4.47E+00	5.99E-03	4.12E+00	1.41E-01
ENSBTAG00000021672	1.89E+00	9.91E-02	2.01E+00	1.28E-02	1.63E+00	5.12E-02	2.51E+00	1.63E-03	-3.04E-01	1.00E+00
ENSBTAG00000037494	1.98E-01	1.00E+00	-1.07E+00	1.20E-02	-1.01E+00	1.57E-02	-1.61E-01	9.72E-01	-4.18E-01	8.00E-01
ENSBTAG00000013064	3.41E-01	1.00E+00	-3.96E-01	8.34E-01	2.62E-01	9.66E-01	9.73E-01	1.36E-01	6.10E-01	8.04E-01
ENSBTAG00000036945	0.00E+00	1.00E+00	0.00E+00	1.00E+00	-5.13E-15	1.00E+00	0.00E+00	1.00E+00	0.00E+00	1.00E+00
ENSBTAG00000044324	0.00E+00	1.00E+00	0.00E+00	1.00E+00	-5.13E-15	1.00E+00	0.00E+00	1.00E+00	0.00E+00	1.00E+00
ENSBTAG00000008420	2.47E-01	1.00E+00	9.71E-01	2.21E-03	1.09E+00	3.90E-04	1.10E+00	3.87E-04	1.93E-01	1.00E+00
ENSBTAG00000045416	0.00E+00	1.00E+00	0.00E+00	1.00E+00	-5.13E-15	1.00E+00	0.00E+00	1.00E+00	0.00E+00	1.00E+00
ENSBTAG00000028022	0.00E+00	1.00E+00	0.00E+00	1.00E+00	-5.13E-15	1.00E+00	0.00E+00	1.00E+00	0.00E+00	1.00E+00
ENSBTAG00000043147	0.00E+00	1.00E+00	0.00E+00	1.00E+00	-5.13E-15	1.00E+00	0.00E+00	1.00E+00	0.00E+00	1.00E+00
ENSBTAG00000045713	0.00E+00	1.00E+00	2.79E+00	2.10E-01	1.78E+00	4.79E-01	1.76E+00	4.97E-01	0.00E+00	1.00E+00
ENSBTAG00000046655	-1.02E-01	1.00E+00	-6.32E-01	3.64E-02	-6.71E-01	2.35E-02	-1.70E-01	8.09E-01	3.62E-01	7.10E-01
ENSBTAG00000043691	0.00E+00	1.00E+00	0.00E+00	1.00E+00	-5.13E-15	1.00E+00	0.00E+00	1.00E+00	0.00E+00	1.00E+00
ENSBTAG00000043370	0.00E+00	1.00E+00	0.00E+00	1.00E+00	-5.13E-15	1.00E+00	0.00E+00	1.00E+00	0.00E+00	1.00E+00
ENSBTAG00000006186	8.93E-03	1.00E+00	5.52E-02	1.00E+00	-1.24E-01	8.82E-01	8.56E-02	1.00E+00	2.06E-01	9.13E-01
ENSBTAG00000006188	-1.51E-01	1.00E+00	9.29E-01	3.37E-01	-6.77E-01	5.53E-01	7.12E-02	1.00E+00	2.65E-02	1.00E+00
ENSBTAG00000007997	0.00E+00	1.00E+00	0.00E+00	1.00E+00	-5.13E-15	1.00E+00	0.00E+00	1.00E+00	0.00E+00	1.00E+00
ENSBTAG00000010392	1.27E-02	1.00E+00	9.79E-01	4.07E-01	-1.00E+00	4.79E-01	1.27E+00	2.05E-01	7.78E-01	8.97E-01
ENSBTAG00000001574	3.72E-01	5.74E-01	1.20E+00	2.42E-07	6.05E-01	1.71E-02	6.79E-01	7.15E-03	-3.40E-01	6.75E-01
ENSBTAG00000009838	3.03E-01	8.23E-01	2.08E+00	7.13E-15	2.02E+00	2.67E-14	1.33E+00	1.17E-06	-3.18E-01	8.00E-01
ENSBTAG00000005359	-2.75E-01	1.00E+00	2.32E-01	9.76E-01	-4.76E-01	5.89E-01	-1.85E-01	1.00E+00	2.34E-01	1.00E+00
ENSBTAG00000045417	0.00E+00	1.00E+00	0.00E+00	1.00E+00	-5.13E-15	1.00E+00	0.00E+00	1.00E+00	0.00E+00	1.00E+00
ENSBTAG00000015534	-5.92E-01	3.69E-01	-3.17E+00	1.16E-19	-1.16E+00	3.80E-04	-5.42E-01	1.60E-01	-3.72E-01	8.00E-01
ENSBTAG00000045048	0.00E+00	1.00E+00	0.00E+00	1.00E+00	-5.13E-15	1.00E+00	0.00E+00	1.00E+00	0.00E+00	1.00E+00

ENSBTAG00000004414	-2.26E+00	3.99E-06	-4.81E+00	1.54E-21	-4.42E+00	9.85E-20	-5.52E-01	3.49E-01	7.13E-01	5.32E-01
ENSBTAG00000006898	2.50E-01	8.23E-01	1.54E+00	2.30E-12	1.56E+00	1.05E-12	1.57E+00	9.70E-13	-7.79E-02	1.00E+00
ENSBTAG00000004064	-2.11E-01	9.52E-01	-3.50E-01	2.47E-01	-4.26E-01	1.30E-01	-4.49E-01	1.14E-01	-1.95E-01	9.65E-01
ENSBTAG00000012582	4.93E-02	1.00E+00	-3.45E-01	1.48E-01	-5.29E-01	1.34E-02	-2.73E-01	3.07E-01	8.91E-02	1.00E+00
ENSBTAG00000030056	0.00E+00	1.00E+00	0.00E+00	1.00E+00	-5.13E-15	1.00E+00	0.00E+00	1.00E+00	0.00E+00	1.00E+00
ENSBTAG00000030053	0.00E+00	1.00E+00	0.00E+00	1.00E+00	-5.13E-15	1.00E+00	0.00E+00	1.00E+00	0.00E+00	1.00E+00
ENSBTAG00000012585	-2.57E-01	7.29E-01	-5.91E-01	4.15E-03	-3.34E-02	1.00E+00	2.95E-01	2.35E-01	1.23E-01	1.00E+00
ENSBTAG00000042602	0.00E+00	1.00E+00	0.00E+00	1.00E+00	-5.13E-15	1.00E+00	0.00E+00	1.00E+00	0.00E+00	1.00E+00
ENSBTAG00000010460	-1.47E-01	1.00E+00	-1.80E+00	1.21E-07	1.39E-03	1.00E+00	5.85E-01	1.21E-01	-4.65E-01	6.40E-01
ENSBTAG00000006403	-1.36E+00	4.19E-05	-2.89E+00	3.81E-21	-3.59E+00	2.27E-30	-2.68E+00	5.90E-19	3.72E-02	1.00E+00
ENSBTAG00000016277	-3.20E-02	1.00E+00	-5.43E-01	6.11E-02	-1.44E+00	1.88E-08	-1.97E+00	1.63E-14	-3.52E-01	6.93E-01
ENSBTAG00000047287	3.80E-01	9.32E-01	-1.02E+00	2.64E-02	-3.30E+00	3.52E-13	-2.87E+00	1.58E-10	8.71E-01	3.12E-01
ENSBTAG00000044740	1.92E+00	8.23E-01	1.98E+00	4.43E-01	-5.13E-15	1.00E+00	0.00E+00	1.00E+00	0.00E+00	1.00E+00
ENSBTAG00000014560	-5.70E-02	1.00E+00	-6.80E-02	1.00E+00	-5.85E-01	1.59E-01	-6.42E-01	1.19E-01	-2.83E-01	9.62E-01
ENSBTAG00000001729	-5.58E-01	6.85E-01	-8.07E-01	8.68E-02	-7.95E-01	9.19E-02	-4.00E-01	5.21E-01	2.78E-02	1.00E+00
ENSBTAG00000042448	0.00E+00	1.00E+00	0.00E+00	1.00E+00	-5.13E-15	1.00E+00	0.00E+00	1.00E+00	0.00E+00	1.00E+00
ENSBTAG00000017561	-1.06E+00	9.82E-02	-5.51E+00	3.92E-21	-6.32E+00	5.47E-25	-4.75E+00	1.33E-19	5.94E-01	6.25E-01
ENSBTAG00000013483	-1.06E-01	1.00E+00	2.97E-01	3.12E-01	-1.96E-01	5.94E-01	-3.61E-01	2.00E-01	1.44E-01	1.00E+00
ENSBTAG00000018824	-4.05E-02	1.00E+00	2.96E-01	1.79E-01	1.85E-01	4.79E-01	1.68E-01	5.63E-01	1.60E-01	9.15E-01
ENSBTAG00000007593	4.23E-02	1.00E+00	-2.04E-01	7.15E-01	-5.58E-01	7.86E-02	-7.53E-01	1.34E-02	1.68E-01	1.00E+00
ENSBTAG00000007595	-4.73E-01	4.66E-01	-6.16E-01	4.83E-02	-1.58E-01	8.49E-01	3.86E-01	2.85E-01	2.78E-01	8.38E-01
ENSBTAG00000044081	1.60E-01	1.00E+00	-3.75E-01	1.50E-01	-1.32E+00	1.20E-09	-1.99E+00	1.90E-19	-2.39E-01	8.00E-01
ENSBTAG00000013463	2.43E-02	1.00E+00	-9.62E-01	8.10E-03	-2.50E-01	6.62E-01	1.50E-01	9.36E-01	-5.64E-02	1.00E+00
ENSBTAG00000000477	2.08E+00	1.64E-01	-1.22E+00	6.19E-01	1.30E+00	3.11E-01	4.85E-01	9.69E-01	1.14E+00	8.00E-01
ENSBTAG00000020989	-2.32E-01	1.00E+00	4.88E-01	5.86E-01	-8.45E-01	2.72E-01	2.70E-02	1.00E+00	-4.44E-02	1.00E+00
ENSBTAG00000006704	-7.81E-01	1.00E+00	-2.70E+00	2.42E-01	-2.70E+00	1.99E-01	-2.70E+00	2.05E-01	-2.70E+00	6.01E-01
ENSBTAG00000003600	-2.56E-01	1.00E+00	-9.63E-02	1.00E+00	-1.54E+00	3.04E-03	-1.47E+00	5.38E-03	1.31E-01	1.00E+00
ENSBTAG00000012778	5.32E-02	1.00E+00	1.35E+00	2.11E-03	1.83E+00	1.94E-05	1.52E+00	5.49E-04	-4.19E-01	8.44E-01
ENSBTAG00000012781	6.99E-01	2.21E-01	1.54E+00	2.56E-06	1.02E+00	2.55E-03	-1.71E-01	8.99E-01	1.70E-01	1.00E+00
ENSBTAG00000019538	3.24E-01	8.59E-01	1.37E+00	1.54E-05	1.54E+00	8.82E-07	7.86E-01	2.37E-02	1.45E-01	1.00E+00
ENSBTAG00000022942	-6.19E-04	1.00E+00	-1.91E+00	4.69E-01	-1.91E+00	4.28E-01	-1.53E-01	1.00E+00	-1.91E+00	8.00E-01
ENSBTAG00000012705	-9.35E-02	1.00E+00	7.61E-02	1.00E+00	2.72E-01	4.79E-01	-7.93E-02	1.00E+00	-2.85E-01	8.00E-01
ENSBTAG00000006718	1.27E-01	1.00E+00	8.97E-01	5.51E-05	8.25E-01	2.19E-04	4.27E-01	9.96E-02	-3.65E-03	1.00E+00
ENSBTAG00000019471	2.76E-01	6.62E-01	3.54E-01	1.29E-01	5.20E-01	1.38E-02	2.95E-01	2.46E-01	9.68E-02	1.00E+00
ENSBTAG00000011785	-8.66E-04	1.00E+00	9.04E-01	2.72E-03	1.67E+00	5.95E-09	8.88E-01	3.92E-03	1.32E-01	1.00E+00

ENSBTAG00000044171	2.69E-01	1.00E+00	-6.99E-01	6.02E-01	-1.41E-01	1.00E+00	5.78E-01	7.06E-01	7.31E-02	1.00E+00
ENSBTAG00000008449	-2.35E-01	1.00E+00	4.84E-01	8.63E-01	2.22E-01	1.00E+00	4.96E-01	8.30E-01	-9.00E-04	1.00E+00
ENSBTAG00000008453	-4.00E-01	4.15E-01	-1.92E+00	6.17E-16	-9.74E-01	2.08E-05	-3.66E-01	1.89E-01	6.60E-02	1.00E+00
ENSBTAG00000016185	7.44E-01	3.05E-01	8.92E-01	2.91E-02	7.40E-01	8.20E-02	1.05E+00	7.32E-03	4.41E-01	8.00E-01
ENSBTAG00000033322	-3.70E-02	1.00E+00	9.22E-01	2.74E-04	1.17E+00	2.29E-06	8.12E-01	1.88E-03	-1.66E-01	1.00E+00
ENSBTAG00000000140	-3.64E-01	6.37E-01	-7.25E-01	7.43E-03	-1.59E+00	3.71E-10	-2.15E+00	3.80E-17	-4.67E-01	4.39E-01
ENSBTAG00000002854	3.84E-02	1.00E+00	-1.20E+00	1.12E-07	-1.44E+00	1.35E-10	-8.12E-01	5.72E-04	2.85E-01	7.69E-01
ENSBTAG00000002855	-5.82E-01	1.00E+00	-2.86E+00	7.31E-03	-3.88E+00	5.16E-04	-2.64E+00	8.24E-03	-5.53E-01	1.00E+00
ENSBTAG00000005835	-3.47E-01	5.00E-01	-7.08E-01	1.27E-03	3.50E-01	1.59E-01	5.64E-01	1.14E-02	-1.75E-01	9.51E-01
ENSBTAG00000037896	-5.22E-01	4.55E-01	-1.57E+00	1.29E-06	-3.11E+00	9.23E-19	-4.20E+00	1.38E-26	1.10E-01	1.00E+00
ENSBTAG00000012787	1.35E-01	1.00E+00	2.41E-01	4.57E-01	6.68E-01	3.34E-03	5.08E-01	3.92E-02	-2.99E-01	7.35E-01
ENSBTAG00000024561	-8.98E-02	1.00E+00	-5.33E-01	2.35E-02	-8.24E-01	1.78E-04	-9.79E-01	8.09E-06	-4.57E-01	3.10E-01
ENSBTAG00000013128	3.04E-01	6.18E-01	1.52E+00	1.15E-13	1.37E+00	2.32E-11	1.31E+00	2.73E-10	-7.62E-02	1.00E+00
ENSBTAG00000002139	-3.23E+00	3.75E-01	-4.36E-01	1.00E+00	-1.83E-01	1.00E+00	-1.46E+00	4.93E-01	-1.31E+00	8.99E-01
ENSBTAG00000033267	1.43E-01	1.00E+00	-4.31E-01	5.02E-01	5.61E-01	2.72E-01	2.54E-01	8.11E-01	1.83E-01	1.00E+00
ENSBTAG00000000837	-1.36E-02	1.00E+00	-8.90E-01	4.28E-04	-7.19E-01	5.40E-03	-2.02E-01	6.47E-01	2.60E-01	8.04E-01
ENSBTAG00000047233	0.00E+00	1.00E+00	0.00E+00	1.00E+00	-5.13E-15	1.00E+00	0.00E+00	1.00E+00	0.00E+00	1.00E+00
ENSBTAG00000024560	5.95E-01	9.62E-01	3.07E-01	9.80E-01	-2.80E-01	1.00E+00	3.62E-01	8.97E-01	-2.83E-01	1.00E+00
ENSBTAG00000033252	1.60E-01	1.00E+00	6.46E-01	6.02E-02	4.54E-01	2.37E-01	5.95E-01	9.62E-02	1.10E-01	1.00E+00
ENSBTAG00000019339	1.06E+00	2.06E-02	1.38E+00	6.92E-05	4.06E-01	4.01E-01	-1.47E-01	9.92E-01	1.64E+00	6.41E-05
ENSBTAG00000013197	8.23E-02	1.00E+00	2.86E-01	3.02E-01	4.51E-01	5.65E-02	-1.78E-01	6.31E-01	-1.81E-01	9.37E-01
ENSBTAG00000021880	-4.31E-01	4.71E-01	-1.55E+00	1.22E-09	-2.21E+00	6.09E-18	-1.86E+00	3.56E-13	-7.65E-02	1.00E+00
ENSBTAG00000022777	1.37E-01	1.00E+00	-1.52E-01	7.59E-01	-7.11E-01	2.09E-03	-6.45E-01	6.86E-03	3.11E-01	6.61E-01
ENSBTAG00000047824	4.63E-02	1.00E+00	6.86E-01	1.28E-03	6.85E-01	1.26E-03	6.02E-01	6.43E-03	-1.11E-01	1.00E+00
ENSBTAG00000036628	0.00E+00	1.00E+00	0.00E+00	1.00E+00	-5.13E-15	1.00E+00	0.00E+00	1.00E+00	0.00E+00	1.00E+00
ENSBTAG00000013949	6.47E-02	1.00E+00	6.36E-01	9.51E-04	5.07E-01	1.08E-02	2.69E-01	2.71E-01	-2.06E-01	8.00E-01
ENSBTAG00000044816	0.00E+00	1.00E+00	0.00E+00	1.00E+00	-5.13E-15	1.00E+00	0.00E+00	1.00E+00	0.00E+00	1.00E+00
ENSBTAG00000003804	9.24E-02	1.00E+00	-2.40E-01	4.95E-01	-8.60E-01	4.18E-04	-7.73E-01	2.11E-03	-1.14E-02	1.00E+00
ENSBTAG00000011508	2.70E+00	5.76E-01	1.98E+00	4.43E-01	1.78E+00	4.79E-01	2.53E+00	2.71E-01	2.71E+00	6.01E-01
ENSBTAG00000021109	0.00E+00	1.00E+00	0.00E+00	1.00E+00	3.06E+00	1.36E-01	0.00E+00	1.00E+00	0.00E+00	1.00E+00
ENSBTAG00000000511	3.00E-01	8.23E-01	7.49E-01	7.55E-03	5.83E-01	4.70E-02	8.42E-02	1.00E+00	1.98E-01	1.00E+00
ENSBTAG00000000072	3.90E-01	3.44E-01	2.49E+00	3.06E-36	2.28E+00	3.97E-31	1.31E+00	4.14E-11	-2.46E-01	8.00E-01
ENSBTAG00000033180	3.57E-02	1.00E+00	-6.15E-01	4.12E-02	-4.59E-01	1.56E-01	-2.59E-01	5.36E-01	-1.55E-01	1.00E+00
ENSBTAG00000042363	0.00E+00	1.00E+00	0.00E+00	1.00E+00	-5.13E-15	1.00E+00	0.00E+00	1.00E+00	0.00E+00	1.00E+00
ENSBTAG00000043288	0.00E+00	1.00E+00	0.00E+00	1.00E+00	-5.13E-15	1.00E+00	0.00E+00	1.00E+00	0.00E+00	1.00E+00

ENSBTAG00000012715	1.25E+00	3.09E-01	1.35E+00	5.73E-02	4.92E-01	7.18E-01	9.72E-01	2.34E-01	3.86E-01	1.00E+00
ENSBTAG00000045161	0.00E+00	1.00E+00	0.00E+00	1.00E+00	-5.13E-15	1.00E+00	0.00E+00	1.00E+00	0.00E+00	1.00E+00
ENSBTAG00000024555	9.23E-02	1.00E+00	1.59E+00	8.19E-08	7.08E-02	1.00E+00	-5.36E-01	1.53E-01	-1.32E-01	1.00E+00
ENSBTAG00000047437	0.00E+00	1.00E+00	0.00E+00	1.00E+00	-5.13E-15	1.00E+00	0.00E+00	1.00E+00	0.00E+00	1.00E+00
ENSBTAG00000043425	0.00E+00	1.00E+00	0.00E+00	1.00E+00	-5.13E-15	1.00E+00	0.00E+00	1.00E+00	0.00E+00	1.00E+00
ENSBTAG00000043181	0.00E+00	1.00E+00	0.00E+00	1.00E+00	-5.13E-15	1.00E+00	0.00E+00	1.00E+00	0.00E+00	1.00E+00
ENSBTAG00000007606	2.97E-01	6.30E-01	1.78E+00	2.89E-18	1.44E+00	1.24E-12	1.12E+00	6.23E-08	-2.11E-01	8.33E-01
ENSBTAG00000033136	2.02E-01	8.78E-01	1.16E+00	5.66E-09	-2.67E-01	3.19E-01	-1.02E+00	5.39E-07	-4.95E-01	1.83E-01
ENSBTAG00000001478	1.34E+00	1.47E-03	2.20E+00	2.75E-10	1.45E+00	4.51E-05	9.59E-01	1.23E-02	8.59E-02	1.00E+00
ENSBTAG00000044066	1.65E-01	1.00E+00	-1.12E+00	1.11E-01	-8.94E-01	1.97E-01	-2.81E-01	8.90E-01	4.33E-02	1.00E+00
ENSBTAG00000013885	3.37E-01	1.00E+00	1.77E+00	1.66E-04	1.87E+00	5.94E-05	1.05E+00	4.06E-02	-5.38E-01	8.00E-01
ENSBTAG00000033127	-1.63E+00	5.81E-01	-1.12E-01	1.00E+00	-1.79E+00	1.69E-01	-1.81E+00	1.70E-01	-7.22E-01	1.00E+00
ENSBTAG00000042709	0.00E+00	1.00E+00	0.00E+00	1.00E+00	-5.13E-15	1.00E+00	0.00E+00	1.00E+00	0.00E+00	1.00E+00
ENSBTAG00000000385	-6.48E-01	1.33E-01	2.28E-01	6.08E-01	3.12E-01	3.98E-01	3.66E-01	2.93E-01	1.09E-01	1.00E+00
ENSBTAG00000017788	5.46E-01	4.38E-01	3.31E-01	4.69E-01	1.06E-01	1.00E+00	4.37E-01	2.91E-01	-9.18E-02	1.00E+00
ENSBTAG00000017775	-1.05E-02	1.00E+00	-9.81E-01	2.32E-02	-5.98E-01	2.13E-01	-3.02E-01	6.73E-01	2.53E-01	1.00E+00
ENSBTAG00000045516	0.00E+00	1.00E+00	0.00E+00	1.00E+00	-5.13E-15	1.00E+00	0.00E+00	1.00E+00	0.00E+00	1.00E+00
ENSBTAG00000036644	0.00E+00	1.00E+00	0.00E+00	1.00E+00	-5.13E-15	1.00E+00	0.00E+00	1.00E+00	0.00E+00	1.00E+00
ENSBTAG00000007566	-1.96E-01	1.00E+00	-7.00E-01	2.10E-02	-4.11E-01	2.36E-01	-1.69E-01	8.21E-01	-2.22E-01	9.73E-01
ENSBTAG00000044744	0.00E+00	1.00E+00	0.00E+00	1.00E+00	-5.13E-15	1.00E+00	0.00E+00	1.00E+00	0.00E+00	1.00E+00
ENSBTAG00000033056	1.98E-01	1.00E+00	-4.81E-04	1.00E+00	-5.84E-02	1.00E+00	9.96E-01	1.75E-01	-7.89E-01	8.10E-01
ENSBTAG00000013700	0.00E+00	1.00E+00	0.00E+00	1.00E+00	-5.13E-15	1.00E+00	0.00E+00	1.00E+00	0.00E+00	1.00E+00
ENSBTAG00000012462	3.60E+00	2.29E-01	0.00E+00	1.00E+00	4.52E+00	4.00E-03	2.53E+00	2.71E-01	1.91E+00	8.00E-01
ENSBTAG00000009396	-3.32E-01	1.00E+00	-3.70E-01	8.94E-01	-9.57E-01	3.18E-01	-6.80E-01	5.27E-01	-1.41E+00	4.73E-01
ENSBTAG00000005200	7.42E-01	9.52E-01	1.27E+00	2.10E-01	8.07E-01	5.12E-01	1.04E+00	3.47E-01	6.68E-01	9.85E-01
ENSBTAG00000010803	5.51E-02	1.00E+00	1.90E+00	9.98E-08	1.96E+00	2.74E-08	2.24E+00	2.60E-10	3.27E-01	9.15E-01
ENSBTAG00000014439	-2.19E-01	1.00E+00	-8.68E-01	9.67E-03	-2.41E+00	4.69E-14	-3.19E+00	2.97E-22	3.74E-01	8.00E-01
ENSBTAG00000044285	0.00E+00	1.00E+00	0.00E+00	1.00E+00	-5.13E-15	1.00E+00	0.00E+00	1.00E+00	0.00E+00	1.00E+00
ENSBTAG00000021688	-2.06E-01	9.63E-01	-7.69E-01	1.87E-03	-1.38E+00	5.26E-09	-1.29E+00	7.07E-08	-2.04E-01	9.35E-01
ENSBTAG00000005410	2.26E-01	1.00E+00	1.92E-01	9.91E-01	-4.93E-02	1.00E+00	4.13E-01	5.61E-01	6.90E-02	1.00E+00
ENSBTAG00000009943	1.09E+00	3.85E-02	9.01E-01	3.22E-02	6.64E-02	1.00E+00	-4.18E-01	5.09E-01	-9.24E-01	3.44E-01
ENSBTAG00000009938	2.11E+00	1.39E-01	7.76E-01	7.28E-01	-1.53E-01	1.00E+00	-1.82E-01	1.00E+00	-1.29E+00	9.02E-01
ENSBTAG00000021211	3.02E-01	8.77E-01	1.90E-01	8.14E-01	2.98E-01	5.03E-01	4.52E-01	2.37E-01	-6.83E-02	1.00E+00
ENSBTAG00000002688	-2.49E-01	7.70E-01	4.37E-02	1.00E+00	3.56E-02	1.00E+00	3.46E-01	1.52E-01	-1.82E-01	9.00E-01
ENSBTAG00000042126	0.00E+00	1.00E+00	0.00E+00	1.00E+00	-5.13E-15	1.00E+00	0.00E+00	1.00E+00	0.00E+00	1.00E+00

ENSBTAG00000002689	7.59E-02	1.00E+00	-4.92E-01	2.40E-02	-3.86E-01	9.21E-02	-3.72E-01	1.15E-01	-2.50E-01	7.71E-01
ENSBTAG00000002690	6.11E-01	5.92E-02	2.36E+00	4.08E-27	1.76E+00	5.86E-16	1.44E+00	5.35E-11	-7.38E-02	1.00E+00
ENSBTAG00000024548	1.26E-02	1.00E+00	9.82E-01	6.54E-01	1.45E+00	3.19E-01	-2.70E+00	2.06E-01	-7.71E-01	1.00E+00
ENSBTAG00000020264	-4.42E-01	1.00E+00	5.80E-01	5.79E-01	1.13E+00	1.12E-01	2.00E-01	1.00E+00	-4.06E-01	1.00E+00
ENSBTAG00000020266	-7.24E-01	3.92E-01	-3.44E-01	6.06E-01	-2.93E-02	1.00E+00	9.58E-01	3.02E-02	-2.13E-01	1.00E+00
ENSBTAG00000017722	6.37E-01	9.70E-02	1.15E+00	5.09E-06	1.16E+00	4.38E-06	1.24E+00	1.06E-06	1.43E+00	5.73E-07
ENSBTAG00000020755	1.59E+00	9.56E-07	2.82E+00	9.26E-21	1.64E+00	3.09E-08	8.81E-01	5.73E-03	-4.28E-01	6.45E-01
ENSBTAG00000011515	4.79E-01	4.11E-01	1.25E+00	2.23E-06	1.41E+00	7.06E-08	8.88E-01	1.45E-03	3.09E-01	8.00E-01
ENSBTAG00000007307	7.99E-01	9.37E-01	7.31E-01	6.47E-01	7.68E-02	1.00E+00	2.76E-01	1.00E+00	2.72E-01	1.00E+00
ENSBTAG00000021671	-3.72E-01	5.03E-01	9.58E-01	1.15E-05	9.33E-01	1.76E-05	3.07E-01	2.90E-01	-4.60E-01	3.47E-01
ENSBTAG00000014793	7.16E-02	1.00E+00	4.91E-01	1.25E-01	9.26E-01	6.21E-04	4.33E-01	2.00E-01	-2.84E-01	8.44E-01
ENSBTAG00000014794	2.96E-02	1.00E+00	7.57E-01	2.67E-05	7.22E-01	6.17E-05	4.52E-01	2.24E-02	3.56E-03	1.00E+00
ENSBTAG00000028273	0.00E+00	1.00E+00	0.00E+00	1.00E+00	-5.13E-15	1.00E+00	0.00E+00	1.00E+00	0.00E+00	1.00E+00
ENSBTAG00000017727	1.19E-01	1.00E+00	-8.51E-01	1.95E-04	-1.25E+00	1.68E-08	-7.20E-01	2.14E-03	-1.38E-01	1.00E+00
ENSBTAG00000042655	0.00E+00	1.00E+00	0.00E+00	1.00E+00	-5.13E-15	1.00E+00	0.00E+00	1.00E+00	0.00E+00	1.00E+00
ENSBTAG00000021298	-3.21E+00	3.75E-01	-4.17E-01	1.00E+00	-6.55E-01	8.96E-01	-1.44E+00	4.97E-01	-1.28E+00	9.06E-01
ENSBTAG00000045831	0.00E+00	1.00E+00	0.00E+00	1.00E+00	-5.13E-15	1.00E+00	0.00E+00	1.00E+00	0.00E+00	1.00E+00
ENSBTAG00000003295	-4.15E-02	1.00E+00	6.62E-01	1.08E-02	1.04E+00	1.72E-05	9.37E-01	1.54E-04	-7.58E-02	1.00E+00
ENSBTAG00000004570	-1.72E+00	3.75E-01	2.37E-01	1.00E+00	-4.38E-01	8.43E-01	-6.20E-01	6.61E-01	-5.81E-01	1.00E+00
ENSBTAG00000006408	2.14E-01	1.00E+00	1.18E+00	3.43E-01	-1.44E-01	1.00E+00	8.84E-01	5.40E-01	-4.69E-01	1.00E+00
ENSBTAG00000020597	3.83E-03	1.00E+00	-6.87E-01	6.23E-03	-1.04E+00	1.44E-05	-1.05E+00	1.48E-05	2.62E-02	1.00E+00
ENSBTAG00000047256	1.08E+00	1.52E-01	-7.08E-01	3.55E-01	-1.01E+00	1.28E-01	-2.24E+00	1.04E-03	4.49E-01	9.00E-01
ENSBTAG00000002974	-5.91E-02	1.00E+00	-1.25E+00	7.44E-04	-1.02E+00	6.47E-03	-4.67E-01	3.25E-01	9.75E-02	1.00E+00
ENSBTAG00000021408	-2.47E-01	8.40E-01	-1.45E+00	6.52E-10	-3.42E+00	1.08E-44	-4.91E+00	1.03E-78	2.10E-01	9.13E-01
ENSBTAG00000044903	0.00E+00	1.00E+00	0.00E+00	1.00E+00	-5.13E-15	1.00E+00	0.00E+00	1.00E+00	0.00E+00	1.00E+00
ENSBTAG00000016685	1.06E-01	1.00E+00	-1.43E+00	3.92E-07	-1.67E+00	2.18E-09	-1.27E+00	8.60E-06	4.07E-01	6.27E-01
ENSBTAG00000001821	1.31E-01	1.00E+00	5.68E-01	5.62E-02	8.45E-01	2.13E-03	7.00E-01	1.55E-02	2.40E-01	9.05E-01
ENSBTAG00000020342	5.10E-01	1.00E+00	5.92E-01	9.48E-01	-2.71E+00	1.99E-01	6.80E-01	8.65E-01	1.18E+00	8.33E-01
ENSBTAG00000020591	-2.17E-01	9.32E-01	-1.05E+00	2.02E-05	-7.82E-01	1.50E-03	-7.73E-01	2.07E-03	-2.79E-01	8.00E-01
ENSBTAG00000017721	-6.57E-02	1.00E+00	1.21E+00	1.08E-05	1.31E+00	1.77E-06	8.70E-01	2.72E-03	-2.04E-01	1.00E+00
ENSBTAG00000014319	-5.28E-02	1.00E+00	2.12E-01	9.15E-01	-5.48E-01	3.42E-01	-2.18E-01	9.04E-01	-4.59E-01	8.07E-01
ENSBTAG00000045018	0.00E+00	1.00E+00	0.00E+00	1.00E+00	-5.13E-15	1.00E+00	0.00E+00	1.00E+00	0.00E+00	1.00E+00
ENSBTAG00000029886	-5.31E+00	1.14E-03	-3.32E+00	3.30E-03	-7.77E-01	4.46E-01	-1.60E+00	8.36E-02	-4.74E-01	1.00E+00
ENSBTAG00000029931	5.05E-01	1.00E+00	-2.71E+00	2.42E-01	2.09E+00	6.53E-02	-1.72E-01	1.00E+00	1.66E+00	6.01E-01
ENSBTAG00000043754	0.00E+00	1.00E+00	0.00E+00	1.00E+00	-5.13E-15	1.00E+00	0.00E+00	1.00E+00	0.00E+00	1.00E+00

ENSBTAG00000024542	-8.06E-02	1.00E+00	-2.17E-01	4.59E-01	-3.37E-01	1.66E-01	-2.88E-01	2.73E-01	-5.51E-02	1.00E+00
ENSBTAG00000010169	5.89E-01	1.66E-01	2.22E+00	1.15E-18	1.41E+00	2.44E-08	1.99E+00	3.11E-15	1.80E-01	1.00E+00
ENSBTAG00000032808	9.52E-01	1.71E-01	1.16E+00	8.44E-03	-4.13E-01	5.57E-01	-1.21E+00	2.30E-02	-8.53E-01	4.55E-01
ENSBTAG00000016347	1.93E-01	9.53E-01	1.49E+00	3.07E-12	1.98E+00	1.15E-20	1.94E+00	9.16E-20	-5.49E-01	1.67E-01
ENSBTAG00000009621	-2.69E-02	1.00E+00	2.79E-01	2.88E-01	-5.56E-02	1.00E+00	-1.69E-01	6.37E-01	-1.78E-02	1.00E+00
ENSBTAG00000047412	-3.30E-01	1.00E+00	-8.03E-01	1.15E-01	-1.90E+00	2.71E-05	-1.24E+00	8.89E-03	-1.11E+00	1.56E-01
ENSBTAG00000002894	-3.51E-01	1.00E+00	7.85E-01	3.35E-01	-2.72E-01	9.99E-01	-5.69E-01	6.52E-01	9.81E-01	5.25E-01
ENSBTAG00000024493	-4.20E-01	5.30E-01	-1.29E+00	9.02E-07	-1.73E+00	2.24E-11	-9.10E-01	8.53E-04	1.26E-01	1.00E+00
ENSBTAG00000016080	3.84E-01	4.63E-01	3.56E-01	2.04E-01	4.91E-01	5.11E-02	7.63E-01	1.23E-03	2.58E-01	8.00E-01
ENSBTAG00000044521	0.00E+00	1.00E+00	0.00E+00	1.00E+00	-5.13E-15	1.00E+00	0.00E+00	1.00E+00	0.00E+00	1.00E+00
ENSBTAG00000024928	2.12E-01	1.00E+00	2.20E-01	6.70E-01	9.51E-01	9.16E-04	1.19E+00	2.80E-05	4.65E-01	5.32E-01
ENSBTAG00000039937	3.47E-02	1.00E+00	-1.86E+00	3.81E-05	-2.97E+00	1.88E-10	-2.00E+00	9.27E-06	1.00E+00	1.77E-01
ENSBTAG00000019556	3.85E-03	1.00E+00	-9.10E-01	1.12E-04	-1.23E+00	9.46E-08	-1.33E+00	9.42E-09	1.88E-01	9.66E-01
ENSBTAG00000005314	-1.23E-01	1.00E+00	-4.46E-01	1.90E-01	-3.40E-01	3.70E-01	3.25E-01	4.17E-01	3.96E-01	6.45E-01
ENSBTAG00000002052	3.61E-03	1.00E+00	-1.37E-01	7.94E-01	-8.05E-01	1.95E-04	-7.47E-01	7.52E-04	6.14E-01	6.44E-02
ENSBTAG00000045716	-2.70E+00	5.76E-01	-2.70E+00	2.42E-01	3.46E-01	1.00E+00	-2.70E+00	2.05E-01	-2.70E+00	6.01E-01
ENSBTAG00000019388	-4.90E-01	1.70E-01	-4.53E-01	6.11E-02	-9.15E-02	9.79E-01	1.35E-01	8.06E-01	1.17E-01	1.00E+00
ENSBTAG00000021739	1.92E+00	8.23E-01	0.00E+00	1.00E+00	1.78E+00	4.79E-01	0.00E+00	1.00E+00	0.00E+00	1.00E+00
ENSBTAG00000006709	0.00E+00	1.00E+00	0.00E+00	1.00E+00	-5.13E-15	1.00E+00	0.00E+00	1.00E+00	0.00E+00	1.00E+00
ENSBTAG00000020700	-3.84E-01	8.23E-01	-9.18E-01	9.02E-03	-4.32E-01	3.15E-01	-7.88E-01	3.17E-02	1.17E-01	1.00E+00
ENSBTAG00000020698	-3.52E-01	8.23E-01	-3.61E-01	3.90E-01	2.80E-01	5.46E-01	-5.36E-01	1.40E-01	-3.53E-01	8.00E-01
ENSBTAG00000009386	-5.03E-01	7.34E-01	3.22E-01	6.25E-01	5.92E-01	2.12E-01	-8.52E-01	5.30E-02	-2.29E-01	1.00E+00
ENSBTAG00000009384	-1.43E-01	1.00E+00	-4.50E-01	9.04E-02	-9.93E-01	1.96E-05	-1.00E+00	2.00E-05	-2.63E-01	8.00E-01
ENSBTAG00000014359	-1.91E+00	8.23E-01	3.16E+00	7.51E-03	2.06E+00	1.80E-01	1.49E+00	4.53E-01	2.62E+00	2.78E-01
ENSBTAG00000013275	-1.78E-01	1.00E+00	2.67E-01	4.65E-01	2.96E-01	4.00E-01	7.50E-02	1.00E+00	-1.56E-01	1.00E+00
ENSBTAG00000014777	-3.39E-01	6.97E-01	-8.47E-01	1.38E-03	-1.29E-01	8.96E-01	-2.91E-01	4.23E-01	1.02E-01	1.00E+00
ENSBTAG00000032617	-3.33E-01	9.80E-01	-9.51E-01	2.82E-02	-1.80E+00	7.85E-06	-1.67E+00	4.75E-05	-4.51E-02	1.00E+00
ENSBTAG00000007223	1.46E-01	1.00E+00	3.36E-01	8.23E-01	-4.50E-02	1.00E+00	-4.27E-01	6.90E-01	5.07E-01	8.96E-01
ENSBTAG00000015533	-1.68E+00	7.75E-01	-2.98E-01	1.00E+00	-1.82E+00	2.91E-01	3.31E-01	1.00E+00	-1.18E-03	1.00E+00
ENSBTAG00000014014	-1.07E-01	1.00E+00	1.28E+00	5.69E-05	2.15E+00	1.15E-12	1.59E+00	2.82E-07	6.26E-02	1.00E+00
ENSBTAG00000015325	4.90E-01	2.17E-01	1.26E+00	1.77E-08	1.67E+00	5.22E-14	1.36E+00	1.16E-09	2.68E-01	8.00E-01
ENSBTAG00000015340	0.00E+00	1.00E+00	0.00E+00	1.00E+00	-5.13E-15	1.00E+00	0.00E+00	1.00E+00	0.00E+00	1.00E+00
ENSBTAG00000002590	4.91E-01	1.67E-01	1.60E-01	7.08E-01	1.05E+00	7.84E-07	1.04E+00	1.21E-06	8.90E-02	1.00E+00
ENSBTAG00000005304	1.29E+00	1.16E-04	3.07E+00	7.23E-27	3.80E+00	1.76E-39	2.84E+00	2.44E-23	-3.03E-02	1.00E+00
ENSBTAG00000012808	6.41E-01	3.81E-02	1.27E-01	8.56E-01	-1.34E-01	8.22E-01	-8.37E-01	2.80E-04	5.73E-01	1.38E-01

ENSBTAG00000003697	5.80E-02	1.00E+00	3.09E-01	1.83E-01	3.97E-01	6.36E-02	-8.88E-03	1.00E+00	1.11E-02	1.00E+00
ENSBTAG00000019818	-1.98E-01	1.00E+00	-1.02E-01	1.00E+00	1.92E-01	9.03E-01	6.22E-01	1.70E-01	4.51E-01	8.00E-01
ENSBTAG00000013538	-1.35E-01	1.00E+00	-5.71E-01	1.25E-02	-1.57E+00	1.02E-13	-1.46E+00	7.33E-12	1.25E-01	1.00E+00
ENSBTAG00000013535	-5.52E-02	1.00E+00	1.72E-02	1.00E+00	3.13E-01	3.46E-01	7.58E-01	3.66E-03	1.22E-01	1.00E+00
ENSBTAG00000045105	0.00E+00	1.00E+00	0.00E+00	1.00E+00	-5.13E-15	1.00E+00	0.00E+00	1.00E+00	0.00E+00	1.00E+00
ENSBTAG00000046494	-3.21E+00	3.75E-01	8.99E-02	1.00E+00	-3.21E+00	9.44E-02	-1.45E+00	4.94E-01	-1.30E+00	9.00E-01
ENSBTAG00000013531	-3.35E-01	6.08E-01	-5.56E-01	2.80E-02	-6.34E-01	9.41E-03	-4.71E-01	7.53E-02	-4.04E-01	4.79E-01
ENSBTAG00000013527	-3.91E-01	4.82E-01	-6.67E-01	6.96E-03	-5.74E-01	2.34E-02	-2.93E-01	3.65E-01	-4.07E-01	4.87E-01
ENSBTAG00000004966	6.99E-02	1.00E+00	3.26E-02	1.00E+00	2.73E-01	4.48E-01	9.39E-01	2.78E-04	1.17E-01	1.00E+00
ENSBTAG00000044699	0.00E+00	1.00E+00	0.00E+00	1.00E+00	-5.13E-15	1.00E+00	0.00E+00	1.00E+00	0.00E+00	1.00E+00
ENSBTAG00000010401	3.78E-02	1.00E+00	-4.64E-01	2.24E-02	-4.96E-01	1.32E-02	-1.71E-01	5.71E-01	2.35E-01	7.70E-01
ENSBTAG00000015574	1.91E-02	1.00E+00	9.81E-01	6.50E-01	-9.22E-01	8.08E-01	6.95E-01	8.55E-01	1.81E-02	1.00E+00
ENSBTAG00000001361	4.36E-01	5.75E-01	1.00E+00	4.38E-04	1.05E-01	1.00E+00	2.56E-01	5.72E-01	3.23E-01	8.00E-01
ENSBTAG00000005933	3.79E-01	5.54E-01	-4.53E-01	1.15E-01	-1.08E+00	1.67E-05	-1.20E+00	2.00E-06	1.25E-02	1.00E+00
ENSBTAG00000011823	-1.68E-01	1.00E+00	-1.05E+00	8.86E-06	-7.40E-01	2.45E-03	-2.73E-01	4.25E-01	7.17E-01	4.48E-02
ENSBTAG00000018984	-7.00E-01	8.03E-02	-2.11E-01	6.63E-01	1.25E+00	1.29E-06	3.89E-01	2.47E-01	-3.27E-02	1.00E+00
ENSBTAG00000042888	0.00E+00	1.00E+00	0.00E+00	1.00E+00	-5.13E-15	1.00E+00	0.00E+00	1.00E+00	0.00E+00	1.00E+00
ENSBTAG00000036516	0.00E+00	1.00E+00	0.00E+00	1.00E+00	-5.13E-15	1.00E+00	1.76E+00	4.97E-01	0.00E+00	1.00E+00
ENSBTAG00000042532	0.00E+00	1.00E+00	0.00E+00	1.00E+00	-5.13E-15	1.00E+00	0.00E+00	1.00E+00	0.00E+00	1.00E+00
ENSBTAG00000003180	5.31E-02	1.00E+00	-2.55E-01	4.56E-01	5.23E-01	4.07E-02	7.83E-01	1.15E-03	2.15E-01	8.98E-01
ENSBTAG00000003177	-2.07E-01	1.00E+00	-4.49E-01	2.99E-01	2.77E-01	6.17E-01	-4.84E-01	2.58E-01	-5.62E-01	5.09E-01
ENSBTAG00000024539	3.67E-01	8.87E-01	2.25E+00	2.85E-09	1.55E+00	4.56E-05	-3.74E-02	1.00E+00	3.10E-01	9.56E-01
ENSBTAG00000004246	6.41E-01	4.47E-01	5.98E-01	1.89E-01	1.49E+00	8.07E-05	2.28E+00	1.63E-09	1.17E+00	3.65E-02
ENSBTAG00000029924	-1.91E+00	8.23E-01	-1.91E+00	4.69E-01	-1.30E-01	1.00E+00	-1.91E+00	4.39E-01	-1.91E+00	8.00E-01
ENSBTAG00000037072	0.00E+00	1.00E+00	0.00E+00	1.00E+00	-5.13E-15	1.00E+00	0.00E+00	1.00E+00	0.00E+00	1.00E+00
ENSBTAG00000012605	-9.46E-01	7.57E-01	-1.62E+00	9.19E-02	-1.68E-01	1.00E+00	-1.03E-01	1.00E+00	-1.96E-01	1.00E+00
ENSBTAG00000034323	1.35E+00	1.00E+00	-2.92E-01	1.00E+00	-1.34E+00	7.81E-01	-3.59E+00	2.04E-01	1.03E+00	1.00E+00
ENSBTAG00000009515	-2.70E+00	5.76E-01	-7.17E-01	9.45E-01	-9.22E-01	8.08E-01	6.95E-01	8.55E-01	-7.85E-01	1.00E+00
ENSBTAG00000013411	-1.17E-01	1.00E+00	-2.15E-01	6.81E-01	1.46E-01	8.93E-01	7.62E-01	1.18E-02	-3.23E-01	8.00E-01
ENSBTAG00000042245	3.88E+00	1.42E-01	1.98E+00	4.43E-01	3.73E+00	3.99E-02	1.77E+00	4.97E-01	0.00E+00	1.00E+00
ENSBTAG00000018272	1.98E-01	1.00E+00	5.62E-01	1.69E-01	-9.43E-02	1.00E+00	-4.47E-01	3.29E-01	3.49E-01	8.24E-01
ENSBTAG00000001401	1.16E+00	8.23E-01	1.72E+00	1.19E-01	5.06E-01	9.43E-01	1.80E-01	1.00E+00	3.85E-01	1.00E+00
ENSBTAG00000011650	-1.27E-01	1.00E+00	2.58E-01	5.54E-01	5.20E-01	9.97E-02	3.43E-01	3.71E-01	-5.94E-01	3.29E-01
ENSBTAG00000006609	4.16E-01	8.23E-01	5.59E-01	2.12E-01	-3.29E-01	5.65E-01	-4.21E-01	4.24E-01	7.44E-02	1.00E+00
ENSBTAG00000020518	-3.85E-02	1.00E+00	-2.07E-03	1.00E+00	-1.86E-01	6.11E-01	-2.76E-01	3.62E-01	-9.15E-03	1.00E+00

ENSBTAG00000003313	1.10E+00	8.23E-01	6.98E-02	1.00E+00	-2.13E-01	1.00E+00	5.43E-01	8.89E-01	-5.49E-01	1.00E+00
ENSBTAG00000014726	-1.05E-01	1.00E+00	-7.78E-01	5.07E-04	-1.56E-01	7.13E-01	4.66E-03	1.00E+00	-2.84E-01	7.33E-01
ENSBTAG00000014602	2.58E-01	1.00E+00	2.65E+00	6.39E-10	2.11E+00	1.14E-06	1.95E+00	1.16E-05	-4.43E-01	9.31E-01
ENSBTAG00000011217	-1.04E-01	1.00E+00	1.45E-01	8.24E-01	5.99E-01	1.82E-02	6.11E-01	1.78E-02	-6.18E-02	1.00E+00
ENSBTAG00000011214	-6.15E-01	2.45E-02	-1.50E+00	1.23E-13	-1.66E+00	1.27E-16	-1.53E+00	3.64E-14	4.53E-02	1.00E+00
ENSBTAG00000015384	-3.73E-01	8.77E-01	-3.31E-01	5.91E-01	4.83E-01	2.96E-01	9.98E-01	8.11E-03	5.64E-01	5.91E-01
ENSBTAG00000008350	-4.48E-01	9.05E-01	7.87E-02	1.00E+00	5.34E-01	4.26E-01	7.10E-01	2.36E-01	6.30E-01	7.08E-01
ENSBTAG00000016735	-3.11E-02	1.00E+00	6.65E-01	5.65E-03	4.05E-01	1.33E-01	-3.38E-01	2.64E-01	-2.91E-02	1.00E+00
ENSBTAG00000016734	-1.96E+00	3.74E-01	6.27E-01	6.24E-01	-7.19E-01	6.19E-01	-5.35E-01	7.99E-01	-2.97E-01	1.00E+00
ENSBTAG00000016733	-2.10E-01	1.00E+00	1.85E-01	8.78E-01	1.31E+00	1.33E-04	5.16E-01	2.29E-01	2.61E-01	9.94E-01
ENSBTAG00000016728	-2.41E-01	1.00E+00	-1.48E+00	4.09E-05	-1.72E+00	1.13E-06	-1.28E+00	2.87E-04	-2.11E-01	1.00E+00
ENSBTAG00000006519	7.09E-01	2.68E-01	-2.16E-01	8.53E-01	-1.22E+00	3.45E-03	-7.95E-01	7.16E-02	-3.65E-01	8.61E-01
ENSBTAG00000006515	-5.96E-01	2.64E-01	-1.26E+00	1.99E-05	-2.13E+00	3.31E-13	-2.66E+00	5.14E-19	-6.67E-01	2.36E-01
ENSBTAG00000021210	-1.68E+00	4.10E-05	-1.95E+00	1.88E-07	-6.51E-01	1.00E-01	-6.89E-01	8.42E-02	-4.88E-01	6.51E-01
ENSBTAG00000021202	1.41E-01	1.00E+00	1.24E+00	5.31E-06	-3.69E-02	1.00E+00	-4.38E-03	1.00E+00	1.27E-02	1.00E+00
ENSBTAG00000013071	1.16E+00	5.24E-01	4.40E-01	8.79E-01	1.96E+00	3.14E-03	9.50E-01	3.30E-01	-1.96E-01	1.00E+00
ENSBTAG00000024525	1.92E+00	8.23E-01	1.98E+00	4.43E-01	-5.13E-15	1.00E+00	0.00E+00	1.00E+00	0.00E+00	1.00E+00
ENSBTAG00000048308	-1.72E-01	1.00E+00	-8.03E-01	3.36E-03	4.09E-02	1.00E+00	6.35E-01	2.41E-02	-1.43E-01	1.00E+00
ENSBTAG00000009075	-7.03E-02	1.00E+00	-6.89E-01	1.47E-02	-2.20E-01	6.14E-01	-1.30E-01	9.22E-01	-8.65E-02	1.00E+00
ENSBTAG00000014423	-1.12E-01	1.00E+00	9.48E-02	1.00E+00	-2.47E-02	1.00E+00	3.15E-02	1.00E+00	-7.24E-01	6.02E-02
ENSBTAG00000040477	-1.05E+00	2.27E-01	-2.77E+00	1.21E-07	-1.05E+00	5.75E-02	-2.17E-01	9.68E-01	2.72E-01	1.00E+00
ENSBTAG00000008125	-2.95E-01	1.00E+00	-6.35E-01	2.43E-01	-7.63E-01	1.29E-01	-1.07E+00	2.76E-02	-3.85E-01	9.00E-01
ENSBTAG00000008126	-4.52E-01	7.14E-01	-1.38E+00	2.73E-04	-1.35E+00	2.25E-04	-6.64E-01	9.59E-02	-1.98E-01	1.00E+00
ENSBTAG00000047285	0.00E+00	1.00E+00	1.98E+00	4.43E-01	3.06E+00	1.36E-01	0.00E+00	1.00E+00	0.00E+00	1.00E+00
ENSBTAG00000045789	-1.42E+00	4.07E-01	-2.53E+00	1.35E-02	-1.89E+00	3.71E-02	-1.91E+00	3.91E-02	-6.11E-01	9.35E-01
ENSBTAG00000048267	-3.64E-01	1.00E+00	-3.59E+00	6.66E-02	1.31E-01	1.00E+00	-3.59E+00	4.90E-02	2.06E-02	1.00E+00
ENSBTAG00000024663	-1.20E+00	2.72E-01	-1.07E+00	1.22E-01	-1.58E+00	1.43E-02	-1.77E+00	6.64E-03	-6.06E-01	8.01E-01
ENSBTAG00000047231	3.96E-01	1.00E+00	9.80E-01	5.31E-01	-7.03E-01	7.60E-01	9.92E-01	5.28E-01	3.19E-01	1.00E+00
ENSBTAG00000016057	3.12E-01	8.23E-01	-1.06E+00	2.86E-04	-1.03E+00	3.78E-04	-7.49E-01	1.51E-02	-2.69E-01	8.75E-01
ENSBTAG00000046062	1.64E+00	4.38E-01	9.54E-02	1.00E+00	1.71E+00	1.09E-01	1.45E+00	2.32E-01	1.50E+00	6.01E-01
ENSBTAG00000021919	-1.28E-01	1.00E+00	-9.18E-01	1.03E-03	-1.08E-01	9.87E-01	-2.37E-01	5.74E-01	-1.49E-01	1.00E+00
ENSBTAG00000004452	2.28E-02	1.00E+00	7.19E-01	6.59E-05	7.94E-01	7.96E-06	5.06E-01	8.43E-03	1.64E-01	9.00E-01
ENSBTAG00000030805	5.19E-01	6.41E-01	1.38E-01	1.00E+00	2.92E-01	6.56E-01	1.91E-01	9.09E-01	1.22E-01	1.00E+00
ENSBTAG00000021576	-6.19E-01	4.41E-01	-1.07E+00	4.78E-03	5.47E-02	1.00E+00	-2.54E-01	7.33E-01	-1.64E-01	1.00E+00
ENSBTAG00000047059	2.79E-02	1.00E+00	1.48E+00	2.33E-09	2.56E+00	3.57E-25	2.60E+00	6.51E-26	-2.17E-01	9.48E-01

ENSBTAG00000000792	-7.87E-01	1.00E+00	-7.15E-01	9.45E-01	-9.17E-01	8.09E-01	1.24E+00	4.54E-01	7.06E-03	1.00E+00
ENSBTAG00000024520	-3.44E-01	5.95E-01	3.28E-01	2.64E-01	4.24E-01	1.12E-01	1.25E-01	8.83E-01	1.19E-02	1.00E+00
ENSBTAG00000020015	-3.80E-01	5.56E-01	-4.52E-01	1.07E-01	-4.26E-01	1.32E-01	-4.56E-01	1.07E-01	3.07E-01	7.42E-01
ENSBTAG00000020014	-5.27E-02	1.00E+00	1.22E+00	4.13E-04	1.06E+00	2.55E-03	8.21E-01	2.81E-02	4.11E-01	7.88E-01
ENSBTAG00000045373	0.00E+00	1.00E+00	0.00E+00	1.00E+00	-5.13E-15	1.00E+00	0.00E+00	1.00E+00	0.00E+00	1.00E+00
ENSBTAG00000004738	-9.75E-02	1.00E+00	9.80E-01	1.03E-06	1.20E+00	1.18E-09	8.64E-01	2.39E-05	-1.30E-01	1.00E+00
ENSBTAG00000045754	-3.09E-01	1.00E+00	-1.02E+00	2.13E-02	-1.43E+00	7.31E-04	-3.11E-01	6.73E-01	-7.12E-02	1.00E+00
ENSBTAG00000046007	1.32E+00	9.30E-01	1.39E+00	5.16E-01	6.45E-01	9.78E-01	6.26E-01	9.94E-01	7.99E-01	1.00E+00
ENSBTAG00000005812	2.52E-01	1.00E+00	6.57E-01	2.45E-01	1.63E-01	1.00E+00	-2.58E-01	8.87E-01	-1.53E-01	1.00E+00
ENSBTAG00000037296	0.00E+00	1.00E+00	0.00E+00	1.00E+00	-5.13E-15	1.00E+00	0.00E+00	1.00E+00	0.00E+00	1.00E+00
ENSBTAG00000015859	-3.02E-01	6.30E-01	3.21E-01	2.14E-01	5.62E-01	1.03E-02	1.92E-01	5.67E-01	2.72E-01	7.36E-01
ENSBTAG00000015857	-2.68E-01	7.07E-01	-8.15E-01	5.79E-05	-1.24E+00	3.73E-10	-9.22E-01	5.27E-06	-9.44E-02	1.00E+00
ENSBTAG00000020839	5.53E-02	1.00E+00	-1.11E+00	8.50E-06	-9.60E-01	1.02E-04	-1.04E+00	2.92E-05	9.60E-02	1.00E+00
ENSBTAG00000030099	0.00E+00	1.00E+00	0.00E+00	1.00E+00	-5.13E-15	1.00E+00	0.00E+00	1.00E+00	0.00E+00	1.00E+00
ENSBTAG00000020837	-2.20E-02	1.00E+00	-2.55E-01	6.55E-01	-7.86E-01	2.32E-02	-1.49E+00	5.83E-06	-1.50E-01	1.00E+00
ENSBTAG00000018965	9.12E-03	1.00E+00	-8.86E-01	3.26E-01	-1.31E+00	9.95E-02	-6.65E-01	4.83E-01	-6.96E-01	8.00E-01
ENSBTAG00000017929	0.00E+00	1.00E+00	0.00E+00	1.00E+00	-5.13E-15	1.00E+00	0.00E+00	1.00E+00	1.91E+00	8.00E-01
ENSBTAG00000001642	1.12E-02	1.00E+00	7.57E-02	1.00E+00	-1.91E+00	4.28E-01	-1.47E-01	1.00E+00	6.46E-03	1.00E+00
ENSBTAG00000009349	-1.61E+00	4.51E-01	-2.05E+00	7.85E-02	-1.79E+00	9.48E-02	-9.02E-01	4.53E-01	-6.90E-01	9.46E-01
ENSBTAG00000001727	-2.86E-01	7.80E-01	-8.16E-01	6.81E-04	-1.25E+00	6.22E-08	-1.39E+00	2.27E-09	-6.46E-01	8.94E-02
ENSBTAG00000043866	0.00E+00	1.00E+00	0.00E+00	1.00E+00	-5.13E-15	1.00E+00	0.00E+00	1.00E+00	0.00E+00	1.00E+00
ENSBTAG00000002483	2.71E+00	5.76E-01	0.00E+00	1.00E+00	2.55E+00	2.54E-01	0.00E+00	1.00E+00	0.00E+00	1.00E+00
ENSBTAG00000002479	-6.32E-01	4.91E-02	-8.84E-01	1.32E-04	-2.44E-01	4.49E-01	-3.70E-01	1.84E-01	3.21E-02	1.00E+00
ENSBTAG00000002464	1.09E-02	1.00E+00	9.84E-01	2.70E-01	-1.27E+00	2.74E-01	-5.44E-01	7.80E-01	-5.38E-01	1.00E+00
ENSBTAG00000009309	-4.78E-01	1.70E-01	-1.83E+00	1.85E-18	-2.36E+00	1.88E-29	-2.03E+00	2.02E-22	1.71E-01	9.64E-01
ENSBTAG00000009307	3.90E-02	1.00E+00	9.04E-02	9.66E-01	4.90E-02	1.00E+00	6.32E-02	1.00E+00	-1.58E-01	9.90E-01
ENSBTAG00000009306	-3.54E-01	8.23E-01	-1.12E+00	7.14E-04	-1.67E-01	8.66E-01	1.33E-01	9.68E-01	-1.91E-02	1.00E+00
ENSBTAG00000045918	0.00E+00	1.00E+00	0.00E+00	1.00E+00	-5.13E-15	1.00E+00	0.00E+00	1.00E+00	0.00E+00	1.00E+00
ENSBTAG00000038716	-2.37E-01	8.85E-01	-3.40E-01	2.70E-01	-5.08E-01	6.01E-02	-6.79E-02	1.00E+00	4.62E-01	4.01E-01
ENSBTAG00000014136	-3.47E-01	6.46E-01	-9.28E-01	3.22E-04	-4.21E-01	1.50E-01	-5.79E-01	3.57E-02	-5.01E-01	3.49E-01
ENSBTAG00000014119	-1.35E-01	1.00E+00	-7.05E-01	2.46E-03	-7.45E-01	1.21E-03	-3.70E-01	1.79E-01	-9.16E-02	1.00E+00
ENSBTAG00000045361	0.00E+00	1.00E+00	0.00E+00	1.00E+00	-5.13E-15	1.00E+00	0.00E+00	1.00E+00	0.00E+00	1.00E+00
ENSBTAG00000008672	1.98E-01	1.00E+00	-1.21E+00	3.80E-01	-5.63E-01	7.81E-01	-1.11E+00	4.04E-01	4.31E-01	1.00E+00
ENSBTAG00000005231	-3.41E-01	1.00E+00	-5.58E-01	5.93E-01	-6.51E-01	4.71E-01	-1.53E+00	4.98E-02	-2.72E-01	1.00E+00
ENSBTAG00000024510	-1.29E+00	9.36E-01	-4.22E-01	1.00E+00	-6.61E-01	8.93E-01	-3.21E+00	9.91E-02	7.18E-04	1.00E+00

ENSBTAG00000024509	-1.05E+00	7.01E-01	-2.10E+00	3.73E-02	-5.93E-01	6.35E-01	5.89E-01	5.64E-01	-2.57E-01	1.00E+00
ENSBTAG00000024508	-7.87E-01	1.00E+00	-2.70E+00	2.42E-01	-2.70E+00	1.99E-01	1.44E+00	3.29E-01	1.19E+00	8.27E-01
ENSBTAG00000043783	0.00E+00	1.00E+00	0.00E+00	1.00E+00	-5.13E-15	1.00E+00	1.76E+00	4.97E-01	0.00E+00	1.00E+00
ENSBTAG00000000215	7.45E-02	1.00E+00	1.59E-01	6.33E-01	-1.98E-01	4.79E-01	-2.10E-01	4.57E-01	-9.82E-02	1.00E+00
ENSBTAG00000000212	-4.10E-01	5.52E-01	-7.58E-01	5.38E-03	-7.08E-01	9.97E-03	-3.13E-01	3.96E-01	2.19E-01	9.41E-01
ENSBTAG00000031749	-8.24E-01	6.33E-02	-2.09E+00	1.27E-11	-2.93E+00	8.42E-21	-2.69E+00	6.73E-18	5.80E-01	4.15E-01
ENSBTAG00000010737	-1.77E-01	9.59E-01	7.52E-01	2.11E-04	6.37E-01	2.04E-03	3.80E-01	1.08E-01	-1.39E-02	1.00E+00
ENSBTAG00000039728	-5.40E-01	1.85E-01	-2.38E-01	5.05E-01	-5.18E-01	5.20E-02	-8.40E-01	8.54E-04	-3.78E-01	5.90E-01
ENSBTAG00000010732	0.00E+00	1.00E+00	0.00E+00	1.00E+00	-5.13E-15	1.00E+00	0.00E+00	1.00E+00	0.00E+00	1.00E+00
ENSBTAG00000010720	0.00E+00	1.00E+00	0.00E+00	1.00E+00	-5.13E-15	1.00E+00	0.00E+00	1.00E+00	0.00E+00	1.00E+00
ENSBTAG00000047755	0.00E+00	1.00E+00	0.00E+00	1.00E+00	-5.13E-15	1.00E+00	0.00E+00	1.00E+00	0.00E+00	1.00E+00
ENSBTAG00000002575	0.00E+00	1.00E+00	0.00E+00	1.00E+00	-5.13E-15	1.00E+00	0.00E+00	1.00E+00	0.00E+00	1.00E+00
ENSBTAG00000046073	0.00E+00	1.00E+00	0.00E+00	1.00E+00	-5.13E-15	1.00E+00	0.00E+00	1.00E+00	0.00E+00	1.00E+00
ENSBTAG00000002219	0.00E+00	1.00E+00	0.00E+00	1.00E+00	-5.13E-15	1.00E+00	0.00E+00	1.00E+00	0.00E+00	1.00E+00
ENSBTAG00000021294	0.00E+00	1.00E+00	0.00E+00	1.00E+00	-5.13E-15	1.00E+00	0.00E+00	1.00E+00	0.00E+00	1.00E+00
ENSBTAG00000023784	1.92E+00	8.23E-01	1.99E+00	4.43E-01	1.78E+00	4.79E-01	0.00E+00	1.00E+00	0.00E+00	1.00E+00
ENSBTAG00000039055	-3.21E+00	3.75E-01	-4.25E-01	1.00E+00	-1.43E+00	4.79E-01	-6.79E-01	8.87E-01	-5.47E-05	1.00E+00
ENSBTAG00000004872	4.48E-02	1.00E+00	9.13E-01	8.75E-06	1.19E+00	2.84E-09	8.37E-01	6.09E-05	-1.78E-01	9.30E-01
ENSBTAG00000004873	5.20E-01	9.78E-02	1.17E+00	9.42E-09	9.36E-01	5.06E-06	1.79E-01	6.09E-01	4.14E-02	1.00E+00
ENSBTAG00000004874	-2.41E-01	8.65E-01	-2.46E-01	4.76E-01	1.39E-01	8.35E-01	7.43E-02	1.00E+00	-2.79E-01	8.00E-01
ENSBTAG00000012247	-3.97E-01	6.07E-01	-1.94E+00	1.78E-11	-2.40E+00	1.14E-16	-1.86E+00	5.13E-11	-2.49E-01	8.99E-01
ENSBTAG00000010696	-3.31E-01	7.42E-01	-6.30E-01	2.33E-02	-3.45E-02	1.00E+00	-9.33E-02	1.00E+00	-2.02E-01	9.80E-01
ENSBTAG00000015882	-2.05E-01	8.87E-01	8.75E-01	2.66E-05	8.04E-01	1.20E-04	3.93E-01	1.10E-01	-1.61E-01	9.97E-01
ENSBTAG00000031631	-6.88E-01	1.21E-01	-1.11E+00	1.15E-04	-3.17E+00	1.57E-27	-3.75E+00	7.97E-36	-2.34E-01	9.53E-01
ENSBTAG00000037523	5.47E-01	6.68E-01	3.06E+00	7.20E-20	2.25E+00	5.07E-11	1.46E+00	8.30E-05	6.04E-01	6.01E-01
ENSBTAG00000015642	-3.92E-02	1.00E+00	-1.65E-01	6.34E-01	-4.82E-01	2.59E-02	-6.68E-01	1.36E-03	1.85E-01	8.97E-01
ENSBTAG00000015635	1.48E-01	1.00E+00	1.02E+00	1.42E-03	-1.48E-01	9.46E-01	-1.36E+00	5.32E-05	3.41E-01	8.00E-01
ENSBTAG00000015632	-3.34E-02	1.00E+00	5.90E-01	4.40E-01	-1.08E-01	1.00E+00	-1.25E-02	1.00E+00	-1.31E+00	3.67E-01
ENSBTAG00000018028	-2.93E-01	1.00E+00	-1.54E+00	6.05E-02	-6.75E-01	4.62E-01	-2.45E-01	1.00E+00	2.73E-01	1.00E+00
ENSBTAG00000036395	0.00E+00	1.00E+00	0.00E+00	1.00E+00	-5.13E-15	1.00E+00	0.00E+00	1.00E+00	1.91E+00	8.00E-01
ENSBTAG00000029998	0.00E+00	1.00E+00	0.00E+00	1.00E+00	-5.13E-15	1.00E+00	0.00E+00	1.00E+00	0.00E+00	1.00E+00
ENSBTAG00000030062	0.00E+00	1.00E+00	0.00E+00	1.00E+00	-5.13E-15	1.00E+00	0.00E+00	1.00E+00	0.00E+00	1.00E+00
ENSBTAG00000007623	-5.16E-01	5.00E-01	-1.39E+00	1.56E-05	2.79E-02	1.00E+00	1.24E-01	1.00E+00	-8.90E-02	1.00E+00
ENSBTAG00000007620	-5.91E-02	1.00E+00	4.32E-01	7.85E-02	6.30E-01	5.22E-03	6.83E-01	2.62E-03	7.85E-02	1.00E+00
ENSBTAG00000007619	-5.21E-02	1.00E+00	1.64E-01	8.98E-01	1.47E+00	3.81E-07	9.92E-01	1.43E-03	-1.07E-01	1.00E+00

ENSBTAG00000017674	-2.23E-01	1.00E+00	-1.43E+00	8.06E-03	-1.67E+00	1.35E-03	-1.66E+00	1.72E-03	1.94E-01	1.00E+00
ENSBTAG00000009856	-1.77E-01	1.00E+00	-1.25E+00	4.80E-06	-7.54E-01	7.61E-03	-8.48E-01	2.76E-03	2.05E-01	9.93E-01
ENSBTAG00000014511	2.19E-02	1.00E+00	6.96E-02	1.00E+00	5.58E-01	5.37E-02	2.13E-01	6.56E-01	1.86E-01	1.00E+00
ENSBTAG00000046758	-3.21E+00	3.76E-01	1.99E+00	5.36E-02	7.54E-01	7.20E-01	-3.21E+00	1.01E-01	-3.21E+00	4.35E-01
ENSBTAG00000024492	1.84E+00	8.36E-02	-4.26E-01	8.88E-01	9.73E-02	1.00E+00	-2.64E-02	1.00E+00	-6.84E-01	9.45E-01
ENSBTAG00000013191	-3.99E-01	7.89E-01	-1.27E+00	1.75E-04	-1.80E+00	5.48E-08	-1.78E+00	1.04E-07	5.41E-02	1.00E+00
ENSBTAG00000014707	-1.10E+00	8.23E-01	-3.09E-01	1.00E+00	-8.12E-01	6.35E-01	-1.41E+00	2.82E-01	-1.46E+00	6.24E-01
ENSBTAG00000014705	-1.60E-01	1.00E+00	-1.61E+00	4.08E-04	-1.18E-01	1.00E+00	1.47E-01	1.00E+00	4.59E-01	7.74E-01
ENSBTAG00000014540	8.83E-02	1.00E+00	2.79E+00	2.84E-07	1.42E+00	2.17E-02	7.65E-01	3.50E-01	1.32E+00	2.49E-01
ENSBTAG00000014537	3.33E-01	9.46E-01	5.88E-01	1.97E-01	1.08E+00	4.08E-03	2.29E-01	8.19E-01	9.75E-01	1.10E-01
ENSBTAG00000013813	1.73E-01	1.00E+00	5.27E-01	7.62E-02	5.20E-01	8.03E-02	-1.03E-01	1.00E+00	2.69E-01	8.27E-01
ENSBTAG00000016528	-1.12E-01	1.00E+00	1.28E+00	2.48E-10	1.93E+00	5.78E-22	1.41E+00	2.88E-12	-1.26E-01	1.00E+00
ENSBTAG00000008739	-8.38E-01	7.88E-02	1.18E+00	2.29E-04	-2.12E+00	7.92E-11	-1.78E+00	5.21E-08	-9.69E-02	1.00E+00
ENSBTAG00000018730	3.04E-01	5.78E-01	1.29E+00	4.28E-11	6.06E-01	3.91E-03	2.83E-01	2.86E-01	-1.31E-02	1.00E+00
ENSBTAG00000018729	-1.55E-01	1.00E+00	3.72E-01	2.03E-01	5.28E-01	4.34E-02	2.64E-01	4.45E-01	1.84E-01	9.90E-01
ENSBTAG00000005722	3.42E-01	8.81E-01	6.57E-01	1.00E-01	6.44E-01	1.09E-01	1.11E+00	2.31E-03	9.02E-01	1.28E-01
ENSBTAG00000018725	1.60E-01	1.00E+00	-5.15E-01	4.43E-01	-4.17E-01	5.69E-01	1.06E-01	1.00E+00	1.57E-01	1.00E+00
ENSBTAG00000018724	-2.91E-01	8.23E-01	-7.27E-01	1.27E-02	-1.68E+00	7.33E-10	-1.94E+00	1.60E-12	-2.65E-01	8.61E-01
ENSBTAG00000018723	-1.14E+00	1.21E-01	-1.71E+00	5.13E-04	-2.14E+00	1.10E-05	-1.98E+00	5.67E-05	1.00E+00	2.61E-01
ENSBTAG00000018722	3.08E-01	6.62E-01	6.32E-01	6.60E-03	1.18E+00	4.08E-08	1.41E+00	7.41E-11	1.90E-01	9.37E-01
ENSBTAG00000039662	1.78E+00	8.37E-10	1.71E+00	4.30E-10	7.98E-01	6.07E-03	8.30E-02	1.00E+00	8.63E-01	3.78E-02
ENSBTAG00000018718	1.71E+00	2.70E-06	1.35E+00	6.54E-05	5.42E-01	1.81E-01	2.70E-02	1.00E+00	6.26E-01	4.31E-01
ENSBTAG00000043486	0.00E+00	1.00E+00	0.00E+00	1.00E+00	-5.13E-15	1.00E+00	0.00E+00	1.00E+00	0.00E+00	1.00E+00
ENSBTAG00000002478	-1.96E-01	9.60E-01	-7.76E-01	8.26E-04	-1.76E+00	2.43E-15	-2.13E+00	2.11E-21	-3.78E-01	5.24E-01
ENSBTAG00000002476	-1.23E-01	1.00E+00	-1.01E+00	6.98E-10	-1.07E+00	5.18E-11	-8.40E-01	4.14E-07	-4.05E-01	1.81E-01
ENSBTAG00000002472	-6.43E-01	1.13E-01	-2.13E+00	1.22E-14	-1.09E+00	3.51E-05	-1.19E-01	9.52E-01	7.11E-02	1.00E+00
ENSBTAG00000024496	-3.69E-01	1.00E+00	-7.97E-01	7.84E-01	-5.32E-01	9.37E-01	-1.83E+00	2.98E-01	-3.62E-01	1.00E+00
ENSBTAG00000024497	1.24E+00	5.55E-01	6.04E-01	7.20E-01	9.98E-01	3.47E-01	1.80E+00	3.19E-02	2.21E+00	5.87E-02
ENSBTAG00000009048	3.71E-01	7.02E-01	1.21E+00	1.39E-05	1.42E+00	2.30E-07	1.58E+00	1.09E-08	-1.38E-01	1.00E+00
ENSBTAG00000002408	-1.06E-01	1.00E+00	-1.57E-01	1.00E+00	-5.46E-01	3.55E-01	-5.72E-01	3.35E-01	-2.57E-03	1.00E+00
ENSBTAG00000005300	-3.85E-01	5.76E-01	-1.87E-01	7.09E-01	-6.45E-01	1.87E-02	-4.46E-01	1.45E-01	-1.18E-01	1.00E+00
ENSBTAG00000015606	-5.19E-02	1.00E+00	5.08E-01	7.67E-02	8.17E-01	1.56E-03	3.53E-02	1.00E+00	-6.80E-01	1.28E-01
ENSBTAG00000020552	2.29E-01	8.91E-01	6.84E-01	5.48E-03	1.00E+00	1.72E-05	8.57E-01	3.83E-04	2.09E-01	9.15E-01
ENSBTAG00000006175	-2.36E-01	8.23E-01	-2.74E-01	3.59E-01	8.60E-02	1.00E+00	2.04E-01	5.55E-01	1.21E-01	1.00E+00
ENSBTAG00000001788	1.94E-01	1.00E+00	2.45E-01	1.00E+00	2.02E-01	1.00E+00	1.48E+00	1.94E-01	-5.71E-01	1.00E+00

ENSBTAG00000016189	3.89E+00	1.42E-01	3.98E+00	2.58E-02	3.43E+00	7.35E-02	1.77E+00	4.97E-01	4.15E+00	1.33E-01
ENSBTAG00000013560	-8.28E-02	1.00E+00	6.48E-01	4.15E-02	1.08E+00	1.57E-04	9.87E-01	8.07E-04	4.14E-01	6.47E-01
ENSBTAG00000009726	-1.34E-01	1.00E+00	-3.40E-01	1.65E-01	9.30E-02	9.48E-01	-5.83E-02	1.00E+00	2.51E-01	7.71E-01
ENSBTAG00000009727	1.20E-01	1.00E+00	1.88E-01	5.18E-01	6.23E-01	1.58E-03	6.41E-01	1.32E-03	4.45E-01	2.23E-01
ENSBTAG00000019927	-5.07E-01	4.57E-01	-1.05E+00	7.12E-04	-1.40E+00	3.62E-06	-9.04E-01	4.43E-03	1.84E-02	1.00E+00
ENSBTAG00000019931	-1.91E+00	8.23E-01	7.51E-02	1.00E+00	-1.91E+00	4.28E-01	6.27E-01	9.94E-01	6.47E-03	1.00E+00
ENSBTAG00000017883	1.13E+00	1.23E-01	4.67E-02	1.00E+00	-1.78E+00	3.10E-03	-1.40E+00	2.08E-02	-4.45E-01	9.31E-01
ENSBTAG00000024791	-1.91E+00	8.23E-01	-1.91E+00	4.69E-01	-1.91E+00	4.28E-01	-1.91E+00	4.39E-01	-1.91E+00	8.00E-01
ENSBTAG00000038000	0.00E+00	1.00E+00	0.00E+00	1.00E+00	1.78E+00	4.79E-01	0.00E+00	1.00E+00	0.00E+00	1.00E+00
ENSBTAG00000040570	0.00E+00	1.00E+00	0.00E+00	1.00E+00	-5.13E-15	1.00E+00	0.00E+00	1.00E+00	0.00E+00	1.00E+00
ENSBTAG00000038222	0.00E+00	1.00E+00	0.00E+00	1.00E+00	-5.13E-15	1.00E+00	0.00E+00	1.00E+00	1.93E+00	8.00E-01
ENSBTAG00000012389	-1.91E+00	8.23E-01	-1.91E+00	4.69E-01	-1.91E+00	4.28E-01	-1.91E+00	4.39E-01	-1.91E+00	8.00E-01
ENSBTAG00000038264	0.00E+00	1.00E+00	1.99E+00	4.43E-01	1.78E+00	4.79E-01	0.00E+00	1.00E+00	0.00E+00	1.00E+00
ENSBTAG00000031507	1.92E+00	8.23E-01	0.00E+00	1.00E+00	1.78E+00	4.79E-01	1.76E+00	4.97E-01	0.00E+00	1.00E+00
ENSBTAG00000038261	0.00E+00	1.00E+00	1.98E+00	4.43E-01	-5.13E-15	1.00E+00	0.00E+00	1.00E+00	0.00E+00	1.00E+00
ENSBTAG00000040419	0.00E+00	1.00E+00	1.99E+00	4.43E-01	-5.13E-15	1.00E+00	0.00E+00	1.00E+00	1.91E+00	8.00E-01
ENSBTAG00000015600	0.00E+00	1.00E+00	0.00E+00	1.00E+00	-5.13E-15	1.00E+00	0.00E+00	1.00E+00	0.00E+00	1.00E+00
ENSBTAG00000001210	6.04E-03	1.00E+00	-1.91E+00	4.69E-01	-1.91E+00	4.28E-01	-1.91E+00	4.39E-01	-1.91E+00	8.00E-01
ENSBTAG00000046630	0.00E+00	1.00E+00	1.98E+00	4.43E-01	-5.13E-15	1.00E+00	1.76E+00	4.97E-01	0.00E+00	1.00E+00
ENSBTAG00000012158	-2.70E+00	5.76E-01	-2.70E+00	2.42E-01	-9.17E-01	8.09E-01	-1.66E-01	1.00E+00	1.80E-02	1.00E+00
ENSBTAG00000015373	5.37E-01	8.23E-01	-8.27E-01	2.05E-01	-6.58E-01	3.33E-01	-1.22E+00	4.19E-02	6.02E-01	7.77E-01
ENSBTAG00000004855	6.18E-02	1.00E+00	-6.00E-01	3.39E-03	-8.10E-01	3.94E-05	-6.92E-01	6.81E-04	-2.90E-01	6.13E-01
ENSBTAG00000002231	7.18E-01	8.23E-01	-3.64E-01	9.19E-01	-3.80E-01	8.75E-01	4.34E-01	7.63E-01	1.47E+00	1.56E-01
ENSBTAG00000044117	6.54E-01	7.57E-01	-6.99E-01	4.24E-01	-1.49E+00	3.75E-02	-1.79E-01	1.00E+00	1.79E+00	3.86E-03
ENSBTAG00000046799	0.00E+00	1.00E+00	0.00E+00	1.00E+00	-5.13E-15	1.00E+00	0.00E+00	1.00E+00	0.00E+00	1.00E+00
ENSBTAG00000024485	2.33E-01	8.23E-01	2.92E-01	2.87E-01	1.72E-02	1.00E+00	-2.29E-01	4.72E-01	2.54E-04	1.00E+00
ENSBTAG00000006925	2.07E-01	1.00E+00	4.50E-01	1.33E-01	3.90E-01	2.08E-01	6.07E-01	2.75E-02	1.91E-01	1.00E+00
ENSBTAG00000004358	8.60E-02	1.00E+00	-1.07E+00	2.38E-06	-1.51E+00	1.88E-11	-1.11E+00	9.90E-07	8.30E-02	1.00E+00
ENSBTAG00000043378	2.96E+00	4.51E-25	2.06E+00	7.75E-13	1.79E+00	4.67E-10	1.12E+00	3.25E-04	-1.01E+00	8.28E-02
ENSBTAG00000042180	2.86E+00	9.09E-21	2.05E+00	6.37E-12	1.94E+00	6.55E-11	1.32E+00	2.21E-05	-7.90E-01	2.05E-01
ENSBTAG00000042339	2.95E+00	2.69E-25	2.17E+00	1.27E-14	2.08E+00	1.05E-13	1.67E+00	5.62E-09	-8.23E-01	1.79E-01
ENSBTAG00000043268	3.11E+00	8.67E-28	2.42E+00	1.63E-17	2.18E+00	1.83E-14	1.83E+00	3.96E-10	-6.66E-01	4.34E-01
ENSBTAG00000042504	3.43E+00	8.15E-26	2.87E+00	6.26E-19	2.82E+00	1.40E-18	2.05E+00	7.05E-10	-4.24E-01	8.18E-01
ENSBTAG00000043432	3.41E+00	4.45E-32	2.71E+00	9.96E-21	2.59E+00	2.82E-19	1.94E+00	1.11E-10	-4.30E-01	8.00E-01
ENSBTAG00000042217	3.30E+00	9.41E-35	2.62E+00	1.04E-22	2.50E+00	5.19E-21	1.82E+00	3.67E-11	-3.27E-01	8.45E-01

ENSBTAG00000042974	3.28E+00	4.10E-38	2.63E+00	3.25E-25	2.31E+00	5.14E-20	2.01E+00	5.27E-15	-2.45E-01	9.73E-01
ENSBTAG00000042931	4.24E+00	9.87E-19	3.39E+00	1.45E-11	3.04E+00	2.68E-09	2.83E+00	8.89E-08	4.96E-01	1.00E+00
ENSBTAG00000043207	4.07E+00	3.71E-25	3.46E+00	3.08E-18	3.10E+00	1.11E-14	2.45E+00	9.78E-09	1.01E+00	3.65E-01
ENSBTAG00000004361	1.12E+00	4.66E-03	7.33E-01	4.17E-02	-3.54E-01	4.74E-01	-5.17E-01	2.34E-01	2.34E-01	1.00E+00
ENSBTAG00000004362	9.21E-02	1.00E+00	-3.16E-01	1.08E-01	-9.65E-01	5.94E-09	-1.17E+00	1.71E-12	1.20E-01	1.00E+00
ENSBTAG00000042396	0.00E+00	1.00E+00	2.79E+00	2.10E-01	1.78E+00	4.79E-01	1.76E+00	4.97E-01	0.00E+00	1.00E+00
ENSBTAG00000027809	2.32E-02	1.00E+00	9.86E-01	2.77E-04	1.09E+00	4.09E-05	8.77E-01	1.60E-03	-2.87E-01	8.38E-01
ENSBTAG00000038189	-1.67E+00	7.80E-01	3.84E-01	1.00E+00	1.45E+00	1.60E-01	1.52E+00	1.39E-01	-8.76E-01	1.00E+00
ENSBTAG00000024482	3.40E-01	7.74E-01	7.38E-01	1.04E-02	3.15E-01	4.15E-01	4.31E-01	2.05E-01	6.66E-02	1.00E+00
ENSBTAG00000002204	0.00E+00	1.00E+00	0.00E+00	1.00E+00	-5.13E-15	1.00E+00	0.00E+00	1.00E+00	0.00E+00	1.00E+00
ENSBTAG00000047352	0.00E+00	1.00E+00	0.00E+00	1.00E+00	-5.13E-15	1.00E+00	0.00E+00	1.00E+00	0.00E+00	1.00E+00
ENSBTAG00000001107	9.89E-01	6.29E-03	2.74E+00	6.36E-21	1.56E+00	5.64E-08	6.25E-01	5.79E-02	-2.22E-03	1.00E+00
ENSBTAG00000001109	-6.38E-02	1.00E+00	-3.55E-01	2.69E-01	-3.28E-01	3.22E-01	-5.96E-01	3.36E-02	-3.26E-01	7.42E-01
ENSBTAG00000015650	9.25E-01	9.45E-01	1.02E+00	5.15E-01	1.31E+00	2.95E-01	1.43E+00	2.36E-01	1.32E+00	7.08E-01
ENSBTAG00000017691	8.54E-02	1.00E+00	-3.87E-01	6.00E-01	-5.07E-01	4.18E-01	2.23E-01	9.03E-01	5.30E-02	1.00E+00
ENSBTAG00000011706	-6.20E-01	9.59E-01	-1.26E-01	1.00E+00	-9.22E-01	3.36E-01	7.00E-01	4.39E-01	-7.12E-01	8.52E-01
ENSBTAG00000028880	0.00E+00	1.00E+00	0.00E+00	1.00E+00	-5.13E-15	1.00E+00	0.00E+00	1.00E+00	0.00E+00	1.00E+00
ENSBTAG00000042851	0.00E+00	1.00E+00	0.00E+00	1.00E+00	-5.13E-15	1.00E+00	0.00E+00	1.00E+00	0.00E+00	1.00E+00
ENSBTAG00000031385	-6.16E-02	1.00E+00	-4.21E-01	5.86E-02	-4.65E-01	3.21E-02	-7.12E-02	1.00E+00	-9.91E-02	1.00E+00
ENSBTAG00000044308	0.00E+00	1.00E+00	0.00E+00	1.00E+00	-5.13E-15	1.00E+00	0.00E+00	1.00E+00	0.00E+00	1.00E+00
ENSBTAG00000018303	2.10E+00	1.87E-03	5.75E+00	1.28E-24	3.78E+00	2.84E-12	2.31E+00	4.58E-05	-4.20E-01	1.00E+00
ENSBTAG00000012953	-2.64E-01	1.00E+00	-1.25E-01	1.00E+00	-1.36E+00	6.27E-02	-2.37E-01	9.93E-01	-5.02E-02	1.00E+00
ENSBTAG00000029898	0.00E+00	1.00E+00	0.00E+00	1.00E+00	-5.13E-15	1.00E+00	0.00E+00	1.00E+00	0.00E+00	1.00E+00
ENSBTAG00000013715	-7.32E-01	1.00E+00	-6.61E-01	7.55E-01	1.74E-01	1.00E+00	-2.14E-02	1.00E+00	-1.63E+00	6.03E-01
ENSBTAG00000038238	1.07E-01	1.00E+00	1.03E+00	1.81E-03	1.93E+00	6.73E-10	1.85E+00	4.65E-09	-3.23E-01	8.46E-01
ENSBTAG00000000854	-7.44E-01	3.75E-01	-1.56E+00	1.73E-04	-1.78E+00	1.47E-05	-2.95E+00	1.90E-12	1.72E-01	1.00E+00
ENSBTAG00000046172	-1.97E-01	9.97E-01	-2.50E-01	4.69E-01	-5.02E-01	6.26E-02	-7.49E-01	3.17E-03	-4.42E-01	4.34E-01
ENSBTAG00000015537	5.43E-01	4.03E-01	1.36E+00	4.31E-06	1.06E+00	4.62E-04	8.61E-01	7.09E-03	-4.21E-01	7.33E-01
ENSBTAG00000004941	-1.31E+00	9.32E-01	-3.22E+00	1.25E-01	-3.22E+00	9.40E-02	-3.22E+00	9.87E-02	-3.22E+00	4.34E-01
ENSBTAG00000010718	6.15E-01	3.30E-01	-1.57E+00	3.22E-06	-1.76E+00	1.17E-07	-2.67E-02	1.00E+00	1.37E+00	5.67E-04
ENSBTAG00000010719	0.00E+00	1.00E+00	0.00E+00	1.00E+00	-5.13E-15	1.00E+00	0.00E+00	1.00E+00	0.00E+00	1.00E+00
ENSBTAG00000013999	-7.18E-02	1.00E+00	-1.06E-01	1.00E+00	7.92E-01	3.74E-02	1.72E+00	5.99E-07	4.02E-01	8.00E-01
ENSBTAG00000014006	8.28E-02	1.00E+00	-6.76E-01	9.43E-03	-5.35E-02	1.00E+00	1.12E-01	9.43E-01	1.04E-01	1.00E+00
ENSBTAG00000015026	1.48E-02	1.00E+00	3.28E-01	4.26E-01	9.15E-01	1.43E-03	9.10E-01	1.86E-03	-1.17E-01	1.00E+00
ENSBTAG00000004059	3.05E-02	1.00E+00	1.39E+00	1.92E-07	1.39E+00	1.45E-07	1.45E+00	5.30E-08	2.39E-01	9.17E-01

ENSBTAG00000002164	2.22E+00	4.70E-01	5.14E+00	1.56E-07	3.66E+00	1.37E-03	4.07E+00	2.01E-04	2.78E+00	2.52E-01
ENSBTAG00000002167	-1.66E+00	7.81E-01	-2.77E-01	1.00E+00	-5.20E-01	9.47E-01	-3.58E+00	4.95E-02	-1.67E+00	7.81E-01
ENSBTAG00000002407	1.21E-02	1.00E+00	2.27E-01	1.00E+00	2.31E-01	1.00E+00	6.70E-01	4.63E-01	-4.71E-01	1.00E+00
ENSBTAG00000021492	2.72E+00	5.76E-01	0.00E+00	1.00E+00	1.78E+00	4.79E-01	0.00E+00	1.00E+00	0.00E+00	1.00E+00
ENSBTAG00000035230	7.49E-02	1.00E+00	9.57E-01	1.58E-02	7.88E-01	5.62E-02	6.45E-01	1.50E-01	5.43E-01	6.18E-01
ENSBTAG00000037813	0.00E+00	1.00E+00	0.00E+00	1.00E+00	-5.13E-15	1.00E+00	0.00E+00	1.00E+00	0.00E+00	1.00E+00
ENSBTAG00000035226	3.99E-01	6.06E-01	4.97E-01	1.21E-01	6.96E-01	1.77E-02	8.91E-01	1.87E-03	5.11E-01	4.32E-01
ENSBTAG00000042838	0.00E+00	1.00E+00	0.00E+00	1.00E+00	-5.13E-15	1.00E+00	0.00E+00	1.00E+00	1.93E+00	8.00E-01
ENSBTAG00000008082	4.29E-01	5.26E-01	6.78E-01	1.77E-02	3.77E-01	2.65E-01	6.71E-01	2.13E-02	4.99E-02	1.00E+00
ENSBTAG00000014191	-6.71E-02	1.00E+00	-3.51E-01	2.55E-01	-9.04E-01	2.72E-04	-4.52E-01	1.19E-01	8.90E-01	7.81E-03
ENSBTAG00000044507	0.00E+00	1.00E+00	0.00E+00	1.00E+00	-5.13E-15	1.00E+00	0.00E+00	1.00E+00	0.00E+00	1.00E+00
ENSBTAG00000003992	1.43E+00	7.56E-01	1.28E+00	4.43E-01	-1.60E-01	1.00E+00	1.93E+00	1.14E-01	1.21E+00	8.20E-01
ENSBTAG00000046502	4.26E-01	5.49E-01	2.29E-01	6.18E-01	-4.40E-01	1.88E-01	-6.88E-02	1.00E+00	2.68E-01	8.46E-01
ENSBTAG00000046930	0.00E+00	1.00E+00	0.00E+00	1.00E+00	-5.13E-15	1.00E+00	0.00E+00	1.00E+00	0.00E+00	1.00E+00
ENSBTAG00000009169	2.46E-01	9.43E-01	-8.14E-01	7.72E-03	3.42E-01	3.51E-01	9.84E-01	3.58E-04	1.23E-01	1.00E+00
ENSBTAG00000006260	2.24E-01	1.00E+00	4.43E-02	1.00E+00	4.90E-01	4.83E-01	2.20E-01	9.69E-01	3.77E-01	1.00E+00
ENSBTAG00000020458	2.55E-01	8.78E-01	-3.75E-01	2.68E-01	7.82E-02	1.00E+00	3.44E-01	3.09E-01	1.93E-02	1.00E+00
ENSBTAG00000009924	-9.28E-02	1.00E+00	4.27E-01	4.69E-01	3.88E-01	5.21E-01	2.11E-01	9.04E-01	2.73E-01	1.00E+00
ENSBTAG00000039764	2.11E+00	1.42E-09	2.27E+00	5.14E-12	1.84E+00	2.12E-08	1.45E+00	1.66E-05	-1.40E-01	1.00E+00
ENSBTAG00000020887	3.09E-02	1.00E+00	3.76E-01	8.55E-01	2.16E-02	1.00E+00	1.01E+00	1.88E-01	-2.91E-01	1.00E+00
ENSBTAG00000046243	0.00E+00	1.00E+00	0.00E+00	1.00E+00	-5.13E-15	1.00E+00	0.00E+00	1.00E+00	0.00E+00	1.00E+00
ENSBTAG00000027426	1.37E-01	1.00E+00	2.42E-01	6.62E-01	7.34E-01	2.54E-02	9.59E-01	2.63E-03	-5.26E-01	5.03E-01
ENSBTAG00000013631	-8.54E-02	1.00E+00	-8.21E-01	2.08E-01	-1.00E+00	1.00E-01	-1.19E-01	1.00E+00	-7.54E-01	6.43E-01
ENSBTAG00000021432	-7.92E-01	1.00E+00	-2.71E+00	2.42E-01	3.49E-01	1.00E+00	6.96E-01	8.54E-01	1.43E-03	1.00E+00
ENSBTAG00000000772	0.00E+00	1.00E+00	0.00E+00	1.00E+00	-5.13E-15	1.00E+00	0.00E+00	1.00E+00	0.00E+00	1.00E+00
ENSBTAG00000018220	-1.23E-01	1.00E+00	-1.18E+00	7.47E-03	-2.51E+00	4.02E-09	-2.84E+00	5.82E-11	-1.26E-01	1.00E+00
ENSBTAG00000009091	3.45E-01	1.00E+00	6.42E-01	3.53E-01	8.65E-01	1.56E-01	5.12E-01	5.03E-01	-7.47E-02	1.00E+00
ENSBTAG00000004305	3.13E+00	9.09E-24	8.65E-01	6.07E-03	1.37E+00	5.07E-06	1.21E+00	8.14E-05	8.51E-01	7.36E-02
ENSBTAG00000026481	7.25E-01	8.23E-01	-3.46E-01	9.60E-01	-4.41E-01	8.31E-01	-6.37E-01	6.35E-01	4.60E-01	1.00E+00
ENSBTAG00000002266	-7.80E-01	4.90E-02	-6.44E-01	3.58E-02	4.94E-02	1.00E+00	3.77E-02	1.00E+00	-1.87E-01	1.00E+00
ENSBTAG00000019821	-6.44E-03	1.00E+00	5.34E-01	5.02E-03	7.06E-01	1.16E-04	6.64E-01	3.91E-04	7.36E-02	1.00E+00
ENSBTAG00000031238	5.80E-01	9.42E-01	2.01E+00	3.39E-04	4.89E-01	6.79E-01	-4.41E-01	8.20E-01	-7.36E-01	8.76E-01
ENSBTAG00000011966	-4.61E-02	1.00E+00	-2.63E-01	4.44E-01	-6.63E-01	9.18E-03	6.79E-02	1.00E+00	5.99E-01	1.53E-01
ENSBTAG00000000793	-4.75E-02	1.00E+00	5.37E-01	6.73E-01	1.26E+00	1.13E-01	2.17E+00	2.92E-03	1.52E-01	1.00E+00
ENSBTAG00000000795	-8.14E-03	1.00E+00	3.72E-01	2.33E-01	-8.93E-02	1.00E+00	-6.98E-01	1.07E-02	5.45E-01	2.75E-01

ENSBTAG00000028988	-1.91E+00	8.23E-01	-1.91E+00	4.69E-01	-1.91E+00	4.28E-01	-1.91E+00	4.39E-01	-1.91E+00	8.00E-01
ENSBTAG00000008003	3.29E-01	4.82E-01	1.22E-01	8.18E-01	1.27E-01	7.86E-01	1.43E-01	7.25E-01	-1.49E-01	1.00E+00
ENSBTAG00000008004	-1.54E-01	1.00E+00	-1.12E-01	1.00E+00	-1.53E-01	9.42E-01	4.12E-01	3.37E-01	-1.98E-01	1.00E+00
ENSBTAG00000045582	7.36E-02	1.00E+00	1.87E-01	4.90E-01	1.67E-01	5.68E-01	1.93E-01	4.93E-01	-4.34E-01	2.11E-01
ENSBTAG00000047395	1.91E+00	8.23E-01	0.00E+00	1.00E+00	-5.13E-15	1.00E+00	0.00E+00	1.00E+00	0.00E+00	1.00E+00
ENSBTAG00000019630	9.59E-01	6.41E-03	3.19E+00	3.48E-30	2.91E+00	6.29E-26	2.09E+00	2.68E-14	-3.48E-01	8.00E-01
ENSBTAG00000042393	0.00E+00	1.00E+00	0.00E+00	1.00E+00	-5.13E-15	1.00E+00	0.00E+00	1.00E+00	0.00E+00	1.00E+00
ENSBTAG00000035836	7.26E-01	1.40E-01	-7.40E-01	3.34E-02	-1.40E+00	1.35E-05	-5.33E-01	1.62E-01	8.05E-01	1.20E-01
ENSBTAG00000016369	8.08E-02	1.00E+00	-9.69E-01	1.23E-04	-4.61E-01	1.06E-01	-2.86E-01	4.24E-01	-1.44E-01	1.00E+00
ENSBTAG00000046091	0.00E+00	1.00E+00	0.00E+00	1.00E+00	-5.13E-15	1.00E+00	0.00E+00	1.00E+00	0.00E+00	1.00E+00
ENSBTAG00000029119	0.00E+00	1.00E+00	0.00E+00	1.00E+00	2.55E+00	2.54E-01	0.00E+00	1.00E+00	0.00E+00	1.00E+00
ENSBTAG00000038079	-6.11E-02	1.00E+00	1.00E-02	1.00E+00	6.87E-01	1.05E-01	6.05E-01	1.81E-01	-1.30E-01	1.00E+00
ENSBTAG00000019264	6.22E-01	3.16E-01	2.36E+00	6.13E-14	1.79E+00	1.12E-08	2.32E+00	1.79E-13	7.24E-01	2.41E-01
ENSBTAG00000014548	7.85E-01	1.54E-01	1.50E+00	7.61E-06	4.93E-01	2.50E-01	5.43E-01	2.00E-01	2.52E-02	1.00E+00
ENSBTAG00000000023	5.12E-01	1.77E-01	9.19E-01	4.92E-05	9.87E-01	9.11E-06	7.42E-01	1.57E-03	1.31E-01	1.00E+00
ENSBTAG00000011285	-1.14E-01	1.00E+00	-7.22E-01	4.23E-03	-6.71E-01	8.38E-03	-5.57E-01	3.94E-02	-2.27E-01	8.85E-01
ENSBTAG00000004492	1.82E-01	1.00E+00	-8.40E-01	1.11E-02	6.10E-01	6.97E-02	5.56E-01	1.15E-01	-1.92E-01	1.00E+00
ENSBTAG00000014249	3.07E-01	7.72E-01	2.12E+00	2.66E-18	1.72E+00	1.03E-12	1.30E+00	1.30E-07	2.04E-01	9.56E-01
ENSBTAG00000018211	8.02E-01	1.00E+00	7.71E-02	1.00E+00	-1.91E+00	4.28E-01	-1.48E-01	1.00E+00	-1.91E+00	8.00E-01
ENSBTAG00000045729	-1.01E-01	1.00E+00	-4.15E-01	1.34E-01	-1.19E+00	5.31E-07	-9.84E-01	4.56E-05	1.72E-01	1.00E+00
ENSBTAG00000015235	-2.03E-01	1.00E+00	-5.08E-01	4.30E-01	-1.25E+00	1.08E-02	-7.94E-01	1.40E-01	-5.60E-01	7.71E-01
ENSBTAG00000011932	1.33E-01	1.00E+00	2.31E-01	5.31E-01	-1.95E-01	6.45E-01	-3.31E-01	3.01E-01	6.80E-01	7.82E-02
ENSBTAG00000011943	1.42E-01	1.00E+00	1.01E+00	1.66E-05	9.28E-01	7.89E-05	7.38E-01	2.73E-03	8.12E-02	1.00E+00
ENSBTAG00000047559	0.00E+00	1.00E+00	0.00E+00	1.00E+00	-5.13E-15	1.00E+00	0.00E+00	1.00E+00	0.00E+00	1.00E+00
ENSBTAG00000011946	2.70E-01	6.72E-01	7.89E-01	5.87E-05	5.55E-01	6.80E-03	4.72E-01	2.93E-02	-1.71E-01	9.25E-01
ENSBTAG00000004176	4.29E-01	1.00E+00	1.83E+00	1.36E-02	7.16E-01	5.39E-01	-1.11E+00	4.25E-01	3.26E-01	1.00E+00
ENSBTAG00000014127	5.20E+00	7.17E-06	4.60E+00	8.48E-06	1.30E+00	2.66E-01	4.50E+00	1.55E-05	-9.21E-01	8.40E-01
ENSBTAG00000013298	7.89E-01	2.14E-02	2.84E-01	4.52E-01	6.14E-02	1.00E+00	6.19E-02	1.00E+00	-5.02E-01	4.34E-01
ENSBTAG00000004407	-1.39E-01	1.00E+00	1.01E-01	1.00E+00	-5.95E-01	1.79E-01	-4.09E-01	4.38E-01	-2.03E-01	1.00E+00
ENSBTAG00000042774	0.00E+00	1.00E+00	0.00E+00	1.00E+00	-5.13E-15	1.00E+00	0.00E+00	1.00E+00	0.00E+00	1.00E+00
ENSBTAG00000024449	4.05E-01	9.63E-01	-2.22E-01	9.47E-01	-6.13E-01	3.38E-01	-2.20E-01	9.53E-01	-9.57E-01	3.89E-01
ENSBTAG00000021553	4.92E-01	7.44E-01	2.58E-01	7.78E-01	-7.42E-01	1.28E-01	3.33E-01	6.01E-01	9.80E-02	1.00E+00
ENSBTAG00000013166	-2.67E-01	8.47E-01	-9.57E-01	3.32E-04	-9.74E-01	2.29E-04	-5.15E-01	8.72E-02	-1.06E-01	1.00E+00
ENSBTAG00000013726	-2.46E-01	8.47E-01	-1.07E+00	7.84E-06	-1.47E+00	4.36E-10	-1.63E+00	6.36E-12	-4.95E-02	1.00E+00
ENSBTAG00000008756	1.95E+00	2.62E-02	1.97E+00	3.68E-03	3.94E-01	8.07E-01	1.59E+00	2.59E-02	8.98E-01	6.55E-01

ENSBTAG00000014992	5.81E-01	9.02E-01	3.24E-01	8.98E-01	-7.77E-01	3.99E-01	-2.28E+00	5.44E-03	1.18E+00	3.49E-01
ENSBTAG00000006574	-1.57E-01	1.00E+00	3.94E-01	1.95E-01	6.26E-01	1.73E-02	6.19E-01	2.13E-02	-1.14E-01	1.00E+00
ENSBTAG00000003016	9.82E-02	1.00E+00	-6.86E-01	6.63E-02	-6.03E-01	1.11E-01	-8.78E-01	1.43E-02	-8.07E-02	1.00E+00
ENSBTAG00000047073	8.01E-02	1.00E+00	-5.54E-01	4.64E-01	-1.54E+00	9.44E-03	-1.37E+00	2.42E-02	2.96E-01	1.00E+00
ENSBTAG00000004789	-3.88E-01	1.00E+00	-2.62E-02	1.00E+00	-1.16E-01	1.00E+00	-2.35E-02	1.00E+00	-1.49E+00	1.74E-01
ENSBTAG00000004790	-2.82E-01	8.65E-01	-1.37E+00	1.72E-06	-8.68E-01	3.01E-03	-4.46E-01	2.00E-01	-1.72E-01	1.00E+00
ENSBTAG00000011772	-7.63E-02	1.00E+00	-7.91E-01	6.37E-03	1.12E-01	9.84E-01	1.72E-01	7.98E-01	4.16E-01	6.01E-01
ENSBTAG00000033994	0.00E+00	1.00E+00	0.00E+00	1.00E+00	-5.13E-15	1.00E+00	0.00E+00	1.00E+00	0.00E+00	1.00E+00
ENSBTAG00000047648	2.92E-01	8.23E-01	-2.42E-02	1.00E+00	-2.03E-01	7.03E-01	-1.34E+00	7.49E-06	5.52E-01	3.27E-01
ENSBTAG00000010978	1.41E-01	1.00E+00	-8.90E-03	1.00E+00	1.45E+00	3.58E-06	1.52E+00	1.49E-06	5.04E-01	5.62E-01
ENSBTAG00000001181	3.37E-01	6.68E-01	8.37E-01	7.08E-04	1.38E+00	4.38E-09	1.28E+00	6.89E-08	-1.10E-01	1.00E+00
ENSBTAG00000046070	0.00E+00	1.00E+00	0.00E+00	1.00E+00	-5.13E-15	1.00E+00	0.00E+00	1.00E+00	0.00E+00	1.00E+00
ENSBTAG00000003702	4.16E-01	2.14E-01	2.60E-01	3.02E-01	3.01E-01	2.01E-01	-3.61E-02	1.00E+00	-6.13E-02	1.00E+00
ENSBTAG00000003701	8.14E-01	9.19E-01	3.42E-01	1.00E+00	2.88E-01	1.00E+00	6.15E-01	7.41E-01	1.94E-02	1.00E+00
ENSBTAG00000015974	1.09E+00	3.48E-01	4.59E+00	1.32E-13	3.24E+00	4.67E-08	2.64E+00	9.31E-06	-1.85E-01	1.00E+00
ENSBTAG00000010016	3.12E-01	1.00E+00	2.53E-01	1.00E+00	7.80E-01	4.44E-01	-8.26E-01	5.57E-01	-3.92E-01	1.00E+00
ENSBTAG00000024443	-1.04E-01	1.00E+00	-4.88E-01	4.28E-02	-6.17E-02	1.00E+00	-2.82E-01	3.40E-01	-7.74E-02	1.00E+00
ENSBTAG00000011340	-3.75E-03	1.00E+00	-1.20E+00	2.20E-03	-4.11E-01	4.25E-01	-1.99E-01	8.72E-01	-3.97E-02	1.00E+00
ENSBTAG00000044819	0.00E+00	1.00E+00	0.00E+00	1.00E+00	-5.13E-15	1.00E+00	0.00E+00	1.00E+00	0.00E+00	1.00E+00
ENSBTAG00000011982	2.40E+00	2.59E-12	2.83E+00	7.51E-18	1.36E+00	5.78E-05	6.07E-01	1.37E-01	-1.76E-01	1.00E+00
ENSBTAG00000021166	3.19E+00	8.77E-07	4.13E+00	2.63E-14	1.34E+00	1.19E-01	9.59E-01	3.73E-01	-5.42E-01	1.00E+00
ENSBTAG00000008545	4.08E+00	1.90E-26	3.92E+00	8.99E-26	2.35E+00	4.27E-11	1.48E+00	4.64E-05	-1.02E+00	7.36E-02
ENSBTAG00000000759	-4.73E-02	1.00E+00	-5.86E-01	1.87E-02	-8.10E-01	6.07E-04	-4.62E-01	8.41E-02	1.71E-02	1.00E+00
ENSBTAG00000000758	-1.74E-01	1.00E+00	-3.99E-01	4.43E-01	9.32E-02	1.00E+00	-9.03E-02	1.00E+00	-2.73E-01	1.00E+00
ENSBTAG00000000754	-3.45E-01	2.87E-01	-1.14E+00	1.80E-11	-1.69E+00	8.50E-24	-1.72E+00	1.55E-24	-3.46E-02	1.00E+00
ENSBTAG00000043028	0.00E+00	1.00E+00	0.00E+00	1.00E+00	-5.13E-15	1.00E+00	0.00E+00	1.00E+00	0.00E+00	1.00E+00
ENSBTAG00000018142	-1.02E+00	4.46E-04	-3.85E+00	9.75E-40	-5.24E+00	6.66E-59	-3.45E+00	8.38E-36	-1.26E-01	1.00E+00
ENSBTAG00000018548	-8.82E-02	1.00E+00	-1.68E+00	8.66E-09	-2.90E+00	1.21E-22	-2.14E+00	2.39E-13	8.49E-02	1.00E+00
ENSBTAG00000005137	6.48E-01	2.31E-01	4.50E-01	2.42E-01	4.04E-01	3.14E-01	9.89E-01	2.12E-03	4.65E-01	6.01E-01
ENSBTAG00000045009	0.00E+00	1.00E+00	0.00E+00	1.00E+00	-5.13E-15	1.00E+00	0.00E+00	1.00E+00	0.00E+00	1.00E+00
ENSBTAG00000009618	5.24E-01	1.00E+00	-9.05E-01	3.63E-01	-1.46E+00	8.66E-02	-7.12E-01	4.97E-01	-1.29E+00	4.89E-01
ENSBTAG00000010909	7.26E-01	1.74E-01	1.35E+00	3.20E-05	4.29E-01	3.04E-01	9.47E-02	1.00E+00	7.61E-01	2.01E-01
ENSBTAG00000024453	1.91E+00	8.23E-01	4.44E+00	6.74E-03	3.97E+00	2.23E-02	1.77E+00	4.97E-01	3.22E+00	4.34E-01
ENSBTAG00000012020	1.34E+00	3.55E-03	1.02E+00	1.24E-02	2.85E-01	7.10E-01	-8.85E-02	1.00E+00	-2.17E-01	1.00E+00
ENSBTAG00000007110	-4.12E-01	4.84E-01	-2.06E+00	1.54E-16	-9.36E-01	1.98E-04	-6.71E-01	1.23E-02	-2.28E-01	8.97E-01

ENSBTAG00000008710	4.91E-01	6.31E-01	1.26E+00	3.00E-04	-8.36E-01	2.99E-02	-8.16E-01	3.90E-02	9.54E-01	8.59E-02
ENSBTAG00000042538	0.00E+00	1.00E+00	0.00E+00	1.00E+00	-5.13E-15	1.00E+00	0.00E+00	1.00E+00	0.00E+00	1.00E+00
ENSBTAG00000029007	0.00E+00	1.00E+00	0.00E+00	1.00E+00	-5.13E-15	1.00E+00	0.00E+00	1.00E+00	1.93E+00	8.00E-01
ENSBTAG00000043319	0.00E+00	1.00E+00	0.00E+00	1.00E+00	-5.13E-15	1.00E+00	0.00E+00	1.00E+00	0.00E+00	1.00E+00
ENSBTAG00000010850	-1.96E+00	2.56E-01	1.36E+00	6.60E-02	3.26E-01	9.52E-01	1.03E+00	2.13E-01	1.54E-01	1.00E+00
ENSBTAG00000046723	5.49E-01	8.25E-01	1.43E+00	4.24E-03	1.50E+00	1.99E-03	1.74E+00	2.62E-04	-9.03E-02	1.00E+00
ENSBTAG00000020886	-5.39E-03	1.00E+00	1.19E-01	8.28E-01	7.84E-01	5.50E-05	8.07E-01	4.03E-05	-3.01E-02	1.00E+00
ENSBTAG00000002849	-3.75E-01	1.00E+00	-2.58E+00	1.02E-07	-4.93E+00	6.86E-21	-2.71E+00	2.74E-08	1.32E-01	1.00E+00
ENSBTAG00000002848	-1.01E-01	1.00E+00	-1.28E+00	5.93E-04	1.80E-01	8.83E-01	3.25E-01	5.30E-01	5.40E-02	1.00E+00
ENSBTAG00000002846	-2.13E-01	9.25E-01	-5.75E-01	2.09E-02	-5.59E-01	2.42E-02	-8.00E-01	8.22E-04	3.96E-02	1.00E+00
ENSBTAG00000015086	1.76E-01	1.00E+00	1.26E-01	8.78E-01	-4.85E-01	6.07E-02	-8.36E-01	4.67E-04	-3.83E-01	5.32E-01
ENSBTAG00000047766	1.86E+00	1.63E-02	2.82E+00	3.83E-06	1.40E+00	2.83E-02	-4.22E-01	7.22E-01	-6.94E-01	8.00E-01
ENSBTAG00000016542	-1.60E-01	1.00E+00	2.36E-01	7.41E-01	-4.24E-01	3.47E-01	-1.04E+00	3.49E-03	-4.98E-01	6.09E-01
ENSBTAG00000016541	-6.13E-01	6.85E-01	-1.53E+00	2.95E-03	-7.63E-01	1.54E-01	-5.64E-02	1.00E+00	-8.96E-02	1.00E+00
ENSBTAG00000029854	0.00E+00	1.00E+00	0.00E+00	1.00E+00	-5.13E-15	1.00E+00	0.00E+00	1.00E+00	0.00E+00	1.00E+00
ENSBTAG00000044497	0.00E+00	1.00E+00	0.00E+00	1.00E+00	-5.13E-15	1.00E+00	0.00E+00	1.00E+00	0.00E+00	1.00E+00
ENSBTAG00000042659	0.00E+00	1.00E+00	0.00E+00	1.00E+00	-5.13E-15	1.00E+00	0.00E+00	1.00E+00	0.00E+00	1.00E+00
ENSBTAG00000001173	9.93E-02	1.00E+00	2.62E-01	2.92E-01	6.00E-01	2.09E-03	8.11E-01	2.04E-05	1.62E-01	9.43E-01
ENSBTAG00000010562	-2.51E-01	1.00E+00	7.84E-01	3.82E-02	1.23E-01	1.00E+00	3.68E-01	4.76E-01	-4.26E-01	8.00E-01
ENSBTAG00000029765	2.92E+00	3.32E-03	5.14E+00	1.72E-15	2.29E+00	1.33E-02	1.67E+00	1.35E-01	1.33E+00	7.14E-01
ENSBTAG00000029942	4.15E-01	9.59E-01	2.22E+00	3.97E-08	-9.47E-02	1.00E+00	-6.88E-01	3.39E-01	-1.08E+00	4.26E-01
ENSBTAG00000005397	1.31E+00	2.99E-08	2.45E+00	5.55E-28	2.20E+00	2.09E-23	1.67E+00	4.58E-14	3.79E-01	5.13E-01
ENSBTAG00000036365	1.92E+00	8.23E-01	3.30E+00	1.02E-01	-5.13E-15	1.00E+00	0.00E+00	1.00E+00	0.00E+00	1.00E+00
ENSBTAG00000036430	0.00E+00	1.00E+00	0.00E+00	1.00E+00	-5.13E-15	1.00E+00	1.76E+00	4.97E-01	0.00E+00	1.00E+00
ENSBTAG00000030910	-8.71E-01	1.00E+00	-1.60E+00	4.08E-01	-1.80E+00	2.96E-01	-1.83E+00	2.99E-01	-3.59E+00	3.09E-01
ENSBTAG00000000070	2.47E-01	7.56E-01	-2.93E-01	2.26E-01	-1.00E+00	1.86E-07	-9.66E-01	6.92E-07	3.91E-01	3.52E-01
ENSBTAG00000038171	1.23E+00	1.32E-03	1.59E+00	8.34E-07	1.70E+00	1.09E-07	1.74E+00	7.91E-08	1.28E+00	2.57E-03
ENSBTAG00000007860	6.05E-01	7.86E-01	1.31E+00	1.08E-02	3.53E-01	7.28E-01	1.20E-01	1.00E+00	3.36E-01	1.00E+00
ENSBTAG00000002291	1.16E-01	1.00E+00	1.47E+00	4.36E-08	9.99E-01	3.05E-04	1.11E+00	6.64E-05	2.53E-01	9.06E-01
ENSBTAG00000008944	2.30E-01	1.00E+00	1.65E+00	3.91E-05	1.46E+00	2.92E-04	1.05E-01	1.00E+00	6.17E-01	6.01E-01
ENSBTAG00000044926	0.00E+00	1.00E+00	0.00E+00	1.00E+00	-5.13E-15	1.00E+00	0.00E+00	1.00E+00	0.00E+00	1.00E+00
ENSBTAG00000012433	2.17E-01	1.00E+00	-6.35E-01	1.10E-01	-5.92E-01	1.39E-01	2.69E-01	6.52E-01	3.17E-02	1.00E+00
ENSBTAG00000037661	2.81E-01	8.38E-01	-3.45E-01	3.66E-01	-1.71E-01	8.02E-01	3.94E-01	2.48E-01	1.32E-01	1.00E+00
ENSBTAG00000013499	2.66E-03	1.00E+00	8.47E-01	6.13E-01	-5.23E-01	9.54E-01	1.16E+00	3.62E-01	-3.80E-01	1.00E+00
ENSBTAG00000016943	-4.07E-02	1.00E+00	-1.51E+00	1.06E-08	-7.29E-01	5.31E-03	-2.37E-01	5.30E-01	3.43E-01	6.46E-01

ENSBTAG00000019890	1.78E-01	1.00E+00	-6.72E-01	7.46E-01	5.58E-01	7.20E-01	-2.12E-01	1.00E+00	-8.39E-03	1.00E+00
ENSBTAG00000023144	2.58E-01	6.79E-01	1.51E-01	6.58E-01	3.46E-02	1.00E+00	-2.95E-01	2.15E-01	-3.57E-01	4.29E-01
ENSBTAG00000044619	0.00E+00	1.00E+00	0.00E+00	1.00E+00	-5.13E-15	1.00E+00	0.00E+00	1.00E+00	0.00E+00	1.00E+00
ENSBTAG00000046345	0.00E+00	1.00E+00	0.00E+00	1.00E+00	-5.13E-15	1.00E+00	0.00E+00	1.00E+00	0.00E+00	1.00E+00
ENSBTAG00000029993	0.00E+00	1.00E+00	0.00E+00	1.00E+00	2.56E+00	2.54E-01	0.00E+00	1.00E+00	0.00E+00	1.00E+00
ENSBTAG00000029849	0.00E+00	1.00E+00	0.00E+00	1.00E+00	-5.13E-15	1.00E+00	0.00E+00	1.00E+00	0.00E+00	1.00E+00
ENSBTAG00000004487	0.00E+00	1.00E+00	0.00E+00	1.00E+00	-5.13E-15	1.00E+00	0.00E+00	1.00E+00	0.00E+00	1.00E+00
ENSBTAG00000009265	5.55E-01	3.18E-01	3.39E+00	6.45E-32	1.72E+00	6.35E-10	1.01E+00	5.71E-04	2.15E-01	1.00E+00
ENSBTAG00000047428	5.06E-01	4.33E-01	1.26E+00	1.26E-05	1.63E+00	5.63E-09	1.26E+00	1.40E-05	4.80E-01	5.38E-01
ENSBTAG00000021874	-4.16E-01	1.00E+00	-1.25E+00	2.08E-01	-8.62E-01	4.48E-01	-3.84E-01	9.31E-01	3.98E-01	1.00E+00
ENSBTAG00000042576	0.00E+00	1.00E+00	0.00E+00	1.00E+00	-5.13E-15	1.00E+00	0.00E+00	1.00E+00	0.00E+00	1.00E+00
ENSBTAG00000020563	1.94E-01	9.64E-01	4.07E-01	1.21E-01	8.17E-01	2.90E-04	4.13E-01	1.20E-01	4.90E-01	2.80E-01
ENSBTAG00000008153	-4.95E-02	1.00E+00	7.85E-01	3.27E-03	8.35E-01	1.56E-03	6.20E-01	2.89E-02	1.77E-02	1.00E+00
ENSBTAG00000047972	-6.99E-01	9.83E-01	-1.53E+00	1.93E-01	-1.11E+00	3.31E-01	-1.92E-01	1.00E+00	-1.24E+00	6.83E-01
ENSBTAG00000047274	2.87E-01	9.05E-01	1.08E+00	3.30E-04	9.74E-01	1.37E-03	6.87E-01	3.91E-02	-2.14E-01	1.00E+00
ENSBTAG00000047491	1.42E-01	1.00E+00	1.63E+00	9.55E-04	-4.82E-01	5.97E-01	-1.02E-01	1.00E+00	-9.10E-02	1.00E+00
ENSBTAG00000048234	-3.52E-01	8.23E-01	-1.14E+00	4.17E-04	-1.54E+00	1.07E-06	-1.30E+00	5.92E-05	-3.80E-01	7.91E-01
ENSBTAG00000046177	-1.73E+00	2.21E-01	5.63E-01	6.72E-01	-2.24E+00	1.09E-02	-1.07E+00	2.83E-01	-2.05E+00	1.74E-01
ENSBTAG00000046350	-1.33E+00	4.89E-01	5.08E-01	6.74E-01	-7.03E-01	5.10E-01	-1.16E-01	1.00E+00	-3.72E-01	1.00E+00
ENSBTAG00000048129	1.91E+00	8.23E-01	0.00E+00	1.00E+00	-5.13E-15	1.00E+00	0.00E+00	1.00E+00	0.00E+00	1.00E+00
ENSBTAG00000046698	-1.96E+00	5.77E-01	-3.88E+00	3.63E-02	-3.88E+00	2.27E-02	-3.88E+00	2.47E-02	-1.16E+00	8.44E-01
ENSBTAG00000035265	2.19E-01	1.00E+00	-7.87E-01	1.02E-02	7.10E-01	1.74E-02	1.40E-01	9.21E-01	2.17E-01	9.93E-01
ENSBTAG00000005262	0.00E+00	1.00E+00	1.98E+00	4.43E-01	-5.13E-15	1.00E+00	2.53E+00	2.71E-01	1.91E+00	8.00E-01
ENSBTAG00000005263	-1.44E-01	1.00E+00	4.57E-01	2.21E-02	9.50E-02	9.01E-01	-5.46E-01	5.84E-03	-3.33E-01	4.55E-01
ENSBTAG00000003068	-4.38E-01	6.83E-01	-6.06E-01	1.03E-01	-1.14E+00	5.32E-04	-1.36E+00	3.78E-05	-3.55E-01	8.00E-01
ENSBTAG00000016514	-9.00E-02	1.00E+00	-6.83E-01	3.03E-01	-2.21E+00	8.24E-05	-1.73E+00	1.95E-03	-4.77E-01	8.76E-01
ENSBTAG00000043688	0.00E+00	1.00E+00	0.00E+00	1.00E+00	-5.13E-15	1.00E+00	0.00E+00	1.00E+00	0.00E+00	1.00E+00
ENSBTAG00000044433	0.00E+00	1.00E+00	0.00E+00	1.00E+00	-5.13E-15	1.00E+00	0.00E+00	1.00E+00	0.00E+00	1.00E+00
ENSBTAG00000005968	9.32E-02	1.00E+00	-1.59E-02	1.00E+00	-2.89E-01	8.92E-01	-1.66E-01	1.00E+00	-9.64E-01	5.69E-01
ENSBTAG00000019053	9.21E-02	1.00E+00	-1.09E+00	1.73E-01	-3.12E-01	8.74E-01	-2.07E-01	1.00E+00	-1.72E+00	1.90E-01
ENSBTAG00000016633	-7.86E-01	1.00E+00	-2.71E+00	2.42E-01	-2.71E+00	1.99E-01	-9.46E-01	8.04E-01	-7.81E-01	1.00E+00
ENSBTAG00000001696	-2.99E-01	1.00E+00	-3.33E-01	9.34E-01	-3.78E-01	8.50E-01	-1.25E+00	1.69E-01	-3.19E-01	1.00E+00
ENSBTAG00000017167	-7.35E-01	6.84E-02	-2.52E+00	5.29E-17	-6.66E-01	2.43E-02	-1.97E-01	7.20E-01	-1.85E-01	1.00E+00
ENSBTAG00000006745	1.46E+00	1.53E-07	1.85E+00	7.41E-13	2.00E+00	8.22E-15	2.11E+00	3.19E-16	1.54E+00	1.45E-07
ENSBTAG00000001638	1.48E+00	4.53E-08	1.82E+00	5.56E-13	2.04E+00	6.18E-16	2.13E+00	4.38E-17	1.67E+00	1.95E-09

ENSBTAG00000022120	1.33E+00	1.42E-04	1.53E+00	7.43E-07	1.55E+00	4.50E-07	1.69E+00	4.76E-08	1.67E+00	2.90E-06
ENSBTAG00000007234	1.52E-01	1.00E+00	1.60E+00	3.99E-12	2.21E+00	8.68E-22	2.43E+00	8.97E-26	-1.21E-01	1.00E+00
ENSBTAG00000024545	4.93E-01	1.00E+00	6.67E-01	5.63E-01	-2.56E-02	1.00E+00	6.86E-01	5.26E-01	-1.61E-01	1.00E+00
ENSBTAG00000018563	-7.58E-01	8.97E-01	-1.44E+00	1.33E-01	-2.82E+00	1.76E-03	-4.03E+00	3.94E-05	-2.17E-01	1.00E+00
ENSBTAG00000019426	-6.77E-01	1.00E+00	-2.01E-01	1.00E+00	2.02E-01	1.00E+00	-4.44E-02	1.00E+00	-1.38E-01	1.00E+00
ENSBTAG00000008008	6.76E-01	5.10E-01	2.64E+00	2.64E-10	2.45E+00	4.06E-09	1.68E+00	7.66E-05	-4.85E-01	8.00E-01
ENSBTAG00000016625	-5.22E-01	9.95E-02	-2.10E+00	8.35E-24	-1.90E+00	2.37E-20	-1.53E+00	1.17E-13	-1.76E-01	9.40E-01
ENSBTAG00000000281	1.29E-01	1.00E+00	-2.83E-01	9.07E-01	-7.06E-02	1.00E+00	-1.02E-01	1.00E+00	-1.21E-01	1.00E+00
ENSBTAG00000008816	-3.10E-01	8.85E-01	-1.64E+00	3.60E-07	-1.37E+00	2.01E-05	-7.49E-01	3.42E-02	-4.37E-01	6.78E-01
ENSBTAG00000043963	1.46E-02	1.00E+00	1.19E+00	8.05E-02	5.42E-01	5.99E-01	2.00E-01	1.00E+00	-3.82E-01	1.00E+00
ENSBTAG00000008438	2.15E-02	1.00E+00	2.61E-01	4.27E-01	7.41E-01	1.59E-03	5.55E-01	2.79E-02	-3.15E-01	6.90E-01
ENSBTAG00000009161	1.92E+00	8.23E-01	3.31E+00	1.02E-01	-5.13E-15	1.00E+00	2.54E+00	2.71E-01	3.89E+00	2.04E-01
ENSBTAG00000046689	-2.05E+00	7.12E-03	-1.50E+00	1.53E-02	-1.98E+00	9.96E-04	-1.53E+00	1.09E-02	-5.86E-02	1.00E+00
ENSBTAG00000043438	1.19E-02	1.00E+00	7.64E-02	1.00E+00	-1.91E+00	4.28E-01	-1.91E+00	4.39E-01	1.84E-02	1.00E+00
ENSBTAG00000046300	-1.91E+00	8.23E-01	-1.91E+00	4.69E-01	-1.91E+00	4.28E-01	-1.48E-01	1.00E+00	-1.91E+00	8.00E-01
ENSBTAG00000007953	2.99E-01	8.23E-01	3.18E-01	4.16E-01	4.90E-01	1.27E-01	3.35E-01	3.84E-01	5.04E-02	1.00E+00
ENSBTAG00000043241	0.00E+00	1.00E+00	0.00E+00	1.00E+00	-5.13E-15	1.00E+00	0.00E+00	1.00E+00	0.00E+00	1.00E+00
ENSBTAG00000042816	0.00E+00	1.00E+00	0.00E+00	1.00E+00	-5.13E-15	1.00E+00	0.00E+00	1.00E+00	0.00E+00	1.00E+00
ENSBTAG00000013914	-1.00E-01	1.00E+00	5.17E-02	1.00E+00	1.36E-01	7.94E-01	2.04E-01	5.43E-01	-3.46E-02	1.00E+00
ENSBTAG00000025263	-1.74E-01	1.00E+00	4.24E-01	2.91E-01	3.22E-01	4.79E-01	-4.53E-01	2.61E-01	2.60E-01	9.46E-01
ENSBTAG00000043071	0.00E+00	1.00E+00	0.00E+00	1.00E+00	-5.13E-15	1.00E+00	0.00E+00	1.00E+00	0.00E+00	1.00E+00
ENSBTAG00000043800	0.00E+00	1.00E+00	0.00E+00	1.00E+00	-5.13E-15	1.00E+00	1.76E+00	4.97E-01	0.00E+00	1.00E+00
ENSBTAG00000044732	-1.91E+00	8.23E-01	-1.91E+00	4.69E-01	-1.91E+00	4.28E-01	-1.91E+00	4.39E-01	-1.91E+00	8.00E-01
ENSBTAG00000042384	0.00E+00	1.00E+00	0.00E+00	1.00E+00	-5.13E-15	1.00E+00	0.00E+00	1.00E+00	0.00E+00	1.00E+00
ENSBTAG00000004633	-1.46E-01	1.00E+00	-1.01E+00	5.79E-02	-1.17E+00	1.96E-02	-8.70E-01	9.87E-02	2.60E-01	1.00E+00
ENSBTAG00000016813	-1.43E-01	1.00E+00	-5.55E-01	3.74E-02	-1.51E+00	3.43E-10	-1.04E+00	2.57E-05	3.27E-01	6.96E-01
ENSBTAG00000009908	9.27E-02	1.00E+00	2.24E-01	5.65E-01	1.36E-01	8.65E-01	-4.93E-02	1.00E+00	-5.22E-01	3.09E-01
ENSBTAG00000042696	0.00E+00	1.00E+00	0.00E+00	1.00E+00	-5.13E-15	1.00E+00	0.00E+00	1.00E+00	0.00E+00	1.00E+00
ENSBTAG00000018546	7.41E-02	1.00E+00	-6.34E-01	2.06E-02	-1.67E+00	3.26E-11	-1.05E+00	5.11E-05	5.69E-01	2.45E-01
ENSBTAG00000024563	0.00E+00	1.00E+00	0.00E+00	1.00E+00	-5.13E-15	1.00E+00	0.00E+00	1.00E+00	0.00E+00	1.00E+00
ENSBTAG00000047924	0.00E+00	1.00E+00	0.00E+00	1.00E+00	-5.13E-15	1.00E+00	0.00E+00	1.00E+00	0.00E+00	1.00E+00
ENSBTAG00000008098	9.13E-02	1.00E+00	-5.11E-01	2.66E-01	-1.56E-01	9.59E-01	1.62E-01	9.37E-01	-8.67E-02	1.00E+00
ENSBTAG00000045580	1.92E+00	8.23E-01	0.00E+00	1.00E+00	-5.13E-15	1.00E+00	1.76E+00	4.97E-01	0.00E+00	1.00E+00
ENSBTAG00000046538	0.00E+00	1.00E+00	0.00E+00	1.00E+00	-5.13E-15	1.00E+00	0.00E+00	1.00E+00	0.00E+00	1.00E+00
ENSBTAG00000042563	3.51E-01	1.00E+00	9.93E-01	4.53E-02	-4.39E-02	1.00E+00	1.60E-01	1.00E+00	5.77E-01	7.68E-01

ENSBTAG00000027182	2.15E-02	1.00E+00	-3.59E-01	2.73E-01	-7.61E-01	4.48E-03	-3.88E-01	2.31E-01	1.57E-01	1.00E+00
ENSBTAG00000002531	1.30E-01	1.00E+00	-7.17E-01	2.11E-02	-1.92E+00	5.11E-11	-7.77E-01	1.21E-02	-8.46E-02	1.00E+00
ENSBTAG00000045072	0.00E+00	1.00E+00	0.00E+00	1.00E+00	-5.13E-15	1.00E+00	0.00E+00	1.00E+00	0.00E+00	1.00E+00
ENSBTAG00000010381	-2.20E-02	1.00E+00	4.81E-01	3.02E-02	-2.84E-01	2.80E-01	-6.16E-01	4.80E-03	-3.60E-02	1.00E+00
ENSBTAG00000010376	3.06E-01	5.80E-01	4.04E-01	8.31E-02	1.69E-01	6.27E-01	-1.19E-01	8.52E-01	6.13E-02	1.00E+00
ENSBTAG00000013674	-3.50E-01	9.36E-01	-2.82E+00	4.05E-12	-2.18E+00	3.47E-08	-1.55E+00	1.23E-04	-2.10E-01	1.00E+00
ENSBTAG00000043066	0.00E+00	1.00E+00	0.00E+00	1.00E+00	-5.13E-15	1.00E+00	0.00E+00	1.00E+00	0.00E+00	1.00E+00
ENSBTAG00000004167	9.96E-01	5.76E-01	6.47E-03	1.00E+00	-1.23E+00	2.15E-01	4.92E-01	7.16E-01	-1.04E+00	7.51E-01
ENSBTAG00000042146	0.00E+00	1.00E+00	0.00E+00	1.00E+00	-5.13E-15	1.00E+00	0.00E+00	1.00E+00	0.00E+00	1.00E+00
ENSBTAG00000036310	8.34E-01	4.59E-01	1.42E+00	3.27E-03	1.63E+00	3.01E-04	1.35E+00	5.21E-03	2.70E-01	1.00E+00
ENSBTAG00000005648	2.70E+00	5.76E-01	0.00E+00	1.00E+00	1.78E+00	4.79E-01	0.00E+00	1.00E+00	2.71E+00	6.01E-01
ENSBTAG00000043898	0.00E+00	1.00E+00	1.99E+00	4.43E-01	-5.13E-15	1.00E+00	0.00E+00	1.00E+00	0.00E+00	1.00E+00
ENSBTAG00000034522	3.23E-01	8.60E-01	2.06E+00	4.30E-12	1.47E+00	1.21E-06	1.27E+00	4.67E-05	-1.80E-01	1.00E+00
ENSBTAG00000048020	6.29E-01	6.15E-01	1.77E+00	3.11E-05	1.85E+00	1.10E-05	1.37E+00	2.05E-03	4.05E-01	8.97E-01
ENSBTAG00000014060	1.37E-01	1.00E+00	6.26E-01	7.79E-03	6.16E-01	8.76E-03	3.42E-01	2.26E-01	-1.88E-01	9.46E-01
ENSBTAG00000042480	0.00E+00	1.00E+00	0.00E+00	1.00E+00	-5.13E-15	1.00E+00	0.00E+00	1.00E+00	0.00E+00	1.00E+00
ENSBTAG00000012738	1.72E-01	1.00E+00	-6.43E-01	3.55E-01	-7.75E-01	2.19E-01	1.41E+00	5.65E-03	5.59E-02	1.00E+00
ENSBTAG00000045724	0.00E+00	1.00E+00	0.00E+00	1.00E+00	-5.13E-15	1.00E+00	0.00E+00	1.00E+00	0.00E+00	1.00E+00
ENSBTAG00000019880	0.00E+00	1.00E+00	0.00E+00	1.00E+00	-5.13E-15	1.00E+00	0.00E+00	1.00E+00	0.00E+00	1.00E+00
ENSBTAG00000012800	8.32E-02	1.00E+00	-1.58E-02	1.00E+00	1.64E-01	9.00E-01	-1.74E-02	1.00E+00	-1.02E-01	1.00E+00
ENSBTAG00000002835	9.89E-01	1.35E-04	2.30E+00	2.18E-24	2.08E+00	1.63E-20	1.66E+00	1.66E-13	5.06E-01	2.70E-01
ENSBTAG00000045265	0.00E+00	1.00E+00	0.00E+00	1.00E+00	-5.13E-15	1.00E+00	0.00E+00	1.00E+00	0.00E+00	1.00E+00
ENSBTAG00000019857	4.42E-01	5.11E-01	1.11E+00	4.91E-05	1.21E+00	8.11E-06	4.15E-01	2.28E-01	2.28E-01	9.45E-01
ENSBTAG00000019854	4.72E-01	2.71E-01	1.54E+00	1.30E-11	2.04E+00	1.94E-19	2.07E+00	6.75E-20	1.02E-01	1.00E+00
ENSBTAG00000013326	2.44E-01	9.55E-01	-5.24E-01	1.18E-01	1.40E-02	1.00E+00	9.54E-02	1.00E+00	5.65E-02	1.00E+00
ENSBTAG00000042647	0.00E+00	1.00E+00	0.00E+00	1.00E+00	-5.13E-15	1.00E+00	0.00E+00	1.00E+00	0.00E+00	1.00E+00
ENSBTAG00000016071	-7.78E-02	1.00E+00	-9.39E-01	6.96E-04	-5.93E-01	4.62E-02	8.88E-02	1.00E+00	-2.63E-01	8.55E-01
ENSBTAG00000039325	-5.41E-01	1.00E+00	-1.09E+00	3.24E-01	-1.64E+00	9.77E-02	-7.49E-01	5.15E-01	-5.34E-01	1.00E+00
ENSBTAG00000047331	7.13E-01	7.52E-01	-1.75E+00	4.22E-03	-3.16E+00	3.54E-07	-3.74E+00	6.06E-09	2.13E-01	1.00E+00
ENSBTAG00000045252	0.00E+00	1.00E+00	0.00E+00	1.00E+00	1.78E+00	4.79E-01	1.76E+00	4.97E-01	0.00E+00	1.00E+00
ENSBTAG00000003399	2.04E-01	8.39E-01	3.72E-01	9.95E-02	9.78E-01	3.75E-07	1.13E+00	4.97E-09	6.85E-02	1.00E+00
ENSBTAG00000002813	6.48E-02	1.00E+00	-8.70E-01	6.10E-04	-8.63E-01	6.31E-04	-7.78E-01	2.80E-03	8.93E-02	1.00E+00
ENSBTAG00000001356	1.75E+00	2.47E-11	3.19E+00	2.06E-35	2.57E+00	1.74E-24	7.19E-01	8.09E-03	-1.40E-01	1.00E+00
ENSBTAG00000045665	-5.54E-02	1.00E+00	-4.00E-01	4.43E-01	-9.06E-01	2.21E-02	-8.44E-01	3.98E-02	1.25E-01	1.00E+00
ENSBTAG00000014111	1.84E-01	1.00E+00	5.94E-01	1.13E-01	-9.18E-02	1.00E+00	6.24E-01	9.23E-02	-3.86E-01	8.00E-01

ENSBTAG00000018200	5.73E-01	7.28E-01	2.99E-01	7.71E-01	-4.97E-01	4.59E-01	-2.90E-01	8.04E-01	5.18E-01	8.00E-01
ENSBTAG00000046082	0.00E+00	1.00E+00	0.00E+00	1.00E+00	-5.13E-15	1.00E+00	0.00E+00	1.00E+00	0.00E+00	1.00E+00
ENSBTAG00000000297	-2.96E-01	6.61E-01	-8.69E-01	5.66E-05	-8.11E-01	1.69E-04	-1.15E+00	7.24E-08	-1.65E-01	9.91E-01
ENSBTAG00000018951	1.49E+00	5.73E-03	2.18E+00	6.04E-07	4.99E-01	4.14E-01	3.42E-01	6.67E-01	1.88E-01	1.00E+00
ENSBTAG00000004647	1.92E+00	8.23E-01	3.98E+00	2.59E-02	-5.13E-15	1.00E+00	0.00E+00	1.00E+00	0.00E+00	1.00E+00
ENSBTAG00000014007	-1.43E-01	1.00E+00	-7.52E-01	1.02E-04	-3.35E-01	1.33E-01	1.69E-01	5.84E-01	-4.80E-01	1.51E-01
ENSBTAG00000034281	-6.03E-03	1.00E+00	4.08E-01	9.02E-01	8.74E-01	3.68E-01	8.43E-01	4.11E-01	4.57E-01	1.00E+00
ENSBTAG00000001580	1.57E+00	9.82E-02	2.65E+00	2.31E-06	9.92E-01	2.19E-01	1.23E+00	9.66E-02	1.46E+00	2.25E-01
ENSBTAG00000022109	1.43E-02	1.00E+00	1.20E-01	9.00E-01	3.15E-01	2.86E-01	3.83E-01	1.74E-01	-4.21E-01	4.45E-01
ENSBTAG00000011373	1.23E-01	1.00E+00	-7.24E-01	2.55E-01	-1.38E+00	1.17E-02	-1.32E+00	1.80E-02	2.49E-01	1.00E+00
ENSBTAG00000021779	1.00E-01	1.00E+00	2.40E-01	3.96E-01	-3.37E-01	1.64E-01	-8.47E-01	3.66E-05	-1.43E-01	1.00E+00
ENSBTAG00000003581	5.88E-01	5.76E-01	4.18E-01	4.66E-01	6.33E-01	1.70E-01	3.49E-01	5.83E-01	3.07E-01	9.90E-01
ENSBTAG00000043769	0.00E+00	1.00E+00	0.00E+00	1.00E+00	-5.13E-15	1.00E+00	0.00E+00	1.00E+00	0.00E+00	1.00E+00
ENSBTAG00000003208	-1.95E-01	1.00E+00	-9.49E-01	3.02E-04	7.56E-02	1.00E+00	3.06E-01	3.84E-01	-5.89E-01	2.25E-01
ENSBTAG00000004745	9.14E-02	1.00E+00	1.31E+00	1.73E-08	1.49E+00	8.86E-11	1.51E+00	8.21E-11	-5.34E-01	2.50E-01
ENSBTAG00000039582	-7.78E-02	1.00E+00	4.93E-02	1.00E+00	3.47E-02	1.00E+00	-1.76E-01	7.82E-01	-3.98E-01	6.14E-01
ENSBTAG00000002599	-4.43E-01	5.75E-01	-3.98E-01	2.77E-01	-1.02E+00	5.38E-04	-2.02E+00	7.90E-12	-1.07E-01	1.00E+00
ENSBTAG00000009110	-4.91E-02	1.00E+00	-5.65E-01	2.44E-02	-2.33E-01	4.79E-01	-2.35E-01	4.97E-01	-6.28E-01	1.01E-01
ENSBTAG00000015811	0.00E+00	1.00E+00	0.00E+00	1.00E+00	-5.13E-15	1.00E+00	0.00E+00	1.00E+00	0.00E+00	1.00E+00
ENSBTAG00000020594	2.88E-01	8.23E-01	1.77E+00	1.61E-11	1.25E+00	2.23E-06	7.99E-01	4.62E-03	1.30E-01	1.00E+00
ENSBTAG00000005998	3.11E+00	1.34E-13	1.97E+00	8.38E-07	1.82E+00	5.38E-06	1.06E+00	1.46E-02	7.02E-02	1.00E+00
ENSBTAG00000042152	0.00E+00	1.00E+00	0.00E+00	1.00E+00	-5.13E-15	1.00E+00	0.00E+00	1.00E+00	0.00E+00	1.00E+00
ENSBTAG00000034089	2.68E-01	7.52E-01	7.43E-01	4.95E-04	8.07E-01	1.23E-04	1.28E+00	4.04E-10	-2.63E-01	7.76E-01
ENSBTAG00000034075	-9.55E-01	8.86E-01	4.38E+00	2.82E-07	1.33E+00	1.99E-01	1.22E+00	2.68E-01	-9.92E-01	8.33E-01
ENSBTAG00000046209	0.00E+00	1.00E+00	0.00E+00	1.00E+00	-5.13E-15	1.00E+00	0.00E+00	1.00E+00	0.00E+00	1.00E+00
ENSBTAG00000015549	-1.50E+00	1.08E-05	-1.86E+00	1.88E-09	-2.92E-01	5.26E-01	-1.14E-01	1.00E+00	-6.47E-01	3.09E-01
ENSBTAG00000023895	-9.07E-01	9.63E-01	-4.38E-01	9.69E-01	-6.99E-01	7.28E-01	-4.16E-01	9.73E-01	-2.41E-01	1.00E+00
ENSBTAG00000044703	0.00E+00	1.00E+00	0.00E+00	1.00E+00	-5.13E-15	1.00E+00	0.00E+00	1.00E+00	0.00E+00	1.00E+00
ENSBTAG00000043885	-7.93E-01	1.00E+00	-7.23E-01	9.45E-01	-2.71E+00	1.99E-01	-2.71E+00	2.05E-01	-2.71E+00	6.01E-01
ENSBTAG00000045176	0.00E+00	1.00E+00	0.00E+00	1.00E+00	-5.13E-15	1.00E+00	0.00E+00	1.00E+00	0.00E+00	1.00E+00
ENSBTAG00000033967	0.00E+00	1.00E+00	0.00E+00	1.00E+00	-5.13E-15	1.00E+00	0.00E+00	1.00E+00	0.00E+00	1.00E+00
ENSBTAG00000042760	0.00E+00	1.00E+00	0.00E+00	1.00E+00	-5.13E-15	1.00E+00	0.00E+00	1.00E+00	0.00E+00	1.00E+00
ENSBTAG00000046902	2.12E+00	1.30E-02	1.07E+00	2.68E-01	1.07E+00	2.49E-01	1.77E+00	1.69E-02	1.66E+00	2.05E-01
ENSBTAG00000009722	0.00E+00	1.00E+00	0.00E+00	1.00E+00	1.78E+00	4.79E-01	0.00E+00	1.00E+00	0.00E+00	1.00E+00
ENSBTAG00000035012	8.04E-01	1.00E+00	-1.91E+00	4.69E-01	-1.91E+00	4.28E-01	-1.48E-01	1.00E+00	-1.91E+00	8.00E-01

ENSBTAG00000040370	0.00E+00	1.00E+00	1.98E+00	4.43E-01	-5.13E-15	1.00E+00	0.00E+00	1.00E+00	1.91E+00	8.00E-01
ENSBTAG00000036321	0.00E+00	1.00E+00	0.00E+00	1.00E+00	-5.13E-15	1.00E+00	0.00E+00	1.00E+00	0.00E+00	1.00E+00
ENSBTAG00000046669	0.00E+00	1.00E+00	0.00E+00	1.00E+00	-5.13E-15	1.00E+00	0.00E+00	1.00E+00	0.00E+00	1.00E+00
ENSBTAG00000019977	1.69E-02	1.00E+00	2.23E+00	7.82E-02	2.43E+00	3.89E-02	2.16E+00	8.89E-02	1.64E+00	6.65E-01
ENSBTAG00000036959	0.00E+00	1.00E+00	0.00E+00	1.00E+00	-5.13E-15	1.00E+00	0.00E+00	1.00E+00	0.00E+00	1.00E+00
ENSBTAG00000045079	0.00E+00	1.00E+00	0.00E+00	1.00E+00	-5.13E-15	1.00E+00	0.00E+00	1.00E+00	0.00E+00	1.00E+00
ENSBTAG00000043720	0.00E+00	1.00E+00	0.00E+00	1.00E+00	-5.13E-15	1.00E+00	0.00E+00	1.00E+00	0.00E+00	1.00E+00
ENSBTAG00000021011	1.03E-01	1.00E+00	2.15E-01	4.60E-01	6.80E-01	8.64E-04	9.97E-01	5.05E-07	-1.78E-01	9.20E-01
ENSBTAG00000044159	-1.25E-02	1.00E+00	-9.97E-01	4.13E-05	-1.21E+00	3.63E-07	-5.87E-01	2.35E-02	1.21E-01	1.00E+00
ENSBTAG00000013611	1.19E-01	1.00E+00	-5.25E-01	2.09E-01	-3.86E-01	3.97E-01	2.91E-01	5.53E-01	-1.56E-02	1.00E+00
ENSBTAG00000044504	0.00E+00	1.00E+00	0.00E+00	1.00E+00	-5.13E-15	1.00E+00	0.00E+00	1.00E+00	0.00E+00	1.00E+00
ENSBTAG00000017493	-4.04E-01	6.05E-01	-7.04E-02	1.00E+00	-1.97E-01	7.20E-01	-8.50E-02	1.00E+00	9.64E-02	1.00E+00
ENSBTAG00000004743	0.00E+00	1.00E+00	0.00E+00	1.00E+00	-5.13E-15	1.00E+00	0.00E+00	1.00E+00	0.00E+00	1.00E+00
ENSBTAG00000045423	0.00E+00	1.00E+00	0.00E+00	1.00E+00	-5.13E-15	1.00E+00	0.00E+00	1.00E+00	0.00E+00	1.00E+00
ENSBTAG00000009824	-1.65E-03	1.00E+00	-3.34E-02	1.00E+00	-1.70E-01	8.64E-01	-9.60E-02	1.00E+00	-5.54E-01	4.57E-01
ENSBTAG00000010843	-5.68E-03	1.00E+00	-1.18E+00	4.00E-08	-1.38E+00	9.66E-11	-1.01E+00	3.79E-06	-8.20E-02	1.00E+00
ENSBTAG00000012135	5.17E-01	2.10E-01	1.38E+00	2.70E-09	1.12E+00	1.71E-06	8.25E-01	7.85E-04	-3.34E-01	6.72E-01
ENSBTAG00000040242	6.09E-01	9.30E-01	5.30E-01	6.68E-01	-1.01E+00	3.47E-01	-1.57E+00	1.31E-01	-6.38E-01	9.66E-01
ENSBTAG00000043806	0.00E+00	1.00E+00	2.79E+00	2.10E-01	-5.13E-15	1.00E+00	0.00E+00	1.00E+00	0.00E+00	1.00E+00
ENSBTAG00000010630	-3.38E-01	8.06E-01	-2.62E+00	2.56E-19	-3.00E+00	1.74E-24	-1.47E+00	3.08E-07	3.17E-01	8.00E-01
ENSBTAG00000042423	0.00E+00	1.00E+00	0.00E+00	1.00E+00	-5.13E-15	1.00E+00	0.00E+00	1.00E+00	0.00E+00	1.00E+00
ENSBTAG00000044058	-1.94E-01	1.00E+00	-2.39E+00	2.92E-16	-1.98E+00	5.16E-12	-9.37E-01	1.87E-03	-2.86E-01	8.40E-01
ENSBTAG00000039552	2.23E-02	1.00E+00	-1.20E+00	9.12E-05	-1.42E+00	2.35E-06	-9.64E-01	2.11E-03	-2.99E-01	8.32E-01
ENSBTAG00000015692	5.55E-01	8.23E-01	2.74E+00	6.01E-08	7.67E-01	2.18E-01	8.36E-01	1.77E-01	2.62E-01	1.00E+00
ENSBTAG00000012826	1.26E-02	1.00E+00	8.80E-01	8.53E-01	-1.30E-01	1.00E+00	1.78E+00	3.08E-01	1.31E+00	9.00E-01
ENSBTAG00000012824	3.37E-01	8.23E-01	7.96E-01	1.53E-02	9.44E-01	2.32E-03	9.36E-01	3.13E-03	-1.89E-01	1.00E+00
ENSBTAG00000043375	0.00E+00	1.00E+00	0.00E+00	1.00E+00	-5.13E-15	1.00E+00	0.00E+00	1.00E+00	0.00E+00	1.00E+00
ENSBTAG00000005435	0.00E+00	1.00E+00	0.00E+00	1.00E+00	-5.13E-15	1.00E+00	0.00E+00	1.00E+00	0.00E+00	1.00E+00
ENSBTAG00000003345	-4.61E-01	6.60E-01	-1.68E+00	1.72E-06	-1.32E+00	1.33E-04	-7.83E-01	3.52E-02	-1.59E-01	1.00E+00
ENSBTAG00000001117	-1.97E-01	1.00E+00	-2.45E+00	5.84E-09	-2.14E+00	1.34E-07	-4.15E-01	4.56E-01	-3.37E-02	1.00E+00
ENSBTAG00000021245	7.39E-01	1.52E-02	4.38E-01	1.06E-01	3.75E-01	1.89E-01	1.51E-01	7.97E-01	-3.14E-01	7.14E-01
ENSBTAG00000000125	3.66E-01	7.82E-01	1.77E+00	7.93E-10	2.20E+00	1.12E-14	1.93E+00	1.95E-11	-3.69E-01	8.00E-01
ENSBTAG00000042522	0.00E+00	1.00E+00	0.00E+00	1.00E+00	-5.13E-15	1.00E+00	0.00E+00	1.00E+00	0.00E+00	1.00E+00
ENSBTAG00000019840	0.00E+00	1.00E+00	0.00E+00	1.00E+00	-5.13E-15	1.00E+00	0.00E+00	1.00E+00	0.00E+00	1.00E+00
ENSBTAG00000005695	-1.05E-01	1.00E+00	-6.41E-02	1.00E+00	-1.92E-01	7.08E-01	-4.18E-01	2.06E-01	9.52E-02	1.00E+00

ENSBTAG00000043841	0.00E+00	1.00E+00	0.00E+00	1.00E+00	-5.13E-15	1.00E+00	0.00E+00	1.00E+00	0.00E+00	1.00E+00
ENSBTAG00000005691	4.17E-01	6.61E-01	3.49E-01	4.11E-01	2.11E-02	1.00E+00	-6.90E-01	4.43E-02	-2.19E-01	1.00E+00
ENSBTAG00000007564	-2.75E-01	9.32E-01	-2.57E+00	2.08E-13	-1.02E+00	1.32E-03	-6.92E-01	4.19E-02	2.61E-01	9.12E-01
ENSBTAG00000016147	-1.78E-02	1.00E+00	-3.85E-01	4.10E-01	-8.42E-01	1.92E-02	-2.35E-01	7.17E-01	-7.11E-01	3.11E-01
ENSBTAG00000012368	1.17E+00	8.70E-01	-2.71E+00	2.42E-01	3.49E-01	1.00E+00	-1.71E-01	1.00E+00	-7.92E-01	1.00E+00
ENSBTAG00000020883	0.00E+00	1.00E+00	0.00E+00	1.00E+00	1.78E+00	4.79E-01	2.53E+00	2.71E-01	0.00E+00	1.00E+00
ENSBTAG00000046302	0.00E+00	1.00E+00	0.00E+00	1.00E+00	-5.13E-15	1.00E+00	0.00E+00	1.00E+00	0.00E+00	1.00E+00
ENSBTAG00000045738	0.00E+00	1.00E+00	0.00E+00	1.00E+00	-5.13E-15	1.00E+00	1.76E+00	4.97E-01	0.00E+00	1.00E+00
ENSBTAG00000045938	0.00E+00	1.00E+00	0.00E+00	1.00E+00	-5.13E-15	1.00E+00	0.00E+00	1.00E+00	0.00E+00	1.00E+00
ENSBTAG00000035606	0.00E+00	1.00E+00	0.00E+00	1.00E+00	-5.13E-15	1.00E+00	0.00E+00	1.00E+00	0.00E+00	1.00E+00
ENSBTAG00000046191	1.69E+00	7.72E-01	1.39E+00	5.18E-01	-1.30E-01	1.00E+00	1.78E+00	3.04E-01	1.33E+00	8.94E-01
ENSBTAG00000046515	0.00E+00	1.00E+00	0.00E+00	1.00E+00	-5.13E-15	1.00E+00	0.00E+00	1.00E+00	0.00E+00	1.00E+00
ENSBTAG00000019021	-3.53E-01	1.00E+00	-3.40E-01	7.27E-01	-7.42E-01	2.08E-01	-1.25E+00	2.44E-02	-4.45E-01	8.93E-01
ENSBTAG00000046852	-1.18E+00	8.62E-01	-1.11E+00	5.38E-01	-8.58E-01	6.52E-01	-5.02E-01	9.29E-01	-1.99E+00	6.01E-01
ENSBTAG00000043467	1.92E+00	8.23E-01	0.00E+00	1.00E+00	-5.13E-15	1.00E+00	0.00E+00	1.00E+00	0.00E+00	1.00E+00
ENSBTAG00000046833	0.00E+00	1.00E+00	0.00E+00	1.00E+00	-5.13E-15	1.00E+00	0.00E+00	1.00E+00	0.00E+00	1.00E+00
ENSBTAG00000047595	-1.23E+00	3.75E-01	-2.98E-01	9.37E-01	-3.60E-01	8.29E-01	-2.05E-01	1.00E+00	-3.70E-01	1.00E+00
ENSBTAG00000016059	-1.24E+00	6.62E-01	-3.92E-01	9.28E-01	-1.76E-01	1.00E+00	-6.78E-01	6.17E-01	-9.47E-01	8.00E-01
ENSBTAG00000048198	0.00E+00	1.00E+00	0.00E+00	1.00E+00	-5.13E-15	1.00E+00	0.00E+00	1.00E+00	0.00E+00	1.00E+00
ENSBTAG00000026437	4.72E-01	1.00E+00	4.05E-02	1.00E+00	4.65E-01	1.00E+00	1.84E-01	1.00E+00	-2.82E-02	1.00E+00
ENSBTAG00000012450	1.75E-01	9.62E-01	1.96E-01	5.24E-01	-6.76E-01	1.22E-03	-4.71E-01	3.85E-02	1.31E-01	1.00E+00
ENSBTAG00000047107	6.84E+00	1.72E-14	7.01E+00	4.12E-16	4.63E+00	4.13E-08	3.35E+00	1.71E-04	-1.16E+00	8.99E-01
ENSBTAG00000029127	0.00E+00	1.00E+00	0.00E+00	1.00E+00	-5.13E-15	1.00E+00	0.00E+00	1.00E+00	0.00E+00	1.00E+00
ENSBTAG00000033547	1.75E-01	1.00E+00	-6.15E-01	6.47E-02	-6.14E-01	6.50E-02	3.10E-01	4.85E-01	-2.73E-01	8.97E-01
ENSBTAG00000011970	4.70E-01	7.69E-01	1.67E-01	9.66E-01	2.66E-01	7.27E-01	1.51E+00	9.60E-05	6.80E-01	4.47E-01
ENSBTAG00000016680	2.16E-01	1.00E+00	1.29E+00	1.83E-05	2.77E-01	5.47E-01	-3.88E-01	3.37E-01	-4.30E-01	6.32E-01
ENSBTAG00000016679	-1.53E-01	1.00E+00	-5.97E-01	2.68E-02	-1.61E+00	5.16E-11	-1.86E+00	4.79E-14	2.50E-01	8.36E-01
ENSBTAG00000033486	-2.60E-01	9.26E-01	-1.27E+00	2.18E-05	-9.99E-01	6.74E-04	-3.71E-01	3.20E-01	-3.03E-01	8.00E-01
ENSBTAG00000010306	-3.29E-01	1.00E+00	-4.71E-01	8.93E-01	-2.50E-02	1.00E+00	5.75E-01	6.91E-01	-1.10E+00	8.00E-01
ENSBTAG00000019229	-4.82E-01	8.81E-01	-3.60E+00	6.28E-11	-3.51E+00	6.84E-11	-2.01E+00	1.08E-04	3.42E-02	1.00E+00
ENSBTAG00000017069	-7.54E-01	3.55E-01	-1.51E+00	4.55E-04	-1.09E+00	1.11E-02	2.78E-01	7.20E-01	1.37E+00	7.44E-03
ENSBTAG00000043594	-1.91E+00	8.23E-01	-1.91E+00	4.69E-01	-1.91E+00	4.28E-01	-1.91E+00	4.39E-01	-1.91E+00	8.00E-01
ENSBTAG00000004775	5.11E-01	1.00E+00	8.35E-01	4.74E-01	5.70E-01	7.30E-01	9.15E-01	4.10E-01	-1.65E+00	6.13E-01
ENSBTAG00000047318	0.00E+00	1.00E+00	0.00E+00	1.00E+00	-5.13E-15	1.00E+00	0.00E+00	1.00E+00	0.00E+00	1.00E+00
ENSBTAG00000021764	-6.02E-01	4.65E-01	-6.34E-01	1.34E-01	-8.81E-01	2.28E-02	-5.54E-01	2.05E-01	-5.44E-01	6.01E-01

ENSBTAG00000043959	-1.27E-01	1.00E+00	-4.50E-01	1.55E-01	-1.40E-01	8.75E-01	-5.10E-01	9.60E-02	3.31E-01	7.43E-01
ENSBTAG00000011063	-1.98E-01	9.74E-01	-5.65E-01	2.77E-02	-9.45E-01	7.29E-05	-1.06E+00	9.44E-06	4.49E-01	3.92E-01
ENSBTAG00000042385	0.00E+00	1.00E+00	0.00E+00	1.00E+00	-5.13E-15	1.00E+00	0.00E+00	1.00E+00	0.00E+00	1.00E+00
ENSBTAG00000011062	5.10E-01	7.11E-01	-1.44E-01	1.00E+00	4.24E-01	4.34E-01	-1.11E+00	7.23E-03	5.41E-01	6.57E-01
ENSBTAG00000044109	-2.03E-01	1.00E+00	-1.21E-01	1.00E+00	-9.20E-01	3.24E-02	-4.55E-01	4.22E-01	-8.74E-01	2.64E-01
ENSBTAG00000003840	4.78E-02	1.00E+00	-5.33E-01	8.19E-02	-8.88E-01	1.37E-03	-4.56E-01	1.63E-01	-4.65E-02	1.00E+00
ENSBTAG00000014576	-2.96E-01	9.28E-01	3.05E-01	5.16E-01	1.78E-01	8.49E-01	4.94E-01	2.00E-01	1.43E-01	1.00E+00
ENSBTAG00000005240	1.26E-01	1.00E+00	-6.07E-01	4.49E-02	-6.76E-01	2.17E-02	-9.55E-01	8.09E-04	-1.15E-01	1.00E+00
ENSBTAG00000013001	1.62E-01	1.00E+00	-1.10E+00	2.73E-02	-7.52E-01	1.52E-01	-1.45E+00	2.97E-03	6.84E-01	5.52E-01
ENSBTAG00000040072	-1.15E+00	1.81E-03	-9.48E-01	2.73E-03	2.95E-01	4.74E-01	-1.60E-02	1.00E+00	-1.87E-01	1.00E+00
ENSBTAG00000038806	-2.12E+00	2.45E-01	-3.86E-01	9.39E-01	1.39E+00	4.06E-02	7.26E-01	4.57E-01	-9.23E-01	8.00E-01
ENSBTAG00000039242	-3.69E-01	1.00E+00	-6.37E-01	3.25E-01	-4.30E-01	5.62E-01	-3.66E-01	6.78E-01	1.21E-01	1.00E+00
ENSBTAG00000040336	-7.72E-01	2.79E-02	5.26E-02	1.00E+00	1.17E+00	1.44E-06	3.01E-01	3.91E-01	1.90E-01	1.00E+00
ENSBTAG00000039316	2.16E-01	1.00E+00	8.48E-01	1.94E-03	2.03E-01	6.81E-01	-5.76E-01	6.55E-02	-5.60E-02	1.00E+00
ENSBTAG00000006753	0.00E+00	1.00E+00	0.00E+00	1.00E+00	-5.13E-15	1.00E+00	1.77E+00	4.97E-01	0.00E+00	1.00E+00
ENSBTAG00000006758	-2.33E-01	1.00E+00	-3.86E-01	8.82E-01	-1.13E+00	2.30E-01	-5.43E-01	6.76E-01	-1.42E+00	4.79E-01
ENSBTAG00000026626	4.13E-03	1.00E+00	9.80E-01	6.50E-01	7.27E-01	8.22E-01	7.06E-01	8.48E-01	6.22E-03	1.00E+00
ENSBTAG00000013951	2.53E-01	8.02E-01	1.23E+00	2.07E-09	1.32E+00	7.95E-11	1.00E+00	1.64E-06	3.34E-01	5.82E-01
ENSBTAG00000009755	1.62E-01	1.00E+00	1.31E+00	1.29E-07	1.06E+00	2.26E-05	8.69E-01	7.97E-04	5.62E-01	2.52E-01
ENSBTAG00000033339	-1.40E-01	1.00E+00	5.00E-01	6.48E-02	9.07E-01	1.91E-04	6.56E-01	1.14E-02	1.73E-01	1.00E+00
ENSBTAG00000007513	1.55E-01	9.74E-01	1.22E+00	4.38E-12	1.25E+00	1.02E-12	8.99E-01	6.21E-07	-2.51E-01	6.85E-01
ENSBTAG00000007510	2.70E-01	5.97E-01	-4.21E-01	3.70E-02	-1.12E+00	5.11E-10	-1.06E+00	6.89E-09	2.49E-01	6.95E-01
ENSBTAG00000000590	1.21E+00	2.94E-02	3.74E+00	1.56E-18	1.98E+00	1.60E-06	1.49E+00	4.50E-04	7.58E-01	4.34E-01
ENSBTAG00000042877	0.00E+00	1.00E+00	0.00E+00	1.00E+00	-5.13E-15	1.00E+00	0.00E+00	1.00E+00	0.00E+00	1.00E+00
ENSBTAG00000007496	1.34E+00	9.43E-01	6.69E+00	7.26E-11	2.61E+00	7.67E-02	2.05E+00	2.41E-01	2.66E-03	1.00E+00
ENSBTAG00000039766	-1.58E-01	1.00E+00	4.15E-01	6.82E-02	4.57E-02	1.00E+00	-2.35E-01	4.26E-01	2.45E-01	7.90E-01
ENSBTAG00000005644	1.02E-01	1.00E+00	-3.88E-01	8.68E-01	7.97E-02	1.00E+00	5.57E-02	1.00E+00	-4.71E-01	1.00E+00
ENSBTAG00000011889	-3.83E-02	1.00E+00	1.45E+00	2.14E-06	2.06E+00	5.91E-12	1.78E+00	5.04E-09	5.81E-03	1.00E+00
ENSBTAG00000017219	-1.60E-01	1.00E+00	1.36E+00	1.33E-09	1.49E+00	2.27E-11	9.65E-01	3.13E-05	-4.18E-01	4.68E-01
ENSBTAG00000022084	-2.23E-01	1.00E+00	1.57E+00	3.04E-06	1.49E+00	9.18E-06	9.28E-01	1.10E-02	5.62E-01	5.43E-01
ENSBTAG00000020423	-2.21E-01	9.42E-01	-4.23E-01	1.49E-01	-1.16E-01	9.39E-01	-1.60E-01	7.91E-01	4.00E-01	5.49E-01
ENSBTAG00000043329	0.00E+00	1.00E+00	0.00E+00	1.00E+00	-5.13E-15	1.00E+00	0.00E+00	1.00E+00	0.00E+00	1.00E+00
ENSBTAG00000012990	-3.99E-01	3.96E-01	3.52E-01	1.95E-01	2.57E-01	4.11E-01	-4.37E-01	9.24E-02	-5.13E-01	2.27E-01
ENSBTAG00000012987	-7.11E-01	4.84E-02	-1.63E+00	1.15E-10	-2.78E+00	2.38E-27	-3.15E+00	1.46E-33	5.29E-01	3.19E-01
ENSBTAG00000002145	-1.76E+00	1.94E-02	-1.18E+00	4.72E-02	-2.19E+00	2.11E-04	-1.98E+00	7.61E-04	2.34E-01	1.00E+00

ENSBTAG00000000439	9.46E-02	1.00E+00	5.44E-01	1.20E-02	7.64E-01	1.96E-04	2.56E-01	3.64E-01	-2.71E-02	1.00E+00
ENSBTAG00000036440	0.00E+00	1.00E+00	0.00E+00	1.00E+00	-5.13E-15	1.00E+00	0.00E+00	1.00E+00	0.00E+00	1.00E+00
ENSBTAG00000048211	1.78E-02	1.00E+00	-7.93E-01	7.88E-01	-5.22E-01	9.46E-01	1.18E+00	3.18E-01	-3.56E-01	1.00E+00
ENSBTAG00000013848	2.84E-02	1.00E+00	1.47E-01	1.00E+00	1.29E-01	1.00E+00	4.58E-01	4.81E-01	4.11E-01	8.97E-01
ENSBTAG00000007988	3.91E-02	1.00E+00	-4.55E-02	1.00E+00	-6.94E-02	1.00E+00	-2.22E-01	4.50E-01	-4.29E-02	1.00E+00
ENSBTAG00000009797	-4.23E-01	8.49E-01	-1.16E+00	1.31E-02	-1.44E+00	1.40E-03	-1.67E+00	2.52E-04	8.67E-02	1.00E+00
ENSBTAG00000006468	-1.95E+00	5.84E-01	-5.74E-01	8.97E-01	-2.09E+00	1.81E-01	5.17E-02	1.00E+00	-1.17E+00	8.44E-01
ENSBTAG00000007047	-1.18E+00	8.03E-01	-5.59E+00	6.90E-04	-1.93E+00	1.04E-01	-8.06E-01	6.13E-01	7.43E-01	9.37E-01
ENSBTAG00000044838	0.00E+00	1.00E+00	0.00E+00	1.00E+00	-5.13E-15	1.00E+00	0.00E+00	1.00E+00	0.00E+00	1.00E+00
ENSBTAG00000002950	5.32E-01	1.00E+00	-2.70E+00	2.42E-01	-2.70E+00	1.99E-01	-2.70E+00	2.05E-01	6.80E-03	1.00E+00
ENSBTAG00000020430	2.69E-01	8.23E-01	-2.38E-02	1.00E+00	-7.49E-01	4.50E-03	-9.34E-01	3.37E-04	1.44E-01	1.00E+00
ENSBTAG00000046676	0.00E+00	1.00E+00	0.00E+00	1.00E+00	-5.13E-15	1.00E+00	0.00E+00	1.00E+00	0.00E+00	1.00E+00
ENSBTAG00000009167	8.20E-02	1.00E+00	1.16E+00	3.47E-04	8.15E-01	1.64E-02	-6.51E-02	1.00E+00	3.56E-02	1.00E+00
ENSBTAG00000046256	2.46E-01	1.00E+00	1.47E-01	1.00E+00	-1.31E+00	7.38E-02	-5.05E-02	1.00E+00	-9.60E-01	6.01E-01
ENSBTAG00000046439	0.00E+00	1.00E+00	0.00E+00	1.00E+00	-5.13E-15	1.00E+00	0.00E+00	1.00E+00	0.00E+00	1.00E+00
ENSBTAG00000046657	5.54E-01	1.52E-01	2.02E+00	1.22E-19	1.79E+00	6.08E-16	1.29E+00	1.61E-08	-6.25E-02	1.00E+00
ENSBTAG00000047597	0.00E+00	1.00E+00	0.00E+00	1.00E+00	-5.13E-15	1.00E+00	0.00E+00	1.00E+00	0.00E+00	1.00E+00
ENSBTAG00000033558	0.00E+00	1.00E+00	0.00E+00	1.00E+00	-5.13E-15	1.00E+00	0.00E+00	1.00E+00	0.00E+00	1.00E+00
ENSBTAG00000046306	0.00E+00	1.00E+00	0.00E+00	1.00E+00	-5.13E-15	1.00E+00	0.00E+00	1.00E+00	0.00E+00	1.00E+00
ENSBTAG00000047085	-1.97E+00	5.76E-01	3.41E-01	1.00E+00	-3.89E+00	2.22E-02	-2.13E+00	1.76E-01	-1.18E+00	8.33E-01
ENSBTAG00000039769	0.00E+00	1.00E+00	0.00E+00	1.00E+00	-5.13E-15	1.00E+00	0.00E+00	1.00E+00	0.00E+00	1.00E+00
ENSBTAG00000034761	0.00E+00	1.00E+00	0.00E+00	1.00E+00	-5.13E-15	1.00E+00	0.00E+00	1.00E+00	0.00E+00	1.00E+00
ENSBTAG00000020532	-3.83E-03	1.00E+00	-3.32E-02	1.00E+00	-1.64E-01	7.76E-01	-6.33E-01	2.48E-02	-4.06E-01	5.83E-01
ENSBTAG00000046986	1.92E+00	8.23E-01	1.98E+00	4.43E-01	-5.13E-15	1.00E+00	1.76E+00	4.97E-01	0.00E+00	1.00E+00
ENSBTAG00000033620	-1.91E+00	8.23E-01	-1.91E+00	4.69E-01	-1.91E+00	4.28E-01	-1.91E+00	4.39E-01	-1.91E+00	8.00E-01
ENSBTAG00000047832	0.00E+00	1.00E+00	0.00E+00	1.00E+00	-5.13E-15	1.00E+00	0.00E+00	1.00E+00	0.00E+00	1.00E+00
ENSBTAG00000006626	-7.37E-01	9.32E-01	3.90E+00	1.53E-06	2.49E+00	2.03E-03	1.04E+00	3.10E-01	-4.75E-02	1.00E+00
ENSBTAG00000043850	0.00E+00	1.00E+00	0.00E+00	1.00E+00	1.78E+00	4.79E-01	0.00E+00	1.00E+00	0.00E+00	1.00E+00
ENSBTAG00000005678	3.32E-01	9.16E-01	7.55E-01	5.01E-02	4.04E-01	4.11E-01	4.73E-01	3.03E-01	-1.41E-01	1.00E+00
ENSBTAG00000001543	-3.32E-01	8.70E-01	9.61E-02	1.00E+00	5.81E-01	1.30E-01	3.99E-01	3.86E-01	2.62E-01	9.74E-01
ENSBTAG00000020414	-1.22E-01	1.00E+00	1.01E+00	1.96E-07	1.33E+00	2.30E-12	8.10E-01	4.85E-05	2.36E-01	8.00E-01
ENSBTAG00000044428	0.00E+00	1.00E+00	0.00E+00	1.00E+00	-5.13E-15	1.00E+00	1.76E+00	4.97E-01	0.00E+00	1.00E+00
ENSBTAG00000032436	1.21E+00	9.52E-02	2.57E+00	1.77E-07	6.61E-01	2.78E-01	7.95E-01	1.76E-01	-5.73E-01	7.96E-01
ENSBTAG00000014269	4.35E-02	1.00E+00	1.65E+00	3.79E-17	1.68E+00	8.21E-18	1.45E+00	1.73E-13	2.88E-01	6.40E-01
ENSBTAG00000024603	4.08E-01	3.73E-01	1.32E+00	9.07E-10	9.46E-01	1.64E-05	1.02E+00	3.74E-06	4.49E-01	3.39E-01

ENSBTAG00000043935	0.00E+00	1.00E+00	0.00E+00	1.00E+00	-5.13E-15	1.00E+00	0.00E+00	1.00E+00	0.00E+00	1.00E+00
ENSBTAG00000039688	2.34E+00	7.85E-06	4.94E+00	4.83E-22	5.16E+00	1.10E-23	4.12E+00	1.04E-16	8.60E-01	4.34E-01
ENSBTAG00000044717	0.00E+00	1.00E+00	0.00E+00	1.00E+00	-5.13E-15	1.00E+00	0.00E+00	1.00E+00	0.00E+00	1.00E+00
ENSBTAG00000039023	-1.50E-01	1.00E+00	1.34E-01	1.00E+00	1.08E+00	3.10E-03	1.83E+00	2.03E-07	5.87E-01	5.35E-01
ENSBTAG00000020346	-7.60E-01	5.56E-01	-1.67E+00	2.02E-03	-8.90E-01	1.10E-01	-8.92E-01	1.18E-01	1.77E-01	1.00E+00
ENSBTAG00000016523	6.82E-01	8.23E-01	1.60E+00	1.22E-02	6.22E-01	5.03E-01	7.93E-01	3.49E-01	3.70E-02	1.00E+00
ENSBTAG00000029491	0.00E+00	1.00E+00	0.00E+00	1.00E+00	-5.13E-15	1.00E+00	0.00E+00	1.00E+00	0.00E+00	1.00E+00
ENSBTAG00000007272	8.56E-02	1.00E+00	1.80E-01	7.07E-01	9.57E-01	8.57E-05	1.11E+00	5.33E-06	3.28E-02	1.00E+00
ENSBTAG00000007271	-5.25E-02	1.00E+00	-1.66E-02	1.00E+00	-3.20E-01	3.86E-01	-7.38E-01	1.15E-02	1.61E-01	1.00E+00
ENSBTAG00000007270	4.69E-01	2.39E-01	2.19E+00	3.18E-24	2.03E+00	2.25E-21	1.45E+00	2.33E-11	-4.58E-02	1.00E+00
ENSBTAG00000007269	6.39E-02	1.00E+00	9.97E-01	8.15E-06	1.54E+00	1.42E-12	1.20E+00	7.15E-08	-1.06E-01	1.00E+00
ENSBTAG00000007266	3.14E-01	5.22E-01	6.35E-01	1.42E-03	4.83E-01	2.00E-02	1.26E-01	7.94E-01	2.36E-01	7.92E-01
ENSBTAG00000039630	8.36E-01	6.55E-03	1.87E+00	1.06E-14	2.03E+00	4.68E-17	2.14E+00	1.23E-18	6.43E-01	1.25E-01
ENSBTAG00000008061	4.54E-01	5.76E-01	2.64E-01	5.97E-01	4.10E-02	1.00E+00	1.11E+00	3.57E-04	-3.03E-01	8.47E-01
ENSBTAG00000008060	-1.37E-01	1.00E+00	-5.25E-01	4.83E-02	-1.49E-01	7.99E-01	-4.84E-01	7.77E-02	-6.67E-02	1.00E+00
ENSBTAG00000007804	3.12E-01	8.50E-01	-3.64E-01	3.93E-01	-8.46E-01	8.96E-03	-1.38E-01	9.58E-01	5.80E-02	1.00E+00
ENSBTAG00000044721	0.00E+00	1.00E+00	0.00E+00	1.00E+00	-5.13E-15	1.00E+00	0.00E+00	1.00E+00	0.00E+00	1.00E+00
ENSBTAG00000000139	1.72E-01	1.00E+00	1.09E+00	2.49E-07	1.33E+00	1.84E-10	9.49E-01	9.91E-06	-9.24E-02	1.00E+00
ENSBTAG00000020505	3.96E-01	7.84E-01	1.32E+00	5.95E-05	1.62E+00	5.79E-07	1.78E+00	4.54E-08	3.14E-01	8.65E-01
ENSBTAG00000020504	-4.87E-02	1.00E+00	-6.14E-01	2.00E-02	-8.38E-01	8.20E-04	-7.92E-01	2.02E-03	-1.37E-01	1.00E+00
ENSBTAG00000020503	-2.17E-01	1.00E+00	-2.40E-01	6.57E-01	1.02E-01	1.00E+00	1.66E-01	8.65E-01	-1.84E-02	1.00E+00
ENSBTAG00000008470	1.42E-02	1.00E+00	-9.23E-01	2.19E-05	-4.14E-01	9.01E-02	-5.20E-02	1.00E+00	1.53E-01	1.00E+00
ENSBTAG00000036794	0.00E+00	1.00E+00	0.00E+00	1.00E+00	-5.13E-15	1.00E+00	0.00E+00	1.00E+00	0.00E+00	1.00E+00
ENSBTAG00000001597	-1.19E-01	1.00E+00	-1.53E+00	1.83E-05	-5.62E-01	1.51E-01	-1.69E-01	8.98E-01	1.63E-01	1.00E+00
ENSBTAG00000001594	-2.89E-01	7.75E-01	-4.64E-01	8.31E-02	-7.68E-01	1.50E-03	-7.06E-01	4.56E-03	-2.66E-01	8.00E-01
ENSBTAG00000001593	-3.16E-01	7.92E-01	-6.28E-01	2.89E-02	-3.06E-01	4.03E-01	-8.20E-02	1.00E+00	-3.32E-01	7.62E-01
ENSBTAG00000008464	1.79E-01	1.00E+00	-2.27E+00	4.64E-04	-1.51E+00	1.44E-02	-1.28E+00	4.39E-02	-3.09E-02	1.00E+00
ENSBTAG00000021595	5.83E-02	1.00E+00	6.36E-01	1.91E-03	7.55E-01	1.58E-04	8.93E-01	7.35E-06	-3.10E-01	6.01E-01
ENSBTAG00000003527	1.13E-01	1.00E+00	4.26E-01	4.08E-01	4.69E-01	3.19E-01	-3.01E-01	6.64E-01	5.47E-02	1.00E+00
ENSBTAG00000032433	7.97E-02	1.00E+00	8.97E-01	4.65E-04	1.76E+00	3.91E-13	7.99E-01	2.43E-03	-4.58E-01	4.68E-01
ENSBTAG00000045026	0.00E+00	1.00E+00	0.00E+00	1.00E+00	-5.13E-15	1.00E+00	0.00E+00	1.00E+00	0.00E+00	1.00E+00
ENSBTAG00000000908	0.00E+00	1.00E+00	4.93E+00	1.20E-03	6.04E+00	1.15E-06	5.39E+00	1.10E-04	0.00E+00	1.00E+00
ENSBTAG00000047904	3.95E+00	4.34E-05	5.51E+00	4.22E-10	5.38E+00	8.57E-10	5.10E+00	6.80E-09	-5.51E-01	1.00E+00
ENSBTAG00000019278	-3.03E-01	1.00E+00	2.31E-02	1.00E+00	-3.16E-01	6.61E-01	-1.12E+00	1.49E-02	-5.20E-01	7.68E-01
ENSBTAG00000006118	3.86E-01	3.75E-01	7.88E-01	1.90E-04	3.95E-01	9.98E-02	2.06E-02	1.00E+00	-3.10E-01	6.10E-01

ENSBTAG00000006114	5.24E-01	1.46E-01	1.10E+00	6.69E-07	5.17E-01	3.46E-02	-7.22E-02	1.00E+00	1.10E-01	1.00E+00
ENSBTAG00000044855	0.00E+00	1.00E+00	0.00E+00	1.00E+00	-5.13E-15	1.00E+00	0.00E+00	1.00E+00	0.00E+00	1.00E+00
ENSBTAG00000016779	-2.12E-01	1.00E+00	-1.37E+00	2.46E-05	-1.01E+00	2.40E-03	-1.52E+00	3.21E-06	-4.45E-02	1.00E+00
ENSBTAG00000037243	0.00E+00	1.00E+00	0.00E+00	1.00E+00	-5.13E-15	1.00E+00	0.00E+00	1.00E+00	0.00E+00	1.00E+00
ENSBTAG00000004203	1.56E-02	1.00E+00	-9.06E-01	5.86E-07	-4.64E-01	1.69E-02	-3.75E-01	7.20E-02	6.10E-02	1.00E+00
ENSBTAG00000004199	2.59E-02	1.00E+00	3.55E-01	1.37E-01	7.13E-01	5.20E-04	3.74E-01	1.19E-01	-5.54E-03	1.00E+00
ENSBTAG00000004197	-2.71E+00	5.76E-01	-2.71E+00	2.42E-01	-1.66E-01	1.00E+00	-1.73E-01	1.00E+00	-7.87E-01	1.00E+00
ENSBTAG00000004192	1.25E-02	1.00E+00	1.39E+00	5.16E-01	-1.91E+00	4.28E-01	6.27E-01	9.94E-01	1.32E+00	8.97E-01
ENSBTAG00000004189	-7.19E-02	1.00E+00	3.08E-01	2.46E-01	6.55E-01	2.54E-03	5.70E-01	1.20E-02	-6.40E-02	1.00E+00
ENSBTAG00000032517	-7.13E-01	2.71E-01	-2.02E+00	2.87E-07	2.28E-01	7.42E-01	1.24E+00	1.21E-04	2.95E-01	9.11E-01
ENSBTAG00000029069	0.00E+00	1.00E+00	0.00E+00	1.00E+00	-5.13E-15	1.00E+00	0.00E+00	1.00E+00	0.00E+00	1.00E+00
ENSBTAG00000039412	0.00E+00	1.00E+00	0.00E+00	1.00E+00	-5.13E-15	1.00E+00	0.00E+00	1.00E+00	0.00E+00	1.00E+00
ENSBTAG00000004187	-1.22E+00	7.05E-01	7.70E-01	4.49E-01	-3.72E-02	1.00E+00	6.19E-01	5.91E-01	-2.79E-01	1.00E+00
ENSBTAG00000004179	-3.37E-01	4.42E-01	-2.88E-01	2.42E-01	4.87E-01	1.91E-02	1.03E+00	7.21E-08	-1.62E-02	1.00E+00
ENSBTAG00000004175	-2.06E-01	1.00E+00	-7.87E-01	1.76E-02	-1.78E+00	6.31E-09	-1.45E+00	3.43E-06	-3.19E-02	1.00E+00
ENSBTAG00000004172	1.08E-01	1.00E+00	1.34E+00	9.39E-05	1.53E+00	6.08E-06	1.48E+00	1.59E-05	2.45E-01	1.00E+00
ENSBTAG00000032534	7.06E-02	1.00E+00	1.57E+00	3.82E-10	-1.27E-01	9.19E-01	-6.46E-01	2.29E-02	-3.19E-01	7.77E-01
ENSBTAG00000009602	-9.59E-02	1.00E+00	-1.72E+00	1.11E-08	-6.91E-01	3.36E-02	1.42E-01	9.38E-01	1.20E-01	1.00E+00
ENSBTAG00000004458	-2.33E-01	1.00E+00	-2.15E+00	1.62E-01	-7.00E-01	7.27E-01	-4.44E-01	9.48E-01	-5.48E-01	1.00E+00
ENSBTAG00000004457	-4.69E-01	4.33E-01	-1.06E+00	1.42E-04	-8.33E-01	3.11E-03	-4.66E-01	1.52E-01	-6.10E-01	2.51E-01
ENSBTAG00000002328	2.16E-02	1.00E+00	-1.84E+00	7.03E-15	-1.17E+00	4.24E-07	-6.53E-01	8.64E-03	5.46E-01	1.97E-01
ENSBTAG00000002315	-2.96E-01	8.23E-01	-4.72E-01	1.47E-01	9.53E-01	2.41E-04	9.91E-01	1.57E-04	8.57E-01	2.10E-02
ENSBTAG00000003092	-1.94E-01	9.24E-01	-6.09E-01	5.22E-03	-6.14E-01	4.61E-03	-6.02E-01	6.61E-03	1.22E-01	1.00E+00
ENSBTAG00000048014	0.00E+00	1.00E+00	0.00E+00	1.00E+00	-5.13E-15	1.00E+00	0.00E+00	1.00E+00	0.00E+00	1.00E+00
ENSBTAG00000010815	-6.93E-02	1.00E+00	1.93E-01	6.90E-01	-5.88E-01	3.68E-02	-5.99E-01	3.69E-02	2.74E-01	8.00E-01
ENSBTAG00000010812	-4.12E-01	2.27E-01	-1.51E+00	2.23E-15	-2.40E+00	3.15E-35	-2.58E+00	7.87E-40	1.40E-01	1.00E+00
ENSBTAG00000008948	-7.51E-01	5.79E-01	3.89E-02	1.00E+00	3.84E-01	5.93E-01	6.26E-02	1.00E+00	-4.53E-01	8.95E-01
ENSBTAG00000020584	2.70E-01	8.23E-01	2.77E-01	4.43E-01	-1.22E-01	9.32E-01	-3.23E-03	1.00E+00	3.01E-01	8.00E-01
ENSBTAG00000001398	7.91E-01	1.90E-02	3.21E+00	4.89E-35	1.95E+00	7.63E-15	1.71E+00	1.35E-11	2.06E-01	9.69E-01
ENSBTAG00000016675	-1.81E-01	1.00E+00	1.04E-01	9.99E-01	3.22E-01	3.39E-01	1.67E-01	7.83E-01	-2.86E-01	8.00E-01
ENSBTAG00000005345	-3.17E-03	1.00E+00	-2.78E-02	1.00E+00	-7.92E-02	9.72E-01	-2.06E-03	1.00E+00	-2.82E-01	6.01E-01
ENSBTAG00000005347	-2.21E-01	8.50E-01	1.18E-02	1.00E+00	6.76E-01	1.57E-03	6.21E-01	4.92E-03	-4.37E-01	3.49E-01
ENSBTAG00000022067	-3.93E-01	9.43E-01	-1.09E-01	1.00E+00	-9.78E-01	5.42E-02	-5.97E-01	3.05E-01	5.86E-02	1.00E+00
ENSBTAG00000008417	2.82E-03	1.00E+00	-2.22E-02	1.00E+00	-1.03E-01	8.61E-01	-8.04E-02	9.76E-01	-2.48E-01	7.08E-01
ENSBTAG00000018330	8.95E-02	1.00E+00	-1.27E+00	1.14E-01	-2.14E+00	3.94E-03	-1.82E+00	1.80E-02	1.52E-01	1.00E+00

ENSBTAG00000018332	-1.02E+00	3.69E-03	-5.70E-01	7.77E-02	-3.41E-01	3.77E-01	-1.24E-01	9.69E-01	1.10E-01	1.00E+00
ENSBTAG00000009208	-2.23E-01	8.28E-01	-1.31E+00	1.08E-09	-1.91E+00	5.37E-19	-1.59E+00	1.13E-13	4.28E-01	3.35E-01
ENSBTAG00000009216	8.81E-02	1.00E+00	-2.54E-01	8.88E-01	-9.37E-01	1.00E-01	-4.40E-01	5.51E-01	4.95E-01	8.00E-01
ENSBTAG00000011198	-8.76E-02	1.00E+00	1.53E-01	7.22E-01	-3.84E-01	1.21E-01	-2.11E-01	5.08E-01	-2.07E-01	8.55E-01
ENSBTAG00000043200	2.70E+00	5.76E-01	1.98E+00	4.43E-01	-5.13E-15	1.00E+00	0.00E+00	1.00E+00	0.00E+00	1.00E+00
ENSBTAG00000032353	6.17E-02	1.00E+00	-2.00E+00	4.01E-03	-3.01E+00	3.78E-05	-2.76E+00	1.14E-04	-7.15E-01	7.57E-01
ENSBTAG00000018369	5.92E-01	1.00E+00	1.57E+00	2.67E-01	-6.05E-01	8.65E-01	1.22E+00	4.46E-01	8.33E-01	9.99E-01
ENSBTAG00000008571	2.16E-01	1.00E+00	8.97E-01	3.97E-01	1.25E+00	1.72E-01	9.38E-01	3.72E-01	1.27E+00	5.22E-01
ENSBTAG00000002122	2.74E-01	1.00E+00	-3.04E-01	7.89E-01	7.08E-01	1.56E-01	9.59E-01	3.63E-02	2.93E-01	1.00E+00
ENSBTAG00000009101	4.94E-01	1.77E-01	4.34E-01	8.27E-02	-6.23E-01	8.14E-03	-6.19E-01	9.87E-03	-6.71E-01	5.30E-02
ENSBTAG00000022069	-4.88E-01	1.09E-01	-1.66E+00	1.49E-17	-1.12E+00	7.49E-09	-7.03E-01	5.57E-04	-4.24E-03	1.00E+00
ENSBTAG00000001168	-9.86E-02	1.00E+00	3.14E-01	3.40E-01	5.78E-01	2.91E-02	5.78E-01	3.32E-02	2.34E-01	8.76E-01
ENSBTAG00000001164	-5.35E-01	3.16E-01	-4.51E-01	1.72E-01	-1.50E+00	3.60E-08	-1.43E+00	2.09E-07	-1.45E-01	1.00E+00
ENSBTAG00000008963	-6.85E-02	1.00E+00	1.02E-01	1.00E+00	-4.77E-01	3.95E-01	-7.97E-01	9.37E-02	-1.34E-01	1.00E+00
ENSBTAG00000005940	5.37E-02	1.00E+00	5.33E-01	2.06E-02	9.70E-01	4.28E-06	7.55E-01	6.03E-04	-7.30E-02	1.00E+00
ENSBTAG00000004693	3.87E-01	1.00E+00	-5.79E-01	4.99E-01	-2.01E+00	3.97E-03	-9.75E-01	1.73E-01	1.19E-01	1.00E+00
ENSBTAG00000001303	-8.01E-02	1.00E+00	1.52E+00	3.36E-02	-1.02E+00	1.97E-01	-8.83E-01	3.01E-01	-4.22E-01	1.00E+00
ENSBTAG00000008676	3.26E-01	1.00E+00	-2.76E-01	1.00E+00	-1.03E+00	6.02E-01	1.09E-01	1.00E+00	-3.55E-01	1.00E+00
ENSBTAG00000043239	0.00E+00	1.00E+00	0.00E+00	1.00E+00	-5.13E-15	1.00E+00	0.00E+00	1.00E+00	0.00E+00	1.00E+00
ENSBTAG00000019757	1.34E-01	1.00E+00	2.30E-01	4.43E-01	7.35E-01	3.24E-04	9.09E-01	6.67E-06	2.51E-01	7.87E-01
ENSBTAG00000012550	4.21E-02	1.00E+00	-4.67E-01	1.26E-01	-2.20E-01	6.08E-01	2.22E-01	6.07E-01	-8.59E-02	1.00E+00
ENSBTAG00000018115	-2.99E-01	7.00E-01	-9.64E-01	1.67E-05	-2.07E+00	4.27E-21	-2.39E+00	4.53E-27	-3.24E-01	6.27E-01
ENSBTAG00000007855	-1.73E-01	1.00E+00	-1.32E+00	7.55E-06	-1.73E+00	2.76E-09	-1.76E+00	2.04E-09	8.44E-01	3.98E-02
ENSBTAG00000007139	3.55E-01	5.33E-01	1.61E+00	9.25E-14	1.45E+00	1.98E-11	1.05E+00	1.90E-06	1.69E-02	1.00E+00
ENSBTAG00000007137	-1.21E-01	1.00E+00	-4.87E-01	2.98E-02	-5.88E-01	6.37E-03	-5.87E-01	7.52E-03	-1.48E-01	1.00E+00
ENSBTAG00000044119	-6.79E-01	9.44E-01	-3.26E-01	9.74E-01	-6.44E-01	6.03E-01	4.68E-01	7.89E-01	-1.48E+00	4.34E-01
ENSBTAG00000042710	0.00E+00	1.00E+00	0.00E+00	1.00E+00	-5.13E-15	1.00E+00	0.00E+00	1.00E+00	0.00E+00	1.00E+00
ENSBTAG00000045549	0.00E+00	1.00E+00	0.00E+00	1.00E+00	-5.13E-15	1.00E+00	0.00E+00	1.00E+00	0.00E+00	1.00E+00
ENSBTAG00000002023	-1.18E+00	8.79E-01	3.21E-01	1.00E+00	2.56E-01	1.00E+00	1.08E+00	3.62E-01	2.34E-01	1.00E+00
ENSBTAG00000046747	3.21E+00	3.75E-01	2.79E+00	2.10E-01	-5.13E-15	1.00E+00	2.53E+00	2.71E-01	1.91E+00	8.00E-01
ENSBTAG00000037415	2.06E-01	1.00E+00	-2.31E-01	8.81E-01	-8.09E-01	1.09E-01	-4.00E-01	5.48E-01	1.89E-01	1.00E+00
ENSBTAG00000032097	-6.03E-01	2.21E-01	-2.45E+00	4.58E-18	-2.65E+00	7.53E-21	-1.76E+00	3.61E-10	4.03E-02	1.00E+00
ENSBTAG00000045851	1.07E+00	6.07E-01	4.58E-01	8.63E-01	6.90E-01	5.62E-01	1.13E+00	2.05E-01	1.69E+00	1.64E-01
ENSBTAG00000044078	4.49E-01	6.26E-01	9.32E-01	4.36E-03	7.64E-01	2.37E-02	9.95E-01	2.49E-03	1.20E-01	1.00E+00
ENSBTAG00000036183	3.27E-01	5.76E-01	2.19E-01	4.82E-01	-2.58E-01	3.84E-01	-4.94E-01	4.10E-02	-7.90E-02	1.00E+00

ENSBTAG00000047484	1.32E+00	9.29E-01	8.81E-01	8.52E-01	-1.30E-01	1.00E+00	1.12E+00	6.71E-01	7.99E-01	1.00E+00
ENSBTAG00000043976	-6.89E-01	3.43E-01	-1.78E+00	1.46E-05	-1.52E+00	8.27E-05	-1.36E+00	4.64E-04	3.82E-01	8.00E-01
ENSBTAG00000047139	-1.87E-01	1.00E+00	-4.18E-01	1.40E-01	-3.64E-01	2.19E-01	-4.01E-01	1.71E-01	-1.61E-01	1.00E+00
ENSBTAG00000044329	0.00E+00	1.00E+00	0.00E+00	1.00E+00	-5.13E-15	1.00E+00	0.00E+00	1.00E+00	0.00E+00	1.00E+00
ENSBTAG00000001888	-5.55E-02	1.00E+00	-2.43E-02	1.00E+00	2.82E-01	4.17E-01	2.56E-01	4.93E-01	1.93E-01	9.85E-01
ENSBTAG00000037250	0.00E+00	1.00E+00	0.00E+00	1.00E+00	-5.13E-15	1.00E+00	0.00E+00	1.00E+00	0.00E+00	1.00E+00
ENSBTAG00000043390	-1.91E+00	8.23E-01	6.97E-02	1.00E+00	-1.91E+00	4.28E-01	-1.91E+00	4.39E-01	7.93E-01	1.00E+00
ENSBTAG00000021706	3.38E-01	8.23E-01	-2.72E-01	5.55E-01	1.92E-01	7.76E-01	1.04E+00	6.14E-04	3.60E-01	7.80E-01
ENSBTAG00000011384	-1.29E+00	9.37E-01	1.72E+00	1.21E-01	2.09E-01	1.00E+00	1.97E+00	4.95E-02	6.91E-01	1.00E+00
ENSBTAG00000031824	-3.58E-02	1.00E+00	9.20E-01	4.08E-05	1.29E+00	3.17E-09	1.32E+00	1.77E-09	-7.76E-02	1.00E+00
ENSBTAG00000003107	-1.91E+00	8.23E-01	8.74E-01	8.53E-01	-1.31E-01	1.00E+00	1.11E+00	6.72E-01	4.64E-04	1.00E+00
ENSBTAG00000008945	-4.25E-02	1.00E+00	3.19E+00	6.50E-12	3.68E+00	5.30E-16	2.23E+00	5.32E-06	-6.85E-02	1.00E+00
ENSBTAG00000031814	2.13E+00	5.06E-10	4.67E+00	6.05E-41	6.36E+00	6.83E-65	5.81E+00	4.85E-57	2.11E+00	3.46E-09
ENSBTAG00000019040	6.54E-02	1.00E+00	2.69E-01	3.08E-01	5.57E-01	8.50E-03	6.11E-01	4.02E-03	2.82E-01	6.60E-01
ENSBTAG00000016738	4.77E-01	8.23E-01	1.21E+00	4.46E-03	2.37E+00	2.35E-09	1.89E+00	3.22E-06	1.45E-01	1.00E+00
ENSBTAG00000016739	7.78E-01	5.76E-01	5.52E-01	4.49E-01	2.44E-01	9.34E-01	-1.19E-01	1.00E+00	-1.04E-01	1.00E+00
ENSBTAG00000011924	-6.84E-02	1.00E+00	8.17E-01	3.73E-03	1.28E+00	9.92E-07	1.00E+00	2.36E-04	-6.94E-01	2.05E-01
ENSBTAG00000011930	2.70E-02	1.00E+00	9.68E-01	8.06E-06	1.15E+00	7.53E-08	1.12E+00	2.04E-07	-2.51E-01	8.00E-01
ENSBTAG00000011937	1.29E+00	2.15E-05	2.65E+00	6.44E-24	2.59E+00	3.84E-23	1.91E+00	6.22E-13	1.26E-01	1.00E+00
ENSBTAG00000001806	-4.61E-01	9.28E-01	1.75E-01	1.00E+00	5.14E-01	4.26E-01	1.92E-01	9.94E-01	-6.30E-01	7.77E-01
ENSBTAG00000011102	1.73E-01	1.00E+00	-2.52E+00	2.85E-26	-2.50E+00	9.43E-27	-2.11E+00	4.64E-20	6.57E-01	6.01E-02
ENSBTAG00000011103	3.49E-01	5.29E-01	2.42E-01	4.43E-01	-1.22E-01	8.60E-01	-2.03E-01	5.59E-01	2.01E-01	8.97E-01
ENSBTAG00000014628	-1.79E-02	1.00E+00	1.36E+00	1.09E-02	2.11E-01	9.79E-01	-7.88E-01	2.12E-01	1.16E-02	1.00E+00
ENSBTAG00000039861	-2.78E-01	1.00E+00	6.09E-01	5.79E-01	-3.73E-02	1.00E+00	-9.03E-01	3.28E-01	-1.93E-01	1.00E+00
ENSBTAG00000037527	6.87E-01	5.76E-01	1.06E+00	3.07E-02	3.76E-01	6.20E-01	-2.45E-01	8.81E-01	7.62E-01	5.09E-01
ENSBTAG00000004247	1.65E+00	5.76E-01	8.37E-02	1.00E+00	1.66E+00	2.05E-01	1.44E+00	3.27E-01	2.52E+00	1.28E-01
ENSBTAG00000002048	1.87E-01	1.00E+00	7.71E-01	4.94E-03	1.17E+00	7.78E-06	1.44E+00	2.82E-08	3.90E-01	6.06E-01
ENSBTAG00000042345	0.00E+00	1.00E+00	0.00E+00	1.00E+00	-5.13E-15	1.00E+00	0.00E+00	1.00E+00	0.00E+00	1.00E+00
ENSBTAG00000031723	8.97E-02	1.00E+00	5.49E-01	9.91E-02	4.27E-01	2.37E-01	4.64E-01	1.97E-01	-2.97E-01	8.24E-01
ENSBTAG00000043928	0.00E+00	1.00E+00	1.99E+00	4.43E-01	-5.13E-15	1.00E+00	0.00E+00	1.00E+00	0.00E+00	1.00E+00
ENSBTAG00000010958	2.30E-01	1.00E+00	-7.38E-01	7.30E-02	-3.17E-01	5.60E-01	-5.28E-01	2.39E-01	4.60E-01	6.93E-01
ENSBTAG00000046730	2.71E-01	1.00E+00	-5.05E-01	2.35E-01	-7.05E-01	6.42E-02	-4.47E-01	3.23E-01	5.54E-01	5.24E-01
ENSBTAG00000008762	1.74E-01	1.00E+00	-4.78E-01	1.34E-01	-5.44E-01	7.59E-02	-6.56E-01	2.87E-02	6.17E-01	2.25E-01
ENSBTAG00000008761	1.59E-01	1.00E+00	-6.35E-01	3.15E-03	-2.68E-01	3.23E-01	-3.63E-01	1.43E-01	6.76E-02	1.00E+00
ENSBTAG00000006666	1.27E-01	1.00E+00	1.14E+00	5.12E-06	1.32E+00	8.27E-08	8.73E-01	7.94E-04	-2.43E-01	8.73E-01

ENSBTAG00000006665	7.82E-03	1.00E+00	1.75E-01	7.36E-01	7.10E-02	1.00E+00	1.55E-01	8.09E-01	3.89E-01	5.92E-01
ENSBTAG00000006659	-1.41E-01	1.00E+00	-1.01E+00	2.41E-02	-1.37E+00	1.37E-03	-9.66E-01	2.91E-02	3.51E-01	8.87E-01
ENSBTAG00000043619	0.00E+00	1.00E+00	0.00E+00	1.00E+00	-5.13E-15	1.00E+00	0.00E+00	1.00E+00	0.00E+00	1.00E+00
ENSBTAG00000031693	-1.43E+00	7.58E-01	-4.66E-01	9.45E-01	-1.59E+00	2.60E-01	-1.62E+00	2.61E-01	-9.07E-03	1.00E+00
ENSBTAG00000009346	-1.44E-01	1.00E+00	8.52E-02	9.68E-01	1.81E-01	5.37E-01	-1.03E-02	1.00E+00	3.16E-01	5.43E-01
ENSBTAG00000008743	-8.04E-02	1.00E+00	-2.82E-01	3.10E-01	-7.72E-01	3.18E-04	-9.62E-01	6.19E-06	-8.76E-02	1.00E+00
ENSBTAG00000003696	-2.73E-01	9.36E-01	-8.66E-01	6.81E-03	-1.33E+00	1.47E-05	-1.23E+00	7.40E-05	-4.99E-01	5.36E-01
ENSBTAG00000022044	-6.99E-03	1.00E+00	5.87E-01	4.49E-03	6.67E-01	9.56E-04	6.72E-01	1.08E-03	9.08E-02	1.00E+00
ENSBTAG00000017379	2.81E-02	1.00E+00	7.90E-01	1.82E-04	9.29E-01	7.28E-06	8.58E-01	4.97E-05	1.64E-01	9.85E-01
ENSBTAG00000042215	0.00E+00	1.00E+00	0.00E+00	1.00E+00	-5.13E-15	1.00E+00	0.00E+00	1.00E+00	0.00E+00	1.00E+00
ENSBTAG00000017389	-6.35E-02	1.00E+00	1.81E-01	5.87E-01	2.93E-01	2.56E-01	5.71E-01	9.33E-03	-5.84E-02	1.00E+00
ENSBTAG00000018108	-2.05E-01	9.77E-01	-1.08E+00	1.12E-05	-1.09E+00	7.69E-06	-5.16E-01	6.31E-02	7.36E-01	4.48E-02
ENSBTAG00000048167	-1.99E+00	2.53E-02	-7.39E-01	3.97E-01	-2.34E+00	6.27E-04	-2.91E+00	4.13E-05	-3.60E+00	6.77E-05
ENSBTAG00000021168	-1.02E-01	1.00E+00	-1.05E+00	4.61E-04	-2.00E-01	7.28E-01	2.33E-02	1.00E+00	-3.27E-01	8.00E-01
ENSBTAG00000046842	0.00E+00	1.00E+00	0.00E+00	1.00E+00	-5.13E-15	1.00E+00	0.00E+00	1.00E+00	0.00E+00	1.00E+00
ENSBTAG00000026732	-7.80E-01	9.44E-01	1.23E+00	1.17E-01	-2.33E-02	1.00E+00	-2.14E+00	6.92E-02	1.51E-01	1.00E+00
ENSBTAG00000047084	0.00E+00	1.00E+00	1.99E+00	4.43E-01	-5.13E-15	1.00E+00	0.00E+00	1.00E+00	0.00E+00	1.00E+00
ENSBTAG00000009800	5.53E-01	1.00E+00	8.48E-01	5.91E-01	9.16E-01	4.95E-01	1.87E+00	4.03E-02	-3.81E-01	1.00E+00
ENSBTAG00000012788	-9.63E-02	1.00E+00	-1.86E-01	6.85E-01	-3.19E-01	3.22E-01	-1.53E-01	8.07E-01	2.94E-02	1.00E+00
ENSBTAG00000012790	-6.93E-02	1.00E+00	-5.81E-01	3.83E-03	-7.40E-01	1.38E-04	-6.09E-01	2.63E-03	-2.97E-01	6.01E-01
ENSBTAG00000012791	-2.26E-01	9.51E-01	-4.16E-01	1.85E-01	2.83E-01	4.28E-01	4.95E-01	9.33E-02	-1.74E-01	1.00E+00
ENSBTAG00000012792	1.07E-01	1.00E+00	2.81E-01	3.88E-01	2.04E-01	5.91E-01	1.53E-01	7.89E-01	-3.43E-01	6.25E-01
ENSBTAG00000024605	4.97E-01	3.98E-01	1.74E+00	5.85E-11	1.04E+00	1.61E-04	8.99E-01	1.66E-03	2.70E-01	8.64E-01
ENSBTAG00000015761	3.70E-02	1.00E+00	-5.70E-02	1.00E+00	-1.90E-01	6.70E-01	-1.20E-03	1.00E+00	6.23E-02	1.00E+00
ENSBTAG00000015767	-8.89E-02	1.00E+00	-2.66E-02	1.00E+00	-7.44E-02	1.00E+00	6.52E-02	1.00E+00	-1.47E-03	1.00E+00
ENSBTAG00000005385	-1.61E-01	1.00E+00	3.56E-01	2.51E-01	7.88E-01	1.79E-03	7.43E-01	4.21E-03	-5.30E-01	3.07E-01
ENSBTAG00000020049	1.17E+00	8.67E-01	7.55E-02	1.00E+00	1.46E+00	3.08E-01	2.05E+00	7.91E-02	5.38E-03	1.00E+00
ENSBTAG00000020050	4.38E-01	7.46E-01	1.25E+00	3.26E-04	1.35E+00	9.91E-05	1.58E+00	5.39E-06	7.32E-01	2.99E-01
ENSBTAG00000007483	-2.50E-01	9.60E-01	4.15E-01	2.51E-01	4.61E-01	1.84E-01	7.61E-01	1.40E-02	2.13E-01	1.00E+00
ENSBTAG00000007484	-2.17E-01	8.78E-01	-4.32E-01	8.32E-02	-1.29E+00	1.88E-09	-1.71E+00	2.11E-15	-2.73E-01	7.74E-01
ENSBTAG00000016338	-2.75E-01	7.14E-01	-1.03E+00	9.22E-07	-5.58E-01	1.14E-02	-5.10E-01	2.62E-02	1.84E-01	9.10E-01
ENSBTAG00000045977	-1.91E+00	8.23E-01	7.58E-02	1.00E+00	-1.91E+00	4.28E-01	6.22E-01	9.94E-01	7.05E-03	1.00E+00
ENSBTAG00000021795	-1.03E+00	2.16E-03	-2.37E+00	2.57E-17	-1.83E+00	3.71E-11	-6.30E-01	4.44E-02	4.49E-01	5.59E-01
ENSBTAG00000021796	-1.08E-01	1.00E+00	5.49E-01	1.85E-02	7.68E-01	4.46E-04	7.79E-01	4.46E-04	9.40E-02	1.00E+00
ENSBTAG00000003297	3.11E-01	1.00E+00	4.03E-01	8.02E-01	6.64E-01	4.46E-01	-1.46E-01	1.00E+00	1.38E-01	1.00E+00

ENSBTAG00000046887	0.00E+00	1.00E+00	0.00E+00	1.00E+00	-5.13E-15	1.00E+00	0.00E+00	1.00E+00	0.00E+00	1.00E+00
ENSBTAG00000032047	-5.03E-01	4.78E-01	6.78E-01	2.71E-02	8.98E-01	1.68E-03	4.14E-01	2.57E-01	-2.05E-01	1.00E+00
ENSBTAG00000014376	-3.47E-02	1.00E+00	-5.40E-01	1.05E-01	5.18E-01	1.16E-01	9.75E-01	9.10E-04	5.67E-01	3.60E-01
ENSBTAG00000006506	1.73E-02	1.00E+00	5.72E-02	1.00E+00	7.66E-01	5.18E-03	4.72E-01	1.33E-01	-9.75E-03	1.00E+00
ENSBTAG00000006504	5.13E-02	1.00E+00	6.32E-01	1.01E-01	1.02E+00	3.01E-03	6.19E-01	1.17E-01	1.46E-01	1.00E+00
ENSBTAG00000002591	1.52E-01	1.00E+00	-3.95E-01	1.01E-01	-3.44E-01	1.67E-01	-1.64E-01	6.67E-01	1.28E-01	1.00E+00
ENSBTAG00000000031	4.41E-02	1.00E+00	7.00E-01	2.15E-01	-9.03E-01	1.33E-01	-2.19E-01	9.56E-01	-7.93E-02	1.00E+00
ENSBTAG00000031993	1.97E-01	1.00E+00	1.22E+00	2.02E-03	2.23E+00	5.46E-10	1.29E+00	1.01E-03	2.33E-02	1.00E+00
ENSBTAG00000005183	9.50E-03	1.00E+00	-8.31E-02	1.00E+00	5.18E-01	2.54E-01	5.56E-01	2.20E-01	-4.70E-01	7.34E-01
ENSBTAG00000005178	2.38E-02	1.00E+00	-4.03E-01	1.87E-01	-8.18E-01	1.54E-03	-1.37E+00	4.78E-08	-1.94E-01	9.91E-01
ENSBTAG00000002698	-4.22E-02	1.00E+00	3.84E-01	2.21E-01	1.25E+00	5.08E-07	7.51E-01	5.14E-03	6.80E-01	1.04E-01
ENSBTAG00000002697	3.01E-01	7.83E-01	1.32E+00	6.01E-08	2.23E+00	1.92E-20	1.43E+00	4.04E-09	-2.71E-01	8.00E-01
ENSBTAG00000002695	-4.95E-01	1.00E+00	1.41E+00	2.58E-01	1.14E+00	4.14E-01	4.79E-01	9.69E-01	1.51E+00	5.97E-01
ENSBTAG00000010847	-1.05E-03	1.00E+00	1.77E+00	3.28E-01	1.50E+00	4.32E-01	1.78E+00	3.04E-01	7.92E-01	1.00E+00
ENSBTAG00000022058	1.45E+00	5.02E-01	2.08E+00	3.38E-02	2.26E+00	1.99E-02	3.72E+00	1.26E-04	2.27E+00	1.53E-01
ENSBTAG00000019848	-3.77E-01	8.23E-01	-1.96E+00	6.40E-08	-5.88E-01	1.64E-01	7.74E-01	5.01E-02	-1.94E-01	1.00E+00
ENSBTAG00000019846	-4.36E-01	4.38E-01	-1.62E+00	1.58E-10	-1.46E+00	6.41E-09	-1.11E+00	1.50E-05	-9.31E-02	1.00E+00
ENSBTAG00000019845	1.70E-02	1.00E+00	-6.58E-01	1.72E-03	-5.11E-01	1.86E-02	-3.30E-01	1.86E-01	3.81E-01	4.16E-01
ENSBTAG00000018833	-4.26E-01	9.62E-01	-1.51E+00	1.14E-02	-1.67E+00	3.43E-03	-6.06E-01	3.75E-01	5.39E-01	8.00E-01
ENSBTAG00000018831	-7.78E-01	8.92E-02	-3.86E+00	9.53E-32	-6.45E+00	1.23E-62	-4.87E+00	5.73E-45	2.68E-01	9.13E-01
ENSBTAG00000018825	1.88E-01	1.00E+00	-8.05E-01	7.27E-03	-4.30E-01	2.12E-01	-1.73E-01	8.12E-01	6.33E-01	2.32E-01
ENSBTAG00000007993	1.31E-01	1.00E+00	6.08E-01	1.47E-02	-5.81E-01	2.16E-02	-4.19E-01	1.36E-01	-1.10E-01	1.00E+00
ENSBTAG00000023431	0.00E+00	1.00E+00	0.00E+00	1.00E+00	-5.13E-15	1.00E+00	0.00E+00	1.00E+00	0.00E+00	1.00E+00
ENSBTAG00000031851	-6.19E-02	1.00E+00	1.53E-01	8.77E-01	3.19E-01	4.18E-01	4.68E-01	1.65E-01	1.88E-01	1.00E+00
ENSBTAG00000031849	-9.68E-01	5.75E-01	-1.97E+00	1.13E-02	-1.78E+00	1.38E-02	-1.46E+00	4.42E-02	-5.39E-01	8.99E-01
ENSBTAG00000031846	-1.21E-01	1.00E+00	4.88E-01	2.67E-02	1.09E+00	4.12E-08	6.27E-01	3.37E-03	-3.39E-02	1.00E+00
ENSBTAG00000011771	9.61E-01	4.95E-03	2.52E+00	3.06E-20	1.42E+00	2.00E-07	7.61E-01	1.07E-02	6.00E-01	2.92E-01
ENSBTAG00000011787	-2.00E-01	9.82E-01	-5.85E-01	2.37E-02	-7.78E-01	1.59E-03	-8.21E-01	9.79E-04	-3.41E-01	6.43E-01
ENSBTAG00000022039	-1.67E+00	2.66E-06	-1.69E+00	2.75E-07	-3.81E-01	3.37E-01	-5.99E-01	8.70E-02	-6.08E-01	3.63E-01
ENSBTAG00000047446	0.00E+00	1.00E+00	0.00E+00	1.00E+00	1.78E+00	4.79E-01	1.76E+00	4.97E-01	1.93E+00	8.00E-01
ENSBTAG00000013686	-3.64E-01	8.23E-01	-7.25E-01	4.02E-02	-1.20E+00	2.64E-04	-1.08E+00	1.29E-03	-4.09E-01	7.36E-01
ENSBTAG00000015526	1.79E-02	1.00E+00	6.04E-01	9.45E-01	1.02E+00	5.89E-01	3.25E-01	1.00E+00	-7.85E-01	1.00E+00
ENSBTAG00000006592	6.55E-01	4.39E-01	-2.03E+00	1.19E-05	-1.32E+00	2.46E-03	-6.21E-01	2.17E-01	4.61E-01	7.93E-01
ENSBTAG00000006594	2.72E+00	5.76E-01	0.00E+00	1.00E+00	1.78E+00	4.79E-01	2.53E+00	2.71E-01	0.00E+00	1.00E+00
ENSBTAG00000044022	5.83E-01	7.69E-01	4.88E-01	4.69E-01	5.78E-01	3.61E-01	8.57E-01	1.24E-01	1.26E+00	1.07E-01

ENSBTAG00000043857	0.00E+00	1.00E+00	0.00E+00	1.00E+00	-5.13E-15	1.00E+00	0.00E+00	1.00E+00	0.00E+00	1.00E+00
ENSBTAG00000001255	1.45E+00	4.41E-01	7.09E-01	6.67E-01	2.71E+00	6.48E-04	1.06E+00	3.78E-01	1.06E+00	7.83E-01
ENSBTAG00000004088	5.86E-01	1.00E+00	-1.73E-01	1.00E+00	5.94E-01	8.76E-01	-8.52E-01	7.61E-01	1.39E+00	7.25E-01
ENSBTAG00000000005	4.12E-01	4.85E-01	3.62E-01	2.47E-01	1.68E-01	7.55E-01	4.89E-01	8.73E-02	4.29E-01	4.91E-01
ENSBTAG00000000698	2.93E-01	1.00E+00	3.56E-01	7.42E-01	-3.23E-02	1.00E+00	3.65E-01	7.20E-01	-3.02E-02	1.00E+00
ENSBTAG00000006569	-5.30E-01	1.00E+00	-1.38E+00	2.67E-01	2.50E-01	1.00E+00	-1.28E+00	2.80E-01	-7.98E-01	9.01E-01
ENSBTAG00000037832	1.39E+00	8.23E-01	1.55E+00	3.35E-01	2.08E+00	1.53E-01	1.98E+00	1.90E-01	1.03E+00	9.28E-01
ENSBTAG00000017038	3.10E-01	6.85E-01	6.23E-01	9.76E-03	8.31E-01	3.08E-04	4.86E-01	6.10E-02	4.36E-01	4.09E-01
ENSBTAG00000012277	4.43E-01	3.44E-01	1.74E+00	9.54E-15	1.05E+00	3.97E-06	4.99E-01	5.85E-02	3.36E-01	6.32E-01
ENSBTAG00000012288	1.10E-01	1.00E+00	5.68E-01	8.25E-03	7.78E-01	1.45E-04	6.53E-01	2.22E-03	2.31E-01	8.00E-01
ENSBTAG00000010368	8.99E-02	1.00E+00	-3.99E-01	3.69E-01	-1.18E-01	1.00E+00	-4.92E-02	1.00E+00	-1.14E-01	1.00E+00
ENSBTAG00000019280	-6.36E-02	1.00E+00	-9.74E-01	1.82E-02	-5.38E-01	2.30E-01	-5.67E-01	2.07E-01	-5.33E-01	6.25E-01
ENSBTAG00000019282	-5.69E-01	9.70E-01	-2.16E+00	7.71E-03	-3.33E+00	9.32E-05	-1.26E+00	1.25E-01	-2.48E-01	1.00E+00
ENSBTAG00000031468	4.27E-01	1.00E+00	5.56E+00	6.94E-02	4.29E+00	1.63E-01	4.40E+00	1.61E-01	8.19E-01	1.00E+00
ENSBTAG00000044660	0.00E+00	1.00E+00	0.00E+00	1.00E+00	-5.13E-15	1.00E+00	0.00E+00	1.00E+00	0.00E+00	1.00E+00
ENSBTAG00000010464	-1.75E-01	1.00E+00	-1.37E+00	8.90E-03	-4.90E-01	4.52E-01	1.97E+00	3.38E-06	-5.87E-01	7.62E-01
ENSBTAG00000017799	2.71E-01	6.32E-01	7.60E-01	6.70E-05	8.66E-01	3.92E-06	6.05E-01	2.34E-03	-1.25E-01	1.00E+00
ENSBTAG00000012193	1.82E-01	9.91E-01	-4.32E-01	9.17E-02	-7.49E-01	1.09E-03	-4.56E-01	7.54E-02	4.17E-01	4.15E-01
ENSBTAG00000004956	1.79E-03	1.00E+00	-4.06E-01	4.69E-01	-9.82E-02	1.00E+00	-2.14E-01	8.70E-01	2.85E-01	1.00E+00
ENSBTAG00000015075	-6.60E-02	1.00E+00	4.50E-01	7.83E-02	5.29E-01	2.93E-02	4.45E-01	8.66E-02	-9.13E-03	1.00E+00
ENSBTAG00000003557	1.44E+00	1.07E-06	3.21E+00	8.44E-31	1.38E+00	3.39E-07	1.05E+00	1.73E-04	4.60E-02	1.00E+00
ENSBTAG00000005970	1.63E+00	1.92E-06	3.70E+00	1.26E-29	4.12E+00	2.45E-35	3.66E+00	3.81E-29	7.38E-01	2.05E-01
ENSBTAG00000022036	-1.31E-01	1.00E+00	-1.54E+00	7.10E-05	-2.00E-01	8.75E-01	6.37E-01	1.50E-01	1.79E-01	1.00E+00
ENSBTAG00000012892	5.07E-01	7.59E-01	-7.57E-01	1.30E-01	-4.40E-01	4.62E-01	-8.01E-01	1.04E-01	-1.99E-01	1.00E+00
ENSBTAG00000009773	-2.26E-01	1.00E+00	-1.55E+00	9.49E-05	-5.64E-01	2.29E-01	2.70E-01	7.24E-01	5.48E-01	6.13E-01
ENSBTAG00000017738	5.10E-01	7.81E-01	9.06E-01	5.12E-02	1.18E-01	1.00E+00	2.04E-01	9.30E-01	1.52E-01	1.00E+00
ENSBTAG00000017750	-2.87E-01	7.33E-01	-6.87E-01	2.88E-03	-7.25E-01	1.45E-03	-7.89E-01	5.73E-04	-3.04E-01	6.83E-01
ENSBTAG00000017755	-6.41E-02	1.00E+00	-4.43E-01	3.03E-02	2.73E-01	2.49E-01	1.77E-01	5.49E-01	-3.59E-02	1.00E+00
ENSBTAG00000012504	-1.53E-01	1.00E+00	-8.56E-01	2.56E-03	-7.52E-02	1.00E+00	7.83E-01	6.65E-03	1.64E-01	1.00E+00
ENSBTAG00000001187	-3.59E-01	9.36E-01	-2.21E+00	2.40E-07	-1.77E-01	9.60E-01	-7.06E-01	1.41E-01	4.27E-01	8.00E-01
ENSBTAG00000001189	-1.81E-01	1.00E+00	-4.37E-01	8.53E-02	4.68E-02	1.00E+00	2.19E-01	5.15E-01	1.98E-02	1.00E+00
ENSBTAG00000045197	0.00E+00	1.00E+00	0.00E+00	1.00E+00	-5.13E-15	1.00E+00	0.00E+00	1.00E+00	0.00E+00	1.00E+00
ENSBTAG00000013147	-3.82E-01	7.83E-01	-1.59E+00	1.46E-06	-4.19E+00	3.13E-27	-4.49E+00	2.61E-29	7.64E-01	1.58E-01
ENSBTAG00000013150	-7.94E-02	1.00E+00	2.34E-01	4.01E-01	6.80E-01	7.48E-04	6.27E-01	2.56E-03	-1.67E-02	1.00E+00
ENSBTAG00000013152	-1.16E-01	1.00E+00	-1.01E+00	8.81E-05	-2.58E+00	5.53E-24	-2.42E+00	2.50E-21	4.87E-02	1.00E+00

ENSBTAG00000013153	3.38E-01	6.26E-01	7.13E-01	3.67E-03	4.65E-01	8.39E-02	8.09E-01	9.21E-04	4.18E-01	4.70E-01
ENSBTAG00000017886	-3.92E-02	1.00E+00	3.09E+00	7.18E-13	1.39E+00	4.12E-03	6.02E-01	3.77E-01	-4.62E-01	9.43E-01
ENSBTAG00000017890	-7.26E-03	1.00E+00	2.77E-01	4.78E-01	5.00E-01	1.06E-01	6.13E-01	4.05E-02	2.03E-02	1.00E+00
ENSBTAG00000000913	3.34E-02	1.00E+00	-7.10E-02	1.00E+00	-1.07E-01	9.47E-01	9.40E-03	1.00E+00	2.87E-02	1.00E+00
ENSBTAG00000002452	3.03E-01	6.86E-01	1.60E+00	2.37E-13	1.18E+00	7.02E-08	5.00E-01	4.73E-02	7.93E-03	1.00E+00
ENSBTAG00000043525	0.00E+00	1.00E+00	1.98E+00	4.43E-01	-5.13E-15	1.00E+00	0.00E+00	1.00E+00	0.00E+00	1.00E+00
ENSBTAG00000001030	1.32E-01	1.00E+00	6.53E-01	6.74E-03	5.33E-01	3.31E-02	3.18E-01	2.90E-01	2.21E-01	8.69E-01
ENSBTAG00000013877	-1.37E-01	1.00E+00	3.82E-01	8.38E-01	1.20E-01	1.00E+00	-9.78E-02	1.00E+00	-4.07E-01	1.00E+00
ENSBTAG00000007424	-3.55E-01	1.00E+00	-2.25E-01	1.00E+00	1.96E+00	5.50E-02	4.57E+00	2.43E-06	-4.12E-01	1.00E+00
ENSBTAG00000016163	2.93E-01	1.00E+00	-2.12E-01	1.00E+00	1.15E+00	4.19E-01	4.38E+00	1.71E-05	-1.46E+00	7.98E-01
ENSBTAG00000018084	-4.97E-01	2.20E-01	-1.99E+00	3.82E-18	-3.12E+00	1.20E-39	-2.24E+00	2.04E-22	2.75E-03	1.00E+00
ENSBTAG00000018086	-3.69E-01	7.81E-01	-1.48E+00	1.34E-06	-7.55E-01	2.09E-02	-1.68E-01	8.71E-01	1.80E-01	1.00E+00
ENSBTAG00000003390	-1.72E-01	9.52E-01	4.31E-01	4.51E-02	5.53E-01	6.79E-03	4.59E-01	3.45E-02	3.15E-01	5.58E-01
ENSBTAG00000003393	1.68E-01	1.00E+00	-7.31E-01	9.10E-02	-2.55E+00	1.15E-09	-2.42E+00	6.58E-09	-3.97E-01	8.00E-01
ENSBTAG00000003394	-3.69E-01	5.52E-01	-1.45E+00	9.83E-10	-2.19E+00	7.23E-20	-1.77E+00	9.09E-14	-1.96E-01	9.43E-01
ENSBTAG00000017404	-3.13E-01	4.52E-01	-1.96E+00	7.67E-28	-3.38E+00	5.52E-72	-1.63E+00	7.03E-20	2.99E-01	5.49E-01
ENSBTAG00000018116	-5.60E-01	3.27E-01	-1.27E+00	1.57E-05	-2.32E+00	3.94E-15	-1.92E+00	6.46E-11	-2.11E-01	1.00E+00
ENSBTAG00000014464	-1.44E-01	1.00E+00	-1.05E+00	5.09E-02	-1.96E+00	7.81E-05	-2.39E+00	2.18E-06	3.07E-01	1.00E+00
ENSBTAG00000031351	9.01E-01	3.33E-01	1.37E+00	5.46E-03	1.26E+00	1.15E-02	1.02E+00	5.45E-02	6.89E-01	6.13E-01
ENSBTAG00000037993	0.00E+00	1.00E+00	0.00E+00	1.00E+00	-5.13E-15	1.00E+00	0.00E+00	1.00E+00	0.00E+00	1.00E+00
ENSBTAG00000014232	-6.40E-01	4.07E-01	-2.66E+00	3.55E-11	-3.24E+00	2.01E-15	-2.21E+00	8.67E-09	5.04E-01	6.34E-01
ENSBTAG00000007728	2.09E-02	1.00E+00	1.85E+00	1.05E-22	2.04E+00	1.54E-27	1.82E+00	3.30E-22	-2.84E-01	6.23E-01
ENSBTAG00000002770	-2.26E-01	8.02E-01	-6.02E-01	2.00E-03	-9.52E-01	2.99E-07	-1.17E+00	2.92E-10	-1.83E-01	8.62E-01
ENSBTAG00000002385	1.53E-01	1.00E+00	2.75E+00	7.60E-08	1.02E+00	7.20E-02	-5.05E-01	5.20E-01	-2.17E-01	1.00E+00
ENSBTAG00000017459	7.65E-01	3.57E-01	1.87E+00	8.33E-07	1.32E+00	9.05E-04	1.68E+00	1.33E-05	3.32E-01	9.82E-01
ENSBTAG00000017438	-9.77E-01	3.44E-02	-1.07E+00	2.35E-03	-9.28E-01	8.17E-03	-4.68E-01	2.71E-01	-8.48E-02	1.00E+00
ENSBTAG00000019146	5.07E-01	8.23E-01	7.60E-01	1.57E-01	-4.70E-03	1.00E+00	3.18E-01	7.46E-01	-4.80E-01	8.60E-01
ENSBTAG00000009963	-4.24E-02	1.00E+00	1.11E+00	2.43E-09	1.46E+00	1.92E-15	4.92E-01	1.75E-02	3.06E-01	5.66E-01
ENSBTAG00000003100	-1.74E-01	1.00E+00	-1.76E+00	3.58E-14	-8.43E-01	3.71E-04	-9.29E-01	9.55E-05	-1.30E-01	1.00E+00
ENSBTAG00000031279	-1.25E-01	1.00E+00	-3.23E-01	5.65E-01	2.91E-01	6.12E-01	2.21E-01	7.99E-01	-4.47E-01	7.70E-01
ENSBTAG00000017023	4.75E-01	1.00E+00	9.12E-02	1.00E+00	-1.75E-01	1.00E+00	4.55E-01	9.13E-01	-6.65E-01	1.00E+00
ENSBTAG00000011962	1.91E+00	8.23E-01	0.00E+00	1.00E+00	1.78E+00	4.79E-01	0.00E+00	1.00E+00	1.91E+00	8.00E-01
ENSBTAG00000002828	-3.55E-01	4.47E-01	-3.28E-03	1.00E+00	-8.54E-02	9.88E-01	-1.36E-01	7.81E-01	1.13E-01	1.00E+00
ENSBTAG00000010662	-3.16E-01	6.87E-01	-1.13E+00	2.08E-06	-3.10E-01	3.05E-01	4.26E-01	1.17E-01	3.71E-02	1.00E+00
ENSBTAG00000010667	-1.46E+00	1.70E-05	-3.18E+00	1.84E-21	-2.80E+00	1.38E-18	-2.39E+00	2.20E-14	-2.15E-01	1.00E+00

ENSBTAG00000005478	-3.19E-01	8.09E-01	-1.38E+00	8.17E-07	-1.00E+00	3.01E-04	-8.90E-01	1.77E-03	1.35E-01	1.00E+00
ENSBTAG00000008990	-8.48E-02	1.00E+00	1.17E+00	1.83E-07	1.72E+00	2.12E-15	1.57E+00	7.33E-13	-3.56E-01	6.07E-01
ENSBTAG00000008991	-1.94E-01	8.95E-01	3.92E-01	8.47E-02	4.39E-01	4.62E-02	4.71E-02	1.00E+00	2.02E-01	8.34E-01
ENSBTAG00000008996	-3.35E-01	8.23E-01	-5.84E-01	1.03E-01	-4.91E-01	1.90E-01	-5.18E-01	1.68E-01	2.31E-01	9.99E-01
ENSBTAG00000008998	3.96E-01	3.98E-01	1.09E+00	5.63E-07	1.02E+00	2.88E-06	9.28E-01	2.97E-05	-1.07E-01	1.00E+00
ENSBTAG00000009006	-3.16E-01	7.05E-01	-8.68E-03	1.00E+00	1.47E+00	6.47E-11	1.07E+00	4.33E-06	9.48E-02	1.00E+00
ENSBTAG00000001295	5.67E-03	1.00E+00	-1.29E+00	3.59E-07	-3.72E-01	2.34E-01	4.80E-01	1.00E-01	1.23E-01	1.00E+00
ENSBTAG00000043690	0.00E+00	1.00E+00	0.00E+00	1.00E+00	-5.13E-15	1.00E+00	0.00E+00	1.00E+00	0.00E+00	1.00E+00
ENSBTAG00000045372	0.00E+00	1.00E+00	0.00E+00	1.00E+00	-5.13E-15	1.00E+00	0.00E+00	1.00E+00	0.00E+00	1.00E+00
ENSBTAG00000031134	-1.59E-01	1.00E+00	-1.08E-01	9.45E-01	3.54E-01	2.05E-01	3.85E-01	1.67E-01	-4.52E-01	3.81E-01
ENSBTAG00000012851	-8.04E-01	5.48E-03	-1.73E+00	2.44E-13	-2.54E+00	5.09E-26	-2.58E+00	1.36E-26	1.12E-01	1.00E+00
ENSBTAG00000008117	-5.55E-01	1.00E+00	-4.70E+00	3.28E-03	-1.27E+00	2.75E-01	-1.66E+00	1.51E-01	-1.18E-02	1.00E+00
ENSBTAG00000046584	6.38E-03	1.00E+00	-2.09E-01	1.00E+00	-2.11E+00	1.74E-01	-8.60E-01	6.61E-01	-5.96E-03	1.00E+00
ENSBTAG00000048030	-5.96E-01	8.37E-01	-1.04E+00	1.49E-01	-7.26E-01	3.35E-01	-1.32E+00	5.33E-02	-1.39E-01	1.00E+00
ENSBTAG00000031160	2.37E-01	1.00E+00	1.60E-01	1.00E+00	-3.27E-01	9.99E-01	-3.69E-01	9.61E-01	6.80E-01	9.48E-01
ENSBTAG00000047529	0.00E+00	1.00E+00	0.00E+00	1.00E+00	-5.13E-15	1.00E+00	0.00E+00	1.00E+00	0.00E+00	1.00E+00
ENSBTAG00000039237	0.00E+00	1.00E+00	0.00E+00	1.00E+00	-5.13E-15	1.00E+00	0.00E+00	1.00E+00	0.00E+00	1.00E+00
ENSBTAG00000047986	0.00E+00	1.00E+00	0.00E+00	1.00E+00	-5.13E-15	1.00E+00	0.00E+00	1.00E+00	0.00E+00	1.00E+00
ENSBTAG00000045810	0.00E+00	1.00E+00	0.00E+00	1.00E+00	-5.13E-15	1.00E+00	0.00E+00	1.00E+00	0.00E+00	1.00E+00
ENSBTAG00000001005	0.00E+00	1.00E+00	0.00E+00	1.00E+00	-5.13E-15	1.00E+00	0.00E+00	1.00E+00	0.00E+00	1.00E+00
ENSBTAG00000046083	0.00E+00	1.00E+00	0.00E+00	1.00E+00	-5.13E-15	1.00E+00	0.00E+00	1.00E+00	0.00E+00	1.00E+00
ENSBTAG00000033890	0.00E+00	1.00E+00	0.00E+00	1.00E+00	-5.13E-15	1.00E+00	0.00E+00	1.00E+00	0.00E+00	1.00E+00
ENSBTAG00000047121	0.00E+00	1.00E+00	0.00E+00	1.00E+00	-5.13E-15	1.00E+00	0.00E+00	1.00E+00	0.00E+00	1.00E+00
ENSBTAG00000047718	-1.11E-01	1.00E+00	1.02E+00	6.32E-03	-5.80E-02	1.00E+00	1.82E-01	9.30E-01	2.24E-01	1.00E+00
ENSBTAG00000005128	-5.49E-01	1.00E+00	-4.68E+00	3.50E-03	-2.90E+00	1.89E-02	-1.26E+00	2.90E-01	-5.22E-01	1.00E+00
ENSBTAG00000022030	1.32E+00	9.30E-01	1.39E+00	5.17E-01	2.27E+00	1.12E-01	-1.47E-01	1.00E+00	8.11E-01	1.00E+00
ENSBTAG00000022032	-3.56E-01	8.06E-01	-6.43E-01	5.33E-02	-1.40E+00	3.08E-06	-1.70E+00	1.41E-08	-4.52E-01	6.01E-01
ENSBTAG00000006108	-1.64E+00	7.05E-03	-3.68E+00	1.10E-12	-5.33E+00	1.18E-21	-4.72E+00	3.01E-18	-7.16E-01	6.01E-01
ENSBTAG00000009988	4.89E-02	1.00E+00	1.03E+00	1.26E-05	6.13E-01	1.50E-02	7.29E-01	3.39E-03	-2.06E-01	9.28E-01
ENSBTAG00000022028	1.14E+00	1.26E-01	3.17E+00	2.94E-11	3.48E+00	3.86E-13	3.28E+00	8.70E-12	1.33E+00	7.68E-02
ENSBTAG00000018647	-4.62E-01	1.00E+00	-8.66E-01	4.22E-01	-1.58E+00	8.19E-02	-8.13E-01	4.39E-01	2.64E-01	1.00E+00
ENSBTAG00000007375	-3.24E-01	7.57E-01	-6.47E-01	1.86E-02	-1.29E+00	3.49E-07	-1.48E+00	7.15E-09	-5.83E-01	2.36E-01
ENSBTAG00000000170	-1.15E+00	2.10E-01	-3.89E+00	9.40E-12	-4.38E+00	4.10E-14	-3.66E+00	1.22E-10	2.63E-02	1.00E+00
ENSBTAG00000040298	-6.08E-01	3.47E-01	-1.98E+00	7.49E-10	-3.17E+00	5.36E-22	-3.25E+00	1.01E-22	-2.49E-01	9.88E-01
ENSBTAG00000008587	-5.80E-01	2.32E-01	-9.49E-01	7.62E-04	-1.82E+00	2.19E-11	-2.25E+00	2.63E-16	-5.14E-01	4.30E-01

ENSBTAG00000022027	-2.68E-01	8.23E-01	-2.77E-01	4.01E-01	-6.32E-01	1.14E-02	-9.99E-01	2.91E-05	-1.54E-01	1.00E+00
ENSBTAG00000024708	-2.43E-01	8.23E-01	-4.96E-01	3.79E-02	-9.54E-01	1.13E-05	-9.15E-01	3.48E-05	-2.41E-02	1.00E+00
ENSBTAG00000046067	0.00E+00	1.00E+00	0.00E+00	1.00E+00	-5.13E-15	1.00E+00	0.00E+00	1.00E+00	0.00E+00	1.00E+00
ENSBTAG00000006730	8.28E-01	3.26E-02	1.52E+00	6.39E-08	1.56E+00	1.99E-08	1.22E+00	2.22E-05	1.51E+00	2.91E-06
ENSBTAG00000006608	3.80E-01	5.13E-01	-2.17E-01	5.55E-01	-3.03E-01	3.30E-01	9.36E-02	1.00E+00	6.79E-02	1.00E+00
ENSBTAG00000046900	1.85E-01	9.17E-01	-9.01E-02	9.50E-01	-6.29E-01	1.91E-03	-1.84E-01	5.54E-01	6.43E-01	2.27E-02
ENSBTAG00000021262	2.08E-01	9.71E-01	3.27E-01	3.07E-01	4.61E-01	1.02E-01	5.16E-01	6.53E-02	-1.55E-02	1.00E+00
ENSBTAG00000022022	3.02E-01	6.83E-01	5.92E-02	1.00E+00	1.73E-01	6.66E-01	8.89E-02	1.00E+00	5.17E-01	2.17E-01
ENSBTAG00000001325	-7.81E-02	1.00E+00	-1.08E+00	9.35E-04	-1.69E+00	8.53E-08	-1.92E+00	1.60E-09	8.24E-02	1.00E+00
ENSBTAG00000016944	8.64E-01	1.73E-01	7.60E-01	8.03E-02	4.92E-01	3.33E-01	7.77E-01	7.83E-02	-3.96E-01	8.33E-01
ENSBTAG00000021656	-2.93E-02	1.00E+00	7.98E-03	1.00E+00	6.93E-01	3.45E-03	5.99E-01	1.57E-02	1.41E-01	1.00E+00
ENSBTAG00000045094	0.00E+00	1.00E+00	0.00E+00	1.00E+00	-5.13E-15	1.00E+00	0.00E+00	1.00E+00	0.00E+00	1.00E+00
ENSBTAG00000020566	-2.17E-01	1.00E+00	-9.31E-01	2.19E-03	-7.93E-01	1.07E-02	-1.43E-01	9.29E-01	1.31E-01	1.00E+00
ENSBTAG00000017277	-1.15E-01	1.00E+00	-1.75E+00	1.33E-02	-1.40E+00	3.36E-02	-1.30E+00	5.14E-02	5.92E-01	7.83E-01
ENSBTAG00000036113	3.08E-01	1.00E+00	7.16E-01	1.02E-01	1.48E-01	1.00E+00	6.69E-02	1.00E+00	1.49E-01	1.00E+00
ENSBTAG00000047664	0.00E+00	1.00E+00	0.00E+00	1.00E+00	-5.13E-15	1.00E+00	0.00E+00	1.00E+00	0.00E+00	1.00E+00
ENSBTAG00000023007	1.77E+00	7.40E-02	3.96E-01	9.25E-01	-5.77E-01	7.74E-01	-2.10E-01	1.00E+00	1.85E-01	1.00E+00
ENSBTAG00000017305	9.44E-02	1.00E+00	6.23E-01	2.06E-02	9.57E-01	1.18E-04	3.05E-01	3.85E-01	-6.42E-02	1.00E+00
ENSBTAG00000006938	-4.13E-01	6.21E-01	-3.72E-01	3.32E-01	-1.24E-01	9.73E-01	3.66E-02	1.00E+00	8.36E-02	1.00E+00
ENSBTAG00000042240	-1.67E+00	7.80E-01	-3.59E+00	6.69E-02	-1.03E+00	6.04E-01	-1.87E-01	1.00E+00	-1.67E+00	7.79E-01
ENSBTAG00000010312	-8.47E-02	1.00E+00	1.40E-01	8.09E-01	1.70E-01	6.79E-01	5.19E-01	3.64E-02	4.14E-01	4.34E-01
ENSBTAG00000047805	0.00E+00	1.00E+00	0.00E+00	1.00E+00	-5.13E-15	1.00E+00	0.00E+00	1.00E+00	0.00E+00	1.00E+00
ENSBTAG00000047299	-2.15E-01	9.36E-01	-6.55E-01	9.51E-03	-1.15E+00	1.28E-06	-1.34E+00	2.03E-08	-1.85E-01	9.88E-01
ENSBTAG00000017867	-1.53E-01	1.00E+00	-7.39E-01	7.42E-03	-1.85E+00	3.06E-12	-2.45E+00	5.70E-19	3.41E-02	1.00E+00
ENSBTAG00000021121	5.97E-02	1.00E+00	-6.07E-02	1.00E+00	-4.52E-02	1.00E+00	6.95E-02	1.00E+00	3.17E-01	6.01E-01
ENSBTAG00000022019	1.86E-03	1.00E+00	5.99E+00	7.32E-22	4.26E+00	1.17E-11	2.50E+00	4.11E-04	-3.60E-01	1.00E+00
ENSBTAG00000029891	0.00E+00	1.00E+00	1.99E+00	4.43E-01	1.78E+00	4.79E-01	0.00E+00	1.00E+00	0.00E+00	1.00E+00
ENSBTAG00000036443	0.00E+00	1.00E+00	0.00E+00	1.00E+00	-5.13E-15	1.00E+00	0.00E+00	1.00E+00	0.00E+00	1.00E+00
ENSBTAG00000000067	1.45E+00	1.02E-02	3.85E+00	1.35E-16	3.48E+00	2.92E-14	2.72E+00	1.93E-09	1.15E+00	1.27E-01
ENSBTAG00000006173	-7.81E-01	1.00E+00	-7.17E-01	9.45E-01	-9.18E-01	8.09E-01	-1.70E-01	1.00E+00	-2.70E+00	6.01E-01
ENSBTAG00000013036	-5.77E-02	1.00E+00	-1.36E+00	2.09E-03	-1.84E+00	2.33E-05	-1.12E+00	1.20E-02	-2.10E-01	1.00E+00
ENSBTAG00000013038	1.27E-01	1.00E+00	7.46E-01	1.18E-04	6.71E-01	6.06E-04	4.45E-01	3.85E-02	1.03E-01	1.00E+00
ENSBTAG00000047538	-1.05E+00	6.15E-01	9.38E-01	1.65E-01	-2.75E-01	9.76E-01	4.65E-01	6.56E-01	5.51E-01	8.85E-01
ENSBTAG00000005951	-1.78E+00	4.11E-02	-7.95E-01	2.66E-01	4.70E-01	5.07E-01	4.76E-01	5.10E-01	6.28E-02	1.00E+00
ENSBTAG00000030927	-7.23E-01	9.60E-01	-1.56E+00	1.78E-01	-9.00E-01	4.46E-01	-2.33E+00	3.95E-02	1.32E-01	1.00E+00

ENSBTAG00000013971	-3.06E-02	1.00E+00	-4.19E-01	6.42E-02	-5.02E-01	2.08E-02	-5.27E-01	1.64E-02	1.01E-01	1.00E+00
ENSBTAG00000013973	0.00E+00	1.00E+00	0.00E+00	1.00E+00	-5.13E-15	1.00E+00	0.00E+00	1.00E+00	0.00E+00	1.00E+00
ENSBTAG00000020760	-5.00E-02	1.00E+00	8.37E-01	5.87E-05	1.01E+00	6.71E-07	9.36E-01	6.74E-06	-1.86E-01	9.14E-01
ENSBTAG00000045548	8.83E-02	1.00E+00	1.10E+00	1.61E-06	9.05E-01	9.87E-05	7.05E-01	3.95E-03	5.14E-01	2.61E-01
ENSBTAG00000038132	-2.12E-01	1.00E+00	-1.15E+00	2.77E-02	-4.99E-01	4.16E-01	-8.67E-01	9.88E-02	6.90E-01	4.91E-01
ENSBTAG00000011292	5.47E-03	1.00E+00	-2.85E-01	2.31E-01	2.86E-01	2.23E-01	5.99E-02	1.00E+00	7.37E-03	1.00E+00
ENSBTAG00000044198	-8.66E-02	1.00E+00	-6.37E-02	1.00E+00	4.11E-01	2.14E-01	3.79E-01	2.77E-01	-3.28E-01	8.00E-01
ENSBTAG00000030979	1.05E-01	1.00E+00	9.18E-01	2.34E-03	2.36E-01	6.53E-01	4.75E-01	1.93E-01	5.30E-01	4.55E-01
ENSBTAG00000019244	-1.96E+00	5.76E-01	-5.80E-01	8.88E-01	-8.23E-01	6.76E-01	-1.35E+00	4.17E-01	4.52E-01	1.00E+00
ENSBTAG00000009145	6.63E-01	8.23E-01	-7.92E-01	4.43E-01	-5.94E-01	5.95E-01	-5.23E-01	6.83E-01	5.46E-01	9.20E-01
ENSBTAG00000038258	1.30E+00	2.32E-01	2.83E+00	3.48E-08	1.52E+00	1.58E-02	1.15E+00	1.14E-01	1.50E+00	1.56E-01
ENSBTAG00000014646	-7.67E-03	1.00E+00	-7.15E-02	1.00E+00	3.48E-01	2.62E-01	9.71E-02	1.00E+00	-3.54E-01	6.46E-01
ENSBTAG00000002130	-3.65E-02	1.00E+00	5.00E-01	1.54E-02	4.02E-01	6.42E-02	7.58E-02	1.00E+00	-6.32E-02	1.00E+00
ENSBTAG00000031018	8.77E-01	8.23E-01	1.64E+00	3.01E-02	1.42E+00	6.89E-02	-3.97E-01	9.62E-01	1.30E-02	1.00E+00
ENSBTAG00000018048	-3.08E-02	1.00E+00	8.15E-01	1.29E-04	5.55E-01	1.34E-02	5.36E-02	1.00E+00	4.69E-02	1.00E+00
ENSBTAG00000047676	0.00E+00	1.00E+00	0.00E+00	1.00E+00	-5.13E-15	1.00E+00	0.00E+00	1.00E+00	0.00E+00	1.00E+00
ENSBTAG00000046165	0.00E+00	1.00E+00	0.00E+00	1.00E+00	-5.13E-15	1.00E+00	0.00E+00	1.00E+00	0.00E+00	1.00E+00
ENSBTAG00000020161	-5.96E-01	4.79E-02	-1.97E+00	2.27E-20	-1.77E+00	4.18E-17	-1.63E+00	9.68E-15	5.66E-02	1.00E+00
ENSBTAG00000019375	3.21E-01	8.48E-01	-2.16E-01	7.55E-01	-1.65E-01	8.88E-01	-8.87E-01	9.78E-03	-4.00E-01	7.62E-01
ENSBTAG00000016257	1.67E-02	1.00E+00	2.64E-01	3.92E-01	6.13E-01	8.38E-03	8.33E-02	1.00E+00	3.51E-01	5.79E-01
ENSBTAG00000000429	-1.62E-01	9.59E-01	-1.86E-01	5.00E-01	-3.96E-01	5.72E-02	-5.04E-01	1.25E-02	3.24E-01	4.84E-01
ENSBTAG00000045955	3.78E-03	1.00E+00	2.30E+00	1.51E-06	1.67E+00	9.24E-04	8.50E-01	1.90E-01	7.50E-02	1.00E+00
ENSBTAG00000048123	-6.38E-01	4.30E-01	7.86E-01	2.53E-02	1.22E+00	9.76E-05	1.02E+00	1.88E-03	-6.77E-02	1.00E+00
ENSBTAG00000018534	-3.82E-01	5.75E-01	7.56E-02	1.00E+00	1.25E+00	8.71E-08	1.14E+00	1.56E-06	-1.81E-01	1.00E+00
ENSBTAG00000047526	1.95E-01	1.00E+00	-4.51E+00	6.07E-03	-1.09E+00	4.02E-01	-4.51E+00	3.29E-03	2.28E-02	1.00E+00
ENSBTAG00000008528	-5.53E-01	6.31E-01	-1.92E+00	1.12E-06	-2.59E+00	6.47E-11	-1.68E+00	2.80E-05	-2.23E-01	1.00E+00
ENSBTAG00000019888	1.60E-01	1.00E+00	1.12E+00	3.85E-06	1.45E+00	1.09E-09	1.02E+00	3.10E-05	-6.58E-02	1.00E+00
ENSBTAG00000047765	0.00E+00	1.00E+00	0.00E+00	1.00E+00	-5.13E-15	1.00E+00	0.00E+00	1.00E+00	0.00E+00	1.00E+00
ENSBTAG00000019891	1.94E-02	1.00E+00	-7.49E-02	1.00E+00	-3.10E-03	1.00E+00	2.43E-01	5.63E-01	-3.00E-01	8.00E-01
ENSBTAG00000019894	4.31E-01	2.32E-01	6.78E-01	1.18E-03	-2.25E-02	1.00E+00	-5.00E-01	2.89E-02	1.24E-01	1.00E+00
ENSBTAG00000004284	5.00E-03	1.00E+00	2.61E-01	3.53E-01	-2.51E-03	1.00E+00	-2.20E-01	4.80E-01	-2.05E-01	8.47E-01
ENSBTAG00000004286	-1.78E-01	1.00E+00	3.29E-01	3.47E-01	-8.80E-01	1.22E-03	-1.08E+00	7.29E-05	4.48E-01	4.90E-01
ENSBTAG00000022020	-4.31E-01	8.54E-01	-3.53E+00	3.24E-14	-1.30E+00	3.45E-03	-1.01E+00	3.23E-02	-7.74E-01	4.34E-01
ENSBTAG00000008320	7.29E-01	4.71E-01	6.47E-01	2.39E-01	1.41E+00	9.58E-04	1.63E+00	1.08E-04	5.10E-01	8.00E-01
ENSBTAG00000039059	-2.71E+00	5.76E-01	9.65E-01	6.61E-01	7.21E-01	8.27E-01	-9.47E-01	8.05E-01	-7.99E-01	1.00E+00

ENSBTAG00000021052	3.24E-02	1.00E+00	-3.53E-01	2.63E-01	-2.16E-02	1.00E+00	4.85E-02	1.00E+00	9.17E-02	1.00E+00
ENSBTAG00000043581	-1.82E-01	1.00E+00	-6.20E-01	2.62E-02	-8.79E-01	8.42E-04	-1.11E+00	2.35E-05	-1.80E-01	1.00E+00
ENSBTAG00000047896	-3.17E-01	9.50E-01	-4.37E-01	3.81E-01	-1.35E+00	2.67E-04	-1.24E+00	1.05E-03	5.71E-01	5.80E-01
ENSBTAG00000019516	-3.76E-01	7.03E-01	-5.37E-01	9.74E-02	-1.46E+00	1.92E-07	-1.35E+00	2.10E-06	6.42E-01	2.35E-01
ENSBTAG00000001473	-9.44E-01	1.32E-02	-2.57E+00	6.19E-18	-3.24E+00	1.64E-26	-2.42E+00	3.98E-16	-2.09E-01	1.00E+00
ENSBTAG00000029769	0.00E+00	1.00E+00	0.00E+00	1.00E+00	2.55E+00	2.54E-01	3.41E+00	8.27E-02	2.72E+00	6.01E-01
ENSBTAG00000045910	-6.89E-01	6.67E-02	-2.12E+00	7.10E-16	-2.18E+00	5.34E-17	-1.76E+00	1.14E-11	-3.06E-01	8.00E-01
ENSBTAG00000012421	-4.72E-01	5.40E-01	-2.27E+00	1.59E-13	-2.60E+00	2.67E-17	-1.79E+00	2.82E-09	-6.03E-01	3.44E-01
ENSBTAG00000047795	-2.12E-01	1.00E+00	6.29E-01	4.41E-01	1.66E+00	1.35E-03	1.68E+00	1.32E-03	2.45E-01	1.00E+00
ENSBTAG00000019869	-3.33E-01	5.21E-01	7.12E-02	1.00E+00	1.07E+00	9.20E-08	8.60E-01	3.13E-05	1.50E-01	1.00E+00
ENSBTAG00000039349	0.00E+00	1.00E+00	0.00E+00	1.00E+00	-5.13E-15	1.00E+00	0.00E+00	1.00E+00	0.00E+00	1.00E+00
ENSBTAG00000019871	-1.89E-01	9.62E-01	8.55E-01	8.91E-05	1.34E+00	1.97E-10	7.66E-01	6.14E-04	-1.29E-01	1.00E+00
ENSBTAG00000046005	3.97E-01	8.23E-01	1.43E+00	4.51E-05	1.45E+00	3.26E-05	1.84E+00	1.37E-07	-3.68E-01	8.06E-01
ENSBTAG00000006656	6.46E-03	1.00E+00	9.46E-01	1.00E-03	1.50E+00	4.36E-08	1.45E+00	1.86E-07	-3.45E-01	7.92E-01
ENSBTAG00000042154	0.00E+00	1.00E+00	0.00E+00	1.00E+00	-5.13E-15	1.00E+00	0.00E+00	1.00E+00	0.00E+00	1.00E+00
ENSBTAG00000006657	-1.25E+00	4.78E-02	-2.70E+00	3.78E-09	-4.09E+00	1.10E-17	-2.46E+00	7.65E-08	1.24E-01	1.00E+00
ENSBTAG00000047626	1.66E-01	1.00E+00	9.72E-02	1.00E+00	5.08E-01	7.51E-01	3.57E-01	9.43E-01	8.78E-01	7.90E-01
ENSBTAG00000046677	-1.14E+00	5.78E-02	-3.02E+00	2.26E-11	-2.65E+00	1.15E-09	-1.57E+00	3.24E-04	-5.80E-01	6.58E-01
ENSBTAG00000046322	-5.70E-02	1.00E+00	-1.03E+00	2.47E-01	-7.27E-01	4.30E-01	-5.52E-01	6.08E-01	5.96E-01	8.16E-01
ENSBTAG00000022183	0.00E+00	1.00E+00	0.00E+00	1.00E+00	-5.13E-15	1.00E+00	0.00E+00	1.00E+00	1.91E+00	8.00E-01
ENSBTAG00000046786	-9.43E-02	1.00E+00	-4.50E-02	1.00E+00	1.33E-01	8.88E-01	2.01E-01	6.61E-01	-3.21E-01	7.58E-01
ENSBTAG00000018244	-1.91E+00	8.23E-01	-1.91E+00	4.69E-01	-1.91E+00	4.28E-01	-1.91E+00	4.39E-01	1.31E+00	8.99E-01
ENSBTAG00000023795	-7.58E-02	1.00E+00	-8.02E-03	1.00E+00	3.48E-02	1.00E+00	1.44E-01	7.31E-01	1.80E-02	1.00E+00
ENSBTAG00000009358	8.18E-02	1.00E+00	-5.03E-01	6.03E-02	-2.82E-01	3.93E-01	-6.32E-02	1.00E+00	5.69E-02	1.00E+00
ENSBTAG00000009357	8.44E-01	4.08E-02	1.19E+00	7.55E-05	-4.01E-01	3.02E-01	-1.72E+00	1.81E-08	-2.02E-01	1.00E+00
ENSBTAG00000044665	0.00E+00	1.00E+00	0.00E+00	1.00E+00	-5.13E-15	1.00E+00	0.00E+00	1.00E+00	0.00E+00	1.00E+00
ENSBTAG00000047399	0.00E+00	1.00E+00	0.00E+00	1.00E+00	-5.13E-15	1.00E+00	0.00E+00	1.00E+00	0.00E+00	1.00E+00
ENSBTAG00000042893	0.00E+00	1.00E+00	0.00E+00	1.00E+00	-5.13E-15	1.00E+00	0.00E+00	1.00E+00	0.00E+00	1.00E+00
ENSBTAG00000010627	1.74E-01	9.30E-01	1.18E+00	1.30E-10	1.22E+00	2.29E-11	1.05E+00	1.33E-08	1.00E-01	1.00E+00
ENSBTAG00000044280	0.00E+00	1.00E+00	0.00E+00	1.00E+00	-5.13E-15	1.00E+00	0.00E+00	1.00E+00	0.00E+00	1.00E+00
ENSBTAG00000043049	0.00E+00	1.00E+00	0.00E+00	1.00E+00	-5.13E-15	1.00E+00	0.00E+00	1.00E+00	0.00E+00	1.00E+00
ENSBTAG00000042806	0.00E+00	1.00E+00	0.00E+00	1.00E+00	-5.13E-15	1.00E+00	0.00E+00	1.00E+00	0.00E+00	1.00E+00
ENSBTAG00000012988	9.21E-02	1.00E+00	2.35E-02	1.00E+00	4.15E-01	1.29E-01	3.43E-01	2.53E-01	1.78E-01	9.98E-01
ENSBTAG00000012985	-2.53E-01	1.00E+00	-8.71E-01	8.67E-03	-7.46E-01	2.83E-02	-7.54E-01	2.99E-02	3.42E-02	1.00E+00
ENSBTAG00000046156	-3.93E-01	6.14E-01	-2.70E+00	1.17E-19	-1.81E+00	7.44E-11	-1.54E+00	3.26E-08	3.35E-01	7.71E-01

ENSBTAG00000003570	6.15E-02	1.00E+00	7.24E-01	6.18E-05	6.22E-01	6.84E-04	3.78E-01	6.76E-02	-8.11E-02	1.00E+00
ENSBTAG00000020714	6.61E-02	1.00E+00	5.77E-01	1.71E-02	7.64E-01	8.87E-04	6.73E-01	4.80E-03	4.86E-02	1.00E+00
ENSBTAG00000019810	-3.51E-02	1.00E+00	2.12E+00	1.13E-15	1.80E+00	6.80E-12	1.19E+00	9.39E-06	-1.27E-01	1.00E+00
ENSBTAG00000004766	-1.29E+00	9.38E-01	-3.21E+00	1.26E-01	-1.52E-01	1.00E+00	-1.44E+00	4.97E-01	-5.01E-01	1.00E+00
ENSBTAG00000047937	4.15E+00	8.24E-02	3.30E+00	1.02E-01	1.78E+00	4.79E-01	2.53E+00	2.71E-01	2.71E+00	6.01E-01
ENSBTAG00000016471	9.25E-02	1.00E+00	-1.33E-01	9.95E-01	8.82E-03	1.00E+00	-5.95E-02	1.00E+00	8.46E-01	1.19E-01
ENSBTAG00000002303	-1.16E-02	1.00E+00	-2.80E-01	5.25E-01	2.87E-01	4.97E-01	8.65E-01	4.84E-03	7.68E-01	1.15E-01
ENSBTAG00000004526	5.18E-01	4.59E-01	1.78E+00	1.98E-09	6.61E-01	4.96E-02	4.02E-01	3.26E-01	-2.33E-01	1.00E+00
ENSBTAG00000004460	6.19E-01	6.72E-01	2.21E+00	5.70E-07	9.55E-01	5.38E-02	1.37E+00	3.43E-03	5.93E-01	7.26E-01
ENSBTAG00000012216	9.57E-01	3.75E-01	1.40E+00	8.89E-03	1.56E+00	2.53E-03	8.43E-01	1.87E-01	1.50E-01	1.00E+00
ENSBTAG00000012222	-4.02E-01	1.00E+00	-1.53E-01	1.00E+00	4.62E-01	8.52E-01	1.08E+00	3.11E-01	-5.06E-01	1.00E+00
ENSBTAG00000016878	-2.02E-01	1.00E+00	1.11E+00	8.92E-05	9.03E-02	1.00E+00	-5.33E-03	1.00E+00	-7.95E-02	1.00E+00
ENSBTAG00000034689	2.88E-01	8.11E-01	9.61E-01	5.82E-05	2.52E-01	4.76E-01	2.73E-01	4.39E-01	-1.63E-01	1.00E+00
ENSBTAG00000006779	-5.02E-01	5.72E-01	-8.97E-01	8.87E-03	-1.95E+00	2.03E-09	-2.46E+00	9.07E-14	-4.45E-01	6.79E-01
ENSBTAG00000000868	-4.12E-01	9.41E-01	-1.46E+00	6.80E-03	-3.51E-01	6.66E-01	-4.11E-01	5.73E-01	-2.95E-01	1.00E+00
ENSBTAG00000042634	0.00E+00	1.00E+00	0.00E+00	1.00E+00	-5.13E-15	1.00E+00	0.00E+00	1.00E+00	0.00E+00	1.00E+00
ENSBTAG00000008500	1.33E-02	1.00E+00	1.39E+00	5.17E-01	-1.91E+00	4.28E-01	6.21E-01	9.94E-01	-1.91E+00	8.00E-01
ENSBTAG00000011995	-5.03E-01	1.00E+00	9.87E-02	1.00E+00	-3.21E+00	9.50E-02	-1.44E+00	4.97E-01	-3.21E+00	4.34E-01
ENSBTAG00000039914	5.69E-01	1.00E+00	-1.45E-01	1.00E+00	-2.35E+00	1.00E-01	-4.12E+00	1.24E-02	-1.42E+00	7.68E-01
ENSBTAG00000012002	9.66E-01	8.21E-05	2.24E+00	2.78E-25	1.34E+00	4.49E-10	6.53E-01	4.94E-03	1.35E-01	1.00E+00
ENSBTAG00000015427	-9.63E-02	1.00E+00	-6.21E-01	1.15E-02	-6.64E-01	5.99E-03	-5.40E-01	3.53E-02	-1.65E-01	1.00E+00
ENSBTAG00000009805	1.13E-01	1.00E+00	7.08E-01	1.40E-03	1.12E+00	1.17E-07	1.18E+00	2.75E-08	1.94E-01	9.05E-01
ENSBTAG00000030600	-5.70E-02	1.00E+00	-9.73E-02	9.45E-01	-1.16E-01	8.56E-01	-7.70E-02	1.00E+00	3.07E-01	6.07E-01
ENSBTAG00000031301	0.00E+00	1.00E+00	0.00E+00	1.00E+00	-5.13E-15	1.00E+00	0.00E+00	1.00E+00	0.00E+00	1.00E+00
ENSBTAG00000015428	-1.05E-01	1.00E+00	-7.68E-01	1.50E-04	-9.84E-01	6.50E-07	-7.08E-01	6.08E-04	-3.54E-02	1.00E+00
ENSBTAG00000040000	0.00E+00	1.00E+00	0.00E+00	1.00E+00	-5.13E-15	1.00E+00	1.77E+00	4.97E-01	1.91E+00	8.00E-01
ENSBTAG00000015432	-2.70E-01	1.00E+00	-1.94E+00	3.59E-05	-6.30E-01	2.06E-01	2.03E-01	8.97E-01	2.74E-01	1.00E+00
ENSBTAG00000006550	-1.50E-01	1.00E+00	-6.84E-01	3.76E-04	-9.87E-01	1.14E-07	-7.88E-01	3.82E-05	-2.94E-01	5.97E-01
ENSBTAG00000015669	2.87E-01	9.62E-01	1.86E+00	4.68E-09	1.97E+00	3.95E-10	1.00E+00	3.43E-03	4.52E-01	6.86E-01
ENSBTAG00000034185	0.00E+00	1.00E+00	0.00E+00	1.00E+00	-5.13E-15	1.00E+00	0.00E+00	1.00E+00	0.00E+00	1.00E+00
ENSBTAG00000015677	-1.34E-01	1.00E+00	-2.08E-02	1.00E+00	6.33E-02	1.00E+00	1.53E-01	8.14E-01	-4.04E-01	5.47E-01
ENSBTAG00000015686	2.76E-01	7.13E-01	1.23E+00	1.20E-09	1.30E+00	7.33E-11	7.92E-01	1.75E-04	-1.60E-01	9.99E-01
ENSBTAG00000026004	2.76E-01	1.00E+00	6.83E-01	4.38E-01	-6.72E-01	5.12E-01	6.87E-01	4.22E-01	-1.57E-01	1.00E+00
ENSBTAG00000044475	0.00E+00	1.00E+00	0.00E+00	1.00E+00	-5.13E-15	1.00E+00	0.00E+00	1.00E+00	0.00E+00	1.00E+00
ENSBTAG00000012131	0.00E+00	1.00E+00	0.00E+00	1.00E+00	1.78E+00	4.79E-01	0.00E+00	1.00E+00	0.00E+00	1.00E+00

ENSBTAG00000015694	-3.02E-01	8.50E-01	2.00E+00	1.13E-12	2.21E+00	2.35E-15	2.04E+00	3.41E-13	4.07E-01	6.55E-01
ENSBTAG00000023780	-5.19E-01	4.74E-01	5.17E-01	1.35E-01	1.34E-01	9.59E-01	-5.24E-01	1.58E-01	-2.15E-02	1.00E+00
ENSBTAG00000001010	-8.46E-01	8.23E-01	-2.07E-01	1.00E+00	-1.04E+00	3.14E-01	7.04E-01	4.81E-01	-1.82E+00	3.47E-01
ENSBTAG00000007184	-9.19E-02	1.00E+00	-1.06E+00	1.24E-06	-2.91E-01	2.99E-01	-5.52E-01	1.98E-02	3.19E-01	6.07E-01
ENSBTAG00000018146	4.69E-01	8.23E-01	1.77E-01	1.00E+00	-4.25E-01	5.43E-01	-1.74E-01	1.00E+00	-6.78E-02	1.00E+00
ENSBTAG00000044653	0.00E+00	1.00E+00	0.00E+00	1.00E+00	-5.13E-15	1.00E+00	0.00E+00	1.00E+00	0.00E+00	1.00E+00
ENSBTAG00000046772	1.92E+00	8.23E-01	0.00E+00	1.00E+00	1.78E+00	4.79E-01	1.76E+00	4.97E-01	0.00E+00	1.00E+00
ENSBTAG00000015894	-1.10E-01	1.00E+00	-1.48E+00	2.94E-12	-1.34E+00	1.54E-10	-1.20E+00	1.57E-08	5.04E-01	1.95E-01
ENSBTAG00000044192	-4.15E-01	4.35E-01	-4.95E-01	6.72E-02	-7.12E-01	4.44E-03	-3.91E-01	1.84E-01	5.37E-01	2.38E-01
ENSBTAG00000017752	1.90E-01	1.00E+00	-1.16E+00	3.49E-03	-1.44E+00	1.82E-04	-2.28E+00	1.80E-08	1.68E-02	1.00E+00
ENSBTAG00000017112	3.45E-01	9.65E-01	-3.15E-01	6.82E-01	-4.73E-01	4.13E-01	3.75E-01	5.52E-01	5.26E-01	7.28E-01
ENSBTAG00000017009	-6.64E-01	1.19E-01	-7.74E-01	7.09E-03	-4.52E-01	1.65E-01	-5.29E-01	9.52E-02	-1.12E-01	1.00E+00
ENSBTAG00000011635	-1.65E-01	1.00E+00	8.89E-01	5.87E-02	5.66E-01	2.98E-01	-1.47E-01	1.00E+00	3.40E-01	9.77E-01
ENSBTAG00000008213	-6.28E-01	5.86E-02	-7.36E-01	2.17E-03	6.61E-02	1.00E+00	-4.03E-01	1.48E-01	1.75E-02	1.00E+00
ENSBTAG00000023765	-6.05E-01	6.37E-01	-2.88E+00	4.19E-07	-1.03E+00	3.05E-02	3.87E-01	5.01E-01	9.19E-01	1.78E-01
ENSBTAG00000044410	0.00E+00	1.00E+00	0.00E+00	1.00E+00	-5.13E-15	1.00E+00	0.00E+00	1.00E+00	0.00E+00	1.00E+00
ENSBTAG00000006795	-6.79E-01	3.18E-01	-1.35E+00	1.44E-04	-2.18E+00	5.91E-10	-2.61E+00	2.79E-13	-6.84E-01	3.80E-01
ENSBTAG00000012693	-8.65E-02	1.00E+00	-2.78E+00	2.45E-13	-2.66E+00	1.05E-12	-2.96E+00	8.31E-15	-8.85E-01	1.78E-01
ENSBTAG00000001026	9.73E-01	8.40E-01	1.20E+00	3.17E-01	7.79E-01	6.23E-01	2.16E+00	9.88E-03	9.46E-01	8.33E-01
ENSBTAG00000009428	6.09E-01	4.55E-01	1.12E+00	1.77E-03	1.44E+00	2.42E-05	1.77E+00	1.08E-07	2.14E-01	1.00E+00
ENSBTAG00000048093	1.92E+00	8.23E-01	0.00E+00	1.00E+00	1.78E+00	4.79E-01	1.77E+00	4.97E-01	0.00E+00	1.00E+00
ENSBTAG00000013524	-1.39E-01	1.00E+00	-1.08E+00	1.13E-07	-1.28E+00	2.30E-10	-5.92E-01	6.70E-03	2.41E-01	8.00E-01
ENSBTAG00000002103	7.66E-02	1.00E+00	-1.08E+00	6.98E-04	-1.27E+00	5.13E-05	-3.44E-01	4.39E-01	5.69E-01	4.30E-01
ENSBTAG00000028336	0.00E+00	1.00E+00	0.00E+00	1.00E+00	-5.13E-15	1.00E+00	0.00E+00	1.00E+00	0.00E+00	1.00E+00
ENSBTAG00000048177	0.00E+00	1.00E+00	0.00E+00	1.00E+00	-5.13E-15	1.00E+00	0.00E+00	1.00E+00	0.00E+00	1.00E+00
ENSBTAG00000045367	0.00E+00	1.00E+00	0.00E+00	1.00E+00	-5.13E-15	1.00E+00	0.00E+00	1.00E+00	1.91E+00	8.00E-01
ENSBTAG00000018394	-3.76E-01	6.21E-01	-2.09E+00	5.39E-15	-1.53E+00	4.33E-09	-1.16E+00	1.41E-05	2.02E-01	9.90E-01
ENSBTAG00000003234	-4.20E-01	3.88E-01	-1.03E+00	1.12E-05	-1.94E+00	4.71E-17	-2.53E+00	8.52E-27	-4.29E-01	4.34E-01
ENSBTAG00000044237	0.00E+00	1.00E+00	0.00E+00	1.00E+00	-5.13E-15	1.00E+00	0.00E+00	1.00E+00	0.00E+00	1.00E+00
ENSBTAG00000009746	-3.47E-01	8.23E-01	7.38E-01	1.93E-02	6.28E-01	5.33E-02	9.04E-01	3.22E-03	-5.35E-01	4.73E-01
ENSBTAG00000046525	0.00E+00	1.00E+00	0.00E+00	1.00E+00	-5.13E-15	1.00E+00	0.00E+00	1.00E+00	0.00E+00	1.00E+00
ENSBTAG00000034373	-5.97E-01	9.32E-01	-1.26E+00	1.01E-01	-2.24E+00	1.42E-03	-2.12E+00	3.08E-03	7.18E-01	8.00E-01
ENSBTAG00000024889	-1.05E-02	1.00E+00	-6.73E-02	1.00E+00	-2.10E-01	4.43E-01	5.66E-02	1.00E+00	3.91E-02	1.00E+00
ENSBTAG00000004248	-3.99E-01	4.02E-01	-1.57E+00	1.55E-12	-8.85E-01	8.72E-05	-6.42E-01	7.33E-03	8.12E-02	1.00E+00
ENSBTAG00000012508	-2.28E-01	9.32E-01	-1.79E-01	7.25E-01	-3.67E-01	2.40E-01	-5.07E-02	1.00E+00	-2.91E-02	1.00E+00

ENSBTAG00000020236	-6.11E-01	4.86E-01	2.57E-01	7.42E-01	-1.55E+00	3.29E-05	-2.84E+00	6.16E-14	-7.45E-01	3.47E-01
ENSBTAG00000040086	1.86E-01	1.00E+00	6.26E-01	3.67E-01	1.05E+00	4.48E-02	3.77E-02	1.00E+00	1.45E+00	3.37E-02
ENSBTAG00000010170	-8.65E-02	1.00E+00	-6.33E-01	2.43E-03	-1.52E-01	6.86E-01	9.59E-02	9.40E-01	3.96E-01	3.71E-01
ENSBTAG00000037239	0.00E+00	1.00E+00	0.00E+00	1.00E+00	-5.13E-15	1.00E+00	0.00E+00	1.00E+00	0.00E+00	1.00E+00
ENSBTAG00000018770	-2.67E-01	8.23E-01	-2.58E+00	3.03E-23	-1.49E+00	6.76E-10	4.18E-02	1.00E+00	-1.29E-01	1.00E+00
ENSBTAG00000018771	4.02E-01	1.00E+00	3.86E+00	1.09E-06	1.32E+00	2.20E-01	9.38E-01	4.82E-01	-1.35E-01	1.00E+00
ENSBTAG00000018772	-7.35E-02	1.00E+00	6.01E-01	4.37E-03	2.13E-01	4.72E-01	-2.24E-01	4.54E-01	-1.83E-01	9.07E-01
ENSBTAG00000034283	-5.22E-01	1.00E+00	-1.24E+00	6.07E-01	-6.69E-01	8.89E-01	-6.92E-01	8.81E-01	-3.23E+00	4.34E-01
ENSBTAG00000016695	3.60E+00	2.29E-01	0.00E+00	1.00E+00	2.56E+00	2.54E-01	1.76E+00	4.97E-01	0.00E+00	1.00E+00
ENSBTAG00000000945	1.66E-01	1.00E+00	-9.84E-01	2.12E-04	-1.19E+00	4.38E-06	-1.33E+00	3.56E-07	2.59E-01	8.31E-01
ENSBTAG00000000946	-7.02E-03	1.00E+00	-1.13E+00	8.41E-05	-2.54E-01	5.40E-01	3.53E-01	3.26E-01	2.48E-01	8.97E-01
ENSBTAG00000016315	2.74E-02	1.00E+00	-1.62E-01	8.55E-01	-5.17E-02	1.00E+00	-2.38E-02	1.00E+00	-2.93E-01	8.22E-01
ENSBTAG00000008385	-7.99E-01	1.03E-02	-9.09E-01	2.68E-04	-4.06E-01	1.61E-01	-3.58E-02	1.00E+00	2.68E-01	8.00E-01
ENSBTAG00000009446	-9.64E-02	1.00E+00	4.08E-01	1.42E-01	4.83E-01	6.76E-02	3.87E-01	1.80E-01	-7.39E-02	1.00E+00
ENSBTAG00000021176	2.03E+00	2.99E-08	2.35E+00	5.73E-12	1.73E+00	4.20E-07	2.50E+00	2.99E-13	1.12E-01	1.00E+00
ENSBTAG00000004109	-7.92E-01	8.23E-01	-1.33E+00	1.73E-01	-1.15E+00	2.18E-01	-1.17E+00	2.18E-01	-2.15E-01	1.00E+00
ENSBTAG00000002639	1.86E-01	1.00E+00	9.74E-01	5.38E-05	1.62E+00	2.36E-12	1.72E+00	1.32E-13	4.71E-01	3.88E-01
ENSBTAG00000020835	5.98E-01	6.25E-01	-1.86E-01	9.84E-01	8.01E-01	8.94E-02	1.75E+00	1.82E-05	8.12E-01	3.84E-01
ENSBTAG00000020836	1.17E-02	1.00E+00	-2.71E+00	2.42E-01	7.22E-01	8.25E-01	-1.71E-01	1.00E+00	1.45E-03	1.00E+00
ENSBTAG00000009918	5.76E-01	3.56E-01	-9.04E-01	6.31E-03	-1.59E+00	5.19E-07	-8.04E-01	1.83E-02	6.90E-02	1.00E+00
ENSBTAG00000044006	3.15E-01	1.00E+00	2.12E+00	8.05E-09	2.53E+00	1.26E-12	2.04E+00	2.77E-08	-1.03E+00	2.63E-01
ENSBTAG00000016075	-1.53E-01	1.00E+00	8.98E-01	3.85E-04	1.17E+00	1.78E-06	6.91E-01	9.61E-03	-1.53E-01	1.00E+00
ENSBTAG00000016079	-1.00E-01	1.00E+00	-1.29E-01	8.44E-01	-2.69E-01	3.67E-01	-3.16E-01	2.65E-01	-9.04E-02	1.00E+00
ENSBTAG00000017824	1.51E-01	1.00E+00	-3.76E-01	1.23E-01	-6.21E-01	4.29E-03	-4.07E-01	9.35E-02	9.93E-02	1.00E+00
ENSBTAG00000000009	1.73E-01	1.00E+00	-1.03E+00	3.10E-02	4.12E-01	4.68E-01	2.77E-01	7.44E-01	-3.90E-02	1.00E+00
ENSBTAG00000012446	-2.04E-01	1.00E+00	-1.52E+00	1.24E-07	-2.71E-01	4.94E-01	-4.49E-01	1.82E-01	2.46E-01	8.99E-01
ENSBTAG00000012465	1.49E+00	5.76E-01	2.89E+00	8.67E-04	3.18E+00	8.67E-05	3.08E+00	2.16E-04	-3.21E+00	4.37E-01
ENSBTAG00000040605	-5.04E-01	1.00E+00	-1.23E+00	6.14E-01	-1.54E-01	1.00E+00	9.39E-01	5.63E-01	-3.21E+00	4.34E-01
ENSBTAG00000019087	-1.47E+00	5.76E-01	-1.39E+00	2.58E-01	-2.91E+00	1.80E-02	-2.16E+00	6.62E-02	-5.53E-01	1.00E+00
ENSBTAG00000002226	-4.32E-01	2.56E-01	-1.53E+00	2.03E-13	-1.10E+00	1.16E-07	-9.58E-01	6.22E-06	1.33E-01	1.00E+00
ENSBTAG00000011632	-1.18E-01	1.00E+00	-5.18E-01	3.41E-02	-3.17E-01	2.59E-01	-6.46E-01	6.65E-03	-2.32E-01	8.12E-01
ENSBTAG00000014267	-2.44E-01	1.00E+00	2.38E+00	1.19E-12	9.60E-01	6.17E-03	3.24E-01	5.28E-01	1.85E-01	1.00E+00
ENSBTAG00000006899	-7.87E-01	8.23E-01	-8.57E-02	1.00E+00	-3.54E-01	8.57E-01	-9.89E-01	2.67E-01	-7.99E-01	8.00E-01
ENSBTAG00000006905	2.50E-01	7.57E-01	1.35E+00	1.25E-12	1.34E+00	1.38E-12	5.54E-01	8.26E-03	-4.00E-02	1.00E+00
ENSBTAG00000006731	9.59E-01	8.12E-01	5.39E+00	6.98E-09	5.14E+00	2.16E-08	3.10E+00	4.58E-04	-2.53E-01	1.00E+00

ENSBTAG00000010151	-4.02E-01	6.88E-01	-1.47E+00	9.41E-07	-2.79E+00	9.35E-20	-3.75E+00	3.64E-31	8.27E-01	8.10E-02
ENSBTAG00000023745	4.58E-01	8.81E-01	1.96E+00	4.06E-05	5.58E-01	3.84E-01	-7.28E-01	2.15E-01	7.52E-03	1.00E+00
ENSBTAG00000045507	-1.50E+00	4.40E-02	-3.72E+00	2.17E-07	-7.50E-01	2.02E-01	-2.41E-01	8.94E-01	6.42E-02	1.00E+00
ENSBTAG00000015766	-1.36E+00	3.56E-03	-3.86E+00	3.31E-21	-3.70E+00	3.16E-20	-1.92E+00	7.10E-07	2.02E-01	1.00E+00
ENSBTAG00000018507	7.73E-01	1.00E+00	-2.80E-01	1.00E+00	-3.58E+00	4.66E-02	-3.58E+00	5.03E-02	8.00E-01	9.58E-01
ENSBTAG00000019520	-1.38E-01	1.00E+00	1.26E+00	7.36E-08	9.01E-01	1.64E-04	5.18E-01	5.38E-02	1.57E-01	1.00E+00
ENSBTAG00000047128	1.98E+00	5.76E-01	2.88E+00	2.18E-02	-1.91E+00	4.28E-01	-1.48E-01	1.00E+00	2.45E+00	3.59E-01
ENSBTAG00000003895	1.88E-02	1.00E+00	-1.77E-01	7.05E-01	-3.93E-01	1.71E-01	-3.35E-01	2.80E-01	-2.32E-01	8.59E-01
ENSBTAG00000012059	-4.45E-01	8.00E-01	-1.04E+00	8.33E-03	-1.54E+00	4.40E-05	-1.25E+00	1.32E-03	-6.67E-01	4.54E-01
ENSBTAG00000017528	1.26E-02	1.00E+00	6.05E-01	9.45E-01	1.03E+00	5.86E-01	-2.70E+00	2.05E-01	7.57E-03	1.00E+00
ENSBTAG00000020942	-1.84E-01	9.61E-01	8.92E-01	1.83E-05	9.97E-01	1.11E-06	2.17E-01	4.97E-01	-3.07E-01	6.40E-01
ENSBTAG00000020943	-3.34E-01	5.86E-01	5.81E-01	1.44E-02	9.34E-01	2.39E-05	8.38E-02	1.00E+00	-4.47E-01	3.68E-01
ENSBTAG00000020944	7.85E-01	2.66E-03	1.15E+00	6.67E-08	2.41E-01	4.30E-01	-1.88E-01	6.12E-01	-2.00E-01	9.00E-01
ENSBTAG00000044808	0.00E+00	1.00E+00	0.00E+00	1.00E+00	-5.13E-15	1.00E+00	0.00E+00	1.00E+00	0.00E+00	1.00E+00
ENSBTAG00000000638	-1.23E+00	7.64E-02	-1.45E+00	3.98E-03	-2.03E+00	4.05E-05	-1.15E+00	2.37E-02	-1.27E+00	1.08E-01
ENSBTAG00000000639	-1.62E-01	1.00E+00	-9.69E-02	1.00E+00	2.02E-01	6.27E-01	-9.20E-03	1.00E+00	-2.36E-01	8.64E-01
ENSBTAG00000004568	-3.54E-02	1.00E+00	-1.14E-01	9.45E-01	3.57E-01	2.32E-01	8.73E-02	1.00E+00	3.53E-01	6.21E-01
ENSBTAG00000019184	-1.61E-01	1.00E+00	-2.13E-01	5.12E-01	-1.68E-01	6.66E-01	-2.19E-01	4.97E-01	-3.12E-01	6.34E-01
ENSBTAG00000023752	-3.59E+00	2.32E-01	-2.84E-01	1.00E+00	-1.04E+00	5.97E-01	-1.06E+00	5.96E-01	3.28E-01	1.00E+00
ENSBTAG00000010927	-6.71E-01	2.29E-01	-1.51E+00	5.60E-06	-1.63E+00	5.14E-07	-1.96E+00	3.49E-09	2.12E-01	1.00E+00
ENSBTAG00000015968	-1.98E-02	1.00E+00	-5.33E-02	1.00E+00	-4.27E-01	8.75E-02	-8.32E-01	2.28E-04	4.25E-02	1.00E+00
ENSBTAG00000011530	3.58E-01	1.00E+00	1.68E+00	2.04E-03	7.42E-01	3.28E-01	8.68E-01	2.22E-01	1.05E-01	1.00E+00
ENSBTAG00000011533	-5.64E-01	2.30E-01	-1.50E+00	3.73E-08	-6.60E-01	2.11E-02	-8.37E-01	2.92E-03	-3.77E-01	6.47E-01
ENSBTAG00000016006	-2.83E-01	5.76E-01	-2.04E-01	4.50E-01	-4.90E-01	1.45E-02	-5.74E-01	3.90E-03	1.96E-01	8.09E-01
ENSBTAG00000012041	-4.55E-01	2.73E-01	-9.15E-01	5.38E-05	-1.55E+00	2.24E-12	-1.86E+00	4.78E-17	-7.11E-02	1.00E+00
ENSBTAG00000012044	-1.77E-02	1.00E+00	4.98E-01	1.55E-01	4.80E-01	1.75E-01	4.92E-01	1.72E-01	-1.86E-01	1.00E+00
ENSBTAG00000042786	0.00E+00	1.00E+00	0.00E+00	1.00E+00	-5.13E-15	1.00E+00	0.00E+00	1.00E+00	0.00E+00	1.00E+00
ENSBTAG00000012215	1.96E-02	1.00E+00	2.82E+00	2.15E-06	2.32E+00	2.26E-04	-1.23E-02	1.00E+00	9.94E-01	7.15E-01
ENSBTAG00000033326	8.03E-01	1.00E+00	2.07E+00	1.96E-01	-1.91E+00	4.28E-01	-1.91E+00	4.39E-01	6.02E-04	1.00E+00
ENSBTAG00000033331	-3.32E-01	5.76E-01	-1.07E+00	6.62E-07	-5.37E-01	1.97E-02	1.87E-03	1.00E+00	3.34E-02	1.00E+00
ENSBTAG00000033333	-3.72E-01	6.28E-01	-1.02E+00	1.33E-04	-1.49E+00	1.03E-08	-1.30E+00	7.80E-07	-5.28E-02	1.00E+00
ENSBTAG00000033334	-1.17E+00	7.91E-05	-1.38E+00	1.97E-07	-8.34E-01	1.70E-03	-4.70E-01	1.21E-01	-2.14E-01	9.45E-01
ENSBTAG00000021165	-5.59E-01	1.57E-01	-1.42E+00	7.88E-09	-7.48E-01	2.85E-03	-4.60E-01	1.03E-01	2.46E-02	1.00E+00
ENSBTAG00000001904	-1.25E-01	1.00E+00	8.01E-01	8.25E-05	6.71E-01	1.20E-03	2.08E-02	1.00E+00	-1.98E-01	8.60E-01
ENSBTAG00000001906	-2.88E-01	8.23E-01	3.66E-01	2.45E-01	-4.95E-01	9.05E-02	-1.81E-01	7.32E-01	1.23E-01	1.00E+00

ENSBTAG00000040356	-5.64E-01	8.29E-01	-1.04E+00	1.17E-01	-1.94E-01	1.00E+00	3.09E-01	8.02E-01	-2.76E-01	1.00E+00
ENSBTAG00000012102	-1.67E-01	1.00E+00	-2.05E-01	5.38E-01	4.00E-01	1.09E-01	5.42E-01	2.20E-02	1.64E-01	1.00E+00
ENSBTAG00000023731	0.00E+00	1.00E+00	5.59E+00	2.80E-05	1.78E+00	4.79E-01	3.69E+00	4.71E-02	0.00E+00	1.00E+00
ENSBTAG00000023730	3.05E-01	1.00E+00	1.32E+00	1.93E-02	3.82E-01	7.60E-01	4.45E-01	6.68E-01	-4.72E-01	1.00E+00
ENSBTAG00000039014	-2.64E-01	9.06E-01	-9.12E-01	1.69E-03	-5.74E-01	6.63E-02	-5.10E-01	1.23E-01	2.88E-01	8.13E-01
ENSBTAG00000038051	-9.66E-02	1.00E+00	-9.52E-01	3.04E-04	-4.77E-01	1.02E-01	3.61E-01	2.62E-01	3.54E-01	6.48E-01
ENSBTAG00000020318	-1.63E-01	1.00E+00	-1.22E+00	8.37E-06	-7.10E-01	1.10E-02	-3.30E-01	3.53E-01	-2.87E-03	1.00E+00
ENSBTAG00000007096	-3.26E-01	7.65E-01	-1.06E+00	5.22E-05	-2.34E+00	1.76E-19	-2.75E+00	1.82E-25	-2.63E-01	8.33E-01
ENSBTAG00000029640	8.05E-01	1.00E+00	6.91E-02	1.00E+00	-1.91E+00	4.28E-01	-1.91E+00	4.39E-01	3.85E-04	1.00E+00
ENSBTAG00000033441	2.71E-01	1.00E+00	6.34E-02	1.00E+00	-4.04E-01	7.06E-01	-5.70E-01	4.97E-01	-2.67E-01	1.00E+00
ENSBTAG00000025283	-1.98E-01	9.58E-01	-3.51E-01	2.13E-01	2.76E-01	3.70E-01	3.95E-01	1.48E-01	9.68E-02	1.00E+00
ENSBTAG00000002493	-1.18E-01	1.00E+00	-3.73E-01	9.05E-02	-3.39E-01	1.34E-01	-8.25E-02	9.82E-01	-2.50E-01	7.38E-01
ENSBTAG00000001872	-4.49E-01	3.04E-01	-3.83E-01	1.60E-01	-2.75E-01	3.70E-01	-1.59E-01	7.38E-01	-1.65E-01	1.00E+00
ENSBTAG00000023726	2.94E+00	8.57E-02	8.82E-01	8.52E-01	-1.91E+00	4.28E-01	6.21E-01	9.94E-01	2.24E+00	4.65E-01
ENSBTAG00000014818	9.63E-01	2.48E-01	5.41E-01	4.17E-01	-1.11E+00	4.31E-02	-1.42E+00	9.03E-03	1.66E+00	9.27E-03
ENSBTAG00000003503	-9.07E-02	1.00E+00	-6.27E-01	1.54E-02	-6.21E-01	1.57E-02	-1.16E+00	1.85E-06	-1.03E-01	1.00E+00
ENSBTAG00000042430	0.00E+00	1.00E+00	0.00E+00	1.00E+00	-5.13E-15	1.00E+00	1.76E+00	4.97E-01	0.00E+00	1.00E+00
ENSBTAG00000002980	-7.04E-01	3.75E-01	-1.01E+00	1.44E-02	-1.45E+00	2.36E-04	-1.01E+00	1.54E-02	2.02E-01	1.00E+00
ENSBTAG00000003757	2.49E-01	7.00E-01	1.03E+00	1.62E-08	6.28E-01	8.95E-04	2.53E-01	3.02E-01	-2.39E-01	7.57E-01
ENSBTAG00000004817	3.66E-01	9.08E-01	1.09E-01	1.00E+00	3.55E-01	5.46E-01	2.01E-02	1.00E+00	-6.78E-01	5.30E-01
ENSBTAG00000014370	2.08E-01	1.00E+00	-1.69E+00	3.00E-04	-2.67E+00	1.37E-08	-2.17E+00	3.22E-06	4.54E-01	8.06E-01
ENSBTAG00000004805	5.20E-02	1.00E+00	-2.39E-01	2.57E-01	-3.75E-01	4.12E-02	-4.04E-01	2.84E-02	1.89E-02	1.00E+00
ENSBTAG00000042102	0.00E+00	1.00E+00	0.00E+00	1.00E+00	-5.13E-15	1.00E+00	0.00E+00	1.00E+00	0.00E+00	1.00E+00
ENSBTAG00000004806	-7.83E-02	1.00E+00	-1.39E+00	2.40E-08	-6.88E-01	8.53E-03	-4.13E-01	1.74E-01	-8.46E-02	1.00E+00
ENSBTAG00000044191	-2.32E-01	1.00E+00	-8.19E-01	6.70E-01	-1.07E+00	4.79E-01	3.74E-01	9.70E-01	-2.21E+00	4.81E-01
ENSBTAG00000010858	-5.82E-01	5.75E-01	-1.86E+00	3.35E-06	-2.95E+00	1.06E-12	-2.91E+00	2.26E-12	-1.96E-01	1.00E+00
ENSBTAG00000010867	-4.16E-01	3.52E-01	-2.08E+00	1.62E-21	-3.11E+00	1.08E-43	-2.52E+00	2.11E-30	-3.37E-01	6.01E-01
ENSBTAG00000010868	-7.50E-01	7.26E-03	-1.48E+00	2.29E-11	-1.53E+00	3.11E-12	-1.78E+00	8.03E-16	-2.11E-02	1.00E+00
ENSBTAG00000019938	3.98E-01	3.44E-01	3.35E-01	1.92E-01	5.14E-01	2.17E-02	8.40E-01	6.27E-05	7.58E-02	1.00E+00
ENSBTAG00000015194	5.17E-01	1.00E+00	-2.70E+00	2.42E-01	-2.70E+00	1.99E-01	-2.70E+00	2.05E-01	-2.70E+00	6.01E-01
ENSBTAG00000008720	-7.94E-01	1.00E+00	-2.71E+00	2.42E-01	-9.24E-01	8.08E-01	9.78E-01	6.30E-01	-7.93E-01	1.00E+00
ENSBTAG00000017397	3.63E-01	8.23E-01	1.50E+00	5.63E-07	3.68E-01	3.94E-01	2.20E-01	7.43E-01	-2.28E-01	1.00E+00
ENSBTAG00000005535	-1.12E+00	8.23E-01	-1.55E+00	2.72E-01	-1.79E+00	1.70E-01	-2.57E+00	6.11E-02	3.49E-01	1.00E+00
ENSBTAG00000033078	2.58E-01	7.61E-01	5.00E-01	2.34E-02	6.17E-01	3.23E-03	3.33E-01	1.85E-01	-2.65E-01	7.60E-01
ENSBTAG00000016640	4.66E-02	1.00E+00	1.00E+00	1.09E-04	9.23E-01	3.77E-04	2.95E-01	4.31E-01	-1.36E-01	1.00E+00

ENSBTAG00000045104	0.00E+00	1.00E+00	0.00E+00	1.00E+00	-5.13E-15	1.00E+00	0.00E+00	1.00E+00	0.00E+00	1.00E+00
ENSBTAG00000042433	4.53E+00	2.86E-02	3.68E+00	5.08E-02	-5.13E-15	1.00E+00	1.76E+00	4.97E-01	3.59E+00	3.09E-01
ENSBTAG00000042439	6.87E-01	1.00E+00	-1.22E+00	6.14E-01	2.19E-01	1.00E+00	-6.80E-01	8.87E-01	5.13E-03	1.00E+00
ENSBTAG00000007942	-4.75E-02	1.00E+00	1.76E-01	7.76E-01	-1.07E+00	7.90E-05	-7.32E-01	1.14E-02	1.85E-01	1.00E+00
ENSBTAG00000006208	-3.31E-01	6.48E-01	-4.26E-01	1.23E-01	-4.38E-01	1.09E-01	-4.89E-01	7.09E-02	-1.78E-01	1.00E+00
ENSBTAG00000021575	-1.38E-02	1.00E+00	-6.08E-02	1.00E+00	1.69E-01	6.53E-01	5.02E-02	1.00E+00	-2.46E-01	8.00E-01
ENSBTAG00000012295	-9.78E-01	4.06E-02	-3.45E+00	2.39E-18	-2.95E+00	1.59E-15	-1.10E+00	2.34E-03	-7.94E-01	2.20E-01
ENSBTAG00000003961	-5.18E-01	6.24E-01	-1.99E+00	1.90E-07	-1.15E+00	2.44E-03	-5.56E-01	2.19E-01	3.26E-01	8.98E-01
ENSBTAG00000020936	9.78E-01	2.63E-02	4.12E-01	3.62E-01	-6.27E-01	1.13E-01	5.40E-01	1.82E-01	9.68E-01	5.66E-02
ENSBTAG00000006291	8.48E-01	4.03E-02	1.81E+00	8.75E-10	1.17E+00	9.24E-05	7.26E-01	2.72E-02	5.56E-01	4.31E-01
ENSBTAG00000028470	0.00E+00	1.00E+00	0.00E+00	1.00E+00	-5.13E-15	1.00E+00	0.00E+00	1.00E+00	0.00E+00	1.00E+00
ENSBTAG00000008544	2.34E+00	2.65E-17	4.37E+00	4.99E-52	4.03E+00	3.67E-46	2.14E+00	1.11E-15	7.05E-01	1.26E-01
ENSBTAG00000040550	0.00E+00	1.00E+00	3.30E+00	1.02E-01	1.78E+00	4.79E-01	0.00E+00	1.00E+00	1.93E+00	8.00E-01
ENSBTAG00000039104	-1.91E+00	8.23E-01	-1.91E+00	4.69E-01	-1.91E+00	4.28E-01	-1.91E+00	4.39E-01	-1.91E+00	8.00E-01
ENSBTAG00000000418	-2.82E-01	1.00E+00	-1.54E+00	6.99E-02	-1.20E+00	1.61E-01	-7.50E-01	4.58E-01	-6.10E-01	9.21E-01
ENSBTAG00000044597	0.00E+00	1.00E+00	0.00E+00	1.00E+00	-5.13E-15	1.00E+00	0.00E+00	1.00E+00	0.00E+00	1.00E+00
ENSBTAG00000002287	-8.29E-02	1.00E+00	-8.71E-01	2.55E-03	-1.03E+00	2.47E-04	-3.88E-02	1.00E+00	-3.61E-01	7.35E-01
ENSBTAG00000016243	-9.38E-02	1.00E+00	-3.44E-01	1.67E-01	-3.63E-01	1.36E-01	-6.49E-02	1.00E+00	2.20E-02	1.00E+00
ENSBTAG00000016250	1.31E-01	1.00E+00	1.46E+00	3.28E-06	1.44E+00	4.51E-06	1.38E+00	1.47E-05	2.01E-01	1.00E+00
ENSBTAG00000012499	2.95E-01	8.28E-01	-1.06E+00	7.32E-04	1.28E-01	9.54E-01	5.84E-03	1.00E+00	2.01E-01	1.00E+00
ENSBTAG00000012501	-3.49E-02	1.00E+00	-5.19E-01	2.12E-02	-6.43E-01	2.90E-03	-5.28E-01	2.04E-02	4.15E-02	1.00E+00
ENSBTAG00000042096	0.00E+00	1.00E+00	0.00E+00	1.00E+00	-5.13E-15	1.00E+00	0.00E+00	1.00E+00	0.00E+00	1.00E+00
ENSBTAG00000032615	0.00E+00	1.00E+00	0.00E+00	1.00E+00	-5.13E-15	1.00E+00	0.00E+00	1.00E+00	0.00E+00	1.00E+00
ENSBTAG00000012083	-4.13E-01	1.00E+00	-1.67E+00	1.70E-02	1.58E+00	4.73E-03	4.03E+00	5.57E-14	-2.16E-02	1.00E+00
ENSBTAG00000004838	-1.91E+00	8.23E-01	7.51E-02	1.00E+00	6.50E-01	9.78E-01	2.74E+00	3.28E-02	1.33E+00	8.94E-01
ENSBTAG00000000171	0.00E+00	1.00E+00	0.00E+00	1.00E+00	-5.13E-15	1.00E+00	0.00E+00	1.00E+00	0.00E+00	1.00E+00
ENSBTAG00000016407	5.11E-01	1.00E+00	-6.71E-01	7.50E-01	6.97E-01	5.71E-01	5.34E-01	7.64E-01	-7.52E-01	9.90E-01
ENSBTAG00000019267	-1.64E-01	1.00E+00	-9.41E-02	1.00E+00	-5.14E-01	3.73E-01	-5.53E-01	3.30E-01	-1.02E-01	1.00E+00
ENSBTAG00000019272	3.07E-01	9.88E-01	9.72E-01	1.14E-02	1.08E+00	3.77E-03	9.88E-01	1.06E-02	3.37E-01	9.00E-01
ENSBTAG00000043455	0.00E+00	1.00E+00	0.00E+00	1.00E+00	-5.13E-15	1.00E+00	0.00E+00	1.00E+00	0.00E+00	1.00E+00
ENSBTAG00000003353	-5.33E-01	1.00E+00	6.52E-01	6.83E-01	2.22E-01	1.00E+00	8.56E-01	4.69E-01	-9.23E-01	9.19E-01
ENSBTAG00000045183	0.00E+00	1.00E+00	0.00E+00	1.00E+00	-5.13E-15	1.00E+00	0.00E+00	1.00E+00	0.00E+00	1.00E+00
ENSBTAG00000032702	-7.71E-02	1.00E+00	3.17E-01	5.68E-01	3.34E-01	5.24E-01	2.62E-01	7.00E-01	-2.14E-01	1.00E+00
ENSBTAG00000011021	-9.07E-02	1.00E+00	-4.53E-01	2.66E-02	-1.20E+00	7.15E-11	-1.59E+00	4.10E-18	-6.21E-01	2.18E-02
ENSBTAG00000001851	-2.22E-01	9.85E-01	-5.80E-01	5.38E-02	-1.97E+00	1.48E-12	-2.26E+00	2.37E-15	-9.85E-01	9.18E-03

ENSBTAG00000038067	6.12E+00	5.11E-43	8.67E+00	4.42E-70	8.60E+00	1.45E-69	8.09E+00	2.71E-64	5.37E+00	4.83E-35
ENSBTAG00000038706	5.82E+00	2.24E-26	8.33E+00	3.29E-45	7.90E+00	2.36E-42	7.57E+00	7.61E-40	4.58E+00	2.63E-17
ENSBTAG00000001595	4.36E+00	4.92E-17	6.37E+00	2.96E-31	6.58E+00	7.14E-33	6.48E+00	4.48E-32	4.96E+00	2.36E-20
ENSBTAG00000023659	3.94E+00	1.53E-19	6.10E+00	2.54E-39	5.51E+00	2.83E-34	3.96E+00	1.08E-20	2.40E+00	1.63E-07
ENSBTAG00000016886	2.06E-02	1.00E+00	1.14E+00	9.81E-02	-4.06E-01	8.39E-01	-1.79E+00	6.26E-02	-8.53E-01	8.00E-01
ENSBTAG00000015084	-1.91E+00	8.23E-01	-1.91E+00	4.69E-01	6.50E-01	9.78E-01	-1.91E+00	4.39E-01	-1.91E+00	8.00E-01
ENSBTAG00000014923	-1.02E-01	1.00E+00	-1.93E+00	3.15E-20	-1.56E+00	1.81E-14	-1.16E+00	1.43E-08	4.31E-02	1.00E+00
ENSBTAG00000001171	-1.86E-01	1.00E+00	5.49E-02	1.00E+00	6.55E-01	1.30E-02	4.66E-01	1.12E-01	-6.75E-02	1.00E+00
ENSBTAG00000042592	0.00E+00	1.00E+00	0.00E+00	1.00E+00	-5.13E-15	1.00E+00	0.00E+00	1.00E+00	0.00E+00	1.00E+00
ENSBTAG00000010207	-2.78E-02	1.00E+00	9.19E-02	1.00E+00	5.36E-01	1.05E-01	7.33E-01	1.89E-02	-3.81E-02	1.00E+00
ENSBTAG00000003798	-3.18E-01	8.54E-01	-8.16E-01	1.42E-02	-1.03E+00	1.39E-03	-6.45E-01	7.17E-02	-9.92E-03	1.00E+00
ENSBTAG00000003794	-1.17E-01	1.00E+00	-3.71E-01	7.53E-01	-9.75E-03	1.00E+00	8.54E-01	1.98E-01	5.30E-01	8.44E-01
ENSBTAG00000011164	2.22E-02	1.00E+00	1.23E+00	3.82E-01	-1.42E+00	4.82E-01	4.84E-01	9.67E-01	6.99E-01	1.00E+00
ENSBTAG00000001852	-5.12E-02	1.00E+00	-8.43E-01	1.93E-01	-1.58E+00	4.98E-03	-1.83E+00	1.13E-03	-1.39E+00	1.30E-01
ENSBTAG00000006611	-1.16E-01	1.00E+00	4.48E-01	4.22E-02	9.78E-01	5.92E-07	6.49E-01	1.84E-03	-1.65E-01	9.62E-01
ENSBTAG00000029938	-3.21E+00	3.75E-01	-1.22E+00	6.14E-01	-6.54E-01	8.97E-01	-6.71E-01	8.92E-01	-3.21E+00	4.34E-01
ENSBTAG00000006630	-1.18E+00	8.63E-01	-5.89E-01	8.80E-01	-1.34E+00	4.09E-01	-1.95E-01	1.00E+00	4.14E-03	1.00E+00
ENSBTAG00000016896	1.46E+00	5.93E-02	3.86E+00	1.40E-11	5.73E-01	4.51E-01	6.24E-01	4.15E-01	-6.28E-02	1.00E+00
ENSBTAG00000005413	5.04E-01	8.62E-01	3.22E+00	6.46E-10	1.60E+00	2.20E-03	3.47E-01	7.48E-01	-3.25E-01	1.00E+00
ENSBTAG00000002233	5.99E-01	6.66E-01	3.10E+00	1.00E-13	1.68E+00	7.67E-05	9.96E-01	3.59E-02	-3.69E-01	9.65E-01
ENSBTAG00000002242	2.28E-01	8.40E-01	1.02E+00	1.99E-06	8.62E-01	6.61E-05	3.90E-01	1.35E-01	1.15E-01	1.00E+00
ENSBTAG00000004842	1.58E+00	6.19E-05	2.69E+00	3.32E-14	2.04E+00	5.05E-09	1.06E+00	4.57E-03	-2.73E-01	9.89E-01
ENSBTAG00000004844	-1.46E-01	1.00E+00	1.16E+00	7.00E-05	-1.78E-01	8.22E-01	-3.46E-02	1.00E+00	-1.98E-01	1.00E+00
ENSBTAG00000011700	-2.43E-01	1.00E+00	-1.74E+00	7.78E-08	-3.18E+00	5.57E-21	-2.56E+00	8.85E-15	2.50E-01	9.75E-01
ENSBTAG00000017718	8.64E-01	8.29E-01	-3.09E-01	1.00E+00	-1.47E+00	2.13E-01	-7.75E-01	6.12E-01	8.95E-01	8.00E-01
ENSBTAG00000024869	1.20E+00	2.55E-02	1.07E+00	1.16E-02	1.01E+00	1.77E-02	4.64E-01	4.18E-01	5.88E-01	6.09E-01
ENSBTAG00000001191	6.21E+00	5.59E-05	3.68E+00	6.04E-02	2.57E+00	2.62E-01	0.00E+00	1.00E+00	0.00E+00	1.00E+00
ENSBTAG00000001854	-3.93E-01	6.75E-01	-1.02E+00	5.55E-04	1.06E-01	1.00E+00	6.26E-01	5.29E-02	-3.77E-01	7.27E-01
ENSBTAG00000001855	-1.24E-01	1.00E+00	-3.59E-01	1.83E-01	1.32E-01	8.22E-01	3.20E-01	2.64E-01	-3.27E-02	1.00E+00
ENSBTAG00000001856	1.24E-01	1.00E+00	7.12E-01	1.27E-04	8.05E-01	1.07E-05	3.11E-01	1.68E-01	-1.52E-01	9.62E-01
ENSBTAG00000001857	5.10E-02	1.00E+00	-1.73E+00	4.23E-07	-2.15E+00	2.58E-10	-1.02E+00	4.01E-03	3.18E-02	1.00E+00
ENSBTAG00000000462	-3.91E-01	6.87E-01	-1.70E+00	1.59E-08	-1.81E+00	9.35E-10	-1.45E+00	1.09E-06	5.52E-01	3.84E-01
ENSBTAG00000005170	3.33E-02	1.00E+00	-4.40E-01	6.09E-01	-2.06E-01	9.92E-01	-8.17E-01	2.05E-01	-9.46E-01	4.49E-01
ENSBTAG00000038141	-2.24E-01	1.00E+00	-6.85E-01	1.02E-01	7.00E-02	1.00E+00	-9.04E-02	1.00E+00	-1.58E-01	1.00E+00
ENSBTAG00000046323	0.00E+00	1.00E+00	0.00E+00	1.00E+00	-5.13E-15	1.00E+00	0.00E+00	1.00E+00	0.00E+00	1.00E+00

ENSBTAG00000006221	4.06E+00	3.02E-04	4.31E+00	5.62E-06	4.74E+00	4.50E-08	5.51E+00	2.60E-12	3.18E+00	6.79E-02
ENSBTAG00000008005	5.64E-01	7.77E-01	9.85E-01	4.38E-02	1.66E+00	5.36E-05	9.53E-01	5.27E-02	1.04E+00	1.98E-01
ENSBTAG00000001848	-4.19E-01	5.76E-01	-1.62E+00	1.50E-08	-1.34E+00	2.01E-06	-3.96E-01	2.71E-01	2.32E-01	9.44E-01
ENSBTAG00000007181	1.93E-01	1.00E+00	5.11E-01	6.60E-02	1.10E+00	8.17E-06	1.14E+00	4.44E-06	5.26E-02	1.00E+00
ENSBTAG00000005702	1.11E+00	7.18E-01	3.44E-01	1.00E+00	-1.68E-01	1.00E+00	2.65E-01	1.00E+00	-6.69E-01	1.00E+00
ENSBTAG00000012785	-1.95E+00	4.79E-04	-3.69E+00	8.22E-10	-5.12E+00	5.38E-14	-3.02E+00	1.16E-08	-7.67E-01	4.59E-01
ENSBTAG00000045572	-9.58E-01	1.35E-02	-8.78E-01	5.22E-03	2.09E-01	6.79E-01	1.13E-01	9.96E-01	2.98E-01	8.00E-01
ENSBTAG00000015758	-4.63E-01	1.97E-01	-1.14E-01	8.78E-01	2.63E-01	3.39E-01	-8.74E-02	9.90E-01	-8.77E-03	1.00E+00
ENSBTAG00000012919	-4.92E-01	2.70E-01	-1.72E+00	1.01E-12	-2.97E+00	5.13E-33	-2.14E+00	9.31E-19	1.82E-01	1.00E+00
ENSBTAG00000013874	-1.25E-01	1.00E+00	-1.07E+00	1.54E-05	1.26E-01	8.87E-01	3.91E-01	1.80E-01	-8.95E-03	1.00E+00
ENSBTAG00000013784	-3.45E-01	2.87E-01	-3.76E-01	5.00E-02	-1.56E-01	5.64E-01	-1.22E-01	7.40E-01	-1.62E-01	8.88E-01
ENSBTAG00000014144	-1.38E+00	1.61E-01	-3.35E+00	1.57E-06	-5.74E+00	1.13E-11	-3.68E+00	1.35E-07	-9.63E-02	1.00E+00
ENSBTAG00000014147	-4.97E-01	1.00E+00	9.11E-02	1.00E+00	-1.44E+00	4.79E-01	-6.86E-01	8.84E-01	5.60E-04	1.00E+00
ENSBTAG00000043897	0.00E+00	1.00E+00	0.00E+00	1.00E+00	-5.13E-15	1.00E+00	0.00E+00	1.00E+00	0.00E+00	1.00E+00
ENSBTAG00000009363	-3.94E-01	8.23E-01	-1.38E+00	4.90E-04	-7.00E-02	1.00E+00	6.53E-01	1.23E-01	7.00E-02	1.00E+00
ENSBTAG00000003894	-3.92E-01	1.00E+00	2.84E-02	1.00E+00	-3.70E-01	8.34E-01	-1.40E+00	8.58E-02	-7.79E-02	1.00E+00
ENSBTAG00000020781	-6.75E-01	7.39E-03	-2.11E+00	3.70E-25	-1.57E+00	2.23E-15	-1.36E+00	6.69E-12	-3.86E-01	3.99E-01
ENSBTAG00000020782	-4.22E-01	1.73E-01	1.02E-01	8.78E-01	-5.79E-01	2.67E-03	-6.26E-01	1.27E-03	-2.85E-01	6.01E-01
ENSBTAG00000043528	0.00E+00	1.00E+00	0.00E+00	1.00E+00	-5.13E-15	1.00E+00	0.00E+00	1.00E+00	0.00E+00	1.00E+00
ENSBTAG00000043192	0.00E+00	1.00E+00	0.00E+00	1.00E+00	-5.13E-15	1.00E+00	0.00E+00	1.00E+00	0.00E+00	1.00E+00
ENSBTAG00000007170	1.36E+00	5.61E-06	1.04E+00	1.91E-04	-3.93E-01	2.55E-01	-1.57E+00	1.30E-08	-2.56E-01	8.89E-01
ENSBTAG00000007172	-1.45E-01	1.00E+00	-3.13E-01	3.16E-01	-8.02E-01	9.88E-04	-9.24E-01	1.45E-04	-1.56E-01	1.00E+00
ENSBTAG00000008786	0.00E+00	1.00E+00	0.00E+00	1.00E+00	-5.13E-15	1.00E+00	0.00E+00	1.00E+00	1.91E+00	8.00E-01
ENSBTAG00000047938	-1.91E+00	8.23E-01	-1.91E+00	4.69E-01	-1.91E+00	4.28E-01	-1.91E+00	4.39E-01	-1.91E+00	8.00E-01
ENSBTAG00000046851	0.00E+00	1.00E+00	0.00E+00	1.00E+00	-5.13E-15	1.00E+00	0.00E+00	1.00E+00	0.00E+00	1.00E+00
ENSBTAG00000045255	0.00E+00	1.00E+00	0.00E+00	1.00E+00	-5.13E-15	1.00E+00	0.00E+00	1.00E+00	0.00E+00	1.00E+00
ENSBTAG00000047460	0.00E+00	1.00E+00	0.00E+00	1.00E+00	-5.13E-15	1.00E+00	0.00E+00	1.00E+00	0.00E+00	1.00E+00
ENSBTAG00000042756	0.00E+00	1.00E+00	0.00E+00	1.00E+00	-5.13E-15	1.00E+00	0.00E+00	1.00E+00	0.00E+00	1.00E+00
ENSBTAG00000046749	8.23E-01	1.00E+00	1.40E+00	5.34E-01	-1.31E-01	1.00E+00	3.06E+00	1.72E-02	7.95E-01	1.00E+00
ENSBTAG00000022783	-4.51E-01	1.00E+00	7.58E-01	4.25E-01	-4.38E-02	1.00E+00	1.34E+00	5.48E-02	-3.16E-01	1.00E+00
ENSBTAG00000042598	0.00E+00	1.00E+00	0.00E+00	1.00E+00	-5.13E-15	1.00E+00	0.00E+00	1.00E+00	0.00E+00	1.00E+00
ENSBTAG00000032092	1.08E+00	1.63E-01	3.34E+00	2.98E-12	3.96E+00	2.96E-16	4.36E+00	8.87E-19	3.11E-01	1.00E+00
ENSBTAG00000037322	0.00E+00	1.00E+00	0.00E+00	1.00E+00	-5.13E-15	1.00E+00	0.00E+00	1.00E+00	0.00E+00	1.00E+00
ENSBTAG00000042792	1.92E+00	8.23E-01	0.00E+00	1.00E+00	-5.13E-15	1.00E+00	0.00E+00	1.00E+00	0.00E+00	1.00E+00
ENSBTAG00000042254	0.00E+00	1.00E+00	0.00E+00	1.00E+00	-5.13E-15	1.00E+00	0.00E+00	1.00E+00	0.00E+00	1.00E+00

ENSBTAG00000045692	-4.82E-02	1.00E+00	-4.73E-01	3.61E-01	2.51E-01	7.43E-01	3.83E-01	4.79E-01	-5.28E-02	1.00E+00
ENSBTAG00000007421	-2.87E-01	8.81E-01	-3.00E-01	4.75E-01	-7.42E-02	1.00E+00	3.09E-01	4.69E-01	-2.37E-02	1.00E+00
ENSBTAG00000009762	1.01E-01	1.00E+00	-2.11E+00	1.08E-02	-2.36E+00	3.07E-03	-2.10E+00	7.92E-03	1.94E+00	1.10E-03
ENSBTAG00000005152	-1.28E-01	1.00E+00	-7.54E-01	2.76E-03	-9.57E-01	8.81E-05	-5.24E-01	5.66E-02	2.38E-02	1.00E+00
ENSBTAG00000038858	-2.89E-01	1.00E+00	-2.20E-01	1.00E+00	-1.68E-01	1.00E+00	-2.14E+00	1.77E-01	2.27E-01	1.00E+00
ENSBTAG00000032576	5.23E-01	1.00E+00	6.04E-01	9.45E-01	-1.42E-01	1.00E+00	-2.70E+00	2.05E-01	1.79E-02	1.00E+00
ENSBTAG00000047654	-2.03E-01	9.43E-01	-8.86E-01	1.73E-04	-5.86E-01	1.65E-02	-6.77E-01	5.35E-03	-6.83E-01	5.64E-02
ENSBTAG00000001104	-5.28E-01	6.52E-02	-2.89E+00	8.14E-46	-3.33E+00	8.54E-59	-1.78E+00	5.93E-20	-1.09E-01	1.00E+00
ENSBTAG00000000458	1.09E-01	1.00E+00	5.46E-01	1.04E-02	7.34E-01	2.91E-04	7.03E-01	7.00E-04	1.87E-01	8.97E-01
ENSBTAG00000016438	3.04E-01	8.33E-01	8.81E-01	3.10E-03	-5.98E-01	7.60E-02	-1.37E+00	1.08E-05	-3.00E-01	8.34E-01
ENSBTAG00000010132	-1.33E-01	1.00E+00	-1.09E+00	6.38E-06	-7.39E-01	3.00E-03	-5.89E-01	2.60E-02	-6.64E-01	8.75E-02
ENSBTAG00000020889	-5.02E-01	1.00E+00	1.57E+00	1.77E-01	-1.43E+00	4.79E-01	-3.21E+00	9.91E-02	1.12E-02	1.00E+00
ENSBTAG00000046708	-1.12E+00	2.10E-02	-1.85E+00	6.81E-07	-7.77E-01	5.43E-02	1.11E+00	3.55E-03	7.16E-01	3.63E-01
ENSBTAG00000009585	1.32E+00	9.30E-01	1.39E+00	5.17E-01	1.82E+00	2.75E-01	6.21E-01	9.94E-01	3.51E+00	2.10E-02
ENSBTAG00000013929	1.75E+00	6.31E-02	4.72E+00	4.14E-12	4.53E+00	1.93E-11	2.79E+00	2.82E-05	1.13E+00	4.90E-01
ENSBTAG00000013931	-2.40E-02	1.00E+00	4.86E-01	1.26E-01	8.28E-01	3.21E-03	1.01E+00	2.85E-04	-3.49E-01	7.59E-01
ENSBTAG00000005093	-2.23E-01	1.00E+00	-3.94E-01	4.43E-01	-1.06E+00	4.70E-03	-1.36E+00	2.43E-04	-4.40E-01	7.79E-01
ENSBTAG00000040413	-5.27E-01	6.86E-01	-1.07E+00	9.38E-03	-1.75E+00	8.85E-06	-1.70E+00	2.02E-05	1.15E-01	1.00E+00
ENSBTAG00000038325	6.04E-01	1.00E+00	5.04E-01	8.66E-01	-1.37E-01	1.00E+00	-4.31E-01	9.70E-01	2.58E+00	8.40E-03
ENSBTAG00000016103	-1.19E-01	1.00E+00	1.03E-01	1.00E+00	4.13E-01	1.79E-01	7.34E-01	6.67E-03	-1.03E-01	1.00E+00
ENSBTAG00000005058	-1.27E+00	8.59E-03	-3.28E+00	1.74E-16	-2.88E+00	1.62E-13	-1.98E+00	3.35E-07	-1.64E-01	1.00E+00
ENSBTAG00000046879	-5.95E-01	7.72E-01	-2.11E+00	2.10E-04	-1.40E+00	8.24E-03	-1.01E+00	6.95E-02	-1.86E-02	1.00E+00
ENSBTAG00000012642	-3.99E-01	4.82E-01	-2.12E-01	5.85E-01	6.91E-01	4.59E-03	6.38E-01	1.13E-02	-2.26E-01	8.79E-01
ENSBTAG00000023632	-9.29E-01	6.49E-02	-3.22E+00	2.39E-15	-2.59E+00	3.73E-12	-7.04E-01	7.29E-02	-3.96E-01	8.00E-01
ENSBTAG00000003514	-3.77E-01	5.42E-01	-1.01E+00	2.50E-05	-1.91E+00	4.90E-16	-2.27E+00	1.98E-21	-6.95E-01	6.10E-02
ENSBTAG00000003519	-1.27E-01	1.00E+00	-9.07E-01	7.93E-02	-9.97E-01	4.53E-02	-8.09E-01	1.29E-01	-2.98E-01	1.00E+00
ENSBTAG00000003521	-1.09E+00	2.20E-01	-3.38E+00	7.63E-07	6.18E-02	1.00E+00	6.07E-01	2.78E-01	-1.04E-01	1.00E+00
ENSBTAG00000012035	-2.68E-01	1.00E+00	-1.55E+00	8.78E-05	-2.68E-01	7.08E-01	-3.16E-01	6.12E-01	-2.03E-01	1.00E+00
ENSBTAG00000012063	3.05E-02	1.00E+00	1.42E+00	4.80E-12	1.80E+00	6.27E-19	1.56E+00	2.02E-14	-7.28E-03	1.00E+00
ENSBTAG00000001286	-1.35E-01	1.00E+00	-9.28E-01	9.54E-06	-1.29E+00	3.38E-10	-1.26E+00	1.27E-09	-1.99E-01	8.69E-01
ENSBTAG00000029999	0.00E+00	1.00E+00	0.00E+00	1.00E+00	-5.13E-15	1.00E+00	0.00E+00	1.00E+00	0.00E+00	1.00E+00
ENSBTAG00000001287	9.99E-02	1.00E+00	-6.69E-01	5.29E-02	-1.97E-01	7.74E-01	-7.54E-01	2.63E-02	2.27E-01	1.00E+00
ENSBTAG00000002327	-1.12E-01	1.00E+00	-2.92E-01	3.59E-01	-4.43E-01	1.00E-01	-2.70E-01	4.27E-01	-4.19E-01	4.57E-01
ENSBTAG00000032427	4.40E-02	1.00E+00	-1.34E+00	8.02E-06	-3.84E-01	3.20E-01	-4.97E-01	1.66E-01	-1.37E-01	1.00E+00
ENSBTAG00000039190	2.08E-01	1.00E+00	4.83E-01	1.96E-01	-3.82E-02	1.00E+00	-7.37E-01	4.09E-02	-4.88E-02	1.00E+00

ENSBTAG00000021570	-4.90E-01	1.00E+00	-1.22E+00	6.14E-01	5.18E-01	9.34E-01	-3.21E+00	9.97E-02	7.61E-03	1.00E+00
ENSBTAG00000032396	-4.36E-01	1.00E+00	-2.08E+00	3.38E-02	-3.10E+00	1.66E-03	-1.98E+00	4.22E-02	-4.56E-01	1.00E+00
ENSBTAG00000001838	2.39E-01	1.00E+00	-1.32E+00	4.71E-01	-2.03E+00	2.05E-01	-1.73E+00	3.13E-01	4.42E-02	1.00E+00
ENSBTAG00000046690	0.00E+00	1.00E+00	0.00E+00	1.00E+00	-5.13E-15	1.00E+00	0.00E+00	1.00E+00	0.00E+00	1.00E+00
ENSBTAG00000019822	-1.51E-02	1.00E+00	-1.35E+00	1.45E-01	-2.52E+00	6.16E-03	-6.92E-01	5.03E-01	4.33E-01	1.00E+00
ENSBTAG00000038134	-2.83E-01	8.23E-01	-1.61E+00	2.13E-10	-1.57E+00	2.80E-10	-1.79E+00	9.79E-13	-4.52E-03	1.00E+00
ENSBTAG00000005685	-1.84E+00	1.41E-01	-2.39E-01	1.00E+00	-2.31E+00	6.16E-03	-1.24E+00	1.39E-01	-1.18E+00	5.43E-01
ENSBTAG00000014553	-4.89E-02	1.00E+00	2.82E-01	2.42E-01	1.66E-01	5.95E-01	1.83E-01	5.39E-01	3.64E-03	1.00E+00
ENSBTAG00000014556	1.92E+00	8.23E-01	0.00E+00	1.00E+00	-5.13E-15	1.00E+00	0.00E+00	1.00E+00	0.00E+00	1.00E+00
ENSBTAG00000008860	6.54E-02	1.00E+00	-2.34E-01	6.71E-01	-1.14E-01	1.00E+00	3.27E-02	1.00E+00	1.27E-01	1.00E+00
ENSBTAG00000013757	0.00E+00	1.00E+00	0.00E+00	1.00E+00	-5.13E-15	1.00E+00	0.00E+00	1.00E+00	0.00E+00	1.00E+00
ENSBTAG00000018900	-1.46E-01	1.00E+00	1.76E-02	1.00E+00	3.58E-01	4.14E-01	-2.58E-01	6.64E-01	3.91E-01	7.73E-01
ENSBTAG00000018903	-2.70E-01	8.80E-01	-1.45E+00	5.07E-07	-5.28E-01	9.32E-02	-2.93E-01	4.66E-01	-5.08E-02	1.00E+00
ENSBTAG00000023734	-3.73E-01	1.00E+00	-6.08E-01	3.47E-01	-1.70E+00	1.83E-03	-1.29E+00	1.82E-02	1.19E-01	1.00E+00
ENSBTAG00000018904	-5.72E-01	6.71E-01	-2.92E+00	2.93E-09	-1.73E+00	9.58E-05	-1.39E+00	2.08E-03	2.37E-01	1.00E+00
ENSBTAG00000018906	-1.14E-01	1.00E+00	-2.70E+00	7.57E-05	-1.68E+00	5.21E-03	-2.99E+00	8.90E-06	-2.47E-01	1.00E+00
ENSBTAG00000018908	-1.23E+00	1.07E-03	-2.65E+00	4.07E-16	-1.92E+00	1.18E-09	-1.12E+00	6.36E-04	-3.22E-01	8.26E-01
ENSBTAG00000032763	-2.04E-01	9.76E-01	-1.15E+00	2.01E-06	-5.64E-01	3.18E-02	-2.06E-01	6.17E-01	5.32E-01	2.64E-01
ENSBTAG00000006387	-6.18E-01	1.00E+00	-9.77E-01	4.15E-01	-7.85E-01	4.91E-01	-1.55E+00	1.40E-01	5.30E-01	9.83E-01
ENSBTAG00000006404	-6.10E-01	7.01E-01	-2.03E+00	8.66E-05	-2.11E+00	2.40E-05	-2.34E+00	5.33E-06	2.17E-01	1.00E+00
ENSBTAG00000006411	-8.76E-01	7.29E-04	-1.31E+00	3.24E-09	-1.33E+00	1.41E-09	-8.75E-01	1.07E-04	-1.48E-01	1.00E+00
ENSBTAG00000006414	1.53E-01	1.00E+00	1.05E+00	3.51E-08	8.11E-01	2.61E-05	4.82E-01	2.35E-02	-2.05E-01	8.10E-01
ENSBTAG00000006417	4.52E-02	1.00E+00	5.07E-01	2.20E-02	9.71E-01	1.50E-06	8.38E-01	5.30E-05	1.02E-01	1.00E+00
ENSBTAG00000018037	-2.05E-01	1.00E+00	1.73E-01	8.52E-01	4.92E-01	1.69E-01	5.24E-01	1.45E-01	6.30E-01	3.09E-01
ENSBTAG00000018040	-1.20E-01	1.00E+00	4.90E-01	4.25E-01	1.08E+00	2.00E-02	9.19E-01	6.20E-02	-4.05E-01	8.86E-01
ENSBTAG00000018043	-1.10E-01	1.00E+00	-7.67E-01	1.02E-04	-1.66E+00	3.80E-18	-1.88E+00	1.18E-22	4.91E-01	1.54E-01
ENSBTAG00000018045	3.49E-01	6.84E-01	6.83E-01	1.21E-02	8.74E-01	8.22E-04	9.56E-01	2.69E-04	4.45E-01	4.87E-01
ENSBTAG00000002824	-4.52E-01	1.00E+00	-1.29E+00	1.22E-01	-2.05E+00	8.69E-03	-1.57E+00	5.19E-02	6.94E-01	8.06E-01
ENSBTAG00000039426	-2.46E-01	1.00E+00	-1.29E+00	1.32E-01	-7.10E-01	4.36E-01	-1.40E+00	8.67E-02	9.58E-01	4.81E-01
ENSBTAG00000004290	-4.38E-01	5.76E-01	-5.58E-02	1.00E+00	1.47E-01	8.93E-01	-2.87E-02	1.00E+00	-5.15E-02	1.00E+00
ENSBTAG00000014644	-2.90E-01	8.23E-01	-1.96E+00	1.69E-13	-1.55E+00	2.50E-09	-1.18E+00	7.31E-06	-7.25E-02	1.00E+00
ENSBTAG00000004368	-4.34E-01	1.82E-01	-1.50E+00	7.10E-15	-1.34E+00	2.30E-12	-8.95E-01	4.62E-06	-2.64E-01	6.93E-01
ENSBTAG00000042313	0.00E+00	1.00E+00	0.00E+00	1.00E+00	-5.13E-15	1.00E+00	0.00E+00	1.00E+00	0.00E+00	1.00E+00
ENSBTAG00000004375	-6.34E-01	5.81E-02	-3.28E+00	5.21E-42	-4.35E+00	4.31E-66	-1.27E+00	5.15E-08	1.95E-01	9.46E-01
ENSBTAG00000007512	-3.05E-01	9.78E-01	-1.90E+00	5.97E-06	-4.58E-01	3.44E-01	8.33E-02	1.00E+00	-5.62E-01	6.01E-01

ENSBTAG00000009195	0.00E+00	1.00E+00	0.00E+00	1.00E+00	-5.13E-15	1.00E+00	0.00E+00	1.00E+00	0.00E+00	1.00E+00
ENSBTAG00000012112	3.37E-01	7.59E-01	-9.92E-02	1.00E+00	8.81E-02	1.00E+00	1.27E-01	9.41E-01	-4.13E-01	6.03E-01
ENSBTAG00000009636	-1.46E-01	1.00E+00	1.05E+00	1.48E-08	1.03E+00	2.32E-08	5.37E-01	8.31E-03	-4.24E-01	2.84E-01
ENSBTAG00000009638	-5.58E-02	1.00E+00	4.78E-01	3.66E-02	9.46E-01	4.21E-06	7.55E-01	4.13E-04	7.03E-02	1.00E+00
ENSBTAG00000043719	0.00E+00	1.00E+00	0.00E+00	1.00E+00	-5.13E-15	1.00E+00	0.00E+00	1.00E+00	0.00E+00	1.00E+00
ENSBTAG00000009639	-4.42E-01	7.68E-01	-2.64E+00	7.50E-13	-2.17E+00	1.59E-09	-2.35E+00	1.15E-10	5.18E-01	6.13E-01
ENSBTAG00000007488	3.75E-01	5.99E-01	1.67E-01	7.90E-01	4.65E-01	1.14E-01	5.07E-01	8.38E-02	-2.46E-01	8.85E-01
ENSBTAG00000033248	-4.23E-01	7.56E-01	-2.05E+00	7.61E-08	4.78E-02	1.00E+00	-2.29E-01	7.44E-01	2.26E-01	1.00E+00
ENSBTAG00000015991	3.95E-01	9.20E-01	3.82E-01	5.71E-01	-5.23E-01	3.63E-01	2.47E-01	8.47E-01	2.46E-01	1.00E+00
ENSBTAG00000013941	1.02E-01	1.00E+00	2.86E-01	2.86E-01	4.30E-01	6.42E-02	3.45E-01	1.76E-01	-1.24E-01	1.00E+00
ENSBTAG00000047471	0.00E+00	1.00E+00	0.00E+00	1.00E+00	-5.13E-15	1.00E+00	0.00E+00	1.00E+00	0.00E+00	1.00E+00
ENSBTAG00000007992	1.47E-01	1.00E+00	1.41E-01	1.00E+00	8.86E-01	8.61E-02	5.48E-01	4.13E-01	9.65E-01	3.09E-01
ENSBTAG00000001698	7.96E-02	1.00E+00	8.07E-01	2.17E-04	7.28E-01	9.36E-04	5.19E-01	2.95E-02	-6.04E-02	1.00E+00
ENSBTAG00000006347	3.19E-01	6.14E-01	2.33E+00	4.00E-28	2.37E+00	2.23E-29	1.53E+00	4.76E-13	-1.49E-01	1.00E+00
ENSBTAG00000001654	-3.27E-01	8.23E-01	7.50E-01	1.31E-02	6.61E-01	3.21E-02	3.53E-01	3.74E-01	-4.19E-01	6.43E-01
ENSBTAG00000001659	-1.36E-01	1.00E+00	2.48E-01	3.74E-01	2.10E-01	4.79E-01	1.04E-01	9.10E-01	3.39E-02	1.00E+00
ENSBTAG00000001663	-1.71E-01	1.00E+00	-1.38E+00	1.41E-07	-8.04E-01	2.22E-03	-4.57E-01	1.27E-01	-2.40E-01	8.73E-01
ENSBTAG00000001665	-2.50E-01	7.67E-01	-2.39E-01	3.90E-01	2.53E-01	3.34E-01	2.13E-01	4.66E-01	-6.20E-02	1.00E+00
ENSBTAG00000001666	4.69E-02	1.00E+00	2.24E+00	1.39E-07	2.71E+00	1.60E-10	2.42E+00	1.35E-08	-2.82E-01	1.00E+00
ENSBTAG00000007616	6.73E-02	1.00E+00	1.49E+00	5.00E-12	1.96E+00	5.48E-20	1.21E+00	2.56E-08	-3.01E-01	7.03E-01
ENSBTAG00000007617	6.15E-02	1.00E+00	5.13E-01	1.84E-02	6.53E-01	1.69E-03	4.59E-01	4.34E-02	-2.29E-02	1.00E+00
ENSBTAG00000002412	2.65E-01	9.32E-01	1.29E+00	9.23E-06	1.03E+00	4.73E-04	9.87E-01	1.10E-03	1.71E-01	1.00E+00
ENSBTAG00000013412	3.59E-01	8.98E-01	8.91E-01	2.74E-02	-2.41E-01	7.87E-01	5.38E-01	2.64E-01	-3.03E-02	1.00E+00
ENSBTAG00000020632	-4.80E-01	4.12E-01	-1.17E+00	2.05E-05	-2.20E+00	7.14E-16	-2.16E+00	2.88E-15	-5.84E-01	2.96E-01
ENSBTAG00000020633	-2.77E-01	7.29E-01	1.01E+00	1.02E-06	1.55E+00	1.68E-14	9.01E-01	1.91E-05	-1.34E-01	1.00E+00
ENSBTAG00000018421	-1.22E-01	1.00E+00	-1.00E+00	6.13E-07	-7.52E-01	2.33E-04	-8.44E-01	3.87E-05	6.10E-02	1.00E+00
ENSBTAG00000045115	0.00E+00	1.00E+00	0.00E+00	1.00E+00	-5.13E-15	1.00E+00	0.00E+00	1.00E+00	0.00E+00	1.00E+00
ENSBTAG00000029771	0.00E+00	1.00E+00	0.00E+00	1.00E+00	-5.13E-15	1.00E+00	0.00E+00	1.00E+00	0.00E+00	1.00E+00
ENSBTAG00000018425	-2.75E-02	1.00E+00	7.44E-01	4.05E-04	5.77E-01	8.05E-03	4.74E-01	4.10E-02	-1.31E-01	1.00E+00
ENSBTAG00000014636	-1.01E-01	1.00E+00	7.39E-01	1.52E-02	6.45E-01	3.91E-02	2.62E-01	5.67E-01	1.60E-01	1.00E+00
ENSBTAG00000042231	0.00E+00	1.00E+00	0.00E+00	1.00E+00	-5.13E-15	1.00E+00	0.00E+00	1.00E+00	0.00E+00	1.00E+00
ENSBTAG00000044267	0.00E+00	1.00E+00	0.00E+00	1.00E+00	-5.13E-15	1.00E+00	0.00E+00	1.00E+00	0.00E+00	1.00E+00
ENSBTAG00000013266	-3.60E+00	2.45E-01	3.82E-01	1.00E+00	-1.82E+00	3.14E-01	1.13E+00	4.06E-01	-8.76E-01	1.00E+00
ENSBTAG00000013265	4.88E-01	3.51E-01	1.32E+00	2.14E-07	1.28E+00	4.58E-07	1.01E+00	1.35E-04	3.51E-02	1.00E+00
ENSBTAG00000006353	1.48E+00	2.21E-05	3.00E+00	5.01E-21	1.93E+00	5.69E-10	8.50E-01	1.24E-02	-3.82E-02	1.00E+00

ENSBTAG00000006354	5.82E+00	3.90E-32	7.44E+00	2.81E-46	7.84E+00	7.05E-50	7.94E+00	1.13E-50	7.34E+00	1.85E-44
ENSBTAG00000019887	5.16E+00	3.12E-41	6.27E+00	7.62E-56	5.51E+00	1.17E-46	4.55E+00	6.63E-35	1.63E-01	1.00E+00
ENSBTAG00000046908	0.00E+00	1.00E+00	0.00E+00	1.00E+00	-5.13E-15	1.00E+00	0.00E+00	1.00E+00	0.00E+00	1.00E+00
ENSBTAG00000019884	2.40E+00	8.16E-24	2.85E+00	2.33E-33	1.48E+00	1.67E-10	6.20E-02	1.00E+00	3.94E-01	5.25E-01
ENSBTAG00000019883	8.10E-01	1.09E-03	8.31E-01	9.14E-05	1.03E-01	9.27E-01	-1.34E-01	8.04E-01	-3.09E-01	6.21E-01
ENSBTAG00000021438	1.79E-01	1.00E+00	-1.17E+00	3.54E-03	-8.92E-01	3.05E-02	-8.42E-01	4.82E-02	-4.72E-01	7.69E-01
ENSBTAG00000044454	0.00E+00	1.00E+00	0.00E+00	1.00E+00	-5.13E-15	1.00E+00	0.00E+00	1.00E+00	0.00E+00	1.00E+00
ENSBTAG00000042970	1.06E+00	1.84E-01	6.31E-01	3.56E-01	2.10E-01	9.92E-01	5.62E-01	4.38E-01	6.64E-01	7.13E-01
ENSBTAG00000046255	-1.63E-02	1.00E+00	-1.03E-01	1.00E+00	-3.67E-01	2.70E-01	-1.23E-01	9.44E-01	4.19E-01	5.53E-01
ENSBTAG00000047787	7.19E-02	1.00E+00	-5.77E-01	5.14E-01	-1.37E+00	4.69E-02	-8.04E-01	2.92E-01	4.07E-01	9.88E-01
ENSBTAG00000002769	5.24E-02	1.00E+00	-4.24E-01	1.77E-01	-1.88E-01	7.12E-01	1.25E-01	9.33E-01	2.90E-01	8.00E-01
ENSBTAG00000043472	0.00E+00	1.00E+00	0.00E+00	1.00E+00	-5.13E-15	1.00E+00	0.00E+00	1.00E+00	0.00E+00	1.00E+00
ENSBTAG00000007545	-7.27E-01	3.86E-01	-9.91E-01	2.91E-02	4.65E-02	1.00E+00	1.07E+00	3.45E-03	-8.35E-02	1.00E+00
ENSBTAG00000007544	-2.86E-01	1.00E+00	-1.25E+00	2.36E-01	-2.06E+00	2.94E-02	-1.49E+00	1.43E-01	-1.00E+00	7.96E-01
ENSBTAG00000002214	-4.42E-02	1.00E+00	-2.40E+00	9.77E-05	-1.56E+00	1.44E-02	-2.06E-01	1.00E+00	2.46E-01	1.00E+00
ENSBTAG00000043286	0.00E+00	1.00E+00	0.00E+00	1.00E+00	-5.13E-15	1.00E+00	0.00E+00	1.00E+00	0.00E+00	1.00E+00
ENSBTAG00000016968	0.00E+00	1.00E+00	3.30E+00	1.02E-01	3.05E+00	1.36E-01	1.77E+00	4.97E-01	0.00E+00	1.00E+00
ENSBTAG00000032018	1.11E-02	1.00E+00	-1.26E+00	8.66E-05	1.84E-01	8.24E-01	-1.94E-01	8.08E-01	2.38E-01	9.93E-01
ENSBTAG00000023617	2.72E+00	5.76E-01	0.00E+00	1.00E+00	-5.13E-15	1.00E+00	1.77E+00	4.97E-01	2.71E+00	6.01E-01
ENSBTAG00000001075	-2.88E-01	1.00E+00	8.72E-02	1.00E+00	-1.34E+00	4.12E-01	9.86E-01	4.17E-01	-1.18E+00	8.34E-01
ENSBTAG00000047367	2.58E-01	1.00E+00	2.23E+00	2.16E-09	2.05E+00	3.28E-08	1.33E+00	5.63E-04	-1.88E-02	1.00E+00
ENSBTAG00000010878	8.26E-02	1.00E+00	2.34E-01	1.00E+00	-5.88E-01	5.12E-01	1.89E-01	1.00E+00	-8.14E-02	1.00E+00
ENSBTAG00000029040	0.00E+00	1.00E+00	0.00E+00	1.00E+00	-5.13E-15	1.00E+00	0.00E+00	1.00E+00	0.00E+00	1.00E+00
ENSBTAG00000045240	0.00E+00	1.00E+00	0.00E+00	1.00E+00	-5.13E-15	1.00E+00	0.00E+00	1.00E+00	0.00E+00	1.00E+00
ENSBTAG00000043877	0.00E+00	1.00E+00	0.00E+00	1.00E+00	-5.13E-15	1.00E+00	0.00E+00	1.00E+00	0.00E+00	1.00E+00
ENSBTAG00000002223	1.69E-01	1.00E+00	1.02E+00	1.39E-06	8.48E-01	7.55E-05	4.22E-01	9.15E-02	-7.38E-02	1.00E+00
ENSBTAG00000008625	-1.85E+00	1.39E-04	-2.99E+00	5.92E-11	-3.05E+00	1.00E-11	-2.72E+00	7.91E-10	-1.09E+00	1.20E-01
ENSBTAG00000047453	0.00E+00	1.00E+00	0.00E+00	1.00E+00	-5.13E-15	1.00E+00	0.00E+00	1.00E+00	0.00E+00	1.00E+00
ENSBTAG00000020434	-4.42E-01	7.25E-01	7.78E-01	3.21E-02	6.47E-01	8.86E-02	7.60E-01	4.09E-02	-2.83E-01	9.43E-01
ENSBTAG00000004735	-7.06E-01	4.75E-01	-6.63E-01	2.04E-01	1.71E+00	4.24E-07	1.57E+00	5.44E-06	-2.10E-02	1.00E+00
ENSBTAG00000007559	-1.44E-01	1.00E+00	1.14E-01	9.24E-01	1.18E+00	1.33E-07	6.69E-01	5.95E-03	-3.20E-01	6.79E-01
ENSBTAG00000007262	-7.00E-01	9.59E-01	3.67E-03	1.00E+00	-7.09E-01	5.66E-01	2.38E-02	1.00E+00	-5.57E-01	1.00E+00
ENSBTAG00000046065	0.00E+00	1.00E+00	0.00E+00	1.00E+00	-5.13E-15	1.00E+00	0.00E+00	1.00E+00	0.00E+00	1.00E+00
ENSBTAG00000002687	7.83E-01	5.76E-01	2.81E-01	9.08E-01	5.15E-01	4.99E-01	4.76E-01	5.73E-01	3.28E-01	1.00E+00
ENSBTAG00000047269	0.00E+00	1.00E+00	0.00E+00	1.00E+00	-5.13E-15	1.00E+00	0.00E+00	1.00E+00	0.00E+00	1.00E+00

ENSBTAG00000040514	0.00E+00	1.00E+00	0.00E+00	1.00E+00	-5.13E-15	1.00E+00	0.00E+00	1.00E+00	0.00E+00	1.00E+00
ENSBTAG00000048104	3.15E+00	5.35E-17	4.18E+00	3.58E-33	3.31E+00	2.34E-20	2.87E+00	8.55E-15	8.47E-01	4.34E-01
ENSBTAG00000044724	0.00E+00	1.00E+00	0.00E+00	1.00E+00	-5.13E-15	1.00E+00	0.00E+00	1.00E+00	0.00E+00	1.00E+00
ENSBTAG00000044307	0.00E+00	1.00E+00	0.00E+00	1.00E+00	-5.13E-15	1.00E+00	0.00E+00	1.00E+00	0.00E+00	1.00E+00
ENSBTAG00000003856	-1.04E-01	1.00E+00	1.89E-01	9.73E-01	1.29E+00	2.26E-03	1.10E+00	1.31E-02	3.19E-01	1.00E+00
ENSBTAG00000014123	4.18E-01	7.79E-01	1.98E+00	2.78E-09	1.58E+00	2.15E-06	1.25E+00	2.96E-04	5.35E-01	5.90E-01
ENSBTAG00000015165	-4.21E-01	8.63E-01	-2.05E+00	3.25E-06	-4.55E-01	4.41E-01	9.27E-02	1.00E+00	6.02E-02	1.00E+00
ENSBTAG00000009304	-1.89E-01	9.78E-01	-6.05E-01	1.19E-02	-1.25E-01	8.61E-01	4.54E-02	1.00E+00	-4.16E-01	4.34E-01
ENSBTAG00000009305	1.15E-01	1.00E+00	-2.32E-01	4.19E-01	-3.11E-01	2.08E-01	-2.45E-01	3.85E-01	2.84E-01	6.47E-01
ENSBTAG00000039441	0.00E+00	1.00E+00	0.00E+00	1.00E+00	-5.13E-15	1.00E+00	0.00E+00	1.00E+00	3.21E+00	4.34E-01
ENSBTAG00000023614	-1.20E-02	1.00E+00	-1.75E-01	8.53E-01	6.05E-01	7.49E-02	9.19E-01	3.73E-03	-3.42E-03	1.00E+00
ENSBTAG00000019266	3.25E-01	1.00E+00	-6.49E-01	6.36E-01	-4.80E-02	1.00E+00	-7.63E-01	5.08E-01	3.33E-01	1.00E+00
ENSBTAG00000013338	-3.54E-01	9.23E-01	-1.95E+00	6.10E-07	-3.79E+00	7.23E-20	-2.75E+00	4.77E-12	1.17E+00	4.00E-02
ENSBTAG00000004864	3.43E-01	7.23E-01	3.67E-01	2.71E-01	1.06E+00	3.38E-05	7.93E-01	3.39E-03	1.30E-01	1.00E+00
ENSBTAG00000010403	-4.58E-01	6.05E-01	-1.11E+00	8.67E-04	4.33E-01	2.55E-01	9.44E-01	2.60E-03	7.10E-02	1.00E+00
ENSBTAG00000003089	-2.85E-01	6.49E-01	-1.79E+00	1.61E-18	-1.08E+00	8.17E-08	-2.22E-01	4.56E-01	1.86E-01	8.98E-01
ENSBTAG00000000721	-1.28E-01	1.00E+00	3.41E-01	2.33E-01	7.02E-01	2.76E-03	5.14E-01	4.41E-02	6.40E-02	1.00E+00
ENSBTAG00000028430	0.00E+00	1.00E+00	0.00E+00	1.00E+00	-5.13E-15	1.00E+00	0.00E+00	1.00E+00	0.00E+00	1.00E+00
ENSBTAG00000031810	-8.81E-01	6.00E-01	-6.02E-02	1.00E+00	-5.16E-01	5.76E-01	-2.13E-01	1.00E+00	-9.72E-01	5.64E-01
ENSBTAG00000047062	4.36E-01	1.00E+00	1.02E+00	4.04E-01	8.95E-01	4.76E-01	1.29E+00	2.05E-01	2.11E-01	1.00E+00
ENSBTAG00000046544	-6.29E-01	1.00E+00	9.84E-01	2.95E-01	4.10E-01	8.75E-01	-1.12E+00	3.93E-01	-3.82E-01	1.00E+00
ENSBTAG00000004994	-2.08E+00	3.62E-13	-1.94E+00	1.51E-12	-1.73E+00	2.35E-10	-9.85E-01	5.66E-04	-3.00E-01	8.00E-01
ENSBTAG00000017560	4.88E-01	3.75E-01	2.94E+00	2.15E-29	2.17E+00	3.13E-17	1.50E+00	8.45E-09	8.31E-02	1.00E+00
ENSBTAG00000031941	-2.70E-01	7.29E-01	-1.00E+00	1.19E-06	-1.71E+00	2.86E-17	-2.02E+00	3.99E-23	-2.31E-01	8.00E-01
ENSBTAG00000047670	-1.18E+00	6.53E-01	-1.50E-01	1.00E+00	-1.13E+00	2.75E-01	-1.83E-01	1.00E+00	-7.30E-01	8.97E-01
ENSBTAG00000017956	-1.58E-01	1.00E+00	1.78E+00	2.40E-06	1.35E+00	4.41E-04	6.50E-01	1.58E-01	-3.53E-01	9.01E-01
ENSBTAG00000000630	1.12E-01	1.00E+00	9.04E-02	8.82E-01	5.91E-01	4.54E-04	5.64E-01	1.09E-03	5.16E-02	1.00E+00
ENSBTAG00000044844	0.00E+00	1.00E+00	0.00E+00	1.00E+00	-5.13E-15	1.00E+00	0.00E+00	1.00E+00	0.00E+00	1.00E+00
ENSBTAG00000045370	1.32E+00	9.30E-01	-1.91E+00	4.69E-01	-1.91E+00	4.28E-01	-1.91E+00	4.39E-01	1.75E-02	1.00E+00
ENSBTAG00000013175	1.23E-01	1.00E+00	7.44E-01	1.21E-03	7.30E-01	1.43E-03	2.07E-01	5.66E-01	4.03E-01	4.54E-01
ENSBTAG00000006396	-7.79E-01	6.59E-03	-1.35E+00	2.42E-09	-1.83E+00	3.82E-16	-1.65E+00	3.24E-13	-6.71E-01	6.09E-02
ENSBTAG00000002571	-1.59E-01	1.00E+00	9.54E-01	1.88E-03	1.16E+00	7.81E-05	2.13E-01	7.43E-01	-1.95E-01	1.00E+00
ENSBTAG00000002573	1.10E-01	1.00E+00	1.15E+00	4.28E-09	1.34E+00	4.23E-12	7.08E-01	5.95E-04	-8.58E-02	1.00E+00
ENSBTAG00000007841	4.93E-01	5.76E-01	2.65E-01	6.50E-01	2.99E-01	5.49E-01	-1.60E-01	9.37E-01	7.60E-02	1.00E+00
ENSBTAG00000018080	-8.78E-01	1.00E+00	-1.60E+00	4.08E-01	3.73E-01	1.00E+00	-5.46E-01	9.30E-01	-8.82E-01	1.00E+00

ENSBTAG00000043477	0.00E+00	1.00E+00	0.00E+00	1.00E+00	-5.13E-15	1.00E+00	0.00E+00	1.00E+00	0.00E+00	1.00E+00
ENSBTAG00000045737	0.00E+00	1.00E+00	0.00E+00	1.00E+00	-5.13E-15	1.00E+00	1.77E+00	4.97E-01	1.91E+00	8.00E-01
ENSBTAG00000045778	0.00E+00	1.00E+00	0.00E+00	1.00E+00	-5.13E-15	1.00E+00	0.00E+00	1.00E+00	0.00E+00	1.00E+00
ENSBTAG00000047571	0.00E+00	1.00E+00	0.00E+00	1.00E+00	-5.13E-15	1.00E+00	1.77E+00	4.97E-01	0.00E+00	1.00E+00
ENSBTAG00000047724	-1.91E+00	8.23E-01	-1.91E+00	4.69E-01	-1.91E+00	4.28E-01	-1.91E+00	4.39E-01	-1.91E+00	8.00E-01
ENSBTAG00000023998	6.04E-03	1.00E+00	7.78E-02	1.00E+00	-1.91E+00	4.28E-01	-1.91E+00	4.39E-01	1.84E-02	1.00E+00
ENSBTAG00000009889	-3.25E-01	7.66E-01	4.53E-01	1.29E-01	6.58E-01	1.50E-02	2.37E-01	5.59E-01	-5.75E-01	2.72E-01
ENSBTAG00000009895	2.77E-01	1.00E+00	2.59E-01	7.61E-01	3.46E-01	5.44E-01	-1.22E-01	1.00E+00	1.69E-01	1.00E+00
ENSBTAG00000002380	0.00E+00	1.00E+00	0.00E+00	1.00E+00	1.78E+00	4.79E-01	0.00E+00	1.00E+00	0.00E+00	1.00E+00
ENSBTAG00000002370	1.73E+00	2.99E-04	2.99E+00	6.88E-13	2.41E+00	4.90E-09	1.78E+00	2.39E-05	3.70E-01	9.20E-01
ENSBTAG00000003820	1.55E+00	7.77E-09	4.61E-01	1.27E-01	-2.61E-01	4.89E-01	-3.10E-01	4.05E-01	-2.85E-01	8.00E-01
ENSBTAG00000043683	0.00E+00	1.00E+00	0.00E+00	1.00E+00	-5.13E-15	1.00E+00	0.00E+00	1.00E+00	0.00E+00	1.00E+00
ENSBTAG00000003826	-2.32E-01	1.00E+00	-1.54E+00	5.90E-05	-1.26E+00	8.68E-04	-5.42E-01	2.36E-01	1.45E-01	1.00E+00
ENSBTAG00000003829	-6.80E-02	1.00E+00	-8.34E-01	1.57E-05	-1.35E+00	7.80E-13	-1.29E+00	1.01E-11	2.08E-01	8.00E-01
ENSBTAG00000040005	-3.19E-01	1.00E+00	-1.48E+00	7.92E-03	-2.03E+00	2.28E-04	-1.35E+00	1.33E-02	6.36E-01	6.20E-01
ENSBTAG00000017814	-3.91E-01	7.76E-01	-2.46E+00	1.77E-14	-4.62E+00	2.04E-40	-4.20E+00	8.05E-35	5.89E-01	4.34E-01
ENSBTAG00000017816	-3.13E-01	5.80E-01	-8.80E-01	2.91E-05	-1.89E+00	3.67E-20	-2.34E+00	2.53E-29	-9.02E-02	1.00E+00
ENSBTAG00000018326	-1.13E+00	2.69E-02	-4.37E+00	2.14E-23	-3.97E+00	2.56E-21	-2.89E+00	3.08E-13	1.81E-01	1.00E+00
ENSBTAG00000031441	-2.69E-01	8.23E-01	-8.89E-01	2.60E-04	-8.50E-01	4.55E-04	-7.07E-01	5.08E-03	1.83E-01	9.89E-01
ENSBTAG00000001994	1.91E+00	8.23E-01	1.99E+00	4.43E-01	-5.13E-15	1.00E+00	1.76E+00	4.97E-01	0.00E+00	1.00E+00
ENSBTAG00000017033	-3.65E-01	7.76E-01	-2.21E-01	7.02E-01	1.50E-01	9.05E-01	2.76E-01	5.54E-01	1.94E-01	1.00E+00
ENSBTAG00000017035	-5.54E-02	1.00E+00	-1.18E-01	8.53E-01	1.27E-01	8.07E-01	2.58E-02	1.00E+00	2.35E-01	8.00E-01
ENSBTAG00000017042	8.08E-01	6.13E-02	2.21E+00	1.30E-13	2.02E+00	1.01E-11	1.50E+00	6.60E-07	-2.60E-01	9.23E-01
ENSBTAG00000017044	-6.31E-01	1.00E+00	-1.22E+00	3.68E-01	-2.74E+00	3.18E-02	-3.73E-01	9.65E-01	-9.28E-01	8.44E-01
ENSBTAG00000031420	0.00E+00	1.00E+00	0.00E+00	1.00E+00	-5.13E-15	1.00E+00	0.00E+00	1.00E+00	0.00E+00	1.00E+00
ENSBTAG00000011887	-2.62E-01	1.00E+00	-1.47E+00	1.13E-05	-9.54E-01	5.38E-03	-1.15E+00	7.54E-04	-2.01E-01	1.00E+00
ENSBTAG00000045806	1.26E+00	6.51E-01	9.23E-02	1.00E+00	3.79E-01	1.00E+00	-1.83E+00	3.01E-01	-3.37E-01	1.00E+00
ENSBTAG00000013791	1.19E-01	1.00E+00	-2.81E-02	1.00E+00	-7.61E-02	1.00E+00	-1.81E+00	6.03E-02	-5.38E-01	1.00E+00
ENSBTAG00000045656	1.92E+00	8.23E-01	1.98E+00	4.43E-01	-5.13E-15	1.00E+00	0.00E+00	1.00E+00	0.00E+00	1.00E+00
ENSBTAG00000006566	3.27E+00	4.72E-04	3.37E+00	3.29E-05	2.19E+00	2.37E-02	2.99E+00	4.34E-04	1.66E+00	5.09E-01
ENSBTAG00000005330	1.10E-02	1.00E+00	-7.23E-01	9.45E-01	-1.54E-01	1.00E+00	-1.73E-01	1.00E+00	6.97E-04	1.00E+00
ENSBTAG00000015915	-3.37E-02	1.00E+00	1.75E+00	1.44E-02	-7.31E-01	6.14E-01	-4.69E+00	1.98E-03	-8.00E-01	9.13E-01
ENSBTAG00000015912	-4.89E-01	1.00E+00	-1.22E+00	6.13E-01	2.15E-01	1.00E+00	4.86E-01	9.63E-01	-1.29E+00	9.02E-01
ENSBTAG00000015917	-6.12E-01	5.86E-02	-7.12E-01	2.37E-03	-2.51E-01	4.28E-01	-1.10E+00	1.16E-06	-1.91E-02	1.00E+00
ENSBTAG00000015920	-1.56E-01	1.00E+00	-7.59E-01	5.66E-03	-1.08E+00	4.05E-05	-8.20E-01	2.94E-03	-1.47E-01	1.00E+00

ENSBTAG00000008777	-1.01E+00	1.09E-01	-2.70E+00	2.12E-09	-4.19E+00	2.82E-17	-3.12E+00	7.50E-12	3.23E-01	9.62E-01
ENSBTAG00000045598	-1.76E+00	2.10E-03	-3.05E+00	6.56E-08	-4.07E+00	1.92E-11	-2.86E+00	5.44E-08	3.56E-01	8.99E-01
ENSBTAG00000045997	-1.91E+00	8.23E-01	7.71E-02	1.00E+00	1.15E+00	6.44E-01	-1.91E+00	4.39E-01	6.46E-03	1.00E+00
ENSBTAG00000047277	-1.73E-01	1.00E+00	-7.82E-01	3.83E-03	-1.64E+00	1.88E-10	-1.49E+00	9.38E-09	-2.71E-01	8.06E-01
ENSBTAG00000013436	1.04E-01	1.00E+00	7.14E-02	1.00E+00	1.42E-01	6.94E-01	-1.91E-01	5.03E-01	-1.70E-01	9.08E-01
ENSBTAG00000013440	-2.51E-02	1.00E+00	4.28E-01	8.97E-02	5.79E-01	1.36E-02	4.77E-01	5.69E-02	-3.26E-01	6.16E-01
ENSBTAG00000013444	-6.03E-01	6.39E-01	-1.71E+00	5.66E-04	-2.15E+00	1.17E-05	-5.40E-01	3.36E-01	-4.22E-01	8.38E-01
ENSBTAG00000020543	8.98E-01	4.59E-01	2.20E+00	2.15E-06	1.94E+00	3.64E-05	8.06E-01	2.22E-01	8.34E-01	5.91E-01
ENSBTAG00000023611	-9.35E-01	2.27E-02	-1.81E+00	1.20E-08	-1.00E+00	1.53E-03	-1.48E+00	2.33E-06	2.40E-01	9.70E-01
ENSBTAG00000002763	-1.15E-01	1.00E+00	5.37E-01	2.69E-02	9.08E-01	4.93E-05	5.47E-01	2.66E-02	1.76E-01	9.85E-01
ENSBTAG00000002764	4.87E-01	3.59E-01	2.76E-01	4.68E-01	-1.34E-01	9.03E-01	-1.07E+00	9.34E-05	1.26E-01	1.00E+00
ENSBTAG00000023610	-1.29E-01	1.00E+00	-7.61E-01	1.44E-03	-4.69E-01	6.87E-02	-5.05E-01	5.07E-02	-1.49E-01	1.00E+00
ENSBTAG00000018593	-1.06E-01	1.00E+00	-5.84E-01	1.98E-02	-9.58E-01	4.22E-05	-7.40E-01	2.48E-03	-2.24E-01	8.67E-01
ENSBTAG00000018594	-3.18E-01	5.76E-01	-1.44E-01	7.57E-01	2.50E-01	3.91E-01	-1.19E-01	8.64E-01	-9.01E-02	1.00E+00
ENSBTAG00000018598	-3.70E-01	7.04E-01	-6.75E-01	2.54E-02	-1.46E+00	1.53E-07	-1.72E+00	7.48E-10	-6.06E-01	2.83E-01
ENSBTAG00000018599	-7.32E-01	4.95E-01	-1.81E+00	3.36E-04	-6.18E-01	2.61E-01	-7.24E-01	1.78E-01	-1.17E-02	1.00E+00
ENSBTAG00000009714	-2.44E-01	1.00E+00	-1.34E+00	2.47E-02	-9.85E-01	9.33E-02	-1.08E+00	6.85E-02	-2.55E-01	1.00E+00
ENSBTAG00000031772	-3.41E-01	1.00E+00	-9.00E-01	5.20E-01	-2.11E+00	4.63E-02	-2.19E+00	4.29E-02	5.49E-01	1.00E+00
ENSBTAG00000007821	-7.79E-01	1.00E+00	-7.17E-01	9.45E-01	-2.70E+00	1.99E-01	-2.70E+00	2.05E-01	1.79E-02	1.00E+00
ENSBTAG00000042589	0.00E+00	1.00E+00	0.00E+00	1.00E+00	-5.13E-15	1.00E+00	0.00E+00	1.00E+00	0.00E+00	1.00E+00
ENSBTAG00000007826	1.01E+00	9.01E-01	9.08E-01	6.03E-01	3.08E-01	1.00E+00	-4.87E-02	1.00E+00	-1.95E-03	1.00E+00
ENSBTAG00000001151	3.26E-01	8.68E-01	7.32E-01	3.74E-02	4.22E-01	3.17E-01	3.32E-01	4.97E-01	3.60E-03	1.00E+00
ENSBTAG00000007334	5.36E-01	1.78E-01	1.73E-01	7.26E-01	-5.16E-01	5.71E-02	-7.43E-01	4.13E-03	4.70E-03	1.00E+00
ENSBTAG00000007336	-3.72E-01	9.36E-01	-1.62E+00	6.18E-04	-1.54E+00	6.98E-04	-1.32E+00	4.17E-03	-4.41E-01	8.00E-01
ENSBTAG00000007338	1.03E-01	1.00E+00	-3.58E-01	4.69E-01	-3.18E-01	5.39E-01	3.23E-01	5.32E-01	-9.89E-02	1.00E+00
ENSBTAG00000000153	-3.47E-01	7.70E-01	-1.17E+00	6.31E-05	-2.17E-01	6.59E-01	1.90E-01	7.49E-01	9.67E-02	1.00E+00
ENSBTAG00000037461	-4.68E-01	4.61E-01	-2.45E-01	5.81E-01	5.53E-01	6.39E-02	4.01E-01	2.39E-01	-2.13E-01	9.89E-01
ENSBTAG00000019679	-5.95E-01	6.64E-01	-1.30E+00	8.62E-03	-1.39E+00	3.47E-03	-2.34E+00	4.98E-06	-1.02E+00	2.38E-01
ENSBTAG00000019678	-1.71E-01	1.00E+00	-1.03E+00	1.07E-03	-8.74E-01	6.26E-03	-8.86E-01	6.44E-03	-1.46E-01	1.00E+00
ENSBTAG00000019674	2.88E-01	9.83E-01	6.59E-01	9.23E-02	-3.04E-01	5.98E-01	-7.29E-01	7.92E-02	1.57E-01	1.00E+00
ENSBTAG00000014995	-2.81E-01	9.20E-01	-1.07E+00	1.07E-03	-8.98E-01	5.19E-03	-8.50E-01	9.73E-03	1.96E-03	1.00E+00
ENSBTAG00000014996	-5.05E-01	7.79E-01	-1.02E+00	2.69E-02	-2.78E-01	7.46E-01	3.84E-01	5.32E-01	3.48E-01	9.25E-01
ENSBTAG00000015001	2.37E-01	1.00E+00	1.04E+00	3.74E-01	2.65E+00	9.13E-05	1.73E+00	4.04E-02	-1.18E+00	8.32E-01
ENSBTAG00000015002	-8.41E-02	1.00E+00	1.25E-01	9.31E-01	7.25E-01	6.55E-03	7.34E-01	6.75E-03	-3.89E-01	6.01E-01
ENSBTAG00000015004	-2.49E-01	8.23E-01	-6.91E-01	2.47E-03	-2.83E-01	3.26E-01	8.68E-02	1.00E+00	-3.77E-01	4.95E-01

ENSBTAG00000014872	-1.37E-01	1.00E+00	-7.95E-01	1.63E-04	1.20E-01	8.40E-01	3.75E-01	1.30E-01	-2.58E-01	7.74E-01
ENSBTAG00000014878	3.26E-01	8.84E-01	7.25E-01	4.52E-02	4.32E-01	3.15E-01	2.63E-01	6.65E-01	6.00E-01	4.36E-01
ENSBTAG00000009521	-3.12E-01	6.90E-01	1.19E+00	7.99E-08	1.17E+00	1.10E-07	5.62E-01	2.40E-02	2.49E-02	1.00E+00
ENSBTAG00000031687	2.87E-01	6.86E-01	1.34E+00	4.63E-11	1.40E+00	4.25E-12	1.05E+00	4.17E-07	-6.32E-02	1.00E+00
ENSBTAG00000002398	4.65E-01	6.27E-01	3.41E-01	4.69E-01	-7.25E-01	4.74E-02	-4.84E-01	2.54E-01	-2.14E-01	1.00E+00
ENSBTAG00000038951	-1.87E-01	1.00E+00	1.32E-01	8.91E-01	-8.80E-01	5.94E-04	-1.07E+00	2.75E-05	-8.87E-02	1.00E+00
ENSBTAG00000008084	-9.89E-04	1.00E+00	1.99E-01	9.45E-01	1.96E-01	9.39E-01	4.95E-02	1.00E+00	-3.46E-01	9.94E-01
ENSBTAG00000033563	3.11E-02	1.00E+00	7.71E-01	6.26E-04	2.34E-01	4.67E-01	3.96E-02	1.00E+00	-1.20E-01	1.00E+00
ENSBTAG00000031686	-2.24E-01	1.00E+00	1.63E+00	2.92E-07	1.02E+00	2.07E-03	3.45E-01	4.72E-01	-4.08E-01	7.79E-01
ENSBTAG00000023464	-4.47E-01	4.39E-01	-1.11E-01	9.75E-01	-4.11E-01	1.88E-01	-3.61E-01	2.79E-01	-6.32E-01	1.86E-01
ENSBTAG00000042182	0.00E+00	1.00E+00	0.00E+00	1.00E+00	-5.13E-15	1.00E+00	0.00E+00	1.00E+00	0.00E+00	1.00E+00
ENSBTAG00000021742	9.81E-02	1.00E+00	-3.47E-01	6.53E-01	-1.97E-01	9.52E-01	-7.61E-01	1.48E-01	1.35E-01	1.00E+00
ENSBTAG00000042735	0.00E+00	1.00E+00	0.00E+00	1.00E+00	-5.13E-15	1.00E+00	0.00E+00	1.00E+00	0.00E+00	1.00E+00
ENSBTAG00000040108	-7.68E-02	1.00E+00	-1.86E-01	1.00E+00	6.87E-01	2.77E-01	1.67E-01	1.00E+00	3.29E-01	1.00E+00
ENSBTAG00000047219	4.08E-01	9.05E-01	9.53E-01	3.33E-02	1.06E+00	1.24E-02	6.83E-01	1.75E-01	-2.43E-01	1.00E+00
ENSBTAG00000040063	-1.58E-01	1.00E+00	-3.68E-01	4.43E-01	2.78E-02	1.00E+00	-2.84E-02	1.00E+00	-2.60E-01	9.83E-01
ENSBTAG00000038869	-1.83E-01	1.00E+00	4.12E-01	4.03E-01	4.28E-01	3.69E-01	-5.42E-02	1.00E+00	2.58E-02	1.00E+00
ENSBTAG00000039075	3.16E-03	1.00E+00	3.45E-01	2.22E-01	2.48E-01	4.43E-01	-2.38E-01	4.96E-01	-7.76E-02	1.00E+00
ENSBTAG00000031677	0.00E+00	1.00E+00	0.00E+00	1.00E+00	2.55E+00	2.55E-01	0.00E+00	1.00E+00	2.72E+00	6.01E-01
ENSBTAG00000004939	-4.36E-02	1.00E+00	5.85E-01	2.27E-02	6.21E-01	1.35E-02	1.57E-01	7.85E-01	-2.63E-01	8.00E-01
ENSBTAG00000000472	-2.30E-01	1.00E+00	7.31E-01	2.15E-02	7.65E-01	1.46E-02	4.91E-01	1.75E-01	-2.67E-01	9.14E-01
ENSBTAG00000038050	-6.35E-01	3.06E-01	-9.75E-01	4.86E-03	-9.94E-02	1.00E+00	-2.45E-01	6.73E-01	-7.40E-01	2.38E-01
ENSBTAG00000008902	-8.15E-01	3.79E-02	-9.78E-01	9.64E-04	-7.27E-01	1.49E-02	-4.35E-01	2.07E-01	-4.15E-01	6.07E-01
ENSBTAG00000046367	-1.17E+00	4.18E-01	1.03E+00	7.81E-02	8.42E-02	1.00E+00	1.82E-01	1.00E+00	-6.32E-01	8.33E-01
ENSBTAG00000040318	1.12E-02	1.00E+00	-2.70E+00	2.42E-01	-2.70E+00	1.99E-01	-2.70E+00	2.05E-01	-7.86E-01	1.00E+00
ENSBTAG00000038442	9.93E-01	3.11E-01	-1.03E+00	2.10E-01	-9.02E-01	2.52E-01	-1.05E+00	1.77E-01	5.96E-01	8.00E-01
ENSBTAG00000044426	0.00E+00	1.00E+00	0.00E+00	1.00E+00	1.78E+00	4.79E-01	0.00E+00	1.00E+00	0.00E+00	1.00E+00
ENSBTAG00000018225	2.44E-01	1.00E+00	6.82E-01	4.90E-02	1.79E+00	5.86E-09	1.88E+00	1.39E-09	1.87E-01	1.00E+00
ENSBTAG00000042990	0.00E+00	1.00E+00	0.00E+00	1.00E+00	-5.13E-15	1.00E+00	0.00E+00	1.00E+00	0.00E+00	1.00E+00
ENSBTAG00000007216	-4.70E-01	1.00E+00	-1.46E+00	9.41E-02	-1.97E+00	1.80E-02	-2.00E+00	1.78E-02	-8.32E-01	7.90E-01
ENSBTAG00000023472	7.84E-01	1.05E-02	8.03E-01	1.17E-03	4.58E-02	1.00E+00	1.72E-01	7.43E-01	-3.69E-01	6.15E-01
ENSBTAG00000000182	-9.95E-02	1.00E+00	-3.90E-01	4.43E-01	-7.34E-01	6.29E-02	-5.81E-01	1.77E-01	-6.33E-02	1.00E+00
ENSBTAG00000031648	1.00E-01	1.00E+00	1.81E+00	4.86E-03	8.30E-01	3.40E-01	4.11E-01	8.26E-01	-2.48E+00	1.74E-01
ENSBTAG00000027711	1.24E-01	1.00E+00	6.59E-02	1.00E+00	1.09E-01	9.41E-01	1.47E-01	8.00E-01	-1.49E-01	1.00E+00
ENSBTAG00000023562	1.26E+00	8.53E-03	9.18E-01	3.10E-02	6.93E-01	1.30E-01	4.20E-01	4.83E-01	1.27E-01	1.00E+00

ENSBTAG00000000621	-1.07E-01	1.00E+00	-1.78E+00	3.10E-11	-2.24E+00	7.35E-17	-1.84E+00	6.48E-12	2.07E-01	9.88E-01
ENSBTAG00000031641	-2.34E-02	1.00E+00	1.12E-01	9.45E-01	1.56E-01	7.73E-01	2.32E-01	5.25E-01	-3.77E-01	5.85E-01
ENSBTAG00000005363	-6.58E-01	1.00E+00	9.56E-02	1.00E+00	-1.33E+00	4.16E-01	-2.12E+00	1.77E-01	-1.15E+00	8.46E-01
ENSBTAG00000017678	1.78E+00	1.85E-01	4.03E+00	3.35E-10	3.34E+00	6.35E-07	2.57E+00	6.12E-04	1.14E+00	7.34E-01
ENSBTAG00000039593	-2.42E-01	9.46E-01	-1.20E+00	4.24E-05	-1.37E+00	1.85E-06	-8.14E-01	6.12E-03	-7.87E-01	8.33E-02
ENSBTAG00000006998	7.36E-01	6.41E-02	5.73E-01	6.54E-02	6.59E-01	2.65E-02	6.03E-01	5.21E-02	1.41E-01	1.00E+00
ENSBTAG00000046064	0.00E+00	1.00E+00	0.00E+00	1.00E+00	-5.13E-15	1.00E+00	0.00E+00	1.00E+00	0.00E+00	1.00E+00
ENSBTAG00000006999	9.72E-03	1.00E+00	-1.27E+00	2.08E-04	-2.26E+00	2.93E-11	-1.84E+00	6.31E-08	3.63E-01	8.00E-01
ENSBTAG00000044225	0.00E+00	1.00E+00	0.00E+00	1.00E+00	-5.13E-15	1.00E+00	0.00E+00	1.00E+00	0.00E+00	1.00E+00
ENSBTAG00000021145	1.41E-01	1.00E+00	5.65E-01	9.60E-02	4.53E-01	2.11E-01	-3.36E-02	1.00E+00	-1.44E-01	1.00E+00
ENSBTAG00000011212	-5.44E-02	1.00E+00	5.56E-01	1.45E-02	3.56E-01	1.64E-01	1.50E-01	7.45E-01	-3.76E-01	4.75E-01
ENSBTAG00000011215	-2.03E-01	8.97E-01	-4.89E-01	3.42E-02	-4.09E-01	9.01E-02	7.76E-02	1.00E+00	8.42E-02	1.00E+00
ENSBTAG00000013239	-6.47E-01	1.00E+00	-8.52E-02	1.00E+00	-3.57E-01	9.78E-01	-8.28E-01	5.57E-01	3.17E-01	1.00E+00
ENSBTAG00000046365	3.75E-01	1.00E+00	-4.23E-01	1.00E+00	-1.43E+00	4.79E-01	4.78E-01	9.70E-01	-7.70E-04	1.00E+00
ENSBTAG00000031538	1.99E+00	3.39E-01	6.00E-01	9.45E-01	-9.28E-01	8.06E-01	6.99E-01	8.52E-01	1.22E-02	1.00E+00
ENSBTAG00000016312	5.32E-01	2.44E-01	9.29E-01	3.53E-04	4.31E-01	1.58E-01	-3.33E-01	3.35E-01	2.33E-01	9.00E-01
ENSBTAG00000016313	8.74E-02	1.00E+00	1.36E-02	1.00E+00	-4.22E-01	5.29E-02	-4.81E-01	2.64E-02	3.35E-01	5.01E-01
ENSBTAG00000001055	4.43E-01	1.45E-01	9.43E-01	4.82E-07	6.31E-01	1.15E-03	4.37E-01	3.94E-02	1.23E-01	1.00E+00
ENSBTAG00000001775	-9.00E-02	1.00E+00	3.34E-01	4.01E-01	-5.60E-01	9.77E-02	-8.94E-01	5.22E-03	-2.97E-01	8.33E-01
ENSBTAG00000001776	-3.01E-01	5.90E-01	-5.67E-01	8.33E-03	-1.24E+00	6.01E-10	-1.33E+00	3.94E-11	-1.36E-01	1.00E+00
ENSBTAG00000001778	1.25E+00	2.46E-07	2.82E+00	1.72E-35	2.07E+00	1.31E-20	1.17E+00	2.95E-07	4.73E-02	1.00E+00
ENSBTAG00000046295	-1.56E-01	1.00E+00	-7.08E-01	3.87E-02	-4.60E-01	2.19E-01	-8.24E-01	1.34E-02	-1.59E-02	1.00E+00
ENSBTAG00000001780	-1.55E-01	1.00E+00	3.07E-01	2.35E-01	4.83E-02	1.00E+00	5.41E-02	1.00E+00	-1.68E-01	9.66E-01
ENSBTAG00000001782	-1.89E-01	1.00E+00	4.72E-01	1.74E-01	-2.45E-01	6.10E-01	-2.38E-01	6.40E-01	-4.14E-01	6.44E-01
ENSBTAG00000001783	-2.64E-01	1.00E+00	-1.23E+00	1.99E-03	-2.15E+00	2.44E-08	-2.49E+00	2.11E-10	3.96E-01	8.04E-01
ENSBTAG00000012590	-1.67E+00	7.78E-01	-1.60E+00	4.08E-01	-1.81E+00	2.95E-01	-1.94E-01	1.00E+00	-3.59E+00	3.09E-01
ENSBTAG00000031266	0.00E+00	1.00E+00	0.00E+00	1.00E+00	-5.13E-15	1.00E+00	0.00E+00	1.00E+00	0.00E+00	1.00E+00
ENSBTAG00000013958	-1.97E-01	1.00E+00	-1.43E+00	4.21E-06	-4.05E-01	3.07E-01	9.84E-02	1.00E+00	2.88E-01	8.75E-01
ENSBTAG00000014296	0.00E+00	1.00E+00	0.00E+00	1.00E+00	1.78E+00	4.79E-01	2.53E+00	2.71E-01	0.00E+00	1.00E+00
ENSBTAG00000045930	0.00E+00	1.00E+00	0.00E+00	1.00E+00	-5.13E-15	1.00E+00	0.00E+00	1.00E+00	0.00E+00	1.00E+00
ENSBTAG00000047307	0.00E+00	1.00E+00	1.99E+00	4.43E-01	-5.13E-15	1.00E+00	0.00E+00	1.00E+00	0.00E+00	1.00E+00
ENSBTAG00000047691	-2.71E+00	5.76E-01	-7.23E-01	9.45E-01	-9.24E-01	8.08E-01	-2.71E+00	2.05E-01	-2.71E+00	6.01E-01
ENSBTAG00000047158	3.51E-01	1.00E+00	6.60E-01	3.02E-01	1.04E+00	4.11E-02	1.70E+00	1.24E-04	-1.70E-01	1.00E+00
ENSBTAG00000005390	2.50E-01	9.04E-01	2.18E-01	6.28E-01	4.97E-02	1.00E+00	1.78E-02	1.00E+00	-1.44E-01	1.00E+00
ENSBTAG00000009785	4.83E-01	5.83E-01	1.60E+00	6.00E-07	9.27E-01	6.02E-03	7.81E-01	2.88E-02	4.80E-01	6.13E-01

ENSBTAG00000020428	0.00E+00	1.00E+00	0.00E+00	1.00E+00	-5.13E-15	1.00E+00	0.00E+00	1.00E+00	0.00E+00	1.00E+00
ENSBTAG00000020429	0.00E+00	1.00E+00	0.00E+00	1.00E+00	-5.13E-15	1.00E+00	0.00E+00	1.00E+00	0.00E+00	1.00E+00
ENSBTAG00000008573	4.06E+00	3.04E-32	3.17E+00	3.69E-22	3.08E+00	2.51E-21	2.47E+00	1.71E-14	2.13E-01	1.00E+00
ENSBTAG00000004017	4.83E-01	3.09E-01	-7.37E-01	6.07E-03	-1.06E+00	3.70E-05	-1.01E+00	1.08E-04	2.00E-01	9.69E-01
ENSBTAG00000021093	2.51E-01	8.23E-01	4.62E-01	7.49E-02	4.95E-01	5.18E-02	3.55E-01	2.19E-01	-9.42E-02	1.00E+00
ENSBTAG00000010467	5.14E-02	1.00E+00	9.63E-01	6.46E-06	1.05E+00	7.32E-07	6.12E-01	7.55E-03	6.09E-01	7.20E-02
ENSBTAG00000010480	5.72E-02	1.00E+00	2.81E-01	4.43E-01	3.58E-01	2.61E-01	4.29E-01	1.61E-01	2.35E-02	1.00E+00
ENSBTAG00000010483	6.56E-01	9.87E-01	9.60E-02	1.00E+00	9.14E-03	1.00E+00	-1.81E+00	1.70E-01	8.69E-01	8.00E-01
ENSBTAG00000010501	1.68E+00	7.75E-01	8.80E-01	8.52E-01	1.51E+00	4.28E-01	1.49E+00	4.55E-01	2.62E+00	2.76E-01
ENSBTAG00000015644	9.75E-02	1.00E+00	2.47E-02	1.00E+00	3.14E-01	5.74E-01	2.40E-01	7.61E-01	1.40E-01	1.00E+00
ENSBTAG00000047030	6.83E-01	9.78E-01	-1.62E+00	2.08E-01	2.33E-01	1.00E+00	-8.03E-01	5.92E-01	2.65E-01	1.00E+00
ENSBTAG00000015260	-7.80E-01	1.00E+00	-2.70E+00	2.42E-01	-9.16E-01	8.10E-01	3.26E-01	1.00E+00	-7.85E-01	1.00E+00
ENSBTAG00000046425	0.00E+00	1.00E+00	1.98E+00	4.43E-01	-5.13E-15	1.00E+00	0.00E+00	1.00E+00	0.00E+00	1.00E+00
ENSBTAG00000012509	1.41E-01	1.00E+00	1.96E+00	6.18E-16	-3.28E-01	3.30E-01	-6.37E-01	2.40E-02	7.62E-01	4.52E-02
ENSBTAG00000002579	3.30E-01	5.12E-01	2.05E+00	1.45E-25	2.37E+00	1.77E-33	1.93E+00	6.09E-23	-2.72E-01	7.13E-01
ENSBTAG00000009414	0.00E+00	1.00E+00	0.00E+00	1.00E+00	-5.13E-15	1.00E+00	0.00E+00	1.00E+00	0.00E+00	1.00E+00
ENSBTAG00000008502	0.00E+00	1.00E+00	0.00E+00	1.00E+00	-5.13E-15	1.00E+00	0.00E+00	1.00E+00	0.00E+00	1.00E+00
ENSBTAG00000005261	-6.83E-01	7.05E-01	-1.90E+00	1.49E-03	-1.60E+00	4.92E-03	-4.64E-01	5.50E-01	6.20E-01	7.24E-01
ENSBTAG00000018242	1.33E+00	6.99E-01	-3.21E+00	1.26E-01	-1.42E+00	4.80E-01	1.89E-01	1.00E+00	-1.28E+00	9.06E-01
ENSBTAG00000017618	2.00E-01	9.01E-01	-2.43E-01	4.24E-01	-1.53E-01	7.11E-01	-2.19E-01	4.93E-01	3.94E-01	4.15E-01
ENSBTAG00000046666	8.90E-01	1.00E+00	-7.16E-01	9.45E-01	-1.52E-01	1.00E+00	-9.40E-01	8.06E-01	8.98E-01	1.00E+00
ENSBTAG00000046626	0.00E+00	1.00E+00	1.98E+00	4.43E-01	1.78E+00	4.79E-01	2.53E+00	2.71E-01	0.00E+00	1.00E+00
ENSBTAG00000001400	-2.46E-01	8.23E-01	-5.20E-01	3.88E-02	-3.81E-01	1.64E-01	1.66E-01	7.24E-01	4.07E-01	4.57E-01
ENSBTAG00000018859	-5.93E-03	1.00E+00	7.04E-02	1.00E+00	5.31E-01	1.02E-01	9.05E-01	1.83E-03	2.63E-01	9.00E-01
ENSBTAG00000019150	-2.24E-01	9.24E-01	-8.14E-01	1.07E-03	-1.78E+00	1.06E-13	-1.88E+00	4.78E-15	6.11E-01	1.43E-01
ENSBTAG00000040067	-7.09E-01	6.53E-01	-2.16E+00	7.20E-04	-3.18E+00	2.90E-06	-1.61E+00	6.16E-03	4.71E-01	8.02E-01
ENSBTAG00000017333	-7.33E-01	1.26E-01	-3.36E+00	2.20E-18	-2.54E+00	5.36E-14	-1.01E+00	1.51E-03	-2.39E-01	9.68E-01
ENSBTAG00000010493	8.79E-01	7.26E-03	1.70E+00	3.49E-11	9.67E-01	2.50E-04	9.87E-01	2.23E-04	-1.08E-01	1.00E+00
ENSBTAG00000010502	-9.61E-01	2.85E-04	-1.04E+00	6.91E-06	-4.75E-01	6.52E-02	8.50E-02	1.00E+00	1.59E-01	1.00E+00
ENSBTAG00000010508	-3.21E-01	7.76E-01	-6.88E-01	1.34E-02	-1.22E+00	2.50E-06	-1.61E+00	5.30E-10	-6.44E-01	1.69E-01
ENSBTAG00000004751	-1.58E-01	1.00E+00	-1.10E+00	7.80E-02	-1.24E+00	3.50E-02	-8.29E-01	1.97E-01	7.11E-02	1.00E+00
ENSBTAG00000004755	-9.57E-02	1.00E+00	-5.34E-01	2.36E-02	-7.30E-02	1.00E+00	1.48E-01	7.60E-01	-2.53E-02	1.00E+00
ENSBTAG00000004757	-1.26E-01	1.00E+00	2.00E-01	8.15E-01	-5.58E-01	1.54E-01	-6.16E-01	1.12E-01	-2.21E-01	1.00E+00
ENSBTAG00000019461	1.69E-01	1.00E+00	4.49E-01	4.08E-01	-1.43E-01	1.00E+00	-9.69E-01	3.03E-02	3.04E-02	1.00E+00
ENSBTAG00000019462	9.38E-02	1.00E+00	-1.45E-01	8.27E-01	-2.87E-01	3.81E-01	-5.01E-01	6.80E-02	8.74E-02	1.00E+00

ENSBTAG00000019465	-1.48E-02	1.00E+00	5.34E-01	1.44E-01	8.79E-01	6.58E-03	8.61E-01	9.38E-03	1.01E-01	1.00E+00
ENSBTAG00000023453	-4.18E-01	4.48E-01	-1.59E+00	1.26E-10	-1.07E+00	1.25E-05	-1.10E+00	9.96E-06	3.57E-01	6.11E-01
ENSBTAG00000009077	-2.97E-01	7.16E-01	2.19E-01	5.11E-01	9.76E-01	7.92E-06	4.32E-01	9.45E-02	-1.50E-01	1.00E+00
ENSBTAG00000009078	-1.24E-01	1.00E+00	6.62E-01	2.31E-01	1.33E+00	3.51E-03	-2.47E-02	1.00E+00	-2.59E-01	1.00E+00
ENSBTAG00000009079	-1.96E-01	8.70E-01	-9.43E-01	2.08E-06	-3.99E-01	7.02E-02	-2.33E-01	3.99E-01	2.75E-01	6.47E-01
ENSBTAG00000017996	-2.55E-01	8.16E-01	-9.08E-01	3.20E-05	-4.69E-01	5.07E-02	-2.95E-01	3.03E-01	-4.21E-02	1.00E+00
ENSBTAG00000001350	1.61E-02	1.00E+00	-3.14E-01	3.90E-01	-2.12E-01	6.39E-01	-1.39E-01	8.89E-01	3.23E-01	7.72E-01
ENSBTAG00000046805	-1.91E+00	8.23E-01	8.83E-01	8.52E-01	-1.91E+00	4.28E-01	-1.91E+00	4.39E-01	-1.91E+00	8.00E-01
ENSBTAG00000037966	-1.40E-01	1.00E+00	-2.14E-01	4.76E-01	-6.59E-01	2.21E-03	-6.53E-01	2.94E-03	-8.28E-02	1.00E+00
ENSBTAG00000047815	-1.03E-01	1.00E+00	-4.28E-01	1.33E-01	-1.63E+00	7.51E-12	-2.73E+00	5.45E-29	2.11E-01	9.23E-01
ENSBTAG00000003272	7.12E-02	1.00E+00	-9.69E-01	2.63E-01	-2.17E+00	4.20E-03	-4.28E+00	1.71E-06	-8.46E-01	7.70E-01
ENSBTAG00000003871	-2.28E-01	8.58E-01	-1.17E+00	1.18E-07	-2.34E+00	5.92E-26	-2.95E+00	1.28E-38	1.57E-01	1.00E+00
ENSBTAG00000047196	-2.48E-01	9.80E-01	-1.26E+00	3.81E-05	-1.86E+00	7.72E-10	-6.32E-01	6.13E-02	-2.38E-01	9.68E-01
ENSBTAG00000018240	-1.18E+00	2.29E-01	-8.95E-01	1.74E-01	-2.40E+00	1.78E-04	-1.36E+00	2.52E-02	-5.57E-01	8.00E-01
ENSBTAG00000003166	-3.43E-01	7.57E-01	-1.19E+00	1.33E-05	-1.41E+00	1.80E-07	-1.51E+00	2.97E-08	1.38E-01	1.00E+00
ENSBTAG00000048174	1.92E+00	8.23E-01	0.00E+00	1.00E+00	-5.13E-15	1.00E+00	0.00E+00	1.00E+00	0.00E+00	1.00E+00
ENSBTAG00000045494	-2.71E+00	5.76E-01	8.41E-02	1.00E+00	3.45E-01	1.00E+00	-1.76E-01	1.00E+00	-2.71E+00	6.01E-01
ENSBTAG00000047918	-1.91E+00	8.23E-01	-1.91E+00	4.69E-01	-1.91E+00	4.28E-01	-1.48E-01	1.00E+00	1.17E-02	1.00E+00
ENSBTAG00000045632	-8.35E-01	2.91E-01	-5.68E-01	2.90E-01	-1.11E-01	1.00E+00	-5.07E-01	3.73E-01	-1.34E+00	3.84E-02
ENSBTAG00000047370	0.00E+00	1.00E+00	0.00E+00	1.00E+00	-5.13E-15	1.00E+00	0.00E+00	1.00E+00	0.00E+00	1.00E+00
ENSBTAG00000048000	0.00E+00	1.00E+00	0.00E+00	1.00E+00	-5.13E-15	1.00E+00	0.00E+00	1.00E+00	0.00E+00	1.00E+00
ENSBTAG00000003168	4.01E-03	1.00E+00	3.09E-01	1.90E-01	4.85E-01	1.92E-02	4.04E-01	6.84E-02	1.63E-01	9.41E-01
ENSBTAG00000039635	-1.31E-01	1.00E+00	5.22E-01	4.66E-02	5.95E-01	1.91E-02	5.26E-01	4.90E-02	4.82E-02	1.00E+00
ENSBTAG00000020457	7.38E-01	5.46E-02	6.23E-01	3.50E-02	-3.65E-02	1.00E+00	-3.39E-01	3.69E-01	-4.27E-01	5.96E-01
ENSBTAG00000023452	-7.92E-02	1.00E+00	-6.59E-01	4.16E-02	-1.14E+00	1.39E-04	-4.38E-01	2.23E-01	1.26E-01	1.00E+00
ENSBTAG00000038711	-7.93E-01	1.00E+00	-2.71E+00	2.42E-01	-1.54E-01	1.00E+00	-9.46E-01	8.04E-01	-7.92E-01	1.00E+00
ENSBTAG00000016034	4.14E-02	1.00E+00	1.70E+00	1.75E-06	2.21E+00	3.06E-10	1.47E+00	4.85E-05	8.18E-02	1.00E+00
ENSBTAG00000016037	-1.10E-01	1.00E+00	-4.67E-01	5.79E-02	-1.02E+00	3.50E-06	-1.39E+00	2.11E-10	4.88E-02	1.00E+00
ENSBTAG00000016041	7.51E-01	1.00E+00	2.74E+00	5.02E-04	5.64E-01	8.41E-01	-5.63E-01	9.24E-01	1.49E+00	5.15E-01
ENSBTAG00000009449	-1.79E-01	1.00E+00	-5.14E-01	1.19E-01	-8.12E-01	6.19E-03	-1.46E-01	9.01E-01	1.07E-01	1.00E+00
ENSBTAG00000047423	0.00E+00	1.00E+00	0.00E+00	1.00E+00	-5.13E-15	1.00E+00	0.00E+00	1.00E+00	0.00E+00	1.00E+00
ENSBTAG00000005615	6.89E-01	1.43E-01	9.37E-01	2.02E-03	-4.76E-01	1.80E-01	-1.06E+00	4.88E-04	7.65E-02	1.00E+00
ENSBTAG00000038321	2.20E-01	1.00E+00	-8.60E-01	1.00E-01	-1.23E-01	1.00E+00	-3.06E-01	7.15E-01	-3.51E-01	9.61E-01
ENSBTAG00000031004	0.00E+00	1.00E+00	2.79E+00	2.10E-01	1.78E+00	4.79E-01	0.00E+00	1.00E+00	0.00E+00	1.00E+00
ENSBTAG00000031001	-7.19E-02	1.00E+00	-1.62E+00	3.59E-10	-1.77E+00	5.52E-12	-1.53E+00	3.45E-09	6.61E-01	1.36E-01

ENSBTAG00000039015	1.02E-01	1.00E+00	-7.22E-01	1.65E-01	-7.73E-01	1.14E-01	-3.36E-01	6.31E-01	-2.32E-02	1.00E+00
ENSBTAG00000013283	-4.43E-01	1.00E+00	-1.72E+00	2.62E-02	-1.37E+00	6.21E-02	-8.07E-01	3.29E-01	1.57E-01	1.00E+00
ENSBTAG00000019787	-5.00E-01	7.67E-01	-1.58E+00	3.50E-04	-1.07E+00	1.43E-02	-7.11E-01	1.39E-01	-7.62E-01	4.30E-01
ENSBTAG00000019785	1.93E-01	9.87E-01	2.83E-01	3.74E-01	1.93E-01	6.24E-01	6.39E-01	1.03E-02	3.29E-01	6.48E-01
ENSBTAG00000000332	-1.22E-01	1.00E+00	2.97E-01	4.21E-01	3.59E-01	2.78E-01	2.12E-01	6.47E-01	4.08E-01	5.88E-01
ENSBTAG00000020756	5.18E-01	1.05E-01	1.95E+00	5.11E-22	1.96E+00	1.80E-22	1.32E+00	1.07E-10	1.62E-01	9.90E-01
ENSBTAG00000020754	2.63E-01	8.63E-01	1.73E-02	1.00E+00	3.32E-01	3.33E-01	3.88E-01	2.37E-01	4.54E-01	4.77E-01
ENSBTAG00000005761	4.99E-01	4.63E-01	2.28E+00	7.05E-15	1.09E+00	2.61E-04	6.85E-01	3.75E-02	4.47E-01	6.01E-01
ENSBTAG00000047826	0.00E+00	1.00E+00	0.00E+00	1.00E+00	-5.13E-15	1.00E+00	0.00E+00	1.00E+00	0.00E+00	1.00E+00
ENSBTAG00000008556	5.08E-01	1.63E-01	3.74E-01	1.62E-01	3.50E-01	1.99E-01	-3.49E-01	2.22E-01	3.12E-01	6.67E-01
ENSBTAG00000021789	-4.43E-01	3.79E-01	-7.29E-02	1.00E+00	2.94E-01	3.69E-01	-1.47E-01	8.25E-01	2.56E-02	1.00E+00
ENSBTAG00000011723	6.74E-03	1.00E+00	6.42E-01	5.59E-01	-3.12E-01	9.68E-01	-9.56E-01	3.21E-01	-9.40E-01	7.43E-01
ENSBTAG00000018635	-5.25E-01	1.00E+00	-8.17E-01	6.73E-01	-6.99E-01	7.25E-01	-4.30E-01	9.59E-01	-4.12E+00	1.39E-01
ENSBTAG00000018633	4.25E-02	1.00E+00	3.75E-01	2.93E-01	4.70E-01	1.50E-01	5.59E-01	7.83E-02	1.48E-01	1.00E+00
ENSBTAG00000048100	0.00E+00	1.00E+00	0.00E+00	1.00E+00	-5.13E-15	1.00E+00	0.00E+00	1.00E+00	0.00E+00	1.00E+00
ENSBTAG00000018912	5.73E-02	1.00E+00	-4.14E-01	7.62E-02	-1.44E-02	1.00E+00	-1.32E-01	7.95E-01	1.30E-01	1.00E+00
ENSBTAG00000001882	9.16E-02	1.00E+00	-7.58E-01	1.27E-01	-2.67E-01	7.68E-01	-9.01E-01	5.81E-02	-1.51E-01	1.00E+00
ENSBTAG00000011963	2.99E-01	7.78E-01	9.31E-01	1.49E-04	1.44E+00	1.36E-09	7.22E-01	5.06E-03	1.18E-01	1.00E+00
ENSBTAG00000019273	-1.91E+00	8.23E-01	8.80E-01	8.52E-01	-1.91E+00	4.28E-01	6.26E-01	9.94E-01	7.04E-03	1.00E+00
ENSBTAG00000020251	-7.87E-01	1.00E+00	-7.23E-01	9.45E-01	-2.71E+00	1.99E-01	-2.71E+00	2.05E-01	-2.71E+00	6.01E-01
ENSBTAG00000006859	2.19E-01	1.00E+00	4.33E-01	2.63E-01	6.66E-01	4.62E-02	4.12E-01	3.06E-01	3.82E-01	7.69E-01
ENSBTAG00000043005	0.00E+00	1.00E+00	0.00E+00	1.00E+00	-5.13E-15	1.00E+00	0.00E+00	1.00E+00	0.00E+00	1.00E+00
ENSBTAG00000018652	-6.90E-01	8.23E-01	-1.19E+00	9.28E-02	-9.39E-01	2.16E-01	-1.76E+00	8.89E-03	-6.37E-01	8.15E-01
ENSBTAG00000002976	-1.48E-01	1.00E+00	-9.12E-01	2.88E-01	-1.02E+00	2.01E-01	-1.23E+00	1.18E-01	-8.12E-01	7.77E-01
ENSBTAG00000016777	0.00E+00	1.00E+00	1.99E+00	4.43E-01	1.78E+00	4.79E-01	0.00E+00	1.00E+00	1.91E+00	8.00E-01
ENSBTAG00000003886	0.00E+00	1.00E+00	1.99E+00	4.43E-01	1.78E+00	4.79E-01	2.53E+00	2.71E-01	0.00E+00	1.00E+00
ENSBTAG00000018882	0.00E+00	1.00E+00	3.30E+00	1.02E-01	2.55E+00	2.55E-01	3.03E+00	1.50E-01	0.00E+00	1.00E+00
ENSBTAG00000023434	5.88E-03	1.00E+00	1.40E+00	5.17E-01	6.51E-01	9.78E-01	1.49E+00	4.57E-01	-1.91E+00	8.00E-01
ENSBTAG00000018077	1.08E+00	1.81E-02	1.72E+00	3.61E-07	7.10E-01	7.40E-02	4.35E-01	3.84E-01	-1.45E-01	1.00E+00
ENSBTAG00000004377	7.74E-01	2.72E-01	1.79E+00	2.79E-06	8.08E-01	5.78E-02	7.37E-01	9.93E-02	-1.90E-01	1.00E+00
ENSBTAG00000004379	1.97E-01	1.00E+00	2.09E-01	9.19E-01	1.89E-01	9.59E-01	-4.17E-02	1.00E+00	1.45E-02	1.00E+00
ENSBTAG00000030939	-4.39E-01	5.76E-01	-1.92E+00	9.37E-10	-2.01E+00	5.74E-11	-1.74E+00	1.09E-08	-1.66E-01	1.00E+00
ENSBTAG00000016268	-2.29E-01	8.53E-01	-1.06E+00	2.31E-06	-1.14E+00	2.66E-07	-9.86E-01	1.30E-05	1.00E-01	1.00E+00
ENSBTAG00000001260	0.00E+00	1.00E+00	0.00E+00	1.00E+00	-5.13E-15	1.00E+00	1.76E+00	4.97E-01	0.00E+00	1.00E+00
ENSBTAG00000001262	2.40E-01	1.00E+00	1.44E+00	3.56E-05	1.10E+00	2.09E-03	1.39E+00	7.56E-05	6.56E-01	4.34E-01

ENSBTAG00000030933	-9.35E-04	1.00E+00	4.99E-01	6.95E-02	3.86E-01	1.96E-01	-2.18E-01	5.90E-01	1.08E-01	1.00E+00
ENSBTAG00000012796	-1.66E-01	1.00E+00	3.58E-01	3.48E-01	-1.80E-01	8.01E-01	-5.53E-01	1.04E-01	-1.23E-02	1.00E+00
ENSBTAG00000015984	-4.10E-01	8.62E-01	-1.00E-01	1.00E+00	-2.65E-01	7.54E-01	-2.07E-01	8.91E-01	6.33E-01	4.98E-01
ENSBTAG00000013125	2.06E+00	2.07E-02	3.55E+00	2.47E-07	3.08E+00	6.26E-06	4.20E+00	2.25E-09	2.56E-01	1.00E+00
ENSBTAG00000023439	0.00E+00	1.00E+00	0.00E+00	1.00E+00	-5.13E-15	1.00E+00	0.00E+00	1.00E+00	0.00E+00	1.00E+00
ENSBTAG00000013766	3.23E+00	3.75E-01	5.40E+00	1.04E-04	3.97E+00	2.27E-02	4.33E+00	9.27E-03	3.24E+00	4.34E-01
ENSBTAG00000030922	8.10E-01	1.00E+00	4.43E+00	2.91E-06	1.82E+00	2.80E-01	1.12E+00	6.72E-01	2.01E-02	1.00E+00
ENSBTAG00000011815	9.37E-02	1.00E+00	1.27E+00	5.67E-06	1.08E+00	1.25E-04	7.43E-01	1.42E-02	3.86E-02	1.00E+00
ENSBTAG00000004588	9.46E-01	3.24E-01	-1.51E-01	1.00E+00	-3.06E-01	8.51E-01	-8.58E-02	1.00E+00	-4.43E-02	1.00E+00
ENSBTAG00000037888	7.97E-01	1.00E+00	8.83E-01	8.52E-01	-1.24E-01	1.00E+00	1.50E+00	4.51E-01	-1.91E+00	8.00E-01
ENSBTAG00000012522	2.08E-01	9.96E-01	6.26E-01	2.04E-02	7.03E-01	7.02E-03	-1.67E-01	7.86E-01	4.00E-01	5.79E-01
ENSBTAG00000012915	3.90E-01	1.00E+00	7.30E-01	3.61E-01	-8.48E-01	3.56E-01	-2.62E+00	3.79E-03	2.26E-01	1.00E+00
ENSBTAG00000026263	-1.15E-02	1.00E+00	7.80E-01	5.59E-04	1.31E-01	8.33E-01	-6.10E-01	1.28E-02	-7.24E-02	1.00E+00
ENSBTAG00000027431	2.09E-01	1.00E+00	1.80E+00	4.20E-09	1.41E+00	4.84E-06	2.18E-02	1.00E+00	1.30E-01	1.00E+00
ENSBTAG00000007714	3.13E-01	1.00E+00	3.84E+00	4.80E-20	3.24E+00	2.87E-15	1.29E+00	2.46E-03	4.70E-01	8.00E-01
ENSBTAG00000040209	1.02E+00	2.21E-02	3.72E+00	2.77E-28	3.56E+00	1.47E-26	2.06E+00	4.05E-10	4.25E-01	7.68E-01
ENSBTAG00000006805	-1.09E-01	1.00E+00	1.23E+00	1.57E-05	6.55E-01	3.71E-02	2.20E-01	6.96E-01	2.63E-01	9.04E-01
ENSBTAG00000011036	3.67E-02	1.00E+00	5.08E-01	8.53E-01	2.32E-01	1.00E+00	2.30E-01	1.00E+00	-1.41E+00	7.77E-01
ENSBTAG00000044517	0.00E+00	1.00E+00	0.00E+00	1.00E+00	-5.13E-15	1.00E+00	0.00E+00	1.00E+00	0.00E+00	1.00E+00
ENSBTAG00000048075	1.36E+00	5.40E-01	2.49E+00	2.08E-03	2.91E+00	1.21E-04	6.01E-01	7.71E-01	6.69E-01	1.00E+00
ENSBTAG00000015313	-2.51E-01	1.00E+00	-6.18E-01	1.18E-01	-5.88E-01	1.39E-01	-6.44E-01	1.04E-01	4.24E-01	7.58E-01
ENSBTAG00000037983	-1.75E-01	1.00E+00	1.99E+00	4.49E-03	1.30E+00	1.08E-01	-1.91E-01	1.00E+00	1.03E+00	6.46E-01
ENSBTAG00000009493	4.12E+00	6.78E-79	4.48E+00	7.45E-91	3.04E+00	9.68E-48	2.41E+00	1.82E-31	1.44E+00	1.43E-10
ENSBTAG00000009494	-1.76E-01	1.00E+00	-1.48E+00	9.74E-06	-2.11E+00	2.39E-10	-1.97E+00	3.91E-09	-2.14E-01	1.00E+00
ENSBTAG00000009495	3.39E-03	1.00E+00	-2.68E-01	4.69E-01	-8.46E-01	1.36E-03	-6.75E-01	1.54E-02	4.65E-01	4.41E-01
ENSBTAG00000015318	-3.61E-02	1.00E+00	1.45E-01	9.16E-01	-9.63E-02	1.00E+00	7.21E-02	1.00E+00	2.80E-01	8.58E-01
ENSBTAG00000047345	7.08E-02	1.00E+00	1.57E-01	7.59E-01	-2.74E-01	3.91E-01	-4.66E-01	8.11E-02	-1.10E-02	1.00E+00
ENSBTAG00000001781	-2.65E-01	9.57E-01	4.23E-01	2.72E-01	1.31E+00	1.45E-05	1.53E+00	4.19E-07	-4.74E-01	5.98E-01
ENSBTAG00000010123	-3.02E-02	1.00E+00	-2.69E-02	1.00E+00	-4.77E-01	5.42E-02	-4.70E-01	6.53E-02	4.70E-01	3.09E-01
ENSBTAG00000020556	-2.84E-01	9.74E-01	-8.77E-01	1.45E-02	-1.68E+00	5.88E-07	-1.81E+00	9.98E-08	-1.59E-02	1.00E+00
ENSBTAG00000020558	-1.62E-01	1.00E+00	-7.25E-01	3.61E-02	-1.44E+00	5.31E-06	-1.55E+00	1.31E-06	6.12E-02	1.00E+00
ENSBTAG00000020560	5.63E-02	1.00E+00	8.15E-02	1.00E+00	3.17E-01	2.58E-01	7.07E-01	2.42E-03	2.83E-01	7.59E-01
ENSBTAG00000038428	3.84E+00	9.43E-08	4.62E+00	1.80E-11	2.85E+00	1.28E-05	2.43E+00	2.54E-04	1.76E-01	1.00E+00
ENSBTAG00000013697	-1.67E-01	1.00E+00	2.92E-01	3.64E-01	1.04E-01	9.68E-01	-4.63E-01	9.40E-02	-7.09E-02	1.00E+00
ENSBTAG00000013702	-2.73E-01	1.00E+00	5.83E-03	1.00E+00	-4.04E-01	6.31E-01	-3.29E-01	7.67E-01	8.26E-02	1.00E+00

ENSBTAG00000010668	-4.83E-01	4.84E-01	-1.64E-01	8.56E-01	6.85E-01	2.23E-02	1.33E-01	9.44E-01	-4.03E-02	1.00E+00
ENSBTAG00000018834	-6.08E-02	1.00E+00	8.27E-01	9.00E-04	6.44E-01	1.27E-02	6.30E-01	1.74E-02	2.54E-02	1.00E+00
ENSBTAG00000020074	2.31E-01	1.00E+00	5.63E-01	2.57E-01	7.07E-01	1.08E-01	-2.89E-03	1.00E+00	1.45E-01	1.00E+00
ENSBTAG00000007065	8.53E-02	1.00E+00	-1.67E-02	1.00E+00	-3.55E-01	2.38E-01	-4.25E-01	1.44E-01	3.90E-02	1.00E+00
ENSBTAG00000007070	-4.11E-01	8.23E-01	9.13E-01	1.55E-02	2.55E-01	7.27E-01	5.45E-01	2.23E-01	5.58E-01	5.99E-01
ENSBTAG00000030882	-8.20E-01	2.11E-03	-1.98E+00	1.58E-19	-3.58E+00	9.04E-55	-4.36E+00	5.55E-75	-2.77E-01	7.71E-01
ENSBTAG00000015836	-9.95E-01	8.48E-02	-3.26E+00	2.61E-15	-3.38E-01	5.82E-01	-8.30E-01	5.80E-02	-2.35E-01	1.00E+00
ENSBTAG00000002682	7.70E-02	1.00E+00	-5.44E-01	4.79E-02	3.65E-01	2.11E-01	7.09E-01	4.31E-03	1.47E-02	1.00E+00
ENSBTAG00000042445	0.00E+00	1.00E+00	0.00E+00	1.00E+00	-5.13E-15	1.00E+00	0.00E+00	1.00E+00	0.00E+00	1.00E+00
ENSBTAG00000013921	-2.75E-01	1.00E+00	1.16E+00	4.04E-01	-6.15E-01	8.05E-01	9.78E-01	5.11E-01	5.61E-01	1.00E+00
ENSBTAG00000002070	-6.57E-01	8.28E-01	-6.90E-01	4.45E-01	8.91E-02	1.00E+00	-5.85E-01	5.22E-01	-4.84E-01	9.76E-01
ENSBTAG00000002072	-1.73E-01	1.00E+00	-4.27E-01	9.89E-02	3.23E-02	1.00E+00	4.05E-01	1.26E-01	8.03E-02	1.00E+00
ENSBTAG00000020689	1.25E-01	1.00E+00	1.74E+00	1.33E-10	1.42E+00	1.47E-07	1.04E+00	2.18E-04	-3.03E-01	8.00E-01
ENSBTAG00000023601	3.53E-01	6.23E-01	1.77E+00	5.38E-14	1.86E+00	1.96E-15	1.51E+00	2.11E-10	-3.52E-01	6.65E-01
ENSBTAG00000017355	-4.86E-02	1.00E+00	-8.88E-02	9.96E-01	1.79E-01	6.23E-01	2.87E-01	3.15E-01	-1.52E-02	1.00E+00
ENSBTAG00000008182	2.22E+00	2.20E-01	-3.10E-01	1.00E+00	2.08E+00	7.46E-02	5.58E+00	1.96E-06	5.41E-01	1.00E+00
ENSBTAG00000008185	-5.88E-01	1.73E-01	-1.45E+00	5.30E-08	-2.49E+00	8.34E-20	-2.84E+00	3.77E-24	-5.40E-01	3.19E-01
ENSBTAG00000014187	1.33E-01	1.00E+00	-4.61E-01	8.43E-01	-1.11E+00	2.94E-01	4.38E-01	7.90E-01	2.68E-03	1.00E+00
ENSBTAG00000019604	1.37E-01	1.00E+00	-5.50E-01	2.08E-02	2.06E-01	5.38E-01	3.57E-01	1.89E-01	-2.59E-01	8.00E-01
ENSBTAG00000019605	-4.63E-01	2.87E-01	-9.10E-01	1.23E-04	-5.88E-01	1.78E-02	1.09E-01	9.41E-01	-9.16E-02	1.00E+00
ENSBTAG00000018398	4.27E-01	8.23E-01	1.63E-01	1.00E+00	-4.84E-01	4.28E-01	-3.17E-01	6.96E-01	-2.58E-01	1.00E+00
ENSBTAG00000015764	1.12E-01	1.00E+00	-7.26E-01	2.21E-01	-7.80E-02	1.00E+00	3.15E-01	7.75E-01	1.03E-01	1.00E+00
ENSBTAG00000029933	0.00E+00	1.00E+00	0.00E+00	1.00E+00	-5.13E-15	1.00E+00	0.00E+00	1.00E+00	0.00E+00	1.00E+00
ENSBTAG00000005527	-5.45E-01	1.00E+00	-1.36E+00	3.91E-01	-4.18E-01	9.68E-01	-2.39E+00	1.01E-01	-5.60E-01	1.00E+00
ENSBTAG00000012177	-7.20E-02	1.00E+00	2.34E-01	5.04E-01	6.04E-01	1.64E-02	6.43E-01	1.12E-02	-3.59E-01	6.01E-01
ENSBTAG00000012176	-5.53E-01	5.49E-01	-1.64E+00	4.87E-06	-1.88E-01	8.75E-01	9.31E-01	1.28E-02	4.30E-01	7.74E-01
ENSBTAG00000045708	-2.74E-01	8.23E-01	1.02E-01	9.74E-01	-3.27E-02	1.00E+00	-1.95E-01	6.38E-01	3.71E-02	1.00E+00
ENSBTAG00000013346	-3.33E-02	1.00E+00	-7.24E-01	2.63E-02	-4.34E-01	2.49E-01	-3.98E-01	3.20E-01	4.01E-01	7.00E-01
ENSBTAG00000013347	-3.86E-01	9.28E-01	-5.01E-01	3.92E-01	-1.01E+00	2.90E-02	-6.30E-01	2.38E-01	7.47E-01	4.43E-01
ENSBTAG00000021417	-1.93E-01	9.85E-01	-3.66E-01	2.01E-01	3.47E-01	2.18E-01	4.36E-01	1.02E-01	2.55E-01	8.00E-01
ENSBTAG00000012097	7.71E-03	1.00E+00	1.04E+00	4.42E-01	-1.02E+00	6.06E-01	-5.54E-01	9.25E-01	-3.56E-01	1.00E+00
ENSBTAG00000012101	2.07E-01	8.72E-01	1.62E+00	8.07E-16	1.29E+00	1.48E-10	8.04E-01	1.37E-04	2.94E-01	6.49E-01
ENSBTAG00000012104	2.83E-01	9.98E-01	1.51E+00	1.48E-05	1.29E+00	2.53E-04	1.48E+00	3.01E-05	8.77E-01	1.49E-01
ENSBTAG00000047505	0.00E+00	1.00E+00	0.00E+00	1.00E+00	-5.13E-15	1.00E+00	0.00E+00	1.00E+00	0.00E+00	1.00E+00
ENSBTAG00000013157	8.01E-01	5.50E-01	4.26E-01	6.35E-01	3.17E-02	1.00E+00	2.13E-01	9.94E-01	-1.93E-02	1.00E+00

ENSBTAG00000005819	-9.69E-02	1.00E+00	-5.78E-01	1.95E-02	-1.19E-01	8.94E-01	-4.43E-01	9.78E-02	2.00E-01	9.15E-01
ENSBTAG00000030108	1.91E+00	8.23E-01	0.00E+00	1.00E+00	-5.13E-15	1.00E+00	0.00E+00	1.00E+00	1.93E+00	8.00E-01
ENSBTAG00000002635	9.44E-01	8.23E-01	1.70E+00	5.38E-02	2.31E+00	1.83E-03	1.56E+00	8.49E-02	2.41E-01	1.00E+00
ENSBTAG00000023367	-2.66E-01	8.23E-01	-8.80E-01	5.71E-04	-1.91E+00	9.87E-15	-2.18E+00	2.04E-18	-4.94E-02	1.00E+00
ENSBTAG00000000027	-2.70E+00	5.76E-01	-2.70E+00	2.42E-01	-2.70E+00	1.99E-01	-1.70E-01	1.00E+00	-2.70E+00	6.01E-01
ENSBTAG00000018948	2.80E-01	1.00E+00	-2.53E-02	1.00E+00	-5.43E-01	3.74E-01	-3.92E-01	5.85E-01	4.94E-01	8.00E-01
ENSBTAG00000008014	3.42E-01	3.79E-01	4.15E-01	4.60E-02	6.37E-01	9.00E-04	7.34E-01	1.25E-04	-1.48E-01	9.85E-01
ENSBTAG00000009337	1.49E-01	1.00E+00	-1.33E+00	1.19E-01	-1.70E+00	3.43E-02	-3.00E+00	5.83E-04	6.39E-01	8.27E-01
ENSBTAG00000046451	5.62E-02	1.00E+00	4.20E-02	1.00E+00	1.10E-01	1.00E+00	-5.94E-02	1.00E+00	-5.80E-01	3.81E-01
ENSBTAG00000018181	-1.26E+00	7.89E-05	-3.47E+00	7.90E-27	-4.19E+00	7.94E-36	-3.30E+00	1.83E-26	5.78E-01	3.05E-01
ENSBTAG00000048021	-2.92E+00	9.10E-02	-2.05E+00	7.87E-02	-4.84E+00	7.57E-04	-3.08E+00	1.07E-02	-6.96E-01	9.45E-01
ENSBTAG00000014583	-3.09E-01	7.75E-01	-7.66E-01	3.36E-03	-8.95E-01	4.61E-04	-7.80E-01	3.24E-03	-1.24E-01	1.00E+00
ENSBTAG00000014585	2.05E-01	1.00E+00	-1.91E+00	1.14E-02	7.98E-02	1.00E+00	2.86E-01	8.81E-01	4.28E-01	9.63E-01
ENSBTAG00000017762	0.00E+00	1.00E+00	0.00E+00	1.00E+00	-5.13E-15	1.00E+00	0.00E+00	1.00E+00	0.00E+00	1.00E+00
ENSBTAG00000030705	-9.95E-01	8.51E-02	-3.54E+00	1.71E-12	-2.42E+00	1.38E-08	-1.05E+00	1.10E-02	-9.32E-02	1.00E+00
ENSBTAG00000015599	-6.27E-01	2.66E-02	-1.24E+00	2.19E-09	-2.02E+00	1.66E-22	-1.47E+00	1.54E-12	-3.24E-01	6.01E-01
ENSBTAG00000046388	0.00E+00	1.00E+00	0.00E+00	1.00E+00	-5.13E-15	1.00E+00	0.00E+00	1.00E+00	0.00E+00	1.00E+00
ENSBTAG00000005830	-4.21E-02	1.00E+00	2.09E-03	1.00E+00	3.39E-01	1.94E-01	1.31E-01	8.21E-01	1.71E-01	9.73E-01
ENSBTAG00000013985	1.99E-01	9.66E-01	3.48E-01	2.29E-01	6.64E-01	5.65E-03	7.92E-01	8.50E-04	1.86E-01	9.74E-01
ENSBTAG00000013577	1.48E+00	2.81E-02	3.23E+00	2.55E-10	2.27E+00	6.41E-06	2.87E+00	1.83E-08	-7.26E-02	1.00E+00
ENSBTAG00000010584	9.18E-02	1.00E+00	-4.32E-02	1.00E+00	-7.11E-02	1.00E+00	7.13E-02	1.00E+00	1.17E-01	1.00E+00
ENSBTAG00000015543	1.08E-01	1.00E+00	2.14E+00	5.25E-08	1.89E+00	1.46E-06	9.59E-01	2.58E-02	4.59E-01	8.00E-01
ENSBTAG00000015576	2.82E-01	1.00E+00	6.08E+00	2.37E-17	3.63E+00	1.70E-06	2.87E+00	4.50E-04	3.57E-02	1.00E+00
ENSBTAG00000015579	-3.21E-02	1.00E+00	-4.50E-01	2.87E-01	-9.52E-01	5.93E-03	-8.34E-01	2.04E-02	-3.82E-01	8.00E-01
ENSBTAG00000009286	-1.04E-01	1.00E+00	6.21E-01	8.06E-03	4.52E-01	7.20E-02	1.60E-01	7.30E-01	7.83E-01	1.10E-02
ENSBTAG00000002676	1.05E-01	1.00E+00	1.22E+00	5.14E-09	1.30E+00	2.94E-10	1.20E+00	1.01E-08	-2.22E-01	8.17E-01
ENSBTAG00000014117	3.69E-02	1.00E+00	2.51E-01	4.46E-01	6.38E-01	8.37E-03	4.75E-01	7.26E-02	-4.85E-02	1.00E+00
ENSBTAG00000020872	5.07E-01	8.23E-01	1.54E-01	1.00E+00	1.20E+00	1.15E-02	1.06E+00	3.41E-02	4.24E-01	8.91E-01
ENSBTAG00000037735	9.23E-01	2.17E-01	5.89E-01	3.11E-01	7.58E-01	1.32E-01	8.89E-01	6.73E-02	7.09E-01	5.53E-01
ENSBTAG00000006848	6.05E-02	1.00E+00	8.64E-01	5.92E-04	1.15E+00	1.87E-06	6.97E-01	8.12E-03	5.49E-03	1.00E+00
ENSBTAG00000006862	1.04E-01	1.00E+00	-5.94E-01	2.19E-01	-6.65E-01	1.45E-01	-4.93E-01	3.38E-01	-8.56E-03	1.00E+00
ENSBTAG00000010856	5.28E-01	1.00E+00	1.28E+00	4.43E-01	1.47E+00	2.98E-01	6.96E-01	8.54E-01	-7.75E-01	1.00E+00
ENSBTAG00000030769	-2.64E-01	8.60E-01	-6.85E-01	1.45E-02	-7.10E-01	1.01E-02	-6.36E-01	2.68E-02	1.96E-02	1.00E+00
ENSBTAG00000013084	1.01E-02	1.00E+00	5.55E-01	1.28E-02	7.81E-01	2.26E-04	6.50E-01	3.28E-03	-5.90E-02	1.00E+00
ENSBTAG00000012606	-1.82E+00	4.50E-01	-2.55E+00	5.90E-02	2.74E-01	1.00E+00	1.05E-01	1.00E+00	-1.31E+00	7.18E-01

ENSBTAG00000004138	-8.34E-02	1.00E+00	-4.82E-01	1.07E-01	5.98E-01	3.06E-02	2.47E-01	5.29E-01	2.77E-01	8.00E-01
ENSBTAG00000021191	3.68E-01	8.23E-01	-2.31E-01	7.28E-01	9.33E-02	1.00E+00	3.61E-01	4.39E-01	9.06E-03	1.00E+00
ENSBTAG00000021192	-1.80E-01	1.00E+00	4.55E-01	9.76E-02	3.87E-01	1.81E-01	-6.11E-02	1.00E+00	-7.00E-01	6.15E-02
ENSBTAG00000043240	0.00E+00	1.00E+00	0.00E+00	1.00E+00	-5.13E-15	1.00E+00	0.00E+00	1.00E+00	0.00E+00	1.00E+00
ENSBTAG00000008203	-1.30E-01	1.00E+00	2.92E-01	4.60E-01	1.14E+00	3.13E-05	1.42E+00	2.12E-07	-5.88E-01	3.07E-01
ENSBTAG00000021185	-7.88E-01	1.00E+00	4.10E+00	1.23E-06	1.27E+00	4.36E-01	-9.38E-01	8.10E-01	-2.70E+00	6.03E-01
ENSBTAG00000023419	-2.14E-02	1.00E+00	-9.57E-03	1.00E+00	-5.75E-01	5.45E-02	-7.54E-01	8.98E-03	-4.69E-01	4.79E-01
ENSBTAG00000039971	9.74E-03	1.00E+00	1.07E+00	5.90E-04	1.10E+00	3.62E-04	1.22E+00	9.45E-05	-3.72E-02	1.00E+00
ENSBTAG00000040054	-1.07E+00	8.23E-01	2.65E+00	6.62E-04	1.21E+00	2.08E-01	3.65E-01	9.68E-01	2.13E-01	1.00E+00
ENSBTAG00000016113	4.51E-01	1.00E+00	-4.00E-01	8.79E-01	-6.23E-01	6.10E-01	-1.08E+00	2.64E-01	7.26E-01	8.26E-01
ENSBTAG00000016114	-1.91E+00	8.23E-01	1.39E+00	5.17E-01	-1.91E+00	4.28E-01	-1.47E-01	1.00E+00	7.05E-03	1.00E+00
ENSBTAG00000014595	-2.88E-01	7.58E-01	-1.38E+00	1.26E-09	-1.96E+00	7.20E-18	-1.49E+00	6.38E-11	1.18E-01	1.00E+00
ENSBTAG00000014597	-3.51E-01	8.23E-01	-8.01E-01	2.04E-02	-2.93E-01	5.26E-01	-4.73E-01	2.26E-01	-2.25E-02	1.00E+00
ENSBTAG00000038322	1.19E-02	1.00E+00	-3.89E+00	3.57E-02	-1.65E-01	1.00E+00	4.33E-02	1.00E+00	1.51E+00	4.15E-01
ENSBTAG00000038986	0.00E+00	1.00E+00	0.00E+00	1.00E+00	-5.13E-15	1.00E+00	0.00E+00	1.00E+00	0.00E+00	1.00E+00
ENSBTAG00000011119	0.00E+00	1.00E+00	0.00E+00	1.00E+00	-5.13E-15	1.00E+00	0.00E+00	1.00E+00	0.00E+00	1.00E+00
ENSBTAG00000047616	1.70E+00	7.68E-01	8.80E-01	8.52E-01	6.45E-01	9.78E-01	1.50E+00	4.52E-01	-1.91E+00	8.00E-01
ENSBTAG00000030667	-1.34E-01	1.00E+00	-1.38E+00	1.64E-02	-2.90E+00	2.46E-06	-1.04E+00	6.93E-02	6.85E-01	6.01E-01
ENSBTAG00000018013	2.17E-01	1.00E+00	-3.01E-01	4.85E-01	-3.37E-01	4.15E-01	-9.97E-02	1.00E+00	-2.78E-01	8.84E-01
ENSBTAG00000007107	-3.60E-01	7.79E-01	-2.00E+00	4.72E-11	-1.59E+00	8.27E-08	-1.08E+00	4.20E-04	4.58E-01	5.87E-01
ENSBTAG00000008546	-7.10E-01	8.70E-01	-2.11E+00	2.76E-02	-2.35E+00	1.01E-02	-1.47E+00	9.73E-02	-4.25E-01	1.00E+00
ENSBTAG00000004463	-3.31E-02	1.00E+00	-2.07E-01	5.81E-01	-1.89E-01	6.36E-01	7.73E-02	1.00E+00	1.52E-01	1.00E+00
ENSBTAG00000023369	9.41E-01	7.72E-01	1.22E+00	1.73E-01	1.28E-01	1.00E+00	1.11E+00	2.31E-01	-1.45E-01	1.00E+00
ENSBTAG00000000080	2.22E-01	9.44E-01	1.81E+00	6.77E-14	2.50E+00	7.15E-25	2.08E+00	7.79E-18	-2.00E-01	9.73E-01
ENSBTAG00000000082	3.49E-01	1.00E+00	4.91E+00	6.01E-12	2.97E+00	4.68E-04	1.78E+00	1.12E-01	-5.21E-01	1.00E+00
ENSBTAG00000001498	3.44E-01	4.65E-01	3.98E-02	1.00E+00	2.35E-01	4.14E-01	4.05E-01	8.49E-02	3.34E-01	5.45E-01
ENSBTAG00000012016	-3.96E-01	9.44E-01	-2.12E+00	1.07E-05	-3.02E+00	8.84E-10	-1.55E+00	1.29E-03	3.56E-01	9.66E-01
ENSBTAG00000045498	5.37E-01	1.00E+00	-1.13E+00	2.21E-01	-1.60E+00	5.65E-02	-7.07E-01	5.31E-01	6.37E-01	9.14E-01
ENSBTAG00000046291	-1.97E+00	3.53E-01	-4.57E-01	8.83E-01	-2.91E+00	1.85E-02	-1.66E+00	1.52E-01	-7.81E-01	9.11E-01
ENSBTAG00000048127	0.00E+00	1.00E+00	0.00E+00	1.00E+00	-5.13E-15	1.00E+00	0.00E+00	1.00E+00	0.00E+00	1.00E+00
ENSBTAG00000015388	-1.10E-02	1.00E+00	1.23E-01	9.22E-01	-7.21E-02	1.00E+00	-1.04E-01	9.93E-01	-2.87E-01	8.00E-01
ENSBTAG00000009872	-2.65E-01	8.88E-01	-2.09E+00	2.34E-14	-2.31E+00	3.10E-17	-3.02E-01	4.45E-01	5.96E-01	2.73E-01
ENSBTAG00000006754	-1.75E-01	1.00E+00	-2.34E+00	2.04E-08	-4.12E+00	5.80E-21	-3.30E+00	1.11E-14	1.65E-01	1.00E+00
ENSBTAG00000006757	5.65E-01	9.28E-01	-1.09E+00	2.72E-01	-1.34E+00	1.39E-01	-3.22E-01	9.35E-01	-1.97E-01	1.00E+00
ENSBTAG00000044132	-6.07E-01	2.84E-01	-2.20E+00	4.58E-13	-4.50E+00	1.05E-41	-2.67E+00	4.10E-18	8.15E-03	1.00E+00

ENSBTAG00000014514	-5.54E-01	4.29E-01	-3.28E+00	5.64E-20	-4.83E+00	1.24E-33	-3.81E+00	1.79E-25	6.88E-02	1.00E+00
ENSBTAG00000045968	0.00E+00	1.00E+00	0.00E+00	1.00E+00	-5.13E-15	1.00E+00	0.00E+00	1.00E+00	0.00E+00	1.00E+00
ENSBTAG00000021557	-1.40E+00	2.79E-02	-9.24E-01	6.89E-02	-2.83E+00	1.43E-07	-2.59E+00	8.60E-07	-2.22E-01	1.00E+00
ENSBTAG00000011617	-3.67E-01	1.00E+00	-1.48E+00	3.04E-02	-1.75E+00	8.26E-03	-1.71E+00	1.15E-02	-1.61E-01	1.00E+00
ENSBTAG00000011619	7.36E-01	1.80E-01	3.76E-01	4.21E-01	-4.22E-04	1.00E+00	-6.11E-01	1.17E-01	-3.64E-01	8.00E-01
ENSBTAG00000011621	4.99E+00	5.42E-03	6.90E+00	2.55E-10	5.82E+00	4.14E-06	5.01E+00	6.55E-04	3.93E+00	1.99E-01
ENSBTAG00000023374	2.32E-01	1.00E+00	1.37E+00	4.21E-02	5.76E-02	1.00E+00	3.60E-01	8.45E-01	-8.01E-01	7.79E-01
ENSBTAG00000011624	-7.53E-01	1.00E+00	-8.50E-01	6.83E-01	-4.25E+00	1.01E-03	-3.55E+00	6.32E-03	-1.01E+00	9.00E-01
ENSBTAG00000009172	1.37E-01	1.00E+00	2.66E+00	1.13E-14	1.60E+00	2.92E-06	2.73E-01	6.62E-01	9.62E-01	7.82E-02
ENSBTAG00000009174	8.15E-01	2.17E-02	1.35E+00	3.82E-07	1.37E+00	2.21E-07	8.05E-01	4.47E-03	6.68E-01	1.56E-01
ENSBTAG00000009177	2.56E-02	1.00E+00	1.61E+00	1.89E-05	1.26E+00	9.24E-04	4.57E-01	3.79E-01	2.10E-01	1.00E+00
ENSBTAG00000001294	2.05E+00	2.92E-02	3.34E+00	3.96E-06	2.80E+00	9.55E-05	2.58E+00	3.98E-04	8.35E-02	1.00E+00
ENSBTAG00000002368	1.91E-01	1.00E+00	7.45E-01	4.16E-03	-1.42E+00	2.64E-08	-1.12E+00	1.78E-05	4.78E-01	4.09E-01
ENSBTAG00000002378	2.48E-01	7.75E-01	1.20E+00	1.18E-09	4.36E-01	5.06E-02	2.77E-01	2.95E-01	6.24E-01	3.78E-02
ENSBTAG00000013337	-1.19E-01	1.00E+00	4.89E+00	1.08E-08	2.10E+00	1.54E-02	1.48E+00	1.22E-01	4.01E-01	1.00E+00
ENSBTAG00000013340	2.33E-01	8.86E-01	-7.41E-02	1.00E+00	3.06E-01	3.29E-01	8.11E-01	9.24E-04	-9.92E-02	1.00E+00
ENSBTAG00000013343	1.22E-01	1.00E+00	-5.67E-02	1.00E+00	-1.36E-01	7.91E-01	-5.45E-02	1.00E+00	-1.12E-01	1.00E+00
ENSBTAG00000039958	2.48E-01	8.50E-01	1.76E+00	5.24E-14	6.23E-01	1.48E-02	3.43E-01	2.71E-01	2.65E-01	8.00E-01
ENSBTAG00000023417	-4.57E-02	1.00E+00	1.74E+00	1.26E-15	1.86E+00	6.77E-18	1.32E+00	1.77E-09	-5.64E-02	1.00E+00
ENSBTAG00000038735	-1.41E+00	7.70E-01	-2.14E+00	1.64E-01	-4.12E+00	1.15E-02	-2.36E+00	1.05E-01	-9.10E-01	9.25E-01
ENSBTAG00000047078	7.98E-01	1.00E+00	7.77E-02	1.00E+00	-1.25E-01	1.00E+00	-1.46E-01	1.00E+00	2.45E+00	3.61E-01
ENSBTAG00000016125	1.32E+00	9.28E-01	-1.91E+00	4.69E-01	-1.25E-01	1.00E+00	6.26E-01	9.94E-01	-1.91E+00	8.00E-01
ENSBTAG00000001996	9.81E-02	1.00E+00	2.99E-01	2.20E-01	4.18E-01	5.70E-02	-3.26E-01	1.79E-01	-8.65E-03	1.00E+00
ENSBTAG00000023415	-8.32E-01	9.71E-02	-3.04E+00	2.13E-16	-1.95E+00	8.11E-09	-1.41E+00	3.45E-05	-3.38E-01	8.17E-01
ENSBTAG00000002844	-3.59E-01	7.22E-01	-2.22E+00	5.17E-14	-3.33E+00	1.96E-26	-1.98E+00	4.37E-12	1.93E-01	1.00E+00
ENSBTAG00000002853	5.74E-01	6.26E-01	-7.33E-02	1.00E+00	-5.71E-01	3.06E-01	-3.98E-01	5.45E-01	5.90E-01	6.16E-01
ENSBTAG00000006139	-6.81E-03	1.00E+00	-1.36E+00	1.51E-11	-1.66E+00	8.47E-17	-1.56E+00	7.54E-15	2.43E-01	7.87E-01
ENSBTAG00000030505	-1.91E+00	8.23E-01	8.84E-01	8.52E-01	-1.91E+00	4.28E-01	1.12E+00	6.71E-01	8.23E-01	1.00E+00
ENSBTAG00000030502	9.44E-01	8.23E-01	2.96E-01	1.00E+00	9.94E-01	3.70E-01	3.64E-01	9.94E-01	1.72E+00	2.44E-01
ENSBTAG00000030543	4.08E-01	9.93E-01	-1.51E+00	1.04E-02	2.02E-01	9.94E-01	-4.36E-02	1.00E+00	1.28E+00	1.18E-01
ENSBTAG00000030544	1.12E-02	1.00E+00	-1.91E+00	4.69E-01	6.39E-01	9.79E-01	-1.91E+00	4.39E-01	8.10E-01	1.00E+00
ENSBTAG00000011421	2.23E-01	9.25E-01	-4.12E-01	1.62E-01	-8.01E-01	1.57E-03	-6.86E-01	9.29E-03	-7.09E-02	1.00E+00
ENSBTAG00000016349	2.31E-01	9.43E-01	-3.90E-01	2.30E-01	-6.75E-01	1.51E-02	3.88E-01	2.38E-01	1.65E-01	1.00E+00
ENSBTAG00000045307	0.00E+00	1.00E+00	0.00E+00	1.00E+00	-5.13E-15	1.00E+00	0.00E+00	1.00E+00	0.00E+00	1.00E+00
ENSBTAG00000012186	-6.10E-01	1.34E-01	-1.42E+00	6.01E-08	-2.44E+00	8.85E-20	-1.44E+00	2.68E-08	-2.15E-01	9.37E-01

ENSBTAG00000008614	-7.62E-01	8.84E-01	1.86E-01	1.00E+00	1.13E-01	1.00E+00	-6.20E-01	6.27E-01	5.54E-01	9.35E-01
ENSBTAG00000043230	0.00E+00	1.00E+00	0.00E+00	1.00E+00	-5.13E-15	1.00E+00	0.00E+00	1.00E+00	0.00E+00	1.00E+00
ENSBTAG00000001062	0.00E+00	1.00E+00	0.00E+00	1.00E+00	-5.13E-15	1.00E+00	0.00E+00	1.00E+00	0.00E+00	1.00E+00
ENSBTAG00000007036	-2.70E+00	5.76E-01	1.28E+00	4.43E-01	-9.18E-01	8.09E-01	3.31E-01	1.00E+00	-7.75E-01	1.00E+00
ENSBTAG00000016526	3.56E-01	4.99E-01	1.61E+00	1.98E-14	5.26E-01	2.41E-02	1.40E-01	7.94E-01	1.13E-01	1.00E+00
ENSBTAG00000000330	-9.65E-02	1.00E+00	-6.22E-01	4.67E-03	-1.29E+00	3.48E-10	-1.28E+00	7.36E-10	1.56E-01	1.00E+00
ENSBTAG00000038045	-1.90E-01	9.97E-01	8.95E-01	1.02E-04	4.84E-01	5.83E-02	-4.30E-01	1.18E-01	-1.52E-01	1.00E+00
ENSBTAG00000005296	-5.85E-02	1.00E+00	3.00E-01	2.31E-01	4.67E-01	3.28E-02	5.28E-01	1.55E-02	7.78E-02	1.00E+00
ENSBTAG00000043197	0.00E+00	1.00E+00	0.00E+00	1.00E+00	-5.13E-15	1.00E+00	0.00E+00	1.00E+00	0.00E+00	1.00E+00
ENSBTAG00000042377	0.00E+00	1.00E+00	0.00E+00	1.00E+00	-5.13E-15	1.00E+00	0.00E+00	1.00E+00	0.00E+00	1.00E+00
ENSBTAG00000043453	0.00E+00	1.00E+00	0.00E+00	1.00E+00	-5.13E-15	1.00E+00	0.00E+00	1.00E+00	0.00E+00	1.00E+00
ENSBTAG00000042883	0.00E+00	1.00E+00	0.00E+00	1.00E+00	-5.13E-15	1.00E+00	0.00E+00	1.00E+00	0.00E+00	1.00E+00
ENSBTAG00000013924	-4.08E-02	1.00E+00	7.57E-02	1.00E+00	-1.52E-01	7.18E-01	-1.27E-01	8.33E-01	-1.55E-01	1.00E+00
ENSBTAG00000043168	0.00E+00	1.00E+00	0.00E+00	1.00E+00	-5.13E-15	1.00E+00	0.00E+00	1.00E+00	0.00E+00	1.00E+00
ENSBTAG00000030068	0.00E+00	1.00E+00	1.98E+00	4.43E-01	-5.13E-15	1.00E+00	1.76E+00	4.97E-01	0.00E+00	1.00E+00
ENSBTAG00000013926	-2.40E-01	7.46E-01	-4.97E-01	1.24E-02	-1.25E+00	6.40E-12	-1.51E+00	1.08E-16	2.73E-01	6.20E-01
ENSBTAG00000021799	-2.25E-01	9.86E-01	-1.70E-01	8.05E-01	-2.84E-01	4.69E-01	-2.85E-01	4.81E-01	-3.71E-01	6.85E-01
ENSBTAG00000014450	-1.69E-01	1.00E+00	2.30E-01	4.70E-01	3.75E-03	1.00E+00	-2.03E-01	5.69E-01	-2.12E-01	8.73E-01
ENSBTAG00000030563	-2.15E-01	1.00E+00	-1.22E+00	1.40E-04	-2.17E+00	9.79E-12	-2.11E+00	4.69E-11	-1.82E-01	1.00E+00
ENSBTAG00000014454	-6.64E-02	1.00E+00	-8.63E-01	1.72E-04	-1.95E+00	4.06E-18	-1.04E+00	4.91E-06	4.47E-01	3.59E-01
ENSBTAG00000020983	6.24E-01	2.22E-01	7.26E-01	2.08E-02	3.60E-01	3.63E-01	3.63E-01	3.71E-01	4.98E-02	1.00E+00
ENSBTAG00000006612	-1.89E-01	9.17E-01	1.41E-01	7.39E-01	2.33E-01	4.08E-01	1.03E-01	9.09E-01	4.39E-02	1.00E+00
ENSBTAG00000006633	1.93E-01	9.59E-01	2.56E-01	4.16E-01	-6.67E-01	3.96E-03	-8.39E-01	2.41E-04	1.44E-01	1.00E+00
ENSBTAG00000006638	3.29E-01	6.74E-01	6.43E-01	1.13E-02	3.07E-01	3.35E-01	1.82E-01	6.96E-01	2.43E-01	8.33E-01
ENSBTAG00000006646	9.27E-02	1.00E+00	5.85E-01	6.30E-02	9.76E-01	6.62E-04	1.15E+00	5.83E-05	-3.97E-01	6.53E-01
ENSBTAG00000012205	-6.75E-01	3.36E-01	-2.15E+00	1.08E-08	-1.73E+00	1.77E-06	-1.95E+00	1.03E-07	-4.11E-01	8.00E-01
ENSBTAG00000037818	0.00E+00	1.00E+00	1.98E+00	4.43E-01	-5.13E-15	1.00E+00	0.00E+00	1.00E+00	0.00E+00	1.00E+00
ENSBTAG00000019179	-1.18E+00	3.68E-01	-2.04E+00	8.07E-03	-2.05E+00	4.54E-03	-2.07E+00	4.84E-03	5.84E-02	1.00E+00
ENSBTAG00000019180	-7.14E-02	1.00E+00	-2.67E-01	3.16E-01	-2.26E-01	4.28E-01	2.20E-01	4.57E-01	1.41E-01	1.00E+00
ENSBTAG00000019183	-1.44E-01	1.00E+00	-5.69E-01	6.14E-02	-1.27E+00	3.74E-06	-1.15E+00	3.47E-05	-1.72E-01	1.00E+00
ENSBTAG00000008518	-1.64E-01	1.00E+00	-8.18E-01	1.08E-04	-6.56E-01	2.36E-03	-1.91E-01	5.68E-01	3.67E-01	4.70E-01
ENSBTAG00000030578	-8.78E-04	1.00E+00	5.60E-01	4.70E-02	-3.35E-01	3.16E-01	-6.04E-01	3.37E-02	2.87E-01	8.00E-01
ENSBTAG00000001834	-3.11E-02	1.00E+00	3.34E-01	3.94E-01	1.30E+00	2.67E-06	1.43E+00	3.15E-07	-1.22E-02	1.00E+00
ENSBTAG00000005763	-2.29E-02	1.00E+00	4.91E-01	2.56E-02	2.64E-01	3.24E-01	1.68E-01	6.39E-01	2.67E-02	1.00E+00
ENSBTAG00000047035	-4.04E-01	5.78E-01	5.14E-02	1.00E+00	6.33E-01	3.09E-02	5.48E-01	7.79E-02	1.01E-01	1.00E+00

ENSBTAG00000005766	-1.22E+00	4.80E-06	-1.80E+00	1.01E-13	-2.31E+00	1.76E-21	-2.47E+00	5.41E-24	-5.58E-01	2.36E-01
ENSBTAG00000003457	-1.35E+00	1.04E-04	-1.39E+00	6.96E-06	-2.29E+00	8.09E-14	-3.04E+00	3.24E-22	-7.53E-01	1.74E-01
ENSBTAG00000016997	-9.56E-01	2.84E-01	-2.90E+00	1.02E-06	-1.82E+00	4.03E-04	-8.99E-01	9.98E-02	-1.35E-01	1.00E+00
ENSBTAG00000005367	-1.08E+00	2.50E-06	-1.39E+00	3.36E-11	-1.18E+00	1.18E-08	-1.20E+00	9.13E-09	-4.81E-02	1.00E+00
ENSBTAG00000012064	6.21E-01	8.48E-02	3.01E+00	3.04E-37	1.60E+00	5.28E-12	1.43E+00	1.33E-09	-1.70E-01	1.00E+00
ENSBTAG00000044113	-8.86E-02	1.00E+00	1.63E+00	4.80E-05	-2.36E-01	8.46E-01	-9.54E-01	4.79E-02	-1.05E-01	1.00E+00
ENSBTAG00000002580	-1.14E-01	1.00E+00	-6.89E-01	4.69E-02	-1.44E+00	4.34E-06	-9.08E-01	6.77E-03	1.30E-01	1.00E+00
ENSBTAG00000039935	3.10E-02	1.00E+00	-1.32E+00	1.15E-01	-6.22E-01	5.12E-01	8.35E-02	1.00E+00	-1.42E-01	1.00E+00
ENSBTAG00000006780	2.02E-01	1.00E+00	-1.29E+00	1.47E-03	-1.06E+00	7.29E-03	-1.15E+00	3.99E-03	1.98E-01	1.00E+00
ENSBTAG00000048283	0.00E+00	1.00E+00	0.00E+00	1.00E+00	-5.13E-15	1.00E+00	0.00E+00	1.00E+00	0.00E+00	1.00E+00
ENSBTAG00000003264	3.19E-01	5.63E-01	1.33E+00	1.75E-11	1.02E+00	3.65E-07	2.99E-01	2.52E-01	1.60E-01	9.85E-01
ENSBTAG00000011083	-2.15E-01	9.95E-01	1.10E+00	1.68E-05	8.80E-01	7.73E-04	4.41E-01	1.59E-01	6.22E-03	1.00E+00
ENSBTAG00000043953	1.56E-01	1.00E+00	5.73E-01	4.16E-01	8.50E-02	1.00E+00	-7.05E-01	3.37E-01	-4.04E-01	1.00E+00
ENSBTAG00000020080	2.38E-01	1.00E+00	1.72E+00	6.36E-08	1.15E+00	4.93E-04	7.57E-01	3.91E-02	4.92E-02	1.00E+00
ENSBTAG00000020082	3.29E-02	1.00E+00	4.75E-01	4.47E-01	-2.38E-02	1.00E+00	-4.87E-01	4.73E-01	1.80E-01	1.00E+00
ENSBTAG00000045510	-1.42E-01	1.00E+00	-3.00E-01	3.09E-01	-4.69E-01	6.38E-02	-3.69E-01	1.85E-01	4.84E-02	1.00E+00
ENSBTAG00000006839	-2.70E-01	9.19E-01	3.72E-01	3.25E-01	3.00E-01	4.68E-01	2.85E-01	5.04E-01	3.58E-02	1.00E+00
ENSBTAG00000006840	-3.91E-01	6.60E-01	-8.64E-01	3.22E-03	-1.89E+00	1.33E-11	-2.29E+00	5.78E-16	-1.60E-01	1.00E+00
ENSBTAG00000006841	3.06E-02	1.00E+00	1.43E+00	1.61E-01	6.40E-01	7.29E-01	2.52E-01	1.00E+00	1.82E+00	2.39E-01
ENSBTAG00000013105	3.04E-01	1.00E+00	-3.41E-01	6.19E-01	-1.88E-01	9.41E-01	-5.91E-01	2.57E-01	8.58E-03	1.00E+00
ENSBTAG00000013106	2.60E-01	1.00E+00	1.91E-01	1.00E+00	1.71E-01	1.00E+00	-3.59E-01	1.00E+00	-3.12E-01	1.00E+00
ENSBTAG00000013107	8.59E-02	1.00E+00	8.18E-02	1.00E+00	-3.70E-01	7.99E-01	-4.33E-02	1.00E+00	2.80E-01	1.00E+00
ENSBTAG00000005576	-8.27E-01	3.98E-01	-1.67E+00	8.25E-04	-2.73E+00	7.30E-08	-1.52E+00	2.19E-03	-4.59E-01	8.34E-01
ENSBTAG00000015115	-1.48E-01	1.00E+00	-1.25E+00	5.33E-04	-1.98E+00	4.15E-08	-1.51E+00	2.19E-05	9.94E-02	1.00E+00
ENSBTAG00000011079	2.04E+00	5.22E-09	4.37E+00	6.35E-37	4.01E+00	2.04E-32	2.69E+00	2.79E-16	-5.50E-01	5.93E-01
ENSBTAG00000042784	0.00E+00	1.00E+00	0.00E+00	1.00E+00	-5.13E-15	1.00E+00	0.00E+00	1.00E+00	0.00E+00	1.00E+00
ENSBTAG00000042742	0.00E+00	1.00E+00	0.00E+00	1.00E+00	-5.13E-15	1.00E+00	0.00E+00	1.00E+00	0.00E+00	1.00E+00
ENSBTAG00000042613	0.00E+00	1.00E+00	0.00E+00	1.00E+00	-5.13E-15	1.00E+00	0.00E+00	1.00E+00	0.00E+00	1.00E+00
ENSBTAG00000043260	0.00E+00	1.00E+00	0.00E+00	1.00E+00	-5.13E-15	1.00E+00	0.00E+00	1.00E+00	0.00E+00	1.00E+00
ENSBTAG00000018539	-1.19E-02	1.00E+00	-9.83E-01	3.56E-01	-1.78E+00	5.75E-02	-1.81E+00	5.75E-02	-2.00E+00	2.61E-01
ENSBTAG00000018545	6.82E-01	1.00E+00	1.22E+00	3.88E-01	-6.63E-01	8.92E-01	-1.92E-01	1.00E+00	-5.13E-01	1.00E+00
ENSBTAG00000020415	-1.22E+00	3.06E-01	-2.51E+00	1.19E-03	-2.47E+00	6.60E-04	-2.79E+00	2.34E-04	-7.46E-01	7.59E-01
ENSBTAG00000015125	-2.70E+00	5.76E-01	6.02E-01	9.45E-01	-1.47E-01	1.00E+00	-2.70E+00	2.05E-01	-2.70E+00	6.01E-01
ENSBTAG00000030482	-7.92E-01	1.00E+00	8.16E-02	1.00E+00	-9.28E-01	8.07E-01	-9.45E-01	8.05E-01	-2.71E+00	6.01E-01
ENSBTAG00000030483	1.12E-02	1.00E+00	-1.91E+00	4.69E-01	-1.31E-01	1.00E+00	6.20E-01	9.94E-01	1.78E-02	1.00E+00

ENSBTAG00000015128	6.04E-03	1.00E+00	7.58E-02	1.00E+00	-1.91E+00	4.28E-01	-1.91E+00	4.39E-01	-1.91E+00	8.00E-01
ENSBTAG00000040177	2.71E+00	5.76E-01	0.00E+00	1.00E+00	1.78E+00	4.79E-01	0.00E+00	1.00E+00	1.91E+00	8.00E-01
ENSBTAG00000015129	-1.49E+00	4.84E-01	-1.39E+00	2.10E-01	-3.29E+00	4.63E-03	-2.05E+00	5.20E-02	-1.47E+00	5.40E-01
ENSBTAG00000002257	1.92E+00	8.23E-01	0.00E+00	1.00E+00	1.78E+00	4.79E-01	0.00E+00	1.00E+00	1.93E+00	8.00E-01
ENSBTAG00000030484	0.00E+00	1.00E+00	1.98E+00	4.43E-01	3.06E+00	1.36E-01	1.76E+00	4.97E-01	1.91E+00	8.00E-01
ENSBTAG00000009382	9.70E-03	1.00E+00	-3.21E+00	1.25E-01	-1.43E+00	4.79E-01	-6.83E-01	8.85E-01	-1.30E+00	9.00E-01
ENSBTAG00000012538	-2.59E+00	2.27E-01	-1.20E+00	3.97E-01	-1.44E+00	2.52E-01	-2.75E+00	3.92E-02	-4.51E+00	6.37E-02
ENSBTAG00000039212	-8.10E-02	1.00E+00	2.28E+00	9.28E-19	2.00E+00	7.94E-15	8.34E-01	4.12E-03	-8.34E-01	1.01E-01
ENSBTAG00000004611	0.00E+00	1.00E+00	0.00E+00	1.00E+00	-5.13E-15	1.00E+00	0.00E+00	1.00E+00	1.91E+00	8.00E-01
ENSBTAG00000004608	6.33E-01	5.42E-01	2.66E-01	7.86E-01	1.31E-01	1.00E+00	2.49E-02	1.00E+00	-5.21E-01	7.87E-01
ENSBTAG00000037537	-2.12E-01	1.00E+00	-1.18E+00	2.04E-02	2.03E-01	9.52E-01	2.54E-01	8.55E-01	6.61E-02	1.00E+00
ENSBTAG00000030440	1.92E+00	8.23E-01	1.98E+00	4.43E-01	-5.13E-15	1.00E+00	0.00E+00	1.00E+00	0.00E+00	1.00E+00
ENSBTAG00000047301	1.91E+00	8.23E-01	0.00E+00	1.00E+00	-5.13E-15	1.00E+00	1.76E+00	4.97E-01	0.00E+00	1.00E+00
ENSBTAG00000037710	0.00E+00	1.00E+00	0.00E+00	1.00E+00	-5.13E-15	1.00E+00	0.00E+00	1.00E+00	0.00E+00	1.00E+00
ENSBTAG00000037699	-7.38E-01	1.00E+00	1.05E-01	1.00E+00	1.84E-01	1.00E+00	7.75E-01	4.97E-01	4.65E-03	1.00E+00
ENSBTAG00000038024	-1.30E+00	9.34E-01	-4.26E-01	1.00E+00	-1.59E-01	1.00E+00	-1.45E+00	4.94E-01	-3.21E+00	4.34E-01
ENSBTAG00000038526	0.00E+00	1.00E+00	3.30E+00	1.02E-01	-5.13E-15	1.00E+00	0.00E+00	1.00E+00	0.00E+00	1.00E+00
ENSBTAG00000019473	-2.55E-01	1.00E+00	-1.76E+00	4.23E-03	-1.60E+00	6.51E-03	-1.60E+00	7.83E-03	-2.95E-01	1.00E+00
ENSBTAG00000020447	-2.13E-01	1.00E+00	1.18E-01	1.00E+00	-1.92E-03	1.00E+00	-3.41E-01	4.39E-01	1.39E-01	1.00E+00
ENSBTAG00000020449	-3.42E-02	1.00E+00	-3.87E-01	2.82E-01	-7.49E-01	1.19E-02	-7.46E-01	1.42E-02	-1.30E-01	1.00E+00
ENSBTAG00000020450	-2.14E+00	2.01E-04	-2.78E+00	3.80E-07	-1.46E+00	2.21E-03	-1.45E+00	2.76E-03	-3.24E-01	1.00E+00
ENSBTAG00000009137	-5.18E-01	3.59E-01	-1.31E+00	3.90E-06	-6.40E-01	3.36E-02	-3.44E-01	3.64E-01	-8.20E-01	5.89E-02
ENSBTAG00000003231	-2.40E-01	1.00E+00	-1.22E-01	1.00E+00	-2.00E-01	1.00E+00	7.00E-03	1.00E+00	6.56E-03	1.00E+00
ENSBTAG00000008852	6.97E-01	2.27E-01	-1.74E+00	2.81E-05	-3.48E-01	4.94E-01	-5.41E-01	2.27E-01	-8.60E-02	1.00E+00
ENSBTAG00000008851	8.37E-02	1.00E+00	-1.11E-01	1.00E+00	1.30E+00	1.48E-02	1.06E+00	6.39E-02	1.08E+00	2.96E-01
ENSBTAG00000047675	-4.56E-01	7.79E-01	-9.97E-01	1.57E-02	1.43E-01	1.00E+00	-2.24E-01	8.19E-01	1.07E-01	1.00E+00
ENSBTAG00000045164	0.00E+00	1.00E+00	0.00E+00	1.00E+00	-5.13E-15	1.00E+00	0.00E+00	1.00E+00	0.00E+00	1.00E+00
ENSBTAG00000040603	-2.94E-02	1.00E+00	1.10E+00	4.79E-06	1.07E+00	8.13E-06	4.53E-01	1.19E-01	-3.03E-02	1.00E+00
ENSBTAG00000023365	-7.90E-01	5.76E-01	1.13E+00	1.49E-02	1.32E+00	2.79E-03	6.70E-01	2.33E-01	2.09E-02	1.00E+00
ENSBTAG00000045880	-6.65E-02	1.00E+00	3.67E-01	4.25E-01	5.75E-01	1.25E-01	3.91E-01	3.88E-01	-1.39E-01	1.00E+00
ENSBTAG00000035868	1.82E-01	1.00E+00	-9.08E-01	1.40E-01	-8.65E-01	1.49E-01	-4.21E-01	6.07E-01	-6.19E-02	1.00E+00
ENSBTAG00000019227	2.08E-01	1.00E+00	5.58E-01	4.04E-01	6.08E-01	3.36E-01	5.07E-01	4.73E-01	4.58E-01	8.67E-01
ENSBTAG00000029918	0.00E+00	1.00E+00	0.00E+00	1.00E+00	-5.13E-15	1.00E+00	1.77E+00	4.97E-01	0.00E+00	1.00E+00
ENSBTAG00000029880	0.00E+00	1.00E+00	0.00E+00	1.00E+00	-5.13E-15	1.00E+00	0.00E+00	1.00E+00	1.91E+00	8.00E-01
ENSBTAG00000029844	2.70E+00	5.76E-01	0.00E+00	1.00E+00	1.78E+00	4.79E-01	0.00E+00	1.00E+00	0.00E+00	1.00E+00

ENSBTAG00000048204	1.46E-01	1.00E+00	-2.12E+00	4.01E-08	-4.55E-01	3.15E-01	-1.31E+00	4.11E-04	-3.06E-01	9.15E-01
ENSBTAG00000017672	3.91E-01	1.00E+00	-1.34E+00	4.01E-01	-1.71E-01	1.00E+00	-2.36E+00	1.05E-01	-2.21E+00	4.81E-01
ENSBTAG00000039491	1.67E+00	5.76E-01	-7.17E-01	9.45E-01	1.48E+00	2.98E-01	-1.72E-01	1.00E+00	5.13E-01	1.00E+00
ENSBTAG00000014593	-6.34E-02	1.00E+00	-4.68E-01	6.31E-02	-4.06E-02	1.00E+00	2.55E-01	4.09E-01	-1.07E-01	1.00E+00
ENSBTAG00000039111	1.97E+00	5.76E-01	-1.91E+00	4.69E-01	-1.37E-01	1.00E+00	-1.48E-01	1.00E+00	1.34E-04	1.00E+00
ENSBTAG00000038487	-1.14E-02	1.00E+00	-4.25E-01	7.69E-01	-5.03E-01	6.54E-01	-8.62E-01	3.09E-01	-2.95E-02	1.00E+00
ENSBTAG00000006954	-3.62E-01	8.09E-01	7.51E-02	1.00E+00	4.99E-01	1.50E-01	3.84E-01	3.32E-01	1.51E-01	1.00E+00
ENSBTAG00000003541	-1.04E-01	1.00E+00	1.09E+00	1.29E-03	1.74E+00	6.82E-08	1.10E+00	1.29E-03	3.91E-01	8.00E-01
ENSBTAG00000037906	-9.09E-02	1.00E+00	-4.33E-01	4.43E-01	-1.01E-01	1.00E+00	2.73E-01	7.07E-01	5.62E-03	1.00E+00
ENSBTAG00000010046	-3.62E-01	6.90E-01	5.77E-01	4.21E-02	6.33E-01	2.10E-02	3.31E-01	3.45E-01	3.53E-01	6.93E-01
ENSBTAG00000019851	-3.39E-02	1.00E+00	2.58E-01	3.37E-01	2.23E-01	4.32E-01	1.07E-01	8.92E-01	9.35E-02	1.00E+00
ENSBTAG00000029317	0.00E+00	1.00E+00	0.00E+00	1.00E+00	-5.13E-15	1.00E+00	0.00E+00	1.00E+00	0.00E+00	1.00E+00
ENSBTAG00000046864	6.04E-03	1.00E+00	-1.91E+00	4.69E-01	-1.25E-01	1.00E+00	-1.91E+00	4.39E-01	-1.91E+00	8.00E-01
ENSBTAG00000018162	0.00E+00	1.00E+00	1.98E+00	4.43E-01	2.55E+00	2.54E-01	0.00E+00	1.00E+00	0.00E+00	1.00E+00
ENSBTAG00000046472	-1.91E+00	8.23E-01	-1.91E+00	4.69E-01	-1.24E-01	1.00E+00	-1.91E+00	4.39E-01	-1.91E+00	8.00E-01
ENSBTAG00000047617	0.00E+00	1.00E+00	0.00E+00	1.00E+00	-5.13E-15	1.00E+00	0.00E+00	1.00E+00	0.00E+00	1.00E+00
ENSBTAG00000046510	0.00E+00	1.00E+00	0.00E+00	1.00E+00	-5.13E-15	1.00E+00	0.00E+00	1.00E+00	0.00E+00	1.00E+00
ENSBTAG00000047791	0.00E+00	1.00E+00	1.98E+00	4.43E-01	-5.13E-15	1.00E+00	0.00E+00	1.00E+00	0.00E+00	1.00E+00
ENSBTAG00000047761	3.50E-01	8.49E-01	2.06E+00	3.67E-10	5.77E-01	1.49E-01	-6.65E-01	1.05E-01	6.40E-01	4.20E-01
ENSBTAG00000038903	-6.61E-01	3.56E-01	-1.57E+00	2.35E-05	-5.43E-01	2.02E-01	-1.72E+00	3.33E-06	-6.15E-01	4.79E-01
ENSBTAG00000030470	-1.28E-01	1.00E+00	-2.69E-02	1.00E+00	1.72E-01	6.27E-01	-3.77E-01	1.29E-01	-1.28E-03	1.00E+00
ENSBTAG00000038125	-5.44E-01	1.00E+00	-5.40E+00	1.13E-04	-1.98E+00	2.46E-02	-3.64E+00	6.41E-04	6.12E-01	8.16E-01
ENSBTAG00000017651	3.27E-02	1.00E+00	2.89E-02	1.00E+00	7.83E-01	8.57E-02	4.74E-01	4.13E-01	-3.60E-02	1.00E+00
ENSBTAG00000011926	2.09E-01	8.82E-01	1.32E+00	1.39E-10	1.03E+00	6.55E-07	2.64E-01	3.66E-01	-1.55E-03	1.00E+00
ENSBTAG00000033604	-2.70E+00	5.76E-01	6.03E-01	9.45E-01	-2.70E+00	1.99E-01	-2.70E+00	2.05E-01	7.73E-03	1.00E+00
ENSBTAG00000045571	-4.41E-01	9.32E-01	1.04E-01	1.00E+00	2.49E-01	8.48E-01	7.06E-01	1.66E-01	2.50E-01	1.00E+00
ENSBTAG00000037440	2.43E-01	8.60E-01	1.56E+00	2.04E-11	1.57E+00	1.13E-11	6.20E-01	1.66E-02	3.50E-02	1.00E+00
ENSBTAG00000047712	-5.20E-02	1.00E+00	-2.41E-01	6.24E-01	7.10E-01	1.37E-02	6.86E-01	2.04E-02	-3.79E-02	1.00E+00
ENSBTAG00000030454	7.70E-01	8.99E-01	-3.45E-01	1.00E+00	-1.55E-01	1.00E+00	-2.56E+00	6.62E-02	-4.03E-01	1.00E+00
ENSBTAG00000011052	6.27E-01	3.64E-01	2.22E+00	1.79E-11	1.96E+00	2.78E-09	7.05E-01	6.67E-02	-2.39E-01	1.00E+00
ENSBTAG00000048191	-1.50E+00	2.81E-01	-3.63E-01	8.72E-01	-9.43E-01	2.76E-01	-9.65E-01	2.72E-01	-3.95E+00	4.52E-03
ENSBTAG00000040411	2.74E-01	6.99E-01	1.10E+00	3.60E-08	8.78E-01	1.31E-05	6.00E-01	5.35E-03	-3.02E-02	1.00E+00
ENSBTAG00000021433	5.03E-01	6.41E-01	1.05E+00	4.89E-03	6.38E-01	1.26E-01	2.77E-01	6.80E-01	1.66E-01	1.00E+00
ENSBTAG00000015899	0.00E+00	1.00E+00	0.00E+00	1.00E+00	-5.13E-15	1.00E+00	0.00E+00	1.00E+00	0.00E+00	1.00E+00
ENSBTAG00000011844	3.21E+00	3.75E-01	0.00E+00	1.00E+00	-5.13E-15	1.00E+00	1.77E+00	4.97E-01	0.00E+00	1.00E+00

ENSBTAG00000047053	-1.87E-02	1.00E+00	8.36E-01	1.63E-01	8.99E-01	1.21E-01	2.29E-01	9.59E-01	-7.53E-02	1.00E+00
ENSBTAG00000045985	-3.45E-02	1.00E+00	1.13E-01	1.00E+00	5.76E-01	2.47E-01	1.47E-01	1.00E+00	-4.20E-02	1.00E+00
ENSBTAG00000004925	2.59E-01	1.00E+00	1.68E+00	3.59E-07	1.43E+00	1.61E-05	4.73E-01	2.78E-01	5.81E-01	4.90E-01
ENSBTAG00000038674	7.30E-01	6.85E-01	7.13E-01	3.20E-01	5.61E-01	4.78E-01	3.60E-01	7.80E-01	-8.91E-01	6.27E-01
ENSBTAG00000039969	-2.67E-01	1.00E+00	7.01E-01	4.57E-01	6.05E-01	5.35E-01	8.78E-02	1.00E+00	-5.34E-01	1.00E+00
ENSBTAG00000017993	2.71E+00	5.76E-01	0.00E+00	1.00E+00	-5.13E-15	1.00E+00	0.00E+00	1.00E+00	0.00E+00	1.00E+00
ENSBTAG00000030444	0.00E+00	1.00E+00	0.00E+00	1.00E+00	-5.13E-15	1.00E+00	0.00E+00	1.00E+00	0.00E+00	1.00E+00
ENSBTAG00000038702	3.60E-01	1.00E+00	1.57E+00	8.83E-02	1.38E+00	1.48E-01	1.91E-01	1.00E+00	2.63E-01	1.00E+00
ENSBTAG00000039849	0.00E+00	1.00E+00	0.00E+00	1.00E+00	-5.13E-15	1.00E+00	0.00E+00	1.00E+00	0.00E+00	1.00E+00
ENSBTAG00000034090	0.00E+00	1.00E+00	0.00E+00	1.00E+00	-5.13E-15	1.00E+00	0.00E+00	1.00E+00	0.00E+00	1.00E+00
ENSBTAG00000045581	2.98E-01	1.00E+00	1.32E+00	9.91E-04	1.08E+00	8.45E-03	2.63E-01	7.65E-01	3.33E-01	9.31E-01
ENSBTAG00000033642	0.00E+00	1.00E+00	0.00E+00	1.00E+00	-5.13E-15	1.00E+00	0.00E+00	1.00E+00	0.00E+00	1.00E+00
ENSBTAG00000038755	-8.21E-01	8.07E-01	-7.05E-01	4.81E-01	-1.26E+00	1.31E-01	-1.03E+00	2.41E-01	5.85E-01	8.49E-01
ENSBTAG00000046403	0.00E+00	1.00E+00	0.00E+00	1.00E+00	-5.13E-15	1.00E+00	0.00E+00	1.00E+00	0.00E+00	1.00E+00
ENSBTAG00000016513	-2.22E-01	1.00E+00	7.84E-02	1.00E+00	1.20E+00	2.52E-05	5.87E-01	7.71E-02	1.96E-01	1.00E+00
ENSBTAG00000015061	3.02E+00	5.82E-05	3.79E+00	7.88E-10	2.79E+00	4.25E-05	2.42E+00	9.79E-04	2.96E-03	1.00E+00
ENSBTAG00000038149	5.09E-01	1.00E+00	-7.94E-02	1.00E+00	-1.29E+00	1.64E-01	-1.66E-01	1.00E+00	1.11E+00	5.92E-01
ENSBTAG00000036392	6.67E-01	1.00E+00	-5.71E-01	8.99E-01	-3.87E+00	2.36E-02	-3.87E+00	2.58E-02	-2.62E-01	1.00E+00
ENSBTAG00000040392	-8.60E-01	2.52E-01	-1.82E+00	2.07E-05	-1.47E+00	5.93E-04	-1.40E+00	1.45E-03	-1.98E-01	1.00E+00
ENSBTAG00000000336	6.06E-03	1.00E+00	7.64E-02	1.00E+00	-1.91E+00	4.28E-01	-1.47E-01	1.00E+00	8.11E-01	1.00E+00
ENSBTAG00000046961	0.00E+00	1.00E+00	0.00E+00	1.00E+00	3.43E+00	7.38E-02	2.53E+00	2.71E-01	1.91E+00	8.00E-01
ENSBTAG00000036224	6.94E-01	2.25E-01	9.61E-01	4.54E-03	6.26E-01	9.28E-02	1.55E-01	9.43E-01	6.96E-01	3.02E-01
ENSBTAG00000014953	0.00E+00	1.00E+00	0.00E+00	1.00E+00	-5.13E-15	1.00E+00	0.00E+00	1.00E+00	0.00E+00	1.00E+00
ENSBTAG00000009171	1.91E+00	8.23E-01	3.30E+00	1.03E-01	-5.13E-15	1.00E+00	1.76E+00	4.97E-01	2.73E+00	6.01E-01
ENSBTAG00000014328	6.93E-01	1.00E+00	7.73E-01	7.31E-01	1.32E+00	2.93E-01	7.32E-01	7.47E-01	6.86E-01	1.00E+00
ENSBTAG00000047602	1.32E+00	9.29E-01	-1.91E+00	4.69E-01	-1.91E+00	4.28E-01	-1.91E+00	4.39E-01	6.29E-03	1.00E+00
ENSBTAG00000002290	1.44E+00	1.00E+00	-5.39E-02	1.00E+00	-4.07E-01	1.00E+00	-4.01E-01	1.00E+00	8.80E-01	1.00E+00
ENSBTAG00000009364	6.04E-03	1.00E+00	-1.91E+00	4.69E-01	-1.91E+00	4.28E-01	-1.91E+00	4.39E-01	-1.91E+00	8.00E-01
ENSBTAG00000013345	0.00E+00	1.00E+00	0.00E+00	1.00E+00	-5.13E-15	1.00E+00	0.00E+00	1.00E+00	1.93E+00	8.00E-01
ENSBTAG00000015987	2.72E+00	5.76E-01	3.30E+00	1.02E-01	-5.13E-15	1.00E+00	3.03E+00	1.50E-01	3.59E+00	3.09E-01
ENSBTAG00000030416	1.79E+00	3.69E-01	3.10E+00	1.20E-03	2.53E+00	1.07E-02	4.79E+00	2.44E-07	-2.52E-01	1.00E+00
ENSBTAG00000015139	0.00E+00	1.00E+00	1.99E+00	4.43E-01	2.55E+00	2.54E-01	1.76E+00	4.97E-01	0.00E+00	1.00E+00
ENSBTAG00000018152	1.82E-02	1.00E+00	-1.33E+00	1.34E-07	-5.96E-01	2.94E-02	7.21E-01	7.00E-03	3.11E-02	1.00E+00
ENSBTAG00000013550	6.22E-01	3.71E-01	-3.03E-01	6.08E-01	-1.13E+00	2.95E-03	-1.57E+00	4.51E-05	7.83E-01	2.08E-01
ENSBTAG00000007506	-3.21E+00	3.75E-01	9.86E-02	1.00E+00	5.18E-01	9.35E-01	-1.81E-01	1.00E+00	1.89E-02	1.00E+00

ENSBTAG00000007509	-1.67E+00	7.81E-01	3.88E-01	1.00E+00	-1.72E-01	1.00E+00	1.16E+00	3.42E-01	2.99E-01	1.00E+00
ENSBTAG00000037338	0.00E+00	1.00E+00	0.00E+00	1.00E+00	-5.13E-15	1.00E+00	0.00E+00	1.00E+00	0.00E+00	1.00E+00
ENSBTAG00000021657	-2.70E+00	5.76E-01	8.76E-02	1.00E+00	1.02E+00	5.89E-01	1.23E+00	4.63E-01	5.13E-01	1.00E+00
ENSBTAG00000002596	3.63E-01	1.00E+00	-1.62E+00	1.29E-02	1.09E+00	3.13E-02	1.25E+00	1.28E-02	1.53E+00	2.05E-02
ENSBTAG00000023349	0.00E+00	1.00E+00	0.00E+00	1.00E+00	-5.13E-15	1.00E+00	0.00E+00	1.00E+00	0.00E+00	1.00E+00
ENSBTAG00000004725	3.22E+00	3.75E-01	2.79E+00	2.10E-01	-5.13E-15	1.00E+00	3.03E+00	1.50E-01	0.00E+00	1.00E+00
ENSBTAG00000040294	0.00E+00	1.00E+00	0.00E+00	1.00E+00	-5.13E-15	1.00E+00	0.00E+00	1.00E+00	0.00E+00	1.00E+00
ENSBTAG00000046996	-2.21E-01	8.80E-01	2.56E-01	4.22E-01	2.01E-01	5.68E-01	1.02E-01	9.57E-01	-2.19E-02	1.00E+00
ENSBTAG00000002830	1.18E-01	1.00E+00	9.14E-01	5.08E-07	9.30E-01	2.74E-07	5.76E-01	2.97E-03	2.67E-01	6.26E-01
ENSBTAG00000011517	-1.84E-01	1.00E+00	1.59E+00	4.66E-11	-4.60E-01	1.13E-01	1.10E-01	9.68E-01	-1.20E-01	1.00E+00
ENSBTAG00000045864	-2.14E-01	1.00E+00	-1.24E+00	9.09E-03	-1.60E+00	5.53E-04	-7.56E-01	1.36E-01	2.41E-02	1.00E+00
ENSBTAG00000047508	-4.83E-01	9.32E-01	-1.64E+00	7.88E-03	-1.37E+00	2.18E-02	-2.60E-02	1.00E+00	-3.66E-01	1.00E+00
ENSBTAG00000016299	-4.77E-01	3.75E-01	-2.33E+00	3.22E-17	-6.07E-01	3.11E-02	-5.21E-01	8.11E-02	2.77E-01	8.00E-01
ENSBTAG00000037582	-1.25E-01	1.00E+00	5.72E-01	1.23E-01	6.00E-01	9.73E-02	7.17E-01	4.17E-02	1.82E-01	1.00E+00
ENSBTAG00000012333	-2.62E-01	9.07E-01	-1.08E+00	1.89E-04	-3.21E-01	4.04E-01	-1.16E-01	9.87E-01	1.54E-01	1.00E+00
ENSBTAG00000030403	-2.74E-01	8.32E-01	-1.26E-01	9.35E-01	-1.21E-01	9.48E-01	-2.45E-01	5.51E-01	6.18E-02	1.00E+00
ENSBTAG00000006121	-3.75E-01	1.00E+00	-1.46E+00	8.27E-02	-1.36E+00	9.04E-02	-1.13E+00	1.74E-01	-1.04E-01	1.00E+00
ENSBTAG00000030885	-2.22E-01	1.00E+00	-1.26E+00	5.73E-05	-1.90E+00	1.10E-09	-6.46E-01	5.30E-02	1.02E-01	1.00E+00
ENSBTAG00000043361	0.00E+00	1.00E+00	0.00E+00	1.00E+00	-5.13E-15	1.00E+00	0.00E+00	1.00E+00	0.00E+00	1.00E+00
ENSBTAG00000019051	-3.59E-01	8.53E-01	-3.18E-01	5.67E-01	-1.40E+00	1.39E-04	-1.31E+00	4.68E-04	-1.42E-01	1.00E+00
ENSBTAG00000038034	1.41E-02	1.00E+00	1.73E+00	8.07E-14	1.34E+00	1.16E-08	7.45E-01	3.82E-03	1.86E-01	1.00E+00
ENSBTAG00000015950	-1.94E-01	1.00E+00	-9.26E-01	6.43E-04	-2.19E+00	8.76E-17	-2.33E+00	1.56E-18	-2.38E-01	9.06E-01
ENSBTAG00000008932	1.10E+00	7.29E-01	1.71E+00	5.04E-02	1.33E+00	1.67E-01	1.58E+00	7.75E-02	1.43E+00	4.64E-01
ENSBTAG00000009211	5.00E-01	2.14E-01	1.30E+00	8.41E-09	1.54E+00	5.01E-12	1.10E+00	1.81E-06	-3.96E-01	5.14E-01
ENSBTAG00000023343	-2.06E-02	1.00E+00	7.21E-02	1.00E+00	-4.49E-03	1.00E+00	3.58E-02	1.00E+00	-4.47E-03	1.00E+00
ENSBTAG00000021057	7.43E-01	8.23E-01	1.24E+00	7.18E-02	5.76E-01	5.68E-01	-6.37E-03	1.00E+00	-1.05E-01	1.00E+00
ENSBTAG00000047400	8.99E-01	1.00E+00	9.91E-01	7.08E-01	1.07E+00	6.33E-01	3.41E+00	3.15E-03	1.08E-03	1.00E+00
ENSBTAG00000044157	3.61E+00	2.29E-01	2.79E+00	2.11E-01	2.55E+00	2.55E-01	3.68E+00	4.85E-02	2.71E+00	6.01E-01
ENSBTAG00000007200	1.68E+00	7.75E-01	7.74E-02	1.00E+00	2.27E+00	1.15E-01	2.43E+00	8.34E-02	2.61E+00	2.86E-01
ENSBTAG00000014087	-6.53E-01	7.89E-02	-1.95E+00	1.92E-14	-1.52E+00	1.08E-09	-1.19E+00	2.70E-06	7.41E-02	1.00E+00
ENSBTAG00000022813	-5.47E-01	1.59E-01	-1.73E+00	3.61E-13	-2.42E+00	7.68E-24	-2.49E+00	5.92E-25	7.16E-02	1.00E+00
ENSBTAG00000015226	-2.39E-01	1.00E+00	-9.03E-01	1.64E-02	-1.38E+00	1.24E-04	-1.50E+00	3.73E-05	-4.39E-01	7.37E-01
ENSBTAG00000003644	-2.02E-01	9.50E-01	9.88E-01	1.11E-05	1.13E+00	3.26E-07	8.20E-01	4.01E-04	-2.72E-01	8.00E-01
ENSBTAG00000021157	3.83E-02	1.00E+00	1.87E-01	5.06E-01	3.04E-01	1.84E-01	9.91E-02	8.97E-01	4.21E-02	1.00E+00
ENSBTAG00000044492	0.00E+00	1.00E+00	0.00E+00	1.00E+00	-5.13E-15	1.00E+00	0.00E+00	1.00E+00	0.00E+00	1.00E+00

ENSBTAG00000012189	-4.14E-01	9.97E-01	-1.46E+00	6.80E-03	-3.16E+00	2.89E-09	-2.93E+00	4.31E-08	-1.18E-02	1.00E+00
ENSBTAG00000040559	-6.98E-02	1.00E+00	-4.81E-01	3.62E-01	-1.57E+00	8.48E-05	-2.56E+00	4.29E-10	2.44E-01	1.00E+00
ENSBTAG00000002522	-3.52E-01	8.23E-01	-1.30E+00	1.22E-04	-2.99E+00	9.65E-17	-2.20E+00	1.24E-10	4.59E-02	1.00E+00
ENSBTAG00000002515	-1.48E-01	1.00E+00	-8.94E-01	8.34E-02	-1.65E+00	5.37E-04	-2.30E+00	4.37E-06	3.87E-01	8.97E-01
ENSBTAG00000006424	-7.86E-01	1.00E+00	9.03E-02	1.00E+00	1.03E+00	5.83E-01	-1.64E-01	1.00E+00	8.99E-01	1.00E+00
ENSBTAG00000006419	4.91E-01	1.00E+00	1.60E+00	2.64E-01	-5.77E-01	8.97E-01	1.26E+00	4.39E-01	7.55E-01	1.00E+00
ENSBTAG00000017847	2.48E-01	8.23E-01	1.14E+00	7.70E-08	1.16E+00	4.39E-08	7.63E-01	6.38E-04	1.30E-01	1.00E+00
ENSBTAG00000009512	1.20E-02	1.00E+00	1.02E+00	6.20E-02	-2.47E-01	9.52E-01	-4.00E-01	7.17E-01	2.63E-01	1.00E+00
ENSBTAG00000030393	7.82E-02	1.00E+00	2.40E+00	6.14E-25	2.64E+00	8.81E-30	1.82E+00	6.29E-15	-3.34E-01	7.05E-01
ENSBTAG00000039215	-1.62E-01	1.00E+00	1.04E-01	1.00E+00	7.15E-01	4.96E-01	8.68E-01	3.77E-01	-1.56E-01	1.00E+00
ENSBTAG00000021647	-6.89E-01	1.00E+00	3.45E-01	9.83E-01	2.34E+00	8.76E-04	2.92E+00	1.71E-05	6.38E-01	9.68E-01
ENSBTAG00000045529	5.65E-02	1.00E+00	2.85E-01	9.02E-01	1.06E-01	1.00E+00	1.74E-01	1.00E+00	-3.68E-01	1.00E+00
ENSBTAG00000047473	-1.17E+00	8.70E-01	-1.09E+00	5.49E-01	-8.27E-01	6.74E-01	6.13E-01	7.48E-01	6.45E-01	1.00E+00
ENSBTAG00000045854	-4.13E-01	9.66E-01	-2.61E-01	8.78E-01	6.54E-01	2.54E-01	4.64E-01	4.97E-01	-4.04E-01	9.44E-01
ENSBTAG00000048184	-3.79E-01	8.23E-01	-2.25E-01	7.92E-01	4.63E-01	2.94E-01	3.05E-01	5.88E-01	-2.02E-01	1.00E+00
ENSBTAG00000047953	0.00E+00	1.00E+00	2.79E+00	2.10E-01	-5.13E-15	1.00E+00	1.76E+00	4.97E-01	0.00E+00	1.00E+00
ENSBTAG00000047175	-8.63E-01	1.00E+00	-2.80E-01	1.00E+00	3.79E-01	1.00E+00	-1.05E+00	5.99E-01	-8.76E-01	1.00E+00
ENSBTAG00000045795	-4.93E-01	1.00E+00	-5.52E-01	7.63E-01	-8.61E-01	4.66E-01	-8.00E-01	5.17E-01	9.14E-02	1.00E+00
ENSBTAG00000039413	4.44E-01	9.74E-01	6.86E-01	3.18E-01	-5.26E-03	1.00E+00	3.59E-01	7.56E-01	-3.81E-01	1.00E+00
ENSBTAG00000038797	9.14E-01	5.76E-01	1.13E+00	9.28E-02	1.37E+00	2.36E-02	7.79E-01	3.30E-01	-3.20E-01	1.00E+00
ENSBTAG00000026080	-1.01E+00	2.31E-01	-2.58E+00	5.09E-06	-5.01E-01	4.33E-01	-2.90E-01	7.90E-01	2.08E-01	1.00E+00
ENSBTAG00000003470	-4.04E-01	8.18E-01	-1.45E+00	6.63E-05	-2.70E+00	5.61E-13	-2.37E+00	1.49E-10	-2.84E-01	9.50E-01
ENSBTAG00000011689	-1.53E-01	1.00E+00	2.99E-01	3.28E-01	-1.52E-02	1.00E+00	-8.98E-01	1.41E-04	-2.81E-02	1.00E+00
ENSBTAG00000011693	-1.17E-01	1.00E+00	8.45E-01	9.86E-03	1.09E+00	4.56E-04	4.13E-01	3.22E-01	-4.79E-02	1.00E+00
ENSBTAG00000045989	4.04E-01	7.67E-01	4.78E-01	2.37E-01	5.17E-01	1.84E-01	-5.51E-02	1.00E+00	4.67E-01	6.27E-01
ENSBTAG00000019547	8.23E-01	5.12E-01	8.23E-01	1.86E-01	3.69E-01	7.23E-01	-2.93E-01	8.92E-01	-3.08E-02	1.00E+00
ENSBTAG00000004043	1.18E+00	1.82E-05	1.80E+00	1.86E-13	1.43E+00	4.48E-09	9.29E-01	3.17E-04	3.19E-04	1.00E+00
ENSBTAG00000039892	1.26E+00	1.01E-01	1.89E+00	7.79E-05	1.23E+00	2.11E-02	7.17E-01	3.00E-01	-2.60E-01	1.00E+00
ENSBTAG00000039932	0.00E+00	1.00E+00	0.00E+00	1.00E+00	-5.13E-15	1.00E+00	0.00E+00	1.00E+00	0.00E+00	1.00E+00
ENSBTAG00000026944	1.14E+00	1.99E-01	1.86E+00	1.85E-04	1.68E+00	8.31E-04	1.31E+00	1.74E-02	-1.84E-01	1.00E+00
ENSBTAG00000037830	2.47E-01	1.00E+00	9.76E-01	1.88E-01	3.09E-01	9.21E-01	4.00E-01	7.98E-01	5.61E-02	1.00E+00
ENSBTAG00000000930	8.18E-01	8.15E-01	1.41E+00	4.92E-02	2.14E-01	1.00E+00	-3.66E-01	9.52E-01	-5.69E-01	1.00E+00
ENSBTAG00000048145	1.12E+00	9.01E-02	1.53E+00	5.06E-04	1.05E+00	2.67E-02	1.30E+00	4.54E-03	-2.83E-01	1.00E+00
ENSBTAG00000039086	1.30E+00	3.53E-02	1.79E+00	5.58E-05	1.54E+00	5.87E-04	1.32E+00	5.31E-03	-2.00E-01	1.00E+00
ENSBTAG00000019348	1.27E+00	5.10E-07	1.84E+00	6.48E-16	7.06E-01	4.65E-03	3.47E-01	2.71E-01	-4.96E-01	3.87E-01

ENSBTAG00000039691	8.66E-02	1.00E+00	6.10E-01	4.10E-01	6.05E-01	3.97E-01	1.78E-01	1.00E+00	-1.23E+00	4.01E-01
ENSBTAG00000005841	1.40E+00	1.48E-04	1.72E+00	6.58E-08	8.68E-01	1.33E-02	3.97E-01	4.11E-01	-3.23E-01	9.00E-01
ENSBTAG00000006487	1.04E-01	1.00E+00	6.80E-01	3.87E-03	7.57E-01	1.08E-03	5.18E-01	4.09E-02	-9.33E-02	1.00E+00
ENSBTAG00000006486	1.06E-01	1.00E+00	1.14E+00	2.33E-07	9.18E-01	3.85E-05	3.88E-01	1.52E-01	-2.24E-02	1.00E+00
ENSBTAG00000015908	-1.00E-01	1.00E+00	4.04E-01	1.25E-01	7.92E-01	5.32E-04	7.29E-01	1.95E-03	-7.62E-02	1.00E+00
ENSBTAG00000020062	-1.92E-01	1.00E+00	4.93E-01	1.16E-01	1.36E-01	9.10E-01	-1.56E-01	8.53E-01	9.85E-02	1.00E+00
ENSBTAG00000023333	4.13E-01	6.70E-01	1.96E+00	1.96E-11	1.13E+00	1.56E-04	7.14E-01	3.06E-02	-4.45E-02	1.00E+00
ENSBTAG00000014900	4.34E-01	3.33E-01	1.84E+00	6.67E-17	8.02E-01	4.62E-04	2.12E-01	5.53E-01	3.56E-01	5.88E-01
ENSBTAG00000007757	-1.59E-02	1.00E+00	7.85E-01	3.38E-04	5.28E-01	2.33E-02	4.19E-01	9.65E-02	-8.91E-02	1.00E+00
ENSBTAG00000007756	-1.32E-01	1.00E+00	1.33E+00	1.72E-06	7.62E-01	1.00E-02	-1.09E-01	1.00E+00	-2.78E-02	1.00E+00
ENSBTAG00000007754	-1.01E-01	1.00E+00	2.53E-01	7.01E-01	4.06E-01	3.81E-01	-1.89E-01	8.72E-01	7.38E-02	1.00E+00
ENSBTAG00000001051	8.58E-01	3.69E-01	2.13E+00	1.38E-06	1.85E+00	3.20E-05	1.62E+00	4.37E-04	1.47E+00	2.34E-02
ENSBTAG00000045861	1.81E+00	1.73E-02	2.19E+00	1.33E-04	2.10E+00	2.18E-04	1.91E+00	1.25E-03	1.81E+00	3.78E-02
ENSBTAG00000038890	0.00E+00	1.00E+00	3.68E+00	5.09E-02	-5.13E-15	1.00E+00	2.53E+00	2.71E-01	0.00E+00	1.00E+00
ENSBTAG00000030360	0.00E+00	1.00E+00	0.00E+00	1.00E+00	-5.13E-15	1.00E+00	0.00E+00	1.00E+00	0.00E+00	1.00E+00
ENSBTAG00000038416	0.00E+00	1.00E+00	0.00E+00	1.00E+00	-5.13E-15	1.00E+00	0.00E+00	1.00E+00	0.00E+00	1.00E+00
ENSBTAG00000036215	0.00E+00	1.00E+00	0.00E+00	1.00E+00	-5.13E-15	1.00E+00	0.00E+00	1.00E+00	0.00E+00	1.00E+00
ENSBTAG00000006961	9.50E-01	8.54E-01	1.91E+00	3.17E-02	1.54E+00	1.11E-01	1.62E+00	9.13E-02	1.11E+00	7.79E-01
ENSBTAG00000006965	5.18E-01	1.00E+00	9.74E-01	6.53E-01	-9.29E-01	8.06E-01	-9.46E-01	8.04E-01	-7.92E-01	1.00E+00
ENSBTAG00000013247	-1.69E+00	1.96E-01	-1.14E+00	1.94E-01	-7.81E-01	3.86E-01	-2.55E+00	3.86E-03	1.26E+00	1.81E-01
ENSBTAG00000002904	-1.60E-01	1.00E+00	1.22E+00	3.57E-08	8.80E-01	1.01E-04	4.48E-01	8.86E-02	-1.27E-01	1.00E+00
ENSBTAG00000034302	-1.36E-01	1.00E+00	1.92E-03	1.00E+00	4.05E-01	6.28E-01	-3.84E-01	6.88E-01	-5.68E-01	8.00E-01
ENSBTAG00000046111	-4.70E-01	3.71E-01	-8.03E-01	2.66E-03	-1.64E-01	7.71E-01	3.86E-01	2.08E-01	-2.24E-01	9.11E-01
ENSBTAG00000039081	0.00E+00	1.00E+00	0.00E+00	1.00E+00	-5.13E-15	1.00E+00	0.00E+00	1.00E+00	0.00E+00	1.00E+00
ENSBTAG00000046087	-1.91E+00	8.23E-01	-1.91E+00	4.69E-01	-1.91E+00	4.28E-01	-1.91E+00	4.39E-01	8.00E-01	1.00E+00
ENSBTAG00000024895	0.00E+00	1.00E+00	0.00E+00	1.00E+00	-5.13E-15	1.00E+00	0.00E+00	1.00E+00	0.00E+00	1.00E+00
ENSBTAG00000045803	1.91E+00	8.23E-01	1.98E+00	4.43E-01	-5.13E-15	1.00E+00	1.76E+00	4.97E-01	1.91E+00	8.00E-01
ENSBTAG00000047236	1.91E+00	8.23E-01	0.00E+00	1.00E+00	-5.13E-15	1.00E+00	0.00E+00	1.00E+00	0.00E+00	1.00E+00
ENSBTAG00000000688	0.00E+00	1.00E+00	0.00E+00	1.00E+00	-5.13E-15	1.00E+00	0.00E+00	1.00E+00	0.00E+00	1.00E+00
ENSBTAG00000030344	0.00E+00	1.00E+00	1.98E+00	4.43E-01	1.78E+00	4.79E-01	0.00E+00	1.00E+00	0.00E+00	1.00E+00
ENSBTAG00000039628	0.00E+00	1.00E+00	1.98E+00	4.43E-01	1.78E+00	4.79E-01	0.00E+00	1.00E+00	0.00E+00	1.00E+00
ENSBTAG00000038022	0.00E+00	1.00E+00	0.00E+00	1.00E+00	-5.13E-15	1.00E+00	2.53E+00	2.71E-01	1.91E+00	8.00E-01
ENSBTAG00000000803	4.10E-01	1.00E+00	2.68E+00	2.64E-05	1.65E+00	1.16E-02	1.03E+00	1.69E-01	1.34E-02	1.00E+00
ENSBTAG00000000801	8.71E-02	1.00E+00	1.12E+00	1.25E-07	1.87E+00	1.72E-19	1.56E+00	7.37E-14	-9.18E-02	1.00E+00
ENSBTAG00000037881	-2.35E-01	1.00E+00	6.74E-01	4.67E-01	3.95E-01	8.07E-01	5.25E-01	6.30E-01	-3.86E-01	1.00E+00

ENSBTAG00000046947	4.00E-03	1.00E+00	-2.70E+00	2.42E-01	-2.70E+00	1.99E-01	-2.70E+00	2.05E-01	-7.86E-01	1.00E+00
ENSBTAG00000030348	-1.44E-01	1.00E+00	4.93E-01	1.84E-02	9.49E-01	5.96E-07	5.46E-01	8.62E-03	2.48E-02	1.00E+00
ENSBTAG00000046101	3.59E-02	1.00E+00	4.66E-01	1.64E-01	2.13E-01	6.78E-01	-1.12E-02	1.00E+00	-3.29E-02	1.00E+00
ENSBTAG00000002055	3.28E-01	7.15E-01	2.27E+00	1.98E-21	1.53E+00	1.44E-10	6.75E-01	1.07E-02	3.73E-01	6.01E-01
ENSBTAG00000025288	0.00E+00	1.00E+00	0.00E+00	1.00E+00	-5.13E-15	1.00E+00	0.00E+00	1.00E+00	0.00E+00	1.00E+00
ENSBTAG00000038911	1.92E+00	8.23E-01	1.99E+00	4.43E-01	-5.13E-15	1.00E+00	0.00E+00	1.00E+00	0.00E+00	1.00E+00
ENSBTAG00000011664	0.00E+00	1.00E+00	3.30E+00	1.02E-01	-5.13E-15	1.00E+00	1.77E+00	4.97E-01	0.00E+00	1.00E+00
ENSBTAG00000045731	0.00E+00	1.00E+00	0.00E+00	1.00E+00	-5.13E-15	1.00E+00	2.53E+00	2.71E-01	0.00E+00	1.00E+00
ENSBTAG00000038326	-8.81E-01	1.00E+00	-1.26E+00	5.02E-01	2.64E-01	1.00E+00	5.69E-01	9.30E-01	-2.88E-01	1.00E+00
ENSBTAG00000048005	5.18E-01	1.00E+00	6.03E-01	9.45E-01	1.02E+00	5.89E-01	3.28E-01	1.00E+00	1.18E+00	8.32E-01
ENSBTAG00000023338	1.96E-01	1.00E+00	-5.28E-01	3.09E-01	3.62E-02	1.00E+00	4.18E-01	4.58E-01	2.31E-01	1.00E+00
ENSBTAG00000047251	0.00E+00	1.00E+00	0.00E+00	1.00E+00	-5.13E-15	1.00E+00	0.00E+00	1.00E+00	0.00E+00	1.00E+00
ENSBTAG00000044457	0.00E+00	1.00E+00	0.00E+00	1.00E+00	-5.13E-15	1.00E+00	0.00E+00	1.00E+00	0.00E+00	1.00E+00
ENSBTAG00000003261	-9.81E-02	1.00E+00	2.20E-01	4.69E-01	6.76E-01	1.50E-03	-9.99E-02	9.42E-01	1.76E-01	9.46E-01
ENSBTAG00000038926	6.31E-01	1.41E-01	2.07E+00	3.92E-16	1.71E+00	1.71E-11	1.08E+00	5.30E-05	2.60E-01	8.68E-01
ENSBTAG00000017872	-1.37E-01	1.00E+00	1.25E+00	5.62E-03	1.26E+00	5.03E-03	4.62E-01	4.63E-01	-1.64E-01	1.00E+00
ENSBTAG00000037721	-2.70E-02	1.00E+00	2.00E+00	1.76E-05	1.18E+00	2.23E-02	-4.12E-01	6.82E-01	-1.59E-02	1.00E+00
ENSBTAG00000039341	1.10E-02	1.00E+00	1.21E+00	5.12E-05	3.99E-01	3.15E-01	-2.97E-01	5.43E-01	1.69E-01	1.00E+00
ENSBTAG00000037375	3.25E-01	8.96E-01	9.79E-01	5.62E-03	4.62E-01	2.86E-01	-2.68E-01	6.66E-01	-1.15E-01	1.00E+00
ENSBTAG00000011262	4.12E-01	8.23E-01	1.41E+00	1.01E-04	1.18E+00	1.33E-03	5.47E-01	2.42E-01	5.72E-01	6.01E-01
ENSBTAG00000000195	-5.59E-01	4.47E-01	6.02E-01	1.01E-01	8.98E-01	6.82E-03	3.17E-01	5.08E-01	-3.09E-01	8.73E-01
ENSBTAG00000040358	3.75E-01	8.23E-01	5.10E-01	2.02E-01	7.43E-01	3.23E-02	-7.21E-01	6.73E-02	2.63E-01	9.71E-01
ENSBTAG00000017613	0.00E+00	1.00E+00	0.00E+00	1.00E+00	-5.13E-15	1.00E+00	0.00E+00	1.00E+00	0.00E+00	1.00E+00
ENSBTAG00000009460	3.87E-01	8.25E-01	5.95E-01	1.78E-01	1.14E+00	2.58E-03	4.37E-01	4.01E-01	3.36E-01	8.97E-01
ENSBTAG00000037981	8.63E-02	1.00E+00	1.93E+00	5.99E-11	9.81E-01	1.67E-03	1.84E-01	8.39E-01	3.87E-01	7.72E-01
ENSBTAG00000048261	0.00E+00	1.00E+00	0.00E+00	1.00E+00	-5.13E-15	1.00E+00	0.00E+00	1.00E+00	0.00E+00	1.00E+00
ENSBTAG00000043770	0.00E+00	1.00E+00	0.00E+00	1.00E+00	-5.13E-15	1.00E+00	0.00E+00	1.00E+00	0.00E+00	1.00E+00
ENSBTAG00000038240	7.54E-02	1.00E+00	8.96E-01	1.57E-02	4.84E-01	2.75E-01	1.81E-01	9.03E-01	4.13E-01	8.00E-01
ENSBTAG00000046880	0.00E+00	1.00E+00	2.79E+00	2.10E-01	-5.13E-15	1.00E+00	0.00E+00	1.00E+00	0.00E+00	1.00E+00
ENSBTAG00000047568	6.47E-01	1.00E+00	1.80E+00	3.30E-02	1.24E+00	2.08E-01	-8.41E-01	6.71E-01	9.69E-01	8.00E-01
ENSBTAG00000038635	1.44E-01	1.00E+00	1.37E+00	7.26E-09	8.89E-01	2.72E-04	2.04E-01	6.29E-01	2.65E-01	8.00E-01
ENSBTAG00000040169	-4.92E-02	1.00E+00	4.23E-01	6.61E-01	1.14E+00	5.58E-02	4.98E-01	5.54E-01	2.73E-02	1.00E+00
ENSBTAG00000015866	-3.37E-03	1.00E+00	8.50E-01	1.09E-02	4.10E-01	3.29E-01	1.03E-01	1.00E+00	3.39E-01	8.04E-01
ENSBTAG00000038947	0.00E+00	1.00E+00	0.00E+00	1.00E+00	-5.13E-15	1.00E+00	0.00E+00	1.00E+00	0.00E+00	1.00E+00
ENSBTAG00000003447	6.13E-03	1.00E+00	8.66E-01	9.68E-05	3.02E-01	2.91E-01	-5.07E-01	4.75E-02	2.27E-01	8.33E-01

ENSBTAG00000045814	9.37E-01	9.44E-01	8.77E-02	1.00E+00	-6.66E-01	8.92E-01	-1.88E-01	1.00E+00	3.73E-01	1.00E+00
ENSBTAG00000038088	3.49E-01	8.03E-01	1.53E+00	4.31E-08	1.10E+00	1.15E-04	5.93E-01	7.30E-02	5.69E-01	3.80E-01
ENSBTAG00000038715	1.00E+00	7.69E-01	8.68E-01	4.69E-01	1.48E+00	8.23E-02	9.24E-01	4.25E-01	8.72E-01	8.00E-01
ENSBTAG00000013020	-9.17E-02	1.00E+00	1.24E+00	8.93E-03	1.93E+00	5.06E-06	1.29E+00	5.83E-03	-6.90E-02	1.00E+00
ENSBTAG00000040442	1.85E-01	1.00E+00	1.71E+00	2.39E-08	5.25E-01	1.58E-01	-2.18E-01	7.54E-01	-1.43E-01	1.00E+00
ENSBTAG00000039946	3.16E-01	1.00E+00	7.73E-01	1.23E-01	-2.63E-01	8.55E-01	-1.17E+00	4.53E-02	7.59E-01	4.62E-01
ENSBTAG00000043658	0.00E+00	1.00E+00	0.00E+00	1.00E+00	-5.13E-15	1.00E+00	0.00E+00	1.00E+00	0.00E+00	1.00E+00
ENSBTAG00000027787	-4.34E-03	1.00E+00	-4.28E-01	3.27E-01	-7.71E-01	3.24E-02	-7.54E-01	4.18E-02	1.33E-01	1.00E+00
ENSBTAG00000003456	-5.01E-02	1.00E+00	5.80E-01	2.08E-02	3.73E-01	1.89E-01	-2.13E-01	5.74E-01	4.64E-02	1.00E+00
ENSBTAG00000043433	0.00E+00	1.00E+00	0.00E+00	1.00E+00	-5.13E-15	1.00E+00	0.00E+00	1.00E+00	0.00E+00	1.00E+00
ENSBTAG00000003462	-3.52E-01	8.53E-01	-1.57E+00	2.66E-05	-1.18E-02	1.00E+00	-8.35E-02	1.00E+00	3.89E-01	8.00E-01
ENSBTAG00000003465	-9.67E-02	1.00E+00	1.30E+00	7.43E-10	1.53E+00	1.22E-13	6.86E-01	2.82E-03	8.63E-02	1.00E+00
ENSBTAG00000013353	-2.28E-01	8.23E-01	4.44E-01	3.95E-02	5.61E-01	6.17E-03	-1.79E-01	5.73E-01	-8.53E-02	1.00E+00
ENSBTAG00000044508	0.00E+00	1.00E+00	0.00E+00	1.00E+00	-5.13E-15	1.00E+00	0.00E+00	1.00E+00	0.00E+00	1.00E+00
ENSBTAG00000025023	-6.03E-02	1.00E+00	-5.95E-02	1.00E+00	-6.60E-03	1.00E+00	-1.61E-01	9.17E-01	1.93E-01	1.00E+00
ENSBTAG00000047405	1.18E-01	1.00E+00	-1.22E-02	1.00E+00	-2.79E-01	6.05E-01	-6.71E-01	7.92E-02	-1.20E-01	1.00E+00
ENSBTAG00000009735	2.23E-01	8.91E-01	1.05E-01	9.54E-01	-6.50E-01	7.26E-03	-7.87E-01	1.03E-03	-2.07E-01	9.05E-01
ENSBTAG00000015989	-2.65E-02	1.00E+00	1.69E-01	7.53E-01	1.69E-01	7.46E-01	4.89E-02	1.00E+00	-8.72E-02	1.00E+00
ENSBTAG00000037882	-9.04E-01	4.04E-02	-7.15E-01	3.92E-02	-2.61E-01	6.01E-01	-2.34E-01	6.80E-01	2.09E-02	1.00E+00
ENSBTAG00000003267	-7.18E-01	1.76E-01	3.22E-01	4.43E-01	4.82E-01	1.56E-01	4.21E-01	2.51E-01	-1.30E-01	1.00E+00
ENSBTAG00000039453	-3.08E-01	8.90E-01	2.33E+00	2.19E-16	2.12E+00	5.71E-14	1.35E+00	4.46E-06	6.45E-04	1.00E+00
ENSBTAG00000044536	0.00E+00	1.00E+00	0.00E+00	1.00E+00	-5.13E-15	1.00E+00	0.00E+00	1.00E+00	0.00E+00	1.00E+00
ENSBTAG00000038534	-1.43E+00	2.38E-01	1.55E+00	4.16E-03	-3.17E-01	8.76E-01	2.71E-01	9.32E-01	3.35E-01	1.00E+00
ENSBTAG00000015164	-3.69E-01	5.05E-01	-8.29E-01	2.96E-04	-1.86E+00	4.01E-17	-2.26E+00	5.09E-24	-1.47E-01	1.00E+00
ENSBTAG00000006422	-3.29E-01	4.64E-01	-2.39E-01	3.70E-01	1.46E-01	6.86E-01	1.50E-01	6.84E-01	-8.18E-02	1.00E+00
ENSBTAG00000013882	-3.47E-02	1.00E+00	-3.96E-02	1.00E+00	-2.99E-01	2.70E-01	-3.81E-01	1.37E-01	-2.08E-01	8.56E-01
ENSBTAG00000012744	-1.73E-01	1.00E+00	-1.61E-01	7.48E-01	7.29E-02	1.00E+00	3.47E-01	2.41E-01	-1.94E-01	9.48E-01
ENSBTAG00000037581	1.14E-01	1.00E+00	1.61E-01	7.02E-01	2.80E-01	3.25E-01	-2.46E-02	1.00E+00	3.26E-01	6.01E-01
ENSBTAG00000046987	-1.27E-01	1.00E+00	-7.36E-01	3.47E-02	-1.35E+00	3.02E-05	-2.20E+00	9.78E-11	-1.54E-01	1.00E+00
ENSBTAG00000037595	-1.73E-01	1.00E+00	-3.60E-01	3.83E-01	-2.77E-01	5.28E-01	-7.59E-01	2.14E-02	-8.83E-02	1.00E+00
ENSBTAG00000037903	1.92E+00	8.23E-01	0.00E+00	1.00E+00	-5.13E-15	1.00E+00	0.00E+00	1.00E+00	0.00E+00	1.00E+00
ENSBTAG00000048260	0.00E+00	1.00E+00	0.00E+00	1.00E+00	-5.13E-15	1.00E+00	0.00E+00	1.00E+00	0.00E+00	1.00E+00
ENSBTAG00000045560	0.00E+00	1.00E+00	0.00E+00	1.00E+00	-5.13E-15	1.00E+00	0.00E+00	1.00E+00	0.00E+00	1.00E+00
ENSBTAG00000042840	0.00E+00	1.00E+00	0.00E+00	1.00E+00	-5.13E-15	1.00E+00	3.39E+00	8.39E-02	0.00E+00	1.00E+00
ENSBTAG00000034681	-3.23E-01	1.00E+00	-2.22E-02	1.00E+00	-8.45E-01	3.22E-01	7.66E-01	3.77E-01	-2.22E-01	1.00E+00

ENSBTAG00000047948	0.00E+00	1.00E+00	0.00E+00	1.00E+00	-5.13E-15	1.00E+00	0.00E+00	1.00E+00	0.00E+00	1.00E+00
ENSBTAG00000010674	0.00E+00	1.00E+00	0.00E+00	1.00E+00	-5.13E-15	1.00E+00	1.76E+00	4.97E-01	0.00E+00	1.00E+00
ENSBTAG00000017345	0.00E+00	1.00E+00	0.00E+00	1.00E+00	1.78E+00	4.79E-01	0.00E+00	1.00E+00	0.00E+00	1.00E+00
ENSBTAG00000044618	0.00E+00	1.00E+00	0.00E+00	1.00E+00	-5.13E-15	1.00E+00	0.00E+00	1.00E+00	0.00E+00	1.00E+00
ENSBTAG00000036115	-1.97E+00	5.80E-01	-5.82E-01	8.93E-01	-4.59E-01	9.67E-01	4.30E-01	9.44E-01	-6.56E-01	1.00E+00
ENSBTAG00000017221	5.72E-02	1.00E+00	1.13E-01	9.95E-01	2.53E-01	5.47E-01	3.61E-01	3.23E-01	-1.28E-01	1.00E+00
ENSBTAG00000007013	9.03E-02	1.00E+00	-1.27E+00	6.31E-08	-2.21E+00	5.02E-21	-7.69E-01	2.01E-03	2.02E-01	9.34E-01
ENSBTAG00000007015	-2.83E-01	7.59E-01	-4.12E-01	1.14E-01	-2.64E-01	3.97E-01	-3.28E-01	2.54E-01	-1.98E-01	9.13E-01
ENSBTAG00000016078	-2.05E-01	1.00E+00	-5.36E-01	7.67E-02	-2.03E-01	6.67E-01	5.43E-02	1.00E+00	-9.65E-02	1.00E+00
ENSBTAG00000043089	0.00E+00	1.00E+00	0.00E+00	1.00E+00	-5.13E-15	1.00E+00	0.00E+00	1.00E+00	0.00E+00	1.00E+00
ENSBTAG00000007443	-1.26E-01	1.00E+00	-6.62E-01	3.56E-02	-1.13E+00	1.09E-04	-8.43E-01	5.50E-03	4.60E-01	5.16E-01
ENSBTAG00000006618	-5.72E-01	7.27E-01	-2.55E+00	3.66E-08	-2.57E+00	2.06E-08	-6.10E-01	2.86E-01	6.75E-01	5.99E-01
ENSBTAG00000000205	-3.77E-02	1.00E+00	-5.66E-02	1.00E+00	-5.66E-01	6.96E-02	-3.79E-01	2.93E-01	-3.61E-01	7.35E-01
ENSBTAG00000009292	-1.13E+00	9.74E-02	-3.66E+00	2.20E-12	-2.37E+00	4.02E-07	-1.02E+00	4.00E-02	-2.28E-01	1.00E+00
ENSBTAG00000042962	0.00E+00	1.00E+00	0.00E+00	1.00E+00	-5.13E-15	1.00E+00	0.00E+00	1.00E+00	0.00E+00	1.00E+00
ENSBTAG00000042227	0.00E+00	1.00E+00	0.00E+00	1.00E+00	-5.13E-15	1.00E+00	1.76E+00	4.97E-01	0.00E+00	1.00E+00
ENSBTAG00000038770	-4.28E-01	8.87E-01	-1.07E+00	3.78E-02	-1.32E+00	7.24E-03	-1.78E+00	4.01E-04	2.59E-01	1.00E+00
ENSBTAG00000040237	0.00E+00	1.00E+00	0.00E+00	1.00E+00	1.78E+00	4.79E-01	1.76E+00	4.97E-01	3.21E+00	4.34E-01
ENSBTAG00000039563	1.31E-01	1.00E+00	-1.41E+00	1.86E-01	2.25E-01	1.00E+00	-5.39E-02	1.00E+00	6.78E-01	8.13E-01
ENSBTAG00000047859	0.00E+00	1.00E+00	0.00E+00	1.00E+00	-5.13E-15	1.00E+00	0.00E+00	1.00E+00	0.00E+00	1.00E+00
ENSBTAG00000018706	-2.04E-01	9.66E-01	-1.38E+00	5.24E-09	-3.17E+00	6.02E-39	-3.34E+00	1.92E-42	-2.76E-01	8.00E-01
ENSBTAG00000035172	-3.57E-02	1.00E+00	-1.56E+00	2.25E-04	-1.73E+00	3.45E-05	-1.25E+00	4.00E-03	6.69E-01	5.12E-01
ENSBTAG00000047723	1.65E+00	3.62E-01	-1.59E+00	4.21E-01	-3.58E+00	4.81E-02	-3.58E+00	5.19E-02	2.16E+00	1.31E-01
ENSBTAG00000021292	1.79E-01	1.00E+00	-1.60E-01	1.00E+00	-1.53E+00	1.80E-01	9.47E-01	3.29E-01	-2.25E+00	3.10E-01
ENSBTAG00000044601	0.00E+00	1.00E+00	0.00E+00	1.00E+00	-5.13E-15	1.00E+00	0.00E+00	1.00E+00	0.00E+00	1.00E+00
ENSBTAG00000038823	1.26E-02	1.00E+00	-1.91E+00	4.69E-01	1.15E+00	6.44E-01	6.26E-01	9.94E-01	-1.91E+00	8.00E-01
ENSBTAG00000040282	4.16E-03	1.00E+00	-4.16E-01	1.00E+00	5.18E-01	9.34E-01	-1.44E+00	4.97E-01	4.03E-01	1.00E+00
ENSBTAG00000035147	1.42E-01	1.00E+00	-1.28E+00	3.78E-02	-1.35E+00	2.17E-02	-4.53E-01	5.67E-01	-6.51E-01	7.68E-01
ENSBTAG00000004449	-5.90E-01	3.68E-01	-9.60E-01	4.83E-03	-1.25E-01	9.94E-01	-6.59E-02	1.00E+00	2.96E-01	8.64E-01
ENSBTAG00000009948	4.69E-01	9.18E-01	1.05E+00	6.33E-02	2.20E-01	9.69E-01	3.42E-01	7.59E-01	6.54E-01	7.32E-01
ENSBTAG00000017275	-7.30E-02	1.00E+00	1.54E-01	6.83E-01	3.30E-01	1.70E-01	2.01E-01	5.04E-01	-1.83E-01	9.03E-01
ENSBTAG00000007893	-1.13E-01	1.00E+00	-7.54E-01	4.14E-05	-1.28E+00	6.36E-13	-1.14E+00	2.94E-10	5.31E-01	6.18E-02
ENSBTAG00000046559	0.00E+00	1.00E+00	0.00E+00	1.00E+00	-5.13E-15	1.00E+00	0.00E+00	1.00E+00	1.93E+00	8.00E-01
ENSBTAG00000025320	-1.17E-01	1.00E+00	8.33E-01	1.74E-03	8.54E-01	1.24E-03	1.49E-01	8.52E-01	-7.56E-02	1.00E+00
ENSBTAG00000004406	2.00E-01	1.00E+00	5.34E-01	2.44E-01	-3.09E-01	6.35E-01	7.08E-01	9.04E-02	2.89E-01	9.85E-01

ENSBTAG00000048085	0.00E+00	1.00E+00	0.00E+00	1.00E+00	-5.13E-15	1.00E+00	0.00E+00	1.00E+00	0.00E+00	1.00E+00
ENSBTAG00000047296	0.00E+00	1.00E+00	0.00E+00	1.00E+00	-5.13E-15	1.00E+00	0.00E+00	1.00E+00	0.00E+00	1.00E+00
ENSBTAG00000047272	0.00E+00	1.00E+00	0.00E+00	1.00E+00	-5.13E-15	1.00E+00	0.00E+00	1.00E+00	0.00E+00	1.00E+00
ENSBTAG00000045351	0.00E+00	1.00E+00	0.00E+00	1.00E+00	-5.13E-15	1.00E+00	0.00E+00	1.00E+00	0.00E+00	1.00E+00
ENSBTAG00000012366	2.19E-02	1.00E+00	-7.43E-02	1.00E+00	3.67E-02	1.00E+00	5.86E-02	1.00E+00	-2.70E-01	7.79E-01
ENSBTAG00000007147	-4.61E-01	4.45E-01	-1.22E+00	1.10E-05	-1.30E+00	1.88E-06	-1.14E+00	3.58E-05	-7.40E-01	9.53E-02
ENSBTAG00000004193	1.26E-01	1.00E+00	-1.92E-02	1.00E+00	3.20E-01	1.67E-01	-5.36E-02	1.00E+00	-9.14E-02	1.00E+00
ENSBTAG00000014766	9.96E-03	1.00E+00	8.51E-01	7.42E-08	7.46E-01	2.69E-06	3.84E-01	3.12E-02	-1.39E-02	1.00E+00
ENSBTAG00000025313	-3.20E-01	5.72E-01	-1.05E+00	2.96E-07	-1.80E+00	3.90E-19	-8.48E-01	4.97E-05	-2.21E-01	8.00E-01
ENSBTAG00000035001	1.92E+00	8.23E-01	0.00E+00	1.00E+00	1.78E+00	4.79E-01	3.02E+00	1.50E-01	2.71E+00	6.01E-01
ENSBTAG00000037424	0.00E+00	1.00E+00	0.00E+00	1.00E+00	-5.13E-15	1.00E+00	0.00E+00	1.00E+00	1.91E+00	8.00E-01
ENSBTAG00000047870	0.00E+00	1.00E+00	1.99E+00	4.43E-01	-5.13E-15	1.00E+00	0.00E+00	1.00E+00	1.93E+00	8.00E-01
ENSBTAG00000045606	0.00E+00	1.00E+00	0.00E+00	1.00E+00	-5.13E-15	1.00E+00	0.00E+00	1.00E+00	1.91E+00	8.00E-01
ENSBTAG00000047689	1.91E+00	8.23E-01	0.00E+00	1.00E+00	1.78E+00	4.79E-01	1.76E+00	4.97E-01	2.71E+00	6.01E-01
ENSBTAG00000045769	0.00E+00	1.00E+00	0.00E+00	1.00E+00	-5.13E-15	1.00E+00	0.00E+00	1.00E+00	0.00E+00	1.00E+00
ENSBTAG00000047770	-1.91E+00	8.23E-01	-1.91E+00	4.69E-01	-1.91E+00	4.28E-01	-1.91E+00	4.39E-01	-1.91E+00	8.00E-01
ENSBTAG00000045921	-1.91E+00	8.23E-01	-1.91E+00	4.69E-01	-1.91E+00	4.28E-01	-1.91E+00	4.39E-01	7.16E-03	1.00E+00
ENSBTAG00000034963	-2.92E-01	8.23E-01	-2.02E+00	1.82E-13	-1.23E+00	3.80E-06	-1.03E+00	1.65E-04	1.10E-02	1.00E+00
ENSBTAG00000012780	-1.22E-01	1.00E+00	-1.04E+00	1.21E-01	-2.13E+00	8.74E-04	-6.18E-01	4.25E-01	8.81E-01	4.46E-01
ENSBTAG00000043244	0.00E+00	1.00E+00	0.00E+00	1.00E+00	-5.13E-15	1.00E+00	0.00E+00	1.00E+00	0.00E+00	1.00E+00
ENSBTAG00000011616	-1.14E+00	2.68E-01	-1.25E+00	4.67E-02	-2.60E+00	3.27E-05	-1.76E+00	3.95E-03	-3.12E-01	1.00E+00
ENSBTAG00000043039	0.00E+00	1.00E+00	0.00E+00	1.00E+00	-5.13E-15	1.00E+00	0.00E+00	1.00E+00	0.00E+00	1.00E+00
ENSBTAG00000046321	-2.27E-01	1.00E+00	-9.06E-01	3.77E-03	-9.46E-01	1.98E-03	-1.08E+00	4.24E-04	3.58E-01	7.66E-01
ENSBTAG00000029982	2.22E-01	1.00E+00	-9.70E-02	1.00E+00	1.91E-01	1.00E+00	-1.89E-01	1.00E+00	3.36E-01	1.00E+00
ENSBTAG00000003386	-2.62E-01	7.86E-01	-4.53E-02	1.00E+00	7.48E-01	5.50E-04	1.05E+00	6.84E-07	-2.61E-01	7.91E-01
ENSBTAG00000020119	-1.73E+00	3.28E-09	-4.94E+00	7.47E-54	-6.37E+00	9.31E-74	-3.36E+00	2.08E-31	-4.20E-01	6.01E-01
ENSBTAG00000001166	2.84E-01	1.00E+00	-1.10E-02	1.00E+00	-1.96E+00	1.54E-05	-2.12E+00	4.38E-06	-2.38E-01	1.00E+00
ENSBTAG00000015352	1.06E-01	1.00E+00	2.44E-02	1.00E+00	-7.61E-01	4.07E-03	-2.02E-01	6.67E-01	6.36E-01	1.55E-01
ENSBTAG00000015368	1.97E-02	1.00E+00	-1.56E+00	7.70E-07	-1.33E+00	2.15E-05	-1.08E+00	8.20E-04	-7.81E-03	1.00E+00
ENSBTAG00000048278	1.26E-02	1.00E+00	7.57E-02	1.00E+00	-1.31E-01	1.00E+00	-1.48E-01	1.00E+00	-1.91E+00	8.00E-01
ENSBTAG00000010167	6.55E-01	4.03E-01	1.01E+00	7.72E-03	7.28E-01	7.43E-02	2.36E-01	8.06E-01	7.74E-01	3.09E-01
ENSBTAG00000028359	0.00E+00	1.00E+00	0.00E+00	1.00E+00	-5.13E-15	1.00E+00	0.00E+00	1.00E+00	0.00E+00	1.00E+00
ENSBTAG00000036927	0.00E+00	1.00E+00	0.00E+00	1.00E+00	-5.13E-15	1.00E+00	0.00E+00	1.00E+00	0.00E+00	1.00E+00
ENSBTAG00000047470	0.00E+00	1.00E+00	0.00E+00	1.00E+00	-5.13E-15	1.00E+00	0.00E+00	1.00E+00	0.00E+00	1.00E+00
ENSBTAG00000046246	0.00E+00	1.00E+00	0.00E+00	1.00E+00	-5.13E-15	1.00E+00	0.00E+00	1.00E+00	0.00E+00	1.00E+00

ENSBTAG00000028696	0.00E+00	1.00E+00	0.00E+00	1.00E+00	-5.13E-15	1.00E+00	0.00E+00	1.00E+00	0.00E+00	1.00E+00
ENSBTAG00000046532	0.00E+00	1.00E+00	0.00E+00	1.00E+00	-5.13E-15	1.00E+00	0.00E+00	1.00E+00	0.00E+00	1.00E+00
ENSBTAG00000034770	0.00E+00	1.00E+00	0.00E+00	1.00E+00	-5.13E-15	1.00E+00	0.00E+00	1.00E+00	0.00E+00	1.00E+00
ENSBTAG00000034769	0.00E+00	1.00E+00	0.00E+00	1.00E+00	-5.13E-15	1.00E+00	0.00E+00	1.00E+00	0.00E+00	1.00E+00
ENSBTAG00000003280	1.98E-01	1.00E+00	3.97E-01	7.10E-01	5.64E-02	1.00E+00	-4.97E-01	6.07E-01	1.11E+00	2.72E-01
ENSBTAG00000034768	0.00E+00	1.00E+00	1.98E+00	4.43E-01	1.78E+00	4.79E-01	0.00E+00	1.00E+00	0.00E+00	1.00E+00
ENSBTAG00000036997	0.00E+00	1.00E+00	1.99E+00	4.43E-01	-5.13E-15	1.00E+00	0.00E+00	1.00E+00	0.00E+00	1.00E+00
ENSBTAG00000034767	3.23E+00	3.75E-01	0.00E+00	1.00E+00	3.43E+00	7.39E-02	2.53E+00	2.71E-01	1.91E+00	8.00E-01
ENSBTAG00000034766	-4.84E+00	9.80E-03	-1.16E+00	3.32E-01	-2.29E+00	3.88E-02	-4.84E+00	8.75E-04	-4.84E+00	2.28E-02
ENSBTAG00000015036	-1.16E-03	1.00E+00	4.32E-02	1.00E+00	2.51E-01	6.57E-01	1.89E-01	8.35E-01	-1.39E-01	1.00E+00
ENSBTAG00000046283	0.00E+00	1.00E+00	0.00E+00	1.00E+00	-5.13E-15	1.00E+00	0.00E+00	1.00E+00	0.00E+00	1.00E+00
ENSBTAG00000043781	0.00E+00	1.00E+00	0.00E+00	1.00E+00	-5.13E-15	1.00E+00	0.00E+00	1.00E+00	0.00E+00	1.00E+00
ENSBTAG00000042415	0.00E+00	1.00E+00	1.98E+00	4.43E-01	-5.13E-15	1.00E+00	0.00E+00	1.00E+00	0.00E+00	1.00E+00
ENSBTAG00000010788	-7.17E-01	9.80E-01	7.65E-01	4.60E-01	-1.86E-01	1.00E+00	-1.90E-01	1.00E+00	-1.25E+00	6.91E-01
ENSBTAG00000021677	-2.65E-01	1.00E+00	-1.39E+00	3.30E-04	7.02E-01	9.33E-02	8.08E-01	4.97E-02	-3.67E-01	8.44E-01
ENSBTAG00000025310	0.00E+00	1.00E+00	0.00E+00	1.00E+00	-5.13E-15	1.00E+00	0.00E+00	1.00E+00	0.00E+00	1.00E+00
ENSBTAG00000021680	-4.11E-01	5.76E-01	-5.71E-01	5.76E-02	-4.13E-01	2.03E-01	-3.95E-01	2.43E-01	-4.57E-01	5.02E-01
ENSBTAG00000030066	0.00E+00	1.00E+00	0.00E+00	1.00E+00	-5.13E-15	1.00E+00	0.00E+00	1.00E+00	0.00E+00	1.00E+00
ENSBTAG00000029787	0.00E+00	1.00E+00	0.00E+00	1.00E+00	-5.13E-15	1.00E+00	0.00E+00	1.00E+00	0.00E+00	1.00E+00
ENSBTAG00000021681	1.87E-01	1.00E+00	1.04E-01	1.00E+00	-3.38E-01	9.90E-01	7.30E-02	1.00E+00	2.95E-02	1.00E+00
ENSBTAG00000003365	4.55E-01	4.07E-01	1.76E+00	7.19E-13	1.09E+00	1.45E-05	7.21E-01	7.89E-03	1.80E-01	1.00E+00
ENSBTAG00000007176	1.81E-01	1.00E+00	-1.59E-01	9.90E-01	-1.67E+00	1.94E-05	-1.99E+00	4.76E-07	3.20E-01	9.40E-01
ENSBTAG00000019806	4.78E-01	7.37E-01	1.19E-01	1.00E+00	-1.34E+00	4.88E-04	-1.13E+00	4.46E-03	6.73E-01	4.35E-01
ENSBTAG00000020774	1.32E-01	1.00E+00	-5.48E-01	5.68E-01	-6.21E-01	4.67E-01	-4.46E-02	1.00E+00	1.08E+00	2.76E-01
ENSBTAG00000017868	-1.28E-01	1.00E+00	-9.54E-01	2.05E-05	-7.33E-01	1.28E-03	-3.94E-01	1.38E-01	1.95E-01	9.13E-01
ENSBTAG00000016708	1.73E-01	1.00E+00	1.22E+00	1.42E-07	1.22E+00	1.33E-07	1.29E+00	3.22E-08	3.90E-02	1.00E+00
ENSBTAG00000016710	3.55E-01	5.89E-01	2.03E+00	1.44E-18	2.10E+00	4.59E-20	1.26E+00	8.26E-08	-1.30E-01	1.00E+00
ENSBTAG00000011623	1.04E+00	7.01E-07	2.30E+00	3.93E-33	2.56E+00	1.95E-40	2.15E+00	2.24E-29	-1.76E-03	1.00E+00
ENSBTAG00000029897	2.51E+00	1.51E-09	4.04E+00	8.63E-25	3.37E+00	3.75E-18	2.61E+00	3.03E-11	-4.24E-01	8.96E-01
ENSBTAG00000001290	1.87E-01	1.00E+00	5.76E-01	1.00E-01	1.11E-01	1.00E+00	-6.17E-02	1.00E+00	3.44E-01	8.00E-01
ENSBTAG00000016851	3.00E-01	6.68E-01	1.12E+00	1.55E-07	9.38E-01	1.27E-05	6.15E-01	7.88E-03	9.55E-02	1.00E+00
ENSBTAG00000044394	0.00E+00	1.00E+00	0.00E+00	1.00E+00	-5.13E-15	1.00E+00	0.00E+00	1.00E+00	0.00E+00	1.00E+00
ENSBTAG00000009747	-2.55E-04	1.00E+00	3.37E-01	3.13E-01	8.16E-01	1.80E-03	9.79E-01	1.67E-04	-5.29E-02	1.00E+00
ENSBTAG00000011721	5.50E-01	3.37E-01	6.30E-01	4.76E-02	8.47E-01	4.66E-03	1.08E+00	2.60E-04	3.98E-01	6.75E-01
ENSBTAG00000012070	2.66E-01	9.62E-01	1.74E+00	7.90E-09	9.07E-01	4.31E-03	1.08E+00	6.36E-04	-2.73E-01	9.19E-01

ENSBTAG00000047313	0.00E+00	1.00E+00	0.00E+00	1.00E+00	-5.13E-15	1.00E+00	1.76E+00	4.97E-01	0.00E+00	1.00E+00
ENSBTAG00000012068	4.15E-02	1.00E+00	2.38E+00	5.44E-11	1.33E+00	4.96E-04	7.11E-01	1.16E-01	-4.77E-01	8.00E-01
ENSBTAG00000047777	0.00E+00	1.00E+00	0.00E+00	1.00E+00	-5.13E-15	1.00E+00	0.00E+00	1.00E+00	0.00E+00	1.00E+00
ENSBTAG00000009968	1.03E-02	1.00E+00	1.52E+00	2.99E-01	3.44E-01	1.00E+00	1.23E+00	4.56E-01	5.08E-01	1.00E+00
ENSBTAG00000014278	-1.88E+00	1.08E-07	-2.32E+00	7.67E-12	-2.00E-01	7.82E-01	-7.72E-01	2.52E-02	-3.11E-02	1.00E+00
ENSBTAG00000034506	1.34E-01	1.00E+00	1.08E+00	1.53E-07	1.54E+00	2.56E-14	1.57E+00	1.01E-14	-3.73E-01	4.81E-01
ENSBTAG00000002663	-2.47E-01	9.00E-01	-8.68E-01	1.42E-03	-5.14E-01	7.97E-02	5.18E-02	1.00E+00	1.44E-01	1.00E+00
ENSBTAG00000033841	0.00E+00	1.00E+00	0.00E+00	1.00E+00	-5.13E-15	1.00E+00	0.00E+00	1.00E+00	0.00E+00	1.00E+00
ENSBTAG00000045964	0.00E+00	1.00E+00	0.00E+00	1.00E+00	-5.13E-15	1.00E+00	0.00E+00	1.00E+00	0.00E+00	1.00E+00
ENSBTAG00000043437	0.00E+00	1.00E+00	0.00E+00	1.00E+00	-5.13E-15	1.00E+00	0.00E+00	1.00E+00	0.00E+00	1.00E+00
ENSBTAG00000009427	3.06E-01	8.23E-01	9.94E-01	1.77E-04	7.57E-01	5.70E-03	5.71E-01	5.47E-02	2.89E-01	8.00E-01
ENSBTAG00000046974	0.00E+00	1.00E+00	0.00E+00	1.00E+00	-5.13E-15	1.00E+00	0.00E+00	1.00E+00	0.00E+00	1.00E+00
ENSBTAG00000004489	8.45E-01	2.26E-02	1.78E+00	7.41E-11	1.54E+00	1.52E-08	1.63E+00	2.93E-09	7.84E-02	1.00E+00
ENSBTAG00000018391	8.02E-01	1.00E+00	-1.91E+00	4.69E-01	6.44E-01	9.78E-01	6.22E-01	9.94E-01	-1.91E+00	8.00E-01
ENSBTAG00000034433	-1.01E-01	1.00E+00	-8.90E-02	1.00E+00	-2.57E-01	9.53E-01	-9.70E-02	1.00E+00	5.50E-02	1.00E+00
ENSBTAG00000043422	0.00E+00	1.00E+00	0.00E+00	1.00E+00	-5.13E-15	1.00E+00	0.00E+00	1.00E+00	0.00E+00	1.00E+00
ENSBTAG00000020089	2.41E-01	9.52E-01	1.41E+00	1.93E-07	1.54E+00	9.93E-09	1.03E+00	2.46E-04	2.94E-01	8.00E-01
ENSBTAG00000017969	-6.72E-01	8.64E-01	-1.28E+00	1.01E-01	-1.32E+00	8.74E-02	-1.83E+00	1.33E-02	-6.11E-01	8.98E-01
ENSBTAG00000012672	2.86E-01	6.72E-01	7.64E-01	2.71E-04	9.24E-01	6.27E-06	2.50E-01	3.95E-01	1.19E-01	1.00E+00
ENSBTAG00000012675	-5.85E-02	1.00E+00	4.55E-01	7.59E-02	6.23E-01	8.91E-03	3.47E-01	2.24E-01	-1.57E-01	1.00E+00
ENSBTAG00000043093	0.00E+00	1.00E+00	0.00E+00	1.00E+00	-5.13E-15	1.00E+00	0.00E+00	1.00E+00	0.00E+00	1.00E+00
ENSBTAG00000034396	2.04E-01	8.53E-01	8.59E-01	1.35E-05	1.29E+00	1.73E-11	8.48E-01	2.09E-05	-2.78E-01	6.72E-01
ENSBTAG00000010290	-9.60E-02	1.00E+00	-8.24E-01	4.33E-06	-2.32E-01	3.26E-01	-1.68E-01	5.52E-01	6.45E-02	1.00E+00
ENSBTAG00000010297	-3.23E-01	6.25E-01	-5.01E-01	4.48E-02	-2.45E-01	4.46E-01	-3.32E-01	2.54E-01	9.25E-02	1.00E+00
ENSBTAG00000010311	-6.56E-01	2.42E-01	-1.48E+00	4.37E-06	-6.73E-01	5.35E-02	-5.89E-01	1.10E-01	-3.65E-01	8.00E-01
ENSBTAG00000000560	-1.82E-03	1.00E+00	2.93E-01	2.46E-01	4.71E-01	3.15E-02	6.12E-01	4.02E-03	-7.10E-01	1.10E-02
ENSBTAG00000033562	-4.13E+00	8.59E-02	-8.29E-01	6.63E-01	-1.58E+00	2.63E-01	-1.10E+00	4.79E-01	-1.42E+00	7.65E-01
ENSBTAG00000021927	-3.97E-02	1.00E+00	2.18E-01	4.54E-01	5.90E-01	5.02E-03	6.15E-01	3.83E-03	1.40E-01	1.00E+00
ENSBTAG00000047820	0.00E+00	1.00E+00	0.00E+00	1.00E+00	-5.13E-15	1.00E+00	0.00E+00	1.00E+00	0.00E+00	1.00E+00
ENSBTAG00000017567	6.34E-01	6.95E-01	-1.18E+00	2.09E-02	-1.12E+00	3.04E-02	-8.42E-01	1.38E-01	-5.91E-01	7.69E-01
ENSBTAG00000043388	0.00E+00	1.00E+00	0.00E+00	1.00E+00	-5.13E-15	1.00E+00	0.00E+00	1.00E+00	0.00E+00	1.00E+00
ENSBTAG00000025242	4.61E-01	1.00E+00	-1.03E+00	2.54E-01	-2.41E+00	4.50E-03	-2.43E+00	4.82E-03	2.23E-01	1.00E+00
ENSBTAG00000014677	-1.15E-01	1.00E+00	5.00E-01	2.87E-02	9.62E-01	3.39E-06	2.51E-01	4.03E-01	-3.42E-01	5.62E-01
ENSBTAG00000010279	-3.30E-01	7.65E-01	-6.52E-01	2.50E-02	-7.31E-01	9.16E-03	-3.46E-01	3.21E-01	2.55E-01	8.44E-01
ENSBTAG00000010292	1.90E-01	1.00E+00	5.42E-01	4.31E-02	7.79E-01	1.78E-03	6.92E-01	7.59E-03	3.30E-01	6.96E-01

ENSBTAG00000010313	-1.14E-03	1.00E+00	8.95E-01	1.60E-05	1.13E+00	2.33E-08	9.58E-01	3.98E-06	-2.46E-01	8.00E-01
ENSBTAG00000004710	-6.58E-01	7.06E-01	-9.46E-01	1.02E-01	-6.70E-01	2.90E-01	-9.06E-01	1.24E-01	-3.04E-01	1.00E+00
ENSBTAG00000009635	-1.14E-01	1.00E+00	5.46E-01	5.56E-03	4.83E-01	1.61E-02	1.24E-01	7.85E-01	1.68E-01	9.12E-01
ENSBTAG00000025258	8.68E-01	8.23E-01	2.20E+00	7.91E-03	2.84E+00	5.13E-04	2.54E+00	2.33E-03	2.20E+00	7.92E-02
ENSBTAG00000046975	4.35E+00	2.58E-16	4.92E+00	8.96E-21	4.34E+00	2.45E-17	2.44E+00	7.75E-07	2.72E-01	1.00E+00
ENSBTAG00000046944	1.80E+00	6.83E-08	2.93E+00	6.00E-21	2.37E+00	1.23E-14	2.10E+00	1.37E-11	1.85E+00	1.39E-07
ENSBTAG00000025260	6.44E-01	8.78E-02	1.46E+00	2.09E-09	-6.77E-02	1.00E+00	-5.16E-01	8.56E-02	2.15E-02	1.00E+00
ENSBTAG00000039504	6.10E-01	1.00E+00	3.96E-02	1.00E+00	-4.40E+00	6.22E-03	1.19E-01	1.00E+00	-7.36E-02	1.00E+00
ENSBTAG00000025257	4.26E+00	2.00E-09	2.09E+00	1.30E-03	4.42E-01	7.19E-01	4.15E-01	7.69E-01	-6.04E-01	8.60E-01
ENSBTAG00000025250	3.78E+00	1.08E-10	2.26E+00	3.97E-05	8.80E-01	1.84E-01	1.24E+00	4.37E-02	-4.10E-01	1.00E+00
ENSBTAG00000003289	2.80E-01	1.00E+00	5.21E-01	2.33E-01	3.01E-02	1.00E+00	-3.88E-01	4.49E-01	-3.07E-01	9.19E-01
ENSBTAG00000010155	-2.12E-01	1.00E+00	-8.61E-01	5.49E-02	-1.24E+00	3.01E-03	-1.43E+00	6.88E-04	-3.91E-01	8.57E-01
ENSBTAG00000010738	-6.12E-01	1.75E-01	-1.28E+00	3.39E-06	-1.13E+00	3.94E-05	-1.07E+00	1.24E-04	-7.73E-01	7.31E-02
ENSBTAG00000007193	-4.31E-01	3.99E-01	-9.08E-01	2.08E-04	-2.01E+00	2.51E-17	-2.71E+00	4.50E-29	-1.24E-01	1.00E+00
ENSBTAG00000007191	-7.52E-02	1.00E+00	-5.61E-01	3.20E-01	-8.29E-01	9.07E-02	-7.61E-01	1.39E-01	-9.67E-01	2.49E-01
ENSBTAG00000002850	1.49E+00	1.25E-02	3.37E+00	7.41E-18	2.14E+00	5.68E-07	7.84E-01	2.00E-01	1.03E+00	3.09E-01
ENSBTAG00000006916	1.36E-01	1.00E+00	5.78E-01	4.59E-03	9.01E-01	3.29E-06	4.84E-01	2.45E-02	9.47E-02	1.00E+00
ENSBTAG00000006086	-3.16E-01	1.00E+00	-1.36E+00	3.68E-02	-9.90E-01	1.28E-01	-1.03E+00	1.19E-01	-9.40E-01	5.24E-01
ENSBTAG00000045621	-2.17E-01	1.00E+00	-2.69E+00	3.77E-04	-2.95E+00	5.76E-05	-2.68E+00	1.90E-04	3.34E-01	1.00E+00
ENSBTAG00000018519	0.00E+00	1.00E+00	3.30E+00	1.02E-01	3.06E+00	1.36E-01	1.76E+00	4.97E-01	0.00E+00	1.00E+00
ENSBTAG00000018518	-5.61E-01	2.33E-01	-1.50E+00	4.17E-08	-2.09E+00	2.69E-14	-1.48E+00	5.29E-08	1.27E-01	1.00E+00
ENSBTAG00000020316	2.31E-01	1.00E+00	2.58E-01	6.18E-01	6.65E-02	1.00E+00	4.68E-01	2.23E-01	3.50E-01	8.00E-01
ENSBTAG00000019723	-3.30E-01	7.14E-01	9.41E-01	1.13E-04	4.08E-01	1.61E-01	2.41E-01	5.14E-01	-8.00E-02	1.00E+00
ENSBTAG00000042305	1.41E-02	1.00E+00	1.46E+00	6.00E-02	1.95E+00	3.39E-03	6.71E-01	5.73E-01	-1.57E-01	1.00E+00
ENSBTAG00000039206	6.50E-01	9.58E-01	-2.31E-01	1.00E+00	-1.80E-01	1.00E+00	-6.94E-01	6.31E-01	1.30E+00	4.39E-01
ENSBTAG00000019437	4.59E-02	1.00E+00	3.69E-01	9.07E-01	-5.96E-02	1.00E+00	-4.02E-01	8.70E-01	2.60E-01	1.00E+00
ENSBTAG00000025219	4.88E-01	8.54E-01	9.94E-01	6.36E-02	7.22E-01	2.20E-01	2.40E-01	9.18E-01	2.81E-01	1.00E+00
ENSBTAG00000028421	2.50E-01	1.00E+00	4.75E-01	3.34E-01	3.87E-01	4.66E-01	-1.81E-01	9.35E-01	2.15E-01	1.00E+00
ENSBTAG00000044968	0.00E+00	1.00E+00	1.99E+00	4.43E-01	-5.13E-15	1.00E+00	0.00E+00	1.00E+00	1.91E+00	8.00E-01
ENSBTAG00000002898	-2.29E-01	1.00E+00	3.19E-01	1.00E+00	4.03E-01	9.38E-01	-1.88E-01	1.00E+00	2.63E-02	1.00E+00
ENSBTAG00000019094	-8.31E-02	1.00E+00	1.35E+00	5.54E-06	1.67E+00	1.23E-08	9.73E-01	1.82E-03	-2.78E-01	8.99E-01
ENSBTAG00000019092	-4.94E-01	1.00E+00	1.86E+00	7.68E-02	1.82E+00	7.36E-02	-1.82E-01	1.00E+00	1.19E-02	1.00E+00
ENSBTAG00000019082	-2.39E-01	1.00E+00	7.25E-01	1.22E-01	1.86E-01	9.48E-01	-7.02E-01	1.50E-01	9.86E-03	1.00E+00
ENSBTAG00000045174	0.00E+00	1.00E+00	0.00E+00	1.00E+00	-5.13E-15	1.00E+00	0.00E+00	1.00E+00	0.00E+00	1.00E+00
ENSBTAG00000013645	-3.05E-01	8.84E-01	2.11E-01	7.58E-01	6.17E-03	1.00E+00	-2.76E-02	1.00E+00	7.80E-01	1.51E-01

ENSBTAG00000018689	-1.84E-01	9.72E-01	7.80E-02	1.00E+00	9.39E-01	8.08E-06	9.31E-01	1.29E-05	6.96E-02	1.00E+00
ENSBTAG00000020340	1.24E-01	1.00E+00	8.10E-01	1.11E-04	8.92E-01	1.49E-05	2.62E-01	3.66E-01	1.45E-01	1.00E+00
ENSBTAG00000020338	-2.18E-01	1.00E+00	-1.67E+00	3.06E-06	-1.93E+00	3.96E-08	-1.41E+00	6.08E-05	-9.87E-02	1.00E+00
ENSBTAG00000044530	0.00E+00	1.00E+00	0.00E+00	1.00E+00	-5.13E-15	1.00E+00	0.00E+00	1.00E+00	0.00E+00	1.00E+00
ENSBTAG00000002680	3.36E-01	1.00E+00	6.56E-01	4.07E-01	1.59E+00	3.88E-03	2.06E+00	1.12E-04	5.66E-01	8.40E-01
ENSBTAG00000008832	2.18E-01	1.00E+00	-3.92E-01	7.49E-01	1.21E+00	1.34E-02	2.96E-01	8.44E-01	5.70E-01	8.00E-01
ENSBTAG00000014113	5.53E+00	1.99E-06	4.01E+00	9.96E-05	2.31E+00	3.03E-02	2.12E+00	5.61E-02	8.17E-01	9.49E-01
ENSBTAG00000004129	0.00E+00	1.00E+00	0.00E+00	1.00E+00	1.78E+00	4.79E-01	3.03E+00	1.50E-01	0.00E+00	1.00E+00
ENSBTAG00000037811	6.12E+00	1.35E-08	5.72E+00	9.13E-09	2.90E+00	1.76E-03	5.30E+00	7.91E-08	-6.32E-01	1.00E+00
ENSBTAG00000042700	0.00E+00	1.00E+00	2.79E+00	2.10E-01	3.72E+00	4.07E-02	3.41E+00	8.26E-02	0.00E+00	1.00E+00
ENSBTAG00000025200	-3.05E-01	1.00E+00	5.03E-02	1.00E+00	-3.29E-01	6.88E-01	5.09E-01	3.99E-01	-8.37E-01	4.34E-01
ENSBTAG00000044815	0.00E+00	1.00E+00	0.00E+00	1.00E+00	-5.13E-15	1.00E+00	0.00E+00	1.00E+00	0.00E+00	1.00E+00
ENSBTAG00000043750	0.00E+00	1.00E+00	0.00E+00	1.00E+00	-5.13E-15	1.00E+00	0.00E+00	1.00E+00	0.00E+00	1.00E+00
ENSBTAG00000043123	0.00E+00	1.00E+00	0.00E+00	1.00E+00	-5.13E-15	1.00E+00	0.00E+00	1.00E+00	0.00E+00	1.00E+00
ENSBTAG00000000344	9.86E-01	7.60E-01	4.15E-03	1.00E+00	-1.79E-01	1.00E+00	-1.87E+00	9.19E-02	1.67E+00	2.80E-01
ENSBTAG00000008913	-3.34E-01	9.27E-01	-1.16E+00	1.97E-03	-1.48E+00	5.39E-05	-1.18E+00	1.99E-03	-1.86E-02	1.00E+00
ENSBTAG00000015527	-4.68E-02	1.00E+00	-6.06E-01	3.48E-02	-1.07E+00	5.35E-05	-6.57E-01	2.20E-02	1.43E-01	1.00E+00
ENSBTAG00000042351	0.00E+00	1.00E+00	0.00E+00	1.00E+00	-5.13E-15	1.00E+00	0.00E+00	1.00E+00	0.00E+00	1.00E+00
ENSBTAG00000004475	6.32E-01	8.23E-01	2.75E+00	2.68E-08	1.44E+00	9.77E-03	5.56E-01	5.12E-01	5.54E-01	8.62E-01
ENSBTAG00000018897	1.62E-01	1.00E+00	8.89E-01	3.64E-04	1.31E+00	5.65E-08	1.08E+00	1.30E-05	-4.49E-02	1.00E+00
ENSBTAG00000008883	4.42E-01	4.03E-01	1.55E+00	1.80E-10	4.78E-01	9.21E-02	8.33E-01	1.23E-03	2.46E-01	8.44E-01
ENSBTAG00000020994	-4.05E-01	5.75E-01	-1.59E+00	2.12E-09	-1.09E+00	3.85E-05	-8.57E-01	1.83E-03	-7.26E-02	1.00E+00
ENSBTAG00000020992	-8.21E-02	1.00E+00	-2.35E+00	1.03E-02	-8.59E-01	3.38E-01	-2.06E-01	1.00E+00	-2.59E-01	1.00E+00
ENSBTAG00000010001	1.82E-01	9.43E-01	-3.95E-01	9.58E-02	3.60E-01	1.33E-01	1.25E-01	8.19E-01	-4.96E-01	1.92E-01
ENSBTAG00000017225	-3.44E-01	4.04E-01	-6.84E-01	5.44E-04	-6.94E-01	4.16E-04	-7.69E-01	9.64E-05	1.07E-01	1.00E+00
ENSBTAG00000003334	9.14E-01	1.22E-01	-2.29E-01	8.32E-01	7.07E-01	1.03E-01	5.35E-01	2.73E-01	1.43E-01	1.00E+00
ENSBTAG00000018386	-6.14E-02	1.00E+00	8.31E-01	2.94E-03	1.06E+00	7.73E-05	4.84E-01	1.33E-01	-5.25E-01	4.20E-01
ENSBTAG00000018383	-5.87E-02	1.00E+00	-6.55E-01	2.74E-01	5.84E-01	2.89E-01	5.00E-01	4.22E-01	-9.28E-02	1.00E+00
ENSBTAG00000018381	5.27E-01	2.30E-01	7.94E-01	1.94E-03	9.00E-01	3.19E-04	5.74E-01	3.81E-02	3.92E-02	1.00E+00
ENSBTAG00000025202	0.00E+00	1.00E+00	0.00E+00	1.00E+00	-5.13E-15	1.00E+00	0.00E+00	1.00E+00	0.00E+00	1.00E+00
ENSBTAG00000042787	0.00E+00	1.00E+00	0.00E+00	1.00E+00	-5.13E-15	1.00E+00	0.00E+00	1.00E+00	0.00E+00	1.00E+00
ENSBTAG00000018752	-1.71E-01	1.00E+00	-1.75E-01	7.91E-01	5.04E-01	1.04E-01	6.00E-01	4.80E-02	-3.58E-01	7.24E-01
ENSBTAG00000015612	2.88E-01	6.62E-01	9.86E-01	1.57E-06	1.28E+00	2.70E-10	1.02E+00	6.92E-07	-2.32E-01	8.00E-01
ENSBTAG00000042680	0.00E+00	1.00E+00	0.00E+00	1.00E+00	-5.13E-15	1.00E+00	0.00E+00	1.00E+00	0.00E+00	1.00E+00
ENSBTAG00000001885	-2.49E-01	1.00E+00	9.82E-01	1.79E-02	-6.25E-01	2.32E-01	-7.69E-02	1.00E+00	4.49E-02	1.00E+00

ENSBTAG00000044401	0.00E+00	1.00E+00	0.00E+00	1.00E+00	-5.13E-15	1.00E+00	0.00E+00	1.00E+00	0.00E+00	1.00E+00
ENSBTAG00000042969	0.00E+00	1.00E+00	0.00E+00	1.00E+00	-5.13E-15	1.00E+00	0.00E+00	1.00E+00	0.00E+00	1.00E+00
ENSBTAG00000042105	0.00E+00	1.00E+00	0.00E+00	1.00E+00	-5.13E-15	1.00E+00	0.00E+00	1.00E+00	0.00E+00	1.00E+00
ENSBTAG00000029914	4.60E-01	1.00E+00	4.18E-01	8.93E-01	9.05E-01	3.47E-01	9.38E-01	3.27E-01	1.20E+00	5.01E-01
ENSBTAG00000047797	-2.45E-01	1.00E+00	-1.63E-01	1.00E+00	5.31E-01	7.87E-01	7.47E-01	5.54E-01	1.07E+00	6.91E-01
ENSBTAG00000045149	-9.41E-01	8.23E-01	-5.99E-01	7.25E-01	7.50E-01	4.28E-01	2.85E-01	1.00E+00	1.27E-01	1.00E+00
ENSBTAG00000029955	2.77E+00	1.47E-01	8.84E-01	8.53E-01	-1.23E-01	1.00E+00	3.02E+00	1.41E-02	7.98E-01	1.00E+00
ENSBTAG00000016972	-6.43E-01	2.07E-01	-3.07E+00	1.13E-21	-4.19E+00	2.01E-34	-2.26E+00	9.60E-14	-4.57E-02	1.00E+00
ENSBTAG00000011829	1.89E-01	1.00E+00	7.62E-01	1.58E-03	6.86E-01	4.85E-03	9.26E-01	9.79E-05	3.98E-01	5.07E-01
ENSBTAG00000009354	0.00E+00	1.00E+00	0.00E+00	1.00E+00	-5.13E-15	1.00E+00	0.00E+00	1.00E+00	0.00E+00	1.00E+00
ENSBTAG00000009353	0.00E+00	1.00E+00	0.00E+00	1.00E+00	-5.13E-15	1.00E+00	0.00E+00	1.00E+00	0.00E+00	1.00E+00
ENSBTAG00000025213	0.00E+00	1.00E+00	0.00E+00	1.00E+00	-5.13E-15	1.00E+00	0.00E+00	1.00E+00	0.00E+00	1.00E+00
ENSBTAG00000005008	1.69E-01	1.00E+00	2.70E-02	1.00E+00	6.81E-01	6.67E-03	2.73E-01	4.38E-01	-3.34E-01	6.69E-01
ENSBTAG00000006601	4.82E-01	7.69E-01	5.36E-01	2.80E-01	2.57E-01	7.66E-01	1.46E-01	1.00E+00	1.98E-01	1.00E+00
ENSBTAG00000043664	0.00E+00	1.00E+00	0.00E+00	1.00E+00	-5.13E-15	1.00E+00	0.00E+00	1.00E+00	0.00E+00	1.00E+00
ENSBTAG00000046372	-2.70E+00	5.76E-01	8.95E-02	1.00E+00	-9.17E-01	8.09E-01	3.25E-01	1.00E+00	-7.74E-01	1.00E+00
ENSBTAG00000006846	-6.52E-02	1.00E+00	4.42E-01	4.92E-02	4.27E-01	5.92E-02	-1.52E-01	7.03E-01	-5.47E-02	1.00E+00
ENSBTAG00000006894	3.74E-01	7.96E-01	-5.52E-01	1.56E-01	-6.87E-02	1.00E+00	3.95E-01	3.46E-01	-3.44E-01	8.00E-01
ENSBTAG00000024490	0.00E+00	1.00E+00	0.00E+00	1.00E+00	-5.13E-15	1.00E+00	0.00E+00	1.00E+00	0.00E+00	1.00E+00
ENSBTAG00000046595	0.00E+00	1.00E+00	0.00E+00	1.00E+00	-5.13E-15	1.00E+00	0.00E+00	1.00E+00	0.00E+00	1.00E+00
ENSBTAG00000023970	0.00E+00	1.00E+00	0.00E+00	1.00E+00	-5.13E-15	1.00E+00	0.00E+00	1.00E+00	0.00E+00	1.00E+00
ENSBTAG00000001836	0.00E+00	1.00E+00	0.00E+00	1.00E+00	-5.13E-15	1.00E+00	0.00E+00	1.00E+00	0.00E+00	1.00E+00
ENSBTAG00000007238	0.00E+00	1.00E+00	0.00E+00	1.00E+00	-5.13E-15	1.00E+00	0.00E+00	1.00E+00	0.00E+00	1.00E+00
ENSBTAG00000046154	-1.91E+00	8.23E-01	-1.91E+00	4.69E-01	-1.25E-01	1.00E+00	-1.91E+00	4.39E-01	8.22E-01	1.00E+00
ENSBTAG00000033298	-1.66E-01	1.00E+00	-1.18E+00	9.76E-05	-8.61E-01	5.81E-03	-3.36E-01	4.39E-01	-3.47E-01	8.00E-01
ENSBTAG00000045001	2.70E+00	5.76E-01	0.00E+00	1.00E+00	-5.13E-15	1.00E+00	0.00E+00	1.00E+00	3.89E+00	2.05E-01
ENSBTAG00000014825	7.67E-02	1.00E+00	-1.83E-01	7.77E-01	-2.59E-01	5.43E-01	-3.02E-01	4.59E-01	-4.69E-01	5.12E-01
ENSBTAG00000008109	-6.75E-01	9.20E-02	-9.44E-01	5.03E-04	-1.53E+00	5.80E-09	-1.47E+00	2.89E-08	-5.11E-01	4.01E-01
ENSBTAG00000008110	-3.83E-01	7.05E-01	-8.27E-01	6.39E-03	-1.58E+00	3.49E-08	-1.37E+00	2.65E-06	-3.62E-01	7.67E-01
ENSBTAG00000008115	5.12E-01	3.59E-01	9.71E-01	4.88E-04	1.02E+00	2.25E-04	1.51E+00	2.90E-08	2.90E-01	8.01E-01
ENSBTAG00000008116	-7.53E-02	1.00E+00	-4.88E-01	1.08E-01	-8.45E-01	1.83E-03	-6.30E-01	3.04E-02	2.04E-03	1.00E+00
ENSBTAG00000008120	-4.99E-03	1.00E+00	-1.24E-01	8.25E-01	8.08E-02	1.00E+00	4.22E-01	6.81E-02	-8.04E-02	1.00E+00
ENSBTAG00000033270	8.11E-01	3.57E-01	-1.81E+00	1.42E-04	-2.73E+00	1.58E-08	-1.86E+00	8.41E-05	5.40E-01	7.72E-01
ENSBTAG00000016151	-8.78E-02	1.00E+00	-4.48E-01	5.36E-02	-1.22E+00	2.42E-09	-1.47E+00	7.33E-13	-5.51E-02	1.00E+00
ENSBTAG00000002816	1.86E-01	1.00E+00	-8.61E-01	1.53E-02	-9.33E-01	6.61E-03	-6.78E-01	6.73E-02	4.94E-01	5.81E-01

ENSBTAG00000002817	-3.43E-01	8.29E-01	-2.36E+00	1.03E-11	-1.59E+00	1.91E-06	-1.08E+00	1.74E-03	-6.39E-03	1.00E+00
ENSBTAG00000044525	0.00E+00	1.00E+00	0.00E+00	1.00E+00	-5.13E-15	1.00E+00	0.00E+00	1.00E+00	0.00E+00	1.00E+00
ENSBTAG00000008788	-2.52E-01	1.00E+00	-1.68E+00	2.12E-02	-1.80E+00	1.28E-02	-1.10E+00	1.82E-01	-4.65E-01	1.00E+00
ENSBTAG00000013095	-1.32E+00	3.75E-01	-2.00E+00	2.12E-02	-2.26E+00	6.71E-03	-2.65E+00	2.51E-03	-2.47E+00	6.28E-02
ENSBTAG00000013096	-2.32E-01	8.57E-01	-1.07E+00	4.30E-06	-1.64E+00	7.45E-13	-1.10E+00	2.02E-06	-1.11E-01	1.00E+00
ENSBTAG00000013098	-3.61E-02	1.00E+00	-1.07E+00	3.40E-03	-3.48E-01	4.90E-01	7.48E-02	1.00E+00	3.89E-01	8.00E-01
ENSBTAG00000013099	-6.13E-02	1.00E+00	-8.73E-01	5.41E-02	-1.19E+00	5.50E-03	-5.24E-01	3.32E-01	4.02E-01	8.33E-01
ENSBTAG00000013100	3.17E-01	1.00E+00	-1.68E-02	1.00E+00	-8.03E-01	1.51E-01	-1.42E-01	1.00E+00	-3.50E-01	1.00E+00
ENSBTAG00000011984	9.41E-02	1.00E+00	1.70E-01	9.07E-01	-9.87E-02	1.00E+00	-4.47E-01	3.22E-01	3.06E-01	8.94E-01
ENSBTAG00000011988	-3.30E-02	1.00E+00	-5.20E-01	3.92E-02	-3.55E-01	2.05E-01	3.93E-02	1.00E+00	3.84E-01	5.09E-01
ENSBTAG00000012845	-4.22E-02	1.00E+00	4.18E-01	1.47E-01	4.11E-01	1.57E-01	3.22E-01	3.23E-01	1.01E-01	1.00E+00
ENSBTAG00000009036	2.52E-01	8.28E-01	1.51E+00	1.31E-10	1.42E+00	1.37E-09	1.17E+00	8.50E-07	2.41E-01	8.33E-01
ENSBTAG00000017268	-5.70E-01	6.84E-01	-1.59E+00	8.88E-04	-2.05E+00	1.40E-05	-1.50E+00	1.16E-03	-5.20E-02	1.00E+00
ENSBTAG00000046129	1.75E-01	1.00E+00	-5.75E-01	1.75E-01	-2.51E-01	6.94E-01	-2.75E-01	6.46E-01	4.87E-02	1.00E+00
ENSBTAG00000046394	4.04E-01	5.88E-01	1.00E+00	2.51E-04	1.75E+00	1.60E-11	7.54E-01	9.08E-03	-4.59E-01	5.54E-01
ENSBTAG00000045641	0.00E+00	1.00E+00	0.00E+00	1.00E+00	-5.13E-15	1.00E+00	0.00E+00	1.00E+00	0.00E+00	1.00E+00
ENSBTAG00000046524	0.00E+00	1.00E+00	0.00E+00	1.00E+00	-5.13E-15	1.00E+00	0.00E+00	1.00E+00	0.00E+00	1.00E+00
ENSBTAG00000048217	0.00E+00	1.00E+00	0.00E+00	1.00E+00	-5.13E-15	1.00E+00	0.00E+00	1.00E+00	0.00E+00	1.00E+00
ENSBTAG00000006777	3.16E-02	1.00E+00	-1.01E+00	2.80E-03	-1.08E+00	9.20E-04	-5.80E-02	1.00E+00	6.21E-03	1.00E+00
ENSBTAG00000015535	3.93E-02	1.00E+00	7.11E-01	1.90E-02	5.64E-01	7.55E-02	1.68E-01	8.43E-01	1.68E-01	1.00E+00
ENSBTAG00000038990	-3.78E-01	8.28E-01	-8.34E-01	3.39E-02	-1.04E+00	6.06E-03	-6.60E-01	1.21E-01	1.27E-01	1.00E+00
ENSBTAG00000047717	-6.01E-01	6.43E-02	-1.51E+00	2.87E-11	-7.55E-01	9.78E-04	-4.03E-01	1.29E-01	6.03E-01	9.10E-02
ENSBTAG00000045484	0.00E+00	1.00E+00	0.00E+00	1.00E+00	-5.13E-15	1.00E+00	0.00E+00	1.00E+00	0.00E+00	1.00E+00
ENSBTAG00000015639	-2.03E-01	8.29E-01	1.58E-01	6.36E-01	2.98E-01	2.03E-01	1.53E-01	6.63E-01	-6.80E-02	1.00E+00
ENSBTAG00000036414	0.00E+00	1.00E+00	0.00E+00	1.00E+00	-5.13E-15	1.00E+00	0.00E+00	1.00E+00	0.00E+00	1.00E+00
ENSBTAG00000029953	0.00E+00	1.00E+00	0.00E+00	1.00E+00	-5.13E-15	1.00E+00	0.00E+00	1.00E+00	0.00E+00	1.00E+00
ENSBTAG00000010073	6.74E-01	2.28E-02	1.05E+00	2.16E-06	8.44E-01	1.63E-04	8.51E-01	1.79E-04	5.01E-01	2.48E-01
ENSBTAG00000013501	3.86E-01	7.21E-01	1.46E-01	9.23E-01	-3.00E-01	4.82E-01	2.65E-01	5.78E-01	3.92E-01	7.12E-01
ENSBTAG00000015976	-1.64E-01	1.00E+00	-1.24E-01	8.68E-01	3.14E-01	2.63E-01	1.43E-01	7.96E-01	-5.10E-02	1.00E+00
ENSBTAG00000015979	-4.94E-01	9.42E-01	-2.73E-01	9.19E-01	-8.10E-01	2.51E-01	-1.38E+00	3.77E-02	-1.02E-01	1.00E+00
ENSBTAG00000007946	-3.75E-01	1.00E+00	-1.42E+00	4.69E-02	-2.69E+00	6.78E-05	-2.86E+00	2.91E-05	-4.79E-02	1.00E+00
ENSBTAG00000017380	-4.11E-02	1.00E+00	-1.03E+00	2.33E-03	-1.67E+00	3.07E-07	-1.51E+00	5.51E-06	3.73E-01	8.00E-01
ENSBTAG00000042201	3.58E+00	2.32E-01	0.00E+00	1.00E+00	-5.13E-15	1.00E+00	0.00E+00	1.00E+00	2.72E+00	6.01E-01
ENSBTAG00000039440	-9.45E-01	7.23E-01	9.15E-02	1.00E+00	-2.61E-01	9.94E-01	-1.01E+00	2.66E-01	-6.62E-01	8.71E-01
ENSBTAG00000005352	5.38E-01	1.00E+00	2.57E+00	9.52E-04	5.73E-01	8.23E-01	-1.99E-01	1.00E+00	-3.63E-01	1.00E+00

ENSBTAG00000033077	8.20E-01	2.86E-02	2.17E+00	2.23E-15	1.67E+00	9.29E-10	1.23E+00	1.13E-05	2.07E-01	1.00E+00
ENSBTAG00000043316	0.00E+00	1.00E+00	0.00E+00	1.00E+00	-5.13E-15	1.00E+00	0.00E+00	1.00E+00	0.00E+00	1.00E+00
ENSBTAG00000047540	9.55E-01	8.54E-01	1.21E+00	3.12E-01	7.80E-01	6.19E-01	1.63E+00	9.16E-02	3.03E-01	1.00E+00
ENSBTAG00000000827	1.16E-01	1.00E+00	8.98E-01	2.68E-03	7.48E-01	1.48E-02	1.12E+00	1.21E-04	-1.19E-01	1.00E+00
ENSBTAG00000025167	-4.66E-01	8.20E-01	-2.48E+00	3.96E-09	-2.82E+00	2.39E-11	-4.21E-01	4.75E-01	6.34E-01	5.58E-01
ENSBTAG00000014687	-3.20E-01	7.76E-01	-1.48E+00	1.47E-08	-2.57E+00	3.43E-22	-1.55E+00	3.04E-09	-1.01E-01	1.00E+00
ENSBTAG00000012040	-1.23E+00	1.34E-06	-2.23E+00	1.21E-20	-1.43E+00	7.33E-10	-6.36E-01	1.14E-02	-5.44E-02	1.00E+00
ENSBTAG00000001119	-7.16E-02	1.00E+00	2.00E-01	8.97E-01	6.57E-01	1.20E-01	1.23E-01	1.00E+00	-5.12E-02	1.00E+00
ENSBTAG00000001120	-3.55E-02	1.00E+00	-1.34E+00	6.60E-04	-2.23E+00	1.43E-08	-2.26E+00	1.24E-08	4.91E-01	6.93E-01
ENSBTAG00000044956	0.00E+00	1.00E+00	0.00E+00	1.00E+00	-5.13E-15	1.00E+00	0.00E+00	1.00E+00	0.00E+00	1.00E+00
ENSBTAG00000011011	2.64E-01	8.82E-01	8.49E-01	1.91E-03	2.70E-01	4.83E-01	7.22E-01	1.12E-02	5.62E-01	3.09E-01
ENSBTAG00000042873	0.00E+00	1.00E+00	0.00E+00	1.00E+00	-5.13E-15	1.00E+00	0.00E+00	1.00E+00	0.00E+00	1.00E+00
ENSBTAG00000042268	0.00E+00	1.00E+00	0.00E+00	1.00E+00	-5.13E-15	1.00E+00	0.00E+00	1.00E+00	0.00E+00	1.00E+00
ENSBTAG00000043915	0.00E+00	1.00E+00	0.00E+00	1.00E+00	-5.13E-15	1.00E+00	0.00E+00	1.00E+00	0.00E+00	1.00E+00
ENSBTAG00000019122	-5.26E-02	1.00E+00	1.01E+00	3.31E-02	-1.19E+00	1.72E-02	-1.49E+00	2.80E-03	1.30E-01	1.00E+00
ENSBTAG00000019341	4.79E-01	2.99E-01	1.49E+00	3.37E-10	1.38E+00	5.04E-09	6.60E-01	1.13E-02	-1.33E-01	1.00E+00
ENSBTAG00000029822	0.00E+00	1.00E+00	0.00E+00	1.00E+00	-5.13E-15	1.00E+00	0.00E+00	1.00E+00	0.00E+00	1.00E+00
ENSBTAG00000019349	-3.44E-01	5.81E-01	7.57E-01	1.17E-03	4.20E-01	1.11E-01	-8.17E-01	6.86E-04	-1.52E-01	1.00E+00
ENSBTAG00000021370	5.10E-02	1.00E+00	8.37E-01	5.86E-06	9.00E-01	8.30E-07	7.36E-01	9.63E-05	-2.00E-02	1.00E+00
ENSBTAG00000002609	1.11E-02	1.00E+00	8.81E-01	8.52E-01	-1.31E-01	1.00E+00	-1.91E+00	4.39E-01	-1.91E+00	8.00E-01
ENSBTAG00000043798	8.24E-03	1.00E+00	-7.90E-01	7.90E-01	-1.02E+00	6.10E-01	-3.58E+00	4.99E-02	9.69E-01	8.24E-01
ENSBTAG00000012371	2.53E-01	1.00E+00	5.67E-01	1.62E-01	1.25E+00	2.69E-04	2.05E+00	1.35E-09	8.82E-01	1.28E-01
ENSBTAG00000004961	3.38E-01	5.89E-01	1.46E+00	3.44E-11	1.36E+00	7.29E-10	1.05E+00	3.71E-06	3.15E-01	6.79E-01
ENSBTAG00000046743	-2.70E+00	5.76E-01	-2.70E+00	2.42E-01	-2.70E+00	1.99E-01	-2.70E+00	2.05E-01	-2.70E+00	6.01E-01
ENSBTAG00000019379	5.52E-03	1.00E+00	7.62E-02	1.00E+00	6.45E-01	9.79E-01	2.23E+00	1.36E-01	1.71E+00	7.74E-01
ENSBTAG00000048216	0.00E+00	1.00E+00	0.00E+00	1.00E+00	1.78E+00	4.79E-01	0.00E+00	1.00E+00	0.00E+00	1.00E+00
ENSBTAG00000044642	0.00E+00	1.00E+00	0.00E+00	1.00E+00	-5.13E-15	1.00E+00	0.00E+00	1.00E+00	0.00E+00	1.00E+00
ENSBTAG00000008424	-1.96E-01	1.00E+00	5.20E-01	7.74E-02	8.40E-01	1.62E-03	6.87E-01	1.44E-02	-2.01E-01	9.93E-01
ENSBTAG00000008423	1.39E-01	1.00E+00	8.80E-01	1.04E-02	9.49E-01	4.79E-03	9.06E-01	8.85E-03	2.63E-02	1.00E+00
ENSBTAG00000000855	1.49E-02	1.00E+00	3.42E-01	3.76E-01	-5.27E-01	1.11E-01	-5.70E-01	8.44E-02	-5.93E-01	3.25E-01
ENSBTAG00000019162	2.24E-01	1.00E+00	1.65E+00	3.59E-06	2.72E+00	1.62E-14	2.28E+00	1.27E-10	-3.62E-02	1.00E+00
ENSBTAG00000019160	1.77E-01	9.56E-01	2.10E-01	4.70E-01	5.88E-03	1.00E+00	-3.28E-01	1.93E-01	-1.34E-01	1.00E+00
ENSBTAG00000013395	5.32E-01	7.46E-01	3.22E+00	3.75E-21	2.20E+00	4.98E-10	9.14E-01	3.76E-02	5.03E-01	7.90E-01
ENSBTAG00000013393	1.97E-02	1.00E+00	2.08E+00	2.69E-27	2.00E+00	1.25E-25	1.33E+00	8.15E-12	1.11E-01	1.00E+00
ENSBTAG00000046273	9.72E-01	3.13E-02	1.27E-01	1.00E+00	4.11E-01	3.85E-01	1.19E+00	3.17E-04	-1.64E-02	1.00E+00

ENSBTAG00000018126	4.44E-02	1.00E+00	3.25E-01	3.02E-01	8.23E-01	8.40E-04	7.63E-01	2.63E-03	-3.59E-01	6.12E-01
ENSBTAG00000044395	0.00E+00	1.00E+00	0.00E+00	1.00E+00	-5.13E-15	1.00E+00	0.00E+00	1.00E+00	1.91E+00	8.00E-01
ENSBTAG00000006606	1.26E+00	8.54E-02	4.15E-01	6.70E-01	2.14E-01	9.95E-01	1.21E+00	2.32E-02	4.67E-01	9.12E-01
ENSBTAG00000029295	1.92E+00	8.23E-01	0.00E+00	1.00E+00	-5.13E-15	1.00E+00	0.00E+00	1.00E+00	3.24E+00	4.34E-01
ENSBTAG00000046148	-3.59E+00	2.32E-01	-3.59E+00	6.66E-02	-1.04E+00	5.96E-01	-5.56E-01	9.24E-01	-3.59E+00	3.09E-01
ENSBTAG00000039819	-5.19E-02	1.00E+00	-4.56E-01	3.04E-01	5.30E-01	1.79E-01	1.11E+00	9.21E-04	-3.64E-01	8.00E-01
ENSBTAG00000025140	-1.81E-01	1.00E+00	1.66E-01	9.24E-01	-6.43E-01	1.05E-01	-1.21E+00	1.08E-03	-3.00E-01	9.17E-01
ENSBTAG00000005664	4.70E-02	1.00E+00	-2.39E-02	1.00E+00	6.22E-02	1.00E+00	2.20E-01	4.41E-01	-2.09E-01	8.00E-01
ENSBTAG00000005665	3.49E-01	2.42E-01	1.12E+00	5.01E-12	1.04E+00	1.43E-10	7.61E-01	5.69E-06	-1.97E-01	8.00E-01
ENSBTAG00000001332	-5.70E-02	1.00E+00	-6.74E-01	1.79E-03	-5.25E-01	1.90E-02	1.63E-02	1.00E+00	-6.72E-03	1.00E+00
ENSBTAG00000011479	-6.48E-02	1.00E+00	-4.34E-01	1.29E-01	-3.31E-01	2.92E-01	-5.74E-01	3.46E-02	2.01E-01	9.54E-01
ENSBTAG00000011480	2.02E-01	9.18E-01	3.72E-01	1.42E-01	1.39E+00	2.19E-11	1.21E+00	8.80E-09	1.04E-01	1.00E+00
ENSBTAG00000044568	0.00E+00	1.00E+00	0.00E+00	1.00E+00	-5.13E-15	1.00E+00	0.00E+00	1.00E+00	0.00E+00	1.00E+00
ENSBTAG00000011482	-8.19E-01	1.77E-01	-2.31E+00	4.50E-10	-4.95E-01	2.89E-01	-3.11E-01	6.13E-01	-6.93E-01	4.10E-01
ENSBTAG00000011483	2.36E-01	9.32E-01	1.61E-01	8.11E-01	9.00E-01	6.34E-04	1.08E-01	9.94E-01	4.96E-01	4.03E-01
ENSBTAG00000038889	3.01E-01	8.04E-01	4.80E-01	9.79E-02	1.26E+00	3.28E-07	1.29E+00	2.47E-07	6.75E-01	1.06E-01
ENSBTAG00000011488	3.65E-02	1.00E+00	3.70E-01	1.71E-01	9.22E-01	3.93E-05	1.05E+00	2.70E-06	3.89E-01	4.83E-01
ENSBTAG00000011490	-1.75E-01	1.00E+00	1.26E+00	1.55E-01	2.28E+00	4.97E-04	1.93E+00	7.02E-03	8.91E-01	8.00E-01
ENSBTAG00000029930	4.67E-01	7.79E-01	5.64E-01	2.36E-01	2.37E+00	6.64E-12	2.17E+00	5.81E-10	6.05E-01	5.69E-01
ENSBTAG00000011491	-1.65E-01	1.00E+00	7.84E-02	1.00E+00	1.10E+00	1.26E-03	7.71E-01	3.76E-02	2.48E-01	1.00E+00
ENSBTAG00000020859	5.65E-01	6.45E-02	7.54E-01	4.61E-04	6.53E-01	2.82E-03	7.29E-01	8.91E-04	1.23E+00	1.63E-07
ENSBTAG00000009705	3.92E-01	3.85E-01	3.08E-01	2.62E-01	-3.18E-02	1.00E+00	7.16E-03	1.00E+00	9.46E-01	4.35E-04
ENSBTAG00000009708	-6.71E-02	1.00E+00	6.23E-02	1.00E+00	8.42E-01	3.00E-03	5.36E-01	9.56E-02	3.51E-01	7.62E-01
ENSBTAG00000009711	-4.36E-01	3.75E-01	-7.53E-01	2.40E-03	2.56E-01	4.52E-01	3.23E-01	3.04E-01	-1.17E-01	1.00E+00
ENSBTAG00000012302	1.48E+00	5.76E-01	4.72E-01	9.90E-01	-1.53E-01	1.00E+00	-1.45E+00	4.96E-01	4.00E-01	1.00E+00
ENSBTAG00000009376	-1.63E-01	1.00E+00	-5.95E-02	1.00E+00	9.29E-02	1.00E+00	1.77E-01	7.09E-01	-1.99E-02	1.00E+00
ENSBTAG00000047926	0.00E+00	1.00E+00	0.00E+00	1.00E+00	-5.13E-15	1.00E+00	0.00E+00	1.00E+00	0.00E+00	1.00E+00
ENSBTAG00000029905	0.00E+00	1.00E+00	0.00E+00	1.00E+00	-5.13E-15	1.00E+00	1.76E+00	4.97E-01	0.00E+00	1.00E+00
ENSBTAG00000036391	-1.91E+00	8.23E-01	-1.91E+00	4.69E-01	-1.25E-01	1.00E+00	1.12E+00	6.68E-01	-1.91E+00	8.00E-01
ENSBTAG00000025130	-3.18E-01	8.23E-01	-1.56E-01	8.55E-01	1.93E-02	1.00E+00	-3.47E-02	1.00E+00	-5.03E-01	4.40E-01
ENSBTAG00000000267	-2.64E-02	1.00E+00	-1.10E+00	5.98E-07	-3.76E-01	1.45E-01	3.99E-01	1.19E-01	1.33E-01	1.00E+00
ENSBTAG00000043786	0.00E+00	1.00E+00	0.00E+00	1.00E+00	-5.13E-15	1.00E+00	0.00E+00	1.00E+00	0.00E+00	1.00E+00
ENSBTAG00000004223	2.53E-01	8.48E-01	1.96E+00	9.92E-17	1.79E+00	2.18E-14	1.11E+00	4.65E-06	-1.39E-01	1.00E+00
ENSBTAG00000045793	0.00E+00	1.00E+00	0.00E+00	1.00E+00	-5.13E-15	1.00E+00	0.00E+00	1.00E+00	0.00E+00	1.00E+00
ENSBTAG00000004225	3.00E-01	8.23E-01	2.32E+00	1.16E-20	2.56E+00	8.24E-25	1.98E+00	1.68E-15	-1.93E-01	1.00E+00

ENSBTAG00000042780	0.00E+00	1.00E+00	0.00E+00	1.00E+00	-5.13E-15	1.00E+00	0.00E+00	1.00E+00	0.00E+00	1.00E+00
ENSBTAG00000042387	0.00E+00	1.00E+00	0.00E+00	1.00E+00	-5.13E-15	1.00E+00	0.00E+00	1.00E+00	0.00E+00	1.00E+00
ENSBTAG00000045500	5.20E-02	1.00E+00	-1.16E+00	5.75E-05	-1.12E+00	7.90E-05	-1.99E-01	7.26E-01	-4.02E-01	6.30E-01
ENSBTAG00000025129	-4.45E-01	8.28E-01	5.44E-01	3.25E-01	1.03E+00	2.14E-02	8.75E-01	6.61E-02	1.05E+00	1.53E-01
ENSBTAG00000017440	3.06E-01	7.23E-01	1.81E+00	1.55E-17	2.07E+00	3.14E-23	1.69E+00	1.77E-15	2.53E-01	8.00E-01
ENSBTAG00000016806	3.51E-01	6.68E-01	5.27E-01	6.47E-02	6.92E-01	1.00E-02	9.59E-01	2.40E-04	1.91E-01	1.00E+00
ENSBTAG00000011786	7.13E-02	1.00E+00	-4.73E-03	1.00E+00	5.31E-01	7.67E-02	1.02E+00	1.84E-04	5.62E-01	3.07E-01
ENSBTAG00000020000	1.47E+00	1.89E-01	1.08E+00	2.02E-01	2.25E+00	2.55E-04	2.43E+00	7.16E-05	1.20E+00	4.66E-01
ENSBTAG00000008481	-1.91E+00	8.23E-01	-1.91E+00	4.69E-01	-1.91E+00	4.28E-01	-1.91E+00	4.39E-01	-1.91E+00	8.00E-01
ENSBTAG00000008324	0.00E+00	1.00E+00	0.00E+00	1.00E+00	-5.13E-15	1.00E+00	0.00E+00	1.00E+00	1.93E+00	8.00E-01
ENSBTAG00000039018	0.00E+00	1.00E+00	0.00E+00	1.00E+00	-5.13E-15	1.00E+00	0.00E+00	1.00E+00	0.00E+00	1.00E+00
ENSBTAG00000040362	0.00E+00	1.00E+00	1.99E+00	4.43E-01	-5.13E-15	1.00E+00	1.77E+00	4.97E-01	0.00E+00	1.00E+00
ENSBTAG00000039411	0.00E+00	1.00E+00	0.00E+00	1.00E+00	-5.13E-15	1.00E+00	0.00E+00	1.00E+00	1.93E+00	8.00E-01
ENSBTAG00000039633	-1.91E+00	8.23E-01	-1.91E+00	4.69E-01	-1.91E+00	4.28E-01	-1.91E+00	4.39E-01	-1.91E+00	8.00E-01
ENSBTAG00000042590	0.00E+00	1.00E+00	0.00E+00	1.00E+00	-5.13E-15	1.00E+00	0.00E+00	1.00E+00	0.00E+00	1.00E+00
ENSBTAG00000019391	0.00E+00	1.00E+00	0.00E+00	1.00E+00	-5.13E-15	1.00E+00	0.00E+00	1.00E+00	0.00E+00	1.00E+00
ENSBTAG00000048282	0.00E+00	1.00E+00	0.00E+00	1.00E+00	-5.13E-15	1.00E+00	0.00E+00	1.00E+00	1.91E+00	8.00E-01
ENSBTAG00000048099	-1.91E+00	8.23E-01	7.57E-02	1.00E+00	-1.91E+00	4.28E-01	-1.91E+00	4.39E-01	6.35E-03	1.00E+00
ENSBTAG00000047049	0.00E+00	1.00E+00	0.00E+00	1.00E+00	-5.13E-15	1.00E+00	0.00E+00	1.00E+00	0.00E+00	1.00E+00
ENSBTAG00000026859	1.91E+00	8.23E-01	1.98E+00	4.43E-01	-5.13E-15	1.00E+00	1.76E+00	4.97E-01	0.00E+00	1.00E+00
ENSBTAG00000018221	0.00E+00	1.00E+00	0.00E+00	1.00E+00	-5.13E-15	1.00E+00	1.76E+00	4.97E-01	0.00E+00	1.00E+00
ENSBTAG00000046652	-1.91E+00	8.23E-01	6.91E-02	1.00E+00	-1.91E+00	4.28E-01	-1.91E+00	4.39E-01	-1.91E+00	8.00E-01
ENSBTAG00000037529	1.91E+00	8.23E-01	0.00E+00	1.00E+00	1.78E+00	4.79E-01	0.00E+00	1.00E+00	2.72E+00	6.01E-01
ENSBTAG00000038059	-7.87E-01	1.00E+00	-2.70E+00	2.42E-01	-2.70E+00	1.99E-01	-2.70E+00	2.05E-01	5.14E-01	1.00E+00
ENSBTAG00000046960	-1.91E+00	8.23E-01	-1.91E+00	4.69E-01	-1.91E+00	4.28E-01	-1.91E+00	4.39E-01	-1.91E+00	8.00E-01
ENSBTAG00000045667	-1.71E+00	3.79E-01	-3.17E+00	3.88E-03	-5.42E+00	1.75E-05	-4.60E+00	1.19E-04	-1.53E-01	1.00E+00
ENSBTAG00000032467	3.21E+00	3.75E-01	0.00E+00	1.00E+00	-5.13E-15	1.00E+00	0.00E+00	1.00E+00	1.93E+00	8.00E-01
ENSBTAG00000046217	0.00E+00	1.00E+00	1.98E+00	4.43E-01	-5.13E-15	1.00E+00	0.00E+00	1.00E+00	0.00E+00	1.00E+00
ENSBTAG00000047458	0.00E+00	1.00E+00	0.00E+00	1.00E+00	-5.13E-15	1.00E+00	0.00E+00	1.00E+00	0.00E+00	1.00E+00
ENSBTAG00000045899	-7.93E-01	1.00E+00	-7.27E-01	9.45E-01	-2.71E+00	1.99E-01	-2.71E+00	2.05E-01	-2.71E+00	6.01E-01
ENSBTAG00000032457	-2.71E+00	5.76E-01	-7.27E-01	9.45E-01	-2.71E+00	1.99E-01	-1.85E-01	1.00E+00	-2.71E+00	6.01E-01
ENSBTAG00000015763	-1.91E+00	1.40E-01	-1.43E+00	1.17E-01	-8.30E-01	4.34E-01	-1.22E+00	1.98E-01	-5.36E-01	1.00E+00
ENSBTAG00000003629	-2.74E-01	1.00E+00	-1.90E+00	7.02E-04	-2.68E+00	3.18E-06	-1.82E+00	7.64E-04	7.72E-02	1.00E+00
ENSBTAG00000000020	-1.69E+00	1.02E-03	-5.25E+00	1.04E-16	-4.02E+00	1.53E-14	-2.47E+00	5.41E-08	-5.77E-01	6.47E-01
ENSBTAG00000018880	1.35E+00	5.79E-01	2.23E+00	1.09E-02	1.56E+00	1.28E-01	1.49E+00	1.60E-01	3.19E-01	1.00E+00

ENSBTAG00000000829	-1.73E-01	1.00E+00	-1.50E+00	1.32E-07	-2.90E+00	3.18E-23	-2.22E+00	8.73E-15	3.05E-01	8.00E-01
ENSBTAG00000000831	1.55E-01	1.00E+00	-1.18E+00	2.39E-07	-1.36E+00	1.66E-09	-1.02E+00	8.68E-06	1.52E-01	1.00E+00
ENSBTAG00000000833	-5.22E-01	2.21E-01	-9.77E-01	7.27E-05	-1.35E+00	2.26E-08	-1.05E+00	2.18E-05	1.31E-01	1.00E+00
ENSBTAG00000025121	-3.61E-02	1.00E+00	3.44E-01	2.10E-01	4.57E-01	6.75E-02	2.92E-01	3.29E-01	-1.85E-01	9.56E-01
ENSBTAG00000015258	2.94E-01	1.00E+00	2.91E-01	8.31E-01	-3.98E-02	1.00E+00	-3.42E-01	7.48E-01	2.90E-04	1.00E+00
ENSBTAG00000015264	-5.82E-02	1.00E+00	-8.84E-01	6.05E-04	-3.98E-01	1.76E-01	-2.37E-01	5.20E-01	-7.76E-02	1.00E+00
ENSBTAG00000032372	0.00E+00	1.00E+00	0.00E+00	1.00E+00	-5.13E-15	1.00E+00	0.00E+00	1.00E+00	0.00E+00	1.00E+00
ENSBTAG00000016621	-8.20E-02	1.00E+00	-2.56E-01	4.31E-01	3.52E-01	1.98E-01	-7.44E-02	1.00E+00	-2.14E-02	1.00E+00
ENSBTAG00000007166	-9.49E-01	2.81E-02	-1.75E+00	8.46E-08	-3.92E-01	3.56E-01	-7.47E-01	3.43E-02	3.93E-01	7.67E-01
ENSBTAG00000007169	-1.11E-01	1.00E+00	-8.19E-01	3.60E-01	5.32E-01	5.64E-01	8.50E-01	2.52E-01	2.55E-01	1.00E+00
ENSBTAG00000014806	-1.94E-01	1.00E+00	-1.19E+00	1.35E-06	-1.32E-01	8.71E-01	-1.08E-02	1.00E+00	-5.44E-02	1.00E+00
ENSBTAG00000016316	-6.67E-02	1.00E+00	9.08E-01	1.29E-04	9.05E-01	1.28E-04	8.19E-01	7.59E-04	2.22E-01	8.86E-01
ENSBTAG00000016319	-3.91E-02	1.00E+00	-4.88E-01	1.30E-01	-4.65E-01	1.53E-01	-4.89E-01	1.37E-01	2.45E-01	9.04E-01
ENSBTAG00000021091	6.34E-01	3.01E-01	1.28E+00	8.08E-05	1.39E+00	1.53E-05	1.31E+00	6.30E-05	6.14E-01	4.06E-01
ENSBTAG00000014433	3.99E-01	1.55E-01	9.15E-01	6.75E-08	7.81E-01	4.74E-06	3.54E-01	7.56E-02	1.17E-01	1.00E+00
ENSBTAG00000016175	8.80E-01	5.33E-03	1.92E+00	1.19E-14	1.99E+00	9.32E-16	1.33E+00	1.65E-07	2.24E-01	9.20E-01
ENSBTAG00000007429	1.21E+00	2.38E-04	2.13E+00	8.89E-14	7.98E-01	8.93E-03	7.42E-01	1.88E-02	3.58E-01	7.79E-01
ENSBTAG00000007430	3.28E-01	6.83E-01	2.27E+00	1.33E-21	2.47E+00	3.39E-25	2.44E+00	1.32E-24	-2.63E-01	8.00E-01
ENSBTAG00000007436	1.25E+00	1.70E-01	2.85E+00	8.04E-10	1.46E+00	8.86E-03	4.97E-01	6.27E-01	6.43E-01	8.00E-01
ENSBTAG00000007437	1.52E+00	6.21E-01	5.81E+00	5.81E-13	2.84E+00	3.02E-03	2.05E+00	6.74E-02	9.06E-02	1.00E+00
ENSBTAG00000020605	2.74E-01	1.00E+00	-1.87E-01	8.97E-01	-4.37E-01	3.67E-01	-7.07E-01	9.61E-02	6.97E-01	3.15E-01
ENSBTAG00000018345	1.07E+00	8.23E-01	4.25E+00	5.14E-09	3.78E+00	3.24E-07	1.86E+00	6.07E-02	5.65E-01	1.00E+00
ENSBTAG00000011343	3.23E-01	1.00E+00	1.27E+00	7.41E-02	4.25E-01	7.48E-01	-8.69E-01	2.89E-01	-3.55E-01	1.00E+00
ENSBTAG00000004680	3.77E+00	1.17E-09	5.59E+00	4.73E-19	5.95E+00	5.60E-21	6.11E+00	1.30E-21	2.28E+00	1.88E-03
ENSBTAG00000021865	6.40E-01	3.66E-02	6.72E-01	3.70E-03	4.74E-01	5.58E-02	8.60E-02	1.00E+00	7.65E-02	1.00E+00
ENSBTAG00000021864	1.61E-01	1.00E+00	2.70E-01	5.58E-01	2.71E-01	5.52E-01	3.95E-01	3.12E-01	2.59E-01	9.15E-01
ENSBTAG00000015593	6.83E-01	1.62E-01	1.88E+00	1.94E-10	1.61E+00	5.06E-08	6.99E-01	3.52E-02	4.97E-01	5.23E-01
ENSBTAG00000047858	0.00E+00	1.00E+00	0.00E+00	1.00E+00	-5.13E-15	1.00E+00	0.00E+00	1.00E+00	0.00E+00	1.00E+00
ENSBTAG00000002982	3.00E-02	1.00E+00	-1.58E+00	9.09E-03	-6.51E-01	3.55E-01	6.52E-01	3.26E-01	-3.85E-01	1.00E+00
ENSBTAG00000002981	1.65E-01	1.00E+00	-6.05E-01	7.18E-01	-6.56E-01	6.35E-01	1.71E-01	1.00E+00	-1.34E-01	1.00E+00
ENSBTAG00000008318	-1.91E+00	8.23E-01	-1.91E+00	4.69E-01	-1.26E-01	1.00E+00	-1.42E-01	1.00E+00	6.46E-03	1.00E+00
ENSBTAG00000015656	-2.29E-01	1.00E+00	-1.75E+00	1.10E-03	-6.90E-01	2.26E-01	-2.37E-01	8.90E-01	-4.76E-01	8.19E-01
ENSBTAG00000020433	-9.14E-01	2.16E-01	-2.35E+00	1.43E-06	5.99E-02	1.00E+00	1.31E-01	1.00E+00	3.10E-01	9.85E-01
ENSBTAG00000025126	-6.47E-01	8.23E-01	-7.62E-01	3.28E-01	-1.25E+00	6.28E-02	1.39E-02	1.00E+00	-5.94E-01	8.27E-01
ENSBTAG00000020432	8.96E-01	1.69E-02	2.27E+00	8.51E-16	8.21E-01	6.21E-03	6.70E-01	3.60E-02	3.77E-01	7.29E-01

ENSBTAG00000020431	5.30E-01	1.06E-01	1.01E+00	1.80E-06	5.61E-01	1.34E-02	5.09E-01	3.17E-02	1.57E-01	1.00E+00
ENSBTAG00000032083	4.64E-01	4.19E-01	9.77E-01	2.35E-04	1.19E+00	4.22E-06	1.16E+00	9.47E-06	1.00E+00	3.97E-03
ENSBTAG00000012739	7.86E-02	1.00E+00	5.49E-01	3.05E-02	9.87E-01	2.22E-05	1.08E+00	4.32E-06	-2.80E-01	8.00E-01
ENSBTAG00000025149	6.33E-01	7.88E-02	1.32E+00	2.30E-08	6.71E-01	8.52E-03	-1.96E-01	6.69E-01	3.25E-02	1.00E+00
ENSBTAG00000011507	4.08E-01	2.23E-01	1.13E+00	9.67E-10	8.35E-01	9.36E-06	3.11E-01	1.87E-01	7.58E-02	1.00E+00
ENSBTAG00000044450	0.00E+00	1.00E+00	0.00E+00	1.00E+00	-5.13E-15	1.00E+00	0.00E+00	1.00E+00	0.00E+00	1.00E+00
ENSBTAG00000011505	2.81E-01	7.61E-01	2.31E-01	4.71E-01	6.15E-02	1.00E+00	3.22E-01	2.66E-01	1.24E-01	1.00E+00
ENSBTAG00000035975	-4.54E-01	8.87E-01	7.01E-01	1.78E-01	1.15E-01	1.00E+00	-4.18E-01	5.67E-01	4.22E-02	1.00E+00
ENSBTAG00000013906	7.97E-01	1.00E+00	-1.91E+00	4.69E-01	-1.91E+00	4.28E-01	-1.91E+00	4.39E-01	1.68E+00	7.78E-01
ENSBTAG00000003699	5.50E-01	3.37E-01	1.97E+00	5.97E-14	1.73E+00	4.18E-11	8.28E-01	5.31E-03	6.46E-01	2.44E-01
ENSBTAG00000001349	-2.77E-01	7.74E-01	-4.42E-01	8.22E-02	-8.87E-01	8.18E-05	-4.91E-01	5.29E-02	2.19E-01	8.54E-01
ENSBTAG00000008593	2.17E-02	1.00E+00	3.37E-01	5.78E-01	-6.48E-01	1.74E-01	-1.19E+00	7.16E-03	2.42E-01	1.00E+00
ENSBTAG00000008591	2.60E-02	1.00E+00	-1.34E-01	7.51E-01	-3.81E-01	8.46E-02	-6.85E-01	6.53E-04	2.15E-01	8.00E-01
ENSBTAG00000008590	-2.18E-02	1.00E+00	-3.95E-01	2.14E-01	-6.79E-01	1.34E-02	-6.20E-01	3.00E-02	-1.23E-01	1.00E+00
ENSBTAG00000005534	-3.95E-01	5.43E-01	-6.99E-01	8.53E-03	-1.92E+00	5.85E-14	-1.51E+00	3.03E-09	-4.95E-01	3.68E-01
ENSBTAG00000004915	2.44E-02	1.00E+00	-1.76E-02	1.00E+00	-1.56E-01	7.79E-01	4.92E-02	1.00E+00	-5.71E-02	1.00E+00
ENSBTAG00000004913	-1.48E-02	1.00E+00	4.04E-01	1.02E-01	4.31E-01	7.43E-02	-1.02E-02	1.00E+00	-6.74E-02	1.00E+00
ENSBTAG00000004910	-2.41E-01	8.76E-01	-8.29E-01	8.72E-04	-1.21E+00	5.61E-07	-7.58E-01	3.01E-03	-5.88E-02	1.00E+00
ENSBTAG00000004909	-5.78E-01	7.76E-01	-1.33E+00	1.60E-02	-1.47E+00	5.20E-03	-1.69E+00	1.77E-03	3.08E-01	1.00E+00
ENSBTAG00000004908	-3.19E-01	6.21E-01	-7.91E-01	6.80E-04	-1.15E+00	3.06E-07	-1.07E+00	2.37E-06	-8.02E-02	1.00E+00
ENSBTAG00000047992	0.00E+00	1.00E+00	0.00E+00	1.00E+00	-5.13E-15	1.00E+00	0.00E+00	1.00E+00	0.00E+00	1.00E+00
ENSBTAG00000004907	-3.64E-01	4.54E-01	-2.00E+00	2.91E-21	-2.47E+00	3.76E-31	-1.71E+00	3.99E-16	1.53E-01	1.00E+00
ENSBTAG00000013392	-6.49E-02	1.00E+00	-1.05E+00	9.77E-06	-1.22E+00	1.94E-07	-7.12E-01	4.02E-03	2.13E-01	8.97E-01
ENSBTAG00000013390	-6.43E-02	1.00E+00	-2.50E-01	5.63E-01	-4.74E-01	1.45E-01	-1.74E-01	8.02E-01	-4.00E-01	6.23E-01
ENSBTAG00000048034	-7.95E-01	2.03E-02	-1.44E+00	2.88E-08	-2.45E+00	1.24E-20	-2.79E+00	2.86E-25	9.20E-01	8.40E-03
ENSBTAG00000045946	-3.83E-01	1.00E+00	1.39E+00	9.30E-03	6.85E-01	3.27E-01	-5.84E-01	5.30E-01	1.42E-01	1.00E+00
ENSBTAG00000003733	-2.15E-01	9.37E-01	-6.38E-01	1.17E-02	-1.01E+00	2.36E-05	-1.03E+00	2.12E-05	-1.38E-01	1.00E+00
ENSBTAG00000018316	-4.90E-01	3.06E-01	-6.00E-01	2.67E-02	-1.44E+00	5.86E-09	-1.81E+00	4.37E-13	3.96E-02	1.00E+00
ENSBTAG00000031998	3.05E-01	6.21E-01	7.22E-01	8.73E-04	4.63E-01	4.81E-02	3.38E-01	2.01E-01	-2.02E-01	8.75E-01
ENSBTAG00000018314	-1.22E-01	1.00E+00	-9.10E-01	1.50E-03	-5.19E-01	9.95E-02	-5.33E-01	9.56E-02	-1.17E-01	1.00E+00
ENSBTAG00000035587	-1.32E+00	3.78E-07	-2.80E+00	2.94E-29	-2.50E+00	1.23E-24	-1.74E+00	5.35E-13	2.46E-01	8.30E-01
ENSBTAG00000018315	1.30E-02	1.00E+00	6.74E-01	1.14E-03	6.49E-01	1.73E-03	4.20E-01	6.95E-02	2.90E-01	6.40E-01
ENSBTAG00000044862	0.00E+00	1.00E+00	0.00E+00	1.00E+00	-5.13E-15	1.00E+00	0.00E+00	1.00E+00	0.00E+00	1.00E+00
ENSBTAG00000011990	1.38E-01	1.00E+00	-1.62E+00	3.81E-04	-2.97E+00	1.18E-09	-3.96E+00	3.91E-13	3.32E-01	9.30E-01
ENSBTAG00000031933	-5.03E-02	1.00E+00	-1.18E+00	7.39E-02	-2.12E+00	1.02E-03	-3.45E+00	4.96E-06	8.88E-01	4.02E-01

ENSBTAG00000047925	-2.75E-02	1.00E+00	-5.23E-01	9.96E-02	-1.06E+00	1.54E-04	-1.04E+00	2.76E-04	-2.74E-01	8.40E-01
ENSBTAG00000021933	1.78E-01	1.00E+00	-1.30E+00	1.07E-03	-1.20E+00	2.37E-03	-8.18E-01	5.65E-02	-9.04E-02	1.00E+00
ENSBTAG00000021932	1.73E-01	1.00E+00	4.04E-01	1.15E-01	6.91E-01	2.36E-03	7.04E-01	2.34E-03	-1.92E-01	9.23E-01
ENSBTAG00000021931	-3.69E-02	1.00E+00	2.06E-02	1.00E+00	1.34E-01	9.55E-01	1.35E-01	9.56E-01	-2.41E-01	9.63E-01
ENSBTAG00000030113	0.00E+00	1.00E+00	0.00E+00	1.00E+00	-5.13E-15	1.00E+00	0.00E+00	1.00E+00	0.00E+00	1.00E+00
ENSBTAG00000029868	0.00E+00	1.00E+00	1.99E+00	4.43E-01	-5.13E-15	1.00E+00	0.00E+00	1.00E+00	0.00E+00	1.00E+00
ENSBTAG00000020854	2.20E+00	2.30E-04	1.35E+00	1.81E-02	1.99E+00	1.67E-04	1.12E+00	6.52E-02	6.15E-01	8.00E-01
ENSBTAG00000020853	-6.79E-01	3.19E-01	-2.33E+00	4.15E-11	-4.08E+00	8.50E-28	-3.73E+00	4.75E-24	2.98E-01	9.32E-01
ENSBTAG00000020852	-4.80E-01	4.77E-01	-2.36E+00	5.37E-16	-4.15E+00	7.92E-40	-2.28E+00	3.46E-15	5.49E-01	4.03E-01
ENSBTAG00000017895	1.20E+00	1.24E-01	1.06E+00	6.40E-02	4.60E-01	5.90E-01	-1.55E+00	3.99E-02	4.12E-02	1.00E+00
ENSBTAG00000025101	-3.75E-01	3.11E-01	-8.74E-01	4.59E-06	-1.73E+00	1.37E-20	-1.74E+00	1.29E-20	1.93E-01	8.34E-01
ENSBTAG00000012071	-4.51E-02	1.00E+00	-6.97E-01	7.19E-04	-1.51E+00	1.35E-14	-1.80E+00	9.26E-20	8.01E-02	1.00E+00
ENSBTAG00000042630	0.00E+00	1.00E+00	0.00E+00	1.00E+00	-5.13E-15	1.00E+00	0.00E+00	1.00E+00	0.00E+00	1.00E+00
ENSBTAG00000002564	1.89E-01	1.00E+00	-7.19E-01	5.57E-02	-8.12E-01	2.50E-02	-8.08E-01	2.89E-02	4.38E-02	1.00E+00
ENSBTAG00000003072	-2.18E-01	9.17E-01	-5.35E-01	3.58E-02	-1.04E+00	7.45E-06	-1.03E+00	1.13E-05	2.35E-02	1.00E+00
ENSBTAG00000029812	0.00E+00	1.00E+00	0.00E+00	1.00E+00	-5.13E-15	1.00E+00	0.00E+00	1.00E+00	0.00E+00	1.00E+00
ENSBTAG00000003075	-9.50E-01	8.44E-04	-2.33E+00	5.08E-20	-1.89E+00	9.31E-15	-1.27E+00	1.80E-07	-1.63E-01	1.00E+00
ENSBTAG00000014881	-9.41E-01	1.64E-02	-1.74E+00	7.52E-09	3.83E-01	3.30E-01	3.88E-01	3.34E-01	-2.54E-01	9.35E-01
ENSBTAG00000014883	1.75E-02	1.00E+00	-3.18E-01	2.36E-01	-5.69E-01	1.27E-02	-4.60E-01	6.02E-02	-2.09E-01	8.60E-01
ENSBTAG00000019443	1.63E-01	9.96E-01	7.86E-01	7.20E-05	6.98E-01	4.78E-04	4.17E-01	6.47E-02	1.15E-01	1.00E+00
ENSBTAG00000019446	1.41E-03	1.00E+00	-5.34E-02	1.00E+00	1.74E-01	5.58E-01	1.45E-01	6.91E-01	2.13E-01	8.00E-01
ENSBTAG00000019448	1.24E-01	1.00E+00	-1.23E+00	2.80E-06	-1.81E+00	2.45E-12	-4.14E-01	2.05E-01	1.51E-01	1.00E+00
ENSBTAG00000009190	-5.20E-01	8.02E-01	-3.97E+00	2.79E-11	-1.17E+00	1.37E-02	1.67E-02	1.00E+00	-2.00E-01	1.00E+00
ENSBTAG00000009126	-1.14E-01	1.00E+00	-9.21E-01	3.35E-02	-2.44E+00	3.65E-08	-1.65E+00	8.72E-05	-4.33E-01	8.00E-01
ENSBTAG00000002018	-3.43E-02	1.00E+00	5.59E-01	2.77E-02	1.02E+00	1.34E-05	1.22E+00	1.86E-07	-1.64E-01	1.00E+00
ENSBTAG00000010513	7.98E-02	1.00E+00	3.48E-01	2.23E-01	5.81E-01	1.75E-02	4.23E-01	1.19E-01	-1.64E-02	1.00E+00
ENSBTAG00000010519	-3.22E-02	1.00E+00	5.46E-01	1.02E-02	5.90E-01	4.72E-03	2.14E-01	4.75E-01	-3.65E-02	1.00E+00
ENSBTAG00000045892	-6.87E-02	1.00E+00	-8.22E-01	9.14E-03	-1.30E+00	1.57E-05	-1.58E+00	2.42E-07	3.17E-01	8.00E-01
ENSBTAG00000010527	1.27E-02	1.00E+00	-3.59E-01	2.25E-01	-8.75E-01	3.19E-04	-1.17E+00	1.37E-06	-1.19E-01	1.00E+00
ENSBTAG00000010532	9.13E-03	1.00E+00	-6.66E-01	7.09E-02	-1.42E+00	2.47E-05	-6.37E-01	8.70E-02	1.75E-01	1.00E+00
ENSBTAG00000017739	-4.18E-01	9.78E-01	-5.67E-01	4.43E-01	9.12E-03	1.00E+00	-1.59E-01	1.00E+00	3.36E-01	1.00E+00
ENSBTAG00000017747	-8.89E-03	1.00E+00	-1.26E-01	9.18E-01	-1.96E-01	6.61E-01	-4.55E-01	1.31E-01	-3.30E-02	1.00E+00
ENSBTAG00000031752	-1.27E-01	1.00E+00	-5.51E-01	1.02E-02	-1.08E+00	5.83E-08	-1.13E+00	1.87E-08	-2.56E-01	7.63E-01
ENSBTAG00000020252	3.33E-01	9.47E-01	-5.81E-01	2.42E-01	-7.19E-01	1.15E-01	-3.76E-01	5.21E-01	-5.12E-01	7.37E-01
ENSBTAG00000020253	6.52E-03	1.00E+00	7.54E-02	1.00E+00	-2.71E+00	1.99E-01	-1.79E-01	1.00E+00	-7.98E-01	1.00E+00

ENSBTAG00000031737	-4.50E-01	9.92E-01	-2.60E+00	5.81E-04	-2.32E+00	7.56E-04	-2.59E+00	3.03E-04	-2.14E-01	1.00E+00
ENSBTAG00000019241	1.81E-01	1.00E+00	-1.36E+00	3.95E-01	-7.39E-01	6.98E-01	-1.12E+00	4.72E-01	5.36E-01	1.00E+00
ENSBTAG00000019242	-6.84E-02	1.00E+00	-3.91E-01	4.07E-01	-4.87E-01	2.43E-01	-9.17E-01	1.17E-02	-5.97E-03	1.00E+00
ENSBTAG00000008289	-4.06E-01	5.03E-01	-1.79E+00	2.54E-12	-1.18E+00	2.15E-06	-8.04E-01	2.09E-03	5.60E-03	1.00E+00
ENSBTAG00000016370	2.66E-01	1.00E+00	-5.82E-01	8.88E-01	-1.34E+00	4.13E-01	-1.84E-01	1.00E+00	1.60E+00	3.49E-01
ENSBTAG00000046765	6.50E-01	2.32E-01	7.01E-01	3.84E-02	1.59E+00	1.99E-07	1.61E+00	2.20E-07	3.53E-01	8.00E-01
ENSBTAG00000031725	5.16E-01	1.37E-01	5.96E-01	9.29E-03	2.31E-02	1.00E+00	-4.65E-01	6.70E-02	2.70E-02	1.00E+00
ENSBTAG00000000130	-1.25E-02	1.00E+00	4.88E-01	2.14E-01	-1.80E-02	1.00E+00	-8.98E-02	1.00E+00	-2.24E-01	1.00E+00
ENSBTAG00000000131	7.08E-03	1.00E+00	8.61E-01	7.06E-06	1.34E+00	4.64E-13	6.50E-01	1.18E-03	-7.85E-02	1.00E+00
ENSBTAG00000000132	5.82E-01	1.15E-01	2.53E+00	1.01E-26	2.97E+00	1.80E-35	2.63E+00	1.64E-28	-4.36E-01	4.34E-01
ENSBTAG00000042836	0.00E+00	1.00E+00	0.00E+00	1.00E+00	-5.13E-15	1.00E+00	0.00E+00	1.00E+00	0.00E+00	1.00E+00
ENSBTAG00000045293	0.00E+00	1.00E+00	0.00E+00	1.00E+00	-5.13E-15	1.00E+00	0.00E+00	1.00E+00	0.00E+00	1.00E+00
ENSBTAG00000044856	0.00E+00	1.00E+00	0.00E+00	1.00E+00	-5.13E-15	1.00E+00	0.00E+00	1.00E+00	0.00E+00	1.00E+00
ENSBTAG00000042868	0.00E+00	1.00E+00	0.00E+00	1.00E+00	-5.13E-15	1.00E+00	0.00E+00	1.00E+00	0.00E+00	1.00E+00
ENSBTAG00000000133	2.01E-01	1.00E+00	1.04E+00	3.45E-05	4.15E-01	1.68E-01	6.02E-02	1.00E+00	-2.67E-01	8.02E-01
ENSBTAG00000000134	1.14E-01	1.00E+00	1.55E-01	6.66E-01	3.11E-01	1.98E-01	3.94E-01	8.38E-02	-2.57E-01	7.39E-01
ENSBTAG00000005461	-1.11E-01	1.00E+00	-1.18E+00	1.07E-03	-6.55E-01	8.51E-02	-1.13E+00	1.63E-03	2.17E-01	1.00E+00
ENSBTAG00000005462	-1.57E-01	1.00E+00	-5.45E-01	1.02E-02	-2.63E-01	3.14E-01	-1.39E-01	7.48E-01	1.02E-01	1.00E+00
ENSBTAG00000004222	-7.07E-01	1.12E-01	-2.77E+00	2.08E-20	-3.15E+00	1.45E-25	-1.57E+00	6.30E-08	6.78E-02	1.00E+00
ENSBTAG00000004226	-3.60E-01	5.42E-01	-9.45E-01	3.38E-05	-1.84E+00	1.26E-16	-2.22E+00	6.77E-23	-2.06E-02	1.00E+00
ENSBTAG00000013680	-3.05E-01	1.00E+00	-1.03E+00	2.31E-02	-1.79E+00	4.63E-05	-6.24E-01	2.13E-01	5.98E-01	5.85E-01
ENSBTAG00000001069	3.73E-01	6.68E-01	1.43E+00	9.71E-08	1.25E+00	3.22E-06	5.61E-01	7.01E-02	-2.06E-01	9.99E-01
ENSBTAG00000001071	-3.37E-01	7.16E-01	-8.61E-02	1.00E+00	6.77E-01	1.06E-02	5.71E-01	4.23E-02	6.98E-02	1.00E+00
ENSBTAG00000037604	-1.29E+00	9.36E-01	-1.23E+00	6.11E-01	-3.21E+00	9.44E-02	-6.79E-01	8.87E-01	-3.21E+00	4.34E-01
ENSBTAG00000008605	8.14E-01	4.65E-01	1.21E+00	1.71E-02	6.21E-01	3.32E-01	3.69E-01	7.01E-01	4.66E-01	8.85E-01
ENSBTAG00000048062	8.78E-01	4.34E-02	1.55E+00	5.63E-07	1.13E+00	3.53E-04	9.24E-01	5.13E-03	5.17E-01	5.28E-01
ENSBTAG00000014693	9.28E-01	1.13E-01	1.33E+00	5.51E-04	1.37E-01	1.00E+00	-2.28E-01	8.43E-01	2.95E-01	9.93E-01
ENSBTAG00000014695	-1.71E-01	1.00E+00	-2.76E-01	4.38E-01	-6.04E-01	2.29E-02	-3.27E-01	3.17E-01	-1.69E-01	1.00E+00
ENSBTAG00000014696	-6.09E-02	1.00E+00	-8.86E-01	2.48E-02	2.14E-01	7.81E-01	6.58E-01	7.76E-02	4.71E-01	6.44E-01
ENSBTAG00000014377	1.02E-01	1.00E+00	2.18E-01	5.47E-01	-3.65E-01	2.03E-01	1.06E-01	9.60E-01	3.40E-01	6.24E-01
ENSBTAG00000044837	0.00E+00	1.00E+00	0.00E+00	1.00E+00	-5.13E-15	1.00E+00	0.00E+00	1.00E+00	0.00E+00	1.00E+00
ENSBTAG00000014940	-7.25E-01	8.25E-01	-1.12E+00	2.12E-01	-1.56E+00	5.85E-02	-1.22E+00	1.52E-01	-3.16E-01	1.00E+00
ENSBTAG00000047214	-2.14E-01	1.00E+00	-1.19E+00	7.91E-06	-1.33E+00	4.37E-07	-8.98E-01	1.09E-03	-2.35E-02	1.00E+00
ENSBTAG00000047756	-1.81E-02	1.00E+00	-1.39E+00	4.37E-09	-1.80E+00	2.49E-14	-1.57E+00	3.10E-11	2.46E-01	8.13E-01
ENSBTAG00000008773	-6.10E-02	1.00E+00	-1.66E+00	2.14E-03	-1.79E+00	6.77E-04	-9.63E-01	9.10E-02	7.16E-01	6.01E-01

ENSBTAG00000008779	2.67E-01	1.00E+00	-1.12E+00	1.28E-01	-3.06E+00	5.18E-06	-1.31E+00	7.05E-02	5.41E-01	9.15E-01
ENSBTAG00000012475	6.68E-03	1.00E+00	2.53E+00	6.65E-02	1.14E+00	6.48E-01	1.78E+00	3.04E-01	1.15E-02	1.00E+00
ENSBTAG00000012478	4.13E+00	1.94E-02	7.67E+00	3.35E-07	8.26E+00	6.05E-08	6.90E+00	3.16E-06	1.75E+00	7.06E-01
ENSBTAG00000012436	1.70E+00	7.69E-01	8.79E-01	8.52E-01	2.27E+00	1.12E-01	1.12E+00	6.70E-01	2.24E+00	4.66E-01
ENSBTAG00000043025	0.00E+00	1.00E+00	0.00E+00	1.00E+00	-5.13E-15	1.00E+00	0.00E+00	1.00E+00	0.00E+00	1.00E+00
ENSBTAG00000003889	-1.43E-01	1.00E+00	-1.42E+00	1.16E-05	-1.45E+00	7.41E-06	-9.69E-01	4.51E-03	1.08E-01	1.00E+00
ENSBTAG00000003891	-2.10E-03	1.00E+00	5.92E-01	2.05E-02	2.32E-01	5.10E-01	-4.02E-01	1.69E-01	4.71E-03	1.00E+00
ENSBTAG00000000386	-1.03E-01	1.00E+00	-3.55E-01	1.77E-01	2.53E-01	3.97E-01	2.41E-01	4.39E-01	6.90E-02	1.00E+00
ENSBTAG00000043508	-7.08E-01	8.04E-01	-1.79E+00	1.73E-02	-2.29E+00	1.88E-03	-1.01E+00	1.69E-01	1.23E-01	1.00E+00
ENSBTAG00000001702	-5.89E-01	7.80E-01	-2.44E+00	2.71E-05	-1.04E+00	5.73E-02	-4.37E-01	5.60E-01	5.08E-01	8.00E-01
ENSBTAG00000001711	-1.28E+00	1.24E-05	-2.72E+00	1.22E-21	-1.61E+00	9.40E-10	-1.01E+00	1.55E-04	-6.26E-01	1.92E-01
ENSBTAG00000001717	-3.61E-01	1.00E+00	-7.83E-01	2.86E-01	-8.73E-01	2.03E-01	-1.56E+00	1.44E-02	-6.08E-01	8.00E-01
ENSBTAG00000007117	-6.02E-03	1.00E+00	-7.44E-01	2.96E-03	4.98E-02	1.00E+00	-1.99E-01	6.34E-01	2.01E-01	9.40E-01
ENSBTAG00000031509	1.33E-01	1.00E+00	1.26E+00	4.81E-05	1.95E+00	1.55E-10	2.11E+00	5.24E-12	5.99E-01	3.92E-01
ENSBTAG00000018809	4.58E-03	1.00E+00	-3.74E-01	3.59E-01	-4.64E-01	2.06E-01	-5.90E-01	9.21E-02	-4.44E-03	1.00E+00
ENSBTAG00000018808	-6.54E-01	6.42E-01	-1.87E+00	2.43E-04	-6.46E-01	2.71E-01	7.07E-01	2.02E-01	-2.94E-02	1.00E+00
ENSBTAG00000020688	-5.00E-01	6.16E-01	-2.43E+00	1.45E-10	-5.02E-01	2.47E-01	-7.68E-01	5.05E-02	1.05E-01	1.00E+00
ENSBTAG00000014443	2.00E-02	1.00E+00	1.58E+00	1.74E-01	2.16E-01	1.00E+00	-3.21E+00	9.96E-02	6.94E-03	1.00E+00
ENSBTAG00000012344	2.77E-01	7.66E-01	9.09E-01	3.88E-05	6.99E-01	2.06E-03	7.36E-01	1.34E-03	-1.15E-01	1.00E+00
ENSBTAG00000039299	4.35E+00	5.54E-02	6.34E+00	3.10E-07	8.00E+00	2.24E-15	5.74E+00	1.91E-05	2.70E+00	6.01E-01
ENSBTAG00000011994	4.42E-01	5.90E-01	2.07E+00	4.06E-12	3.13E+00	6.68E-25	6.45E-01	5.84E-02	-4.22E-02	1.00E+00
ENSBTAG00000021151	-3.30E-02	1.00E+00	6.97E-01	6.86E-03	8.60E-01	5.67E-04	8.03E-01	1.73E-03	1.69E-01	1.00E+00
ENSBTAG00000043649	0.00E+00	1.00E+00	0.00E+00	1.00E+00	-5.13E-15	1.00E+00	0.00E+00	1.00E+00	0.00E+00	1.00E+00
ENSBTAG00000043290	0.00E+00	1.00E+00	0.00E+00	1.00E+00	-5.13E-15	1.00E+00	0.00E+00	1.00E+00	0.00E+00	1.00E+00
ENSBTAG00000018416	1.44E-01	1.00E+00	-4.91E-01	7.62E-02	-9.47E-01	1.33E-04	-1.37E+00	2.51E-08	2.76E-02	1.00E+00
ENSBTAG00000039012	1.49E+00	2.23E-03	7.93E-01	1.09E-01	-3.70E-01	6.46E-01	1.62E-01	1.00E+00	2.57E-01	1.00E+00
ENSBTAG00000016344	4.23E-01	4.24E-01	-4.35E-01	1.34E-01	-1.33E+00	8.33E-08	-1.70E+00	1.32E-11	9.78E-03	1.00E+00
ENSBTAG00000005978	9.04E-01	2.54E-02	9.61E-01	2.03E-03	3.60E-01	3.93E-01	-1.11E-01	1.00E+00	-3.01E-02	1.00E+00
ENSBTAG00000012137	2.00E-01	9.88E-01	-3.29E-01	3.11E-01	-4.69E-01	9.75E-02	2.06E-01	6.14E-01	-9.05E-03	1.00E+00
ENSBTAG00000017815	4.95E-02	1.00E+00	-6.90E-01	2.72E-04	-1.04E+00	1.27E-08	-1.17E+00	2.34E-10	6.99E-01	4.11E-03
ENSBTAG00000006397	0.00E+00	1.00E+00	0.00E+00	1.00E+00	-5.13E-15	1.00E+00	0.00E+00	1.00E+00	0.00E+00	1.00E+00
ENSBTAG00000017823	-1.30E+00	7.42E-01	-2.54E+00	6.52E-02	-4.52E+00	3.23E-03	-5.30E-02	1.00E+00	-1.30E+00	7.46E-01
ENSBTAG00000000120	-5.95E-01	9.36E-01	-1.76E-01	1.00E+00	-4.44E-01	7.25E-01	2.80E-01	9.27E-01	-7.14E-01	8.00E-01
ENSBTAG00000005048	-8.65E-01	1.00E+00	-7.91E-01	7.88E-01	-1.03E+00	6.04E-01	-1.05E+00	6.02E-01	-3.67E-01	1.00E+00
ENSBTAG00000005051	8.03E-01	1.00E+00	7.58E-02	1.00E+00	-1.91E+00	4.28E-01	6.26E-01	9.94E-01	1.32E+00	8.97E-01

ENSBTAG00000005052	5.25E-01	5.76E-01	-1.84E+00	6.26E-07	-2.30E+00	3.92E-10	-1.05E+00	5.89E-03	9.74E-02	1.00E+00
ENSBTAG00000025088	1.66E+00	5.76E-01	-7.16E-01	9.45E-01	1.02E+00	5.91E-01	-9.39E-01	8.06E-01	-2.70E+00	6.01E-01
ENSBTAG00000019107	-1.28E-01	1.00E+00	-1.10E-02	1.00E+00	-1.07E-01	1.00E+00	3.96E-01	5.55E-01	-6.08E-01	7.19E-01
ENSBTAG00000043853	0.00E+00	1.00E+00	0.00E+00	1.00E+00	-5.13E-15	1.00E+00	0.00E+00	1.00E+00	0.00E+00	1.00E+00
ENSBTAG00000025337	-5.49E-01	1.00E+00	-1.39E+00	2.55E-01	-9.68E-01	4.28E-01	-1.29E+00	2.71E-01	-1.48E+00	6.01E-01
ENSBTAG00000009702	3.20E-01	1.00E+00	1.04E+00	4.43E-01	-1.03E+00	6.00E-01	1.05E-01	1.00E+00	-1.66E+00	7.83E-01
ENSBTAG00000037794	2.78E-01	1.00E+00	7.39E-01	6.46E-01	-3.88E+00	2.33E-02	7.64E-01	6.03E-01	-3.88E+00	2.10E-01
ENSBTAG00000018204	-3.50E+00	7.51E-02	-1.40E-01	1.00E+00	-4.10E+00	4.30E-03	-2.05E+00	1.82E-01	-1.84E+00	6.07E-01
ENSBTAG00000007090	-5.15E-01	1.00E+00	1.21E+00	5.26E-01	-1.55E+00	3.80E-01	-5.45E-01	9.77E-01	-1.80E-01	1.00E+00
ENSBTAG00000011803	5.83E-01	5.82E-01	-3.25E-01	6.92E-01	3.20E-01	6.40E-01	-6.13E-01	2.71E-01	3.49E-01	9.21E-01
ENSBTAG00000021780	-9.68E-02	1.00E+00	1.07E-01	9.85E-01	7.10E-01	7.35E-03	8.57E-01	1.08E-03	4.91E-02	1.00E+00
ENSBTAG00000046919	-3.20E-01	8.66E-01	-6.84E-01	6.21E-02	3.51E-01	4.17E-01	2.00E-01	7.89E-01	-3.05E-01	8.70E-01
ENSBTAG00000003958	-1.32E-01	1.00E+00	-8.27E-01	8.38E-03	-1.44E+00	1.15E-06	-1.57E+00	1.62E-07	-6.41E-01	2.91E-01
ENSBTAG00000046290	0.00E+00	1.00E+00	0.00E+00	1.00E+00	-5.13E-15	1.00E+00	0.00E+00	1.00E+00	0.00E+00	1.00E+00
ENSBTAG00000022509	-5.95E-01	6.62E-01	-9.46E-01	5.88E-02	-1.46E+00	2.05E-03	8.09E-02	1.00E+00	-9.96E-03	1.00E+00
ENSBTAG00000004675	-1.05E-01	1.00E+00	-9.47E-01	7.06E-05	-4.89E-01	6.06E-02	-3.08E-01	3.22E-01	3.82E-01	5.32E-01
ENSBTAG00000012103	2.54E-01	6.68E-01	7.96E-01	1.36E-05	2.81E-01	2.15E-01	1.90E-01	4.97E-01	1.67E-01	9.02E-01
ENSBTAG00000030109	0.00E+00	1.00E+00	0.00E+00	1.00E+00	-5.13E-15	1.00E+00	0.00E+00	1.00E+00	0.00E+00	1.00E+00
ENSBTAG00000035706	1.93E-02	1.00E+00	3.41E-01	9.49E-01	-5.39E-02	1.00E+00	2.53E-01	1.00E+00	-5.06E-01	1.00E+00
ENSBTAG00000021938	-4.07E-02	1.00E+00	4.28E-01	1.41E-01	-5.98E-03	1.00E+00	-4.68E-01	1.14E-01	-5.59E-02	1.00E+00
ENSBTAG00000021939	1.00E+00	3.03E-08	2.34E+00	7.07E-44	2.15E+00	7.24E-38	1.45E+00	3.22E-18	-2.26E-02	1.00E+00
ENSBTAG00000044265	0.00E+00	1.00E+00	0.00E+00	1.00E+00	-5.13E-15	1.00E+00	0.00E+00	1.00E+00	0.00E+00	1.00E+00
ENSBTAG00000031107	1.63E+00	6.58E-05	2.33E+00	6.84E-11	6.84E-01	1.10E-01	8.45E-01	4.01E-02	8.26E-02	1.00E+00
ENSBTAG00000046021	0.00E+00	1.00E+00	0.00E+00	1.00E+00	-5.13E-15	1.00E+00	0.00E+00	1.00E+00	0.00E+00	1.00E+00
ENSBTAG00000015294	4.25E-02	1.00E+00	1.55E+00	6.03E-12	2.04E+00	4.90E-20	1.65E+00	2.22E-13	-4.20E-02	1.00E+00
ENSBTAG00000007866	3.46E-01	8.23E-01	4.48E-01	2.65E-01	1.87E-02	1.00E+00	5.64E-01	1.26E-01	4.33E-01	6.86E-01
ENSBTAG00000019070	3.32E-01	1.00E+00	-2.89E-01	9.39E-01	6.03E-01	4.45E-01	1.31E+00	2.64E-02	1.22E-01	1.00E+00
ENSBTAG00000003802	6.32E-01	9.32E-01	-3.55E-01	9.61E-01	-8.18E-01	4.82E-01	4.63E-01	7.67E-01	3.89E-01	1.00E+00
ENSBTAG00000031084	1.09E+00	1.94E-01	1.81E+00	1.16E-04	6.40E-01	3.47E-01	3.93E-02	1.00E+00	1.29E+00	1.11E-01
ENSBTAG00000042416	1.91E+00	8.23E-01	0.00E+00	1.00E+00	1.78E+00	4.79E-01	0.00E+00	1.00E+00	0.00E+00	1.00E+00
ENSBTAG00000004496	6.93E-01	4.25E-02	2.46E+00	3.07E-24	2.05E+00	1.07E-17	1.31E+00	7.64E-08	1.11E-02	1.00E+00
ENSBTAG00000002605	5.04E-01	8.64E-01	2.06E+00	3.39E-05	1.80E+00	3.22E-04	1.14E+00	3.95E-02	1.65E-01	1.00E+00
ENSBTAG00000000943	-1.61E-01	1.00E+00	-1.03E+00	4.64E-05	-4.85E-01	8.69E-02	-5.55E-01	4.77E-02	3.03E-01	7.79E-01
ENSBTAG00000031071	-5.55E-01	2.04E-01	-9.33E-01	3.77E-04	-5.58E-01	4.58E-02	-7.10E-01	9.12E-03	-7.69E-03	1.00E+00
ENSBTAG00000025078	-7.60E-01	6.79E-01	8.33E-01	2.11E-01	2.44E+00	9.39E-06	8.61E-01	2.01E-01	-4.19E-01	9.99E-01

ENSBTAG00000043442	-7.15E-01	2.83E-01	8.28E-01	1.79E-02	1.73E+00	2.00E-08	3.67E-01	4.42E-01	-1.36E-01	1.00E+00
ENSBTAG00000042098	-6.50E-01	5.76E-01	9.12E-01	3.27E-02	1.94E+00	1.64E-07	1.33E+00	8.02E-04	2.48E-01	1.00E+00
ENSBTAG00000042447	-7.65E-01	1.09E-01	9.15E-01	2.08E-03	1.75E+00	1.82E-10	7.40E-01	1.80E-02	-2.45E-01	9.63E-01
ENSBTAG00000003014	-3.90E-01	7.46E-01	-1.49E+00	2.48E-06	-9.20E-01	3.95E-03	-8.55E-01	9.29E-03	-5.36E-01	4.75E-01
ENSBTAG00000017246	3.14E-01	1.00E+00	1.66E+00	3.84E-04	-7.89E-01	1.39E-01	-4.40E-01	5.23E-01	1.45E-03	1.00E+00
ENSBTAG00000013274	-3.28E-01	6.78E-01	-6.68E-01	9.05E-03	-7.07E-01	4.89E-03	-7.39E-01	3.63E-03	1.43E-02	1.00E+00
ENSBTAG00000013273	-1.31E-01	1.00E+00	2.37E-01	4.10E-01	1.11E-01	8.72E-01	-3.75E-01	1.26E-01	-1.60E-01	9.90E-01
ENSBTAG00000013271	-7.47E-02	1.00E+00	3.25E-01	4.69E-01	5.11E-01	1.72E-01	4.25E-01	2.99E-01	2.54E-01	9.62E-01
ENSBTAG00000013270	1.19E-01	1.00E+00	1.10E+00	4.47E-06	1.53E+00	8.44E-11	1.26E+00	1.46E-07	-2.79E-01	8.00E-01
ENSBTAG00000044099	-8.05E-03	1.00E+00	-9.90E-01	5.10E-05	-1.15E+00	1.62E-06	-1.28E+00	1.16E-07	1.34E-02	1.00E+00
ENSBTAG00000007581	1.33E+00	2.92E-02	1.33E+00	4.73E-03	1.31E+00	5.55E-03	1.52E+00	1.14E-03	2.66E-01	1.00E+00
ENSBTAG00000000928	-4.32E-01	5.76E-01	-9.74E-01	1.29E-03	1.31E+00	2.98E-07	2.00E+00	1.55E-15	1.94E-01	1.00E+00
ENSBTAG00000045261	0.00E+00	1.00E+00	0.00E+00	1.00E+00	1.78E+00	4.79E-01	0.00E+00	1.00E+00	0.00E+00	1.00E+00
ENSBTAG00000003439	2.21E-01	8.23E-01	1.10E+00	2.58E-09	1.06E+00	1.04E-08	4.98E-01	1.60E-02	5.96E-02	1.00E+00
ENSBTAG00000030977	3.07E-01	6.99E-01	3.22E-01	2.70E-01	2.77E-01	3.76E-01	2.25E-01	5.16E-01	3.12E-01	6.88E-01
ENSBTAG00000021113	2.15E+00	3.16E-01	2.41E+00	4.51E-02	2.90E+00	9.20E-03	4.12E+00	7.23E-05	1.02E+00	9.28E-01
ENSBTAG00000038548	1.75E+00	1.18E-01	4.40E+00	4.49E-10	2.98E+00	2.22E-05	2.62E+00	2.87E-04	1.63E+00	2.44E-01
ENSBTAG00000039161	2.07E-01	9.04E-01	-3.45E-01	1.92E-01	-1.23E+00	7.71E-09	-1.39E+00	7.92E-11	1.07E-01	1.00E+00
ENSBTAG00000016544	-2.56E-01	1.00E+00	-2.34E+00	1.55E-11	-3.61E+00	1.07E-22	-3.73E+00	1.20E-23	3.37E-01	8.20E-01
ENSBTAG00000000540	-3.24E-01	9.87E-01	-1.19E+00	8.73E-03	-1.53E+00	4.84E-04	-1.96E+00	1.89E-05	2.49E-01	1.00E+00
ENSBTAG00000006187	-5.13E-01	7.87E-01	-6.00E-01	2.71E-01	-9.43E-01	4.64E-02	-1.17E+00	1.25E-02	-1.69E-01	1.00E+00
ENSBTAG00000001014	-5.76E-01	2.01E-01	-8.07E-01	3.39E-03	1.78E-01	7.43E-01	2.69E-01	4.91E-01	2.11E-01	9.62E-01
ENSBTAG00000017790	-1.90E-01	1.00E+00	2.39E-01	5.95E-01	1.15E+00	1.58E-05	7.21E-01	1.33E-02	1.06E-01	1.00E+00
ENSBTAG00000017771	1.44E-01	1.00E+00	-7.66E-01	9.04E-03	-1.02E-02	1.00E+00	4.23E-01	2.02E-01	3.22E-01	7.93E-01
ENSBTAG00000003707	-8.30E-01	5.54E-02	-1.60E+00	2.59E-07	-1.19E+00	1.05E-04	-8.60E-01	7.88E-03	-3.75E-01	7.73E-01
ENSBTAG00000003700	-6.85E-01	4.05E-01	-2.38E+00	6.24E-08	-2.17E+00	2.27E-07	-1.34E+00	1.24E-03	3.35E-01	9.00E-01
ENSBTAG00000003705	0.00E+00	1.00E+00	0.00E+00	1.00E+00	-5.13E-15	1.00E+00	0.00E+00	1.00E+00	0.00E+00	1.00E+00
ENSBTAG00000014858	2.93E-01	5.76E-01	-1.36E-01	7.44E-01	3.66E-03	1.00E+00	-2.19E-01	4.39E-01	2.12E-01	8.00E-01
ENSBTAG00000017094	3.23E-01	4.47E-01	-5.59E-01	4.45E-03	-1.83E+00	2.52E-23	-2.28E+00	9.39E-35	3.82E-01	3.46E-01
ENSBTAG00000017090	6.58E-01	3.08E-01	1.18E+00	5.36E-04	8.54E-01	1.68E-02	1.03E+00	3.56E-03	4.02E-01	8.00E-01
ENSBTAG00000017087	-5.99E-02	1.00E+00	1.19E-01	8.69E-01	3.25E-01	2.20E-01	-4.92E-02	1.00E+00	2.90E-01	7.02E-01
ENSBTAG00000017081	-1.87E-01	1.00E+00	-2.19E-01	7.06E-01	7.26E-01	1.73E-02	1.12E+00	1.05E-04	1.69E-01	1.00E+00
ENSBTAG00000016161	-1.02E-01	1.00E+00	-1.80E-02	1.00E+00	5.49E-01	1.38E-02	9.84E-01	2.74E-06	-4.91E-02	1.00E+00
ENSBTAG00000004635	-7.77E-02	1.00E+00	-7.76E-02	1.00E+00	-3.86E-01	2.62E-01	-7.88E-01	7.59E-03	-2.25E-01	9.55E-01
ENSBTAG00000025046	-3.79E-02	1.00E+00	9.44E-01	2.97E-05	1.01E+00	6.16E-06	8.83E-01	1.22E-04	2.21E-01	8.55E-01

ENSBTAG00000006520	-2.85E-01	1.00E+00	2.59E+00	7.51E-08	2.32E+00	1.84E-06	3.70E-01	8.06E-01	1.61E-01	1.00E+00
ENSBTAG00000006517	-1.15E-01	1.00E+00	1.45E-01	7.86E-01	3.99E-01	1.24E-01	3.53E-01	2.02E-01	-2.66E-01	8.00E-01
ENSBTAG00000008802	-1.12E-01	1.00E+00	4.39E-01	1.15E-01	1.17E+00	5.73E-07	1.04E+00	1.59E-05	-1.11E-01	1.00E+00
ENSBTAG00000008801	7.94E-02	1.00E+00	2.14E-01	5.61E-01	-1.55E-01	7.69E-01	-1.10E-01	9.44E-01	1.63E-01	1.00E+00
ENSBTAG00000008798	5.70E-01	1.00E+00	1.06E+00	1.99E-01	8.51E-01	3.38E-01	-9.36E-02	1.00E+00	3.50E-01	1.00E+00
ENSBTAG00000003983	-1.85E-01	9.80E-01	-5.72E-01	1.55E-02	2.05E-01	5.45E-01	3.13E-01	2.71E-01	1.73E-01	9.83E-01
ENSBTAG00000007884	-1.15E+00	1.97E-03	-1.83E+00	3.65E-09	-1.26E+00	5.63E-05	-6.15E-01	8.73E-02	-4.70E-01	6.01E-01
ENSBTAG00000036437	0.00E+00	1.00E+00	0.00E+00	1.00E+00	-5.13E-15	1.00E+00	0.00E+00	1.00E+00	0.00E+00	1.00E+00
ENSBTAG00000004768	-2.71E-01	8.23E-01	-1.25E+00	1.53E-06	-1.72E-01	7.45E-01	-2.25E-01	5.80E-01	-3.44E-01	6.91E-01
ENSBTAG00000016432	-5.71E-01	2.00E-01	-1.35E+00	1.91E-07	-2.47E+00	1.61E-21	-3.06E+00	8.57E-31	2.18E-01	9.44E-01
ENSBTAG00000020520	4.85E-01	1.00E+00	8.23E-01	4.81E-01	1.82E+00	4.43E-02	1.35E+00	1.77E-01	6.79E-01	9.40E-01
ENSBTAG00000020517	-5.51E-01	3.07E-01	2.45E-01	5.79E-01	1.10E+00	5.06E-05	7.90E-01	6.22E-03	-2.39E-01	9.30E-01
ENSBTAG00000030820	-4.22E-01	7.68E-01	-7.64E-01	4.98E-02	-7.69E-01	4.07E-02	-7.19E-01	6.38E-02	1.76E-01	1.00E+00
ENSBTAG00000018973	-1.24E-01	1.00E+00	-3.87E-01	9.32E-02	2.88E-01	2.54E-01	2.24E-01	4.41E-01	-8.41E-02	1.00E+00
ENSBTAG00000008010	7.24E-02	1.00E+00	6.00E-02	1.00E+00	3.08E-01	4.84E-01	1.40E-01	9.56E-01	2.61E-01	9.33E-01
ENSBTAG00000008009	3.89E+00	1.52E-01	3.98E+00	2.91E-02	4.68E+00	3.13E-03	3.69E+00	5.22E-02	3.21E+00	4.41E-01
ENSBTAG00000010534	-3.08E-02	1.00E+00	-5.15E-01	7.61E-03	-3.31E-01	1.21E-01	-2.93E-01	1.99E-01	2.12E-01	8.00E-01
ENSBTAG00000015298	3.63E-01	1.00E+00	-5.94E-01	6.59E-01	2.98E-03	1.00E+00	-8.91E-01	3.99E-01	-3.45E-01	1.00E+00
ENSBTAG00000008978	-1.47E-01	1.00E+00	-5.75E-01	2.22E-02	-6.23E-01	1.17E-02	-9.06E-02	1.00E+00	6.70E-02	1.00E+00
ENSBTAG00000010559	-1.52E-02	1.00E+00	8.00E-01	2.56E-05	5.77E-01	3.42E-03	-7.75E-03	1.00E+00	-3.05E-02	1.00E+00
ENSBTAG00000010566	-1.48E-01	1.00E+00	9.01E-01	1.75E-04	1.06E+00	6.73E-06	5.36E-01	4.49E-02	-4.64E-02	1.00E+00
ENSBTAG00000046966	-1.23E-01	1.00E+00	-3.59E-01	4.43E-01	2.49E-01	6.55E-01	6.00E-01	9.97E-02	2.09E-01	1.00E+00
ENSBTAG00000010576	-2.20E-03	1.00E+00	1.14E+00	2.64E-04	7.20E-01	3.16E-02	6.19E-01	8.22E-02	-1.72E-02	1.00E+00
ENSBTAG00000020061	-9.03E-01	4.74E-02	-2.58E+00	2.57E-13	-4.15E+00	6.37E-26	-4.46E+00	5.24E-28	5.69E-01	4.34E-01
ENSBTAG00000018840	-1.91E+00	8.23E-01	-1.91E+00	4.69E-01	-1.91E+00	4.28E-01	-1.91E+00	4.39E-01	-1.91E+00	8.00E-01
ENSBTAG00000020629	-2.50E-01	8.23E-01	9.94E-01	3.16E-06	3.33E-01	2.07E-01	-3.24E-01	2.39E-01	-4.22E-01	3.84E-01
ENSBTAG00000009407	1.09E-01	1.00E+00	-6.78E-01	5.28E-02	-3.80E-01	3.71E-01	-7.64E-02	1.00E+00	4.96E-01	5.63E-01
ENSBTAG00000047186	3.53E-01	7.05E-01	1.26E+00	1.18E-06	1.46E+00	1.07E-08	1.43E+00	2.90E-08	1.81E-01	1.00E+00
ENSBTAG00000004651	2.65E-01	9.06E-01	1.24E+00	8.29E-06	1.47E+00	7.35E-08	1.46E+00	1.47E-07	-3.51E-02	1.00E+00
ENSBTAG00000012293	8.44E-02	1.00E+00	-3.71E-02	1.00E+00	1.93E-01	5.86E-01	3.32E-01	2.33E-01	-3.47E-02	1.00E+00
ENSBTAG00000042159	0.00E+00	1.00E+00	0.00E+00	1.00E+00	-5.13E-15	1.00E+00	0.00E+00	1.00E+00	0.00E+00	1.00E+00
ENSBTAG00000037660	0.00E+00	1.00E+00	0.00E+00	1.00E+00	1.78E+00	4.79E-01	0.00E+00	1.00E+00	0.00E+00	1.00E+00
ENSBTAG00000000546	-1.12E+00	6.74E-05	-2.63E+00	3.10E-25	-1.39E+00	1.68E-08	-8.06E-01	2.06E-03	2.31E-01	8.93E-01
ENSBTAG00000000731	2.70E+00	5.76E-01	3.30E+00	1.02E-01	-5.13E-15	1.00E+00	2.53E+00	2.71E-01	1.91E+00	8.00E-01
ENSBTAG00000042852	0.00E+00	1.00E+00	1.98E+00	4.43E-01	-5.13E-15	1.00E+00	0.00E+00	1.00E+00	0.00E+00	1.00E+00

ENSBTAG00000025183	-6.69E-01	9.09E-01	-9.82E-02	1.00E+00	-6.95E-01	4.86E-01	-1.35E+00	1.32E-01	-6.58E-01	8.92E-01
ENSBTAG00000002279	-2.07E-01	8.78E-01	-4.59E-02	1.00E+00	-2.50E-01	3.88E-01	-9.13E-01	1.56E-05	-2.48E-01	8.00E-01
ENSBTAG00000002267	-9.47E-02	1.00E+00	-1.37E-02	1.00E+00	4.41E-01	3.45E-02	1.61E-01	6.28E-01	8.24E-03	1.00E+00
ENSBTAG00000020070	-6.40E-01	1.46E-01	-1.33E+00	1.75E-06	-1.43E+00	1.85E-07	-1.61E+00	5.34E-09	-4.36E-01	5.70E-01
ENSBTAG00000009835	-1.17E-01	1.00E+00	-1.71E+00	3.89E-02	-2.77E+00	5.99E-04	-2.39E+00	3.37E-03	-4.26E-01	1.00E+00
ENSBTAG00000004155	-6.18E-01	8.23E-01	-9.46E-01	1.27E-01	-1.76E+00	1.55E-03	-2.20E+00	7.70E-05	-3.31E-01	1.00E+00
ENSBTAG00000015893	-1.23E+00	6.96E-06	-2.58E+00	8.25E-22	-2.56E+00	9.29E-23	-2.30E+00	2.68E-19	-2.87E-01	8.00E-01
ENSBTAG00000014355	-8.05E-01	2.87E-02	-3.33E+00	1.11E-27	-3.79E+00	2.01E-34	-3.39E+00	1.59E-29	-4.12E-01	6.01E-01
ENSBTAG00000021303	-1.88E-01	1.00E+00	-6.68E-01	9.28E-03	-1.05E-01	9.69E-01	7.04E-02	1.00E+00	-7.30E-03	1.00E+00
ENSBTAG00000021301	-4.71E-01	3.31E-01	-2.28E+00	7.80E-19	-2.00E+00	9.32E-16	-1.40E+00	1.41E-08	-2.66E-01	8.00E-01
ENSBTAG00000030060	0.00E+00	1.00E+00	0.00E+00	1.00E+00	-5.13E-15	1.00E+00	0.00E+00	1.00E+00	0.00E+00	1.00E+00
ENSBTAG00000021302	0.00E+00	1.00E+00	0.00E+00	1.00E+00	-5.13E-15	1.00E+00	0.00E+00	1.00E+00	0.00E+00	1.00E+00
ENSBTAG00000006072	5.63E-01	2.77E-01	2.26E+00	3.88E-16	2.25E+00	3.88E-16	1.99E+00	5.96E-13	3.12E-01	8.00E-01
ENSBTAG00000021723	1.16E-01	1.00E+00	9.94E-01	1.35E-05	1.31E+00	4.01E-09	6.54E-01	7.51E-03	7.95E-02	1.00E+00
ENSBTAG00000021720	-4.33E-02	1.00E+00	-2.33E-01	5.18E-01	-3.56E-01	2.32E-01	-1.47E-01	8.18E-01	-5.21E-02	1.00E+00
ENSBTAG00000009298	-5.43E-01	4.19E-01	-1.67E+00	1.10E-07	-1.79E+00	9.84E-09	-8.52E-01	1.07E-02	3.17E-01	8.19E-01
ENSBTAG00000040316	0.00E+00	1.00E+00	1.98E+00	4.43E-01	-5.13E-15	1.00E+00	0.00E+00	1.00E+00	2.71E+00	6.01E-01
ENSBTAG00000042867	0.00E+00	1.00E+00	0.00E+00	1.00E+00	-5.13E-15	1.00E+00	0.00E+00	1.00E+00	0.00E+00	1.00E+00
ENSBTAG00000013103	-5.65E-02	1.00E+00	-9.55E-01	2.51E-02	-1.23E+00	2.57E-03	-8.52E-01	5.57E-02	8.61E-01	2.66E-01
ENSBTAG00000003956	2.71E-03	1.00E+00	-4.17E-01	8.74E-01	-1.33E+00	1.72E-01	-5.46E-01	7.16E-01	4.80E-04	1.00E+00
ENSBTAG00000043979	0.00E+00	1.00E+00	0.00E+00	1.00E+00	-5.13E-15	1.00E+00	0.00E+00	1.00E+00	0.00E+00	1.00E+00
ENSBTAG00000040521	-6.37E-01	7.71E-01	-2.32E+00	1.96E-04	-2.58E+00	2.25E-05	-1.64E+00	4.95E-03	-5.69E-01	8.00E-01
ENSBTAG00000008744	-7.04E-01	3.80E-02	-3.00E+00	7.37E-33	-2.96E+00	1.83E-32	-1.50E+00	7.53E-10	1.65E-01	1.00E+00
ENSBTAG00000019498	7.19E-02	1.00E+00	-1.73E+00	1.58E-03	-1.08E+00	5.35E-02	1.88E-01	1.00E+00	1.08E-01	1.00E+00
ENSBTAG00000017409	1.31E+00	9.31E-01	-1.91E+00	4.69E-01	-1.91E+00	4.28E-01	-1.91E+00	4.39E-01	-1.91E+00	8.00E-01
ENSBTAG00000007353	-1.40E+00	7.72E-01	-1.33E+00	4.06E-01	-6.98E-01	7.28E-01	-4.29E-01	9.61E-01	-2.39E-01	1.00E+00
ENSBTAG00000045805	-1.16E+00	8.75E-01	-1.94E-01	1.00E+00	-3.88E+00	2.27E-02	-8.45E-01	6.69E-01	2.61E-01	1.00E+00
ENSBTAG00000042876	0.00E+00	1.00E+00	0.00E+00	1.00E+00	-5.13E-15	1.00E+00	0.00E+00	1.00E+00	0.00E+00	1.00E+00
ENSBTAG00000018699	-1.01E-01	1.00E+00	3.93E-01	9.23E-02	4.47E-01	4.56E-02	5.38E-01	1.42E-02	9.62E-02	1.00E+00
ENSBTAG00000006886	-5.08E-01	3.50E-01	-1.09E+00	7.91E-05	-1.25E+00	3.98E-06	-7.48E-01	9.63E-03	3.06E-01	8.00E-01
ENSBTAG00000006881	9.88E-01	3.46E-03	2.78E+00	8.83E-24	2.97E+00	6.44E-27	2.58E+00	7.05E-21	4.38E-01	5.85E-01
ENSBTAG00000043165	0.00E+00	1.00E+00	0.00E+00	1.00E+00	-5.13E-15	1.00E+00	0.00E+00	1.00E+00	0.00E+00	1.00E+00
ENSBTAG00000005824	-8.40E-02	1.00E+00	6.31E-01	1.27E-03	8.42E-01	8.13E-06	6.09E-01	2.28E-03	1.53E-01	9.74E-01
ENSBTAG00000015293	5.23E-01	1.00E+00	-7.16E-01	9.45E-01	-1.42E-01	1.00E+00	-9.33E-01	8.08E-01	8.99E-01	1.00E+00
ENSBTAG00000020979	-2.38E-01	1.00E+00	-2.29E+00	8.48E-12	-7.66E-01	3.49E-02	1.89E-01	8.58E-01	2.71E-02	1.00E+00

ENSBTAG00000017120	-7.31E-02	1.00E+00	7.85E-01	4.31E-04	8.78E-01	6.43E-05	9.99E-01	5.76E-06	4.92E-01	2.50E-01
ENSBTAG00000001919	-5.54E-01	3.83E-01	-1.83E+00	8.09E-09	-1.06E+00	7.98E-04	-2.92E-01	5.30E-01	-1.62E-02	1.00E+00
ENSBTAG00000000033	-6.25E-01	4.40E-01	-9.37E-01	1.84E-02	-1.51E+00	7.00E-05	-1.78E+00	4.11E-06	-2.54E-01	1.00E+00
ENSBTAG00000000032	-1.45E+00	6.35E-06	-1.79E+00	1.66E-09	-2.73E-01	5.14E-01	-3.73E-01	3.25E-01	-4.08E-01	6.39E-01
ENSBTAG00000047325	-1.96E+00	3.43E-04	-7.70E-01	1.21E-01	-1.11E-01	1.00E+00	-2.04E-01	9.08E-01	-4.55E-01	8.00E-01
ENSBTAG00000009864	3.75E-01	1.00E+00	-1.14E+00	3.59E-01	-1.40E+00	2.10E-01	-1.13E+00	3.38E-01	3.91E-01	1.00E+00
ENSBTAG00000011736	-3.57E-01	1.00E+00	-8.38E-01	5.92E-01	-3.33E-01	1.00E+00	8.47E-01	4.29E-01	-1.85E-01	1.00E+00
ENSBTAG00000005045	0.00E+00	1.00E+00	1.99E+00	4.43E-01	1.78E+00	4.79E-01	1.77E+00	4.97E-01	0.00E+00	1.00E+00
ENSBTAG00000018803	3.58E-02	1.00E+00	9.18E-02	9.68E-01	1.10E-01	8.88E-01	6.13E-02	1.00E+00	-1.87E-01	9.07E-01
ENSBTAG00000018802	1.69E-01	9.43E-01	1.04E+00	2.44E-08	8.23E-01	1.20E-05	8.14E-01	1.96E-05	9.67E-02	1.00E+00
ENSBTAG00000018339	-2.15E-01	1.00E+00	-3.03E-01	5.22E-01	-2.91E-01	5.48E-01	-9.97E-03	1.00E+00	-1.67E-01	1.00E+00
ENSBTAG00000044230	0.00E+00	1.00E+00	0.00E+00	1.00E+00	-5.13E-15	1.00E+00	0.00E+00	1.00E+00	0.00E+00	1.00E+00
ENSBTAG00000030047	0.00E+00	1.00E+00	0.00E+00	1.00E+00	-5.13E-15	1.00E+00	0.00E+00	1.00E+00	0.00E+00	1.00E+00
ENSBTAG00000007935	4.00E-01	2.68E-01	1.56E+00	2.47E-16	1.08E+00	2.15E-08	7.51E-01	1.81E-04	-1.23E-01	1.00E+00
ENSBTAG00000009332	-6.80E-01	1.00E+00	9.36E-01	4.63E-01	2.20E+00	1.24E-02	2.20E+00	1.39E-02	6.99E-01	9.80E-01
ENSBTAG00000044840	0.00E+00	1.00E+00	0.00E+00	1.00E+00	-5.13E-15	1.00E+00	0.00E+00	1.00E+00	0.00E+00	1.00E+00
ENSBTAG00000038765	-1.91E+00	8.23E-01	-1.91E+00	4.69E-01	-1.91E+00	4.28E-01	-1.91E+00	4.39E-01	-1.91E+00	8.00E-01
ENSBTAG00000044274	0.00E+00	1.00E+00	0.00E+00	1.00E+00	-5.13E-15	1.00E+00	0.00E+00	1.00E+00	0.00E+00	1.00E+00
ENSBTAG00000014663	0.00E+00	1.00E+00	0.00E+00	1.00E+00	-5.13E-15	1.00E+00	0.00E+00	1.00E+00	0.00E+00	1.00E+00
ENSBTAG00000036386	0.00E+00	1.00E+00	0.00E+00	1.00E+00	-5.13E-15	1.00E+00	0.00E+00	1.00E+00	0.00E+00	1.00E+00
ENSBTAG00000025009	0.00E+00	1.00E+00	0.00E+00	1.00E+00	-5.13E-15	1.00E+00	1.76E+00	4.97E-01	0.00E+00	1.00E+00
ENSBTAG00000005546	-2.42E+00	3.09E-01	-4.34E+00	1.09E-02	1.59E-01	1.00E+00	-6.49E-01	7.42E-01	-1.13E+00	8.00E-01
ENSBTAG00000045835	-1.30E+00	7.29E-01	-1.21E+00	3.81E-01	-3.42E-01	9.94E-01	2.62E-01	1.00E+00	-1.36E-01	1.00E+00
ENSBTAG00000045840	0.00E+00	1.00E+00	0.00E+00	1.00E+00	-5.13E-15	1.00E+00	0.00E+00	1.00E+00	0.00E+00	1.00E+00
ENSBTAG00000029892	-2.70E+00	5.76E-01	-7.17E-01	9.45E-01	-2.70E+00	1.99E-01	-1.70E-01	1.00E+00	-2.70E+00	6.01E-01
ENSBTAG00000039599	-2.09E+00	6.08E-05	-5.52E-01	2.93E-01	2.95E-01	6.62E-01	-1.95E-03	1.00E+00	2.01E-01	1.00E+00
ENSBTAG00000021427	-1.31E+00	1.67E-03	-1.85E+00	2.05E-07	-1.22E+00	4.98E-04	-1.31E+00	2.35E-04	-1.66E-01	1.00E+00
ENSBTAG00000009979	-1.38E+00	4.18E-02	-7.68E-01	1.84E-01	-4.41E-01	5.35E-01	-2.49E-01	8.88E-01	-1.68E-01	1.00E+00
ENSBTAG00000007603	0.00E+00	1.00E+00	0.00E+00	1.00E+00	-5.13E-15	1.00E+00	0.00E+00	1.00E+00	0.00E+00	1.00E+00
ENSBTAG00000007601	0.00E+00	1.00E+00	0.00E+00	1.00E+00	1.78E+00	4.79E-01	1.77E+00	4.97E-01	1.93E+00	8.00E-01
ENSBTAG00000000641	7.63E-02	1.00E+00	1.82E-01	7.40E-01	1.73E-01	7.59E-01	-6.24E-01	3.40E-02	-7.35E-01	9.55E-02
ENSBTAG00000043418	0.00E+00	1.00E+00	0.00E+00	1.00E+00	-5.13E-15	1.00E+00	0.00E+00	1.00E+00	0.00E+00	1.00E+00
ENSBTAG00000020321	-3.72E-01	4.85E-01	3.01E-01	2.90E-01	9.60E-01	8.09E-06	1.06E+00	8.50E-07	-1.31E-01	1.00E+00
ENSBTAG00000002949	-2.56E-01	9.32E-01	5.63E-02	1.00E+00	-3.99E-01	2.61E-01	-2.80E-01	5.05E-01	8.54E-02	1.00E+00
ENSBTAG00000013653	-6.55E-02	1.00E+00	-5.72E-01	1.15E-02	-4.94E-01	3.37E-02	1.29E-01	8.28E-01	2.90E-01	6.93E-01

ENSBTAG00000039954	-4.08E-01	5.76E-01	-1.16E+00	1.52E-05	-2.03E+00	1.39E-14	-2.52E+00	5.18E-21	-1.19E-01	1.00E+00
ENSBTAG00000036361	0.00E+00	1.00E+00	0.00E+00	1.00E+00	-5.13E-15	1.00E+00	0.00E+00	1.00E+00	0.00E+00	1.00E+00
ENSBTAG00000014202	2.02E-01	9.30E-01	3.64E-01	1.66E-01	6.00E-01	9.28E-03	5.80E-01	1.44E-02	1.89E-01	9.27E-01
ENSBTAG00000012249	-1.38E-01	1.00E+00	1.94E+00	2.08E-10	-2.47E-01	6.71E-01	-7.69E-01	3.30E-02	5.39E-02	1.00E+00
ENSBTAG00000012259	-2.79E-01	9.43E-01	3.99E-01	3.37E-01	8.84E-02	1.00E+00	2.68E-01	6.11E-01	-3.71E-02	1.00E+00
ENSBTAG00000013740	1.67E-01	1.00E+00	9.53E-01	1.41E-05	5.13E-01	3.35E-02	1.58E-01	7.41E-01	2.15E-01	8.71E-01
ENSBTAG00000001825	0.00E+00	1.00E+00	0.00E+00	1.00E+00	-5.13E-15	1.00E+00	0.00E+00	1.00E+00	0.00E+00	1.00E+00
ENSBTAG00000001828	0.00E+00	1.00E+00	0.00E+00	1.00E+00	-5.13E-15	1.00E+00	0.00E+00	1.00E+00	0.00E+00	1.00E+00
ENSBTAG00000001830	0.00E+00	1.00E+00	0.00E+00	1.00E+00	-5.13E-15	1.00E+00	0.00E+00	1.00E+00	0.00E+00	1.00E+00
ENSBTAG00000001832	0.00E+00	1.00E+00	0.00E+00	1.00E+00	-5.13E-15	1.00E+00	0.00E+00	1.00E+00	0.00E+00	1.00E+00
ENSBTAG00000013523	0.00E+00	1.00E+00	0.00E+00	1.00E+00	-5.13E-15	1.00E+00	0.00E+00	1.00E+00	0.00E+00	1.00E+00
ENSBTAG00000014199	3.68E-01	8.13E-01	2.42E-01	6.79E-01	-4.52E-01	2.63E-01	-9.55E-01	6.10E-03	-4.42E-01	6.84E-01
ENSBTAG00000011280	-2.51E-01	8.23E-01	-1.33E+00	7.82E-10	-1.19E+00	3.18E-08	2.75E-01	3.51E-01	3.83E-01	4.64E-01
ENSBTAG00000006586	-1.05E-01	1.00E+00	-1.01E-01	1.00E+00	7.82E-01	8.40E-03	1.54E+00	4.50E-08	-1.58E-01	1.00E+00
ENSBTAG00000011441	1.30E-02	1.00E+00	2.07E+00	1.94E-01	1.15E+00	6.43E-01	1.12E+00	6.68E-01	8.00E-01	1.00E+00
ENSBTAG00000011435	2.22E-02	1.00E+00	-8.50E-01	5.49E-06	-7.05E-01	1.79E-04	9.66E-02	8.99E-01	2.45E-02	1.00E+00
ENSBTAG00000042555	0.00E+00	1.00E+00	0.00E+00	1.00E+00	-5.13E-15	1.00E+00	0.00E+00	1.00E+00	0.00E+00	1.00E+00
ENSBTAG00000025001	8.47E-03	1.00E+00	4.03E-01	5.37E-02	3.66E-01	8.77E-02	4.95E-01	1.47E-02	-2.81E-01	6.06E-01
ENSBTAG00000001804	-1.97E+00	3.49E-01	-4.54E-01	8.84E-01	-9.63E-01	4.30E-01	-1.65E+00	1.53E-01	-4.68E+00	3.67E-02
ENSBTAG00000008535	3.25E-02	1.00E+00	1.45E+00	2.92E-04	1.56E+00	7.86E-05	1.37E+00	8.27E-04	5.72E-01	6.47E-01
ENSBTAG00000016911	1.67E-01	1.00E+00	-1.11E+00	1.49E-02	1.58E-01	1.00E+00	1.22E+00	2.95E-03	2.72E-01	1.00E+00
ENSBTAG00000046615	0.00E+00	1.00E+00	0.00E+00	1.00E+00	-5.13E-15	1.00E+00	0.00E+00	1.00E+00	0.00E+00	1.00E+00
ENSBTAG00000013469	-4.46E-01	1.00E+00	-9.66E-01	2.10E-01	-9.40E-01	2.01E-01	-1.64E+00	2.12E-02	-7.30E-02	1.00E+00
ENSBTAG00000047623	0.00E+00	1.00E+00	0.00E+00	1.00E+00	-5.13E-15	1.00E+00	0.00E+00	1.00E+00	0.00E+00	1.00E+00
ENSBTAG00000048067	2.15E+00	7.73E-03	1.06E+00	2.30E-01	2.09E+00	1.43E-03	3.31E+00	2.44E-08	9.64E-01	6.96E-01
ENSBTAG00000011532	1.16E-01	1.00E+00	-9.60E-01	1.74E-04	-4.35E-01	1.41E-01	-1.93E-01	6.82E-01	1.54E-01	1.00E+00
ENSBTAG00000046569	-2.72E-01	9.66E-01	-1.11E+00	8.51E-04	-1.09E+00	9.59E-04	-6.99E-01	5.28E-02	-2.27E-01	1.00E+00
ENSBTAG00000011539	-2.69E-01	9.28E-01	-1.46E+00	7.25E-07	-1.59E+00	4.98E-08	-4.05E-01	2.85E-01	1.09E-01	1.00E+00
ENSBTAG00000003830	-2.76E-02	1.00E+00	7.22E-02	1.00E+00	1.06E-01	9.59E-01	2.06E-01	5.96E-01	-3.98E-01	5.06E-01
ENSBTAG00000006499	-3.16E-01	8.23E-01	-7.71E-01	9.92E-03	-1.24E+00	1.31E-05	-8.10E-02	1.00E+00	4.99E-01	4.41E-01
ENSBTAG00000030591	-6.37E-01	4.18E-02	-2.02E-01	5.70E-01	-2.07E-02	1.00E+00	-1.80E-01	6.52E-01	-3.00E-01	7.05E-01
ENSBTAG00000044056	3.45E-01	1.00E+00	4.35E+00	3.68E-03	2.96E+00	5.53E-02	5.45E-01	1.00E+00	-7.07E-02	1.00E+00
ENSBTAG00000003229	-9.49E-02	1.00E+00	-8.45E-02	1.00E+00	-2.99E-01	3.46E-01	-2.48E-01	4.87E-01	-5.79E-01	1.77E-01
ENSBTAG00000045022	0.00E+00	1.00E+00	0.00E+00	1.00E+00	-5.13E-15	1.00E+00	0.00E+00	1.00E+00	0.00E+00	1.00E+00
ENSBTAG00000043359	0.00E+00	1.00E+00	0.00E+00	1.00E+00	-5.13E-15	1.00E+00	0.00E+00	1.00E+00	0.00E+00	1.00E+00

ENSBTAG00000030587	-1.28E-01	1.00E+00	-3.89E-01	2.02E-01	-4.23E-02	1.00E+00	5.26E-01	6.31E-02	-9.15E-02	1.00E+00
ENSBTAG00000024983	-9.31E-03	1.00E+00	-1.00E+00	1.08E-04	-1.50E+00	3.04E-09	-1.20E+00	2.83E-06	-4.40E-02	1.00E+00
ENSBTAG00000019347	5.91E-01	9.31E-01	3.19E-01	9.45E-01	-1.12E+00	2.64E-01	5.16E-01	6.48E-01	-9.74E-02	1.00E+00
ENSBTAG00000002036	-1.33E-01	1.00E+00	2.22E-01	7.58E-01	-2.40E-01	7.06E-01	-1.12E+00	1.83E-03	1.72E-01	1.00E+00
ENSBTAG00000002035	4.86E-01	1.00E+00	-5.61E-01	6.96E-01	-6.68E-01	5.53E-01	-1.24E+00	2.05E-01	7.78E-01	7.69E-01
ENSBTAG00000002060	8.89E-02	1.00E+00	2.11E-01	5.50E-01	8.73E-02	1.00E+00	2.23E-02	1.00E+00	-3.41E-01	6.01E-01
ENSBTAG00000008366	3.59E-01	1.00E+00	-1.04E+00	4.79E-01	-9.16E-01	5.19E-01	-1.32E+00	3.29E-01	-7.55E-01	9.81E-01
ENSBTAG00000008368	1.21E+00	8.50E-01	1.92E+00	1.26E-01	-1.53E-01	1.00E+00	7.00E-01	8.54E-01	1.21E+00	8.24E-01
ENSBTAG00000008371	9.69E-02	1.00E+00	-3.92E-01	4.66E-01	-1.98E-01	8.79E-01	-3.31E-01	5.85E-01	5.36E-01	6.23E-01
ENSBTAG00000013236	1.75E-01	1.00E+00	1.41E+00	3.78E-07	1.77E+00	1.40E-10	1.41E+00	5.08E-07	2.39E-01	9.37E-01
ENSBTAG00000044355	3.88E+00	1.42E-01	3.31E+00	1.02E-01	-5.13E-15	1.00E+00	0.00E+00	1.00E+00	1.93E+00	8.00E-01
ENSBTAG00000013238	1.34E-01	1.00E+00	8.29E-01	1.58E-03	9.43E-01	2.52E-04	8.95E-01	6.84E-04	4.65E-01	4.36E-01
ENSBTAG00000004119	0.00E+00	1.00E+00	1.98E+00	4.43E-01	1.78E+00	4.79E-01	0.00E+00	1.00E+00	0.00E+00	1.00E+00
ENSBTAG00000006035	3.21E-01	1.00E+00	-6.50E-01	4.48E-01	-2.60E+00	2.54E-04	-1.13E+00	1.16E-01	8.12E-01	6.13E-01
ENSBTAG00000011729	1.09E-01	1.00E+00	2.25E-01	4.69E-01	2.29E-01	4.52E-01	1.48E-01	7.51E-01	2.47E-01	8.00E-01
ENSBTAG00000011730	-8.53E-02	1.00E+00	5.73E-01	2.75E-01	-3.59E-01	5.96E-01	-1.43E-01	1.00E+00	5.07E-01	7.78E-01
ENSBTAG00000011731	-1.66E+00	7.82E-01	-1.60E+00	4.10E-01	-1.80E+00	2.99E-01	-3.58E+00	4.96E-02	1.74E+00	3.27E-01
ENSBTAG00000011732	-1.89E-01	9.42E-01	-1.05E+00	6.20E-07	-1.36E+00	6.66E-11	-1.21E+00	1.01E-08	9.69E-02	1.00E+00
ENSBTAG00000021798	-9.99E-02	1.00E+00	-2.15E+00	5.11E-12	-2.34E+00	6.43E-14	-5.40E-01	1.52E-01	1.04E+00	2.07E-02
ENSBTAG00000021802	-2.20E-02	1.00E+00	-6.36E-01	5.70E-02	-6.39E-01	5.30E-02	1.37E-01	9.48E-01	8.98E-01	3.90E-02
ENSBTAG00000017366	-5.90E-01	6.14E-01	-2.51E+00	3.78E-09	-3.18E+00	1.96E-13	-8.34E-01	7.77E-02	1.52E+00	5.89E-03
ENSBTAG00000011003	5.47E-02	1.00E+00	3.88E-01	4.43E-01	1.91E-01	8.68E-01	-2.09E-01	8.46E-01	-4.21E-01	8.00E-01
ENSBTAG00000030523	-9.69E-02	1.00E+00	-1.60E+00	1.05E-02	-1.14E+00	5.53E-02	-4.61E-01	5.50E-01	-6.48E-01	7.68E-01
ENSBTAG00000012854	2.09E-01	1.00E+00	-1.45E+00	9.13E-05	-1.30E+00	4.97E-04	-1.71E+00	4.08E-06	2.61E-01	1.00E+00
ENSBTAG00000013855	-1.60E-01	1.00E+00	-1.28E+00	2.97E-07	-2.46E+00	7.58E-23	-1.67E+00	2.04E-11	1.09E-01	1.00E+00
ENSBTAG00000039933	-1.91E+00	8.23E-01	6.97E-02	1.00E+00	6.39E-01	9.79E-01	-1.91E+00	4.39E-01	5.52E-04	1.00E+00
ENSBTAG00000010070	-5.02E-01	1.00E+00	8.67E-02	1.00E+00	1.15E+00	4.08E-01	1.78E+00	9.00E-02	6.90E-01	1.00E+00
ENSBTAG00000021461	-2.27E-01	8.23E-01	5.02E-02	1.00E+00	8.93E-03	1.00E+00	-5.52E-02	1.00E+00	-1.68E-01	9.37E-01
ENSBTAG00000021462	-1.31E-01	1.00E+00	1.81E+00	4.83E-02	-7.84E-01	6.67E-01	9.58E-01	4.42E-01	-1.76E+00	6.01E-01
ENSBTAG00000021468	-1.03E-01	1.00E+00	7.91E-02	1.00E+00	-1.94E-01	6.69E-01	-8.83E-03	1.00E+00	3.41E-01	6.88E-01
ENSBTAG00000045067	0.00E+00	1.00E+00	0.00E+00	1.00E+00	-5.13E-15	1.00E+00	0.00E+00	1.00E+00	0.00E+00	1.00E+00
ENSBTAG00000010745	-6.25E-01	1.50E-01	-2.28E+00	2.41E-17	-3.49E+00	1.57E-35	-1.96E+00	2.61E-13	1.80E-01	1.00E+00
ENSBTAG00000012178	-2.07E+00	3.21E-03	-1.42E+00	1.95E-02	-2.38E+00	4.74E-05	-1.34E-01	1.00E+00	1.59E+00	7.31E-02
ENSBTAG00000010375	-3.82E-01	5.48E-01	-1.23E+00	3.51E-07	-5.80E-01	2.64E-02	3.76E-02	1.00E+00	1.15E-02	1.00E+00
ENSBTAG00000006250	1.03E-01	1.00E+00	4.23E-01	7.96E-02	7.89E-01	2.36E-04	9.48E-01	8.95E-06	2.35E-01	8.00E-01

ENSBTAG00000015232	-1.43E+00	1.40E-03	-4.21E+00	7.92E-21	-3.99E+00	1.68E-20	-2.02E+00	1.43E-07	-7.49E-01	3.44E-01
ENSBTAG00000038126	1.19E-01	1.00E+00	5.83E-01	4.43E-02	4.79E-01	1.15E-01	6.58E-01	2.17E-02	4.40E-01	5.22E-01
ENSBTAG00000010384	-1.30E+00	8.72E-02	-3.44E-01	6.95E-01	-6.91E-01	2.28E-01	-9.33E-01	8.89E-02	-6.08E-01	7.38E-01
ENSBTAG00000012500	-6.17E-01	1.59E-01	-2.73E+00	1.60E-23	-1.47E+00	3.60E-08	-1.27E-01	9.43E-01	4.24E-01	5.79E-01
ENSBTAG00000018759	-1.94E-01	1.00E+00	-1.14E-01	1.00E+00	2.80E-01	9.78E-01	1.48E-01	1.00E+00	-2.11E-01	1.00E+00
ENSBTAG00000019262	-5.53E-02	1.00E+00	-9.41E-01	1.06E-01	-8.06E-01	1.83E-01	-9.27E-01	1.19E-01	-6.98E-01	6.64E-01
ENSBTAG00000008611	1.35E-01	1.00E+00	-4.04E-01	1.37E-01	-2.16E-01	5.39E-01	4.00E-01	1.52E-01	7.08E-01	4.17E-02
ENSBTAG00000007958	5.88E-01	1.00E+00	2.68E+00	2.35E-03	2.83E+00	1.21E-03	2.46E+00	6.35E-03	3.49E-01	1.00E+00
ENSBTAG00000015133	1.29E+00	6.33E-02	5.59E-01	4.28E-01	3.38E-01	7.45E-01	7.77E-01	1.96E-01	-2.25E-01	1.00E+00
ENSBTAG00000015308	2.32E-01	7.31E-01	6.95E-01	9.88E-05	1.08E+00	2.97E-10	6.37E-01	4.80E-04	1.54E-02	1.00E+00
ENSBTAG00000030539	5.64E-01	1.00E+00	-5.06E-01	8.20E-01	-2.99E-01	1.00E+00	-1.56E+00	1.54E-01	-3.97E-01	1.00E+00
ENSBTAG00000040279	-6.54E-01	1.00E+00	-1.89E+00	2.58E-01	-2.09E+00	1.78E-01	-1.34E+00	4.22E-01	-2.55E-01	1.00E+00
ENSBTAG00000030542	-1.91E+00	8.23E-01	-1.91E+00	4.69E-01	-1.91E+00	4.28E-01	-1.47E-01	1.00E+00	8.10E-01	1.00E+00
ENSBTAG00000030546	1.92E+00	8.23E-01	1.99E+00	4.43E-01	-5.13E-15	1.00E+00	0.00E+00	1.00E+00	0.00E+00	1.00E+00
ENSBTAG00000030548	-1.91E+00	8.23E-01	7.57E-02	1.00E+00	-1.91E+00	4.28E-01	-1.91E+00	4.39E-01	6.35E-03	1.00E+00
ENSBTAG00000030555	0.00E+00	1.00E+00	0.00E+00	1.00E+00	-5.13E-15	1.00E+00	1.76E+00	4.97E-01	0.00E+00	1.00E+00
ENSBTAG00000020824	3.06E-01	1.00E+00	7.72E-01	1.71E-01	8.11E-01	1.37E-01	1.33E-01	1.00E+00	8.74E-01	4.29E-01
ENSBTAG00000015518	-1.91E+00	8.23E-01	-1.91E+00	4.69E-01	-1.31E-01	1.00E+00	-1.91E+00	4.39E-01	-1.91E+00	8.00E-01
ENSBTAG00000007794	0.00E+00	1.00E+00	0.00E+00	1.00E+00	1.78E+00	4.79E-01	0.00E+00	1.00E+00	0.00E+00	1.00E+00
ENSBTAG00000046458	0.00E+00	1.00E+00	0.00E+00	1.00E+00	-5.13E-15	1.00E+00	0.00E+00	1.00E+00	0.00E+00	1.00E+00
ENSBTAG00000043117	-1.91E+00	8.23E-01	-1.91E+00	4.69E-01	-1.91E+00	4.28E-01	-1.91E+00	4.39E-01	7.16E-03	1.00E+00
ENSBTAG00000040460	-2.71E+00	5.76E-01	9.72E-01	6.55E-01	-2.71E+00	1.99E-01	-1.78E-01	1.00E+00	5.08E-01	1.00E+00
ENSBTAG00000020078	1.92E+00	8.23E-01	1.99E+00	4.43E-01	1.78E+00	4.79E-01	0.00E+00	1.00E+00	1.91E+00	8.00E-01
ENSBTAG00000017408	-1.91E+00	8.23E-01	-1.91E+00	4.69E-01	-1.91E+00	4.28E-01	-1.41E-01	1.00E+00	-1.91E+00	8.00E-01
ENSBTAG00000047515	0.00E+00	1.00E+00	0.00E+00	1.00E+00	-5.13E-15	1.00E+00	0.00E+00	1.00E+00	0.00E+00	1.00E+00
ENSBTAG00000046225	0.00E+00	1.00E+00	0.00E+00	1.00E+00	-5.13E-15	1.00E+00	0.00E+00	1.00E+00	0.00E+00	1.00E+00
ENSBTAG00000048083	-1.91E+00	8.23E-01	-1.91E+00	4.69E-01	-1.91E+00	4.28E-01	-1.91E+00	4.39E-01	-1.91E+00	8.00E-01
ENSBTAG00000024995	0.00E+00	1.00E+00	0.00E+00	1.00E+00	-5.13E-15	1.00E+00	0.00E+00	1.00E+00	0.00E+00	1.00E+00
ENSBTAG00000030559	0.00E+00	1.00E+00	0.00E+00	1.00E+00	-5.13E-15	1.00E+00	0.00E+00	1.00E+00	0.00E+00	1.00E+00
ENSBTAG00000038759	0.00E+00	1.00E+00	0.00E+00	1.00E+00	-5.13E-15	1.00E+00	0.00E+00	1.00E+00	0.00E+00	1.00E+00
ENSBTAG00000037665	0.00E+00	1.00E+00	0.00E+00	1.00E+00	-5.13E-15	1.00E+00	0.00E+00	1.00E+00	0.00E+00	1.00E+00
ENSBTAG00000039644	0.00E+00	1.00E+00	2.79E+00	2.10E-01	-5.13E-15	1.00E+00	0.00E+00	1.00E+00	0.00E+00	1.00E+00
ENSBTAG00000024839	-1.78E-01	1.00E+00	-5.05E-04	1.00E+00	-1.01E+00	2.21E-01	-1.53E+00	5.91E-02	-2.08E+00	1.28E-01
ENSBTAG00000046075	0.00E+00	1.00E+00	0.00E+00	1.00E+00	-5.13E-15	1.00E+00	0.00E+00	1.00E+00	0.00E+00	1.00E+00
ENSBTAG00000046907	0.00E+00	1.00E+00	0.00E+00	1.00E+00	-5.13E-15	1.00E+00	0.00E+00	1.00E+00	0.00E+00	1.00E+00

ENSBTAG00000037943	0.00E+00	1.00E+00	0.00E+00	1.00E+00	-5.13E-15	1.00E+00	0.00E+00	1.00E+00	0.00E+00	1.00E+00
ENSBTAG00000040118	0.00E+00	1.00E+00	0.00E+00	1.00E+00	-5.13E-15	1.00E+00	0.00E+00	1.00E+00	0.00E+00	1.00E+00
ENSBTAG00000038386	0.00E+00	1.00E+00	0.00E+00	1.00E+00	-5.13E-15	1.00E+00	0.00E+00	1.00E+00	0.00E+00	1.00E+00
ENSBTAG00000039587	0.00E+00	1.00E+00	0.00E+00	1.00E+00	-5.13E-15	1.00E+00	0.00E+00	1.00E+00	0.00E+00	1.00E+00
ENSBTAG00000039880	0.00E+00	1.00E+00	0.00E+00	1.00E+00	-5.13E-15	1.00E+00	0.00E+00	1.00E+00	0.00E+00	1.00E+00
ENSBTAG00000040229	0.00E+00	1.00E+00	0.00E+00	1.00E+00	-5.13E-15	1.00E+00	0.00E+00	1.00E+00	0.00E+00	1.00E+00
ENSBTAG00000001672	0.00E+00	1.00E+00	0.00E+00	1.00E+00	1.78E+00	4.79E-01	0.00E+00	1.00E+00	0.00E+00	1.00E+00
ENSBTAG00000037553	0.00E+00	1.00E+00	1.98E+00	4.43E-01	-5.13E-15	1.00E+00	0.00E+00	1.00E+00	1.91E+00	8.00E-01
ENSBTAG00000030519	-1.91E+00	8.23E-01	-1.91E+00	4.69E-01	-1.91E+00	4.28E-01	-1.91E+00	4.39E-01	-1.91E+00	8.00E-01
ENSBTAG00000037560	-1.91E+00	8.23E-01	7.58E-02	1.00E+00	-1.91E+00	4.28E-01	1.51E+00	4.48E-01	6.34E-03	1.00E+00
ENSBTAG00000013685	0.00E+00	1.00E+00	0.00E+00	1.00E+00	-5.13E-15	1.00E+00	0.00E+00	1.00E+00	0.00E+00	1.00E+00
ENSBTAG00000000573	-7.86E-01	1.00E+00	-2.71E+00	2.42E-01	-9.29E-01	8.06E-01	1.44E+00	3.29E-01	3.88E-04	1.00E+00
ENSBTAG00000016384	0.00E+00	1.00E+00	1.98E+00	4.43E-01	-5.13E-15	1.00E+00	1.77E+00	4.97E-01	1.93E+00	8.00E-01
ENSBTAG00000006807	0.00E+00	1.00E+00	0.00E+00	1.00E+00	-5.13E-15	1.00E+00	0.00E+00	1.00E+00	0.00E+00	1.00E+00
ENSBTAG00000046124	2.72E+00	5.76E-01	1.98E+00	4.43E-01	1.78E+00	4.79E-01	0.00E+00	1.00E+00	0.00E+00	1.00E+00
ENSBTAG00000004903	0.00E+00	1.00E+00	0.00E+00	1.00E+00	-5.13E-15	1.00E+00	1.76E+00	4.97E-01	3.60E+00	3.09E-01
ENSBTAG00000004905	-7.23E-01	8.23E-01	-1.56E+00	2.37E-02	-1.87E+00	5.46E-03	-1.20E+00	1.06E-01	-5.02E-01	9.70E-01
ENSBTAG00000047165	-1.91E+00	8.23E-01	-1.91E+00	4.69E-01	-1.37E-01	1.00E+00	-1.91E+00	4.39E-01	-1.91E+00	8.00E-01
ENSBTAG00000007583	1.57E-02	1.00E+00	9.52E-02	1.00E+00	5.02E-01	9.47E-01	-1.79E-01	1.00E+00	-5.01E-01	1.00E+00
ENSBTAG00000033766	-1.91E+00	8.23E-01	8.82E-01	8.52E-01	-1.24E-01	1.00E+00	6.23E-01	9.94E-01	7.16E-03	1.00E+00
ENSBTAG00000006806	1.41E-02	1.00E+00	2.07E+00	2.01E-01	1.14E+00	6.50E-01	3.57E+00	1.13E-03	3.91E+00	4.11E-03
ENSBTAG00000047262	0.00E+00	1.00E+00	0.00E+00	1.00E+00	-5.13E-15	1.00E+00	0.00E+00	1.00E+00	0.00E+00	1.00E+00
ENSBTAG00000042827	0.00E+00	1.00E+00	0.00E+00	1.00E+00	-5.13E-15	1.00E+00	0.00E+00	1.00E+00	0.00E+00	1.00E+00
ENSBTAG00000042897	0.00E+00	1.00E+00	0.00E+00	1.00E+00	-5.13E-15	1.00E+00	0.00E+00	1.00E+00	0.00E+00	1.00E+00
ENSBTAG00000002282	5.39E-01	5.76E-01	2.03E+00	9.30E-09	9.35E-01	1.26E-02	-3.86E-02	1.00E+00	-3.24E-01	8.97E-01
ENSBTAG00000017663	-2.70E+00	5.78E-01	9.81E-01	6.56E-01	2.23E+00	4.56E-02	2.66E+00	9.29E-03	6.24E-03	1.00E+00
ENSBTAG00000017685	-2.16E-01	1.00E+00	-2.08E+00	1.44E-10	-2.27E+00	2.10E-12	-1.30E+00	8.01E-05	-2.04E-01	1.00E+00
ENSBTAG00000025752	-1.87E-01	1.00E+00	-7.31E-01	1.69E-01	-1.32E+00	5.15E-03	-9.10E-01	7.01E-02	2.95E-01	1.00E+00
ENSBTAG00000011454	-2.11E-01	1.00E+00	-9.47E-01	3.57E-03	-1.01E+00	1.31E-03	-1.17E+00	2.20E-04	-6.43E-02	1.00E+00
ENSBTAG00000011456	-1.24E-01	1.00E+00	5.91E-01	1.34E-02	9.67E-01	1.48E-05	2.08E-01	5.61E-01	2.00E-03	1.00E+00
ENSBTAG00000011459	0.00E+00	1.00E+00	1.98E+00	4.43E-01	4.53E+00	3.97E-03	3.03E+00	1.50E-01	1.91E+00	8.00E-01
ENSBTAG00000011460	-7.50E-02	1.00E+00	1.50E+00	4.93E-05	1.15E+00	2.47E-03	1.17E+00	2.44E-03	4.66E-01	7.91E-01
ENSBTAG00000016740	-6.97E-01	4.65E-01	-2.40E+00	1.47E-08	-1.51E+00	3.72E-04	-7.17E-01	1.51E-01	-1.03E+00	1.66E-01
ENSBTAG00000013698	9.33E-01	5.76E-01	-5.91E-01	6.67E-01	-8.63E-01	4.15E-01	-1.51E+00	1.19E-01	3.56E-01	1.00E+00
ENSBTAG00000025762	-8.17E-02	1.00E+00	4.39E-01	1.41E-01	6.22E-01	2.21E-02	6.19E-01	2.60E-02	2.96E-01	8.00E-01

ENSBTAG00000002654	7.19E-02	1.00E+00	5.09E-01	1.71E-02	7.42E-01	2.18E-04	5.63E-01	8.16E-03	-3.26E-01	5.52E-01
ENSBTAG00000002657	1.40E-02	1.00E+00	4.82E-01	5.03E-02	6.77E-01	3.06E-03	7.71E-01	7.36E-04	-1.82E-01	9.62E-01
ENSBTAG00000046162	-2.93E-01	1.00E+00	-1.44E+00	3.88E-02	-4.73E-01	5.87E-01	4.53E-02	1.00E+00	4.23E-01	9.40E-01
ENSBTAG00000046580	2.58E-01	1.00E+00	6.77E-02	1.00E+00	-1.11E+00	3.24E-02	-1.65E+00	9.50E-04	1.51E-01	1.00E+00
ENSBTAG00000044019	6.58E-01	2.17E-02	2.34E+00	1.60E-29	1.34E+00	1.09E-10	6.24E-01	6.56E-03	2.20E-01	8.34E-01
ENSBTAG00000024979	1.03E-02	1.00E+00	2.48E+00	1.83E-02	1.48E+00	2.97E-01	3.22E-01	1.00E+00	1.63E-03	1.00E+00
ENSBTAG00000006982	7.06E-02	1.00E+00	7.57E-01	4.50E-05	1.10E+00	1.04E-09	1.03E+00	1.69E-08	-4.06E-02	1.00E+00
ENSBTAG00000001195	1.99E+00	3.62E-01	4.20E+00	3.12E-07	4.37E+00	3.31E-08	3.77E+00	1.10E-05	2.43E+00	1.92E-01
ENSBTAG00000000665	6.75E-01	1.00E+00	1.40E+00	2.62E-01	-1.44E+00	4.79E-01	1.80E-01	1.00E+00	-3.22E+00	4.34E-01
ENSBTAG00000000666	-1.49E-01	1.00E+00	-7.55E-01	5.15E-03	-1.15E+00	8.68E-06	-1.32E+00	3.10E-07	-3.22E-01	7.68E-01
ENSBTAG00000010125	5.56E-01	1.13E-01	-1.86E-01	6.39E-01	6.90E-01	3.26E-03	3.08E-01	3.05E-01	6.64E-01	5.98E-02
ENSBTAG00000009496	8.84E-01	7.73E-03	9.43E-01	4.67E-04	1.38E+00	1.06E-07	6.78E-01	1.87E-02	2.54E-01	8.71E-01
ENSBTAG00000021523	1.75E+00	2.91E-15	3.33E+00	4.51E-50	3.26E+00	1.17E-48	2.68E+00	1.21E-34	5.90E-01	1.02E-01
ENSBTAG00000039684	5.49E-01	3.80E-01	7.93E-01	1.38E-02	9.28E-01	3.04E-03	1.00E+00	1.48E-03	-1.91E-01	1.00E+00
ENSBTAG00000019218	-6.89E-02	1.00E+00	-4.40E-01	1.04E-01	-9.16E-01	1.09E-04	-9.40E-01	8.75E-05	-2.66E-01	8.00E-01
ENSBTAG00000046078	0.00E+00	1.00E+00	0.00E+00	1.00E+00	-5.13E-15	1.00E+00	0.00E+00	1.00E+00	0.00E+00	1.00E+00
ENSBTAG00000047956	-1.05E-01	1.00E+00	-3.78E-01	1.84E-01	-1.97E-01	6.07E-01	3.33E-01	2.59E-01	6.85E-02	1.00E+00
ENSBTAG00000047604	1.40E+00	2.77E-02	1.84E+00	7.07E-05	4.20E-01	6.20E-01	1.15E-01	1.00E+00	4.07E-01	9.80E-01
ENSBTAG00000046746	-1.56E-01	1.00E+00	-2.02E-01	6.72E-01	-3.77E-01	2.48E-01	-1.19E-01	9.56E-01	-2.95E-02	1.00E+00
ENSBTAG00000046670	-6.92E-02	1.00E+00	-9.71E-01	1.61E-06	-1.63E+00	3.26E-16	-6.07E-01	4.81E-03	2.92E-01	6.28E-01
ENSBTAG00000020907	-3.54E-02	1.00E+00	1.02E+00	3.30E-03	9.18E-01	8.25E-03	4.47E-01	3.30E-01	7.56E-02	1.00E+00
ENSBTAG00000032493	-2.73E-01	8.23E-01	-6.36E-01	1.32E-02	-5.79E-01	2.64E-02	-3.02E-01	3.62E-01	2.12E-01	9.13E-01
ENSBTAG00000048152	-3.34E-01	7.40E-01	-1.31E-01	9.12E-01	2.31E-01	5.69E-01	6.41E-01	2.22E-02	-4.71E-01	4.39E-01
ENSBTAG00000030347	-5.13E-01	6.14E-01	-1.74E+00	3.60E-06	-1.50E+00	4.89E-05	-8.27E-01	3.76E-02	5.13E-01	6.07E-01
ENSBTAG00000019090	-9.93E-01	8.86E-02	-2.48E+00	5.01E-10	-2.12E+00	7.22E-08	-2.01E+00	4.09E-07	-2.96E-01	9.93E-01
ENSBTAG00000019095	-5.47E-01	6.22E-01	-1.20E+00	5.43E-03	-2.74E+00	3.10E-09	-2.11E+00	1.49E-06	6.21E-01	4.62E-01
ENSBTAG00000019097	3.53E-02	1.00E+00	-6.98E-01	5.15E-02	-1.50E+00	5.27E-06	-1.66E+00	5.99E-07	5.58E-01	4.57E-01
ENSBTAG00000021918	-2.03E-01	9.13E-01	-6.60E-01	3.41E-03	-3.92E-01	1.18E-01	-1.82E-01	6.24E-01	2.15E-01	8.40E-01
ENSBTAG00000019903	-6.35E-02	1.00E+00	-6.29E-01	7.08E-02	-7.45E-01	2.58E-02	-6.63E-01	5.88E-02	-6.09E-01	3.72E-01
ENSBTAG00000019906	-7.21E-02	1.00E+00	-2.79E-01	3.00E-01	-5.48E-01	1.26E-02	-4.05E-01	9.29E-02	-5.46E-02	1.00E+00
ENSBTAG00000019907	-4.36E-01	8.23E-01	1.33E+00	1.23E-04	-3.91E-01	4.66E-01	-2.54E-01	7.49E-01	3.38E-01	8.82E-01
ENSBTAG00000019911	-1.21E-01	1.00E+00	-4.95E-01	3.98E-02	-7.07E-01	1.70E-03	-4.99E-01	4.18E-02	-2.14E-01	8.54E-01
ENSBTAG00000019912	6.38E-02	1.00E+00	-2.26E-01	5.90E-01	-1.26E-01	9.18E-01	-8.27E-02	1.00E+00	-9.08E-01	1.34E-02
ENSBTAG00000019914	-1.34E-03	1.00E+00	1.25E-01	7.94E-01	5.41E-01	8.26E-03	6.01E-01	3.46E-03	-1.65E-02	1.00E+00
ENSBTAG00000019918	1.80E-01	9.74E-01	1.40E+00	9.78E-12	2.03E+00	4.30E-23	1.91E+00	1.62E-20	1.94E-02	1.00E+00

ENSBTAG00000030334	1.72E-01	1.00E+00	3.68E+00	4.64E-27	2.60E+00	2.98E-15	7.38E-01	4.77E-02	1.04E-02	1.00E+00
ENSBTAG00000030333	-9.28E-02	1.00E+00	-4.04E-01	9.85E-02	-8.44E-01	7.87E-05	-9.02E-01	2.80E-05	-2.10E-01	8.47E-01
ENSBTAG00000046264	0.00E+00	1.00E+00	0.00E+00	1.00E+00	-5.13E-15	1.00E+00	0.00E+00	1.00E+00	0.00E+00	1.00E+00
ENSBTAG00000046001	0.00E+00	1.00E+00	0.00E+00	1.00E+00	-5.13E-15	1.00E+00	0.00E+00	1.00E+00	0.00E+00	1.00E+00
ENSBTAG00000001041	2.12E-01	8.94E-01	5.03E-01	3.65E-02	2.98E-01	2.92E-01	2.40E-01	4.57E-01	9.98E-01	1.94E-04
ENSBTAG00000039321	1.56E-01	1.00E+00	8.91E-01	1.14E-03	3.66E-01	2.85E-01	5.23E-01	9.25E-02	1.05E+00	2.71E-03
ENSBTAG00000010184	1.10E-01	1.00E+00	4.65E-01	4.36E-01	2.04E+00	1.23E-06	8.24E-01	8.73E-02	4.08E-01	8.47E-01
ENSBTAG00000044538	2.70E+00	5.76E-01	0.00E+00	1.00E+00	-5.13E-15	1.00E+00	0.00E+00	1.00E+00	2.71E+00	6.01E-01
ENSBTAG00000015136	4.38E-02	1.00E+00	1.98E-01	5.93E-01	7.35E-01	1.49E-03	7.74E-01	9.26E-04	-2.50E-01	8.00E-01
ENSBTAG00000015140	-3.53E-01	8.12E-01	-1.05E-01	1.00E+00	9.71E-01	9.21E-04	1.32E+00	4.06E-06	1.66E-01	1.00E+00
ENSBTAG00000017441	6.11E-04	1.00E+00	3.54E-01	2.56E-01	2.52E-01	4.79E-01	3.54E-01	2.70E-01	3.51E-02	1.00E+00
ENSBTAG00000007389	1.38E-02	1.00E+00	-5.85E-02	1.00E+00	-1.45E-02	1.00E+00	-2.01E-01	6.44E-01	-2.61E-01	8.00E-01
ENSBTAG00000007390	-2.33E-01	8.23E-01	-1.47E+00	4.76E-13	-2.05E+00	6.13E-24	-8.63E-01	3.79E-05	-5.34E-01	1.31E-01
ENSBTAG00000007393	-8.58E-01	1.62E-01	-1.87E+00	5.46E-06	-2.14E+00	1.19E-07	-1.89E+00	2.44E-06	-1.25E+00	2.46E-02
ENSBTAG00000022520	1.27E+00	2.90E-02	2.49E+00	1.11E-08	2.80E-01	7.74E-01	6.12E-03	1.00E+00	1.12E+00	1.26E-01
ENSBTAG00000001632	7.96E-01	2.86E-02	1.17E+00	1.53E-05	1.00E+00	2.42E-04	6.30E-01	3.56E-02	5.90E-01	2.74E-01
ENSBTAG00000006801	1.14E-01	1.00E+00	9.31E-01	5.27E-05	4.45E-01	9.01E-02	-6.10E-01	1.58E-02	-8.92E-02	1.00E+00
ENSBTAG00000030305	-1.18E-01	1.00E+00	1.94E-01	6.72E-01	5.34E-01	4.58E-02	3.66E-01	2.38E-01	-5.59E-01	3.00E-01
ENSBTAG00000045973	-7.53E-01	1.00E+00	-1.21E-01	1.00E+00	-1.79E+00	1.68E-01	-1.82E+00	1.69E-01	-1.64E+00	6.01E-01
ENSBTAG00000047023	-2.22E+00	4.21E-01	1.05E+00	3.21E-01	-1.58E+00	2.63E-01	-1.60E+00	2.70E-01	-1.42E+00	7.68E-01
ENSBTAG00000046891	0.00E+00	1.00E+00	0.00E+00	1.00E+00	-5.13E-15	1.00E+00	1.76E+00	4.97E-01	0.00E+00	1.00E+00
ENSBTAG00000030314	0.00E+00	1.00E+00	1.98E+00	4.43E-01	2.55E+00	2.54E-01	0.00E+00	1.00E+00	0.00E+00	1.00E+00
ENSBTAG00000045620	1.35E-02	1.00E+00	1.39E+00	5.17E-01	-1.91E+00	4.28E-01	-1.41E-01	1.00E+00	-1.91E+00	8.00E-01
ENSBTAG00000030308	0.00E+00	1.00E+00	0.00E+00	1.00E+00	-5.13E-15	1.00E+00	2.53E+00	2.71E-01	0.00E+00	1.00E+00
ENSBTAG00000048293	-1.91E+00	8.23E-01	-1.91E+00	4.69E-01	6.39E-01	9.79E-01	6.27E-01	9.94E-01	6.46E-03	1.00E+00
ENSBTAG00000045916	-2.71E+00	5.76E-01	2.08E+00	8.02E-02	-2.71E+00	1.99E-01	-2.71E+00	2.05E-01	-7.99E-01	1.00E+00
ENSBTAG00000030306	-4.84E+00	9.72E-03	-4.84E+00	1.87E-03	-4.84E+00	7.64E-04	-3.08E+00	1.08E-02	-4.84E+00	2.27E-02
ENSBTAG00000048183	8.02E-01	1.00E+00	7.51E-02	1.00E+00	-1.91E+00	4.28E-01	-1.47E-01	1.00E+00	-1.91E+00	8.00E-01
ENSBTAG00000046183	0.00E+00	1.00E+00	0.00E+00	1.00E+00	-5.13E-15	1.00E+00	0.00E+00	1.00E+00	0.00E+00	1.00E+00
ENSBTAG00000039550	0.00E+00	1.00E+00	0.00E+00	1.00E+00	1.78E+00	4.79E-01	0.00E+00	1.00E+00	0.00E+00	1.00E+00
ENSBTAG00000040600	-3.23E+00	3.75E-01	-3.23E+00	1.24E-01	-6.74E-01	8.87E-01	-1.88E-01	1.00E+00	-1.30E+00	9.00E-01
ENSBTAG00000016847	1.49E+00	1.81E-05	1.06E+00	1.03E-03	6.07E-02	1.00E+00	3.01E-01	5.32E-01	3.85E-01	7.79E-01
ENSBTAG00000044513	0.00E+00	1.00E+00	0.00E+00	1.00E+00	-5.13E-15	1.00E+00	0.00E+00	1.00E+00	0.00E+00	1.00E+00
ENSBTAG00000008339	1.48E+00	8.16E-09	1.93E+00	1.40E-15	1.16E+00	1.97E-06	3.18E-01	3.38E-01	8.82E-01	9.10E-03
ENSBTAG00000008333	2.15E-01	1.00E+00	1.40E+00	1.25E-04	8.86E-03	1.00E+00	-5.99E-01	2.13E-01	-6.78E-01	4.82E-01

ENSBTAG00000015751	6.52E-02	1.00E+00	-1.20E+00	4.08E-01	-1.94E+00	1.28E-01	-1.96E+00	1.32E-01	-5.57E-01	1.00E+00
ENSBTAG00000013134	1.68E+00	7.75E-01	3.02E+00	1.37E-02	2.25E+00	1.18E-01	1.12E+00	6.72E-01	1.68E+00	7.79E-01
ENSBTAG00000003966	-2.89E-01	8.14E-01	-4.05E-02	1.00E+00	9.05E-01	2.35E-04	1.22E+00	5.46E-07	-3.18E-01	7.36E-01
ENSBTAG00000045732	-5.08E-01	8.76E-01	-7.91E-01	2.33E-01	-4.36E-01	5.86E-01	-6.38E-01	3.58E-01	7.57E-01	5.32E-01
ENSBTAG00000017536	-1.04E+00	6.96E-02	-9.73E-01	2.16E-02	-1.03E+00	1.23E-02	-8.81E-01	3.99E-02	6.33E-01	4.78E-01
ENSBTAG00000007434	-7.07E-03	1.00E+00	-4.27E-01	2.31E-01	-3.97E-01	2.76E-01	-5.62E-01	9.12E-02	-6.40E-02	1.00E+00
ENSBTAG00000001279	9.01E-03	1.00E+00	-3.03E-01	9.98E-01	-2.94E-01	9.88E-01	-1.39E+00	1.65E-01	-1.87E+00	3.37E-01
ENSBTAG00000044287	0.00E+00	1.00E+00	0.00E+00	1.00E+00	-5.13E-15	1.00E+00	0.00E+00	1.00E+00	0.00E+00	1.00E+00
ENSBTAG00000002441	0.00E+00	1.00E+00	0.00E+00	1.00E+00	1.78E+00	4.79E-01	0.00E+00	1.00E+00	1.93E+00	8.00E-01
ENSBTAG00000046022	4.13E+00	8.48E-02	3.98E+00	2.58E-02	1.78E+00	4.79E-01	2.53E+00	2.71E-01	2.71E+00	6.01E-01
ENSBTAG00000045652	-2.24E-01	1.00E+00	-6.73E-01	7.48E-01	-9.22E-01	5.23E-01	-4.36E+00	6.27E-03	-1.64E+00	6.06E-01
ENSBTAG00000016249	-8.59E-01	1.20E-01	-3.09E+00	2.52E-17	-1.11E+00	2.42E-03	9.58E-02	1.00E+00	8.08E-01	2.29E-01
ENSBTAG00000016251	-1.01E-01	1.00E+00	-7.75E-01	3.85E-03	-9.25E-01	4.05E-04	-8.36E-01	1.90E-03	-1.04E-02	1.00E+00
ENSBTAG00000016252	-6.84E-02	1.00E+00	7.90E-01	6.07E-05	1.21E+00	2.24E-10	1.01E+00	2.31E-07	-2.65E-01	7.08E-01
ENSBTAG00000016253	-3.26E-01	5.76E-01	-9.13E-01	2.92E-05	-8.73E-01	5.72E-05	-1.14E+00	1.38E-07	1.69E-01	9.80E-01
ENSBTAG00000016254	-4.12E-01	5.76E-01	-1.26E+00	3.85E-06	-1.13E+00	3.61E-05	-7.83E-01	6.95E-03	1.28E-01	1.00E+00
ENSBTAG00000004464	-2.08E+00	3.14E-03	-1.16E+00	4.41E-02	-5.44E-01	4.17E-01	-3.47E-02	1.00E+00	4.26E-02	1.00E+00
ENSBTAG00000019658	-2.77E+00	1.42E-01	-2.70E+00	3.78E-02	-2.90E+00	1.86E-02	-5.28E-02	1.00E+00	-8.03E-01	8.99E-01
ENSBTAG00000019660	-1.05E-01	1.00E+00	2.11E-01	4.43E-01	4.12E-01	5.40E-02	1.95E-01	4.97E-01	4.21E-02	1.00E+00
ENSBTAG00000019662	-4.25E-01	2.52E-01	-3.00E-01	2.45E-01	6.40E-01	2.32E-03	8.50E-01	3.69E-05	1.02E-01	1.00E+00
ENSBTAG00000005067	-2.95E-01	6.71E-01	-6.09E-02	1.00E+00	8.60E-01	5.76E-05	5.21E-01	2.68E-02	2.38E-01	8.00E-01
ENSBTAG00000001610	-3.76E-01	1.00E+00	6.43E-01	7.87E-01	-1.04E+00	5.97E-01	-1.06E+00	5.96E-01	5.51E-01	1.00E+00
ENSBTAG00000001615	-8.81E-02	1.00E+00	-3.08E-01	9.16E-01	6.45E-02	1.00E+00	1.76E-01	1.00E+00	-1.12E+00	5.22E-01
ENSBTAG00000019463	-2.11E-01	1.00E+00	-1.49E-01	9.38E-01	-6.12E-01	8.46E-02	-7.35E-01	3.42E-02	1.16E-02	1.00E+00
ENSBTAG00000018823	-2.90E-01	7.96E-01	-8.75E-01	3.74E-04	-8.21E-01	8.92E-04	-6.16E-01	1.98E-02	1.59E-01	1.00E+00
ENSBTAG00000011447	-8.65E-01	4.79E-01	-3.06E+00	1.13E-06	-2.44E+00	2.21E-05	-1.61E+00	4.55E-03	-7.17E-01	6.63E-01
ENSBTAG00000008165	-4.93E-01	5.76E-01	-1.53E+00	2.70E-06	-1.53E+00	2.17E-06	-2.00E+00	9.96E-10	3.24E-01	8.29E-01
ENSBTAG00000038316	6.86E-02	1.00E+00	1.01E+00	3.07E-05	1.16E+00	1.22E-06	6.57E-01	1.18E-02	4.07E-01	5.16E-01
ENSBTAG00000020590	-4.68E-01	1.00E+00	-3.00E+00	1.38E-02	-1.02E+00	3.52E-01	5.40E-01	6.50E-01	6.43E-01	8.76E-01
ENSBTAG00000039485	-6.26E-01	1.00E+00	-2.93E-01	1.00E+00	-8.10E-01	5.60E-01	-1.99E+00	1.06E-01	-6.28E-01	1.00E+00
ENSBTAG00000005693	-2.12E-01	9.57E-01	1.19E-01	9.28E-01	6.06E-01	1.78E-02	7.01E-01	5.66E-03	1.46E-01	1.00E+00
ENSBTAG00000005696	-1.10E-01	1.00E+00	-8.84E-01	5.74E-02	-1.61E+00	2.46E-04	-1.73E+00	1.15E-04	1.61E-01	1.00E+00
ENSBTAG00000001735	-4.72E-01	9.53E-01	-1.34E+00	4.39E-02	-8.17E-01	2.31E-01	5.54E-01	4.33E-01	-3.44E-01	1.00E+00
ENSBTAG00000004036	-5.46E-01	9.13E-01	6.31E-02	1.00E+00	-7.36E-01	3.38E-01	-5.84E-01	4.97E-01	-9.20E-01	6.01E-01
ENSBTAG00000016736	-2.68E-01	1.00E+00	1.32E+00	5.10E-02	5.18E-01	6.54E-01	-1.87E-01	1.00E+00	-1.13E-01	1.00E+00

ENSBTAG00000013526	-1.21E-01	1.00E+00	2.80E-01	3.81E-01	1.05E+00	4.34E-06	1.10E+00	1.90E-06	2.22E-01	8.70E-01
ENSBTAG00000044884	0.00E+00	1.00E+00	0.00E+00	1.00E+00	-5.13E-15	1.00E+00	0.00E+00	1.00E+00	0.00E+00	1.00E+00
ENSBTAG00000013529	4.86E-01	8.37E-01	-2.30E-01	9.56E-01	-1.10E+00	7.35E-02	-1.87E+00	2.99E-03	7.77E-01	5.01E-01
ENSBTAG00000013532	1.31E+00	9.30E-01	7.78E-02	1.00E+00	-1.25E-01	1.00E+00	-1.41E-01	1.00E+00	-1.91E+00	8.00E-01
ENSBTAG00000013534	8.10E-01	1.00E+00	2.71E+00	3.89E-02	6.50E-01	9.78E-01	-1.47E-01	1.00E+00	1.32E+00	8.97E-01
ENSBTAG00000008088	-5.21E-02	1.00E+00	4.90E-01	7.05E-01	-1.72E-01	1.00E+00	-3.58E-01	8.97E-01	-2.69E-01	1.00E+00
ENSBTAG00000011759	-4.89E-01	1.00E+00	4.72E-01	9.91E-01	-1.48E-01	1.00E+00	1.94E-01	1.00E+00	-1.29E+00	9.02E-01
ENSBTAG00000006048	2.85E-01	8.35E-01	1.71E+00	1.86E-11	1.94E+00	1.02E-14	1.71E+00	1.64E-11	3.84E-01	6.52E-01
ENSBTAG00000006051	-4.80E-02	1.00E+00	6.20E-01	7.54E-04	7.68E-01	1.76E-05	4.37E-01	2.80E-02	-1.25E-01	1.00E+00
ENSBTAG00000006052	-5.90E-01	4.96E-01	-1.72E+00	8.86E-06	-1.73E+00	4.42E-06	-1.85E+00	1.25E-06	-2.96E-01	9.46E-01
ENSBTAG00000006054	-6.54E-01	2.04E-01	-2.48E+00	2.19E-16	-3.26E+00	4.50E-26	-2.48E+00	2.09E-16	-5.89E-01	3.72E-01
ENSBTAG00000006056	-2.59E-02	1.00E+00	-1.09E+00	4.73E-06	-1.15E+00	1.03E-06	-2.82E-01	4.03E-01	9.12E-02	1.00E+00
ENSBTAG00000024974	1.93E+00	1.84E-04	1.62E-01	1.00E+00	-4.30E-01	5.23E-01	2.62E-01	8.45E-01	3.54E-01	9.77E-01
ENSBTAG00000004196	1.16E-01	1.00E+00	1.11E-02	1.00E+00	6.32E-02	1.00E+00	-7.76E-02	1.00E+00	-1.67E-01	1.00E+00
ENSBTAG00000024975	-3.57E-01	1.00E+00	-1.60E+00	4.11E-01	-1.03E+00	6.05E-01	-3.58E+00	4.97E-02	7.55E-01	9.84E-01
ENSBTAG00000007083	-2.77E+00	1.38E-01	-1.01E+00	4.43E-01	-1.64E+00	1.48E-01	-9.99E-01	4.28E-01	6.53E-03	1.00E+00
ENSBTAG00000007084	3.39E-01	8.15E-01	1.36E+00	2.03E-06	1.26E+00	1.11E-05	1.42E+00	8.40E-07	2.76E-01	8.68E-01
ENSBTAG00000043534	0.00E+00	1.00E+00	0.00E+00	1.00E+00	-5.13E-15	1.00E+00	0.00E+00	1.00E+00	0.00E+00	1.00E+00
ENSBTAG00000000571	-1.44E-01	1.00E+00	-5.06E-01	1.58E-01	-7.77E-02	1.00E+00	-2.62E-01	5.94E-01	7.50E-02	1.00E+00
ENSBTAG00000020359	-3.81E-01	4.64E-01	6.90E-01	2.85E-03	4.57E-01	6.92E-02	4.57E-01	7.53E-02	-8.19E-02	1.00E+00
ENSBTAG00000027274	-1.94E-01	1.00E+00	-1.03E+00	4.90E-01	-2.56E+00	5.71E-02	-6.39E-01	7.46E-01	-7.24E-01	1.00E+00
ENSBTAG00000000030	-1.57E-01	1.00E+00	-2.39E-01	9.45E-01	-1.06E+00	7.93E-02	-8.19E-01	2.05E-01	-1.91E-01	1.00E+00
ENSBTAG00000000029	-1.91E+00	8.23E-01	7.64E-02	1.00E+00	6.51E-01	9.78E-01	6.21E-01	9.94E-01	1.68E+00	7.78E-01
ENSBTAG00000024942	4.68E-02	1.00E+00	1.49E+00	1.72E-01	1.12E+00	3.68E-01	5.36E-01	8.62E-01	-4.87E-01	1.00E+00
ENSBTAG00000044940	0.00E+00	1.00E+00	0.00E+00	1.00E+00	-5.13E-15	1.00E+00	0.00E+00	1.00E+00	0.00E+00	1.00E+00
ENSBTAG00000015958	2.15E-03	1.00E+00	-1.27E-01	7.74E-01	-2.77E-02	1.00E+00	-2.65E-01	2.94E-01	1.26E-01	1.00E+00
ENSBTAG00000002664	9.91E-01	2.91E-01	-1.26E+00	5.78E-02	6.74E-01	2.98E-01	3.78E-01	7.08E-01	1.51E-01	1.00E+00
ENSBTAG00000016012	8.10E-01	1.00E+00	2.07E+00	1.95E-01	1.15E+00	6.41E-01	6.27E-01	9.94E-01	-1.91E+00	8.00E-01
ENSBTAG00000008442	3.31E-01	7.28E-01	5.84E-01	3.46E-02	1.16E+00	2.76E-06	1.62E+00	4.66E-11	2.93E-01	8.00E-01
ENSBTAG00000006785	-7.79E-02	1.00E+00	9.01E-01	8.71E-03	1.01E+00	2.60E-03	1.67E+00	2.00E-07	-2.71E-01	9.66E-01
ENSBTAG00000042764	0.00E+00	1.00E+00	1.98E+00	4.43E-01	2.56E+00	2.54E-01	0.00E+00	1.00E+00	1.91E+00	8.00E-01
ENSBTAG00000002749	1.01E-02	1.00E+00	-1.10E+00	5.46E-01	-1.68E-01	1.00E+00	-8.57E-01	6.62E-01	-1.15E+00	8.45E-01
ENSBTAG00000010317	-1.91E+00	8.23E-01	-1.91E+00	4.69E-01	-1.91E+00	4.28E-01	-1.91E+00	4.39E-01	1.32E+00	8.97E-01
ENSBTAG00000017512	-2.02E-01	1.00E+00	5.34E-01	2.18E-01	-2.36E-01	7.80E-01	3.03E-01	6.09E-01	1.43E-01	1.00E+00
ENSBTAG00000012564	8.34E-02	1.00E+00	1.93E-01	5.55E-01	1.47E-02	1.00E+00	1.65E-01	6.70E-01	1.93E-01	8.97E-01

ENSBTAG00000046260	0.00E+00	1.00E+00	0.00E+00	1.00E+00	-5.13E-15	1.00E+00	0.00E+00	1.00E+00	0.00E+00	1.00E+00
ENSBTAG00000002726	-3.92E-01	2.70E-01	-8.63E-02	9.61E-01	2.68E-01	2.73E-01	3.82E-01	8.58E-02	-4.98E-01	1.36E-01
ENSBTAG00000021916	-3.31E-01	1.00E+00	-4.03E-01	8.89E-01	-4.30E-01	8.29E-01	-1.51E+00	1.32E-01	-3.71E-01	1.00E+00
ENSBTAG00000025165	2.20E-01	1.00E+00	1.80E+00	1.02E-06	1.09E+00	5.35E-03	6.89E-01	1.26E-01	1.08E-01	1.00E+00
ENSBTAG00000009987	-3.46E-01	1.00E+00	-9.01E-01	1.74E-01	-4.44E-01	6.39E-01	6.99E-02	1.00E+00	2.19E-01	1.00E+00
ENSBTAG00000001621	9.47E-01	8.23E-01	1.52E+00	9.92E-02	-1.78E-01	1.00E+00	2.54E-01	1.00E+00	1.21E+00	6.15E-01
ENSBTAG00000045837	0.00E+00	1.00E+00	0.00E+00	1.00E+00	-5.13E-15	1.00E+00	0.00E+00	1.00E+00	0.00E+00	1.00E+00
ENSBTAG00000046063	2.72E+00	5.76E-01	1.98E+00	4.43E-01	1.78E+00	4.79E-01	3.71E+00	4.65E-02	0.00E+00	1.00E+00
ENSBTAG00000006943	2.71E-02	1.00E+00	-3.58E+00	6.76E-02	1.32E-01	1.00E+00	7.36E-01	6.73E-01	4.88E-03	1.00E+00
ENSBTAG00000009458	3.00E-01	5.75E-01	9.39E-01	7.92E-07	4.81E-01	2.03E-02	-1.28E-03	1.00E+00	1.27E-02	1.00E+00
ENSBTAG00000015713	2.45E-03	1.00E+00	1.58E-01	6.49E-01	4.94E-01	1.72E-02	1.78E-01	5.71E-01	-2.25E-01	8.00E-01
ENSBTAG00000015739	-4.16E-01	8.01E-01	-1.20E+00	1.45E-03	-1.23E+00	8.19E-04	-1.31E+00	4.51E-04	-2.02E-01	1.00E+00
ENSBTAG00000004637	-6.24E-01	1.00E+00	-1.75E+00	2.13E-01	-1.09E+00	4.42E-01	1.70E+00	4.46E-02	-1.33E+00	7.68E-01
ENSBTAG00000045325	0.00E+00	1.00E+00	0.00E+00	1.00E+00	-5.13E-15	1.00E+00	0.00E+00	1.00E+00	0.00E+00	1.00E+00
ENSBTAG00000044674	0.00E+00	1.00E+00	0.00E+00	1.00E+00	-5.13E-15	1.00E+00	0.00E+00	1.00E+00	0.00E+00	1.00E+00
ENSBTAG00000004249	4.75E-01	8.40E-01	8.46E-03	1.00E+00	2.06E-01	9.64E-01	6.78E-01	2.28E-01	1.69E-01	1.00E+00
ENSBTAG00000020810	-2.76E-01	1.00E+00	-7.23E-01	3.72E-01	5.46E-02	1.00E+00	-1.09E+00	1.19E-01	-3.93E-01	1.00E+00
ENSBTAG00000047866	-1.91E+00	8.23E-01	7.64E-02	1.00E+00	6.40E-01	9.79E-01	1.12E+00	6.68E-01	7.17E-03	1.00E+00
ENSBTAG00000024950	6.94E-01	7.69E-01	2.17E+00	3.90E-06	1.74E+00	3.85E-04	1.40E+00	8.92E-03	6.62E-01	8.00E-01
ENSBTAG00000038138	1.20E+00	8.54E-01	-7.17E-01	9.45E-01	-1.42E-01	1.00E+00	6.92E-01	8.59E-01	5.78E-03	1.00E+00
ENSBTAG00000016613	-3.21E+00	3.75E-01	7.67E-01	7.36E-01	7.58E-01	7.14E-01	9.52E-01	5.55E-01	3.61E-02	1.00E+00
ENSBTAG00000003500	-2.61E-01	8.23E-01	-7.83E-02	1.00E+00	4.23E-01	1.03E-01	5.71E-01	1.99E-02	5.07E-01	2.49E-01
ENSBTAG00000009885	1.30E-01	1.00E+00	1.26E+00	3.06E-03	1.59E+00	1.16E-04	1.74E+00	2.56E-05	4.97E-01	7.96E-01
ENSBTAG00000008151	-5.25E-01	1.45E-01	-9.63E-01	2.28E-05	-2.03E-01	5.60E-01	-3.34E-01	2.40E-01	3.31E-02	1.00E+00
ENSBTAG00000008154	1.45E-01	1.00E+00	-4.52E-01	3.12E-01	-3.52E-01	4.76E-01	-6.30E-01	1.18E-01	-3.81E-01	8.00E-01
ENSBTAG00000000056	3.84E-03	1.00E+00	-7.11E-01	2.35E-03	-4.11E-01	1.12E-01	-4.43E-01	8.75E-02	3.74E-01	5.09E-01
ENSBTAG00000011834	-5.91E-02	1.00E+00	2.11E-02	1.00E+00	3.55E-01	2.18E-01	5.90E-01	2.09E-02	4.11E-01	4.72E-01
ENSBTAG00000021058	6.03E-02	1.00E+00	-3.82E-01	7.83E-02	6.66E-01	5.16E-04	5.07E-01	1.32E-02	9.05E-02	1.00E+00
ENSBTAG00000021060	3.56E-01	5.76E-01	2.87E+00	3.18E-35	2.74E+00	1.46E-32	2.13E+00	9.99E-21	-2.50E-01	8.04E-01
ENSBTAG00000021061	-5.16E-02	1.00E+00	2.06E-01	6.05E-01	4.25E-01	1.32E-01	3.62E-01	2.34E-01	-2.03E-01	9.38E-01
ENSBTAG00000021062	-4.36E-02	1.00E+00	-3.13E-01	2.26E-01	-3.25E-01	1.99E-01	-1.25E-01	8.28E-01	-1.08E-01	1.00E+00
ENSBTAG00000024947	1.55E-01	1.00E+00	8.72E-02	1.00E+00	-1.19E-02	1.00E+00	-3.82E-01	9.55E-01	-3.98E-01	1.00E+00
ENSBTAG00000017220	-3.21E+00	3.75E-01	-3.21E+00	1.26E-01	-3.21E+00	9.49E-02	-3.21E+00	9.96E-02	-3.21E+00	4.34E-01
ENSBTAG00000044204	1.72E-01	1.00E+00	-2.52E-01	8.82E-01	-5.11E-01	4.39E-01	-3.65E-01	6.62E-01	4.69E-01	8.00E-01
ENSBTAG00000004770	1.92E-01	1.00E+00	-5.09E-01	8.16E-01	-5.73E-01	7.19E-01	-7.98E-01	5.24E-01	-2.63E-01	1.00E+00

ENSBTAG00000019432	1.48E+00	3.15E-03	1.70E+00	4.72E-05	1.11E+00	1.10E-02	7.71E-01	1.15E-01	-5.46E-02	1.00E+00
ENSBTAG00000016799	1.63E+00	3.70E-09	2.31E+00	7.21E-19	1.33E+00	3.00E-07	7.73E-01	5.97E-03	8.61E-01	2.43E-02
ENSBTAG00000004723	2.67E-02	1.00E+00	-7.74E-01	3.89E-03	-5.08E-01	8.01E-02	6.16E-02	1.00E+00	2.09E-01	9.56E-01
ENSBTAG00000042741	-2.25E-01	1.00E+00	1.59E+00	8.05E-05	1.50E+00	1.70E-04	1.11E+00	1.13E-02	-1.36E+00	1.56E-01
ENSBTAG00000042398	2.28E-01	1.00E+00	3.27E-01	9.07E-01	6.35E-01	4.74E-01	4.29E-01	7.48E-01	-1.08E+00	6.25E-01
ENSBTAG00000012066	-1.84E-01	1.00E+00	-1.23E+00	4.77E-04	-6.04E-01	1.32E-01	-1.87E-01	8.76E-01	-1.29E-01	1.00E+00
ENSBTAG00000007409	3.42E-01	9.71E-01	-6.43E-01	2.56E-01	4.09E-01	4.79E-01	8.03E-01	6.66E-02	8.67E-01	2.49E-01
ENSBTAG00000018420	-5.92E-01	1.94E-01	-1.08E+00	7.87E-05	-9.54E-01	4.87E-04	-4.85E-01	1.25E-01	-3.59E-04	1.00E+00
ENSBTAG00000018423	9.27E-01	1.91E-04	2.06E+00	9.91E-22	1.88E+00	1.46E-18	1.16E+00	8.95E-08	-8.61E-02	1.00E+00
ENSBTAG00000045885	0.00E+00	1.00E+00	0.00E+00	1.00E+00	-5.13E-15	1.00E+00	0.00E+00	1.00E+00	0.00E+00	1.00E+00
ENSBTAG00000047875	0.00E+00	1.00E+00	0.00E+00	1.00E+00	-5.13E-15	1.00E+00	0.00E+00	1.00E+00	0.00E+00	1.00E+00
ENSBTAG00000014002	2.74E-01	6.70E-01	2.12E-01	4.63E-01	3.91E-01	8.47E-02	-6.40E-01	2.26E-03	-2.54E-01	7.59E-01
ENSBTAG00000019853	-1.16E-01	1.00E+00	-9.02E-01	4.52E-04	-5.42E-01	4.84E-02	-1.64E-02	1.00E+00	2.21E-02	1.00E+00
ENSBTAG00000012225	-1.39E-01	1.00E+00	4.42E-01	7.47E-02	1.10E+00	2.46E-07	1.07E+00	7.61E-07	-5.78E-01	1.30E-01
ENSBTAG00000014598	2.26E-01	8.90E-01	1.84E-01	6.62E-01	8.19E-02	1.00E+00	2.61E-01	4.39E-01	2.54E-01	8.00E-01
ENSBTAG00000015951	2.62E-01	7.49E-01	8.98E-01	9.52E-06	7.43E-01	3.08E-04	6.27E-01	3.49E-03	-6.44E-02	1.00E+00
ENSBTAG00000044065	3.10E-01	1.00E+00	6.89E-01	2.16E-01	4.97E-02	1.00E+00	-1.14E+00	2.34E-02	9.35E-02	1.00E+00
ENSBTAG00000044269	0.00E+00	1.00E+00	0.00E+00	1.00E+00	-5.13E-15	1.00E+00	0.00E+00	1.00E+00	0.00E+00	1.00E+00
ENSBTAG00000007354	-1.13E-02	1.00E+00	-1.26E-01	8.95E-01	1.82E-01	6.82E-01	5.00E-01	6.76E-02	-4.52E-01	4.28E-01
ENSBTAG00000028692	-5.11E-01	1.00E+00	-1.24E+00	6.06E-01	2.06E-01	1.00E+00	-3.23E+00	9.84E-02	-1.30E+00	9.01E-01
ENSBTAG00000002423	1.66E-02	1.00E+00	9.37E-01	4.28E-08	8.16E-01	2.08E-06	5.46E-01	2.84E-03	-5.65E-01	2.68E-02
ENSBTAG00000004787	-3.42E-01	9.52E-01	-1.11E-01	1.00E+00	-4.37E-01	4.28E-01	-1.47E+00	2.96E-04	-5.18E-01	7.15E-01
ENSBTAG00000015414	-2.16E-01	9.27E-01	-1.00E+00	2.08E-05	-1.08E+00	3.22E-06	-7.98E-01	1.01E-03	6.74E-02	1.00E+00
ENSBTAG00000044470	0.00E+00	1.00E+00	0.00E+00	1.00E+00	-5.13E-15	1.00E+00	0.00E+00	1.00E+00	0.00E+00	1.00E+00
ENSBTAG00000015406	0.00E+00	1.00E+00	0.00E+00	1.00E+00	-5.13E-15	1.00E+00	0.00E+00	1.00E+00	0.00E+00	1.00E+00
ENSBTAG00000042663	0.00E+00	1.00E+00	0.00E+00	1.00E+00	-5.13E-15	1.00E+00	0.00E+00	1.00E+00	0.00E+00	1.00E+00
ENSBTAG00000015394	-7.34E-01	3.11E-02	-1.62E+00	1.55E-10	-3.22E+00	1.31E-32	-2.95E+00	1.32E-28	4.29E-01	4.80E-01
ENSBTAG00000015390	-6.76E-01	4.75E-03	-1.59E+00	9.23E-17	-2.36E+00	4.13E-34	-2.39E+00	1.27E-34	1.62E-02	1.00E+00
ENSBTAG00000015387	-9.03E-01	9.80E-03	-2.61E+00	7.36E-20	-4.22E+00	2.79E-41	-3.39E+00	2.00E-30	-5.09E-01	4.33E-01
ENSBTAG00000017645	8.49E-01	4.33E-01	6.56E-01	3.21E-01	3.59E-01	7.23E-01	8.60E-01	1.35E-01	6.96E-01	6.58E-01
ENSBTAG00000003695	2.03E-01	8.54E-01	9.82E-01	4.20E-07	9.18E-01	2.23E-06	7.75E-01	1.07E-04	-1.93E-02	1.00E+00
ENSBTAG00000003687	2.77E-01	5.83E-01	2.01E+00	9.86E-30	1.57E+00	4.52E-19	1.16E+00	9.14E-11	-1.21E-01	1.00E+00
ENSBTAG00000046136	0.00E+00	1.00E+00	0.00E+00	1.00E+00	-5.13E-15	1.00E+00	0.00E+00	1.00E+00	0.00E+00	1.00E+00
ENSBTAG00000047408	0.00E+00	1.00E+00	0.00E+00	1.00E+00	-5.13E-15	1.00E+00	0.00E+00	1.00E+00	0.00E+00	1.00E+00
ENSBTAG00000030230	-2.61E-01	8.76E-01	-7.82E-02	1.00E+00	-4.53E-01	1.45E-01	-5.90E-01	4.66E-02	-4.58E-01	4.82E-01

ENSBTAG00000018178	-4.49E-01	2.32E-01	-9.03E-01	2.84E-05	-4.07E-01	9.44E-02	-1.78E-01	6.27E-01	7.98E-02	1.00E+00
ENSBTAG00000018176	-3.29E-01	5.76E-01	2.01E-01	5.58E-01	-1.30E-01	8.24E-01	-2.39E-02	1.00E+00	-6.99E-02	1.00E+00
ENSBTAG00000018175	-2.78E-01	7.80E-01	-6.75E-01	4.87E-03	-3.29E-01	2.51E-01	-7.98E-01	7.77E-04	1.06E-01	1.00E+00
ENSBTAG00000018172	-1.26E-01	1.00E+00	-8.34E-02	1.00E+00	-2.55E-01	5.68E-01	-1.55E-01	8.78E-01	2.60E-01	8.97E-01
ENSBTAG00000018170	0.00E+00	1.00E+00	1.99E+00	4.43E-01	-5.13E-15	1.00E+00	0.00E+00	1.00E+00	0.00E+00	1.00E+00
ENSBTAG00000015299	1.32E-02	1.00E+00	1.40E+00	5.16E-01	6.45E-01	9.78E-01	-1.46E-01	1.00E+00	-1.91E+00	8.00E-01
ENSBTAG00000047667	2.71E+00	5.76E-01	3.68E+00	5.07E-02	-5.13E-15	1.00E+00	0.00E+00	1.00E+00	0.00E+00	1.00E+00
ENSBTAG00000046791	0.00E+00	1.00E+00	0.00E+00	1.00E+00	-5.13E-15	1.00E+00	0.00E+00	1.00E+00	0.00E+00	1.00E+00
ENSBTAG00000039765	1.02E+00	1.00E+00	2.39E+00	2.36E-01	1.47E+00	5.44E-01	2.58E-01	1.00E+00	1.35E+00	9.29E-01
ENSBTAG00000045277	0.00E+00	1.00E+00	0.00E+00	1.00E+00	-5.13E-15	1.00E+00	0.00E+00	1.00E+00	0.00E+00	1.00E+00
ENSBTAG00000024934	-1.36E-01	1.00E+00	5.12E-01	5.24E-01	1.67E-01	1.00E+00	-2.56E-01	9.43E-01	8.31E-02	1.00E+00
ENSBTAG00000044288	0.00E+00	1.00E+00	0.00E+00	1.00E+00	-5.13E-15	1.00E+00	0.00E+00	1.00E+00	0.00E+00	1.00E+00
ENSBTAG00000024915	-6.21E-01	1.00E+00	2.51E-01	1.00E+00	6.99E-01	1.00E+00	3.80E-02	1.00E+00	1.29E-01	1.00E+00
ENSBTAG00000045084	0.00E+00	1.00E+00	0.00E+00	1.00E+00	-5.13E-15	1.00E+00	0.00E+00	1.00E+00	0.00E+00	1.00E+00
ENSBTAG00000038910	-2.42E-02	1.00E+00	1.84E+00	1.01E-01	3.15E+00	2.75E-03	2.59E+00	1.68E-02	8.43E-01	9.13E-01
ENSBTAG00000047245	0.00E+00	1.00E+00	0.00E+00	1.00E+00	-5.13E-15	1.00E+00	0.00E+00	1.00E+00	0.00E+00	1.00E+00
ENSBTAG00000045705	5.36E-03	1.00E+00	-1.91E+00	4.69E-01	6.49E-01	9.78E-01	-1.91E+00	4.39E-01	6.47E-03	1.00E+00
ENSBTAG00000040323	1.05E-01	1.00E+00	6.36E-01	9.39E-01	1.03E+00	6.72E-01	3.01E-01	1.00E+00	2.78E-01	1.00E+00
ENSBTAG00000046226	0.00E+00	1.00E+00	0.00E+00	1.00E+00	-5.13E-15	1.00E+00	0.00E+00	1.00E+00	0.00E+00	1.00E+00
ENSBTAG00000011359	-2.68E-01	1.00E+00	-1.53E+00	2.74E-04	-7.76E-01	7.36E-02	-1.07E+00	1.06E-02	-4.46E-02	1.00E+00
ENSBTAG00000019986	-1.35E-01	1.00E+00	-6.83E-02	1.00E+00	2.39E-01	3.04E-01	-4.96E-04	1.00E+00	3.11E-01	4.83E-01
ENSBTAG00000017461	6.46E-01	6.05E-01	7.29E-01	1.72E-01	1.26E+00	6.10E-03	1.56E+00	5.72E-04	2.59E-01	1.00E+00
ENSBTAG00000019321	-6.38E-02	1.00E+00	-1.63E+00	6.37E-09	-6.89E-01	1.72E-02	-8.41E-01	3.30E-03	2.03E-01	9.98E-01
ENSBTAG00000015980	-1.19E+00	2.37E-01	-2.81E+00	2.05E-06	-1.91E+00	1.25E-03	-2.15E+00	3.34E-04	-1.30E+00	2.36E-01
ENSBTAG00000047760	-2.88E-01	8.23E-01	-1.11E-01	9.70E-01	4.07E-01	1.83E-01	5.02E-01	8.68E-02	-1.82E-01	1.00E+00
ENSBTAG00000019317	-1.86E-01	9.32E-01	-2.53E-01	3.58E-01	-4.63E-02	1.00E+00	1.81E-01	5.84E-01	-3.82E-02	1.00E+00
ENSBTAG00000011575	-2.60E-01	7.48E-01	-6.63E-01	1.59E-03	-1.27E+00	1.90E-10	-1.32E+00	4.84E-11	1.41E-01	1.00E+00
ENSBTAG00000047043	3.21E+00	3.75E-01	0.00E+00	1.00E+00	-5.13E-15	1.00E+00	0.00E+00	1.00E+00	3.22E+00	4.34E-01
ENSBTAG00000008747	-3.04E-01	8.23E-01	-1.45E+00	3.98E-07	-2.71E+00	4.09E-21	-2.36E+00	1.89E-16	-1.43E-01	1.00E+00
ENSBTAG00000022927	-1.82E-01	1.00E+00	-1.06E+00	2.38E-02	-2.26E+00	1.57E-06	-1.63E+00	3.69E-04	-4.95E-01	7.87E-01
ENSBTAG00000004111	-7.44E-02	1.00E+00	-1.04E+00	4.36E-03	-2.23E+00	2.27E-10	-1.88E+00	9.98E-08	4.30E-01	7.73E-01
ENSBTAG00000004108	-3.21E-02	1.00E+00	-6.64E-01	1.38E-02	-1.25E+00	7.83E-07	-9.37E-01	3.07E-04	2.04E-01	9.55E-01
ENSBTAG00000004632	-2.69E-01	7.75E-01	-1.18E+00	5.76E-08	-1.37E+00	2.18E-10	-1.27E+00	5.88E-09	2.17E-01	8.38E-01
ENSBTAG00000047973	0.00E+00	1.00E+00	0.00E+00	1.00E+00	-5.13E-15	1.00E+00	0.00E+00	1.00E+00	0.00E+00	1.00E+00
ENSBTAG00000044643	0.00E+00	1.00E+00	0.00E+00	1.00E+00	-5.13E-15	1.00E+00	0.00E+00	1.00E+00	0.00E+00	1.00E+00

ENSBTAG00000000044	7.97E-01	1.00E+00	-1.91E+00	4.69E-01	-1.91E+00	4.28E-01	-1.48E-01	1.00E+00	1.78E-02	1.00E+00
ENSBTAG00000000042	-4.86E-03	1.00E+00	1.20E+00	2.63E-02	6.46E-02	1.00E+00	2.44E-01	9.27E-01	3.30E-01	1.00E+00
ENSBTAG00000000040	-5.85E-01	3.30E-01	2.27E-01	6.87E-01	3.45E-01	4.09E-01	4.97E-01	1.71E-01	-1.52E-01	1.00E+00
ENSBTAG00000000039	-9.92E-01	2.43E-05	-7.29E-01	7.81E-04	1.57E-01	6.87E-01	1.51E-01	7.24E-01	-3.76E-02	1.00E+00
ENSBTAG00000001868	-6.40E-01	4.33E-01	-1.87E+00	5.45E-07	-2.88E+00	2.05E-14	-1.92E+00	3.02E-07	-4.99E-01	6.95E-01
ENSBTAG00000013898	1.44E+00	7.47E-01	2.08E+00	7.80E-02	-9.24E-01	8.08E-01	-9.46E-01	8.04E-01	5.08E-01	1.00E+00
ENSBTAG00000001937	-2.93E-01	8.23E-01	-6.77E-01	1.27E-02	-8.12E-01	2.05E-03	-6.28E-01	2.52E-02	-1.26E-01	1.00E+00
ENSBTAG00000008498	4.91E-01	1.67E-01	1.92E+00	2.80E-20	1.83E+00	1.08E-18	1.52E+00	3.87E-13	-5.23E-01	1.97E-01
ENSBTAG00000030209	1.30E-02	1.00E+00	3.97E-01	1.62E-01	7.78E-01	1.38E-03	9.65E-01	6.14E-05	-2.46E-01	8.17E-01
ENSBTAG00000006045	3.49E-01	8.03E-01	7.43E-01	1.77E-02	6.50E-01	4.35E-02	7.27E-01	2.38E-02	1.15E+00	2.76E-03
ENSBTAG00000024929	-2.14E+00	4.81E-02	-2.25E+00	6.54E-03	-9.20E-01	2.83E-01	-5.93E-01	5.70E-01	-7.98E-01	8.00E-01
ENSBTAG00000019812	-2.07E-01	9.28E-01	-6.37E-01	7.13E-03	-1.23E+00	3.02E-08	-1.03E+00	5.61E-06	-6.57E-01	6.10E-02
ENSBTAG00000003786	-3.05E-01	7.65E-01	-1.12E+00	3.91E-06	-2.29E+00	1.59E-21	-2.03E+00	3.43E-17	1.66E-01	1.00E+00
ENSBTAG00000021914	-2.20E-01	1.00E+00	-2.45E+00	1.28E-02	-5.75E+00	1.44E-05	-3.99E+00	2.88E-04	3.79E-02	1.00E+00
ENSBTAG00000000417	-5.52E-01	2.60E-01	-1.11E+00	4.46E-05	-1.23E+00	4.55E-06	-6.63E-01	2.55E-02	5.77E-03	1.00E+00
ENSBTAG00000000411	-5.91E-02	1.00E+00	6.72E-01	7.88E-04	8.25E-01	2.37E-05	5.97E-01	3.92E-03	1.21E-01	1.00E+00
ENSBTAG00000030200	-1.87E-01	1.00E+00	-7.54E-01	1.72E-02	-7.67E-01	1.33E-02	-6.21E-01	5.90E-02	3.49E-01	7.73E-01
ENSBTAG00000000356	-8.95E-02	1.00E+00	1.57E+00	7.61E-11	1.85E+00	1.35E-14	1.29E+00	1.39E-07	-2.43E-01	8.63E-01
ENSBTAG00000000355	-5.36E-01	3.83E-01	6.74E-01	2.69E-02	5.72E-01	6.94E-02	5.62E-01	8.22E-02	1.91E-01	1.00E+00
ENSBTAG00000000354	8.73E-03	1.00E+00	2.47E+00	8.04E-05	1.37E+00	8.50E-02	8.47E-01	4.34E-01	3.31E-01	1.00E+00
ENSBTAG00000040573	-1.91E+00	8.23E-01	8.77E-01	8.53E-01	-1.31E-01	1.00E+00	-1.91E+00	4.39E-01	-1.91E+00	8.00E-01
ENSBTAG00000019105	1.02E-02	1.00E+00	9.27E-01	2.17E-04	9.29E-01	2.00E-04	8.44E-01	1.02E-03	5.71E-02	1.00E+00
ENSBTAG00000019104	-4.54E-01	7.71E-01	-1.37E-01	1.00E+00	-9.87E-01	1.32E-02	1.18E-01	1.00E+00	2.82E-01	9.70E-01
ENSBTAG00000024932	1.70E+00	7.71E-01	2.07E+00	2.00E-01	-1.30E-01	1.00E+00	-1.91E+00	4.40E-01	7.01E-03	1.00E+00
ENSBTAG00000006189	1.50E-01	1.00E+00	-9.77E-01	1.40E-02	5.29E-01	2.60E-01	1.48E+00	1.16E-04	-1.41E+00	5.27E-03
ENSBTAG00000047677	0.00E+00	1.00E+00	0.00E+00	1.00E+00	-5.13E-15	1.00E+00	0.00E+00	1.00E+00	0.00E+00	1.00E+00
ENSBTAG00000016776	-3.46E-01	8.23E-01	-2.25E+00	5.74E-11	-9.79E-01	2.58E-03	-7.99E-01	1.85E-02	3.91E-01	7.35E-01
ENSBTAG00000043745	0.00E+00	1.00E+00	0.00E+00	1.00E+00	-5.13E-15	1.00E+00	0.00E+00	1.00E+00	0.00E+00	1.00E+00
ENSBTAG00000018271	3.37E-02	1.00E+00	3.66E-01	1.05E-01	3.07E-01	1.98E-01	2.88E-01	2.50E-01	5.62E-01	6.62E-02
ENSBTAG00000019972	4.75E-02	1.00E+00	1.41E-01	7.79E-01	1.05E+00	4.07E-07	8.36E-01	1.02E-04	7.96E-02	1.00E+00
ENSBTAG00000000875	-2.75E-01	8.23E-01	-1.36E+00	3.60E-07	-1.18E+00	5.25E-06	-1.03E+00	9.95E-05	7.56E-01	4.02E-02
ENSBTAG00000019049	3.14E-01	1.00E+00	1.16E-01	1.00E+00	1.21E+00	3.91E-03	9.44E-01	4.15E-02	2.66E-01	1.00E+00
ENSBTAG00000029775	0.00E+00	1.00E+00	0.00E+00	1.00E+00	-5.13E-15	1.00E+00	0.00E+00	1.00E+00	0.00E+00	1.00E+00
ENSBTAG00000019044	4.09E-01	7.28E-01	7.42E-01	2.93E-02	1.97E+00	2.09E-10	1.53E+00	1.11E-06	1.51E-01	1.00E+00
ENSBTAG00000038745	-1.25E-01	1.00E+00	-3.07E-01	3.13E-01	1.86E-01	6.41E-01	3.93E-01	1.56E-01	-1.26E-01	1.00E+00

ENSBTAG00000002883	-6.12E-02	1.00E+00	2.87E-01	4.00E-01	7.17E-01	4.45E-03	5.49E-01	4.40E-02	1.60E-02	1.00E+00
ENSBTAG00000036851	0.00E+00	1.00E+00	0.00E+00	1.00E+00	-5.13E-15	1.00E+00	0.00E+00	1.00E+00	0.00E+00	1.00E+00
ENSBTAG00000008940	7.76E-01	9.29E-01	1.72E+00	4.58E-02	1.69E+00	4.61E-02	2.19E+00	5.41E-03	-1.68E+00	6.22E-01
ENSBTAG00000015339	-1.92E-01	8.96E-01	1.44E+00	3.28E-14	1.36E+00	4.93E-13	2.37E-02	1.00E+00	-1.43E-01	1.00E+00
ENSBTAG00000022489	-1.41E-01	1.00E+00	1.10E+00	7.98E-02	5.32E-01	5.15E-01	-3.65E-01	7.76E-01	2.54E-01	1.00E+00
ENSBTAG00000017233	-3.23E-01	1.00E+00	-1.34E-01	1.00E+00	8.08E-02	1.00E+00	-4.45E-01	7.06E-01	-6.71E-01	8.00E-01
ENSBTAG00000015276	-3.22E-01	6.88E-01	-1.98E+00	2.59E-16	-2.73E+00	2.32E-28	-1.82E+00	2.66E-14	2.88E-02	1.00E+00
ENSBTAG00000015267	-7.35E-01	9.03E-03	-1.48E+00	3.00E-11	-1.12E+00	3.82E-07	-7.83E-01	6.50E-04	-9.59E-02	1.00E+00
ENSBTAG00000015261	-5.70E-01	8.23E-01	-1.21E+00	4.74E-02	-8.27E-01	1.86E-01	-7.10E-01	2.89E-01	3.25E-01	1.00E+00
ENSBTAG00000016023	6.82E-02	1.00E+00	7.79E-01	2.61E-05	1.11E+00	8.12E-10	8.16E-01	1.18E-05	-4.46E-02	1.00E+00
ENSBTAG00000016021	-1.29E-01	1.00E+00	-3.70E-01	1.91E-01	-2.87E-01	3.59E-01	-1.70E-01	7.17E-01	6.31E-01	9.47E-02
ENSBTAG00000030186	0.00E+00	1.00E+00	0.00E+00	1.00E+00	-5.13E-15	1.00E+00	0.00E+00	1.00E+00	0.00E+00	1.00E+00
ENSBTAG00000016017	-2.66E-01	1.00E+00	-7.46E-01	1.23E-01	-1.02E+00	2.22E-02	-1.39E+00	1.85E-03	2.09E-01	1.00E+00
ENSBTAG00000003192	-2.74E-02	1.00E+00	-2.71E-01	5.53E-01	-6.93E-01	3.08E-02	-8.86E-01	5.02E-03	5.01E-01	4.66E-01
ENSBTAG00000009782	-1.29E+00	4.70E-03	-8.25E-01	4.04E-02	-7.43E-01	7.08E-02	-2.06E-01	8.61E-01	2.11E-01	1.00E+00
ENSBTAG00000009107	-5.89E-01	6.06E-01	-1.49E+00	6.96E-04	-9.67E-01	2.90E-02	-2.45E-01	8.11E-01	5.21E-01	7.06E-01
ENSBTAG00000038306	-5.51E-01	1.00E+00	-1.38E+00	2.64E-01	-1.66E-01	1.00E+00	5.02E-01	7.44E-01	5.54E-01	1.00E+00
ENSBTAG00000001965	0.00E+00	1.00E+00	1.99E+00	4.43E-01	-5.13E-15	1.00E+00	1.76E+00	4.97E-01	0.00E+00	1.00E+00
ENSBTAG00000042973	0.00E+00	1.00E+00	0.00E+00	1.00E+00	-5.13E-15	1.00E+00	0.00E+00	1.00E+00	0.00E+00	1.00E+00
ENSBTAG00000043887	0.00E+00	1.00E+00	0.00E+00	1.00E+00	-5.13E-15	1.00E+00	0.00E+00	1.00E+00	0.00E+00	1.00E+00
ENSBTAG00000006280	-6.57E-02	1.00E+00	-3.55E-01	6.50E-01	-6.95E-01	1.99E-01	-5.01E-01	4.26E-01	-9.50E-01	3.30E-01
ENSBTAG00000006277	-2.86E-02	1.00E+00	3.67E-01	4.40E-01	1.59E-01	9.28E-01	-3.49E-02	1.00E+00	9.53E-02	1.00E+00
ENSBTAG00000006276	-1.36E-01	1.00E+00	-3.19E-01	3.33E-01	-8.08E-01	1.57E-03	-7.99E-01	2.15E-03	-4.30E-01	4.85E-01
ENSBTAG00000009991	-4.45E-01	3.19E-01	-8.37E-01	3.68E-04	-1.92E-01	6.13E-01	3.04E-01	3.11E-01	8.77E-01	2.98E-03
ENSBTAG00000001368	-4.22E-01	5.81E-01	-1.50E+00	1.05E-07	-1.69E+00	1.91E-09	-7.93E-01	9.29E-03	5.56E-01	3.80E-01
ENSBTAG00000010899	1.94E-01	1.00E+00	2.82E-01	4.94E-01	4.89E-02	1.00E+00	3.74E-01	3.10E-01	2.30E-01	9.57E-01
ENSBTAG00000039283	0.00E+00	1.00E+00	0.00E+00	1.00E+00	-5.13E-15	1.00E+00	0.00E+00	1.00E+00	0.00E+00	1.00E+00
ENSBTAG00000021505	-4.05E-01	4.85E-01	8.52E-01	5.07E-04	1.36E+00	7.64E-09	6.48E-01	1.24E-02	4.49E-01	4.32E-01
ENSBTAG00000045449	-1.91E+00	8.23E-01	6.91E-02	1.00E+00	-1.91E+00	4.28E-01	1.12E+00	6.71E-01	-1.91E+00	8.00E-01
ENSBTAG00000006663	3.78E-01	3.50E-01	3.55E-01	1.30E-01	7.32E-02	1.00E+00	1.76E-01	5.97E-01	-3.00E-01	6.11E-01
ENSBTAG00000044681	0.00E+00	1.00E+00	0.00E+00	1.00E+00	-5.13E-15	1.00E+00	0.00E+00	1.00E+00	0.00E+00	1.00E+00
ENSBTAG00000000920	-6.61E-01	1.54E-01	-1.31E-01	9.45E-01	-1.52E-01	8.76E-01	-7.10E-01	2.32E-02	3.96E-01	6.51E-01
ENSBTAG00000047060	0.00E+00	1.00E+00	0.00E+00	1.00E+00	-5.13E-15	1.00E+00	0.00E+00	1.00E+00	0.00E+00	1.00E+00
ENSBTAG00000000675	2.35E-01	8.80E-01	1.10E+00	3.09E-06	1.32E+00	1.17E-08	4.94E-01	6.88E-02	-1.77E-01	1.00E+00
ENSBTAG00000008441	5.36E+00	7.07E-54	6.06E+00	1.60E-65	5.33E+00	2.43E-54	4.92E+00	6.42E-48	2.76E+00	1.74E-16

ENSBTAG00000009029	1.55E-01	1.00E+00	-1.83E-01	7.93E-01	-4.72E-01	1.69E-01	-7.77E-01	1.19E-02	6.73E-01	2.01E-01
ENSBTAG00000024926	-9.27E-01	8.83E-01	-1.73E+00	1.89E-01	-2.74E+00	3.34E-02	-1.12E+00	3.98E-01	-9.35E-01	8.46E-01
ENSBTAG00000013573	-1.71E-01	1.00E+00	-1.03E+00	1.18E-02	-1.68E+00	1.89E-05	-1.23E+00	2.20E-03	-7.86E-01	3.14E-01
ENSBTAG00000007125	-9.90E-01	1.52E-04	-1.51E+00	3.71E-11	-1.57E+00	3.78E-12	-2.34E+00	3.50E-24	1.86E-01	9.65E-01
ENSBTAG00000024914	0.00E+00	1.00E+00	0.00E+00	1.00E+00	-5.13E-15	1.00E+00	0.00E+00	1.00E+00	0.00E+00	1.00E+00
ENSBTAG00000007121	-5.85E-01	2.68E-01	-6.67E-02	1.00E+00	-6.41E-01	4.19E-02	-6.82E-01	3.19E-02	-3.14E-01	8.00E-01
ENSBTAG00000019069	-1.74E-01	1.00E+00	-3.80E-01	1.40E-01	1.43E-01	7.69E-01	3.85E-01	1.40E-01	1.92E-01	9.13E-01
ENSBTAG00000019067	-4.22E-01	5.93E-01	-8.28E-01	7.09E-03	-5.63E-01	8.50E-02	-6.31E-01	5.25E-02	-1.65E-01	1.00E+00
ENSBTAG00000018661	1.40E-01	1.00E+00	-6.65E-01	5.33E-03	-8.30E-01	3.25E-04	-6.29E-01	1.01E-02	2.23E-01	8.49E-01
ENSBTAG00000044955	0.00E+00	1.00E+00	0.00E+00	1.00E+00	-5.13E-15	1.00E+00	0.00E+00	1.00E+00	0.00E+00	1.00E+00
ENSBTAG00000018658	1.81E-01	1.00E+00	6.48E-02	1.00E+00	1.50E-01	8.79E-01	4.81E-01	1.55E-01	4.72E-01	5.09E-01
ENSBTAG00000043529	0.00E+00	1.00E+00	0.00E+00	1.00E+00	-5.13E-15	1.00E+00	0.00E+00	1.00E+00	0.00E+00	1.00E+00
ENSBTAG00000002633	-9.18E-02	1.00E+00	-1.09E+00	1.59E-05	-6.41E-01	1.60E-02	-2.17E-01	5.98E-01	-1.79E-01	1.00E+00
ENSBTAG00000013677	1.74E-01	1.00E+00	1.11E-01	9.24E-01	3.88E-01	1.34E-01	7.38E-01	1.14E-03	4.44E-02	1.00E+00
ENSBTAG00000044443	8.29E-01	4.32E-01	1.88E+00	1.72E-05	1.44E+00	1.60E-03	-8.77E-01	2.17E-01	-1.24E-01	1.00E+00
ENSBTAG00000005104	3.19E-03	1.00E+00	7.72E-01	4.43E-01	1.90E-01	1.00E+00	6.26E-01	5.71E-01	2.82E-02	1.00E+00
ENSBTAG00000018260	-3.18E-01	9.28E-01	-8.26E-01	2.83E-02	-6.37E-01	1.11E-01	4.03E-02	1.00E+00	2.20E-01	1.00E+00
ENSBTAG00000018258	-6.19E-01	3.53E-02	-9.44E-01	1.03E-05	-1.17E-01	8.70E-01	-3.50E-01	1.86E-01	-6.01E-01	7.82E-02
ENSBTAG00000044138	-1.96E-01	1.00E+00	-7.05E-01	3.19E-02	-1.28E+00	2.47E-05	-1.94E+00	2.11E-10	-4.23E-01	6.52E-01
ENSBTAG00000019153	7.41E-01	8.53E-03	1.90E+00	1.38E-18	1.80E-01	6.48E-01	1.05E-01	9.51E-01	-1.66E-01	1.00E+00
ENSBTAG00000001998	-1.91E+00	8.23E-01	7.57E-02	1.00E+00	-1.25E-01	1.00E+00	-1.42E-01	1.00E+00	7.99E-01	1.00E+00
ENSBTAG00000015255	-3.77E-01	6.01E-01	-1.74E+00	2.44E-10	-4.71E-01	1.14E-01	-5.35E-01	7.00E-02	-1.53E-01	1.00E+00
ENSBTAG00000043414	1.28E+00	1.32E-01	1.67E+00	1.92E-03	2.15E+00	1.34E-05	9.43E-01	1.53E-01	4.25E-03	1.00E+00
ENSBTAG00000042461	3.29E-01	1.00E+00	-1.60E+00	4.24E-01	2.46E+00	4.05E-03	1.41E+00	2.14E-01	-3.59E+00	3.17E-01
ENSBTAG00000042706	1.26E-02	1.00E+00	1.40E+00	5.16E-01	1.15E+00	6.42E-01	-1.91E+00	4.39E-01	-1.91E+00	8.00E-01
ENSBTAG00000039607	1.91E+00	8.23E-01	0.00E+00	1.00E+00	3.06E+00	1.36E-01	1.77E+00	4.97E-01	1.91E+00	8.00E-01
ENSBTAG00000005556	-3.67E-01	8.65E-01	-2.85E+00	6.64E-11	-1.16E+00	3.09E-03	-8.92E-01	2.96E-02	2.38E-01	1.00E+00
ENSBTAG00000003436	7.54E-01	6.41E-02	1.13E+00	6.10E-05	1.61E+00	2.73E-09	1.05E+00	2.52E-04	-2.21E-01	1.00E+00
ENSBTAG00000039119	3.31E+00	3.11E-02	3.14E+00	1.08E-02	3.51E+00	1.77E-03	3.06E+00	1.31E-02	2.23E+00	4.90E-01
ENSBTAG00000014682	3.23E+00	3.75E-01	1.98E+00	4.43E-01	1.78E+00	4.79E-01	0.00E+00	1.00E+00	1.91E+00	8.00E-01
ENSBTAG00000020115	-2.12E-01	9.47E-01	1.04E+00	1.06E-05	8.87E-01	2.13E-04	5.89E-01	2.40E-02	9.98E-02	1.00E+00
ENSBTAG00000008507	4.31E-01	8.63E-01	8.20E-01	8.77E-02	9.81E-01	2.94E-02	8.29E-01	8.64E-02	1.48E-01	1.00E+00
ENSBTAG00000030172	-2.36E-01	9.06E-01	-1.34E+00	6.75E-08	-1.08E+00	1.33E-05	-7.05E-01	7.98E-03	1.67E-01	1.00E+00
ENSBTAG00000030173	-5.44E-01	5.54E-01	-1.09E+00	3.88E-03	-6.23E-01	1.20E-01	-1.00E+00	7.66E-03	5.50E-01	5.18E-01
ENSBTAG00000037261	0.00E+00	1.00E+00	0.00E+00	1.00E+00	-5.13E-15	1.00E+00	0.00E+00	1.00E+00	0.00E+00	1.00E+00

ENSBTAG00000013792	1.85E+00	4.17E-03	1.56E+00	4.18E-03	1.16E+00	4.17E-02	-2.32E-02	1.00E+00	1.11E-01	1.00E+00
ENSBTAG00000016240	9.84E-02	1.00E+00	-9.68E-01	1.49E-03	-8.32E-01	6.82E-03	-6.79E-01	3.73E-02	2.17E-01	1.00E+00
ENSBTAG00000007916	-2.51E-01	1.00E+00	-1.09E+00	1.41E-01	-1.01E+00	1.70E-01	-1.14E+00	1.16E-01	-2.88E-01	1.00E+00
ENSBTAG00000007910	-3.77E-02	1.00E+00	-8.88E-01	1.57E-05	-7.57E-01	2.55E-04	-4.38E-01	5.97E-02	1.73E-01	9.37E-01
ENSBTAG00000007906	-1.92E-01	1.00E+00	-3.96E-01	3.52E-01	-5.93E-01	1.02E-01	-5.81E-01	1.19E-01	-3.09E-01	8.50E-01
ENSBTAG00000024910	0.00E+00	1.00E+00	2.79E+00	2.10E-01	-5.13E-15	1.00E+00	0.00E+00	1.00E+00	0.00E+00	1.00E+00
ENSBTAG00000009228	1.41E-01	1.00E+00	1.14E+00	2.72E-08	1.16E+00	1.04E-08	9.52E-01	5.05E-06	-6.97E-02	1.00E+00
ENSBTAG00000010517	-1.51E+00	1.13E-01	5.50E-01	5.08E-01	-1.47E+00	2.49E-02	-1.55E+00	1.92E-02	-9.89E-01	5.26E-01
ENSBTAG00000010509	-5.33E-01	1.44E-01	-1.28E+00	1.33E-08	-2.37E+00	1.27E-25	-3.40E+00	1.40E-46	2.75E-01	7.87E-01
ENSBTAG00000044038	-3.52E-01	3.78E-01	-1.06E+00	2.73E-08	-1.85E+00	1.37E-22	-2.21E+00	6.04E-31	2.85E-01	6.09E-01
ENSBTAG00000030174	-8.72E-01	6.12E-02	-1.32E+00	5.25E-05	-1.90E+00	3.76E-09	-1.56E+00	1.84E-06	4.13E-01	7.53E-01
ENSBTAG00000030175	1.64E-01	1.00E+00	-4.35E-01	1.07E-01	-1.57E-01	7.50E-01	-4.78E-01	7.25E-02	4.17E-01	4.34E-01
ENSBTAG00000044918	0.00E+00	1.00E+00	0.00E+00	1.00E+00	-5.13E-15	1.00E+00	0.00E+00	1.00E+00	0.00E+00	1.00E+00
ENSBTAG00000022009	-2.46E-02	1.00E+00	-7.32E-01	9.28E-03	-3.69E-01	2.75E-01	2.03E-01	6.86E-01	1.44E-01	1.00E+00
ENSBTAG00000022006	-4.15E-01	5.76E-01	-1.52E+00	3.61E-08	-2.99E-01	4.30E-01	-3.16E-01	4.17E-01	1.19E-01	1.00E+00
ENSBTAG00000018920	-4.43E-01	6.64E-01	-2.25E+00	5.89E-12	-1.74E+00	6.66E-08	-8.17E-01	2.00E-02	6.56E-01	3.28E-01
ENSBTAG00000008634	-1.75E-01	9.69E-01	-7.16E-01	6.11E-04	-9.40E-01	3.69E-06	-4.90E-01	3.04E-02	1.00E-01	1.00E+00
ENSBTAG00000013556	-5.61E-03	1.00E+00	-2.31E-02	1.00E+00	-2.22E-01	7.31E-01	-5.27E-01	1.77E-01	2.35E-01	1.00E+00
ENSBTAG00000016287	2.65E-02	1.00E+00	3.75E-01	1.07E-01	3.89E-01	8.90E-02	2.91E-01	2.62E-01	2.86E-01	6.49E-01
ENSBTAG00000024909	2.43E-01	7.81E-01	5.91E-01	4.33E-03	8.94E-01	5.72E-06	4.20E-01	6.48E-02	-1.69E-01	9.37E-01
ENSBTAG00000014964	2.84E-02	1.00E+00	-6.67E-01	1.40E-01	-1.76E+00	5.70E-06	-2.38E+00	1.32E-09	3.46E-01	9.01E-01
ENSBTAG00000018169	-3.54E-01	8.23E-01	-1.43E+00	2.01E-05	-2.67E+00	5.62E-15	-2.70E+00	3.99E-15	5.50E-01	5.03E-01
ENSBTAG00000011717	-1.15E-02	1.00E+00	1.19E+00	1.27E-10	1.33E+00	5.00E-13	9.65E-01	3.26E-07	-3.84E-02	1.00E+00
ENSBTAG00000011715	1.18E-02	1.00E+00	-6.25E-01	1.76E-02	-7.68E-01	2.42E-03	-1.32E+00	7.43E-08	2.47E-01	8.31E-01
ENSBTAG00000047510	0.00E+00	1.00E+00	0.00E+00	1.00E+00	-5.13E-15	1.00E+00	0.00E+00	1.00E+00	0.00E+00	1.00E+00
ENSBTAG00000010758	0.00E+00	1.00E+00	0.00E+00	1.00E+00	-5.13E-15	1.00E+00	0.00E+00	1.00E+00	0.00E+00	1.00E+00
ENSBTAG00000011713	-3.86E-02	1.00E+00	2.93E-02	1.00E+00	-1.08E+00	9.23E-02	-1.63E+00	9.50E-03	5.34E-01	8.23E-01
ENSBTAG00000020067	-5.44E-01	2.15E-01	-1.90E+00	3.78E-14	-3.04E+00	1.25E-31	-1.80E+00	8.69E-13	1.26E-01	1.00E+00
ENSBTAG00000000824	-8.51E-01	4.02E-03	-1.24E+00	2.84E-07	-4.77E-02	1.00E+00	-1.55E-01	7.89E-01	-1.01E-01	1.00E+00
ENSBTAG00000007220	-8.66E-01	2.80E-03	-2.86E+00	1.83E-31	-2.09E+00	1.04E-18	-1.18E+00	8.40E-07	1.67E-01	1.00E+00
ENSBTAG00000007217	-2.42E-01	8.23E-01	-1.48E+00	3.93E-11	-1.73E+00	7.84E-15	-8.74E-01	1.69E-04	5.95E-01	1.18E-01
ENSBTAG00000043339	0.00E+00	1.00E+00	0.00E+00	1.00E+00	-5.13E-15	1.00E+00	0.00E+00	1.00E+00	0.00E+00	1.00E+00
ENSBTAG00000004736	1.75E-01	9.82E-01	2.08E-01	5.00E-01	2.56E-01	3.66E-01	1.29E-01	8.15E-01	4.65E-01	2.54E-01
ENSBTAG00000000202	9.31E-01	1.41E-02	1.07E+00	3.53E-04	3.03E-02	1.00E+00	-6.91E-01	3.92E-02	-6.37E-02	1.00E+00
ENSBTAG00000045475	0.00E+00	1.00E+00	0.00E+00	1.00E+00	-5.13E-15	1.00E+00	0.00E+00	1.00E+00	0.00E+00	1.00E+00

ENSBTAG00000030168	-3.46E-01	6.83E-01	-1.20E+00	3.14E-06	-1.49E+00	4.22E-09	-5.58E-01	5.25E-02	3.46E-01	6.83E-01
ENSBTAG00000016133	-1.66E-01	1.00E+00	1.82E-01	6.80E-01	4.36E-01	1.10E-01	3.84E-01	1.87E-01	-1.68E-01	1.00E+00
ENSBTAG00000016128	-1.58E-01	9.90E-01	1.15E-01	8.27E-01	1.58E-01	6.23E-01	-7.57E-02	1.00E+00	3.06E-01	5.59E-01
ENSBTAG00000016126	-1.23E-01	1.00E+00	4.38E-01	3.66E-02	7.87E-01	3.50E-05	2.48E-01	3.37E-01	-1.49E-01	9.93E-01
ENSBTAG00000030169	-1.95E-01	9.28E-01	-2.68E-01	3.43E-01	-1.30E-01	8.06E-01	-1.29E-01	8.18E-01	-7.10E-01	1.84E-02
ENSBTAG00000030170	2.36E-02	1.00E+00	2.76E-01	2.91E-01	3.73E-01	1.07E-01	1.56E-01	6.79E-01	-3.49E-01	5.03E-01
ENSBTAG00000011527	-1.33E-01	1.00E+00	-1.48E-01	9.30E-01	2.60E-01	5.99E-01	2.48E-01	6.41E-01	-3.84E-01	7.62E-01
ENSBTAG00000014799	-5.66E-01	1.25E-01	-1.44E-01	8.15E-01	-1.87E-01	6.43E-01	-4.85E-01	6.97E-02	-1.91E-01	9.59E-01
ENSBTAG00000001110	-1.17E+00	1.94E-02	-3.27E+00	1.24E-16	-4.23E+00	5.05E-25	-4.18E+00	1.75E-24	-1.50E+00	2.69E-03
ENSBTAG00000026645	-5.57E-01	1.00E+00	-1.91E+00	1.17E-01	1.08E-01	1.00E+00	3.14E-01	9.76E-01	6.08E-01	9.34E-01
ENSBTAG00000045522	0.00E+00	1.00E+00	0.00E+00	1.00E+00	-5.13E-15	1.00E+00	0.00E+00	1.00E+00	0.00E+00	1.00E+00
ENSBTAG00000021231	-3.27E-01	8.31E-01	-3.42E-01	4.43E-01	3.73E-01	3.77E-01	8.76E-01	7.71E-03	-4.88E-02	1.00E+00
ENSBTAG00000021227	-1.17E-01	1.00E+00	-2.22E-01	5.68E-01	-3.90E-01	1.94E-01	-3.54E-01	2.66E-01	-2.76E-01	8.00E-01
ENSBTAG00000003303	-6.83E-02	1.00E+00	2.22E-01	5.28E-01	3.11E-01	3.00E-01	2.54E-01	4.53E-01	-2.69E-01	8.00E-01
ENSBTAG00000039466	2.61E-01	8.96E-01	-9.57E-01	1.61E-03	7.81E-02	1.00E+00	3.35E-01	3.64E-01	2.14E-02	1.00E+00
ENSBTAG00000010809	-2.71E-01	9.32E-01	-8.90E-01	4.67E-03	-2.82E-01	5.28E-01	-7.50E-01	2.21E-02	-2.03E-01	1.00E+00
ENSBTAG00000040111	-3.69E-01	1.00E+00	8.47E-02	1.00E+00	1.33E+00	2.19E-01	-5.66E-01	9.20E-01	-3.68E-01	1.00E+00
ENSBTAG00000021257	8.96E-01	1.00E+00	5.96E-01	9.45E-01	3.48E-01	1.00E+00	-9.46E-01	8.04E-01	8.92E-01	1.00E+00
ENSBTAG00000017252	1.99E+00	5.76E-01	-1.91E+00	4.69E-01	2.06E+00	1.82E-01	1.78E+00	3.05E-01	2.46E+00	3.61E-01
ENSBTAG00000017248	2.66E-01	9.02E-01	5.01E-03	1.00E+00	-1.72E-01	8.10E-01	-1.35E-01	9.38E-01	-2.17E-01	9.98E-01
ENSBTAG00000017242	-1.31E-01	1.00E+00	-8.10E-01	4.47E-06	-1.55E+00	1.99E-19	-1.94E+00	6.15E-29	1.30E-02	1.00E+00
ENSBTAG00000006525	1.62E-01	1.00E+00	-1.39E-02	1.00E+00	1.07E-01	9.69E-01	3.78E-01	2.17E-01	1.45E-01	1.00E+00
ENSBTAG00000040042	-6.35E-01	6.88E-01	-1.81E+00	9.80E-04	-1.35E+00	9.42E-03	-7.94E-01	1.61E-01	1.88E-01	1.00E+00
ENSBTAG00000011389	6.51E-01	3.75E-01	7.75E-01	4.79E-02	1.51E+00	1.14E-05	2.21E+00	5.84E-11	7.03E-01	3.64E-01
ENSBTAG00000011387	-2.36E-01	8.23E-01	2.23E-01	4.69E-01	6.50E-01	2.61E-03	5.06E-01	2.89E-02	2.42E-02	1.00E+00
ENSBTAG00000011382	4.83E-02	1.00E+00	2.11E-01	5.78E-01	3.45E-02	1.00E+00	1.38E-01	8.44E-01	-1.00E-02	1.00E+00
ENSBTAG00000042961	0.00E+00	1.00E+00	0.00E+00	1.00E+00	-5.13E-15	1.00E+00	0.00E+00	1.00E+00	0.00E+00	1.00E+00
ENSBTAG00000008092	7.43E-02	1.00E+00	-6.21E-01	6.55E-02	-4.15E-01	2.71E-01	-6.79E-01	4.23E-02	-2.70E-01	9.00E-01
ENSBTAG00000002773	0.00E+00	1.00E+00	0.00E+00	1.00E+00	-5.13E-15	1.00E+00	0.00E+00	1.00E+00	0.00E+00	1.00E+00
ENSBTAG00000004690	-6.67E-01	1.00E+00	-1.90E+00	2.55E-01	-1.33E+00	4.15E-01	4.99E-02	1.00E+00	-1.96E+00	6.03E-01
ENSBTAG00000039449	0.00E+00	1.00E+00	0.00E+00	1.00E+00	-5.13E-15	1.00E+00	0.00E+00	1.00E+00	0.00E+00	1.00E+00
ENSBTAG00000046693	-9.52E-01	8.23E-01	-1.78E+00	9.66E-02	-7.59E-01	5.01E-01	-5.80E-01	6.83E-01	-3.18E+00	7.44E-02
ENSBTAG00000024901	-9.94E-01	3.75E-01	-2.68E+00	1.80E-05	-7.45E-01	2.61E-01	-3.97E-01	6.86E-01	-5.73E-01	8.00E-01
ENSBTAG00000008328	-2.90E-01	1.00E+00	-5.74E-01	8.95E-01	7.84E-01	5.65E-01	-4.78E-01	9.48E-01	2.59E-01	1.00E+00
ENSBTAG00000008327	1.98E-01	1.00E+00	8.63E-01	3.26E-01	1.45E+00	3.18E-02	2.37E+00	7.16E-05	2.14E-01	1.00E+00

ENSBTAG00000000812	2.71E-01	1.00E+00	7.21E-01	5.77E-02	1.20E+00	4.16E-04	5.38E-01	2.05E-01	7.24E-01	3.06E-01
ENSBTAG00000018663	1.16E+00	8.21E-05	3.35E+00	7.94E-36	2.30E+00	5.50E-19	1.16E+00	1.08E-05	6.28E-01	1.92E-01
ENSBTAG00000013959	5.22E-03	1.00E+00	8.73E-01	8.53E-01	-1.91E+00	4.28E-01	-1.91E+00	4.39E-01	1.68E+00	7.77E-01
ENSBTAG00000030166	-4.29E-01	1.00E+00	-3.49E-01	9.61E-01	-1.55E+00	1.35E-01	-1.95E+00	6.69E-02	-1.76E+00	4.29E-01
ENSBTAG00000004617	1.13E-01	1.00E+00	9.00E-02	1.00E+00	-2.66E+00	1.11E-02	-1.07E+00	3.15E-01	1.40E+00	2.49E-01
ENSBTAG00000016732	7.72E-01	1.00E+00	1.03E+00	4.66E-01	-1.81E+00	3.05E-01	1.39E+00	2.19E-01	-1.61E-02	1.00E+00
ENSBTAG00000011007	-5.94E-01	6.33E-02	-1.94E+00	7.89E-19	-2.42E+00	4.32E-28	-1.21E+00	3.65E-08	-3.36E-01	6.01E-01
ENSBTAG00000030164	2.37E-02	1.00E+00	2.47E-01	3.90E-01	1.91E-01	5.48E-01	-9.00E-03	1.00E+00	-3.42E-01	5.32E-01
ENSBTAG00000047075	0.00E+00	1.00E+00	0.00E+00	1.00E+00	-5.13E-15	1.00E+00	0.00E+00	1.00E+00	0.00E+00	1.00E+00
ENSBTAG00000044195	1.83E-01	1.00E+00	-3.44E-01	4.67E-01	-6.38E-01	8.55E-02	3.97E-01	3.50E-01	6.96E-01	2.52E-01
ENSBTAG00000019115	-4.91E-01	5.00E-01	-1.89E+00	2.31E-10	-1.75E+00	3.76E-09	-8.25E-01	1.04E-02	-2.17E-02	1.00E+00
ENSBTAG00000006089	2.74E-01	7.00E-01	1.90E+00	3.84E-22	1.71E+00	2.27E-18	1.04E+00	2.68E-07	-6.99E-02	1.00E+00
ENSBTAG00000006088	-2.31E-02	1.00E+00	1.01E+00	8.07E-06	1.25E+00	1.72E-08	1.10E+00	1.12E-06	1.52E-01	1.00E+00
ENSBTAG00000006087	-8.00E-02	1.00E+00	3.50E-01	1.70E-01	3.58E-01	1.56E-01	1.40E-01	7.80E-01	4.40E-02	1.00E+00
ENSBTAG00000017136	0.00E+00	1.00E+00	0.00E+00	1.00E+00	-5.13E-15	1.00E+00	4.32E+00	9.50E-03	0.00E+00	1.00E+00
ENSBTAG00000018837	-5.06E-02	1.00E+00	2.63E-01	5.80E-01	3.05E-01	4.79E-01	2.53E-01	6.14E-01	2.82E-01	8.74E-01
ENSBTAG00000045824	2.24E+00	5.96E-04	2.67E+00	7.79E-07	3.79E+00	7.97E-15	2.80E+00	1.27E-07	1.79E+00	4.52E-02
ENSBTAG00000043304	0.00E+00	1.00E+00	0.00E+00	1.00E+00	-5.13E-15	1.00E+00	0.00E+00	1.00E+00	1.91E+00	8.00E-01
ENSBTAG00000008294	8.39E-01	4.15E-01	-2.86E+00	2.27E-08	-2.99E+00	3.73E-09	-2.74E+00	7.09E-08	7.03E-01	6.09E-01
ENSBTAG00000024903	1.33E+00	4.99E-01	9.10E-01	4.69E-01	1.04E+00	3.50E-01	-1.89E-01	1.00E+00	1.22E+00	6.12E-01
ENSBTAG00000001609	1.14E+00	6.08E-02	1.62E+00	1.78E-04	1.21E+00	6.54E-03	5.43E-01	3.42E-01	6.82E-01	5.58E-01
ENSBTAG00000002747	1.26E-01	1.00E+00	-8.92E-01	2.39E-03	-5.73E-01	7.23E-02	-8.09E-01	7.46E-03	2.59E-01	8.97E-01
ENSBTAG00000006937	7.73E-01	6.41E-01	-1.09E+00	8.07E-02	-1.90E+00	9.32E-04	-2.23E+00	1.14E-04	8.68E-01	5.82E-01
ENSBTAG00000006921	1.26E+00	5.64E-07	1.23E+00	1.47E-07	1.21E+00	1.71E-07	7.92E-01	1.25E-03	1.42E+00	4.67E-08
ENSBTAG00000006896	3.57E-01	1.00E+00	2.59E+00	1.36E-07	5.57E-01	4.10E-01	-7.29E-01	2.36E-01	5.97E-01	7.74E-01
ENSBTAG00000024899	2.27E-01	1.00E+00	-1.77E+00	3.96E-03	-3.85E+00	2.72E-09	-5.24E+00	5.19E-13	5.68E-01	8.28E-01
ENSBTAG00000001085	4.65E-01	2.24E-01	2.18E-02	1.00E+00	-1.25E+00	6.52E-09	-1.25E+00	8.85E-09	7.37E-01	1.67E-02
ENSBTAG00000008621	-1.47E-01	1.00E+00	-8.24E-02	9.55E-01	-4.44E-02	1.00E+00	-1.70E-01	5.54E-01	1.14E-01	1.00E+00
ENSBTAG00000010637	-1.92E-01	1.00E+00	-1.30E+00	2.06E-07	-2.02E+00	4.64E-16	-1.63E+00	5.50E-11	9.17E-01	5.04E-03
ENSBTAG00000039556	8.60E-02	1.00E+00	-9.78E-01	1.93E-03	-5.86E-01	8.74E-02	5.05E-01	1.59E-01	8.92E-01	4.80E-02
ENSBTAG00000003074	8.40E-01	3.75E-01	-1.27E+00	1.03E-02	-1.02E+00	4.63E-02	-2.46E-01	8.87E-01	7.53E-01	5.43E-01
ENSBTAG00000009345	-2.04E-01	9.29E-01	-4.67E-01	5.87E-02	-4.41E-01	7.79E-02	-3.77E-01	1.60E-01	-1.07E-02	1.00E+00
ENSBTAG00000045292	0.00E+00	1.00E+00	0.00E+00	1.00E+00	-5.13E-15	1.00E+00	0.00E+00	1.00E+00	0.00E+00	1.00E+00
ENSBTAG00000046174	5.20E-01	7.38E-01	1.20E+00	4.06E-03	1.45E+00	3.45E-04	1.55E+00	1.48E-04	1.69E-01	1.00E+00
ENSBTAG00000047055	-2.38E-01	1.00E+00	-4.50E-01	4.37E-01	-7.91E-01	8.34E-02	-9.17E-01	4.28E-02	2.26E-01	1.00E+00

ENSBTAG00000017751	1.86E-01	1.00E+00	4.67E-01	3.51E-01	1.64E-02	1.00E+00	-1.30E-01	1.00E+00	1.52E-01	1.00E+00
ENSBTAG00000030162	-1.62E+00	1.98E-07	-3.13E+00	9.69E-25	-1.18E+00	5.21E-05	-8.15E-01	8.17E-03	-8.14E-01	7.60E-02
ENSBTAG00000033680	2.00E-01	1.00E+00	-5.95E-02	1.00E+00	-8.14E-01	5.44E-02	-1.09E+00	7.81E-03	1.52E+00	1.32E-03
ENSBTAG00000001915	-1.03E-01	1.00E+00	-8.58E-01	2.38E-06	-1.64E+00	2.95E-20	-1.96E+00	8.17E-28	-2.67E-01	6.23E-01
ENSBTAG00000044628	0.00E+00	1.00E+00	0.00E+00	1.00E+00	-5.13E-15	1.00E+00	1.76E+00	4.97E-01	0.00E+00	1.00E+00
ENSBTAG00000001061	2.72E-02	1.00E+00	-7.28E-02	1.00E+00	1.14E-01	9.84E-01	7.88E-01	5.72E-03	2.43E-01	9.11E-01
ENSBTAG00000012311	-1.98E+00	5.76E-01	-1.91E+00	2.53E-01	-8.34E-01	6.68E-01	-2.12E+00	1.78E-01	-1.97E+00	6.01E-01
ENSBTAG00000048289	0.00E+00	1.00E+00	0.00E+00	1.00E+00	-5.13E-15	1.00E+00	0.00E+00	1.00E+00	0.00E+00	1.00E+00
ENSBTAG00000000589	-1.53E-01	1.00E+00	-2.24E+00	3.45E-03	-3.44E+00	7.91E-06	-3.51E+00	7.29E-06	6.99E-01	8.44E-01
ENSBTAG00000007547	-2.07E+00	1.53E-01	-6.70E-01	5.70E-01	-7.66E-01	4.62E-01	-1.15E+00	2.28E-01	-9.27E-01	7.83E-01
ENSBTAG00000004746	1.96E-01	9.17E-01	-3.59E-01	1.50E-01	-7.00E-01	1.14E-03	-4.30E-01	7.43E-02	4.94E-01	2.01E-01
ENSBTAG00000039227	0.00E+00	1.00E+00	2.79E+00	2.10E-01	-5.13E-15	1.00E+00	0.00E+00	1.00E+00	1.91E+00	8.00E-01
ENSBTAG00000010051	-3.21E+00	3.75E-01	4.71E-01	9.93E-01	7.53E-01	7.19E-01	1.88E+00	6.90E-02	-1.29E+00	9.03E-01
ENSBTAG00000009831	-5.96E-01	9.74E-01	3.18E-01	9.31E-01	-7.73E-01	4.35E-01	9.18E-01	2.41E-01	-9.71E-01	7.14E-01
ENSBTAG00000040536	0.00E+00	1.00E+00	0.00E+00	1.00E+00	-5.13E-15	1.00E+00	0.00E+00	1.00E+00	0.00E+00	1.00E+00
ENSBTAG00000043266	0.00E+00	1.00E+00	0.00E+00	1.00E+00	-5.13E-15	1.00E+00	0.00E+00	1.00E+00	0.00E+00	1.00E+00
ENSBTAG00000013916	8.43E-02	1.00E+00	1.61E-01	7.52E-01	2.17E-01	5.48E-01	-2.47E-02	1.00E+00	3.28E-01	6.63E-01
ENSBTAG00000002316	-5.19E-01	8.82E-01	-2.16E+00	1.72E-03	-1.16E+00	6.30E-02	-1.40E+00	2.66E-02	1.57E-01	1.00E+00
ENSBTAG00000016895	-1.88E-02	1.00E+00	-8.27E-03	1.00E+00	5.14E-02	1.00E+00	-1.68E-02	1.00E+00	7.82E-02	1.00E+00
ENSBTAG00000016894	1.33E-01	1.00E+00	-1.42E-01	8.21E-01	-5.08E-01	4.69E-02	-3.36E-01	2.62E-01	1.88E-01	9.63E-01
ENSBTAG00000014714	-3.36E-01	6.76E-01	-2.27E+00	5.67E-20	-7.49E-01	3.35E-03	-7.15E-01	6.39E-03	-2.09E-01	9.37E-01
ENSBTAG00000015841	-4.64E-01	3.36E-01	-1.37E+00	1.23E-08	-1.73E+00	3.76E-13	-1.04E+00	2.51E-05	-4.25E-01	4.75E-01
ENSBTAG00000003225	-2.18E-01	9.92E-01	4.63E-01	1.36E-01	1.03E+00	1.08E-04	1.08E+00	5.85E-05	-1.85E-01	1.00E+00
ENSBTAG00000011658	-6.04E-01	3.75E-01	3.95E-01	3.78E-01	-2.75E-01	6.14E-01	-8.81E-01	1.32E-02	-4.03E-01	7.79E-01
ENSBTAG00000038318	0.00E+00	1.00E+00	0.00E+00	1.00E+00	1.78E+00	4.79E-01	0.00E+00	1.00E+00	0.00E+00	1.00E+00
ENSBTAG00000015834	-3.07E-01	1.00E+00	1.86E+00	2.75E-06	3.03E-02	1.00E+00	-1.40E-01	1.00E+00	-3.60E-01	1.00E+00
ENSBTAG00000034978	-1.36E-01	1.00E+00	7.12E-01	5.28E-05	9.30E-01	6.11E-08	7.48E-01	2.39E-05	-4.14E-02	1.00E+00
ENSBTAG00000000941	-3.49E-01	4.15E-01	-3.99E-01	7.50E-02	-1.86E-01	5.32E-01	-6.90E-02	1.00E+00	-2.17E-01	8.00E-01
ENSBTAG00000047403	0.00E+00	1.00E+00	0.00E+00	1.00E+00	-5.13E-15	1.00E+00	0.00E+00	1.00E+00	0.00E+00	1.00E+00
ENSBTAG00000043294	0.00E+00	1.00E+00	0.00E+00	1.00E+00	-5.13E-15	1.00E+00	0.00E+00	1.00E+00	0.00E+00	1.00E+00
ENSBTAG00000042866	0.00E+00	1.00E+00	0.00E+00	1.00E+00	-5.13E-15	1.00E+00	0.00E+00	1.00E+00	0.00E+00	1.00E+00
ENSBTAG00000047034	0.00E+00	1.00E+00	0.00E+00	1.00E+00	-5.13E-15	1.00E+00	0.00E+00	1.00E+00	0.00E+00	1.00E+00
ENSBTAG00000006157	2.10E-01	1.00E+00	1.71E+00	3.97E-10	4.02E-01	2.48E-01	2.00E-01	7.27E-01	7.07E-01	1.39E-01
ENSBTAG00000005444	1.63E-01	1.00E+00	1.80E+00	3.10E-07	1.78E+00	3.39E-07	2.07E+00	4.08E-09	8.60E-01	1.74E-01
ENSBTAG00000006003	-2.13E-01	1.00E+00	-1.64E+00	9.11E-09	-5.26E-01	1.03E-01	5.88E-01	6.24E-02	5.38E-02	1.00E+00

ENSBTAG00000008089	1.32E-01	1.00E+00	6.30E-01	6.85E-02	1.65E+00	2.02E-08	1.47E+00	1.06E-06	4.39E-01	6.46E-01
ENSBTAG00000010225	3.55E-02	1.00E+00	6.93E-01	4.33E-04	7.14E-01	2.54E-04	1.81E-01	5.56E-01	-7.76E-02	1.00E+00
ENSBTAG00000005987	-1.06E-01	1.00E+00	-1.15E-01	8.02E-01	-2.11E-02	1.00E+00	-4.75E-01	1.62E-02	-2.05E-01	8.00E-01
ENSBTAG00000005986	-2.23E-01	1.00E+00	-3.02E-01	5.08E-01	-3.47E-01	4.17E-01	-3.10E-01	4.97E-01	4.34E-01	6.09E-01
ENSBTAG00000006262	-7.30E-01	2.23E-01	-2.54E+00	2.27E-13	-3.87E+00	7.86E-27	-2.77E+00	1.76E-15	3.69E-01	8.00E-01
ENSBTAG00000006261	0.00E+00	1.00E+00	0.00E+00	1.00E+00	-5.13E-15	1.00E+00	0.00E+00	1.00E+00	0.00E+00	1.00E+00
ENSBTAG00000039803	6.35E-01	9.06E-01	-1.21E+00	1.32E-01	-2.74E+00	1.81E-04	-2.27E+00	2.27E-03	2.15E+00	3.59E-02
ENSBTAG00000021172	3.93E-01	2.23E-01	1.73E+00	1.21E-22	1.58E+00	2.40E-19	1.12E+00	3.70E-10	1.82E-01	8.48E-01
ENSBTAG00000018275	-5.52E-02	1.00E+00	7.43E-01	1.96E-02	8.86E-01	3.64E-03	1.34E+00	4.13E-06	1.08E-01	1.00E+00
ENSBTAG00000003950	2.06E-01	8.26E-01	-5.70E-01	5.08E-03	-1.42E+00	7.16E-14	-1.57E+00	1.49E-16	5.60E-01	6.65E-02
ENSBTAG00000004216	2.00E-01	1.00E+00	-1.49E-01	9.47E-01	-4.11E-01	3.35E-01	3.64E-01	4.25E-01	3.48E-01	8.00E-01
ENSBTAG00000020777	-1.52E-01	1.00E+00	-7.90E-01	7.52E-05	-1.69E-01	5.97E-01	-1.80E-01	5.66E-01	8.26E-02	1.00E+00
ENSBTAG00000038275	7.08E-01	1.00E+00	9.28E-01	4.83E-01	-1.59E+00	3.00E-01	8.22E-01	5.62E-01	-2.21E-01	1.00E+00
ENSBTAG00000034949	0.00E+00	1.00E+00	0.00E+00	1.00E+00	-5.13E-15	1.00E+00	0.00E+00	1.00E+00	0.00E+00	1.00E+00
ENSBTAG00000019177	5.08E-02	1.00E+00	-7.86E-01	3.58E-03	-9.22E-01	4.68E-04	-3.26E-01	3.58E-01	-1.47E-01	1.00E+00
ENSBTAG00000045010	0.00E+00	1.00E+00	0.00E+00	1.00E+00	-5.13E-15	1.00E+00	0.00E+00	1.00E+00	0.00E+00	1.00E+00
ENSBTAG00000017214	4.93E+00	8.25E-03	1.99E+00	4.44E-01	-5.13E-15	1.00E+00	0.00E+00	1.00E+00	3.26E+00	4.34E-01
ENSBTAG00000000266	7.63E-01	6.61E-02	1.21E+00	2.66E-05	1.16E-01	9.95E-01	3.88E-01	3.06E-01	1.37E-01	1.00E+00
ENSBTAG00000015678	-2.28E-01	1.00E+00	-8.47E-01	7.19E-02	3.97E-01	4.84E-01	7.54E-01	9.85E-02	3.32E-01	9.44E-01
ENSBTAG00000007719	-2.89E-01	7.52E-01	-1.68E+00	2.21E-12	-8.16E-02	1.00E+00	5.21E-01	3.31E-02	6.91E-02	1.00E+00
ENSBTAG00000007584	-4.43E-01	8.23E-01	-1.02E+00	1.32E-02	-1.33E+00	7.97E-04	-1.63E+00	3.90E-05	3.27E-02	1.00E+00
ENSBTAG00000007787	1.38E-02	1.00E+00	-4.26E-01	2.00E-01	-9.57E-01	5.41E-04	-1.06E+00	1.43E-04	-3.33E-01	7.79E-01
ENSBTAG00000026994	4.74E-01	4.10E-01	1.45E-01	8.86E-01	-2.59E-01	5.25E-01	-3.59E-01	3.24E-01	-6.36E-02	1.00E+00
ENSBTAG00000011808	-1.91E+00	8.23E-01	-1.91E+00	4.69E-01	-1.91E+00	4.28E-01	-1.91E+00	4.39E-01	7.05E-03	1.00E+00
ENSBTAG00000018795	-5.19E-02	1.00E+00	1.55E-01	7.61E-01	6.15E-01	1.04E-02	4.09E-01	1.33E-01	-3.77E-01	5.50E-01
ENSBTAG00000012561	-7.98E-03	1.00E+00	3.71E-01	1.60E-01	1.01E+00	3.42E-06	1.02E+00	3.43E-06	-3.82E-01	5.01E-01
ENSBTAG00000012557	4.95E-01	3.51E-01	1.81E+00	1.01E-12	3.42E-01	3.20E-01	2.17E-01	6.39E-01	-3.29E-01	7.78E-01
ENSBTAG00000012554	4.68E-01	7.73E-01	2.21E+00	1.15E-09	-3.02E-01	6.63E-01	-5.74E-01	2.55E-01	-8.54E-01	3.07E-01
ENSBTAG00000000492	-1.09E-01	1.00E+00	6.86E-01	1.43E-03	7.33E-01	5.67E-04	6.06E-01	6.52E-03	-1.35E-01	1.00E+00
ENSBTAG00000010498	-4.98E-01	2.94E-01	-1.88E+00	3.42E-14	-1.68E+00	9.86E-12	-6.31E-01	2.17E-02	-1.17E-01	1.00E+00
ENSBTAG00000003002	5.30E-02	1.00E+00	1.61E+00	1.54E-17	1.66E+00	7.89E-19	1.01E+00	2.11E-07	-4.16E-01	3.27E-01
ENSBTAG00000027020	2.38E-01	9.04E-01	7.36E-01	4.38E-03	7.27E-01	4.73E-03	7.21E-01	6.04E-03	-3.22E-02	1.00E+00
ENSBTAG00000045103	0.00E+00	1.00E+00	0.00E+00	1.00E+00	-5.13E-15	1.00E+00	0.00E+00	1.00E+00	0.00E+00	1.00E+00
ENSBTAG00000021466	-7.32E-02	1.00E+00	4.62E-01	4.14E-02	5.09E-01	2.17E-02	5.67E-01	1.04E-02	-4.57E-04	1.00E+00
ENSBTAG00000007141	-3.00E-01	1.00E+00	1.23E+00	5.61E-02	4.27E-01	7.09E-01	1.43E-01	1.00E+00	-1.24E+00	4.24E-01

ENSBTAG00000042939	0.00E+00	1.00E+00	0.00E+00	1.00E+00	-5.13E-15	1.00E+00	0.00E+00	1.00E+00	0.00E+00	1.00E+00
ENSBTAG00000046000	3.54E-02	1.00E+00	-4.48E-01	4.27E-01	-5.58E-01	2.54E-01	5.69E-01	1.90E-01	2.42E-01	1.00E+00
ENSBTAG00000008483	1.48E-01	1.00E+00	7.37E-01	4.02E-02	-8.16E-01	2.14E-02	4.75E-02	1.00E+00	-9.38E-01	7.31E-02
ENSBTAG00000007580	1.30E+00	9.32E-01	8.75E-01	8.53E-01	-1.91E+00	4.28E-01	1.78E+00	3.04E-01	5.36E-04	1.00E+00
ENSBTAG00000001683	-2.52E-01	1.00E+00	-4.06E-02	1.00E+00	-5.03E-02	1.00E+00	5.71E-01	6.03E-01	-2.76E-01	1.00E+00
ENSBTAG00000019929	1.12E+00	2.51E-02	2.15E+00	7.88E-09	2.51E+00	1.70E-11	2.75E+00	2.68E-13	8.98E-01	1.88E-01
ENSBTAG00000047275	0.00E+00	1.00E+00	0.00E+00	1.00E+00	-5.13E-15	1.00E+00	0.00E+00	1.00E+00	0.00E+00	1.00E+00
ENSBTAG00000021762	3.02E-04	1.00E+00	9.54E-01	1.03E-06	1.28E+00	2.67E-11	7.93E-01	7.62E-05	-5.54E-01	8.70E-02
ENSBTAG00000003645	2.09E-01	1.00E+00	9.40E-01	4.41E-01	3.19E-02	1.00E+00	9.13E-01	4.45E-01	-4.95E-01	1.00E+00
ENSBTAG00000048130	-4.54E+00	2.92E-02	8.22E-02	1.00E+00	-2.75E+00	3.20E-02	-2.78E+00	3.37E-02	-4.54E+00	5.75E-02
ENSBTAG00000026122	0.00E+00	1.00E+00	1.99E+00	4.43E-01	-5.13E-15	1.00E+00	0.00E+00	1.00E+00	0.00E+00	1.00E+00
ENSBTAG00000038064	-6.10E-01	8.23E-01	-7.17E-01	3.19E-01	-3.98E-01	6.66E-01	-3.16E-01	8.12E-01	-1.03E+00	4.34E-01
ENSBTAG00000042196	0.00E+00	1.00E+00	0.00E+00	1.00E+00	-5.13E-15	1.00E+00	0.00E+00	1.00E+00	0.00E+00	1.00E+00
ENSBTAG00000045221	0.00E+00	1.00E+00	0.00E+00	1.00E+00	-5.13E-15	1.00E+00	0.00E+00	1.00E+00	0.00E+00	1.00E+00
ENSBTAG00000009777	3.03E-01	8.23E-01	1.40E+00	2.20E-07	9.68E-01	4.93E-04	4.70E-01	1.58E-01	-1.76E-01	1.00E+00
ENSBTAG00000020961	1.12E-01	1.00E+00	-4.29E-01	2.10E-01	-1.07E+00	1.47E-04	-9.79E-01	7.17E-04	1.11E-01	1.00E+00
ENSBTAG00000033662	1.74E-01	1.00E+00	4.19E-01	1.21E-01	7.42E-01	1.77E-03	9.21E-01	8.88E-05	5.28E-02	1.00E+00
ENSBTAG00000010977	-6.06E-01	7.57E-01	-1.76E+00	5.25E-04	-2.72E+00	1.01E-07	-1.88E+00	2.16E-04	-6.42E-01	7.08E-01
ENSBTAG00000042402	0.00E+00	1.00E+00	0.00E+00	1.00E+00	-5.13E-15	1.00E+00	0.00E+00	1.00E+00	0.00E+00	1.00E+00
ENSBTAG00000008390	-1.41E-01	1.00E+00	3.50E-01	1.49E-01	7.07E-01	6.24E-04	6.00E-01	5.39E-03	-3.31E-01	5.69E-01
ENSBTAG00000012100	-1.58E-01	1.00E+00	-3.82E-01	1.48E-01	-1.92E-01	5.96E-01	-5.62E-01	2.10E-02	-1.12E-01	1.00E+00
ENSBTAG00000033523	0.00E+00	1.00E+00	0.00E+00	1.00E+00	-5.13E-15	1.00E+00	0.00E+00	1.00E+00	0.00E+00	1.00E+00
ENSBTAG00000044009	-3.87E-01	1.00E+00	-8.73E-02	1.00E+00	-1.10E+00	3.86E-01	-3.53E-02	1.00E+00	4.56E-01	1.00E+00
ENSBTAG00000000937	2.01E-01	9.32E-01	3.65E-01	1.65E-01	3.87E-01	1.33E-01	5.26E-01	2.95E-02	2.39E-01	8.00E-01
ENSBTAG00000001755	0.00E+00	1.00E+00	1.98E+00	4.43E-01	-5.13E-15	1.00E+00	1.76E+00	4.97E-01	0.00E+00	1.00E+00
ENSBTAG00000009257	1.36E-01	1.00E+00	-8.84E-01	2.19E-02	-1.27E+00	5.06E-04	-1.49E+00	5.38E-05	-2.99E-01	9.32E-01
ENSBTAG00000009256	2.94E-01	8.75E-01	3.78E-01	3.39E-01	1.86E-01	8.00E-01	-4.68E-01	2.14E-01	-3.47E-01	8.00E-01
ENSBTAG00000037365	0.00E+00	1.00E+00	0.00E+00	1.00E+00	-5.13E-15	1.00E+00	0.00E+00	1.00E+00	0.00E+00	1.00E+00
ENSBTAG00000043956	-1.96E-01	1.00E+00	-9.84E-01	3.12E-04	-6.90E-01	1.49E-02	-1.37E-02	1.00E+00	-9.91E-02	1.00E+00
ENSBTAG00000045838	0.00E+00	1.00E+00	2.79E+00	2.10E-01	-5.13E-15	1.00E+00	0.00E+00	1.00E+00	0.00E+00	1.00E+00
ENSBTAG00000028219	-1.91E+00	8.23E-01	-1.91E+00	4.69E-01	-1.91E+00	4.28E-01	-1.91E+00	4.39E-01	-1.91E+00	8.00E-01
ENSBTAG00000021726	1.53E-01	1.00E+00	9.48E-01	3.38E-06	1.16E+00	6.17E-09	6.09E-01	5.23E-03	-2.30E-01	8.00E-01
ENSBTAG00000045485	0.00E+00	1.00E+00	0.00E+00	1.00E+00	-5.13E-15	1.00E+00	0.00E+00	1.00E+00	0.00E+00	1.00E+00
ENSBTAG00000003120	3.60E-01	8.23E-01	3.89E+00	1.49E-28	3.95E+00	1.99E-29	2.82E+00	9.00E-17	-6.09E-01	4.47E-01
ENSBTAG00000046904	0.00E+00	1.00E+00	0.00E+00	1.00E+00	-5.13E-15	1.00E+00	0.00E+00	1.00E+00	0.00E+00	1.00E+00

ENSBTAG00000001861	6.43E-01	2.18E-01	9.84E-01	1.38E-03	1.33E+00	7.83E-06	9.45E-01	2.60E-03	2.07E-01	1.00E+00
ENSBTAG00000027875	2.03E-01	1.00E+00	9.77E-01	3.42E-03	9.03E-01	6.98E-03	2.96E-02	1.00E+00	-5.39E-01	5.92E-01
ENSBTAG00000042279	1.92E+00	8.23E-01	0.00E+00	1.00E+00	-5.13E-15	1.00E+00	0.00E+00	1.00E+00	0.00E+00	1.00E+00
ENSBTAG00000026986	-3.51E-02	1.00E+00	1.33E+00	2.31E-01	-1.04E+00	4.09E-01	7.13E-01	6.55E-01	8.69E-01	8.53E-01
ENSBTAG00000001098	6.32E-01	1.37E-01	1.80E+00	1.99E-12	1.11E+00	2.35E-05	9.11E-01	8.47E-04	2.83E-01	8.06E-01
ENSBTAG00000001097	7.34E-01	4.50E-01	1.63E+00	2.08E-04	1.12E+00	1.51E-02	8.81E-01	7.48E-02	8.35E-01	3.88E-01
ENSBTAG00000033254	3.04E-01	1.00E+00	1.90E+00	1.73E-02	-1.40E+00	2.12E-01	-1.23E+00	2.93E-01	-1.80E+00	4.34E-01
ENSBTAG00000001096	-5.45E-01	7.52E-01	-7.82E-01	1.28E-01	-1.31E+00	5.18E-03	-4.54E-01	4.62E-01	-5.67E-01	7.23E-01
ENSBTAG00000014405	-1.09E-01	1.00E+00	1.23E+00	2.09E-08	8.25E-01	2.56E-04	-4.03E-01	1.35E-01	-2.97E-01	7.21E-01
ENSBTAG00000042858	0.00E+00	1.00E+00	0.00E+00	1.00E+00	-5.13E-15	1.00E+00	0.00E+00	1.00E+00	0.00E+00	1.00E+00
ENSBTAG00000044684	3.90E+00	1.40E-01	3.30E+00	1.02E-01	3.05E+00	1.36E-01	0.00E+00	1.00E+00	0.00E+00	1.00E+00
ENSBTAG00000006168	4.09E-02	1.00E+00	-9.26E-02	1.00E+00	8.77E-01	1.73E-04	1.01E+00	1.37E-05	-1.79E-01	9.95E-01
ENSBTAG00000039522	1.09E+00	7.46E-01	3.55E-01	1.00E+00	2.87E-01	1.00E+00	6.07E-01	7.59E-01	-1.96E+00	6.03E-01
ENSBTAG00000016396	2.15E-01	1.00E+00	-3.57E-02	1.00E+00	1.82E-01	1.00E+00	3.19E-01	8.35E-01	9.53E-02	1.00E+00
ENSBTAG00000027694	-5.49E-01	2.30E-01	-1.09E+00	5.45E-05	-5.19E-01	7.41E-02	-1.32E-01	9.00E-01	1.14E-02	1.00E+00
ENSBTAG00000027696	-9.33E-01	1.40E-01	-3.62E+00	9.16E-12	-9.43E-01	2.49E-02	3.12E-01	6.03E-01	3.23E-01	9.09E-01
ENSBTAG00000009897	2.08E-01	9.46E-01	1.25E-01	8.88E-01	6.66E-01	6.27E-03	9.37E-01	7.24E-05	-8.08E-02	1.00E+00
ENSBTAG00000019255	2.00E-02	1.00E+00	9.30E-01	4.13E-04	-1.57E-02	1.00E+00	-5.54E-01	6.00E-02	-6.08E-02	1.00E+00
ENSBTAG00000021580	1.40E-01	1.00E+00	7.60E-01	4.23E-04	6.04E-01	6.54E-03	6.43E-01	4.09E-03	-1.14E-02	1.00E+00
ENSBTAG00000045536	0.00E+00	1.00E+00	3.68E+00	5.09E-02	1.78E+00	4.79E-01	0.00E+00	1.00E+00	0.00E+00	1.00E+00
ENSBTAG00000043318	0.00E+00	1.00E+00	0.00E+00	1.00E+00	-5.13E-15	1.00E+00	0.00E+00	1.00E+00	0.00E+00	1.00E+00
ENSBTAG00000006693	-1.22E-01	1.00E+00	1.78E-01	5.85E-01	4.53E-02	1.00E+00	-6.57E-02	1.00E+00	-2.54E-01	7.68E-01
ENSBTAG00000015840	0.00E+00	1.00E+00	2.79E+00	2.10E-01	2.55E+00	2.54E-01	0.00E+00	1.00E+00	1.91E+00	8.00E-01
ENSBTAG00000004835	6.71E-01	1.00E+00	-1.24E+00	6.09E-01	-1.44E+00	4.79E-01	-1.46E+00	4.92E-01	-5.12E-01	1.00E+00
ENSBTAG00000039581	9.70E-03	1.00E+00	8.93E-02	1.00E+00	-6.57E-01	8.95E-01	-3.21E+00	9.91E-02	-5.06E-01	1.00E+00
ENSBTAG00000029927	0.00E+00	1.00E+00	0.00E+00	1.00E+00	-5.13E-15	1.00E+00	0.00E+00	1.00E+00	1.91E+00	8.00E-01
ENSBTAG00000016033	-1.29E+00	9.38E-01	-1.22E+00	6.13E-01	9.70E-01	5.26E-01	-1.71E-01	1.00E+00	-3.21E+00	4.34E-01
ENSBTAG00000016030	0.00E+00	1.00E+00	1.98E+00	4.43E-01	-5.13E-15	1.00E+00	0.00E+00	1.00E+00	0.00E+00	1.00E+00
ENSBTAG00000033330	-1.95E+00	1.70E-01	-3.56E+00	1.61E-03	-1.04E+00	2.72E-01	-3.78E+00	5.44E-04	-3.63E+00	1.93E-02
ENSBTAG00000004314	-3.21E+00	3.75E-01	-1.22E+00	6.14E-01	-3.21E+00	9.50E-02	-1.44E+00	4.97E-01	-3.21E+00	4.34E-01
ENSBTAG00000004313	1.33E-02	1.00E+00	-1.91E+00	4.69E-01	-1.24E-01	1.00E+00	-1.91E+00	4.39E-01	7.16E-03	1.00E+00
ENSBTAG00000002009	1.33E-01	1.00E+00	-4.09E-02	1.00E+00	-6.29E-01	9.79E-01	-5.02E-01	1.00E+00	-1.05E+00	1.00E+00
ENSBTAG00000018061	2.27E-01	8.85E-01	1.74E-01	6.98E-01	5.79E-01	1.85E-02	2.08E-01	5.81E-01	-1.31E-01	1.00E+00
ENSBTAG00000031711	-5.02E-01	7.80E-01	-2.42E+00	6.34E-08	5.04E-02	1.00E+00	1.10E+00	1.06E-02	-6.19E-01	6.01E-01
ENSBTAG00000043840	0.00E+00	1.00E+00	0.00E+00	1.00E+00	-5.13E-15	1.00E+00	0.00E+00	1.00E+00	0.00E+00	1.00E+00

ENSBTAG00000002944	-4.52E-02	1.00E+00	-2.47E-01	6.40E-01	-1.55E-02	1.00E+00	3.79E-01	3.63E-01	-3.37E-01	8.00E-01
ENSBTAG00000002295	1.74E-01	9.93E-01	1.19E+00	5.23E-09	8.17E-01	9.62E-05	6.45E-01	3.40E-03	-5.46E-02	1.00E+00
ENSBTAG00000045843	0.00E+00	1.00E+00	0.00E+00	1.00E+00	-5.13E-15	1.00E+00	0.00E+00	1.00E+00	0.00E+00	1.00E+00
ENSBTAG00000008028	-7.30E-02	1.00E+00	-1.93E+00	8.09E-07	-8.11E-01	3.79E-02	-7.35E-01	6.98E-02	-6.38E-01	4.55E-01
ENSBTAG00000018253	3.45E+00	1.70E-01	4.53E+00	3.70E-03	2.48E+00	1.92E-01	4.34E+00	6.43E-03	3.46E+00	2.41E-01
ENSBTAG00000001585	-2.51E-01	8.28E-01	-3.68E-01	2.12E-01	-2.37E-01	4.95E-01	-1.91E-01	6.57E-01	-6.97E-01	6.37E-02
ENSBTAG00000043001	0.00E+00	1.00E+00	0.00E+00	1.00E+00	-5.13E-15	1.00E+00	0.00E+00	1.00E+00	0.00E+00	1.00E+00
ENSBTAG00000021636	3.29E-01	7.11E-01	-8.62E-01	9.41E-04	-9.99E-01	8.75E-05	-6.44E-01	1.88E-02	-1.40E-01	1.00E+00
ENSBTAG00000012966	2.22E-01	1.00E+00	-6.78E-02	1.00E+00	-2.28E-01	7.95E-01	1.32E-01	1.00E+00	1.74E-01	1.00E+00
ENSBTAG00000038620	9.86E-03	1.00E+00	-2.02E-01	4.69E-01	-6.61E-02	1.00E+00	-1.53E-01	6.67E-01	-6.18E-02	1.00E+00
ENSBTAG00000014949	-7.99E-01	1.00E+00	-7.29E-01	9.45E-01	-2.71E+00	1.99E-01	-1.85E-01	1.00E+00	-2.71E+00	6.01E-01
ENSBTAG00000043499	0.00E+00	1.00E+00	0.00E+00	1.00E+00	-5.13E-15	1.00E+00	0.00E+00	1.00E+00	0.00E+00	1.00E+00
ENSBTAG00000006970	3.70E-02	1.00E+00	7.60E-01	5.84E-03	9.22E-01	5.70E-04	6.64E-01	2.10E-02	-6.92E-01	1.29E-01
ENSBTAG00000000176	-1.23E-02	1.00E+00	-6.91E-02	1.00E+00	6.32E-02	1.00E+00	2.44E-01	4.66E-01	-1.30E-01	1.00E+00
ENSBTAG00000040494	-1.34E-01	1.00E+00	-3.59E-01	3.30E-01	-4.08E-01	2.40E-01	-3.47E-01	3.66E-01	-2.19E-01	9.81E-01
ENSBTAG00000003458	-2.37E-01	1.00E+00	-1.03E+00	1.81E-03	-7.76E-01	1.89E-02	-1.10E+00	7.07E-04	-6.12E-01	3.72E-01
ENSBTAG00000001083	1.46E-03	1.00E+00	3.21E-02	1.00E+00	2.33E-01	8.27E-01	3.91E-01	5.09E-01	-3.14E-01	9.74E-01
ENSBTAG00000043175	0.00E+00	1.00E+00	0.00E+00	1.00E+00	-5.13E-15	1.00E+00	0.00E+00	1.00E+00	0.00E+00	1.00E+00
ENSBTAG00000020984	-1.64E-01	1.00E+00	-2.31E+00	9.50E-12	-1.66E+00	1.63E-07	-2.44E-01	6.50E-01	-6.56E-01	3.07E-01
ENSBTAG00000040105	0.00E+00	1.00E+00	0.00E+00	1.00E+00	-5.13E-15	1.00E+00	0.00E+00	1.00E+00	0.00E+00	1.00E+00
ENSBTAG00000043489	0.00E+00	1.00E+00	0.00E+00	1.00E+00	-5.13E-15	1.00E+00	0.00E+00	1.00E+00	0.00E+00	1.00E+00
ENSBTAG00000016836	-6.47E-01	1.34E-01	-1.28E+00	2.51E-06	-1.62E+00	1.70E-09	-1.54E+00	1.66E-08	-2.45E-01	9.01E-01
ENSBTAG00000042826	0.00E+00	1.00E+00	0.00E+00	1.00E+00	-5.13E-15	1.00E+00	0.00E+00	1.00E+00	0.00E+00	1.00E+00
ENSBTAG00000017266	2.93E-01	1.00E+00	-5.92E-01	1.91E-01	-4.30E-01	4.09E-01	-1.71E-01	9.51E-01	-3.67E-01	8.44E-01
ENSBTAG00000045375	0.00E+00	1.00E+00	0.00E+00	1.00E+00	-5.13E-15	1.00E+00	0.00E+00	1.00E+00	0.00E+00	1.00E+00
ENSBTAG00000005741	2.33E+00	6.88E-02	9.74E-02	1.00E+00	2.11E+00	2.82E-02	1.57E+00	1.76E-01	3.25E-02	1.00E+00
ENSBTAG00000015217	0.00E+00	1.00E+00	2.79E+00	2.10E-01	1.78E+00	4.79E-01	2.53E+00	2.71E-01	0.00E+00	1.00E+00
ENSBTAG00000019075	1.87E-01	9.74E-01	-5.21E-01	3.25E-02	-5.76E-01	1.52E-02	-5.20E-01	3.56E-02	-1.62E-01	1.00E+00
ENSBTAG00000010118	7.47E-02	1.00E+00	5.32E-01	2.24E-02	4.12E-01	9.61E-02	4.71E-01	5.38E-02	-3.75E-01	4.96E-01
ENSBTAG00000016762	-3.20E-01	9.06E-01	-1.93E+00	9.35E-08	-1.27E+00	3.20E-04	-3.69E-01	4.52E-01	-7.31E-01	3.02E-01
ENSBTAG00000002646	-4.20E-02	1.00E+00	-3.68E-01	1.12E-01	-3.13E-01	1.99E-01	-9.20E-02	9.53E-01	-7.66E-02	1.00E+00
ENSBTAG00000007898	-2.90E-01	1.00E+00	1.01E-03	1.00E+00	-1.27E-01	1.00E+00	-1.64E-01	9.99E-01	-4.56E-01	8.00E-01
ENSBTAG00000007683	-4.88E-01	3.68E-01	4.07E-01	1.89E-01	9.30E-01	2.19E-04	7.42E-01	5.13E-03	-4.22E-01	5.62E-01
ENSBTAG00000016430	1.95E-01	1.00E+00	-1.20E-01	1.00E+00	3.26E-01	4.30E-01	5.41E-02	1.00E+00	-1.45E-01	1.00E+00
ENSBTAG00000006918	1.26E-01	1.00E+00	-6.68E-01	1.23E-02	-9.58E-01	1.58E-04	-5.48E-01	5.36E-02	3.41E-01	6.87E-01

ENSBTAG00000013218	4.00E-01	2.17E-01	1.23E+00	8.36E-12	7.05E-01	1.59E-04	4.93E-01	1.40E-02	4.14E-02	1.00E+00
ENSBTAG00000036258	0.00E+00	1.00E+00	0.00E+00	1.00E+00	-5.13E-15	1.00E+00	0.00E+00	1.00E+00	0.00E+00	1.00E+00
ENSBTAG00000007258	-2.97E-01	1.00E+00	2.50E+00	4.89E-04	1.80E+00	2.55E-02	2.58E-01	1.00E+00	-1.98E+00	6.01E-01
ENSBTAG00000032846	3.98E-01	8.30E-01	-3.64E-01	5.42E-01	-1.96E+00	2.05E-06	-2.36E+00	2.37E-08	5.85E-01	6.01E-01
ENSBTAG00000003207	2.07E+00	1.88E-12	9.82E-01	8.47E-04	-2.15E+00	2.21E-12	-3.66E-01	3.66E-01	3.57E-01	7.79E-01
ENSBTAG00000045962	0.00E+00	1.00E+00	0.00E+00	1.00E+00	-5.13E-15	1.00E+00	0.00E+00	1.00E+00	0.00E+00	1.00E+00
ENSBTAG00000003626	1.03E-01	1.00E+00	-5.58E-01	5.48E-01	-8.82E-01	2.41E-01	-4.44E-01	6.88E-01	-7.49E-01	7.79E-01
ENSBTAG00000043675	0.00E+00	1.00E+00	0.00E+00	1.00E+00	2.55E+00	2.55E-01	0.00E+00	1.00E+00	1.91E+00	8.00E-01
ENSBTAG00000020296	8.72E-02	1.00E+00	-2.02E-01	6.17E-01	-3.07E-01	3.32E-01	-7.68E-02	1.00E+00	-5.33E-02	1.00E+00
ENSBTAG00000026008	2.95E-02	1.00E+00	3.60E-02	1.00E+00	-5.29E-02	1.00E+00	-4.36E-01	5.19E-02	-8.41E-02	1.00E+00
ENSBTAG00000008716	1.97E-01	8.78E-01	1.15E+00	3.20E-09	1.59E+00	9.83E-17	1.31E+00	1.22E-11	-3.31E-01	5.32E-01
ENSBTAG00000042734	0.00E+00	1.00E+00	0.00E+00	1.00E+00	-5.13E-15	1.00E+00	0.00E+00	1.00E+00	0.00E+00	1.00E+00
ENSBTAG00000001093	3.43E-01	8.53E-01	-9.62E-01	1.32E-02	4.07E-02	1.00E+00	-4.02E-01	4.19E-01	1.16E+00	9.54E-03
ENSBTAG00000001092	-3.09E-01	7.49E-01	-5.81E-01	2.76E-02	-7.55E-01	2.61E-03	-8.24E-01	1.10E-03	-2.41E-01	8.40E-01
ENSBTAG00000001090	6.57E-02	1.00E+00	-1.43E+00	5.55E-04	-1.41E+00	4.01E-04	-7.98E-01	5.97E-02	4.82E-01	6.71E-01
ENSBTAG00000027789	1.42E-01	1.00E+00	-8.34E-02	9.88E-01	3.36E-01	1.53E-01	2.58E-01	3.35E-01	-7.39E-02	1.00E+00
ENSBTAG00000010743	6.95E-02	1.00E+00	5.06E-01	3.85E-02	3.22E-01	2.52E-01	3.10E-01	2.88E-01	-1.10E-01	1.00E+00
ENSBTAG00000046878	0.00E+00	1.00E+00	0.00E+00	1.00E+00	-5.13E-15	1.00E+00	0.00E+00	1.00E+00	0.00E+00	1.00E+00
ENSBTAG00000044248	0.00E+00	1.00E+00	0.00E+00	1.00E+00	-5.13E-15	1.00E+00	0.00E+00	1.00E+00	0.00E+00	1.00E+00
ENSBTAG00000010741	1.10E+00	3.82E-01	1.16E+00	9.73E-02	3.51E-01	8.46E-01	9.24E-01	2.29E-01	6.25E-01	8.28E-01
ENSBTAG00000010736	-2.67E-01	8.74E-01	-8.55E-01	2.98E-03	4.16E-01	1.86E-01	6.26E-01	2.75E-02	-3.83E-01	6.47E-01
ENSBTAG00000004555	-5.34E-01	1.00E+00	-1.45E+00	1.87E-01	-2.60E+00	1.02E-02	-1.37E+00	2.15E-01	3.06E-01	1.00E+00
ENSBTAG00000004557	1.07E+00	6.02E-01	-9.07E-02	1.00E+00	-1.57E-01	1.00E+00	9.21E-01	3.57E-01	1.62E+00	2.06E-01
ENSBTAG00000026885	-1.40E-01	1.00E+00	-1.75E+00	7.86E-08	-3.78E+00	1.74E-28	-3.68E+00	4.22E-27	7.53E-01	2.18E-01
ENSBTAG00000004241	1.92E+00	8.23E-01	0.00E+00	1.00E+00	-5.13E-15	1.00E+00	1.76E+00	4.97E-01	0.00E+00	1.00E+00
ENSBTAG00000010048	-6.21E-01	7.91E-01	-1.07E+00	8.65E-02	-9.09E-01	1.37E-01	4.15E-01	5.90E-01	-2.20E-01	1.00E+00
ENSBTAG00000042879	0.00E+00	1.00E+00	0.00E+00	1.00E+00	-5.13E-15	1.00E+00	0.00E+00	1.00E+00	0.00E+00	1.00E+00
ENSBTAG00000010362	2.65E-01	8.87E-01	-7.36E-01	1.20E-02	-1.17E+00	2.51E-05	-5.00E-01	1.24E-01	2.78E-01	8.28E-01
ENSBTAG00000044179	-2.39E-02	1.00E+00	6.13E-01	6.85E-02	1.76E-01	8.29E-01	-5.17E-01	1.55E-01	-3.58E-01	7.99E-01
ENSBTAG00000043862	0.00E+00	1.00E+00	0.00E+00	1.00E+00	-5.13E-15	1.00E+00	0.00E+00	1.00E+00	0.00E+00	1.00E+00
ENSBTAG00000044462	0.00E+00	1.00E+00	0.00E+00	1.00E+00	-5.13E-15	1.00E+00	0.00E+00	1.00E+00	0.00E+00	1.00E+00
ENSBTAG00000017162	9.65E-01	3.88E-01	2.52E+00	2.21E-06	2.11E+00	7.93E-05	1.02E+00	9.96E-02	9.86E-01	4.34E-01
ENSBTAG00000021954	5.21E-03	1.00E+00	-1.91E+00	4.69E-01	-1.31E-01	1.00E+00	-1.91E+00	4.39E-01	1.30E+00	9.00E-01
ENSBTAG00000046605	0.00E+00	1.00E+00	0.00E+00	1.00E+00	-5.13E-15	1.00E+00	0.00E+00	1.00E+00	0.00E+00	1.00E+00
ENSBTAG00000003164	3.81E-01	6.98E-01	1.31E+00	2.98E-06	1.27E+00	5.15E-06	1.05E+00	2.72E-04	1.46E+00	6.81E-06

ENSBTAG00000017743	1.94E+00	5.65E-01	1.85E+00	2.32E-01	-1.22E+00	5.10E-01	1.25E+00	4.97E-01	1.06E+00	9.25E-01
ENSBTAG00000046347	0.00E+00	1.00E+00	0.00E+00	1.00E+00	-5.13E-15	1.00E+00	0.00E+00	1.00E+00	0.00E+00	1.00E+00
ENSBTAG00000019137	1.54E-02	1.00E+00	-1.66E-01	1.00E+00	3.80E-01	8.30E-01	-4.55E-01	8.04E-01	1.28E-02	1.00E+00
ENSBTAG00000026882	1.99E-02	1.00E+00	-6.56E-01	1.61E-02	-9.10E-01	4.31E-04	-6.67E-01	1.47E-02	-2.27E-01	9.01E-01
ENSBTAG00000002425	-5.12E-01	1.00E+00	4.57E-03	1.00E+00	-8.24E-01	3.90E-01	-1.17E+00	1.88E-01	-1.36E+00	4.65E-01
ENSBTAG00000042597	0.00E+00	1.00E+00	0.00E+00	1.00E+00	-5.13E-15	1.00E+00	1.76E+00	4.97E-01	0.00E+00	1.00E+00
ENSBTAG00000018520	9.53E-01	8.60E-01	1.04E+00	4.43E-01	1.09E+00	3.77E-01	1.71E+00	7.31E-02	5.46E-01	1.00E+00
ENSBTAG00000016512	2.40E-02	1.00E+00	-1.81E+00	8.06E-11	-8.34E-01	3.34E-03	8.34E-02	1.00E+00	2.91E-01	8.00E-01
ENSBTAG00000013957	-1.07E-01	1.00E+00	-6.20E-01	3.09E-01	-4.64E-01	4.79E-01	-8.04E-01	1.48E-01	-8.76E-01	4.34E-01
ENSBTAG00000039091	-9.66E-01	2.37E-01	-1.87E+00	2.08E-04	-1.66E+00	6.72E-04	-1.69E+00	6.23E-04	-3.82E-01	9.37E-01
ENSBTAG00000042315	0.00E+00	1.00E+00	0.00E+00	1.00E+00	-5.13E-15	1.00E+00	0.00E+00	1.00E+00	0.00E+00	1.00E+00
ENSBTAG00000038180	9.61E-01	8.02E-01	7.99E-01	5.25E-01	-1.70E-01	1.00E+00	-2.02E-01	1.00E+00	-8.99E-01	9.31E-01
ENSBTAG00000019385	1.83E-01	1.00E+00	1.81E+00	1.82E-02	8.90E-01	4.09E-01	1.27E+00	1.54E-01	3.59E-01	1.00E+00
ENSBTAG00000042818	0.00E+00	1.00E+00	0.00E+00	1.00E+00	-5.13E-15	1.00E+00	0.00E+00	1.00E+00	0.00E+00	1.00E+00
ENSBTAG00000007650	5.87E-01	1.00E+00	-1.33E+00	4.22E-01	-2.34E+00	1.11E-01	1.15E+00	2.71E-01	2.41E-01	1.00E+00
ENSBTAG00000046685	-1.91E+00	8.23E-01	-1.91E+00	4.69E-01	-1.91E+00	4.28E-01	-1.91E+00	4.39E-01	8.00E-01	1.00E+00
ENSBTAG00000005501	3.45E-01	8.23E-01	-1.94E-01	7.86E-01	-1.40E+00	3.11E-06	-6.02E-01	8.22E-02	3.68E-01	7.77E-01
ENSBTAG00000047880	3.82E-01	4.76E-01	-3.02E-01	3.18E-01	-1.49E+00	4.64E-11	-1.10E+00	1.90E-06	-3.54E-02	1.00E+00
ENSBTAG00000019291	8.17E-01	1.93E-02	1.63E-01	8.18E-01	-1.48E+00	1.92E-08	-9.67E-01	4.17E-04	1.90E-01	1.00E+00
ENSBTAG00000008704	1.12E+00	1.51E-02	-1.37E+00	1.15E-03	-1.06E+00	1.01E-02	-3.03E-01	6.51E-01	8.84E-01	1.69E-01
ENSBTAG00000045366	0.00E+00	1.00E+00	0.00E+00	1.00E+00	-5.13E-15	1.00E+00	0.00E+00	1.00E+00	0.00E+00	1.00E+00
ENSBTAG00000047523	0.00E+00	1.00E+00	0.00E+00	1.00E+00	-5.13E-15	1.00E+00	0.00E+00	1.00E+00	0.00E+00	1.00E+00
ENSBTAG00000001899	1.55E+00	3.02E-01	6.60E-01	6.99E-01	5.45E-01	8.00E-01	2.12E+00	6.38E-03	9.48E-01	8.00E-01
ENSBTAG00000046572	0.00E+00	1.00E+00	0.00E+00	1.00E+00	-5.13E-15	1.00E+00	0.00E+00	1.00E+00	0.00E+00	1.00E+00
ENSBTAG00000018446	4.88E-01	8.23E-01	8.63E-01	9.27E-02	8.07E-01	1.16E-01	1.09E+00	2.20E-02	4.09E-01	9.13E-01
ENSBTAG00000043713	0.00E+00	1.00E+00	0.00E+00	1.00E+00	-5.13E-15	1.00E+00	0.00E+00	1.00E+00	0.00E+00	1.00E+00
ENSBTAG00000008142	3.53E-01	1.00E+00	2.79E+00	1.31E-06	2.20E+00	1.24E-04	4.52E-01	6.43E-01	-4.17E-01	1.00E+00
ENSBTAG00000008140	1.39E-01	1.00E+00	3.49E+00	1.56E-11	3.74E+00	1.12E-13	1.46E+00	3.42E-02	4.73E-01	1.00E+00
ENSBTAG00000000730	-2.71E+00	5.76E-01	-7.23E-01	9.45E-01	-9.24E-01	8.08E-01	-1.77E-01	1.00E+00	-2.71E+00	6.01E-01
ENSBTAG00000015330	5.83E-02	1.00E+00	-1.06E+00	3.97E-06	-1.93E+00	1.54E-17	-1.88E+00	1.38E-16	3.57E-01	5.99E-01
ENSBTAG00000048246	0.00E+00	1.00E+00	0.00E+00	1.00E+00	-5.13E-15	1.00E+00	0.00E+00	1.00E+00	0.00E+00	1.00E+00
ENSBTAG00000015317	-1.66E-01	1.00E+00	-1.24E+00	6.28E-06	-2.43E+00	1.70E-18	-2.78E+00	5.19E-23	-4.56E-01	5.16E-01
ENSBTAG00000028572	0.00E+00	1.00E+00	0.00E+00	1.00E+00	-5.13E-15	1.00E+00	0.00E+00	1.00E+00	0.00E+00	1.00E+00
ENSBTAG00000006294	0.00E+00	1.00E+00	0.00E+00	1.00E+00	-5.13E-15	1.00E+00	0.00E+00	1.00E+00	0.00E+00	1.00E+00
ENSBTAG00000021692	1.92E+00	8.23E-01	5.18E+00	3.06E-04	3.06E+00	1.36E-01	3.94E+00	2.67E-02	0.00E+00	1.00E+00

ENSBTAG00000021691	6.60E-02	1.00E+00	9.64E-01	5.31E-06	8.75E-01	3.85E-05	6.64E-01	3.04E-03	-4.52E-01	3.09E-01
ENSBTAG00000045139	0.00E+00	1.00E+00	0.00E+00	1.00E+00	-5.13E-15	1.00E+00	0.00E+00	1.00E+00	0.00E+00	1.00E+00
ENSBTAG00000006654	4.58E-01	1.13E-01	1.40E+00	4.56E-15	7.99E-01	1.45E-05	3.75E-01	8.18E-02	-4.89E-01	1.35E-01
ENSBTAG00000005180	4.58E-01	2.07E-01	2.44E+00	7.60E-33	2.39E+00	6.67E-32	1.68E+00	1.31E-16	-9.78E-02	1.00E+00
ENSBTAG00000009080	-1.35E-01	1.00E+00	-1.83E-01	6.05E-01	-2.78E-01	3.16E-01	-1.31E-02	1.00E+00	-2.36E-01	8.00E-01
ENSBTAG00000032515	5.87E-01	1.81E-01	2.06E-02	1.00E+00	-2.06E-01	6.75E-01	-7.14E-02	1.00E+00	-6.39E-01	2.32E-01
ENSBTAG00000042933	0.00E+00	1.00E+00	0.00E+00	1.00E+00	-5.13E-15	1.00E+00	0.00E+00	1.00E+00	0.00E+00	1.00E+00
ENSBTAG00000017797	6.08E-05	1.00E+00	-1.16E+00	2.88E-04	-6.91E-02	1.00E+00	-2.99E-01	5.09E-01	-6.81E-01	2.64E-01
ENSBTAG00000038195	-8.38E-03	1.00E+00	-4.88E-01	2.94E-01	-1.08E+00	4.10E-03	-1.25E+00	9.24E-04	-4.33E-01	7.92E-01
ENSBTAG00000008755	-3.55E-02	1.00E+00	-5.76E-02	1.00E+00	-1.42E-01	7.70E-01	-4.18E-01	9.78E-02	-4.52E-01	3.11E-01
ENSBTAG00000020654	2.51E-01	1.00E+00	-4.06E-01	4.69E-01	-1.27E+00	1.60E-03	-1.18E+00	4.48E-03	1.76E-01	1.00E+00
ENSBTAG00000004184	-3.11E-01	7.58E-01	-1.42E+00	1.14E-08	-2.21E+00	1.20E-18	-1.70E+00	7.56E-12	-4.91E-01	3.64E-01
ENSBTAG00000004177	-4.14E-01	3.87E-01	-1.74E+00	2.21E-14	-2.03E+00	4.89E-19	-1.44E+00	2.44E-10	1.07E-01	1.00E+00
ENSBTAG00000032481	4.86E-03	1.00E+00	8.74E-01	8.54E-01	-1.31E-01	1.00E+00	2.41E+00	8.58E-02	1.97E+00	6.01E-01
ENSBTAG00000013773	-2.06E-01	8.33E-01	-9.65E-01	1.00E-06	-1.39E+00	8.86E-13	-9.76E-01	7.85E-07	-2.12E-01	8.00E-01
ENSBTAG00000004267	1.79E-01	1.00E+00	-4.52E-01	7.94E-01	-1.00E+00	2.81E-01	-6.35E-01	5.68E-01	-6.77E-01	8.82E-01
ENSBTAG00000036754	0.00E+00	1.00E+00	0.00E+00	1.00E+00	-5.13E-15	1.00E+00	0.00E+00	1.00E+00	0.00E+00	1.00E+00
ENSBTAG00000012801	-4.36E-01	5.76E-01	-2.70E+00	3.53E-21	-4.74E+00	3.02E-52	-3.10E+00	9.46E-27	4.57E-01	5.55E-01
ENSBTAG00000043308	5.30E-01	1.00E+00	-2.70E+00	2.42E-01	-2.70E+00	1.99E-01	-1.70E-01	1.00E+00	-2.70E+00	6.01E-01
ENSBTAG00000019337	-1.31E-01	1.00E+00	-1.43E+00	4.01E-02	-3.78E-01	7.77E-01	5.29E-01	5.41E-01	2.03E-01	1.00E+00
ENSBTAG00000008329	-4.65E-01	5.76E-01	6.32E-02	1.00E+00	1.50E-01	8.99E-01	1.24E-02	1.00E+00	-5.26E-01	4.80E-01
ENSBTAG00000043593	0.00E+00	1.00E+00	0.00E+00	1.00E+00	-5.13E-15	1.00E+00	0.00E+00	1.00E+00	0.00E+00	1.00E+00
ENSBTAG00000012912	0.00E+00	1.00E+00	1.98E+00	4.43E-01	-5.13E-15	1.00E+00	2.53E+00	2.71E-01	0.00E+00	1.00E+00
ENSBTAG00000017376	3.07E-01	1.00E+00	-3.58E+00	6.70E-02	-5.27E-01	9.41E-01	-1.82E+00	3.02E-01	5.76E-01	1.00E+00
ENSBTAG00000011909	3.84E-01	6.62E-01	-1.42E+00	3.81E-07	-5.24E-01	1.02E-01	-4.15E-01	2.41E-01	3.77E-01	6.92E-01
ENSBTAG00000032405	9.17E-01	6.61E-01	-6.00E-01	7.18E-01	8.23E-01	3.50E-01	1.78E+00	4.11E-03	-1.38E-01	1.00E+00
ENSBTAG00000009770	3.09E-01	9.94E-01	-6.01E-01	2.28E-01	4.15E-01	4.28E-01	1.01E+00	8.89E-03	-2.01E-02	1.00E+00
ENSBTAG00000003650	-5.18E-01	7.67E-01	-4.01E-01	5.20E-01	-1.34E+00	2.35E-03	-6.12E-01	2.48E-01	-3.01E-01	1.00E+00
ENSBTAG00000043505	0.00E+00	1.00E+00	0.00E+00	1.00E+00	-5.13E-15	1.00E+00	0.00E+00	1.00E+00	0.00E+00	1.00E+00
ENSBTAG00000028119	0.00E+00	1.00E+00	0.00E+00	1.00E+00	-5.13E-15	1.00E+00	0.00E+00	1.00E+00	0.00E+00	1.00E+00
ENSBTAG00000006159	-2.19E-01	1.00E+00	-3.40E+00	3.14E-21	-2.97E+00	1.88E-18	-1.98E+00	8.01E-10	-1.43E-01	1.00E+00
ENSBTAG00000005562	-3.18E-01	1.00E+00	-2.54E-02	1.00E+00	-5.78E-01	3.15E-01	2.74E-01	7.79E-01	-3.85E-01	9.23E-01
ENSBTAG00000021526	-1.62E+00	3.61E-01	-3.24E+00	5.64E-03	-8.78E-01	4.12E-01	-1.27E+00	2.05E-01	-6.69E-01	9.17E-01
ENSBTAG00000012816	1.08E-01	1.00E+00	6.70E-01	9.47E-04	8.79E-01	7.70E-06	8.53E-01	1.92E-05	-1.03E-02	1.00E+00
ENSBTAG00000033143	-1.86E-01	1.00E+00	2.57E-01	8.51E-01	2.57E-01	8.37E-01	2.84E-01	7.95E-01	-4.44E-01	8.44E-01

ENSBTAG00000001435	2.99E-01	7.41E-01	6.02E-01	1.45E-02	2.06E-01	5.77E-01	-3.27E-01	2.78E-01	-1.04E-01	1.00E+00
ENSBTAG00000047365	0.00E+00	1.00E+00	0.00E+00	1.00E+00	-5.13E-15	1.00E+00	0.00E+00	1.00E+00	0.00E+00	1.00E+00
ENSBTAG00000042278	0.00E+00	1.00E+00	0.00E+00	1.00E+00	-5.13E-15	1.00E+00	0.00E+00	1.00E+00	0.00E+00	1.00E+00
ENSBTAG00000048151	8.68E-03	1.00E+00	8.02E-01	4.54E-04	1.06E+00	1.75E-06	1.15E+00	2.75E-07	-1.66E-01	1.00E+00
ENSBTAG00000026851	-1.46E-01	1.00E+00	-5.92E-01	1.09E-01	-7.90E-01	2.14E-02	-6.66E-01	6.73E-02	-3.38E-01	8.00E-01
ENSBTAG00000045119	0.00E+00	1.00E+00	0.00E+00	1.00E+00	-5.13E-15	1.00E+00	0.00E+00	1.00E+00	0.00E+00	1.00E+00
ENSBTAG00000034503	0.00E+00	1.00E+00	0.00E+00	1.00E+00	-5.13E-15	1.00E+00	0.00E+00	1.00E+00	0.00E+00	1.00E+00
ENSBTAG00000044916	0.00E+00	1.00E+00	0.00E+00	1.00E+00	-5.13E-15	1.00E+00	1.76E+00	4.97E-01	0.00E+00	1.00E+00
ENSBTAG00000048306	-7.51E-02	1.00E+00	6.13E-01	3.25E-01	1.45E+00	1.91E-03	1.88E+00	3.81E-05	5.53E-01	8.00E-01
ENSBTAG00000001887	2.29E-01	9.66E-01	4.51E-01	1.56E-01	1.01E+00	1.26E-04	1.13E+00	2.04E-05	2.05E-01	9.96E-01
ENSBTAG00000002297	-2.15E-01	1.00E+00	-1.76E-01	1.00E+00	-3.93E-01	7.16E-01	-1.81E-01	1.00E+00	-1.25E-01	1.00E+00
ENSBTAG00000002293	3.31E-02	1.00E+00	1.41E-01	8.53E-01	1.41E-01	8.45E-01	3.58E-01	2.56E-01	4.17E-01	5.03E-01
ENSBTAG00000006907	3.79E-01	1.00E+00	1.95E+00	8.25E-03	1.57E-01	1.00E+00	1.00E+00	2.60E-01	1.01E+00	6.23E-01
ENSBTAG00000021020	4.01E-01	5.79E-01	1.29E+00	1.21E-06	1.54E+00	3.93E-09	1.57E+00	2.72E-09	2.58E-02	1.00E+00
ENSBTAG00000007239	4.25E+00	6.90E-07	5.91E+00	2.96E-18	4.30E+00	3.19E-08	4.58E+00	1.57E-09	1.97E+00	4.34E-01
ENSBTAG00000032369	7.96E-01	1.96E-01	3.32E+00	4.63E-19	2.41E+00	2.85E-11	1.39E+00	1.98E-04	-2.06E-01	1.00E+00
ENSBTAG00000016217	6.02E-01	6.98E-01	2.02E+00	1.02E-05	1.36E+00	3.73E-03	3.55E-01	6.68E-01	-2.99E-01	1.00E+00
ENSBTAG00000039731	5.54E-01	9.20E-02	-8.29E-01	1.91E-04	-1.10E+00	3.91E-07	-1.24E+00	1.11E-08	-4.39E-01	3.54E-01
ENSBTAG00000045819	9.84E-01	3.07E-02	1.38E+00	4.24E-05	2.23E+00	1.19E-11	2.58E+00	6.92E-15	1.00E+00	5.32E-02
ENSBTAG00000037325	1.91E+00	8.23E-01	0.00E+00	1.00E+00	-5.13E-15	1.00E+00	0.00E+00	1.00E+00	0.00E+00	1.00E+00
ENSBTAG00000015962	1.88E-01	9.17E-01	2.25E-01	4.35E-01	-8.31E-05	1.00E+00	-6.09E-02	1.00E+00	-1.67E-01	9.49E-01
ENSBTAG00000047426	1.72E-01	1.00E+00	-2.14E+00	3.77E-05	-3.32E+00	2.16E-09	-1.65E+00	9.05E-04	4.91E-01	8.00E-01
ENSBTAG00000019683	-7.88E-02	1.00E+00	-2.42E+00	3.23E-03	-3.22E+00	1.20E-04	-3.82E+00	1.64E-05	1.43E+00	2.78E-01
ENSBTAG00000018125	-1.16E+00	2.37E-01	-5.13E-01	5.08E-01	-7.88E-01	2.19E-01	-8.79E-01	1.68E-01	-7.24E-01	6.83E-01
ENSBTAG00000018581	3.83E-01	5.75E-01	1.32E+00	4.45E-08	7.57E-01	2.87E-03	6.84E-01	9.49E-03	2.49E-01	8.38E-01
ENSBTAG00000042622	0.00E+00	1.00E+00	0.00E+00	1.00E+00	-5.13E-15	1.00E+00	0.00E+00	1.00E+00	0.00E+00	1.00E+00
ENSBTAG00000019592	2.40E-01	9.05E-01	-1.07E-02	1.00E+00	-4.60E-01	1.24E-01	-1.55E+00	5.70E-09	3.74E-01	6.03E-01
ENSBTAG00000016081	-5.15E-02	1.00E+00	6.38E-01	8.38E-03	7.43E-01	1.64E-03	7.49E-01	1.81E-03	-2.83E-01	7.79E-01
ENSBTAG00000015291	4.34E-01	1.70E-01	1.05E+00	1.74E-08	6.06E-01	1.99E-03	7.17E-02	1.00E+00	-1.35E-03	1.00E+00
ENSBTAG00000036993	0.00E+00	1.00E+00	0.00E+00	1.00E+00	-5.13E-15	1.00E+00	0.00E+00	1.00E+00	0.00E+00	1.00E+00
ENSBTAG00000018114	-1.29E-01	1.00E+00	3.45E-01	2.37E-01	1.96E-01	6.15E-01	-1.81E-01	6.83E-01	-2.13E-02	1.00E+00
ENSBTAG00000046032	0.00E+00	1.00E+00	0.00E+00	1.00E+00	-5.13E-15	1.00E+00	1.76E+00	4.97E-01	0.00E+00	1.00E+00
ENSBTAG00000012376	-2.86E+00	1.91E-02	4.75E-01	6.52E-01	2.11E-01	1.00E+00	-9.38E-01	2.93E-01	-2.34E+00	9.77E-02
ENSBTAG00000044491	0.00E+00	1.00E+00	0.00E+00	1.00E+00	-5.13E-15	1.00E+00	0.00E+00	1.00E+00	0.00E+00	1.00E+00
ENSBTAG00000012615	2.19E-01	9.17E-01	8.13E-01	5.81E-04	5.60E-01	2.54E-02	5.23E-01	4.48E-02	3.45E-02	1.00E+00

ENSBTAG00000001132	2.19E-02	1.00E+00	-6.58E-02	1.00E+00	2.84E-02	1.00E+00	-9.15E-02	1.00E+00	-5.02E-01	3.36E-01
ENSBTAG00000032289	-5.76E-02	1.00E+00	-7.80E-01	1.46E-02	-6.93E-01	3.13E-02	-3.97E-01	3.06E-01	-3.62E-01	7.79E-01
ENSBTAG00000032277	5.90E-01	6.41E-01	1.42E+00	1.11E-03	1.92E-01	9.52E-01	-7.70E-01	1.24E-01	-9.19E-02	1.00E+00
ENSBTAG00000047778	0.00E+00	1.00E+00	0.00E+00	1.00E+00	-5.13E-15	1.00E+00	0.00E+00	1.00E+00	0.00E+00	1.00E+00
ENSBTAG00000043666	0.00E+00	1.00E+00	0.00E+00	1.00E+00	-5.13E-15	1.00E+00	0.00E+00	1.00E+00	0.00E+00	1.00E+00
ENSBTAG00000047455	-1.18E+00	8.64E-01	-3.89E+00	3.55E-02	-3.89E+00	2.22E-02	-3.89E+00	2.41E-02	-1.18E+00	8.33E-01
ENSBTAG00000048039	0.00E+00	1.00E+00	0.00E+00	1.00E+00	-5.13E-15	1.00E+00	0.00E+00	1.00E+00	0.00E+00	1.00E+00
ENSBTAG00000032264	0.00E+00	1.00E+00	0.00E+00	1.00E+00	-5.13E-15	1.00E+00	0.00E+00	1.00E+00	0.00E+00	1.00E+00
ENSBTAG00000024632	-8.73E-03	1.00E+00	9.95E-01	2.62E-01	-7.44E-02	1.00E+00	1.39E+00	6.80E-02	-9.35E-01	8.00E-01
ENSBTAG00000017629	-3.39E-01	9.43E-01	-1.28E+00	2.54E-03	-1.48E-01	1.00E+00	2.65E-01	7.18E-01	-5.72E-01	6.05E-01
ENSBTAG00000018147	-1.63E+00	4.50E-01	-2.28E-01	1.00E+00	-1.43E+00	1.89E-01	1.48E-01	1.00E+00	-3.22E-01	1.00E+00
ENSBTAG00000045146	0.00E+00	1.00E+00	0.00E+00	1.00E+00	-5.13E-15	1.00E+00	0.00E+00	1.00E+00	0.00E+00	1.00E+00
ENSBTAG00000013455	-2.06E-01	1.00E+00	1.53E-01	9.08E-01	5.05E-01	1.45E-01	7.61E-01	1.61E-02	1.10E-02	1.00E+00
ENSBTAG00000001627	2.11E-01	8.69E-01	3.25E-01	2.08E-01	9.72E-01	1.41E-06	1.13E+00	2.06E-08	2.52E-01	7.97E-01
ENSBTAG00000014432	-4.20E-01	8.50E-01	-2.75E+00	4.52E-08	-8.85E-01	5.65E-02	9.52E-03	1.00E+00	-4.90E-01	8.00E-01
ENSBTAG00000039437	0.00E+00	1.00E+00	1.99E+00	4.43E-01	1.78E+00	4.79E-01	2.53E+00	2.71E-01	0.00E+00	1.00E+00
ENSBTAG00000044055	2.26E-01	1.00E+00	2.70E-01	9.16E-01	-5.47E-01	4.97E-01	5.86E-01	4.39E-01	-6.68E-01	8.00E-01
ENSBTAG00000043532	0.00E+00	1.00E+00	0.00E+00	1.00E+00	-5.13E-15	1.00E+00	0.00E+00	1.00E+00	0.00E+00	1.00E+00
ENSBTAG00000021755	3.25E-01	8.23E-01	2.23E-01	6.83E-01	7.30E-01	1.93E-02	1.42E+00	1.03E-06	-3.50E-01	8.00E-01
ENSBTAG00000001060	1.92E+00	1.41E-02	1.66E+00	9.48E-03	4.70E-01	6.78E-01	2.11E+00	7.21E-04	1.39E-01	1.00E+00
ENSBTAG00000009949	3.02E-02	1.00E+00	5.04E-01	8.79E-03	9.53E-01	9.41E-08	8.51E-01	2.96E-06	-2.19E-01	7.99E-01
ENSBTAG00000015172	-7.79E-02	1.00E+00	-1.17E+00	3.38E-04	9.26E-02	1.00E+00	9.25E-01	3.51E-03	-3.38E-02	1.00E+00
ENSBTAG00000015170	-6.52E-01	7.71E-01	-6.52E-01	3.69E-01	-7.79E-01	2.30E-01	-1.33E+00	2.81E-02	6.92E-01	6.24E-01
ENSBTAG00000015169	1.12E-01	1.00E+00	2.38E-01	3.17E-01	4.65E-02	1.00E+00	4.79E-02	1.00E+00	-2.58E-01	6.46E-01
ENSBTAG00000013302	7.26E-02	1.00E+00	3.86E-01	1.42E-01	1.08E+00	6.38E-07	8.96E-01	6.09E-05	-3.60E-02	1.00E+00
ENSBTAG00000029990	0.00E+00	1.00E+00	0.00E+00	1.00E+00	-5.13E-15	1.00E+00	0.00E+00	1.00E+00	0.00E+00	1.00E+00
ENSBTAG00000043334	0.00E+00	1.00E+00	0.00E+00	1.00E+00	-5.13E-15	1.00E+00	0.00E+00	1.00E+00	2.71E+00	6.01E-01
ENSBTAG00000026842	1.50E-01	1.00E+00	1.09E+00	3.23E-07	2.10E-01	5.23E-01	-7.32E-01	1.70E-03	2.35E-01	8.00E-01
ENSBTAG00000013449	-2.55E-03	1.00E+00	7.55E-01	7.07E-05	7.88E-01	2.91E-05	8.51E-01	7.12E-06	4.91E-02	1.00E+00
ENSBTAG00000014409	3.09E-01	1.00E+00	6.40E-01	8.05E-01	-1.02E+00	6.17E-01	1.40E+00	2.07E-01	1.25E-02	1.00E+00
ENSBTAG00000048182	0.00E+00	1.00E+00	0.00E+00	1.00E+00	-5.13E-15	1.00E+00	0.00E+00	1.00E+00	0.00E+00	1.00E+00
ENSBTAG00000014158	-2.10E-01	8.33E-01	-5.47E-01	1.03E-02	-2.64E-01	3.14E-01	-4.74E-01	3.37E-02	-2.15E-01	8.00E-01
ENSBTAG00000008039	3.07E-01	1.00E+00	-8.44E-01	7.67E-02	-5.96E-01	2.62E-01	-4.69E-02	1.00E+00	5.88E-01	6.60E-01
ENSBTAG00000018753	5.68E-02	1.00E+00	9.82E-02	1.00E+00	-5.04E-01	4.89E-01	-1.24E-02	1.00E+00	-9.11E-02	1.00E+00
ENSBTAG00000018744	1.83E-01	1.00E+00	-2.47E-01	7.61E-01	-6.53E-01	1.30E-01	2.70E-01	6.93E-01	-9.80E-02	1.00E+00

ENSBTAG00000042177	0.00E+00	1.00E+00	0.00E+00	1.00E+00	-5.13E-15	1.00E+00	1.76E+00	4.97E-01	0.00E+00	1.00E+00
ENSBTAG00000043111	0.00E+00	1.00E+00	0.00E+00	1.00E+00	-5.13E-15	1.00E+00	0.00E+00	1.00E+00	0.00E+00	1.00E+00
ENSBTAG00000005238	9.24E-02	1.00E+00	-5.80E-01	1.16E-01	-2.57E+00	8.28E-14	-1.77E+00	7.84E-08	1.94E-01	1.00E+00
ENSBTAG00000005234	-2.35E-01	9.51E-01	-2.14E+00	4.90E-14	-3.29E+00	7.72E-28	-2.39E+00	2.73E-17	-3.61E-01	7.03E-01
ENSBTAG00000047466	0.00E+00	1.00E+00	0.00E+00	1.00E+00	-5.13E-15	1.00E+00	0.00E+00	1.00E+00	0.00E+00	1.00E+00
ENSBTAG00000047036	-2.88E-01	1.00E+00	-2.00E+00	2.64E-06	-3.28E+00	6.51E-13	-1.74E+00	2.27E-05	2.68E-01	1.00E+00
ENSBTAG00000021103	-6.51E-02	1.00E+00	-6.16E-01	5.55E-03	-5.40E-01	1.75E-02	-1.86E-01	6.00E-01	-2.69E-01	7.62E-01
ENSBTAG00000003401	2.16E-01	8.85E-01	3.55E-01	1.87E-01	6.55E-01	4.12E-03	8.62E-01	1.12E-04	-2.50E-01	8.00E-01
ENSBTAG00000046351	-6.09E-01	7.16E-01	-2.25E-01	9.27E-01	2.79E-01	8.11E-01	-4.75E-01	4.97E-01	-7.66E-01	5.32E-01
ENSBTAG00000045710	-7.52E-01	4.52E-01	-5.93E-01	2.98E-01	-3.19E-01	6.98E-01	-9.62E-01	5.41E-02	-1.25E+00	8.43E-02
ENSBTAG00000047913	0.00E+00	1.00E+00	0.00E+00	1.00E+00	-5.13E-15	1.00E+00	0.00E+00	1.00E+00	0.00E+00	1.00E+00
ENSBTAG00000047445	0.00E+00	1.00E+00	1.98E+00	4.43E-01	-5.13E-15	1.00E+00	0.00E+00	1.00E+00	0.00E+00	1.00E+00
ENSBTAG00000047042	-2.17E-01	1.00E+00	-2.75E-01	9.45E-01	1.79E-02	1.00E+00	-8.26E-01	2.89E-01	-9.77E-01	5.90E-01
ENSBTAG00000047790	2.49E-01	1.00E+00	2.66E-01	9.19E-01	-1.48E-01	1.00E+00	-9.21E-01	1.71E-01	-2.57E+00	1.15E-03
ENSBTAG00000002016	-1.91E+00	8.23E-01	-1.91E+00	4.69E-01	-1.91E+00	4.28E-01	-1.91E+00	4.39E-01	-1.91E+00	8.00E-01
ENSBTAG00000044578	0.00E+00	1.00E+00	0.00E+00	1.00E+00	-5.13E-15	1.00E+00	0.00E+00	1.00E+00	0.00E+00	1.00E+00
ENSBTAG00000005235	-5.08E-01	8.29E-01	-5.93E-01	3.81E-01	-1.36E+00	1.32E-02	-9.57E-01	9.60E-02	-1.60E+00	6.02E-02
ENSBTAG00000046281	2.70E+00	5.76E-01	0.00E+00	1.00E+00	-5.13E-15	1.00E+00	0.00E+00	1.00E+00	0.00E+00	1.00E+00
ENSBTAG00000042773	-1.91E+00	8.23E-01	-1.91E+00	4.69E-01	-1.91E+00	4.28E-01	-1.91E+00	4.39E-01	-1.91E+00	8.00E-01
ENSBTAG00000042265	0.00E+00	1.00E+00	0.00E+00	1.00E+00	-5.13E-15	1.00E+00	0.00E+00	1.00E+00	0.00E+00	1.00E+00
ENSBTAG00000015077	9.31E-01	2.76E-02	3.08E+00	6.13E-24	3.15E+00	4.39E-25	1.86E+00	1.12E-09	1.37E-01	1.00E+00
ENSBTAG00000016785	2.72E+00	5.76E-01	2.79E+00	2.11E-01	3.05E+00	1.37E-01	3.68E+00	4.84E-02	0.00E+00	1.00E+00
ENSBTAG00000010221	9.09E-02	1.00E+00	-7.05E-01	2.67E-04	-2.83E-01	2.29E-01	1.45E-01	6.82E-01	7.67E-02	1.00E+00
ENSBTAG00000002112	-1.84E-01	1.00E+00	-1.62E+00	5.51E-12	-1.40E+00	1.82E-09	-8.51E-01	4.84E-04	1.32E-01	1.00E+00
ENSBTAG00000021494	0.00E+00	1.00E+00	0.00E+00	1.00E+00	1.78E+00	4.79E-01	0.00E+00	1.00E+00	1.91E+00	8.00E-01
ENSBTAG00000018473	5.47E-01	9.12E-01	8.85E-01	2.17E-01	8.90E-01	2.13E-01	5.51E-01	5.40E-01	1.85E-01	1.00E+00
ENSBTAG00000003771	5.12E-03	1.00E+00	-2.71E+00	2.42E-01	-9.35E-01	8.05E-01	-9.52E-01	8.02E-01	-7.98E-01	1.00E+00
ENSBTAG00000020794	0.00E+00	1.00E+00	0.00E+00	1.00E+00	-5.13E-15	1.00E+00	0.00E+00	1.00E+00	0.00E+00	1.00E+00
ENSBTAG00000007111	1.19E+00	2.04E-04	8.95E-01	2.05E-03	8.15E-01	5.48E-03	1.07E+00	2.02E-04	1.21E+00	5.76E-04
ENSBTAG00000010599	3.23E-01	7.66E-01	7.10E-01	8.33E-03	-7.81E-01	4.83E-03	-7.56E-01	7.59E-03	-2.23E-01	9.37E-01
ENSBTAG00000009517	1.37E-01	1.00E+00	3.96E-01	2.53E-01	2.17E-02	1.00E+00	-1.55E-01	8.62E-01	-3.90E-01	6.48E-01
ENSBTAG00000026825	6.30E-01	4.68E-01	-1.06E+00	9.33E-03	-2.16E+00	4.36E-08	-1.20E+00	3.09E-03	-7.23E-01	4.20E-01
ENSBTAG00000013619	-7.49E-03	1.00E+00	1.99E-02	1.00E+00	1.54E-01	1.00E+00	-6.67E-01	2.62E-01	-1.83E-01	1.00E+00
ENSBTAG00000009057	-9.24E-02	1.00E+00	-6.23E-01	4.31E-01	-3.20E-01	8.23E-01	-5.39E-01	5.05E-01	-6.01E-01	8.00E-01
ENSBTAG00000009058	-3.27E-01	8.23E-01	-1.03E+00	1.16E-03	-4.63E-02	1.00E+00	1.93E-01	7.82E-01	-2.22E-01	1.00E+00

ENSBTAG00000020855	2.22E-01	1.00E+00	-7.37E-01	9.23E-02	-4.22E-01	4.26E-01	6.31E-01	1.47E-01	2.07E-01	1.00E+00
ENSBTAG00000048209	0.00E+00	1.00E+00	0.00E+00	1.00E+00	-5.13E-15	1.00E+00	0.00E+00	1.00E+00	0.00E+00	1.00E+00
ENSBTAG00000018236	-2.30E-01	8.02E-01	-1.08E+00	1.34E-08	-1.29E+00	6.48E-12	-1.11E+00	6.07E-09	-1.93E-01	8.36E-01
ENSBTAG00000043360	0.00E+00	1.00E+00	0.00E+00	1.00E+00	-5.13E-15	1.00E+00	0.00E+00	1.00E+00	0.00E+00	1.00E+00
ENSBTAG00000026829	1.83E-01	1.00E+00	1.57E+00	3.37E-10	1.87E+00	3.83E-14	1.12E+00	1.31E-05	4.10E-02	1.00E+00
ENSBTAG00000032021	-1.56E-01	1.00E+00	-7.26E-01	2.20E-03	-2.02E-01	5.78E-01	-8.68E-02	1.00E+00	-3.27E-01	6.36E-01
ENSBTAG00000032017	-1.33E+00	4.47E-01	-1.27E+00	1.79E-01	3.70E-01	8.34E-01	1.93E+00	1.36E-03	7.70E-03	1.00E+00
ENSBTAG00000019464	3.09E-01	5.76E-01	1.90E+00	2.24E-23	1.74E+00	4.11E-20	1.59E+00	8.15E-17	-2.98E-01	6.20E-01
ENSBTAG00000011682	2.44E-01	1.00E+00	9.51E-01	1.90E-01	-2.59E-01	1.00E+00	1.28E+00	4.17E-02	3.35E-01	1.00E+00
ENSBTAG00000007765	-3.84E-01	1.00E+00	-1.97E-01	1.00E+00	-8.22E-01	3.84E-01	-8.53E-01	3.69E-01	-3.62E-01	1.00E+00
ENSBTAG00000048035	0.00E+00	1.00E+00	0.00E+00	1.00E+00	-5.13E-15	1.00E+00	0.00E+00	1.00E+00	0.00E+00	1.00E+00
ENSBTAG00000019781	7.13E-02	1.00E+00	-6.80E-01	9.89E-03	-3.35E-01	2.89E-01	-4.17E-02	1.00E+00	3.16E-01	7.37E-01
ENSBTAG00000043135	0.00E+00	1.00E+00	0.00E+00	1.00E+00	-5.13E-15	1.00E+00	0.00E+00	1.00E+00	0.00E+00	1.00E+00
ENSBTAG00000019387	4.42E-01	1.90E-01	2.15E+00	3.44E-29	2.39E+00	1.50E-35	1.63E+00	1.99E-17	-4.31E-01	3.07E-01
ENSBTAG00000006059	2.21E-01	9.00E-01	9.42E-01	4.22E-05	1.04E+00	4.96E-06	9.78E-01	2.38E-05	2.25E-01	8.56E-01
ENSBTAG00000026822	-2.28E-01	1.00E+00	-5.53E-01	2.00E-01	-1.35E+00	2.19E-04	-7.88E-01	4.59E-02	2.03E-01	1.00E+00
ENSBTAG00000031918	-1.29E+00	9.38E-01	-3.21E+00	1.26E-01	-3.21E+00	9.49E-02	-1.45E+00	4.97E-01	-5.00E-01	1.00E+00
ENSBTAG00000047681	1.91E+00	8.23E-01	0.00E+00	1.00E+00	-5.13E-15	1.00E+00	0.00E+00	1.00E+00	0.00E+00	1.00E+00
ENSBTAG00000031898	-8.07E-01	6.32E-01	3.28E-01	8.09E-01	-7.43E-01	3.01E-01	2.00E-01	1.00E+00	-3.81E-01	1.00E+00
ENSBTAG00000044835	0.00E+00	1.00E+00	0.00E+00	1.00E+00	-5.13E-15	1.00E+00	0.00E+00	1.00E+00	0.00E+00	1.00E+00
ENSBTAG00000014863	-3.87E-01	8.82E-01	-1.45E+00	3.99E-04	-6.96E-01	1.33E-01	-8.19E-01	7.11E-02	-5.10E-01	7.42E-01
ENSBTAG00000007863	1.96E-02	1.00E+00	4.72E-01	1.30E-01	1.46E-01	8.69E-01	-1.65E-01	8.14E-01	-5.93E-01	2.72E-01
ENSBTAG00000007867	-6.77E-02	1.00E+00	7.29E-01	3.22E-01	6.13E-01	4.36E-01	-9.13E-02	1.00E+00	-4.66E-01	9.45E-01
ENSBTAG00000046699	-4.50E-02	1.00E+00	-1.18E+00	1.16E-02	-2.93E+00	9.07E-10	-2.41E+00	2.33E-07	3.39E-01	9.62E-01
ENSBTAG00000011256	1.35E-01	1.00E+00	-3.60E-01	2.17E-01	-3.01E-01	3.37E-01	-2.88E-01	3.86E-01	2.98E-01	7.64E-01
ENSBTAG00000018653	1.17E+00	3.74E-04	1.67E+00	3.02E-09	1.99E+00	1.27E-12	1.06E+00	2.89E-04	4.15E-01	6.24E-01
ENSBTAG00000018497	-7.90E-01	1.09E-01	-2.23E+00	4.74E-12	-1.17E+00	3.36E-04	-1.04E+00	1.99E-03	-7.36E-01	2.25E-01
ENSBTAG00000014832	-1.07E+00	3.75E-01	-1.26E+00	5.84E-02	-1.78E+00	5.21E-03	-5.67E-01	4.95E-01	-8.99E-01	5.90E-01
ENSBTAG00000046400	0.00E+00	1.00E+00	0.00E+00	1.00E+00	-5.13E-15	1.00E+00	0.00E+00	1.00E+00	0.00E+00	1.00E+00
ENSBTAG00000044658	0.00E+00	1.00E+00	0.00E+00	1.00E+00	-5.13E-15	1.00E+00	0.00E+00	1.00E+00	0.00E+00	1.00E+00
ENSBTAG00000047197	0.00E+00	1.00E+00	0.00E+00	1.00E+00	-5.13E-15	1.00E+00	0.00E+00	1.00E+00	0.00E+00	1.00E+00
ENSBTAG00000047783	2.62E-01	1.00E+00	-9.54E-01	7.71E-03	-1.04E+00	2.89E-03	-5.20E-01	2.06E-01	1.50E-01	1.00E+00
ENSBTAG00000037816	0.00E+00	1.00E+00	0.00E+00	1.00E+00	-5.13E-15	1.00E+00	1.76E+00	4.97E-01	0.00E+00	1.00E+00
ENSBTAG00000009976	-9.14E-02	1.00E+00	-4.42E-01	1.23E-01	-7.16E-01	5.20E-03	-6.86E-01	9.11E-03	1.49E-02	1.00E+00
ENSBTAG00000016782	-4.74E-01	7.39E-01	-2.43E-01	7.72E-01	1.44E+00	5.54E-05	2.13E+00	1.67E-09	-7.10E-01	4.04E-01

ENSBTAG00000016784	3.76E-01	9.08E-01	-7.98E-01	1.26E-01	-4.73E-01	4.28E-01	1.66E-02	1.00E+00	-7.17E-02	1.00E+00
ENSBTAG00000013196	4.71E-02	1.00E+00	-2.90E-01	4.50E-01	-2.61E-01	5.04E-01	-4.77E-01	1.37E-01	-1.37E-01	1.00E+00
ENSBTAG00000047862	-1.06E-01	1.00E+00	1.03E+00	6.70E-02	-2.92E-01	8.95E-01	1.57E-01	1.00E+00	-1.41E-01	1.00E+00
ENSBTAG00000020696	9.31E-01	3.17E-01	1.42E+00	4.01E-03	6.99E-01	2.43E-01	1.08E+00	4.31E-02	2.55E-01	1.00E+00
ENSBTAG00000017695	4.57E-01	5.76E-01	1.70E-01	8.72E-01	6.67E-01	4.00E-02	6.57E-01	4.90E-02	6.53E-02	1.00E+00
ENSBTAG00000007779	-2.61E-01	8.23E-01	1.20E-01	9.18E-01	6.48E-01	9.51E-03	4.38E-01	1.19E-01	1.94E-01	9.66E-01
ENSBTAG00000047452	0.00E+00	1.00E+00	0.00E+00	1.00E+00	1.78E+00	4.79E-01	0.00E+00	1.00E+00	0.00E+00	1.00E+00
ENSBTAG00000020416	2.51E-01	1.00E+00	-1.03E+00	1.74E-02	-4.48E-01	3.87E-01	6.68E-01	9.98E-02	4.70E-01	7.20E-01
ENSBTAG00000031696	1.46E-01	1.00E+00	2.59E-02	1.00E+00	1.23E+00	7.87E-05	6.31E-01	8.06E-02	1.34E-01	1.00E+00
ENSBTAG00000044361	0.00E+00	1.00E+00	0.00E+00	1.00E+00	-5.13E-15	1.00E+00	0.00E+00	1.00E+00	0.00E+00	1.00E+00
ENSBTAG00000012171	0.00E+00	1.00E+00	2.79E+00	2.10E-01	-5.13E-15	1.00E+00	1.76E+00	4.97E-01	0.00E+00	1.00E+00
ENSBTAG00000008915	1.34E-01	1.00E+00	3.79E-01	1.09E-01	8.65E-02	9.80E-01	-2.57E-01	3.62E-01	-1.12E-01	1.00E+00
ENSBTAG00000004659	1.00E+00	4.72E-03	2.12E+00	9.09E-14	1.32E+00	3.68E-06	6.47E-01	4.55E-02	-5.74E-01	3.72E-01
ENSBTAG00000043296	0.00E+00	1.00E+00	0.00E+00	1.00E+00	-5.13E-15	1.00E+00	0.00E+00	1.00E+00	0.00E+00	1.00E+00
ENSBTAG00000012586	9.93E-01	2.85E-03	2.39E+00	2.21E-18	1.96E+00	4.52E-13	1.76E+00	1.00E-10	1.67E-01	1.00E+00
ENSBTAG00000012589	2.95E-01	7.27E-01	1.54E+00	3.17E-12	1.19E+00	8.19E-08	9.45E-01	3.57E-05	1.69E-01	1.00E+00
ENSBTAG00000019116	3.02E-01	7.04E-01	3.18E-01	2.71E-01	4.82E-01	5.49E-02	1.01E-01	9.69E-01	-4.38E-01	4.13E-01
ENSBTAG00000007652	6.36E-01	6.01E-01	1.48E+00	3.87E-04	-1.70E-02	1.00E+00	-1.08E-01	1.00E+00	8.50E-02	1.00E+00
ENSBTAG00000002056	4.68E-03	1.00E+00	9.71E-01	7.84E-04	1.63E+00	2.33E-09	1.02E+00	4.26E-04	-7.26E-02	1.00E+00
ENSBTAG00000031614	-1.74E+00	1.10E-01	5.12E-01	5.49E-01	4.11E-01	6.84E-01	-4.49E-01	6.86E-01	-9.77E-01	6.01E-01
ENSBTAG00000007635	-6.02E-01	1.67E-01	-9.06E-01	1.07E-03	-9.78E-01	2.86E-04	-6.25E-01	3.10E-02	-2.87E-01	8.00E-01
ENSBTAG00000042540	5.09E-03	1.00E+00	8.74E-01	8.53E-01	-1.32E-01	1.00E+00	6.14E-01	9.97E-01	7.92E-01	1.00E+00
ENSBTAG00000044781	0.00E+00	1.00E+00	0.00E+00	1.00E+00	-5.13E-15	1.00E+00	1.76E+00	4.97E-01	0.00E+00	1.00E+00
ENSBTAG00000042810	0.00E+00	1.00E+00	0.00E+00	1.00E+00	-5.13E-15	1.00E+00	0.00E+00	1.00E+00	0.00E+00	1.00E+00
ENSBTAG00000044950	0.00E+00	1.00E+00	0.00E+00	1.00E+00	-5.13E-15	1.00E+00	0.00E+00	1.00E+00	0.00E+00	1.00E+00
ENSBTAG00000016334	9.79E-02	1.00E+00	-5.54E-01	3.41E-01	9.93E-01	1.58E-02	1.52E+00	7.87E-05	3.93E-02	1.00E+00
ENSBTAG00000042887	0.00E+00	1.00E+00	0.00E+00	1.00E+00	-5.13E-15	1.00E+00	0.00E+00	1.00E+00	0.00E+00	1.00E+00
ENSBTAG00000031595	4.30E-01	5.39E-01	1.47E+00	3.62E-08	5.01E-01	1.08E-01	-2.77E-01	4.97E-01	-2.64E-01	8.55E-01
ENSBTAG00000009952	2.03E-01	1.00E+00	4.60E-01	2.43E-01	8.42E-01	8.38E-03	1.29E+00	1.71E-05	1.73E-01	1.00E+00
ENSBTAG00000019232	6.11E-01	1.51E-01	1.21E+00	3.10E-06	8.87E-01	8.76E-04	1.02E+00	1.17E-04	1.01E-01	1.00E+00
ENSBTAG00000019231	-2.66E-02	1.00E+00	1.37E-01	8.17E-01	3.51E-01	1.95E-01	1.29E-01	8.51E-01	-1.13E-01	1.00E+00
ENSBTAG00000024003	0.00E+00	1.00E+00	0.00E+00	1.00E+00	-5.13E-15	1.00E+00	0.00E+00	1.00E+00	0.00E+00	1.00E+00
ENSBTAG00000016092	-1.33E-01	1.00E+00	-1.31E+00	4.55E-11	-2.00E+00	1.35E-23	-1.53E+00	1.65E-14	-9.79E-03	1.00E+00
ENSBTAG00000002272	-3.87E-01	5.79E-01	-1.56E+00	5.90E-09	-5.47E-01	5.86E-02	-1.10E-01	9.81E-01	7.38E-02	1.00E+00
ENSBTAG00000031579	-2.06E-01	9.86E-01	-9.38E-01	2.28E-04	-2.69E-01	4.43E-01	2.90E-01	4.08E-01	2.06E-01	9.46E-01

ENSBTAG00000009725	-5.98E-02	1.00E+00	-9.38E-01	1.10E-04	-1.94E+00	1.89E-16	-2.23E+00	7.56E-21	5.06E-01	3.09E-01
ENSBTAG00000038878	2.67E+00	1.55E-03	1.45E+00	6.47E-02	8.26E-01	3.93E-01	3.61E-01	9.05E-01	2.64E+00	5.89E-03
ENSBTAG00000047849	0.00E+00	1.00E+00	0.00E+00	1.00E+00	-5.13E-15	1.00E+00	0.00E+00	1.00E+00	0.00E+00	1.00E+00
ENSBTAG00000026812	1.01E-01	1.00E+00	2.64E+00	2.40E-05	3.93E+00	1.26E-10	3.47E+00	1.74E-08	1.54E+00	1.86E-01
ENSBTAG00000006049	1.15E-01	1.00E+00	6.28E-01	8.53E-03	1.33E+00	1.81E-09	1.74E+00	4.38E-15	-6.30E-02	1.00E+00
ENSBTAG00000005408	8.46E-01	1.00E-03	4.73E-01	5.16E-02	-4.09E-01	1.06E-01	-6.28E-01	7.34E-03	-1.25E-01	1.00E+00
ENSBTAG00000005416	1.12E-01	1.00E+00	3.22E-01	2.94E-01	1.36E-01	8.43E-01	2.52E-01	4.72E-01	-1.94E-01	9.70E-01
ENSBTAG00000018282	1.06E-01	1.00E+00	-8.82E-02	1.00E+00	4.77E-01	1.02E-01	7.04E-01	9.53E-03	-9.20E-02	1.00E+00
ENSBTAG00000031473	-2.69E-02	1.00E+00	-5.06E-01	1.37E-01	-1.01E+00	6.39E-04	-5.19E-01	1.32E-01	2.00E-02	1.00E+00
ENSBTAG00000021201	7.58E-01	1.73E-01	2.25E+00	1.16E-11	1.67E+00	4.45E-07	1.13E+00	1.14E-03	-4.60E-02	1.00E+00
ENSBTAG00000045431	0.00E+00	1.00E+00	0.00E+00	1.00E+00	-5.13E-15	1.00E+00	0.00E+00	1.00E+00	0.00E+00	1.00E+00
ENSBTAG00000012760	5.02E-02	1.00E+00	3.95E-01	7.86E-02	1.75E-01	5.84E-01	-2.39E-01	3.99E-01	-2.80E-01	6.60E-01
ENSBTAG00000012763	9.70E-03	1.00E+00	8.98E-02	1.00E+00	-1.44E+00	4.79E-01	-1.87E-01	1.00E+00	-1.30E+00	9.00E-01
ENSBTAG00000010998	1.37E+00	1.41E-07	1.80E+00	5.99E-14	1.15E+00	2.49E-06	6.86E-01	9.46E-03	1.79E-01	1.00E+00
ENSBTAG00000003731	-1.91E+00	8.23E-01	-1.91E+00	4.69E-01	-1.25E-01	1.00E+00	-1.48E-01	1.00E+00	-1.91E+00	8.00E-01
ENSBTAG00000015718	2.90E-01	1.00E+00	1.63E+00	5.61E-05	1.04E+00	1.40E-02	3.85E-01	5.25E-01	-5.41E-02	1.00E+00
ENSBTAG00000012334	1.47E-01	1.00E+00	1.88E-01	8.71E-01	-8.16E-01	3.66E-02	-4.89E-01	2.81E-01	6.43E-02	1.00E+00
ENSBTAG00000014933	-1.47E-01	1.00E+00	-6.02E-01	5.60E-02	-9.36E-01	1.28E-03	-4.29E-02	1.00E+00	4.55E-01	5.43E-01
ENSBTAG00000019298	-3.55E-01	6.15E-01	-9.96E-01	6.76E-05	-1.07E+00	1.48E-05	-5.08E-01	7.30E-02	-3.61E-02	1.00E+00
ENSBTAG00000017436	2.46E-02	1.00E+00	1.62E-01	8.62E-01	-3.86E-01	3.06E-01	-1.54E-01	8.88E-01	-2.94E-01	8.34E-01
ENSBTAG00000017437	-2.73E-02	1.00E+00	-6.50E-01	5.87E-02	1.66E-01	8.47E-01	4.93E-01	1.55E-01	3.05E-01	8.07E-01
ENSBTAG00000046831	0.00E+00	1.00E+00	0.00E+00	1.00E+00	-5.13E-15	1.00E+00	0.00E+00	1.00E+00	0.00E+00	1.00E+00
ENSBTAG00000007395	-4.29E-02	1.00E+00	-1.02E+00	2.09E-06	-9.71E-01	5.44E-06	-3.60E-01	1.67E-01	2.30E-01	8.00E-01
ENSBTAG00000002283	1.76E-01	1.00E+00	-1.07E+00	2.02E-02	5.19E-01	2.90E-01	8.36E-01	4.85E-02	2.05E-01	1.00E+00
ENSBTAG00000034192	-1.84E-01	1.00E+00	-1.41E+00	1.01E-03	-1.50E+00	4.06E-04	-4.93E-01	3.98E-01	5.94E-02	1.00E+00
ENSBTAG00000009859	-1.92E-01	9.09E-01	-3.16E-01	2.06E-01	-4.33E-01	5.45E-02	-5.92E-01	6.11E-03	-3.95E-01	3.85E-01
ENSBTAG00000043194	0.00E+00	1.00E+00	0.00E+00	1.00E+00	-5.13E-15	1.00E+00	0.00E+00	1.00E+00	0.00E+00	1.00E+00
ENSBTAG00000005517	5.51E-01	2.86E-02	2.23E+00	1.30E-34	2.37E+00	5.61E-39	1.63E+00	1.82E-19	-5.50E-01	6.06E-02
ENSBTAG00000043261	0.00E+00	1.00E+00	0.00E+00	1.00E+00	-5.13E-15	1.00E+00	0.00E+00	1.00E+00	0.00E+00	1.00E+00
ENSBTAG00000042181	0.00E+00	1.00E+00	0.00E+00	1.00E+00	-5.13E-15	1.00E+00	0.00E+00	1.00E+00	0.00E+00	1.00E+00
ENSBTAG00000045062	0.00E+00	1.00E+00	0.00E+00	1.00E+00	-5.13E-15	1.00E+00	0.00E+00	1.00E+00	0.00E+00	1.00E+00
ENSBTAG00000045316	0.00E+00	1.00E+00	0.00E+00	1.00E+00	-5.13E-15	1.00E+00	0.00E+00	1.00E+00	0.00E+00	1.00E+00
ENSBTAG00000006420	2.50E-01	9.53E-01	6.21E-01	4.92E-02	-1.47E-01	9.01E-01	6.93E-01	2.68E-02	6.95E-01	1.76E-01
ENSBTAG00000018746	-8.63E-02	1.00E+00	7.63E-01	3.69E-02	9.10E-02	1.00E+00	7.47E-01	4.62E-02	-2.08E-01	1.00E+00
ENSBTAG00000008673	4.43E-01	9.69E-01	2.47E+00	7.66E-09	1.65E+00	3.41E-04	4.85E-01	5.35E-01	-2.46E-01	1.00E+00

ENSBTAG00000015424	1.85E-01	9.37E-01	1.94E+00	4.71E-24	2.24E+00	1.27E-31	1.50E+00	6.96E-15	-3.98E-01	4.10E-01
ENSBTAG00000016000	-1.48E-01	1.00E+00	-1.08E+00	1.61E-06	-5.48E-01	2.21E-02	-8.54E-01	1.91E-04	-2.45E-01	8.00E-01
ENSBTAG00000019994	0.00E+00	1.00E+00	0.00E+00	1.00E+00	-5.13E-15	1.00E+00	0.00E+00	1.00E+00	0.00E+00	1.00E+00
ENSBTAG00000014469	9.79E-02	1.00E+00	-6.10E-01	8.96E-02	-1.28E+00	5.96E-05	-4.70E-01	2.37E-01	7.41E-04	1.00E+00
ENSBTAG00000000851	4.09E-02	1.00E+00	-6.06E-01	1.08E-02	-5.63E-01	1.89E-02	-3.48E-01	2.13E-01	-2.63E-01	8.00E-01
ENSBTAG00000011017	3.63E-01	8.57E-01	3.66E+00	4.20E-25	2.35E+00	7.49E-12	5.70E-01	1.93E-01	-1.50E-01	1.00E+00
ENSBTAG00000027201	1.10E+00	4.20E-03	2.44E+00	6.12E-15	1.71E+00	3.92E-08	9.40E-01	4.73E-03	-9.83E-02	1.00E+00
ENSBTAG00000007445	4.90E-01	5.99E-01	2.07E+00	1.77E-11	5.98E-01	1.18E-01	-6.13E-01	1.51E-01	1.03E-01	1.00E+00
ENSBTAG00000013170	3.59E-01	1.00E+00	1.17E+00	2.02E-01	1.07E-02	1.00E+00	1.24E-02	1.00E+00	-1.89E-01	1.00E+00
ENSBTAG00000021596	-1.10E-01	1.00E+00	7.33E-01	2.45E-01	2.33E-01	9.59E-01	-8.99E-02	1.00E+00	-5.41E-01	8.85E-01
ENSBTAG00000040367	1.16E-01	1.00E+00	1.60E-01	7.26E-01	-1.64E-01	7.06E-01	-1.60E-01	7.35E-01	-9.75E-02	1.00E+00
ENSBTAG00000045092	0.00E+00	1.00E+00	0.00E+00	1.00E+00	-5.13E-15	1.00E+00	0.00E+00	1.00E+00	0.00E+00	1.00E+00
ENSBTAG00000046521	-1.48E+00	5.76E-01	-1.41E+00	2.54E-01	-3.55E-01	9.68E-01	-7.57E-01	5.85E-01	-5.51E-01	1.00E+00
ENSBTAG00000010293	-2.97E-01	1.00E+00	-2.39E-01	9.04E-01	-2.03E-01	9.60E-01	9.50E-02	1.00E+00	-2.14E-01	1.00E+00
ENSBTAG00000046783	-5.07E-01	1.00E+00	-3.57E-01	8.52E-01	-1.11E+00	1.46E-01	-8.29E-01	3.24E-01	1.61E-01	1.00E+00
ENSBTAG00000046029	-4.40E-01	1.00E+00	8.35E-01	3.23E-01	-1.82E-01	1.00E+00	-3.03E-01	9.76E-01	7.02E-01	8.00E-01
ENSBTAG00000011971	6.06E-01	1.41E-01	7.61E-01	4.49E-03	9.62E-02	1.00E+00	1.55E-01	8.30E-01	-5.13E-01	3.75E-01
ENSBTAG00000028809	0.00E+00	1.00E+00	0.00E+00	1.00E+00	-5.13E-15	1.00E+00	0.00E+00	1.00E+00	0.00E+00	1.00E+00
ENSBTAG00000014407	4.84E-01	6.42E-01	7.45E-01	4.88E-02	2.49E-01	7.13E-01	5.57E-01	1.84E-01	2.80E-01	9.69E-01
ENSBTAG00000021976	-1.17E-01	1.00E+00	-5.41E-01	6.97E-02	-9.62E-01	3.62E-04	-4.61E-01	1.50E-01	1.09E-01	1.00E+00
ENSBTAG00000021979	1.07E-01	1.00E+00	5.47E-01	4.16E-02	5.28E-01	5.09E-02	3.69E-01	2.33E-01	-6.98E-01	7.10E-02
ENSBTAG00000043166	0.00E+00	1.00E+00	0.00E+00	1.00E+00	-5.13E-15	1.00E+00	0.00E+00	1.00E+00	0.00E+00	1.00E+00
ENSBTAG00000042507	0.00E+00	1.00E+00	0.00E+00	1.00E+00	-5.13E-15	1.00E+00	0.00E+00	1.00E+00	0.00E+00	1.00E+00
ENSBTAG00000017043	-5.62E-01	4.50E-01	-2.15E+00	1.02E-10	-3.35E+00	2.06E-22	-3.25E+00	3.63E-21	5.98E-01	4.35E-01
ENSBTAG00000031166	3.48E-01	1.00E+00	-8.06E-01	2.06E-01	-1.56E+00	5.00E-03	-2.27E+00	6.62E-05	-2.43E-01	1.00E+00
ENSBTAG00000019694	3.31E-02	1.00E+00	1.26E+00	2.34E-06	1.64E+00	4.33E-10	1.27E+00	2.23E-06	-4.66E-01	5.13E-01
ENSBTAG00000046454	2.36E-01	1.00E+00	-1.40E-01	1.00E+00	-1.07E+00	4.79E-01	-7.29E-01	7.09E-01	-2.40E-01	1.00E+00
ENSBTAG00000016192	3.15E-01	8.90E-01	-1.92E+00	8.33E-07	-2.10E-01	7.95E-01	3.96E-01	3.75E-01	5.36E-01	5.25E-01
ENSBTAG00000016193	-1.84E-01	1.00E+00	-2.75E-01	4.70E-01	1.20E+00	2.92E-06	1.13E+00	1.42E-05	-5.74E-03	1.00E+00
ENSBTAG00000019190	-7.87E-01	1.00E+00	9.77E-01	6.52E-01	-2.71E+00	1.99E-01	9.95E-01	6.18E-01	-7.92E-01	1.00E+00
ENSBTAG00000044097	6.76E-01	2.43E-01	1.44E+00	8.13E-06	1.86E-01	8.48E-01	-2.73E-01	6.35E-01	-2.63E-02	1.00E+00
ENSBTAG00000045074	0.00E+00	1.00E+00	0.00E+00	1.00E+00	-5.13E-15	1.00E+00	0.00E+00	1.00E+00	0.00E+00	1.00E+00
ENSBTAG00000012625	-1.08E-02	1.00E+00	-5.15E-01	1.23E-01	-8.63E-01	3.80E-03	-4.88E-01	1.53E-01	-2.76E-01	8.56E-01
ENSBTAG00000005474	5.43E-01	1.61E-01	1.28E+00	2.94E-08	9.42E-01	6.10E-05	5.21E-01	5.05E-02	-2.82E-01	8.00E-01
ENSBTAG00000005476	3.81E-01	4.70E-01	1.67E+00	9.31E-15	1.99E+00	1.14E-20	1.76E+00	2.28E-16	-1.77E-01	9.98E-01

ENSBTAG00000044599	0.00E+00	1.00E+00	0.00E+00	1.00E+00	-5.13E-15	1.00E+00	0.00E+00	1.00E+00	0.00E+00	1.00E+00
ENSBTAG00000016297	-4.17E-01	5.76E-01	9.22E-01	1.06E-03	1.10E+00	6.40E-05	2.09E-01	6.93E-01	-3.49E-02	1.00E+00
ENSBTAG00000020957	-7.64E-01	2.29E-01	3.80E-01	4.64E-01	6.90E-01	9.29E-02	6.26E-02	1.00E+00	6.79E-01	4.16E-01
ENSBTAG00000007830	2.87E-01	1.00E+00	-2.90E-01	7.44E-01	1.02E-01	1.00E+00	-7.23E-02	1.00E+00	8.44E-01	3.07E-01
ENSBTAG00000014094	0.00E+00	1.00E+00	1.98E+00	4.43E-01	-5.13E-15	1.00E+00	3.39E+00	8.39E-02	0.00E+00	1.00E+00
ENSBTAG00000015054	0.00E+00	1.00E+00	0.00E+00	1.00E+00	-5.13E-15	1.00E+00	0.00E+00	1.00E+00	0.00E+00	1.00E+00
ENSBTAG00000014783	1.91E+00	8.23E-01	0.00E+00	1.00E+00	-5.13E-15	1.00E+00	0.00E+00	1.00E+00	0.00E+00	1.00E+00
ENSBTAG00000021770	0.00E+00	1.00E+00	1.98E+00	4.43E-01	-5.13E-15	1.00E+00	1.76E+00	4.97E-01	0.00E+00	1.00E+00
ENSBTAG00000046832	0.00E+00	1.00E+00	0.00E+00	1.00E+00	-5.13E-15	1.00E+00	0.00E+00	1.00E+00	0.00E+00	1.00E+00
ENSBTAG00000012888	-3.21E+00	3.75E-01	-4.20E-01	1.00E+00	-1.43E+00	4.79E-01	-6.79E-01	8.87E-01	2.92E-02	1.00E+00
ENSBTAG00000020527	-4.34E-01	3.60E-01	-1.35E+00	4.23E-09	-2.65E+00	3.04E-30	-2.98E+00	9.20E-37	-7.45E-02	1.00E+00
ENSBTAG00000002177	-2.19E-02	1.00E+00	-6.94E-01	4.16E-03	2.18E-01	5.20E-01	3.31E-01	2.55E-01	-1.72E-01	1.00E+00
ENSBTAG00000002180	-1.64E-01	1.00E+00	1.07E-01	1.00E+00	3.67E-01	4.77E-01	3.91E-02	1.00E+00	2.65E-01	1.00E+00
ENSBTAG00000011992	-8.64E-01	1.00E+00	1.04E+00	4.43E-01	3.90E-01	1.00E+00	1.61E+00	1.00E-01	2.81E-02	1.00E+00
ENSBTAG00000047386	0.00E+00	1.00E+00	0.00E+00	1.00E+00	-5.13E-15	1.00E+00	0.00E+00	1.00E+00	0.00E+00	1.00E+00
ENSBTAG00000046940	-2.71E+00	5.76E-01	-2.71E+00	2.42E-01	3.49E-01	1.00E+00	-2.71E+00	2.05E-01	-2.71E+00	6.01E-01
ENSBTAG00000018130	-5.47E-01	6.83E-01	-1.51E+00	2.87E-04	-2.57E+00	7.06E-10	-2.63E+00	3.84E-10	8.61E-01	2.95E-01
ENSBTAG00000015415	-1.85E-01	1.00E+00	-5.77E-01	2.35E-01	-1.05E+00	1.21E-02	-1.03E+00	1.52E-02	8.55E-02	1.00E+00
ENSBTAG00000045083	0.00E+00	1.00E+00	0.00E+00	1.00E+00	-5.13E-15	1.00E+00	0.00E+00	1.00E+00	0.00E+00	1.00E+00
ENSBTAG00000047806	-3.70E-02	1.00E+00	6.96E-01	2.71E-02	1.03E+00	4.49E-04	1.02E+00	6.22E-04	5.35E-02	1.00E+00
ENSBTAG00000021600	1.54E-01	1.00E+00	6.54E-01	1.03E-02	-2.35E-01	5.13E-01	-4.48E-01	1.20E-01	-2.16E-01	9.12E-01
ENSBTAG00000010579	-1.92E-01	1.00E+00	-7.75E-01	4.30E-02	-1.31E+00	2.03E-04	-1.70E+00	1.51E-06	-4.10E-01	8.00E-01
ENSBTAG00000010722	-3.52E-01	7.76E-01	-1.97E+00	8.80E-12	-2.58E+00	8.22E-19	-1.01E+00	6.71E-04	-1.77E-01	1.00E+00
ENSBTAG00000009707	-1.59E+00	4.49E-01	-1.49E-03	1.00E+00	-2.86E+00	5.75E-03	-5.53E-01	8.11E-01	-1.69E+00	4.41E-01
ENSBTAG00000015066	-5.04E-02	1.00E+00	-8.25E-01	4.84E-03	-6.73E-01	2.61E-02	-3.95E-01	2.76E-01	-6.20E-02	1.00E+00
ENSBTAG00000016662	4.25E-02	1.00E+00	-4.40E-01	4.04E-01	-8.19E-01	4.90E-02	-5.06E-02	1.00E+00	4.60E-01	7.79E-01
ENSBTAG00000012647	8.02E-02	1.00E+00	-2.00E-01	1.00E+00	-9.69E-01	2.36E-01	-1.65E-01	1.00E+00	8.16E-02	1.00E+00
ENSBTAG00000047490	0.00E+00	1.00E+00	0.00E+00	1.00E+00	-5.13E-15	1.00E+00	0.00E+00	1.00E+00	0.00E+00	1.00E+00
ENSBTAG00000005239	-8.85E-02	1.00E+00	-3.96E-01	3.52E-01	-1.09E+00	8.42E-04	-4.76E-01	2.32E-01	3.59E-01	8.00E-01
ENSBTAG00000044975	0.00E+00	1.00E+00	0.00E+00	1.00E+00	-5.13E-15	1.00E+00	0.00E+00	1.00E+00	0.00E+00	1.00E+00
ENSBTAG00000043939	0.00E+00	1.00E+00	0.00E+00	1.00E+00	-5.13E-15	1.00E+00	0.00E+00	1.00E+00	0.00E+00	1.00E+00
ENSBTAG00000037804	2.35E-01	1.00E+00	8.60E-02	1.00E+00	-1.56E-01	1.00E+00	3.74E-01	5.94E-01	-5.59E-01	7.93E-01
ENSBTAG00000044588	0.00E+00	1.00E+00	0.00E+00	1.00E+00	-5.13E-15	1.00E+00	0.00E+00	1.00E+00	0.00E+00	1.00E+00
ENSBTAG00000018549	-1.22E+00	6.87E-01	-1.15E+00	3.42E-01	-2.27E+00	4.12E-02	-5.11E-01	7.89E-01	-4.63E-01	1.00E+00
ENSBTAG00000043739	0.00E+00	1.00E+00	0.00E+00	1.00E+00	-5.13E-15	1.00E+00	0.00E+00	1.00E+00	0.00E+00	1.00E+00

ENSBTAG00000048219	0.00E+00	1.00E+00	0.00E+00	1.00E+00	-5.13E-15	1.00E+00	0.00E+00	1.00E+00	0.00E+00	1.00E+00
ENSBTAG00000018552	1.78E-01	1.00E+00	-1.42E+00	1.38E-01	-1.99E+00	2.87E-02	1.90E-01	1.00E+00	-1.87E-01	1.00E+00
ENSBTAG00000033690	-1.02E+00	2.64E-01	-3.97E-01	6.22E-01	1.09E-01	1.00E+00	1.39E-01	1.00E+00	-5.32E-01	8.00E-01
ENSBTAG00000003472	-8.43E-01	8.54E-01	-5.38E-01	7.67E-01	2.64E-01	1.00E+00	6.86E-01	4.91E-01	-1.08E+00	7.63E-01
ENSBTAG00000045368	0.00E+00	1.00E+00	0.00E+00	1.00E+00	-5.13E-15	1.00E+00	0.00E+00	1.00E+00	0.00E+00	1.00E+00
ENSBTAG00000019274	-2.07E-01	1.00E+00	-1.22E+00	2.66E-05	-9.90E-01	7.65E-04	-2.29E-01	6.61E-01	-4.44E-01	6.01E-01
ENSBTAG00000008300	3.06E-01	9.02E-01	1.35E-01	9.84E-01	-4.56E-01	2.58E-01	-5.26E-01	1.82E-01	1.14E+00	1.00E-02
ENSBTAG00000013215	5.95E-01	4.59E-01	-1.60E+00	5.29E-05	-2.84E+00	8.83E-12	-1.92E+00	1.05E-06	4.00E-01	8.00E-01
ENSBTAG00000043119	-6.44E-02	1.00E+00	-3.17E+00	4.15E-04	-2.90E+00	3.72E-04	-4.20E+00	1.36E-05	-2.27E-01	1.00E+00
ENSBTAG00000010413	-1.73E-01	1.00E+00	-8.10E-01	6.11E-03	-1.81E+00	7.64E-11	-2.74E+00	6.50E-22	-8.15E-01	6.45E-02
ENSBTAG00000006821	0.00E+00	1.00E+00	0.00E+00	1.00E+00	-5.13E-15	1.00E+00	0.00E+00	1.00E+00	0.00E+00	1.00E+00
ENSBTAG00000010426	3.86E-02	1.00E+00	-1.21E+00	3.21E-05	-2.81E-01	5.00E-01	1.14E-01	1.00E+00	-1.92E-01	1.00E+00
ENSBTAG00000016183	-1.76E+00	3.45E-01	-2.99E+00	1.34E-02	-1.92E+00	6.17E-02	-1.28E+00	2.21E-01	-1.41E-01	1.00E+00
ENSBTAG00000026788	-1.29E+00	9.38E-01	1.42E+00	2.56E-01	-1.48E-01	1.00E+00	-6.79E-01	8.87E-01	-1.29E+00	9.02E-01
ENSBTAG00000003843	-3.05E-01	5.86E-01	-6.67E-01	1.77E-03	-6.35E-02	1.00E+00	-2.13E-01	4.94E-01	3.75E-02	1.00E+00
ENSBTAG00000003846	-7.65E-02	1.00E+00	-1.15E-01	8.81E-01	-5.87E-01	8.54E-03	-6.85E-01	2.02E-03	-7.20E-01	1.63E-02
ENSBTAG00000005596	-1.79E-02	1.00E+00	-2.86E-01	4.74E-01	-1.23E+00	8.88E-06	-1.69E+00	1.02E-09	-7.33E-01	1.17E-01
ENSBTAG00000048210	-1.70E-01	1.00E+00	-6.82E-01	2.75E-01	-1.83E+00	4.94E-04	-1.60E+00	2.62E-03	1.43E-01	1.00E+00
ENSBTAG00000007062	-5.27E-01	9.09E-01	-4.06E-01	7.13E-01	-1.26E+00	4.45E-02	-1.26E+00	4.92E-02	6.43E-01	8.00E-01
ENSBTAG00000019003	0.00E+00	1.00E+00	0.00E+00	1.00E+00	1.78E+00	4.79E-01	0.00E+00	1.00E+00	0.00E+00	1.00E+00
ENSBTAG00000044973	0.00E+00	1.00E+00	0.00E+00	1.00E+00	-5.13E-15	1.00E+00	0.00E+00	1.00E+00	0.00E+00	1.00E+00
ENSBTAG00000002485	-1.27E-01	1.00E+00	-1.39E+00	1.57E-06	-5.63E-01	8.62E-02	9.49E-02	1.00E+00	2.56E-01	9.08E-01
ENSBTAG00000044630	0.00E+00	1.00E+00	0.00E+00	1.00E+00	-5.13E-15	1.00E+00	0.00E+00	1.00E+00	0.00E+00	1.00E+00
ENSBTAG00000045124	0.00E+00	1.00E+00	0.00E+00	1.00E+00	-5.13E-15	1.00E+00	0.00E+00	1.00E+00	0.00E+00	1.00E+00
ENSBTAG00000003327	8.82E-01	1.00E+00	1.73E+00	1.95E-01	1.25E+00	4.30E-01	9.86E-01	6.23E-01	1.22E-02	1.00E+00
ENSBTAG00000038042	1.21E+00	2.69E-01	1.23E+00	6.88E-02	1.77E+00	4.07E-03	2.71E+00	5.44E-06	9.40E-01	5.98E-01
ENSBTAG00000026753	2.81E+00	3.32E-04	3.42E+00	2.81E-07	3.79E+00	6.65E-09	4.48E+00	5.91E-12	2.19E+00	3.68E-02
ENSBTAG00000010386	-4.48E-02	1.00E+00	-3.66E-01	9.35E-02	-3.59E-01	1.01E-01	-1.30E-01	7.59E-01	-3.11E-01	5.46E-01
ENSBTAG00000042880	0.00E+00	1.00E+00	0.00E+00	1.00E+00	-5.13E-15	1.00E+00	0.00E+00	1.00E+00	0.00E+00	1.00E+00
ENSBTAG00000003923	-7.54E-02	1.00E+00	1.62E-01	8.80E-01	4.88E-01	1.66E-01	-8.52E-02	1.00E+00	1.11E-01	1.00E+00
ENSBTAG00000003927	1.10E-01	1.00E+00	2.28E-02	1.00E+00	1.73E-02	1.00E+00	3.74E-01	9.24E-02	1.57E-01	9.55E-01
ENSBTAG00000003936	9.24E-02	1.00E+00	-3.10E-01	2.32E-01	-1.01E+00	8.95E-07	-3.00E-01	2.64E-01	3.50E-01	5.09E-01
ENSBTAG00000003935	0.00E+00	1.00E+00	0.00E+00	1.00E+00	-5.13E-15	1.00E+00	0.00E+00	1.00E+00	0.00E+00	1.00E+00
ENSBTAG00000003944	1.23E-01	1.00E+00	-1.48E+00	7.34E-04	-2.57E+00	6.06E-09	-2.05E+00	2.90E-06	9.96E-01	1.69E-01
ENSBTAG00000015520	2.59E-01	1.00E+00	3.64E-01	4.77E-01	1.27E+00	4.09E-04	8.87E-01	2.18E-02	8.55E-01	1.85E-01

ENSBTAG00000008362	-2.91E-01	6.06E-01	-1.34E+00	1.08E-11	-1.08E+00	3.64E-08	-3.05E-01	2.27E-01	4.26E-01	3.01E-01
ENSBTAG00000029850	0.00E+00	1.00E+00	0.00E+00	1.00E+00	-5.13E-15	1.00E+00	0.00E+00	1.00E+00	0.00E+00	1.00E+00
ENSBTAG00000018543	-1.22E-01	1.00E+00	-1.23E+00	6.80E-02	-1.93E-01	1.00E+00	9.17E-02	1.00E+00	-1.08E-01	1.00E+00
ENSBTAG00000016572	-8.59E-02	1.00E+00	2.11E-01	6.47E-01	-8.96E-02	1.00E+00	-5.28E-02	1.00E+00	-1.52E-01	1.00E+00
ENSBTAG00000003807	-4.85E-01	1.09E-01	-5.45E-01	8.48E-03	-9.71E-01	5.38E-07	-1.09E+00	2.17E-08	4.44E-02	1.00E+00
ENSBTAG00000003809	-6.14E-01	3.26E-01	-8.07E-01	1.85E-02	-2.61E+00	1.16E-15	-3.42E+00	9.62E-24	2.28E-01	1.00E+00
ENSBTAG00000026772	-4.22E-02	1.00E+00	7.01E-01	8.03E-03	1.26E+00	3.69E-07	7.17E-01	7.43E-03	8.46E-02	1.00E+00
ENSBTAG00000003813	-7.63E-02	1.00E+00	1.21E-01	9.54E-01	4.68E-01	1.33E-01	7.11E-01	1.27E-02	-6.95E-02	1.00E+00
ENSBTAG00000003817	-1.03E-01	1.00E+00	4.10E-01	6.45E-02	3.34E-01	1.54E-01	-1.99E-02	1.00E+00	-6.46E-03	1.00E+00
ENSBTAG00000003819	-5.93E-02	1.00E+00	-2.18E+00	3.28E-14	-7.23E-01	8.78E-03	1.11E-01	9.71E-01	3.54E-01	6.61E-01
ENSBTAG00000018415	1.66E-01	1.00E+00	1.47E-01	8.02E-01	1.07E-01	9.50E-01	9.11E-02	1.00E+00	6.39E-01	8.47E-02
ENSBTAG00000013489	-1.30E-01	1.00E+00	-4.88E-01	4.12E-02	-1.30E+00	9.51E-10	-1.81E+00	2.08E-17	-3.25E-01	6.01E-01
ENSBTAG00000013492	-4.12E+00	9.01E-02	-8.18E-01	6.77E-01	-4.12E+00	1.19E-02	-2.36E+00	1.08E-01	-1.41E+00	7.74E-01
ENSBTAG00000013990	-1.29E+00	9.40E-01	-4.18E-01	1.00E+00	-1.52E-01	1.00E+00	4.85E-01	9.65E-01	-1.29E+00	9.03E-01
ENSBTAG00000009217	-5.04E-01	1.00E+00	7.65E-01	7.36E-01	7.47E-01	7.20E-01	-1.45E+00	4.94E-01	3.73E-01	1.00E+00
ENSBTAG00000031052	0.00E+00	1.00E+00	3.98E+00	2.57E-02	1.78E+00	4.79E-01	3.03E+00	1.50E-01	0.00E+00	1.00E+00
ENSBTAG00000020191	5.17E-01	1.00E+00	-7.17E-01	9.45E-01	3.54E-01	1.00E+00	3.27E-01	1.00E+00	5.24E-01	1.00E+00
ENSBTAG00000014819	-1.79E-03	1.00E+00	8.20E-02	1.00E+00	-2.71E+00	1.99E-01	-1.72E-01	1.00E+00	5.07E-01	1.00E+00
ENSBTAG00000029763	-7.87E-01	1.00E+00	-2.71E+00	2.42E-01	-2.71E+00	1.99E-01	-2.71E+00	2.05E-01	-7.81E-01	1.00E+00
ENSBTAG00000026751	-4.53E-01	1.00E+00	2.79E-01	1.00E+00	-1.71E-01	1.00E+00	-6.45E-01	7.38E-01	1.94E-01	1.00E+00
ENSBTAG00000008452	-2.65E+00	2.00E-10	-5.74E+00	1.39E-35	-7.11E+00	3.45E-46	-4.06E+00	2.25E-22	3.16E-01	9.56E-01
ENSBTAG00000031041	-5.26E-02	1.00E+00	-8.48E-01	4.70E-03	-1.88E+00	5.18E-11	-2.20E+00	3.37E-14	-1.20E-01	1.00E+00
ENSBTAG00000012618	-7.12E-01	3.57E-01	-2.16E+00	8.88E-08	-2.29E+00	8.47E-09	-2.55E+00	3.40E-10	1.44E-01	1.00E+00
ENSBTAG00000012619	-7.64E-01	1.43E-02	-8.17E-01	9.43E-04	-1.43E-01	8.24E-01	2.27E-01	5.43E-01	-2.15E-01	9.05E-01
ENSBTAG00000012620	-2.84E-01	7.57E-01	-7.50E-01	1.12E-03	-9.09E-01	5.22E-05	-4.80E-01	5.99E-02	-1.13E-01	1.00E+00
ENSBTAG00000020606	-2.00E-01	9.09E-01	-1.20E-01	8.58E-01	-2.79E-01	3.10E-01	-5.29E-01	2.27E-02	-2.43E-01	8.00E-01
ENSBTAG00000020607	-4.60E-01	2.37E-01	-1.56E+00	8.50E-13	-2.21E+00	5.83E-24	-1.90E+00	2.99E-18	-3.34E-01	6.01E-01
ENSBTAG00000020608	-3.54E-01	4.08E-01	-6.31E-01	2.39E-03	-5.34E-01	1.16E-02	7.63E-02	1.00E+00	4.25E-01	2.97E-01
ENSBTAG00000020610	-3.25E-01	4.87E-01	7.03E-01	3.26E-04	1.10E+00	4.18E-09	5.44E-01	8.34E-03	4.58E-03	1.00E+00
ENSBTAG00000020611	-1.37E-01	1.00E+00	-7.94E-01	1.57E-03	-1.38E+00	1.10E-08	-1.64E+00	1.97E-11	3.15E-01	7.16E-01
ENSBTAG00000020613	-1.67E-01	9.68E-01	-5.57E-01	6.97E-03	-9.19E-01	2.34E-06	-7.23E-01	3.34E-04	-1.26E-01	1.00E+00
ENSBTAG00000030974	-8.40E-01	8.30E-02	-9.13E-01	7.79E-03	8.04E-01	2.08E-02	1.36E+00	3.59E-05	-1.27E+00	3.48E-03
ENSBTAG00000030973	-5.74E-01	8.75E-01	-1.71E+00	7.42E-03	-5.93E-01	4.79E-01	-7.67E-01	3.21E-01	-6.65E-01	8.00E-01
ENSBTAG00000013782	-1.45E-01	1.00E+00	4.44E-02	1.00E+00	-4.06E-01	1.07E-01	-3.46E-01	2.04E-01	-2.70E-01	7.77E-01
ENSBTAG00000013798	1.71E-02	1.00E+00	8.49E-01	5.95E-01	5.81E-01	8.23E-01	1.19E+00	3.23E-01	1.42E+00	5.79E-01

ENSBTAG00000036436	0.00E+00	1.00E+00	0.00E+00	1.00E+00	-5.13E-15	1.00E+00	0.00E+00	1.00E+00	0.00E+00	1.00E+00
ENSBTAG00000013799	5.12E-03	1.00E+00	-2.97E-01	3.59E-01	-8.14E-01	8.75E-04	-5.23E-01	5.29E-02	4.37E-02	1.00E+00
ENSBTAG00000010897	-3.21E+00	3.75E-01	-4.23E-01	1.00E+00	-1.43E+00	4.79E-01	-1.86E-01	1.00E+00	-3.21E+00	4.34E-01
ENSBTAG00000021514	1.90E-01	1.00E+00	6.06E-01	6.00E-02	6.55E-01	3.81E-02	7.65E-01	1.43E-02	-3.58E-01	7.76E-01
ENSBTAG00000046244	0.00E+00	1.00E+00	0.00E+00	1.00E+00	-5.13E-15	1.00E+00	0.00E+00	1.00E+00	0.00E+00	1.00E+00
ENSBTAG00000005353	2.90E-01	9.90E-01	4.24E-01	3.81E-01	-1.70E-01	9.28E-01	3.59E-01	4.97E-01	2.82E-01	9.70E-01
ENSBTAG00000046176	1.80E-02	1.00E+00	-1.46E+00	1.68E-06	-1.97E+00	8.69E-11	-1.39E+00	4.87E-06	5.61E-02	1.00E+00
ENSBTAG00000002995	6.39E-01	3.33E-01	9.36E-01	7.56E-03	1.14E+00	8.20E-04	1.17E+00	7.06E-04	4.09E-01	7.79E-01
ENSBTAG00000020537	1.32E+00	6.91E-01	5.92E+00	1.85E-26	2.54E+00	2.43E-03	1.79E+00	8.53E-02	6.71E-01	1.00E+00
ENSBTAG00000020539	2.18E-01	1.00E+00	3.92E-01	4.32E-01	1.15E+00	1.29E-03	1.88E+00	6.02E-08	6.25E-01	4.47E-01
ENSBTAG00000020540	9.27E-01	3.51E-01	3.70E-01	7.42E-01	1.08E+00	3.88E-02	1.32E+00	8.08E-03	4.26E-01	9.60E-01
ENSBTAG00000040338	-4.30E-01	5.81E-01	-1.52E+00	1.69E-07	-2.50E+00	1.35E-17	-1.58E+00	5.76E-08	-3.84E-01	7.14E-01
ENSBTAG00000009972	1.49E-01	1.00E+00	-1.73E+00	2.77E-03	-2.01E+00	3.56E-04	-2.15E+00	1.77E-04	-9.24E-03	1.00E+00
ENSBTAG00000038992	-3.08E-01	7.02E-01	-3.98E-01	1.45E-01	3.71E-01	1.78E-01	6.13E-01	1.26E-02	3.82E-02	1.00E+00
ENSBTAG00000014975	-2.51E-01	1.00E+00	-2.00E+00	3.78E-04	-4.02E-01	5.51E-01	-4.06E-01	5.55E-01	7.93E-02	1.00E+00
ENSBTAG00000037288	0.00E+00	1.00E+00	0.00E+00	1.00E+00	-5.13E-15	1.00E+00	0.00E+00	1.00E+00	0.00E+00	1.00E+00
ENSBTAG00000010030	-2.50E-01	1.00E+00	3.81E-01	8.17E-01	-1.00E+00	2.58E-01	4.20E-01	7.44E-01	-5.49E-01	9.69E-01
ENSBTAG00000042746	0.00E+00	1.00E+00	0.00E+00	1.00E+00	-5.13E-15	1.00E+00	0.00E+00	1.00E+00	0.00E+00	1.00E+00
ENSBTAG00000009950	1.22E+00	7.53E-01	4.38E-02	1.00E+00	2.41E-01	1.00E+00	1.64E+00	1.30E-01	-7.32E-01	1.00E+00
ENSBTAG00000046649	4.05E-01	1.00E+00	-6.24E-01	6.09E-01	-8.29E-02	1.00E+00	7.50E-01	3.64E-01	3.34E-01	1.00E+00
ENSBTAG00000011649	2.23E-02	1.00E+00	1.06E+00	2.59E-05	1.15E+00	3.80E-06	5.83E-01	3.94E-02	-2.98E-01	8.00E-01
ENSBTAG00000000301	8.52E-01	1.10E-01	1.16E+00	9.51E-04	1.18E+00	6.71E-04	1.33E+00	1.22E-04	8.20E-01	2.00E-01
ENSBTAG00000008431	-7.52E-01	7.36E-02	-1.81E+00	4.77E-10	-3.13E+00	1.27E-24	-3.11E+00	2.03E-24	7.58E-01	1.02E-01
ENSBTAG00000017258	-2.35E-01	1.00E+00	-3.29E-01	4.43E-01	2.10E-01	7.22E-01	7.31E-01	2.35E-02	-8.52E-03	1.00E+00
ENSBTAG00000005315	0.00E+00	1.00E+00	0.00E+00	1.00E+00	-5.13E-15	1.00E+00	0.00E+00	1.00E+00	0.00E+00	1.00E+00
ENSBTAG00000006432	1.54E+00	4.33E-01	1.25E+00	2.71E-01	-2.53E-01	1.00E+00	3.49E+00	1.62E-04	6.74E-01	1.00E+00
ENSBTAG00000042580	0.00E+00	1.00E+00	0.00E+00	1.00E+00	-5.13E-15	1.00E+00	0.00E+00	1.00E+00	0.00E+00	1.00E+00
ENSBTAG00000043213	0.00E+00	1.00E+00	0.00E+00	1.00E+00	-5.13E-15	1.00E+00	0.00E+00	1.00E+00	0.00E+00	1.00E+00
ENSBTAG00000021588	8.10E-01	1.00E+00	8.82E-01	8.52E-01	6.45E-01	9.78E-01	6.22E-01	9.94E-01	1.81E-02	1.00E+00
ENSBTAG00000027962	-1.77E-01	1.00E+00	-2.97E-01	7.08E-01	-8.25E-01	6.99E-02	-5.68E-01	2.78E-01	1.27E-01	1.00E+00
ENSBTAG00000011133	5.24E-02	1.00E+00	1.66E+00	2.70E-08	1.47E+00	7.59E-07	1.03E-01	1.00E+00	-2.00E-01	1.00E+00
ENSBTAG00000016951	1.19E-01	1.00E+00	8.70E-01	6.04E-06	9.41E-01	7.67E-07	4.33E-01	4.74E-02	4.63E-01	1.95E-01
ENSBTAG00000042664	6.01E-03	1.00E+00	1.39E+00	5.17E-01	1.81E+00	2.79E-01	-1.91E+00	4.39E-01	7.99E-01	1.00E+00
ENSBTAG00000011514	5.75E-02	1.00E+00	6.07E-01	2.54E-03	8.30E-01	1.68E-05	4.15E-01	6.13E-02	-1.97E-01	8.38E-01
ENSBTAG00000008717	-2.51E-01	1.00E+00	-4.19E-01	5.28E-01	-2.31E-01	8.76E-01	2.02E-01	9.37E-01	-7.85E-01	4.72E-01

ENSBTAG00000040156	0.00E+00	1.00E+00	0.00E+00	1.00E+00	-5.13E-15	1.00E+00	0.00E+00	1.00E+00	0.00E+00	1.00E+00
ENSBTAG00000038700	-1.91E+00	8.23E-01	7.78E-02	1.00E+00	-1.25E-01	1.00E+00	6.22E-01	9.94E-01	7.05E-03	1.00E+00
ENSBTAG00000021769	1.84E-01	8.90E-01	7.56E-01	6.42E-05	5.05E-01	1.14E-02	4.94E-01	1.57E-02	-5.68E-02	1.00E+00
ENSBTAG00000000581	5.03E-01	3.79E-01	1.08E+00	9.82E-05	1.02E-01	1.00E+00	-4.62E-01	1.80E-01	-3.35E-01	8.00E-01
ENSBTAG00000019152	6.33E-01	9.42E-01	-1.01E-01	1.00E+00	-8.51E-01	4.60E-01	3.83E-01	8.83E-01	-1.53E+00	4.82E-01
ENSBTAG00000044367	0.00E+00	1.00E+00	0.00E+00	1.00E+00	-5.13E-15	1.00E+00	0.00E+00	1.00E+00	0.00E+00	1.00E+00
ENSBTAG00000009013	2.94E-02	1.00E+00	3.15E-01	3.01E-01	9.82E-02	9.82E-01	7.37E-02	1.00E+00	-2.37E-01	8.40E-01
ENSBTAG00000044948	0.00E+00	1.00E+00	0.00E+00	1.00E+00	-5.13E-15	1.00E+00	0.00E+00	1.00E+00	0.00E+00	1.00E+00
ENSBTAG00000021308	-8.08E-02	1.00E+00	-1.79E+00	1.71E-04	-5.52E-01	3.81E-01	2.68E-01	8.44E-01	8.29E-01	4.45E-01
ENSBTAG00000023964	0.00E+00	1.00E+00	6.33E+00	7.23E-08	3.96E+00	2.25E-02	1.76E+00	4.97E-01	2.72E+00	6.01E-01
ENSBTAG00000023963	2.24E-01	1.00E+00	2.95E+00	9.75E-18	1.55E+00	6.83E-06	7.50E-01	5.62E-02	4.54E-02	1.00E+00
ENSBTAG00000021310	1.19E-01	1.00E+00	-2.33E-01	8.91E-01	-3.51E-01	6.39E-01	-1.59E-01	1.00E+00	6.55E-01	5.63E-01
ENSBTAG00000021316	2.77E-04	1.00E+00	1.75E+00	1.29E-06	3.78E-01	4.79E-01	-8.08E-01	6.95E-02	2.48E-01	1.00E+00
ENSBTAG00000021319	-8.73E-03	1.00E+00	1.73E-01	6.84E-01	8.07E-01	4.46E-04	1.03E-01	9.60E-01	-1.50E-01	1.00E+00
ENSBTAG00000021322	-1.48E-01	1.00E+00	1.32E+00	1.86E-02	1.13E+00	4.89E-02	7.31E-01	2.79E-01	-2.43E-01	1.00E+00
ENSBTAG00000021323	2.17E-02	1.00E+00	8.19E-01	1.09E-03	1.02E+00	3.16E-05	2.45E-01	5.03E-01	-7.61E-01	3.82E-02
ENSBTAG00000021325	-2.50E-01	1.00E+00	-1.28E+00	3.67E-04	-1.91E+00	5.93E-08	-1.86E+00	1.67E-07	-2.66E-02	1.00E+00
ENSBTAG00000007127	8.45E-01	2.01E-03	3.34E+00	2.30E-47	1.50E+00	1.32E-11	8.52E-01	2.73E-04	8.06E-02	1.00E+00
ENSBTAG00000021326	5.05E+00	4.18E-05	5.70E+00	4.25E-07	1.51E+00	2.35E-01	1.52E+00	2.41E-01	-6.44E-01	1.00E+00
ENSBTAG00000021327	-2.34E-01	1.00E+00	3.05E-01	1.00E+00	-7.02E-01	7.20E-01	-7.37E-01	7.00E-01	-1.41E+00	7.71E-01
ENSBTAG00000021328	-7.47E-01	1.00E+00	-1.56E+00	2.70E-01	-4.34E+00	5.77E-03	-6.57E-01	7.34E-01	-1.64E+00	6.06E-01
ENSBTAG00000010035	2.86E-02	1.00E+00	-1.93E-01	1.00E+00	4.75E-01	8.85E-01	1.12E+00	2.96E-01	1.66E-02	1.00E+00
ENSBTAG00000037640	6.09E-02	1.00E+00	3.23E-01	3.40E-01	-2.35E-02	1.00E+00	-4.55E-01	1.35E-01	1.46E-01	1.00E+00
ENSBTAG00000044908	0.00E+00	1.00E+00	0.00E+00	1.00E+00	-5.13E-15	1.00E+00	0.00E+00	1.00E+00	0.00E+00	1.00E+00
ENSBTAG00000016063	-1.55E-01	1.00E+00	-1.07E+00	6.98E-01	-1.23E+00	6.00E-01	-7.05E-01	9.21E-01	5.49E-03	1.00E+00
ENSBTAG00000042788	0.00E+00	1.00E+00	0.00E+00	1.00E+00	-5.13E-15	1.00E+00	0.00E+00	1.00E+00	0.00E+00	1.00E+00
ENSBTAG00000045470	0.00E+00	1.00E+00	0.00E+00	1.00E+00	-5.13E-15	1.00E+00	0.00E+00	1.00E+00	0.00E+00	1.00E+00
ENSBTAG00000039046	3.50E-01	9.56E-01	-6.28E-02	1.00E+00	6.21E-01	1.99E-01	8.86E-01	4.66E-02	5.01E-01	7.68E-01
ENSBTAG00000021653	1.95E-01	8.23E-01	4.73E-01	9.09E-03	2.31E-01	3.00E-01	2.95E-01	1.57E-01	6.67E-02	1.00E+00
ENSBTAG00000010338	-8.78E-02	1.00E+00	-1.36E+00	1.07E-03	-5.06E-01	3.18E-01	-5.00E-01	3.39E-01	-3.07E-01	9.77E-01
ENSBTAG00000043024	0.00E+00	1.00E+00	0.00E+00	1.00E+00	-5.13E-15	1.00E+00	0.00E+00	1.00E+00	0.00E+00	1.00E+00
ENSBTAG00000042668	0.00E+00	1.00E+00	0.00E+00	1.00E+00	-5.13E-15	1.00E+00	0.00E+00	1.00E+00	0.00E+00	1.00E+00
ENSBTAG00000010344	3.92E-03	1.00E+00	1.63E+00	1.11E-01	7.52E-01	6.27E-01	1.00E+00	4.39E-01	-1.37E-01	1.00E+00
ENSBTAG00000009664	1.45E-01	1.00E+00	1.17E+00	1.95E-04	1.45E+00	2.54E-06	9.29E-01	4.66E-03	2.87E-01	8.97E-01
ENSBTAG00000044419	1.33E+00	5.14E-01	1.51E+00	1.03E-01	1.83E+00	2.42E-02	4.43E-02	1.00E+00	1.10E+00	7.33E-01

ENSBTAG00000007280	1.82E+00	4.03E-02	3.37E+00	1.51E-09	1.58E+00	2.43E-02	1.07E+00	2.09E-01	2.66E-01	1.00E+00
ENSBTAG00000005584	-1.16E-01	1.00E+00	-1.28E-02	1.00E+00	2.10E-01	5.86E-01	2.65E-01	4.47E-01	-6.67E-02	1.00E+00
ENSBTAG00000015752	1.41E-01	1.00E+00	6.84E-01	4.52E-02	3.23E-01	4.75E-01	-1.92E-01	8.17E-01	1.51E-01	1.00E+00
ENSBTAG00000047133	0.00E+00	1.00E+00	0.00E+00	1.00E+00	-5.13E-15	1.00E+00	0.00E+00	1.00E+00	0.00E+00	1.00E+00
ENSBTAG00000037989	1.60E-01	1.00E+00	2.54E-01	9.00E-01	-5.23E-01	4.64E-01	-7.35E-01	2.45E-01	-1.01E-01	1.00E+00
ENSBTAG00000037702	2.91E-01	1.00E+00	1.10E+00	4.29E-02	4.58E-01	5.43E-01	-2.06E-01	9.93E-01	4.57E-01	8.93E-01
ENSBTAG00000038710	4.27E-01	7.86E-01	9.57E-01	1.06E-02	3.26E-01	5.48E-01	-3.25E-01	5.63E-01	-2.83E-01	9.70E-01
ENSBTAG00000040055	7.84E-02	1.00E+00	3.68E-01	7.67E-02	-8.79E-03	1.00E+00	2.36E-02	1.00E+00	-3.30E-01	4.56E-01
ENSBTAG00000017855	6.36E-01	7.88E-02	8.57E-01	5.86E-04	3.07E-01	3.50E-01	-4.47E-02	1.00E+00	-2.62E-01	8.00E-01
ENSBTAG00000043220	0.00E+00	1.00E+00	0.00E+00	1.00E+00	-5.13E-15	1.00E+00	2.53E+00	2.71E-01	0.00E+00	1.00E+00
ENSBTAG00000017431	-1.96E+00	5.76E-01	3.50E-01	1.00E+00	-2.10E+00	1.74E-01	-1.85E-01	1.00E+00	-2.80E-01	1.00E+00
ENSBTAG00000030718	1.46E+00	1.90E-01	1.85E+00	7.95E-03	-5.35E-01	6.58E-01	-1.84E+00	1.99E-02	1.22E+00	4.34E-01
ENSBTAG00000026710	9.64E-01	4.23E-03	1.11E+00	5.83E-05	4.94E-01	1.24E-01	1.08E+00	1.13E-04	4.37E-01	5.72E-01
ENSBTAG00000005119	1.26E-01	1.00E+00	4.05E-01	4.33E-02	5.74E-01	2.16E-03	7.08E-01	1.32E-04	-4.43E-01	1.81E-01
ENSBTAG00000039772	0.00E+00	1.00E+00	0.00E+00	1.00E+00	-5.13E-15	1.00E+00	0.00E+00	1.00E+00	0.00E+00	1.00E+00
ENSBTAG00000018502	1.33E-02	1.00E+00	-3.19E-01	4.43E-01	5.07E-01	1.11E-01	2.77E-01	5.02E-01	3.50E-01	7.68E-01
ENSBTAG00000044922	0.00E+00	1.00E+00	0.00E+00	1.00E+00	-5.13E-15	1.00E+00	0.00E+00	1.00E+00	0.00E+00	1.00E+00
ENSBTAG00000043107	0.00E+00	1.00E+00	0.00E+00	1.00E+00	-5.13E-15	1.00E+00	0.00E+00	1.00E+00	0.00E+00	1.00E+00
ENSBTAG00000001359	-2.10E+00	3.94E-02	-1.21E+00	1.14E-01	-1.64E+00	2.31E-02	-1.78E-01	1.00E+00	-5.26E-01	9.12E-01
ENSBTAG00000001357	-3.59E+00	2.32E-01	-3.59E+00	6.69E-02	-3.59E+00	4.55E-02	-1.06E+00	5.96E-01	-8.80E-01	1.00E+00
ENSBTAG00000016174	2.46E-01	8.23E-01	2.11E+00	4.31E-24	2.37E+00	5.79E-30	2.13E+00	1.61E-24	-9.16E-02	1.00E+00
ENSBTAG00000042435	0.00E+00	1.00E+00	0.00E+00	1.00E+00	-5.13E-15	1.00E+00	0.00E+00	1.00E+00	0.00E+00	1.00E+00
ENSBTAG00000043297	0.00E+00	1.00E+00	0.00E+00	1.00E+00	-5.13E-15	1.00E+00	0.00E+00	1.00E+00	0.00E+00	1.00E+00
ENSBTAG00000017684	5.62E-01	1.00E+00	1.35E+00	2.19E-01	9.30E-01	4.79E-01	1.19E+00	3.07E-01	1.51E+00	4.72E-01
ENSBTAG00000047784	0.00E+00	1.00E+00	0.00E+00	1.00E+00	-5.13E-15	1.00E+00	0.00E+00	1.00E+00	0.00E+00	1.00E+00
ENSBTAG00000002549	1.17E-01	1.00E+00	5.94E-01	6.60E-03	7.31E-01	5.60E-04	6.57E-01	2.69E-03	-5.91E-01	7.31E-02
ENSBTAG00000019480	1.49E-01	1.00E+00	-6.55E-01	1.04E-02	-6.77E-01	6.97E-03	-4.04E-01	1.60E-01	2.09E-01	9.07E-01
ENSBTAG00000014988	0.00E+00	1.00E+00	0.00E+00	1.00E+00	-5.13E-15	1.00E+00	0.00E+00	1.00E+00	0.00E+00	1.00E+00
ENSBTAG00000005207	-2.41E-01	8.23E-01	1.66E-02	1.00E+00	1.36E-01	7.66E-01	-1.61E-01	6.73E-01	8.59E-03	1.00E+00
ENSBTAG00000003253	1.48E+00	9.82E-02	-6.81E-01	4.41E-01	-1.96E+00	3.39E-03	-5.32E-01	5.91E-01	-1.44E+00	2.36E-01
ENSBTAG00000043514	0.00E+00	1.00E+00	1.98E+00	4.43E-01	-5.13E-15	1.00E+00	0.00E+00	1.00E+00	1.91E+00	8.00E-01
ENSBTAG00000011964	-1.19E-01	1.00E+00	-3.18E-01	3.42E-01	2.68E-01	4.43E-01	-4.32E-02	1.00E+00	8.40E-03	1.00E+00
ENSBTAG00000016748	0.00E+00	1.00E+00	1.98E+00	4.43E-01	1.78E+00	4.79E-01	1.76E+00	4.97E-01	0.00E+00	1.00E+00
ENSBTAG00000039346	0.00E+00	1.00E+00	0.00E+00	1.00E+00	-5.13E-15	1.00E+00	2.53E+00	2.71E-01	0.00E+00	1.00E+00
ENSBTAG00000004519	0.00E+00	1.00E+00	0.00E+00	1.00E+00	1.78E+00	4.79E-01	0.00E+00	1.00E+00	0.00E+00	1.00E+00

ENSBTAG00000012363	0.00E+00	1.00E+00	0.00E+00	1.00E+00	1.78E+00	4.79E-01	1.76E+00	4.97E-01	0.00E+00	1.00E+00
ENSBTAG00000021050	3.81E+00	8.91E-18	5.09E+00	8.44E-31	7.60E+00	3.30E-57	7.97E+00	3.55E-61	7.25E+00	3.86E-52
ENSBTAG00000011390	1.98E-01	1.00E+00	1.59E-01	1.00E+00	-8.29E-01	3.20E-01	-8.67E-01	3.01E-01	-4.49E-01	1.00E+00
ENSBTAG00000045943	1.32E+00	9.28E-01	7.78E-02	1.00E+00	-1.30E-01	1.00E+00	1.12E+00	6.68E-01	7.05E-03	1.00E+00
ENSBTAG00000010598	-8.10E-02	1.00E+00	7.65E-01	1.58E-03	1.13E+00	8.23E-07	4.65E-01	8.93E-02	2.20E-01	8.97E-01
ENSBTAG00000045513	7.53E-02	1.00E+00	-5.09E-01	2.90E-01	-5.60E-01	2.10E-01	-4.69E-01	3.35E-01	-2.44E-01	1.00E+00
ENSBTAG00000004603	4.24E-01	4.26E-01	2.42E+00	5.43E-24	2.00E+00	2.88E-17	1.04E+00	2.24E-05	-5.47E-02	1.00E+00
ENSBTAG00000044093	-8.48E-01	8.23E-01	-1.70E+00	7.47E-02	-3.60E-01	8.79E-01	-5.38E+00	4.97E-05	-1.02E+00	6.95E-01
ENSBTAG00000012629	-6.29E-01	7.22E-02	-2.60E+00	1.99E-25	-3.04E+00	2.07E-33	-3.23E+00	1.51E-36	1.47E-01	1.00E+00
ENSBTAG00000020671	-3.16E-01	9.32E-01	-1.70E+00	1.36E-05	2.64E-01	6.52E-01	1.61E+00	3.75E-07	4.72E-01	6.47E-01
ENSBTAG00000020548	-2.67E-02	1.00E+00	-1.10E+00	1.31E-01	-7.00E-02	1.00E+00	-6.53E-01	4.27E-01	1.83E-01	1.00E+00
ENSBTAG00000017605	-8.27E-02	1.00E+00	2.14E-01	5.17E-01	6.70E-02	1.00E+00	-8.98E-02	9.98E-01	1.07E-01	1.00E+00
ENSBTAG00000044284	-6.31E-01	1.00E+00	1.38E+00	7.27E-02	-4.14E-03	1.00E+00	4.69E-01	8.05E-01	4.85E-01	1.00E+00
ENSBTAG00000008330	1.53E+00	6.50E-02	3.85E+00	1.85E-10	2.02E+00	6.18E-04	1.20E+00	6.30E-02	3.33E-01	1.00E+00
ENSBTAG00000008331	-2.07E+00	1.70E-01	-1.07E+00	3.12E-01	-1.11E+00	2.55E-01	-1.15E+00	2.49E-01	-9.68E-01	7.77E-01
ENSBTAG00000008336	1.64E+00	4.36E-01	1.41E+00	2.61E-01	-6.61E-01	8.94E-01	-1.45E+00	4.95E-01	1.13E+00	8.00E-01
ENSBTAG00000002184	-2.61E+00	2.08E-01	-4.52E+00	6.00E-03	-1.47E+00	2.27E-01	-8.32E-01	5.55E-01	-1.69E-01	1.00E+00
ENSBTAG00000018067	-3.61E-01	6.49E-01	-1.92E+00	1.12E-13	-2.32E+00	4.28E-19	-1.43E+00	3.22E-08	-6.28E-02	1.00E+00
ENSBTAG00000018065	-4.83E-02	1.00E+00	1.86E+00	1.83E-18	1.80E+00	1.26E-17	1.30E+00	1.27E-09	-3.05E-01	6.61E-01
ENSBTAG00000018062	-1.04E+00	3.85E-02	-3.32E+00	3.25E-17	-7.16E-01	8.02E-02	6.65E-01	1.15E-01	-6.10E-02	1.00E+00
ENSBTAG00000026684	2.48E-01	1.00E+00	1.67E+00	7.96E-09	1.15E+00	1.27E-04	5.75E-01	1.07E-01	-1.41E-01	1.00E+00
ENSBTAG00000005904	7.31E-02	1.00E+00	8.06E-01	6.90E-05	7.58E-01	1.94E-04	6.08E-01	4.36E-03	-1.02E-01	1.00E+00
ENSBTAG00000027159	2.33E-01	8.23E-01	2.62E-01	3.42E-01	2.64E-01	3.33E-01	1.46E-01	7.37E-01	1.53E-01	1.00E+00
ENSBTAG00000018199	-2.33E-01	1.00E+00	9.42E-01	1.41E-02	8.52E-01	2.87E-02	5.72E-01	2.05E-01	-5.64E-01	6.27E-01
ENSBTAG00000046044	0.00E+00	1.00E+00	0.00E+00	1.00E+00	-5.13E-15	1.00E+00	2.54E+00	2.71E-01	0.00E+00	1.00E+00
ENSBTAG00000020655	-7.17E-02	1.00E+00	-2.95E-01	3.34E-01	-4.00E-01	1.39E-01	-2.99E-01	3.33E-01	1.92E-01	9.45E-01
ENSBTAG00000020652	-6.53E-01	3.10E-01	-5.07E-01	2.16E-01	-1.46E+00	2.18E-05	-2.09E+00	3.66E-09	-2.32E-01	1.00E+00
ENSBTAG00000037902	-5.84E-01	4.07E-01	-9.57E-01	6.75E-03	-2.02E+00	5.37E-09	-2.17E+00	6.03E-10	-3.67E-01	8.00E-01
ENSBTAG00000046862	1.32E+00	9.04E-03	1.38E+00	6.90E-04	5.87E-01	2.35E-01	5.78E-01	2.59E-01	-1.96E-01	1.00E+00
ENSBTAG00000012698	-1.27E-01	1.00E+00	-8.66E-01	5.53E-04	-2.47E-01	4.83E-01	3.60E-01	2.49E-01	1.96E-01	9.69E-01
ENSBTAG00000012695	2.41E-01	8.85E-01	-5.55E-01	4.72E-02	-8.55E-01	9.45E-04	-9.93E-01	1.30E-04	-6.48E-01	1.39E-01
ENSBTAG00000002027	-3.92E-01	9.21E-01	-2.00E-01	9.41E-01	-4.37E-01	4.76E-01	-5.25E-01	3.65E-01	-4.69E-01	8.00E-01
ENSBTAG00000014390	-1.26E-01	1.00E+00	-9.21E-01	4.13E-04	-1.59E+00	3.45E-10	-1.77E+00	4.53E-12	-2.69E-01	8.00E-01
ENSBTAG00000014388	1.38E-01	1.00E+00	6.50E-01	3.83E-04	6.92E-01	1.37E-04	6.42E-01	5.56E-04	-3.04E-01	5.15E-01
ENSBTAG00000009163	-2.76E-01	8.23E-01	-1.69E+00	5.12E-12	-1.23E+00	2.39E-07	-8.31E-01	8.26E-04	-1.45E-01	1.00E+00

ENSBTAG00000026696	2.51E-01	1.00E+00	2.23E-01	1.00E+00	4.36E-01	7.66E-01	-1.03E+00	3.40E-01	7.91E-01	7.79E-01
ENSBTAG00000015103	-1.06E+00	1.51E-02	-1.57E+00	9.26E-06	-7.95E-01	2.27E-02	-1.73E-01	8.69E-01	-4.04E-01	7.63E-01
ENSBTAG00000015104	-4.13E-02	1.00E+00	-8.05E-01	2.65E-03	-1.82E+00	3.46E-12	-1.32E+00	3.81E-07	-1.81E-01	1.00E+00
ENSBTAG00000027930	1.83E-02	1.00E+00	1.29E-01	9.44E-01	3.95E-01	2.47E-01	5.61E-01	7.22E-02	-4.64E-01	5.01E-01
ENSBTAG00000016954	7.74E-03	1.00E+00	-1.91E-01	5.84E-01	3.42E-01	1.83E-01	2.67E-01	3.61E-01	3.35E-01	5.85E-01
ENSBTAG00000012497	-1.34E-01	1.00E+00	-2.07E-01	4.25E-01	2.69E-01	2.31E-01	3.40E-01	1.08E-01	-1.70E-01	8.86E-01
ENSBTAG00000040518	1.15E+00	6.58E-05	3.70E+00	1.49E-43	3.50E+00	4.97E-40	2.61E+00	3.13E-24	-6.24E-01	1.94E-01
ENSBTAG00000039121	1.86E-01	9.31E-01	-6.41E-02	1.00E+00	-2.80E-01	2.81E-01	-7.97E-02	1.00E+00	6.31E-02	1.00E+00
ENSBTAG00000015715	-2.59E-01	1.00E+00	-1.41E+00	2.10E-01	-1.12E+00	3.10E-01	-9.49E-01	4.27E-01	1.07E+00	5.80E-01
ENSBTAG00000010657	-3.31E-01	1.00E+00	-1.01E+00	4.46E-01	-7.36E-01	5.96E-01	-9.99E-01	4.39E-01	-1.09E+00	8.00E-01
ENSBTAG00000010050	-6.56E-03	1.00E+00	-4.87E-01	3.85E-01	-6.43E-01	1.87E-01	-6.59E-01	1.81E-01	7.71E-01	3.24E-01
ENSBTAG00000010378	-1.88E-01	1.00E+00	-7.98E-01	1.35E-03	-6.25E-01	1.48E-02	-4.96E-01	7.21E-02	-1.05E-02	1.00E+00
ENSBTAG00000045347	0.00E+00	1.00E+00	0.00E+00	1.00E+00	-5.13E-15	1.00E+00	0.00E+00	1.00E+00	0.00E+00	1.00E+00
ENSBTAG00000010366	-2.49E-01	1.00E+00	-1.40E+00	2.54E-02	-2.64E+00	5.19E-05	-1.29E+00	3.29E-02	3.41E-01	1.00E+00
ENSBTAG00000013235	1.11E-01	1.00E+00	4.97E-01	6.14E-02	-8.28E-02	1.00E+00	-7.81E-01	1.78E-03	-3.88E-02	1.00E+00
ENSBTAG00000016819	5.50E-01	2.76E-01	1.34E+00	3.20E-07	1.44E+00	2.78E-08	6.67E-01	2.45E-02	5.48E-01	3.54E-01
ENSBTAG00000039994	0.00E+00	1.00E+00	0.00E+00	1.00E+00	-5.13E-15	1.00E+00	0.00E+00	1.00E+00	0.00E+00	1.00E+00
ENSBTAG00000016818	-8.59E-02	1.00E+00	-4.20E-01	7.54E-02	-5.12E-01	2.26E-02	-5.78E-01	9.86E-03	9.31E-02	1.00E+00
ENSBTAG00000011582	0.00E+00	1.00E+00	0.00E+00	1.00E+00	-5.13E-15	1.00E+00	0.00E+00	1.00E+00	0.00E+00	1.00E+00
ENSBTAG00000035081	0.00E+00	1.00E+00	0.00E+00	1.00E+00	-5.13E-15	1.00E+00	0.00E+00	1.00E+00	0.00E+00	1.00E+00
ENSBTAG00000016817	3.33E-01	5.07E-01	1.56E+00	2.67E-15	1.06E+00	8.64E-08	3.82E-01	1.11E-01	-2.25E-03	1.00E+00
ENSBTAG00000020159	1.91E+00	8.23E-01	3.30E+00	1.02E-01	3.43E+00	7.38E-02	0.00E+00	1.00E+00	0.00E+00	1.00E+00
ENSBTAG00000018979	-2.19E-01	7.31E-01	5.60E-02	1.00E+00	-2.23E-01	3.09E-01	-3.99E-01	3.23E-02	-3.08E-01	4.34E-01
ENSBTAG00000043864	0.00E+00	1.00E+00	0.00E+00	1.00E+00	-5.13E-15	1.00E+00	0.00E+00	1.00E+00	0.00E+00	1.00E+00
ENSBTAG00000042860	0.00E+00	1.00E+00	0.00E+00	1.00E+00	-5.13E-15	1.00E+00	0.00E+00	1.00E+00	0.00E+00	1.00E+00
ENSBTAG00000042142	0.00E+00	1.00E+00	0.00E+00	1.00E+00	-5.13E-15	1.00E+00	0.00E+00	1.00E+00	0.00E+00	1.00E+00
ENSBTAG00000042775	0.00E+00	1.00E+00	0.00E+00	1.00E+00	-5.13E-15	1.00E+00	0.00E+00	1.00E+00	0.00E+00	1.00E+00
ENSBTAG00000026676	-2.86E-01	8.52E-01	5.50E-01	8.01E-02	3.32E-01	3.83E-01	1.58E-01	8.57E-01	7.55E-02	1.00E+00
ENSBTAG00000005477	-5.17E-02	1.00E+00	-5.40E-01	4.12E-02	-3.89E-01	1.81E-01	-4.14E-01	1.55E-01	-4.54E-01	4.20E-01
ENSBTAG00000003479	-1.31E+00	9.32E-01	-3.22E+00	1.25E-01	7.52E-01	7.20E-01	7.14E-01	7.63E-01	-5.15E-01	1.00E+00
ENSBTAG00000012848	-1.21E-01	1.00E+00	-1.24E-01	1.00E+00	-1.65E-01	9.20E-01	-5.98E-01	1.39E-01	-1.90E-01	1.00E+00
ENSBTAG00000017253	-4.46E-02	1.00E+00	3.52E-01	2.36E-01	1.72E-01	7.13E-01	-7.59E-02	1.00E+00	-7.96E-02	1.00E+00
ENSBTAG00000043464	0.00E+00	1.00E+00	0.00E+00	1.00E+00	-5.13E-15	1.00E+00	0.00E+00	1.00E+00	0.00E+00	1.00E+00
ENSBTAG00000013309	6.48E-02	1.00E+00	-1.31E-01	7.25E-01	2.14E-01	3.98E-01	-4.45E-01	2.65E-02	-7.52E-02	1.00E+00
ENSBTAG00000047524	-6.92E-01	6.75E-01	-2.15E+00	5.97E-04	-6.54E-01	3.08E-01	-6.35E-01	3.38E-01	-3.00E-02	1.00E+00

ENSBTAG00000006667	1.97E-01	1.00E+00	9.22E-01	1.44E-03	7.01E-01	2.01E-02	5.05E-01	1.32E-01	3.40E-01	7.90E-01
ENSBTAG00000003202	-1.18E+00	8.68E-01	-3.89E+00	3.61E-02	-4.68E-01	9.54E-01	9.87E-01	4.13E-01	-6.65E-01	1.00E+00
ENSBTAG00000015771	2.34E-01	8.20E-01	1.39E+00	3.33E-13	1.36E+00	1.11E-12	7.74E-01	1.18E-04	-9.78E-02	1.00E+00
ENSBTAG00000043380	0.00E+00	1.00E+00	0.00E+00	1.00E+00	-5.13E-15	1.00E+00	0.00E+00	1.00E+00	1.91E+00	8.00E-01
ENSBTAG00000001160	4.74E-01	2.24E-01	1.27E+00	2.90E-09	1.15E+00	7.52E-08	3.20E-01	2.67E-01	3.96E-01	4.60E-01
ENSBTAG00000042723	-3.88E+00	1.44E-01	-1.89E+00	2.57E-01	-1.32E+00	4.17E-01	-2.12E+00	1.79E-01	1.68E-02	1.00E+00
ENSBTAG00000026660	5.31E-02	1.00E+00	5.66E-01	1.83E-02	9.12E-01	3.60E-05	5.47E-01	2.54E-02	-6.35E-02	1.00E+00
ENSBTAG00000021525	-1.31E+00	7.05E-01	2.06E+00	1.26E-03	2.05E+00	9.25E-04	1.35E+00	7.62E-02	-6.37E-01	1.00E+00
ENSBTAG00000048228	6.43E-01	3.58E-02	1.95E+00	8.68E-20	2.13E+00	1.53E-23	1.51E+00	2.03E-12	-7.68E-01	2.10E-02
ENSBTAG00000042986	0.00E+00	1.00E+00	0.00E+00	1.00E+00	-5.13E-15	1.00E+00	0.00E+00	1.00E+00	0.00E+00	1.00E+00
ENSBTAG00000042871	0.00E+00	1.00E+00	0.00E+00	1.00E+00	-5.13E-15	1.00E+00	0.00E+00	1.00E+00	0.00E+00	1.00E+00
ENSBTAG00000043315	0.00E+00	1.00E+00	0.00E+00	1.00E+00	-5.13E-15	1.00E+00	0.00E+00	1.00E+00	0.00E+00	1.00E+00
ENSBTAG00000042553	0.00E+00	1.00E+00	0.00E+00	1.00E+00	-5.13E-15	1.00E+00	0.00E+00	1.00E+00	0.00E+00	1.00E+00
ENSBTAG00000010393	1.80E-02	1.00E+00	9.89E-02	9.56E-01	4.15E-01	9.36E-02	1.60E-01	7.14E-01	1.90E-02	1.00E+00
ENSBTAG00000003788	-4.68E-01	6.09E-01	-1.84E-01	8.52E-01	1.12E+00	1.41E-04	1.20E+00	5.09E-05	8.11E-02	1.00E+00
ENSBTAG00000046050	-8.85E-01	3.74E-01	9.33E-02	1.00E+00	7.55E-01	1.05E-01	-2.92E-01	7.77E-01	-1.92E+00	1.10E-02
ENSBTAG00000046681	-4.75E-01	8.25E-01	6.84E-01	1.54E-01	1.20E+00	2.17E-03	8.16E-01	6.77E-02	-6.80E-01	6.13E-01
ENSBTAG00000045668	-5.65E-01	8.89E-01	7.09E-01	2.85E-01	1.61E+00	8.53E-04	5.88E-01	4.25E-01	-4.76E-01	9.55E-01
ENSBTAG00000002727	-4.04E-02	1.00E+00	-1.13E+00	5.45E-09	-1.23E+00	1.41E-10	-1.36E+00	1.41E-12	2.33E-01	7.95E-01
ENSBTAG00000003676	-3.50E-01	1.00E+00	2.99E-01	9.45E-01	9.55E-01	1.63E-01	1.08E+00	1.03E-01	-8.57E-01	8.00E-01
ENSBTAG00000040210	-1.80E-01	1.00E+00	1.44E-01	1.00E+00	3.41E-01	6.33E-01	3.37E-01	6.54E-01	-2.65E-01	1.00E+00
ENSBTAG00000045032	0.00E+00	1.00E+00	0.00E+00	1.00E+00	-5.13E-15	1.00E+00	0.00E+00	1.00E+00	0.00E+00	1.00E+00
ENSBTAG00000002678	-1.87E-01	1.00E+00	-4.06E-01	2.02E-01	-3.11E-01	3.73E-01	-3.92E-01	2.26E-01	-2.91E-01	8.00E-01
ENSBTAG00000002363	1.49E+00	2.66E-02	2.28E+00	6.74E-06	6.39E-03	1.00E+00	1.88E-01	1.00E+00	3.38E-01	1.00E+00
ENSBTAG00000006342	-1.83E-01	1.00E+00	-7.16E-01	3.79E-03	-1.32E+00	1.53E-08	-1.18E+00	6.26E-07	-2.74E-01	8.00E-01
ENSBTAG00000018385	4.12E-02	1.00E+00	-5.51E-02	1.00E+00	-3.44E-02	1.00E+00	-2.40E-01	4.93E-01	7.72E-02	1.00E+00
ENSBTAG00000027051	-2.93E-01	8.95E-01	-1.31E-03	1.00E+00	4.67E-01	2.04E-01	5.01E-01	1.74E-01	-3.36E-01	8.00E-01
ENSBTAG00000009937	9.69E-03	1.00E+00	1.01E+00	5.17E-01	-6.61E-01	8.92E-01	-1.82E-01	1.00E+00	-1.30E+00	9.00E-01
ENSBTAG00000043989	-3.78E-01	6.82E-01	-9.93E-02	1.00E+00	-1.95E-01	7.28E-01	6.01E-02	1.00E+00	2.97E-01	8.00E-01
ENSBTAG00000008800	-4.96E-01	4.39E-01	-2.18E+00	2.19E-13	-2.00E+00	7.56E-12	-6.48E-01	4.48E-02	8.34E-02	1.00E+00
ENSBTAG00000012997	2.61E-01	9.19E-01	-1.15E+00	5.44E-05	-2.96E+00	1.03E-24	-2.98E+00	7.30E-25	1.68E-01	1.00E+00
ENSBTAG00000006225	1.17E-01	1.00E+00	2.01E-01	6.51E-01	1.77E-01	7.23E-01	1.19E-01	9.38E-01	-3.79E-01	6.01E-01
ENSBTAG00000006223	1.44E+00	8.41E-09	8.35E-01	7.43E-04	6.30E-01	1.47E-02	3.32E-02	1.00E+00	-1.28E-03	1.00E+00
ENSBTAG00000003867	1.91E+00	8.23E-01	2.79E+00	2.10E-01	2.55E+00	2.54E-01	1.76E+00	4.97E-01	0.00E+00	1.00E+00
ENSBTAG00000003866	1.80E-02	1.00E+00	7.01E-01	1.40E-03	8.68E-01	4.56E-05	7.10E-01	1.38E-03	4.26E-02	1.00E+00

ENSBTAG00000044441	0.00E+00	1.00E+00	0.00E+00	1.00E+00	-5.13E-15	1.00E+00	0.00E+00	1.00E+00	0.00E+00	1.00E+00
ENSBTAG00000001192	-2.13E-02	1.00E+00	-1.24E-01	8.51E-01	-3.56E-02	1.00E+00	2.15E-03	1.00E+00	-1.98E-01	8.93E-01
ENSBTAG00000019614	-5.86E-01	3.55E-01	-6.65E-01	5.83E-02	1.99E-01	7.72E-01	-2.42E-01	6.68E-01	2.27E-01	1.00E+00
ENSBTAG00000007554	-1.41E-01	1.00E+00	-2.03E-01	1.00E+00	-1.09E+00	7.57E-01	-1.41E+00	6.10E-01	-1.79E+00	8.00E-01
ENSBTAG00000011784	2.00E+00	1.00E-12	2.00E+00	1.15E-13	9.22E-01	1.16E-03	5.55E-01	8.59E-02	-1.84E-02	1.00E+00
ENSBTAG00000037456	-7.69E-01	2.12E-02	-3.43E+00	3.65E-34	-2.53E+00	1.88E-22	-1.19E+00	2.70E-06	-3.22E-01	7.46E-01
ENSBTAG00000018374	2.53E-01	7.69E-01	6.34E-01	2.51E-03	5.95E-01	4.86E-03	5.82E-01	7.10E-03	1.19E-01	1.00E+00
ENSBTAG00000043205	0.00E+00	1.00E+00	0.00E+00	1.00E+00	-5.13E-15	1.00E+00	0.00E+00	1.00E+00	0.00E+00	1.00E+00
ENSBTAG00000002505	0.00E+00	1.00E+00	2.79E+00	2.10E-01	2.55E+00	2.54E-01	1.76E+00	4.97E-01	0.00E+00	1.00E+00
ENSBTAG00000019728	-8.08E-03	1.00E+00	-3.89E+00	3.57E-02	-1.34E+00	4.11E-01	-1.36E+00	4.13E-01	2.47E-01	1.00E+00
ENSBTAG00000010254	6.37E-01	1.32E-01	1.95E-01	7.24E-01	4.75E-02	1.00E+00	-1.75E-01	7.96E-01	4.97E-01	4.34E-01
ENSBTAG00000010253	-8.87E-01	1.93E-02	-1.78E+00	4.46E-09	-2.59E+00	8.89E-17	-1.46E+00	6.14E-07	3.68E-02	1.00E+00
ENSBTAG00000010249	2.51E-01	8.23E-01	5.06E-01	3.85E-02	7.63E-01	7.48E-04	9.69E-01	1.43E-05	1.38E-01	1.00E+00
ENSBTAG00000008766	4.04E-01	4.08E-01	1.05E+00	2.46E-06	4.15E-01	1.15E-01	6.61E-01	5.74E-03	2.37E-01	8.22E-01
ENSBTAG00000018413	-1.64E+00	4.90E-04	-2.90E+00	3.08E-10	-2.79E+00	1.60E-10	-1.60E+00	8.15E-05	-3.99E-01	8.00E-01
ENSBTAG00000019252	-7.90E-01	3.74E-02	-3.27E+00	2.14E-25	-3.55E+00	6.22E-30	-2.77E+00	5.26E-21	1.17E-01	1.00E+00
ENSBTAG00000004416	1.22E-01	1.00E+00	7.86E-01	1.97E-04	3.26E-01	2.00E-01	2.35E-01	4.39E-01	-8.74E-03	1.00E+00
ENSBTAG00000014848	-3.20E+00	1.95E-13	-4.25E+00	1.36E-21	-1.20E+00	3.43E-03	-3.70E-01	5.45E-01	2.34E-01	1.00E+00
ENSBTAG00000014849	-7.13E-01	2.89E-02	-4.86E-02	1.00E+00	3.42E-01	2.41E-01	5.43E-01	3.59E-02	-1.04E-01	1.00E+00
ENSBTAG00000014850	2.02E-01	9.67E-01	1.44E+00	1.19E-10	1.43E+00	1.18E-10	9.61E-01	3.74E-05	-2.17E-01	9.00E-01
ENSBTAG00000009223	-3.66E-01	1.00E+00	9.17E-02	1.00E+00	5.70E-01	8.27E-01	1.12E-01	1.00E+00	2.52E+00	2.21E-02
ENSBTAG00000046672	-1.17E-01	1.00E+00	2.72E-01	6.22E-01	9.20E-01	6.92E-03	5.17E-01	2.01E-01	2.26E-02	1.00E+00
ENSBTAG00000009796	-2.85E-01	6.80E-01	-1.65E+00	1.88E-14	-8.30E-01	9.23E-05	-8.45E-01	8.57E-05	3.69E-01	4.54E-01
ENSBTAG00000001024	-2.40E-01	8.23E-01	-6.19E-01	7.48E-03	-1.78E-01	6.40E-01	6.92E-02	1.00E+00	2.67E-01	7.82E-01
ENSBTAG00000047382	1.26E+00	5.32E-01	9.62E-02	1.00E+00	6.73E-01	6.40E-01	1.35E+00	1.41E-01	-2.36E-01	1.00E+00
ENSBTAG00000014447	-7.06E-01	2.21E-02	-1.39E+00	2.02E-09	-8.86E-01	1.50E-04	-7.69E-01	1.44E-03	1.13E-01	1.00E+00
ENSBTAG00000038104	4.19E-02	1.00E+00	-4.80E-01	8.19E-02	-6.37E-01	1.38E-02	-2.29E-01	5.46E-01	-3.20E-01	7.23E-01
ENSBTAG00000003235	5.19E-01	3.49E-01	3.00E+00	8.03E-29	2.27E+00	9.55E-18	1.79E+00	2.06E-11	3.99E-01	6.26E-01
ENSBTAG00000040497	6.62E-03	1.00E+00	-1.91E+00	4.69E-01	6.44E-01	9.78E-01	-1.91E+00	4.39E-01	-1.91E+00	8.00E-01
ENSBTAG00000009631	-3.32E-01	1.00E+00	-9.52E-01	2.35E-01	-1.17E+00	1.15E-01	-1.48E+00	4.17E-02	2.40E-01	1.00E+00
ENSBTAG00000005629	-4.92E-01	5.47E-01	-3.99E+00	7.68E-33	-5.72E+00	2.42E-55	-2.38E+00	3.79E-14	3.34E-01	8.00E-01
ENSBTAG00000005628	-1.39E-01	1.00E+00	-1.24E+00	2.45E-04	-1.24E+00	2.04E-04	-1.16E+00	6.68E-04	-9.37E-01	7.29E-02
ENSBTAG00000009581	3.82E-01	6.39E-01	-4.53E-01	1.83E-01	9.51E-02	1.00E+00	-8.00E-02	1.00E+00	5.21E-01	3.96E-01
ENSBTAG00000009580	7.70E-02	1.00E+00	-4.87E-01	9.37E-02	-3.60E-01	2.59E-01	1.07E-01	9.85E-01	4.66E-01	4.34E-01
ENSBTAG00000009579	-6.68E-01	6.81E-02	-1.48E+00	8.54E-09	-3.60E-01	2.51E-01	1.55E-01	8.05E-01	3.98E-01	5.55E-01

ENSBTAG00000039276	3.22E+00	3.75E-01	3.68E+00	5.08E-02	-5.13E-15	1.00E+00	0.00E+00	1.00E+00	2.71E+00	6.01E-01
ENSBTAG00000026638	-1.12E+00	2.02E-01	-5.63E-01	4.11E-01	-2.13E+00	1.98E-04	-1.69E+00	2.61E-03	6.32E-01	6.04E-01
ENSBTAG00000047148	-1.96E-01	1.00E+00	3.72E-01	5.88E-01	-7.71E-01	1.56E-01	-1.44E+00	5.40E-03	7.74E-01	4.19E-01
ENSBTAG00000019399	-6.10E-02	1.00E+00	8.79E-02	1.00E+00	-1.07E+00	1.30E-02	-1.29E+00	3.05E-03	5.62E-01	6.01E-01
ENSBTAG00000009562	9.72E-02	1.00E+00	1.42E+00	2.33E-07	1.95E+00	3.72E-13	1.53E+00	2.62E-08	-3.79E-01	7.35E-01
ENSBTAG00000013131	-3.16E-01	1.00E+00	-8.75E-01	8.97E-02	9.82E-03	1.00E+00	-7.31E-01	1.63E-01	-1.28E+00	9.37E-02
ENSBTAG00000001513	-4.53E-01	6.88E-01	-3.16E-01	5.38E-01	5.51E-01	1.39E-01	1.18E+00	1.81E-04	4.26E-01	7.12E-01
ENSBTAG00000005085	-2.15E+00	6.17E-01	2.73E+00	2.04E-02	-3.39E-01	1.00E+00	2.76E+00	2.02E-02	4.58E-01	1.00E+00
ENSBTAG00000006355	-3.64E-01	1.00E+00	4.49E+00	8.16E-11	1.09E+00	2.96E-01	2.58E-01	1.00E+00	5.05E-01	1.00E+00
ENSBTAG00000006349	-5.91E-01	5.39E-01	7.92E-02	1.00E+00	-1.72E+00	4.49E-06	-2.26E+00	2.10E-09	-5.38E-02	1.00E+00
ENSBTAG00000005105	1.08E-02	1.00E+00	-2.11E-01	6.68E-01	-4.21E-01	2.02E-01	-4.43E-01	1.83E-01	-5.65E-02	1.00E+00
ENSBTAG00000048095	0.00E+00	1.00E+00	0.00E+00	1.00E+00	-5.13E-15	1.00E+00	0.00E+00	1.00E+00	0.00E+00	1.00E+00
ENSBTAG00000013761	2.89E-01	8.91E-01	-8.04E-02	1.00E+00	-8.14E-01	1.34E-02	-2.06E-01	7.59E-01	-4.43E-01	6.29E-01
ENSBTAG00000021787	1.32E-01	1.00E+00	1.90E+00	9.61E-04	1.33E+00	3.79E-02	-7.69E-02	1.00E+00	4.61E-01	1.00E+00
ENSBTAG00000021786	2.54E-01	1.00E+00	3.74E-01	4.74E-01	2.79E-02	1.00E+00	1.74E-02	1.00E+00	-3.31E-01	9.14E-01
ENSBTAG00000021781	3.06E-01	6.81E-01	1.13E+00	3.24E-07	6.09E-01	1.03E-02	1.55E-01	7.55E-01	-2.16E-01	8.70E-01
ENSBTAG00000021778	2.09E-01	8.70E-01	3.07E-01	2.42E-01	7.41E-01	4.22E-04	8.85E-01	2.32E-05	2.89E-01	6.74E-01
ENSBTAG00000003069	2.16E-01	1.00E+00	-7.89E-01	3.52E-02	5.08E-02	1.00E+00	1.64E-01	9.33E-01	-3.75E-02	1.00E+00
ENSBTAG00000001050	-2.69E-01	8.62E-01	-1.85E+00	1.08E-11	-3.57E-03	1.00E+00	3.68E-01	2.84E-01	3.49E-01	7.18E-01
ENSBTAG00000002988	-1.85E-01	8.37E-01	-1.11E+00	2.35E-10	-7.97E-01	6.01E-06	8.48E-02	9.38E-01	8.69E-03	1.00E+00
ENSBTAG00000001297	1.47E+00	5.76E-01	4.68E-01	9.95E-01	-1.54E-01	1.00E+00	4.85E-01	9.66E-01	9.13E-01	9.23E-01
ENSBTAG00000042129	0.00E+00	1.00E+00	0.00E+00	1.00E+00	-5.13E-15	1.00E+00	0.00E+00	1.00E+00	0.00E+00	1.00E+00
ENSBTAG00000001296	-1.95E-01	8.23E-01	-3.38E-01	1.07E-01	-1.85E-01	4.79E-01	-7.75E-02	9.84E-01	-3.59E-01	3.69E-01
ENSBTAG00000015222	-1.13E-01	1.00E+00	5.45E-01	1.57E-02	2.06E-01	5.12E-01	-3.09E-01	2.59E-01	-1.75E-02	1.00E+00
ENSBTAG00000001651	9.30E-02	1.00E+00	8.30E-01	5.18E-04	8.38E-01	4.24E-04	4.40E-01	1.12E-01	1.84E-01	9.87E-01
ENSBTAG00000044989	0.00E+00	1.00E+00	0.00E+00	1.00E+00	-5.13E-15	1.00E+00	0.00E+00	1.00E+00	0.00E+00	1.00E+00
ENSBTAG00000019800	-6.85E-02	1.00E+00	-6.00E-02	1.00E+00	-4.85E-02	1.00E+00	-4.39E-01	3.96E-01	-2.07E-01	1.00E+00
ENSBTAG00000009470	2.57E-01	9.58E-01	6.53E-01	4.51E-02	-1.68E-01	8.47E-01	-3.58E-02	1.00E+00	3.12E-01	8.02E-01
ENSBTAG00000013772	-1.76E-03	1.00E+00	3.95E-02	1.00E+00	3.65E-01	2.12E-01	1.17E-01	9.28E-01	9.79E-02	1.00E+00
ENSBTAG00000012604	-1.13E-01	1.00E+00	-1.73E+00	3.31E-02	-2.45E+00	2.24E-03	-2.75E+00	1.06E-03	5.21E-01	9.12E-01
ENSBTAG00000017452	1.67E-01	1.00E+00	-1.43E+00	2.35E-03	-6.32E-01	2.26E-01	4.91E-01	3.82E-01	2.26E-01	1.00E+00
ENSBTAG00000013048	-4.31E-01	7.19E-01	-1.11E+00	1.62E-03	-8.95E-01	1.08E-02	5.61E-01	1.38E-01	7.51E-01	1.97E-01
ENSBTAG00000010278	1.10E-02	1.00E+00	-1.51E+00	6.31E-02	-1.08E+00	1.69E-01	-5.40E-01	5.85E-01	8.29E-01	5.84E-01
ENSBTAG00000019791	6.89E-01	1.00E+00	8.75E-02	1.00E+00	-1.54E-01	1.00E+00	4.81E-01	9.67E-01	1.13E-02	1.00E+00
ENSBTAG00000045407	0.00E+00	1.00E+00	0.00E+00	1.00E+00	-5.13E-15	1.00E+00	0.00E+00	1.00E+00	0.00E+00	1.00E+00

ENSBTAG00000001101	-1.50E-01	1.00E+00	-4.75E-01	1.33E-01	-1.61E+00	2.47E-09	-1.41E+00	2.39E-07	3.00E-01	8.00E-01
ENSBTAG00000001100	2.41E-03	1.00E+00	7.69E-01	7.37E-01	1.16E+00	4.12E-01	1.42E+00	2.49E-01	1.76E+00	4.30E-01
ENSBTAG00000014885	-4.12E-01	1.00E+00	-9.97E-02	1.00E+00	-6.20E-01	5.47E-01	7.82E-01	4.02E-01	3.66E-01	1.00E+00
ENSBTAG00000042918	0.00E+00	1.00E+00	0.00E+00	1.00E+00	-5.13E-15	1.00E+00	0.00E+00	1.00E+00	0.00E+00	1.00E+00
ENSBTAG00000048024	2.72E+00	5.76E-01	0.00E+00	1.00E+00	-5.13E-15	1.00E+00	1.76E+00	4.97E-01	1.93E+00	8.00E-01
ENSBTAG00000008072	2.22E-01	7.84E-01	1.18E+00	3.39E-11	1.03E+00	7.07E-09	3.20E-01	1.46E-01	-5.30E-01	6.40E-02
ENSBTAG00000030435	-2.47E-01	8.63E-01	-5.94E-01	2.48E-02	-5.82E-01	2.84E-02	-2.04E-01	6.33E-01	6.54E-02	1.00E+00
ENSBTAG00000019371	-1.45E+00	7.23E-02	-1.59E+00	6.55E-03	-5.67E-01	4.54E-01	-4.66E-01	5.92E-01	-4.74E-02	1.00E+00
ENSBTAG00000030434	-1.75E-01	9.54E-01	-1.03E+00	2.37E-07	-1.13E+00	9.50E-09	-8.29E-01	4.80E-05	4.27E-01	2.99E-01
ENSBTAG00000043449	0.00E+00	1.00E+00	0.00E+00	1.00E+00	-5.13E-15	1.00E+00	0.00E+00	1.00E+00	0.00E+00	1.00E+00
ENSBTAG00000021832	-2.82E-01	6.18E-01	-9.23E-01	2.19E-06	-1.67E+00	2.24E-18	-1.69E+00	1.08E-18	-2.04E-01	8.16E-01
ENSBTAG00000005002	1.11E-01	1.00E+00	3.95E-01	4.75E-01	8.30E-01	5.11E-02	1.80E+00	4.14E-06	7.76E-01	3.47E-01
ENSBTAG00000011625	-2.15E-01	1.00E+00	-2.48E-01	5.52E-01	1.40E-01	8.88E-01	3.69E-01	2.86E-01	-6.86E-02	1.00E+00
ENSBTAG00000021620	-4.12E-01	3.51E-01	-1.12E+00	2.95E-07	-5.54E-01	1.80E-02	-2.75E-01	3.58E-01	-2.61E-01	7.98E-01
ENSBTAG00000047467	-1.65E-01	1.00E+00	1.57E-01	8.59E-01	3.27E-01	3.95E-01	7.20E-01	1.61E-02	1.04E-01	1.00E+00
ENSBTAG00000026585	1.01E-01	1.00E+00	1.48E+00	1.72E-07	1.26E+00	9.08E-06	1.15E+00	8.01E-05	6.82E-01	1.97E-01
ENSBTAG00000020905	-1.09E-01	1.00E+00	8.69E-02	1.00E+00	6.08E-02	1.00E+00	1.02E-01	9.93E-01	-1.15E-01	1.00E+00
ENSBTAG00000030425	-2.45E+00	4.08E-13	-8.36E-01	1.25E-02	-2.37E-01	6.79E-01	-3.05E-01	5.20E-01	-4.41E-01	6.53E-01
ENSBTAG00000014400	1.10E+00	3.04E-01	5.34E-01	5.72E-01	1.39E+00	1.63E-02	1.06E+00	1.00E-01	4.44E-01	1.00E+00
ENSBTAG00000012263	-3.15E-01	8.23E-01	-1.98E+00	1.10E-12	-3.57E+00	9.75E-34	-2.89E+00	3.63E-24	3.67E-01	7.09E-01
ENSBTAG00000043152	0.00E+00	1.00E+00	0.00E+00	1.00E+00	-5.13E-15	1.00E+00	0.00E+00	1.00E+00	0.00E+00	1.00E+00
ENSBTAG00000038865	-2.22E-01	1.00E+00	-1.61E+00	9.31E-06	-2.82E+00	1.94E-14	-3.90E+00	7.89E-24	-5.36E-01	6.01E-01
ENSBTAG00000002594	-4.23E-01	5.03E-01	-5.19E-01	7.97E-02	-2.35E-01	5.50E-01	-2.91E-01	4.36E-01	3.66E-01	6.21E-01
ENSBTAG00000016578	-5.51E-02	1.00E+00	3.62E-02	1.00E+00	3.12E-01	1.84E-01	6.44E-01	1.38E-03	1.34E-01	1.00E+00
ENSBTAG00000040329	-1.41E+00	7.66E-01	-4.13E+00	1.96E-02	-2.35E+00	1.01E-01	-4.13E+00	1.23E-02	-5.41E-01	1.00E+00
ENSBTAG00000009502	7.62E-01	5.79E-02	1.08E+00	1.04E-04	1.54E+00	5.29E-09	2.15E+00	1.12E-16	5.35E-01	4.25E-01
ENSBTAG00000009500	-5.21E-02	1.00E+00	-2.23E-01	4.66E-01	2.03E-01	5.13E-01	3.35E-01	1.95E-01	-1.55E-01	1.00E+00
ENSBTAG00000030413	0.00E+00	1.00E+00	1.98E+00	4.43E-01	1.78E+00	4.79E-01	3.40E+00	8.35E-02	3.22E+00	4.34E-01
ENSBTAG00000045902	2.22E-02	1.00E+00	6.67E-01	6.65E-01	4.91E-02	1.00E+00	6.41E-01	6.71E-01	-2.29E-01	1.00E+00
ENSBTAG00000048247	0.00E+00	1.00E+00	0.00E+00	1.00E+00	-5.13E-15	1.00E+00	0.00E+00	1.00E+00	0.00E+00	1.00E+00
ENSBTAG00000011196	-2.40E-01	9.05E-01	-2.26E-01	5.76E-01	-6.44E-01	1.70E-02	-8.09E-01	2.25E-03	-2.69E-01	8.00E-01
ENSBTAG00000011195	-3.00E-01	1.00E+00	-1.55E-01	1.00E+00	-3.27E-01	6.53E-01	-7.81E-01	1.15E-01	-2.70E-01	1.00E+00
ENSBTAG00000011193	-2.62E-01	8.53E-01	-3.92E-01	2.12E-01	-8.12E-01	2.09E-03	-1.06E+00	4.39E-05	-3.31E-01	7.37E-01
ENSBTAG00000007153	-2.86E-01	8.23E-01	-5.60E-01	3.99E-02	-9.10E-01	2.99E-04	-9.81E-01	1.09E-04	-3.00E-01	7.90E-01
ENSBTAG00000020102	-9.16E-01	9.54E-01	-4.55E-01	9.51E-01	-4.18E-01	9.67E-01	-2.03E-01	1.00E+00	-1.42E+00	7.68E-01

ENSBTAG00000016448	2.87E-01	8.75E-01	1.10E+00	1.76E-04	8.10E-01	7.61E-03	7.10E-02	1.00E+00	6.29E-01	2.81E-01
ENSBTAG00000045330	0.00E+00	1.00E+00	0.00E+00	1.00E+00	-5.13E-15	1.00E+00	0.00E+00	1.00E+00	0.00E+00	1.00E+00
ENSBTAG00000022671	-9.06E-01	9.60E-01	1.01E-01	1.00E+00	2.23E-01	1.00E+00	-1.83E-01	1.00E+00	-5.09E-01	1.00E+00
ENSBTAG00000042891	2.72E+00	5.76E-01	0.00E+00	1.00E+00	3.05E+00	1.36E-01	0.00E+00	1.00E+00	0.00E+00	1.00E+00
ENSBTAG00000001700	4.49E-02	1.00E+00	1.06E-01	9.27E-01	3.49E-01	1.82E-01	4.68E-01	5.55E-02	-1.90E-01	9.15E-01
ENSBTAG00000040602	-7.57E-02	1.00E+00	-3.57E-01	1.75E-01	-2.78E-01	3.38E-01	-1.61E-01	7.12E-01	-5.29E-01	1.83E-01
ENSBTAG00000013085	-1.66E+00	7.81E-01	-3.58E+00	6.70E-02	-3.58E+00	4.56E-02	-1.82E+00	3.01E-01	1.65E-02	1.00E+00
ENSBTAG00000030269	-2.71E+00	5.76E-01	-2.71E+00	2.42E-01	-2.71E+00	1.99E-01	-2.71E+00	2.05E-01	-2.71E+00	6.01E-01
ENSBTAG00000017122	2.82E-02	1.00E+00	-3.82E-01	2.42E-01	-4.65E-01	1.28E-01	-5.65E-01	5.65E-02	-1.50E-02	1.00E+00
ENSBTAG00000001784	-8.66E-01	1.00E+00	-1.60E+00	4.12E-01	-1.04E+00	6.02E-01	-5.56E-01	9.25E-01	-1.66E+00	7.88E-01
ENSBTAG00000006940	-2.45E-01	7.69E-01	-3.77E-01	9.62E-02	9.31E-02	9.37E-01	-1.91E-01	5.26E-01	-1.40E-01	1.00E+00
ENSBTAG00000044807	0.00E+00	1.00E+00	0.00E+00	1.00E+00	-5.13E-15	1.00E+00	0.00E+00	1.00E+00	0.00E+00	1.00E+00
ENSBTAG00000008952	1.71E+00	3.27E-05	2.54E-01	7.50E-01	-6.94E-01	1.04E-01	7.16E-01	9.60E-02	1.13E+00	4.39E-02
ENSBTAG00000008951	4.18E-01	5.76E-01	-1.03E-01	1.00E+00	-4.82E-01	1.30E-01	-4.34E-01	2.00E-01	8.88E-01	2.29E-02
ENSBTAG00000042825	0.00E+00	1.00E+00	0.00E+00	1.00E+00	-5.13E-15	1.00E+00	0.00E+00	1.00E+00	0.00E+00	1.00E+00
ENSBTAG00000002977	1.91E-01	1.00E+00	8.18E-02	1.00E+00	-3.66E-01	2.06E-01	2.49E-01	4.83E-01	2.61E-01	8.00E-01
ENSBTAG00000040215	-5.13E-02	1.00E+00	2.14E-02	1.00E+00	2.46E-01	4.44E-01	6.57E-01	6.01E-03	1.35E-01	1.00E+00
ENSBTAG00000018656	-1.17E-01	1.00E+00	-9.69E-01	9.56E-08	-2.80E-01	2.15E-01	1.24E-01	7.63E-01	-6.71E-02	1.00E+00
ENSBTAG00000001082	-2.75E-02	1.00E+00	1.44E+00	1.23E-01	2.38E+00	1.22E-03	2.02E+00	1.09E-02	-4.14E+00	1.44E-01
ENSBTAG00000017644	-9.32E-01	1.38E-01	-6.48E-01	1.62E-01	-8.65E-01	4.07E-02	-5.20E-01	2.89E-01	-4.20E-01	8.00E-01
ENSBTAG00000006085	5.73E-01	1.52E-01	1.50E+00	7.80E-10	1.78E+00	1.96E-13	1.87E+00	1.60E-14	7.49E-01	4.64E-02
ENSBTAG00000006084	-5.04E-01	2.21E-01	-8.75E-01	2.52E-04	-1.54E+00	2.77E-11	-1.25E+00	9.85E-08	-2.19E-01	8.92E-01
ENSBTAG00000006136	-6.42E-01	8.26E-01	-2.13E+00	1.12E-03	-2.83E+00	1.45E-05	-1.25E+00	7.99E-02	-6.84E-02	1.00E+00
ENSBTAG00000046988	8.91E-01	1.00E+00	5.88E-01	9.51E-01	1.25E+00	4.33E-01	9.90E-01	6.22E-01	8.85E-01	1.00E+00
ENSBTAG00000020850	-5.07E-01	2.23E-01	5.81E-01	2.09E-02	1.13E+00	8.08E-07	8.70E-01	2.63E-04	5.40E-03	1.00E+00
ENSBTAG00000009156	7.64E-01	1.43E-01	2.64E-01	6.48E-01	3.52E-01	4.49E-01	1.40E-01	9.83E-01	-4.10E-01	7.78E-01
ENSBTAG00000013511	-8.19E-01	4.99E-01	-1.66E+00	3.83E-03	-1.38E+00	1.18E-02	-1.79E+00	1.37E-03	9.90E-02	1.00E+00
ENSBTAG00000040547	0.00E+00	1.00E+00	0.00E+00	1.00E+00	-5.13E-15	1.00E+00	0.00E+00	1.00E+00	1.91E+00	8.00E-01
ENSBTAG00000016877	1.25E-02	1.00E+00	1.77E+00	3.25E-01	1.14E+00	6.46E-01	1.12E+00	6.68E-01	1.31E+00	8.99E-01
ENSBTAG00000012217	5.17E-01	1.00E+00	9.72E-01	6.54E-01	-1.54E-01	1.00E+00	1.23E+00	4.57E-01	5.19E-01	1.00E+00
ENSBTAG00000046977	9.43E-01	1.41E-01	1.47E+00	2.81E-04	2.16E+00	4.30E-08	1.71E+00	2.37E-05	-6.60E-01	5.79E-01
ENSBTAG00000020499	7.13E-01	6.18E-01	7.10E-01	2.58E-01	2.30E+00	3.00E-06	2.72E+00	4.04E-08	-2.87E-01	1.00E+00
ENSBTAG00000039122	1.11E+00	7.92E-01	3.39E+00	8.99E-07	2.46E+00	1.53E-03	1.30E+00	2.41E-01	-8.76E-01	1.00E+00
ENSBTAG00000002700	-2.70E+00	5.76E-01	-7.15E-01	9.45E-01	-2.70E+00	1.99E-01	-9.39E-01	8.06E-01	-2.70E+00	6.01E-01
ENSBTAG00000005759	2.72E+00	5.76E-01	0.00E+00	1.00E+00	-5.13E-15	1.00E+00	1.76E+00	4.97E-01	0.00E+00	1.00E+00

ENSBTAG00000013039	1.26E-02	1.00E+00	7.51E-02	1.00E+00	-1.91E+00	4.28E-01	-1.91E+00	4.39E-01	-1.91E+00	8.00E-01
ENSBTAG00000004418	-1.91E+00	8.23E-01	2.07E+00	1.95E-01	6.50E-01	9.78E-01	-1.46E-01	1.00E+00	-1.91E+00	8.00E-01
ENSBTAG00000017108	8.00E-02	1.00E+00	3.46E-01	2.08E-01	4.90E-01	4.39E-02	3.31E-01	2.41E-01	4.76E-01	2.97E-01
ENSBTAG00000030367	-1.16E+00	3.88E-01	-2.89E+00	1.36E-04	1.04E+00	1.36E-01	2.94E-01	9.30E-01	9.38E-01	5.88E-01
ENSBTAG00000007998	2.38E-01	8.35E-01	-9.17E-01	1.02E-04	-2.86E-01	3.52E-01	-1.94E-01	6.22E-01	4.20E-01	4.34E-01
ENSBTAG00000007996	-5.48E-01	5.22E-01	-2.43E+00	1.66E-09	-2.42E+00	3.57E-10	-2.74E+00	7.04E-12	-3.28E-01	8.52E-01
ENSBTAG00000005140	-3.48E-01	8.23E-01	1.89E-01	8.40E-01	-6.52E-02	1.00E+00	-2.09E-01	7.90E-01	-4.02E-01	7.83E-01
ENSBTAG00000044904	-2.70E+00	5.76E-01	6.02E-01	9.45E-01	1.48E+00	2.97E-01	-1.64E-01	1.00E+00	7.61E-03	1.00E+00
ENSBTAG00000004554	4.19E-02	1.00E+00	-2.45E-01	4.08E-01	5.46E-03	1.00E+00	2.40E-01	4.33E-01	-1.49E-02	1.00E+00
ENSBTAG00000013650	-1.03E-01	1.00E+00	-4.82E-01	8.15E-02	-5.36E-02	1.00E+00	1.09E-01	9.61E-01	-7.37E-02	1.00E+00
ENSBTAG00000012289	-4.52E-01	5.07E-01	-1.09E+00	1.09E-04	-1.36E+00	8.52E-07	-1.52E+00	4.72E-08	-3.00E-01	8.00E-01
ENSBTAG00000026758	-6.43E-02	1.00E+00	-1.23E+00	7.14E-06	-9.48E-01	6.71E-04	-7.15E-01	1.58E-02	8.74E-02	1.00E+00
ENSBTAG00000011951	2.83E-01	8.14E-01	1.89E+00	5.85E-16	2.25E+00	6.37E-22	1.98E+00	2.13E-17	2.11E-02	1.00E+00
ENSBTAG00000011950	-7.53E-02	1.00E+00	1.28E-01	9.70E-01	4.01E-01	2.84E-01	9.02E-01	3.45E-03	8.33E-01	7.11E-02
ENSBTAG00000044375	0.00E+00	1.00E+00	0.00E+00	1.00E+00	-5.13E-15	1.00E+00	0.00E+00	1.00E+00	0.00E+00	1.00E+00
ENSBTAG00000015240	-1.94E-02	1.00E+00	4.82E-01	3.42E-02	3.62E-01	1.40E-01	-7.48E-02	1.00E+00	2.13E-01	8.28E-01
ENSBTAG00000028707	0.00E+00	1.00E+00	0.00E+00	1.00E+00	-5.13E-15	1.00E+00	0.00E+00	1.00E+00	0.00E+00	1.00E+00
ENSBTAG00000030341	-2.58E+00	6.70E-02	-3.68E-01	9.06E-01	-2.74E+00	5.99E-03	-1.60E+00	8.96E-02	-1.40E+00	4.95E-01
ENSBTAG00000030340	1.48E-01	1.00E+00	-5.81E-01	3.84E-01	3.05E-01	7.38E-01	7.46E-01	1.49E-01	-1.10E-01	1.00E+00
ENSBTAG00000030335	-4.31E-01	2.28E-01	-9.31E-01	4.06E-06	-1.94E+00	1.49E-22	-1.64E+00	1.29E-16	4.62E-01	2.36E-01
ENSBTAG00000015246	5.88E-01	1.00E+00	8.57E-02	1.00E+00	-2.36E+00	1.02E-01	-2.05E-01	1.00E+00	1.26E+00	5.66E-01
ENSBTAG00000014967	-5.05E-01	8.81E-01	-8.47E-01	2.02E-01	-1.04E+00	8.78E-02	-1.01E+00	1.04E-01	-2.29E-01	1.00E+00
ENSBTAG00000030322	1.48E-04	1.00E+00	-1.37E+00	1.35E-01	-8.63E-01	4.13E-01	-4.70E-01	8.01E-01	1.06E-01	1.00E+00
ENSBTAG00000019172	1.95E-02	1.00E+00	-5.09E-02	1.00E+00	-1.41E+00	1.92E-01	3.57E-01	9.04E-01	-2.92E+00	1.41E-01
ENSBTAG00000006438	0.00E+00	1.00E+00	1.98E+00	4.43E-01	-5.13E-15	1.00E+00	0.00E+00	1.00E+00	1.91E+00	8.00E-01
ENSBTAG00000000684	-1.21E-01	1.00E+00	-1.22E+00	7.65E-03	-7.80E-01	1.20E-01	-6.74E-01	2.09E-01	1.27E-01	1.00E+00
ENSBTAG00000008579	8.35E-02	1.00E+00	8.62E-01	7.00E-05	7.32E-01	8.70E-04	6.38E-01	5.32E-03	1.41E-02	1.00E+00
ENSBTAG00000045172	-3.83E-01	8.23E-01	2.64E-01	6.68E-01	-9.03E-02	1.00E+00	-2.32E-01	7.62E-01	-2.75E-01	9.79E-01
ENSBTAG00000038945	5.11E-01	1.00E+00	9.82E-01	6.49E-01	3.50E-01	1.00E+00	1.44E+00	3.29E-01	8.98E-01	1.00E+00
ENSBTAG00000012052	1.47E+00	3.82E-02	-5.29E-01	5.96E-01	2.14E+00	1.55E-05	1.99E+00	9.14E-05	7.76E-01	6.29E-01
ENSBTAG00000012043	9.56E-01	8.10E-01	3.11E-01	1.00E+00	2.15E-01	1.00E+00	1.09E+00	2.82E-01	-5.17E-01	1.00E+00
ENSBTAG00000002138	-9.41E-01	8.23E-01	-3.96E-01	9.23E-01	-1.12E+00	3.09E-01	-2.02E-01	1.00E+00	-9.44E-01	8.00E-01
ENSBTAG00000043637	0.00E+00	1.00E+00	0.00E+00	1.00E+00	-5.13E-15	1.00E+00	0.00E+00	1.00E+00	0.00E+00	1.00E+00
ENSBTAG00000003403	-1.50E-01	1.00E+00	-1.06E+00	5.33E-02	-5.04E-01	4.29E-01	-3.09E-01	7.54E-01	1.27E-01	1.00E+00
ENSBTAG00000008314	-2.51E-01	8.23E-01	-5.84E-01	1.26E-02	-9.47E-01	1.66E-05	-1.29E+00	3.14E-09	-2.74E-01	7.72E-01

ENSBTAG00000008309	-4.53E-01	4.48E-01	-1.27E+00	2.07E-06	-7.17E-01	1.05E-02	-4.33E-01	1.84E-01	-1.15E-01	1.00E+00
ENSBTAG00000003832	-5.79E-01	6.80E-01	-1.23E+00	9.51E-03	-1.86E+00	6.10E-05	-1.74E+00	2.04E-04	-4.19E-01	8.39E-01
ENSBTAG00000003822	-1.47E-01	1.00E+00	-7.94E-01	1.88E-03	-1.44E+00	3.63E-09	-1.45E+00	3.80E-09	2.48E-01	8.33E-01
ENSBTAG00000013282	-1.09E-01	1.00E+00	-1.45E-01	6.90E-01	-9.55E-02	9.13E-01	6.82E-02	1.00E+00	-5.07E-02	1.00E+00
ENSBTAG00000013281	-3.52E-01	8.23E-01	5.47E-02	1.00E+00	-9.41E-01	3.47E-03	-9.83E-01	2.57E-03	1.81E-02	1.00E+00
ENSBTAG00000009251	2.01E-01	1.00E+00	8.45E-01	1.28E-03	9.23E-01	3.56E-04	1.02E+00	8.86E-05	2.36E-01	8.97E-01
ENSBTAG00000047837	1.32E+00	5.27E-07	3.77E+00	3.27E-49	2.36E+00	3.57E-22	1.46E+00	2.29E-09	2.33E-01	8.84E-01
ENSBTAG00000008510	-2.07E-01	8.63E-01	7.79E-01	1.11E-04	-3.46E-01	1.53E-01	-2.33E-01	4.33E-01	2.10E-01	8.15E-01
ENSBTAG00000014221	-1.15E-01	1.00E+00	-1.11E+00	4.24E-04	-1.48E+00	1.62E-06	-4.23E-01	2.55E-01	1.29E-01	1.00E+00
ENSBTAG00000005670	-6.30E-01	1.58E-01	-2.40E+00	2.15E-18	-4.61E+00	2.46E-53	-3.93E+00	9.44E-42	-2.55E-01	8.88E-01
ENSBTAG00000000815	1.25E+00	9.35E-04	1.72E+00	7.61E-08	1.01E+00	2.25E-03	7.98E-01	2.38E-02	-2.70E-01	9.43E-01
ENSBTAG00000043154	0.00E+00	1.00E+00	0.00E+00	1.00E+00	-5.13E-15	1.00E+00	0.00E+00	1.00E+00	0.00E+00	1.00E+00
ENSBTAG00000034680	-7.02E-01	5.39E-01	-2.08E+00	5.72E-05	-3.62E+00	5.08E-10	-1.48E+00	2.51E-03	2.66E-01	1.00E+00
ENSBTAG00000034674	1.19E-02	1.00E+00	4.66E-01	9.96E-01	5.13E-01	9.39E-01	-1.87E-01	1.00E+00	-5.06E-01	1.00E+00
ENSBTAG00000010940	3.66E+00	9.62E-02	4.37E+00	3.22E-03	1.85E+00	3.94E-01	4.63E+00	1.82E-03	4.83E+00	1.68E-02
ENSBTAG00000010938	0.00E+00	1.00E+00	0.00E+00	1.00E+00	1.78E+00	4.79E-01	1.76E+00	4.97E-01	0.00E+00	1.00E+00
ENSBTAG00000048232	0.00E+00	1.00E+00	0.00E+00	1.00E+00	-5.13E-15	1.00E+00	0.00E+00	1.00E+00	0.00E+00	1.00E+00
ENSBTAG00000046826	0.00E+00	1.00E+00	0.00E+00	1.00E+00	-5.13E-15	1.00E+00	0.00E+00	1.00E+00	0.00E+00	1.00E+00
ENSBTAG00000038537	1.91E+00	8.23E-01	0.00E+00	1.00E+00	-5.13E-15	1.00E+00	0.00E+00	1.00E+00	0.00E+00	1.00E+00
ENSBTAG00000038364	0.00E+00	1.00E+00	0.00E+00	1.00E+00	-5.13E-15	1.00E+00	0.00E+00	1.00E+00	0.00E+00	1.00E+00
ENSBTAG00000000617	0.00E+00	1.00E+00	0.00E+00	1.00E+00	-5.13E-15	1.00E+00	0.00E+00	1.00E+00	0.00E+00	1.00E+00
ENSBTAG00000045490	0.00E+00	1.00E+00	0.00E+00	1.00E+00	-5.13E-15	1.00E+00	0.00E+00	1.00E+00	0.00E+00	1.00E+00
ENSBTAG00000047333	0.00E+00	1.00E+00	0.00E+00	1.00E+00	-5.13E-15	1.00E+00	0.00E+00	1.00E+00	0.00E+00	1.00E+00
ENSBTAG00000024593	0.00E+00	1.00E+00	1.98E+00	4.43E-01	-5.13E-15	1.00E+00	0.00E+00	1.00E+00	1.93E+00	8.00E-01
ENSBTAG00000011895	2.22E-01	9.36E-01	-1.35E+00	3.41E-08	-1.59E+00	4.93E-11	-1.07E+00	1.65E-05	2.79E-01	8.00E-01
ENSBTAG00000029967	0.00E+00	1.00E+00	0.00E+00	1.00E+00	-5.13E-15	1.00E+00	0.00E+00	1.00E+00	0.00E+00	1.00E+00
ENSBTAG00000017746	5.34E-02	1.00E+00	5.07E-01	5.73E-01	4.81E-01	6.05E-01	-4.18E-01	7.13E-01	-3.76E-02	1.00E+00
ENSBTAG00000029895	0.00E+00	1.00E+00	0.00E+00	1.00E+00	-5.13E-15	1.00E+00	0.00E+00	1.00E+00	0.00E+00	1.00E+00
ENSBTAG00000008180	-8.60E-01	5.81E-01	-5.16E-01	5.57E-01	3.18E-01	7.87E-01	1.04E+00	4.59E-02	-2.08E-01	1.00E+00
ENSBTAG00000014612	1.72E-01	1.00E+00	-8.42E-01	1.42E-03	-7.65E-01	3.73E-03	-2.81E-01	4.39E-01	-2.67E-01	8.02E-01
ENSBTAG00000002134	0.00E+00	1.00E+00	0.00E+00	1.00E+00	-5.13E-15	1.00E+00	0.00E+00	1.00E+00	0.00E+00	1.00E+00
ENSBTAG00000014620	-1.91E+00	8.23E-01	7.78E-02	1.00E+00	-1.91E+00	4.28E-01	-1.47E-01	1.00E+00	-1.91E+00	8.00E-01
ENSBTAG00000043132	0.00E+00	1.00E+00	0.00E+00	1.00E+00	-5.13E-15	1.00E+00	0.00E+00	1.00E+00	0.00E+00	1.00E+00
ENSBTAG00000046449	-7.13E-01	5.21E-01	-1.83E+00	4.61E-04	-8.82E-01	7.75E-02	-6.29E-01	2.54E-01	-8.94E-01	3.68E-01
ENSBTAG00000009381	5.03E-01	2.59E-01	3.37E-01	2.88E-01	-1.71E-01	7.41E-01	-4.30E-01	1.51E-01	-2.42E-01	8.55E-01

ENSBTAG00000002985	-1.13E-01	1.00E+00	1.37E-01	1.00E+00	-1.93E+00	1.01E-03	-8.75E-01	1.55E-01	2.47E-01	1.00E+00
ENSBTAG00000002865	-2.81E-01	1.00E+00	-8.71E-01	2.24E-01	-1.15E+00	7.67E-02	-4.55E-01	6.10E-01	-4.94E-01	9.01E-01
ENSBTAG00000002779	1.53E-02	1.00E+00	1.40E+00	5.26E-01	1.54E+00	4.32E-01	2.84E+00	3.02E-02	7.97E-01	1.00E+00
ENSBTAG00000024801	4.49E-02	1.00E+00	-6.81E-01	1.10E-02	-4.44E-01	1.29E-01	-1.63E-01	7.81E-01	-4.16E-01	5.30E-01
ENSBTAG00000034824	0.00E+00	1.00E+00	0.00E+00	1.00E+00	-5.13E-15	1.00E+00	0.00E+00	1.00E+00	0.00E+00	1.00E+00
ENSBTAG00000022684	0.00E+00	1.00E+00	0.00E+00	1.00E+00	-5.13E-15	1.00E+00	0.00E+00	1.00E+00	0.00E+00	1.00E+00
ENSBTAG00000010003	8.05E-01	1.00E+00	7.11E-02	1.00E+00	-1.37E-01	1.00E+00	6.15E-01	9.96E-01	3.33E-04	1.00E+00
ENSBTAG00000015316	6.25E-01	1.07E-01	2.00E+00	2.37E-16	2.46E+00	5.62E-24	1.41E+00	1.31E-08	-3.78E-01	6.18E-01
ENSBTAG00000000128	-8.93E-02	1.00E+00	1.08E-01	1.00E+00	3.54E-01	3.36E-01	4.09E-01	2.50E-01	2.69E-01	8.62E-01
ENSBTAG00000042429	0.00E+00	1.00E+00	0.00E+00	1.00E+00	-5.13E-15	1.00E+00	0.00E+00	1.00E+00	0.00E+00	1.00E+00
ENSBTAG00000017013	0.00E+00	1.00E+00	0.00E+00	1.00E+00	-5.13E-15	1.00E+00	0.00E+00	1.00E+00	0.00E+00	1.00E+00
ENSBTAG00000008357	2.37E+00	1.40E-01	2.07E+00	8.36E-02	1.25E+00	4.38E-01	6.94E-01	8.59E-01	-7.10E-04	1.00E+00
ENSBTAG00000015376	9.47E-02	1.00E+00	5.02E-01	5.64E-02	6.62E-01	7.13E-03	7.30E-01	3.13E-03	2.82E-02	1.00E+00
ENSBTAG00000017457	4.63E-02	1.00E+00	-9.42E-02	1.00E+00	-4.45E-01	9.84E-02	-1.22E-01	9.02E-01	3.28E-01	6.55E-01
ENSBTAG00000008876	-1.42E+00	7.67E-01	4.99E-01	8.53E-01	-1.07E+00	4.79E-01	-4.12E+00	1.27E-02	-5.23E-01	1.00E+00
ENSBTAG00000034659	3.73E-01	7.71E-01	-6.02E-01	9.97E-02	1.99E-01	7.68E-01	5.27E-01	1.39E-01	3.60E-01	7.98E-01
ENSBTAG00000044301	0.00E+00	1.00E+00	0.00E+00	1.00E+00	-5.13E-15	1.00E+00	0.00E+00	1.00E+00	0.00E+00	1.00E+00
ENSBTAG00000009019	3.65E-01	7.20E-01	-1.19E+00	6.31E-05	-1.32E+00	6.37E-06	-9.22E-01	2.30E-03	8.24E-01	5.09E-02
ENSBTAG00000001429	2.69E-01	1.00E+00	-7.88E-01	6.83E-02	-1.12E-01	1.00E+00	-2.25E-02	1.00E+00	6.77E-02	1.00E+00
ENSBTAG00000013863	-2.55E-01	1.00E+00	2.08E+00	2.38E-05	1.03E+00	5.35E-02	5.40E-01	4.39E-01	-2.07E-02	1.00E+00
ENSBTAG00000015955	7.48E-02	1.00E+00	-5.50E-01	1.38E-02	-1.18E+00	1.04E-08	-1.24E+00	2.65E-09	5.32E-01	1.41E-01
ENSBTAG00000015099	4.66E-02	1.00E+00	3.63E-01	2.39E-01	5.97E-01	2.31E-02	-1.71E-02	1.00E+00	-1.70E-01	1.00E+00
ENSBTAG00000015100	1.29E-01	1.00E+00	2.01E-03	1.00E+00	9.63E-02	1.00E+00	8.27E-02	1.00E+00	-9.73E-03	1.00E+00
ENSBTAG00000002020	7.01E-01	5.76E-01	8.44E-01	1.15E-01	2.64E-01	8.44E-01	6.35E-01	2.92E-01	5.21E-01	8.00E-01
ENSBTAG00000002021	-1.87E-01	9.78E-01	1.24E+00	4.22E-09	8.64E-01	6.47E-05	7.32E-01	1.11E-03	-3.78E-01	5.11E-01
ENSBTAG00000029583	0.00E+00	1.00E+00	0.00E+00	1.00E+00	-5.13E-15	1.00E+00	0.00E+00	1.00E+00	0.00E+00	1.00E+00
ENSBTAG00000020568	-4.54E+00	2.94E-02	-5.59E-01	8.17E-01	-2.13E-02	1.00E+00	-1.51E+00	2.23E-01	-1.83E+00	5.02E-01
ENSBTAG00000001823	1.33E+00	9.31E-01	8.82E-01	8.53E-01	2.76E+00	3.25E-02	3.87E+00	2.68E-04	1.73E+00	7.72E-01
ENSBTAG00000034598	2.81E-01	7.56E-01	1.94E+00	7.01E-20	2.68E+00	3.92E-36	8.96E-01	5.38E-05	-9.88E-02	1.00E+00
ENSBTAG00000016216	0.00E+00	1.00E+00	0.00E+00	1.00E+00	-5.13E-15	1.00E+00	0.00E+00	1.00E+00	0.00E+00	1.00E+00
ENSBTAG00000009995	-2.87E-01	7.82E-01	4.01E-01	1.50E-01	5.83E-01	2.09E-02	-4.70E-01	8.56E-02	-4.12E-01	4.78E-01
ENSBTAG00000045543	0.00E+00	1.00E+00	0.00E+00	1.00E+00	-5.13E-15	1.00E+00	0.00E+00	1.00E+00	0.00E+00	1.00E+00
ENSBTAG00000000564	8.69E-02	1.00E+00	1.19E+00	1.03E-07	-1.31E-01	8.40E-01	-1.92E-01	6.22E-01	8.19E-03	1.00E+00
ENSBTAG00000007729	-3.80E-01	1.00E+00	9.04E-02	1.00E+00	-1.81E+00	2.97E-01	3.71E-01	1.00E+00	2.31E-02	1.00E+00
ENSBTAG00000030064	0.00E+00	1.00E+00	0.00E+00	1.00E+00	-5.13E-15	1.00E+00	0.00E+00	1.00E+00	0.00E+00	1.00E+00

ENSBTAG00000013873	2.71E+00	5.76E-01	0.00E+00	1.00E+00	1.78E+00	4.79E-01	3.03E+00	1.49E-01	1.93E+00	8.00E-01
ENSBTAG00000016002	-3.86E-03	1.00E+00	-1.09E+00	5.85E-01	1.37E+00	2.15E-01	2.77E+00	9.54E-04	-6.24E-01	1.00E+00
ENSBTAG00000003066	-3.41E-02	1.00E+00	5.43E-01	1.10E-01	4.96E-01	1.50E-01	8.00E-02	1.00E+00	-4.92E-01	6.01E-01
ENSBTAG00000015519	-5.20E-02	1.00E+00	-9.94E-02	9.45E-01	2.71E-02	1.00E+00	1.75E-02	1.00E+00	-3.09E-02	1.00E+00
ENSBTAG00000015512	6.62E-02	1.00E+00	-4.93E-01	2.36E-02	-7.17E-01	4.59E-04	-8.71E-01	1.91E-05	-1.17E-02	1.00E+00
ENSBTAG00000034138	9.03E-02	1.00E+00	-5.01E-01	1.63E-01	-5.11E-01	1.44E-01	-9.16E-01	4.09E-03	1.93E-01	1.00E+00
ENSBTAG00000026369	9.48E-02	1.00E+00	-2.35E+00	1.02E-04	-1.22E+00	5.86E-02	7.97E-01	2.91E-01	-9.55E-02	1.00E+00
ENSBTAG00000044229	0.00E+00	1.00E+00	0.00E+00	1.00E+00	-5.13E-15	1.00E+00	0.00E+00	1.00E+00	0.00E+00	1.00E+00
ENSBTAG00000045014	0.00E+00	1.00E+00	0.00E+00	1.00E+00	-5.13E-15	1.00E+00	0.00E+00	1.00E+00	0.00E+00	1.00E+00
ENSBTAG00000005633	-4.96E-01	6.14E-01	-1.05E+00	4.24E-03	-2.09E+00	5.02E-09	-2.01E+00	2.22E-08	-1.77E-01	1.00E+00
ENSBTAG00000006843	2.31E-01	8.52E-01	1.57E+00	3.93E-13	1.92E+00	2.73E-19	1.21E+00	3.11E-08	-2.88E-01	7.62E-01
ENSBTAG00000018452	-1.10E-01	1.00E+00	1.61E-01	6.28E-01	9.92E-02	8.98E-01	-4.44E-01	4.19E-02	-2.67E-01	6.84E-01
ENSBTAG00000010012	8.85E-02	1.00E+00	5.97E-01	2.56E-03	6.17E-01	1.71E-03	4.76E-01	2.34E-02	-5.57E-01	6.36E-02
ENSBTAG00000032705	-1.39E-01	1.00E+00	-1.25E+00	2.92E-05	-6.84E-01	3.46E-02	-6.39E-02	1.00E+00	1.81E-03	1.00E+00
ENSBTAG00000042972	0.00E+00	1.00E+00	0.00E+00	1.00E+00	-5.13E-15	1.00E+00	0.00E+00	1.00E+00	0.00E+00	1.00E+00
ENSBTAG00000003377	0.00E+00	1.00E+00	0.00E+00	1.00E+00	-5.13E-15	1.00E+00	0.00E+00	1.00E+00	0.00E+00	1.00E+00
ENSBTAG00000008494	1.82E+00	2.26E-13	2.15E+00	2.22E-19	2.41E+00	5.11E-24	1.12E+00	4.94E-06	5.13E-01	3.11E-01
ENSBTAG00000017545	7.30E-02	1.00E+00	-2.68E-01	4.75E-01	-1.98E-01	6.77E-01	6.19E-02	1.00E+00	7.47E-02	1.00E+00
ENSBTAG00000007979	-6.06E-02	1.00E+00	3.38E-01	2.16E-01	8.12E-01	2.55E-04	1.09E+00	5.52E-07	1.50E-01	1.00E+00
ENSBTAG00000012775	0.00E+00	1.00E+00	0.00E+00	1.00E+00	-5.13E-15	1.00E+00	0.00E+00	1.00E+00	0.00E+00	1.00E+00
ENSBTAG00000020601	-3.53E-01	9.74E-01	5.68E-01	2.36E-01	6.56E-01	1.35E-01	2.18E-01	8.63E-01	-9.80E-02	1.00E+00
ENSBTAG00000014675	2.75E-02	1.00E+00	3.14E-01	2.29E-01	6.87E-01	1.23E-03	5.71E-01	1.07E-02	-1.28E-01	1.00E+00
ENSBTAG00000001962	-1.39E-01	1.00E+00	6.46E-01	1.15E-02	7.65E-01	2.03E-03	8.95E-01	2.90E-04	-1.34E-01	1.00E+00
ENSBTAG00000001961	5.27E-01	8.23E-01	2.01E+00	2.57E-06	9.85E-01	5.24E-02	4.65E-01	5.23E-01	4.71E-01	8.62E-01
ENSBTAG00000047486	1.92E+00	8.23E-01	1.99E+00	4.43E-01	-5.13E-15	1.00E+00	0.00E+00	1.00E+00	0.00E+00	1.00E+00
ENSBTAG00000044160	2.39E-01	8.23E-01	5.48E-02	1.00E+00	2.40E-01	4.28E-01	3.52E-01	1.77E-01	3.81E-01	4.54E-01
ENSBTAG00000000363	2.63E-01	9.52E-01	5.53E-01	1.10E-01	2.99E-02	1.00E+00	1.92E-01	7.98E-01	-1.21E-02	1.00E+00
ENSBTAG00000034360	3.33E-01	9.12E-01	1.70E-01	9.45E-01	3.51E-01	4.84E-01	1.02E+00	3.96E-03	-1.70E-01	1.00E+00
ENSBTAG00000005743	1.75E-01	9.98E-01	1.54E+00	1.65E-14	1.69E+00	1.51E-17	1.11E+00	6.44E-08	-2.12E-01	8.57E-01
ENSBTAG00000003326	1.54E+00	2.29E-04	2.24E+00	1.00E-09	6.26E-01	1.49E-01	1.45E-01	1.00E+00	6.21E-02	1.00E+00
ENSBTAG00000027983	-1.38E-01	1.00E+00	-1.91E+00	2.39E-13	-1.85E+00	9.35E-13	-1.12E+00	2.20E-05	-1.08E-01	1.00E+00
ENSBTAG00000000561	-4.14E-01	5.60E-01	-4.42E+00	1.14E-47	-4.37E+00	2.02E-48	-2.16E+00	5.73E-16	3.75E-01	6.44E-01
ENSBTAG00000044954	0.00E+00	1.00E+00	0.00E+00	1.00E+00	-5.13E-15	1.00E+00	0.00E+00	1.00E+00	0.00E+00	1.00E+00
ENSBTAG00000040001	-1.04E-02	1.00E+00	-5.84E-02	1.00E+00	4.07E-01	3.70E-01	5.89E-01	1.40E-01	3.07E-01	8.97E-01
ENSBTAG00000002073	1.67E-02	1.00E+00	-4.81E-01	3.89E-02	2.39E-01	4.18E-01	3.60E-01	1.54E-01	2.34E-02	1.00E+00

ENSBTAG00000046192	-2.29E-01	8.23E-01	5.63E-01	1.08E-02	3.20E-01	2.13E-01	-3.36E-01	2.01E-01	-4.84E-01	2.36E-01
ENSBTAG00000027980	0.00E+00	1.00E+00	0.00E+00	1.00E+00	-5.13E-15	1.00E+00	0.00E+00	1.00E+00	0.00E+00	1.00E+00
ENSBTAG00000017011	-1.80E-01	9.59E-01	9.01E-02	9.73E-01	-3.58E-01	1.46E-01	-3.17E-01	2.26E-01	6.99E-03	1.00E+00
ENSBTAG00000011046	2.08E-01	9.62E-01	1.58E+00	5.86E-12	1.68E+00	1.33E-13	1.09E+00	3.83E-06	-3.01E-02	1.00E+00
ENSBTAG00000011045	-1.15E-01	1.00E+00	-5.16E-01	7.10E-02	-5.00E-01	8.28E-02	1.37E-01	8.82E-01	-4.94E-02	1.00E+00
ENSBTAG00000014246	-3.41E-01	9.31E-01	-1.26E+00	2.04E-03	-6.77E-01	1.21E-01	-1.70E-01	9.50E-01	-1.39E-01	1.00E+00
ENSBTAG00000014239	4.28E-01	1.00E+00	1.83E-01	1.00E+00	1.18E-01	1.00E+00	-1.37E-01	1.00E+00	-1.68E-01	1.00E+00
ENSBTAG00000001013	4.41E-01	2.56E-01	1.74E+00	5.59E-17	1.34E+00	1.28E-10	7.21E-01	1.19E-03	-6.80E-02	1.00E+00
ENSBTAG00000010989	1.18E+00	1.25E-02	2.22E+00	1.64E-09	1.42E+00	1.28E-04	5.23E-01	2.71E-01	4.67E-02	1.00E+00
ENSBTAG00000034170	2.50E-01	1.00E+00	1.54E+00	7.50E-07	1.40E+00	8.01E-06	1.10E+00	8.50E-04	2.83E-01	9.38E-01
ENSBTAG00000043699	1.91E+00	8.23E-01	0.00E+00	1.00E+00	-5.13E-15	1.00E+00	0.00E+00	1.00E+00	0.00E+00	1.00E+00
ENSBTAG00000006360	-1.22E+00	4.03E-02	-2.41E+00	2.76E-07	-2.61E-01	7.84E-01	-3.40E-01	6.30E-01	-1.44E-02	1.00E+00
ENSBTAG00000025964	1.71E-01	1.00E+00	-5.48E-01	4.43E-01	-6.01E-01	3.80E-01	-2.40E-01	9.36E-01	2.75E-01	1.00E+00
ENSBTAG00000047291	0.00E+00	1.00E+00	0.00E+00	1.00E+00	-5.13E-15	1.00E+00	0.00E+00	1.00E+00	1.93E+00	8.00E-01
ENSBTAG00000007321	1.12E-01	1.00E+00	3.98E-01	1.40E-01	6.14E-01	1.10E-02	1.29E-01	8.64E-01	-2.09E-01	8.99E-01
ENSBTAG00000000415	1.13E-01	1.00E+00	-8.57E-02	1.00E+00	6.20E-02	1.00E+00	7.84E-02	1.00E+00	-1.77E-02	1.00E+00
ENSBTAG00000016903	1.16E-01	1.00E+00	-1.54E+00	4.04E-05	-5.90E-01	1.75E-01	-5.18E-01	2.69E-01	3.87E-01	8.00E-01
ENSBTAG00000016902	-4.08E-01	9.24E-01	-2.39E-01	8.74E-01	-7.13E-01	1.83E-01	-3.59E-01	6.28E-01	-5.97E-01	7.08E-01
ENSBTAG00000016900	-1.91E-01	9.61E-01	2.07E-01	5.41E-01	-9.75E-02	9.68E-01	-3.04E-01	2.96E-01	-3.04E-01	6.82E-01
ENSBTAG00000047828	0.00E+00	1.00E+00	0.00E+00	1.00E+00	-5.13E-15	1.00E+00	0.00E+00	1.00E+00	0.00E+00	1.00E+00
ENSBTAG00000009519	-3.63E-01	7.19E-01	-9.82E-01	6.31E-04	-3.16E-01	4.15E-01	-3.11E-01	4.39E-01	1.94E-02	1.00E+00
ENSBTAG00000009514	-3.70E-02	1.00E+00	1.94E-01	5.68E-01	-2.57E-01	3.87E-01	-5.95E-01	1.00E-02	-3.21E-01	6.01E-01
ENSBTAG00000044175	-9.09E-02	1.00E+00	-8.60E-01	2.65E-01	-6.87E-01	3.83E-01	-4.29E-01	6.79E-01	-4.23E-01	1.00E+00
ENSBTAG00000046810	0.00E+00	1.00E+00	0.00E+00	1.00E+00	-5.13E-15	1.00E+00	0.00E+00	1.00E+00	0.00E+00	1.00E+00
ENSBTAG00000016963	-1.55E-01	1.00E+00	-4.65E-01	3.48E-01	-4.91E-01	2.99E-01	-5.21E-01	2.71E-01	-1.85E-01	1.00E+00
ENSBTAG00000044452	0.00E+00	1.00E+00	0.00E+00	1.00E+00	-5.13E-15	1.00E+00	0.00E+00	1.00E+00	0.00E+00	1.00E+00
ENSBTAG00000000037	-2.10E-02	1.00E+00	-4.57E-02	1.00E+00	3.46E-01	1.56E-01	1.42E-01	7.51E-01	-3.23E-01	5.99E-01
ENSBTAG00000033897	0.00E+00	1.00E+00	0.00E+00	1.00E+00	-5.13E-15	1.00E+00	0.00E+00	1.00E+00	0.00E+00	1.00E+00
ENSBTAG00000009469	-1.69E-01	1.00E+00	-9.73E-01	1.91E-04	-8.01E-01	2.21E-03	-7.39E-01	6.05E-03	-2.39E-01	8.76E-01
ENSBTAG00000009468	-2.06E-01	1.00E+00	-4.15E-01	8.61E-01	-1.24E+00	1.99E-01	-1.79E+00	6.54E-02	8.43E-01	6.98E-01
ENSBTAG00000005169	-7.83E-01	9.66E-01	-1.01E+00	4.69E-01	-7.14E-01	6.39E-01	5.88E-01	6.71E-01	-1.45E-01	1.00E+00
ENSBTAG00000043992	-3.28E-01	1.00E+00	-1.14E-01	1.00E+00	2.46E-01	1.00E+00	2.10E-01	1.00E+00	-7.01E-02	1.00E+00
ENSBTAG00000029760	0.00E+00	1.00E+00	0.00E+00	1.00E+00	-5.13E-15	1.00E+00	0.00E+00	1.00E+00	0.00E+00	1.00E+00
ENSBTAG00000040439	0.00E+00	1.00E+00	1.98E+00	4.43E-01	-5.13E-15	1.00E+00	0.00E+00	1.00E+00	0.00E+00	1.00E+00
ENSBTAG00000018616	-2.93E-02	1.00E+00	-5.18E-01	6.80E-03	-2.21E-01	3.68E-01	4.73E-02	1.00E+00	-5.50E-01	4.76E-02

ENSBTAG00000011812	1.51E-01	1.00E+00	1.19E+00	2.77E-09	1.24E+00	4.05E-10	7.76E-01	1.98E-04	-5.43E-01	1.28E-01
ENSBTAG00000015022	4.27E-01	5.76E-01	1.07E+00	1.15E-04	1.36E+00	5.61E-07	1.34E+00	1.17E-06	-8.43E-02	1.00E+00
ENSBTAG00000012876	2.44E-01	7.70E-01	1.07E+00	2.22E-08	1.36E+00	4.84E-13	1.18E+00	6.57E-10	-2.51E-01	7.62E-01
ENSBTAG00000043091	1.92E+00	8.23E-01	0.00E+00	1.00E+00	-5.13E-15	1.00E+00	0.00E+00	1.00E+00	0.00E+00	1.00E+00
ENSBTAG00000044823	0.00E+00	1.00E+00	0.00E+00	1.00E+00	-5.13E-15	1.00E+00	0.00E+00	1.00E+00	0.00E+00	1.00E+00
ENSBTAG00000021224	4.19E-01	5.21E-01	1.12E+00	1.09E-05	8.18E-01	1.81E-03	4.94E-01	1.01E-01	1.84E-01	1.00E+00
ENSBTAG00000046856	0.00E+00	1.00E+00	0.00E+00	1.00E+00	-5.13E-15	1.00E+00	0.00E+00	1.00E+00	0.00E+00	1.00E+00
ENSBTAG00000047951	-2.12E+00	1.63E-01	-1.13E+00	2.87E-01	-5.35E+00	8.94E-05	-2.32E+00	2.04E-02	-5.35E+00	4.99E-03
ENSBTAG00000021607	1.02E-01	1.00E+00	2.32E-01	7.50E-01	1.09E-02	1.00E+00	8.99E-02	1.00E+00	-6.13E-01	4.63E-01
ENSBTAG00000021606	2.00E-01	1.00E+00	7.52E-01	3.94E-03	6.21E-01	2.08E-02	7.15E-01	7.16E-03	-1.03E-01	1.00E+00
ENSBTAG00000007704	1.14E+00	8.23E-01	-4.19E-01	1.00E+00	2.20E-01	1.00E+00	9.35E-01	5.65E-01	6.84E-03	1.00E+00
ENSBTAG00000017026	2.11E+00	1.01E-01	2.10E+00	2.21E-02	1.10E+00	3.76E-01	1.85E+00	5.49E-02	1.07E+00	8.00E-01
ENSBTAG00000036375	0.00E+00	1.00E+00	0.00E+00	1.00E+00	-5.13E-15	1.00E+00	0.00E+00	1.00E+00	0.00E+00	1.00E+00
ENSBTAG00000029651	-1.91E+00	8.23E-01	-1.91E+00	4.69E-01	-1.91E+00	4.28E-01	-1.91E+00	4.39E-01	1.77E-02	1.00E+00
ENSBTAG00000039486	4.69E-01	8.23E-01	-4.36E-01	5.36E-01	-1.10E+00	3.76E-02	-1.01E+00	6.27E-02	3.85E-01	8.97E-01
ENSBTAG00000000494	7.53E-01	4.75E-01	5.18E-02	1.00E+00	-9.30E-01	1.08E-01	4.31E-01	5.41E-01	9.36E-01	3.11E-01
ENSBTAG00000007306	2.18E-01	1.00E+00	-4.26E-01	8.05E-01	1.36E-01	1.00E+00	9.42E-01	1.94E-01	-2.67E-01	1.00E+00
ENSBTAG00000005229	0.00E+00	1.00E+00	0.00E+00	1.00E+00	-5.13E-15	1.00E+00	0.00E+00	1.00E+00	0.00E+00	1.00E+00
ENSBTAG00000043799	0.00E+00	1.00E+00	0.00E+00	1.00E+00	-5.13E-15	1.00E+00	0.00E+00	1.00E+00	0.00E+00	1.00E+00
ENSBTAG00000016770	5.34E-01	8.23E-01	-8.95E-01	1.68E-01	-8.25E-02	1.00E+00	-2.26E-01	9.53E-01	4.52E-01	8.71E-01
ENSBTAG00000016771	-4.68E-02	1.00E+00	-2.43E+00	7.48E-17	-1.53E+00	9.85E-08	-1.48E+00	3.46E-07	-1.32E+00	1.83E-04
ENSBTAG00000042944	0.00E+00	1.00E+00	0.00E+00	1.00E+00	-5.13E-15	1.00E+00	0.00E+00	1.00E+00	0.00E+00	1.00E+00
ENSBTAG00000046468	0.00E+00	1.00E+00	0.00E+00	1.00E+00	-5.13E-15	1.00E+00	0.00E+00	1.00E+00	0.00E+00	1.00E+00
ENSBTAG00000047114	0.00E+00	1.00E+00	0.00E+00	1.00E+00	-5.13E-15	1.00E+00	0.00E+00	1.00E+00	0.00E+00	1.00E+00
ENSBTAG00000042330	0.00E+00	1.00E+00	0.00E+00	1.00E+00	-5.13E-15	1.00E+00	0.00E+00	1.00E+00	0.00E+00	1.00E+00
ENSBTAG00000044319	1.91E+00	8.23E-01	0.00E+00	1.00E+00	-5.13E-15	1.00E+00	0.00E+00	1.00E+00	0.00E+00	1.00E+00
ENSBTAG00000026505	-6.67E-01	6.26E-01	-6.20E-01	3.17E-01	-1.74E+00	7.77E-04	-1.01E+00	6.19E-02	-1.43E+00	7.31E-02
ENSBTAG00000012124	6.09E-01	4.30E-01	2.30E+00	7.39E-11	6.95E-01	8.06E-02	4.38E-01	3.66E-01	-3.05E-01	9.22E-01
ENSBTAG00000047548	1.46E-02	1.00E+00	9.59E-02	1.00E+00	-1.68E-01	1.00E+00	-8.22E-01	5.60E-01	1.55E-01	1.00E+00
ENSBTAG00000014248	3.43E-01	8.64E-01	3.63E-01	4.63E-01	8.22E-01	2.19E-02	1.44E+00	1.33E-05	2.28E-01	1.00E+00
ENSBTAG00000013426	-3.44E-01	8.82E-01	-1.17E+00	1.57E-03	-6.57E-01	1.05E-01	-6.75E-01	1.01E-01	-6.60E-01	4.29E-01
ENSBTAG00000036766	0.00E+00	1.00E+00	0.00E+00	1.00E+00	-5.13E-15	1.00E+00	0.00E+00	1.00E+00	0.00E+00	1.00E+00
ENSBTAG00000013790	-3.37E-01	7.51E-01	-5.74E-01	5.19E-02	-5.93E-01	4.16E-02	-1.08E-01	9.94E-01	1.42E-01	1.00E+00
ENSBTAG00000043083	0.00E+00	1.00E+00	0.00E+00	1.00E+00	-5.13E-15	1.00E+00	0.00E+00	1.00E+00	0.00E+00	1.00E+00
ENSBTAG00000001001	6.21E-01	6.07E-01	-2.46E-01	8.89E-01	-1.42E-02	1.00E+00	2.45E-01	8.61E-01	-8.62E-01	4.71E-01

ENSBTAG00000017745	8.44E-01	2.38E-02	1.10E+00	9.39E-05	6.30E-01	4.01E-02	1.08E+00	1.56E-04	3.50E-01	7.71E-01
ENSBTAG00000004351	2.10E-01	1.00E+00	-4.08E-01	3.03E-01	6.29E-01	4.70E-02	8.07E-01	8.17E-03	7.75E-01	1.03E-01
ENSBTAG00000008871	4.31E-01	1.00E+00	-9.09E-02	1.00E+00	-4.81E-01	1.00E+00	7.85E-02	1.00E+00	-1.89E+00	7.37E-01
ENSBTAG00000033313	-1.76E-01	9.78E-01	-9.08E-01	1.82E-05	-7.36E-01	5.67E-04	-6.69E-01	2.33E-03	-3.64E-01	4.85E-01
ENSBTAG00000044567	0.00E+00	1.00E+00	0.00E+00	1.00E+00	-5.13E-15	1.00E+00	0.00E+00	1.00E+00	0.00E+00	1.00E+00
ENSBTAG00000010526	-1.91E-01	1.00E+00	-5.15E-01	9.30E-02	-5.50E-01	6.71E-02	-4.53E-01	1.64E-01	1.79E-01	1.00E+00
ENSBTAG00000010518	-1.51E-01	1.00E+00	-9.02E-01	1.32E-04	-6.97E-01	4.05E-03	-5.85E-01	2.19E-02	-1.88E-01	9.66E-01
ENSBTAG00000010510	-1.66E-01	9.56E-01	3.71E-02	1.00E+00	6.00E-01	1.92E-03	5.50E-01	6.01E-03	-2.34E-01	7.79E-01
ENSBTAG00000047594	-1.91E+00	8.23E-01	1.77E+00	3.25E-01	6.50E-01	9.78E-01	1.50E+00	4.49E-01	-1.91E+00	8.00E-01
ENSBTAG00000047087	0.00E+00	1.00E+00	0.00E+00	1.00E+00	-5.13E-15	1.00E+00	3.41E+00	8.27E-02	1.91E+00	8.00E-01
ENSBTAG00000030051	1.92E+00	8.23E-01	0.00E+00	1.00E+00	-5.13E-15	1.00E+00	1.76E+00	4.97E-01	0.00E+00	1.00E+00
ENSBTAG00000030027	0.00E+00	1.00E+00	0.00E+00	1.00E+00	-5.13E-15	1.00E+00	1.77E+00	4.97E-01	0.00E+00	1.00E+00
ENSBTAG00000030121	0.00E+00	1.00E+00	0.00E+00	1.00E+00	-5.13E-15	1.00E+00	0.00E+00	1.00E+00	0.00E+00	1.00E+00
ENSBTAG00000021960	-3.30E-02	1.00E+00	-6.03E-01	1.92E-02	-1.40E+00	4.31E-09	-8.19E-01	9.46E-04	-7.50E-01	3.60E-02
ENSBTAG00000021959	1.63E+00	8.23E-01	2.48E+00	1.81E-01	8.27E-01	8.64E-01	1.26E+00	6.31E-01	-3.92E-02	1.00E+00
ENSBTAG00000021958	6.80E-02	1.00E+00	1.93E-01	1.00E+00	2.57E-01	9.60E-01	-1.35E+00	4.51E-02	-1.22E+00	3.33E-01
ENSBTAG00000027865	-6.37E-01	1.00E+00	-8.46E-01	5.72E-01	3.99E-01	8.87E-01	2.51E-01	1.00E+00	-9.27E-01	8.45E-01
ENSBTAG00000005164	-1.22E+00	3.73E-02	-1.47E+00	1.15E-03	-1.46E+00	7.92E-04	-2.19E+00	1.59E-06	-1.89E+00	1.12E-03
ENSBTAG00000004221	1.37E+00	2.11E-01	-1.48E+00	1.12E-01	6.01E-01	5.51E-01	7.12E-02	1.00E+00	-6.81E-01	8.92E-01
ENSBTAG00000003200	1.84E-01	1.00E+00	7.05E-01	1.26E-01	1.15E+00	2.74E-03	3.57E-01	5.80E-01	-3.77E-01	9.32E-01
ENSBTAG00000042490	0.00E+00	1.00E+00	0.00E+00	1.00E+00	-5.13E-15	1.00E+00	0.00E+00	1.00E+00	1.91E+00	8.00E-01
ENSBTAG00000004920	-4.49E-02	1.00E+00	-3.63E-01	2.62E-01	-6.50E-01	1.73E-02	-4.84E-01	1.06E-01	3.59E-02	1.00E+00
ENSBTAG00000027924	3.69E-01	4.52E-01	5.06E-01	3.15E-02	5.14E-01	2.77E-02	4.31E-01	8.38E-02	-3.84E-01	4.75E-01
ENSBTAG00000022714	0.00E+00	1.00E+00	1.98E+00	4.43E-01	-5.13E-15	1.00E+00	0.00E+00	1.00E+00	1.93E+00	8.00E-01
ENSBTAG00000019834	-3.17E-01	1.00E+00	-7.93E-01	9.97E-02	-1.19E+00	7.13E-03	1.10E-01	1.00E+00	-1.62E-02	1.00E+00
ENSBTAG00000003728	-6.39E-02	1.00E+00	-2.11E-01	5.94E-01	-6.09E-01	1.90E-02	-5.70E-01	3.42E-02	-2.45E-01	8.33E-01
ENSBTAG00000003329	4.80E-01	7.46E-01	1.51E+00	5.78E-05	4.52E+00	2.71E-31	3.43E+00	7.84E-20	1.64E-02	1.00E+00
ENSBTAG00000046997	1.91E+00	8.23E-01	0.00E+00	1.00E+00	-5.13E-15	1.00E+00	2.53E+00	2.71E-01	0.00E+00	1.00E+00
ENSBTAG00000005380	6.66E-01	3.25E-02	1.58E+00	1.69E-12	2.24E+00	2.16E-23	2.00E+00	5.19E-19	-1.98E-02	1.00E+00
ENSBTAG00000019289	1.34E+00	1.49E-07	3.99E+00	1.97E-58	4.08E+00	8.96E-61	2.82E+00	1.37E-32	2.08E-01	9.32E-01
ENSBTAG00000016525	6.83E-01	5.67E-02	2.35E+00	4.05E-22	1.75E+00	4.45E-13	1.82E+00	7.40E-14	1.14E+00	1.69E-04
ENSBTAG00000046575	0.00E+00	1.00E+00	0.00E+00	1.00E+00	-5.13E-15	1.00E+00	0.00E+00	1.00E+00	0.00E+00	1.00E+00
ENSBTAG00000003268	3.66E-01	4.41E-01	1.17E+00	1.39E-08	5.42E-01	1.64E-02	4.03E-01	1.05E-01	-1.79E-01	9.48E-01
ENSBTAG00000001241	5.37E-03	1.00E+00	8.81E-01	8.52E-01	-1.91E+00	4.28E-01	1.12E+00	6.68E-01	6.36E-03	1.00E+00
ENSBTAG00000028641	0.00E+00	1.00E+00	0.00E+00	1.00E+00	-5.13E-15	1.00E+00	0.00E+00	1.00E+00	0.00E+00	1.00E+00

ENSBTAG00000042274	0.00E+00	1.00E+00	0.00E+00	1.00E+00	-5.13E-15	1.00E+00	0.00E+00	1.00E+00	1.93E+00	8.00E-01
ENSBTAG00000004066	3.32E-01	6.69E-01	-7.32E-01	4.20E-03	-1.71E+00	2.24E-12	-1.51E+00	7.67E-10	-2.24E-01	8.97E-01
ENSBTAG00000044320	0.00E+00	1.00E+00	0.00E+00	1.00E+00	-5.13E-15	1.00E+00	0.00E+00	1.00E+00	0.00E+00	1.00E+00
ENSBTAG00000044010	1.37E+00	2.40E-01	2.65E-01	1.00E+00	7.26E-01	4.52E-01	1.25E+00	1.01E-01	9.62E-01	6.33E-01
ENSBTAG00000043948	6.41E-01	8.81E-01	7.83E-01	3.80E-01	-8.61E-01	3.71E-01	8.62E-01	3.04E-01	-4.75E-01	1.00E+00
ENSBTAG00000044496	0.00E+00	1.00E+00	0.00E+00	1.00E+00	-5.13E-15	1.00E+00	0.00E+00	1.00E+00	0.00E+00	1.00E+00
ENSBTAG00000000084	-1.02E-01	1.00E+00	2.72E-01	5.65E-01	7.44E-01	2.02E-02	6.58E-01	5.05E-02	-3.71E-01	7.78E-01
ENSBTAG00000011657	-3.00E-01	8.68E-01	-1.21E+00	7.71E-05	-1.83E+00	1.47E-09	-6.43E-01	5.94E-02	3.97E-02	1.00E+00
ENSBTAG00000042246	0.00E+00	1.00E+00	0.00E+00	1.00E+00	-5.13E-15	1.00E+00	0.00E+00	1.00E+00	0.00E+00	1.00E+00
ENSBTAG00000033187	0.00E+00	1.00E+00	0.00E+00	1.00E+00	-5.13E-15	1.00E+00	0.00E+00	1.00E+00	0.00E+00	1.00E+00
ENSBTAG00000011885	-1.05E-01	1.00E+00	-1.04E+00	4.88E-04	-1.86E+00	1.56E-10	-1.59E+00	6.17E-08	2.23E-01	9.98E-01
ENSBTAG00000020376	1.05E-01	1.00E+00	1.42E-01	6.79E-01	3.06E-01	1.65E-01	2.60E-01	2.76E-01	-1.04E-01	1.00E+00
ENSBTAG00000007461	-5.71E-02	1.00E+00	-1.20E+00	5.92E-07	-7.37E-01	2.46E-03	-5.06E-01	5.76E-02	1.62E-01	1.00E+00
ENSBTAG00000001457	-2.78E-01	9.36E-01	-1.97E+00	6.81E-10	-1.08E+00	7.13E-04	-4.92E-01	1.98E-01	-2.48E-01	9.68E-01
ENSBTAG00000001557	2.88E-01	9.67E-01	-2.16E+00	3.06E-07	-2.08E+00	1.97E-07	-1.82E+00	4.10E-06	7.86E-01	1.90E-01
ENSBTAG00000011839	-2.31E-01	1.00E+00	-2.04E+00	4.29E-03	-1.98E+00	5.43E-03	-1.14E+00	1.61E-01	-8.35E-01	7.68E-01
ENSBTAG00000006233	-6.81E-01	1.00E+00	-1.12E+00	5.37E-01	6.66E-02	1.00E+00	-1.39E+00	4.05E-01	-3.01E-01	1.00E+00
ENSBTAG00000015504	3.33E-01	5.65E-01	1.84E+00	8.74E-20	1.50E+00	9.06E-14	8.47E-01	7.07E-05	-3.24E-02	1.00E+00
ENSBTAG00000042376	3.22E+00	3.75E-01	2.79E+00	2.10E-01	-5.13E-15	1.00E+00	1.76E+00	4.97E-01	0.00E+00	1.00E+00
ENSBTAG00000033224	-2.87E-01	1.00E+00	3.21E-01	9.16E-01	6.76E-02	1.00E+00	2.85E-01	9.55E-01	1.13E-01	1.00E+00
ENSBTAG00000033221	-3.49E-01	5.96E-01	-1.34E+00	1.11E-08	-3.02E+00	4.65E-36	-3.94E+00	5.47E-56	-9.26E-01	3.18E-03
ENSBTAG00000001335	2.43E-01	8.23E-01	-3.11E-02	1.00E+00	3.25E-01	2.50E-01	6.51E-02	1.00E+00	3.84E-01	4.99E-01
ENSBTAG00000016981	5.25E-01	9.94E-02	7.20E-01	7.65E-04	5.84E-01	7.97E-03	2.72E-01	3.37E-01	2.34E-01	8.00E-01
ENSBTAG00000004929	9.04E-01	6.29E-02	1.49E+00	9.18E-06	1.19E+00	4.68E-04	1.33E+00	1.07E-04	5.24E-01	6.01E-01
ENSBTAG00000033186	1.17E-01	1.00E+00	-3.03E-01	4.69E-01	2.04E-01	7.08E-01	4.00E-01	2.68E-01	-2.92E-01	8.33E-01
ENSBTAG00000010822	-6.61E-02	1.00E+00	-9.33E-01	2.76E-01	-1.35E+00	7.98E-02	-1.03E+00	2.17E-01	-1.04E+00	6.01E-01
ENSBTAG00000014177	5.53E-01	1.81E-01	1.95E-01	6.64E-01	-4.48E-01	1.20E-01	-5.93E-01	3.04E-02	7.98E-01	2.66E-02
ENSBTAG00000001997	4.68E-01	8.84E-01	-7.10E-01	2.42E-01	-1.82E+00	3.26E-04	-2.77E+00	1.01E-07	4.52E-01	8.78E-01
ENSBTAG00000011766	6.03E-01	7.16E-01	4.17E-01	5.63E-01	4.69E-01	4.79E-01	2.90E-01	8.03E-01	9.18E-01	3.55E-01
ENSBTAG00000014374	-3.17E-01	9.00E-01	-1.36E+00	6.49E-05	-1.45E+00	1.53E-05	-1.14E+00	1.08E-03	2.01E-01	1.00E+00
ENSBTAG00000005142	1.06E-01	1.00E+00	4.08E-01	1.35E-01	-3.76E-02	1.00E+00	-8.26E-02	1.00E+00	-3.72E-01	5.71E-01
ENSBTAG00000042297	0.00E+00	1.00E+00	0.00E+00	1.00E+00	-5.13E-15	1.00E+00	0.00E+00	1.00E+00	0.00E+00	1.00E+00
ENSBTAG00000000013	-3.67E-01	4.92E-01	-6.36E-01	6.65E-03	-4.92E-01	4.41E-02	-5.19E-01	3.60E-02	-3.99E-01	4.51E-01
ENSBTAG00000000012	1.80E-01	1.00E+00	-2.62E-01	6.52E-01	4.32E-01	2.99E-01	4.76E-01	2.45E-01	5.46E-01	5.05E-01
ENSBTAG00000014972	6.99E-01	3.11E-01	4.96E-01	2.83E-01	-1.59E-01	9.71E-01	-1.23E-01	1.00E+00	-4.03E-01	8.00E-01

ENSBTAG00000046658	0.00E+00	1.00E+00	0.00E+00	1.00E+00	-5.13E-15	1.00E+00	0.00E+00	1.00E+00	0.00E+00	1.00E+00
ENSBTAG00000043592	0.00E+00	1.00E+00	0.00E+00	1.00E+00	-5.13E-15	1.00E+00	0.00E+00	1.00E+00	0.00E+00	1.00E+00
ENSBTAG00000043758	0.00E+00	1.00E+00	0.00E+00	1.00E+00	-5.13E-15	1.00E+00	0.00E+00	1.00E+00	0.00E+00	1.00E+00
ENSBTAG00000016152	-4.05E-01	4.60E-01	-4.97E-01	6.43E-02	7.65E-02	1.00E+00	-3.10E-01	3.37E-01	-4.19E-01	4.76E-01
ENSBTAG00000016149	4.89E-01	2.42E-01	1.17E-01	9.20E-01	-5.05E-01	5.29E-02	-7.57E-01	2.14E-03	4.72E-01	3.51E-01
ENSBTAG00000001492	5.01E-01	1.10E-01	1.34E+00	6.63E-12	7.18E-01	4.66E-04	2.96E-01	2.62E-01	-4.15E-01	3.65E-01
ENSBTAG00000006697	4.55E-01	3.59E-01	7.20E-01	4.50E-03	2.75E-01	4.28E-01	1.60E-01	7.82E-01	-2.08E-01	9.38E-01
ENSBTAG00000033107	1.43E+00	3.93E-03	3.13E+00	4.25E-14	1.95E+00	1.50E-06	1.71E+00	3.57E-05	5.60E-01	6.67E-01
ENSBTAG00000010423	-4.24E-02	1.00E+00	5.45E-01	1.95E-01	-6.74E-02	1.00E+00	4.77E-01	2.90E-01	-9.41E-02	1.00E+00
ENSBTAG00000019595	-1.27E-01	1.00E+00	-8.76E-01	1.96E-01	-1.21E+00	4.97E-02	-2.61E-01	9.29E-01	1.12E-01	1.00E+00
ENSBTAG00000044244	0.00E+00	1.00E+00	0.00E+00	1.00E+00	-5.13E-15	1.00E+00	0.00E+00	1.00E+00	0.00E+00	1.00E+00
ENSBTAG00000042537	0.00E+00	1.00E+00	0.00E+00	1.00E+00	-5.13E-15	1.00E+00	0.00E+00	1.00E+00	0.00E+00	1.00E+00
ENSBTAG00000042084	0.00E+00	1.00E+00	0.00E+00	1.00E+00	-5.13E-15	1.00E+00	0.00E+00	1.00E+00	0.00E+00	1.00E+00
ENSBTAG00000005176	3.88E-01	9.98E-01	1.80E+00	4.06E-05	7.78E-01	1.52E-01	-6.27E-01	3.85E-01	3.56E-01	1.00E+00
ENSBTAG00000033095	-1.15E-01	1.00E+00	6.82E-01	9.80E-04	7.96E-01	7.87E-05	6.99E-01	7.91E-04	-1.85E-01	9.07E-01
ENSBTAG00000002458	2.26E-01	8.86E-01	3.79E-01	1.74E-01	5.85E-01	1.77E-02	2.88E-01	3.69E-01	9.90E-02	1.00E+00
ENSBTAG00000045286	0.00E+00	1.00E+00	0.00E+00	1.00E+00	-5.13E-15	1.00E+00	0.00E+00	1.00E+00	0.00E+00	1.00E+00
ENSBTAG00000000586	2.75E-01	1.00E+00	-2.55E-02	1.00E+00	1.46E-02	1.00E+00	-3.26E-01	6.26E-01	-1.31E-01	1.00E+00
ENSBTAG00000000579	2.52E-01	9.17E-01	9.18E-01	8.20E-04	7.15E-01	1.18E-02	5.34E-01	8.57E-02	-1.13E-01	1.00E+00
ENSBTAG00000047208	9.13E-02	1.00E+00	1.07E+00	1.26E-03	1.04E+00	1.47E-03	9.20E-01	7.32E-03	3.84E-01	8.00E-01
ENSBTAG00000018245	1.69E-01	1.00E+00	-4.11E-01	4.81E-01	-7.21E-01	1.27E-01	-5.45E-01	2.99E-01	-1.30E+00	4.48E-02
ENSBTAG00000008430	-4.76E-01	7.53E-01	-1.13E+00	6.11E-03	-8.88E-01	3.13E-02	-1.39E+00	6.34E-04	-6.87E-01	4.43E-01
ENSBTAG00000011334	-1.69E-01	1.00E+00	-2.42E-01	6.53E-01	6.83E-01	3.92E-02	1.25E+00	4.60E-05	-1.29E-02	1.00E+00
ENSBTAG00000013562	-5.54E-01	5.72E-01	-2.08E+00	3.64E-08	-1.36E+00	2.39E-04	-1.21E+00	1.33E-03	3.16E-01	9.04E-01
ENSBTAG00000020142	-2.71E-01	1.00E+00	4.72E-01	2.56E-01	1.43E-01	9.74E-01	8.93E-01	1.07E-02	-8.09E-02	1.00E+00
ENSBTAG00000002701	-1.22E-01	1.00E+00	-7.38E-01	5.01E-02	-1.48E+00	1.57E-05	-9.82E-01	6.80E-03	4.63E-01	6.84E-01
ENSBTAG00000012688	-6.00E-01	1.00E+00	-2.55E+00	7.28E-03	-3.76E+00	8.80E-05	-3.46E+00	3.46E-04	-3.68E-01	1.00E+00
ENSBTAG00000019975	9.99E-01	1.81E-01	1.42E+00	2.05E-03	5.14E-01	4.19E-01	-9.72E-02	1.00E+00	-5.08E-01	8.00E-01
ENSBTAG00000023529	-1.12E-01	1.00E+00	-1.66E+00	5.76E-07	-2.53E+00	5.51E-14	-2.42E+00	5.54E-13	-2.86E-01	9.09E-01
ENSBTAG00000025035	-3.47E-02	1.00E+00	-1.07E+00	2.16E-03	-2.87E+00	5.17E-17	-4.01E+00	7.14E-29	1.46E+00	5.27E-04
ENSBTAG00000007690	9.77E-02	1.00E+00	-9.54E-01	1.38E-05	-1.96E+00	9.46E-20	-2.09E+00	5.00E-22	2.61E-02	1.00E+00
ENSBTAG00000015648	1.56E-01	1.00E+00	9.19E-01	9.72E-06	1.34E+00	2.70E-11	9.62E-01	3.72E-06	-1.54E-01	1.00E+00
ENSBTAG00000019211	5.68E-01	7.49E-02	2.13E+00	2.65E-24	2.09E+00	1.38E-23	1.05E+00	1.08E-06	-6.18E-01	8.22E-02
ENSBTAG00000019209	-6.61E-02	1.00E+00	1.03E+00	5.18E-07	1.54E+00	1.03E-14	1.10E+00	6.47E-08	-1.98E-01	8.94E-01
ENSBTAG00000032933	-4.60E-02	1.00E+00	-1.81E+00	3.49E-06	-2.63E+00	5.36E-11	-2.09E+00	6.43E-08	-1.18E-01	1.00E+00

ENSBTAG00000037206	0.00E+00	1.00E+00	0.00E+00	1.00E+00	-5.13E-15	1.00E+00	0.00E+00	1.00E+00	0.00E+00	1.00E+00
ENSBTAG00000007071	5.04E-01	2.34E-01	6.31E-01	1.34E-02	6.34E-01	1.26E-02	-6.66E-02	1.00E+00	2.64E-01	8.00E-01
ENSBTAG00000017071	-4.09E-02	1.00E+00	-1.47E+00	1.06E-01	-1.43E+00	1.03E-01	-1.62E+00	6.65E-02	5.82E-01	9.13E-01
ENSBTAG00000043322	0.00E+00	1.00E+00	0.00E+00	1.00E+00	-5.13E-15	1.00E+00	0.00E+00	1.00E+00	0.00E+00	1.00E+00
ENSBTAG00000021813	4.02E-02	1.00E+00	-1.71E+00	3.86E-13	-3.77E+00	4.75E-51	-2.65E+00	1.44E-28	4.28E-01	4.46E-01
ENSBTAG00000018235	1.20E-01	1.00E+00	-2.36E+00	2.11E-19	-3.10E+00	5.85E-31	-3.34E+00	1.30E-34	6.13E-01	1.83E-01
ENSBTAG00000039929	3.22E+00	3.75E-01	0.00E+00	1.00E+00	3.05E+00	1.36E-01	0.00E+00	1.00E+00	1.91E+00	8.00E-01
ENSBTAG00000012558	-1.69E-01	1.00E+00	-5.89E-01	2.10E-02	-8.61E-01	3.35E-04	-4.24E-01	1.29E-01	-1.60E-01	1.00E+00
ENSBTAG00000014261	1.13E+00	6.47E-07	3.14E+00	2.28E-48	2.74E+00	2.20E-38	1.92E+00	2.58E-20	-1.81E-01	9.37E-01
ENSBTAG00000007665	-1.16E+00	4.17E-01	-1.97E+00	5.23E-03	-1.97E+00	5.01E-03	-6.60E-01	4.97E-01	1.71E-01	1.00E+00
ENSBTAG00000021965	1.46E-02	1.00E+00	6.89E-03	1.00E+00	-4.96E-02	1.00E+00	1.06E-01	9.40E-01	-5.28E-01	2.03E-01
ENSBTAG00000008414	1.78E-01	9.68E-01	8.83E-01	2.07E-05	1.29E+00	1.69E-10	1.31E+00	1.15E-10	3.14E-02	1.00E+00
ENSBTAG00000043775	0.00E+00	1.00E+00	0.00E+00	1.00E+00	-5.13E-15	1.00E+00	0.00E+00	1.00E+00	0.00E+00	1.00E+00
ENSBTAG00000012278	-7.22E-01	4.70E-02	-2.05E+00	3.43E-14	-1.07E+00	3.80E-05	-9.10E-01	6.58E-04	-3.16E-01	7.77E-01
ENSBTAG00000032853	-2.70E+00	5.76E-01	-2.70E+00	2.42E-01	-9.18E-01	8.09E-01	-9.41E-01	8.06E-01	-2.70E+00	6.01E-01
ENSBTAG00000032848	1.13E-01	1.00E+00	1.01E+00	8.93E-08	6.85E-01	4.05E-04	4.59E-01	3.03E-02	-7.78E-02	1.00E+00
ENSBTAG00000014455	-1.09E-02	1.00E+00	1.01E+00	1.64E-04	-7.43E-01	1.05E-02	-9.75E-01	6.51E-04	-1.89E-01	1.00E+00
ENSBTAG00000038985	5.11E-01	1.00E+00	9.27E-02	1.00E+00	-9.22E-01	8.09E-01	-2.70E+00	2.06E-01	5.15E-01	1.00E+00
ENSBTAG00000017569	6.12E-02	1.00E+00	2.64E-01	4.77E-01	6.22E-02	1.00E+00	-2.89E-01	4.42E-01	1.39E-01	1.00E+00
ENSBTAG00000017551	8.33E-02	1.00E+00	-2.82E-01	3.65E-01	-7.46E-02	1.00E+00	-7.09E-01	3.31E-03	6.73E-02	1.00E+00
ENSBTAG00000012992	-1.58E-01	1.00E+00	6.98E-01	5.18E-01	1.62E-01	1.00E+00	-5.14E-01	7.69E-01	-1.83E+00	3.36E-01
ENSBTAG00000040572	0.00E+00	1.00E+00	0.00E+00	1.00E+00	-5.13E-15	1.00E+00	0.00E+00	1.00E+00	0.00E+00	1.00E+00
ENSBTAG00000043813	0.00E+00	1.00E+00	0.00E+00	1.00E+00	-5.13E-15	1.00E+00	0.00E+00	1.00E+00	0.00E+00	1.00E+00
ENSBTAG00000043184	0.00E+00	1.00E+00	0.00E+00	1.00E+00	-5.13E-15	1.00E+00	0.00E+00	1.00E+00	0.00E+00	1.00E+00
ENSBTAG00000018234	1.33E-02	1.00E+00	7.64E-02	1.00E+00	6.40E-01	9.79E-01	-1.47E-01	1.00E+00	8.00E-01	1.00E+00
ENSBTAG00000048105	0.00E+00	1.00E+00	0.00E+00	1.00E+00	-5.13E-15	1.00E+00	0.00E+00	1.00E+00	0.00E+00	1.00E+00
ENSBTAG00000045813	8.14E-01	6.72E-01	-6.06E-01	6.16E-01	-2.35E+00	1.10E-02	-1.05E+00	2.64E-01	1.03E+00	4.65E-01
ENSBTAG00000002845	-2.23E-01	1.00E+00	9.46E-02	1.00E+00	-1.38E+00	2.14E-01	6.46E-01	6.31E-01	1.33E-01	1.00E+00
ENSBTAG00000042459	1.92E+00	8.23E-01	0.00E+00	1.00E+00	-5.13E-15	1.00E+00	1.76E+00	4.97E-01	0.00E+00	1.00E+00
ENSBTAG00000025893	-4.70E-01	1.00E+00	-3.93E-01	9.33E-01	-8.55E-01	4.79E-01	-3.34E-01	9.76E-01	-3.03E-01	1.00E+00
ENSBTAG00000013660	1.99E+00	3.33E-01	3.73E+00	4.71E-06	3.77E+00	1.85E-06	3.03E+00	8.64E-04	2.15E+00	3.09E-01
ENSBTAG00000037844	-4.60E-01	1.00E+00	-1.40E-01	1.00E+00	-7.01E-01	3.95E-01	5.05E-01	5.70E-01	-1.86E-01	1.00E+00
ENSBTAG00000043021	0.00E+00	1.00E+00	0.00E+00	1.00E+00	-5.13E-15	1.00E+00	0.00E+00	1.00E+00	0.00E+00	1.00E+00
ENSBTAG00000045304	0.00E+00	1.00E+00	0.00E+00	1.00E+00	-5.13E-15	1.00E+00	0.00E+00	1.00E+00	0.00E+00	1.00E+00
ENSBTAG00000012971	-1.91E+00	8.23E-01	-1.91E+00	4.69E-01	-1.91E+00	4.28E-01	-1.46E-01	1.00E+00	-1.91E+00	8.00E-01

ENSBTAG00000005491	0.00E+00	1.00E+00	0.00E+00	1.00E+00	-5.13E-15	1.00E+00	0.00E+00	1.00E+00	0.00E+00	1.00E+00
ENSBTAG00000033565	6.43E-01	6.35E-02	8.62E-01	3.93E-04	5.06E-01	5.86E-02	1.91E-01	6.68E-01	-1.77E-01	1.00E+00
ENSBTAG00000044221	0.00E+00	1.00E+00	0.00E+00	1.00E+00	-5.13E-15	1.00E+00	0.00E+00	1.00E+00	0.00E+00	1.00E+00
ENSBTAG00000042103	0.00E+00	1.00E+00	0.00E+00	1.00E+00	-5.13E-15	1.00E+00	0.00E+00	1.00E+00	0.00E+00	1.00E+00
ENSBTAG00000020126	-1.65E-01	1.00E+00	-1.06E+00	3.55E-06	-1.74E+00	1.18E-14	-9.68E-01	2.92E-05	-2.19E-03	1.00E+00
ENSBTAG00000016444	4.29E-01	9.90E-01	1.99E+00	1.03E-04	3.04E+00	1.49E-09	3.28E+00	1.15E-10	-1.30E-01	1.00E+00
ENSBTAG00000016441	-2.08E-01	9.03E-01	1.26E+00	2.85E-09	1.15E+00	5.66E-08	3.12E-01	2.65E-01	7.34E-02	1.00E+00
ENSBTAG00000037576	-1.69E-01	1.00E+00	-2.66E-01	1.00E+00	-3.53E-01	9.71E-01	6.72E-01	5.40E-01	-8.11E-01	8.99E-01
ENSBTAG00000003219	-1.17E-02	1.00E+00	1.01E-01	1.00E+00	-4.98E-01	2.13E-01	-1.77E-01	8.68E-01	1.06E+00	1.13E-02
ENSBTAG00000045215	0.00E+00	1.00E+00	0.00E+00	1.00E+00	-5.13E-15	1.00E+00	0.00E+00	1.00E+00	0.00E+00	1.00E+00
ENSBTAG00000013391	6.20E-01	2.23E-01	1.49E+00	2.30E-07	1.29E+00	7.88E-06	1.11E+00	2.00E-04	1.01E+00	1.20E-02
ENSBTAG00000045869	0.00E+00	1.00E+00	0.00E+00	1.00E+00	-5.13E-15	1.00E+00	0.00E+00	1.00E+00	0.00E+00	1.00E+00
ENSBTAG00000003186	4.24E-01	2.94E-01	7.79E-01	2.92E-04	9.19E-01	1.21E-05	1.01E+00	1.46E-06	5.64E-01	1.14E-01
ENSBTAG00000000672	1.62E-01	1.00E+00	4.07E-01	4.10E-01	7.56E-01	4.98E-02	4.55E-01	3.33E-01	-3.84E-01	8.00E-01
ENSBTAG00000005514	2.43E-01	8.17E-01	1.31E-01	8.02E-01	3.38E-01	1.72E-01	7.01E-01	9.98E-04	2.62E-01	7.63E-01
ENSBTAG00000039745	0.00E+00	1.00E+00	0.00E+00	1.00E+00	-5.13E-15	1.00E+00	0.00E+00	1.00E+00	0.00E+00	1.00E+00
ENSBTAG00000043365	0.00E+00	1.00E+00	0.00E+00	1.00E+00	-5.13E-15	1.00E+00	0.00E+00	1.00E+00	0.00E+00	1.00E+00
ENSBTAG00000021972	-1.68E-01	1.00E+00	-1.12E-01	1.00E+00	-1.24E+00	5.20E-02	-1.53E-01	1.00E+00	-7.04E-02	1.00E+00
ENSBTAG00000042965	0.00E+00	1.00E+00	0.00E+00	1.00E+00	-5.13E-15	1.00E+00	0.00E+00	1.00E+00	0.00E+00	1.00E+00
ENSBTAG00000038035	0.00E+00	1.00E+00	0.00E+00	1.00E+00	-5.13E-15	1.00E+00	0.00E+00	1.00E+00	0.00E+00	1.00E+00
ENSBTAG00000047588	0.00E+00	1.00E+00	0.00E+00	1.00E+00	-5.13E-15	1.00E+00	0.00E+00	1.00E+00	0.00E+00	1.00E+00
ENSBTAG00000017222	-2.99E-01	1.00E+00	-6.88E-02	1.00E+00	-5.28E-01	4.54E-01	1.53E-01	1.00E+00	2.49E-02	1.00E+00
ENSBTAG00000047394	0.00E+00	1.00E+00	0.00E+00	1.00E+00	-5.13E-15	1.00E+00	0.00E+00	1.00E+00	0.00E+00	1.00E+00
ENSBTAG00000045141	0.00E+00	1.00E+00	0.00E+00	1.00E+00	-5.13E-15	1.00E+00	0.00E+00	1.00E+00	0.00E+00	1.00E+00
ENSBTAG00000042090	0.00E+00	1.00E+00	0.00E+00	1.00E+00	-5.13E-15	1.00E+00	0.00E+00	1.00E+00	0.00E+00	1.00E+00
ENSBTAG00000006346	-2.49E-02	1.00E+00	-3.51E-01	2.66E-01	-5.65E-01	3.78E-02	-1.73E-01	7.49E-01	1.32E-01	1.00E+00
ENSBTAG00000011337	7.79E-01	5.44E-01	2.65E-01	9.09E-01	-3.97E-01	7.02E-01	-7.51E-01	3.11E-01	2.83E-01	1.00E+00
ENSBTAG00000003539	-1.51E-01	1.00E+00	-2.27E-01	1.00E+00	4.66E-01	6.02E-01	-1.07E+00	1.81E-01	7.96E-01	6.11E-01
ENSBTAG00000003536	-1.34E-02	1.00E+00	-4.52E-01	9.85E-02	-5.32E-01	4.16E-02	9.07E-03	1.00E+00	5.69E-01	1.91E-01
ENSBTAG00000022809	-4.06E-03	1.00E+00	-3.22E+00	1.25E-01	-6.62E-01	8.93E-01	1.11E+00	4.41E-01	-1.31E+00	9.00E-01
ENSBTAG00000010801	-3.94E-01	4.21E-01	-8.91E-01	8.65E-05	-1.97E+00	5.74E-19	-2.62E+00	2.85E-31	-2.81E-01	7.71E-01
ENSBTAG00000017889	1.60E-01	1.00E+00	1.49E+00	4.13E-11	1.29E+00	1.20E-08	1.13E+00	7.88E-07	-2.21E-01	8.68E-01
ENSBTAG00000017885	3.32E-01	5.40E-01	8.61E-01	3.26E-05	3.49E-01	1.60E-01	1.06E-02	1.00E+00	1.22E-01	1.00E+00
ENSBTAG00000043875	0.00E+00	1.00E+00	0.00E+00	1.00E+00	-5.13E-15	1.00E+00	0.00E+00	1.00E+00	0.00E+00	1.00E+00
ENSBTAG00000032508	0.00E+00	1.00E+00	0.00E+00	1.00E+00	-5.13E-15	1.00E+00	0.00E+00	1.00E+00	0.00E+00	1.00E+00

ENSBTAG00000044500	8.54E-01	3.76E-01	-1.54E+00	8.25E-03	-1.03E+00	7.63E-02	-1.21E+00	3.85E-02	4.89E-01	8.22E-01
ENSBTAG00000005021	3.57E-01	8.23E-01	1.09E+00	5.18E-04	4.03E-01	3.28E-01	2.26E-01	7.25E-01	6.90E-01	2.61E-01
ENSBTAG00000037147	0.00E+00	1.00E+00	0.00E+00	1.00E+00	-5.13E-15	1.00E+00	0.00E+00	1.00E+00	0.00E+00	1.00E+00
ENSBTAG00000044785	0.00E+00	1.00E+00	0.00E+00	1.00E+00	-5.13E-15	1.00E+00	0.00E+00	1.00E+00	0.00E+00	1.00E+00
ENSBTAG00000009401	5.50E-01	3.88E-01	1.75E+00	6.98E-09	2.29E+00	2.66E-14	1.69E+00	2.45E-08	3.92E-01	7.47E-01
ENSBTAG00000009400	8.36E-01	2.62E-02	2.04E+00	5.17E-14	3.31E+00	1.62E-33	2.69E+00	3.82E-23	4.10E-01	6.25E-01
ENSBTAG00000019210	2.71E+00	2.46E-03	3.84E+00	2.01E-07	3.20E+00	1.45E-05	3.16E+00	2.51E-05	2.34E+00	3.61E-02
ENSBTAG00000032442	-1.91E+00	8.23E-01	-1.91E+00	4.69E-01	-1.91E+00	4.28E-01	-1.47E-01	1.00E+00	8.22E-01	1.00E+00
ENSBTAG00000003381	-4.49E-01	2.31E-01	-3.24E-01	2.17E-01	-2.23E-01	4.70E-01	-8.02E-01	2.37E-04	-4.63E-01	2.82E-01
ENSBTAG00000043275	0.00E+00	1.00E+00	0.00E+00	1.00E+00	-5.13E-15	1.00E+00	0.00E+00	1.00E+00	0.00E+00	1.00E+00
ENSBTAG00000043281	0.00E+00	1.00E+00	0.00E+00	1.00E+00	-5.13E-15	1.00E+00	0.00E+00	1.00E+00	0.00E+00	1.00E+00
ENSBTAG00000015478	-3.57E-01	5.76E-01	-2.03E+00	3.74E-18	-3.28E+00	4.44E-41	-2.86E+00	6.55E-33	-1.54E-02	1.00E+00
ENSBTAG00000015466	-2.02E-01	9.43E-01	-4.12E-01	1.17E-01	4.07E-01	1.20E-01	3.85E-01	1.59E-01	-7.16E-02	1.00E+00
ENSBTAG00000043282	0.00E+00	1.00E+00	0.00E+00	1.00E+00	-5.13E-15	1.00E+00	0.00E+00	1.00E+00	0.00E+00	1.00E+00
ENSBTAG00000008130	1.96E-01	1.00E+00	1.32E+00	2.31E-08	1.34E+00	1.04E-08	7.73E-01	2.06E-03	-2.51E-01	8.20E-01
ENSBTAG00000018501	7.51E-02	1.00E+00	7.32E-01	2.05E-03	8.58E-01	2.13E-04	5.22E-01	4.30E-02	-3.05E-02	1.00E+00
ENSBTAG00000018460	-5.41E-01	7.46E-01	-3.82E-01	5.63E-01	-1.46E+00	1.83E-03	-1.74E+00	2.89E-04	-5.57E-01	7.27E-01
ENSBTAG00000045462	0.00E+00	1.00E+00	0.00E+00	1.00E+00	-5.13E-15	1.00E+00	0.00E+00	1.00E+00	0.00E+00	1.00E+00
ENSBTAG00000044671	0.00E+00	1.00E+00	0.00E+00	1.00E+00	-5.13E-15	1.00E+00	0.00E+00	1.00E+00	0.00E+00	1.00E+00
ENSBTAG00000000809	-8.72E-01	1.00E+00	-2.85E-01	1.00E+00	-1.03E+00	6.02E-01	-5.56E-01	9.24E-01	5.08E-06	1.00E+00
ENSBTAG00000047499	3.22E+00	3.75E-01	0.00E+00	1.00E+00	1.78E+00	4.79E-01	0.00E+00	1.00E+00	3.88E+00	2.05E-01
ENSBTAG00000046558	0.00E+00	1.00E+00	0.00E+00	1.00E+00	-5.13E-15	1.00E+00	0.00E+00	1.00E+00	0.00E+00	1.00E+00
ENSBTAG00000043909	0.00E+00	1.00E+00	0.00E+00	1.00E+00	-5.13E-15	1.00E+00	0.00E+00	1.00E+00	0.00E+00	1.00E+00
ENSBTAG00000015907	-8.66E-01	1.00E+00	-1.60E+00	4.10E-01	-5.28E-01	9.41E-01	-5.56E-01	9.24E-01	5.27E-03	1.00E+00
ENSBTAG00000009914	-6.02E-02	1.00E+00	-4.25E-01	2.00E-01	-4.49E-02	1.00E+00	-7.82E-03	1.00E+00	-6.66E-02	1.00E+00
ENSBTAG00000047906	2.52E-02	1.00E+00	-1.57E-01	7.97E-01	4.51E-02	1.00E+00	3.65E-01	2.40E-01	-3.28E-01	7.11E-01
ENSBTAG00000000944	3.11E-01	7.97E-01	-1.44E-01	8.76E-01	-4.50E-02	1.00E+00	-2.50E-01	5.39E-01	-5.07E-02	1.00E+00
ENSBTAG00000015843	1.52E-01	1.00E+00	-7.26E-01	4.69E-01	-7.14E-01	4.58E-01	4.76E-01	6.37E-01	-6.79E-01	8.44E-01
ENSBTAG00000001329	2.47E-01	8.53E-01	9.13E-01	1.80E-04	7.89E-01	1.41E-03	8.32E-01	8.75E-04	1.73E-01	1.00E+00
ENSBTAG00000012567	-1.71E-01	1.00E+00	7.06E-03	1.00E+00	-5.26E-01	4.42E-01	-4.65E-01	5.24E-01	-2.15E-02	1.00E+00
ENSBTAG00000012559	-3.81E-01	1.00E+00	-2.84E-01	1.00E+00	-1.66E-01	1.00E+00	-1.11E+00	4.04E-01	-1.81E+00	5.09E-01
ENSBTAG00000045695	6.92E-01	1.00E+00	7.64E-01	7.41E-01	7.59E-01	7.16E-01	1.81E-01	1.00E+00	-4.94E-01	1.00E+00
ENSBTAG00000006775	-1.86E-01	9.71E-01	-6.10E-01	8.43E-03	-9.12E-01	3.34E-05	-9.46E-01	2.07E-05	2.70E-01	7.78E-01
ENSBTAG00000034435	-2.80E-01	1.00E+00	-2.51E-01	7.78E-01	-9.25E-01	2.57E-02	-7.35E-01	1.00E-01	4.03E-01	8.00E-01
ENSBTAG00000046181	-2.63E-02	1.00E+00	8.79E-01	1.28E-02	-2.22E-02	1.00E+00	-1.29E+00	1.63E-04	4.36E-02	1.00E+00

ENSBTAG00000034431	0.00E+00	1.00E+00	0.00E+00	1.00E+00	1.78E+00	4.79E-01	0.00E+00	1.00E+00	0.00E+00	1.00E+00
ENSBTAG00000006972	-5.45E-01	6.04E-01	-1.62E-02	1.00E+00	9.82E-01	4.87E-03	4.51E-01	3.30E-01	4.73E-02	1.00E+00
ENSBTAG00000006971	8.91E-02	1.00E+00	4.44E-01	4.23E-02	4.67E-01	3.04E-02	2.14E-01	4.68E-01	1.61E-01	9.66E-01
ENSBTAG00000047116	-9.06E-01	1.74E-01	-2.49E+00	2.71E-08	-1.89E+00	6.87E-06	-5.50E-01	2.82E-01	-1.47E-01	1.00E+00
ENSBTAG00000011405	8.04E-02	1.00E+00	-2.26E+00	9.27E-07	-1.85E+00	3.68E-05	-1.43E+00	1.87E-03	-6.31E-02	1.00E+00
ENSBTAG00000039409	1.92E+00	8.23E-01	1.99E+00	4.43E-01	-5.13E-15	1.00E+00	0.00E+00	1.00E+00	0.00E+00	1.00E+00
ENSBTAG00000004629	-7.79E-01	9.46E-01	-4.58E-01	8.83E-01	1.09E-01	1.00E+00	3.30E-01	9.64E-01	-3.21E-01	1.00E+00
ENSBTAG00000008606	-3.49E-02	1.00E+00	7.03E-01	2.11E-04	7.50E-01	6.38E-05	1.38E-01	7.18E-01	1.38E-01	1.00E+00
ENSBTAG00000026527	1.60E-01	1.00E+00	2.29E-01	9.98E-01	-3.17E-01	8.69E-01	-5.68E-01	5.40E-01	-7.52E-02	1.00E+00
ENSBTAG00000045056	0.00E+00	1.00E+00	0.00E+00	1.00E+00	-5.13E-15	1.00E+00	0.00E+00	1.00E+00	0.00E+00	1.00E+00
ENSBTAG00000048135	1.09E-01	1.00E+00	-9.17E-02	1.00E+00	-5.76E-01	7.49E-01	-8.34E-01	5.15E-01	9.42E-01	8.00E-01
ENSBTAG00000046493	2.27E-01	7.69E-01	7.58E-01	2.75E-05	7.40E-01	4.12E-05	3.32E-01	1.23E-01	5.38E-02	1.00E+00
ENSBTAG00000046019	-1.66E-01	1.00E+00	-3.04E-01	2.74E-01	-8.22E-01	1.79E-04	-1.11E+00	3.37E-07	-2.80E-02	1.00E+00
ENSBTAG00000047780	1.58E-01	1.00E+00	5.35E-01	9.26E-03	7.80E-01	5.80E-05	4.31E-01	4.91E-02	3.58E-01	4.34E-01
ENSBTAG00000046206	0.00E+00	1.00E+00	0.00E+00	1.00E+00	-5.13E-15	1.00E+00	0.00E+00	1.00E+00	0.00E+00	1.00E+00
ENSBTAG00000045954	1.63E+00	2.07E-01	5.05E-01	8.52E-01	-1.07E+00	4.79E-01	-4.12E+00	1.29E-02	1.81E+00	1.74E-01
ENSBTAG00000034577	0.00E+00	1.00E+00	0.00E+00	1.00E+00	-5.13E-15	1.00E+00	0.00E+00	1.00E+00	0.00E+00	1.00E+00
ENSBTAG00000008251	4.83E-02	1.00E+00	-3.37E-01	1.65E-01	-3.84E-01	9.95E-02	-3.47E-01	1.57E-01	-2.73E-01	7.00E-01
ENSBTAG00000002186	-4.71E-01	6.63E-01	-1.39E+00	1.80E-04	-5.86E-01	1.42E-01	-5.25E-01	2.12E-01	-3.74E-01	8.00E-01
ENSBTAG00000045998	3.04E-01	1.00E+00	2.59E-01	1.00E+00	7.06E-01	5.19E-01	-3.78E-01	9.64E-01	8.00E-01	8.00E-01
ENSBTAG00000039698	0.00E+00	1.00E+00	0.00E+00	1.00E+00	-5.13E-15	1.00E+00	0.00E+00	1.00E+00	0.00E+00	1.00E+00
ENSBTAG00000046055	0.00E+00	1.00E+00	0.00E+00	1.00E+00	-5.13E-15	1.00E+00	0.00E+00	1.00E+00	0.00E+00	1.00E+00
ENSBTAG00000029086	0.00E+00	1.00E+00	0.00E+00	1.00E+00	-5.13E-15	1.00E+00	0.00E+00	1.00E+00	0.00E+00	1.00E+00
ENSBTAG00000047328	0.00E+00	1.00E+00	0.00E+00	1.00E+00	-5.13E-15	1.00E+00	0.00E+00	1.00E+00	0.00E+00	1.00E+00
ENSBTAG00000046780	0.00E+00	1.00E+00	0.00E+00	1.00E+00	-5.13E-15	1.00E+00	0.00E+00	1.00E+00	0.00E+00	1.00E+00
ENSBTAG00000042140	0.00E+00	1.00E+00	0.00E+00	1.00E+00	-5.13E-15	1.00E+00	0.00E+00	1.00E+00	0.00E+00	1.00E+00
ENSBTAG00000006621	1.44E+00	7.52E-01	8.35E-02	1.00E+00	1.02E+00	5.90E-01	1.80E+00	1.57E-01	1.43E+00	7.60E-01
ENSBTAG00000044247	0.00E+00	1.00E+00	0.00E+00	1.00E+00	-5.13E-15	1.00E+00	0.00E+00	1.00E+00	0.00E+00	1.00E+00
ENSBTAG00000042316	0.00E+00	1.00E+00	0.00E+00	1.00E+00	-5.13E-15	1.00E+00	0.00E+00	1.00E+00	0.00E+00	1.00E+00
ENSBTAG00000023079	-1.91E+00	8.23E-01	7.08E-02	1.00E+00	-1.91E+00	4.28E-01	2.02E+00	2.04E-01	-1.91E+00	8.00E-01
ENSBTAG00000035268	0.00E+00	1.00E+00	0.00E+00	1.00E+00	-5.13E-15	1.00E+00	0.00E+00	1.00E+00	0.00E+00	1.00E+00
ENSBTAG00000006648	-1.91E+00	8.23E-01	-1.91E+00	4.69E-01	-1.91E+00	4.28E-01	1.49E+00	4.57E-01	7.12E-03	1.00E+00
ENSBTAG00000013567	0.00E+00	1.00E+00	0.00E+00	1.00E+00	-5.13E-15	1.00E+00	1.77E+00	4.97E-01	0.00E+00	1.00E+00
ENSBTAG00000048284	0.00E+00	1.00E+00	0.00E+00	1.00E+00	-5.13E-15	1.00E+00	0.00E+00	1.00E+00	0.00E+00	1.00E+00
ENSBTAG00000035333	1.68E+00	7.74E-01	1.39E+00	5.20E-01	1.82E+00	2.80E-01	2.02E+00	2.05E-01	7.91E-01	1.00E+00

ENSBTAG00000046925	0.00E+00	1.00E+00	1.98E+00	4.43E-01	-5.13E-15	1.00E+00	0.00E+00	1.00E+00	0.00E+00	1.00E+00
ENSBTAG00000047044	3.23E+00	3.75E-01	0.00E+00	1.00E+00	-5.13E-15	1.00E+00	2.53E+00	2.71E-01	0.00E+00	1.00E+00
ENSBTAG00000047252	0.00E+00	1.00E+00	0.00E+00	1.00E+00	-5.13E-15	1.00E+00	0.00E+00	1.00E+00	1.91E+00	8.00E-01
ENSBTAG00000046568	0.00E+00	1.00E+00	0.00E+00	1.00E+00	-5.13E-15	1.00E+00	0.00E+00	1.00E+00	0.00E+00	1.00E+00
ENSBTAG00000046093	0.00E+00	1.00E+00	0.00E+00	1.00E+00	-5.13E-15	1.00E+00	0.00E+00	1.00E+00	0.00E+00	1.00E+00
ENSBTAG00000046213	-1.10E+00	8.23E-01	-2.35E+00	1.03E-01	-4.33E+00	6.04E-03	-4.33E+00	6.70E-03	-2.42E+00	3.84E-01
ENSBTAG00000048121	-3.88E+00	1.44E-01	-3.88E+00	3.63E-02	-2.10E+00	1.77E-01	-1.35E+00	4.16E-01	-3.88E+00	2.08E-01
ENSBTAG00000042812	1.62E+00	3.47E-01	9.77E-02	1.00E+00	-5.19E-01	9.48E-01	-1.82E+00	3.03E-01	5.68E-01	1.00E+00
ENSBTAG00000045376	-7.81E-01	1.00E+00	-2.70E+00	2.42E-01	-9.18E-01	8.09E-01	-2.70E+00	2.05E-01	-2.70E+00	6.01E-01
ENSBTAG00000042115	7.89E-03	1.00E+00	-1.35E+00	3.95E-01	-1.59E+00	2.62E-01	-7.41E-01	7.02E-01	-5.30E-01	1.00E+00
ENSBTAG00000045308	-6.73E-01	1.00E+00	-1.28E+00	2.97E-01	-1.05E+00	3.98E-01	-6.04E-01	7.37E-01	-4.35E-01	1.00E+00
ENSBTAG00000044769	0.00E+00	1.00E+00	0.00E+00	1.00E+00	-5.13E-15	1.00E+00	2.53E+00	2.71E-01	2.71E+00	6.01E-01
ENSBTAG00000045426	-3.90E-01	1.00E+00	-1.62E+00	4.01E-01	-3.61E+00	4.46E-02	-3.61E+00	4.81E-02	-3.61E+00	3.09E-01
ENSBTAG00000042125	0.00E+00	1.00E+00	0.00E+00	1.00E+00	-5.13E-15	1.00E+00	0.00E+00	1.00E+00	3.22E+00	4.34E-01
ENSBTAG00000042397	0.00E+00	1.00E+00	0.00E+00	1.00E+00	-5.13E-15	1.00E+00	0.00E+00	1.00E+00	0.00E+00	1.00E+00
ENSBTAG00000043599	0.00E+00	1.00E+00	0.00E+00	1.00E+00	-5.13E-15	1.00E+00	0.00E+00	1.00E+00	0.00E+00	1.00E+00
ENSBTAG00000042769	0.00E+00	1.00E+00	0.00E+00	1.00E+00	-5.13E-15	1.00E+00	0.00E+00	1.00E+00	0.00E+00	1.00E+00
ENSBTAG00000042719	1.92E+00	8.23E-01	0.00E+00	1.00E+00	1.78E+00	4.79E-01	0.00E+00	1.00E+00	0.00E+00	1.00E+00
ENSBTAG00000043080	1.92E+00	8.23E-01	1.99E+00	4.43E-01	1.78E+00	4.79E-01	0.00E+00	1.00E+00	1.93E+00	8.00E-01
ENSBTAG00000045459	0.00E+00	1.00E+00	1.98E+00	4.43E-01	-5.13E-15	1.00E+00	0.00E+00	1.00E+00	0.00E+00	1.00E+00
ENSBTAG00000002487	1.88E-01	8.82E-01	2.37E-01	3.62E-01	8.07E-01	2.22E-05	5.89E-01	3.45E-03	-2.23E-02	1.00E+00
ENSBTAG00000027081	5.22E-02	1.00E+00	1.40E-01	1.00E+00	-8.00E-02	1.00E+00	-6.46E-01	3.29E-01	-3.38E-01	1.00E+00
ENSBTAG00000043450	0.00E+00	1.00E+00	0.00E+00	1.00E+00	-5.13E-15	1.00E+00	0.00E+00	1.00E+00	0.00E+00	1.00E+00
ENSBTAG00000047865	7.52E-01	4.41E-01	1.23E+00	5.96E-03	1.31E+00	2.55E-03	2.04E+00	7.42E-07	4.64E-01	8.13E-01
ENSBTAG00000046543	-7.80E-01	1.00E+00	1.28E+00	4.44E-01	3.55E-01	1.00E+00	9.94E-01	6.19E-01	8.87E-01	1.00E+00
ENSBTAG00000045489	-1.91E+00	8.23E-01	-1.91E+00	4.69E-01	-1.91E+00	4.28E-01	-1.46E-01	1.00E+00	-1.91E+00	8.00E-01
ENSBTAG00000045975	0.00E+00	1.00E+00	0.00E+00	1.00E+00	-5.13E-15	1.00E+00	0.00E+00	1.00E+00	0.00E+00	1.00E+00
ENSBTAG00000047923	-2.00E+00	5.76E-01	-1.93E+00	2.49E-01	-3.91E+00	2.20E-02	-2.14E+00	1.75E-01	-2.00E+00	6.01E-01
ENSBTAG00000047192	-3.22E+00	3.75E-01	-3.22E+00	1.25E-01	-3.22E+00	9.39E-02	-3.22E+00	9.87E-02	-3.22E+00	4.34E-01
ENSBTAG00000045640	0.00E+00	1.00E+00	0.00E+00	1.00E+00	-5.13E-15	1.00E+00	0.00E+00	1.00E+00	0.00E+00	1.00E+00
ENSBTAG00000047732	0.00E+00	1.00E+00	0.00E+00	1.00E+00	-5.13E-15	1.00E+00	0.00E+00	1.00E+00	0.00E+00	1.00E+00
ENSBTAG00000046053	0.00E+00	1.00E+00	3.30E+00	1.02E-01	-5.13E-15	1.00E+00	0.00E+00	1.00E+00	0.00E+00	1.00E+00
ENSBTAG00000013422	-4.61E-01	1.00E+00	5.80E-01	7.22E-01	-1.80E+00	1.64E-01	-1.96E-01	1.00E+00	-7.62E-01	9.78E-01
ENSBTAG00000003392	1.22E+00	5.95E-01	9.14E-01	4.69E-01	-8.27E-01	6.74E-01	4.44E-01	9.25E-01	2.50E-01	1.00E+00
ENSBTAG00000045042	-2.70E+00	5.76E-01	-2.70E+00	2.42E-01	-2.70E+00	1.99E-01	-2.70E+00	2.05E-01	-2.70E+00	6.01E-01

ENSBTAG00000046858	0.00E+00	1.00E+00	0.00E+00	1.00E+00	-5.13E-15	1.00E+00	0.00E+00	1.00E+00	0.00E+00	1.00E+00
ENSBTAG00000035184	0.00E+00	1.00E+00	0.00E+00	1.00E+00	-5.13E-15	1.00E+00	0.00E+00	1.00E+00	0.00E+00	1.00E+00
ENSBTAG00000034398	0.00E+00	1.00E+00	0.00E+00	1.00E+00	-5.13E-15	1.00E+00	0.00E+00	1.00E+00	0.00E+00	1.00E+00
ENSBTAG00000000640	8.78E-01	9.96E-03	3.17E+00	2.20E-31	2.57E+00	5.02E-22	1.84E+00	4.47E-12	1.56E-03	1.00E+00
ENSBTAG00000007357	6.80E-02	1.00E+00	6.19E-01	1.03E-02	1.49E+00	1.19E-11	1.12E+00	6.83E-07	-2.87E-01	7.78E-01
ENSBTAG00000001567	-6.07E-02	1.00E+00	3.70E-01	2.52E-01	-2.88E-01	4.28E-01	-4.59E-01	1.43E-01	4.88E-02	1.00E+00
ENSBTAG00000044758	0.00E+00	1.00E+00	0.00E+00	1.00E+00	-5.13E-15	1.00E+00	0.00E+00	1.00E+00	0.00E+00	1.00E+00
ENSBTAG00000009125	6.48E-01	3.37E-01	1.21E-01	1.00E+00	3.94E-01	4.08E-01	9.47E-01	8.97E-03	1.03E-01	1.00E+00
ENSBTAG00000045087	0.00E+00	1.00E+00	0.00E+00	1.00E+00	-5.13E-15	1.00E+00	0.00E+00	1.00E+00	0.00E+00	1.00E+00
ENSBTAG00000048048	-1.64E-01	1.00E+00	-2.79E-01	1.00E+00	-8.28E-01	5.11E-01	-8.89E-01	4.92E-01	-1.21E-01	1.00E+00
ENSBTAG00000010514	8.99E-02	1.00E+00	1.63E-01	8.64E-01	2.41E-01	6.22E-01	5.56E-01	9.97E-02	1.20E-01	1.00E+00
ENSBTAG00000016495	8.48E-01	3.17E-03	1.63E+00	1.58E-12	1.09E+00	3.33E-06	4.35E-01	1.23E-01	-1.87E-01	9.88E-01
ENSBTAG00000006987	7.41E-01	8.65E-01	6.29E-01	6.25E-01	-1.61E+00	1.72E-01	6.19E-01	6.20E-01	-5.19E-01	1.00E+00
ENSBTAG00000019823	-5.14E-01	8.62E-01	-3.08E-01	8.20E-01	1.00E-01	1.00E+00	5.97E-01	3.67E-01	-1.84E-01	1.00E+00
ENSBTAG00000010192	-1.96E-01	1.00E+00	-2.61E+00	2.74E-05	-7.56E-02	1.00E+00	8.75E-01	4.85E-02	1.41E-02	1.00E+00
ENSBTAG00000010649	-1.61E-02	1.00E+00	4.78E-02	1.00E+00	-4.53E-02	1.00E+00	-1.48E-02	1.00E+00	-2.36E-01	8.59E-01
ENSBTAG00000048158	0.00E+00	1.00E+00	0.00E+00	1.00E+00	-5.13E-15	1.00E+00	0.00E+00	1.00E+00	0.00E+00	1.00E+00
ENSBTAG00000000539	-2.44E-02	1.00E+00	-6.63E-01	2.21E-03	-8.43E-01	5.89E-05	-8.57E-01	5.47E-05	-4.42E-02	1.00E+00
ENSBTAG00000011185	-2.67E-03	1.00E+00	-5.78E-01	1.48E-01	-1.13E+00	1.16E-03	-7.86E-01	3.67E-02	2.26E-01	1.00E+00
ENSBTAG00000011752	2.40E-01	9.69E-01	-3.14E-02	1.00E+00	-3.62E-01	3.39E-01	1.82E-01	7.90E-01	3.16E-01	8.00E-01
ENSBTAG00000021527	8.02E-01	1.29E-01	9.43E-01	7.13E-03	9.61E-01	5.46E-03	8.97E-01	1.20E-02	5.69E-01	5.11E-01
ENSBTAG00000021531	3.22E+00	3.75E-01	2.79E+00	2.10E-01	2.55E+00	2.55E-01	2.53E+00	2.71E-01	2.71E+00	6.01E-01
ENSBTAG00000020514	7.80E-02	1.00E+00	-1.61E+00	8.27E-03	-2.72E+00	8.70E-06	-1.49E+00	1.54E-02	-3.89E-01	1.00E+00
ENSBTAG00000043137	0.00E+00	1.00E+00	0.00E+00	1.00E+00	-5.13E-15	1.00E+00	0.00E+00	1.00E+00	0.00E+00	1.00E+00
ENSBTAG00000018252	-8.89E-01	1.59E-03	-5.21E-01	4.44E-02	-3.07E-01	3.13E-01	-1.80E-01	6.78E-01	-8.77E-01	6.15E-03
ENSBTAG00000046611	3.93E-01	1.00E+00	1.93E+00	2.68E-02	1.37E+00	1.64E-01	-2.13E-01	1.00E+00	1.02E+00	8.00E-01
ENSBTAG00000018007	-1.18E+00	1.58E-05	-1.02E+00	4.70E-05	-5.36E-01	5.04E-02	-4.53E-01	1.23E-01	-2.16E-01	9.20E-01
ENSBTAG00000043911	0.00E+00	1.00E+00	0.00E+00	1.00E+00	-5.13E-15	1.00E+00	0.00E+00	1.00E+00	0.00E+00	1.00E+00
ENSBTAG00000013689	9.87E-02	1.00E+00	-1.12E+00	3.62E-05	-1.43E+00	8.17E-08	-1.05E+00	1.11E-04	4.61E-01	4.54E-01
ENSBTAG00000043663	0.00E+00	1.00E+00	0.00E+00	1.00E+00	-5.13E-15	1.00E+00	0.00E+00	1.00E+00	0.00E+00	1.00E+00
ENSBTAG00000033335	4.23E-01	1.00E+00	-5.58E-01	6.49E-01	3.47E-01	8.37E-01	-1.97E-01	1.00E+00	-2.94E-01	1.00E+00
ENSBTAG00000027075	-6.87E-02	1.00E+00	-2.14E-02	1.00E+00	-2.18E-01	6.12E-01	-2.14E-01	6.37E-01	8.74E-02	1.00E+00
ENSBTAG00000018603	1.19E+00	3.86E-01	4.99E-01	7.58E-01	2.34E-01	1.00E+00	1.13E+00	1.52E-01	3.97E-01	1.00E+00
ENSBTAG00000017776	-8.13E-02	1.00E+00	5.10E-01	1.25E-02	-2.52E-01	3.20E-01	1.95E-01	4.97E-01	1.63E-01	9.41E-01
ENSBTAG00000048002	-2.06E-01	1.00E+00	-6.85E-01	5.43E-01	-1.89E+00	3.65E-02	-4.17E-01	8.12E-01	3.41E-01	1.00E+00

ENSBTAG00000022990	1.72E-02	1.00E+00	-2.70E+00	2.42E-01	-2.70E+00	1.99E-01	-9.33E-01	8.08E-01	7.76E-03	1.00E+00
ENSBTAG00000047409	1.92E+00	8.23E-01	0.00E+00	1.00E+00	1.78E+00	4.79E-01	0.00E+00	1.00E+00	2.71E+00	6.01E-01
ENSBTAG00000022989	1.63E+00	5.36E-03	2.97E+00	1.07E-10	3.13E+00	8.42E-12	2.21E+00	2.40E-06	7.75E-01	5.70E-01
ENSBTAG00000045559	0.00E+00	1.00E+00	0.00E+00	1.00E+00	1.78E+00	4.79E-01	1.77E+00	4.97E-01	0.00E+00	1.00E+00
ENSBTAG00000017896	-3.70E-01	1.00E+00	2.64E+00	7.15E-04	2.18E+00	8.48E-03	1.78E+00	5.66E-02	-8.68E-04	1.00E+00
ENSBTAG00000001652	-1.15E-01	1.00E+00	3.45E-02	1.00E+00	-5.00E-01	2.21E-01	-4.89E-02	1.00E+00	-5.33E-01	5.77E-01
ENSBTAG00000021064	4.24E-02	1.00E+00	1.90E+00	2.48E-08	1.39E+00	6.22E-05	1.49E-01	9.86E-01	3.00E-01	9.37E-01
ENSBTAG00000027074	-2.63E-01	1.00E+00	4.72E-01	6.09E-01	-1.59E-01	1.00E+00	1.01E+00	8.54E-02	-2.00E-01	1.00E+00
ENSBTAG00000047854	0.00E+00	1.00E+00	0.00E+00	1.00E+00	-5.13E-15	1.00E+00	0.00E+00	1.00E+00	0.00E+00	1.00E+00
ENSBTAG00000037383	2.46E-01	9.62E-01	1.81E+00	1.17E-10	1.46E+00	1.94E-07	4.09E-01	2.69E-01	4.10E-01	6.33E-01
ENSBTAG00000016091	5.90E-01	3.11E-01	5.09E-01	1.58E-01	1.43E-01	9.38E-01	6.53E-01	5.66E-02	4.36E-01	6.29E-01
ENSBTAG00000045960	0.00E+00	1.00E+00	0.00E+00	1.00E+00	-5.13E-15	1.00E+00	0.00E+00	1.00E+00	0.00E+00	1.00E+00
ENSBTAG00000011213	-3.93E-01	1.00E+00	-9.25E-02	1.00E+00	-5.64E-01	7.75E-01	-4.16E-02	1.00E+00	-6.37E-01	1.00E+00
ENSBTAG00000048156	6.06E-03	1.00E+00	7.58E-02	1.00E+00	2.05E+00	1.82E-01	-1.47E-01	1.00E+00	8.00E-01	1.00E+00
ENSBTAG00000047543	5.58E-01	1.00E+00	1.04E+00	4.41E-01	5.96E-01	8.06E-01	1.61E+00	9.71E-02	1.83E+00	2.66E-01
ENSBTAG00000043944	7.97E-01	1.00E+00	6.93E-02	1.00E+00	1.14E+00	6.49E-01	-1.91E+00	4.39E-01	7.93E-01	1.00E+00
ENSBTAG00000019830	-2.95E-01	8.23E-01	-2.73E-01	4.47E-01	1.28E-01	8.99E-01	-3.68E-02	1.00E+00	-5.50E-01	2.81E-01
ENSBTAG00000019831	-4.65E-02	1.00E+00	2.61E-01	4.38E-01	2.75E-01	3.93E-01	2.75E-01	4.07E-01	-3.58E-02	1.00E+00
ENSBTAG00000000967	-3.26E-01	5.76E-01	3.96E-01	1.06E-01	1.10E+00	6.05E-08	6.10E-01	6.15E-03	2.99E-02	1.00E+00
ENSBTAG00000040069	0.00E+00	1.00E+00	0.00E+00	1.00E+00	-5.13E-15	1.00E+00	0.00E+00	1.00E+00	0.00E+00	1.00E+00
ENSBTAG00000036425	0.00E+00	1.00E+00	0.00E+00	1.00E+00	-5.13E-15	1.00E+00	1.77E+00	4.97E-01	0.00E+00	1.00E+00
ENSBTAG00000017632	1.63E+00	2.02E-04	5.05E+00	1.46E-41	4.94E+00	3.00E-40	3.68E+00	5.28E-24	-3.12E-01	1.00E+00
ENSBTAG00000014762	1.34E+00	1.42E-01	3.48E+00	1.45E-09	3.32E+00	6.02E-09	2.95E+00	2.99E-07	9.88E-01	4.92E-01
ENSBTAG00000047729	-6.06E-01	1.00E+00	2.48E-01	1.00E+00	-2.24E-01	1.00E+00	-7.72E-01	7.29E-01	2.84E-01	1.00E+00
ENSBTAG00000001308	-1.17E+00	5.86E-02	-2.65E-01	7.64E-01	-1.86E-01	9.23E-01	-5.11E-01	3.33E-01	-3.22E-01	9.59E-01
ENSBTAG00000045929	0.00E+00	1.00E+00	0.00E+00	1.00E+00	-5.13E-15	1.00E+00	0.00E+00	1.00E+00	0.00E+00	1.00E+00
ENSBTAG00000046591	0.00E+00	1.00E+00	0.00E+00	1.00E+00	-5.13E-15	1.00E+00	0.00E+00	1.00E+00	0.00E+00	1.00E+00
ENSBTAG00000040580	0.00E+00	1.00E+00	0.00E+00	1.00E+00	-5.13E-15	1.00E+00	0.00E+00	1.00E+00	0.00E+00	1.00E+00
ENSBTAG00000002786	0.00E+00	1.00E+00	0.00E+00	1.00E+00	-5.13E-15	1.00E+00	0.00E+00	1.00E+00	0.00E+00	1.00E+00
ENSBTAG00000012326	0.00E+00	1.00E+00	2.79E+00	2.10E-01	1.78E+00	4.79E-01	0.00E+00	1.00E+00	0.00E+00	1.00E+00
ENSBTAG00000038494	-4.48E-01	8.23E-01	7.55E-01	5.88E-02	2.26E-01	8.06E-01	-3.61E-01	5.45E-01	-3.21E-01	9.55E-01
ENSBTAG00000004339	-7.73E-01	3.13E-02	-4.61E-01	1.35E-01	1.98E-01	6.69E-01	-2.86E-01	4.49E-01	-4.18E-01	5.70E-01
ENSBTAG00000001526	-1.91E+00	8.23E-01	7.57E-02	1.00E+00	-1.91E+00	4.28E-01	-1.91E+00	4.39E-01	-1.91E+00	8.00E-01
ENSBTAG00000046377	0.00E+00	1.00E+00	0.00E+00	1.00E+00	-5.13E-15	1.00E+00	1.76E+00	4.97E-01	0.00E+00	1.00E+00
ENSBTAG00000046412	-1.91E+00	8.23E-01	-1.91E+00	4.69E-01	-1.24E-01	1.00E+00	-1.91E+00	4.39E-01	-1.91E+00	8.00E-01

ENSBTAG00000036082	-7.87E-01	1.00E+00	-2.70E+00	2.42E-01	-1.53E-01	1.00E+00	-2.70E+00	2.05E-01	-7.75E-01	1.00E+00
ENSBTAG00000017054	1.67E+00	7.76E-01	-1.91E+00	4.69E-01	1.15E+00	6.44E-01	6.26E-01	9.94E-01	6.39E-03	1.00E+00
ENSBTAG00000048096	0.00E+00	1.00E+00	1.98E+00	4.43E-01	-5.13E-15	1.00E+00	1.76E+00	4.97E-01	0.00E+00	1.00E+00
ENSBTAG00000045202	0.00E+00	1.00E+00	0.00E+00	1.00E+00	-5.13E-15	1.00E+00	0.00E+00	1.00E+00	0.00E+00	1.00E+00
ENSBTAG00000016158	-3.00E-01	1.00E+00	-8.58E-01	5.20E-01	-3.23E-01	9.94E-01	6.69E-01	5.39E-01	-1.61E+00	5.31E-01
ENSBTAG00000001740	2.11E+00	3.84E-02	1.43E+00	8.19E-02	5.84E-01	6.35E-01	2.07E-01	1.00E+00	9.34E-01	7.36E-01
ENSBTAG00000003300	-2.87E-02	1.00E+00	-3.23E-01	3.93E-01	-3.06E-01	4.27E-01	-8.07E-02	1.00E+00	8.92E-02	1.00E+00
ENSBTAG00000019954	-3.33E-01	8.23E-01	-5.93E-01	7.69E-02	-6.84E-01	3.38E-02	6.08E-02	1.00E+00	4.66E-01	5.74E-01
ENSBTAG00000033721	-1.11E-01	1.00E+00	3.04E-01	1.00E+00	-5.20E-01	9.61E-01	-7.28E-02	1.00E+00	-7.48E-01	1.00E+00
ENSBTAG00000009097	0.00E+00	1.00E+00	0.00E+00	1.00E+00	-5.13E-15	1.00E+00	0.00E+00	1.00E+00	0.00E+00	1.00E+00
ENSBTAG00000044709	0.00E+00	1.00E+00	0.00E+00	1.00E+00	-5.13E-15	1.00E+00	0.00E+00	1.00E+00	0.00E+00	1.00E+00
ENSBTAG00000009098	0.00E+00	1.00E+00	0.00E+00	1.00E+00	-5.13E-15	1.00E+00	0.00E+00	1.00E+00	0.00E+00	1.00E+00
ENSBTAG00000029922	0.00E+00	1.00E+00	0.00E+00	1.00E+00	-5.13E-15	1.00E+00	0.00E+00	1.00E+00	0.00E+00	1.00E+00
ENSBTAG00000005471	-3.69E-01	1.00E+00	-2.86E-01	1.00E+00	-3.59E+00	4.53E-02	-1.05E+00	5.97E-01	-1.67E+00	7.79E-01
ENSBTAG00000002418	6.47E-01	7.26E-01	-9.86E-04	1.00E+00	-3.33E-02	1.00E+00	-2.56E-01	9.31E-01	4.91E-01	8.57E-01
ENSBTAG00000002440	-5.56E-02	1.00E+00	-1.58E+00	6.20E-06	-1.10E+00	1.48E-03	-9.94E-01	5.00E-03	1.48E-01	1.00E+00
ENSBTAG00000015350	-2.37E-01	1.00E+00	3.10E-01	4.65E-01	4.26E-01	2.39E-01	2.35E-01	6.55E-01	-3.65E-02	1.00E+00
ENSBTAG00000006140	3.09E-01	7.78E-01	-3.97E-01	2.19E-01	6.75E-01	9.38E-03	6.70E-01	1.16E-02	1.19E-01	1.00E+00
ENSBTAG00000006141	-6.46E-01	8.23E-01	3.75E-01	7.14E-01	7.64E-01	1.89E-01	7.18E-01	2.38E-01	-9.53E-01	5.82E-01
ENSBTAG00000027058	8.11E-01	1.00E+00	7.51E-02	1.00E+00	-1.31E-01	1.00E+00	-1.42E-01	1.00E+00	8.10E-01	1.00E+00
ENSBTAG00000016881	-2.85E-01	1.00E+00	-1.34E+00	1.89E-03	-1.42E+00	8.51E-04	-1.12E+00	1.18E-02	5.35E-02	1.00E+00
ENSBTAG00000001407	-1.13E-01	1.00E+00	7.93E-02	1.00E+00	5.01E-01	3.25E-02	6.91E-01	2.15E-03	-7.38E-02	1.00E+00
ENSBTAG00000027059	-6.43E-02	1.00E+00	-7.76E-01	8.95E-03	-6.37E-01	3.68E-02	-4.36E-01	2.09E-01	-7.83E-02	1.00E+00
ENSBTAG00000019174	1.27E-01	1.00E+00	-2.16E-01	6.67E-01	-1.15E-01	9.80E-01	4.75E-01	1.41E-01	3.26E-01	7.93E-01
ENSBTAG00000014093	3.93E-02	1.00E+00	-6.29E-01	8.79E-02	-1.30E+00	7.51E-05	-4.75E-01	2.51E-01	-2.30E-01	1.00E+00
ENSBTAG00000022947	1.70E+00	7.69E-01	7.57E-02	1.00E+00	-1.25E-01	1.00E+00	-1.42E-01	1.00E+00	-1.91E+00	8.00E-01
ENSBTAG00000021273	-6.58E-01	8.82E-03	-1.79E+00	9.91E-20	-2.36E+00	2.43E-32	-1.42E+00	4.52E-13	8.02E-01	1.44E-03
ENSBTAG00000021275	-3.63E-01	5.76E-01	-1.16E+00	7.27E-07	-1.52E+00	5.55E-11	-9.78E-01	4.18E-05	-7.56E-02	1.00E+00
ENSBTAG00000005174	-8.38E-01	1.09E-01	-1.22E+00	5.81E-04	-4.43E-01	2.98E-01	-9.51E-02	1.00E+00	6.15E-02	1.00E+00
ENSBTAG00000014505	-9.74E-02	1.00E+00	3.05E-01	2.59E-01	-3.30E-01	2.08E-01	-3.57E-01	1.73E-01	-3.04E-01	6.47E-01
ENSBTAG00000000026	-2.95E-02	1.00E+00	-8.75E-01	2.40E-05	-7.05E-01	7.41E-04	-7.48E-01	3.91E-04	4.01E-01	3.65E-01
ENSBTAG00000018643	4.32E-01	8.26E-01	9.24E-02	1.00E+00	2.19E-02	1.00E+00	5.42E-02	1.00E+00	-8.25E-02	1.00E+00
ENSBTAG00000043465	0.00E+00	1.00E+00	0.00E+00	1.00E+00	-5.13E-15	1.00E+00	0.00E+00	1.00E+00	0.00E+00	1.00E+00
ENSBTAG00000018642	-2.68E-01	1.00E+00	-2.59E-01	8.43E-01	6.51E-01	1.92E-01	9.84E-01	2.71E-02	2.42E-01	1.00E+00
ENSBTAG00000018639	-3.55E-01	5.45E-01	-1.32E+00	3.45E-09	-1.44E+00	7.05E-11	-8.62E-01	1.72E-04	7.77E-02	1.00E+00

ENSBTAG00000018637	-3.15E-02	1.00E+00	-1.26E+00	1.50E-06	-2.40E+00	1.31E-19	-2.02E+00	1.11E-14	-1.63E-01	1.00E+00
ENSBTAG00000018905	-1.86E-01	9.19E-01	-1.08E+00	7.34E-08	-6.46E-01	1.68E-03	7.01E-02	1.00E+00	3.54E-01	4.52E-01
ENSBTAG00000002941	-1.46E+00	3.72E-08	-2.12E+00	4.73E-17	-5.39E-01	4.61E-02	-7.78E-01	2.58E-03	-2.23E-01	8.99E-01
ENSBTAG00000002939	-7.36E-01	5.83E-02	-1.86E+00	4.62E-12	-2.62E+00	5.11E-22	-1.65E+00	1.12E-09	2.82E-01	8.11E-01
ENSBTAG00000020301	2.50E-01	1.00E+00	8.48E-01	8.59E-03	-1.97E-01	8.05E-01	-1.61E-01	9.10E-01	1.94E-03	1.00E+00
ENSBTAG00000010915	2.96E-01	8.67E-01	4.20E-01	2.58E-01	5.53E-01	9.79E-02	7.25E-01	2.30E-02	3.68E-01	7.71E-01
ENSBTAG00000013060	4.25E-01	4.62E-01	1.57E+00	1.99E-10	1.64E+00	2.26E-11	1.50E+00	1.44E-09	-1.61E-01	1.00E+00
ENSBTAG00000043693	0.00E+00	1.00E+00	0.00E+00	1.00E+00	-5.13E-15	1.00E+00	0.00E+00	1.00E+00	0.00E+00	1.00E+00
ENSBTAG00000047955	1.87E+00	5.53E-04	2.41E+00	1.16E-07	2.86E+00	7.57E-11	2.37E+00	1.80E-07	1.25E+00	1.39E-01
ENSBTAG00000001385	-1.16E+00	6.13E-03	-2.33E+00	4.34E-10	2.33E-02	1.00E+00	9.18E-02	1.00E+00	1.85E-01	1.00E+00
ENSBTAG00000001387	1.54E-01	1.00E+00	-1.93E-01	8.05E-01	5.68E-01	9.45E-02	1.01E+00	9.00E-04	8.46E-01	7.01E-02
ENSBTAG00000001388	-8.66E-02	1.00E+00	-3.34E-01	7.55E-01	-1.39E+00	1.51E-02	-1.41E+00	1.48E-02	-2.22E-01	1.00E+00
ENSBTAG00000011239	-1.58E-01	1.00E+00	-3.42E-01	1.35E-01	-2.86E-01	2.40E-01	-4.31E-01	4.93E-02	6.93E-02	1.00E+00
ENSBTAG00000001616	-1.19E-01	1.00E+00	-3.01E-01	2.64E-01	-6.92E-02	1.00E+00	-8.13E-02	1.00E+00	4.33E-01	3.29E-01
ENSBTAG00000044900	0.00E+00	1.00E+00	0.00E+00	1.00E+00	-5.13E-15	1.00E+00	0.00E+00	1.00E+00	0.00E+00	1.00E+00
ENSBTAG00000001618	3.43E-01	8.74E-01	7.61E-01	4.46E-02	6.27E-01	1.15E-01	-1.48E-01	9.83E-01	7.55E-01	2.67E-01
ENSBTAG00000020250	3.84E-01	1.00E+00	1.13E+00	9.95E-02	9.99E-02	1.00E+00	6.62E-01	4.60E-01	7.04E-01	8.00E-01
ENSBTAG00000015909	2.91E-02	1.00E+00	-5.81E-01	1.58E-02	2.52E-03	1.00E+00	1.61E-01	7.28E-01	1.92E-01	9.31E-01
ENSBTAG00000044587	0.00E+00	1.00E+00	0.00E+00	1.00E+00	-5.13E-15	1.00E+00	0.00E+00	1.00E+00	0.00E+00	1.00E+00
ENSBTAG00000042777	0.00E+00	1.00E+00	0.00E+00	1.00E+00	-5.13E-15	1.00E+00	0.00E+00	1.00E+00	0.00E+00	1.00E+00
ENSBTAG00000000622	4.53E-03	1.00E+00	8.96E-02	9.77E-01	-9.02E-02	9.75E-01	4.58E-02	1.00E+00	-5.77E-02	1.00E+00
ENSBTAG00000000623	-9.40E-01	7.86E-01	-3.95E+00	8.63E-05	-4.21E+00	2.16E-05	-1.26E+00	1.95E-01	4.93E-01	1.00E+00
ENSBTAG00000042673	0.00E+00	1.00E+00	0.00E+00	1.00E+00	-5.13E-15	1.00E+00	0.00E+00	1.00E+00	0.00E+00	1.00E+00
ENSBTAG00000008495	1.50E-02	1.00E+00	6.71E-01	6.64E-01	-2.34E+00	1.02E-01	-1.86E-01	1.00E+00	-9.08E-01	9.26E-01
ENSBTAG00000015018	1.05E+00	9.15E-02	2.89E-01	7.41E-01	-2.81E-01	7.51E-01	-1.12E+00	1.32E-02	3.68E-01	9.09E-01
ENSBTAG00000017711	1.13E+00	3.83E-03	1.19E+00	2.43E-04	6.16E-01	9.05E-02	-5.12E-02	1.00E+00	7.61E-02	1.00E+00
ENSBTAG00000017715	1.11E-01	1.00E+00	-1.18E-01	9.33E-01	2.29E-01	5.33E-01	-5.05E-02	1.00E+00	-1.16E-01	1.00E+00
ENSBTAG00000027069	7.28E-02	1.00E+00	1.12E+00	1.19E-03	1.39E+00	3.26E-05	2.82E-01	6.27E-01	-4.74E-01	6.70E-01
ENSBTAG00000016522	-2.93E-02	1.00E+00	-2.86E-01	6.25E-01	-1.31E-01	1.00E+00	-4.49E-01	3.30E-01	-5.19E-02	1.00E+00
ENSBTAG00000047366	1.27E-02	1.00E+00	2.88E+00	2.23E-02	3.01E+00	1.09E-02	6.22E-01	9.94E-01	1.79E-02	1.00E+00
ENSBTAG00000016630	-1.30E+00	3.39E-01	-8.14E-01	3.52E-01	-6.21E-01	4.79E-01	-1.05E-01	1.00E+00	5.60E-01	8.32E-01
ENSBTAG00000044418	0.00E+00	1.00E+00	0.00E+00	1.00E+00	-5.13E-15	1.00E+00	0.00E+00	1.00E+00	0.00E+00	1.00E+00
ENSBTAG00000003410	9.20E-02	1.00E+00	7.75E-01	7.20E-05	1.28E+00	7.92E-12	9.76E-01	3.77E-07	1.22E-01	1.00E+00
ENSBTAG00000003414	-5.91E-02	1.00E+00	7.05E-01	1.99E-01	3.01E-01	7.69E-01	2.84E-01	8.08E-01	2.41E-01	1.00E+00
ENSBTAG00000004645	2.75E-01	1.00E+00	2.73E-01	7.92E-01	-4.73E-01	4.45E-01	-7.70E-01	1.50E-01	3.37E-02	1.00E+00

ENSBTAG00000018004	2.65E-01	1.00E+00	-5.84E-01	8.85E-01	-2.11E+00	1.74E-01	-1.85E-01	1.00E+00	-6.75E-01	1.00E+00
ENSBTAG00000015462	-6.65E-01	1.00E+00	-1.90E+00	2.55E-01	-8.22E-01	6.77E-01	-1.35E+00	4.16E-01	-6.69E-01	1.00E+00
ENSBTAG00000018527	2.28E-01	1.00E+00	-3.80E-01	3.92E-01	-9.45E-01	5.05E-03	-1.03E+00	2.56E-03	-4.80E-01	6.01E-01
ENSBTAG00000012926	-1.13E-01	1.00E+00	-1.27E+00	2.63E-05	-2.20E+00	1.45E-13	-2.32E+00	1.04E-14	-7.33E-01	1.74E-01
ENSBTAG00000021341	1.79E-01	1.00E+00	3.35E-01	2.30E-01	1.62E-01	7.12E-01	1.42E-01	8.04E-01	1.77E-02	1.00E+00
ENSBTAG00000010988	3.06E-01	5.30E-01	8.38E-01	9.83E-06	6.13E-01	1.70E-03	4.00E-01	6.65E-02	-2.97E-01	6.01E-01
ENSBTAG00000010992	1.13E+00	7.56E-06	1.84E+00	2.79E-16	1.40E+00	4.87E-10	1.09E+00	2.33E-06	-2.51E-01	8.00E-01
ENSBTAG00000019940	6.32E-01	9.82E-01	6.36E-01	6.27E-01	5.46E-01	7.06E-01	5.32E-01	7.35E-01	-5.36E-01	1.00E+00
ENSBTAG00000045607	0.00E+00	1.00E+00	0.00E+00	1.00E+00	-5.13E-15	1.00E+00	0.00E+00	1.00E+00	0.00E+00	1.00E+00
ENSBTAG00000036380	0.00E+00	1.00E+00	0.00E+00	1.00E+00	-5.13E-15	1.00E+00	0.00E+00	1.00E+00	0.00E+00	1.00E+00
ENSBTAG00000020195	0.00E+00	1.00E+00	1.98E+00	4.43E-01	-5.13E-15	1.00E+00	0.00E+00	1.00E+00	0.00E+00	1.00E+00
ENSBTAG00000000807	2.66E-01	1.00E+00	-3.77E-02	1.00E+00	6.89E-01	5.70E-02	1.11E+00	9.35E-04	7.30E-01	2.50E-01
ENSBTAG00000044373	0.00E+00	1.00E+00	0.00E+00	1.00E+00	-5.13E-15	1.00E+00	0.00E+00	1.00E+00	0.00E+00	1.00E+00
ENSBTAG00000007094	1.92E+00	8.23E-01	0.00E+00	1.00E+00	1.78E+00	4.79E-01	2.53E+00	2.71E-01	2.71E+00	6.01E-01
ENSBTAG00000047935	-2.88E-01	1.00E+00	-1.90E+00	2.54E-01	-2.11E+00	1.72E-01	-8.47E-01	6.68E-01	1.22E+00	6.12E-01
ENSBTAG00000020023	2.14E-02	1.00E+00	-5.11E-01	1.21E-02	-1.10E+00	5.26E-09	-9.98E-01	1.66E-07	-1.19E-01	1.00E+00
ENSBTAG00000000735	2.31E+00	4.88E-10	1.71E+00	1.70E-06	3.43E-02	1.00E+00	-7.18E-02	1.00E+00	-9.05E-01	2.27E-01
ENSBTAG00000012321	2.35E-01	8.00E-01	8.58E-02	9.71E-01	-1.96E-01	4.94E-01	4.18E-02	1.00E+00	6.82E-01	1.14E-02
ENSBTAG00000048314	3.34E-01	5.76E-01	1.00E-01	9.48E-01	-3.91E-01	1.24E-01	-3.20E-01	2.51E-01	2.53E-03	1.00E+00
ENSBTAG00000012338	1.88E-02	1.00E+00	-9.10E-01	9.08E-06	-1.96E+00	2.14E-22	-2.05E+00	3.39E-24	1.41E-01	1.00E+00
ENSBTAG00000047587	-6.37E-01	9.21E-01	7.57E-02	1.00E+00	-1.07E-01	1.00E+00	2.45E-02	1.00E+00	-4.60E-01	1.00E+00
ENSBTAG00000012351	4.45E-01	8.98E-01	1.31E+00	3.93E-03	1.85E-01	1.00E+00	6.67E-01	2.43E-01	5.58E-01	7.87E-01
ENSBTAG00000032951	-1.65E-01	1.00E+00	-1.20E+00	3.01E-06	-1.36E+00	9.15E-08	-9.11E-01	5.96E-04	2.47E-01	8.62E-01
ENSBTAG00000042941	-1.91E+00	8.23E-01	7.58E-02	1.00E+00	-1.91E+00	4.28E-01	-1.41E-01	1.00E+00	-1.91E+00	8.00E-01
ENSBTAG00000007431	6.39E-01	8.23E-01	5.50E+00	3.20E-19	4.55E+00	1.73E-14	3.67E+00	4.31E-10	9.72E-01	5.47E-01
ENSBTAG00000001565	3.80E-01	4.19E-01	1.16E+00	4.47E-08	1.53E+00	3.02E-13	1.67E+00	2.80E-15	3.96E-01	4.34E-01
ENSBTAG00000008624	1.54E+00	1.62E-05	1.39E+00	1.93E-05	7.75E-01	2.81E-02	1.62E-01	9.25E-01	-7.01E-01	3.11E-01
ENSBTAG00000011861	1.72E+00	4.44E-02	3.07E+00	3.17E-09	2.10E+00	2.98E-04	7.67E-01	4.37E-01	-9.09E-03	1.00E+00
ENSBTAG00000010963	-3.17E-01	8.23E-01	-8.27E-01	4.73E-03	-7.55E-01	1.01E-02	-1.08E+00	1.64E-04	-4.08E-01	6.13E-01
ENSBTAG00000010964	-3.69E-01	6.03E-01	-1.87E+00	2.67E-13	-1.35E+00	9.42E-08	-5.62E-01	4.81E-02	-2.68E-02	1.00E+00
ENSBTAG00000012166	1.19E+00	8.63E-01	9.73E-01	6.59E-01	-1.48E-01	1.00E+00	1.77E+00	1.71E-01	-1.64E-03	1.00E+00
ENSBTAG00000035247	-1.12E-01	1.00E+00	-8.02E-01	3.36E-04	-1.32E+00	8.99E-10	-1.46E+00	2.05E-11	-2.66E-01	7.86E-01
ENSBTAG00000009854	2.20E-01	9.36E-01	1.75E+00	5.02E-14	1.73E+00	5.75E-14	9.69E-01	6.49E-05	-8.72E-02	1.00E+00
ENSBTAG00000009855	1.39E-01	1.00E+00	7.02E-01	7.62E-04	9.92E-01	7.63E-07	6.45E-01	2.59E-03	1.95E-01	8.74E-01
ENSBTAG00000014422	-8.12E-02	1.00E+00	-2.24E-01	5.42E-01	-2.74E-02	1.00E+00	2.68E-01	4.39E-01	2.81E-01	8.00E-01

ENSBTAG00000001639	-5.03E-01	9.97E-01	-3.88E-01	8.02E-01	-1.52E+00	3.77E-02	-9.43E-01	2.37E-01	3.73E-01	1.00E+00
ENSBTAG00000036407	0.00E+00	1.00E+00	0.00E+00	1.00E+00	-5.13E-15	1.00E+00	0.00E+00	1.00E+00	0.00E+00	1.00E+00
ENSBTAG00000020413	3.06E-01	1.00E+00	-6.27E-02	1.00E+00	-7.30E-01	5.97E-01	7.19E-02	1.00E+00	-1.46E+00	6.01E-01
ENSBTAG00000003435	9.36E-01	9.40E-01	7.72E-01	7.29E-01	2.20E-01	1.00E+00	9.45E-01	5.55E-01	3.90E-01	1.00E+00
ENSBTAG00000039634	1.19E-01	1.00E+00	6.95E-01	8.28E-03	5.75E-01	3.50E-02	3.75E-01	2.39E-01	-4.30E-03	1.00E+00
ENSBTAG00000042491	0.00E+00	1.00E+00	0.00E+00	1.00E+00	-5.13E-15	1.00E+00	0.00E+00	1.00E+00	0.00E+00	1.00E+00
ENSBTAG00000015398	-1.21E-01	1.00E+00	4.42E-01	1.24E-01	4.22E-01	1.48E-01	6.31E-01	1.85E-02	3.27E-01	7.14E-01
ENSBTAG00000042189	1.92E+00	8.23E-01	0.00E+00	1.00E+00	-5.13E-15	1.00E+00	0.00E+00	1.00E+00	0.00E+00	1.00E+00
ENSBTAG00000007651	-5.88E-01	7.80E-01	-1.47E+00	8.41E-03	-7.07E-01	2.36E-01	-9.33E-01	1.01E-01	-8.51E-01	4.91E-01
ENSBTAG00000007649	-2.66E-02	1.00E+00	2.90E-01	3.15E-01	3.45E-01	1.95E-01	3.65E-02	1.00E+00	6.66E-02	1.00E+00
ENSBTAG00000006675	-5.74E-03	1.00E+00	-6.15E-01	1.72E-03	-1.17E+00	3.00E-10	-1.36E+00	3.02E-13	3.70E-01	3.85E-01
ENSBTAG00000013593	-2.00E-01	1.00E+00	3.45E-01	3.96E-01	6.53E-01	4.00E-02	2.11E-01	7.24E-01	3.32E-03	1.00E+00
ENSBTAG00000003957	1.33E+00	7.36E-01	2.75E+00	1.45E-02	2.23E+00	5.28E-02	1.01E+00	5.13E-01	1.67E+00	5.52E-01
ENSBTAG00000047130	0.00E+00	1.00E+00	0.00E+00	1.00E+00	-5.13E-15	1.00E+00	0.00E+00	1.00E+00	0.00E+00	1.00E+00
ENSBTAG00000015775	-6.63E-01	8.23E-01	-4.12E+00	3.15E-06	-3.17E+00	2.90E-05	-1.46E+00	3.82E-02	-7.75E-02	1.00E+00
ENSBTAG00000042966	0.00E+00	1.00E+00	0.00E+00	1.00E+00	-5.13E-15	1.00E+00	0.00E+00	1.00E+00	0.00E+00	1.00E+00
ENSBTAG00000006868	5.73E-01	1.00E+00	2.98E-01	1.00E+00	2.05E-01	1.00E+00	8.49E-01	4.72E-01	5.57E-01	1.00E+00
ENSBTAG00000001396	1.74E-01	9.93E-01	9.00E-01	1.71E-05	6.01E-01	6.16E-03	-6.37E-02	1.00E+00	1.98E-02	1.00E+00
ENSBTAG00000012022	6.66E-01	8.57E-02	-5.75E-02	1.00E+00	-5.97E-01	4.31E-02	-5.39E-01	8.18E-02	1.05E-01	1.00E+00
ENSBTAG00000047166	-9.58E-01	8.03E-01	-1.12E+00	3.05E-01	-2.04E+00	3.99E-02	-1.70E+00	8.85E-02	-4.22E-01	1.00E+00
ENSBTAG00000010981	-1.92E-01	1.00E+00	-8.69E-01	4.26E-02	-1.14E+00	4.94E-03	-6.41E-01	1.51E-01	3.22E-01	8.97E-01
ENSBTAG00000006227	-1.84E-01	1.00E+00	-2.21E-01	7.85E-01	1.42E+00	2.56E-05	1.32E+00	1.29E-04	-3.84E-01	8.00E-01
ENSBTAG00000006231	-3.40E+00	1.10E-20	-5.51E+00	5.37E-35	-7.59E+00	3.20E-45	-4.85E+00	7.96E-34	2.01E-01	1.00E+00
ENSBTAG00000015942	1.82E+00	1.36E-01	4.77E+00	1.67E-08	2.50E+00	1.95E-03	2.29E+00	5.48E-03	1.09E-01	1.00E+00
ENSBTAG00000015944	1.22E-01	1.00E+00	6.79E-03	1.00E+00	-4.73E-02	1.00E+00	-1.22E-02	1.00E+00	-1.28E-01	1.00E+00
ENSBTAG00000045415	0.00E+00	1.00E+00	0.00E+00	1.00E+00	1.78E+00	4.79E-01	0.00E+00	1.00E+00	0.00E+00	1.00E+00
ENSBTAG00000012909	-1.65E+00	4.52E-01	-2.48E+00	3.68E-02	-8.89E-01	4.79E-01	3.67E-01	9.40E-01	-1.38E+00	6.11E-01
ENSBTAG00000002504	-8.41E-03	1.00E+00	-3.79E-01	2.68E-01	4.24E-01	1.93E-01	1.01E+00	2.37E-04	4.84E-01	4.44E-01
ENSBTAG00000001786	-4.61E-01	3.46E-01	-2.40E+00	4.60E-22	-3.90E+00	1.78E-48	-3.10E+00	3.32E-34	4.03E-01	5.25E-01
ENSBTAG00000014440	-4.29E-02	1.00E+00	6.04E-02	1.00E+00	4.75E-01	8.37E-02	5.50E-01	4.20E-02	-3.05E-01	7.66E-01
ENSBTAG00000013657	-4.34E+00	5.35E-02	-6.58E-01	7.63E-01	-1.56E-01	1.00E+00	-1.31E+00	3.39E-01	7.02E-03	1.00E+00
ENSBTAG00000003130	-4.96E-01	1.00E+00	-4.19E-01	1.00E+00	5.13E-01	9.37E-01	9.41E-01	5.60E-01	6.87E-01	1.00E+00
ENSBTAG00000003132	1.15E+00	8.23E-01	1.23E+00	3.96E-01	7.34E-01	7.42E-01	9.32E-01	5.80E-01	2.30E+00	1.32E-01
ENSBTAG00000004024	3.54E-01	5.08E-01	8.62E-01	7.34E-05	1.34E+00	1.87E-10	1.21E+00	1.40E-08	-1.22E-01	1.00E+00
ENSBTAG00000002833	-2.31E-01	7.53E-01	2.11E-01	4.07E-01	7.83E-01	9.18E-06	5.73E-01	2.14E-03	-9.41E-02	1.00E+00

ENSBTAG00000043187	-8.23E-01	7.03E-01	5.99E-01	4.18E-01	8.03E-01	1.81E-01	1.46E-01	1.00E+00	-4.84E-02	1.00E+00
ENSBTAG00000012489	-5.59E-01	7.16E-01	-2.11E+00	5.22E-06	-1.22E+00	7.12E-03	-1.21E+00	9.09E-03	-1.87E-01	1.00E+00
ENSBTAG00000012490	-4.85E-02	1.00E+00	-4.44E-01	2.21E-01	-7.83E-01	1.17E-02	-8.04E-01	1.08E-02	-2.32E-01	9.77E-01
ENSBTAG00000016651	2.57E-02	1.00E+00	1.58E+00	1.89E-01	-6.45E-01	9.07E-01	1.11E+00	4.54E-01	-4.99E-01	1.00E+00
ENSBTAG00000007382	2.91E-01	8.23E-01	-7.97E-01	4.08E-03	-7.88E-01	4.35E-03	-1.03E-01	1.00E+00	-6.69E-02	1.00E+00
ENSBTAG00000015780	4.78E-01	7.16E-01	2.63E+00	8.40E-13	2.30E+00	2.47E-10	2.04E+00	2.90E-08	8.88E-02	1.00E+00
ENSBTAG00000021144	2.82E-02	1.00E+00	-1.77E-01	7.43E-01	3.34E-01	2.98E-01	-9.22E-03	1.00E+00	-2.40E-01	8.77E-01
ENSBTAG00000002415	-1.39E-01	1.00E+00	-8.95E-01	2.43E-04	-5.82E-01	2.47E-02	-1.98E-01	6.37E-01	1.99E-01	9.49E-01
ENSBTAG00000005145	1.93E-01	1.00E+00	5.24E-02	1.00E+00	2.59E-01	5.73E-01	1.73E-01	8.34E-01	5.56E-01	4.01E-01
ENSBTAG00000020441	-1.52E-01	1.00E+00	-4.40E-02	1.00E+00	-7.72E-02	1.00E+00	3.62E-02	1.00E+00	-5.28E-02	1.00E+00
ENSBTAG00000023147	-4.03E-01	8.81E-01	-7.54E-01	1.22E-01	5.76E-01	2.44E-01	3.49E-01	5.96E-01	-1.03E-01	1.00E+00
ENSBTAG00000019506	-1.91E+00	8.23E-01	-1.91E+00	4.69E-01	-1.91E+00	4.28E-01	-1.91E+00	4.39E-01	-1.91E+00	8.00E-01
ENSBTAG00000012219	-4.53E-01	7.69E-01	6.09E-02	1.00E+00	-5.42E-01	2.29E-01	-1.38E+00	3.34E-04	-2.62E-01	1.00E+00
ENSBTAG00000012206	8.67E-01	9.15E-02	1.09E+00	2.03E-03	1.06E+00	2.84E-03	3.41E-01	5.05E-01	4.96E-01	6.24E-01
ENSBTAG00000012199	-2.52E-01	8.81E-01	3.05E-01	3.87E-01	1.32E-01	8.96E-01	-2.41E-01	5.45E-01	-3.37E-01	7.21E-01
ENSBTAG00000021293	9.70E-03	1.00E+00	-5.00E-01	3.49E-02	-6.43E-01	4.32E-03	-1.11E+00	2.97E-07	-1.35E-01	1.00E+00
ENSBTAG00000016984	5.08E-01	3.75E-01	1.84E+00	1.29E-11	4.49E-01	1.78E-01	3.70E-03	1.00E+00	1.43E-02	1.00E+00
ENSBTAG00000009985	4.72E-01	4.04E-01	-2.54E-01	5.35E-01	3.68E-01	2.76E-01	9.51E-01	5.00E-04	2.52E-01	8.77E-01
ENSBTAG00000007368	8.02E-02	1.00E+00	-4.05E-01	1.07E-01	-3.47E-01	1.88E-01	-5.51E-01	2.05E-02	5.57E-03	1.00E+00
ENSBTAG00000007365	-9.24E-01	4.44E-03	-2.34E+00	1.68E-17	-2.31E+00	1.17E-17	-1.67E+00	3.24E-10	-3.57E-01	6.84E-01
ENSBTAG00000030052	0.00E+00	1.00E+00	0.00E+00	1.00E+00	-5.13E-15	1.00E+00	0.00E+00	1.00E+00	0.00E+00	1.00E+00
ENSBTAG00000007346	-4.25E-02	1.00E+00	-5.76E-01	8.05E-03	-1.21E+00	2.24E-09	-1.40E+00	7.38E-12	-2.11E-01	8.24E-01
ENSBTAG00000032631	1.43E+00	7.63E-01	4.13E+00	3.08E-07	2.87E+00	2.80E-03	2.18E+00	5.73E-02	-7.85E-01	1.00E+00
ENSBTAG00000021021	-1.25E+00	1.24E-05	-2.68E-01	4.69E-01	3.51E-02	1.00E+00	3.48E-01	3.04E-01	3.54E-01	6.74E-01
ENSBTAG00000014089	6.60E-02	1.00E+00	-1.16E+00	5.28E-07	-2.17E+00	1.03E-20	-2.01E+00	5.84E-18	6.03E-02	1.00E+00
ENSBTAG00000021318	-7.05E-01	5.54E-01	-2.45E+00	3.44E-07	-6.32E-01	2.64E-01	-5.42E-01	3.88E-01	-3.16E-01	1.00E+00
ENSBTAG00000021693	-4.82E+00	1.08E-02	-1.51E+00	1.98E-01	-2.61E-02	1.00E+00	1.83E-01	1.00E+00	-9.14E-01	8.04E-01
ENSBTAG00000017267	-5.35E-02	1.00E+00	5.43E-02	1.00E+00	2.27E-01	6.09E-01	4.29E-01	1.96E-01	-4.87E-01	4.36E-01
ENSBTAG00000005846	-3.93E-01	5.76E-01	-1.03E+00	1.21E-04	-4.30E-01	1.68E-01	2.11E-01	6.43E-01	-1.41E-01	1.00E+00
ENSBTAG00000005845	-8.62E-02	1.00E+00	-8.63E-01	2.62E-03	-6.05E-01	4.25E-02	-4.82E-01	1.35E-01	1.67E-01	1.00E+00
ENSBTAG00000005844	-2.78E-02	1.00E+00	2.11E-01	4.08E-01	1.96E-01	4.48E-01	2.37E-01	3.31E-01	8.73E-02	1.00E+00
ENSBTAG00000005838	-2.51E-02	1.00E+00	-3.46E-01	2.95E-01	-4.03E-01	1.94E-01	-5.45E-01	6.00E-02	3.70E-01	6.20E-01
ENSBTAG00000021426	-5.03E-01	1.00E+00	-1.23E+00	6.09E-01	-3.22E+00	9.40E-02	-1.46E+00	4.92E-01	-3.22E+00	4.34E-01
ENSBTAG00000021425	5.11E-01	1.00E+00	6.07E-01	9.45E-01	1.48E+00	2.97E-01	-1.64E-01	1.00E+00	1.97E-02	1.00E+00
ENSBTAG00000021424	-1.66E-01	1.00E+00	-3.57E-01	2.49E-01	2.06E-01	6.18E-01	4.43E-02	1.00E+00	-2.18E-01	9.14E-01

ENSBTAG00000000085	-4.39E-01	3.56E-01	-1.05E+00	6.63E-06	-2.49E+00	7.92E-27	-2.87E+00	3.32E-34	-9.12E-02	1.00E+00
ENSBTAG00000001021	-7.52E-01	1.61E-01	-2.61E+00	3.26E-15	-6.03E+00	8.10E-57	-2.60E+00	4.08E-15	7.27E-02	1.00E+00
ENSBTAG00000007308	1.77E-01	1.00E+00	5.36E-01	4.73E-02	1.02E+00	1.65E-05	1.07E+00	8.18E-06	3.76E-01	6.01E-01
ENSBTAG00000045383	0.00E+00	1.00E+00	0.00E+00	1.00E+00	-5.13E-15	1.00E+00	0.00E+00	1.00E+00	0.00E+00	1.00E+00
ENSBTAG00000007304	-1.05E-01	1.00E+00	1.70E-01	6.67E-01	1.79E-02	1.00E+00	-2.86E-01	3.28E-01	-5.28E-02	1.00E+00
ENSBTAG00000011828	2.87E-01	8.39E-01	7.64E-01	7.02E-03	6.30E-01	3.09E-02	6.40E-01	3.16E-02	4.50E-03	1.00E+00
ENSBTAG00000000010	2.84E-01	8.23E-01	5.32E-01	5.37E-02	4.47E-01	1.22E-01	5.71E-01	3.92E-02	5.80E-02	1.00E+00
ENSBTAG00000011446	8.23E-01	9.69E-02	7.14E-01	4.92E-02	1.07E+00	1.33E-03	2.07E+00	9.28E-11	3.61E-01	8.00E-01
ENSBTAG00000006934	6.51E-01	9.96E-01	-1.31E-02	1.00E+00	-4.57E-01	8.42E-01	3.51E-01	9.69E-01	1.38E+00	4.94E-01
ENSBTAG00000006912	-4.91E-01	9.95E-01	-4.99E-01	6.33E-01	-3.54E-01	8.08E-01	4.81E-01	5.66E-01	-6.02E-01	8.42E-01
ENSBTAG00000007348	1.83E+00	4.41E-01	6.03E-01	9.45E-01	-1.52E-01	1.00E+00	9.93E-01	6.17E-01	1.18E+00	8.31E-01
ENSBTAG00000006024	-1.03E+00	5.93E-01	-1.11E+00	2.09E-01	-3.18E+00	5.89E-04	-2.04E+00	1.44E-02	-2.00E-01	1.00E+00
ENSBTAG00000002661	0.00E+00	1.00E+00	0.00E+00	1.00E+00	-5.13E-15	1.00E+00	3.03E+00	1.50E-01	0.00E+00	1.00E+00
ENSBTAG00000015779	2.28E-01	9.52E-01	1.13E+00	1.20E-05	6.02E-01	3.28E-02	2.65E-02	1.00E+00	-2.24E-02	1.00E+00
ENSBTAG00000015772	4.73E-02	1.00E+00	-3.32E-01	4.10E-01	2.87E-01	4.79E-01	5.41E-01	9.92E-02	1.34E-01	1.00E+00
ENSBTAG00000009086	-2.89E-01	9.84E-01	-2.14E+00	1.97E-09	-1.49E+00	2.41E-05	-5.17E-01	2.45E-01	-1.31E-01	1.00E+00
ENSBTAG00000027033	1.91E+00	8.23E-01	0.00E+00	1.00E+00	1.78E+00	4.79E-01	0.00E+00	1.00E+00	1.91E+00	8.00E-01
ENSBTAG00000037578	-1.31E+00	9.32E-01	-4.31E-01	1.00E+00	-1.44E+00	4.79E-01	-1.46E+00	4.92E-01	1.47E+00	6.01E-01
ENSBTAG00000039813	-4.32E-01	1.00E+00	-1.29E+00	2.43E-01	-4.40E-02	1.00E+00	-2.10E-01	1.00E+00	1.29E-01	1.00E+00
ENSBTAG00000038159	-7.67E-01	9.60E-01	-4.52E-01	8.92E-01	-9.58E-01	4.43E-01	-1.65E+00	1.57E-01	-3.33E-01	1.00E+00
ENSBTAG00000013234	1.91E+00	8.23E-01	1.98E+00	4.43E-01	1.78E+00	4.79E-01	0.00E+00	1.00E+00	2.71E+00	6.01E-01
ENSBTAG00000040134	0.00E+00	1.00E+00	0.00E+00	1.00E+00	1.78E+00	4.79E-01	0.00E+00	1.00E+00	1.91E+00	8.00E-01
ENSBTAG00000010828	4.08E-01	1.00E+00	-1.89E-02	1.00E+00	-3.06E-01	8.75E-01	-5.09E-01	5.89E-01	-5.96E-01	8.34E-01
ENSBTAG00000013055	-2.44E+00	7.10E-03	-2.93E+00	2.05E-04	-1.54E+00	2.44E-02	-1.14E+00	1.11E-01	-1.25E+00	3.69E-01
ENSBTAG00000038080	-8.67E-01	1.00E+00	-1.32E+00	4.63E-01	2.54E-01	1.00E+00	-6.36E-01	8.80E-01	-9.44E-01	9.90E-01
ENSBTAG00000010057	-7.79E-01	9.72E-01	-1.16E+00	3.84E-01	-1.76E-01	1.00E+00	-1.74E+00	1.46E-01	-9.46E-01	8.34E-01
ENSBTAG00000021291	-3.23E-01	9.87E-01	-2.31E+00	1.20E-08	-3.98E+00	5.67E-20	-3.85E+00	4.99E-19	-1.20E-01	1.00E+00
ENSBTAG00000048113	0.00E+00	1.00E+00	0.00E+00	1.00E+00	-5.13E-15	1.00E+00	0.00E+00	1.00E+00	0.00E+00	1.00E+00
ENSBTAG00000002170	3.68E-01	9.57E-01	2.46E-01	8.52E-01	-1.01E-01	1.00E+00	-7.31E-02	1.00E+00	-3.57E-01	9.62E-01
ENSBTAG00000045428	0.00E+00	1.00E+00	0.00E+00	1.00E+00	-5.13E-15	1.00E+00	0.00E+00	1.00E+00	0.00E+00	1.00E+00
ENSBTAG00000031558	-4.92E-01	1.00E+00	-5.12E-01	6.98E-01	-5.36E-01	6.41E-01	-9.24E-01	3.17E-01	-2.75E-01	1.00E+00
ENSBTAG00000011113	-2.71E+00	5.76E-01	5.96E-01	9.45E-01	1.00E+00	6.00E-01	1.62E+00	2.35E-01	2.32E-02	1.00E+00
ENSBTAG00000012789	1.02E-01	1.00E+00	7.48E-01	1.39E-02	4.57E-01	1.89E-01	2.62E-01	5.72E-01	4.71E-02	1.00E+00
ENSBTAG00000045322	0.00E+00	1.00E+00	0.00E+00	1.00E+00	-5.13E-15	1.00E+00	0.00E+00	1.00E+00	0.00E+00	1.00E+00
ENSBTAG00000027863	-2.70E+00	5.76E-01	-2.70E+00	2.42E-01	-2.70E+00	1.99E-01	3.39E-01	1.00E+00	1.88E-02	1.00E+00

ENSBTAG00000001617	-2.67E-02	1.00E+00	-2.67E-01	6.39E-01	-1.21E-01	1.00E+00	-2.26E-01	7.49E-01	-2.84E-01	9.20E-01
ENSBTAG00000017565	7.66E-02	1.00E+00	5.20E-01	1.06E-02	4.26E-01	4.42E-02	2.67E-01	2.90E-01	-2.42E-01	7.71E-01
ENSBTAG00000021844	1.62E+00	5.08E-01	6.23E+00	5.21E-19	3.26E+00	9.69E-05	1.69E+00	1.50E-01	3.93E-01	1.00E+00
ENSBTAG00000021845	9.35E-01	4.44E-05	2.21E+00	7.05E-28	1.40E+00	2.58E-12	5.65E-01	1.11E-02	-2.77E-01	7.14E-01
ENSBTAG00000017201	1.79E+00	6.06E-14	2.94E+00	4.67E-37	2.01E+00	1.18E-18	1.19E+00	4.85E-07	-5.11E-02	1.00E+00
ENSBTAG00000032477	-1.47E-03	1.00E+00	-4.15E-01	9.76E-02	-3.88E-01	1.28E-01	-1.58E-01	7.24E-01	4.19E-01	3.96E-01
ENSBTAG00000022847	-1.24E-01	1.00E+00	-1.44E+00	3.83E-09	-8.63E-01	5.18E-04	-3.44E-03	1.00E+00	2.48E-01	8.24E-01
ENSBTAG00000042753	0.00E+00	1.00E+00	0.00E+00	1.00E+00	-5.13E-15	1.00E+00	0.00E+00	1.00E+00	0.00E+00	1.00E+00
ENSBTAG00000038020	-3.07E-01	9.75E-01	-1.00E+00	1.44E-02	-8.11E-02	1.00E+00	5.22E-01	2.31E-01	2.89E-01	9.55E-01
ENSBTAG00000010536	-7.79E-01	1.00E+00	-2.70E+00	2.42E-01	-9.22E-01	8.08E-01	-2.70E+00	2.05E-01	-2.70E+00	6.01E-01
ENSBTAG00000044121	7.26E-02	1.00E+00	-8.27E-01	3.17E-03	-1.01E+00	1.98E-04	-1.19E+00	1.34E-05	-9.74E-02	1.00E+00
ENSBTAG00000028324	0.00E+00	1.00E+00	0.00E+00	1.00E+00	-5.13E-15	1.00E+00	0.00E+00	1.00E+00	0.00E+00	1.00E+00
ENSBTAG00000027024	-3.15E-01	8.23E-01	-2.34E-01	6.24E-01	6.35E-01	3.68E-02	1.03E+00	3.20E-04	5.74E-02	1.00E+00
ENSBTAG00000043440	-1.40E-01	1.00E+00	2.72E-01	1.00E+00	-7.03E-01	6.23E-01	-5.45E-01	7.88E-01	-1.69E-01	1.00E+00
ENSBTAG00000042097	6.62E-03	1.00E+00	-1.91E+00	4.69E-01	-1.91E+00	4.28E-01	-1.91E+00	4.39E-01	-1.91E+00	8.00E-01
ENSBTAG00000044894	0.00E+00	1.00E+00	0.00E+00	1.00E+00	1.78E+00	4.79E-01	0.00E+00	1.00E+00	1.91E+00	8.00E-01
ENSBTAG00000017719	1.65E-01	1.00E+00	1.46E-01	1.00E+00	-6.30E-01	5.52E-01	1.04E+00	1.85E-01	-4.23E-01	1.00E+00
ENSBTAG00000042823	0.00E+00	1.00E+00	0.00E+00	1.00E+00	-5.13E-15	1.00E+00	0.00E+00	1.00E+00	0.00E+00	1.00E+00
ENSBTAG00000037302	0.00E+00	1.00E+00	0.00E+00	1.00E+00	-5.13E-15	1.00E+00	0.00E+00	1.00E+00	0.00E+00	1.00E+00
ENSBTAG00000004462	8.10E-01	1.00E+00	2.32E+00	1.16E-01	1.80E+00	2.88E-01	2.58E+00	5.64E-02	2.00E+00	6.01E-01
ENSBTAG00000008172	-4.70E-02	1.00E+00	-4.75E-01	1.89E-01	-7.56E-01	1.76E-02	-4.87E-01	1.80E-01	-2.75E-01	8.91E-01
ENSBTAG00000047905	8.14E-01	5.52E-01	8.94E-01	1.42E-01	8.82E-02	1.00E+00	-3.26E-01	8.36E-01	-2.26E-01	1.00E+00
ENSBTAG00000013981	0.00E+00	1.00E+00	0.00E+00	1.00E+00	-5.13E-15	1.00E+00	0.00E+00	1.00E+00	0.00E+00	1.00E+00
ENSBTAG00000035392	0.00E+00	1.00E+00	0.00E+00	1.00E+00	-5.13E-15	1.00E+00	0.00E+00	1.00E+00	0.00E+00	1.00E+00
ENSBTAG00000003419	4.26E-01	3.51E-01	1.11E+00	5.71E-07	7.19E-01	1.88E-03	5.38E-01	3.10E-02	1.05E-01	1.00E+00
ENSBTAG00000046113	-2.78E+00	1.40E-01	-1.02E+00	4.43E-01	-4.45E-02	1.00E+00	-2.18E+00	6.58E-02	-5.44E-01	1.00E+00
ENSBTAG00000037013	1.18E+00	8.62E-01	6.05E-01	9.45E-01	1.02E+00	5.93E-01	1.00E+00	6.15E-01	1.43E+00	7.64E-01
ENSBTAG00000028419	2.72E+00	5.76E-01	0.00E+00	1.00E+00	2.55E+00	2.54E-01	0.00E+00	1.00E+00	1.91E+00	8.00E-01
ENSBTAG00000047504	-7.89E-01	9.43E-01	-1.91E+00	1.22E-01	-2.15E+00	6.50E-02	2.05E-01	1.00E+00	-1.99E+00	4.16E-01
ENSBTAG00000045508	0.00E+00	1.00E+00	0.00E+00	1.00E+00	-5.13E-15	1.00E+00	0.00E+00	1.00E+00	0.00E+00	1.00E+00
ENSBTAG00000012873	-4.46E-03	1.00E+00	-2.55E-01	4.99E-01	-8.55E-02	1.00E+00	1.13E-01	9.67E-01	-2.99E-01	8.00E-01
ENSBTAG00000037179	0.00E+00	1.00E+00	0.00E+00	1.00E+00	-5.13E-15	1.00E+00	0.00E+00	1.00E+00	0.00E+00	1.00E+00
ENSBTAG00000015143	2.70E+00	5.76E-01	1.98E+00	4.43E-01	1.78E+00	4.79E-01	1.76E+00	4.97E-01	0.00E+00	1.00E+00
ENSBTAG00000015053	1.77E-01	1.00E+00	2.88E-01	4.31E-01	6.29E-01	2.10E-02	1.28E+00	3.21E-07	-5.71E-02	1.00E+00
ENSBTAG00000020164	1.30E+00	9.64E-06	2.43E+00	4.70E-20	2.07E+00	3.16E-15	1.68E+00	2.55E-10	-6.96E-01	1.44E-01

ENSBTAG00000037938	1.36E-02	1.00E+00	7.59E-01	2.30E-03	6.55E-01	9.81E-03	4.02E-02	1.00E+00	-2.50E-02	1.00E+00
ENSBTAG00000019085	4.51E-01	2.17E-01	1.97E+00	3.96E-22	1.97E+00	3.40E-22	1.75E+00	9.53E-18	1.44E-01	1.00E+00
ENSBTAG00000025764	-7.27E-01	7.47E-01	1.54E+00	1.42E-03	2.43E-01	9.33E-01	-9.04E-01	1.95E-01	-1.28E+00	3.07E-01
ENSBTAG00000009680	4.09E-01	4.77E-01	7.15E-02	1.00E+00	1.73E-01	7.27E-01	1.48E-01	8.27E-01	-4.42E-01	4.62E-01
ENSBTAG00000009681	7.45E-01	2.75E-02	1.34E+00	5.78E-08	1.00E+00	6.88E-05	8.50E-01	1.10E-03	-6.78E-01	1.15E-01
ENSBTAG00000009682	6.27E-01	5.81E-02	1.79E+00	4.95E-16	1.75E+00	1.62E-15	1.20E+00	1.23E-07	1.35E-01	1.00E+00
ENSBTAG00000043216	0.00E+00	1.00E+00	0.00E+00	1.00E+00	-5.13E-15	1.00E+00	0.00E+00	1.00E+00	0.00E+00	1.00E+00
ENSBTAG00000009683	1.25E-01	1.00E+00	7.26E-01	1.89E-03	6.72E-01	4.40E-03	5.10E-01	4.55E-02	-4.40E-01	3.89E-01
ENSBTAG00000016683	3.62E+00	5.33E-19	2.87E+00	1.13E-13	1.22E+00	1.86E-03	8.15E-01	5.83E-02	-1.83E-01	1.00E+00
ENSBTAG00000042175	0.00E+00	1.00E+00	0.00E+00	1.00E+00	-5.13E-15	1.00E+00	0.00E+00	1.00E+00	0.00E+00	1.00E+00
ENSBTAG00000027013	6.10E-03	1.00E+00	5.97E-01	9.45E-01	3.44E-01	1.00E+00	-2.71E+00	2.05E-01	1.24E-03	1.00E+00
ENSBTAG00000001282	2.32E-01	8.75E-01	-5.87E-01	2.11E-02	-3.00E-01	3.36E-01	1.23E-02	1.00E+00	-3.97E-01	5.10E-01
ENSBTAG00000042200	0.00E+00	1.00E+00	0.00E+00	1.00E+00	-5.13E-15	1.00E+00	0.00E+00	1.00E+00	0.00E+00	1.00E+00
ENSBTAG00000045730	-7.87E-01	1.00E+00	-7.16E-01	9.45E-01	-9.22E-01	8.08E-01	7.11E-01	8.46E-01	1.19E+00	8.29E-01
ENSBTAG00000018400	-3.99E-01	7.41E-01	-1.27E+00	9.16E-05	3.07E-02	1.00E+00	9.08E-02	1.00E+00	-7.43E-01	2.00E-01
ENSBTAG00000009941	-6.00E-01	1.21E-01	-9.26E-01	2.33E-04	-5.56E-01	4.02E-02	-3.68E-01	2.42E-01	-3.52E-01	6.44E-01
ENSBTAG00000000541	-1.91E+00	8.23E-01	8.82E-01	8.52E-01	6.45E-01	9.78E-01	1.12E+00	6.71E-01	-1.91E+00	8.00E-01
ENSBTAG00000013352	6.61E-03	1.00E+00	7.11E-02	1.00E+00	-1.91E+00	4.28E-01	2.22E+00	1.32E-01	7.93E-01	1.00E+00
ENSBTAG00000009186	-1.65E-01	1.00E+00	-1.02E+00	1.11E-01	-2.00E+00	1.14E-03	-2.57E+00	9.59E-05	2.36E-02	1.00E+00
ENSBTAG00000019350	4.05E-02	1.00E+00	-1.17E+00	3.83E-05	-1.29E+00	3.32E-06	-1.54E+00	3.96E-08	8.09E-02	1.00E+00
ENSBTAG00000000655	2.07E-01	1.00E+00	-1.42E+00	2.56E-06	-1.32E+00	1.01E-05	-9.35E-01	2.66E-03	-4.04E-01	6.82E-01
ENSBTAG00000013888	-1.14E+00	1.49E-02	-2.72E+00	3.34E-13	-1.50E+00	3.32E-05	3.83E-01	4.58E-01	5.44E-02	1.00E+00
ENSBTAG00000015095	-2.85E-01	1.00E+00	1.68E-01	1.00E+00	-1.22E+00	9.63E-02	-2.75E-01	9.29E-01	3.60E-01	1.00E+00
ENSBTAG00000006582	7.00E-01	1.00E+00	9.42E-02	1.00E+00	-1.59E-01	1.00E+00	-6.66E-01	8.97E-01	6.72E-01	1.00E+00
ENSBTAG00000006130	-1.87E-02	1.00E+00	-7.04E-01	4.83E-02	-6.65E-02	1.00E+00	-4.19E-02	1.00E+00	-2.72E-01	9.34E-01
ENSBTAG00000044793	0.00E+00	1.00E+00	0.00E+00	1.00E+00	-5.13E-15	1.00E+00	0.00E+00	1.00E+00	0.00E+00	1.00E+00
ENSBTAG00000003708	8.81E-02	1.00E+00	-1.02E+00	8.06E-06	-8.10E-01	4.71E-04	4.23E-01	1.14E-01	2.77E-01	7.83E-01
ENSBTAG00000048042	0.00E+00	1.00E+00	0.00E+00	1.00E+00	-5.13E-15	1.00E+00	0.00E+00	1.00E+00	0.00E+00	1.00E+00
ENSBTAG00000020930	-2.27E-01	1.00E+00	-4.73E-01	1.89E-01	1.39E-01	9.34E-01	6.43E-01	4.77E-02	-4.52E-01	6.01E-01
ENSBTAG00000015023	7.47E-02	1.00E+00	1.59E-01	6.53E-01	4.48E-02	1.00E+00	-5.56E-01	9.63E-03	-3.43E-01	5.00E-01
ENSBTAG00000026995	-3.61E-02	1.00E+00	3.73E-01	1.43E-01	1.80E-01	6.22E-01	-4.24E-01	9.24E-02	8.39E-02	1.00E+00
ENSBTAG00000045986	1.84E-01	1.00E+00	-5.70E-01	3.12E-01	-2.13E+00	1.28E-06	-1.40E+00	2.25E-03	8.64E-01	3.49E-01
ENSBTAG00000006819	1.38E-01	1.00E+00	-4.54E-02	1.00E+00	-4.95E-01	2.34E-02	-5.45E-01	1.26E-02	2.16E-01	8.00E-01
ENSBTAG00000006836	-1.19E-02	1.00E+00	1.33E-01	8.79E-01	1.09E-01	9.65E-01	9.35E-02	1.00E+00	5.13E-01	3.09E-01
ENSBTAG00000017924	1.00E+00	3.15E-01	1.36E+00	1.08E-02	2.65E+00	1.03E-08	2.88E+00	4.66E-10	-3.85E-01	1.00E+00

ENSBTAG00000009980	-7.92E-01	1.00E+00	-2.71E+00	2.42E-01	1.26E+00	4.28E-01	-9.40E-01	8.06E-01	1.19E+00	8.29E-01
ENSBTAG00000003684	-1.85E-01	1.00E+00	-1.18E-01	1.00E+00	-7.66E-01	6.14E-01	1.35E+00	1.81E-01	-4.67E-01	1.00E+00
ENSBTAG00000043018	0.00E+00	1.00E+00	0.00E+00	1.00E+00	-5.13E-15	1.00E+00	0.00E+00	1.00E+00	0.00E+00	1.00E+00
ENSBTAG00000047864	0.00E+00	1.00E+00	0.00E+00	1.00E+00	-5.13E-15	1.00E+00	0.00E+00	1.00E+00	0.00E+00	1.00E+00
ENSBTAG00000006363	5.97E-01	7.61E-01	-1.32E-01	1.00E+00	-8.31E-01	1.41E-01	-6.14E-01	3.42E-01	1.26E+00	1.18E-01
ENSBTAG00000047710	1.99E+00	5.76E-01	2.88E+00	2.27E-02	2.06E+00	1.80E-01	2.43E+00	8.18E-02	1.69E+00	7.74E-01
ENSBTAG00000047240	1.58E-01	1.00E+00	1.13E+00	1.40E-03	9.16E-01	1.18E-02	7.84E-01	4.17E-02	8.40E-04	1.00E+00
ENSBTAG00000031934	0.00E+00	1.00E+00	0.00E+00	1.00E+00	1.78E+00	4.79E-01	0.00E+00	1.00E+00	0.00E+00	1.00E+00
ENSBTAG00000016062	-1.04E+00	1.57E-04	-1.51E+00	1.01E-09	-8.93E-01	2.25E-04	-1.02E+00	3.01E-05	-2.21E-01	8.85E-01
ENSBTAG00000020042	-5.04E-01	6.63E-01	-2.98E-01	6.38E-01	6.52E-02	1.00E+00	3.34E-01	5.53E-01	-3.52E-01	8.60E-01
ENSBTAG00000007351	0.00E+00	1.00E+00	0.00E+00	1.00E+00	-5.13E-15	1.00E+00	0.00E+00	1.00E+00	0.00E+00	1.00E+00
ENSBTAG00000039117	-4.11E-02	1.00E+00	-3.61E-01	2.63E-01	1.42E-01	8.49E-01	4.93E-01	8.68E-02	-1.97E-01	9.87E-01
ENSBTAG00000002603	-3.91E-02	1.00E+00	-3.46E-01	1.59E-01	-2.68E-02	1.00E+00	-3.17E-01	2.20E-01	-3.58E-01	4.81E-01
ENSBTAG00000045184	0.00E+00	1.00E+00	0.00E+00	1.00E+00	-5.13E-15	1.00E+00	0.00E+00	1.00E+00	0.00E+00	1.00E+00
ENSBTAG00000002610	3.76E-02	1.00E+00	-2.20E-01	5.22E-01	-9.84E-02	9.76E-01	2.92E-01	3.43E-01	-1.97E-01	9.27E-01
ENSBTAG00000047002	7.58E-02	1.00E+00	-1.20E+00	1.65E-03	-4.71E-01	2.68E-01	5.71E-01	1.21E-01	3.78E-01	8.00E-01
ENSBTAG00000002612	2.68E-01	8.82E-01	-1.78E+00	1.98E-09	-5.30E-01	9.72E-02	-1.52E-01	8.71E-01	-1.47E-01	1.00E+00
ENSBTAG00000002613	2.64E-02	1.00E+00	-9.93E-01	5.62E-02	-2.98E-01	7.65E-01	-4.89E-01	4.62E-01	2.66E-01	1.00E+00
ENSBTAG00000002616	-1.20E-01	1.00E+00	-1.42E-01	8.72E-01	3.21E-01	3.53E-01	5.20E-01	7.89E-02	-1.26E-01	1.00E+00
ENSBTAG00000032059	2.07E-01	1.00E+00	3.70E-01	8.48E-01	-2.55E-01	9.99E-01	-9.86E-01	2.24E-01	-9.76E-01	6.01E-01
ENSBTAG00000015412	6.95E-01	9.50E-01	1.10E-01	1.00E+00	-5.23E-02	1.00E+00	-5.48E-01	8.02E-01	-2.97E-01	1.00E+00
ENSBTAG00000014472	-1.06E-01	1.00E+00	3.24E-01	4.43E-01	1.67E+00	1.18E-09	1.07E+00	2.36E-04	2.82E-01	8.62E-01
ENSBTAG00000000425	-5.00E-02	1.00E+00	2.14E-01	6.87E-01	1.97E-01	7.34E-01	2.16E-01	6.88E-01	-3.22E-01	8.00E-01
ENSBTAG00000014476	-4.41E-02	1.00E+00	-5.08E-01	4.00E-02	-6.12E-01	9.49E-03	-8.26E-01	3.60E-04	2.02E-01	8.98E-01
ENSBTAG00000014478	1.12E+00	3.21E-04	1.62E+00	1.53E-09	1.40E+00	1.94E-07	5.46E-01	8.42E-02	2.54E-01	8.96E-01
ENSBTAG00000014479	-7.61E-02	1.00E+00	-5.34E-01	1.42E-02	1.79E-01	5.75E-01	1.31E-01	7.89E-01	-1.30E-02	1.00E+00
ENSBTAG00000021304	-5.29E-02	1.00E+00	-1.32E+00	4.73E-10	-6.15E-01	5.90E-03	-6.31E-02	1.00E+00	2.06E-01	8.60E-01
ENSBTAG00000048076	0.00E+00	1.00E+00	0.00E+00	1.00E+00	-5.13E-15	1.00E+00	0.00E+00	1.00E+00	0.00E+00	1.00E+00
ENSBTAG00000028355	-2.70E+00	5.76E-01	-2.70E+00	2.42E-01	-2.70E+00	1.99E-01	-2.70E+00	2.05E-01	-2.70E+00	6.01E-01
ENSBTAG00000043150	0.00E+00	1.00E+00	0.00E+00	1.00E+00	-5.13E-15	1.00E+00	1.76E+00	4.97E-01	0.00E+00	1.00E+00
ENSBTAG00000014001	-7.02E-03	1.00E+00	-1.45E-01	1.00E+00	3.37E-02	1.00E+00	4.03E-01	5.46E-01	4.12E-01	8.74E-01
ENSBTAG00000045096	0.00E+00	1.00E+00	0.00E+00	1.00E+00	-5.13E-15	1.00E+00	0.00E+00	1.00E+00	0.00E+00	1.00E+00
ENSBTAG00000014005	1.58E-01	1.00E+00	3.26E-01	3.50E-01	3.65E-01	2.64E-01	6.54E-01	2.01E-02	-1.20E-01	1.00E+00
ENSBTAG00000007502	-1.64E+00	3.33E-01	-1.92E+00	4.75E-02	-2.01E+00	3.20E-02	-2.09E+00	2.82E-02	-1.46E+00	4.79E-01
ENSBTAG00000007503	-1.98E-01	1.00E+00	-3.60E-01	1.00E+00	-1.28E+00	3.37E-01	-6.44E-01	7.41E-01	-4.33E-01	1.00E+00

ENSBTAG00000043787	0.00E+00	1.00E+00	0.00E+00	1.00E+00	-5.13E-15	1.00E+00	0.00E+00	1.00E+00	0.00E+00	1.00E+00
ENSBTAG00000032130	2.78E-01	1.00E+00	-2.64E-01	6.99E-01	-1.60E+00	1.07E-05	-1.39E+00	1.77E-04	2.70E-01	9.91E-01
ENSBTAG00000017196	7.13E-01	3.03E-02	1.77E+00	1.40E-13	1.83E+00	1.70E-14	1.66E+00	4.84E-12	8.28E-01	1.58E-02
ENSBTAG00000043223	0.00E+00	1.00E+00	0.00E+00	1.00E+00	-5.13E-15	1.00E+00	0.00E+00	1.00E+00	0.00E+00	1.00E+00
ENSBTAG00000032132	1.08E+00	6.70E-02	2.37E+00	3.33E-10	1.89E+00	8.18E-07	1.98E+00	2.57E-07	3.89E-01	9.01E-01
ENSBTAG00000047694	8.91E-02	1.00E+00	1.17E-01	1.00E+00	-4.59E-01	2.17E-01	-5.60E-01	1.15E-01	4.51E-01	6.01E-01
ENSBTAG00000037953	0.00E+00	1.00E+00	0.00E+00	1.00E+00	-5.13E-15	1.00E+00	0.00E+00	1.00E+00	0.00E+00	1.00E+00
ENSBTAG00000017211	3.53E-01	6.27E-01	8.45E-01	8.43E-04	-2.27E-01	5.59E-01	-6.79E-01	1.15E-02	-3.32E-01	7.12E-01
ENSBTAG00000017212	4.08E-01	3.91E-01	1.63E+00	1.49E-13	1.32E+00	2.56E-09	6.79E-01	4.55E-03	-3.33E-01	6.22E-01
ENSBTAG00000017213	2.21E-01	9.01E-01	1.11E+00	1.05E-06	9.32E-01	4.93E-05	8.59E-01	2.60E-04	1.20E-01	1.00E+00
ENSBTAG00000017215	-1.48E-01	1.00E+00	-1.34E+00	6.14E-03	-1.40E+00	3.61E-03	-9.77E-01	6.08E-02	-2.40E-01	1.00E+00
ENSBTAG00000017216	7.23E-02	1.00E+00	3.06E-01	8.80E-01	-6.90E-01	4.24E-01	-1.76E+00	1.92E-02	-4.20E-01	1.00E+00
ENSBTAG00000042421	0.00E+00	1.00E+00	0.00E+00	1.00E+00	-5.13E-15	1.00E+00	0.00E+00	1.00E+00	0.00E+00	1.00E+00
ENSBTAG00000044101	1.45E-01	1.00E+00	-9.05E-01	1.88E-01	-8.64E-01	1.94E-01	-1.12E+00	8.26E-02	1.88E-01	1.00E+00
ENSBTAG00000006738	-5.08E-01	1.00E+00	4.62E-01	9.98E-01	-6.62E-01	8.92E-01	-6.91E-01	8.80E-01	1.62E+00	4.96E-01
ENSBTAG00000045399	0.00E+00	1.00E+00	0.00E+00	1.00E+00	-5.13E-15	1.00E+00	0.00E+00	1.00E+00	0.00E+00	1.00E+00
ENSBTAG00000000838	1.81E-01	9.80E-01	-2.37E-01	4.44E-01	-4.92E-01	3.90E-02	7.44E-03	1.00E+00	-9.28E-04	1.00E+00
ENSBTAG00000005040	-1.51E-01	9.61E-01	4.98E-01	6.33E-03	5.22E-01	3.76E-03	2.33E-01	3.15E-01	-1.50E-01	9.32E-01
ENSBTAG00000018708	-5.31E-03	1.00E+00	8.57E-01	4.15E-02	5.80E-01	2.18E-01	1.90E-01	9.22E-01	-3.29E-01	9.34E-01
ENSBTAG00000018709	3.33E-01	9.35E-01	8.58E-02	1.00E+00	-7.61E-01	7.89E-02	-1.21E+00	3.54E-03	1.74E-01	1.00E+00
ENSBTAG00000003375	-1.84E-01	1.00E+00	3.04E-01	7.79E-01	-2.48E-01	8.90E-01	5.62E-01	3.57E-01	4.48E-02	1.00E+00
ENSBTAG00000018123	-2.25E-01	1.00E+00	-1.33E+00	3.58E-06	-2.15E+00	1.01E-13	-9.21E-01	1.77E-03	-3.28E-01	8.00E-01
ENSBTAG00000026953	-1.97E-01	1.00E+00	9.90E-01	5.26E-03	1.46E+00	1.40E-05	1.56E+00	4.54E-06	-1.78E-01	1.00E+00
ENSBTAG00000046316	-3.70E-01	8.53E-01	7.92E-01	2.94E-02	1.61E+00	2.95E-07	9.87E-01	4.39E-03	-7.08E-01	4.14E-01
ENSBTAG00000005786	-3.42E-01	8.23E-01	-1.38E+00	5.51E-05	-4.34E-01	2.77E-01	2.48E-01	6.44E-01	1.41E-01	1.00E+00
ENSBTAG00000015156	5.75E-02	1.00E+00	6.68E-01	1.41E-02	6.26E-01	2.26E-02	4.08E-01	1.99E-01	-8.21E-02	1.00E+00
ENSBTAG00000010227	-7.35E-02	1.00E+00	3.70E-01	8.13E-02	6.19E-01	1.11E-03	5.57E-01	4.62E-03	1.11E-01	1.00E+00
ENSBTAG00000006620	-2.72E-01	1.00E+00	2.27E-01	1.00E+00	-1.71E-01	1.00E+00	8.98E-01	2.65E-01	-8.16E-01	8.33E-01
ENSBTAG00000044369	0.00E+00	1.00E+00	0.00E+00	1.00E+00	-5.13E-15	1.00E+00	0.00E+00	1.00E+00	0.00E+00	1.00E+00
ENSBTAG00000045963	0.00E+00	1.00E+00	1.99E+00	4.43E-01	-5.13E-15	1.00E+00	0.00E+00	1.00E+00	0.00E+00	1.00E+00
ENSBTAG00000010416	6.48E-02	1.00E+00	-1.33E-01	8.85E-01	-2.34E-01	5.32E-01	4.32E-02	1.00E+00	1.46E-01	1.00E+00
ENSBTAG00000046979	-4.38E-01	1.44E-01	-8.13E-01	1.33E-05	-1.19E+00	5.68E-11	-1.36E+00	9.73E-14	2.99E-03	1.00E+00
ENSBTAG00000004598	4.23E-01	3.67E-01	1.68E+00	2.97E-14	1.72E+00	5.00E-15	1.33E+00	2.46E-09	2.61E-01	8.00E-01
ENSBTAG00000009836	2.49E+00	8.57E-02	9.81E-01	6.49E-01	1.96E+00	9.52E-02	1.24E+00	4.52E-01	5.25E-01	1.00E+00
ENSBTAG00000009845	2.54E-03	1.00E+00	-8.87E-01	1.39E-03	-9.71E-01	3.60E-04	-6.37E-01	3.14E-02	-1.59E-01	1.00E+00

ENSBTAG00000042548	0.00E+00	1.00E+00	0.00E+00	1.00E+00	-5.13E-15	1.00E+00	0.00E+00	1.00E+00	0.00E+00	1.00E+00
ENSBTAG00000031885	-2.42E-02	1.00E+00	4.14E-01	2.22E-01	1.00E+00	2.33E-04	9.39E-01	7.97E-04	-1.88E-01	1.00E+00
ENSBTAG00000046990	1.78E-02	1.00E+00	6.58E-01	4.65E-03	1.04E+00	1.57E-06	5.98E-01	1.24E-02	-4.12E-01	4.59E-01
ENSBTAG00000004310	2.40E-02	1.00E+00	-2.61E-01	3.43E-01	3.30E-01	1.74E-01	3.12E-01	2.17E-01	-4.60E-02	1.00E+00
ENSBTAG00000046185	2.57E-02	1.00E+00	4.82E-01	1.80E-01	9.14E-01	2.36E-03	1.09E+00	2.55E-04	5.22E-01	4.71E-01
ENSBTAG00000008017	-3.43E-01	1.00E+00	-1.06E+00	1.89E-01	-7.45E-01	3.94E-01	4.27E-02	1.00E+00	1.31E+00	2.15E-01
ENSBTAG00000015880	7.32E-01	8.35E-01	2.50E-01	1.00E+00	1.08E+00	1.69E-01	1.50E+00	2.90E-02	3.04E-01	1.00E+00
ENSBTAG00000046590	0.00E+00	1.00E+00	0.00E+00	1.00E+00	1.78E+00	4.79E-01	0.00E+00	1.00E+00	0.00E+00	1.00E+00
ENSBTAG00000007109	-6.89E-01	1.70E-01	-1.36E+00	1.54E-05	-5.77E-01	9.37E-02	-9.82E-01	2.11E-03	-2.09E-01	1.00E+00
ENSBTAG00000016112	5.21E-03	1.00E+00	1.39E+00	5.19E-01	-1.91E+00	4.28E-01	1.11E+00	6.72E-01	4.19E-04	1.00E+00
ENSBTAG00000014981	-2.95E-01	9.64E-01	-1.38E+00	2.64E-04	-5.61E-01	1.77E-01	1.75E-01	8.94E-01	-3.18E-01	8.96E-01
ENSBTAG00000003423	-1.37E-01	1.00E+00	1.17E+00	1.53E-07	1.26E+00	1.17E-08	7.10E-01	2.79E-03	-2.47E-01	8.00E-01
ENSBTAG00000031834	-2.81E-01	8.23E-01	-1.79E-01	7.45E-01	-5.90E-01	3.65E-02	-7.97E-01	3.52E-03	-2.03E-01	9.80E-01
ENSBTAG00000004272	-1.59E+00	1.00E+00	-2.22E+00	4.71E-01	-2.76E+00	3.44E-01	-2.19E+00	4.97E-01	-3.86E+00	5.15E-01
ENSBTAG00000003152	-4.96E-01	1.00E+00	-1.87E-01	1.00E+00	-8.87E-01	8.40E-01	-1.25E+00	6.50E-01	-1.99E+00	7.63E-01
ENSBTAG00000003155	-1.85E-01	1.00E+00	-7.83E-01	4.74E-02	-4.93E-01	2.52E-01	-2.86E-02	1.00E+00	-3.24E-02	1.00E+00
ENSBTAG00000009891	5.66E-01	1.37E-01	1.33E+00	1.37E-08	-1.78E-01	6.94E-01	-9.60E-01	9.41E-05	-6.36E-02	1.00E+00
ENSBTAG00000009894	1.26E+00	2.65E-05	2.08E+00	1.02E-14	1.73E+00	9.18E-11	1.42E+00	1.59E-07	8.87E-01	2.30E-02
ENSBTAG00000039808	2.70E-01	8.18E-01	-1.45E-01	8.05E-01	-1.29E+00	1.30E-08	-2.04E+00	3.95E-19	6.29E-01	9.36E-02
ENSBTAG00000018843	9.14E-02	1.00E+00	-8.45E-02	9.90E-01	-5.85E-01	5.76E-03	-8.14E-01	7.80E-05	-1.94E-01	8.70E-01
ENSBTAG00000038361	-1.10E-02	1.00E+00	-2.46E-01	4.09E-01	-6.36E-01	3.83E-03	-7.21E-01	1.05E-03	2.33E-01	8.00E-01
ENSBTAG00000007392	1.06E+00	8.50E-01	2.53E+00	1.71E-02	4.95E+00	6.95E-07	5.65E+00	3.07E-08	3.73E+00	4.68E-03
ENSBTAG00000006191	-3.87E+00	1.47E-01	-5.71E-01	8.99E-01	4.95E-01	8.74E-01	-8.29E-01	6.86E-01	-3.87E+00	2.11E-01
ENSBTAG00000039864	-6.87E-01	9.90E-01	-2.03E+00	8.31E-02	-1.40E+00	1.99E-01	-1.79E+00	1.04E-01	1.68E-01	1.00E+00
ENSBTAG00000004063	-4.52E-01	2.36E-01	-1.19E+00	2.08E-08	-1.97E+00	1.19E-20	-1.99E+00	5.76E-21	-3.26E-01	6.01E-01
ENSBTAG00000046540	1.23E+00	6.35E-05	1.43E+00	1.76E-07	1.37E+00	5.26E-07	1.47E+00	1.06E-07	2.10E+00	6.56E-13
ENSBTAG00000017096	2.35E-01	9.36E-01	-9.76E-03	1.00E+00	1.59E-01	8.07E-01	-5.99E-02	1.00E+00	-3.46E-01	7.36E-01
ENSBTAG00000045894	0.00E+00	1.00E+00	0.00E+00	1.00E+00	-5.13E-15	1.00E+00	0.00E+00	1.00E+00	0.00E+00	1.00E+00
ENSBTAG00000045844	0.00E+00	1.00E+00	0.00E+00	1.00E+00	-5.13E-15	1.00E+00	0.00E+00	1.00E+00	0.00E+00	1.00E+00
ENSBTAG00000024311	-2.34E-01	9.32E-01	-3.83E-01	2.38E-01	7.09E-01	6.03E-03	8.36E-01	1.06E-03	-4.63E-01	4.55E-01
ENSBTAG00000047122	-2.34E+00	1.11E-01	-4.13E-01	9.46E-01	-3.27E+00	1.32E-03	-3.47E+00	8.85E-04	-4.61E+00	9.52E-04
ENSBTAG00000047040	2.83E+00	3.51E-15	2.91E+00	5.48E-17	2.84E+00	1.89E-16	2.80E+00	6.79E-16	3.27E+00	4.61E-19
ENSBTAG00000007043	3.19E+00	1.42E-12	3.80E+00	6.02E-18	3.82E+00	3.24E-18	4.22E+00	4.32E-21	3.73E+00	8.22E-16
ENSBTAG00000026917	0.00E+00	1.00E+00	0.00E+00	1.00E+00	-5.13E-15	1.00E+00	0.00E+00	1.00E+00	0.00E+00	1.00E+00
ENSBTAG00000007041	2.01E+00	2.24E-15	2.21E+00	2.41E-19	2.31E+00	4.29E-21	2.35E+00	1.59E-21	2.61E+00	1.74E-24

ENSBTAG00000003492	4.15E+00	1.46E-17	4.86E+00	1.26E-23	5.44E+00	4.64E-28	6.19E+00	1.42E-33	3.60E+00	3.24E-13
ENSBTAG00000010407	1.92E+00	8.23E-01	0.00E+00	1.00E+00	-5.13E-15	1.00E+00	3.04E+00	1.48E-01	1.93E+00	8.00E-01
ENSBTAG00000042640	0.00E+00	1.00E+00	0.00E+00	1.00E+00	-5.13E-15	1.00E+00	0.00E+00	1.00E+00	0.00E+00	1.00E+00
ENSBTAG00000044551	0.00E+00	1.00E+00	0.00E+00	1.00E+00	-5.13E-15	1.00E+00	0.00E+00	1.00E+00	0.00E+00	1.00E+00
ENSBTAG00000012852	2.08E-01	1.00E+00	8.16E-02	1.00E+00	7.62E-01	5.51E-03	1.04E+00	1.07E-04	3.74E-01	6.48E-01
ENSBTAG00000019916	3.72E-02	1.00E+00	-2.10E-01	5.16E-01	-2.82E-01	3.16E-01	1.75E-01	6.44E-01	6.36E-01	5.22E-02
ENSBTAG00000048094	2.43E+00	1.73E-01	2.40E+00	4.25E-02	2.35E+00	4.69E-02	2.30E+00	5.88E-02	2.92E+00	1.10E-01
ENSBTAG00000003526	-2.52E-01	1.00E+00	-1.64E+00	1.79E-05	-2.29E+00	1.63E-09	-1.15E+00	3.61E-03	-2.06E-01	1.00E+00
ENSBTAG00000044659	-7.22E-01	3.27E-01	2.64E-01	6.87E-01	3.01E-01	5.81E-01	-9.18E-01	2.50E-02	-1.09E-01	1.00E+00
ENSBTAG00000013423	-3.12E-01	8.23E-01	-1.28E+00	1.15E-05	-1.99E+00	4.86E-12	-1.57E+00	7.07E-08	-4.86E-01	5.15E-01
ENSBTAG00000009653	1.92E+00	8.23E-01	0.00E+00	1.00E+00	-5.13E-15	1.00E+00	0.00E+00	1.00E+00	0.00E+00	1.00E+00
ENSBTAG00000019580	0.00E+00	1.00E+00	1.98E+00	4.43E-01	1.78E+00	4.79E-01	0.00E+00	1.00E+00	1.91E+00	8.00E-01
ENSBTAG00000021717	3.97E-01	1.00E+00	6.58E-01	6.71E-01	3.31E-02	1.00E+00	-4.35E-01	9.53E-01	7.08E-01	9.33E-01
ENSBTAG00000007052	3.92E-01	1.00E+00	4.76E-01	9.89E-01	2.25E-01	1.00E+00	1.98E-01	1.00E+00	-3.21E+00	4.34E-01
ENSBTAG00000012833	7.44E-01	3.11E-01	1.86E+00	1.08E-06	8.54E-01	4.07E-02	2.04E-02	1.00E+00	3.36E-01	9.12E-01
ENSBTAG00000019836	1.89E-01	9.06E-01	-1.76E-01	5.86E-01	-6.86E-01	7.34E-04	-1.08E+00	4.81E-08	-2.39E-01	7.96E-01
ENSBTAG00000044547	0.00E+00	1.00E+00	0.00E+00	1.00E+00	-5.13E-15	1.00E+00	0.00E+00	1.00E+00	0.00E+00	1.00E+00
ENSBTAG00000044757	0.00E+00	1.00E+00	0.00E+00	1.00E+00	-5.13E-15	1.00E+00	0.00E+00	1.00E+00	0.00E+00	1.00E+00
ENSBTAG00000016854	-4.96E-02	1.00E+00	-5.74E-01	3.39E-01	-1.32E+00	6.13E-03	-1.45E+00	2.98E-03	-4.94E-01	8.00E-01
ENSBTAG00000004054	2.40E-01	7.84E-01	9.99E-01	3.02E-07	2.70E-01	2.87E-01	-1.82E-01	5.64E-01	-3.01E-01	6.01E-01
ENSBTAG00000014230	-1.10E-02	1.00E+00	-7.39E-01	4.15E-02	-3.39E-01	4.59E-01	-3.00E-01	5.47E-01	-3.12E-01	8.57E-01
ENSBTAG00000028043	0.00E+00	1.00E+00	0.00E+00	1.00E+00	-5.13E-15	1.00E+00	0.00E+00	1.00E+00	0.00E+00	1.00E+00
ENSBTAG00000031727	0.00E+00	1.00E+00	0.00E+00	1.00E+00	-5.13E-15	1.00E+00	0.00E+00	1.00E+00	0.00E+00	1.00E+00
ENSBTAG00000042869	0.00E+00	1.00E+00	0.00E+00	1.00E+00	-5.13E-15	1.00E+00	0.00E+00	1.00E+00	0.00E+00	1.00E+00
ENSBTAG00000018019	1.13E-01	1.00E+00	2.88E-01	7.89E-01	-1.93E-01	9.79E-01	6.45E-02	1.00E+00	2.99E-02	1.00E+00
ENSBTAG00000018310	-3.83E-01	2.30E-01	-1.15E+00	1.40E-10	-1.13E+00	2.27E-10	-1.10E+00	8.81E-10	-1.88E-02	1.00E+00
ENSBTAG00000018636	-9.39E-02	1.00E+00	5.95E-01	4.44E-03	4.49E-01	4.13E-02	2.63E-01	3.32E-01	-5.46E-02	1.00E+00
ENSBTAG00000025181	-4.40E-01	7.68E-01	5.93E-01	1.58E-01	-5.44E-01	2.08E-01	-1.21E+00	1.08E-03	-2.17E-01	1.00E+00
ENSBTAG00000026913	2.19E-02	1.00E+00	-3.12E-01	4.62E-01	-1.27E+00	1.84E-05	-7.00E-01	3.20E-02	9.91E-01	1.68E-02
ENSBTAG00000035544	-6.06E-01	8.23E-01	-6.59E-01	4.08E-01	-1.77E+00	6.31E-03	-9.60E-01	1.61E-01	4.92E-01	8.56E-01
ENSBTAG00000013491	4.87E-01	6.20E-01	1.45E+00	2.25E-05	9.72E-01	6.15E-03	1.08E+00	2.44E-03	7.79E-01	2.28E-01
ENSBTAG00000047143	0.00E+00	1.00E+00	0.00E+00	1.00E+00	-5.13E-15	1.00E+00	0.00E+00	1.00E+00	0.00E+00	1.00E+00
ENSBTAG00000009293	-3.68E-01	4.21E-01	-1.18E+00	1.35E-08	-1.71E+00	8.55E-17	-1.41E+00	1.18E-11	-4.69E-02	1.00E+00
ENSBTAG00000030073	0.00E+00	1.00E+00	0.00E+00	1.00E+00	-5.13E-15	1.00E+00	0.00E+00	1.00E+00	0.00E+00	1.00E+00
ENSBTAG00000009294	-5.31E-01	9.66E-01	-1.47E+00	3.89E-02	-1.62E+00	1.96E-02	-5.21E-01	6.22E-01	1.84E-01	1.00E+00

ENSBTAG00000045497	2.26E-02	1.00E+00	-4.15E-02	1.00E+00	-6.29E-01	5.87E-02	-4.09E-01	2.93E-01	3.74E-03	1.00E+00
ENSBTAG00000020819	-7.99E-02	1.00E+00	1.83E-01	5.64E-01	3.83E-01	9.62E-02	1.13E-01	8.66E-01	2.36E-02	1.00E+00
ENSBTAG00000013666	-2.54E-01	1.00E+00	-9.50E-01	4.30E-03	-8.32E-01	1.39E-02	3.23E-01	4.97E-01	-9.71E-02	1.00E+00
ENSBTAG00000029887	-3.88E+00	1.44E-01	-3.88E+00	3.63E-02	-3.88E+00	2.27E-02	-2.12E+00	1.79E-01	4.90E-01	1.00E+00
ENSBTAG00000008384	-2.80E-01	8.59E-01	-9.64E-01	6.35E-04	-2.46E+00	4.00E-19	-2.91E+00	2.74E-25	4.78E-01	4.82E-01
ENSBTAG00000004679	1.32E-02	1.00E+00	3.15E+00	8.21E-15	2.29E+00	7.21E-09	1.14E+00	6.75E-03	-2.01E-01	1.00E+00
ENSBTAG00000007233	2.12E-01	1.00E+00	3.46E+00	4.42E-30	2.07E+00	2.54E-12	6.05E-01	8.51E-02	-4.33E-04	1.00E+00
ENSBTAG00000017716	-4.15E-02	1.00E+00	-6.63E-02	1.00E+00	4.05E-02	1.00E+00	-2.12E-01	6.30E-01	4.27E-02	1.00E+00
ENSBTAG00000037899	4.38E-01	1.00E+00	-6.86E-01	5.50E-01	-7.19E-01	5.09E-01	-9.06E-01	3.80E-01	-4.85E-02	1.00E+00
ENSBTAG00000029792	0.00E+00	1.00E+00	0.00E+00	1.00E+00	-5.13E-15	1.00E+00	0.00E+00	1.00E+00	0.00E+00	1.00E+00
ENSBTAG00000037265	0.00E+00	1.00E+00	0.00E+00	1.00E+00	-5.13E-15	1.00E+00	0.00E+00	1.00E+00	0.00E+00	1.00E+00
ENSBTAG00000046585	0.00E+00	1.00E+00	0.00E+00	1.00E+00	-5.13E-15	1.00E+00	0.00E+00	1.00E+00	0.00E+00	1.00E+00
ENSBTAG00000030002	0.00E+00	1.00E+00	0.00E+00	1.00E+00	-5.13E-15	1.00E+00	0.00E+00	1.00E+00	0.00E+00	1.00E+00
ENSBTAG00000030004	0.00E+00	1.00E+00	0.00E+00	1.00E+00	-5.13E-15	1.00E+00	0.00E+00	1.00E+00	0.00E+00	1.00E+00
ENSBTAG00000030003	0.00E+00	1.00E+00	0.00E+00	1.00E+00	-5.13E-15	1.00E+00	0.00E+00	1.00E+00	0.00E+00	1.00E+00
ENSBTAG00000029911	0.00E+00	1.00E+00	0.00E+00	1.00E+00	-5.13E-15	1.00E+00	0.00E+00	1.00E+00	0.00E+00	1.00E+00
ENSBTAG00000029829	0.00E+00	1.00E+00	0.00E+00	1.00E+00	-5.13E-15	1.00E+00	0.00E+00	1.00E+00	0.00E+00	1.00E+00
ENSBTAG00000044345	0.00E+00	1.00E+00	0.00E+00	1.00E+00	-5.13E-15	1.00E+00	0.00E+00	1.00E+00	0.00E+00	1.00E+00
ENSBTAG00000047901	0.00E+00	1.00E+00	0.00E+00	1.00E+00	-5.13E-15	1.00E+00	0.00E+00	1.00E+00	0.00E+00	1.00E+00
ENSBTAG00000029782	0.00E+00	1.00E+00	0.00E+00	1.00E+00	-5.13E-15	1.00E+00	0.00E+00	1.00E+00	0.00E+00	1.00E+00
ENSBTAG00000044326	0.00E+00	1.00E+00	0.00E+00	1.00E+00	-5.13E-15	1.00E+00	0.00E+00	1.00E+00	0.00E+00	1.00E+00
ENSBTAG00000044330	0.00E+00	1.00E+00	0.00E+00	1.00E+00	-5.13E-15	1.00E+00	0.00E+00	1.00E+00	0.00E+00	1.00E+00
ENSBTAG00000044645	0.00E+00	1.00E+00	0.00E+00	1.00E+00	-5.13E-15	1.00E+00	0.00E+00	1.00E+00	0.00E+00	1.00E+00
ENSBTAG00000042727	0.00E+00	1.00E+00	0.00E+00	1.00E+00	-5.13E-15	1.00E+00	0.00E+00	1.00E+00	0.00E+00	1.00E+00
ENSBTAG00000045326	0.00E+00	1.00E+00	0.00E+00	1.00E+00	1.78E+00	4.79E-01	0.00E+00	1.00E+00	1.91E+00	8.00E-01
ENSBTAG00000043859	1.92E+00	8.23E-01	0.00E+00	1.00E+00	-5.13E-15	1.00E+00	0.00E+00	1.00E+00	2.71E+00	6.01E-01
ENSBTAG00000044760	0.00E+00	1.00E+00	0.00E+00	1.00E+00	-5.13E-15	1.00E+00	0.00E+00	1.00E+00	0.00E+00	1.00E+00
ENSBTAG00000042739	0.00E+00	1.00E+00	0.00E+00	1.00E+00	2.56E+00	2.54E-01	0.00E+00	1.00E+00	0.00E+00	1.00E+00
ENSBTAG00000045159	0.00E+00	1.00E+00	0.00E+00	1.00E+00	1.78E+00	4.79E-01	0.00E+00	1.00E+00	0.00E+00	1.00E+00
ENSBTAG00000043342	0.00E+00	1.00E+00	0.00E+00	1.00E+00	-5.13E-15	1.00E+00	0.00E+00	1.00E+00	0.00E+00	1.00E+00
ENSBTAG00000042401	-6.19E-04	1.00E+00	-1.91E+00	4.69E-01	-1.37E-01	1.00E+00	-1.91E+00	4.39E-01	1.17E-02	1.00E+00
ENSBTAG00000042882	0.00E+00	1.00E+00	1.98E+00	4.43E-01	3.96E+00	2.25E-02	2.53E+00	2.71E-01	3.21E+00	4.34E-01
ENSBTAG00000043354	0.00E+00	1.00E+00	0.00E+00	1.00E+00	-5.13E-15	1.00E+00	0.00E+00	1.00E+00	0.00E+00	1.00E+00
ENSBTAG00000042346	0.00E+00	1.00E+00	0.00E+00	1.00E+00	-5.13E-15	1.00E+00	0.00E+00	1.00E+00	1.91E+00	8.00E-01
ENSBTAG00000043040	0.00E+00	1.00E+00	0.00E+00	1.00E+00	1.78E+00	4.79E-01	0.00E+00	1.00E+00	0.00E+00	1.00E+00

ENSBTAG00000042588	0.00E+00	1.00E+00	0.00E+00	1.00E+00	-5.13E-15	1.00E+00	0.00E+00	1.00E+00	0.00E+00	1.00E+00
ENSBTAG00000042199	0.00E+00	1.00E+00	1.98E+00	4.43E-01	-5.13E-15	1.00E+00	1.76E+00	4.97E-01	0.00E+00	1.00E+00
ENSBTAG00000042508	1.91E+00	8.23E-01	0.00E+00	1.00E+00	-5.13E-15	1.00E+00	0.00E+00	1.00E+00	2.71E+00	6.01E-01
ENSBTAG00000043417	0.00E+00	1.00E+00	0.00E+00	1.00E+00	-5.13E-15	1.00E+00	0.00E+00	1.00E+00	0.00E+00	1.00E+00
ENSBTAG00000042412	0.00E+00	1.00E+00	1.98E+00	4.43E-01	-5.13E-15	1.00E+00	0.00E+00	1.00E+00	0.00E+00	1.00E+00
ENSBTAG00000043179	-1.91E+00	8.23E-01	-1.91E+00	4.69E-01	-1.91E+00	4.28E-01	-1.91E+00	4.39E-01	-1.91E+00	8.00E-01
ENSBTAG00000042679	1.03E-02	1.00E+00	-1.49E-01	1.00E+00	-1.68E-01	1.00E+00	-7.36E-01	7.07E-01	-1.43E+00	7.66E-01
ENSBTAG00000046420	-8.99E-03	1.00E+00	-5.37E-01	7.13E-01	-1.15E+00	2.27E-01	-4.42E-01	8.03E-01	-5.05E-01	1.00E+00
ENSBTAG00000042845	1.92E+00	8.23E-01	0.00E+00	1.00E+00	-5.13E-15	1.00E+00	0.00E+00	1.00E+00	0.00E+00	1.00E+00
ENSBTAG00000048101	0.00E+00	1.00E+00	0.00E+00	1.00E+00	1.78E+00	4.79E-01	0.00E+00	1.00E+00	0.00E+00	1.00E+00
ENSBTAG00000042603	0.00E+00	1.00E+00	0.00E+00	1.00E+00	-5.13E-15	1.00E+00	0.00E+00	1.00E+00	0.00E+00	1.00E+00
ENSBTAG00000042690	-1.10E+00	7.96E-01	-2.71E+00	3.64E-02	-4.69E+00	1.43E-03	-2.16E+00	6.55E-02	-2.78E+00	2.00E-01
ENSBTAG00000043034	0.00E+00	1.00E+00	0.00E+00	1.00E+00	-5.13E-15	1.00E+00	0.00E+00	1.00E+00	0.00E+00	1.00E+00
ENSBTAG00000042543	5.64E-01	1.00E+00	-1.47E-01	1.00E+00	2.23E-01	1.00E+00	-4.25E-01	9.61E-01	4.28E-01	1.00E+00
ENSBTAG00000043490	3.59E+00	5.84E-03	1.77E+00	3.26E-01	1.82E+00	2.76E-01	1.13E+00	6.66E-01	2.22E+00	4.75E-01
ENSBTAG00000042442	1.84E-02	1.00E+00	-3.21E+00	1.26E-01	-3.21E+00	9.50E-02	-6.71E-01	8.92E-01	-3.21E+00	4.34E-01
ENSBTAG00000043413	0.00E+00	1.00E+00	0.00E+00	1.00E+00	-5.13E-15	1.00E+00	0.00E+00	1.00E+00	0.00E+00	1.00E+00
ENSBTAG00000042519	0.00E+00	1.00E+00	0.00E+00	1.00E+00	-5.13E-15	1.00E+00	0.00E+00	1.00E+00	0.00E+00	1.00E+00
ENSBTAG00000042953	0.00E+00	1.00E+00	2.79E+00	2.10E-01	-5.13E-15	1.00E+00	0.00E+00	1.00E+00	0.00E+00	1.00E+00
ENSBTAG00000042365	0.00E+00	1.00E+00	0.00E+00	1.00E+00	-5.13E-15	1.00E+00	0.00E+00	1.00E+00	0.00E+00	1.00E+00
ENSBTAG00000043933	0.00E+00	1.00E+00	0.00E+00	1.00E+00	-5.13E-15	1.00E+00	0.00E+00	1.00E+00	0.00E+00	1.00E+00
ENSBTAG00000042438	0.00E+00	1.00E+00	0.00E+00	1.00E+00	-5.13E-15	1.00E+00	0.00E+00	1.00E+00	0.00E+00	1.00E+00
ENSBTAG00000043314	1.92E+00	8.23E-01	0.00E+00	1.00E+00	-5.13E-15	1.00E+00	0.00E+00	1.00E+00	0.00E+00	1.00E+00
ENSBTAG00000044977	0.00E+00	1.00E+00	0.00E+00	1.00E+00	-5.13E-15	1.00E+00	0.00E+00	1.00E+00	0.00E+00	1.00E+00
ENSBTAG00000042805	1.92E+00	8.23E-01	0.00E+00	1.00E+00	-5.13E-15	1.00E+00	0.00E+00	1.00E+00	0.00E+00	1.00E+00
ENSBTAG00000043670	0.00E+00	1.00E+00	0.00E+00	1.00E+00	-5.13E-15	1.00E+00	0.00E+00	1.00E+00	0.00E+00	1.00E+00
ENSBTAG00000045422	0.00E+00	1.00E+00	0.00E+00	1.00E+00	-5.13E-15	1.00E+00	0.00E+00	1.00E+00	0.00E+00	1.00E+00
ENSBTAG00000043694	0.00E+00	1.00E+00	0.00E+00	1.00E+00	-5.13E-15	1.00E+00	0.00E+00	1.00E+00	0.00E+00	1.00E+00
ENSBTAG00000036379	0.00E+00	1.00E+00	0.00E+00	1.00E+00	-5.13E-15	1.00E+00	0.00E+00	1.00E+00	0.00E+00	1.00E+00
ENSBTAG00000029845	0.00E+00	1.00E+00	0.00E+00	1.00E+00	-5.13E-15	1.00E+00	0.00E+00	1.00E+00	0.00E+00	1.00E+00
ENSBTAG00000036399	0.00E+00	1.00E+00	0.00E+00	1.00E+00	-5.13E-15	1.00E+00	0.00E+00	1.00E+00	0.00E+00	1.00E+00
ENSBTAG00000029770	0.00E+00	1.00E+00	0.00E+00	1.00E+00	-5.13E-15	1.00E+00	0.00E+00	1.00E+00	0.00E+00	1.00E+00
ENSBTAG00000045611	0.00E+00	1.00E+00	0.00E+00	1.00E+00	-5.13E-15	1.00E+00	0.00E+00	1.00E+00	0.00E+00	1.00E+00
ENSBTAG00000039219	0.00E+00	1.00E+00	0.00E+00	1.00E+00	-5.13E-15	1.00E+00	0.00E+00	1.00E+00	0.00E+00	1.00E+00
ENSBTAG00000029820	0.00E+00	1.00E+00	0.00E+00	1.00E+00	-5.13E-15	1.00E+00	0.00E+00	1.00E+00	0.00E+00	1.00E+00

ENSBTAG00000036444	0.00E+00	1.00E+00	0.00E+00	1.00E+00	-5.13E-15	1.00E+00	0.00E+00	1.00E+00	0.00E+00	1.00E+00
ENSBTAG00000036383	0.00E+00	1.00E+00	0.00E+00	1.00E+00	-5.13E-15	1.00E+00	0.00E+00	1.00E+00	0.00E+00	1.00E+00
ENSBTAG00000036356	0.00E+00	1.00E+00	0.00E+00	1.00E+00	-5.13E-15	1.00E+00	0.00E+00	1.00E+00	0.00E+00	1.00E+00
ENSBTAG00000036396	0.00E+00	1.00E+00	0.00E+00	1.00E+00	-5.13E-15	1.00E+00	0.00E+00	1.00E+00	0.00E+00	1.00E+00
ENSBTAG00000040254	0.00E+00	1.00E+00	0.00E+00	1.00E+00	-5.13E-15	1.00E+00	0.00E+00	1.00E+00	0.00E+00	1.00E+00
ENSBTAG00000036431	0.00E+00	1.00E+00	0.00E+00	1.00E+00	-5.13E-15	1.00E+00	0.00E+00	1.00E+00	0.00E+00	1.00E+00
ENSBTAG00000036360	0.00E+00	1.00E+00	0.00E+00	1.00E+00	-5.13E-15	1.00E+00	0.00E+00	1.00E+00	0.00E+00	1.00E+00
ENSBTAG00000048146	0.00E+00	1.00E+00	0.00E+00	1.00E+00	1.78E+00	4.79E-01	0.00E+00	1.00E+00	0.00E+00	1.00E+00
ENSBTAG00000046428	0.00E+00	1.00E+00	0.00E+00	1.00E+00	-5.13E-15	1.00E+00	0.00E+00	1.00E+00	0.00E+00	1.00E+00
ENSBTAG00000044865	0.00E+00	1.00E+00	0.00E+00	1.00E+00	-5.13E-15	1.00E+00	0.00E+00	1.00E+00	0.00E+00	1.00E+00
ENSBTAG00000039118	0.00E+00	1.00E+00	0.00E+00	1.00E+00	-5.13E-15	1.00E+00	0.00E+00	1.00E+00	0.00E+00	1.00E+00
ENSBTAG00000030017	0.00E+00	1.00E+00	0.00E+00	1.00E+00	-5.13E-15	1.00E+00	0.00E+00	1.00E+00	0.00E+00	1.00E+00
ENSBTAG00000040013	0.00E+00	1.00E+00	0.00E+00	1.00E+00	-5.13E-15	1.00E+00	0.00E+00	1.00E+00	0.00E+00	1.00E+00
ENSBTAG00000038423	0.00E+00	1.00E+00	0.00E+00	1.00E+00	-5.13E-15	1.00E+00	0.00E+00	1.00E+00	0.00E+00	1.00E+00
ENSBTAG00000038206	0.00E+00	1.00E+00	0.00E+00	1.00E+00	-5.13E-15	1.00E+00	0.00E+00	1.00E+00	0.00E+00	1.00E+00
ENSBTAG00000047640	0.00E+00	1.00E+00	0.00E+00	1.00E+00	-5.13E-15	1.00E+00	0.00E+00	1.00E+00	0.00E+00	1.00E+00
ENSBTAG00000036368	0.00E+00	1.00E+00	0.00E+00	1.00E+00	-5.13E-15	1.00E+00	0.00E+00	1.00E+00	0.00E+00	1.00E+00
ENSBTAG00000030026	0.00E+00	1.00E+00	0.00E+00	1.00E+00	-5.13E-15	1.00E+00	0.00E+00	1.00E+00	0.00E+00	1.00E+00
ENSBTAG00000036433	0.00E+00	1.00E+00	0.00E+00	1.00E+00	-5.13E-15	1.00E+00	0.00E+00	1.00E+00	0.00E+00	1.00E+00
ENSBTAG00000030035	0.00E+00	1.00E+00	0.00E+00	1.00E+00	-5.13E-15	1.00E+00	0.00E+00	1.00E+00	0.00E+00	1.00E+00
ENSBTAG00000036398	0.00E+00	1.00E+00	0.00E+00	1.00E+00	-5.13E-15	1.00E+00	0.00E+00	1.00E+00	0.00E+00	1.00E+00
ENSBTAG00000046480	0.00E+00	1.00E+00	0.00E+00	1.00E+00	1.78E+00	4.79E-01	0.00E+00	1.00E+00	0.00E+00	1.00E+00
ENSBTAG00000029995	0.00E+00	1.00E+00	0.00E+00	1.00E+00	-5.13E-15	1.00E+00	0.00E+00	1.00E+00	0.00E+00	1.00E+00
ENSBTAG00000029791	0.00E+00	1.00E+00	0.00E+00	1.00E+00	-5.13E-15	1.00E+00	0.00E+00	1.00E+00	0.00E+00	1.00E+00
ENSBTAG00000029789	0.00E+00	1.00E+00	0.00E+00	1.00E+00	-5.13E-15	1.00E+00	0.00E+00	1.00E+00	0.00E+00	1.00E+00
ENSBTAG00000047451	0.00E+00	1.00E+00	0.00E+00	1.00E+00	-5.13E-15	1.00E+00	0.00E+00	1.00E+00	0.00E+00	1.00E+00
ENSBTAG00000036402	0.00E+00	1.00E+00	0.00E+00	1.00E+00	-5.13E-15	1.00E+00	0.00E+00	1.00E+00	0.00E+00	1.00E+00
ENSBTAG00000036445	0.00E+00	1.00E+00	0.00E+00	1.00E+00	-5.13E-15	1.00E+00	0.00E+00	1.00E+00	0.00E+00	1.00E+00
ENSBTAG00000036426	0.00E+00	1.00E+00	0.00E+00	1.00E+00	-5.13E-15	1.00E+00	0.00E+00	1.00E+00	0.00E+00	1.00E+00
ENSBTAG00000046198	0.00E+00	1.00E+00	0.00E+00	1.00E+00	-5.13E-15	1.00E+00	0.00E+00	1.00E+00	0.00E+00	1.00E+00
ENSBTAG00000036415	0.00E+00	1.00E+00	0.00E+00	1.00E+00	-5.13E-15	1.00E+00	0.00E+00	1.00E+00	0.00E+00	1.00E+00
ENSBTAG00000029907	0.00E+00	1.00E+00	0.00E+00	1.00E+00	-5.13E-15	1.00E+00	0.00E+00	1.00E+00	0.00E+00	1.00E+00
ENSBTAG00000037263	0.00E+00	1.00E+00	0.00E+00	1.00E+00	-5.13E-15	1.00E+00	0.00E+00	1.00E+00	0.00E+00	1.00E+00
ENSBTAG00000046141	0.00E+00	1.00E+00	0.00E+00	1.00E+00	-5.13E-15	1.00E+00	0.00E+00	1.00E+00	0.00E+00	1.00E+00
ENSBTAG00000029961	0.00E+00	1.00E+00	0.00E+00	1.00E+00	-5.13E-15	1.00E+00	0.00E+00	1.00E+00	0.00E+00	1.00E+00

ENSBTAG00000029808	0.00E+00	1.00E+00	0.00E+00	1.00E+00	-5.13E-15	1.00E+00	0.00E+00	1.00E+00	0.00E+00	1.00E+00
ENSBTAG00000029794	0.00E+00	1.00E+00	0.00E+00	1.00E+00	-5.13E-15	1.00E+00	0.00E+00	1.00E+00	0.00E+00	1.00E+00
ENSBTAG00000036364	0.00E+00	1.00E+00	0.00E+00	1.00E+00	-5.13E-15	1.00E+00	0.00E+00	1.00E+00	0.00E+00	1.00E+00
ENSBTAG00000047889	3.84E-01	1.00E+00	-3.22E+00	1.25E-01	-3.22E+00	9.40E-02	-3.22E+00	9.88E-02	-1.29E+00	9.02E-01
ENSBTAG00000030016	8.03E-01	1.00E+00	7.58E-02	1.00E+00	1.52E+00	4.28E-01	6.28E-01	9.94E-01	7.16E-03	1.00E+00
ENSBTAG00000038073	1.50E+00	5.76E-01	4.32E+00	1.47E-10	5.72E+00	3.69E-22	4.82E+00	3.36E-14	6.96E-01	1.00E+00
ENSBTAG00000043578	2.01E+00	5.78E-01	6.33E+00	3.92E-13	7.51E+00	8.20E-20	7.71E+00	7.65E-21	1.30E+00	9.11E-01
ENSBTAG00000037977	3.61E+00	2.37E-01	8.25E+00	6.51E-15	9.18E+00	2.72E-20	9.27E+00	1.23E-20	0.00E+00	1.00E+00
ENSBTAG00000020192	5.70E-03	1.00E+00	-6.71E-01	2.76E-04	-1.16E+00	6.95E-11	-1.19E+00	2.69E-11	-1.86E-01	8.27E-01
ENSBTAG00000016598	6.65E-02	1.00E+00	4.59E-01	7.23E-02	5.98E-01	1.29E-02	5.68E-01	2.21E-02	2.18E-01	8.64E-01
ENSBTAG00000016603	1.73E-01	1.00E+00	3.51E-01	5.40E-01	5.19E-01	2.54E-01	-5.06E-01	3.38E-01	-6.04E-01	6.01E-01
ENSBTAG00000006270	9.94E-01	2.19E-02	3.29E+00	1.20E-22	2.63E+00	9.92E-16	1.88E+00	9.87E-09	3.16E-01	8.59E-01
ENSBTAG00000007007	-8.86E-02	1.00E+00	3.38E-01	1.36E-01	5.39E-01	7.19E-03	3.15E-01	1.82E-01	4.82E-02	1.00E+00
ENSBTAG00000007008	2.85E-01	9.68E-01	-1.39E+00	3.28E-04	-1.02E+00	6.93E-03	-1.19E-01	1.00E+00	4.37E-01	7.23E-01
ENSBTAG00000007009	0.00E+00	1.00E+00	0.00E+00	1.00E+00	-5.13E-15	1.00E+00	0.00E+00	1.00E+00	0.00E+00	1.00E+00
ENSBTAG00000044797	0.00E+00	1.00E+00	1.99E+00	4.43E-01	-5.13E-15	1.00E+00	0.00E+00	1.00E+00	0.00E+00	1.00E+00
ENSBTAG00000010993	-3.48E-01	6.15E-01	-8.10E-01	1.22E-03	-3.77E-03	1.00E+00	-3.38E-01	2.79E-01	5.31E-02	1.00E+00
ENSBTAG00000010995	-5.81E-02	1.00E+00	-4.01E-02	1.00E+00	-1.51E-01	7.04E-01	-2.77E-01	3.13E-01	-9.25E-02	1.00E+00
ENSBTAG00000043100	0.00E+00	1.00E+00	0.00E+00	1.00E+00	-5.13E-15	1.00E+00	0.00E+00	1.00E+00	0.00E+00	1.00E+00
ENSBTAG00000012143	-1.56E-01	1.00E+00	-3.03E-01	3.85E-01	3.96E-01	1.84E-01	6.19E-01	2.10E-02	2.37E-01	8.71E-01
ENSBTAG00000010882	-7.83E-02	1.00E+00	-6.63E-02	1.00E+00	1.02E+00	3.64E-03	1.41E+00	3.95E-05	2.59E-02	1.00E+00
ENSBTAG00000002336	5.88E-01	1.26E-01	8.38E-01	7.14E-04	6.78E-02	1.00E+00	4.17E-01	1.59E-01	1.81E-01	1.00E+00
ENSBTAG00000046633	4.49E-03	1.00E+00	-3.95E-01	3.77E-01	-4.96E-01	2.15E-01	-3.84E-01	4.07E-01	-6.19E-01	4.15E-01
ENSBTAG00000013475	4.48E-01	7.32E-01	1.36E+00	7.62E-05	8.55E-01	1.97E-02	1.23E+00	4.44E-04	1.86E-01	1.00E+00
ENSBTAG00000015750	-4.13E-01	1.00E+00	9.51E-02	1.00E+00	-9.16E-01	1.73E-01	-1.08E+00	1.04E-01	3.18E-01	1.00E+00
ENSBTAG00000016456	-4.19E-02	1.00E+00	-4.82E-01	4.10E-02	2.24E-01	4.73E-01	1.85E-01	6.07E-01	4.02E-01	4.23E-01
ENSBTAG00000026893	1.08E-01	1.00E+00	9.39E-01	3.27E-01	1.06E+00	2.40E-01	1.58E-02	1.00E+00	-2.39E-02	1.00E+00
ENSBTAG00000035995	3.52E+00	1.45E-07	2.58E+00	2.41E-05	9.81E-01	1.62E-01	9.81E-01	1.73E-01	-1.05E-01	1.00E+00
ENSBTAG00000047319	-4.43E-01	5.87E-01	-1.66E+00	3.75E-08	-1.50E+00	5.97E-07	-5.36E-01	1.35E-01	8.13E-02	1.00E+00
ENSBTAG00000016282	-1.63E-01	1.00E+00	7.80E-01	2.28E-04	7.13E-01	8.28E-04	6.10E-01	6.12E-03	-2.26E-01	8.00E-01
ENSBTAG00000042713	0.00E+00	1.00E+00	0.00E+00	1.00E+00	-5.13E-15	1.00E+00	0.00E+00	1.00E+00	0.00E+00	1.00E+00
ENSBTAG00000013410	1.26E-01	1.00E+00	8.05E-01	3.94E-05	8.88E-01	4.41E-06	3.67E-01	1.12E-01	-2.04E-01	8.17E-01
ENSBTAG00000035998	-1.89E-01	1.00E+00	-1.26E+00	1.89E-04	-1.27E+00	1.60E-04	-1.05E-01	1.00E+00	-3.82E-01	8.00E-01
ENSBTAG00000031327	-1.38E-01	1.00E+00	1.56E+00	4.34E-13	2.08E+00	3.24E-22	1.88E+00	3.09E-18	-4.53E-01	3.55E-01
ENSBTAG00000017361	3.04E-01	6.21E-01	1.54E+00	1.47E-14	1.27E+00	2.55E-10	1.04E+00	3.83E-07	2.63E-02	1.00E+00

ENSBTAG00000021199	6.92E-02	1.00E+00	-1.82E-01	6.63E-01	-3.23E-01	2.69E-01	-1.26E-01	8.74E-01	-2.74E-02	1.00E+00
ENSBTAG00000017299	8.56E-02	1.00E+00	5.63E-01	1.30E-02	1.29E-01	8.17E-01	-2.11E-01	5.14E-01	-5.31E-02	1.00E+00
ENSBTAG00000009966	-2.04E-01	1.00E+00	8.48E-01	2.54E-03	-6.55E-01	2.64E-02	-1.65E+00	1.60E-09	1.91E-01	1.00E+00
ENSBTAG00000003556	-7.99E-02	1.00E+00	1.44E-01	8.06E-01	-1.06E+00	3.31E-06	-1.16E+00	4.27E-07	8.84E-02	1.00E+00
ENSBTAG00000003559	-3.80E-01	4.03E-01	-4.52E-01	5.79E-02	-3.48E-02	1.00E+00	-4.04E-01	1.07E-01	-1.69E-01	9.77E-01
ENSBTAG00000026886	-2.36E-01	9.80E-01	-4.26E-01	2.22E-01	-4.98E-01	1.32E-01	-5.51E-01	9.30E-02	-2.63E-01	8.89E-01
ENSBTAG00000046186	0.00E+00	1.00E+00	0.00E+00	1.00E+00	-5.13E-15	1.00E+00	0.00E+00	1.00E+00	0.00E+00	1.00E+00
ENSBTAG00000020402	-4.42E-01	1.00E+00	2.19E-01	1.00E+00	-1.43E+00	7.84E-02	-2.45E-01	1.00E+00	-1.65E-01	1.00E+00
ENSBTAG00000046401	0.00E+00	1.00E+00	0.00E+00	1.00E+00	-5.13E-15	1.00E+00	0.00E+00	1.00E+00	0.00E+00	1.00E+00
ENSBTAG00000017194	-1.33E-01	1.00E+00	-3.17E-01	6.89E-01	-1.14E+00	1.05E-02	-1.59E+00	2.86E-04	9.90E-02	1.00E+00
ENSBTAG00000021904	-6.17E-01	3.34E-01	-2.14E+00	2.51E-10	-6.75E-01	5.96E-02	-8.40E-01	1.63E-02	-1.15E-01	1.00E+00
ENSBTAG00000022775	1.18E-01	1.00E+00	-2.49E+00	1.75E-10	-4.58E-01	3.53E-01	-1.74E-01	9.41E-01	3.23E-01	9.12E-01
ENSBTAG00000006673	9.28E-01	9.50E-01	-4.30E-01	1.00E+00	1.46E+00	2.19E-01	9.16E-01	5.84E-01	4.96E-03	1.00E+00
ENSBTAG00000007187	2.03E-02	1.00E+00	-9.45E-02	1.00E+00	-1.33E-01	9.47E-01	-7.67E-01	1.45E-02	-1.21E-03	1.00E+00
ENSBTAG00000017616	-1.60E-01	1.00E+00	-1.09E+00	2.75E-05	-2.08E+00	7.32E-16	-1.93E+00	8.48E-14	-2.18E-01	9.37E-01
ENSBTAG00000017622	-1.38E-01	1.00E+00	-3.53E-01	3.27E-01	-1.16E+00	3.30E-05	-6.15E-01	4.34E-02	-1.91E-02	1.00E+00
ENSBTAG00000017636	-1.81E-02	1.00E+00	9.72E-01	5.16E-08	1.03E+00	6.14E-09	8.03E-01	1.07E-05	7.73E-02	1.00E+00
ENSBTAG00000005038	1.16E-01	1.00E+00	2.14E+00	1.94E-08	1.99E+00	1.61E-07	1.46E+00	2.28E-04	5.37E-01	6.93E-01
ENSBTAG00000004802	-6.71E-01	2.05E-01	-7.63E-01	2.15E-02	-1.36E+00	1.13E-05	-7.46E-01	2.82E-02	-2.86E-01	8.91E-01
ENSBTAG00000015160	-1.87E-01	1.00E+00	-2.01E+00	6.96E-09	-1.26E+00	1.69E-04	-9.23E-01	8.14E-03	4.71E-02	1.00E+00
ENSBTAG00000046828	6.80E-01	1.00E+00	1.23E+00	3.90E-01	1.78E+00	9.02E-02	1.43E+00	2.49E-01	1.32E+00	7.22E-01
ENSBTAG00000010370	2.27E-01	1.00E+00	4.59E-01	4.20E-01	9.00E-01	3.92E-02	6.11E-01	2.24E-01	6.40E-01	5.66E-01
ENSBTAG00000031242	-1.80E-01	1.00E+00	1.84E+00	1.11E-05	2.13E+00	1.52E-07	1.91E+00	4.38E-06	9.95E-02	1.00E+00
ENSBTAG00000005647	1.34E+00	2.98E-02	1.67E-01	1.00E+00	-2.62E-01	8.90E-01	-1.89E-01	1.00E+00	4.90E-02	1.00E+00
ENSBTAG00000007319	-8.85E-01	1.78E-01	-2.32E+00	5.37E-08	-2.83E+00	4.73E-11	-2.59E+00	1.14E-09	-1.59E-01	1.00E+00
ENSBTAG00000048010	-4.76E-01	4.18E-01	-1.43E+00	1.47E-07	-2.50E+00	1.29E-19	-2.79E+00	2.28E-23	-3.30E-01	7.82E-01
ENSBTAG00000012753	-1.55E-01	1.00E+00	-7.29E-01	1.65E-02	2.71E-01	4.94E-01	1.93E-01	7.39E-01	1.05E-02	1.00E+00
ENSBTAG00000046364	-1.03E-01	1.00E+00	-6.11E-01	2.64E-03	-1.26E+00	5.06E-11	-1.48E+00	1.93E-14	3.48E-02	1.00E+00
ENSBTAG00000012751	0.00E+00	1.00E+00	0.00E+00	1.00E+00	-5.13E-15	1.00E+00	0.00E+00	1.00E+00	0.00E+00	1.00E+00
ENSBTAG00000046346	0.00E+00	1.00E+00	0.00E+00	1.00E+00	-5.13E-15	1.00E+00	0.00E+00	1.00E+00	0.00E+00	1.00E+00
ENSBTAG00000047606	0.00E+00	1.00E+00	0.00E+00	1.00E+00	-5.13E-15	1.00E+00	0.00E+00	1.00E+00	0.00E+00	1.00E+00
ENSBTAG00000047229	3.93E-01	9.44E-01	-5.30E-01	3.88E-01	-1.48E+00	1.37E-03	-1.71E+00	2.20E-04	2.43E-02	1.00E+00
ENSBTAG00000046857	-4.57E-03	1.00E+00	-9.26E-01	1.05E-01	-2.90E-01	7.72E-01	1.50E+00	7.34E-05	4.28E-01	8.24E-01
ENSBTAG00000047970	-1.11E-01	1.00E+00	-2.24E-01	9.44E-01	-6.12E-01	3.26E-01	-4.85E-01	4.97E-01	-3.70E-01	9.83E-01
ENSBTAG00000045594	-5.80E-01	1.00E+00	-2.09E+00	4.08E-02	-2.33E+00	1.72E-02	-2.37E+00	1.73E-02	-1.79E+00	3.39E-01

ENSBTAG00000046455	0.00E+00	1.00E+00	1.98E+00	4.43E-01	1.78E+00	4.79E-01	0.00E+00	1.00E+00	0.00E+00	1.00E+00
ENSBTAG00000047700	4.04E-01	6.87E-01	8.19E-02	1.00E+00	-1.74E-01	8.48E-01	1.09E-01	1.00E+00	9.90E-01	1.58E-02
ENSBTAG00000047959	1.33E-02	1.00E+00	-1.91E+00	4.69E-01	-1.91E+00	4.28E-01	-1.91E+00	4.39E-01	7.05E-03	1.00E+00
ENSBTAG00000047769	0.00E+00	1.00E+00	0.00E+00	1.00E+00	-5.13E-15	1.00E+00	0.00E+00	1.00E+00	1.91E+00	8.00E-01
ENSBTAG00000047632	-2.92E-01	1.00E+00	-1.08E+00	2.50E-01	-8.60E-01	3.56E-01	-1.37E+00	1.18E-01	-5.21E-01	9.98E-01
ENSBTAG00000047438	-2.71E+00	5.76E-01	-7.23E-01	9.45E-01	-2.71E+00	1.99E-01	6.99E-01	8.53E-01	-2.71E+00	6.01E-01
ENSBTAG00000048049	1.73E-01	1.00E+00	4.31E-02	1.00E+00	-3.18E-01	9.62E-01	-4.38E-01	8.17E-01	3.55E-01	1.00E+00
ENSBTAG00000045926	2.44E-01	1.00E+00	9.26E-01	4.44E-01	6.43E-01	6.84E-01	3.81E-01	9.79E-01	-2.28E-01	1.00E+00
ENSBTAG00000045836	1.66E-01	1.00E+00	4.46E-01	6.05E-01	-8.34E-02	1.00E+00	4.69E-02	1.00E+00	3.01E-01	1.00E+00
ENSBTAG00000046161	1.22E-01	1.00E+00	-7.60E-02	1.00E+00	-5.21E-01	6.92E-01	-4.77E-01	7.60E-01	5.62E-01	9.66E-01
ENSBTAG00000048268	2.68E-01	1.00E+00	4.95E-02	1.00E+00	-3.57E-01	9.22E-01	-4.27E-01	8.45E-01	6.07E-01	9.49E-01
ENSBTAG00000001213	-1.69E+00	7.74E-01	-1.62E+00	4.03E-01	-5.40E-01	9.32E-01	-5.69E-01	9.15E-01	-1.69E+00	7.78E-01
ENSBTAG00000046076	-2.88E-01	1.00E+00	-1.78E-01	1.00E+00	-4.60E-01	7.04E-01	-3.89E-01	8.13E-01	5.36E-01	9.28E-01
ENSBTAG00000046011	1.66E-01	1.00E+00	6.72E-02	1.00E+00	3.39E-01	6.93E-01	1.13E+00	1.34E-02	7.35E-01	5.37E-01
ENSBTAG00000046634	0.00E+00	1.00E+00	0.00E+00	1.00E+00	-5.13E-15	1.00E+00	0.00E+00	1.00E+00	0.00E+00	1.00E+00
ENSBTAG00000045666	0.00E+00	1.00E+00	0.00E+00	1.00E+00	-5.13E-15	1.00E+00	0.00E+00	1.00E+00	0.00E+00	1.00E+00
ENSBTAG00000037517	0.00E+00	1.00E+00	0.00E+00	1.00E+00	-5.13E-15	1.00E+00	0.00E+00	1.00E+00	0.00E+00	1.00E+00
ENSBTAG00000023954	3.17E-01	1.00E+00	-1.60E+00	4.10E-01	-1.80E+00	2.96E-01	1.17E-01	1.00E+00	-3.67E-01	1.00E+00
ENSBTAG00000007398	-2.38E-01	1.00E+00	-1.70E+00	6.89E-05	-9.62E-01	3.23E-02	2.12E-01	8.98E-01	-1.49E-01	1.00E+00
ENSBTAG00000009453	-3.70E-01	5.76E-01	-9.06E-01	3.21E-04	4.47E-01	9.50E-02	6.15E-01	1.47E-02	1.96E-01	9.52E-01
ENSBTAG00000003997	-1.66E-01	1.00E+00	-7.72E-01	4.35E-04	-1.21E+00	1.12E-08	-1.07E+00	6.67E-07	1.12E-01	1.00E+00
ENSBTAG00000017547	-3.92E-02	1.00E+00	-3.12E-01	2.37E-01	6.43E-02	1.00E+00	1.87E-01	5.88E-01	-2.41E-02	1.00E+00
ENSBTAG00000014547	-7.63E-01	5.64E-01	1.28E+00	2.06E-03	-6.87E-01	2.47E-01	-1.25E+00	2.25E-02	4.18E-01	8.61E-01
ENSBTAG00000012073	-1.37E-01	1.00E+00	1.25E-01	1.00E+00	1.05E-01	1.00E+00	8.76E-02	1.00E+00	-4.15E-01	8.06E-01
ENSBTAG00000018026	2.12E-01	9.43E-01	1.23E+00	8.68E-08	1.39E+00	9.91E-10	1.11E+00	2.10E-06	-2.34E-03	1.00E+00
ENSBTAG00000011628	5.55E-01	1.65E-01	2.20E+00	3.15E-20	2.02E+00	1.70E-17	1.57E+00	4.87E-11	9.35E-01	3.66E-03
ENSBTAG00000029403	0.00E+00	1.00E+00	0.00E+00	1.00E+00	-5.13E-15	1.00E+00	0.00E+00	1.00E+00	0.00E+00	1.00E+00
ENSBTAG00000015897	-1.26E+00	3.85E-01	-1.36E+00	9.99E-02	-9.88E-01	2.23E-01	-2.11E+00	9.30E-03	-2.57E+00	4.38E-02
ENSBTAG00000014971	1.07E+00	6.56E-03	2.59E+00	2.56E-16	2.71E+00	1.19E-17	2.43E+00	1.45E-14	6.18E-02	1.00E+00
ENSBTAG00000000220	-1.40E-03	1.00E+00	1.85E+00	7.03E-04	5.51E-02	1.00E+00	-5.64E-01	4.97E-01	7.28E-02	1.00E+00
ENSBTAG00000044231	0.00E+00	1.00E+00	0.00E+00	1.00E+00	-5.13E-15	1.00E+00	0.00E+00	1.00E+00	0.00E+00	1.00E+00
ENSBTAG00000018227	3.72E-02	1.00E+00	2.17E+00	6.43E-14	1.61E+00	2.36E-08	1.04E+00	6.14E-04	-5.54E-01	4.30E-01
ENSBTAG00000030494	0.00E+00	1.00E+00	0.00E+00	1.00E+00	-5.13E-15	1.00E+00	0.00E+00	1.00E+00	0.00E+00	1.00E+00
ENSBTAG00000044393	0.00E+00	1.00E+00	0.00E+00	1.00E+00	-5.13E-15	1.00E+00	0.00E+00	1.00E+00	0.00E+00	1.00E+00
ENSBTAG00000016920	-8.06E-01	2.60E-01	-1.06E+00	1.41E-02	-1.30E+00	1.74E-03	-1.37E+00	1.07E-03	-1.29E+00	3.40E-02

ENSBTAG00000028620	0.00E+00	1.00E+00	0.00E+00	1.00E+00	-5.13E-15	1.00E+00	0.00E+00	1.00E+00	0.00E+00	1.00E+00
ENSBTAG00000035286	2.54E-02	1.00E+00	-2.65E-01	3.91E-01	-6.67E-01	3.85E-03	-6.78E-01	3.88E-03	-3.45E-01	5.98E-01
ENSBTAG00000004117	1.02E-01	1.00E+00	4.36E-01	2.02E-02	3.86E-01	4.51E-02	2.51E-01	2.70E-01	-8.31E-02	1.00E+00
ENSBTAG00000022751	9.04E-02	1.00E+00	7.16E-01	8.34E-03	-6.71E-01	2.09E-02	-8.07E-01	5.09E-03	-3.47E-01	7.43E-01
ENSBTAG00000044062	2.83E-02	1.00E+00	3.54E-01	5.78E-01	-1.50E-01	1.00E+00	1.13E-01	1.00E+00	-3.02E-01	1.00E+00
ENSBTAG00000042299	0.00E+00	1.00E+00	0.00E+00	1.00E+00	-5.13E-15	1.00E+00	0.00E+00	1.00E+00	0.00E+00	1.00E+00
ENSBTAG00000019832	-5.17E-02	1.00E+00	2.86E-01	2.43E-01	-4.00E-03	1.00E+00	2.68E-01	2.99E-01	-1.99E-01	8.31E-01
ENSBTAG00000007131	-9.89E-01	5.90E-01	-8.81E-01	3.19E-01	-1.23E+00	1.15E-01	-4.55E-01	7.20E-01	-1.27E+00	4.34E-01
ENSBTAG00000042079	0.00E+00	1.00E+00	0.00E+00	1.00E+00	-5.13E-15	1.00E+00	0.00E+00	1.00E+00	0.00E+00	1.00E+00
ENSBTAG00000044966	0.00E+00	1.00E+00	0.00E+00	1.00E+00	-5.13E-15	1.00E+00	0.00E+00	1.00E+00	0.00E+00	1.00E+00
ENSBTAG00000007438	3.75E-01	5.47E-01	1.20E+00	3.18E-07	1.33E+00	8.43E-09	1.54E+00	3.86E-11	6.46E-01	9.37E-02
ENSBTAG00000009194	-5.30E-02	1.00E+00	-5.65E-01	1.32E-01	-7.47E-01	3.12E-02	-5.91E-01	1.14E-01	-2.68E-01	9.37E-01
ENSBTAG00000009826	1.16E-01	1.00E+00	3.66E-01	5.33E-01	6.82E-01	1.12E-01	4.86E-01	3.37E-01	4.11E-01	8.00E-01
ENSBTAG00000001710	2.93E-01	1.00E+00	2.85E-01	6.90E-01	-3.94E-01	4.76E-01	-6.65E-01	1.46E-01	-1.45E-01	1.00E+00
ENSBTAG00000001712	-1.50E-01	1.00E+00	-4.73E-02	1.00E+00	-9.62E-03	1.00E+00	-1.86E-01	8.30E-01	5.86E-02	1.00E+00
ENSBTAG00000019526	2.09E-01	7.76E-01	5.64E-01	1.20E-03	4.80E-01	7.02E-03	2.26E-01	3.21E-01	-1.67E-02	1.00E+00
ENSBTAG00000014700	8.62E-02	1.00E+00	6.64E-01	1.20E-03	7.76E-01	1.02E-04	6.53E-01	1.69E-03	-7.23E-01	1.17E-02
ENSBTAG00000025595	8.24E-02	1.00E+00	-2.16E-02	1.00E+00	-4.31E-01	2.25E-01	-4.15E-01	2.62E-01	-4.40E-01	6.01E-01
ENSBTAG00000004834	-1.21E-01	1.00E+00	3.55E-01	1.62E-01	9.31E-01	7.23E-06	5.58E-01	1.41E-02	-3.48E-01	5.58E-01
ENSBTAG00000022744	3.45E-02	1.00E+00	-6.46E-01	7.82E-01	-3.52E-01	1.00E+00	6.61E-01	6.36E-01	-1.12E+00	8.00E-01
ENSBTAG00000034883	6.37E-01	8.23E-01	-7.01E-02	1.00E+00	-1.23E+00	1.22E-01	-1.14E+00	1.60E-01	-7.94E-02	1.00E+00
ENSBTAG00000043125	2.70E+00	5.76E-01	1.99E+00	4.43E-01	-5.13E-15	1.00E+00	0.00E+00	1.00E+00	2.71E+00	6.01E-01
ENSBTAG00000015254	4.46E-03	1.00E+00	-9.16E-01	2.48E-05	-1.57E+00	1.68E-13	-1.04E+00	1.58E-06	2.49E-01	8.00E-01
ENSBTAG00000034871	1.37E-02	1.00E+00	-2.41E-01	5.33E-01	-1.06E-01	9.84E-01	1.28E-01	9.14E-01	-9.62E-02	1.00E+00
ENSBTAG00000021442	-3.42E-01	8.23E-01	-1.05E+00	1.99E-03	-1.08E+00	1.11E-03	-1.02E+00	2.78E-03	2.88E-01	8.98E-01
ENSBTAG00000005327	2.27E+00	1.77E-01	1.53E+00	2.98E-01	1.46E+00	3.10E-01	2.32E+00	3.40E-02	1.67E+00	6.01E-01
ENSBTAG00000003952	-3.49E-01	8.23E-01	-8.61E-01	1.78E-02	-1.26E+00	2.54E-04	-1.02E+00	3.92E-03	6.18E-01	3.63E-01
ENSBTAG00000042124	0.00E+00	1.00E+00	0.00E+00	1.00E+00	-5.13E-15	1.00E+00	0.00E+00	1.00E+00	0.00E+00	1.00E+00
ENSBTAG00000003968	-3.15E-02	1.00E+00	-2.10E-01	5.91E-01	3.69E-01	2.06E-01	1.26E-01	8.97E-01	-3.60E-02	1.00E+00
ENSBTAG00000042705	0.00E+00	1.00E+00	0.00E+00	1.00E+00	-5.13E-15	1.00E+00	0.00E+00	1.00E+00	0.00E+00	1.00E+00
ENSBTAG00000011333	-3.78E-02	1.00E+00	5.38E-01	2.90E-02	-1.78E-01	6.65E-01	2.26E-01	5.09E-01	-6.42E-02	1.00E+00
ENSBTAG00000028471	0.00E+00	1.00E+00	0.00E+00	1.00E+00	-5.13E-15	1.00E+00	0.00E+00	1.00E+00	0.00E+00	1.00E+00
ENSBTAG00000017426	3.01E-01	8.71E-01	1.36E+00	7.26E-06	1.30E+00	1.70E-05	5.78E-01	1.02E-01	4.88E-02	1.00E+00
ENSBTAG00000043185	0.00E+00	1.00E+00	0.00E+00	1.00E+00	-5.13E-15	1.00E+00	0.00E+00	1.00E+00	0.00E+00	1.00E+00
ENSBTAG00000042722	0.00E+00	1.00E+00	0.00E+00	1.00E+00	-5.13E-15	1.00E+00	0.00E+00	1.00E+00	0.00E+00	1.00E+00

ENSBTAG00000007732	2.71E-03	1.00E+00	3.26E-02	1.00E+00	-1.79E+00	7.86E-02	-7.42E-01	5.91E-01	-8.52E-01	8.40E-01
ENSBTAG00000036357	0.00E+00	1.00E+00	0.00E+00	1.00E+00	-5.13E-15	1.00E+00	0.00E+00	1.00E+00	0.00E+00	1.00E+00
ENSBTAG00000042942	1.92E+00	8.23E-01	0.00E+00	1.00E+00	-5.13E-15	1.00E+00	0.00E+00	1.00E+00	0.00E+00	1.00E+00
ENSBTAG00000006735	-1.12E-01	1.00E+00	-1.27E+00	4.61E-02	-2.25E+00	2.60E-04	-1.53E+00	1.33E-02	-4.07E-01	1.00E+00
ENSBTAG00000016760	9.54E-02	1.00E+00	1.82E-01	7.08E-01	-5.98E-01	2.51E-02	-6.50E-01	1.54E-02	1.39E-01	1.00E+00
ENSBTAG00000016758	9.24E-02	1.00E+00	6.40E-02	1.00E+00	-8.35E-01	2.83E-04	-6.85E-01	4.36E-03	-9.29E-02	1.00E+00
ENSBTAG00000022733	-1.83E-01	1.00E+00	-1.90E-01	9.17E-01	-1.26E-01	1.00E+00	-3.47E-01	5.69E-01	5.11E-01	7.00E-01
ENSBTAG00000021452	4.94E-01	8.79E-01	1.57E+00	2.81E-03	1.23E+00	2.43E-02	-3.66E-01	7.18E-01	4.82E-01	8.65E-01
ENSBTAG00000039727	-2.00E+00	1.41E-07	-1.96E+00	3.10E-08	-7.43E-01	3.34E-02	-1.00E+00	3.46E-03	-6.83E-01	3.09E-01
ENSBTAG00000016563	2.82E-01	7.86E-01	1.47E+00	1.09E-10	1.95E+00	9.66E-18	1.59E+00	3.70E-12	-1.39E-01	1.00E+00
ENSBTAG00000016566	1.38E+00	2.64E-07	2.67E+00	2.08E-26	2.54E+00	2.91E-24	1.80E+00	3.87E-13	7.14E-01	7.31E-02
ENSBTAG00000047965	0.00E+00	1.00E+00	0.00E+00	1.00E+00	-5.13E-15	1.00E+00	0.00E+00	1.00E+00	0.00E+00	1.00E+00
ENSBTAG00000013402	-9.98E-02	1.00E+00	-1.42E+00	4.10E-06	-1.56E+00	3.43E-07	-1.19E+00	1.43E-04	2.09E-01	1.00E+00
ENSBTAG00000044316	0.00E+00	1.00E+00	0.00E+00	1.00E+00	-5.13E-15	1.00E+00	0.00E+00	1.00E+00	0.00E+00	1.00E+00
ENSBTAG00000029937	0.00E+00	1.00E+00	0.00E+00	1.00E+00	-5.13E-15	1.00E+00	0.00E+00	1.00E+00	0.00E+00	1.00E+00
ENSBTAG00000012963	-4.18E-01	1.00E+00	-9.00E-01	2.14E-01	-1.21E+00	6.94E-02	-1.69E+00	8.97E-03	5.80E-01	8.17E-01
ENSBTAG00000037726	-3.60E-01	9.32E-01	-2.32E+00	1.67E-08	-2.62E-01	7.64E-01	-2.37E-01	8.28E-01	-2.96E-01	1.00E+00
ENSBTAG00000008567	-5.40E-01	6.15E-01	-7.86E-01	6.21E-02	-2.79E+00	7.69E-13	-2.51E+00	1.06E-10	6.06E-01	5.50E-01
ENSBTAG00000018283	-2.20E-01	9.32E-01	-9.65E-01	6.57E-05	-2.31E+00	2.03E-22	-2.88E+00	1.29E-32	-3.04E-02	1.00E+00
ENSBTAG00000000563	5.48E-01	2.24E-01	2.03E+00	7.86E-16	1.96E+00	6.74E-15	1.43E+00	1.74E-08	1.30E-01	1.00E+00
ENSBTAG00000014927	1.97E-01	1.00E+00	7.55E-01	8.48E-03	1.07E+00	7.87E-05	1.22E+00	6.73E-06	3.29E-01	7.94E-01
ENSBTAG00000039310	2.31E-01	1.00E+00	1.64E+00	4.51E-02	1.20E+00	1.97E-01	2.11E-01	1.00E+00	2.21E+00	2.56E-02
ENSBTAG00000008463	-5.70E-01	1.00E+00	1.26E+00	1.89E-01	9.40E-01	3.91E-01	-7.37E-01	6.10E-01	1.53E+00	3.84E-01
ENSBTAG00000011278	-2.63E-01	7.80E-01	-1.28E+00	1.99E-09	-1.95E+00	3.96E-20	-1.55E+00	2.64E-13	3.61E-01	5.13E-01
ENSBTAG00000018105	4.55E-02	1.00E+00	-4.20E-01	4.65E-01	-6.13E-01	2.02E-01	-1.03E-01	1.00E+00	6.55E-01	4.73E-01
ENSBTAG00000019305	-1.97E-01	1.00E+00	3.33E-01	6.34E-01	1.36E+00	4.60E-04	1.59E+00	3.89E-05	2.92E-01	1.00E+00
ENSBTAG00000009155	-1.52E-01	1.00E+00	1.03E-01	1.00E+00	4.44E-02	1.00E+00	2.64E-01	9.40E-01	-1.31E+00	4.34E-01
ENSBTAG00000042314	0.00E+00	1.00E+00	0.00E+00	1.00E+00	-5.13E-15	1.00E+00	0.00E+00	1.00E+00	0.00E+00	1.00E+00
ENSBTAG00000046911	-1.12E+00	8.23E-01	-1.04E+00	4.82E-01	4.54E-01	8.39E-01	-4.02E-01	9.56E-01	-1.12E+00	8.00E-01
ENSBTAG00000017174	-6.86E-02	1.00E+00	-1.25E+00	9.68E-02	-2.40E+00	6.24E-04	-3.40E+00	5.26E-06	-1.39E-01	1.00E+00
ENSBTAG00000003745	-5.86E-02	1.00E+00	-2.16E-01	4.43E-01	-2.48E-01	3.47E-01	-1.48E-01	7.00E-01	1.59E-01	9.68E-01
ENSBTAG00000047135	-3.01E-01	6.88E-01	-6.53E-01	5.46E-03	-3.86E-01	1.42E-01	-4.01E-01	1.32E-01	4.47E-02	1.00E+00
ENSBTAG00000010477	-6.31E-01	1.94E-01	-6.18E-01	5.19E-02	-4.15E-01	2.40E-01	-1.09E+00	2.21E-04	-5.36E-02	1.00E+00
ENSBTAG00000003845	2.32E+00	2.86E-09	3.34E+00	5.27E-19	2.34E+00	1.67E-10	1.76E+00	2.11E-06	7.77E-01	3.09E-01
ENSBTAG00000046512	3.05E+00	4.07E-02	5.71E+00	2.96E-07	4.28E+00	8.81E-05	5.51E+00	8.40E-07	2.07E+00	4.10E-01

ENSBTAG00000002923	7.41E-01	2.59E-01	-6.23E-03	1.00E+00	-4.29E-01	4.09E-01	-1.01E+00	1.35E-02	-8.76E-02	1.00E+00
ENSBTAG00000015483	-3.50E-01	1.00E+00	-6.62E-01	4.23E-01	-1.11E+00	1.01E-01	-2.00E+00	3.62E-03	-9.10E-01	6.01E-01
ENSBTAG00000015493	3.67E+00	4.28E-03	3.29E+00	4.66E-03	3.71E+00	3.65E-04	4.92E+00	1.87E-08	1.31E+00	9.00E-01
ENSBTAG00000022721	2.28E-01	8.25E-01	-1.68E-01	6.71E-01	-2.66E-01	3.63E-01	1.14E-02	1.00E+00	1.37E-01	1.00E+00
ENSBTAG00000009760	2.82E+00	1.38E-15	7.62E-01	4.35E-02	-6.94E-02	1.00E+00	6.32E-01	1.19E-01	1.22E+00	9.58E-03
ENSBTAG00000009757	-2.65E-02	1.00E+00	1.64E-01	7.11E-01	2.15E-01	5.17E-01	3.32E-01	2.43E-01	7.30E-01	2.28E-02
ENSBTAG00000042226	0.00E+00	1.00E+00	0.00E+00	1.00E+00	-5.13E-15	1.00E+00	0.00E+00	1.00E+00	0.00E+00	1.00E+00
ENSBTAG00000042875	0.00E+00	1.00E+00	0.00E+00	1.00E+00	-5.13E-15	1.00E+00	0.00E+00	1.00E+00	0.00E+00	1.00E+00
ENSBTAG00000009774	2.77E+00	1.42E-01	8.81E-01	8.52E-01	1.52E+00	4.28E-01	1.49E+00	4.55E-01	8.12E-01	1.00E+00
ENSBTAG00000009430	-4.39E-02	1.00E+00	2.30E-01	8.41E-01	3.02E-01	6.64E-01	2.79E-01	7.28E-01	-8.40E-02	1.00E+00
ENSBTAG00000019106	1.87E-01	1.00E+00	7.88E-01	8.44E-04	9.35E-01	5.14E-05	2.34E-01	4.97E-01	-8.93E-02	1.00E+00
ENSBTAG00000011702	-1.55E-01	1.00E+00	-6.64E-01	1.72E-01	-1.21E+00	4.83E-03	-9.61E-01	3.12E-02	-5.93E-01	6.07E-01
ENSBTAG00000002038	4.58E-03	1.00E+00	3.15E-01	2.42E-01	3.64E-01	1.55E-01	5.14E-01	3.07E-02	-4.49E-01	3.13E-01
ENSBTAG00000018057	-2.70E-01	8.23E-01	5.78E-01	2.06E-02	2.93E-01	3.46E-01	1.72E-01	7.12E-01	1.47E-01	1.00E+00
ENSBTAG00000044586	0.00E+00	1.00E+00	0.00E+00	1.00E+00	-5.13E-15	1.00E+00	0.00E+00	1.00E+00	0.00E+00	1.00E+00
ENSBTAG00000047441	3.93E-01	6.09E-01	2.29E+00	2.42E-17	1.99E+00	9.63E-14	1.75E+00	8.58E-11	8.21E-02	1.00E+00
ENSBTAG00000043427	0.00E+00	1.00E+00	0.00E+00	1.00E+00	-5.13E-15	1.00E+00	0.00E+00	1.00E+00	0.00E+00	1.00E+00
ENSBTAG00000016420	-1.88E-01	9.36E-01	-6.81E-01	1.45E-03	-4.36E-01	6.02E-02	-6.57E-02	1.00E+00	-1.63E-01	9.85E-01
ENSBTAG00000043190	-1.91E+00	8.23E-01	-1.91E+00	4.69E-01	-1.91E+00	4.28E-01	-1.91E+00	4.39E-01	-1.91E+00	8.00E-01
ENSBTAG00000008416	-2.09E-01	9.37E-01	3.36E-01	2.37E-01	8.55E-01	1.24E-04	8.96E-01	6.55E-05	-2.67E-01	8.00E-01
ENSBTAG00000029814	0.00E+00	1.00E+00	0.00E+00	1.00E+00	-5.13E-15	1.00E+00	0.00E+00	1.00E+00	0.00E+00	1.00E+00
ENSBTAG00000017102	-1.48E-01	1.00E+00	-8.43E-01	1.70E-03	-1.15E+00	1.01E-05	-7.91E-01	3.71E-03	-5.44E-02	1.00E+00
ENSBTAG00000025379	1.88E-01	9.86E-01	1.25E+00	8.66E-09	1.50E+00	2.15E-12	1.81E+00	2.63E-17	3.26E-01	6.26E-01
ENSBTAG00000016076	-2.06E-01	1.00E+00	-1.29E+00	1.33E-06	-1.20E+00	6.38E-06	-7.38E-01	9.44E-03	4.52E-01	4.91E-01
ENSBTAG00000047883	6.91E-02	1.00E+00	-1.70E+00	1.52E-14	-1.41E+00	1.31E-10	-1.77E+00	1.09E-15	2.53E-01	8.00E-01
ENSBTAG00000013027	-2.70E+00	5.81E-01	-2.70E+00	2.46E-01	1.25E+00	4.51E-01	2.64E+00	1.34E-02	-7.84E-01	1.00E+00
ENSBTAG00000018601	-3.59E+00	2.32E-01	-1.60E+00	4.08E-01	-1.54E-01	1.00E+00	-1.83E+00	2.99E-01	-8.80E-01	1.00E+00
ENSBTAG00000006567	-3.34E-01	8.16E-01	-1.93E+00	1.00E-11	-9.05E-01	2.03E-03	-5.84E-01	7.40E-02	-2.51E-02	1.00E+00
ENSBTAG00000006570	-9.43E-02	1.00E+00	-2.00E+00	7.16E-09	-8.66E-01	9.10E-03	2.47E-01	6.37E-01	8.51E-01	6.37E-02
ENSBTAG00000002795	1.89E-01	1.00E+00	9.86E-01	3.93E-04	-2.97E-03	1.00E+00	-9.69E-02	1.00E+00	-3.12E-01	8.00E-01
ENSBTAG00000017365	-4.41E-01	7.43E-01	3.54E-01	4.65E-01	-1.25E+00	4.99E-04	-7.38E-01	6.02E-02	8.93E-02	1.00E+00
ENSBTAG00000006563	1.63E+00	6.21E-01	3.41E+00	2.24E-03	2.12E+00	9.38E-02	2.99E+00	9.68E-03	1.27E+00	8.00E-01
ENSBTAG00000033422	-2.61E-03	1.00E+00	1.20E+00	3.36E-01	-1.57E-01	1.00E+00	1.42E+00	1.95E-01	-1.67E+00	7.90E-01
ENSBTAG00000013015	6.53E-01	5.60E-01	-1.28E+00	2.24E-02	-5.09E+00	3.80E-10	-1.99E+00	3.73E-04	9.95E-01	1.75E-01
ENSBTAG00000038640	6.06E-01	2.87E-01	1.87E+00	4.52E-10	1.36E+00	6.64E-06	1.08E+00	5.34E-04	-1.67E-01	1.00E+00

ENSBTAG00000014162	-2.79E-01	1.00E+00	3.49E-01	7.39E-01	4.68E-01	5.29E-01	-3.78E-01	7.00E-01	9.28E-01	4.23E-01
ENSBTAG00000034106	-5.57E-01	7.53E-02	-1.54E+00	1.60E-13	-3.19E+00	3.64E-49	-4.23E+00	1.44E-77	-3.01E-02	1.00E+00
ENSBTAG00000000459	-2.31E-01	9.66E-01	9.78E-01	2.87E-04	1.04E+00	1.00E-04	6.29E-01	3.32E-02	1.66E-01	1.00E+00
ENSBTAG00000008244	-2.70E+00	5.76E-01	-2.70E+00	2.42E-01	-2.70E+00	1.99E-01	-2.70E+00	2.05E-01	-2.70E+00	6.01E-01
ENSBTAG00000003325	3.84E-01	5.83E-01	3.14E-01	3.80E-01	1.49E-01	8.43E-01	3.55E-01	2.97E-01	2.91E-01	8.00E-01
ENSBTAG00000044543	0.00E+00	1.00E+00	0.00E+00	1.00E+00	-5.13E-15	1.00E+00	0.00E+00	1.00E+00	0.00E+00	1.00E+00
ENSBTAG00000008893	4.93E-01	2.31E-01	3.15E-01	3.02E-01	6.18E-01	1.24E-02	4.58E-01	9.18E-02	1.37E-02	1.00E+00
ENSBTAG00000044167	2.10E+00	4.83E-01	2.55E+00	7.76E-02	1.44E+00	4.19E-01	2.36E+00	1.15E-01	9.63E-01	9.98E-01
ENSBTAG00000016622	2.64E-02	1.00E+00	-5.35E-01	1.75E-02	-1.32E-01	7.93E-01	3.69E-01	1.38E-01	2.49E-01	7.98E-01
ENSBTAG00000030533	1.12E-02	1.00E+00	7.51E-02	1.00E+00	1.14E+00	6.46E-01	1.78E+00	3.04E-01	-1.91E+00	8.00E-01
ENSBTAG00000046301	9.99E-02	1.00E+00	1.89E-01	8.23E-01	3.07E-01	4.92E-01	2.63E-01	6.15E-01	4.98E-01	5.57E-01
ENSBTAG00000003622	-2.72E-01	8.29E-01	-8.70E-01	1.42E-03	-3.54E-01	2.84E-01	-3.34E-01	3.36E-01	-2.25E-01	9.22E-01
ENSBTAG00000037803	-9.90E-02	1.00E+00	2.77E-01	2.53E-01	7.78E-02	9.97E-01	-5.55E-02	1.00E+00	-1.63E-01	9.39E-01
ENSBTAG00000034005	-9.89E-02	1.00E+00	1.54E+00	5.66E-09	1.03E+00	1.56E-04	7.29E-01	1.30E-02	-1.37E-01	1.00E+00
ENSBTAG00000007020	-7.89E-02	1.00E+00	1.47E-01	9.31E-01	4.38E-01	2.36E-01	-2.77E-01	5.68E-01	1.32E-01	1.00E+00
ENSBTAG00000046036	0.00E+00	1.00E+00	2.79E+00	2.10E-01	-5.13E-15	1.00E+00	0.00E+00	1.00E+00	2.72E+00	6.01E-01
ENSBTAG00000016725	-1.06E-01	1.00E+00	-8.60E-02	1.00E+00	2.51E-01	4.28E-01	7.58E-02	1.00E+00	-2.31E-01	8.26E-01
ENSBTAG00000016726	-2.64E-01	1.00E+00	-1.05E+00	8.27E-02	-1.21E+00	3.43E-02	-9.77E-01	1.09E-01	-1.06E+00	3.52E-01
ENSBTAG00000047611	1.75E-01	1.00E+00	7.86E-01	2.05E-02	1.35E+00	5.76E-06	1.44E+00	1.34E-06	-2.26E-01	1.00E+00
ENSBTAG00000019479	2.91E-01	8.23E-01	5.54E-01	5.61E-02	7.18E-01	7.97E-03	7.76E-01	4.33E-03	1.25E-01	1.00E+00
ENSBTAG00000006193	1.68E+00	3.33E-04	3.27E+00	2.77E-15	9.64E-01	2.91E-02	5.49E-01	3.09E-01	-1.00E-01	1.00E+00
ENSBTAG00000016101	-4.19E-01	5.76E-01	-9.58E-01	1.17E-03	5.10E-01	9.33E-02	9.82E-01	2.47E-04	2.86E-01	8.00E-01
ENSBTAG00000016098	-4.15E-02	1.00E+00	1.82E-01	5.17E-01	7.40E-02	1.00E+00	1.42E-02	1.00E+00	-1.73E-01	8.97E-01
ENSBTAG00000010094	1.92E+00	8.23E-01	0.00E+00	1.00E+00	-5.13E-15	1.00E+00	0.00E+00	1.00E+00	1.91E+00	8.00E-01
ENSBTAG00000010077	-4.75E-01	6.32E-01	-1.90E+00	1.11E-07	-1.47E+00	2.39E-05	-5.73E-01	1.55E-01	2.02E-01	1.00E+00
ENSBTAG00000003882	-1.84E-01	1.00E+00	-4.74E-01	8.58E-02	-6.03E-01	2.05E-02	-4.07E-01	1.68E-01	-9.21E-02	1.00E+00
ENSBTAG00000010836	1.19E+00	8.62E-01	1.26E+00	4.56E-01	3.41E-01	1.00E+00	-1.85E-01	1.00E+00	1.18E+00	8.38E-01
ENSBTAG00000010835	6.64E-01	1.11E-01	2.54E+00	2.80E-21	1.60E+00	1.43E-09	4.08E-01	2.32E-01	7.94E-01	5.80E-02
ENSBTAG00000033669	-6.91E-01	5.18E-02	-1.62E+00	6.40E-11	-2.03E+00	2.31E-16	-1.49E+00	2.33E-09	-3.13E-01	7.71E-01
ENSBTAG00000010833	-5.56E-01	9.17E-01	-6.59E-01	4.43E-01	-3.87E-01	7.60E-01	-1.94E+00	4.80E-03	3.93E-01	1.00E+00
ENSBTAG00000010832	-4.97E-02	1.00E+00	1.34E-01	8.37E-01	1.87E-01	6.23E-01	1.64E-01	7.22E-01	3.58E-02	1.00E+00
ENSBTAG00000007969	-6.97E-01	1.00E+00	-2.33E+00	9.76E-02	-4.36E+00	2.15E-03	-3.38E+00	1.52E-02	-5.78E-01	1.00E+00
ENSBTAG00000007968	-7.90E-02	1.00E+00	-2.88E-01	2.43E-01	-1.63E-01	6.27E-01	-3.49E-01	1.40E-01	-1.83E-02	1.00E+00
ENSBTAG00000007966	-3.64E-01	4.55E-01	-9.48E-01	1.04E-05	-1.45E+00	6.90E-12	-1.70E+00	1.01E-15	3.78E-01	4.58E-01
ENSBTAG00000007964	-3.06E-02	1.00E+00	2.91E-02	1.00E+00	3.58E-01	1.37E-01	6.07E-01	5.04E-03	-2.56E-01	7.72E-01

ENSBTAG00000007961	-6.84E-02	1.00E+00	-2.32E-01	4.54E-01	-5.74E-01	1.21E-02	-8.71E-01	7.53E-05	6.59E-05	1.00E+00
ENSBTAG00000007780	1.65E-01	1.00E+00	-7.34E-01	8.97E-03	-6.11E-01	3.09E-02	-5.58E-01	5.80E-02	-4.21E-01	5.58E-01
ENSBTAG00000007777	-1.25E-01	1.00E+00	2.54E-02	1.00E+00	1.15E-01	9.55E-01	1.93E-01	6.87E-01	4.23E-01	5.13E-01
ENSBTAG00000007776	-8.09E-02	1.00E+00	4.34E-01	1.15E-01	8.04E-01	8.23E-04	5.38E-01	4.22E-02	-7.60E-02	1.00E+00
ENSBTAG00000011551	2.35E-01	1.00E+00	3.28E+00	1.84E-11	1.61E+00	1.23E-03	-4.28E-01	6.18E-01	6.75E-01	6.82E-01
ENSBTAG00000009765	6.15E-02	1.00E+00	1.06E+00	2.04E-05	7.95E-01	1.79E-03	3.78E-01	2.28E-01	2.57E-01	8.17E-01
ENSBTAG00000042999	2.70E+00	5.76E-01	0.00E+00	1.00E+00	-5.13E-15	1.00E+00	0.00E+00	1.00E+00	0.00E+00	1.00E+00
ENSBTAG00000010916	-1.23E-01	1.00E+00	-7.94E-01	1.67E-02	-8.49E-01	8.95E-03	-4.55E-01	2.44E-01	3.16E-02	1.00E+00
ENSBTAG00000019193	2.45E-02	1.00E+00	5.86E-01	9.09E-03	1.11E-01	8.92E-01	-1.28E-01	8.32E-01	-7.25E-02	1.00E+00
ENSBTAG00000003112	-1.42E-01	1.00E+00	2.98E-01	2.71E-01	-1.29E-01	8.14E-01	-2.43E-01	4.37E-01	2.50E-01	8.00E-01
ENSBTAG00000004685	3.24E-01	1.00E+00	-6.37E-01	3.27E-01	-4.98E-01	4.70E-01	-2.43E-01	8.99E-01	2.89E-01	1.00E+00
ENSBTAG00000012142	1.07E-01	1.00E+00	4.11E-01	2.16E-01	6.26E-01	2.95E-02	-1.22E-03	1.00E+00	-1.82E-01	1.00E+00
ENSBTAG00000019772	-7.93E-01	1.00E+00	5.97E-01	9.45E-01	-9.24E-01	8.08E-01	-2.71E+00	2.05E-01	-7.92E-01	1.00E+00
ENSBTAG00000022699	-3.22E+00	3.75E-01	4.60E-01	1.00E+00	-1.44E+00	4.79E-01	7.16E-01	7.59E-01	-5.13E-01	1.00E+00
ENSBTAG00000005433	-5.36E-01	1.00E+00	-1.00E+00	4.43E-01	-2.12E+00	6.54E-02	-9.87E-01	4.39E-01	-3.25E-01	1.00E+00
ENSBTAG00000005431	-5.38E-01	8.45E-01	-1.64E+00	4.27E-03	-1.51E+00	7.62E-03	-4.20E-01	6.47E-01	-9.58E-01	4.44E-01
ENSBTAG00000013047	1.07E-02	1.00E+00	2.98E-01	9.24E-01	-7.14E-01	4.15E-01	1.14E+00	9.75E-02	-6.34E-01	8.30E-01
ENSBTAG00000042993	0.00E+00	1.00E+00	0.00E+00	1.00E+00	-5.13E-15	1.00E+00	0.00E+00	1.00E+00	0.00E+00	1.00E+00
ENSBTAG00000042811	0.00E+00	1.00E+00	0.00E+00	1.00E+00	-5.13E-15	1.00E+00	0.00E+00	1.00E+00	0.00E+00	1.00E+00
ENSBTAG00000033275	0.00E+00	1.00E+00	0.00E+00	1.00E+00	-5.13E-15	1.00E+00	0.00E+00	1.00E+00	0.00E+00	1.00E+00
ENSBTAG00000012904	4.20E-01	6.54E-01	1.94E+00	8.25E-11	1.73E+00	5.75E-09	1.39E+00	5.06E-06	6.15E-01	3.35E-01
ENSBTAG00000012902	2.74E-01	8.15E-01	1.00E+00	1.42E-05	1.04E+00	6.10E-06	1.14E+00	7.83E-07	1.40E-01	1.00E+00
ENSBTAG00000009863	2.11E+00	4.62E-08	2.50E+00	4.70E-12	1.48E+00	4.20E-05	1.12E+00	3.02E-03	2.36E-01	1.00E+00
ENSBTAG00000020455	2.80E-01	8.23E-01	1.14E+00	5.63E-06	2.38E-01	5.36E-01	-7.41E-01	6.40E-03	4.51E-01	4.59E-01
ENSBTAG00000042403	0.00E+00	1.00E+00	0.00E+00	1.00E+00	-5.13E-15	1.00E+00	0.00E+00	1.00E+00	0.00E+00	1.00E+00
ENSBTAG00000039855	-1.95E-01	8.57E-01	-9.10E-01	1.88E-06	-1.70E+00	1.40E-19	-1.72E+00	7.56E-20	2.72E-01	6.45E-01
ENSBTAG00000034496	-3.28E-02	1.00E+00	-1.69E-02	1.00E+00	-3.06E-01	4.44E-01	-4.86E-01	1.53E-01	-3.22E-01	8.00E-01
ENSBTAG00000018935	-6.05E-01	4.01E-01	-1.24E+00	4.96E-04	-9.12E-01	1.16E-02	-7.98E-01	3.42E-02	-8.24E-02	1.00E+00
ENSBTAG00000001176	5.35E-03	1.00E+00	7.57E-02	1.00E+00	-1.91E+00	4.28E-01	-1.91E+00	4.39E-01	1.32E+00	8.97E-01
ENSBTAG00000019536	3.19E-01	6.06E-01	1.71E-01	6.70E-01	6.31E-02	1.00E+00	1.19E-01	8.83E-01	1.67E-01	9.99E-01
ENSBTAG00000019534	4.28E-01	2.27E-01	9.75E-01	9.30E-07	1.31E+00	1.58E-11	8.76E-01	1.46E-05	1.01E-01	1.00E+00
ENSBTAG00000000273	3.93E-01	1.00E+00	4.74E-01	9.88E-01	-6.55E-01	8.96E-01	1.94E-01	1.00E+00	3.91E-01	1.00E+00
ENSBTAG00000033225	6.87E-01	7.72E-01	5.74E-01	4.82E-01	-6.97E-01	4.17E-01	5.66E-01	4.97E-01	1.36E-03	1.00E+00
ENSBTAG00000003592	-4.48E-01	1.00E+00	-2.47E-01	1.00E+00	-1.14E+00	1.80E-01	-6.60E-02	1.00E+00	-9.71E-01	6.82E-01
ENSBTAG00000045059	0.00E+00	1.00E+00	0.00E+00	1.00E+00	-5.13E-15	1.00E+00	0.00E+00	1.00E+00	0.00E+00	1.00E+00

ENSBTAG00000006727	-2.70E+00	5.76E-01	-2.70E+00	2.42E-01	-2.70E+00	1.99E-01	-2.70E+00	2.05E-01	-7.74E-01	1.00E+00
ENSBTAG00000002471	-1.08E-01	1.00E+00	-1.88E+00	1.58E-09	-3.36E+00	8.68E-25	-3.58E+00	3.72E-27	-3.60E-01	8.00E-01
ENSBTAG00000044734	0.00E+00	1.00E+00	0.00E+00	1.00E+00	-5.13E-15	1.00E+00	2.53E+00	2.71E-01	0.00E+00	1.00E+00
ENSBTAG00000007615	-1.63E-02	1.00E+00	-6.22E-02	1.00E+00	-4.85E-01	3.37E-01	-1.76E-01	9.45E-01	1.81E-02	1.00E+00
ENSBTAG00000018644	1.15E-02	1.00E+00	-2.67E-02	1.00E+00	-1.63E-01	9.78E-01	1.71E-01	9.52E-01	-1.66E-01	1.00E+00
ENSBTAG00000002147	1.66E-01	1.00E+00	1.07E+00	9.08E-05	7.25E-01	1.21E-02	8.68E-01	2.39E-03	1.77E-01	1.00E+00
ENSBTAG00000020481	3.78E-03	1.00E+00	5.44E-01	2.43E-01	1.06E-01	1.00E+00	3.06E-03	1.00E+00	2.61E-01	1.00E+00
ENSBTAG00000005436	2.22E-01	8.23E-01	1.42E+00	2.17E-13	1.59E+00	7.93E-17	9.16E-01	4.67E-06	-9.48E-02	1.00E+00
ENSBTAG00000022689	4.12E-01	6.09E-01	1.01E+00	4.61E-04	5.84E-01	6.52E-02	2.82E-01	5.08E-01	6.38E-01	2.46E-01
ENSBTAG00000048243	0.00E+00	1.00E+00	0.00E+00	1.00E+00	-5.13E-15	1.00E+00	0.00E+00	1.00E+00	0.00E+00	1.00E+00
ENSBTAG00000018862	4.44E-01	7.69E-01	-9.69E-02	1.00E+00	9.73E-01	6.69E-03	1.52E+00	6.50E-06	3.32E-01	8.97E-01
ENSBTAG00000019330	0.00E+00	1.00E+00	0.00E+00	1.00E+00	-5.13E-15	1.00E+00	0.00E+00	1.00E+00	0.00E+00	1.00E+00
ENSBTAG00000038546	0.00E+00	1.00E+00	0.00E+00	1.00E+00	-5.13E-15	1.00E+00	0.00E+00	1.00E+00	0.00E+00	1.00E+00
ENSBTAG00000016533	1.11E-01	1.00E+00	-8.47E-01	5.25E-04	-1.44E+00	1.04E-09	-7.88E-01	1.54E-03	3.50E-01	6.06E-01
ENSBTAG00000036616	0.00E+00	1.00E+00	0.00E+00	1.00E+00	-5.13E-15	1.00E+00	0.00E+00	1.00E+00	0.00E+00	1.00E+00
ENSBTAG00000042996	0.00E+00	1.00E+00	0.00E+00	1.00E+00	-5.13E-15	1.00E+00	0.00E+00	1.00E+00	0.00E+00	1.00E+00
ENSBTAG00000048050	-1.91E+00	8.23E-01	1.39E+00	5.17E-01	-1.91E+00	4.28E-01	1.12E+00	6.71E-01	-1.91E+00	8.00E-01
ENSBTAG00000009220	0.00E+00	1.00E+00	0.00E+00	1.00E+00	-5.13E-15	1.00E+00	0.00E+00	1.00E+00	0.00E+00	1.00E+00
ENSBTAG00000006679	1.35E-01	1.00E+00	-2.55E-01	6.95E-01	3.50E-01	4.70E-01	5.23E-01	2.06E-01	-5.91E-03	1.00E+00
ENSBTAG00000013059	7.64E-01	4.26E-01	9.04E-01	6.34E-02	9.27E-01	4.77E-02	9.51E-01	4.48E-02	2.03E-01	1.00E+00
ENSBTAG00000007624	-7.88E-01	2.75E-01	1.43E+00	3.19E-04	1.80E-02	1.00E+00	-5.33E-02	1.00E+00	-2.78E-01	1.00E+00
ENSBTAG00000021508	-7.23E-01	1.00E+00	1.07E+00	2.95E-01	1.17E-02	1.00E+00	-1.39E-02	1.00E+00	6.35E-02	1.00E+00
ENSBTAG00000021506	4.51E-02	1.00E+00	3.78E-02	1.00E+00	1.11E-01	8.07E-01	1.39E-01	6.79E-01	-1.19E-01	1.00E+00
ENSBTAG00000015556	-7.56E-02	1.00E+00	-5.08E-01	4.13E-02	-6.40E-02	1.00E+00	2.66E-01	4.06E-01	-3.60E-01	5.71E-01
ENSBTAG00000015554	4.49E-01	4.38E-01	1.75E+00	4.77E-12	1.75E+00	4.47E-12	1.56E+00	9.11E-10	7.95E-02	1.00E+00
ENSBTAG00000022681	-1.34E-01	1.00E+00	1.29E+00	1.17E-02	-3.24E-01	7.58E-01	-1.24E-01	1.00E+00	-8.00E-02	1.00E+00
ENSBTAG00000003110	-8.02E-01	7.48E-01	1.03E+00	1.15E-01	-1.11E+00	1.37E-01	-4.81E-01	6.59E-01	-2.11E-01	1.00E+00
ENSBTAG00000019041	-7.36E-02	1.00E+00	7.13E-01	4.19E-01	-2.55E-01	1.00E+00	1.20E+00	8.50E-02	-3.45E-01	1.00E+00
ENSBTAG00000042847	0.00E+00	1.00E+00	0.00E+00	1.00E+00	-5.13E-15	1.00E+00	0.00E+00	1.00E+00	0.00E+00	1.00E+00
ENSBTAG00000009541	4.54E-02	1.00E+00	-4.61E-02	1.00E+00	-4.28E-01	8.20E-02	-6.55E-01	4.19E-03	7.51E-02	1.00E+00
ENSBTAG00000011081	1.69E-01	1.00E+00	1.88E+00	1.14E-09	1.57E+00	4.75E-07	1.51E+00	1.93E-06	-6.74E-03	1.00E+00
ENSBTAG00000042763	0.00E+00	1.00E+00	0.00E+00	1.00E+00	-5.13E-15	1.00E+00	0.00E+00	1.00E+00	0.00E+00	1.00E+00
ENSBTAG00000045749	0.00E+00	1.00E+00	0.00E+00	1.00E+00	-5.13E-15	1.00E+00	0.00E+00	1.00E+00	0.00E+00	1.00E+00
ENSBTAG00000044541	0.00E+00	1.00E+00	0.00E+00	1.00E+00	-5.13E-15	1.00E+00	0.00E+00	1.00E+00	0.00E+00	1.00E+00
ENSBTAG00000047851	0.00E+00	1.00E+00	0.00E+00	1.00E+00	-5.13E-15	1.00E+00	0.00E+00	1.00E+00	0.00E+00	1.00E+00

ENSBTAG00000010360	1.15E-01	1.00E+00	-7.89E-01	6.76E-03	-1.23E+00	8.32E-06	-8.91E-01	2.15E-03	3.02E-01	8.00E-01
ENSBTAG00000046330	0.00E+00	1.00E+00	0.00E+00	1.00E+00	-5.13E-15	1.00E+00	0.00E+00	1.00E+00	0.00E+00	1.00E+00
ENSBTAG00000037972	-4.32E-03	1.00E+00	9.52E-01	9.44E-05	7.66E-01	2.14E-03	3.22E-01	3.22E-01	5.67E-02	1.00E+00
ENSBTAG00000032954	-7.22E-02	1.00E+00	-3.92E-01	2.53E-01	-8.28E-01	3.41E-03	-8.08E-01	5.21E-03	-1.41E-01	1.00E+00
ENSBTAG00000044251	0.00E+00	1.00E+00	0.00E+00	1.00E+00	-5.13E-15	1.00E+00	0.00E+00	1.00E+00	0.00E+00	1.00E+00
ENSBTAG00000030082	0.00E+00	1.00E+00	0.00E+00	1.00E+00	-5.13E-15	1.00E+00	0.00E+00	1.00E+00	1.91E+00	8.00E-01
ENSBTAG00000010581	1.54E-01	1.00E+00	4.26E-01	4.97E-01	-8.82E-01	6.76E-02	-1.65E+00	2.16E-04	6.23E-01	6.21E-01
ENSBTAG00000008534	1.91E+00	8.23E-01	0.00E+00	1.00E+00	-5.13E-15	1.00E+00	0.00E+00	1.00E+00	0.00E+00	1.00E+00
ENSBTAG00000019048	1.81E+00	2.65E-02	1.22E+00	1.09E-01	2.22E+00	1.05E-04	2.34E+00	3.89E-05	1.09E+00	5.50E-01
ENSBTAG00000003165	6.94E-01	3.02E-01	2.43E+00	2.20E-12	1.72E+00	6.95E-07	1.85E+00	1.08E-07	-2.64E-01	1.00E+00
ENSBTAG00000021835	2.92E+00	3.25E-08	6.07E+00	1.22E-29	6.00E+00	2.30E-29	3.94E+00	3.96E-15	1.31E+00	1.24E-01
ENSBTAG00000015112	2.87E-01	6.18E-01	1.01E+00	2.38E-07	9.21E-01	2.87E-06	3.98E-01	8.40E-02	-1.09E-01	1.00E+00
ENSBTAG00000011287	1.02E-01	1.00E+00	5.24E-01	6.51E-02	1.73E-01	7.50E-01	9.60E-02	1.00E+00	2.00E-01	9.80E-01
ENSBTAG00000032872	1.96E-01	1.00E+00	-1.51E-01	9.20E-01	-1.10E-01	1.00E+00	-5.01E-01	1.77E-01	-5.64E-01	4.34E-01
ENSBTAG00000039133	5.11E-01	1.00E+00	5.98E-01	9.45E-01	7.22E-01	8.25E-01	-1.73E-01	1.00E+00	5.08E-04	1.00E+00
ENSBTAG00000013026	1.10E-02	1.00E+00	-7.23E-01	9.45E-01	3.49E-01	1.00E+00	6.99E-01	8.52E-01	7.75E-04	1.00E+00
ENSBTAG00000000265	-1.51E+00	5.46E-02	-1.32E+00	2.45E-02	-1.09E+00	5.37E-02	-4.99E-01	4.81E-01	-1.21E+00	2.34E-01
ENSBTAG00000045546	0.00E+00	1.00E+00	0.00E+00	1.00E+00	-5.13E-15	1.00E+00	0.00E+00	1.00E+00	1.91E+00	8.00E-01
ENSBTAG00000005321	3.95E-01	1.00E+00	8.22E-02	1.00E+00	-3.11E-01	8.87E-01	2.82E-01	9.12E-01	-8.45E-01	7.14E-01
ENSBTAG00000020302	2.72E+00	5.76E-01	1.99E+00	4.43E-01	2.56E+00	2.54E-01	1.76E+00	4.97E-01	3.61E+00	3.07E-01
ENSBTAG00000001889	6.33E-02	1.00E+00	-4.67E-01	5.67E-01	6.11E-01	3.64E-01	9.81E-01	8.79E-02	4.25E-01	9.37E-01
ENSBTAG00000021911	2.77E-01	5.76E-01	1.52E+00	5.88E-18	1.46E+00	8.27E-17	9.20E-01	3.86E-07	-1.67E-01	9.02E-01
ENSBTAG00000044501	0.00E+00	1.00E+00	0.00E+00	1.00E+00	-5.13E-15	1.00E+00	0.00E+00	1.00E+00	0.00E+00	1.00E+00
ENSBTAG00000014418	1.83E-01	1.00E+00	9.07E-02	1.00E+00	-8.68E-01	2.91E-01	-9.56E-01	2.39E-01	-1.37E-01	1.00E+00
ENSBTAG00000047771	0.00E+00	1.00E+00	0.00E+00	1.00E+00	-5.13E-15	1.00E+00	0.00E+00	1.00E+00	0.00E+00	1.00E+00
ENSBTAG00000044432	0.00E+00	1.00E+00	0.00E+00	1.00E+00	-5.13E-15	1.00E+00	0.00E+00	1.00E+00	0.00E+00	1.00E+00
ENSBTAG00000047999	0.00E+00	1.00E+00	0.00E+00	1.00E+00	-5.13E-15	1.00E+00	1.77E+00	4.97E-01	0.00E+00	1.00E+00
ENSBTAG00000045313	0.00E+00	1.00E+00	0.00E+00	1.00E+00	-5.13E-15	1.00E+00	0.00E+00	1.00E+00	0.00E+00	1.00E+00
ENSBTAG00000036442	0.00E+00	1.00E+00	0.00E+00	1.00E+00	-5.13E-15	1.00E+00	0.00E+00	1.00E+00	0.00E+00	1.00E+00
ENSBTAG00000020373	1.21E-02	1.00E+00	-4.20E-01	8.03E-01	6.24E-02	1.00E+00	-2.89E-01	9.73E-01	1.06E-01	1.00E+00
ENSBTAG00000023207	-1.81E+00	1.30E-01	-3.96E+00	1.46E-04	-2.53E+00	2.32E-03	-2.26E+00	5.83E-03	-3.24E+00	1.10E-02
ENSBTAG00000046763	1.79E+00	4.19E-06	2.48E+00	2.63E-12	1.80E+00	3.45E-07	1.79E+00	5.82E-07	1.46E+00	1.57E-03
ENSBTAG00000004178	-9.93E-02	1.00E+00	-1.89E+00	7.90E-16	-3.02E+00	2.87E-36	-2.65E+00	1.05E-28	5.07E-01	3.04E-01
ENSBTAG00000022656	5.78E-01	1.82E-01	2.64E-01	4.87E-01	2.95E-01	4.24E-01	-2.08E-01	6.62E-01	1.23E-01	1.00E+00
ENSBTAG00000021724	-6.26E-02	1.00E+00	-6.54E-01	4.45E-02	-8.13E-01	9.01E-03	-4.24E-01	2.67E-01	-4.47E-02	1.00E+00

ENSBTAG00000019989	-3.48E-01	7.52E-01	-1.62E-01	8.39E-01	-1.02E+00	2.57E-04	-1.09E+00	9.55E-05	-1.75E-02	1.00E+00
ENSBTAG00000019987	-1.44E-01	1.00E+00	4.44E-01	7.83E-02	1.15E+00	1.14E-07	8.50E-01	1.75E-04	-1.10E-01	1.00E+00
ENSBTAG00000016615	-2.01E-01	1.00E+00	-1.91E+00	1.39E-11	-1.18E+00	1.64E-05	-9.99E-01	3.46E-04	-1.93E-01	1.00E+00
ENSBTAG00000043037	0.00E+00	1.00E+00	0.00E+00	1.00E+00	-5.13E-15	1.00E+00	0.00E+00	1.00E+00	0.00E+00	1.00E+00
ENSBTAG00000018294	-2.50E-01	8.23E-01	-5.56E-01	2.26E-02	-6.70E-01	4.36E-03	-6.71E-01	5.07E-03	-5.50E-01	1.81E-01
ENSBTAG00000022004	1.82E-02	1.00E+00	4.69E-01	1.23E-01	6.35E-01	2.41E-02	4.15E-01	2.03E-01	3.39E-01	7.37E-01
ENSBTAG00000044386	0.00E+00	1.00E+00	0.00E+00	1.00E+00	-5.13E-15	1.00E+00	0.00E+00	1.00E+00	0.00E+00	1.00E+00
ENSBTAG00000021164	-2.06E-02	1.00E+00	-1.60E-01	6.29E-01	-3.99E-01	6.56E-02	-3.72E-01	9.93E-02	-3.79E-01	3.76E-01
ENSBTAG00000018433	-1.26E-01	1.00E+00	-9.82E-01	5.22E-03	-7.08E-01	5.52E-02	-5.07E-01	2.24E-01	7.60E-02	1.00E+00
ENSBTAG00000016069	8.84E-01	6.03E-04	2.42E+00	2.25E-28	2.32E+00	1.70E-26	1.88E+00	5.13E-18	-7.44E-02	1.00E+00
ENSBTAG00000015563	4.61E-01	6.35E-01	3.15E+00	6.63E-24	3.08E+00	3.32E-23	2.49E+00	9.08E-16	3.40E-02	1.00E+00
ENSBTAG00000020802	1.68E-01	1.00E+00	-7.95E-01	6.15E-03	-1.12E+00	5.22E-05	-1.46E+00	1.75E-07	-1.50E-01	1.00E+00
ENSBTAG00000016013	3.56E-01	8.23E-01	1.31E-01	9.90E-01	-2.67E-01	6.12E-01	-1.06E+00	2.34E-03	-3.55E-02	1.00E+00
ENSBTAG00000006824	2.74E-01	8.49E-01	-3.53E-01	3.26E-01	-1.43E-01	8.84E-01	3.12E-01	4.13E-01	-5.06E-02	1.00E+00
ENSBTAG00000001439	-6.86E-01	3.76E-01	-1.43E+00	4.02E-04	-2.21E+00	5.53E-08	-2.30E+00	2.39E-08	-1.16E-01	1.00E+00
ENSBTAG00000010999	9.81E-01	6.07E-01	2.33E-01	1.00E+00	-2.48E-02	1.00E+00	9.29E-01	2.86E-01	3.57E-01	1.00E+00
ENSBTAG00000014091	-4.10E-02	1.00E+00	-2.84E-01	5.27E-01	-7.05E-01	2.90E-02	-6.25E-01	6.51E-02	-8.02E-01	1.11E-01
ENSBTAG00000019748	3.89E-03	1.00E+00	-2.70E-01	4.43E-01	2.38E-01	5.04E-01	4.95E-01	7.49E-02	-1.44E-01	1.00E+00
ENSBTAG00000019746	-3.80E-01	3.53E-01	-3.78E-01	1.05E-01	7.61E-03	1.00E+00	-3.03E-01	2.36E-01	-3.77E-01	4.31E-01
ENSBTAG00000010029	5.34E-01	9.69E-01	8.63E-01	2.78E-01	-2.98E-01	9.52E-01	1.10E+00	1.31E-01	-1.09E+00	5.99E-01
ENSBTAG00000043482	-8.74E-01	4.87E-01	-1.12E+00	7.27E-02	-4.55E-01	5.49E-01	-1.23E+00	3.95E-02	-1.11E+00	3.37E-01
ENSBTAG00000020221	-3.12E-01	1.00E+00	-1.16E+00	1.41E-01	-2.29E+00	1.83E-03	-2.02E+00	6.41E-03	3.52E-01	1.00E+00
ENSBTAG00000013117	1.02E-02	1.00E+00	-4.89E-01	3.31E-01	-2.19E+00	7.78E-08	-1.49E+00	2.10E-04	-2.06E-01	1.00E+00
ENSBTAG00000013124	0.00E+00	1.00E+00	0.00E+00	1.00E+00	-5.13E-15	1.00E+00	0.00E+00	1.00E+00	0.00E+00	1.00E+00
ENSBTAG00000043206	-1.91E+00	8.23E-01	-1.91E+00	4.69E-01	-1.91E+00	4.28E-01	-1.91E+00	4.39E-01	-1.91E+00	8.00E-01
ENSBTAG00000045472	0.00E+00	1.00E+00	0.00E+00	1.00E+00	-5.13E-15	1.00E+00	0.00E+00	1.00E+00	0.00E+00	1.00E+00
ENSBTAG00000044233	0.00E+00	1.00E+00	0.00E+00	1.00E+00	-5.13E-15	1.00E+00	0.00E+00	1.00E+00	0.00E+00	1.00E+00
ENSBTAG00000032374	1.08E+00	4.78E-03	3.15E+00	2.65E-23	2.28E+00	1.45E-13	1.45E+00	3.86E-06	-2.10E-02	1.00E+00
ENSBTAG00000011180	2.57E-01	8.52E-01	4.47E-01	1.26E-01	1.95E-01	6.65E-01	5.51E-01	4.92E-02	-9.09E-03	1.00E+00
ENSBTAG00000011178	3.97E-01	9.72E-01	1.44E+00	2.56E-03	9.67E-01	6.12E-02	1.31E+00	7.93E-03	-2.14E-01	1.00E+00
ENSBTAG00000010027	-3.32E-01	9.12E-01	-1.32E+00	2.45E-04	-1.28E+00	4.01E-04	-6.72E-01	1.04E-01	-1.06E-01	1.00E+00
ENSBTAG00000010026	3.41E-02	1.00E+00	-8.99E-02	1.00E+00	-6.35E-01	3.74E-01	5.53E-01	4.38E-01	3.18E-01	1.00E+00
ENSBTAG00000032347	-2.15E-01	8.28E-01	-4.35E-01	5.60E-02	-6.12E-01	3.87E-03	-5.78E-01	7.93E-03	-3.70E-02	1.00E+00
ENSBTAG00000011633	-1.57E-01	1.00E+00	-4.16E-01	1.50E-01	-4.48E-01	1.10E-01	-8.35E-02	1.00E+00	-1.78E-01	1.00E+00
ENSBTAG00000010863	1.96E-01	9.13E-01	1.12E+00	2.70E-08	1.52E+00	1.12E-14	1.11E+00	3.87E-08	4.42E-02	1.00E+00

ENSBTAG00000003758	-6.95E-01	8.07E-02	-1.07E+00	8.15E-05	-1.36E+00	3.09E-07	-1.31E+00	1.18E-06	-7.54E-01	7.81E-02
ENSBTAG00000008719	5.20E-01	2.29E-01	3.13E-01	3.45E-01	4.46E-01	1.18E-01	7.05E-01	6.76E-03	-1.56E-01	1.00E+00
ENSBTAG00000020973	-2.73E-01	9.29E-01	-5.36E-01	1.24E-01	-6.83E-02	1.00E+00	6.32E-02	1.00E+00	2.32E-01	9.77E-01
ENSBTAG00000011345	-2.58E-01	7.73E-01	-1.32E+00	1.98E-10	-3.65E-01	1.37E-01	-7.55E-02	1.00E+00	2.77E-01	7.11E-01
ENSBTAG00000011344	2.44E-01	1.00E+00	8.66E-01	1.29E-02	6.90E-01	5.88E-02	7.47E-01	4.15E-02	-6.05E-02	1.00E+00
ENSBTAG00000032531	4.05E-02	1.00E+00	1.55E+00	5.66E-03	4.27E-02	1.00E+00	1.06E+00	8.81E-02	2.41E-01	1.00E+00
ENSBTAG00000007850	2.37E+00	2.49E-11	3.66E+00	2.62E-25	4.01E+00	1.61E-29	4.22E+00	8.10E-32	4.19E+00	4.13E-30
ENSBTAG00000007846	-1.11E-02	1.00E+00	-4.60E-01	2.83E-01	-1.14E+00	8.65E-04	-1.06E+00	2.53E-03	2.57E-01	9.93E-01
ENSBTAG00000007843	2.04E-01	1.00E+00	-6.96E-02	1.00E+00	-8.65E-01	5.25E-02	-9.86E-01	2.61E-02	9.87E-02	1.00E+00
ENSBTAG00000004749	2.88E-03	1.00E+00	-8.10E-01	1.99E-05	-6.28E-01	1.14E-03	-2.23E-01	4.05E-01	-2.83E-01	6.01E-01
ENSBTAG00000043291	0.00E+00	1.00E+00	0.00E+00	1.00E+00	-5.13E-15	1.00E+00	0.00E+00	1.00E+00	0.00E+00	1.00E+00
ENSBTAG00000004747	5.64E-02	1.00E+00	-6.78E-02	1.00E+00	-1.47E-01	7.68E-01	-1.01E-01	9.59E-01	-2.94E-01	7.14E-01
ENSBTAG00000014550	2.76E-01	6.25E-01	1.11E-01	8.53E-01	2.30E-02	1.00E+00	7.91E-02	9.96E-01	4.21E-01	2.78E-01
ENSBTAG00000014549	9.55E-01	9.87E-05	3.17E+00	1.65E-47	3.06E+00	5.38E-45	2.24E+00	6.11E-26	-5.59E-01	1.51E-01
ENSBTAG00000043140	0.00E+00	1.00E+00	0.00E+00	1.00E+00	-5.13E-15	1.00E+00	0.00E+00	1.00E+00	0.00E+00	1.00E+00
ENSBTAG00000043914	0.00E+00	1.00E+00	0.00E+00	1.00E+00	-5.13E-15	1.00E+00	0.00E+00	1.00E+00	0.00E+00	1.00E+00
ENSBTAG00000044972	0.00E+00	1.00E+00	0.00E+00	1.00E+00	-5.13E-15	1.00E+00	0.00E+00	1.00E+00	0.00E+00	1.00E+00
ENSBTAG00000042117	0.00E+00	1.00E+00	0.00E+00	1.00E+00	-5.13E-15	1.00E+00	0.00E+00	1.00E+00	0.00E+00	1.00E+00
ENSBTAG00000014786	9.87E-02	1.00E+00	-7.21E-01	5.34E-03	-1.19E-01	9.21E-01	3.23E-01	3.14E-01	3.71E-02	1.00E+00
ENSBTAG00000044079	-5.37E-01	6.13E-01	-1.81E+00	3.28E-06	-1.21E+00	1.78E-03	-4.79E-01	3.36E-01	1.68E-02	1.00E+00
ENSBTAG00000014784	6.44E-02	1.00E+00	-3.97E-01	3.03E-01	-4.58E-01	2.01E-01	-2.28E-02	1.00E+00	-8.09E-02	1.00E+00
ENSBTAG00000014782	-5.66E-01	2.22E-01	-1.73E+00	4.25E-11	-1.08E+00	5.05E-05	-1.45E+00	4.10E-08	-2.65E-01	8.38E-01
ENSBTAG00000014779	2.14E-02	1.00E+00	-5.11E-01	1.10E-02	-6.40E-01	9.36E-04	-7.34E-01	1.48E-04	-7.08E-02	1.00E+00
ENSBTAG00000045757	3.69E-01	1.00E+00	1.47E+00	3.54E-01	-3.55E-01	1.00E+00	1.23E+00	4.79E-01	8.87E-01	9.98E-01
ENSBTAG00000046924	-4.63E-01	1.00E+00	-1.63E+00	9.24E-02	-5.16E-01	6.92E-01	-6.81E-01	5.27E-01	1.47E+00	1.16E-01
ENSBTAG00000047658	-2.90E-01	1.00E+00	-2.06E+00	5.49E-06	-1.69E+00	1.78E-04	-1.40E+00	2.64E-03	6.49E-01	6.01E-01
ENSBTAG00000045817	-1.18E-01	1.00E+00	4.08E-02	1.00E+00	4.56E-01	6.61E-02	5.45E-01	2.51E-02	2.42E-01	8.00E-01
ENSBTAG00000047794	-5.42E-01	9.18E-01	8.86E-01	1.43E-01	5.17E-01	4.90E-01	-1.17E-01	1.00E+00	6.32E-01	7.84E-01
ENSBTAG00000029825	0.00E+00	1.00E+00	0.00E+00	1.00E+00	-5.13E-15	1.00E+00	0.00E+00	1.00E+00	0.00E+00	1.00E+00
ENSBTAG00000021695	-6.42E-01	4.75E-02	-2.14E+00	5.96E-21	-2.45E+00	8.42E-27	-2.25E+00	6.14E-23	1.35E-01	1.00E+00
ENSBTAG00000002960	-1.16E-01	1.00E+00	9.10E-02	9.06E-01	2.07E-01	4.02E-01	1.61E-01	5.72E-01	1.70E-02	1.00E+00
ENSBTAG00000029861	0.00E+00	1.00E+00	0.00E+00	1.00E+00	-5.13E-15	1.00E+00	0.00E+00	1.00E+00	0.00E+00	1.00E+00
ENSBTAG00000002962	0.00E+00	1.00E+00	0.00E+00	1.00E+00	-5.13E-15	1.00E+00	0.00E+00	1.00E+00	0.00E+00	1.00E+00
ENSBTAG00000018198	5.95E-01	9.30E-01	8.39E-01	3.19E-01	-2.99E-01	9.79E-01	-4.49E-01	8.06E-01	7.18E-01	8.00E-01
ENSBTAG00000004118	-2.18E+00	3.35E-07	-4.94E+00	4.26E-30	-4.23E+00	4.94E-24	-2.48E+00	5.21E-10	-2.70E-01	1.00E+00

ENSBTAG00000001471	-3.56E-01	8.23E-01	-2.76E+00	4.11E-15	-3.17E+00	3.42E-19	-2.49E+00	4.62E-13	-4.17E-01	7.63E-01
ENSBTAG00000021912	-1.02E-01	1.00E+00	2.70E-03	1.00E+00	7.52E-01	5.39E-03	3.57E-01	2.99E-01	-1.69E-01	1.00E+00
ENSBTAG00000018628	-7.15E-02	1.00E+00	-7.48E-04	1.00E+00	-1.24E-01	8.36E-01	-4.77E-02	1.00E+00	-5.49E-02	1.00E+00
ENSBTAG00000032304	-1.76E-01	1.00E+00	-8.51E-01	9.84E-05	-1.79E+00	2.73E-17	-1.76E+00	1.22E-16	3.87E-02	1.00E+00
ENSBTAG00000037377	-2.76E-01	6.78E-01	-6.68E-01	1.27E-03	-1.45E+00	1.60E-13	-1.78E+00	2.70E-19	-2.37E-01	8.00E-01
ENSBTAG00000018622	-1.32E-01	1.00E+00	-4.01E-01	3.01E-01	-8.25E-01	8.84E-03	-8.92E-01	4.98E-03	1.10E-01	1.00E+00
ENSBTAG00000013442	1.09E+00	7.91E-01	1.70E+00	7.37E-02	1.81E+00	4.13E-02	1.41E+00	1.77E-01	-9.33E-03	1.00E+00
ENSBTAG00000000671	1.90E+00	5.51E-11	3.52E+00	1.55E-35	3.05E+00	5.64E-28	2.24E+00	4.09E-16	-3.84E-02	1.00E+00
ENSBTAG00000000670	1.25E-01	1.00E+00	2.98E+00	8.40E-31	2.60E+00	1.74E-24	1.47E+00	9.81E-09	-3.46E-01	7.35E-01
ENSBTAG00000000805	0.00E+00	1.00E+00	4.44E+00	6.84E-03	-5.13E-15	1.00E+00	2.53E+00	2.71E-01	0.00E+00	1.00E+00
ENSBTAG00000040574	0.00E+00	1.00E+00	0.00E+00	1.00E+00	-5.13E-15	1.00E+00	0.00E+00	1.00E+00	0.00E+00	1.00E+00
ENSBTAG00000032279	0.00E+00	1.00E+00	0.00E+00	1.00E+00	-5.13E-15	1.00E+00	2.53E+00	2.71E-01	1.91E+00	8.00E-01
ENSBTAG00000048044	-1.91E+00	8.23E-01	-1.91E+00	4.69E-01	-1.24E-01	1.00E+00	-1.91E+00	4.39E-01	-1.91E+00	8.00E-01
ENSBTAG00000037410	8.90E-01	1.00E+00	9.13E-02	1.00E+00	7.28E-01	8.21E-01	-9.40E-01	8.06E-01	1.79E-02	1.00E+00
ENSBTAG00000045942	0.00E+00	1.00E+00	0.00E+00	1.00E+00	-5.13E-15	1.00E+00	0.00E+00	1.00E+00	0.00E+00	1.00E+00
ENSBTAG00000039664	-9.80E-02	1.00E+00	-8.04E-01	1.68E-03	-2.24E+00	3.14E-19	-2.82E+00	8.41E-28	-5.99E-02	1.00E+00
ENSBTAG00000001067	-1.80E+00	4.80E-01	-2.53E+00	6.47E-02	-4.52E+00	3.15E-03	-2.76E+00	3.66E-02	-1.81E+00	5.16E-01
ENSBTAG00000034796	-4.18E-01	2.90E-01	-1.11E+00	9.73E-08	-1.60E+00	9.60E-15	-1.18E+00	1.59E-08	-4.00E-02	1.00E+00
ENSBTAG00000032253	8.03E-01	1.26E-02	3.11E-01	3.66E-01	2.28E-01	5.63E-01	3.15E-01	3.62E-01	8.48E-01	1.68E-02
ENSBTAG00000006501	2.84E-01	6.14E-01	9.06E-01	3.00E-06	9.50E-01	7.81E-07	7.34E-01	2.40E-04	1.40E-01	1.00E+00
ENSBTAG00000006500	7.60E-02	1.00E+00	-2.56E-03	1.00E+00	1.40E+00	5.59E-07	6.86E-01	2.87E-02	2.18E-01	9.98E-01
ENSBTAG00000031797	2.39E+00	7.63E-13	4.87E+00	3.18E-44	4.50E+00	1.71E-39	3.35E+00	1.69E-24	5.56E-01	4.81E-01
ENSBTAG00000001874	4.70E-01	5.70E-01	-2.64E-01	6.32E-01	-3.65E-01	4.12E-01	-5.95E-01	1.16E-01	4.58E-01	6.01E-01
ENSBTAG00000047517	0.00E+00	1.00E+00	0.00E+00	1.00E+00	-5.13E-15	1.00E+00	0.00E+00	1.00E+00	0.00E+00	1.00E+00
ENSBTAG00000016532	-6.87E-02	1.00E+00	-5.17E-01	6.25E-02	3.90E-01	1.91E-01	6.40E-01	1.59E-02	6.13E-02	1.00E+00
ENSBTAG00000022622	3.04E+00	3.62E-27	2.58E+00	3.44E-21	1.81E+00	1.65E-11	1.47E+00	7.70E-08	8.00E-01	6.28E-02
ENSBTAG00000004120	1.80E-01	9.85E-01	4.07E-01	1.01E-01	3.37E-01	1.99E-01	3.07E-01	2.71E-01	-2.62E-01	7.90E-01
ENSBTAG00000010165	-3.12E-03	1.00E+00	-1.81E+00	4.83E-03	-2.56E+00	8.92E-05	-4.02E-01	6.68E-01	1.60E-01	1.00E+00
ENSBTAG00000009489	4.72E-01	1.00E+00	-1.09E+00	5.49E-01	-1.33E+00	4.16E-01	-8.53E-01	6.65E-01	2.56E-02	1.00E+00
ENSBTAG00000002634	-2.12E-01	8.46E-01	6.21E-01	3.28E-03	6.10E-01	3.85E-03	2.42E-01	4.06E-01	-1.57E-02	1.00E+00
ENSBTAG00000019163	-1.72E-01	1.00E+00	-6.45E-01	7.97E-03	-4.36E-01	9.47E-02	-9.45E-02	9.93E-01	-1.47E-01	1.00E+00
ENSBTAG00000019161	7.85E-02	1.00E+00	8.40E-02	1.00E+00	-2.75E-01	3.74E-01	-3.68E-01	1.90E-01	6.02E-02	1.00E+00
ENSBTAG00000020965	1.92E-02	1.00E+00	3.36E+00	1.03E-07	4.71E-01	8.89E-01	4.41E-01	9.26E-01	-6.47E-01	1.00E+00
ENSBTAG00000020963	-8.96E-02	1.00E+00	1.94E+00	3.60E-14	1.23E+00	1.76E-06	5.16E-01	8.72E-02	-5.14E-01	3.76E-01
ENSBTAG00000020962	6.19E-02	1.00E+00	4.01E-01	2.21E-01	1.10E+00	1.86E-05	1.19E+00	4.41E-06	3.25E-01	7.80E-01

ENSBTAG00000000484	5.69E-01	5.10E-01	-4.58E-01	3.34E-01	2.49E-01	7.23E-01	3.17E-01	5.75E-01	-2.90E-01	9.60E-01
ENSBTAG00000000483	-4.38E-02	1.00E+00	-1.18E+00	1.21E-03	-1.22E+00	6.08E-04	-1.05E+00	4.24E-03	-7.61E-02	1.00E+00
ENSBTAG00000038084	-3.22E-01	8.74E-01	-1.78E+00	1.15E-06	-1.59E+00	5.33E-06	-1.10E+00	1.80E-03	1.67E-01	1.00E+00
ENSBTAG00000047200	4.89E-02	1.00E+00	-9.88E-01	2.17E-02	-1.19E+00	4.19E-03	-1.07E+00	1.21E-02	-4.25E-02	1.00E+00
ENSBTAG00000000480	-1.96E-01	1.00E+00	8.05E-01	1.13E-03	1.08E+00	6.32E-06	7.49E-01	3.21E-03	-2.82E-01	8.00E-01
ENSBTAG00000000478	-1.67E+00	5.98E-04	-1.17E+00	5.94E-03	-2.17E+00	3.42E-07	-2.42E+00	3.21E-08	-3.87E-01	8.33E-01
ENSBTAG00000010138	-3.49E-01	1.00E+00	-3.29E-01	7.67E-01	-1.40E+00	8.26E-03	-1.68E+00	1.54E-03	-4.26E-01	9.24E-01
ENSBTAG00000020645	8.34E-02	1.00E+00	-2.25E-02	1.00E+00	-2.52E-01	3.08E-01	-1.17E-01	8.13E-01	-1.13E-01	1.00E+00
ENSBTAG00000008509	-1.48E-01	1.00E+00	-1.44E+00	8.49E-08	-2.30E+00	9.39E-18	-1.27E+00	3.01E-06	4.25E-01	5.85E-01
ENSBTAG00000018020	-4.05E-01	4.65E-01	-2.18E+00	5.75E-19	-2.15E+00	7.18E-19	-1.35E+00	2.03E-08	6.31E-01	1.15E-01
ENSBTAG00000018307	8.70E-01	9.82E-02	1.00E+00	5.65E-03	6.50E-01	1.04E-01	5.18E-01	2.41E-01	-4.13E-02	1.00E+00
ENSBTAG00000006330	2.25E-01	8.00E-01	3.57E-01	9.77E-02	-2.05E-01	4.42E-01	-3.85E-01	7.58E-02	1.57E-01	9.47E-01
ENSBTAG00000042160	0.00E+00	1.00E+00	0.00E+00	1.00E+00	-5.13E-15	1.00E+00	2.53E+00	2.71E-01	0.00E+00	1.00E+00
ENSBTAG00000006328	-7.04E-02	1.00E+00	2.21E-01	3.75E-01	4.18E-01	3.43E-02	-1.90E-02	1.00E+00	-7.90E-02	1.00E+00
ENSBTAG00000020047	-3.13E-01	6.69E-01	5.44E-01	2.55E-02	6.14E-01	9.50E-03	4.72E-01	6.65E-02	-6.19E-02	1.00E+00
ENSBTAG00000015046	1.68E+00	8.16E-09	4.91E+00	1.46E-61	4.32E+00	1.69E-50	2.68E+00	2.09E-22	3.67E-01	7.35E-01
ENSBTAG00000040083	0.00E+00	1.00E+00	0.00E+00	1.00E+00	-5.13E-15	1.00E+00	0.00E+00	1.00E+00	0.00E+00	1.00E+00
ENSBTAG00000004878	-4.67E-01	9.02E-01	-3.88E+00	9.54E-08	-1.27E+00	2.24E-02	-1.44E+00	1.06E-02	-2.07E-01	1.00E+00
ENSBTAG00000004877	6.48E-01	8.23E-01	-7.92E-01	4.12E-01	9.63E-01	1.15E-01	9.89E-01	1.09E-01	6.65E-01	8.00E-01
ENSBTAG00000012335	-1.25E-01	1.00E+00	-8.89E-02	1.00E+00	-4.86E-01	6.12E-01	-8.81E-01	2.27E-01	7.93E-02	1.00E+00
ENSBTAG00000012322	-1.89E+00	1.03E-03	-5.79E-01	3.47E-01	-4.07E-01	5.66E-01	-6.14E-01	3.06E-01	-4.46E-01	8.52E-01
ENSBTAG00000005728	1.58E+00	3.91E-02	5.65E+00	9.22E-24	5.99E+00	3.92E-26	4.74E+00	4.97E-18	-6.15E-01	8.48E-01
ENSBTAG00000011595	4.58E-01	3.75E-01	1.41E+00	5.70E-09	1.27E+00	1.62E-07	9.34E-01	2.11E-04	5.08E-01	3.32E-01
ENSBTAG00000032026	1.79E+00	6.09E-07	4.13E+00	2.35E-32	3.63E+00	2.05E-26	3.27E+00	3.47E-22	3.85E-01	8.00E-01
ENSBTAG00000011588	4.10E-01	3.49E-01	2.49E+00	4.48E-31	1.69E+00	1.11E-15	9.42E-01	1.68E-05	2.89E-01	7.14E-01
ENSBTAG00000045547	0.00E+00	1.00E+00	0.00E+00	1.00E+00	-5.13E-15	1.00E+00	0.00E+00	1.00E+00	0.00E+00	1.00E+00
ENSBTAG00000011585	4.49E-01	3.84E-01	1.24E-01	9.21E-01	-6.24E-01	1.93E-02	-1.08E+00	1.71E-05	7.54E-01	4.58E-02
ENSBTAG00000011583	-3.45E-01	3.88E-01	-1.05E+00	3.08E-08	-1.85E+00	4.69E-23	-1.65E+00	1.17E-18	7.05E-03	1.00E+00
ENSBTAG00000011581	-4.24E-01	8.23E-01	-1.48E+00	3.08E-04	-2.21E-01	8.37E-01	3.88E-01	4.93E-01	2.34E-01	1.00E+00
ENSBTAG00000011580	3.47E-02	1.00E+00	-4.18E-01	2.66E-01	1.27E-01	9.76E-01	1.11E+00	2.51E-04	7.19E-01	1.76E-01
ENSBTAG00000022632	-1.39E-01	1.00E+00	-5.85E-01	2.32E-02	-9.20E-01	1.33E-04	-6.43E-01	1.25E-02	-4.88E-01	3.35E-01
ENSBTAG00000002321	-2.29E-01	8.49E-01	-1.62E+00	1.89E-13	-2.36E+00	3.51E-26	-2.83E+00	1.89E-35	1.99E-01	9.07E-01
ENSBTAG00000000387	-1.48E-01	1.00E+00	-1.01E+00	7.82E-07	-1.14E+00	1.34E-08	-1.13E+00	2.90E-08	-1.30E-01	1.00E+00
ENSBTAG00000004279	1.42E-01	1.00E+00	4.10E-01	4.13E-02	5.30E-01	5.41E-03	6.45E-01	6.25E-04	-7.48E-02	1.00E+00
ENSBTAG00000044873	-1.91E+00	8.23E-01	-1.91E+00	4.69E-01	6.49E-01	9.78E-01	-1.47E-01	1.00E+00	-1.91E+00	8.00E-01

ENSBTAG00000011899	9.59E-02	1.00E+00	1.04E+00	8.65E-07	1.42E+00	8.13E-12	1.19E+00	1.50E-08	-5.26E-02	1.00E+00
ENSBTAG00000011894	6.17E-01	5.01E-01	3.75E+00	3.17E-28	2.73E+00	6.30E-16	1.59E+00	9.56E-06	-4.18E-01	8.24E-01
ENSBTAG00000009371	-2.61E-01	1.00E+00	5.42E-01	3.57E-01	-3.87E-01	6.20E-01	-3.10E-01	7.67E-01	3.94E-01	9.04E-01
ENSBTAG00000000982	6.08E-03	1.00E+00	7.63E-02	1.00E+00	-1.91E+00	4.28E-01	-1.91E+00	4.39E-01	1.69E+00	7.74E-01
ENSBTAG00000000981	-3.65E-01	7.76E-01	-9.34E-01	3.35E-03	-5.39E-01	1.19E-01	-3.74E-02	1.00E+00	-3.31E-01	8.00E-01
ENSBTAG00000013932	-4.39E-01	4.33E-01	5.33E-01	5.31E-02	8.93E-01	3.38E-04	4.90E-01	8.74E-02	-1.13E-01	1.00E+00
ENSBTAG00000022635	-1.42E-01	1.00E+00	-5.39E-01	9.29E-02	-6.57E-01	3.06E-02	-9.68E-01	9.16E-04	8.24E-02	1.00E+00
ENSBTAG00000018921	-1.32E-01	1.00E+00	3.91E-01	1.13E-01	8.38E-01	8.38E-05	4.17E-01	9.24E-02	3.68E-01	4.87E-01
ENSBTAG00000018928	9.22E-02	1.00E+00	3.47E-01	1.26E-01	1.76E-01	5.65E-01	1.04E-01	8.87E-01	6.68E-02	1.00E+00
ENSBTAG00000018924	-2.60E-01	8.02E-01	-1.62E-01	7.01E-01	-1.30E-01	8.22E-01	-2.41E-01	4.48E-01	1.74E-02	1.00E+00
ENSBTAG00000031837	1.72E-01	9.85E-01	1.52E+00	2.33E-14	2.21E+00	1.81E-28	1.94E+00	2.35E-22	-1.92E-01	8.95E-01
ENSBTAG00000018918	-1.01E-01	1.00E+00	-8.82E-02	1.00E+00	-3.63E-01	1.97E-01	-6.72E-01	6.24E-03	-2.89E-01	7.69E-01
ENSBTAG00000029957	8.90E-01	3.41E-01	2.31E+00	8.77E-09	1.59E+00	2.34E-04	8.24E-01	1.35E-01	-4.76E-02	1.00E+00
ENSBTAG00000029913	4.83E-01	6.33E-01	2.13E+00	1.64E-12	1.72E+00	1.60E-08	7.10E-01	5.88E-02	7.66E-02	1.00E+00
ENSBTAG00000018913	-8.89E-02	1.00E+00	4.05E-01	2.03E-01	5.45E-01	5.87E-02	7.03E-02	1.00E+00	-4.04E-02	1.00E+00
ENSBTAG00000018910	-7.40E-02	1.00E+00	8.24E-01	2.25E-04	7.72E-01	5.79E-04	5.10E-01	3.84E-02	2.07E-01	8.88E-01
ENSBTAG00000005490	-5.79E-01	2.56E-01	-1.50E+00	3.06E-07	-1.12E+00	1.04E-04	-1.22E+00	2.51E-05	1.54E-01	1.00E+00
ENSBTAG00000005488	-3.32E-02	1.00E+00	9.02E-01	9.25E-07	7.93E-01	1.78E-05	4.80E-01	1.79E-02	2.53E-01	6.95E-01
ENSBTAG00000000191	-3.35E-01	5.77E-01	-9.07E-01	5.94E-05	-1.83E+00	9.78E-17	-1.99E+00	2.19E-19	1.72E-01	9.93E-01
ENSBTAG00000014205	-1.47E-02	1.00E+00	1.71E-01	5.96E-01	-2.31E-02	1.00E+00	2.56E-01	3.31E-01	1.49E-01	1.00E+00
ENSBTAG00000021853	2.03E-03	1.00E+00	-6.43E-01	2.07E-03	-6.84E-01	8.95E-04	-4.24E-01	6.52E-02	6.47E-01	2.54E-02
ENSBTAG00000021850	-9.83E-02	1.00E+00	-5.87E-01	1.74E-01	-9.34E-02	1.00E+00	6.59E-01	9.54E-02	3.35E-01	8.55E-01
ENSBTAG00000021846	1.25E-01	1.00E+00	1.13E+00	5.98E-02	1.17E+00	4.21E-02	-2.61E-01	9.76E-01	-5.84E-01	9.00E-01
ENSBTAG00000045827	0.00E+00	1.00E+00	0.00E+00	1.00E+00	-5.13E-15	1.00E+00	0.00E+00	1.00E+00	0.00E+00	1.00E+00
ENSBTAG00000038381	-6.08E-02	1.00E+00	9.62E-02	1.00E+00	3.09E-01	4.79E-01	-1.18E-01	1.00E+00	-1.33E-01	1.00E+00
ENSBTAG00000021390	-7.50E-01	9.21E-01	-1.51E-01	1.00E+00	-5.65E-03	1.00E+00	-2.92E+00	6.80E-03	-7.62E-01	8.78E-01
ENSBTAG00000019096	-2.13E-01	1.00E+00	-2.83E-01	4.49E-01	-4.36E-01	1.64E-01	-2.46E-01	5.48E-01	-2.80E-01	8.00E-01
ENSBTAG00000019081	2.91E-02	1.00E+00	-5.55E-01	1.40E-01	-8.73E-01	9.68E-03	-1.40E+00	2.85E-05	3.93E-01	7.38E-01
ENSBTAG00000045476	0.00E+00	1.00E+00	0.00E+00	1.00E+00	-5.13E-15	1.00E+00	0.00E+00	1.00E+00	0.00E+00	1.00E+00
ENSBTAG00000019078	5.34E-03	1.00E+00	-1.91E+00	4.69E-01	-1.91E+00	4.28E-01	-1.48E-01	1.00E+00	6.46E-03	1.00E+00
ENSBTAG00000006752	9.82E-02	1.00E+00	-8.70E-01	2.35E-02	-1.58E-02	1.00E+00	-1.47E-01	9.72E-01	9.29E-02	1.00E+00
ENSBTAG00000009183	1.86E-01	1.00E+00	6.09E-01	7.88E-02	2.75E-01	5.69E-01	-5.23E-02	1.00E+00	-1.64E-01	1.00E+00
ENSBTAG00000008406	6.81E-01	2.80E-01	1.68E+00	3.47E-07	1.31E+00	8.56E-05	4.03E-01	4.09E-01	-7.56E-02	1.00E+00
ENSBTAG00000008399	4.19E-01	4.26E-01	1.80E+00	2.60E-15	2.09E+00	1.72E-20	1.11E+00	2.31E-06	-1.35E-01	1.00E+00
ENSBTAG00000013413	-4.02E-01	4.16E-01	-1.27E+00	2.37E-08	-3.67E-01	1.82E-01	-2.75E-01	3.92E-01	3.03E-01	7.08E-01

ENSBTAG00000013407	2.44E-01	1.00E+00	1.05E-01	1.00E+00	7.93E-01	8.62E-02	1.40E+00	9.08E-04	5.44E-01	7.08E-01
ENSBTAG00000031612	0.00E+00	1.00E+00	0.00E+00	1.00E+00	-5.13E-15	1.00E+00	1.76E+00	4.97E-01	1.91E+00	8.00E-01
ENSBTAG00000003025	-1.83E-01	1.00E+00	-6.50E-01	3.03E-02	-3.09E-01	4.27E-01	-5.36E-02	1.00E+00	-2.36E-01	9.27E-01
ENSBTAG00000003022	1.91E+00	8.23E-01	0.00E+00	1.00E+00	-5.13E-15	1.00E+00	0.00E+00	1.00E+00	0.00E+00	1.00E+00
ENSBTAG00000013356	0.00E+00	1.00E+00	0.00E+00	1.00E+00	-5.13E-15	1.00E+00	0.00E+00	1.00E+00	1.91E+00	8.00E-01
ENSBTAG00000016153	0.00E+00	1.00E+00	0.00E+00	1.00E+00	-5.13E-15	1.00E+00	0.00E+00	1.00E+00	0.00E+00	1.00E+00
ENSBTAG00000031599	0.00E+00	1.00E+00	0.00E+00	1.00E+00	1.78E+00	4.79E-01	1.76E+00	4.97E-01	2.71E+00	6.01E-01
ENSBTAG00000039879	0.00E+00	1.00E+00	0.00E+00	1.00E+00	-5.13E-15	1.00E+00	0.00E+00	1.00E+00	1.91E+00	8.00E-01
ENSBTAG00000024852	0.00E+00	1.00E+00	0.00E+00	1.00E+00	-5.13E-15	1.00E+00	3.40E+00	8.36E-02	3.59E+00	3.09E-01
ENSBTAG00000031594	0.00E+00	1.00E+00	0.00E+00	1.00E+00	-5.13E-15	1.00E+00	0.00E+00	1.00E+00	0.00E+00	1.00E+00
ENSBTAG00000020077	0.00E+00	1.00E+00	0.00E+00	1.00E+00	1.78E+00	4.79E-01	3.94E+00	2.68E-02	2.71E+00	6.01E-01
ENSBTAG00000020076	6.83E-01	1.00E+00	-3.51E-01	1.00E+00	-1.28E+00	3.68E-01	-1.78E-01	1.00E+00	1.07E+00	7.13E-01
ENSBTAG00000009586	-3.09E-01	1.00E+00	-1.21E+00	3.76E-02	-6.63E-01	2.77E-01	1.02E+00	2.37E-02	-7.20E-01	6.22E-01
ENSBTAG00000044607	0.00E+00	1.00E+00	0.00E+00	1.00E+00	-5.13E-15	1.00E+00	0.00E+00	1.00E+00	0.00E+00	1.00E+00
ENSBTAG00000015839	-1.39E-01	1.00E+00	2.81E-01	4.43E-01	4.58E-01	1.27E-01	8.21E-01	2.25E-03	6.26E-02	1.00E+00
ENSBTAG00000047174	0.00E+00	1.00E+00	0.00E+00	1.00E+00	-5.13E-15	1.00E+00	0.00E+00	1.00E+00	0.00E+00	1.00E+00
ENSBTAG00000015833	-3.03E-02	1.00E+00	5.80E-01	2.15E-03	6.88E-01	1.89E-04	2.24E-01	3.84E-01	2.06E-02	1.00E+00
ENSBTAG00000031567	1.34E-01	1.00E+00	3.21E-01	4.01E-01	4.50E-01	1.68E-01	9.91E-01	3.94E-04	2.85E-01	8.09E-01
ENSBTAG00000002309	4.74E-01	1.00E+00	5.56E-01	6.49E-01	5.38E-01	6.44E-01	5.80E-01	5.99E-01	2.59E-01	1.00E+00
ENSBTAG00000011478	-6.14E-01	1.16E-01	-2.65E-01	4.62E-01	6.96E-01	5.48E-03	4.96E-01	7.36E-02	4.15E-01	4.99E-01
ENSBTAG00000042921	0.00E+00	1.00E+00	0.00E+00	1.00E+00	-5.13E-15	1.00E+00	1.76E+00	4.97E-01	0.00E+00	1.00E+00
ENSBTAG00000015782	-3.22E-01	7.03E-01	-1.88E-01	6.69E-01	-4.42E-01	1.14E-01	-4.08E-01	1.65E-01	2.24E-01	8.92E-01
ENSBTAG00000002774	7.35E-04	1.00E+00	6.71E-01	4.69E-04	7.23E-01	1.37E-04	3.87E-01	7.48E-02	7.91E-02	1.00E+00
ENSBTAG00000031519	-1.91E+00	8.23E-01	1.39E+00	5.17E-01	-1.91E+00	4.28E-01	1.78E+00	3.04E-01	7.14E-03	1.00E+00
ENSBTAG00000006573	4.84E-02	1.00E+00	-3.31E-01	2.83E-01	2.85E-01	3.77E-01	1.86E-01	6.67E-01	5.58E-01	1.98E-01
ENSBTAG00000019841	-2.18E-01	1.00E+00	-8.23E-02	1.00E+00	1.06E-02	1.00E+00	-6.79E-02	1.00E+00	-3.40E-01	8.00E-01
ENSBTAG00000002948	-1.29E-01	1.00E+00	9.92E-01	1.15E-06	1.25E+00	5.50E-10	6.11E-01	5.12E-03	8.33E-02	1.00E+00
ENSBTAG00000022598	-5.61E-01	2.07E-01	-1.77E+00	3.57E-11	-1.00E+00	1.31E-04	-1.30E+00	7.06E-07	-7.55E-02	1.00E+00
ENSBTAG00000011311	-9.49E-02	1.00E+00	-2.64E-01	4.37E-01	2.51E-01	4.48E-01	6.21E-01	1.28E-02	1.33E-01	1.00E+00
ENSBTAG00000016948	-4.39E-01	6.95E-01	-2.68E+00	2.92E-15	-6.40E-01	8.56E-02	2.77E-02	1.00E+00	-6.30E-02	1.00E+00
ENSBTAG00000008394	0.00E+00	1.00E+00	0.00E+00	1.00E+00	-5.13E-15	1.00E+00	0.00E+00	1.00E+00	0.00E+00	1.00E+00
ENSBTAG00000031454	1.08E+00	5.10E-02	6.63E-02	1.00E+00	1.39E-01	1.00E+00	-9.49E-01	8.72E-02	5.53E-01	7.10E-01
ENSBTAG00000037821	-4.94E-01	1.00E+00	4.67E-01	9.95E-01	7.39E-01	7.28E-01	1.29E+00	3.22E-01	1.15E+00	8.00E-01
ENSBTAG00000031447	1.70E+00	7.70E-01	7.58E-02	1.00E+00	-1.30E-01	1.00E+00	1.49E+00	4.55E-01	7.04E-03	1.00E+00
ENSBTAG00000038616	1.56E-02	1.00E+00	7.72E-01	7.32E-01	-1.43E+00	4.80E-01	1.86E-01	1.00E+00	-3.21E+00	4.34E-01

ENSBTAG00000005019	0.00E+00	1.00E+00	2.79E+00	2.10E-01	3.43E+00	7.35E-02	0.00E+00	1.00E+00	2.71E+00	6.01E-01
ENSBTAG00000004232	1.92E+00	8.23E-01	1.98E+00	4.43E-01	2.56E+00	2.54E-01	1.76E+00	4.97E-01	1.93E+00	8.00E-01
ENSBTAG00000017812	-1.49E-01	1.00E+00	-1.24E+00	6.48E-07	-1.75E-01	7.29E-01	-4.51E-01	1.29E-01	1.09E-01	1.00E+00
ENSBTAG00000021119	-7.86E-01	1.00E+00	-7.23E-01	9.45E-01	-9.24E-01	8.08E-01	6.87E-01	8.61E-01	-7.92E-01	1.00E+00
ENSBTAG00000008013	-3.35E-01	1.00E+00	-1.06E+00	6.47E-02	1.77E-01	1.00E+00	6.65E-01	2.30E-01	2.06E-01	1.00E+00
ENSBTAG00000018767	6.27E-01	1.00E+00	7.28E-01	6.41E-01	6.22E-01	7.22E-01	-8.63E-01	6.57E-01	-2.97E-01	1.00E+00
ENSBTAG00000001292	1.53E-01	1.00E+00	1.00E-01	1.00E+00	3.68E-01	5.91E-01	5.05E-02	1.00E+00	6.59E-01	5.95E-01
ENSBTAG00000006155	-1.94E+00	3.59E-05	-2.60E+00	8.47E-09	-1.04E+00	1.07E-02	-1.23E+00	2.70E-03	-8.87E-01	2.30E-01
ENSBTAG00000008099	-8.44E-01	9.07E-02	-1.29E+00	2.27E-04	-1.07E+00	1.80E-03	-1.09E+00	1.75E-03	-9.16E-01	9.29E-02
ENSBTAG00000013586	-7.08E-02	1.00E+00	-2.71E-01	8.53E-01	-2.43E-01	8.89E-01	-1.26E+00	2.25E-02	-6.78E-01	6.71E-01
ENSBTAG00000001338	-1.02E+00	5.76E-01	-2.58E+00	2.82E-03	-8.75E-01	2.74E-01	-3.44E-01	8.46E-01	-6.65E-01	8.04E-01
ENSBTAG00000031355	-1.03E+00	3.23E-01	-1.91E-01	1.00E+00	-6.29E-01	3.64E-01	-6.66E-01	3.34E-01	-8.23E-01	5.98E-01
ENSBTAG00000019428	9.32E-01	1.43E-01	8.45E-01	5.76E-02	1.54E+00	9.83E-05	2.04E+00	1.62E-07	5.00E-01	7.74E-01
ENSBTAG00000048004	-5.74E-01	6.13E-01	4.16E-01	4.26E-01	8.68E-01	2.06E-02	3.66E-01	4.97E-01	-5.69E-01	6.38E-01
ENSBTAG00000020019	1.21E+00	5.53E-01	1.11E-01	1.00E+00	2.86E-01	1.00E+00	2.99E-01	1.00E+00	6.14E-01	1.00E+00
ENSBTAG00000015698	6.22E-03	1.00E+00	-4.54E-01	3.18E-01	-1.72E-01	9.12E-01	2.76E-01	6.59E-01	6.33E-01	4.34E-01
ENSBTAG00000015708	0.00E+00	1.00E+00	0.00E+00	1.00E+00	-5.13E-15	1.00E+00	0.00E+00	1.00E+00	0.00E+00	1.00E+00
ENSBTAG00000031348	-4.24E-02	1.00E+00	2.71E-01	1.00E+00	9.22E-01	3.33E-01	-1.21E-02	1.00E+00	-3.12E-01	1.00E+00
ENSBTAG00000046434	0.00E+00	1.00E+00	0.00E+00	1.00E+00	-5.13E-15	1.00E+00	0.00E+00	1.00E+00	0.00E+00	1.00E+00
ENSBTAG00000001484	7.68E-01	1.85E-03	7.48E-01	3.77E-04	7.69E-01	2.17E-04	6.98E-01	1.12E-03	1.36E-01	1.00E+00
ENSBTAG00000031340	1.22E+00	9.42E-03	3.16E+00	3.34E-17	2.21E+00	2.02E-09	1.15E+00	3.36E-03	3.49E-01	8.87E-01
ENSBTAG00000003501	3.03E-01	7.98E-01	1.87E+00	6.75E-14	2.06E+00	1.11E-16	1.02E+00	6.89E-05	-3.07E-01	7.89E-01
ENSBTAG00000026097	4.22E-01	7.53E-01	1.94E+00	1.29E-09	1.36E+00	2.97E-05	1.13E+00	8.69E-04	5.41E-01	5.47E-01
ENSBTAG00000007315	1.65E-01	1.00E+00	1.48E+00	1.86E-07	1.19E+00	3.08E-05	5.82E-01	7.76E-02	1.61E-01	1.00E+00
ENSBTAG00000031287	2.06E+00	2.28E-03	4.16E+00	6.71E-17	5.10E+00	2.49E-25	3.60E+00	1.04E-12	3.92E-02	1.00E+00
ENSBTAG00000012482	-1.82E-01	1.00E+00	4.86E-01	2.51E-01	1.87E+00	3.65E-09	1.17E+00	5.10E-04	-4.77E-01	6.98E-01
ENSBTAG00000026650	0.00E+00	1.00E+00	0.00E+00	1.00E+00	-5.13E-15	1.00E+00	0.00E+00	1.00E+00	0.00E+00	1.00E+00
ENSBTAG00000018331	-3.78E-01	6.79E-01	-1.02E+00	3.05E-04	-1.31E+00	2.11E-06	-1.26E+00	6.81E-06	-6.71E-01	1.84E-01
ENSBTAG00000018329	1.69E-01	9.96E-01	8.44E-01	3.97E-05	1.20E+00	1.48E-09	6.69E-01	1.77E-03	1.36E-02	1.00E+00
ENSBTAG00000011484	-1.56E-01	1.00E+00	-3.12E-01	2.55E-01	5.26E-02	1.00E+00	4.12E-02	1.00E+00	-3.63E-02	1.00E+00
ENSBTAG00000016731	-3.80E-01	6.46E-01	-1.63E+00	2.79E-09	-1.02E+00	2.00E-04	-1.21E+00	1.18E-05	-1.90E-01	1.00E+00
ENSBTAG00000012328	0.00E+00	1.00E+00	3.30E+00	1.02E-01	2.55E+00	2.55E-01	0.00E+00	1.00E+00	0.00E+00	1.00E+00
ENSBTAG00000017825	4.14E-01	4.64E-01	1.23E+00	4.42E-07	1.23E+00	3.87E-07	1.05E+00	2.32E-05	1.33E-01	1.00E+00
ENSBTAG00000001305	7.82E-02	1.00E+00	-1.17E+00	1.81E-02	-1.45E+00	2.58E-03	-1.04E+00	4.45E-02	7.73E-01	5.13E-01
ENSBTAG00000037358	0.00E+00	1.00E+00	0.00E+00	1.00E+00	-5.13E-15	1.00E+00	0.00E+00	1.00E+00	0.00E+00	1.00E+00

ENSBTAG00000038962	1.03E-01	1.00E+00	-1.10E+00	2.97E-06	-1.98E+00	2.27E-17	-7.93E-01	1.29E-03	1.05E+00	3.12E-04
ENSBTAG00000045463	0.00E+00	1.00E+00	0.00E+00	1.00E+00	-5.13E-15	1.00E+00	0.00E+00	1.00E+00	0.00E+00	1.00E+00
ENSBTAG00000005857	-1.91E+00	1.52E-01	-7.18E-01	4.84E-01	-9.83E-01	2.70E-01	-1.80E+00	3.95E-02	-1.61E+00	3.34E-01
ENSBTAG00000001447	1.19E-02	1.00E+00	2.08E+00	1.94E-01	-1.30E-01	1.00E+00	6.26E-01	9.94E-01	7.16E-03	1.00E+00
ENSBTAG00000035827	-4.37E-03	1.00E+00	-4.05E-02	1.00E+00	5.83E-02	1.00E+00	1.81E-01	6.15E-01	1.44E-01	1.00E+00
ENSBTAG00000031065	0.00E+00	1.00E+00	0.00E+00	1.00E+00	-5.13E-15	1.00E+00	0.00E+00	1.00E+00	0.00E+00	1.00E+00
ENSBTAG00000018600	-3.97E-01	6.03E-01	-5.17E-01	1.01E-01	-6.80E-01	2.03E-02	-3.69E-01	2.99E-01	3.75E-01	6.52E-01
ENSBTAG00000003328	3.55E-02	1.00E+00	-3.12E-01	4.27E-01	-3.16E-01	4.14E-01	-4.20E-01	2.24E-01	-6.56E-02	1.00E+00
ENSBTAG00000005306	4.35E+00	4.85E-02	1.98E+00	4.43E-01	3.43E+00	7.35E-02	2.53E+00	2.71E-01	2.72E+00	6.01E-01
ENSBTAG00000044332	-2.70E+00	5.76E-01	-7.16E-01	9.45E-01	-2.70E+00	1.99E-01	-2.70E+00	2.05E-01	-7.85E-01	1.00E+00
ENSBTAG00000043073	0.00E+00	1.00E+00	1.98E+00	4.43E-01	1.78E+00	4.79E-01	0.00E+00	1.00E+00	0.00E+00	1.00E+00
ENSBTAG00000045027	0.00E+00	1.00E+00	0.00E+00	1.00E+00	-5.13E-15	1.00E+00	0.00E+00	1.00E+00	0.00E+00	1.00E+00
ENSBTAG00000006614	5.60E-01	1.70E-01	-9.48E-01	1.69E-04	-1.43E+00	5.98E-09	-1.30E+00	1.81E-07	-2.16E-01	9.20E-01
ENSBTAG00000043932	0.00E+00	1.00E+00	0.00E+00	1.00E+00	-5.13E-15	1.00E+00	0.00E+00	1.00E+00	0.00E+00	1.00E+00
ENSBTAG00000001814	-3.51E-01	7.43E-01	-2.03E+00	9.80E-14	-1.37E+00	4.52E-07	-1.27E+00	4.57E-06	-2.67E-01	8.54E-01
ENSBTAG00000001812	-3.44E-01	1.00E+00	-8.92E-01	3.36E-01	-2.52E+00	2.91E-03	-1.80E+00	2.87E-02	-3.87E-01	1.00E+00
ENSBTAG00000001310	1.92E+00	8.23E-01	0.00E+00	1.00E+00	3.05E+00	1.37E-01	0.00E+00	1.00E+00	2.71E+00	6.01E-01
ENSBTAG00000019121	-7.23E-02	1.00E+00	-1.83E+00	2.29E-12	-1.31E+00	1.99E-07	-4.02E-01	1.90E-01	3.32E-01	6.93E-01
ENSBTAG00000021645	-3.81E-01	6.49E-01	-7.84E-01	7.27E-03	1.41E-01	8.87E-01	1.62E-02	1.00E+00	4.33E-02	1.00E+00
ENSBTAG00000021643	5.18E-01	1.00E+00	-7.14E-01	9.45E-01	1.82E+00	1.42E-01	6.94E-01	8.56E-01	-2.70E+00	6.01E-01
ENSBTAG00000015283	7.68E-02	1.00E+00	2.97E-01	3.29E-01	3.78E-02	1.00E+00	6.37E-02	1.00E+00	-2.64E-01	8.00E-01
ENSBTAG00000042863	0.00E+00	1.00E+00	0.00E+00	1.00E+00	-5.13E-15	1.00E+00	0.00E+00	1.00E+00	0.00E+00	1.00E+00
ENSBTAG00000015273	3.21E-01	9.89E-01	1.26E+00	6.80E-04	2.41E-01	8.01E-01	2.11E-01	8.79E-01	-2.15E-01	1.00E+00
ENSBTAG00000000161	-1.38E+00	5.76E-01	-3.59E-01	9.55E-01	-1.02E+00	3.63E-01	5.50E-02	1.00E+00	-1.37E+00	6.01E-01
ENSBTAG00000004514	9.81E-02	1.00E+00	3.25E-01	1.24E-01	2.36E-01	3.21E-01	1.00E-01	8.72E-01	2.87E-01	5.81E-01
ENSBTAG00000045748	0.00E+00	1.00E+00	0.00E+00	1.00E+00	-5.13E-15	1.00E+00	0.00E+00	1.00E+00	0.00E+00	1.00E+00
ENSBTAG00000004512	6.43E-02	1.00E+00	1.37E+00	1.91E-05	1.18E+00	2.63E-04	8.46E-01	1.45E-02	3.33E-01	8.22E-01
ENSBTAG00000024991	-3.59E-01	8.32E-01	-1.73E+00	1.38E-06	-1.41E+00	6.58E-05	-4.88E-01	2.71E-01	-1.46E-01	1.00E+00
ENSBTAG00000037980	1.75E-01	1.00E+00	-5.01E-01	1.12E-01	6.19E-01	3.09E-02	7.33E-01	9.46E-03	2.16E-01	9.66E-01
ENSBTAG00000001333	2.28E+00	2.05E-04	1.17E+00	5.03E-02	4.87E-01	5.74E-01	1.69E-01	1.00E+00	-2.95E-01	1.00E+00
ENSBTAG00000044928	0.00E+00	1.00E+00	0.00E+00	1.00E+00	-5.13E-15	1.00E+00	0.00E+00	1.00E+00	1.91E+00	8.00E-01
ENSBTAG00000020260	-2.12E-02	1.00E+00	-7.04E-01	2.36E-01	-1.72E-01	1.00E+00	2.47E-01	8.63E-01	-3.05E-01	1.00E+00
ENSBTAG00000020263	0.00E+00	1.00E+00	0.00E+00	1.00E+00	-5.13E-15	1.00E+00	0.00E+00	1.00E+00	0.00E+00	1.00E+00
ENSBTAG00000040416	1.92E+00	8.23E-01	0.00E+00	1.00E+00	-5.13E-15	1.00E+00	0.00E+00	1.00E+00	0.00E+00	1.00E+00
ENSBTAG00000037626	0.00E+00	1.00E+00	0.00E+00	1.00E+00	-5.13E-15	1.00E+00	0.00E+00	1.00E+00	0.00E+00	1.00E+00

ENSBTAG00000031115	3.61E-01	1.00E+00	1.75E-02	1.00E+00	-2.97E-01	8.11E-01	-5.74E-01	4.24E-01	3.89E-01	9.57E-01
ENSBTAG00000013725	-1.29E-01	1.00E+00	8.53E-01	5.36E-03	7.34E-01	1.91E-02	9.96E-01	1.00E-03	3.06E-01	8.17E-01
ENSBTAG00000013723	-7.94E-02	1.00E+00	1.21E-01	9.33E-01	1.17E-02	1.00E+00	2.24E-02	1.00E+00	-4.18E-01	5.16E-01
ENSBTAG00000013716	5.14E-01	5.47E-01	8.16E-01	1.90E-02	3.00E-01	5.48E-01	5.18E-01	1.98E-01	9.59E-01	5.53E-02
ENSBTAG00000006789	2.69E-01	8.75E-01	-1.93E-01	7.34E-01	-8.58E-02	1.00E+00	-2.90E-01	4.74E-01	-3.28E-01	7.97E-01
ENSBTAG00000040133	1.37E-01	1.00E+00	9.70E-01	1.36E-01	2.72E+00	2.55E-08	5.69E-01	4.97E-01	-1.94E-01	1.00E+00
ENSBTAG00000038298	4.07E-01	3.37E-01	1.38E+00	1.34E-11	9.02E-01	1.63E-05	7.03E-01	1.36E-03	1.27E-01	1.00E+00
ENSBTAG00000011896	-3.67E-01	1.00E+00	2.69E-01	1.00E+00	6.30E-01	6.05E-01	3.86E-01	9.13E-01	1.19E+00	5.09E-01
ENSBTAG00000045870	0.00E+00	1.00E+00	0.00E+00	1.00E+00	-5.13E-15	1.00E+00	0.00E+00	1.00E+00	0.00E+00	1.00E+00
ENSBTAG00000011088	-6.93E-01	5.30E-01	-1.79E+00	2.13E-04	-1.11E-01	1.00E+00	3.89E-01	5.37E-01	4.85E-01	8.00E-01
ENSBTAG00000007363	-6.30E-02	1.00E+00	-5.66E-01	1.10E-02	7.67E-02	1.00E+00	1.97E-01	5.39E-01	-2.36E-01	8.00E-01
ENSBTAG00000007362	-1.75E-01	1.00E+00	-1.55E+00	1.07E-12	-2.50E+00	6.12E-30	-2.98E+00	1.88E-40	2.45E-01	8.00E-01
ENSBTAG00000028443	0.00E+00	1.00E+00	0.00E+00	1.00E+00	-5.13E-15	1.00E+00	0.00E+00	1.00E+00	0.00E+00	1.00E+00
ENSBTAG00000007360	-2.63E-01	7.67E-01	-8.43E-01	7.00E-05	-3.07E-01	2.44E-01	1.81E-01	6.08E-01	1.27E-01	1.00E+00
ENSBTAG00000015529	-2.45E-01	8.23E-01	6.42E-01	4.42E-03	1.61E+00	1.10E-14	1.04E+00	1.18E-06	-1.83E-01	9.46E-01
ENSBTAG00000023918	0.00E+00	1.00E+00	0.00E+00	1.00E+00	-5.13E-15	1.00E+00	0.00E+00	1.00E+00	0.00E+00	1.00E+00
ENSBTAG00000017173	0.00E+00	1.00E+00	1.99E+00	4.43E-01	-5.13E-15	1.00E+00	1.76E+00	4.97E-01	1.93E+00	8.00E-01
ENSBTAG00000046731	1.92E+00	8.23E-01	1.98E+00	4.43E-01	1.78E+00	4.79E-01	2.53E+00	2.71E-01	0.00E+00	1.00E+00
ENSBTAG00000001668	-2.04E-01	1.00E+00	-3.94E-01	7.92E-01	-2.19E-02	1.00E+00	-3.46E-01	8.39E-01	-2.37E-01	1.00E+00
ENSBTAG00000004014	-4.05E-01	1.00E+00	-7.36E-01	3.79E-01	-1.53E+00	2.12E-02	-1.41E+00	4.05E-02	6.16E-01	8.27E-01
ENSBTAG00000007208	-5.74E-01	3.10E-01	-1.39E+00	2.25E-06	-2.46E+00	7.32E-17	-2.69E+00	1.68E-19	-2.15E-01	1.00E+00
ENSBTAG00000003665	-2.79E-01	7.41E-01	-2.61E+00	3.00E-32	-3.06E+00	8.20E-43	-2.85E+00	6.36E-38	3.85E-01	4.55E-01
ENSBTAG00000003237	-3.45E-01	5.78E-01	-1.55E+00	1.58E-11	-2.39E+00	8.08E-25	-1.54E+00	1.78E-11	2.62E-01	8.00E-01
ENSBTAG00000003242	-2.40E-01	8.23E-01	-1.13E+00	2.36E-07	-9.66E-01	1.05E-05	-1.04E+00	2.62E-06	-2.17E-02	1.00E+00
ENSBTAG00000003244	-4.62E-01	5.76E-01	-2.24E+00	1.83E-11	-2.26E+00	2.73E-12	-2.11E+00	6.57E-11	2.48E-01	9.37E-01
ENSBTAG00000047509	4.80E-01	1.00E+00	-8.41E-01	5.17E-01	-3.10E-02	1.00E+00	-8.83E-01	4.72E-01	4.08E-01	1.00E+00
ENSBTAG00000005922	-7.42E-01	9.82E-02	-3.20E+00	2.67E-21	-1.17E+00	1.01E-04	-1.38E+00	5.35E-06	-3.74E-01	7.55E-01
ENSBTAG00000014666	1.21E-02	1.00E+00	4.32E+00	6.17E-06	1.52E+00	4.28E-01	1.78E+00	3.04E-01	1.68E+00	7.78E-01
ENSBTAG00000030915	1.74E-02	1.00E+00	-5.29E-01	1.15E-02	2.64E-01	2.97E-01	-9.47E-02	9.35E-01	1.86E-01	8.78E-01
ENSBTAG00000012159	-1.21E-03	1.00E+00	2.59E-01	2.61E-01	1.13E-01	8.06E-01	-3.50E-01	1.01E-01	-6.30E-02	1.00E+00
ENSBTAG00000013212	2.12E-01	9.30E-01	7.54E-01	1.45E-03	7.75E-01	9.97E-04	8.03E-01	7.54E-04	3.13E-01	6.91E-01
ENSBTAG00000044986	1.92E+00	8.23E-01	0.00E+00	1.00E+00	-5.13E-15	1.00E+00	1.76E+00	4.97E-01	0.00E+00	1.00E+00
ENSBTAG00000030930	-4.18E-01	2.51E-01	-1.07E+00	1.66E-07	-1.09E+00	6.89E-08	-5.09E-01	2.14E-02	5.99E-02	1.00E+00
ENSBTAG00000040116	-7.18E-01	1.74E-01	-1.93E+00	1.54E-09	-2.48E+00	1.13E-14	-2.19E+00	1.04E-11	-1.12E-01	1.00E+00
ENSBTAG00000010193	-8.51E-02	1.00E+00	3.04E-01	1.80E-01	2.25E-01	3.82E-01	2.65E-01	2.73E-01	2.25E-02	1.00E+00

ENSBTAG00000030941	4.64E-01	1.00E+00	1.18E-01	1.00E+00	-1.51E-01	1.00E+00	-9.30E-02	1.00E+00	-8.83E-01	7.63E-01
ENSBTAG00000005191	4.31E-01	5.22E-01	1.18E+00	1.09E-05	1.33E+00	4.74E-07	1.46E+00	4.11E-08	9.53E-01	9.89E-03
ENSBTAG00000019707	-1.17E+00	3.53E-02	-1.34E+00	1.52E-03	4.81E-01	3.28E-01	-2.10E-01	8.74E-01	-5.71E-01	6.22E-01
ENSBTAG00000006179	0.00E+00	1.00E+00	0.00E+00	1.00E+00	-5.13E-15	1.00E+00	1.76E+00	4.97E-01	0.00E+00	1.00E+00
ENSBTAG00000030962	-2.82E-01	7.22E-01	-4.20E-01	8.77E-02	-4.85E-01	4.02E-02	-4.20E-01	9.37E-02	-4.35E-01	3.49E-01
ENSBTAG00000020998	-2.82E-03	1.00E+00	9.53E-01	3.40E-05	1.27E+00	1.46E-08	1.31E+00	6.37E-09	-2.43E-01	8.07E-01
ENSBTAG00000004937	7.51E-01	1.26E-01	1.95E+00	3.54E-10	2.25E+00	3.61E-13	2.43E+00	7.10E-15	1.27E+00	1.51E-03
ENSBTAG00000011547	-1.81E+00	2.22E-01	8.34E-01	2.87E-01	-1.98E+00	2.90E-02	-6.36E-01	5.69E-01	-8.85E-01	8.00E-01
ENSBTAG00000018248	-1.36E-01	1.00E+00	-3.13E-01	6.22E-01	-7.08E-01	1.00E-01	-9.95E-01	1.45E-02	7.15E-02	1.00E+00
ENSBTAG00000018238	-9.01E-02	1.00E+00	-5.03E-01	4.47E-02	-1.05E+00	3.09E-06	-1.74E+00	8.57E-15	-2.23E-01	8.52E-01
ENSBTAG00000018031	-1.30E-01	1.00E+00	-3.87E-01	4.10E-01	-1.08E+00	2.30E-03	-1.35E+00	1.94E-04	2.25E-01	1.00E+00
ENSBTAG00000014380	-5.20E-01	6.69E-01	-1.19E+00	2.99E-03	-3.75E-01	4.93E-01	1.53E-01	9.96E-01	-3.54E-01	8.85E-01
ENSBTAG00000022575	-2.00E-01	8.71E-01	-2.82E-01	2.69E-01	2.08E-02	1.00E+00	-1.36E-01	7.61E-01	-1.12E-02	1.00E+00
ENSBTAG00000043698	0.00E+00	1.00E+00	0.00E+00	1.00E+00	-5.13E-15	1.00E+00	0.00E+00	1.00E+00	0.00E+00	1.00E+00
ENSBTAG00000009159	3.38E-01	7.74E-01	1.14E+00	2.77E-05	1.02E+00	1.91E-04	1.67E+00	3.24E-10	2.21E-01	9.69E-01
ENSBTAG00000030855	1.94E-02	1.00E+00	-7.14E-01	1.04E-01	-1.04E+00	1.01E-02	-1.05E+00	1.00E-02	7.56E-02	1.00E+00
ENSBTAG00000011795	-8.61E-02	1.00E+00	-9.48E-01	1.06E-04	-9.97E-01	3.83E-05	-8.38E-01	8.12E-04	2.83E-01	8.00E-01
ENSBTAG00000039194	-2.88E-01	1.00E+00	-1.21E+00	2.91E-02	-1.23E+00	2.01E-02	-2.06E+00	1.90E-04	-1.33E-02	1.00E+00
ENSBTAG00000008778	-4.97E-01	5.88E-01	-2.60E+00	2.66E-14	-4.19E+00	4.47E-31	-2.32E+00	1.06E-11	2.13E-01	1.00E+00
ENSBTAG00000008774	-3.11E-01	9.46E-01	-1.43E+00	5.88E-05	-2.90E+00	5.60E-16	-3.22E+00	7.00E-19	1.83E-01	1.00E+00
ENSBTAG00000009874	-5.44E-01	1.29E-01	2.12E-01	5.37E-01	4.94E-01	4.45E-02	2.30E-01	4.97E-01	-6.26E-02	1.00E+00
ENSBTAG00000005617	1.79E-02	1.00E+00	-4.60E-01	2.81E-01	-4.39E-01	2.96E-01	-5.99E-01	1.24E-01	2.02E-01	1.00E+00
ENSBTAG00000014470	-4.17E-01	3.19E-01	-1.51E+00	7.65E-13	-2.86E+00	2.54E-40	-3.10E+00	4.83E-46	7.52E-02	1.00E+00
ENSBTAG00000008313	-1.39E-01	1.00E+00	-2.64E+00	1.72E-15	-5.73E-01	1.36E-01	-2.65E-01	6.50E-01	7.27E-01	2.57E-01
ENSBTAG00000019402	3.05E-01	9.96E-01	-9.90E-01	2.06E-02	-2.17E+00	2.18E-07	-2.60E+00	2.27E-09	1.26E+00	1.27E-02
ENSBTAG00000046534	0.00E+00	1.00E+00	0.00E+00	1.00E+00	-5.13E-15	1.00E+00	0.00E+00	1.00E+00	0.00E+00	1.00E+00
ENSBTAG00000043990	-1.33E-01	1.00E+00	-1.88E-01	8.07E-01	-1.90E-01	7.94E-01	1.75E-01	8.45E-01	-4.77E-01	5.85E-01
ENSBTAG00000007937	-5.49E-02	1.00E+00	4.59E-01	1.16E-01	8.05E-01	1.60E-03	5.12E-01	7.48E-02	-2.00E-01	9.85E-01
ENSBTAG00000047720	0.00E+00	1.00E+00	0.00E+00	1.00E+00	-5.13E-15	1.00E+00	0.00E+00	1.00E+00	0.00E+00	1.00E+00
ENSBTAG00000047911	0.00E+00	1.00E+00	0.00E+00	1.00E+00	-5.13E-15	1.00E+00	0.00E+00	1.00E+00	0.00E+00	1.00E+00
ENSBTAG00000005362	-7.39E-02	1.00E+00	4.08E-01	4.37E-01	9.81E-01	9.06E-03	1.06E+00	4.80E-03	3.60E-01	8.45E-01
ENSBTAG00000005355	-4.85E-02	1.00E+00	1.13E+00	7.44E-07	5.87E-01	1.79E-02	2.54E-01	4.55E-01	-3.14E-01	7.09E-01
ENSBTAG00000018879	1.96E-01	9.91E-01	1.50E+00	5.44E-11	1.50E+00	3.28E-11	1.05E+00	7.10E-06	2.77E-01	8.00E-01
ENSBTAG00000008228	-3.37E-01	1.00E+00	-7.09E-01	6.46E-01	-7.30E-01	5.93E-01	-1.92E-01	1.00E+00	-1.97E+00	4.20E-01
ENSBTAG00000043807	0.00E+00	1.00E+00	0.00E+00	1.00E+00	-5.13E-15	1.00E+00	0.00E+00	1.00E+00	0.00E+00	1.00E+00

ENSBTAG00000045681	0.00E+00	1.00E+00	0.00E+00	1.00E+00	-5.13E-15	1.00E+00	0.00E+00	1.00E+00	0.00E+00	1.00E+00
ENSBTAG00000045698	0.00E+00	1.00E+00	0.00E+00	1.00E+00	-5.13E-15	1.00E+00	0.00E+00	1.00E+00	0.00E+00	1.00E+00
ENSBTAG00000048128	0.00E+00	1.00E+00	0.00E+00	1.00E+00	-5.13E-15	1.00E+00	0.00E+00	1.00E+00	0.00E+00	1.00E+00
ENSBTAG00000021237	8.08E-02	1.00E+00	-6.56E-01	5.64E-02	-1.02E+00	1.37E-03	-1.09E+00	6.63E-04	5.19E-01	5.13E-01
ENSBTAG00000033515	-2.60E-02	1.00E+00	6.62E-02	1.00E+00	-8.36E-01	1.92E-01	-1.90E-01	1.00E+00	5.03E-02	1.00E+00
ENSBTAG00000046193	-4.72E-01	1.00E+00	-8.68E-01	3.30E-01	-1.55E+00	4.10E-02	-1.98E+00	8.92E-03	-5.32E-01	9.69E-01
ENSBTAG00000012870	1.53E-01	1.00E+00	2.40E-01	9.14E-01	9.26E-02	1.00E+00	5.05E-01	4.57E-01	-2.60E-01	1.00E+00
ENSBTAG00000025718	-9.22E-02	1.00E+00	5.15E-01	6.83E-01	9.89E-01	1.89E-01	1.35E+00	4.35E-02	-5.68E-01	1.00E+00
ENSBTAG00000012973	7.97E-01	1.00E+00	7.78E-02	1.00E+00	-1.91E+00	4.28E-01	-1.91E+00	4.39E-01	7.04E-03	1.00E+00
ENSBTAG00000011719	9.58E-01	8.23E-01	4.93E-01	8.88E-01	5.44E-02	1.00E+00	1.21E+00	2.51E-01	4.03E-01	1.00E+00
ENSBTAG00000016058	-2.82E-01	1.00E+00	2.05E-01	1.00E+00	-3.87E-01	9.03E-01	4.45E-01	8.05E-01	-2.90E-01	1.00E+00
ENSBTAG00000044495	0.00E+00	1.00E+00	0.00E+00	1.00E+00	-5.13E-15	1.00E+00	0.00E+00	1.00E+00	0.00E+00	1.00E+00
ENSBTAG00000012652	-7.45E-01	2.37E-01	-8.00E-01	4.32E-02	-5.71E-01	1.73E-01	-7.13E-01	7.78E-02	-1.09E+00	5.43E-02
ENSBTAG00000043056	0.00E+00	1.00E+00	0.00E+00	1.00E+00	-5.13E-15	1.00E+00	0.00E+00	1.00E+00	0.00E+00	1.00E+00
ENSBTAG00000014581	8.11E-02	1.00E+00	-4.18E-01	2.76E-01	-1.77E+00	7.43E-09	-1.09E+00	4.96E-04	6.65E-01	2.42E-01
ENSBTAG00000017533	1.34E-01	1.00E+00	-8.89E-01	1.19E-01	-4.56E-01	4.96E-01	2.18E-01	9.21E-01	3.15E-02	1.00E+00
ENSBTAG00000018167	-1.41E-02	1.00E+00	-7.78E-01	5.58E-01	-1.07E+00	3.51E-01	-1.18E+00	2.91E-01	-4.18E-02	1.00E+00
ENSBTAG00000015571	-1.94E+00	1.94E-03	-2.67E+00	4.98E-07	-1.47E+00	6.56E-03	-1.36E+00	1.48E-02	-6.04E-01	8.00E-01
ENSBTAG00000015565	1.32E+00	9.30E-01	1.77E+00	3.27E-01	6.44E-01	9.78E-01	1.12E+00	6.70E-01	1.97E+00	6.01E-01
ENSBTAG00000002581	-1.17E+00	8.74E-01	-1.09E+00	5.52E-01	-1.33E+00	4.18E-01	-2.12E+00	1.80E-01	-3.88E+00	2.09E-01
ENSBTAG00000045923	-3.22E+00	3.75E-01	-1.24E+00	6.08E-01	-1.44E+00	4.79E-01	-1.46E+00	4.92E-01	-1.29E+00	9.02E-01
ENSBTAG00000047707	-3.54E-01	9.96E-01	-9.16E-01	8.38E-02	-5.29E-01	3.67E-01	-1.78E+00	5.10E-04	-6.04E-01	6.91E-01
ENSBTAG00000003038	1.03E-02	1.00E+00	2.11E-01	6.37E-01	3.15E-01	3.68E-01	1.36E-01	8.95E-01	3.48E-02	1.00E+00
ENSBTAG00000003037	0.00E+00	1.00E+00	0.00E+00	1.00E+00	-5.13E-15	1.00E+00	0.00E+00	1.00E+00	0.00E+00	1.00E+00
ENSBTAG00000003039	-2.02E-01	1.00E+00	-1.59E-01	1.00E+00	-6.53E-02	1.00E+00	-2.21E-01	9.39E-01	-7.14E-01	6.01E-01
ENSBTAG00000008953	1.01E+00	1.65E-01	2.05E+00	5.01E-06	8.82E-01	7.69E-02	-2.04E-01	9.50E-01	-3.39E-01	9.93E-01
ENSBTAG00000008954	2.53E-01	1.00E+00	1.58E+00	5.03E-03	1.14E+00	5.72E-02	4.12E-01	6.80E-01	-5.32E-01	8.42E-01
ENSBTAG00000034582	0.00E+00	1.00E+00	0.00E+00	1.00E+00	1.78E+00	4.79E-01	0.00E+00	1.00E+00	0.00E+00	1.00E+00
ENSBTAG00000012451	-3.03E-01	9.31E-01	-1.62E+00	1.39E-06	-1.34E+00	6.66E-05	-1.31E+00	1.21E-04	-3.70E-01	8.00E-01
ENSBTAG00000015730	-2.35E-01	1.00E+00	-1.87E+00	3.67E-09	-1.62E+00	2.15E-07	-1.38E+00	1.19E-05	-2.81E-01	8.99E-01
ENSBTAG00000006490	-2.22E-01	1.00E+00	-1.48E+00	1.32E-02	-1.48E+00	1.16E-02	-1.72E+00	3.87E-03	-2.08E-01	1.00E+00
ENSBTAG00000011074	5.19E-01	2.51E-02	1.51E+00	2.00E-19	1.03E+00	1.07E-09	5.54E-01	2.21E-03	1.08E-01	1.00E+00
ENSBTAG00000000601	-3.58E-01	9.65E-01	1.60E+00	3.85E-05	-1.06E-02	1.00E+00	-1.51E+00	1.01E-03	1.84E-01	1.00E+00
ENSBTAG00000000602	-3.83E-03	1.00E+00	5.45E-01	1.12E-02	2.40E-01	3.96E-01	3.24E-02	1.00E+00	-3.18E-02	1.00E+00
ENSBTAG00000008191	2.90E-01	8.23E-01	7.66E-01	4.90E-03	1.14E+00	1.13E-05	1.51E+00	5.78E-09	1.02E-01	1.00E+00

ENSBTAG00000020927	-2.84E-01	8.47E-01	-6.78E-01	2.24E-02	-1.18E+00	1.96E-05	-1.39E+00	5.37E-07	-2.78E-01	8.33E-01
ENSBTAG00000046881	5.24E-03	1.00E+00	8.75E-01	8.53E-01	6.44E-01	9.78E-01	6.21E-01	9.94E-01	-1.91E+00	8.00E-01
ENSBTAG00000018884	5.80E-02	1.00E+00	2.42E-01	3.29E-01	3.07E-01	1.70E-01	2.53E-01	3.04E-01	-1.37E-01	1.00E+00
ENSBTAG00000002643	1.44E-01	1.00E+00	6.38E-01	1.21E-03	9.06E-01	1.71E-06	6.20E-01	2.01E-03	1.11E-01	1.00E+00
ENSBTAG00000002648	1.51E-02	1.00E+00	3.56E-01	2.92E-01	3.56E-01	2.90E-01	3.98E-01	2.29E-01	-1.01E-01	1.00E+00
ENSBTAG00000025669	-4.09E-01	4.33E-01	-1.33E+00	3.67E-08	-2.74E+00	3.30E-27	-2.56E+00	1.77E-24	-1.26E-01	1.00E+00
ENSBTAG00000016824	-1.19E-01	1.00E+00	7.43E-01	4.18E-03	1.35E+00	3.23E-08	1.25E+00	4.85E-07	-2.60E-01	8.17E-01
ENSBTAG00000010723	-3.97E-02	1.00E+00	3.61E-01	2.29E-01	3.34E-01	2.78E-01	4.48E-01	1.15E-01	-4.16E-01	4.96E-01
ENSBTAG00000016827	-2.61E-01	8.23E-01	-9.53E-01	1.31E-04	-1.10E+00	8.08E-06	-1.01E+00	5.30E-05	-6.66E-02	1.00E+00
ENSBTAG00000016828	-8.51E-03	1.00E+00	3.71E-01	2.48E-01	2.33E-01	5.62E-01	4.59E-02	1.00E+00	8.85E-02	1.00E+00
ENSBTAG00000045337	0.00E+00	1.00E+00	0.00E+00	1.00E+00	-5.13E-15	1.00E+00	0.00E+00	1.00E+00	0.00E+00	1.00E+00
ENSBTAG00000016829	-4.44E-01	5.78E-01	-1.44E+00	8.10E-06	-1.47E+00	2.41E-06	-4.41E-01	2.39E-01	-9.36E-02	1.00E+00
ENSBTAG00000016830	-3.02E-01	5.76E-01	7.31E-01	2.42E-04	7.13E-01	3.38E-04	2.46E-01	3.68E-01	-1.01E-01	1.00E+00
ENSBTAG00000001631	-1.17E-01	1.00E+00	-4.70E-01	3.48E-01	-7.13E-01	9.35E-02	-9.44E-01	2.18E-02	-6.66E-01	4.58E-01
ENSBTAG00000001634	2.83E-02	1.00E+00	-5.44E-01	3.56E-02	-6.32E-01	1.17E-02	-4.04E-01	1.58E-01	-1.81E-01	9.95E-01
ENSBTAG00000001635	-4.45E-01	3.59E-01	-9.31E-01	1.25E-04	-4.03E-01	1.52E-01	-2.71E-01	4.33E-01	-5.71E-02	1.00E+00
ENSBTAG00000020975	-3.30E-03	1.00E+00	-4.10E-01	2.91E-01	-7.25E-01	2.59E-02	-1.39E+00	8.29E-06	5.00E-02	1.00E+00
ENSBTAG00000038888	-1.74E+00	5.52E-09	-2.38E+00	8.19E-17	-1.37E+00	9.74E-07	-1.03E+00	3.40E-04	5.36E-01	3.96E-01
ENSBTAG00000000428	-3.55E-01	8.88E-01	3.66E-02	1.00E+00	9.75E-01	1.11E-02	1.08E+00	5.26E-03	-2.11E-02	1.00E+00
ENSBTAG00000011057	8.07E-01	9.32E-01	5.55E-01	8.34E-01	2.94E-01	1.00E+00	-2.12E+00	1.79E-01	1.35E+00	5.46E-01
ENSBTAG00000013245	-4.44E-01	4.60E-01	-1.71E+00	1.22E-10	-2.07E+00	5.99E-15	-1.45E+00	4.01E-08	-2.33E-01	9.11E-01
ENSBTAG00000033603	-3.35E-01	8.23E-01	-6.86E-01	3.73E-02	-1.25E+00	3.85E-05	-1.27E+00	3.26E-05	-3.82E-01	7.58E-01
ENSBTAG00000015538	-2.18E-01	8.77E-01	-1.74E-01	6.62E-01	-7.03E-03	1.00E+00	-1.92E-01	5.99E-01	3.90E-02	1.00E+00
ENSBTAG00000015550	-3.70E-01	1.00E+00	2.94E+00	4.21E-05	3.73E-01	1.00E+00	5.54E-01	8.49E-01	1.11E+00	7.87E-01
ENSBTAG00000013632	-2.83E-01	1.00E+00	1.14E+00	1.11E-01	3.07E-01	9.39E-01	7.45E-01	4.02E-01	-5.07E-01	1.00E+00
ENSBTAG00000034529	-1.78E-01	1.00E+00	-5.25E-01	3.69E-01	3.67E-01	5.95E-01	9.38E-01	5.08E-02	-2.59E-01	1.00E+00
ENSBTAG00000034519	-4.90E-01	8.69E-01	1.87E-01	1.00E+00	3.08E-01	7.92E-01	-2.67E-01	8.78E-01	-3.73E-01	9.97E-01
ENSBTAG00000019684	1.13E-01	1.00E+00	-2.28E-01	4.69E-01	-1.87E-01	5.97E-01	2.47E-02	1.00E+00	7.26E-02	1.00E+00
ENSBTAG00000044261	0.00E+00	1.00E+00	0.00E+00	1.00E+00	-5.13E-15	1.00E+00	0.00E+00	1.00E+00	0.00E+00	1.00E+00
ENSBTAG00000015598	-9.66E-02	1.00E+00	1.34E-01	8.50E-01	2.19E-01	5.26E-01	2.00E-01	6.01E-01	2.71E-01	8.00E-01
ENSBTAG00000016124	3.45E-01	1.00E+00	2.03E-02	1.00E+00	-8.26E-01	2.99E-01	1.21E-01	1.00E+00	-7.13E-02	1.00E+00
ENSBTAG00000021993	1.51E-01	1.00E+00	-4.83E-02	1.00E+00	-4.49E-02	1.00E+00	1.53E-01	1.00E+00	6.98E-01	8.35E-01
ENSBTAG00000034493	-2.49E-01	8.23E-01	-8.92E-01	4.43E-05	-1.22E+00	9.95E-09	-7.74E-01	5.45E-04	4.78E-01	2.61E-01
ENSBTAG00000029474	0.00E+00	1.00E+00	0.00E+00	1.00E+00	-5.13E-15	1.00E+00	0.00E+00	1.00E+00	0.00E+00	1.00E+00
ENSBTAG00000024549	-6.86E-02	1.00E+00	7.16E-01	1.28E-04	6.47E-01	6.02E-04	1.43E-01	6.88E-01	-2.35E-01	7.71E-01

ENSBTAG00000044892	5.19E-03	1.00E+00	-1.91E+00	4.69E-01	-1.91E+00	4.28E-01	-1.91E+00	4.39E-01	7.93E-01	1.00E+00
ENSBTAG00000044323	0.00E+00	1.00E+00	0.00E+00	1.00E+00	-5.13E-15	1.00E+00	0.00E+00	1.00E+00	0.00E+00	1.00E+00
ENSBTAG00000009267	-6.38E-01	1.84E-01	-2.35E+00	1.08E-15	-1.86E+00	7.73E-11	-8.48E-01	5.24E-03	4.16E-01	6.14E-01
ENSBTAG00000016887	1.87E-01	1.00E+00	6.79E-01	6.09E-03	7.50E-01	2.00E-03	3.89E-01	1.80E-01	1.45E-01	1.00E+00
ENSBTAG00000016890	2.81E-02	1.00E+00	5.17E-01	4.39E-02	7.26E-01	2.42E-03	5.37E-01	3.85E-02	3.01E-01	7.44E-01
ENSBTAG00000016891	-2.25E-01	1.00E+00	-5.86E-01	4.75E-01	-2.22E+00	1.44E-03	-2.49E+00	6.03E-04	4.45E-01	9.08E-01
ENSBTAG00000009226	2.05E-01	1.00E+00	-1.52E+00	2.57E-03	-2.34E-01	8.46E-01	-6.04E-02	1.00E+00	6.26E-01	5.59E-01
ENSBTAG00000006170	-1.10E-01	1.00E+00	-1.15E+00	2.35E-09	-7.55E-01	1.01E-04	-5.09E-01	1.47E-02	2.05E-01	8.00E-01
ENSBTAG00000025622	-4.41E-01	5.56E-01	-9.54E-01	1.33E-03	-1.09E-01	1.00E+00	-6.85E-01	2.71E-02	-2.67E-01	8.71E-01
ENSBTAG00000017542	4.78E-01	3.88E-01	2.60E+00	6.48E-24	6.27E-01	2.84E-02	8.94E-01	1.06E-03	-1.76E-01	1.00E+00
ENSBTAG00000019492	-9.83E-01	8.59E-03	-1.25E+00	3.65E-05	-7.07E-02	1.00E+00	-4.63E-01	2.00E-01	-3.38E-01	8.00E-01
ENSBTAG00000019494	-1.12E-01	1.00E+00	4.73E-01	7.84E-02	7.53E-01	2.02E-03	3.35E-01	2.76E-01	-6.58E-01	8.62E-02
ENSBTAG00000019495	1.35E+00	1.03E-09	2.27E+00	1.37E-27	1.45E+00	2.80E-12	8.88E-01	3.81E-05	3.63E-01	5.06E-01
ENSBTAG00000034287	5.53E-01	7.84E-01	1.06E+00	2.60E-02	1.45E+00	6.45E-04	1.11E+00	1.68E-02	7.12E-01	5.81E-01
ENSBTAG00000038094	0.00E+00	1.00E+00	0.00E+00	1.00E+00	-5.13E-15	1.00E+00	0.00E+00	1.00E+00	0.00E+00	1.00E+00
ENSBTAG00000047502	2.93E+00	2.98E-10	4.82E+00	1.21E-24	3.23E+00	2.87E-13	2.83E+00	1.60E-10	1.10E+00	1.43E-01
ENSBTAG00000010877	5.91E-01	6.27E-01	1.97E+00	6.27E-08	1.45E+00	1.49E-04	5.70E-01	2.84E-01	4.26E-01	8.33E-01
ENSBTAG00000016833	-1.91E+00	8.23E-01	7.64E-02	1.00E+00	-1.91E+00	4.28E-01	-1.91E+00	4.39E-01	-1.91E+00	8.00E-01
ENSBTAG00000016834	5.19E-03	1.00E+00	8.75E-01	8.53E-01	-1.91E+00	4.28E-01	-1.91E+00	4.39E-01	-1.91E+00	8.00E-01
ENSBTAG00000016838	2.70E-01	9.45E-01	1.29E+00	2.45E-05	1.33E+00	1.29E-05	1.12E+00	3.70E-04	-4.05E-01	7.18E-01
ENSBTAG00000046180	0.00E+00	1.00E+00	0.00E+00	1.00E+00	-5.13E-15	1.00E+00	0.00E+00	1.00E+00	0.00E+00	1.00E+00
ENSBTAG00000017251	7.56E-01	4.87E-01	2.00E+00	1.74E-05	6.93E-01	2.19E-01	-7.44E-01	1.93E-01	8.07E-01	4.66E-01
ENSBTAG00000043084	0.00E+00	1.00E+00	0.00E+00	1.00E+00	-5.13E-15	1.00E+00	0.00E+00	1.00E+00	0.00E+00	1.00E+00
ENSBTAG00000020783	1.33E+00	2.57E-12	2.32E+00	1.08E-36	1.46E+00	4.66E-16	2.07E-01	4.46E-01	-3.44E-03	1.00E+00
ENSBTAG00000010007	5.41E-01	8.41E-01	-1.36E-01	1.00E+00	-1.04E+00	1.24E-01	-1.43E-01	1.00E+00	1.49E+00	5.22E-02
ENSBTAG00000013316	3.91E-01	6.62E-01	4.86E-02	1.00E+00	2.53E-01	5.72E-01	1.02E+00	4.44E-04	9.44E-01	1.86E-02
ENSBTAG00000015118	3.38E-01	1.00E+00	1.70E+00	2.57E-02	9.06E-01	3.51E-01	5.25E-01	7.41E-01	-2.02E+00	3.47E-01
ENSBTAG00000017617	-1.91E+00	8.23E-01	8.74E-01	8.53E-01	6.39E-01	9.79E-01	1.78E+00	3.06E-01	2.15E-05	1.00E+00
ENSBTAG00000034879	3.93E-01	7.86E-01	2.85E-01	5.92E-01	8.10E-01	1.90E-02	1.56E+00	1.18E-06	-1.25E-01	1.00E+00
ENSBTAG00000020595	-2.26E-01	8.23E-01	-6.60E-01	1.23E-03	-2.61E-01	3.12E-01	1.65E-01	6.31E-01	-2.77E-01	6.64E-01
ENSBTAG00000011126	4.04E-01	4.03E-01	5.28E-01	3.31E-02	3.19E-01	2.68E-01	1.22E-01	8.87E-01	-3.15E-01	6.82E-01
ENSBTAG00000004219	0.00E+00	1.00E+00	0.00E+00	1.00E+00	-5.13E-15	1.00E+00	0.00E+00	1.00E+00	0.00E+00	1.00E+00
ENSBTAG00000040006	3.74E-01	2.45E-01	7.37E-01	4.21E-05	1.15E+00	3.32E-11	5.58E-01	3.20E-03	-6.09E-01	1.85E-02
ENSBTAG00000025617	3.41E-01	7.23E-01	2.09E+00	3.11E-16	1.85E+00	3.19E-13	1.40E+00	5.82E-08	-2.45E-01	8.87E-01
ENSBTAG00000008353	1.53E+00	1.28E-01	3.51E+00	1.88E-07	1.01E+00	1.89E-01	8.03E-01	3.49E-01	-1.65E-01	1.00E+00

ENSBTAG00000045551	3.73E-01	9.27E-01	2.42E-02	1.00E+00	-5.65E-01	3.21E-01	4.44E-01	4.39E-01	-7.23E-01	5.68E-01
ENSBTAG00000043057	0.00E+00	1.00E+00	0.00E+00	1.00E+00	-5.13E-15	1.00E+00	0.00E+00	1.00E+00	0.00E+00	1.00E+00
ENSBTAG00000010728	5.63E-01	7.08E-01	4.04E-01	5.46E-01	-3.69E-01	6.36E-01	-3.51E-01	6.74E-01	1.46E-01	1.00E+00
ENSBTAG00000010730	1.92E+00	8.23E-01	2.79E+00	2.10E-01	1.78E+00	4.79E-01	2.53E+00	2.71E-01	0.00E+00	1.00E+00
ENSBTAG00000007514	1.89E+00	1.33E-04	3.67E+00	7.38E-20	1.61E+00	2.92E-04	9.68E-01	6.43E-02	-1.14E+00	3.80E-01
ENSBTAG00000002376	-2.18E-01	9.73E-01	-7.35E-01	6.89E-03	-2.44E-01	5.26E-01	-3.66E-02	1.00E+00	-2.88E-01	8.00E-01
ENSBTAG00000002393	-1.76E-01	9.95E-01	-1.14E+00	1.03E-07	-6.76E-01	2.37E-03	-6.06E-01	8.57E-03	2.34E-01	8.00E-01
ENSBTAG00000002092	-6.40E-01	8.82E-01	-9.63E-01	2.65E-01	-2.44E+00	2.43E-03	-1.53E+00	5.06E-02	9.58E-01	5.22E-01
ENSBTAG00000033453	-4.51E-02	1.00E+00	-4.02E-01	8.75E-02	-5.85E-01	6.81E-03	-4.19E-01	7.83E-02	-5.41E-02	1.00E+00
ENSBTAG00000014172	-5.81E-01	2.31E-01	1.94E-01	7.29E-01	-5.58E-01	7.09E-02	-6.87E-01	2.27E-02	1.76E-01	1.00E+00
ENSBTAG00000000396	3.51E+00	6.09E-50	4.46E+00	3.67E-75	3.62E+00	1.34E-53	3.06E+00	3.71E-40	1.04E+00	3.21E-04
ENSBTAG00000044331	1.92E+00	8.23E-01	2.79E+00	2.10E-01	2.56E+00	2.54E-01	3.03E+00	1.49E-01	0.00E+00	1.00E+00
ENSBTAG00000045936	-5.10E-01	1.00E+00	1.84E+00	8.27E-02	-1.59E-01	1.00E+00	-1.90E-01	1.00E+00	-1.31E+00	9.00E-01
ENSBTAG00000002369	0.00E+00	1.00E+00	0.00E+00	1.00E+00	-5.13E-15	1.00E+00	0.00E+00	1.00E+00	0.00E+00	1.00E+00
ENSBTAG00000044283	0.00E+00	1.00E+00	0.00E+00	1.00E+00	-5.13E-15	1.00E+00	0.00E+00	1.00E+00	0.00E+00	1.00E+00
ENSBTAG00000014253	3.51E-01	8.23E-01	4.96E-01	2.02E-01	5.14E-01	1.74E-01	4.71E-01	2.37E-01	1.84E-04	1.00E+00
ENSBTAG00000008321	1.89E-01	8.36E-01	5.65E-01	2.39E-03	4.64E-01	1.57E-02	2.18E-01	3.93E-01	2.68E-05	1.00E+00
ENSBTAG00000017091	-2.03E-01	9.96E-01	-1.88E-01	6.89E-01	-8.52E-02	1.00E+00	-9.29E-02	1.00E+00	3.71E-01	6.01E-01
ENSBTAG00000012069	-3.11E-02	1.00E+00	-1.06E-01	1.00E+00	-9.20E-01	3.04E-02	-8.43E-01	5.68E-02	-8.28E-02	1.00E+00
ENSBTAG00000005888	-3.78E-03	1.00E+00	1.40E+00	9.08E-02	9.79E-01	3.14E-01	1.37E-01	1.00E+00	1.24E-02	1.00E+00
ENSBTAG00000000527	1.15E-01	1.00E+00	9.86E-01	2.02E-08	6.87E-01	1.35E-04	2.53E-01	2.78E-01	-9.02E-02	1.00E+00
ENSBTAG00000000529	-1.13E-01	1.00E+00	-4.95E-01	9.88E-02	-1.06E+00	5.47E-05	-1.02E+00	1.37E-04	1.66E-01	1.00E+00
ENSBTAG00000012703	-1.32E-01	1.00E+00	-7.79E-01	9.59E-04	-1.48E+00	5.31E-11	-1.34E+00	4.92E-09	-2.09E-01	9.00E-01
ENSBTAG00000014063	-2.91E-01	8.23E-01	-2.51E-01	5.31E-01	-3.60E-01	2.84E-01	-8.32E-02	1.00E+00	-6.49E-01	1.81E-01
ENSBTAG00000018224	-2.68E-01	1.00E+00	-1.95E-01	1.00E+00	-4.70E-01	8.03E-01	-3.50E-01	9.41E-01	-1.43E-01	1.00E+00
ENSBTAG00000001074	-1.79E-01	1.00E+00	-3.26E-01	4.43E-01	-2.39E-01	6.42E-01	-3.98E-01	3.11E-01	3.45E-01	8.00E-01
ENSBTAG00000046576	0.00E+00	1.00E+00	0.00E+00	1.00E+00	-5.13E-15	1.00E+00	0.00E+00	1.00E+00	0.00E+00	1.00E+00
ENSBTAG00000011112	-1.02E+00	1.70E-02	-1.10E+00	1.03E-03	-3.24E+00	6.24E-22	-2.16E+00	3.93E-11	4.38E-02	1.00E+00
ENSBTAG00000044708	0.00E+00	1.00E+00	0.00E+00	1.00E+00	-5.13E-15	1.00E+00	0.00E+00	1.00E+00	0.00E+00	1.00E+00
ENSBTAG00000007140	-1.15E+00	8.23E-01	-2.38E+00	9.84E-02	-4.36E+00	5.62E-03	-1.83E+00	1.70E-01	1.11E+00	6.06E-01
ENSBTAG00000010328	9.78E-01	7.04E-02	3.90E-01	4.69E-01	1.91E-03	1.00E+00	-1.08E-01	1.00E+00	3.03E-01	9.65E-01
ENSBTAG00000010352	5.69E-01	1.00E+00	5.71E-01	6.57E-01	-4.00E-02	1.00E+00	-1.91E-01	1.00E+00	9.41E-01	6.77E-01
ENSBTAG00000027197	-8.21E-01	8.53E-01	-1.07E+00	3.26E-01	-2.10E+00	2.68E-02	-4.30E-01	8.66E-01	3.77E-01	1.00E+00
ENSBTAG00000021381	3.51E-01	7.69E-01	-5.05E-01	1.25E-01	-6.81E-01	2.57E-02	-9.34E-01	1.58E-03	9.19E-02	1.00E+00
ENSBTAG00000010449	-2.96E-01	7.63E-01	-1.76E+00	3.98E-14	-1.44E+00	5.63E-10	-4.76E-01	8.02E-02	2.84E-01	7.90E-01

ENSBTAG00000043518	0.00E+00	1.00E+00	0.00E+00	1.00E+00	-5.13E-15	1.00E+00	0.00E+00	1.00E+00	0.00E+00	1.00E+00
ENSBTAG00000017971	-6.23E-01	1.00E+00	-7.40E-01	5.66E-01	-1.62E-01	1.00E+00	-1.94E+00	6.54E-02	-4.97E+00	1.46E-02
ENSBTAG00000044582	0.00E+00	1.00E+00	0.00E+00	1.00E+00	-5.13E-15	1.00E+00	0.00E+00	1.00E+00	0.00E+00	1.00E+00
ENSBTAG00000048116	0.00E+00	1.00E+00	0.00E+00	1.00E+00	-5.13E-15	1.00E+00	0.00E+00	1.00E+00	0.00E+00	1.00E+00
ENSBTAG00000002919	1.38E+00	1.24E-03	-9.28E-01	1.83E-02	-3.00E+00	4.00E-15	-1.50E+00	7.04E-05	1.38E+00	4.11E-03
ENSBTAG00000010197	-9.06E-01	2.19E-01	-2.31E+00	3.99E-06	-2.95E+00	7.90E-09	-1.69E+00	2.29E-04	1.16E+00	2.81E-02
ENSBTAG00000005974	2.11E-01	1.00E+00	9.96E-02	1.00E+00	-1.12E+00	2.55E-02	-1.46E+00	3.36E-03	1.51E+00	1.28E-02
ENSBTAG00000005975	2.23E-01	8.23E-01	4.97E-01	2.73E-02	5.53E-01	1.20E-02	3.72E-01	1.35E-01	-1.94E-01	8.92E-01
ENSBTAG00000009905	1.42E-01	1.00E+00	4.78E-01	1.01E-01	-2.66E-01	4.74E-01	-4.30E-01	1.66E-01	-7.16E-02	1.00E+00
ENSBTAG00000021359	7.32E-02	1.00E+00	-1.86E+00	2.28E-05	-1.86E+00	1.22E-05	-1.13E+00	8.84E-03	-8.02E-04	1.00E+00
ENSBTAG00000006485	8.04E-01	1.00E+00	1.77E+00	3.28E-01	1.15E+00	6.45E-01	-1.91E+00	4.39E-01	-3.95E-05	1.00E+00
ENSBTAG00000007275	1.23E+00	5.39E-01	8.98E-01	4.33E-01	8.02E-01	4.79E-01	5.58E-01	7.44E-01	9.65E-01	7.81E-01
ENSBTAG00000015707	1.48E+00	4.66E-02	2.67E+00	6.65E-07	2.00E+00	2.11E-04	1.50E+00	8.36E-03	8.80E-01	5.29E-01
ENSBTAG00000017593	2.82E+00	1.11E-05	2.59E+00	1.23E-05	2.79E+00	1.52E-06	2.93E+00	4.90E-07	1.78E+00	6.37E-02
ENSBTAG00000014211	3.60E-02	1.00E+00	5.97E-01	3.22E-02	1.09E+00	1.74E-05	1.21E+00	2.02E-06	7.25E-01	7.31E-02
ENSBTAG00000019343	-2.29E-01	1.00E+00	-2.59E+00	7.01E-04	-1.05E-01	1.00E+00	2.70E-01	9.21E-01	2.07E-01	1.00E+00
ENSBTAG00000012384	-8.96E-02	1.00E+00	-3.87E-01	2.58E-01	-4.77E-01	1.33E-01	-2.25E-01	6.29E-01	1.02E-03	1.00E+00
ENSBTAG00000003818	0.00E+00	1.00E+00	1.99E+00	4.43E-01	3.05E+00	1.36E-01	0.00E+00	1.00E+00	2.71E+00	6.01E-01
ENSBTAG00000009861	-1.68E-01	1.00E+00	-2.36E+00	3.35E-07	-1.88E+00	7.12E-06	-1.89E+00	7.95E-06	3.71E-01	8.00E-01
ENSBTAG00000038916	-5.92E-01	2.33E-01	8.02E-01	3.25E-03	-8.33E-01	4.59E-03	-7.36E-01	1.52E-02	-1.04E-01	1.00E+00
ENSBTAG00000008977	1.15E-01	1.00E+00	1.77E-01	7.20E-01	-5.33E-02	1.00E+00	1.55E-01	8.03E-01	-2.30E-01	8.88E-01
ENSBTAG00000008981	6.46E-02	1.00E+00	9.57E-01	8.84E-03	1.02E+00	4.16E-03	-4.22E-02	1.00E+00	4.65E-01	7.27E-01
ENSBTAG00000010100	7.92E-01	9.42E-03	2.31E+00	1.85E-23	1.74E+00	4.83E-14	1.06E+00	1.15E-05	2.01E-02	1.00E+00
ENSBTAG00000010101	5.81E-01	1.37E-01	2.84E+00	2.50E-34	2.60E+00	1.83E-29	2.02E+00	1.96E-18	-1.19E-01	1.00E+00
ENSBTAG00000010106	-8.67E-02	1.00E+00	5.81E-01	2.11E-02	-3.68E-01	2.05E-01	-2.94E-01	3.73E-01	-9.47E-02	1.00E+00
ENSBTAG00000011339	-2.57E-01	8.23E-01	-2.87E-01	3.48E-01	3.36E-01	2.28E-01	4.91E-01	5.28E-02	3.09E-01	6.81E-01
ENSBTAG00000048230	-5.45E-01	4.49E-01	-4.24E+00	8.12E-29	-4.60E+00	3.11E-33	-2.22E+00	1.67E-11	6.21E-01	3.70E-01
ENSBTAG00000012822	-4.94E-01	7.74E-01	-1.74E+00	4.87E-05	-2.87E+00	9.21E-11	-4.22E+00	2.12E-17	-6.83E-01	5.09E-01
ENSBTAG00000012823	1.75E-02	1.00E+00	-2.06E+00	3.42E-03	-3.25E+00	1.46E-05	-2.98E+00	5.09E-05	3.18E-01	1.00E+00
ENSBTAG00000012439	5.49E-01	1.00E+00	1.35E+00	2.24E-01	1.08E+00	3.78E-01	-5.61E-01	9.19E-01	1.39E+00	5.76E-01
ENSBTAG00000015301	-8.89E-02	1.00E+00	2.43E-01	4.97E-01	6.13E-01	1.81E-02	8.54E-01	6.47E-04	6.83E-02	1.00E+00
ENSBTAG00000015321	5.53E-01	3.51E-01	-1.31E+00	8.81E-05	-9.21E-01	4.95E-03	-8.73E-01	9.28E-03	2.50E-01	9.45E-01
ENSBTAG00000037268	0.00E+00	1.00E+00	0.00E+00	1.00E+00	-5.13E-15	1.00E+00	0.00E+00	1.00E+00	0.00E+00	1.00E+00
ENSBTAG00000043404	7.97E-01	1.00E+00	-1.91E+00	4.69E-01	-1.91E+00	4.28E-01	-1.91E+00	4.39E-01	1.84E-02	1.00E+00
ENSBTAG00000005967	-7.82E-02	1.00E+00	-2.76E-01	4.52E-01	-1.78E-01	7.37E-01	3.89E-02	1.00E+00	1.79E-01	1.00E+00

ENSBTAG00000005971	5.86E-01	8.23E-01	7.44E-01	2.69E-01	7.32E-01	2.66E-01	-4.57E-01	6.66E-01	-1.72E-01	1.00E+00
ENSBTAG00000029173	0.00E+00	1.00E+00	0.00E+00	1.00E+00	-5.13E-15	1.00E+00	0.00E+00	1.00E+00	0.00E+00	1.00E+00
ENSBTAG00000000940	-5.86E-01	6.12E-02	-5.67E-01	1.45E-02	-1.68E-01	6.66E-01	-2.06E-03	1.00E+00	-7.53E-02	1.00E+00
ENSBTAG00000014814	-1.59E-01	1.00E+00	-1.32E+00	3.58E-05	-4.61E-01	2.15E-01	-2.98E-01	5.11E-01	2.19E-01	1.00E+00
ENSBTAG00000018478	1.42E-01	1.00E+00	1.13E+00	1.44E-08	1.42E+00	5.98E-13	1.24E+00	5.26E-10	3.86E-02	1.00E+00
ENSBTAG00000017097	7.34E-01	9.05E-01	3.25E+00	8.54E-09	-4.02E-01	9.55E-01	-2.60E+00	5.65E-02	3.21E-01	1.00E+00
ENSBTAG00000015900	4.35E-01	5.76E-01	1.14E+00	8.64E-05	1.26E+00	1.28E-05	1.10E+00	2.09E-04	3.31E-01	8.00E-01
ENSBTAG00000005530	1.77E+00	2.03E-06	4.03E+00	9.99E-30	3.94E+00	7.41E-29	3.97E+00	4.52E-29	9.19E-01	1.10E-01
ENSBTAG00000005532	-1.48E-01	1.00E+00	-8.96E-03	1.00E+00	-3.59E-01	1.27E-01	-9.19E-01	5.94E-06	-7.85E-02	1.00E+00
ENSBTAG00000005533	-1.59E-01	1.00E+00	-8.57E-01	1.45E-04	-5.11E-01	3.40E-02	4.12E-02	1.00E+00	1.45E-01	1.00E+00
ENSBTAG00000005536	-7.82E-02	1.00E+00	-2.71E-04	1.00E+00	-8.55E-02	1.00E+00	-8.65E-02	1.00E+00	3.35E-02	1.00E+00
ENSBTAG00000012746	-8.89E-02	1.00E+00	5.78E-02	1.00E+00	1.81E-01	5.26E-01	9.13E-02	9.37E-01	2.51E-01	7.21E-01
ENSBTAG00000012749	1.17E-01	1.00E+00	-8.11E-01	1.60E-05	-1.21E+00	4.13E-11	-8.30E-01	1.14E-05	3.84E-01	3.30E-01
ENSBTAG00000012752	-1.89E-01	1.00E+00	-2.31E-01	6.13E-01	2.43E-01	5.71E-01	3.99E-01	2.51E-01	-3.24E-01	8.00E-01
ENSBTAG00000012755	-2.92E-01	7.18E-01	-8.09E-01	3.62E-04	-8.26E-01	2.36E-04	-4.69E-01	6.37E-02	2.28E-01	8.17E-01
ENSBTAG00000012761	2.95E-01	1.00E+00	-1.29E+00	2.21E-02	-8.77E-01	1.41E-01	5.29E-01	4.57E-01	1.66E-01	1.00E+00
ENSBTAG00000012777	2.70E-01	8.23E-01	1.34E+00	2.65E-09	1.26E+00	1.76E-08	8.89E-01	1.48E-04	-9.11E-02	1.00E+00
ENSBTAG00000019908	1.29E-01	1.00E+00	-5.51E-02	1.00E+00	5.15E-01	5.81E-03	3.88E-01	5.58E-02	2.01E-01	8.00E-01
ENSBTAG00000019913	-4.81E-01	5.72E-01	-9.56E-01	3.20E-03	-1.42E+00	5.82E-06	-1.13E+00	4.60E-04	-8.02E-03	1.00E+00
ENSBTAG00000046107	-1.14E+00	4.56E-02	-3.44E+00	7.54E-12	-1.28E+00	2.22E-03	-1.04E-01	1.00E+00	2.88E-01	9.96E-01
ENSBTAG00000045499	-1.27E+00	2.65E-01	-1.81E+00	1.27E-02	-1.72E+00	1.18E-02	3.33E-01	7.86E-01	5.76E-01	8.00E-01
ENSBTAG00000006350	-8.02E-01	3.95E-01	-2.77E+00	1.77E-09	-1.80E+00	8.22E-05	-5.21E-01	4.18E-01	-3.88E-01	9.22E-01
ENSBTAG00000006357	-1.55E-01	1.00E+00	-6.47E-01	1.77E-01	4.40E-02	1.00E+00	8.03E-01	6.22E-02	6.58E-01	4.89E-01
ENSBTAG00000042257	5.32E-03	1.00E+00	2.07E+00	1.95E-01	-1.25E-01	1.00E+00	1.12E+00	6.69E-01	1.78E-02	1.00E+00
ENSBTAG00000010217	-1.49E-01	1.00E+00	-1.58E+00	8.14E-09	9.90E-02	1.00E+00	8.28E-01	3.72E-03	7.61E-02	1.00E+00
ENSBTAG00000005842	9.80E-02	1.00E+00	-1.32E+00	2.10E-04	1.80E-01	8.88E-01	1.79E-02	1.00E+00	5.78E-01	5.09E-01
ENSBTAG00000005850	-1.30E+00	9.34E-01	-4.23E-01	1.00E+00	5.11E-01	9.38E-01	1.56E+00	1.72E-01	3.85E-01	1.00E+00
ENSBTAG00000011189	2.68E-01	9.05E-01	-1.73E-02	1.00E+00	-5.23E-02	1.00E+00	-1.71E-01	8.26E-01	2.55E-01	9.01E-01
ENSBTAG00000025554	-1.91E+00	8.23E-01	8.83E-01	8.52E-01	6.44E-01	9.78E-01	1.13E+00	6.66E-01	1.78E-02	1.00E+00
ENSBTAG00000018967	-8.15E-02	1.00E+00	-2.11E-01	4.47E-01	-9.30E-02	9.33E-01	-5.96E-02	1.00E+00	9.25E-02	1.00E+00
ENSBTAG00000018969	1.98E-01	9.79E-01	1.78E+00	1.08E-15	2.22E+00	8.45E-24	1.56E+00	2.48E-12	-1.36E-01	1.00E+00
ENSBTAG00000018970	3.01E-02	1.00E+00	8.99E-01	9.13E-06	1.84E+00	1.71E-21	1.58E+00	5.54E-16	2.33E-01	8.00E-01
ENSBTAG00000006015	2.61E-01	1.00E+00	3.19E+00	4.13E-20	1.45E+00	2.22E-05	6.77E-01	8.83E-02	-1.70E-01	1.00E+00
ENSBTAG00000006016	-4.84E-03	1.00E+00	1.95E+00	7.25E-07	1.15E+00	4.98E-03	1.05E+00	1.26E-02	2.82E-02	1.00E+00
ENSBTAG00000009548	1.62E-01	1.00E+00	1.32E+00	4.95E-05	1.12E+00	7.31E-04	1.18E+00	3.89E-04	4.85E-01	6.25E-01

ENSBTAG00000009549	-4.73E-01	9.33E-01	1.12E+00	4.99E-02	-1.35E-01	1.00E+00	-9.65E-01	1.19E-01	-1.60E-01	1.00E+00
ENSBTAG00000025571	9.34E-02	1.00E+00	4.43E-01	9.80E-02	6.92E-01	4.12E-03	6.01E-01	1.72E-02	-4.07E-02	1.00E+00
ENSBTAG00000005339	1.53E-01	1.00E+00	3.38E-01	2.19E-01	7.12E-02	1.00E+00	-3.43E-01	2.21E-01	-2.55E-02	1.00E+00
ENSBTAG00000047561	0.00E+00	1.00E+00	0.00E+00	1.00E+00	-5.13E-15	1.00E+00	0.00E+00	1.00E+00	0.00E+00	1.00E+00
ENSBTAG00000047995	0.00E+00	1.00E+00	0.00E+00	1.00E+00	1.78E+00	4.79E-01	0.00E+00	1.00E+00	0.00E+00	1.00E+00
ENSBTAG00000013722	5.14E-01	1.08E-01	3.22E-01	2.10E-01	8.23E-02	1.00E+00	-2.78E-02	1.00E+00	1.54E-01	1.00E+00
ENSBTAG00000011760	0.00E+00	1.00E+00	0.00E+00	1.00E+00	-5.13E-15	1.00E+00	0.00E+00	1.00E+00	0.00E+00	1.00E+00
ENSBTAG00000032680	8.65E-02	1.00E+00	-3.40E-01	2.55E-01	-1.07E+00	6.32E-06	-5.91E-01	2.29E-02	-1.53E-01	1.00E+00
ENSBTAG00000020012	0.00E+00	1.00E+00	0.00E+00	1.00E+00	-5.13E-15	1.00E+00	0.00E+00	1.00E+00	0.00E+00	1.00E+00
ENSBTAG00000021066	3.98E-01	9.42E-01	-7.18E-01	1.87E-01	-2.32E+00	4.16E-07	-3.32E+00	2.22E-12	7.25E-01	5.52E-01
ENSBTAG00000015131	-1.05E-01	1.00E+00	-2.10E-01	6.95E-01	-3.13E-01	4.28E-01	-2.19E-01	6.73E-01	1.43E-01	1.00E+00
ENSBTAG00000000778	7.56E-01	7.68E-02	2.51E+00	5.92E-18	1.38E+00	1.63E-06	9.15E-01	2.72E-03	2.75E-01	8.76E-01
ENSBTAG00000008076	-7.68E-02	1.00E+00	-6.79E-01	1.74E-03	-6.50E-01	2.64E-03	-3.28E-01	2.06E-01	8.57E-02	1.00E+00
ENSBTAG00000013705	2.70E+00	2.87E-13	2.08E+00	3.82E-09	5.36E-01	2.24E-01	9.25E-01	1.66E-02	-1.12E-01	1.00E+00
ENSBTAG00000013711	2.68E+00	6.00E-01	5.32E+00	7.95E-04	4.60E+00	6.61E-03	3.03E+00	1.66E-01	3.56E+00	3.49E-01
ENSBTAG00000032887	1.06E-01	1.00E+00	-6.11E-02	1.00E+00	1.06E-01	9.69E-01	-4.80E-01	9.25E-02	-2.43E-01	8.45E-01
ENSBTAG00000045355	0.00E+00	1.00E+00	0.00E+00	1.00E+00	-5.13E-15	1.00E+00	0.00E+00	1.00E+00	0.00E+00	1.00E+00
ENSBTAG00000004104	7.44E-01	4.88E-01	3.48E+00	5.75E-15	3.64E+00	2.88E-16	1.85E+00	3.88E-05	-4.64E-01	8.46E-01
ENSBTAG00000010244	-4.87E-02	1.00E+00	-2.46E-01	7.59E-01	-1.57E-01	9.68E-01	-6.32E-01	1.50E-01	1.01E-02	1.00E+00
ENSBTAG00000044482	0.00E+00	1.00E+00	0.00E+00	1.00E+00	-5.13E-15	1.00E+00	0.00E+00	1.00E+00	0.00E+00	1.00E+00
ENSBTAG00000003499	3.64E-01	6.99E-01	2.13E-01	6.68E-01	-8.63E-02	1.00E+00	3.31E-01	3.85E-01	-1.19E-01	1.00E+00
ENSBTAG00000015829	-1.99E-01	1.00E+00	-3.77E-01	4.32E-01	-4.92E-01	2.34E-01	2.39E-03	1.00E+00	-3.88E-02	1.00E+00
ENSBTAG00000009302	-2.14E-01	1.00E+00	8.65E-01	9.40E-02	-6.58E-01	3.47E-01	-4.23E-01	6.31E-01	2.87E-01	1.00E+00
ENSBTAG00000022736	0.00E+00	1.00E+00	0.00E+00	1.00E+00	-5.13E-15	1.00E+00	0.00E+00	1.00E+00	0.00E+00	1.00E+00
ENSBTAG00000003632	1.04E-01	1.00E+00	-2.54E-01	5.78E-01	-1.05E+00	2.92E-04	-1.68E+00	3.91E-09	7.71E-02	1.00E+00
ENSBTAG00000043423	0.00E+00	1.00E+00	0.00E+00	1.00E+00	-5.13E-15	1.00E+00	0.00E+00	1.00E+00	0.00E+00	1.00E+00
ENSBTAG00000016302	5.65E-01	1.95E-01	-4.64E-01	1.34E-01	-4.47E-01	1.49E-01	-3.39E-01	3.35E-01	5.19E-01	3.47E-01
ENSBTAG00000009978	-3.48E-02	1.00E+00	-4.73E-01	5.85E-01	-1.80E+00	2.13E-03	-6.73E-01	3.58E-01	-4.12E-01	9.91E-01
ENSBTAG00000019315	-2.42E-01	1.00E+00	-1.27E+00	3.25E-04	-1.63E+00	2.76E-06	-5.51E-01	1.99E-01	-9.14E-01	1.18E-01
ENSBTAG00000047009	-1.16E-03	1.00E+00	7.56E-02	1.00E+00	9.97E-01	6.04E-01	3.24E-01	1.00E+00	-2.71E+00	6.01E-01
ENSBTAG00000004345	-1.39E+00	5.52E-01	-2.99E+00	1.36E-02	-3.20E+00	5.58E-03	-1.05E+00	3.38E-01	-8.53E-01	8.10E-01
ENSBTAG00000004347	3.81E-01	6.80E-01	1.11E+00	8.00E-05	4.75E-01	1.52E-01	1.17E-02	1.00E+00	-1.15E-01	1.00E+00
ENSBTAG00000021609	9.47E-02	1.00E+00	2.56E+00	9.45E-05	2.55E+00	9.12E-05	1.40E+00	5.94E-02	1.68E+00	1.35E-01
ENSBTAG00000020054	4.51E-01	7.56E-01	8.87E-01	2.08E-02	6.43E-01	1.22E-01	-5.79E-02	1.00E+00	-5.82E-01	5.49E-01
ENSBTAG00000000322	3.46E-01	7.34E-01	1.13E+00	1.97E-05	8.44E-01	2.03E-03	9.67E-01	4.04E-04	-1.68E-02	1.00E+00

ENSBTAG00000037687	-9.17E-01	9.57E-01	-1.34E+00	4.03E-01	-1.57E+00	2.67E-01	-1.60E+00	2.71E-01	-2.21E+00	4.82E-01
ENSBTAG00000002934	0.00E+00	1.00E+00	0.00E+00	1.00E+00	-5.13E-15	1.00E+00	3.03E+00	1.50E-01	0.00E+00	1.00E+00
ENSBTAG00000016499	-1.92E-03	1.00E+00	1.51E+00	3.33E-01	-9.24E-01	8.15E-01	2.54E+00	2.21E-02	-7.99E-01	1.00E+00
ENSBTAG00000021415	-5.43E-02	1.00E+00	4.40E-01	7.84E-01	-1.14E+00	2.38E-01	6.69E-01	4.91E-01	-7.77E-01	8.29E-01
ENSBTAG00000046237	5.51E-01	2.01E-01	2.31E+00	1.07E-22	1.98E+00	3.62E-17	1.74E+00	2.14E-13	2.19E-01	9.28E-01
ENSBTAG00000014272	-4.30E-02	1.00E+00	-7.47E-01	2.59E-04	-1.72E+00	1.90E-18	-1.85E+00	6.54E-21	1.11E-01	1.00E+00
ENSBTAG00000020710	-1.92E-01	1.00E+00	-8.83E-01	3.26E-03	-8.15E-01	6.95E-03	-5.22E-01	1.26E-01	7.36E-02	1.00E+00
ENSBTAG00000025535	-4.47E-01	9.19E-01	-1.84E+00	2.09E-04	-2.76E+00	2.28E-08	-3.86E+00	6.17E-14	3.79E-01	9.77E-01
ENSBTAG00000047591	-6.03E-01	7.63E-01	-1.73E-01	1.00E+00	-2.80E-04	1.00E+00	1.03E+00	2.19E-02	3.13E-01	1.00E+00
ENSBTAG00000045363	0.00E+00	1.00E+00	0.00E+00	1.00E+00	-5.13E-15	1.00E+00	0.00E+00	1.00E+00	0.00E+00	1.00E+00
ENSBTAG00000011300	6.52E-03	1.00E+00	7.09E-02	1.00E+00	1.15E+00	6.45E-01	1.48E+00	4.59E-01	-1.78E-04	1.00E+00
ENSBTAG00000002166	-1.10E+00	8.23E-01	-4.75E-01	8.81E-01	-7.20E-01	6.27E-01	-2.17E+00	7.30E-02	-2.67E-03	1.00E+00
ENSBTAG00000012918	-2.88E-02	1.00E+00	-4.13E-02	1.00E+00	-3.53E-01	2.27E-01	-3.06E-01	3.36E-01	-8.68E-01	9.18E-03
ENSBTAG00000042716	0.00E+00	1.00E+00	1.99E+00	4.43E-01	-5.13E-15	1.00E+00	0.00E+00	1.00E+00	0.00E+00	1.00E+00
ENSBTAG00000019762	1.92E+00	8.23E-01	0.00E+00	1.00E+00	-5.13E-15	1.00E+00	0.00E+00	1.00E+00	0.00E+00	1.00E+00
ENSBTAG00000008085	0.00E+00	1.00E+00	0.00E+00	1.00E+00	-5.13E-15	1.00E+00	0.00E+00	1.00E+00	0.00E+00	1.00E+00
ENSBTAG00000046711	0.00E+00	1.00E+00	1.99E+00	4.43E-01	-5.13E-15	1.00E+00	2.53E+00	2.71E-01	1.91E+00	8.00E-01
ENSBTAG00000020425	4.20E-01	1.00E+00	-1.02E+00	4.59E-01	-7.45E-01	6.15E-01	1.67E-01	1.00E+00	9.93E-03	1.00E+00
ENSBTAG00000020426	-5.75E-01	1.00E+00	-3.50E-01	9.43E-01	-2.73E-01	1.00E+00	-9.74E-01	3.48E-01	-9.48E-01	7.92E-01
ENSBTAG00000011237	-2.21E-01	1.00E+00	-3.09E-01	7.29E-01	-9.01E-01	6.65E-02	-9.41E-01	5.82E-02	1.06E-01	1.00E+00
ENSBTAG00000036400	0.00E+00	1.00E+00	0.00E+00	1.00E+00	-5.13E-15	1.00E+00	0.00E+00	1.00E+00	0.00E+00	1.00E+00
ENSBTAG00000029826	0.00E+00	1.00E+00	0.00E+00	1.00E+00	-5.13E-15	1.00E+00	0.00E+00	1.00E+00	0.00E+00	1.00E+00
ENSBTAG00000002150	0.00E+00	1.00E+00	0.00E+00	1.00E+00	1.78E+00	4.79E-01	0.00E+00	1.00E+00	0.00E+00	1.00E+00
ENSBTAG00000016835	0.00E+00	1.00E+00	1.98E+00	4.43E-01	-5.13E-15	1.00E+00	0.00E+00	1.00E+00	0.00E+00	1.00E+00
ENSBTAG00000010721	-4.43E-01	5.76E-01	-1.56E+00	2.58E-07	-3.67E-01	3.43E-01	-2.47E-01	6.16E-01	-3.47E-01	8.00E-01
ENSBTAG00000008588	1.92E+00	8.23E-01	2.79E+00	2.10E-01	-5.13E-15	1.00E+00	1.76E+00	4.97E-01	0.00E+00	1.00E+00
ENSBTAG00000025494	-4.39E-03	1.00E+00	-5.28E-01	7.75E-02	-4.82E-01	1.15E-01	-2.93E-01	4.42E-01	9.85E-02	1.00E+00
ENSBTAG00000017810	-4.50E-01	8.23E-01	-8.21E-01	8.87E-02	-6.70E-01	1.66E-01	-7.64E-01	1.11E-01	-3.65E-01	9.00E-01
ENSBTAG00000009112	8.62E-01	1.57E-03	-2.57E-02	1.00E+00	9.60E-01	2.64E-05	2.04E+00	4.41E-20	6.20E-01	1.03E-01
ENSBTAG00000005206	4.82E-01	3.75E-01	-8.37E-01	2.44E-03	-1.48E+00	2.45E-08	-7.40E-01	9.43E-03	1.75E-01	1.00E+00
ENSBTAG00000006546	-4.44E-01	9.78E-01	-7.58E-01	2.55E-01	-1.75E+00	1.57E-03	-2.66E+00	1.61E-06	6.27E-02	1.00E+00
ENSBTAG00000025485	-8.64E-01	5.76E-01	-2.14E+00	2.53E-04	-3.40E+00	9.61E-09	-4.10E+00	1.95E-11	-6.52E-01	8.00E-01
ENSBTAG00000021516	-6.98E-01	7.22E-02	-1.18E+00	9.49E-06	-2.07E+00	2.86E-15	-2.94E+00	5.19E-28	-5.46E-01	3.26E-01
ENSBTAG00000037949	-7.03E-01	5.64E-02	-1.42E+00	6.75E-08	-3.11E+00	3.11E-28	-4.14E+00	8.65E-41	-1.31E-01	1.00E+00
ENSBTAG00000032642	-4.74E-01	1.00E+00	-4.96E-01	8.56E-01	-1.83E+00	6.98E-02	-2.80E+00	4.54E-03	1.49E+00	5.13E-01

ENSBTAG00000004288	-3.66E-01	5.10E-01	-1.47E+00	2.98E-11	-2.94E+00	1.82E-38	-3.88E+00	1.07E-60	2.15E-02	1.00E+00
ENSBTAG00000043250	-7.48E-01	3.45E-01	-7.79E-01	8.23E-02	-8.91E-01	3.91E-02	-1.79E+00	1.16E-05	-2.05E+00	2.28E-05
ENSBTAG00000015590	-7.74E-01	4.21E-02	-3.66E+00	3.54E-36	-3.72E+00	9.44E-38	-2.26E+00	1.56E-16	2.76E-01	8.31E-01
ENSBTAG00000015083	-1.90E-01	1.00E+00	-2.09E+00	4.74E-17	-2.69E+00	1.45E-26	-2.23E+00	4.50E-19	4.70E-02	1.00E+00
ENSBTAG00000008148	8.12E-01	1.00E+00	7.59E-02	1.00E+00	-1.24E-01	1.00E+00	1.12E+00	6.71E-01	1.31E+00	8.99E-01
ENSBTAG00000032624	0.00E+00	1.00E+00	0.00E+00	1.00E+00	-5.13E-15	1.00E+00	0.00E+00	1.00E+00	0.00E+00	1.00E+00
ENSBTAG00000003359	-3.50E-01	8.23E-01	-9.14E-01	3.87E-03	-1.90E+00	2.75E-10	-1.88E+00	6.82E-10	-9.27E-02	1.00E+00
ENSBTAG00000045034	0.00E+00	1.00E+00	0.00E+00	1.00E+00	-5.13E-15	1.00E+00	1.76E+00	4.97E-01	0.00E+00	1.00E+00
ENSBTAG00000009656	3.15E-01	1.00E+00	1.53E-01	1.00E+00	-3.23E-01	1.00E+00	-6.98E-01	1.00E+00	-4.32E-01	1.00E+00
ENSBTAG00000021077	7.72E-02	1.00E+00	-1.60E-01	1.00E+00	-8.32E-01	6.53E-01	-9.25E-01	5.96E-01	-6.31E-01	1.00E+00
ENSBTAG00000038128	1.68E-01	1.00E+00	4.02E-02	1.00E+00	-6.30E-01	8.95E-01	-1.02E+00	6.31E-01	-5.16E-01	1.00E+00
ENSBTAG00000003352	1.11E-01	1.00E+00	1.05E-01	1.00E+00	-6.37E-01	2.23E-01	-1.12E+00	1.60E-02	-3.69E-01	9.18E-01
ENSBTAG00000037605	3.87E-01	1.00E+00	5.84E-02	1.00E+00	-5.08E-01	1.00E+00	-6.36E-01	9.21E-01	-3.79E-01	1.00E+00
ENSBTAG00000013919	2.06E-01	1.00E+00	4.26E-02	1.00E+00	-3.58E-01	3.87E-01	-6.19E-01	7.27E-02	-4.79E-01	5.70E-01
ENSBTAG00000038397	2.22E+00	4.19E-01	7.51E-02	1.00E+00	-1.31E-01	1.00E+00	-1.48E-01	1.00E+00	6.44E-03	1.00E+00
ENSBTAG00000010645	2.12E-01	1.00E+00	5.36E-02	1.00E+00	-3.87E-01	4.45E-01	-7.62E-01	6.10E-02	-4.28E-01	7.93E-01
ENSBTAG00000034939	-7.87E-01	1.00E+00	-7.21E-01	9.45E-01	3.50E-01	1.00E+00	-1.66E-01	1.00E+00	-7.91E-01	1.00E+00
ENSBTAG00000034945	-1.79E-03	1.00E+00	-2.71E+00	2.42E-01	-2.71E+00	1.99E-01	-9.46E-01	8.04E-01	-7.81E-01	1.00E+00
ENSBTAG00000007618	1.92E+00	8.23E-01	3.68E+00	5.07E-02	-5.13E-15	1.00E+00	2.53E+00	2.71E-01	1.91E+00	8.00E-01
ENSBTAG00000019588	3.25E-01	1.00E+00	1.01E-01	1.00E+00	-4.43E-01	7.49E-01	-6.93E-01	4.73E-01	-2.61E-01	1.00E+00
ENSBTAG00000033979	2.71E+00	5.76E-01	0.00E+00	1.00E+00	-5.13E-15	1.00E+00	0.00E+00	1.00E+00	1.93E+00	8.00E-01
ENSBTAG00000026163	-5.56E-01	1.00E+00	-2.11E+00	6.05E-01	-2.79E+00	4.28E-01	-2.07E+00	6.19E-01	2.20E-01	1.00E+00
ENSBTAG00000046116	0.00E+00	1.00E+00	0.00E+00	1.00E+00	1.78E+00	4.79E-01	0.00E+00	1.00E+00	0.00E+00	1.00E+00
ENSBTAG00000023541	1.32E+00	7.00E-01	1.72E+00	1.16E-01	1.14E+00	4.13E-01	7.26E-01	7.48E-01	6.80E-01	1.00E+00
ENSBTAG00000046920	0.00E+00	1.00E+00	0.00E+00	1.00E+00	-5.13E-15	1.00E+00	0.00E+00	1.00E+00	0.00E+00	1.00E+00
ENSBTAG00000023563	-5.80E-01	8.09E-01	4.32E-02	1.00E+00	-3.91E-01	6.39E-01	-9.84E-01	8.47E-02	-2.45E-01	1.00E+00
ENSBTAG00000048304	-1.67E-01	1.00E+00	4.33E-01	4.39E-01	1.00E-02	1.00E+00	-4.30E-01	4.70E-01	6.54E-02	1.00E+00
ENSBTAG00000025424	9.96E-02	1.00E+00	1.03E+00	2.79E-02	1.10E+00	1.66E-02	5.46E-01	3.53E-01	1.13E-01	1.00E+00
ENSBTAG00000014421	-2.32E-01	8.60E-01	-6.28E-01	1.01E-02	-5.37E-01	3.17E-02	4.90E-02	1.00E+00	2.35E-01	8.20E-01
ENSBTAG00000014420	4.45E-01	5.76E-01	8.94E-01	2.77E-03	-2.35E-01	6.66E-01	-6.33E-01	7.55E-02	1.37E-01	1.00E+00
ENSBTAG00000025413	-2.18E-01	8.23E-01	-4.64E-01	3.91E-02	-4.25E-01	6.33E-02	-1.23E-01	8.29E-01	-3.82E-02	1.00E+00
ENSBTAG00000004442	-2.02E-01	8.85E-01	-2.80E-01	2.98E-01	-3.35E-01	1.80E-01	-3.28E-01	2.03E-01	1.59E-01	9.91E-01
ENSBTAG00000025410	-1.03E+00	1.79E-01	-1.07E+00	3.47E-02	-1.78E+00	2.50E-04	-2.35E+00	3.58E-06	7.39E-02	1.00E+00
ENSBTAG00000004436	-4.45E-01	4.62E-01	-2.08E+00	8.77E-15	-1.78E+00	1.24E-11	-7.11E-01	1.26E-02	3.22E-01	7.79E-01
ENSBTAG00000038904	-1.46E+00	1.35E-01	-3.67E+00	1.28E-05	-2.09E+00	1.64E-03	-1.17E+00	7.67E-02	-6.31E-01	8.00E-01

ENSBTAG00000037781	-1.38E-01	1.00E+00	1.41E+00	3.97E-06	1.23E+00	6.38E-05	9.67E-01	2.95E-03	2.64E-01	9.58E-01
ENSBTAG00000008794	-1.36E-01	1.00E+00	2.04E-01	4.69E-01	6.18E-01	2.01E-03	5.61E-01	6.76E-03	2.44E-01	7.69E-01
ENSBTAG00000047039	5.02E-01	1.00E+00	2.55E+00	5.97E-06	2.63E+00	1.53E-06	1.40E+00	4.93E-02	1.20E+00	4.39E-01
ENSBTAG00000001444	3.56E-01	6.25E-01	5.31E-02	1.00E+00	-1.69E-01	7.56E-01	-4.49E-01	1.37E-01	5.46E-01	2.71E-01
ENSBTAG00000006864	8.04E-01	1.78E-02	6.56E-01	1.78E-02	5.68E-01	4.67E-02	4.05E-01	2.10E-01	1.11E+00	5.76E-04
ENSBTAG00000001443	2.47E+00	1.49E-11	3.36E+00	3.93E-21	2.89E+00	1.61E-16	1.91E+00	5.41E-08	1.67E+00	7.92E-05
ENSBTAG00000037533	4.90E-01	5.77E-01	3.43E-01	4.69E-01	1.71E-01	8.93E-01	1.78E-01	8.81E-01	1.05E+00	2.93E-02
ENSBTAG00000005589	2.84E-01	7.71E-01	9.01E-01	9.60E-05	8.60E-01	2.02E-04	1.80E-01	6.72E-01	1.62E-03	1.00E+00
ENSBTAG00000005588	1.76E-01	1.00E+00	1.14E+00	8.37E-05	1.05E+00	3.04E-04	6.17E-01	5.92E-02	-1.24E-02	1.00E+00
ENSBTAG00000005587	1.70E-01	1.00E+00	4.16E-01	7.96E-02	6.97E-01	1.10E-03	7.18E-01	9.16E-04	6.96E-02	1.00E+00
ENSBTAG00000007453	-4.50E-03	1.00E+00	5.57E-01	2.25E-02	8.64E-01	1.37E-04	1.01E+00	7.59E-06	-2.93E-01	7.57E-01
ENSBTAG00000046158	3.69E-01	2.97E-01	1.02E-01	8.77E-01	-3.93E-01	5.82E-02	-5.12E-01	1.04E-02	4.05E-01	2.82E-01
ENSBTAG00000007450	5.28E-01	3.39E-01	-1.72E-01	8.11E-01	-9.74E-01	5.52E-04	-1.27E+00	5.43E-06	7.75E-01	7.88E-02
ENSBTAG00000032031	5.91E-02	1.00E+00	-1.22E-01	1.00E+00	-6.76E-01	1.99E-01	-1.16E+00	1.64E-02	6.61E-01	5.53E-01
ENSBTAG00000005676	-4.09E-01	4.69E-01	-1.80E+00	1.76E-13	-1.73E+00	7.03E-13	-1.23E+00	5.46E-07	1.85E-01	9.98E-01
ENSBTAG00000005675	8.63E-02	1.00E+00	3.00E+00	2.25E-04	3.79E+00	3.59E-06	3.69E+00	8.17E-06	-1.27E+00	5.91E-01
ENSBTAG00000005674	2.15E-01	1.00E+00	5.32E-01	2.95E-01	7.18E-01	1.15E-01	8.36E-01	6.20E-02	1.36E-01	1.00E+00
ENSBTAG00000042989	2.77E-01	1.00E+00	4.42E-01	4.84E-01	1.53E+00	2.03E-04	5.53E-01	3.34E-01	-1.69E+00	2.38E-02
ENSBTAG00000043151	1.90E-01	1.00E+00	1.20E+00	4.21E-02	1.75E+00	7.92E-04	7.53E-01	2.90E-01	-6.84E-01	8.00E-01
ENSBTAG00000025441	2.14E+00	8.24E-02	4.87E+00	1.12E-07	6.87E-01	6.03E-01	1.17E+00	2.62E-01	-1.09E-01	1.00E+00
ENSBTAG00000025442	1.32E+00	1.34E-01	2.79E+00	2.40E-07	3.66E-01	7.85E-01	6.30E-01	4.39E-01	1.24E+00	2.49E-01
ENSBTAG00000005634	-1.93E-02	1.00E+00	1.18E-02	1.00E+00	-3.13E-02	1.00E+00	1.07E-01	1.00E+00	-4.41E-01	5.33E-01
ENSBTAG00000005630	-8.43E-01	6.77E-02	-9.35E-01	5.47E-03	-2.52E+00	6.25E-13	-2.08E+00	7.99E-10	-2.13E-01	1.00E+00
ENSBTAG00000005631	1.00E-01	1.00E+00	1.49E+00	1.10E-11	2.00E+00	7.90E-20	1.89E+00	9.04E-18	-2.31E-01	8.27E-01
ENSBTAG00000039620	-5.69E-01	8.23E-01	-2.11E+00	7.90E-04	-2.53E+00	4.63E-05	-2.28E+00	2.06E-04	-3.23E-01	1.00E+00
ENSBTAG00000019790	-5.98E-01	6.14E-01	-1.72E+00	1.88E-04	-2.92E+00	1.86E-09	-2.28E+00	9.06E-07	2.17E-01	1.00E+00
ENSBTAG00000013533	-4.22E-01	5.49E-01	-1.42E+00	1.47E-07	-1.12E+00	2.99E-05	-3.25E-01	3.83E-01	-4.48E-01	5.16E-01
ENSBTAG00000013530	-3.95E-01	7.35E-01	-1.53E+00	1.25E-06	-2.76E+00	2.83E-17	-1.88E+00	2.66E-09	-2.30E-01	1.00E+00
ENSBTAG00000013525	8.65E-02	1.00E+00	9.14E-01	1.61E-01	2.05E-01	1.00E+00	4.77E-01	6.06E-01	-8.10E-01	7.37E-01
ENSBTAG00000000585	8.75E-03	1.00E+00	-1.61E+00	4.05E-01	-1.04E+00	5.96E-01	-5.62E-01	9.19E-01	-8.65E-01	1.00E+00
ENSBTAG00000038955	0.00E+00	1.00E+00	0.00E+00	1.00E+00	-5.13E-15	1.00E+00	3.03E+00	1.50E-01	0.00E+00	1.00E+00
ENSBTAG00000000582	-6.22E+00	2.49E-05	-3.43E+00	4.85E-04	-3.66E+00	1.28E-04	-4.46E+00	2.26E-05	-2.99E+00	2.08E-02
ENSBTAG00000000580	3.90E-01	1.00E+00	-1.23E+00	6.12E-01	-1.43E+00	4.79E-01	9.34E-01	5.65E-01	-3.21E+00	4.34E-01
ENSBTAG00000000578	-2.32E-01	9.00E-01	-1.13E+00	2.90E-06	-7.25E-01	3.98E-03	-7.68E-01	2.58E-03	7.19E-02	1.00E+00
ENSBTAG00000025449	-6.19E-04	1.00E+00	8.74E-01	8.53E-01	-1.91E+00	4.28E-01	-1.91E+00	4.39E-01	-1.91E+00	8.00E-01

ENSBTAG00000039740	-4.14E-01	8.45E-01	3.91E-01	5.02E-01	-7.49E-02	1.00E+00	-7.86E-01	9.37E-02	-2.17E-01	1.00E+00
ENSBTAG00000008837	-6.14E-02	1.00E+00	-1.58E-02	1.00E+00	1.88E-01	6.91E-01	3.42E-01	3.02E-01	-2.54E-01	8.34E-01
ENSBTAG00000008835	-1.90E-01	9.70E-01	9.74E-01	1.15E-05	1.08E+00	7.91E-07	6.14E-01	1.01E-02	-8.17E-02	1.00E+00
ENSBTAG00000023628	-5.29E-01	1.66E-01	-1.93E+00	1.40E-15	-1.04E+00	8.35E-06	-5.42E-01	3.43E-02	-2.93E-01	7.59E-01
ENSBTAG00000008833	-2.64E-02	1.00E+00	-6.68E-01	8.34E-02	-1.48E+00	1.60E-05	-1.70E+00	8.07E-07	3.35E-01	8.44E-01
ENSBTAG00000019685	-1.97E-02	1.00E+00	2.50E-01	3.69E-01	5.60E-01	9.07E-03	6.90E-01	1.11E-03	1.69E-01	9.49E-01
ENSBTAG00000019682	-9.27E-02	1.00E+00	3.10E-01	2.33E-01	7.43E-01	4.27E-04	7.60E-01	3.80E-04	2.77E-02	1.00E+00
ENSBTAG00000042984	0.00E+00	1.00E+00	0.00E+00	1.00E+00	-5.13E-15	1.00E+00	0.00E+00	1.00E+00	0.00E+00	1.00E+00
ENSBTAG00000037350	1.92E+00	8.23E-01	0.00E+00	1.00E+00	2.55E+00	2.55E-01	1.77E+00	4.97E-01	1.91E+00	8.00E-01
ENSBTAG00000020554	-2.31E-01	9.80E-01	-7.20E-01	1.47E-02	-9.86E-01	4.64E-04	-8.89E-01	2.16E-03	-5.78E-01	3.11E-01
ENSBTAG00000018343	-4.41E-01	7.57E-01	-1.05E+00	5.91E-03	-8.43E-01	2.75E-02	-1.45E+00	1.07E-04	-4.59E-01	7.29E-01
ENSBTAG00000020674	7.42E-01	4.43E-02	-2.47E-01	5.61E-01	-4.85E-01	1.25E-01	-8.08E-01	5.21E-03	-2.94E-01	8.00E-01
ENSBTAG00000025471	1.59E+00	2.14E-06	5.66E-01	1.39E-01	-1.84E-01	8.65E-01	-5.14E-01	2.32E-01	-2.61E-01	9.93E-01
ENSBTAG00000000016	1.37E+00	8.02E-02	1.63E-01	1.00E+00	4.94E-02	1.00E+00	-5.18E-01	6.29E-01	4.27E-01	1.00E+00
ENSBTAG00000014492	-2.92E-01	8.20E-01	2.29E-01	5.49E-01	-1.57E-01	7.93E-01	-5.04E-01	7.85E-02	-1.53E-01	1.00E+00
ENSBTAG00000014491	-1.88E+00	2.61E-01	8.79E-02	1.00E+00	8.32E-01	3.03E-01	-4.45E-01	8.21E-01	4.81E-01	1.00E+00
ENSBTAG00000014490	3.12E-01	5.40E-01	1.02E+00	1.35E-07	1.01E+00	1.72E-07	4.66E-01	3.20E-02	1.70E-01	9.25E-01
ENSBTAG00000043392	0.00E+00	1.00E+00	0.00E+00	1.00E+00	-5.13E-15	1.00E+00	0.00E+00	1.00E+00	0.00E+00	1.00E+00
ENSBTAG00000043483	0.00E+00	1.00E+00	0.00E+00	1.00E+00	-5.13E-15	1.00E+00	0.00E+00	1.00E+00	0.00E+00	1.00E+00
ENSBTAG00000045577	-8.98E-01	1.00E+00	-8.20E-01	7.71E-01	1.01E-01	1.00E+00	-1.07E+00	5.89E-01	-9.02E-01	1.00E+00
ENSBTAG00000008959	3.89E-01	9.99E-01	4.29E-02	1.00E+00	-3.29E-01	7.44E-01	-8.67E-01	1.34E-01	-2.25E-01	1.00E+00
ENSBTAG00000031913	5.86E-02	1.00E+00	-5.85E-02	1.00E+00	-3.35E-01	3.24E-01	-1.98E-01	6.86E-01	-2.06E-01	9.74E-01
ENSBTAG00000031905	-1.91E-01	1.00E+00	-2.11E-01	9.89E-01	-6.97E-01	2.82E-01	-8.72E-01	1.56E-01	-6.40E-01	7.63E-01
ENSBTAG00000005182	3.81E-02	1.00E+00	5.17E-01	3.97E-01	-3.47E-02	1.00E+00	-2.16E-01	9.24E-01	-4.84E-02	1.00E+00
ENSBTAG00000010166	4.42E-01	1.00E+00	1.28E+00	1.72E-01	5.71E-01	6.94E-01	7.14E-02	1.00E+00	2.45E-02	1.00E+00
ENSBTAG00000012208	0.00E+00	1.00E+00	0.00E+00	1.00E+00	-5.13E-15	1.00E+00	0.00E+00	1.00E+00	0.00E+00	1.00E+00
ENSBTAG00000001476	0.00E+00	1.00E+00	0.00E+00	1.00E+00	-5.13E-15	1.00E+00	0.00E+00	1.00E+00	0.00E+00	1.00E+00
ENSBTAG00000021111	1.92E+00	8.23E-01	1.98E+00	4.43E-01	1.78E+00	4.79E-01	0.00E+00	1.00E+00	0.00E+00	1.00E+00
ENSBTAG00000014435	-3.03E-01	1.00E+00	-1.22E+00	7.96E-03	-1.44E+00	1.11E-03	-1.60E+00	3.91E-04	-6.29E-01	6.01E-01
ENSBTAG00000014434	-1.35E-01	1.00E+00	-1.59E+00	1.12E-09	-1.85E+00	5.90E-13	-1.35E+00	9.04E-08	6.00E-02	1.00E+00
ENSBTAG00000025531	0.00E+00	1.00E+00	0.00E+00	1.00E+00	-5.13E-15	1.00E+00	0.00E+00	1.00E+00	1.91E+00	8.00E-01
ENSBTAG00000021721	1.33E-02	1.00E+00	7.58E-02	1.00E+00	-1.91E+00	4.28E-01	-1.91E+00	4.39E-01	1.84E-02	1.00E+00
ENSBTAG00000004539	0.00E+00	1.00E+00	1.99E+00	4.43E-01	-5.13E-15	1.00E+00	0.00E+00	1.00E+00	0.00E+00	1.00E+00
ENSBTAG00000043004	2.70E+00	5.76E-01	3.30E+00	1.02E-01	3.05E+00	1.36E-01	0.00E+00	1.00E+00	1.93E+00	8.00E-01
ENSBTAG00000007075	-1.68E-01	1.00E+00	-4.23E-02	1.00E+00	-5.06E-01	9.47E-01	-8.33E-01	6.91E-01	-2.72E-01	1.00E+00

ENSBTAG00000022590	6.32E-02	1.00E+00	1.84E-01	9.96E-01	-1.29E-01	1.00E+00	-2.54E-01	8.61E-01	-2.78E-02	1.00E+00
ENSBTAG00000038810	-1.39E-01	1.00E+00	-1.89E+00	1.22E-01	-1.26E+00	2.74E-01	-1.28E+00	2.75E-01	7.44E-03	1.00E+00
ENSBTAG00000043267	0.00E+00	1.00E+00	0.00E+00	1.00E+00	1.78E+00	4.79E-01	0.00E+00	1.00E+00	0.00E+00	1.00E+00
ENSBTAG00000010698	-2.98E-01	8.19E-01	-7.65E-01	4.00E-03	2.07E-01	6.33E-01	2.99E-01	4.06E-01	-2.25E-01	9.12E-01
ENSBTAG00000046757	-2.32E-01	8.54E-01	-9.04E-01	1.15E-04	1.36E-03	1.00E+00	1.60E-01	7.35E-01	-1.81E-01	9.74E-01
ENSBTAG00000010682	-5.61E-02	1.00E+00	-1.25E+00	1.01E-02	-1.12E+00	2.22E-02	-9.74E-01	5.77E-02	2.98E-01	1.00E+00
ENSBTAG00000011358	1.38E+00	6.88E-02	1.05E+00	7.37E-02	1.16E+00	4.10E-02	1.19E+00	4.03E-02	-5.55E-02	1.00E+00
ENSBTAG00000009960	1.71E-01	1.00E+00	2.49E-01	4.41E-01	7.27E-03	1.00E+00	7.43E-03	1.00E+00	-9.31E-03	1.00E+00
ENSBTAG00000006969	1.01E-01	1.00E+00	2.75E-02	1.00E+00	-5.69E-02	1.00E+00	7.52E-01	9.53E-03	-7.82E-01	6.67E-02
ENSBTAG00000006966	6.63E-01	2.70E-01	-1.51E-01	9.57E-01	-5.72E-01	1.53E-01	-5.15E-01	2.25E-01	-8.08E-02	1.00E+00
ENSBTAG00000025526	4.75E-01	5.76E-01	-6.69E-01	5.91E-02	-6.55E-01	6.56E-02	-1.49E-01	9.43E-01	-4.17E-02	1.00E+00
ENSBTAG00000039695	1.91E+00	3.29E-18	8.35E-01	1.62E-04	-3.47E-02	1.00E+00	9.56E-02	9.77E-01	1.39E-01	1.00E+00
ENSBTAG00000006960	5.61E-03	1.00E+00	-1.38E-01	9.06E-01	7.58E-02	1.00E+00	-2.80E-01	4.89E-01	3.02E-01	8.00E-01
ENSBTAG00000006958	-2.52E-01	9.94E-01	-1.36E+00	2.68E-05	-1.47E-01	9.27E-01	5.20E-01	1.50E-01	2.62E-01	9.24E-01
ENSBTAG00000006941	7.11E-02	1.00E+00	1.74E-01	7.60E-01	-4.75E-01	1.11E-01	-5.63E-01	5.36E-02	1.75E-01	1.00E+00
ENSBTAG00000006936	1.45E-01	1.00E+00	4.48E-02	1.00E+00	4.17E-01	1.57E-01	7.33E-01	5.26E-03	-1.89E-01	9.99E-01
ENSBTAG00000006933	1.91E-01	1.00E+00	1.28E+00	1.04E-05	4.19E-01	2.51E-01	-1.27E-01	9.74E-01	1.81E-01	1.00E+00
ENSBTAG00000043729	0.00E+00	1.00E+00	0.00E+00	1.00E+00	-5.13E-15	1.00E+00	0.00E+00	1.00E+00	0.00E+00	1.00E+00
ENSBTAG00000006927	-3.92E-02	1.00E+00	7.51E-01	7.90E-04	1.31E+00	9.23E-10	1.37E+00	2.32E-10	-1.60E-02	1.00E+00
ENSBTAG00000044647	0.00E+00	1.00E+00	0.00E+00	1.00E+00	-5.13E-15	1.00E+00	0.00E+00	1.00E+00	0.00E+00	1.00E+00
ENSBTAG00000037337	0.00E+00	1.00E+00	0.00E+00	1.00E+00	-5.13E-15	1.00E+00	0.00E+00	1.00E+00	0.00E+00	1.00E+00
ENSBTAG00000006914	-6.56E-01	2.38E-01	-1.44E-01	9.45E-01	9.14E-01	3.57E-03	5.23E-01	1.55E-01	-1.15E-01	1.00E+00
ENSBTAG00000025516	-1.49E-01	1.00E+00	-4.41E-01	8.22E-02	8.57E-02	1.00E+00	5.97E-01	1.06E-02	-6.87E-02	1.00E+00
ENSBTAG00000007643	-4.56E-02	1.00E+00	5.47E-01	1.53E-02	6.14E-01	5.22E-03	3.74E-01	1.41E-01	-2.22E-02	1.00E+00
ENSBTAG00000001477	8.80E-02	1.00E+00	1.14E+00	6.92E-06	1.16E+00	3.55E-06	8.50E-01	1.36E-03	-1.45E-01	1.00E+00
ENSBTAG00000038619	2.73E-01	1.00E+00	1.51E+00	4.23E-05	3.95E-01	4.45E-01	3.86E-01	4.77E-01	2.10E-01	1.00E+00
ENSBTAG00000005146	-1.25E-01	1.00E+00	-2.81E-01	8.32E-01	-8.84E-01	1.02E-01	-1.11E+00	3.38E-02	-9.75E-02	1.00E+00
ENSBTAG00000019386	5.15E-02	1.00E+00	7.88E-02	1.00E+00	-4.40E-01	6.46E-01	-8.00E-01	2.61E-01	-2.47E-01	1.00E+00
ENSBTAG00000020116	1.06E-01	1.00E+00	3.57E-01	9.76E-01	-3.40E-01	9.90E-01	-4.79E-01	8.50E-01	-1.55E-01	1.00E+00
ENSBTAG00000002069	7.10E-02	1.00E+00	1.28E-01	1.00E+00	-6.14E-01	2.78E-01	-8.21E-01	1.18E-01	-2.56E-01	1.00E+00
ENSBTAG00000037421	1.48E-02	1.00E+00	-1.32E+00	1.75E-01	-1.97E+00	2.71E-02	-2.42E+00	7.15E-03	2.98E-01	1.00E+00
ENSBTAG00000035744	-4.76E-01	3.21E-01	-1.47E-01	8.31E-01	7.88E-02	1.00E+00	-1.62E-01	7.78E-01	3.14E-01	7.47E-01
ENSBTAG00000007532	1.32E-02	1.00E+00	8.81E-01	8.52E-01	1.15E+00	6.43E-01	-1.47E-01	1.00E+00	8.00E-01	1.00E+00
ENSBTAG00000044550	0.00E+00	1.00E+00	0.00E+00	1.00E+00	-5.13E-15	1.00E+00	0.00E+00	1.00E+00	0.00E+00	1.00E+00
ENSBTAG00000007530	1.93E+00	1.63E-01	8.50E-01	5.97E-01	1.79E+00	5.52E-02	3.69E-01	1.00E+00	7.44E-01	9.99E-01

ENSBTAG00000037381	-5.64E-04	1.00E+00	7.12E-02	1.00E+00	-1.91E+00	4.28E-01	-1.54E-01	1.00E+00	1.30E+00	9.00E-01
ENSBTAG00000004490	9.33E-01	7.16E-01	1.57E+00	3.58E-02	1.33E+00	9.12E-02	1.18E+00	1.59E-01	1.02E+00	6.20E-01
ENSBTAG00000032247	2.40E-02	1.00E+00	1.98E-01	6.00E-01	2.61E-01	4.17E-01	5.39E-02	1.00E+00	2.39E-02	1.00E+00
ENSBTAG00000031792	8.36E-02	1.00E+00	3.66E-01	4.43E-01	7.96E-01	2.42E-02	3.93E-01	3.98E-01	-5.18E-01	5.98E-01
ENSBTAG00000017836	1.33E-01	1.00E+00	-7.97E-02	1.00E+00	3.15E-01	1.00E+00	4.77E-01	1.00E+00	1.95E-01	1.00E+00
ENSBTAG00000017818	-5.29E-02	1.00E+00	-9.02E-02	1.00E+00	8.85E-02	1.00E+00	-4.45E-02	1.00E+00	1.42E-01	1.00E+00
ENSBTAG00000017239	1.38E-02	1.00E+00	-5.05E-01	1.42E-01	-3.49E-01	3.80E-01	-6.63E-01	4.21E-02	1.74E-01	1.00E+00
ENSBTAG00000031825	-3.78E-01	1.00E+00	9.58E-01	1.22E-01	-1.56E-02	1.00E+00	-2.65E-01	9.30E-01	-9.80E-01	4.95E-01
ENSBTAG00000025398	9.05E-01	7.76E-01	1.60E+00	5.20E-02	3.92E-01	8.84E-01	-3.71E-01	9.20E-01	-9.82E-01	7.36E-01
ENSBTAG00000003250	0.00E+00	1.00E+00	0.00E+00	1.00E+00	-5.13E-15	1.00E+00	0.00E+00	1.00E+00	0.00E+00	1.00E+00
ENSBTAG00000043759	0.00E+00	1.00E+00	1.98E+00	4.43E-01	1.78E+00	4.79E-01	0.00E+00	1.00E+00	0.00E+00	1.00E+00
ENSBTAG00000027245	-1.91E+00	8.23E-01	8.80E-01	8.52E-01	-1.91E+00	4.28E-01	6.20E-01	9.94E-01	-1.91E+00	8.00E-01
ENSBTAG00000027246	9.53E-01	2.87E-01	1.79E+00	2.69E-04	4.11E-01	5.97E-01	3.32E-01	7.47E-01	-1.06E+00	2.67E-01
ENSBTAG00000017227	-1.92E+00	5.74E-02	-1.26E+00	9.17E-02	6.30E-01	3.55E-01	-1.17E+00	1.06E-01	-1.34E+00	3.29E-01
ENSBTAG00000045586	2.72E+00	5.76E-01	0.00E+00	1.00E+00	-5.13E-15	1.00E+00	0.00E+00	1.00E+00	0.00E+00	1.00E+00
ENSBTAG00000012956	0.00E+00	1.00E+00	0.00E+00	1.00E+00	-5.13E-15	1.00E+00	0.00E+00	1.00E+00	0.00E+00	1.00E+00
ENSBTAG00000047014	0.00E+00	1.00E+00	2.79E+00	2.10E-01	-5.13E-15	1.00E+00	0.00E+00	1.00E+00	0.00E+00	1.00E+00
ENSBTAG00000046934	0.00E+00	1.00E+00	0.00E+00	1.00E+00	2.56E+00	2.54E-01	0.00E+00	1.00E+00	0.00E+00	1.00E+00
ENSBTAG00000031832	0.00E+00	1.00E+00	0.00E+00	1.00E+00	-5.13E-15	1.00E+00	0.00E+00	1.00E+00	0.00E+00	1.00E+00
ENSBTAG00000048194	0.00E+00	1.00E+00	0.00E+00	1.00E+00	-5.13E-15	1.00E+00	0.00E+00	1.00E+00	0.00E+00	1.00E+00
ENSBTAG00000047644	5.25E-03	1.00E+00	1.39E+00	5.18E-01	-1.91E+00	4.28E-01	-1.54E-01	1.00E+00	-1.91E+00	8.00E-01
ENSBTAG00000047763	0.00E+00	1.00E+00	0.00E+00	1.00E+00	-5.13E-15	1.00E+00	0.00E+00	1.00E+00	0.00E+00	1.00E+00
ENSBTAG00000031835	0.00E+00	1.00E+00	0.00E+00	1.00E+00	-5.13E-15	1.00E+00	0.00E+00	1.00E+00	0.00E+00	1.00E+00
ENSBTAG00000046892	0.00E+00	1.00E+00	0.00E+00	1.00E+00	-5.13E-15	1.00E+00	0.00E+00	1.00E+00	0.00E+00	1.00E+00
ENSBTAG00000047829	0.00E+00	1.00E+00	0.00E+00	1.00E+00	-5.13E-15	1.00E+00	0.00E+00	1.00E+00	0.00E+00	1.00E+00
ENSBTAG00000048019	5.10E-01	1.00E+00	1.27E+00	4.43E-01	-2.71E+00	1.99E-01	-9.45E-01	8.04E-01	8.92E-01	1.00E+00
ENSBTAG00000039390	0.00E+00	1.00E+00	0.00E+00	1.00E+00	-5.13E-15	1.00E+00	0.00E+00	1.00E+00	0.00E+00	1.00E+00
ENSBTAG00000040188	-4.87E-01	1.00E+00	-4.18E-01	1.00E+00	-6.49E-01	9.00E-01	-1.80E-01	1.00E+00	-4.88E-01	1.00E+00
ENSBTAG00000039683	3.91E+00	1.40E-01	0.00E+00	1.00E+00	-5.13E-15	1.00E+00	0.00E+00	1.00E+00	0.00E+00	1.00E+00
ENSBTAG00000039116	0.00E+00	1.00E+00	0.00E+00	1.00E+00	-5.13E-15	1.00E+00	0.00E+00	1.00E+00	0.00E+00	1.00E+00
ENSBTAG00000039562	-1.91E+00	8.23E-01	-1.91E+00	4.69E-01	-1.91E+00	4.28E-01	-1.91E+00	4.39E-01	-1.91E+00	8.00E-01
ENSBTAG00000039901	0.00E+00	1.00E+00	0.00E+00	1.00E+00	-5.13E-15	1.00E+00	0.00E+00	1.00E+00	0.00E+00	1.00E+00
ENSBTAG00000038472	0.00E+00	1.00E+00	0.00E+00	1.00E+00	-5.13E-15	1.00E+00	0.00E+00	1.00E+00	0.00E+00	1.00E+00
ENSBTAG00000013654	0.00E+00	1.00E+00	0.00E+00	1.00E+00	-5.13E-15	1.00E+00	0.00E+00	1.00E+00	0.00E+00	1.00E+00
ENSBTAG00000031843	0.00E+00	1.00E+00	0.00E+00	1.00E+00	-5.13E-15	1.00E+00	0.00E+00	1.00E+00	0.00E+00	1.00E+00

ENSBTAG00000039534	0.00E+00	1.00E+00	0.00E+00	1.00E+00	-5.13E-15	1.00E+00	0.00E+00	1.00E+00	0.00E+00	1.00E+00
ENSBTAG00000046777	0.00E+00	1.00E+00	0.00E+00	1.00E+00	-5.13E-15	1.00E+00	0.00E+00	1.00E+00	0.00E+00	1.00E+00
ENSBTAG00000047086	0.00E+00	1.00E+00	0.00E+00	1.00E+00	-5.13E-15	1.00E+00	1.76E+00	4.97E-01	0.00E+00	1.00E+00
ENSBTAG00000046023	0.00E+00	1.00E+00	0.00E+00	1.00E+00	-5.13E-15	1.00E+00	1.77E+00	4.97E-01	0.00E+00	1.00E+00
ENSBTAG00000038608	0.00E+00	1.00E+00	0.00E+00	1.00E+00	-5.13E-15	1.00E+00	0.00E+00	1.00E+00	0.00E+00	1.00E+00
ENSBTAG00000000228	0.00E+00	1.00E+00	1.98E+00	4.43E-01	-5.13E-15	1.00E+00	0.00E+00	1.00E+00	0.00E+00	1.00E+00
ENSBTAG00000037628	0.00E+00	1.00E+00	0.00E+00	1.00E+00	-5.13E-15	1.00E+00	1.77E+00	4.97E-01	0.00E+00	1.00E+00
ENSBTAG00000031850	0.00E+00	1.00E+00	0.00E+00	1.00E+00	-5.13E-15	1.00E+00	0.00E+00	1.00E+00	0.00E+00	1.00E+00
ENSBTAG00000006339	-2.57E-01	1.00E+00	-6.90E-01	3.72E-01	-1.01E+00	1.27E-01	-1.49E+00	1.94E-02	-5.66E-01	8.32E-01
ENSBTAG00000027279	-4.50E-02	1.00E+00	4.14E-01	2.87E-01	5.32E-01	1.30E-01	4.86E-01	1.90E-01	-3.98E-01	7.43E-01
ENSBTAG00000027955	1.92E+00	8.23E-01	0.00E+00	1.00E+00	-5.13E-15	1.00E+00	0.00E+00	1.00E+00	0.00E+00	1.00E+00
ENSBTAG00000040582	0.00E+00	1.00E+00	0.00E+00	1.00E+00	-5.13E-15	1.00E+00	0.00E+00	1.00E+00	0.00E+00	1.00E+00
ENSBTAG00000047558	0.00E+00	1.00E+00	0.00E+00	1.00E+00	-5.13E-15	1.00E+00	0.00E+00	1.00E+00	0.00E+00	1.00E+00
ENSBTAG00000040280	1.91E+00	8.23E-01	0.00E+00	1.00E+00	-5.13E-15	1.00E+00	0.00E+00	1.00E+00	0.00E+00	1.00E+00
ENSBTAG00000038562	1.91E+00	8.23E-01	0.00E+00	1.00E+00	1.78E+00	4.79E-01	0.00E+00	1.00E+00	0.00E+00	1.00E+00
ENSBTAG00000038928	0.00E+00	1.00E+00	0.00E+00	1.00E+00	-5.13E-15	1.00E+00	0.00E+00	1.00E+00	0.00E+00	1.00E+00
ENSBTAG00000038444	0.00E+00	1.00E+00	0.00E+00	1.00E+00	-5.13E-15	1.00E+00	0.00E+00	1.00E+00	0.00E+00	1.00E+00
ENSBTAG00000047736	0.00E+00	1.00E+00	0.00E+00	1.00E+00	-5.13E-15	1.00E+00	0.00E+00	1.00E+00	0.00E+00	1.00E+00
ENSBTAG00000039052	1.26E-02	1.00E+00	-1.91E+00	4.69E-01	-1.91E+00	4.28E-01	-1.91E+00	4.39E-01	-1.91E+00	8.00E-01
ENSBTAG00000040064	0.00E+00	1.00E+00	0.00E+00	1.00E+00	-5.13E-15	1.00E+00	0.00E+00	1.00E+00	0.00E+00	1.00E+00
ENSBTAG00000037577	0.00E+00	1.00E+00	0.00E+00	1.00E+00	-5.13E-15	1.00E+00	0.00E+00	1.00E+00	0.00E+00	1.00E+00
ENSBTAG00000040187	0.00E+00	1.00E+00	0.00E+00	1.00E+00	-5.13E-15	1.00E+00	1.77E+00	4.97E-01	0.00E+00	1.00E+00
ENSBTAG00000040026	0.00E+00	1.00E+00	0.00E+00	1.00E+00	-5.13E-15	1.00E+00	0.00E+00	1.00E+00	0.00E+00	1.00E+00
ENSBTAG00000040457	-3.23E+00	3.75E-01	-3.23E+00	1.24E-01	-3.23E+00	9.34E-02	-3.23E+00	9.81E-02	-3.23E+00	4.34E-01
ENSBTAG00000015175	0.00E+00	1.00E+00	1.98E+00	4.43E-01	1.78E+00	4.79E-01	0.00E+00	1.00E+00	0.00E+00	1.00E+00
ENSBTAG00000000214	2.70E+00	5.76E-01	0.00E+00	1.00E+00	-5.13E-15	1.00E+00	0.00E+00	1.00E+00	2.71E+00	6.01E-01
ENSBTAG00000047585	0.00E+00	1.00E+00	0.00E+00	1.00E+00	-5.13E-15	1.00E+00	0.00E+00	1.00E+00	0.00E+00	1.00E+00
ENSBTAG00000008349	2.89E-02	1.00E+00	-2.74E+00	2.13E-14	-4.16E+00	4.18E-28	-4.31E+00	1.58E-29	-2.96E-01	9.36E-01
ENSBTAG00000047789	1.59E-02	1.00E+00	-4.26E-01	1.00E+00	9.64E-01	5.34E-01	-1.80E-01	1.00E+00	6.68E-01	1.00E+00
ENSBTAG00000000871	6.73E-01	3.21E-03	1.62E+00	3.61E-19	1.40E+00	1.06E-14	9.27E-01	6.63E-07	1.01E-01	1.00E+00
ENSBTAG00000045477	0.00E+00	1.00E+00	0.00E+00	1.00E+00	-5.13E-15	1.00E+00	0.00E+00	1.00E+00	0.00E+00	1.00E+00
ENSBTAG00000035959	-1.84E-01	1.00E+00	6.73E-02	1.00E+00	-4.27E-02	1.00E+00	1.87E-02	1.00E+00	-6.04E-01	1.60E-01
ENSBTAG00000046381	0.00E+00	1.00E+00	0.00E+00	1.00E+00	-5.13E-15	1.00E+00	0.00E+00	1.00E+00	0.00E+00	1.00E+00
ENSBTAG00000047775	0.00E+00	1.00E+00	0.00E+00	1.00E+00	-5.13E-15	1.00E+00	0.00E+00	1.00E+00	0.00E+00	1.00E+00
ENSBTAG00000038782	0.00E+00	1.00E+00	0.00E+00	1.00E+00	-5.13E-15	1.00E+00	0.00E+00	1.00E+00	0.00E+00	1.00E+00

ENSBTAG00000008943	-3.56E-01	1.00E+00	-1.39E+00	2.55E-01	5.36E-01	6.77E-01	1.29E+00	7.79E-02	8.90E-01	7.36E-01
ENSBTAG00000037988	6.86E-01	9.30E-01	8.77E-01	3.90E-01	1.24E+00	1.15E-01	3.54E-01	9.46E-01	1.32E+00	4.09E-01
ENSBTAG00000046573	-5.05E-01	7.80E-01	-5.96E-01	2.64E-01	-4.60E-01	4.26E-01	-1.07E+00	2.16E-02	-6.96E-02	1.00E+00
ENSBTAG00000045403	0.00E+00	1.00E+00	0.00E+00	1.00E+00	-5.13E-15	1.00E+00	0.00E+00	1.00E+00	0.00E+00	1.00E+00
ENSBTAG00000031869	-3.00E-01	9.13E-01	-9.39E-02	1.00E+00	2.24E-01	7.06E-01	-6.15E-01	1.00E-01	3.19E-01	8.18E-01
ENSBTAG00000043779	0.00E+00	1.00E+00	0.00E+00	1.00E+00	-5.13E-15	1.00E+00	0.00E+00	1.00E+00	0.00E+00	1.00E+00
ENSBTAG00000031871	2.96E-01	1.00E+00	4.93E-01	5.36E-01	2.62E-01	9.07E-01	-1.17E-01	1.00E+00	-1.83E-01	1.00E+00
ENSBTAG00000003575	-3.99E-01	1.00E+00	-2.60E-02	1.00E+00	-1.77E-01	1.00E+00	2.88E-01	9.64E-01	6.73E-01	8.00E-01
ENSBTAG00000040168	0.00E+00	1.00E+00	0.00E+00	1.00E+00	-5.13E-15	1.00E+00	0.00E+00	1.00E+00	0.00E+00	1.00E+00
ENSBTAG00000002641	4.38E-01	2.54E-01	1.48E+00	2.66E-13	1.44E+00	9.15E-13	1.20E+00	5.45E-09	9.79E-02	1.00E+00
ENSBTAG00000007074	3.19E-02	1.00E+00	9.79E-01	1.72E-05	3.33E-01	2.45E-01	-4.87E-01	6.32E-02	-3.83E-02	1.00E+00
ENSBTAG00000031873	-3.89E-02	1.00E+00	1.08E+00	1.86E-04	7.03E-01	2.20E-02	-2.68E-01	5.66E-01	-2.85E-02	1.00E+00
ENSBTAG00000031874	-3.29E-01	1.00E+00	1.06E+00	2.10E-02	1.17E+00	7.50E-03	8.39E-01	9.09E-02	-6.68E-01	6.88E-01
ENSBTAG00000013384	0.00E+00	1.00E+00	0.00E+00	1.00E+00	-5.13E-15	1.00E+00	3.03E+00	1.50E-01	0.00E+00	1.00E+00
ENSBTAG00000039079	0.00E+00	1.00E+00	0.00E+00	1.00E+00	-5.13E-15	1.00E+00	0.00E+00	1.00E+00	0.00E+00	1.00E+00
ENSBTAG00000038908	0.00E+00	1.00E+00	0.00E+00	1.00E+00	-5.13E-15	1.00E+00	0.00E+00	1.00E+00	0.00E+00	1.00E+00
ENSBTAG00000031881	0.00E+00	1.00E+00	0.00E+00	1.00E+00	-5.13E-15	1.00E+00	0.00E+00	1.00E+00	0.00E+00	1.00E+00
ENSBTAG00000040454	0.00E+00	1.00E+00	0.00E+00	1.00E+00	-5.13E-15	1.00E+00	0.00E+00	1.00E+00	0.00E+00	1.00E+00
ENSBTAG00000040583	0.00E+00	1.00E+00	0.00E+00	1.00E+00	-5.13E-15	1.00E+00	0.00E+00	1.00E+00	0.00E+00	1.00E+00
ENSBTAG00000038983	0.00E+00	1.00E+00	0.00E+00	1.00E+00	-5.13E-15	1.00E+00	0.00E+00	1.00E+00	0.00E+00	1.00E+00
ENSBTAG00000040171	0.00E+00	1.00E+00	0.00E+00	1.00E+00	-5.13E-15	1.00E+00	0.00E+00	1.00E+00	0.00E+00	1.00E+00
ENSBTAG00000040307	0.00E+00	1.00E+00	0.00E+00	1.00E+00	-5.13E-15	1.00E+00	0.00E+00	1.00E+00	0.00E+00	1.00E+00
ENSBTAG00000005885	0.00E+00	1.00E+00	0.00E+00	1.00E+00	-5.13E-15	1.00E+00	0.00E+00	1.00E+00	0.00E+00	1.00E+00
ENSBTAG00000038597	0.00E+00	1.00E+00	0.00E+00	1.00E+00	-5.13E-15	1.00E+00	0.00E+00	1.00E+00	0.00E+00	1.00E+00
ENSBTAG00000039677	0.00E+00	1.00E+00	0.00E+00	1.00E+00	-5.13E-15	1.00E+00	0.00E+00	1.00E+00	0.00E+00	1.00E+00
ENSBTAG00000039145	-1.91E+00	8.23E-01	-1.91E+00	4.69E-01	-1.91E+00	4.28E-01	-1.91E+00	4.39E-01	-1.91E+00	8.00E-01
ENSBTAG00000039274	1.92E+00	8.23E-01	0.00E+00	1.00E+00	1.78E+00	4.79E-01	1.76E+00	4.97E-01	0.00E+00	1.00E+00
ENSBTAG00000024188	0.00E+00	1.00E+00	0.00E+00	1.00E+00	1.78E+00	4.79E-01	1.76E+00	4.97E-01	0.00E+00	1.00E+00
ENSBTAG00000024187	-1.91E+00	8.23E-01	7.51E-02	1.00E+00	-1.91E+00	4.28E-01	-1.91E+00	4.39E-01	-1.91E+00	8.00E-01
ENSBTAG00000024186	0.00E+00	1.00E+00	1.98E+00	4.43E-01	-5.13E-15	1.00E+00	0.00E+00	1.00E+00	1.91E+00	8.00E-01
ENSBTAG00000024185	0.00E+00	1.00E+00	0.00E+00	1.00E+00	1.78E+00	4.79E-01	0.00E+00	1.00E+00	0.00E+00	1.00E+00
ENSBTAG00000024184	-1.91E+00	8.23E-01	-1.91E+00	4.69E-01	-1.91E+00	4.28E-01	-1.91E+00	4.39E-01	-1.91E+00	8.00E-01
ENSBTAG00000024183	1.92E+00	8.23E-01	0.00E+00	1.00E+00	-5.13E-15	1.00E+00	0.00E+00	1.00E+00	0.00E+00	1.00E+00
ENSBTAG00000024182	1.07E+00	9.69E-01	1.49E+00	3.91E-01	1.68E+00	2.95E-01	2.46E+00	9.14E-02	1.31E+00	8.07E-01
ENSBTAG00000031886	2.19E-01	1.00E+00	3.37E-01	9.45E-01	9.01E-01	3.50E-01	1.44E+00	8.65E-02	-5.93E-02	1.00E+00

ENSBTAG00000024180	0.00E+00	1.00E+00	2.79E+00	2.10E-01	1.78E+00	4.79E-01	0.00E+00	1.00E+00	1.91E+00	8.00E-01
ENSBTAG00000024179	0.00E+00	1.00E+00	1.98E+00	4.43E-01	-5.13E-15	1.00E+00	0.00E+00	1.00E+00	0.00E+00	1.00E+00
ENSBTAG00000024178	0.00E+00	1.00E+00	0.00E+00	1.00E+00	-5.13E-15	1.00E+00	1.76E+00	4.97E-01	2.71E+00	6.01E-01
ENSBTAG00000024177	2.70E+00	5.76E-01	2.79E+00	2.10E-01	3.04E+00	1.38E-01	3.70E+00	4.69E-02	2.71E+00	6.01E-01
ENSBTAG00000024176	0.00E+00	1.00E+00	1.98E+00	4.43E-01	-5.13E-15	1.00E+00	0.00E+00	1.00E+00	1.91E+00	8.00E-01
ENSBTAG00000024175	0.00E+00	1.00E+00	0.00E+00	1.00E+00	-5.13E-15	1.00E+00	0.00E+00	1.00E+00	0.00E+00	1.00E+00
ENSBTAG00000031888	-1.91E+00	8.23E-01	-1.91E+00	4.69E-01	-1.91E+00	4.28E-01	-1.42E-01	1.00E+00	-1.91E+00	8.00E-01
ENSBTAG00000031889	2.72E+00	5.76E-01	4.23E+00	1.30E-02	5.02E+00	4.70E-04	5.01E+00	6.09E-04	3.24E+00	4.34E-01
ENSBTAG00000045181	-1.91E+00	8.23E-01	-1.91E+00	4.69E-01	-1.91E+00	4.28E-01	-1.91E+00	4.39E-01	-1.91E+00	8.00E-01
ENSBTAG00000044946	0.00E+00	1.00E+00	0.00E+00	1.00E+00	-5.13E-15	1.00E+00	0.00E+00	1.00E+00	0.00E+00	1.00E+00
ENSBTAG00000011498	3.28E-02	1.00E+00	-1.24E-01	1.00E+00	4.02E-04	1.00E+00	1.46E-01	1.00E+00	1.47E-02	1.00E+00
ENSBTAG00000009152	-2.44E-02	1.00E+00	-1.17E+00	4.45E-02	7.56E-01	1.64E-01	1.28E+00	6.84E-03	2.85E-01	1.00E+00
ENSBTAG00000024169	1.91E+00	8.23E-01	0.00E+00	1.00E+00	-5.13E-15	1.00E+00	0.00E+00	1.00E+00	0.00E+00	1.00E+00
ENSBTAG00000012461	-5.22E-01	1.00E+00	-3.23E+00	1.24E-01	-3.23E+00	9.36E-02	-3.23E+00	9.83E-02	-1.31E+00	8.99E-01
ENSBTAG00000040378	0.00E+00	1.00E+00	0.00E+00	1.00E+00	2.56E+00	2.54E-01	2.53E+00	2.71E-01	1.91E+00	8.00E-01
ENSBTAG00000038173	-7.80E-01	1.00E+00	-2.70E+00	2.42E-01	3.54E-01	1.00E+00	3.30E-01	1.00E+00	8.87E-01	1.00E+00
ENSBTAG00000046096	1.92E+00	8.23E-01	0.00E+00	1.00E+00	-5.13E-15	1.00E+00	3.03E+00	1.50E-01	1.93E+00	8.00E-01
ENSBTAG00000039492	0.00E+00	1.00E+00	1.98E+00	4.43E-01	-5.13E-15	1.00E+00	0.00E+00	1.00E+00	0.00E+00	1.00E+00
ENSBTAG00000038277	9.81E-03	1.00E+00	-2.70E+00	2.42E-01	-2.70E+00	1.99E-01	-1.70E-01	1.00E+00	-7.86E-01	1.00E+00
ENSBTAG00000044338	0.00E+00	1.00E+00	0.00E+00	1.00E+00	-5.13E-15	1.00E+00	0.00E+00	1.00E+00	0.00E+00	1.00E+00
ENSBTAG00000031741	4.06E-01	6.15E-01	9.74E-01	4.47E-04	1.46E+00	1.40E-08	1.34E+00	3.35E-07	2.38E-01	9.48E-01
ENSBTAG00000037597	0.00E+00	1.00E+00	0.00E+00	1.00E+00	1.78E+00	4.79E-01	0.00E+00	1.00E+00	0.00E+00	1.00E+00
ENSBTAG00000027547	0.00E+00	1.00E+00	1.99E+00	4.43E-01	1.78E+00	4.79E-01	1.76E+00	4.97E-01	0.00E+00	1.00E+00
ENSBTAG00000010784	2.71E-01	7.44E-01	1.68E+00	2.76E-17	1.64E+00	1.34E-16	9.90E-01	1.55E-06	-1.46E-01	1.00E+00
ENSBTAG00000031746	1.57E-03	1.00E+00	2.71E-01	5.55E-01	3.35E-01	4.17E-01	6.31E-01	5.39E-02	2.97E-02	1.00E+00
ENSBTAG00000031747	-5.84E-02	1.00E+00	-6.88E-01	3.30E-04	-1.00E+00	6.35E-08	-1.04E+00	2.44E-08	-3.02E-01	5.61E-01
ENSBTAG00000010852	5.54E-02	1.00E+00	-5.29E-02	1.00E+00	-5.09E-01	2.34E-01	-1.02E+00	7.29E-03	2.66E-02	1.00E+00
ENSBTAG00000031753	0.00E+00	1.00E+00	0.00E+00	1.00E+00	-5.13E-15	1.00E+00	0.00E+00	1.00E+00	0.00E+00	1.00E+00
ENSBTAG00000031756	-1.14E+00	7.90E-01	-2.91E-01	1.00E+00	3.13E-01	9.88E-01	1.28E+00	1.14E-01	-2.81E+00	2.05E-01
ENSBTAG00000031757	0.00E+00	1.00E+00	0.00E+00	1.00E+00	2.56E+00	2.54E-01	2.53E+00	2.71E-01	1.91E+00	8.00E-01
ENSBTAG00000031758	1.91E+00	8.23E-01	0.00E+00	1.00E+00	2.55E+00	2.55E-01	2.53E+00	2.71E-01	0.00E+00	1.00E+00
ENSBTAG00000031759	0.00E+00	1.00E+00	1.99E+00	4.43E-01	-5.13E-15	1.00E+00	0.00E+00	1.00E+00	0.00E+00	1.00E+00
ENSBTAG00000031760	1.92E+00	8.23E-01	0.00E+00	1.00E+00	1.78E+00	4.79E-01	0.00E+00	1.00E+00	1.91E+00	8.00E-01
ENSBTAG00000031761	0.00E+00	1.00E+00	3.30E+00	1.02E-01	-5.13E-15	1.00E+00	2.53E+00	2.71E-01	0.00E+00	1.00E+00
ENSBTAG00000031762	3.81E-01	1.00E+00	2.20E+00	2.38E-02	2.10E+00	2.94E-02	-1.76E-01	1.00E+00	-4.87E-01	1.00E+00

ENSBTAG00000031763	9.03E-01	1.00E+00	2.47E+00	6.95E-02	1.37E+00	4.49E-01	1.88E+00	2.25E-01	4.89E-01	1.00E+00
ENSBTAG00000039163	-5.42E-01	9.57E-01	-3.66E-01	8.41E-01	-1.09E+00	1.41E-01	-2.87E-01	9.51E-01	1.49E+00	2.00E-01
ENSBTAG00000031766	1.91E+00	8.24E-01	6.68E+00	1.22E-07	4.80E+00	2.38E-03	5.52E+00	1.36E-04	3.20E+00	4.46E-01
ENSBTAG00000031767	6.41E-01	1.00E+00	3.30E+00	4.09E-05	2.41E+00	4.10E-03	2.14E+00	1.54E-02	1.18E+00	6.71E-01
ENSBTAG00000031768	1.90E-01	1.00E+00	4.50E+00	1.86E-08	3.70E+00	4.76E-06	3.47E+00	2.76E-05	2.24E+00	1.26E-01
ENSBTAG00000031769	1.44E-02	1.00E+00	5.98E-01	9.50E-01	3.48E-01	1.00E+00	2.44E+00	2.71E-02	1.32E-02	1.00E+00
ENSBTAG00000031770	1.91E+00	8.23E-01	0.00E+00	1.00E+00	-5.13E-15	1.00E+00	2.53E+00	2.71E-01	0.00E+00	1.00E+00
ENSBTAG00000031771	-1.23E+00	8.77E-01	1.58E+00	1.40E-01	1.68E+00	9.58E-02	1.80E+00	7.16E-02	2.32E+00	9.37E-02
ENSBTAG00000033117	0.00E+00	1.00E+00	0.00E+00	1.00E+00	-5.13E-15	1.00E+00	0.00E+00	1.00E+00	0.00E+00	1.00E+00
ENSBTAG00000031774	0.00E+00	1.00E+00	0.00E+00	1.00E+00	-5.13E-15	1.00E+00	0.00E+00	1.00E+00	0.00E+00	1.00E+00
ENSBTAG00000040277	-2.73E-01	1.00E+00	-5.20E-01	2.82E-01	-1.22E+00	2.32E-03	-1.12E+00	6.18E-03	-2.18E-02	1.00E+00
ENSBTAG00000031776	-1.91E+00	8.23E-01	2.32E+00	1.13E-01	-1.30E-01	1.00E+00	6.26E-01	9.94E-01	8.10E-01	1.00E+00
ENSBTAG00000031778	2.63E-01	9.42E-01	1.43E+00	5.68E-07	1.79E+00	1.92E-10	1.99E+00	1.83E-12	1.21E+00	9.78E-04
ENSBTAG00000047206	-1.63E+00	4.69E-01	-2.06E+00	8.46E-02	-1.83E-01	1.00E+00	8.84E-01	3.37E-01	-9.60E-01	8.00E-01
ENSBTAG00000039657	5.39E-01	1.00E+00	4.96E+00	2.83E-12	4.98E+00	1.86E-12	4.95E+00	3.48E-12	8.33E-01	7.69E-01
ENSBTAG00000031785	-1.20E-01	1.00E+00	2.37E+00	7.51E-06	2.27E+00	1.71E-05	2.18E+00	4.46E-05	1.23E+00	1.95E-01
ENSBTAG00000025391	-2.89E-01	1.00E+00	2.15E+00	5.51E-05	1.98E+00	2.17E-04	1.61E+00	3.60E-03	5.55E-01	8.00E-01
ENSBTAG00000011671	0.00E+00	1.00E+00	0.00E+00	1.00E+00	-5.13E-15	1.00E+00	0.00E+00	1.00E+00	0.00E+00	1.00E+00
ENSBTAG00000013065	-5.64E-01	5.88E-01	-1.33E+00	8.90E-04	-1.35E+00	6.32E-04	-1.29E+00	1.36E-03	2.91E-01	9.93E-01
ENSBTAG00000011677	-7.40E-01	8.08E-01	-1.43E+00	5.51E-02	4.00E-01	7.32E-01	2.50E+00	4.88E-06	1.04E+00	4.34E-01
ENSBTAG00000031717	0.00E+00	1.00E+00	3.68E+00	5.08E-02	1.78E+00	4.79E-01	2.53E+00	2.71E-01	2.71E+00	6.01E-01
ENSBTAG00000007286	2.47E+00	2.91E-01	8.83E-01	8.52E-01	1.16E+00	6.43E-01	1.78E+00	3.09E-01	-1.91E+00	8.00E-01
ENSBTAG00000031780	-1.91E+00	8.23E-01	-1.91E+00	4.69E-01	-1.91E+00	4.28E-01	-1.91E+00	4.39E-01	-1.91E+00	8.00E-01
ENSBTAG00000031738	1.94E+00	6.50E-01	6.80E+00	3.78E-12	5.55E+00	5.43E-08	5.57E+00	6.32E-08	2.61E+00	3.50E-01
ENSBTAG00000031773	3.90E+00	1.83E-01	8.71E+00	1.70E-11	7.91E+00	2.15E-09	7.18E+00	2.02E-07	5.10E+00	2.84E-02
ENSBTAG00000039812	1.95E-01	1.00E+00	1.73E+00	8.80E-03	1.77E+00	7.20E-03	1.71E+00	1.15E-02	1.74E+00	8.59E-02
ENSBTAG00000031719	1.92E+00	8.23E-01	3.30E+00	1.02E-01	3.06E+00	1.36E-01	3.94E+00	2.66E-02	2.71E+00	6.01E-01
ENSBTAG00000031721	0.00E+00	1.00E+00	3.31E+00	1.02E-01	1.78E+00	4.79E-01	0.00E+00	1.00E+00	0.00E+00	1.00E+00
ENSBTAG00000046621	-3.88E+00	1.45E-01	3.49E-01	1.00E+00	4.57E-01	9.07E-01	-8.52E-01	6.69E-01	2.53E-01	1.00E+00
ENSBTAG00000018523	1.46E-01	1.00E+00	3.75E-01	3.57E-01	4.33E-01	2.54E-01	3.35E-01	4.41E-01	-9.23E-02	1.00E+00
ENSBTAG00000002642	1.67E-01	1.00E+00	-1.87E-01	7.55E-01	-1.63E+00	1.64E-09	-2.25E+00	2.24E-16	2.71E-01	8.44E-01
ENSBTAG00000003797	2.09E-01	1.00E+00	-2.09E+00	2.82E-05	-3.10E+00	7.20E-10	-3.45E+00	1.67E-11	2.97E-01	1.00E+00
ENSBTAG00000037917	-2.79E-01	8.23E-01	-8.62E-01	7.96E-04	-1.67E+00	1.32E-11	-1.97E+00	2.21E-15	-1.26E-01	1.00E+00
ENSBTAG00000037738	-6.43E-02	1.00E+00	-5.35E-01	5.37E-02	-1.14E+00	4.18E-06	-1.32E+00	1.35E-07	1.62E-01	1.00E+00
ENSBTAG00000046373	2.45E+00	2.91E-01	6.97E-02	1.00E+00	-1.32E-01	1.00E+00	-1.53E-01	1.00E+00	7.93E-01	1.00E+00

ENSBTAG00000025882	1.70E-02	1.00E+00	9.80E-01	6.50E-01	-9.18E-01	8.09E-01	-2.70E+00	2.05E-01	6.29E-03	1.00E+00
ENSBTAG00000048235	1.86E-02	1.00E+00	-7.17E-01	9.45E-01	-2.70E+00	1.99E-01	-2.70E+00	2.05E-01	-2.70E+00	6.01E-01
ENSBTAG00000046992	1.19E+00	8.56E-01	1.53E+00	2.99E-01	3.50E-01	1.00E+00	-2.70E+00	2.05E-01	6.95E-03	1.00E+00
ENSBTAG00000019926	-1.02E-01	1.00E+00	1.16E+00	3.53E-04	-1.31E+00	3.13E-04	-2.27E+00	3.23E-09	-4.52E-01	7.24E-01
ENSBTAG00000016549	-1.40E-01	1.00E+00	-1.29E+00	5.22E-05	-2.39E+00	4.31E-14	-1.45E+00	5.38E-06	-4.73E-02	1.00E+00
ENSBTAG00000046581	0.00E+00	1.00E+00	0.00E+00	1.00E+00	-5.13E-15	1.00E+00	0.00E+00	1.00E+00	0.00E+00	1.00E+00
ENSBTAG00000038783	0.00E+00	1.00E+00	0.00E+00	1.00E+00	-5.13E-15	1.00E+00	0.00E+00	1.00E+00	0.00E+00	1.00E+00
ENSBTAG00000008491	7.20E-02	1.00E+00	4.56E-02	1.00E+00	3.41E-01	2.53E-01	6.82E-01	6.43E-03	2.10E-01	9.14E-01
ENSBTAG00000003892	1.44E+00	3.44E-02	2.96E+00	6.77E-09	2.44E+00	1.36E-06	2.10E+00	3.96E-05	6.03E-01	7.80E-01
ENSBTAG00000018165	2.34E-01	1.00E+00	3.70E-01	4.04E-01	5.61E-01	1.26E-01	3.40E-01	4.64E-01	2.63E-01	9.50E-01
ENSBTAG00000043012	0.00E+00	1.00E+00	0.00E+00	1.00E+00	-5.13E-15	1.00E+00	0.00E+00	1.00E+00	0.00E+00	1.00E+00
ENSBTAG00000017329	-3.89E-01	5.30E-01	-9.59E-01	1.61E-04	-1.52E-01	7.91E-01	2.86E-01	3.85E-01	-3.00E-01	7.77E-01
ENSBTAG00000020496	7.72E-02	1.00E+00	-2.03E-01	4.25E-01	-2.93E-02	1.00E+00	-2.26E-01	3.56E-01	-4.00E-01	2.49E-01
ENSBTAG00000016337	-1.00E-01	1.00E+00	-5.12E-01	1.06E-02	-1.33E+00	5.62E-13	-1.76E+00	3.43E-21	-3.70E-01	3.81E-01
ENSBTAG00000000365	-1.95E-01	9.09E-01	-1.02E+00	6.48E-07	4.59E-02	1.00E+00	1.84E-01	5.78E-01	-2.61E-01	7.60E-01
ENSBTAG00000021903	-1.46E-01	1.00E+00	-4.61E-01	8.78E-01	-1.72E-01	1.00E+00	7.72E-02	1.00E+00	9.85E-01	6.32E-01
ENSBTAG00000021902	3.21E-04	1.00E+00	-8.53E-02	1.00E+00	-7.77E-01	4.05E-03	-9.13E-01	6.93E-04	3.42E-01	7.22E-01
ENSBTAG00000004982	7.93E-01	5.84E-03	-4.02E-01	1.43E-01	-1.41E+00	7.22E-10	-1.58E+00	6.41E-12	1.07E+00	1.48E-04
ENSBTAG00000004979	7.45E-01	8.44E-04	-2.05E-01	4.59E-01	-5.23E-01	9.67E-03	-5.34E-01	9.43E-03	5.06E-03	1.00E+00
ENSBTAG00000042587	0.00E+00	1.00E+00	0.00E+00	1.00E+00	-5.13E-15	1.00E+00	0.00E+00	1.00E+00	0.00E+00	1.00E+00
ENSBTAG00000009798	8.20E-01	2.68E-02	-6.11E-02	1.00E+00	-9.06E-01	1.99E-03	5.36E-01	9.61E-02	1.36E+00	2.28E-05
ENSBTAG00000043143	0.00E+00	1.00E+00	0.00E+00	1.00E+00	-5.13E-15	1.00E+00	0.00E+00	1.00E+00	0.00E+00	1.00E+00
ENSBTAG00000009180	-1.16E+00	8.75E-01	-5.73E-01	8.94E-01	-1.33E+00	4.15E-01	-4.83E-01	9.42E-01	4.74E-03	1.00E+00
ENSBTAG00000045279	0.00E+00	1.00E+00	0.00E+00	1.00E+00	-5.13E-15	1.00E+00	0.00E+00	1.00E+00	0.00E+00	1.00E+00
ENSBTAG00000039810	1.92E+00	8.23E-01	1.98E+00	4.43E-01	-5.13E-15	1.00E+00	1.76E+00	4.97E-01	0.00E+00	1.00E+00
ENSBTAG00000047106	0.00E+00	1.00E+00	1.98E+00	4.43E-01	-5.13E-15	1.00E+00	0.00E+00	1.00E+00	0.00E+00	1.00E+00
ENSBTAG00000038430	-2.13E-02	1.00E+00	-2.96E-01	3.76E-01	-1.33E-01	8.72E-01	-4.19E-02	1.00E+00	-2.16E-01	9.11E-01
ENSBTAG00000025304	0.00E+00	1.00E+00	1.98E+00	4.43E-01	-5.13E-15	1.00E+00	0.00E+00	1.00E+00	0.00E+00	1.00E+00
ENSBTAG00000043201	0.00E+00	1.00E+00	0.00E+00	1.00E+00	-5.13E-15	1.00E+00	0.00E+00	1.00E+00	0.00E+00	1.00E+00
ENSBTAG00000042150	0.00E+00	1.00E+00	0.00E+00	1.00E+00	-5.13E-15	1.00E+00	0.00E+00	1.00E+00	0.00E+00	1.00E+00
ENSBTAG00000025314	1.91E+00	8.23E-01	0.00E+00	1.00E+00	1.78E+00	4.79E-01	1.77E+00	4.97E-01	0.00E+00	1.00E+00
ENSBTAG00000043055	0.00E+00	1.00E+00	0.00E+00	1.00E+00	-5.13E-15	1.00E+00	0.00E+00	1.00E+00	0.00E+00	1.00E+00
ENSBTAG00000025302	8.03E-01	1.00E+00	-1.91E+00	4.69E-01	-1.30E-01	1.00E+00	-1.46E-01	1.00E+00	7.17E-03	1.00E+00
ENSBTAG00000045324	0.00E+00	1.00E+00	0.00E+00	1.00E+00	-5.13E-15	1.00E+00	0.00E+00	1.00E+00	0.00E+00	1.00E+00
ENSBTAG00000043526	0.00E+00	1.00E+00	0.00E+00	1.00E+00	-5.13E-15	1.00E+00	0.00E+00	1.00E+00	0.00E+00	1.00E+00

ENSBTAG00000015827	0.00E+00	1.00E+00	0.00E+00	1.00E+00	-5.13E-15	1.00E+00	0.00E+00	1.00E+00	0.00E+00	1.00E+00
ENSBTAG00000042317	0.00E+00	1.00E+00	0.00E+00	1.00E+00	-5.13E-15	1.00E+00	0.00E+00	1.00E+00	0.00E+00	1.00E+00
ENSBTAG00000038718	0.00E+00	1.00E+00	0.00E+00	1.00E+00	-5.13E-15	1.00E+00	1.77E+00	4.97E-01	0.00E+00	1.00E+00
ENSBTAG00000047126	0.00E+00	1.00E+00	0.00E+00	1.00E+00	-5.13E-15	1.00E+00	0.00E+00	1.00E+00	0.00E+00	1.00E+00
ENSBTAG00000042344	0.00E+00	1.00E+00	0.00E+00	1.00E+00	-5.13E-15	1.00E+00	0.00E+00	1.00E+00	0.00E+00	1.00E+00
ENSBTAG00000047671	-1.91E+00	8.23E-01	7.57E-02	1.00E+00	-1.26E-01	1.00E+00	-1.48E-01	1.00E+00	-1.91E+00	8.00E-01
ENSBTAG00000046890	0.00E+00	1.00E+00	0.00E+00	1.00E+00	-5.13E-15	1.00E+00	0.00E+00	1.00E+00	0.00E+00	1.00E+00
ENSBTAG00000036106	1.91E+00	8.23E-01	0.00E+00	1.00E+00	-5.13E-15	1.00E+00	0.00E+00	1.00E+00	1.93E+00	8.00E-01
ENSBTAG00000042886	0.00E+00	1.00E+00	0.00E+00	1.00E+00	-5.13E-15	1.00E+00	0.00E+00	1.00E+00	0.00E+00	1.00E+00
ENSBTAG00000033993	2.72E+00	5.76E-01	0.00E+00	1.00E+00	-5.13E-15	1.00E+00	3.40E+00	8.35E-02	0.00E+00	1.00E+00
ENSBTAG00000047117	0.00E+00	1.00E+00	0.00E+00	1.00E+00	-5.13E-15	1.00E+00	0.00E+00	1.00E+00	0.00E+00	1.00E+00
ENSBTAG00000033513	1.21E-02	1.00E+00	-1.91E+00	4.69E-01	-1.91E+00	4.28E-01	1.49E+00	4.57E-01	-1.91E+00	8.00E-01
ENSBTAG00000047057	0.00E+00	1.00E+00	0.00E+00	1.00E+00	-5.13E-15	1.00E+00	0.00E+00	1.00E+00	0.00E+00	1.00E+00
ENSBTAG00000044980	0.00E+00	1.00E+00	0.00E+00	1.00E+00	-5.13E-15	1.00E+00	0.00E+00	1.00E+00	0.00E+00	1.00E+00
ENSBTAG00000043255	0.00E+00	1.00E+00	0.00E+00	1.00E+00	1.78E+00	4.79E-01	0.00E+00	1.00E+00	0.00E+00	1.00E+00
ENSBTAG00000025317	0.00E+00	1.00E+00	0.00E+00	1.00E+00	-5.13E-15	1.00E+00	1.76E+00	4.97E-01	1.91E+00	8.00E-01
ENSBTAG00000044735	0.00E+00	1.00E+00	0.00E+00	1.00E+00	-5.13E-15	1.00E+00	0.00E+00	1.00E+00	0.00E+00	1.00E+00
ENSBTAG00000031640	1.92E+00	8.23E-01	0.00E+00	1.00E+00	-5.13E-15	1.00E+00	2.53E+00	2.71E-01	1.91E+00	8.00E-01
ENSBTAG00000045456	0.00E+00	1.00E+00	0.00E+00	1.00E+00	-5.13E-15	1.00E+00	0.00E+00	1.00E+00	0.00E+00	1.00E+00
ENSBTAG00000043330	0.00E+00	1.00E+00	0.00E+00	1.00E+00	-5.13E-15	1.00E+00	0.00E+00	1.00E+00	0.00E+00	1.00E+00
ENSBTAG00000040136	0.00E+00	1.00E+00	0.00E+00	1.00E+00	1.78E+00	4.79E-01	0.00E+00	1.00E+00	0.00E+00	1.00E+00
ENSBTAG00000006761	1.91E+00	8.23E-01	1.98E+00	4.43E-01	1.78E+00	4.79E-01	0.00E+00	1.00E+00	1.91E+00	8.00E-01
ENSBTAG00000015274	0.00E+00	1.00E+00	0.00E+00	1.00E+00	-5.13E-15	1.00E+00	1.76E+00	4.97E-01	0.00E+00	1.00E+00
ENSBTAG00000015130	1.92E+00	8.23E-01	1.99E+00	4.43E-01	-5.13E-15	1.00E+00	0.00E+00	1.00E+00	0.00E+00	1.00E+00
ENSBTAG00000044561	0.00E+00	1.00E+00	0.00E+00	1.00E+00	-5.13E-15	1.00E+00	0.00E+00	1.00E+00	0.00E+00	1.00E+00
ENSBTAG00000042755	0.00E+00	1.00E+00	0.00E+00	1.00E+00	-5.13E-15	1.00E+00	0.00E+00	1.00E+00	0.00E+00	1.00E+00
ENSBTAG00000046556	-6.44E-02	1.00E+00	1.42E+00	1.00E-02	2.22E+00	1.91E-05	2.07E+00	9.20E-05	-3.33E-01	1.00E+00
ENSBTAG00000025589	1.97E-01	8.60E-01	2.92E-01	2.27E-01	9.53E-01	4.22E-07	8.75E-01	5.22E-06	-3.47E-01	4.72E-01
ENSBTAG00000021045	6.86E-02	1.00E+00	-4.43E-01	4.20E-01	4.55E-01	3.77E-01	4.44E-01	4.11E-01	-2.79E-01	1.00E+00
ENSBTAG00000028794	0.00E+00	1.00E+00	0.00E+00	1.00E+00	-5.13E-15	1.00E+00	0.00E+00	1.00E+00	0.00E+00	1.00E+00
ENSBTAG00000016519	5.67E-01	5.68E-01	1.18E+00	1.51E-03	6.93E-01	9.50E-02	5.88E-01	1.89E-01	-2.35E-01	1.00E+00
ENSBTAG00000043721	0.00E+00	1.00E+00	0.00E+00	1.00E+00	-5.13E-15	1.00E+00	0.00E+00	1.00E+00	0.00E+00	1.00E+00
ENSBTAG00000044747	0.00E+00	1.00E+00	0.00E+00	1.00E+00	-5.13E-15	1.00E+00	0.00E+00	1.00E+00	0.00E+00	1.00E+00
ENSBTAG00000009055	-3.98E-01	8.23E-01	-2.00E+00	2.97E-08	-2.64E+00	3.46E-13	-2.60E+00	1.08E-12	-6.04E-01	5.07E-01
ENSBTAG00000012865	7.46E-02	1.00E+00	1.80E-01	4.69E-01	1.97E-01	4.21E-01	2.93E-01	1.61E-01	-1.48E-01	9.30E-01

ENSBTAG00000014608	8.31E-01	5.44E-03	1.35E+00	1.31E-08	1.23E+00	2.39E-07	4.99E-01	7.19E-02	5.88E-01	1.80E-01
ENSBTAG00000043655	0.00E+00	1.00E+00	0.00E+00	1.00E+00	-5.13E-15	1.00E+00	0.00E+00	1.00E+00	0.00E+00	1.00E+00
ENSBTAG00000019300	1.83E-01	9.20E-01	5.98E-01	3.20E-03	5.99E-01	3.08E-03	3.88E-01	8.73E-02	-1.61E-01	9.58E-01
ENSBTAG00000012087	-8.37E-01	2.39E-01	1.11E+00	5.59E-03	1.50E+00	8.50E-05	6.21E-01	1.89E-01	-3.32E-01	9.40E-01
ENSBTAG00000043020	0.00E+00	1.00E+00	1.99E+00	4.43E-01	-5.13E-15	1.00E+00	0.00E+00	1.00E+00	0.00E+00	1.00E+00
ENSBTAG00000042216	0.00E+00	1.00E+00	0.00E+00	1.00E+00	1.78E+00	4.79E-01	0.00E+00	1.00E+00	0.00E+00	1.00E+00
ENSBTAG00000019217	1.01E-01	1.00E+00	-1.06E+00	3.81E-04	-1.43E-01	9.08E-01	4.07E-01	2.66E-01	4.78E-02	1.00E+00
ENSBTAG00000017128	4.03E-01	5.31E-01	1.27E+00	2.99E-07	1.52E+00	5.37E-10	1.24E+00	6.68E-07	-3.02E-02	1.00E+00
ENSBTAG00000017118	1.46E-01	1.00E+00	1.03E+00	3.04E-04	1.31E+00	2.45E-06	1.24E+00	1.14E-05	3.96E-01	6.54E-01
ENSBTAG00000016924	1.57E-01	1.00E+00	-4.76E-01	4.07E-01	-7.39E-01	1.19E-01	1.18E-01	1.00E+00	-6.53E-02	1.00E+00
ENSBTAG00000015396	-3.59E-01	9.59E-01	-2.66E+00	1.00E-07	-8.21E-01	8.70E-02	1.63E+00	3.01E-05	6.30E-01	5.78E-01
ENSBTAG00000020559	-3.03E-01	1.00E+00	-1.85E+00	1.04E-04	-3.39E+00	7.07E-11	-2.29E+00	1.56E-06	-2.88E-02	1.00E+00
ENSBTAG00000043228	0.00E+00	1.00E+00	0.00E+00	1.00E+00	-5.13E-15	1.00E+00	0.00E+00	1.00E+00	0.00E+00	1.00E+00
ENSBTAG00000019675	3.70E-01	7.06E-01	-1.81E-01	7.95E-01	-6.34E-01	4.28E-02	-7.07E-02	1.00E+00	6.78E-01	1.74E-01
ENSBTAG00000019672	5.86E-01	1.70E-01	-5.76E-01	5.20E-02	-7.85E-01	4.74E-03	-7.75E-01	6.22E-03	3.48E-01	7.07E-01
ENSBTAG00000015743	-1.04E+00	7.41E-02	-1.72E+00	6.03E-05	-2.72E+00	1.29E-09	-1.19E+00	4.50E-03	-6.82E-01	4.65E-01
ENSBTAG00000004115	2.05E-01	1.00E+00	-1.44E-01	9.40E-01	2.83E-01	5.34E-01	1.17E-01	1.00E+00	-7.35E-01	1.86E-01
ENSBTAG00000044502	0.00E+00	1.00E+00	0.00E+00	1.00E+00	-5.13E-15	1.00E+00	0.00E+00	1.00E+00	0.00E+00	1.00E+00
ENSBTAG00000012939	-3.53E-01	5.28E-01	-3.82E-01	1.41E-01	-5.41E-01	2.20E-02	-4.97E-01	4.37E-02	-3.80E-01	4.90E-01
ENSBTAG00000012938	2.38E-02	1.00E+00	1.99E+00	6.67E-15	1.12E+00	1.45E-05	1.09E-01	9.90E-01	-3.93E-02	1.00E+00
ENSBTAG00000043133	0.00E+00	1.00E+00	0.00E+00	1.00E+00	-5.13E-15	1.00E+00	0.00E+00	1.00E+00	0.00E+00	1.00E+00
ENSBTAG00000031430	4.13E+00	6.37E-08	1.83E+00	9.40E-03	2.37E-01	1.00E+00	1.00E+00	2.26E-01	-8.67E-01	7.27E-01
ENSBTAG00000045884	2.22E+00	4.22E-01	7.51E-02	1.00E+00	-1.91E+00	4.28E-01	-1.47E-01	1.00E+00	1.69E+00	7.76E-01
ENSBTAG00000001475	-8.71E-02	1.00E+00	-8.56E-01	2.15E-03	-6.26E-01	3.09E-02	-2.63E-01	5.10E-01	2.53E-01	8.67E-01
ENSBTAG00000043307	0.00E+00	1.00E+00	0.00E+00	1.00E+00	-5.13E-15	1.00E+00	0.00E+00	1.00E+00	0.00E+00	1.00E+00
ENSBTAG00000021556	1.74E-01	9.29E-01	-9.80E-02	9.02E-01	1.03E-02	1.00E+00	-1.70E-02	1.00E+00	-6.75E-02	1.00E+00
ENSBTAG00000021554	1.00E-01	1.00E+00	1.16E-01	8.82E-01	3.78E-01	1.35E-01	1.76E-01	6.45E-01	-3.05E-01	6.52E-01
ENSBTAG00000014904	-4.50E-01	3.92E-01	-1.05E+00	3.10E-05	-1.76E+00	1.11E-12	-1.83E+00	2.01E-13	-3.18E-01	7.60E-01
ENSBTAG00000017160	-1.64E-01	1.00E+00	-1.04E+00	1.47E-05	-1.50E+00	1.91E-10	-1.46E+00	8.00E-10	-3.30E-02	1.00E+00
ENSBTAG00000012039	-1.76E-01	1.00E+00	-1.08E+00	3.80E-04	-7.32E-01	1.80E-02	-3.25E-01	4.36E-01	2.72E-01	8.59E-01
ENSBTAG00000008096	2.22E+00	7.69E-03	2.34E+00	5.16E-04	1.21E+00	1.24E-01	1.98E+00	4.74E-03	8.44E-02	1.00E+00
ENSBTAG00000043628	0.00E+00	1.00E+00	0.00E+00	1.00E+00	-5.13E-15	1.00E+00	0.00E+00	1.00E+00	0.00E+00	1.00E+00
ENSBTAG00000001252	5.98E-01	4.17E-01	5.81E-01	1.50E-01	3.59E-01	4.67E-01	6.38E-01	1.11E-01	2.15E-01	1.00E+00
ENSBTAG00000019533	3.61E-01	2.71E-01	-6.07E-01	8.97E-04	-1.88E+00	2.95E-27	-1.95E+00	8.26E-29	8.35E-02	1.00E+00
ENSBTAG00000010256	-1.78E+00	2.36E-02	-8.89E-01	1.81E-01	-1.49E-01	1.00E+00	1.65E-01	1.00E+00	-2.03E-01	1.00E+00

ENSBTAG00000006287	3.29E+00	6.27E-18	1.97E+00	4.57E-08	2.27E+00	2.93E-10	2.11E+00	6.18E-09	1.64E-01	1.00E+00
ENSBTAG00000046160	8.74E-01	5.05E-01	1.28E+00	2.87E-02	1.35E+00	1.93E-02	9.55E-01	1.37E-01	2.95E-02	1.00E+00
ENSBTAG00000002497	6.76E-01	9.79E-02	2.16E-01	6.65E-01	8.98E-01	9.61E-04	6.73E-01	2.17E-02	3.96E-01	6.24E-01
ENSBTAG00000002498	0.00E+00	1.00E+00	0.00E+00	1.00E+00	-5.13E-15	1.00E+00	0.00E+00	1.00E+00	0.00E+00	1.00E+00
ENSBTAG00000031377	1.62E+00	1.61E-01	7.31E-01	5.59E-01	6.94E-01	5.72E-01	1.58E+00	4.23E-02	1.76E+00	1.44E-01
ENSBTAG00000018787	8.04E-01	1.00E+00	1.40E+00	5.16E-01	1.15E+00	6.42E-01	-1.47E-01	1.00E+00	1.99E+00	6.01E-01
ENSBTAG00000014053	5.16E-01	9.17E-01	4.63E-01	6.26E-01	-2.52E-01	9.72E-01	-1.58E-01	1.00E+00	1.07E+00	3.44E-01
ENSBTAG00000005808	-6.29E-02	1.00E+00	-2.55E-01	2.55E-01	-5.20E-01	4.92E-03	-4.98E-01	8.74E-03	-3.59E-01	3.46E-01
ENSBTAG00000018674	2.90E-01	5.76E-01	1.95E+00	1.44E-26	2.02E+00	1.11E-28	1.39E+00	3.24E-14	-8.76E-02	1.00E+00
ENSBTAG00000039326	3.77E-01	9.74E-01	2.37E+00	1.68E-07	1.44E+00	1.87E-03	-5.60E-01	3.65E-01	9.50E-02	1.00E+00
ENSBTAG00000039513	5.57E-02	1.00E+00	1.57E+00	7.16E-04	2.11E-01	9.84E-01	-2.70E-01	9.17E-01	-1.71E-01	1.00E+00
ENSBTAG00000001250	-5.40E-01	1.00E+00	1.53E+00	6.93E-02	2.11E-01	1.00E+00	6.97E-03	1.00E+00	1.06E+00	7.04E-01
ENSBTAG00000005248	-5.61E-01	1.00E+00	-1.76E+00	1.29E-01	-1.08E+00	3.59E-01	-9.43E-01	4.47E-01	-1.73E-02	1.00E+00
ENSBTAG00000009157	-1.21E-01	1.00E+00	1.02E+00	1.52E-06	8.17E-01	1.42E-04	4.13E-01	1.01E-01	1.96E-02	1.00E+00
ENSBTAG00000002534	5.44E-02	1.00E+00	1.57E+00	7.88E-13	1.61E+00	1.56E-13	1.26E+00	1.41E-08	-4.36E-01	4.29E-01
ENSBTAG00000039732	-1.15E-01	1.00E+00	-6.38E-01	3.23E-02	-7.48E-02	1.00E+00	3.37E-01	3.63E-01	1.70E-01	1.00E+00
ENSBTAG00000046839	-2.59E-02	1.00E+00	1.25E+00	2.99E-05	1.20E+00	5.49E-05	1.10E+00	3.18E-04	6.18E-01	3.16E-01
ENSBTAG00000019237	0.00E+00	1.00E+00	0.00E+00	1.00E+00	-5.13E-15	1.00E+00	0.00E+00	1.00E+00	0.00E+00	1.00E+00
ENSBTAG00000043918	0.00E+00	1.00E+00	0.00E+00	1.00E+00	-5.13E-15	1.00E+00	0.00E+00	1.00E+00	0.00E+00	1.00E+00
ENSBTAG00000019234	2.98E-01	9.81E-01	1.16E-01	1.00E+00	6.88E-01	8.27E-02	1.04E+00	4.39E-03	-2.20E-01	1.00E+00
ENSBTAG00000008517	-2.97E-01	9.19E-01	-8.56E-01	1.29E-02	-5.72E-01	1.24E-01	-5.48E-01	1.55E-01	-4.65E-01	6.23E-01
ENSBTAG00000001123	-1.80E-01	1.00E+00	4.97E-01	5.08E-02	3.29E-01	2.55E-01	-4.10E-01	1.40E-01	-2.44E-01	8.04E-01
ENSBTAG00000015106	-3.68E-03	1.00E+00	-9.13E-01	4.52E-03	-1.23E+00	7.23E-05	-2.00E-01	7.87E-01	5.77E-01	4.24E-01
ENSBTAG00000043917	0.00E+00	1.00E+00	0.00E+00	1.00E+00	-5.13E-15	1.00E+00	0.00E+00	1.00E+00	0.00E+00	1.00E+00
ENSBTAG00000013821	4.87E-01	2.69E-01	2.39E+00	1.38E-25	2.25E+00	4.37E-23	1.53E+00	3.20E-11	9.07E-02	1.00E+00
ENSBTAG00000014587	-1.78E-02	1.00E+00	-3.07E-01	8.97E-01	-7.22E-01	3.69E-01	-1.17E+00	9.21E-02	-1.06E-01	1.00E+00
ENSBTAG00000022731	3.79E-01	5.76E-01	8.98E-01	3.30E-04	1.16E+00	2.06E-06	1.25E+00	3.70E-07	2.80E-01	8.00E-01
ENSBTAG00000005980	1.41E-01	1.00E+00	1.44E-01	8.42E-01	4.80E-02	1.00E+00	1.66E-01	7.64E-01	1.06E-01	1.00E+00
ENSBTAG00000031444	-1.72E-01	1.00E+00	-1.36E+00	1.10E-04	-2.58E-01	6.37E-01	1.52E-02	1.00E+00	2.24E-01	1.00E+00
ENSBTAG00000007268	1.12E+00	1.10E-01	1.14E-01	1.00E+00	3.35E-01	7.02E-01	1.09E+00	2.91E-02	1.36E+00	5.09E-02
ENSBTAG00000009873	-7.85E-01	4.47E-01	-1.38E+00	6.11E-03	-6.13E-01	2.88E-01	3.03E-01	7.44E-01	-6.45E-01	6.49E-01
ENSBTAG00000010640	0.00E+00	1.00E+00	0.00E+00	1.00E+00	1.78E+00	4.79E-01	1.76E+00	4.97E-01	1.93E+00	8.00E-01
ENSBTAG00000018072	-2.47E-01	1.00E+00	-4.72E-01	6.92E-01	-1.28E+00	7.35E-02	-1.87E+00	6.40E-03	-4.54E-01	1.00E+00
ENSBTAG00000031432	9.21E-02	1.00E+00	6.16E-01	2.84E-02	4.59E-01	1.30E-01	1.13E-01	9.77E-01	-2.88E-01	8.00E-01
ENSBTAG00000018309	0.00E+00	1.00E+00	0.00E+00	1.00E+00	-5.13E-15	1.00E+00	0.00E+00	1.00E+00	0.00E+00	1.00E+00

ENSBTAG00000046057	1.92E+00	8.23E-01	1.99E+00	4.43E-01	1.78E+00	4.79E-01	0.00E+00	1.00E+00	1.91E+00	8.00E-01
ENSBTAG00000003332	-7.51E-02	1.00E+00	4.02E-02	1.00E+00	1.85E+00	3.21E-12	2.35E+00	7.21E-19	3.21E-01	8.00E-01
ENSBTAG00000016397	2.13E-01	9.34E-01	1.49E+00	2.26E-11	2.32E+00	3.92E-26	1.50E+00	1.42E-11	6.24E-02	1.00E+00
ENSBTAG00000015178	-2.78E-01	8.22E-01	-9.89E-01	3.27E-05	-1.69E+00	3.88E-13	-1.96E+00	6.03E-17	-1.41E-01	1.00E+00
ENSBTAG00000015176	-2.33E-01	1.00E+00	-1.21E+00	5.98E-05	-2.58E+00	1.53E-17	-3.05E+00	5.32E-23	-7.53E-01	1.49E-01
ENSBTAG00000002089	5.68E-02	1.00E+00	-5.32E-01	3.97E-01	-2.58E+00	9.28E-07	-2.34E+00	6.48E-06	-4.71E-01	8.04E-01
ENSBTAG00000002086	1.38E-01	1.00E+00	8.26E-01	1.60E-04	6.85E-01	2.13E-03	9.27E-02	9.84E-01	5.80E-02	1.00E+00
ENSBTAG00000026919	6.23E-01	5.76E-01	7.25E-02	1.00E+00	1.78E+00	1.08E-05	1.56E+00	1.61E-04	-3.13E-01	9.97E-01
ENSBTAG00000010943	-1.50E-01	1.00E+00	-5.35E-01	4.55E-02	-5.50E-01	3.80E-02	-2.03E-01	6.29E-01	-7.80E-02	1.00E+00
ENSBTAG00000031249	-1.98E-01	1.00E+00	-3.55E-01	2.58E-01	-4.04E-01	1.78E-01	-5.27E-01	6.32E-02	-2.89E-01	8.00E-01
ENSBTAG00000004093	-2.90E-01	1.00E+00	-1.27E+00	5.89E-03	-6.97E-01	1.76E-01	-2.91E-01	7.54E-01	-4.91E-01	8.00E-01
ENSBTAG00000046886	3.99E-01	8.28E-01	1.22E-01	1.00E+00	2.41E-01	7.91E-01	1.86E-01	9.28E-01	-6.74E-01	4.90E-01
ENSBTAG00000006379	-4.77E-01	2.57E-01	-9.74E-01	3.10E-05	-1.62E+00	1.40E-12	-1.95E+00	2.68E-17	-2.48E-01	8.00E-01
ENSBTAG00000045888	0.00E+00	1.00E+00	0.00E+00	1.00E+00	-5.13E-15	1.00E+00	0.00E+00	1.00E+00	0.00E+00	1.00E+00
ENSBTAG00000006378	4.41E-01	6.83E-01	2.78E+00	2.38E-17	1.93E+00	1.98E-09	8.50E-01	1.60E-02	-3.75E-02	1.00E+00
ENSBTAG00000000786	-3.76E-01	7.09E-01	-6.79E-01	2.72E-02	1.70E-01	8.22E-01	-4.82E-01	1.62E-01	-7.51E-02	1.00E+00
ENSBTAG00000017483	-1.05E-01	1.00E+00	-5.34E-01	5.17E-02	-6.67E-01	1.03E-02	-4.03E-01	1.79E-01	-7.48E-02	1.00E+00
ENSBTAG00000043014	0.00E+00	1.00E+00	0.00E+00	1.00E+00	-5.13E-15	1.00E+00	0.00E+00	1.00E+00	0.00E+00	1.00E+00
ENSBTAG00000017949	-5.42E-01	2.34E-01	-4.26E-01	1.70E-01	-8.26E-01	1.98E-03	-8.02E-01	3.25E-03	-7.91E-01	4.91E-02
ENSBTAG00000042636	0.00E+00	1.00E+00	0.00E+00	1.00E+00	-5.13E-15	1.00E+00	0.00E+00	1.00E+00	0.00E+00	1.00E+00
ENSBTAG00000025280	-1.29E-01	1.00E+00	-5.48E-01	6.80E-03	-7.66E-01	7.63E-05	-8.10E-01	3.40E-05	-2.26E-01	8.00E-01
ENSBTAG00000031267	1.88E+00	1.70E-07	7.66E-01	3.98E-02	7.55E-01	4.26E-02	6.29E-01	1.16E-01	-3.18E-01	8.97E-01
ENSBTAG00000011975	5.68E-02	1.00E+00	-7.82E-01	1.12E-01	-7.06E-01	1.63E-01	-5.05E-01	3.98E-01	-7.88E-02	1.00E+00
ENSBTAG00000045364	0.00E+00	1.00E+00	0.00E+00	1.00E+00	-5.13E-15	1.00E+00	0.00E+00	1.00E+00	0.00E+00	1.00E+00
ENSBTAG00000006166	-2.45E-01	7.57E-01	-5.08E-01	1.36E-02	2.29E-02	1.00E+00	-3.63E-01	1.10E-01	-1.28E-01	1.00E+00
ENSBTAG00000012079	-6.23E-01	4.19E-01	-3.39E+00	6.41E-19	-5.25E+00	3.44E-35	-4.27E+00	5.52E-27	2.53E-01	1.00E+00
ENSBTAG00000037624	0.00E+00	1.00E+00	0.00E+00	1.00E+00	-5.13E-15	1.00E+00	1.76E+00	4.97E-01	0.00E+00	1.00E+00
ENSBTAG00000012058	2.95E-02	1.00E+00	6.65E-02	1.00E+00	-2.36E-01	5.35E-01	-2.66E-01	4.76E-01	2.20E-01	9.13E-01
ENSBTAG00000026896	-9.93E-01	6.46E-01	-2.37E+00	1.01E-02	-1.50E+00	7.09E-02	-1.33E+00	1.17E-01	-1.16E+00	5.63E-01
ENSBTAG00000043725	0.00E+00	1.00E+00	0.00E+00	1.00E+00	-5.13E-15	1.00E+00	0.00E+00	1.00E+00	0.00E+00	1.00E+00
ENSBTAG00000020215	2.24E-01	8.23E-01	9.60E-01	1.18E-06	4.80E-01	2.63E-02	8.50E-02	9.87E-01	-9.13E-02	1.00E+00
ENSBTAG00000037257	0.00E+00	1.00E+00	0.00E+00	1.00E+00	-5.13E-15	1.00E+00	0.00E+00	1.00E+00	0.00E+00	1.00E+00
ENSBTAG00000002929	8.83E-02	1.00E+00	3.23E-01	8.36E-01	-3.64E-01	7.72E-01	-9.14E-02	1.00E+00	1.13E-01	1.00E+00
ENSBTAG00000027375	-5.80E-01	8.23E-01	-2.53E-01	9.29E-01	-4.26E-01	6.26E-01	-8.00E-01	2.24E-01	-5.86E-01	8.00E-01
ENSBTAG00000047812	0.00E+00	1.00E+00	0.00E+00	1.00E+00	-5.13E-15	1.00E+00	0.00E+00	1.00E+00	0.00E+00	1.00E+00

ENSBTAG00000038677	0.00E+00	1.00E+00	0.00E+00	1.00E+00	-5.13E-15	1.00E+00	0.00E+00	1.00E+00	0.00E+00	1.00E+00
ENSBTAG00000037293	0.00E+00	1.00E+00	0.00E+00	1.00E+00	-5.13E-15	1.00E+00	0.00E+00	1.00E+00	0.00E+00	1.00E+00
ENSBTAG00000047516	0.00E+00	1.00E+00	0.00E+00	1.00E+00	-5.13E-15	1.00E+00	1.76E+00	4.97E-01	0.00E+00	1.00E+00
ENSBTAG00000047485	0.00E+00	1.00E+00	0.00E+00	1.00E+00	-5.13E-15	1.00E+00	0.00E+00	1.00E+00	0.00E+00	1.00E+00
ENSBTAG00000046606	0.00E+00	1.00E+00	0.00E+00	1.00E+00	-5.13E-15	1.00E+00	0.00E+00	1.00E+00	0.00E+00	1.00E+00
ENSBTAG00000046691	-1.95E+00	5.81E-01	-1.89E+00	2.59E-01	-3.87E+00	2.31E-02	-3.87E+00	2.52E-02	-1.96E+00	6.03E-01
ENSBTAG00000013809	0.00E+00	1.00E+00	0.00E+00	1.00E+00	-5.13E-15	1.00E+00	0.00E+00	1.00E+00	0.00E+00	1.00E+00
ENSBTAG00000046382	0.00E+00	1.00E+00	0.00E+00	1.00E+00	-5.13E-15	1.00E+00	0.00E+00	1.00E+00	1.91E+00	8.00E-01
ENSBTAG00000045511	0.00E+00	1.00E+00	0.00E+00	1.00E+00	-5.13E-15	1.00E+00	0.00E+00	1.00E+00	0.00E+00	1.00E+00
ENSBTAG00000046505	0.00E+00	1.00E+00	0.00E+00	1.00E+00	-5.13E-15	1.00E+00	0.00E+00	1.00E+00	0.00E+00	1.00E+00
ENSBTAG00000001317	0.00E+00	1.00E+00	1.98E+00	4.43E-01	-5.13E-15	1.00E+00	0.00E+00	1.00E+00	0.00E+00	1.00E+00
ENSBTAG00000039099	-2.71E+00	5.76E-01	-2.71E+00	2.42E-01	-2.71E+00	1.99E-01	-2.71E+00	2.05E-01	-2.71E+00	6.01E-01
ENSBTAG00000046170	0.00E+00	1.00E+00	0.00E+00	1.00E+00	-5.13E-15	1.00E+00	0.00E+00	1.00E+00	0.00E+00	1.00E+00
ENSBTAG00000047013	0.00E+00	1.00E+00	0.00E+00	1.00E+00	-5.13E-15	1.00E+00	0.00E+00	1.00E+00	0.00E+00	1.00E+00
ENSBTAG00000039564	0.00E+00	1.00E+00	0.00E+00	1.00E+00	-5.13E-15	1.00E+00	0.00E+00	1.00E+00	0.00E+00	1.00E+00
ENSBTAG00000005917	5.60E-02	1.00E+00	-9.96E-01	2.56E-03	-1.07E+00	8.78E-04	-1.18E+00	2.96E-04	4.66E-01	6.01E-01
ENSBTAG00000005916	1.24E-02	1.00E+00	9.64E-01	5.35E-04	1.42E+00	1.05E-07	1.19E+00	1.37E-05	5.18E-01	4.23E-01
ENSBTAG00000036297	-5.72E-01	1.82E-01	-1.19E+00	5.32E-06	3.81E-01	2.16E-01	1.03E+00	3.80E-05	6.68E-02	1.00E+00
ENSBTAG00000036298	-1.10E+00	2.03E-06	-3.09E+00	1.31E-44	-4.47E+00	1.25E-79	-2.48E+00	5.72E-31	6.06E-02	1.00E+00
ENSBTAG00000013587	-1.20E-01	1.00E+00	-2.76E-01	3.85E-01	1.01E-01	9.65E-01	1.49E-01	7.85E-01	-3.32E-02	1.00E+00
ENSBTAG00000022895	-6.85E-02	1.00E+00	-1.94E-02	1.00E+00	-4.07E-01	9.58E-02	-2.35E-01	4.50E-01	-3.81E-02	1.00E+00
ENSBTAG00000013665	1.77E-01	1.00E+00	5.54E-01	5.78E-01	-1.77E+00	4.48E-02	-4.02E-02	1.00E+00	5.65E-01	8.97E-01
ENSBTAG00000000656	-3.68E-02	1.00E+00	-4.80E-01	1.04E-01	-1.87E-01	6.93E-01	-1.02E-01	9.96E-01	2.46E-01	8.44E-01
ENSBTAG00000001224	7.39E-03	1.00E+00	-1.01E+00	4.91E-07	-5.20E-01	1.60E-02	-3.83E-01	1.06E-01	7.59E-02	1.00E+00
ENSBTAG00000042653	0.00E+00	1.00E+00	0.00E+00	1.00E+00	-5.13E-15	1.00E+00	0.00E+00	1.00E+00	0.00E+00	1.00E+00
ENSBTAG00000021003	-1.15E+00	2.17E-01	3.62E-02	1.00E+00	-3.46E-01	7.50E-01	1.49E-01	1.00E+00	-2.59E-01	1.00E+00
ENSBTAG00000022890	-2.39E-01	1.00E+00	-1.83E+00	8.96E-09	3.12E-01	4.97E-01	9.52E-01	4.34E-03	1.21E-01	1.00E+00
ENSBTAG00000017517	3.19E-01	6.87E-01	7.46E-01	2.06E-03	1.03E+00	9.20E-06	5.55E-01	3.31E-02	2.74E-01	8.00E-01
ENSBTAG00000042712	1.92E+00	8.23E-01	0.00E+00	1.00E+00	-5.13E-15	1.00E+00	0.00E+00	1.00E+00	0.00E+00	1.00E+00
ENSBTAG00000004650	1.26E-01	1.00E+00	-5.57E-01	3.27E-01	-1.39E-01	1.00E+00	-1.73E-01	9.96E-01	7.73E-01	4.34E-01
ENSBTAG00000000422	-3.09E-02	1.00E+00	-7.10E-01	2.94E-02	1.63E-01	8.64E-01	-1.43E-01	9.36E-01	3.92E-01	7.14E-01
ENSBTAG00000001724	2.13E-01	1.00E+00	-8.14E-01	2.50E-02	-1.02E+00	3.41E-03	-7.99E-01	3.20E-02	3.77E-02	1.00E+00
ENSBTAG00000022887	1.66E+00	1.05E-07	7.87E-01	1.21E-02	7.00E-01	2.88E-02	9.20E-01	3.04E-03	5.56E-01	4.27E-01
ENSBTAG00000021428	2.46E-01	1.00E+00	-1.88E+00	1.88E-01	-9.99E-01	5.27E-01	-1.47E+00	3.10E-01	5.26E-01	1.00E+00
ENSBTAG00000009841	-4.36E-02	1.00E+00	-3.74E-01	4.69E-01	-4.54E-01	3.40E-01	-3.68E-01	4.97E-01	1.91E-01	1.00E+00

ENSBTAG00000000106	3.40E-01	1.00E+00	-2.97E-01	1.00E+00	-8.94E-01	5.52E-01	-2.19E+00	7.82E-02	-2.28E-01	1.00E+00
ENSBTAG00000022466	1.62E-03	1.00E+00	8.85E-02	1.00E+00	-1.81E+00	2.93E-01	-1.83E+00	2.96E-01	9.44E-01	8.33E-01
ENSBTAG00000012012	-3.48E-01	5.26E-01	-1.06E+00	8.23E-07	-2.43E+00	8.82E-30	-2.81E+00	4.13E-38	1.70E-01	9.83E-01
ENSBTAG00000009687	-3.72E-01	3.51E-01	-6.80E-01	7.84E-04	-4.63E-01	3.05E-02	-3.31E-01	1.68E-01	-2.25E-01	8.00E-01
ENSBTAG00000009686	-2.86E-01	9.94E-01	-1.89E-01	8.78E-01	-5.30E-01	2.17E-01	-1.09E+00	4.02E-03	3.10E-01	8.98E-01
ENSBTAG00000012889	2.21E-01	1.00E+00	3.50E-01	9.45E-01	2.85E-01	1.00E+00	7.65E-01	4.45E-01	9.40E-03	1.00E+00
ENSBTAG00000005985	-1.96E+00	5.76E-01	-5.73E-01	8.94E-01	-1.33E+00	4.16E-01	-2.12E+00	1.80E-01	-3.88E+00	2.08E-01
ENSBTAG00000017412	4.91E-01	6.14E-01	9.97E-01	3.98E-03	1.27E+00	1.06E-04	1.41E+00	1.72E-05	8.05E-01	1.88E-01
ENSBTAG00000013221	2.50E-01	9.58E-01	8.09E-02	1.00E+00	3.58E-01	3.49E-01	-7.69E-03	1.00E+00	2.42E-01	9.45E-01
ENSBTAG00000009266	1.72E-02	1.00E+00	1.34E+00	7.62E-02	-7.23E-01	4.79E-01	-2.50E-01	1.00E+00	-3.46E-01	1.00E+00
ENSBTAG00000046957	-3.92E-02	1.00E+00	-5.63E-01	8.39E-01	-1.10E+00	4.18E-01	-8.24E-01	6.08E-01	5.41E-01	1.00E+00
ENSBTAG00000036666	1.92E+00	8.23E-01	0.00E+00	1.00E+00	-5.13E-15	1.00E+00	0.00E+00	1.00E+00	0.00E+00	1.00E+00
ENSBTAG00000006726	1.25E-01	1.00E+00	-1.43E+00	2.04E-03	-1.20E+00	6.93E-03	-2.15E-01	8.74E-01	-1.02E-01	1.00E+00
ENSBTAG00000007567	-1.01E-01	1.00E+00	1.18E-01	9.57E-01	1.11E+00	1.46E-05	9.79E-01	2.14E-04	2.79E-01	8.00E-01
ENSBTAG00000034676	8.19E-01	9.20E-01	3.06E+00	3.51E-06	2.42E+00	6.80E-04	2.56E+00	2.68E-04	9.54E-01	8.00E-01
ENSBTAG00000037907	-4.35E-01	8.23E-01	-5.74E-02	1.00E+00	6.92E-01	9.95E-02	7.61E-01	6.86E-02	3.63E-03	1.00E+00
ENSBTAG00000046641	0.00E+00	1.00E+00	0.00E+00	1.00E+00	-5.13E-15	1.00E+00	1.76E+00	4.97E-01	0.00E+00	1.00E+00
ENSBTAG00000023270	9.90E-02	1.00E+00	-2.39E-01	1.00E+00	-1.13E+00	2.22E-01	-1.61E+00	8.06E-02	-3.29E-01	1.00E+00
ENSBTAG00000043797	0.00E+00	1.00E+00	0.00E+00	1.00E+00	-5.13E-15	1.00E+00	0.00E+00	1.00E+00	0.00E+00	1.00E+00
ENSBTAG00000014488	1.88E-02	1.00E+00	8.71E-01	4.12E-01	-2.90E-02	1.00E+00	8.24E-01	4.39E-01	-2.62E+00	2.89E-01
ENSBTAG00000028094	0.00E+00	1.00E+00	0.00E+00	1.00E+00	-5.13E-15	1.00E+00	0.00E+00	1.00E+00	0.00E+00	1.00E+00
ENSBTAG00000029114	0.00E+00	1.00E+00	0.00E+00	1.00E+00	-5.13E-15	1.00E+00	0.00E+00	1.00E+00	1.93E+00	8.00E-01
ENSBTAG00000001207	2.76E+00	8.37E-10	3.58E+00	8.64E-17	2.36E+00	2.47E-08	2.55E+00	2.37E-09	-6.77E-01	6.12E-01
ENSBTAG00000023026	2.05E+00	3.29E-01	5.75E+00	5.75E-08	5.39E+00	2.83E-07	6.59E+00	1.17E-09	-1.97E+00	7.15E-01
ENSBTAG00000034656	-6.05E-01	8.23E-01	-4.65E-01	5.86E-01	-2.07E-01	9.84E-01	-1.40E-01	1.00E+00	-8.41E-01	6.01E-01
ENSBTAG00000039565	0.00E+00	1.00E+00	0.00E+00	1.00E+00	-5.13E-15	1.00E+00	0.00E+00	1.00E+00	0.00E+00	1.00E+00
ENSBTAG00000001801	1.51E-01	1.00E+00	4.94E-01	4.20E-01	1.50E-01	1.00E+00	3.97E-01	5.62E-01	-1.59E-01	1.00E+00
ENSBTAG00000045568	-6.82E-02	1.00E+00	1.32E-01	8.90E-01	9.69E-02	1.00E+00	1.48E-01	8.36E-01	-3.56E-01	6.43E-01
ENSBTAG00000026290	-1.61E-01	1.00E+00	-3.79E-01	1.48E-01	9.51E-02	9.75E-01	-5.18E-02	1.00E+00	-1.47E-02	1.00E+00
ENSBTAG00000045111	5.16E-01	1.00E+00	1.73E+00	2.00E-01	1.80E+00	1.53E-01	9.73E-01	6.35E-01	-7.87E-01	1.00E+00
ENSBTAG00000045320	0.00E+00	1.00E+00	0.00E+00	1.00E+00	-5.13E-15	1.00E+00	0.00E+00	1.00E+00	0.00E+00	1.00E+00
ENSBTAG00000011094	1.46E-01	1.00E+00	1.64E+00	1.81E-13	1.61E+00	3.71E-13	9.28E-01	6.99E-05	-9.34E-02	1.00E+00
ENSBTAG00000004940	-8.78E-01	4.90E-02	-1.90E+00	6.36E-09	-2.44E+00	1.25E-13	-1.80E+00	2.50E-08	2.40E-01	9.85E-01
ENSBTAG00000044087	9.88E-01	5.89E-01	-5.98E-01	7.28E-01	-6.43E-01	6.50E-01	-1.85E-01	1.00E+00	6.86E-01	8.48E-01
ENSBTAG00000021583	-4.36E-02	1.00E+00	9.01E-02	1.00E+00	9.11E-01	3.47E-05	9.15E-01	4.01E-05	-2.40E-01	8.03E-01

ENSBTAG00000021581	-2.63E-01	1.00E+00	-2.27E-01	9.24E-01	-6.57E-01	2.63E-01	4.10E-01	5.40E-01	1.65E-01	1.00E+00
ENSBTAG00000044770	0.00E+00	1.00E+00	0.00E+00	1.00E+00	-5.13E-15	1.00E+00	0.00E+00	1.00E+00	0.00E+00	1.00E+00
ENSBTAG00000036732	0.00E+00	1.00E+00	0.00E+00	1.00E+00	-5.13E-15	1.00E+00	0.00E+00	1.00E+00	0.00E+00	1.00E+00
ENSBTAG00000042194	0.00E+00	1.00E+00	0.00E+00	1.00E+00	-5.13E-15	1.00E+00	0.00E+00	1.00E+00	0.00E+00	1.00E+00
ENSBTAG00000012252	2.65E-01	8.23E-01	1.18E+00	5.79E-07	5.42E-01	3.70E-02	1.52E-01	7.99E-01	-4.21E-02	1.00E+00
ENSBTAG00000018954	4.30E-01	1.67E-01	1.90E+00	4.03E-25	1.02E+00	3.18E-08	4.66E-01	2.51E-02	7.86E-02	1.00E+00
ENSBTAG00000000998	1.52E-01	1.00E+00	1.56E+00	9.34E-10	1.64E+00	8.93E-11	1.33E+00	2.74E-07	1.20E-01	1.00E+00
ENSBTAG00000005497	-3.59E-01	5.61E-01	-5.46E-01	2.89E-02	3.46E-01	2.14E-01	-2.45E-01	4.67E-01	-4.19E-01	4.34E-01
ENSBTAG00000011622	-1.26E-01	1.00E+00	3.75E-01	2.36E-01	7.56E-01	3.56E-03	4.79E-01	1.04E-01	7.73E-03	1.00E+00
ENSBTAG00000029796	0.00E+00	1.00E+00	1.99E+00	4.43E-01	-5.13E-15	1.00E+00	0.00E+00	1.00E+00	0.00E+00	1.00E+00
ENSBTAG00000011206	-1.72E-01	1.00E+00	8.49E-01	1.29E-03	8.15E-01	2.04E-03	5.66E-01	5.10E-02	-3.02E-01	8.00E-01
ENSBTAG00000012426	4.86E-02	1.00E+00	-1.35E-01	7.81E-01	1.47E-01	7.16E-01	-9.88E-03	1.00E+00	2.96E-01	6.25E-01
ENSBTAG00000010255	-4.09E-02	1.00E+00	5.22E-01	1.57E-02	5.56E-01	9.08E-03	2.67E-01	3.26E-01	-8.79E-02	1.00E+00
ENSBTAG00000010252	-2.80E-01	8.18E-01	-3.83E-01	1.88E-01	4.39E-01	1.05E-01	-1.84E-01	6.76E-01	1.42E-01	1.00E+00
ENSBTAG00000015315	0.00E+00	1.00E+00	0.00E+00	1.00E+00	2.56E+00	2.54E-01	0.00E+00	1.00E+00	0.00E+00	1.00E+00
ENSBTAG00000007520	4.07E-02	1.00E+00	1.28E+00	7.74E-05	9.71E-01	3.70E-03	6.21E-01	9.98E-02	-1.70E-01	1.00E+00
ENSBTAG00000043893	0.00E+00	1.00E+00	0.00E+00	1.00E+00	-5.13E-15	1.00E+00	0.00E+00	1.00E+00	0.00E+00	1.00E+00
ENSBTAG00000012307	7.99E-01	8.23E-01	2.88E+00	1.32E-04	1.67E+00	3.41E-02	6.80E-01	5.44E-01	1.75E-01	1.00E+00
ENSBTAG00000023206	0.00E+00	1.00E+00	0.00E+00	1.00E+00	-5.13E-15	1.00E+00	0.00E+00	1.00E+00	0.00E+00	1.00E+00
ENSBTAG00000042637	0.00E+00	1.00E+00	0.00E+00	1.00E+00	-5.13E-15	1.00E+00	0.00E+00	1.00E+00	0.00E+00	1.00E+00
ENSBTAG00000045217	0.00E+00	1.00E+00	0.00E+00	1.00E+00	-5.13E-15	1.00E+00	0.00E+00	1.00E+00	0.00E+00	1.00E+00
ENSBTAG00000010299	-4.32E-01	1.00E+00	1.54E-01	1.00E+00	-1.38E+00	4.81E-02	4.98E-01	5.62E-01	-1.67E+00	1.58E-01
ENSBTAG00000043612	0.00E+00	1.00E+00	0.00E+00	1.00E+00	-5.13E-15	1.00E+00	0.00E+00	1.00E+00	0.00E+00	1.00E+00
ENSBTAG00000047003	-9.71E-01	5.24E-01	-1.65E-01	1.00E+00	-6.69E-01	3.92E-01	-4.60E-02	1.00E+00	-1.54E+00	2.01E-01
ENSBTAG00000000138	3.19E-01	1.00E+00	-1.14E+00	4.33E-01	-1.09E+00	4.28E-01	-2.27E-01	1.00E+00	-6.36E-01	1.00E+00
ENSBTAG00000048303	-1.91E+00	8.23E-01	-1.91E+00	4.69E-01	-1.91E+00	4.28E-01	-1.91E+00	4.39E-01	7.93E-01	1.00E+00
ENSBTAG00000045391	0.00E+00	1.00E+00	0.00E+00	1.00E+00	-5.13E-15	1.00E+00	0.00E+00	1.00E+00	0.00E+00	1.00E+00
ENSBTAG00000020003	5.11E-02	1.00E+00	-4.94E-01	6.56E-01	-5.65E-01	5.39E-01	-6.18E-01	4.97E-01	1.65E-01	1.00E+00
ENSBTAG00000020286	3.54E-02	1.00E+00	-2.07E+00	1.52E-15	-2.53E+00	3.63E-22	-1.30E+00	3.45E-07	4.87E-01	3.84E-01
ENSBTAG00000020391	2.54E-01	1.00E+00	-3.55E-01	7.37E-01	-8.60E-01	1.54E-01	-1.81E+00	7.27E-04	7.56E-01	6.01E-01
ENSBTAG00000020160	5.94E-01	1.46E-01	1.32E+00	8.25E-08	9.15E-01	3.18E-04	2.65E-01	4.94E-01	-1.96E-01	1.00E+00
ENSBTAG00000045379	0.00E+00	1.00E+00	0.00E+00	1.00E+00	-5.13E-15	1.00E+00	0.00E+00	1.00E+00	0.00E+00	1.00E+00
ENSBTAG00000003113	1.57E+00	1.24E-05	3.20E+00	4.65E-22	3.04E+00	2.32E-20	1.48E+00	6.67E-06	-6.30E-01	4.01E-01
ENSBTAG00000039296	1.01E+00	5.48E-02	3.28E+00	8.58E-19	3.49E+00	5.66E-21	2.16E+00	3.86E-09	-1.25E-01	1.00E+00
ENSBTAG00000007696	9.31E-01	3.42E-03	3.32E+00	7.82E-36	3.26E+00	5.40E-35	1.71E+00	3.73E-11	-1.17E-01	1.00E+00

ENSBTAG00000044063	-1.01E+00	1.60E-02	-2.65E+00	1.15E-14	-1.04E+00	1.61E-03	-9.31E-01	6.09E-03	-2.99E-01	8.79E-01
ENSBTAG00000010991	-4.16E-01	4.55E-01	-9.68E-01	8.53E-05	-1.88E+00	5.05E-15	-2.42E+00	2.72E-23	-6.22E-01	1.42E-01
ENSBTAG00000021923	-2.23E-01	9.49E-01	-8.54E-01	1.03E-03	-1.04E+00	4.12E-05	-2.01E-01	6.65E-01	-2.82E-01	8.00E-01
ENSBTAG00000039483	6.37E-02	1.00E+00	-4.47E-02	1.00E+00	-1.96E+00	1.03E-04	-2.34E+00	7.13E-06	-3.53E-02	1.00E+00
ENSBTAG00000040126	2.64E-01	1.00E+00	-4.71E-01	6.42E-01	-2.16E+00	1.88E-03	-8.64E-01	2.44E-01	-4.48E-01	9.85E-01
ENSBTAG00000013831	-1.91E-01	1.00E+00	-9.95E-01	9.99E-02	-1.22E+00	3.21E-02	-1.83E+00	1.42E-03	-5.76E-01	8.00E-01
ENSBTAG00000019876	-6.76E-01	6.30E-01	-3.21E+00	1.75E-07	-3.28E+00	2.96E-08	-2.11E+00	8.68E-05	4.36E-01	8.56E-01
ENSBTAG00000040584	-2.28E-01	1.00E+00	-3.71E+00	1.07E-24	-2.95E+00	9.53E-18	-4.14E-01	3.66E-01	5.18E-01	5.89E-01
ENSBTAG00000015238	-8.69E-01	1.00E+00	-1.60E+00	4.08E-01	-1.81E+00	2.94E-01	-1.06E+00	5.95E-01	-3.52E-01	1.00E+00
ENSBTAG00000044228	0.00E+00	1.00E+00	0.00E+00	1.00E+00	-5.13E-15	1.00E+00	0.00E+00	1.00E+00	0.00E+00	1.00E+00
ENSBTAG00000028575	0.00E+00	1.00E+00	0.00E+00	1.00E+00	1.78E+00	4.79E-01	3.41E+00	8.24E-02	1.91E+00	8.00E-01
ENSBTAG00000021190	-1.53E-01	1.00E+00	-1.08E+00	3.83E-08	-1.33E+00	5.96E-12	-1.13E+00	7.61E-09	3.25E-01	5.43E-01
ENSBTAG00000044212	2.60E-01	9.87E-01	2.22E-01	7.39E-01	5.25E-01	1.52E-01	1.18E+00	1.32E-04	1.76E-01	1.00E+00
ENSBTAG00000046976	0.00E+00	1.00E+00	0.00E+00	1.00E+00	-5.13E-15	1.00E+00	0.00E+00	1.00E+00	0.00E+00	1.00E+00
ENSBTAG00000014082	-3.54E-01	1.00E+00	-7.30E-01	4.43E-01	-1.34E+00	7.67E-02	-1.59E+00	3.40E-02	-1.24E+00	4.34E-01
ENSBTAG00000034032	0.00E+00	1.00E+00	0.00E+00	1.00E+00	-5.13E-15	1.00E+00	0.00E+00	1.00E+00	0.00E+00	1.00E+00
ENSBTAG00000019037	-1.97E+00	5.76E-01	-1.10E+00	5.46E-01	-1.33E+00	4.14E-01	-8.59E-01	6.62E-01	-3.89E+00	2.05E-01
ENSBTAG00000014259	3.47E-01	8.85E-01	1.34E+00	5.73E-05	2.21E+00	7.74E-13	1.51E+00	3.82E-06	2.30E-01	1.00E+00
ENSBTAG00000028847	0.00E+00	1.00E+00	0.00E+00	1.00E+00	-5.13E-15	1.00E+00	0.00E+00	1.00E+00	0.00E+00	1.00E+00
ENSBTAG00000004257	6.12E-01	5.10E-01	3.38E+00	3.92E-24	3.41E+00	9.20E-25	2.39E+00	1.55E-12	-3.93E-01	8.67E-01
ENSBTAG00000017698	-3.22E-01	1.00E+00	6.02E-01	7.74E-01	3.73E-01	9.87E-01	-2.53E-01	1.00E+00	-4.76E-01	1.00E+00
ENSBTAG00000011531	2.01E-01	9.05E-01	8.03E-02	1.00E+00	6.04E-01	5.88E-03	6.71E-01	2.29E-03	-2.33E-01	8.00E-01
ENSBTAG00000045044	0.00E+00	1.00E+00	0.00E+00	1.00E+00	-5.13E-15	1.00E+00	0.00E+00	1.00E+00	0.00E+00	1.00E+00
ENSBTAG00000007383	-5.30E-01	7.60E-01	-7.67E-01	1.26E-01	-4.83E-01	4.10E-01	-5.79E-01	2.89E-01	-7.95E-01	4.34E-01
ENSBTAG00000020467	-3.93E-01	1.00E+00	-1.10E+00	3.14E-01	-5.57E-01	6.86E-01	-1.96E-01	1.00E+00	-7.16E-01	8.97E-01
ENSBTAG00000003035	-1.07E-01	1.00E+00	1.23E-01	8.27E-01	8.26E-01	4.01E-05	4.71E-01	3.64E-02	-2.53E-01	7.77E-01
ENSBTAG00000023259	-8.57E-02	1.00E+00	-1.06E+00	2.35E-07	-1.93E+00	3.14E-21	-1.54E+00	3.71E-14	1.17E-01	1.00E+00
ENSBTAG00000045077	0.00E+00	1.00E+00	0.00E+00	1.00E+00	-5.13E-15	1.00E+00	0.00E+00	1.00E+00	0.00E+00	1.00E+00
ENSBTAG00000005638	-7.27E-02	1.00E+00	-1.34E+00	3.35E-02	-3.19E+00	1.35E-07	-3.76E+00	1.40E-09	1.90E-01	1.00E+00
ENSBTAG00000005635	-1.16E-01	1.00E+00	-1.28E+00	4.98E-03	-3.06E+00	8.68E-12	-2.80E+00	4.47E-10	-2.23E-02	1.00E+00
ENSBTAG00000027181	-4.56E-01	3.10E-01	-1.38E+00	3.00E-09	-2.71E+00	8.52E-30	-2.91E+00	2.36E-33	1.23E-01	1.00E+00
ENSBTAG00000039850	0.00E+00	1.00E+00	0.00E+00	1.00E+00	-5.13E-15	1.00E+00	0.00E+00	1.00E+00	0.00E+00	1.00E+00
ENSBTAG00000043389	0.00E+00	1.00E+00	0.00E+00	1.00E+00	-5.13E-15	1.00E+00	0.00E+00	1.00E+00	0.00E+00	1.00E+00
ENSBTAG00000000517	-6.28E-01	3.95E-01	-1.50E+00	8.26E-05	-7.12E-01	6.89E-02	-1.16E+00	1.91E-03	-1.47E-01	1.00E+00
ENSBTAG00000015195	5.71E-01	7.43E-01	1.23E+00	9.28E-03	1.42E+00	2.19E-03	1.29E+00	7.15E-03	1.91E-01	1.00E+00

ENSBTAG00000015190	3.12E-01	6.97E-01	1.30E+00	1.21E-08	1.26E+00	2.44E-08	7.62E-01	1.60E-03	8.58E-02	1.00E+00
ENSBTAG00000010042	6.00E-01	5.35E-02	1.87E+00	2.17E-18	1.42E+00	2.67E-11	8.96E-01	5.32E-05	-3.73E-01	5.04E-01
ENSBTAG00000010037	5.69E-01	3.39E-01	2.28E+00	9.66E-16	1.49E+00	1.99E-07	1.11E+00	2.01E-04	3.68E-01	7.80E-01
ENSBTAG00000008705	1.12E+00	6.69E-02	7.27E-01	1.43E-01	-3.25E-01	6.73E-01	-6.78E-01	2.00E-01	-3.25E-01	9.94E-01
ENSBTAG00000006743	3.84E-01	3.63E-01	4.73E-01	3.61E-02	2.91E-01	2.66E-01	3.47E-01	1.68E-01	-1.03E-01	1.00E+00
ENSBTAG00000044678	0.00E+00	1.00E+00	0.00E+00	1.00E+00	-5.13E-15	1.00E+00	0.00E+00	1.00E+00	0.00E+00	1.00E+00
ENSBTAG00000043262	1.92E+00	8.23E-01	0.00E+00	1.00E+00	-5.13E-15	1.00E+00	0.00E+00	1.00E+00	0.00E+00	1.00E+00
ENSBTAG00000005734	-2.89E-02	1.00E+00	-8.94E-01	3.06E-02	1.79E-01	9.35E-01	-2.85E-01	6.96E-01	6.02E-01	5.67E-01
ENSBTAG00000047113	-6.13E-01	5.76E-01	-1.36E+00	1.28E-03	-2.67E-01	7.59E-01	-2.65E-01	7.77E-01	9.74E-01	1.70E-01
ENSBTAG00000007467	9.69E-02	1.00E+00	-1.02E-01	1.00E+00	3.85E-01	3.20E-01	1.08E+00	3.75E-04	7.01E-01	1.98E-01
ENSBTAG00000029926	0.00E+00	1.00E+00	0.00E+00	1.00E+00	-5.13E-15	1.00E+00	0.00E+00	1.00E+00	0.00E+00	1.00E+00
ENSBTAG00000029906	0.00E+00	1.00E+00	0.00E+00	1.00E+00	-5.13E-15	1.00E+00	0.00E+00	1.00E+00	0.00E+00	1.00E+00
ENSBTAG00000005709	-1.25E-01	1.00E+00	3.81E-01	1.58E-01	-7.57E-01	1.31E-03	-6.01E-01	1.52E-02	4.21E-01	4.31E-01
ENSBTAG00000008292	4.42E-01	4.59E-01	1.64E+00	3.83E-11	1.73E+00	2.26E-12	1.22E+00	1.88E-06	-2.38E-01	9.12E-01
ENSBTAG00000017597	-3.38E-01	6.80E-01	-2.07E-01	6.26E-01	8.73E-02	1.00E+00	3.26E-02	1.00E+00	-4.24E-01	5.04E-01
ENSBTAG00000008275	-3.27E-02	1.00E+00	-2.12E+00	4.39E-14	-1.33E+00	7.93E-07	-5.44E-01	7.56E-02	4.99E-01	4.10E-01
ENSBTAG00000042470	0.00E+00	1.00E+00	0.00E+00	1.00E+00	-5.13E-15	1.00E+00	0.00E+00	1.00E+00	0.00E+00	1.00E+00
ENSBTAG00000044687	0.00E+00	1.00E+00	0.00E+00	1.00E+00	-5.13E-15	1.00E+00	0.00E+00	1.00E+00	0.00E+00	1.00E+00
ENSBTAG00000019199	1.92E+00	8.23E-01	0.00E+00	1.00E+00	2.55E+00	2.55E-01	0.00E+00	1.00E+00	1.91E+00	8.00E-01
ENSBTAG00000008403	1.81E-02	1.00E+00	-2.13E-01	5.81E-01	-7.56E-02	1.00E+00	3.50E-01	2.55E-01	3.13E-03	1.00E+00
ENSBTAG00000019214	9.90E-02	1.00E+00	7.51E-01	1.42E-03	5.04E-01	4.62E-02	5.45E-01	3.20E-02	-2.61E-01	8.00E-01
ENSBTAG00000019215	8.38E-02	1.00E+00	2.90E-01	2.24E-01	4.50E-01	3.06E-02	1.20E-01	8.10E-01	-3.18E-01	5.41E-01
ENSBTAG00000030086	0.00E+00	1.00E+00	0.00E+00	1.00E+00	-5.13E-15	1.00E+00	0.00E+00	1.00E+00	0.00E+00	1.00E+00
ENSBTAG00000007705	4.29E-01	8.47E-01	8.02E-01	8.71E-02	1.51E+00	2.14E-04	1.14E+00	8.25E-03	-8.25E-03	1.00E+00
ENSBTAG00000012320	0.00E+00	1.00E+00	0.00E+00	1.00E+00	-5.13E-15	1.00E+00	0.00E+00	1.00E+00	0.00E+00	1.00E+00
ENSBTAG00000045055	0.00E+00	1.00E+00	0.00E+00	1.00E+00	-5.13E-15	1.00E+00	0.00E+00	1.00E+00	0.00E+00	1.00E+00
ENSBTAG00000014012	-8.68E-01	1.00E+00	-7.88E-01	7.91E-01	-3.58E+00	4.62E-02	-1.84E-01	1.00E+00	-3.58E+00	3.10E-01
ENSBTAG00000007003	-1.20E-01	1.00E+00	-1.29E+00	6.43E-09	-1.87E+00	2.56E-17	-1.80E+00	4.07E-16	-4.65E-01	3.13E-01
ENSBTAG00000007000	-3.62E-02	1.00E+00	-6.23E-01	2.33E-02	-1.35E+00	8.11E-08	-1.26E+00	9.06E-07	1.78E-01	1.00E+00
ENSBTAG00000001523	9.09E-02	1.00E+00	7.58E-02	1.00E+00	2.33E-02	1.00E+00	4.45E-01	5.04E-02	-7.42E-02	1.00E+00
ENSBTAG00000020650	9.45E-01	9.39E-01	1.72E+00	1.21E-01	2.21E+00	1.73E-02	1.67E+00	1.31E-01	5.56E-04	1.00E+00
ENSBTAG00000015149	-7.98E-02	1.00E+00	-4.24E-01	5.04E-01	-1.11E+00	2.06E-02	-9.12E-01	6.65E-02	-1.71E+00	1.21E-02
ENSBTAG00000043195	0.00E+00	1.00E+00	0.00E+00	1.00E+00	-5.13E-15	1.00E+00	0.00E+00	1.00E+00	0.00E+00	1.00E+00
ENSBTAG00000042228	0.00E+00	1.00E+00	0.00E+00	1.00E+00	-5.13E-15	1.00E+00	0.00E+00	1.00E+00	0.00E+00	1.00E+00
ENSBTAG00000042870	0.00E+00	1.00E+00	0.00E+00	1.00E+00	-5.13E-15	1.00E+00	0.00E+00	1.00E+00	0.00E+00	1.00E+00

ENSBTAG00000021668	-3.98E-01	8.23E-01	-9.39E-01	1.64E-02	5.56E-02	1.00E+00	8.41E-02	1.00E+00	4.63E-01	6.47E-01
ENSBTAG00000021673	-5.23E-02	1.00E+00	-7.96E-01	2.49E-01	-5.73E-01	4.48E-01	1.80E-02	1.00E+00	-7.92E-01	6.27E-01
ENSBTAG00000044303	0.00E+00	1.00E+00	0.00E+00	1.00E+00	-5.13E-15	1.00E+00	0.00E+00	1.00E+00	0.00E+00	1.00E+00
ENSBTAG00000003354	-2.29E-01	9.44E-01	-1.45E-01	8.63E-01	-2.02E-01	6.62E-01	-2.39E-01	5.60E-01	-2.51E-01	8.57E-01
ENSBTAG00000003880	5.24E-01	6.24E-01	1.50E+00	4.50E-05	6.86E-01	1.03E-01	6.32E-01	1.53E-01	-2.54E-02	1.00E+00
ENSBTAG00000042981	0.00E+00	1.00E+00	0.00E+00	1.00E+00	-5.13E-15	1.00E+00	0.00E+00	1.00E+00	0.00E+00	1.00E+00
ENSBTAG00000012060	-3.02E-01	9.85E-01	-9.03E-01	2.14E-02	-8.22E-01	3.95E-02	-5.41E-01	2.43E-01	2.36E-01	1.00E+00
ENSBTAG00000019585	-5.17E-01	6.44E-01	-2.02E+00	6.98E-08	-3.87E+00	2.18E-22	-4.06E+00	6.90E-24	1.01E-01	1.00E+00
ENSBTAG00000016024	-1.48E-01	1.00E+00	-4.18E-01	4.79E-02	-6.89E-01	3.72E-04	-5.91E-01	3.32E-03	-2.64E-01	6.74E-01
ENSBTAG00000026266	2.53E-01	7.97E-01	7.17E-01	8.53E-04	1.39E-01	7.68E-01	1.89E-01	5.81E-01	-7.72E-02	1.00E+00
ENSBTAG00000007718	1.40E+00	2.92E-02	2.98E+00	8.97E-10	2.44E+00	3.58E-07	1.43E+00	4.40E-03	-2.73E-01	1.00E+00
ENSBTAG00000005372	5.17E-01	8.53E-01	8.50E-01	1.60E-01	-5.05E-01	4.92E-01	-1.31E+00	1.88E-02	7.39E-02	1.00E+00
ENSBTAG00000045020	0.00E+00	1.00E+00	0.00E+00	1.00E+00	-5.13E-15	1.00E+00	0.00E+00	1.00E+00	0.00E+00	1.00E+00
ENSBTAG00000046269	0.00E+00	1.00E+00	0.00E+00	1.00E+00	-5.13E-15	1.00E+00	0.00E+00	1.00E+00	0.00E+00	1.00E+00
ENSBTAG00000038970	1.95E-02	1.00E+00	-7.82E-01	2.87E-02	-6.33E-01	8.61E-02	-1.49E-01	9.48E-01	3.89E-01	7.79E-01
ENSBTAG00000019251	-2.19E-01	9.98E-01	-9.03E-01	1.37E-03	-2.96E-01	4.29E-01	-1.85E-01	7.49E-01	1.65E-01	1.00E+00
ENSBTAG00000046396	-2.65E-01	1.00E+00	-7.87E-01	9.11E-02	-6.84E-01	1.44E-01	-4.89E-01	3.66E-01	-1.05E+00	1.41E-01
ENSBTAG00000019036	1.90E-02	1.00E+00	-2.37E-01	1.00E+00	-9.83E-01	2.48E-01	9.17E-02	1.00E+00	-1.52E+00	3.45E-01
ENSBTAG00000018160	5.27E-01	1.00E+00	-4.71E-01	8.66E-01	2.22E-01	1.00E+00	9.07E-01	3.07E-01	5.64E-04	1.00E+00
ENSBTAG00000046046	-3.21E+00	3.75E-01	-4.18E-01	1.00E+00	-3.21E+00	9.51E-02	-3.21E+00	9.99E-02	-3.21E+00	4.34E-01
ENSBTAG00000024015	2.75E-01	6.75E-01	6.09E-01	3.38E-03	6.49E-01	1.58E-03	9.81E-01	8.47E-07	1.20E-01	1.00E+00
ENSBTAG00000043821	0.00E+00	1.00E+00	0.00E+00	1.00E+00	-5.13E-15	1.00E+00	0.00E+00	1.00E+00	0.00E+00	1.00E+00
ENSBTAG00000010356	-1.49E-01	1.00E+00	-8.48E-01	2.49E-05	-4.70E-01	2.96E-02	-4.68E-01	3.41E-02	-2.58E-01	7.37E-01
ENSBTAG00000009459	2.03E-01	1.00E+00	-9.72E-01	6.36E-03	-9.05E-02	1.00E+00	2.45E-01	7.02E-01	-9.75E-02	1.00E+00
ENSBTAG00000004871	5.92E-01	1.22E-01	9.49E-01	1.11E-04	2.55E-01	4.75E-01	-2.60E-01	4.77E-01	-2.36E-01	8.67E-01
ENSBTAG00000002755	7.83E-01	9.46E-02	3.28E+00	5.98E-25	2.55E+00	1.69E-16	1.39E+00	1.03E-05	-2.70E-01	9.23E-01
ENSBTAG00000001805	-2.40E-01	9.36E-01	1.28E-01	9.31E-01	-4.09E-01	2.07E-01	3.00E-01	4.36E-01	-9.90E-02	1.00E+00
ENSBTAG00000021250	-1.04E-01	1.00E+00	-1.16E-01	8.47E-01	4.70E-01	2.60E-02	5.98E-01	3.56E-03	-3.08E-02	1.00E+00
ENSBTAG00000009822	-3.99E-01	3.68E-01	-6.71E-01	2.68E-03	-3.06E-01	2.55E-01	-2.01E-01	5.50E-01	1.56E-01	1.00E+00
ENSBTAG00000046533	3.69E-01	1.00E+00	1.87E+00	1.76E-04	-3.41E-01	7.32E-01	1.35E-01	1.00E+00	5.84E-01	7.93E-01
ENSBTAG00000017276	5.13E-03	1.00E+00	2.52E+00	6.78E-02	-1.37E-01	1.00E+00	-1.91E+00	4.39E-01	1.30E+00	9.00E-01
ENSBTAG00000017279	-3.85E-02	1.00E+00	-2.80E-01	2.59E-01	-9.00E-01	3.68E-06	-7.44E-01	2.09E-04	-7.51E-02	1.00E+00
ENSBTAG00000005154	-4.14E-01	6.26E-01	-1.97E+00	2.30E-11	-2.76E+00	2.49E-20	-1.77E+00	1.72E-09	1.04E-01	1.00E+00
ENSBTAG00000005158	-1.01E-01	1.00E+00	-1.50E-01	6.36E-01	-1.83E-01	4.88E-01	1.63E-02	1.00E+00	1.51E-01	9.51E-01
ENSBTAG00000011171	-5.77E-02	1.00E+00	-1.55E+00	3.04E-03	-1.11E+00	2.69E-02	-4.46E-01	4.96E-01	-7.75E-01	5.09E-01

ENSBTAG00000042783	0.00E+00	1.00E+00	0.00E+00	1.00E+00	-5.13E-15	1.00E+00	0.00E+00	1.00E+00	0.00E+00	1.00E+00
ENSBTAG00000028631	0.00E+00	1.00E+00	0.00E+00	1.00E+00	-5.13E-15	1.00E+00	0.00E+00	1.00E+00	0.00E+00	1.00E+00
ENSBTAG00000002125	6.75E-02	1.00E+00	6.22E-01	6.64E-02	3.36E-01	4.28E-01	1.86E-01	8.17E-01	1.62E-01	1.00E+00
ENSBTAG00000002128	-4.39E-01	3.75E-01	-1.42E+00	2.93E-09	-2.56E+00	3.29E-26	-2.14E+00	4.07E-19	1.74E-01	1.00E+00
ENSBTAG00000043951	-4.33E-01	6.50E-01	-1.43E+00	4.25E-06	-3.33E+00	3.68E-25	-3.44E+00	2.56E-26	4.32E-01	6.63E-01
ENSBTAG00000046547	-4.52E-01	4.03E-01	-1.22E+00	2.29E-06	-1.27E+00	6.09E-07	-5.52E-01	5.43E-02	-5.79E-02	1.00E+00
ENSBTAG00000047609	-3.77E-01	8.23E-01	-1.33E+00	3.39E-04	-1.75E+00	1.62E-06	-1.09E+00	3.87E-03	-4.47E-01	7.65E-01
ENSBTAG00000046337	1.37E+00	1.22E-06	1.34E+00	2.94E-07	1.38E+00	9.97E-08	1.23E+00	3.17E-06	-4.25E-01	5.94E-01
ENSBTAG00000011250	3.09E-02	1.00E+00	5.02E-01	4.12E-02	3.15E-01	2.67E-01	3.11E-01	2.88E-01	6.35E-02	1.00E+00
ENSBTAG00000017983	-1.56E-01	1.00E+00	1.64E+00	6.47E-04	5.06E-01	4.73E-01	8.13E-01	1.62E-01	9.22E-01	4.03E-01
ENSBTAG00000010542	-2.12E-01	1.00E+00	-2.17E-01	7.74E-01	-3.58E-01	4.39E-01	-1.51E-01	9.53E-01	1.21E-02	1.00E+00
ENSBTAG00000046928	0.00E+00	1.00E+00	0.00E+00	1.00E+00	-5.13E-15	1.00E+00	0.00E+00	1.00E+00	0.00E+00	1.00E+00
ENSBTAG00000010547	-9.93E-02	1.00E+00	2.97E-01	4.43E-01	7.69E-01	6.14E-03	5.69E-01	6.39E-02	-9.64E-03	1.00E+00
ENSBTAG00000010552	3.97E-01	5.52E-01	1.31E+00	1.04E-07	1.83E+00	4.17E-14	1.50E+00	9.13E-10	-1.95E-01	9.96E-01
ENSBTAG00000010563	1.85E-01	8.81E-01	9.86E-01	7.29E-08	1.05E+00	8.04E-09	6.52E-01	7.07E-04	-2.15E-02	1.00E+00
ENSBTAG00000010792	2.72E-01	6.59E-01	1.62E+00	1.31E-17	2.39E+00	6.03E-36	1.77E+00	1.02E-20	-3.17E-01	5.62E-01
ENSBTAG00000013380	-2.94E-03	1.00E+00	2.05E+00	1.47E-12	1.26E+00	1.76E-05	-3.05E-01	4.97E-01	-5.91E-02	1.00E+00
ENSBTAG00000009139	-3.33E-02	1.00E+00	-6.65E-01	1.52E-01	9.60E-02	1.00E+00	8.98E-01	3.47E-02	3.17E-01	9.53E-01
ENSBTAG00000009141	-4.18E-02	1.00E+00	7.22E-01	4.77E-04	8.41E-01	3.27E-05	3.04E-01	2.42E-01	-1.88E-01	8.98E-01
ENSBTAG00000009142	4.83E-01	3.72E-01	2.02E+00	1.19E-15	1.93E+00	1.41E-14	1.55E+00	1.14E-09	9.13E-02	1.00E+00
ENSBTAG00000009143	1.92E+00	8.23E-01	0.00E+00	1.00E+00	-5.13E-15	1.00E+00	0.00E+00	1.00E+00	0.00E+00	1.00E+00
ENSBTAG00000011038	6.89E-01	1.00E+00	-4.29E-01	1.00E+00	-3.22E+00	9.40E-02	-6.84E-01	8.84E-01	-3.22E+00	4.34E-01
ENSBTAG00000046153	-7.58E-02	1.00E+00	-5.54E-01	4.69E-01	-7.36E-01	2.77E-01	5.01E-01	5.43E-01	-4.17E-01	9.80E-01
ENSBTAG00000018088	5.45E-01	8.23E-01	7.44E-01	2.04E-01	5.30E-01	4.28E-01	9.69E-01	6.68E-02	4.46E-01	8.97E-01
ENSBTAG00000043469	0.00E+00	1.00E+00	0.00E+00	1.00E+00	-5.13E-15	1.00E+00	0.00E+00	1.00E+00	0.00E+00	1.00E+00
ENSBTAG00000043507	-6.19E-04	1.00E+00	-1.91E+00	4.69E-01	-1.91E+00	4.28E-01	-1.91E+00	4.39E-01	-1.91E+00	8.00E-01
ENSBTAG00000002099	-2.21E-01	1.00E+00	1.81E+00	7.87E-04	7.29E-01	2.94E-01	-5.29E-01	5.70E-01	-2.58E-01	1.00E+00
ENSBTAG00000019870	-1.88E+00	1.63E-01	-1.80E+00	4.99E-02	-1.29E+00	1.34E-01	-2.38E-02	1.00E+00	-3.85E-01	1.00E+00
ENSBTAG00000016620	-3.57E-01	1.00E+00	-2.79E-01	1.00E+00	-1.59E-01	1.00E+00	-1.82E-01	1.00E+00	-3.57E-01	1.00E+00
ENSBTAG00000012067	2.82E-01	9.46E-01	2.95E-01	5.52E-01	6.69E-01	5.77E-02	9.44E-01	4.80E-03	6.18E-01	3.83E-01
ENSBTAG00000028153	0.00E+00	1.00E+00	0.00E+00	1.00E+00	-5.13E-15	1.00E+00	0.00E+00	1.00E+00	0.00E+00	1.00E+00
ENSBTAG00000002624	8.74E-01	2.85E-03	9.22E-01	1.65E-04	2.58E-01	4.64E-01	-1.59E-02	1.00E+00	-3.70E-01	6.01E-01
ENSBTAG00000002507	5.30E-02	1.00E+00	-2.11E-01	6.60E-01	-2.62E-01	5.04E-01	1.23E-01	9.52E-01	2.33E-01	9.20E-01
ENSBTAG00000002512	1.48E-02	1.00E+00	-5.54E-01	3.33E-02	-2.62E-01	4.28E-01	1.61E-01	7.54E-01	-7.13E-03	1.00E+00
ENSBTAG00000042092	0.00E+00	1.00E+00	0.00E+00	1.00E+00	-5.13E-15	1.00E+00	0.00E+00	1.00E+00	1.91E+00	8.00E-01

ENSBTAG00000032964	2.69E-01	8.23E-01	1.36E+00	1.50E-09	1.54E+00	3.53E-12	9.00E-01	1.37E-04	-3.58E-01	6.28E-01
ENSBTAG00000001128	-6.34E-01	1.00E+00	-1.23E+00	3.63E-01	-1.47E+00	2.26E-01	-1.50E+00	2.23E-01	-1.82E+00	5.01E-01
ENSBTAG00000015210	1.11E+00	7.80E-01	1.71E+00	7.27E-02	3.78E-01	1.00E+00	1.18E+00	3.10E-01	5.40E-01	1.00E+00
ENSBTAG00000001770	1.84E+00	1.81E-01	1.60E+00	1.05E-01	1.98E+00	1.94E-02	1.61E+00	9.31E-02	1.11E+00	7.80E-01
ENSBTAG00000007833	1.85E-01	9.32E-01	5.89E-01	5.12E-03	1.21E+00	4.96E-10	1.41E+00	5.00E-13	8.62E-02	1.00E+00
ENSBTAG00000043180	0.00E+00	1.00E+00	0.00E+00	1.00E+00	-5.13E-15	1.00E+00	0.00E+00	1.00E+00	0.00E+00	1.00E+00
ENSBTAG00000019331	9.26E-01	9.49E-01	7.50E-01	7.49E-01	1.12E+00	4.28E-01	1.12E+00	4.39E-01	9.10E-01	9.24E-01
ENSBTAG00000011893	-1.20E-01	1.00E+00	-2.39E-01	5.42E-01	3.58E-01	2.57E-01	5.13E-01	7.45E-02	6.24E-02	1.00E+00
ENSBTAG00000047809	3.20E-01	8.23E-01	2.26E-01	6.45E-01	6.58E-01	2.52E-02	9.87E-01	3.75E-04	3.00E-01	8.00E-01
ENSBTAG00000032961	2.16E-01	1.00E+00	1.07E+00	3.57E-04	1.09E+00	2.61E-04	6.52E-01	5.07E-02	-4.49E-01	6.01E-01
ENSBTAG00000032962	1.32E+00	9.28E-01	2.32E+00	1.14E-01	6.49E-01	9.78E-01	2.03E+00	1.99E-01	1.69E+00	7.74E-01
ENSBTAG00000046756	0.00E+00	1.00E+00	0.00E+00	1.00E+00	-5.13E-15	1.00E+00	0.00E+00	1.00E+00	0.00E+00	1.00E+00
ENSBTAG00000039916	-4.15E-02	1.00E+00	-1.42E+00	3.03E-10	-6.91E-01	2.86E-03	5.76E-02	1.00E+00	3.68E-02	1.00E+00
ENSBTAG00000013306	-4.13E-01	1.00E+00	-7.94E-01	4.34E-01	2.20E-01	1.00E+00	-1.06E+00	2.33E-01	-7.68E-01	8.00E-01
ENSBTAG00000043251	0.00E+00	1.00E+00	0.00E+00	1.00E+00	-5.13E-15	1.00E+00	0.00E+00	1.00E+00	0.00E+00	1.00E+00
ENSBTAG00000043142	0.00E+00	1.00E+00	0.00E+00	1.00E+00	-5.13E-15	1.00E+00	0.00E+00	1.00E+00	0.00E+00	1.00E+00
ENSBTAG00000018901	-5.41E-01	2.65E-01	-1.16E+00	1.46E-05	-6.50E-01	2.33E-02	-3.33E-01	3.61E-01	-8.12E-01	4.48E-02
ENSBTAG00000042168	0.00E+00	1.00E+00	0.00E+00	1.00E+00	-5.13E-15	1.00E+00	0.00E+00	1.00E+00	0.00E+00	1.00E+00
ENSBTAG00000012441	-1.88E+00	3.34E-08	-1.37E+00	1.56E-05	-6.30E-01	7.03E-02	-3.77E-01	3.79E-01	3.82E-01	7.69E-01
ENSBTAG00000046898	0.00E+00	1.00E+00	0.00E+00	1.00E+00	-5.13E-15	1.00E+00	0.00E+00	1.00E+00	0.00E+00	1.00E+00
ENSBTAG00000039363	0.00E+00	1.00E+00	0.00E+00	1.00E+00	-5.13E-15	1.00E+00	0.00E+00	1.00E+00	1.91E+00	8.00E-01
ENSBTAG00000000024	5.23E-02	1.00E+00	3.83E-01	8.34E-02	6.01E-01	2.64E-03	4.10E-01	6.45E-02	-1.62E-01	9.55E-01
ENSBTAG00000021334	1.72E-01	1.00E+00	5.63E-01	1.68E-02	1.80E-01	6.34E-01	1.13E-01	9.09E-01	-2.95E-01	7.11E-01
ENSBTAG00000022902	9.70E-01	2.07E-04	1.88E+00	2.21E-17	1.47E+00	4.14E-11	7.50E-01	1.86E-03	-4.11E-01	4.97E-01
ENSBTAG00000042368	0.00E+00	1.00E+00	0.00E+00	1.00E+00	-5.13E-15	1.00E+00	0.00E+00	1.00E+00	0.00E+00	1.00E+00
ENSBTAG00000043263	0.00E+00	1.00E+00	0.00E+00	1.00E+00	-5.13E-15	1.00E+00	0.00E+00	1.00E+00	0.00E+00	1.00E+00
ENSBTAG00000043301	0.00E+00	1.00E+00	0.00E+00	1.00E+00	-5.13E-15	1.00E+00	0.00E+00	1.00E+00	0.00E+00	1.00E+00
ENSBTAG00000042206	0.00E+00	1.00E+00	0.00E+00	1.00E+00	-5.13E-15	1.00E+00	0.00E+00	1.00E+00	0.00E+00	1.00E+00
ENSBTAG00000044412	0.00E+00	1.00E+00	0.00E+00	1.00E+00	-5.13E-15	1.00E+00	0.00E+00	1.00E+00	0.00E+00	1.00E+00
ENSBTAG00000039248	0.00E+00	1.00E+00	0.00E+00	1.00E+00	-5.13E-15	1.00E+00	0.00E+00	1.00E+00	0.00E+00	1.00E+00
ENSBTAG00000002863	-2.74E-01	8.23E-01	-7.96E-01	1.00E-03	-1.81E+00	4.62E-15	-2.32E+00	3.07E-23	-1.36E-01	1.00E+00
ENSBTAG00000045039	-4.01E-01	9.62E-01	-5.94E-01	3.56E-01	-1.21E+00	2.11E-02	-1.92E+00	3.84E-04	7.80E-02	1.00E+00
ENSBTAG00000045235	6.17E-01	8.88E-01	5.91E-01	5.56E-01	-1.03E+00	2.82E-01	-8.89E-01	3.92E-01	8.59E-02	1.00E+00
ENSBTAG00000019455	-7.45E-02	1.00E+00	-5.91E-01	5.42E-03	-1.27E+00	1.87E-10	-1.04E+00	2.79E-07	5.14E-01	1.39E-01
ENSBTAG00000002660	-1.74E-01	1.00E+00	1.55E+00	5.29E-10	6.59E-01	1.62E-02	-4.90E-01	1.17E-01	-5.61E-01	3.11E-01

ENSBTAG00000003801	1.45E-01	1.00E+00	8.63E-01	3.80E-06	6.21E-01	1.27E-03	4.72E-01	2.22E-02	1.37E-01	1.00E+00
ENSBTAG00000021884	-1.94E-01	8.94E-01	-9.14E-02	9.53E-01	3.17E-01	1.93E-01	1.46E-01	7.17E-01	6.17E-02	1.00E+00
ENSBTAG00000018216	-1.15E+00	3.47E-01	-1.51E+00	3.34E-02	-1.34E+00	4.44E-02	-5.29E-01	5.26E-01	-6.30E-01	8.00E-01
ENSBTAG00000009907	1.68E-01	1.00E+00	3.28E-01	6.38E-01	1.68E-01	9.79E-01	5.59E-01	2.60E-01	7.70E-02	1.00E+00
ENSBTAG00000047635	1.04E+00	4.21E-01	1.32E+00	3.98E-02	6.26E-01	4.70E-01	-2.46E-01	1.00E+00	-2.85E-01	1.00E+00
ENSBTAG00000016269	3.83E-01	5.72E-01	6.53E-01	1.24E-02	1.16E+00	1.51E-06	1.52E+00	1.77E-10	3.61E-01	6.21E-01
ENSBTAG00000005683	-9.98E-02	1.00E+00	-1.65E+00	4.98E-09	-1.33E+00	1.76E-06	-1.33E+00	2.21E-06	-2.90E-01	8.04E-01
ENSBTAG00000006919	2.21E-01	8.23E-01	1.18E+00	3.52E-10	5.99E-01	2.66E-03	4.05E-01	6.73E-02	-1.56E-01	9.71E-01
ENSBTAG00000034551	0.00E+00	1.00E+00	0.00E+00	1.00E+00	-5.13E-15	1.00E+00	0.00E+00	1.00E+00	0.00E+00	1.00E+00
ENSBTAG00000014886	-7.43E-02	1.00E+00	1.64E-01	8.50E-01	7.55E-01	9.23E-03	8.36E-01	3.96E-03	-5.27E-02	1.00E+00
ENSBTAG00000034555	0.00E+00	1.00E+00	0.00E+00	1.00E+00	-5.13E-15	1.00E+00	0.00E+00	1.00E+00	0.00E+00	1.00E+00
ENSBTAG00000045085	0.00E+00	1.00E+00	0.00E+00	1.00E+00	-5.13E-15	1.00E+00	0.00E+00	1.00E+00	0.00E+00	1.00E+00
ENSBTAG00000037457	2.39E-01	8.74E-01	4.33E-01	1.22E-01	6.14E-01	1.65E-02	5.39E-01	4.56E-02	-9.03E-02	1.00E+00
ENSBTAG00000009129	4.31E-02	1.00E+00	-7.38E-01	1.16E-02	-6.41E-01	3.23E-02	-4.86E-01	1.40E-01	-7.97E-02	1.00E+00
ENSBTAG00000008168	7.85E-01	8.68E-01	9.04E-01	4.13E-01	8.75E-01	4.19E-01	3.81E-01	9.38E-01	9.81E-01	7.50E-01
ENSBTAG00000008169	-9.52E-02	1.00E+00	-2.89E-01	7.33E-01	1.50E-01	1.00E+00	-2.67E-02	1.00E+00	2.07E-01	1.00E+00
ENSBTAG00000046901	-3.76E-01	1.00E+00	-1.44E-01	1.00E+00	-5.45E-01	6.82E-01	-1.80E+00	6.29E-02	-5.06E-01	1.00E+00
ENSBTAG00000042220	0.00E+00	1.00E+00	0.00E+00	1.00E+00	-5.13E-15	1.00E+00	0.00E+00	1.00E+00	0.00E+00	1.00E+00
ENSBTAG00000013871	-1.91E+00	8.23E-01	-1.91E+00	4.69E-01	-1.91E+00	4.28E-01	-1.48E-01	1.00E+00	-1.91E+00	8.00E-01
ENSBTAG00000002788	2.58E-01	1.00E+00	-3.80E-01	6.37E-01	4.52E-02	1.00E+00	-1.25E-01	1.00E+00	-1.91E-01	1.00E+00
ENSBTAG00000008158	-1.31E-01	1.00E+00	-4.90E-01	6.39E-01	-1.05E+00	1.47E-01	-1.43E+00	4.37E-02	-2.48E-01	1.00E+00
ENSBTAG00000016462	2.63E-01	1.00E+00	9.65E-01	2.39E-02	1.56E-01	1.00E+00	7.67E-01	9.59E-02	-3.96E-02	1.00E+00
ENSBTAG00000045116	0.00E+00	1.00E+00	0.00E+00	1.00E+00	-5.13E-15	1.00E+00	0.00E+00	1.00E+00	0.00E+00	1.00E+00
ENSBTAG00000015731	-9.72E-02	1.00E+00	-1.91E-02	1.00E+00	-1.09E-01	8.04E-01	-1.26E-01	7.29E-01	-2.96E-01	5.10E-01
ENSBTAG00000004005	-2.66E-01	7.09E-01	1.84E-02	1.00E+00	-5.87E-01	5.05E-03	-8.62E-01	2.28E-05	2.75E-01	6.79E-01
ENSBTAG00000007347	2.79E-01	1.00E+00	-1.44E+00	2.02E-01	-3.33E+00	4.61E-03	-1.42E+00	1.87E-01	4.98E-01	1.00E+00
ENSBTAG00000042835	1.92E+00	8.23E-01	3.30E+00	1.02E-01	1.78E+00	4.79E-01	2.53E+00	2.71E-01	0.00E+00	1.00E+00
ENSBTAG00000022819	2.21E-01	1.00E+00	2.21E+00	3.55E-13	1.43E+00	2.75E-06	1.16E+00	2.26E-04	6.76E-01	2.66E-01
ENSBTAG00000006393	1.68E-01	1.00E+00	-3.70E-01	1.29E-01	-9.73E-01	2.64E-06	-8.02E-01	1.70E-04	4.53E-01	2.73E-01
ENSBTAG00000019271	2.21E-01	8.23E-01	6.33E-01	1.43E-03	3.46E-01	1.26E-01	6.36E-02	1.00E+00	-3.54E-01	4.34E-01
ENSBTAG00000045186	1.92E+00	8.23E-01	2.79E+00	2.10E-01	1.78E+00	4.79E-01	2.53E+00	2.71E-01	3.22E+00	4.34E-01
ENSBTAG00000005738	5.38E-01	2.73E-01	-3.48E-01	3.28E-01	-1.22E+00	6.60E-06	1.61E-01	8.28E-01	2.10E-01	9.81E-01
ENSBTAG00000046411	-1.91E+00	8.23E-01	-1.91E+00	4.69E-01	-1.31E-01	1.00E+00	-1.91E+00	4.39E-01	6.46E-03	1.00E+00
ENSBTAG00000005412	-3.77E-01	6.50E-01	-2.14E+00	1.75E-15	-3.54E+00	9.72E-37	-2.93E+00	9.93E-27	6.40E-01	1.99E-01
ENSBTAG00000029851	-4.56E+00	7.30E-18	-4.24E+00	5.26E-17	-9.26E-01	6.24E-02	-1.04E+00	3.52E-02	3.81E-01	9.24E-01

ENSBTAG00000014284	-5.64E-01	8.22E-01	-1.68E-01	1.00E+00	7.44E-02	1.00E+00	-3.14E-01	7.57E-01	-1.12E+00	2.82E-01
ENSBTAG00000027916	4.70E-01	5.81E-01	1.41E-01	9.65E-01	9.53E-02	1.00E+00	3.43E-01	4.60E-01	2.63E-01	9.51E-01
ENSBTAG00000013588	3.90E-01	8.23E-01	-1.93E-01	8.68E-01	2.38E-01	7.24E-01	3.07E-01	5.66E-01	5.59E-01	5.16E-01
ENSBTAG00000022829	8.31E-01	9.16E-01	-5.79E-01	8.91E-01	4.69E-01	8.94E-01	1.31E+00	1.90E-01	4.86E-01	1.00E+00
ENSBTAG00000037994	0.00E+00	1.00E+00	0.00E+00	1.00E+00	-5.13E-15	1.00E+00	0.00E+00	1.00E+00	0.00E+00	1.00E+00
ENSBTAG00000011954	2.89E-01	8.23E-01	6.91E-01	8.24E-03	4.82E-01	8.97E-02	4.24E-01	1.59E-01	2.11E-01	9.37E-01
ENSBTAG00000004796	5.16E-01	1.00E+00	6.01E-01	9.45E-01	2.78E+00	3.09E-03	3.16E+00	3.49E-04	3.19E+00	7.44E-03
ENSBTAG00000022826	-1.29E+00	9.38E-01	-4.19E-01	1.00E+00	-3.21E+00	9.48E-02	1.94E-01	1.00E+00	-4.90E-01	1.00E+00
ENSBTAG00000038739	1.04E-02	1.00E+00	2.80E+00	4.18E-03	3.55E-01	1.00E+00	3.32E-01	1.00E+00	-7.86E-01	1.00E+00
ENSBTAG00000043771	0.00E+00	1.00E+00	0.00E+00	1.00E+00	-5.13E-15	1.00E+00	0.00E+00	1.00E+00	0.00E+00	1.00E+00
ENSBTAG00000006833	-9.65E-02	1.00E+00	-1.27E-01	9.18E-01	-3.84E-01	2.19E-01	8.40E-02	1.00E+00	8.79E-01	1.26E-02
ENSBTAG00000008723	6.38E-01	6.13E-01	5.91E-01	3.11E-01	4.94E-01	4.28E-01	8.69E-01	8.15E-02	7.58E-01	4.91E-01
ENSBTAG00000019676	-1.91E+00	8.23E-01	-1.91E+00	4.69E-01	-1.24E-01	1.00E+00	1.78E+00	3.07E-01	-1.91E+00	8.00E-01
ENSBTAG00000000531	0.00E+00	1.00E+00	2.79E+00	2.10E-01	2.55E+00	2.54E-01	2.53E+00	2.71E-01	1.93E+00	8.00E-01
ENSBTAG00000047569	-2.75E-01	1.00E+00	-1.29E+00	2.44E-01	-1.79E-01	1.00E+00	-3.20E-01	9.73E-01	-6.22E-01	9.80E-01
ENSBTAG00000001739	-5.60E-01	1.00E+00	-1.21E+00	3.35E-01	-8.80E-01	5.21E-01	-1.08E+00	4.17E-01	-4.65E-02	1.00E+00
ENSBTAG00000016663	2.33E-01	8.23E-01	-4.07E-01	1.02E-01	-2.06E-01	5.24E-01	-3.19E-01	2.48E-01	7.63E-02	1.00E+00
ENSBTAG00000016666	4.14E-02	1.00E+00	1.94E-01	5.36E-01	3.69E-01	1.20E-01	3.52E-02	1.00E+00	-4.24E-02	1.00E+00
ENSBTAG00000042865	0.00E+00	1.00E+00	0.00E+00	1.00E+00	-5.13E-15	1.00E+00	0.00E+00	1.00E+00	0.00E+00	1.00E+00
ENSBTAG00000007569	-6.03E-03	1.00E+00	-1.51E+00	7.61E-02	-2.70E+00	1.69E-03	-1.06E+00	2.25E-01	-7.79E-01	8.00E-01
ENSBTAG00000045832	1.31E-01	1.00E+00	1.21E+00	9.27E-07	7.42E-01	4.18E-03	7.75E-01	3.13E-03	2.54E-01	8.31E-01
ENSBTAG00000019302	1.76E-01	1.00E+00	3.25E-01	5.85E-01	1.79E-01	9.23E-01	8.91E-01	2.55E-02	4.26E-01	8.00E-01
ENSBTAG00000007723	-1.31E-01	1.00E+00	-9.22E-01	9.87E-05	-4.79E-01	6.43E-02	-1.56E-02	1.00E+00	1.92E-01	9.43E-01
ENSBTAG00000043400	-1.17E+00	8.67E-01	1.05E+00	3.71E-01	7.30E-02	1.00E+00	-3.89E+00	2.40E-02	4.58E-01	1.00E+00
ENSBTAG00000010492	-9.41E-02	1.00E+00	-5.48E-01	6.17E-03	2.14E-01	4.28E-01	2.65E-01	2.83E-01	-1.50E-01	9.80E-01
ENSBTAG00000002019	2.43E+00	3.11E-01	7.60E-02	1.00E+00	2.59E+00	4.97E-02	6.19E-01	9.96E-01	2.03E+00	6.01E-01
ENSBTAG00000035171	2.51E-02	1.00E+00	-1.22E+00	6.14E-01	-3.21E+00	9.50E-02	-1.45E+00	4.97E-01	-3.21E+00	4.34E-01
ENSBTAG00000035182	-1.91E+00	8.23E-01	6.90E-02	1.00E+00	-1.37E-01	1.00E+00	-1.91E+00	4.39E-01	8.15E-01	1.00E+00
ENSBTAG00000039037	3.91E+00	5.49E-05	6.27E+00	4.87E-15	7.28E+00	6.05E-20	8.16E+00	4.29E-24	2.22E+00	2.46E-01
ENSBTAG00000037608	0.00E+00	1.00E+00	0.00E+00	1.00E+00	-5.13E-15	1.00E+00	0.00E+00	1.00E+00	0.00E+00	1.00E+00
ENSBTAG00000037554	1.95E+00	6.47E-01	3.58E+00	4.06E-03	3.75E+00	1.69E-03	4.77E+00	1.32E-05	-1.92E+00	8.02E-01
ENSBTAG00000035224	-1.90E+00	8.26E-01	1.78E+00	3.42E-01	3.74E+00	7.55E-04	4.42E+00	1.55E-05	6.54E-03	1.00E+00
ENSBTAG00000040586	0.00E+00	1.00E+00	0.00E+00	1.00E+00	-5.13E-15	1.00E+00	0.00E+00	1.00E+00	0.00E+00	1.00E+00
ENSBTAG00000005324	0.00E+00	1.00E+00	0.00E+00	1.00E+00	-5.13E-15	1.00E+00	0.00E+00	1.00E+00	0.00E+00	1.00E+00
ENSBTAG00000037773	3.22E+00	3.75E-01	0.00E+00	1.00E+00	1.78E+00	4.79E-01	3.04E+00	1.49E-01	1.91E+00	8.00E-01

ENSBTAG00000023198	7.83E-01	1.00E+00	3.02E+00	2.67E-02	2.05E+00	2.20E-01	3.88E+00	1.16E-03	-1.90E+00	8.06E-01
ENSBTAG00000001102	-1.91E+00	8.23E-01	-1.91E+00	4.69E-01	-1.91E+00	4.28E-01	-1.91E+00	4.39E-01	8.00E-01	1.00E+00
ENSBTAG00000036962	-7.85E-01	1.00E+00	-7.23E-01	9.45E-01	1.82E+00	1.42E-01	6.89E-01	8.59E-01	-7.80E-01	1.00E+00
ENSBTAG00000046523	0.00E+00	1.00E+00	0.00E+00	1.00E+00	-5.13E-15	1.00E+00	0.00E+00	1.00E+00	0.00E+00	1.00E+00
ENSBTAG00000047565	0.00E+00	1.00E+00	0.00E+00	1.00E+00	-5.13E-15	1.00E+00	0.00E+00	1.00E+00	0.00E+00	1.00E+00
ENSBTAG00000023249	3.22E+00	3.75E-01	3.98E+00	2.58E-02	4.78E+00	1.43E-03	3.93E+00	2.72E-02	2.71E+00	6.01E-01
ENSBTAG00000007558	7.93E-01	6.26E-01	2.05E+00	1.23E-04	7.08E-01	3.35E-01	-5.12E-01	6.21E-01	9.84E-01	4.54E-01
ENSBTAG00000019803	4.40E-02	1.00E+00	2.37E-01	5.71E-01	3.85E-01	2.41E-01	4.10E-01	2.11E-01	-1.22E-01	1.00E+00
ENSBTAG00000019804	6.75E-02	1.00E+00	-2.63E-02	1.00E+00	4.13E-01	2.82E-01	6.95E-01	3.77E-02	-8.65E-02	1.00E+00
ENSBTAG00000019805	6.05E-01	3.39E-01	-2.64E-01	6.35E-01	-1.02E+00	2.26E-03	-3.88E-01	3.89E-01	2.16E-01	1.00E+00
ENSBTAG00000045693	-4.90E-01	1.00E+00	-3.21E+00	1.26E-01	-3.21E+00	9.49E-02	-3.21E+00	9.96E-02	-5.00E-01	1.00E+00
ENSBTAG00000026422	-4.02E-01	3.79E-01	-1.04E+00	2.73E-06	-1.38E+00	2.39E-10	-1.28E+00	6.00E-09	-1.30E-01	1.00E+00
ENSBTAG00000016564	-3.72E-01	4.69E-01	-1.24E+00	2.26E-08	-8.56E-01	1.32E-04	-6.15E-01	9.61E-03	3.59E-02	1.00E+00
ENSBTAG00000034024	-1.91E+00	8.23E-01	-1.91E+00	4.69E-01	-1.38E-01	1.00E+00	2.57E+00	5.68E-02	8.17E-01	1.00E+00
ENSBTAG00000038921	1.91E+00	8.26E-01	0.00E+00	1.00E+00	3.41E+00	8.68E-02	5.42E+00	3.32E-04	2.73E+00	6.03E-01
ENSBTAG00000038757	0.00E+00	1.00E+00	0.00E+00	1.00E+00	1.78E+00	4.79E-01	4.32E+00	9.50E-03	0.00E+00	1.00E+00
ENSBTAG00000026418	1.14E+00	8.23E-01	7.97E-02	1.00E+00	-6.68E-01	8.91E-01	1.42E+00	2.50E-01	2.07E+00	2.25E-01
ENSBTAG00000026417	-1.91E+00	8.23E-01	1.39E+00	5.17E-01	-1.91E+00	4.28E-01	1.13E+00	6.68E-01	1.70E+00	7.71E-01
ENSBTAG00000016567	-3.44E-01	9.62E-01	-1.23E+00	6.52E-03	-1.70E+00	1.12E-04	-2.29E+00	6.46E-07	3.88E-01	8.27E-01
ENSBTAG00000032198	1.56E-01	1.00E+00	-1.54E+00	1.65E-02	-2.51E+00	1.36E-04	-2.78E+00	4.99E-05	-7.27E-01	6.86E-01
ENSBTAG00000016568	-1.91E-01	9.53E-01	-4.37E-01	7.62E-02	6.34E-02	1.00E+00	-7.38E-01	1.12E-03	1.21E-01	1.00E+00
ENSBTAG00000016571	-2.37E-01	8.23E-01	-8.49E-01	1.78E-04	-1.21E+00	4.12E-08	-1.08E+00	1.52E-06	2.83E-01	7.58E-01
ENSBTAG00000016574	1.29E-03	1.00E+00	1.59E-01	1.00E+00	-8.20E-02	1.00E+00	-3.51E-01	7.97E-01	-5.01E-01	9.16E-01
ENSBTAG00000046883	0.00E+00	1.00E+00	0.00E+00	1.00E+00	-5.13E-15	1.00E+00	0.00E+00	1.00E+00	0.00E+00	1.00E+00
ENSBTAG00000027854	-2.71E+00	5.76E-01	-2.71E+00	2.42E-01	-2.71E+00	1.99E-01	-1.82E-01	1.00E+00	-7.98E-01	1.00E+00
ENSBTAG00000016575	-7.81E-01	1.00E+00	-2.70E+00	2.42E-01	-9.17E-01	8.09E-01	-2.70E+00	2.05E-01	-2.70E+00	6.01E-01
ENSBTAG00000016577	-2.29E-01	8.23E-01	-4.17E-02	1.00E+00	1.08E-01	8.88E-01	-3.92E-03	1.00E+00	-7.27E-02	1.00E+00
ENSBTAG00000016581	-3.23E-01	7.09E-01	-7.11E-01	5.40E-03	-1.03E+00	2.52E-05	-1.06E+00	1.72E-05	-1.88E-01	9.92E-01
ENSBTAG00000016588	-6.48E-01	3.31E-01	-2.42E+00	3.30E-12	-2.24E+00	5.36E-11	-1.77E+00	2.42E-07	4.32E-01	7.42E-01
ENSBTAG00000016590	-2.46E-01	8.23E-01	-1.14E+00	8.62E-08	-2.30E+00	4.02E-27	-2.77E+00	4.77E-37	4.07E-01	4.10E-01
ENSBTAG00000026429	-1.64E-01	9.83E-01	-1.08E+00	1.97E-08	-2.30E+00	1.31E-32	-1.80E+00	7.64E-21	1.44E-01	1.00E+00
ENSBTAG00000016591	-1.65E-01	1.00E+00	-8.57E-01	9.84E-05	-8.85E-01	4.99E-05	-9.43E-01	1.84E-05	4.92E-01	2.36E-01
ENSBTAG00000016592	-1.04E+00	8.16E-05	-6.49E-01	8.97E-03	-2.76E-01	3.99E-01	-6.53E-01	9.64E-03	1.03E-02	1.00E+00
ENSBTAG00000033739	1.69E+00	7.73E-01	1.39E+00	5.18E-01	6.44E-01	9.78E-01	1.50E+00	4.52E-01	1.31E+00	8.98E-01
ENSBTAG00000048251	0.00E+00	1.00E+00	1.98E+00	4.43E-01	-5.13E-15	1.00E+00	1.77E+00	4.97E-01	0.00E+00	1.00E+00

ENSBTAG00000016594	-3.05E-01	8.23E-01	-4.90E-01	1.06E-01	-9.73E-02	1.00E+00	-8.02E-02	1.00E+00	5.13E-02	1.00E+00
ENSBTAG00000007880	-2.08E-01	8.90E-01	-3.05E-02	1.00E+00	-2.74E-02	1.00E+00	3.79E-01	1.38E-01	-3.64E-01	5.09E-01
ENSBTAG00000002737	5.51E-01	8.23E-01	2.04E+00	1.06E-06	1.31E+00	4.08E-03	8.07E-01	1.40E-01	6.52E-01	6.68E-01
ENSBTAG00000002735	-3.02E-01	6.68E-01	-8.72E-01	8.39E-05	-1.34E+00	6.33E-10	-1.41E+00	8.01E-11	-2.41E-01	8.00E-01
ENSBTAG00000004551	-2.71E-01	7.42E-01	-2.99E-01	2.62E-01	-2.04E-02	1.00E+00	-1.40E-01	7.75E-01	2.06E-02	1.00E+00
ENSBTAG00000017284	-4.09E-01	1.00E+00	1.64E-01	1.00E+00	-8.07E-01	2.21E-01	-1.94E+00	1.91E-03	-4.09E-01	9.85E-01
ENSBTAG00000038330	-2.85E-01	8.23E-01	-8.54E-01	1.59E-03	-3.91E-01	2.25E-01	-4.46E-02	1.00E+00	-4.30E-01	5.36E-01
ENSBTAG00000019136	-1.06E-01	1.00E+00	-3.76E-01	1.38E-01	-3.87E-01	1.24E-01	-3.13E-01	2.61E-01	-2.15E-01	8.38E-01
ENSBTAG00000033672	-2.39E-01	8.02E-01	2.84E-01	2.55E-01	-3.86E-01	9.13E-02	-6.58E-01	1.73E-03	-7.86E-02	1.00E+00
ENSBTAG00000019006	1.42E-01	1.00E+00	1.18E+00	1.71E-08	1.46E+00	1.59E-12	9.91E-01	3.45E-06	8.71E-02	1.00E+00
ENSBTAG00000019008	-6.00E-01	3.92E-01	-2.96E+00	1.05E-12	-1.55E+00	1.14E-05	-1.66E+00	3.68E-06	2.79E-01	9.15E-01
ENSBTAG00000045857	0.00E+00	1.00E+00	0.00E+00	1.00E+00	-5.13E-15	1.00E+00	0.00E+00	1.00E+00	0.00E+00	1.00E+00
ENSBTAG00000033677	0.00E+00	1.00E+00	0.00E+00	1.00E+00	-5.13E-15	1.00E+00	0.00E+00	1.00E+00	0.00E+00	1.00E+00
ENSBTAG00000020198	6.83E-01	1.00E+00	7.62E-01	7.38E-01	-6.62E-01	8.92E-01	-6.85E-01	8.84E-01	-1.31E+00	9.00E-01
ENSBTAG00000002467	-6.71E-01	2.07E-01	-9.11E-01	4.86E-03	-9.68E-01	2.44E-03	-1.32E+00	2.71E-05	-4.05E-01	7.22E-01
ENSBTAG00000002470	-7.31E-01	3.14E-02	-1.18E+00	3.59E-06	-2.53E+00	3.54E-21	-2.23E+00	2.00E-17	1.61E-02	1.00E+00
ENSBTAG00000002481	-2.64E-01	8.87E-01	-5.45E-01	7.62E-02	-1.01E+00	2.60E-04	-1.25E+00	5.99E-06	-2.11E-01	9.85E-01
ENSBTAG00000002484	-4.74E-01	2.45E-01	-8.27E-02	1.00E+00	2.09E-01	5.51E-01	-3.64E-02	1.00E+00	-3.55E-01	5.98E-01
ENSBTAG00000000177	-1.04E+00	4.64E-01	-1.26E+00	7.11E-02	-9.43E-01	2.10E-01	-1.74E+00	9.67E-03	-1.35E+00	2.87E-01
ENSBTAG00000033580	-1.65E-01	1.00E+00	-2.92E-01	1.00E+00	-3.72E-04	1.00E+00	2.54E-01	1.00E+00	1.70E-02	1.00E+00
ENSBTAG00000000179	-9.04E-01	1.01E-02	-2.00E+00	5.44E-12	-8.10E-02	1.00E+00	-1.56E-01	8.45E-01	2.36E-02	1.00E+00
ENSBTAG00000019743	-6.76E-01	3.79E-01	-2.64E+00	2.91E-09	-2.29E+00	2.88E-08	-1.53E+00	1.26E-04	-1.69E-01	1.00E+00
ENSBTAG00000033526	0.00E+00	1.00E+00	0.00E+00	1.00E+00	-5.13E-15	1.00E+00	0.00E+00	1.00E+00	0.00E+00	1.00E+00
ENSBTAG00000019745	-4.74E-02	1.00E+00	-7.39E-01	1.05E-03	-8.57E-01	1.04E-04	-9.27E-01	3.09E-05	-2.93E-01	7.09E-01
ENSBTAG00000020737	-8.71E-01	1.00E+00	-1.60E+00	4.08E-01	-1.81E+00	2.94E-01	-1.81E-01	1.00E+00	2.14E-02	1.00E+00
ENSBTAG00000039974	-1.91E+00	8.23E-01	-1.91E+00	4.69E-01	-1.91E+00	4.28E-01	-1.91E+00	4.39E-01	-1.91E+00	8.00E-01
ENSBTAG00000033481	0.00E+00	1.00E+00	1.98E+00	4.43E-01	-5.13E-15	1.00E+00	0.00E+00	1.00E+00	0.00E+00	1.00E+00
ENSBTAG00000012601	2.72E+00	5.76E-01	0.00E+00	1.00E+00	1.78E+00	4.79E-01	1.76E+00	4.97E-01	0.00E+00	1.00E+00
ENSBTAG00000026461	2.00E-01	1.00E+00	-5.24E-01	1.95E-01	-4.17E-01	3.36E-01	-3.10E-01	5.37E-01	3.66E-01	8.00E-01
ENSBTAG00000016520	-7.54E-01	1.00E+00	-6.73E-01	7.45E-01	-2.57E+00	5.59E-02	-9.58E-01	5.04E-01	4.77E-01	1.00E+00
ENSBTAG00000007325	1.61E-02	1.00E+00	6.05E-01	9.45E-01	-9.17E-01	8.09E-01	-2.70E+00	2.05E-01	1.45E+00	7.49E-01
ENSBTAG00000046722	-1.91E+00	8.23E-01	6.91E-02	1.00E+00	-1.91E+00	4.28E-01	-1.91E+00	4.39E-01	3.84E-04	1.00E+00
ENSBTAG00000046965	0.00E+00	1.00E+00	0.00E+00	1.00E+00	2.56E+00	2.54E-01	1.76E+00	4.97E-01	0.00E+00	1.00E+00
ENSBTAG00000038866	1.42E-01	1.00E+00	7.62E-01	6.19E-05	7.21E-01	1.53E-04	2.05E-01	4.67E-01	7.10E-02	1.00E+00
ENSBTAG00000046876	-6.19E-04	1.00E+00	-1.91E+00	4.69E-01	6.44E-01	9.78E-01	-1.91E+00	4.39E-01	-1.91E+00	8.00E-01

ENSBTAG00000014100	-5.16E-01	1.00E+00	7.88E-01	2.80E-01	-1.41E-01	1.00E+00	-1.90E+00	2.01E-02	-8.37E-01	7.77E-01
ENSBTAG00000014101	-1.60E-01	1.00E+00	-4.63E-01	1.75E-01	3.67E-01	3.17E-01	4.91E-01	1.46E-01	-5.26E-01	4.34E-01
ENSBTAG00000014118	5.44E-02	1.00E+00	3.04E-01	1.70E-01	1.87E-01	4.79E-01	-5.08E-01	1.07E-02	-1.43E-01	9.90E-01
ENSBTAG00000014126	-5.93E-01	1.82E-01	-9.38E-01	6.83E-04	-7.97E-01	3.75E-03	-4.20E-01	1.96E-01	1.59E-01	1.00E+00
ENSBTAG00000033395	-6.72E-02	1.00E+00	1.17E+00	4.62E-06	1.29E+00	4.03E-07	1.08E+00	3.44E-05	-5.58E-02	1.00E+00
ENSBTAG00000000568	-1.97E+00	3.50E-01	6.12E-01	6.18E-01	7.22E-01	4.75E-01	2.00E-01	1.00E+00	-3.39E-01	1.00E+00
ENSBTAG00000015889	-1.37E-01	1.00E+00	-2.09E-01	7.42E-01	-3.21E-01	4.53E-01	-3.87E-01	3.39E-01	4.64E-02	1.00E+00
ENSBTAG00000005148	1.91E+00	8.23E-01	0.00E+00	1.00E+00	-5.13E-15	1.00E+00	0.00E+00	1.00E+00	0.00E+00	1.00E+00
ENSBTAG00000033389	4.32E-01	6.25E-01	3.96E-01	3.21E-01	9.05E-01	3.73E-03	1.46E+00	1.12E-06	-1.39E-01	1.00E+00
ENSBTAG00000019086	-3.12E-01	7.43E-01	-5.03E-01	6.75E-02	-1.30E-01	8.75E-01	-4.07E-01	1.68E-01	3.27E-01	6.80E-01
ENSBTAG00000007245	-3.00E-01	8.23E-01	-1.09E+00	1.29E-04	-6.24E-01	3.49E-02	-6.07E-01	4.62E-02	3.23E-01	7.79E-01
ENSBTAG00000007246	0.00E+00	1.00E+00	0.00E+00	1.00E+00	-5.13E-15	1.00E+00	0.00E+00	1.00E+00	0.00E+00	1.00E+00
ENSBTAG00000002202	-6.54E-02	1.00E+00	3.84E-01	2.92E-01	7.12E-01	1.74E-02	5.72E-01	7.64E-02	5.88E-01	3.11E-01
ENSBTAG00000002209	-5.06E-03	1.00E+00	2.69E-01	3.12E-01	7.76E-01	1.28E-04	9.49E-01	2.47E-06	3.93E-02	1.00E+00
ENSBTAG00000002211	2.58E-02	1.00E+00	-1.95E-02	1.00E+00	5.74E-01	3.53E-02	1.39E-01	8.72E-01	5.64E-01	2.45E-01
ENSBTAG00000016552	-4.05E-01	4.65E-01	-8.93E-01	2.90E-04	-1.27E+00	1.23E-07	-1.43E+00	3.11E-09	-5.39E-01	2.53E-01
ENSBTAG00000026415	-3.76E-01	7.57E-01	-8.99E-01	3.77E-03	-6.86E-01	3.45E-02	-3.45E-01	4.21E-01	-3.52E-01	8.00E-01
ENSBTAG00000016553	-2.16E-02	1.00E+00	-3.26E-01	3.66E-01	7.49E-03	1.00E+00	-1.60E-01	8.24E-01	-8.95E-02	1.00E+00
ENSBTAG00000016558	-1.27E-01	1.00E+00	-1.32E-02	1.00E+00	-8.11E-02	1.00E+00	-4.95E-01	4.19E-02	2.04E-01	8.85E-01
ENSBTAG00000016561	2.56E-02	1.00E+00	7.47E-01	1.77E-03	8.66E-01	2.05E-04	7.22E-01	3.05E-03	-2.13E-01	9.02E-01
ENSBTAG00000033299	-2.33E-01	9.83E-01	-1.06E+00	1.83E-04	-2.66E+00	2.94E-21	-3.05E+00	1.45E-26	2.65E-01	8.76E-01
ENSBTAG00000019998	-2.94E-01	8.23E-01	-8.13E-01	2.76E-03	-2.03E+00	4.18E-15	-2.34E+00	2.70E-19	-1.23E-01	1.00E+00
ENSBTAG00000033015	-3.95E-01	6.27E-01	-1.98E+00	9.52E-13	-1.38E+00	5.88E-07	-7.90E-01	7.87E-03	-4.33E-01	5.93E-01
ENSBTAG00000048229	-7.33E-01	5.00E-01	-2.57E+00	3.20E-06	-2.98E+00	5.78E-08	-3.57E+00	1.55E-09	-1.39E+00	6.09E-02
ENSBTAG00000020001	2.84E-02	1.00E+00	-1.56E-01	9.62E-01	-5.30E-01	2.24E-01	-7.26E-01	7.40E-02	-3.30E-01	8.87E-01
ENSBTAG00000019249	-9.24E-01	3.39E-01	-1.20E+00	2.66E-02	-3.70E+00	2.19E-09	-3.74E+00	1.97E-09	1.67E-01	1.00E+00
ENSBTAG00000009533	2.28E-02	1.00E+00	-1.09E+00	4.45E-03	-1.60E+00	1.79E-05	-1.76E+00	3.54E-06	-2.83E-01	9.58E-01
ENSBTAG00000009534	-3.17E-01	7.91E-01	-7.15E-01	1.02E-02	-9.32E-01	4.99E-04	-9.97E-01	2.22E-04	-4.36E-01	5.29E-01
ENSBTAG00000009535	-7.26E-02	1.00E+00	-2.71E-03	1.00E+00	1.97E-02	1.00E+00	2.17E-01	6.59E-01	3.32E-01	7.79E-01
ENSBTAG00000042394	0.00E+00	1.00E+00	0.00E+00	1.00E+00	-5.13E-15	1.00E+00	0.00E+00	1.00E+00	0.00E+00	1.00E+00
ENSBTAG00000042688	0.00E+00	1.00E+00	0.00E+00	1.00E+00	-5.13E-15	1.00E+00	0.00E+00	1.00E+00	0.00E+00	1.00E+00
ENSBTAG00000045327	5.04E-01	7.50E-01	1.83E+00	1.32E-07	2.20E+00	2.78E-11	1.34E+00	2.61E-04	-2.03E-01	1.00E+00
ENSBTAG00000009536	-7.86E-01	1.00E+00	8.80E-02	1.00E+00	1.27E+00	4.28E-01	2.30E+00	3.37E-02	-7.85E-01	1.00E+00
ENSBTAG00000020366	-2.32E-04	1.00E+00	7.49E-01	1.92E-03	1.25E+00	4.87E-08	1.09E+00	3.21E-06	-1.21E-01	1.00E+00
ENSBTAG00000020369	2.55E-01	1.00E+00	1.37E+00	1.04E-02	-7.34E-01	4.02E-01	-1.26E+00	1.09E-01	2.52E-01	1.00E+00

ENSBTAG00000017631	-1.31E-02	1.00E+00	6.67E-01	5.34E-03	6.82E-01	4.09E-03	4.81E-01	6.65E-02	-4.43E-01	4.15E-01
ENSBTAG00000008504	-2.14E-01	1.00E+00	-1.13E+00	1.30E-01	-1.70E+00	1.56E-02	-1.42E+00	4.53E-02	6.22E-01	7.80E-01
ENSBTAG00000001023	2.67E-01	8.12E-01	1.87E+00	2.38E-17	1.87E+00	1.39E-17	1.08E+00	1.49E-06	-1.96E-01	9.23E-01
ENSBTAG00000046703	0.00E+00	1.00E+00	1.98E+00	4.43E-01	-5.13E-15	1.00E+00	0.00E+00	1.00E+00	0.00E+00	1.00E+00
ENSBTAG00000013479	-5.35E-01	1.01E-01	-1.70E+00	8.21E-16	-2.82E+00	7.10E-39	-2.68E+00	1.87E-35	2.67E-01	7.72E-01
ENSBTAG00000006272	-2.24E-01	1.00E+00	-1.69E+00	3.71E-09	-1.98E+00	3.92E-12	-1.79E+00	3.98E-10	2.15E-01	9.96E-01
ENSBTAG00000020617	-8.59E-02	1.00E+00	-3.62E-01	1.20E-01	-2.59E-01	3.25E-01	-3.23E-01	1.93E-01	1.95E-02	1.00E+00
ENSBTAG00000020619	-1.45E-01	1.00E+00	-8.42E-01	4.13E-04	-1.31E-01	8.50E-01	-2.84E-01	3.82E-01	2.30E-01	8.43E-01
ENSBTAG00000037782	0.00E+00	1.00E+00	0.00E+00	1.00E+00	-5.13E-15	1.00E+00	0.00E+00	1.00E+00	0.00E+00	1.00E+00
ENSBTAG00000000279	-8.03E-01	4.40E-02	-1.41E+00	1.25E-06	-2.48E+00	1.02E-16	-2.65E+00	1.94E-18	6.06E-01	2.70E-01
ENSBTAG00000042751	0.00E+00	1.00E+00	0.00E+00	1.00E+00	3.43E+00	7.35E-02	2.53E+00	2.71E-01	0.00E+00	1.00E+00
ENSBTAG00000019073	9.16E-02	1.00E+00	2.85E-01	2.39E-01	2.56E-01	3.09E-01	1.29E-01	7.74E-01	-3.00E-01	6.01E-01
ENSBTAG00000037945	-3.47E-01	7.41E-01	-1.11E+00	6.66E-05	-2.08E+00	1.19E-13	-2.60E+00	3.28E-19	-2.34E-01	9.20E-01
ENSBTAG00000009928	-3.37E-01	6.88E-01	-1.03E+00	5.31E-05	-5.10E-01	6.93E-02	5.53E-03	1.00E+00	-6.41E-02	1.00E+00
ENSBTAG00000009945	-1.12E+00	2.58E-01	7.87E-01	1.48E-01	-7.59E-01	2.37E-01	-1.33E+00	2.66E-02	-1.68E+00	6.79E-02
ENSBTAG00000037500	-1.43E+00	8.30E-02	3.67E-01	7.18E-01	4.88E-01	5.09E-01	-5.99E-01	4.27E-01	-1.86E+00	2.52E-02
ENSBTAG00000044606	-5.33E-01	8.06E-01	2.76E-01	7.96E-01	4.79E-01	4.28E-01	-3.68E-01	6.30E-01	-1.05E+00	2.44E-01
ENSBTAG00000009951	-2.92E-01	1.00E+00	6.98E-01	9.47E-02	1.52E+00	3.08E-05	1.63E+00	1.02E-05	-5.52E-01	6.03E-01
ENSBTAG00000009961	-4.13E-01	3.33E-01	-2.83E-01	3.16E-01	3.13E-02	1.00E+00	-3.07E-01	2.67E-01	-1.16E-01	1.00E+00
ENSBTAG00000009964	-1.84E-02	1.00E+00	1.56E+00	4.10E-03	6.75E-01	3.78E-01	-4.87E-01	6.89E-01	3.76E-01	1.00E+00
ENSBTAG00000009965	-1.98E-01	9.93E-01	-7.29E-01	3.52E-03	-2.09E+00	1.16E-18	-2.78E+00	1.24E-30	1.12E-01	1.00E+00
ENSBTAG00000009969	1.03E-01	1.00E+00	4.74E-01	1.61E-02	3.68E-01	7.81E-02	1.11E-01	8.30E-01	1.72E-01	8.92E-01
ENSBTAG00000037348	-1.82E+00	4.55E-01	-1.23E+00	3.62E-01	-2.75E+00	3.16E-02	-2.78E+00	3.32E-02	1.60E-01	1.00E+00
ENSBTAG00000014903	1.23E+00	2.87E-05	1.94E+00	1.49E-13	1.26E+00	1.80E-06	8.96E-01	1.18E-03	-1.57E-01	1.00E+00
ENSBTAG00000034643	2.27E+00	1.82E-06	4.74E+00	3.52E-25	2.65E+00	9.93E-10	1.02E+00	3.85E-02	-1.48E-01	1.00E+00
ENSBTAG00000006591	-7.65E-02	1.00E+00	2.46E+00	4.94E-05	2.39E+00	7.98E-05	1.10E+00	1.32E-01	1.02E-01	1.00E+00
ENSBTAG00000024884	-1.86E-02	1.00E+00	-4.82E-01	7.55E-02	5.24E-01	4.21E-02	4.63E-01	8.95E-02	3.45E-03	1.00E+00
ENSBTAG00000045625	-8.53E-01	7.80E-01	-1.13E+00	2.12E-01	-3.37E-01	8.84E-01	-3.74E-01	8.42E-01	-1.04E+00	6.24E-01
ENSBTAG00000005458	-1.95E-01	1.00E+00	-8.55E-01	3.15E-03	-9.23E-02	1.00E+00	4.36E-01	1.68E-01	6.51E-02	1.00E+00
ENSBTAG00000026428	3.17E-01	4.49E-01	8.41E-01	4.72E-06	7.85E-01	2.04E-05	6.27E-01	1.11E-03	5.71E-02	1.00E+00
ENSBTAG00000001022	-3.56E-02	1.00E+00	-2.98E-01	2.92E-01	-2.19E-01	4.87E-01	-1.44E-01	7.77E-01	-5.95E-02	1.00E+00
ENSBTAG00000034436	-4.37E-01	5.84E-01	1.10E-01	1.00E+00	8.67E-02	1.00E+00	5.37E-01	1.16E-01	4.35E-01	6.01E-01
ENSBTAG00000040575	1.04E-01	1.00E+00	1.24E+00	7.27E-10	1.56E+00	3.72E-15	7.03E-01	1.10E-03	-1.51E-01	1.00E+00
ENSBTAG00000040394	-2.71E+00	5.76E-01	-7.23E-01	9.45E-01	3.45E-01	1.00E+00	-1.76E-01	1.00E+00	-7.80E-01	1.00E+00
ENSBTAG00000020582	5.09E-02	1.00E+00	-8.34E-01	6.25E-01	4.60E-01	9.01E-01	2.39E+00	1.01E-02	6.58E-03	1.00E+00

ENSBTAG00000019103	0.00E+00	1.00E+00	0.00E+00	1.00E+00	1.78E+00	4.79E-01	0.00E+00	1.00E+00	1.91E+00	8.00E-01
ENSBTAG00000034372	1.66E+00	8.23E-01	-7.49E-01	9.88E-01	2.35E+00	1.89E-01	3.90E+00	1.38E-02	2.94E+00	3.51E-01
ENSBTAG00000047368	5.51E-03	1.00E+00	-1.91E+00	4.70E-01	1.82E+00	2.81E-01	2.02E+00	2.05E-01	6.85E-03	1.00E+00
ENSBTAG00000015045	-1.43E+00	7.58E-01	-1.36E+00	3.91E-01	-2.37E+00	9.73E-02	-4.15E+00	1.20E-02	-5.49E-01	1.00E+00
ENSBTAG00000038568	-2.72E-01	1.00E+00	-2.00E-01	1.00E+00	-1.55E+00	1.54E-01	-6.65E-01	6.46E-01	1.37E+00	3.40E-01
ENSBTAG00000034368	-5.49E-01	1.00E+00	-1.35E+00	3.92E-01	-1.10E+00	4.68E-01	-2.09E-01	1.00E+00	-5.47E-01	1.00E+00
ENSBTAG00000019543	-2.90E-01	8.47E-01	-4.32E-01	2.12E-01	-3.47E-01	3.59E-01	-5.02E-01	1.36E-01	-6.27E-02	1.00E+00
ENSBTAG00000012683	2.10E-02	1.00E+00	3.35E-01	1.60E-01	1.55E-01	6.66E-01	-3.00E-01	2.36E-01	7.03E-01	1.10E-02
ENSBTAG00000038115	-8.47E-01	1.61E-01	-1.95E+00	3.90E-07	-2.55E+00	4.92E-11	-1.03E+00	8.91E-03	7.23E-02	1.00E+00
ENSBTAG00000006117	-3.20E-01	8.85E-01	-1.12E+00	9.86E-04	-2.45E+00	2.16E-13	-1.70E+00	3.39E-07	5.29E-02	1.00E+00
ENSBTAG00000020190	-2.60E-03	1.00E+00	5.89E-01	9.50E-01	-9.36E-01	8.05E-01	6.87E-01	8.60E-01	5.01E-01	1.00E+00
ENSBTAG00000004727	2.11E-01	1.00E+00	-2.38E-01	9.02E-01	2.71E-01	7.91E-01	4.29E-02	1.00E+00	1.36E-01	1.00E+00
ENSBTAG00000004729	-8.64E-01	2.34E-01	-8.65E-01	6.16E-02	-6.96E-01	1.43E-01	-4.65E-01	4.16E-01	-5.62E-01	6.68E-01
ENSBTAG00000047563	0.00E+00	1.00E+00	0.00E+00	1.00E+00	-5.13E-15	1.00E+00	0.00E+00	1.00E+00	0.00E+00	1.00E+00
ENSBTAG00000045901	1.52E+00	2.55E-01	-2.36E+00	1.06E-01	-2.56E+00	6.29E-02	1.25E+00	1.73E-01	-7.45E-01	1.00E+00
ENSBTAG00000012082	1.35E+00	1.70E-01	4.82E+00	2.13E-14	5.08E+00	1.08E-15	4.79E+00	3.08E-14	-5.67E-01	8.99E-01
ENSBTAG00000002367	-2.39E-01	1.00E+00	-7.77E-01	3.42E-02	-9.89E-01	4.65E-03	-1.09E+00	1.89E-03	-7.10E-02	1.00E+00
ENSBTAG00000001415	-1.16E-01	1.00E+00	3.03E-01	5.58E-01	9.35E-01	5.20E-03	9.95E-01	3.18E-03	5.07E-01	6.01E-01
ENSBTAG00000019920	-2.95E-01	1.00E+00	-9.43E-01	1.92E-01	-5.73E-01	4.79E-01	1.50E-01	1.00E+00	4.40E-03	1.00E+00
ENSBTAG00000016424	1.70E+00	1.52E-03	2.73E+00	4.97E-10	2.18E+00	8.22E-07	2.40E+00	6.59E-08	7.87E-01	5.05E-01
ENSBTAG00000034238	2.64E+00	2.05E-01	3.50E+00	1.79E-03	1.82E+00	2.82E-01	6.21E-01	9.96E-01	6.13E-03	1.00E+00
ENSBTAG00000005572	-3.37E-02	1.00E+00	3.79E-01	1.58E-01	6.39E-01	6.34E-03	6.21E-02	1.00E+00	-1.68E-02	1.00E+00
ENSBTAG00000026408	-4.11E-01	8.23E-01	-1.86E-01	9.06E-01	2.21E-01	8.05E-01	5.01E-01	2.77E-01	-3.95E-01	8.00E-01
ENSBTAG00000021407	-1.12E-01	1.00E+00	-3.02E+00	3.10E-01	-4.27E+00	1.29E-01	-2.81E+00	3.67E-01	5.36E-01	1.00E+00
ENSBTAG00000046326	0.00E+00	1.00E+00	0.00E+00	1.00E+00	-5.13E-15	1.00E+00	0.00E+00	1.00E+00	0.00E+00	1.00E+00
ENSBTAG00000043681	0.00E+00	1.00E+00	0.00E+00	1.00E+00	-5.13E-15	1.00E+00	0.00E+00	1.00E+00	0.00E+00	1.00E+00
ENSBTAG00000047634	0.00E+00	1.00E+00	0.00E+00	1.00E+00	1.78E+00	4.79E-01	0.00E+00	1.00E+00	0.00E+00	1.00E+00
ENSBTAG00000047615	3.89E+00	1.42E-01	2.79E+00	2.11E-01	1.78E+00	4.79E-01	3.71E+00	4.66E-02	3.60E+00	3.09E-01
ENSBTAG00000040438	-1.91E+00	8.23E-01	-1.91E+00	4.69E-01	-1.91E+00	4.28E-01	-1.91E+00	4.39E-01	-1.91E+00	8.00E-01
ENSBTAG00000042452	0.00E+00	1.00E+00	0.00E+00	1.00E+00	-5.13E-15	1.00E+00	0.00E+00	1.00E+00	0.00E+00	1.00E+00
ENSBTAG00000005195	-3.96E-01	5.81E-01	1.34E+00	5.13E-08	1.48E+00	1.02E-09	8.24E-01	1.73E-03	-2.21E-01	9.48E-01
ENSBTAG00000042525	0.00E+00	1.00E+00	0.00E+00	1.00E+00	-5.13E-15	1.00E+00	0.00E+00	1.00E+00	0.00E+00	1.00E+00
ENSBTAG00000019123	8.21E-01	6.48E-01	2.64E+00	9.35E-06	1.08E+00	1.11E-01	2.41E+00	6.29E-05	6.39E-01	8.00E-01
ENSBTAG00000018625	-2.36E-01	1.00E+00	6.57E-01	5.10E-02	8.88E-01	4.89E-03	2.16E-01	7.38E-01	-1.91E-01	1.00E+00
ENSBTAG00000020209	-4.79E-01	1.00E+00	5.56E-01	6.84E-01	-5.49E-01	6.95E-01	-1.42E+00	1.19E-01	-4.48E-02	1.00E+00

ENSBTAG00000040381	-6.06E-02	1.00E+00	1.06E+00	2.01E-04	9.70E-01	6.86E-04	7.64E-02	1.00E+00	2.14E-01	1.00E+00
ENSBTAG00000001606	0.00E+00	1.00E+00	0.00E+00	1.00E+00	-5.13E-15	1.00E+00	0.00E+00	1.00E+00	0.00E+00	1.00E+00
ENSBTAG00000001614	-5.42E-01	1.67E-01	-6.02E-01	1.96E-02	-1.76E-01	6.86E-01	-2.92E-01	3.68E-01	1.35E-01	1.00E+00
ENSBTAG00000000728	3.26E-01	1.00E+00	-7.64E-02	1.00E+00	-4.30E-01	7.36E-01	5.84E-01	4.97E-01	8.52E-01	6.07E-01
ENSBTAG00000004875	-1.28E-01	1.00E+00	4.05E-01	6.90E-02	1.30E-01	7.74E-01	6.36E-02	1.00E+00	-1.10E-02	1.00E+00
ENSBTAG00000000611	-7.99E-01	1.00E+00	-7.29E-01	9.45E-01	-2.71E+00	1.99E-01	-9.46E-01	8.04E-01	-7.98E-01	1.00E+00
ENSBTAG00000004502	-4.15E-03	1.00E+00	-9.51E-01	1.17E-05	-2.30E-01	4.41E-01	-1.75E-01	6.34E-01	1.13E-01	1.00E+00
ENSBTAG00000004505	2.61E-01	9.97E-01	-4.85E-01	2.66E-01	-2.34E-01	7.20E-01	-4.18E-01	3.59E-01	-2.28E-01	1.00E+00
ENSBTAG00000004509	-1.56E-01	1.00E+00	-5.34E-01	6.60E-02	-2.95E-01	4.17E-01	-3.32E-01	3.40E-01	7.43E-01	6.28E-02
ENSBTAG00000044712	6.32E-01	7.01E-01	-2.11E-01	1.00E+00	1.91E-01	1.00E+00	1.20E-01	1.00E+00	1.30E+00	6.09E-02
ENSBTAG00000020107	-1.83E-01	1.00E+00	-8.57E-01	2.19E-03	-2.76E-01	4.73E-01	-7.67E-01	7.15E-03	-1.51E-01	1.00E+00
ENSBTAG00000020109	-4.38E-02	1.00E+00	-1.74E-01	7.24E-01	3.76E-01	2.01E-01	2.28E-01	5.44E-01	2.08E-02	1.00E+00
ENSBTAG00000026403	3.82E-01	4.54E-01	1.16E+00	1.71E-07	5.75E-01	1.65E-02	6.10E-01	1.16E-02	5.56E-01	1.64E-01
ENSBTAG00000016629	5.17E-01	2.81E-01	1.16E+00	4.65E-06	1.06E+00	2.98E-05	4.34E-01	1.66E-01	4.85E-01	4.24E-01
ENSBTAG00000026400	2.37E-01	1.00E+00	6.44E-01	1.69E-01	2.08E-01	8.83E-01	-7.47E-01	1.05E-01	-2.73E-01	1.00E+00
ENSBTAG00000001078	1.53E+00	2.24E-01	2.67E+00	1.06E-04	2.12E+00	2.98E-03	2.75E+00	6.46E-05	4.04E-01	1.00E+00
ENSBTAG00000033174	-9.38E-01	9.72E-02	-1.16E+00	3.07E-03	-1.41E-01	1.00E+00	-3.78E-01	4.97E-01	1.77E-01	1.00E+00
ENSBTAG00000009199	-1.96E-01	1.00E+00	-1.21E+00	2.33E-02	-1.35E-01	1.00E+00	1.49E-01	1.00E+00	4.73E-01	8.44E-01
ENSBTAG00000009200	1.02E-02	1.00E+00	2.18E-01	6.55E-01	4.24E-01	2.00E-01	3.34E-01	3.79E-01	-1.29E-01	1.00E+00
ENSBTAG00000009201	-4.73E-01	3.39E-01	-1.30E+00	2.16E-07	-1.34E+00	6.54E-08	-1.38E+00	3.72E-08	-3.83E-01	6.01E-01
ENSBTAG00000046339	0.00E+00	1.00E+00	0.00E+00	1.00E+00	-5.13E-15	1.00E+00	0.00E+00	1.00E+00	0.00E+00	1.00E+00
ENSBTAG00000010013	-1.16E-01	1.00E+00	4.24E-01	7.64E-02	4.80E-01	3.81E-02	4.37E-01	7.16E-02	-1.95E-01	8.97E-01
ENSBTAG00000010015	6.78E-02	1.00E+00	-6.20E-01	1.41E-01	-1.37E+00	1.69E-04	-2.10E+00	6.93E-09	-7.17E-01	3.52E-01
ENSBTAG00000018937	-1.11E-02	1.00E+00	5.03E-01	1.86E-01	8.14E-01	1.48E-02	5.84E-01	1.14E-01	-1.47E-01	1.00E+00
ENSBTAG00000018938	-4.14E-01	5.76E-01	-2.43E+00	3.69E-18	-3.47E+00	1.10E-32	-2.58E+00	3.04E-20	1.32E-01	1.00E+00
ENSBTAG00000047316	1.14E+00	5.43E-01	-4.72E-01	7.95E-01	-2.16E+00	1.08E-02	2.59E-01	1.00E+00	2.01E+00	8.69E-02
ENSBTAG00000026394	-5.85E-01	1.70E-01	6.59E-01	1.55E-02	6.50E-01	1.70E-02	2.56E-01	5.03E-01	-6.48E-02	1.00E+00
ENSBTAG00000042669	5.30E-01	1.00E+00	9.78E-01	6.54E-01	1.02E+00	5.93E-01	1.81E+00	1.57E-01	-2.70E+00	6.01E-01
ENSBTAG00000018999	-1.57E-01	1.00E+00	-8.51E-01	4.23E-05	-1.01E+00	7.09E-07	-7.24E-01	7.21E-04	3.93E-02	1.00E+00
ENSBTAG00000006590	-8.46E-02	1.00E+00	-5.82E-01	1.87E-02	-1.81E+00	2.98E-15	-1.32E+00	1.03E-08	1.94E-01	9.38E-01
ENSBTAG00000006593	3.68E-01	4.94E-01	-1.40E-01	8.14E-01	-9.23E-02	9.89E-01	-4.20E-01	1.15E-01	4.92E-01	2.66E-01
ENSBTAG00000006595	5.19E-01	3.99E-01	1.08E+00	1.58E-04	4.82E-01	1.63E-01	-3.30E-01	4.56E-01	4.94E-01	5.08E-01
ENSBTAG00000037566	2.82E-01	9.30E-01	-2.68E-02	1.00E+00	-2.95E-01	5.28E-01	-5.68E-01	1.28E-01	6.10E-01	3.51E-01
ENSBTAG00000016330	6.61E-02	1.00E+00	8.08E-01	4.74E-03	3.84E-01	2.72E-01	-1.95E-01	7.45E-01	-5.89E-01	3.15E-01
ENSBTAG00000016331	-3.83E-01	5.76E-01	-1.40E+00	4.91E-08	-1.84E+00	4.46E-13	-1.52E+00	3.34E-09	5.06E-01	3.65E-01

ENSBTAG00000001731	1.28E-01	1.00E+00	5.83E-01	2.62E-03	7.62E-01	4.66E-05	7.22E-01	1.56E-04	4.03E-02	1.00E+00
ENSBTAG00000001736	4.17E-01	5.75E-01	1.33E+00	5.57E-07	1.51E+00	1.06E-08	1.11E+00	4.81E-05	4.11E-01	6.01E-01
ENSBTAG00000002178	1.76E+00	4.73E-11	1.94E+00	2.54E-14	1.85E+00	3.23E-13	1.39E+00	5.96E-08	4.29E-01	5.24E-01
ENSBTAG00000007890	1.58E+00	2.38E-03	1.97E+00	3.46E-06	2.05E+00	8.80E-07	1.06E+00	2.84E-02	9.99E-01	2.52E-01
ENSBTAG00000007896	-2.34E-01	8.92E-01	7.60E-02	1.00E+00	6.62E-01	7.95E-03	2.69E-01	4.39E-01	4.38E-01	4.34E-01
ENSBTAG00000002188	1.37E+00	5.73E-01	1.47E+00	1.64E-01	-5.39E-01	9.34E-01	3.58E-01	1.00E+00	-3.80E-01	1.00E+00
ENSBTAG00000002189	-3.03E-02	1.00E+00	9.92E-01	6.49E-05	7.84E-01	2.05E-03	6.50E-01	1.54E-02	4.48E-01	4.50E-01
ENSBTAG00000008222	-1.84E-01	1.00E+00	-5.90E-01	7.44E-02	-1.91E-01	7.73E-01	-1.55E-01	8.89E-01	-6.97E-01	1.99E-01
ENSBTAG00000026383	5.49E-03	1.00E+00	1.52E+00	2.97E-01	7.17E-01	8.27E-01	3.25E-01	1.00E+00	8.15E-04	1.00E+00
ENSBTAG00000037010	0.00E+00	1.00E+00	1.98E+00	4.43E-01	-5.13E-15	1.00E+00	0.00E+00	1.00E+00	0.00E+00	1.00E+00
ENSBTAG00000044570	0.00E+00	1.00E+00	0.00E+00	1.00E+00	-5.13E-15	1.00E+00	0.00E+00	1.00E+00	0.00E+00	1.00E+00
ENSBTAG00000007210	5.86E-01	9.16E-01	5.75E-01	5.61E-01	-2.32E-01	1.00E+00	9.77E-01	1.81E-01	-3.17E-02	1.00E+00
ENSBTAG00000028105	0.00E+00	1.00E+00	0.00E+00	1.00E+00	-5.13E-15	1.00E+00	0.00E+00	1.00E+00	0.00E+00	1.00E+00
ENSBTAG00000026381	5.16E-01	1.00E+00	6.01E-01	9.45E-01	-9.17E-01	8.10E-01	1.45E+00	3.29E-01	1.45E+00	7.53E-01
ENSBTAG00000035782	-1.21E-01	1.00E+00	-2.05E-01	6.68E-01	-2.41E-01	5.54E-01	-5.17E-01	9.11E-02	-8.86E-02	1.00E+00
ENSBTAG00000029291	0.00E+00	1.00E+00	0.00E+00	1.00E+00	-5.13E-15	1.00E+00	0.00E+00	1.00E+00	0.00E+00	1.00E+00
ENSBTAG00000004038	-1.72E-01	1.00E+00	-1.73E+00	8.32E-12	-3.23E+00	9.14E-35	-2.73E+00	4.90E-26	2.49E-01	8.58E-01
ENSBTAG00000022530	2.37E-01	8.74E-01	2.03E+00	7.40E-20	1.89E+00	1.60E-17	1.72E+00	1.14E-14	7.80E-02	1.00E+00
ENSBTAG00000001440	1.39E-01	1.00E+00	1.24E+00	3.36E-09	9.86E-01	3.21E-06	6.59E-01	3.50E-03	3.12E-02	1.00E+00
ENSBTAG00000001441	-2.71E-02	1.00E+00	-1.43E-01	7.50E-01	-7.94E-01	1.45E-04	-1.15E+00	2.47E-08	1.14E-01	1.00E+00
ENSBTAG00000005455	2.12E-01	7.97E-01	4.30E-01	2.35E-02	4.29E-01	2.37E-02	2.56E-01	2.60E-01	1.42E-01	9.70E-01
ENSBTAG00000043252	0.00E+00	1.00E+00	0.00E+00	1.00E+00	-5.13E-15	1.00E+00	0.00E+00	1.00E+00	0.00E+00	1.00E+00
ENSBTAG00000013858	-3.30E-02	1.00E+00	-6.73E-01	8.08E-04	1.44E-01	7.03E-01	2.89E-01	2.51E-01	-1.73E-01	9.14E-01
ENSBTAG00000043829	3.89E+00	1.41E-01	4.23E+00	1.32E-02	4.17E+00	1.28E-02	3.39E+00	8.39E-02	3.90E+00	2.01E-01
ENSBTAG00000007887	-4.17E-01	1.00E+00	-1.37E+00	3.32E-02	-5.28E-01	4.79E-01	-6.91E-01	3.25E-01	-6.52E-01	7.85E-01
ENSBTAG00000045680	0.00E+00	1.00E+00	0.00E+00	1.00E+00	-5.13E-15	1.00E+00	0.00E+00	1.00E+00	0.00E+00	1.00E+00
ENSBTAG00000043160	0.00E+00	1.00E+00	0.00E+00	1.00E+00	-5.13E-15	1.00E+00	0.00E+00	1.00E+00	0.00E+00	1.00E+00
ENSBTAG00000010198	-1.39E-01	1.00E+00	2.19E+00	3.32E-05	9.89E-02	1.00E+00	-6.17E-01	4.65E-01	7.03E-02	1.00E+00
ENSBTAG00000043497	0.00E+00	1.00E+00	0.00E+00	1.00E+00	-5.13E-15	1.00E+00	0.00E+00	1.00E+00	0.00E+00	1.00E+00
ENSBTAG00000007177	2.67E-02	1.00E+00	-9.44E-01	1.92E-03	-1.02E+00	7.25E-04	-3.12E-01	4.75E-01	-1.55E-01	1.00E+00
ENSBTAG00000017015	-9.34E-01	8.23E-01	-6.12E-01	7.08E-01	-2.28E+00	3.92E-02	-1.81E+00	9.82E-02	-1.61E+00	5.04E-01
ENSBTAG00000009560	1.15E-01	1.00E+00	5.88E-01	2.00E-03	9.59E-01	8.80E-08	6.20E-01	1.17E-03	-1.67E-01	9.10E-01
ENSBTAG00000016360	1.34E-02	1.00E+00	-2.03E+00	1.44E-04	-3.73E+00	1.52E-11	-5.69E+00	5.37E-20	4.16E-01	9.58E-01
ENSBTAG00000020649	-8.06E-01	2.41E-01	-3.71E-01	5.21E-01	-9.36E-01	2.51E-02	-1.10E+00	8.13E-03	1.84E-02	1.00E+00
ENSBTAG00000046840	2.03E-01	1.00E+00	8.75E-01	3.93E-03	1.10E+00	1.90E-04	8.19E-01	8.65E-03	2.36E-01	9.69E-01

ENSBTAG00000005208	9.69E-02	1.00E+00	1.67E+00	7.37E-09	2.05E+00	9.75E-13	1.71E+00	3.65E-09	3.16E-01	8.00E-01
ENSBTAG00000004386	3.05E+00	1.29E-14	4.94E+00	7.35E-38	4.36E+00	1.07E-30	2.76E+00	5.61E-13	-1.91E-01	1.00E+00
ENSBTAG00000032884	1.92E+00	8.23E-01	3.98E+00	2.55E-02	1.78E+00	4.79E-01	0.00E+00	1.00E+00	0.00E+00	1.00E+00
ENSBTAG00000013051	0.00E+00	1.00E+00	2.79E+00	2.10E-01	-5.13E-15	1.00E+00	0.00E+00	1.00E+00	0.00E+00	1.00E+00
ENSBTAG00000032880	0.00E+00	1.00E+00	0.00E+00	1.00E+00	1.78E+00	4.79E-01	0.00E+00	1.00E+00	0.00E+00	1.00E+00
ENSBTAG00000021493	0.00E+00	1.00E+00	1.98E+00	4.43E-01	-5.13E-15	1.00E+00	0.00E+00	1.00E+00	0.00E+00	1.00E+00
ENSBTAG00000026375	7.06E-02	1.00E+00	6.73E-02	1.00E+00	7.91E-01	1.50E-01	1.38E+00	4.06E-03	-1.32E-01	1.00E+00
ENSBTAG00000013305	-2.35E-01	1.00E+00	1.05E+00	3.50E-01	1.54E+00	8.36E-02	9.30E-01	4.40E-01	1.89E+00	1.81E-01
ENSBTAG00000005622	1.25E+00	9.00E-07	2.45E+00	3.43E-25	2.18E+00	1.26E-20	1.50E+00	2.03E-10	6.65E-02	1.00E+00
ENSBTAG00000026376	1.73E+00	7.79E-10	1.38E+00	2.99E-07	1.03E-01	1.00E+00	3.01E-01	4.64E-01	6.28E-01	2.44E-01
ENSBTAG00000014823	2.55E-01	8.23E-01	9.34E-01	7.42E-05	6.32E-01	1.07E-02	3.12E-01	3.18E-01	4.32E-01	4.34E-01
ENSBTAG00000014826	-8.35E-02	1.00E+00	-7.73E-01	1.66E-05	-3.39E-01	9.87E-02	-2.98E-01	1.74E-01	4.25E-02	1.00E+00
ENSBTAG00000048279	0.00E+00	1.00E+00	0.00E+00	1.00E+00	-5.13E-15	1.00E+00	0.00E+00	1.00E+00	1.91E+00	8.00E-01
ENSBTAG00000014588	5.38E-01	4.94E-02	2.64E+00	3.18E-44	2.48E+00	1.07E-39	1.41E+00	5.00E-14	-5.87E-01	5.43E-02
ENSBTAG00000003577	4.83E-01	7.36E-02	2.53E+00	8.89E-45	2.79E+00	1.70E-53	2.39E+00	1.72E-40	1.07E-01	1.00E+00
ENSBTAG00000007295	0.00E+00	1.00E+00	0.00E+00	1.00E+00	-5.13E-15	1.00E+00	0.00E+00	1.00E+00	0.00E+00	1.00E+00
ENSBTAG00000026371	-7.11E-03	1.00E+00	-2.57E-01	4.96E-01	-9.01E-02	1.00E+00	1.76E-01	7.38E-01	-1.05E-01	1.00E+00
ENSBTAG00000046216	7.83E-01	9.43E-01	-3.90E+00	3.51E-02	-8.53E-01	6.56E-01	-2.14E+00	1.75E-01	2.03E-03	1.00E+00
ENSBTAG00000007142	1.16E-01	1.00E+00	-8.52E-01	1.41E-02	-1.05E+00	1.69E-03	-7.69E-01	3.32E-02	2.53E-01	9.83E-01
ENSBTAG00000046399	5.16E-03	1.00E+00	1.39E+00	5.18E-01	1.51E+00	4.29E-01	1.11E+00	6.73E-01	-1.91E+00	8.00E-01
ENSBTAG00000032643	0.00E+00	1.00E+00	0.00E+00	1.00E+00	-5.13E-15	1.00E+00	0.00E+00	1.00E+00	0.00E+00	1.00E+00
ENSBTAG00000021773	3.11E-01	8.81E-01	1.02E+00	1.32E-03	1.80E+00	2.41E-09	1.41E+00	5.48E-06	1.89E-01	1.00E+00
ENSBTAG00000026365	-9.90E-02	1.00E+00	7.38E-01	1.88E-01	7.00E-01	2.08E-01	7.22E-01	1.99E-01	2.18E-01	1.00E+00
ENSBTAG00000008728	2.80E-01	9.06E-01	3.48E-01	3.96E-01	1.88E-01	7.88E-01	3.62E-01	3.76E-01	4.09E-01	6.67E-01
ENSBTAG00000036371	0.00E+00	1.00E+00	0.00E+00	1.00E+00	-5.13E-15	1.00E+00	0.00E+00	1.00E+00	1.91E+00	8.00E-01
ENSBTAG00000029848	-3.58E+00	2.33E-01	-1.60E+00	4.09E-01	-1.03E+00	6.03E-01	5.69E-01	8.38E-01	9.47E-01	8.33E-01
ENSBTAG00000018097	-2.90E-01	8.14E-01	-1.61E+00	4.42E-11	-1.57E+00	1.02E-10	-1.53E+00	3.82E-10	3.99E-01	5.51E-01
ENSBTAG00000021521	5.31E-02	1.00E+00	4.09E-01	6.17E-02	2.87E-01	2.43E-01	-2.20E-02	1.00E+00	1.01E-01	1.00E+00
ENSBTAG00000021522	-5.56E-01	7.47E-01	1.04E-02	1.00E+00	-9.98E-02	1.00E+00	-1.11E+00	2.32E-02	-6.64E-01	6.01E-01
ENSBTAG00000043173	0.00E+00	1.00E+00	0.00E+00	1.00E+00	-5.13E-15	1.00E+00	0.00E+00	1.00E+00	0.00E+00	1.00E+00
ENSBTAG00000004804	3.85E-01	3.61E-01	1.69E+00	2.02E-17	1.29E+00	9.36E-11	5.35E-01	1.66E-02	-1.36E-01	1.00E+00
ENSBTAG00000030100	0.00E+00	1.00E+00	0.00E+00	1.00E+00	-5.13E-15	1.00E+00	0.00E+00	1.00E+00	0.00E+00	1.00E+00
ENSBTAG00000004803	-2.43E-01	8.23E-01	-9.30E-01	3.96E-05	-1.68E+00	3.10E-14	-1.79E+00	6.67E-16	-2.23E-02	1.00E+00
ENSBTAG00000004801	8.97E-02	1.00E+00	2.01E-02	1.00E+00	1.43E-01	6.88E-01	3.66E-01	1.01E-01	-1.17E-01	1.00E+00
ENSBTAG00000047520	-1.47E-01	1.00E+00	-9.05E-01	7.30E-03	-2.04E+00	1.79E-10	-2.21E+00	7.16E-12	-1.94E-01	1.00E+00

ENSBTAG00000011692	-9.47E-01	8.23E-01	-4.13E-02	1.00E+00	-3.19E-01	9.84E-01	1.09E+00	1.61E-01	2.61E-01	1.00E+00
ENSBTAG00000020387	-1.75E-01	1.00E+00	-5.33E-01	1.29E-01	-3.74E-01	3.52E-01	2.91E-02	1.00E+00	3.59E-01	7.93E-01
ENSBTAG00000045216	-1.91E+00	8.23E-01	7.78E-02	1.00E+00	-1.30E-01	1.00E+00	-1.91E+00	4.39E-01	7.05E-03	1.00E+00
ENSBTAG00000029976	1.92E+00	8.23E-01	0.00E+00	1.00E+00	-5.13E-15	1.00E+00	2.53E+00	2.71E-01	3.21E+00	4.34E-01
ENSBTAG00000015986	-1.23E-01	1.00E+00	-3.66E-01	3.51E-01	-7.34E-01	1.91E-02	-6.76E-01	3.78E-02	-2.31E-01	9.77E-01
ENSBTAG00000015988	1.03E-01	1.00E+00	-7.55E-01	3.82E-02	-1.77E+00	1.05E-07	-2.32E-01	7.42E-01	4.75E-01	6.29E-01
ENSBTAG00000032640	6.45E-02	1.00E+00	3.94E-01	1.02E-01	6.35E-01	3.12E-03	1.22E-01	8.46E-01	-1.69E-01	9.70E-01
ENSBTAG00000021090	-3.31E-02	1.00E+00	-2.07E-02	1.00E+00	-9.76E-01	6.34E-05	-1.17E+00	1.82E-06	3.88E-01	5.47E-01
ENSBTAG00000015191	3.55E-01	5.76E-01	7.56E-01	1.83E-03	5.64E-01	2.68E-02	-4.22E-02	1.00E+00	3.60E-01	6.01E-01
ENSBTAG00000017759	3.99E-01	6.01E-01	8.34E-01	3.34E-03	9.13E-01	1.12E-03	9.46E-01	8.66E-04	8.38E-01	4.25E-02
ENSBTAG00000005523	6.88E-02	1.00E+00	-1.01E+00	2.54E-02	-1.71E+00	7.03E-05	-5.67E-01	2.74E-01	5.33E-01	6.85E-01
ENSBTAG00000044722	0.00E+00	1.00E+00	0.00E+00	1.00E+00	-5.13E-15	1.00E+00	0.00E+00	1.00E+00	0.00E+00	1.00E+00
ENSBTAG00000020733	1.13E+00	3.43E-05	1.92E+00	1.58E-16	1.84E+00	1.70E-15	1.28E+00	1.27E-07	-3.30E-01	7.87E-01
ENSBTAG00000020734	-4.29E-01	6.76E-01	-1.12E+00	5.21E-04	-8.37E-01	1.21E-02	-5.16E-01	1.81E-01	2.53E-01	9.68E-01
ENSBTAG00000020735	6.66E-01	3.33E-01	7.03E-01	7.23E-02	2.08E-01	8.20E-01	5.70E-01	1.81E-01	4.56E-01	7.23E-01
ENSBTAG00000044528	0.00E+00	1.00E+00	0.00E+00	1.00E+00	-5.13E-15	1.00E+00	0.00E+00	1.00E+00	0.00E+00	1.00E+00
ENSBTAG00000018250	1.33E-02	1.00E+00	8.82E-01	8.52E-01	1.53E+00	4.28E-01	6.27E-01	9.94E-01	-1.91E+00	8.00E-01
ENSBTAG00000014229	2.89E-02	1.00E+00	7.30E-01	5.46E-01	-1.59E-01	1.00E+00	8.29E-01	4.48E-01	4.63E-01	1.00E+00
ENSBTAG00000032503	8.91E-02	1.00E+00	6.08E-01	2.60E-01	1.35E-01	1.00E+00	4.00E-01	5.49E-01	-3.97E-01	9.41E-01
ENSBTAG00000044481	4.84E+00	9.28E-03	3.68E+00	5.07E-02	1.78E+00	4.79E-01	0.00E+00	1.00E+00	0.00E+00	1.00E+00
ENSBTAG00000021242	-7.80E-02	1.00E+00	-9.32E-02	1.00E+00	-2.19E-01	6.62E-01	-4.01E-01	2.71E-01	-1.46E-01	1.00E+00
ENSBTAG00000016505	3.15E-01	8.39E-01	2.69E-01	5.81E-01	-2.79E-01	5.54E-01	1.94E-01	7.98E-01	3.92E-01	7.42E-01
ENSBTAG00000002097	-2.56E-01	1.00E+00	-8.77E-01	6.62E-01	-5.43E-01	9.08E-01	2.36E-01	1.00E+00	-9.37E-01	9.37E-01
ENSBTAG00000002101	3.60E-02	1.00E+00	6.10E-01	1.04E-01	1.54E+00	1.83E-06	1.83E+00	1.48E-08	-9.60E-02	1.00E+00
ENSBTAG00000003960	-4.20E-02	1.00E+00	8.39E-01	3.80E-02	1.32E+00	3.75E-04	1.10E+00	4.45E-03	1.34E-01	1.00E+00
ENSBTAG00000019412	-2.73E-03	1.00E+00	-6.48E-01	5.82E-03	-2.29E-01	4.79E-01	-1.16E-01	9.03E-01	1.09E-01	1.00E+00
ENSBTAG00000019593	-4.32E-01	5.91E-01	2.69E-02	1.00E+00	8.95E-01	2.72E-03	7.76E-01	1.28E-02	-3.75E-01	7.54E-01
ENSBTAG00000044092	-4.87E-01	9.40E-01	1.19E-01	1.00E+00	-9.27E-03	1.00E+00	-8.50E-01	2.13E-01	-1.12E-01	1.00E+00
ENSBTAG00000019596	2.95E-01	1.00E+00	2.24E+00	1.07E-03	3.14E+00	4.26E-06	2.74E+00	7.24E-05	3.25E-01	1.00E+00
ENSBTAG00000046240	-1.66E+00	5.90E-01	-4.38E+00	1.11E-02	2.59E-01	1.00E+00	-4.38E+00	6.61E-03	-2.45E+00	3.84E-01
ENSBTAG00000022535	3.90E+00	1.40E-01	2.79E+00	2.10E-01	3.05E+00	1.36E-01	3.40E+00	8.36E-02	3.59E+00	3.09E-01
ENSBTAG00000006263	8.26E-01	1.00E+00	5.30E+00	2.54E-06	4.76E+00	3.75E-05	4.54E+00	1.28E-04	6.12E+00	9.78E-07
ENSBTAG00000006264	6.62E-03	1.00E+00	6.91E-02	1.00E+00	-1.32E-01	1.00E+00	-1.91E+00	4.39E-01	3.81E-04	1.00E+00
ENSBTAG00000016434	3.12E-01	1.00E+00	-3.03E-01	1.00E+00	-5.71E-01	7.67E-01	-1.13E+00	3.85E-01	7.91E-01	8.00E-01
ENSBTAG00000037449	1.29E-01	1.00E+00	-7.25E-01	9.93E-02	-1.75E+00	5.91E-06	-2.62E+00	2.30E-11	-1.91E-01	1.00E+00

ENSBTAG00000019423	-2.88E-01	6.95E-01	-1.03E+00	1.34E-06	-2.18E+00	5.65E-25	-3.08E+00	1.00E-45	4.05E-02	1.00E+00
ENSBTAG00000001417	-1.21E-01	1.00E+00	-6.24E-01	1.38E-02	-1.46E+00	3.88E-10	-1.90E+00	6.53E-16	4.88E-02	1.00E+00
ENSBTAG00000046098	-4.85E-01	9.29E-01	1.34E-01	1.00E+00	-1.12E-02	1.00E+00	-1.53E+00	1.42E-02	-2.59E-01	1.00E+00
ENSBTAG00000047673	-2.25E-01	9.84E-01	1.08E+00	4.04E-05	1.61E+00	1.72E-10	1.51E+00	3.76E-09	-8.44E-02	1.00E+00
ENSBTAG00000006447	-6.45E-02	1.00E+00	-8.82E-01	1.47E-04	-1.98E+00	2.11E-18	-2.63E+00	3.19E-30	3.94E-01	4.95E-01
ENSBTAG00000006448	-5.70E-01	5.76E-01	-6.03E-01	1.84E-01	4.65E-01	3.07E-01	1.18E+00	8.88E-04	-4.61E-01	7.71E-01
ENSBTAG00000006449	4.84E-02	1.00E+00	-7.47E-02	1.00E+00	5.21E-01	6.49E-02	7.78E-01	2.96E-03	1.00E-02	1.00E+00
ENSBTAG00000032260	2.19E-01	1.00E+00	1.03E+00	1.55E-04	7.71E-01	6.44E-03	7.38E-01	1.13E-02	-1.04E-01	1.00E+00
ENSBTAG00000018559	1.80E-01	1.00E+00	8.44E-01	2.54E-03	6.05E-01	4.14E-02	3.30E-01	3.88E-01	-7.20E-02	1.00E+00
ENSBTAG00000036743	0.00E+00	1.00E+00	0.00E+00	1.00E+00	-5.13E-15	1.00E+00	0.00E+00	1.00E+00	0.00E+00	1.00E+00
ENSBTAG00000018560	1.72E-01	1.00E+00	7.87E-01	1.53E-03	3.00E-01	3.61E-01	-5.04E-01	7.82E-02	3.95E-01	5.52E-01
ENSBTAG00000018562	2.51E-01	9.72E-01	-5.78E-02	1.00E+00	-2.48E-01	6.25E-01	2.07E-01	7.44E-01	-1.94E-01	1.00E+00
ENSBTAG00000011504	-4.13E-01	1.00E+00	-7.15E-01	6.75E-01	-2.34E+00	2.77E-02	-2.88E+00	7.56E-03	1.28E-01	1.00E+00
ENSBTAG00000009837	-5.05E-01	3.84E-01	-6.82E-01	2.37E-02	-5.06E-01	1.19E-01	-3.63E-01	3.34E-01	-2.66E-02	1.00E+00
ENSBTAG00000009842	-1.64E-01	1.00E+00	-1.14E+00	5.61E-05	-9.79E-01	5.36E-04	-3.97E-01	2.62E-01	-2.15E-01	9.85E-01
ENSBTAG00000006717	2.37E-01	1.00E+00	-1.94E+00	3.02E-02	-2.66E+00	2.62E-03	-9.86E-01	3.32E-01	-4.26E-01	1.00E+00
ENSBTAG00000035915	2.36E-01	1.00E+00	9.28E-02	1.00E+00	3.99E-02	1.00E+00	2.21E-01	1.00E+00	4.87E-01	9.84E-01
ENSBTAG00000021651	-1.59E-01	1.00E+00	-7.66E-02	1.00E+00	-2.11E-02	1.00E+00	1.22E-01	9.02E-01	-1.09E-01	1.00E+00
ENSBTAG00000021652	-1.94E-01	1.00E+00	-3.76E-01	4.46E-01	-6.37E-01	1.12E-01	-4.63E-01	3.08E-01	-1.03E+00	6.45E-02
ENSBTAG00000008188	6.06E-01	4.50E-01	1.14E+00	1.48E-03	1.07E+00	2.97E-03	5.43E-01	2.21E-01	4.06E-01	8.00E-01
ENSBTAG00000016244	2.67E-01	1.00E+00	5.49E-01	8.34E-01	7.29E-02	1.00E+00	2.55E-01	1.00E+00	8.19E-01	8.87E-01
ENSBTAG00000042455	0.00E+00	1.00E+00	0.00E+00	1.00E+00	-5.13E-15	1.00E+00	0.00E+00	1.00E+00	0.00E+00	1.00E+00
ENSBTAG00000013052	-1.56E+00	1.11E-01	-1.49E+00	3.15E-02	-9.60E-01	1.58E-01	-2.33E+00	9.26E-04	-7.88E-01	6.80E-01
ENSBTAG00000017662	-2.67E-01	8.34E-01	-1.49E+00	4.82E-09	-6.96E-01	9.96E-03	2.20E-01	6.09E-01	6.73E-01	1.15E-01
ENSBTAG00000018044	8.40E-02	1.00E+00	1.43E+00	1.84E-11	1.73E+00	2.47E-16	1.18E+00	4.73E-08	5.18E-02	1.00E+00
ENSBTAG00000018050	-4.20E-01	7.58E-01	8.29E-01	1.90E-02	2.94E-02	1.00E+00	-3.18E-01	5.34E-01	-4.01E-01	7.92E-01
ENSBTAG00000032373	-1.55E-01	1.00E+00	-1.84E+00	2.62E-03	-1.22E+00	3.52E-02	-2.97E+00	5.59E-06	2.34E-01	1.00E+00
ENSBTAG00000018868	9.46E-01	2.77E-01	2.47E+00	2.35E-07	2.66E+00	2.69E-08	1.85E+00	1.43E-04	2.61E-01	1.00E+00
ENSBTAG00000018869	0.00E+00	1.00E+00	0.00E+00	1.00E+00	-5.13E-15	1.00E+00	0.00E+00	1.00E+00	0.00E+00	1.00E+00
ENSBTAG00000018870	-2.86E-01	1.00E+00	3.60E+00	4.88E-06	3.45E+00	1.12E-05	2.33E+00	4.89E-03	3.14E-01	1.00E+00
ENSBTAG00000039989	0.00E+00	1.00E+00	0.00E+00	1.00E+00	-5.13E-15	1.00E+00	0.00E+00	1.00E+00	0.00E+00	1.00E+00
ENSBTAG00000018195	0.00E+00	1.00E+00	0.00E+00	1.00E+00	-5.13E-15	1.00E+00	2.53E+00	2.71E-01	0.00E+00	1.00E+00
ENSBTAG00000017391	-6.07E-01	1.00E+00	-3.53E-02	1.00E+00	-6.21E-02	1.00E+00	1.14E+00	2.33E-01	-4.50E-01	1.00E+00
ENSBTAG00000011744	4.60E-01	5.90E-01	1.34E+00	1.15E-05	2.02E+00	8.30E-12	1.68E+00	2.49E-08	2.44E-01	9.89E-01
ENSBTAG00000043717	0.00E+00	1.00E+00	0.00E+00	1.00E+00	-5.13E-15	1.00E+00	0.00E+00	1.00E+00	0.00E+00	1.00E+00

ENSBTAG00000010163	-2.70E+00	5.76E-01	-7.16E-01	9.45E-01	7.18E-01	8.28E-01	1.23E+00	4.57E-01	1.20E+00	8.22E-01
ENSBTAG00000012290	2.00E+00	5.76E-01	2.07E+00	2.00E-01	1.15E+00	6.44E-01	2.58E+00	5.67E-02	1.33E+00	8.96E-01
ENSBTAG00000032223	-1.06E-02	1.00E+00	-7.91E-01	8.00E-03	-2.65E-01	5.29E-01	-1.09E-01	1.00E+00	4.13E-02	1.00E+00
ENSBTAG00000002352	-2.33E-01	8.23E-01	-3.58E-01	1.74E-01	2.60E-01	3.81E-01	2.64E-01	3.83E-01	-1.58E-01	1.00E+00
ENSBTAG00000045593	2.44E-02	1.00E+00	2.57E+00	2.52E-10	2.41E+00	2.50E-09	2.51E+00	7.67E-10	-1.98E-01	1.00E+00
ENSBTAG00000046033	8.71E-02	1.00E+00	1.16E+00	1.03E-05	1.01E+00	1.28E-04	9.98E-01	2.12E-04	-7.60E-02	1.00E+00
ENSBTAG00000006391	-1.24E-01	1.00E+00	2.37E-01	4.71E-01	-3.26E-02	1.00E+00	-2.70E-01	4.14E-01	-2.56E-01	8.00E-01
ENSBTAG00000006405	-5.48E-01	1.84E-01	-1.58E+00	1.37E-10	-1.42E+00	5.89E-09	-1.15E+00	3.35E-06	9.84E-02	1.00E+00
ENSBTAG00000006410	6.93E-02	1.00E+00	1.00E-01	9.86E-01	1.40E-01	8.32E-01	2.26E-01	5.39E-01	5.14E-02	1.00E+00
ENSBTAG00000014453	-2.99E-02	1.00E+00	7.81E-01	1.52E-04	-2.69E-01	3.29E-01	-8.60E-01	6.52E-05	1.22E-01	1.00E+00
ENSBTAG00000014456	2.96E-01	1.00E+00	4.12E-02	1.00E+00	7.01E-01	4.19E-01	-4.89E-01	7.17E-01	3.63E-01	1.00E+00
ENSBTAG00000012541	-5.19E-01	6.61E-01	-1.21E+00	1.94E-03	-2.64E+00	4.23E-12	-2.51E+00	5.84E-11	3.22E-02	1.00E+00
ENSBTAG00000020921	4.13E-02	1.00E+00	-6.48E-01	3.00E-02	-6.38E-01	3.18E-02	-4.97E-01	1.25E-01	-7.40E-02	1.00E+00
ENSBTAG00000001404	8.26E-01	9.33E-01	7.45E-01	6.51E-01	6.47E-01	7.23E-01	7.31E-01	6.44E-01	-1.16E+00	8.55E-01
ENSBTAG00000009441	1.34E-01	1.00E+00	1.44E+00	1.68E-11	1.33E+00	5.03E-10	2.31E-01	4.82E-01	-1.12E-01	1.00E+00
ENSBTAG00000017999	2.16E-02	1.00E+00	-9.88E-01	6.82E-05	-9.10E-01	2.59E-04	-9.70E-01	1.14E-04	4.00E-01	5.49E-01
ENSBTAG00000003467	4.16E-01	1.00E+00	-8.57E-01	4.58E-01	-6.70E-02	1.00E+00	3.07E-01	9.47E-01	-9.34E-04	1.00E+00
ENSBTAG00000020043	8.17E-02	1.00E+00	4.65E-01	2.53E-02	6.44E-01	1.01E-03	9.94E-02	9.05E-01	4.04E-02	1.00E+00
ENSBTAG00000046132	-5.31E-01	1.00E+00	7.46E-01	7.61E-01	-1.46E+00	4.79E-01	4.50E-01	9.94E-01	1.75E+00	4.34E-01
ENSBTAG00000013169	8.70E-02	1.00E+00	4.36E-02	1.00E+00	-4.11E-01	7.82E-02	-6.70E-01	1.91E-03	-2.86E-02	1.00E+00
ENSBTAG00000020184	-1.16E-01	1.00E+00	-4.24E-01	4.22E-01	-1.67E+00	8.62E-06	-2.08E+00	3.74E-08	2.56E-03	1.00E+00
ENSBTAG00000011162	-7.45E-01	1.00E+00	-1.68E+00	2.97E-01	-5.91E-01	9.01E-01	-2.11E-01	1.00E+00	1.40E-01	1.00E+00
ENSBTAG00000036807	0.00E+00	1.00E+00	0.00E+00	1.00E+00	-5.13E-15	1.00E+00	0.00E+00	1.00E+00	0.00E+00	1.00E+00
ENSBTAG00000046843	0.00E+00	1.00E+00	0.00E+00	1.00E+00	-5.13E-15	1.00E+00	0.00E+00	1.00E+00	0.00E+00	1.00E+00
ENSBTAG00000020051	0.00E+00	1.00E+00	0.00E+00	1.00E+00	-5.13E-15	1.00E+00	0.00E+00	1.00E+00	1.91E+00	8.00E-01
ENSBTAG00000002176	-6.07E-02	1.00E+00	-4.58E-01	4.58E-02	-1.02E+00	6.76E-07	-1.07E+00	2.14E-07	-5.13E-01	1.67E-01
ENSBTAG00000019530	1.55E-02	1.00E+00	-1.89E-01	7.32E-01	1.83E-01	7.31E-01	-3.82E-02	1.00E+00	5.74E-02	1.00E+00
ENSBTAG00000001602	1.57E+00	9.12E-07	2.93E+00	1.63E-22	2.07E+00	1.97E-12	1.42E+00	2.02E-06	4.79E-01	5.58E-01
ENSBTAG00000019567	1.04E+00	1.45E-01	9.76E-01	4.68E-02	-7.82E-02	1.00E+00	-7.03E-01	2.39E-01	6.93E-03	1.00E+00
ENSBTAG00000006126	-6.64E-02	1.00E+00	-6.69E-01	3.85E-03	-1.36E-01	8.08E-01	-6.06E-03	1.00E+00	1.63E-01	1.00E+00
ENSBTAG00000006129	-7.75E-03	1.00E+00	-4.84E-01	1.08E-01	2.40E-01	5.36E-01	-2.34E-02	1.00E+00	1.91E-01	1.00E+00
ENSBTAG00000004607	2.91E-01	1.00E+00	7.88E-01	3.41E-01	-6.09E-01	5.79E-01	1.09E+00	1.19E-01	-5.70E-01	9.62E-01
ENSBTAG00000010309	-2.55E-01	6.87E-01	-4.96E-01	1.37E-02	-7.30E-01	1.24E-04	-5.08E-01	1.26E-02	2.03E-02	1.00E+00
ENSBTAG00000032140	-9.56E-01	7.22E-02	-3.22E+00	6.95E-16	-4.81E+00	2.02E-27	-4.59E+00	5.03E-26	-5.05E-01	6.56E-01
ENSBTAG00000032136	-5.03E-01	5.76E-01	-1.44E+00	5.13E-05	-9.77E-01	4.86E-03	-3.87E-01	3.90E-01	3.64E-01	8.00E-01

ENSBTAG00000021249	1.40E-01	1.00E+00	5.49E-01	7.44E-02	-3.13E-01	4.28E-01	-6.93E-01	2.66E-02	-1.68E-01	1.00E+00
ENSBTAG00000014653	-9.00E-02	1.00E+00	2.05E-01	4.43E-01	5.37E-01	5.75E-03	5.67E-01	3.85E-03	3.12E-01	5.21E-01
ENSBTAG00000014650	-4.92E-01	2.51E-01	-5.36E-01	4.19E-02	5.72E-01	2.06E-02	3.97E-01	1.55E-01	4.35E-01	4.26E-01
ENSBTAG00000032122	1.26E-01	1.00E+00	9.32E-02	1.00E+00	-3.67E-01	7.44E-01	-7.01E-01	3.38E-01	-4.35E-01	9.69E-01
ENSBTAG00000006542	1.11E-01	1.00E+00	1.75E-01	7.18E-01	7.16E-01	3.82E-03	6.35E-01	1.38E-02	3.65E-01	6.01E-01
ENSBTAG00000006541	-5.75E-01	8.14E-01	2.28E+00	6.20E-07	1.05E+00	3.63E-02	-3.75E-01	6.71E-01	-1.52E-01	1.00E+00
ENSBTAG00000019219	1.50E-01	1.00E+00	1.05E+00	1.41E-07	8.68E-01	1.75E-05	3.41E-01	1.70E-01	1.19E-01	1.00E+00
ENSBTAG00000019216	-5.70E-02	1.00E+00	1.51E-01	8.48E-01	2.26E-01	5.90E-01	3.65E-01	2.78E-01	-1.08E-01	1.00E+00
ENSBTAG00000047397	0.00E+00	1.00E+00	0.00E+00	1.00E+00	-5.13E-15	1.00E+00	0.00E+00	1.00E+00	0.00E+00	1.00E+00
ENSBTAG00000032087	-3.51E-02	1.00E+00	7.62E-01	5.27E-05	8.18E-01	1.18E-05	4.15E-01	5.07E-02	2.52E-01	7.08E-01
ENSBTAG00000006543	1.62E-01	9.54E-01	1.07E+00	3.23E-09	1.35E+00	3.42E-14	1.33E+00	1.30E-13	-2.90E-02	1.00E+00
ENSBTAG00000018000	9.79E-02	1.00E+00	-3.14E-01	2.68E-01	-6.94E-01	2.63E-03	-6.07E-01	1.15E-02	3.78E-02	1.00E+00
ENSBTAG00000018011	-1.27E-01	1.00E+00	-2.87E-01	4.43E-01	-2.83E-01	4.35E-01	-3.90E-01	2.33E-01	-7.31E-02	1.00E+00
ENSBTAG00000018015	2.76E+00	2.47E-14	2.52E+00	7.18E-13	7.28E-01	9.35E-02	5.17E-01	3.08E-01	5.10E-01	7.24E-01
ENSBTAG00000018016	8.19E-01	1.01E-01	1.25E+00	1.84E-04	2.40E-01	7.05E-01	-1.34E-01	9.96E-01	-1.47E-01	1.00E+00
ENSBTAG00000016883	7.94E-01	2.16E-03	1.13E+00	8.59E-08	4.21E-01	9.05E-02	2.78E-01	3.51E-01	1.56E-01	1.00E+00
ENSBTAG00000048253	0.00E+00	1.00E+00	0.00E+00	1.00E+00	-5.13E-15	1.00E+00	0.00E+00	1.00E+00	0.00E+00	1.00E+00
ENSBTAG00000008635	2.22E-01	8.98E-01	-5.01E-02	1.00E+00	-7.69E-01	1.15E-03	-1.03E+00	9.57E-06	-7.83E-02	1.00E+00
ENSBTAG00000008633	4.13E-01	4.65E-01	3.93E-01	1.87E-01	-6.58E-01	1.15E-02	-1.04E+00	3.01E-05	8.09E-01	2.15E-02
ENSBTAG00000008632	2.31E-01	9.48E-01	5.56E-02	1.00E+00	-5.64E-01	5.41E-02	-6.12E-01	3.76E-02	1.86E-01	1.00E+00
ENSBTAG00000008631	2.59E-01	8.39E-01	-2.84E-01	4.28E-01	8.92E-02	1.00E+00	1.01E-01	1.00E+00	-1.64E-01	1.00E+00
ENSBTAG00000016156	-9.50E-02	1.00E+00	-4.08E-01	3.09E-01	2.70E-01	5.78E-01	7.54E-01	2.39E-02	-9.98E-02	1.00E+00
ENSBTAG00000016155	-2.37E-01	1.00E+00	4.81E-01	2.99E-01	7.09E-01	7.36E-02	-2.09E-02	1.00E+00	-2.46E-01	1.00E+00
ENSBTAG00000009047	-6.02E-01	5.02E-02	-1.57E+00	4.20E-13	-9.98E-01	3.93E-06	-7.83E-01	4.77E-04	2.85E-01	7.21E-01
ENSBTAG00000009046	-6.96E-01	7.75E-01	-2.37E-01	9.81E-01	7.10E-01	3.11E-01	7.33E-01	3.02E-01	-9.55E-02	1.00E+00
ENSBTAG00000012928	8.70E-02	1.00E+00	7.29E-01	1.18E-04	1.11E+00	1.32E-09	9.13E-01	1.07E-06	1.52E-01	9.69E-01
ENSBTAG00000012927	2.42E-01	9.09E-01	8.14E-01	2.07E-03	1.59E+00	2.89E-10	1.83E+00	4.75E-13	-2.28E-01	9.10E-01
ENSBTAG00000005033	5.45E-01	1.00E+00	1.03E+00	4.43E-01	3.64E-01	1.00E+00	3.43E-01	1.00E+00	9.35E-01	8.38E-01
ENSBTAG00000005031	-1.05E+00	6.10E-01	6.87E-01	4.43E-01	-3.80E-01	8.46E-01	-1.39E+00	1.09E-01	-3.38E-01	1.00E+00
ENSBTAG00000005030	-1.20E-02	1.00E+00	1.20E+00	1.62E-09	1.18E+00	2.48E-09	5.57E-01	1.12E-02	-6.58E-02	1.00E+00
ENSBTAG00000005028	-4.38E-01	5.53E-01	-8.81E-01	2.56E-03	-4.21E-01	2.18E-01	-5.91E-02	1.00E+00	-1.30E-01	1.00E+00
ENSBTAG00000017422	-1.04E-01	1.00E+00	4.35E-01	6.06E-02	6.75E-01	1.51E-03	2.38E-01	4.33E-01	-1.77E-02	1.00E+00
ENSBTAG00000021785	-1.57E-01	1.00E+00	-3.38E-01	2.25E-01	-5.35E-01	2.75E-02	-4.96E-01	4.95E-02	1.14E-01	1.00E+00
ENSBTAG00000045841	0.00E+00	1.00E+00	0.00E+00	1.00E+00	-5.13E-15	1.00E+00	0.00E+00	1.00E+00	0.00E+00	1.00E+00
ENSBTAG00000015982	3.62E-01	5.40E-01	1.89E+00	1.30E-18	1.75E+00	2.04E-16	6.21E-01	9.94E-03	1.22E-01	1.00E+00

ENSBTAG00000026319	0.00E+00	1.00E+00	0.00E+00	1.00E+00	-5.13E-15	1.00E+00	1.77E+00	4.97E-01	0.00E+00	1.00E+00
ENSBTAG00000007955	-1.12E+00	8.23E-01	-2.35E+00	1.01E-01	-1.28E+00	3.41E-01	-1.96E-01	1.00E+00	-1.63E+00	6.07E-01
ENSBTAG00000007954	-2.24E-01	8.23E-01	-1.29E-01	7.99E-01	8.54E-01	2.31E-05	1.07E+00	8.60E-08	-1.71E-01	9.45E-01
ENSBTAG00000007952	8.91E-01	8.83E-04	1.72E+00	1.49E-14	1.95E+00	2.91E-18	2.08E+00	2.60E-20	3.43E-01	6.01E-01
ENSBTAG00000045147	0.00E+00	1.00E+00	0.00E+00	1.00E+00	-5.13E-15	1.00E+00	0.00E+00	1.00E+00	0.00E+00	1.00E+00
ENSBTAG00000010904	3.44E-01	4.86E-01	8.92E-01	1.35E-05	8.70E-01	2.01E-05	6.32E-01	3.55E-03	6.52E-02	1.00E+00
ENSBTAG00000046752	0.00E+00	1.00E+00	0.00E+00	1.00E+00	-5.13E-15	1.00E+00	0.00E+00	1.00E+00	0.00E+00	1.00E+00
ENSBTAG00000044880	-2.21E-02	1.00E+00	1.81E-01	1.00E+00	1.45E+00	1.77E-03	2.57E-01	9.03E-01	-2.83E-01	1.00E+00
ENSBTAG00000013669	1.83E-01	1.00E+00	4.92E-02	1.00E+00	9.94E-01	7.65E-03	4.69E-02	1.00E+00	2.20E-01	1.00E+00
ENSBTAG00000026325	-1.18E+00	8.65E-01	-1.90E+00	2.54E-01	-4.76E-01	9.46E-01	-2.13E+00	1.76E-01	-2.80E-01	1.00E+00
ENSBTAG00000042494	1.92E+00	8.23E-01	1.98E+00	4.43E-01	2.55E+00	2.55E-01	0.00E+00	1.00E+00	0.00E+00	1.00E+00
ENSBTAG00000047448	1.27E-01	1.00E+00	1.39E-01	8.88E-01	-4.14E-01	2.02E-01	-7.48E-01	1.01E-02	-2.36E-01	9.13E-01
ENSBTAG00000018082	-2.91E-01	8.85E-01	-1.50E+00	4.85E-07	-1.94E+00	5.87E-11	-1.87E+00	3.57E-10	4.05E-02	1.00E+00
ENSBTAG00000026326	-1.41E-01	1.00E+00	-1.42E+00	7.80E-06	-1.04E+00	1.01E-03	-8.22E-01	1.34E-02	-4.19E-01	6.83E-01
ENSBTAG00000042304	0.00E+00	1.00E+00	0.00E+00	1.00E+00	2.55E+00	2.55E-01	0.00E+00	1.00E+00	0.00E+00	1.00E+00
ENSBTAG00000015486	-2.21E-01	9.28E-01	-3.73E-01	2.04E-01	-1.40E-01	8.31E-01	3.64E-02	1.00E+00	-2.11E-01	9.13E-01
ENSBTAG00000046855	-1.62E-01	1.00E+00	-3.97E-01	1.29E-01	-1.73E-01	6.61E-01	-8.73E-02	1.00E+00	-3.82E-01	5.01E-01
ENSBTAG00000031852	2.17E-01	8.94E-01	5.85E-01	1.37E-02	8.03E-01	3.51E-04	7.21E-01	1.87E-03	7.63E-02	1.00E+00
ENSBTAG00000021218	-1.35E+00	5.08E-01	1.19E+00	2.18E-01	-2.32E+00	1.25E-02	-7.10E-01	5.91E-01	-3.76E-01	1.00E+00
ENSBTAG00000021219	5.69E-02	1.00E+00	-7.41E-01	2.68E-02	-9.15E-01	4.22E-03	-1.42E+00	6.72E-06	-8.44E-01	9.57E-02
ENSBTAG00000031806	-9.22E-01	1.56E-03	-4.09E-01	1.54E-01	6.95E-01	3.93E-03	4.92E-01	6.48E-02	-1.15E-01	1.00E+00
ENSBTAG00000021222	-3.07E-01	8.23E-01	-3.85E-01	2.53E-01	-5.20E-01	8.75E-02	1.29E-01	9.32E-01	-5.04E-01	4.23E-01
ENSBTAG00000039962	-7.54E-01	2.42E-02	-1.49E+00	3.66E-09	-1.57E+00	2.88E-10	-1.13E+00	7.38E-06	-3.18E-01	7.48E-01
ENSBTAG00000010851	-1.81E-01	9.74E-01	-1.45E+00	4.45E-12	-1.03E+00	1.18E-06	-7.74E-01	4.44E-04	1.13E-01	1.00E+00
ENSBTAG00000007103	8.70E-02	1.00E+00	2.96E-01	4.12E-01	2.59E-01	4.81E-01	-3.24E-02	1.00E+00	2.19E-02	1.00E+00
ENSBTAG00000043974	-7.54E-01	3.13E-03	-1.13E+00	7.69E-08	-1.13E+00	4.80E-08	-9.04E-01	2.00E-05	-3.57E-01	5.01E-01
ENSBTAG00000019357	-4.98E-01	2.26E-01	-7.97E-01	1.04E-03	-5.27E-01	3.83E-02	-4.78E-01	7.30E-02	8.18E-03	1.00E+00
ENSBTAG00000037984	-3.59E+00	2.32E-01	-3.59E+00	6.66E-02	-1.81E+00	2.94E-01	-5.61E-01	9.20E-01	-3.59E+00	3.09E-01
ENSBTAG00000018094	-2.55E-01	9.66E-01	2.73E-01	5.40E-01	4.50E-01	1.98E-01	3.13E-01	4.58E-01	5.18E-01	4.45E-01
ENSBTAG00000009099	-3.51E-01	5.91E-01	-3.64E-01	2.16E-01	-1.40E-02	1.00E+00	-2.47E-02	1.00E+00	-9.84E-02	1.00E+00
ENSBTAG00000031789	2.97E-01	6.35E-01	9.95E-01	1.36E-06	9.24E-01	7.39E-06	5.50E-01	1.49E-02	8.72E-03	1.00E+00
ENSBTAG00000013917	-1.44E-01	1.00E+00	5.12E-01	3.96E-02	1.16E+00	2.02E-07	8.12E-01	5.03E-04	3.35E-01	6.09E-01
ENSBTAG00000042276	0.00E+00	1.00E+00	0.00E+00	1.00E+00	-5.13E-15	1.00E+00	0.00E+00	1.00E+00	0.00E+00	1.00E+00
ENSBTAG00000043199	0.00E+00	1.00E+00	2.79E+00	2.10E-01	3.72E+00	4.00E-02	1.77E+00	4.97E-01	3.21E+00	4.34E-01
ENSBTAG00000003417	9.61E-02	1.00E+00	4.91E-01	6.33E-02	7.83E-01	1.13E-03	5.17E-01	5.21E-02	1.58E-01	1.00E+00

ENSBTAG00000031765	1.77E-01	1.00E+00	1.72E+00	1.17E-07	7.76E-01	3.74E-02	6.27E-01	1.24E-01	9.54E-02	1.00E+00
ENSBTAG00000003421	2.07E-02	1.00E+00	8.69E-01	1.69E-04	1.11E+00	7.66E-07	8.78E-01	1.71E-04	2.47E-01	8.00E-01
ENSBTAG00000026307	2.44E-01	1.00E+00	1.34E+00	4.50E-06	1.35E+00	3.09E-06	8.87E-01	4.51E-03	1.54E-01	1.00E+00
ENSBTAG00000008412	-4.92E-02	1.00E+00	-4.93E-01	1.39E-01	-2.16E-01	6.67E-01	2.40E-01	6.01E-01	-3.35E-01	8.00E-01
ENSBTAG00000017697	-1.21E+00	7.20E-03	-2.39E+00	8.00E-10	-1.62E+00	8.65E-06	-7.08E-01	7.80E-02	-2.26E-01	1.00E+00
ENSBTAG00000035373	1.91E+00	8.23E-01	0.00E+00	1.00E+00	-5.13E-15	1.00E+00	0.00E+00	1.00E+00	2.73E+00	6.01E-01
ENSBTAG00000040555	1.33E-01	1.00E+00	4.25E-02	1.00E+00	-1.34E-01	9.33E-01	4.15E-01	2.33E-01	3.01E-01	8.00E-01
ENSBTAG00000002344	-2.04E-01	1.00E+00	-1.47E-01	8.56E-01	5.91E-01	3.27E-02	3.72E-01	2.58E-01	-4.53E-02	1.00E+00
ENSBTAG00000002345	-6.82E-02	1.00E+00	1.34E+00	4.82E-09	1.62E+00	8.80E-13	1.01E+00	1.85E-05	2.85E-01	7.87E-01
ENSBTAG00000002346	-4.31E-01	3.92E-01	-2.47E+00	7.42E-25	-2.51E+00	8.88E-26	-2.08E+00	2.43E-18	2.12E-01	9.13E-01
ENSBTAG00000002349	1.32E+00	7.04E-01	-4.19E-01	1.00E+00	2.25E-01	1.00E+00	4.86E-01	9.64E-01	-4.89E-01	1.00E+00
ENSBTAG00000007523	3.72E-02	1.00E+00	-8.01E-01	3.42E-05	-1.28E+00	9.75E-12	-9.78E-01	3.23E-07	1.17E-01	1.00E+00
ENSBTAG00000006650	-8.23E-03	1.00E+00	1.07E+00	3.73E-03	1.60E+00	3.66E-06	1.39E+00	9.34E-05	1.34E-01	1.00E+00
ENSBTAG00000016687	4.50E-02	1.00E+00	4.08E-01	3.40E-01	9.28E-01	4.92E-03	6.46E-01	7.75E-02	3.85E-01	7.93E-01
ENSBTAG00000031551	-2.90E-01	8.23E-01	-2.07E+00	2.24E-16	-1.09E+00	1.59E-05	-1.23E+00	1.23E-06	7.98E-03	1.00E+00
ENSBTAG00000000405	-1.84E-01	9.27E-01	-8.20E-01	4.07E-05	-1.54E+00	2.25E-15	-1.61E+00	1.63E-16	2.15E-01	8.00E-01
ENSBTAG00000010524	-1.41E-01	1.00E+00	-3.97E-01	1.61E-01	-1.77E-01	6.87E-01	-1.17E-01	9.21E-01	5.17E-01	2.61E-01
ENSBTAG00000044656	0.00E+00	1.00E+00	0.00E+00	1.00E+00	-5.13E-15	1.00E+00	0.00E+00	1.00E+00	0.00E+00	1.00E+00
ENSBTAG00000010511	1.87E-01	1.00E+00	1.35E+00	5.19E-09	8.32E-01	5.08E-04	-1.33E-01	8.65E-01	-1.61E-01	1.00E+00
ENSBTAG00000016973	-1.22E-01	1.00E+00	-1.65E+00	7.90E-03	-1.28E+00	3.26E-02	-1.14E+00	6.42E-02	-2.44E-01	1.00E+00
ENSBTAG00000037397	-2.49E+00	8.72E-02	-1.22E+00	2.32E-01	-2.15E+00	2.58E-02	-1.04E+00	3.01E-01	-6.46E-01	9.26E-01
ENSBTAG00000005757	-4.09E-02	1.00E+00	-3.67E-01	1.48E-01	6.26E-01	4.95E-03	6.26E-01	5.84E-03	-1.72E-02	1.00E+00
ENSBTAG00000020535	-9.09E-01	1.34E-02	-1.22E+00	2.80E-05	-3.79E-01	2.90E-01	-2.58E-01	5.56E-01	4.55E-03	1.00E+00
ENSBTAG00000001503	-1.36E+00	8.58E-01	1.21E+00	4.43E-01	-1.25E-01	1.00E+00	1.39E+00	3.32E-01	9.57E-01	9.33E-01
ENSBTAG00000047238	-1.97E-01	1.00E+00	6.35E-02	1.00E+00	1.28E+00	2.88E-05	1.11E+00	4.71E-04	-8.83E-02	1.00E+00
ENSBTAG00000019524	-3.01E-01	1.00E+00	-1.29E-01	1.00E+00	3.22E-01	8.31E-01	3.29E-01	8.26E-01	1.09E+00	3.09E-01
ENSBTAG00000019521	7.82E-01	1.00E+00	-7.93E-01	7.87E-01	-1.48E-01	1.00E+00	7.52E-01	6.59E-01	-3.67E-01	1.00E+00
ENSBTAG00000002374	1.04E-01	1.00E+00	1.01E+00	2.58E-04	5.73E-01	6.16E-02	1.13E-01	9.96E-01	3.86E-01	6.63E-01
ENSBTAG00000002391	8.58E-02	1.00E+00	-6.31E-01	1.41E-01	3.70E-01	4.48E-01	1.26E-01	1.00E+00	4.16E-02	1.00E+00
ENSBTAG00000010096	7.48E-02	1.00E+00	-1.34E-01	8.36E-01	-3.75E-01	1.64E-01	-5.57E-01	2.48E-02	-7.43E-02	1.00E+00
ENSBTAG00000047734	2.54E-02	1.00E+00	2.12E-01	5.34E-01	3.04E-01	2.86E-01	4.91E-02	1.00E+00	2.12E-01	8.75E-01
ENSBTAG00000006457	8.55E-01	8.29E-01	8.02E-01	5.19E-01	-1.68E-01	1.00E+00	-1.99E-01	1.00E+00	9.87E-01	7.78E-01
ENSBTAG00000040319	1.92E+00	8.23E-01	0.00E+00	1.00E+00	-5.13E-15	1.00E+00	0.00E+00	1.00E+00	0.00E+00	1.00E+00
ENSBTAG00000037462	2.70E+00	5.76E-01	0.00E+00	1.00E+00	-5.13E-15	1.00E+00	0.00E+00	1.00E+00	0.00E+00	1.00E+00
ENSBTAG00000009327	6.79E-02	1.00E+00	-1.75E+00	1.67E-04	-2.57E+00	5.88E-08	-3.13E+00	4.81E-10	2.84E-01	1.00E+00

ENSBTAG00000008175	-2.32E-01	1.00E+00	-2.24E+00	7.20E-08	-1.04E+00	5.24E-03	-6.20E-01	1.31E-01	-9.09E-02	1.00E+00
ENSBTAG00000007934	-6.77E-02	1.00E+00	-4.96E-02	1.00E+00	2.10E-01	6.18E-01	-2.60E-01	4.95E-01	-3.38E-01	7.05E-01
ENSBTAG00000031598	-4.26E-01	5.10E-01	-1.11E+00	2.55E-05	-3.57E-01	2.88E-01	-4.54E-01	1.51E-01	-2.13E-01	9.63E-01
ENSBTAG00000013081	-1.26E+00	6.33E-02	-7.68E-01	1.70E-01	-1.76E+00	2.52E-04	-2.05E+00	2.35E-05	-9.46E-01	3.44E-01
ENSBTAG00000008184	3.73E-01	3.75E-01	1.66E+00	2.97E-17	1.38E+00	2.77E-12	1.24E+00	5.45E-10	-2.74E-01	7.03E-01
ENSBTAG00000042366	0.00E+00	1.00E+00	0.00E+00	1.00E+00	-5.13E-15	1.00E+00	0.00E+00	1.00E+00	0.00E+00	1.00E+00
ENSBTAG00000046466	0.00E+00	1.00E+00	0.00E+00	1.00E+00	-5.13E-15	1.00E+00	0.00E+00	1.00E+00	0.00E+00	1.00E+00
ENSBTAG00000008190	-3.46E-01	8.09E-01	-1.41E+00	2.21E-06	-1.37E+00	3.24E-06	-7.20E-01	2.51E-02	-5.66E-03	1.00E+00
ENSBTAG00000008195	1.62E-01	1.00E+00	1.62E+00	4.79E-09	1.73E-01	8.24E-01	-9.25E-01	4.15E-03	-1.35E-01	1.00E+00
ENSBTAG00000040295	5.62E-02	1.00E+00	1.79E-01	6.74E-01	4.05E-01	1.36E-01	5.16E-01	4.65E-02	-4.06E-01	4.77E-01
ENSBTAG00000031583	-5.27E-02	1.00E+00	-8.98E-01	2.28E-02	-1.24E+00	9.02E-04	-8.11E-01	3.88E-02	3.04E-01	8.92E-01
ENSBTAG00000031582	0.00E+00	1.00E+00	0.00E+00	1.00E+00	-5.13E-15	1.00E+00	0.00E+00	1.00E+00	0.00E+00	1.00E+00
ENSBTAG00000014448	1.48E+00	2.08E-05	2.30E+00	1.49E-13	2.20E+00	1.15E-12	1.26E+00	7.78E-05	4.35E-01	6.70E-01
ENSBTAG00000000704	1.64E-01	1.00E+00	-7.12E-02	1.00E+00	-4.49E-01	4.55E-02	-4.96E-01	2.73E-02	4.84E-01	1.97E-01
ENSBTAG00000015314	7.40E-02	1.00E+00	-1.02E+00	6.28E-03	-7.22E-01	7.32E-02	-1.15E+00	2.22E-03	-7.44E-03	1.00E+00
ENSBTAG00000037489	7.59E-01	3.20E-01	-1.42E-02	1.00E+00	9.82E-01	1.94E-02	6.46E-01	1.79E-01	2.88E-01	1.00E+00
ENSBTAG00000000390	4.59E-02	1.00E+00	1.22E-01	8.39E-01	4.24E-01	6.56E-02	8.24E-02	1.00E+00	3.90E-02	1.00E+00
ENSBTAG00000017435	-4.78E-01	6.99E-01	-1.84E+00	1.09E-06	-1.02E+00	7.19E-03	-6.45E-01	1.29E-01	-2.86E-02	1.00E+00
ENSBTAG00000046622	-5.60E-01	1.00E+00	1.13E+00	3.04E-01	6.55E-01	6.85E-01	-2.41E+00	1.08E-01	-4.17E+00	1.43E-01
ENSBTAG00000003842	4.79E-01	6.75E-01	2.16E+00	6.90E-10	2.29E+00	5.07E-11	9.28E-01	1.43E-02	-3.99E-01	8.00E-01
ENSBTAG00000002935	2.02E-01	9.31E-01	9.10E-01	3.63E-05	9.66E-01	9.76E-06	8.22E-01	2.62E-04	2.80E-01	7.62E-01
ENSBTAG00000004051	2.57E-02	1.00E+00	4.27E-01	5.68E-02	5.99E-01	4.16E-03	5.73E-01	7.69E-03	-4.21E-01	3.13E-01
ENSBTAG00000008446	-1.70E-03	1.00E+00	4.24E-02	1.00E+00	4.38E-01	2.40E-01	9.04E-01	3.98E-03	4.33E-01	6.27E-01
ENSBTAG00000043969	9.09E-02	1.00E+00	-2.31E-01	9.25E-01	-3.65E-01	6.57E-01	-3.46E-02	1.00E+00	-4.42E-01	8.86E-01
ENSBTAG00000008718	-5.08E-01	9.02E-01	-2.00E-02	1.00E+00	-9.46E-01	1.37E-01	5.52E-01	4.39E-01	-6.12E-01	8.00E-01
ENSBTAG00000002083	1.71E-01	1.00E+00	-1.97E+00	1.20E-10	-1.87E+00	8.02E-10	-1.84E+00	1.84E-09	5.93E-01	3.84E-01
ENSBTAG00000046557	0.00E+00	1.00E+00	0.00E+00	1.00E+00	-5.13E-15	1.00E+00	0.00E+00	1.00E+00	0.00E+00	1.00E+00
ENSBTAG00000047342	-3.58E+00	2.36E-01	1.00E-01	1.00E+00	-1.80E+00	3.02E-01	1.10E-01	1.00E+00	1.09E+00	8.00E-01
ENSBTAG00000043444	0.00E+00	1.00E+00	0.00E+00	1.00E+00	1.78E+00	4.79E-01	2.53E+00	2.71E-01	0.00E+00	1.00E+00
ENSBTAG00000046179	0.00E+00	1.00E+00	0.00E+00	1.00E+00	-5.13E-15	1.00E+00	0.00E+00	1.00E+00	0.00E+00	1.00E+00
ENSBTAG00000045444	0.00E+00	1.00E+00	0.00E+00	1.00E+00	-5.13E-15	1.00E+00	2.53E+00	2.71E-01	0.00E+00	1.00E+00
ENSBTAG00000047264	-6.37E-01	5.76E-01	-6.40E-01	2.27E-01	-2.16E-01	9.04E-01	-8.58E-01	8.27E-02	-1.72E+00	2.33E-03
ENSBTAG00000048148	-1.91E+00	8.23E-01	1.77E+00	3.28E-01	1.80E+00	2.83E-01	2.24E+00	1.29E-01	3.86E-04	1.00E+00
ENSBTAG00000047401	-1.24E+00	2.07E-01	-4.39E-01	6.71E-01	-1.14E+00	7.79E-02	-1.75E+00	4.13E-03	-1.59E+00	8.49E-02
ENSBTAG00000045825	-1.27E+00	3.84E-01	-1.52E+00	5.85E-02	4.73E-01	6.81E-01	1.44E+00	3.34E-02	-1.21E+00	4.87E-01

ENSBTAG00000045952	-1.91E+00	8.23E-01	-1.91E+00	4.69E-01	6.51E-01	9.78E-01	2.61E+00	5.21E-02	6.07E-03	1.00E+00
ENSBTAG00000045747	8.13E-01	1.00E+00	-1.91E+00	4.69E-01	1.14E+00	6.49E-01	2.27E+00	1.26E-01	6.90E-03	1.00E+00
ENSBTAG00000048255	0.00E+00	1.00E+00	0.00E+00	1.00E+00	-5.13E-15	1.00E+00	0.00E+00	1.00E+00	0.00E+00	1.00E+00
ENSBTAG00000046882	0.00E+00	1.00E+00	0.00E+00	1.00E+00	-5.13E-15	1.00E+00	0.00E+00	1.00E+00	0.00E+00	1.00E+00
ENSBTAG00000046680	-1.05E+00	5.68E-01	-1.84E+00	1.40E-02	-2.78E+00	1.92E-04	-5.21E+00	7.78E-09	-4.99E+00	1.75E-06
ENSBTAG00000045801	0.00E+00	1.00E+00	0.00E+00	1.00E+00	-5.13E-15	1.00E+00	0.00E+00	1.00E+00	0.00E+00	1.00E+00
ENSBTAG00000014417	1.73E-01	1.00E+00	-2.43E-01	7.95E-01	5.19E-01	2.61E-01	2.12E+00	2.06E-09	5.99E-01	5.48E-01
ENSBTAG00000011753	-4.57E-01	2.51E-01	-2.52E-01	4.25E-01	7.86E-02	1.00E+00	2.91E-01	3.25E-01	-4.57E-02	1.00E+00
ENSBTAG00000026286	1.97E-01	1.00E+00	4.14E-01	1.65E-01	2.32E-01	5.41E-01	1.23E-01	9.24E-01	3.11E-01	7.70E-01
ENSBTAG00000003305	1.97E+00	8.58E-05	1.86E+00	3.25E-05	5.70E-01	3.28E-01	4.31E-01	5.21E-01	5.75E-01	7.35E-01
ENSBTAG00000009780	-1.38E-01	1.00E+00	-1.92E+00	2.09E-15	-1.61E+00	2.10E-11	-1.31E+00	7.74E-08	1.31E-01	1.00E+00
ENSBTAG00000006212	-4.02E-03	1.00E+00	-1.09E+00	6.02E-05	-1.55E+00	5.24E-09	-2.09E-01	6.51E-01	-1.44E-01	1.00E+00
ENSBTAG00000044775	0.00E+00	1.00E+00	0.00E+00	1.00E+00	-5.13E-15	1.00E+00	0.00E+00	1.00E+00	0.00E+00	1.00E+00
ENSBTAG00000011875	-1.42E-01	1.00E+00	-2.96E-01	6.86E-01	4.78E-01	3.23E-01	4.82E-01	3.29E-01	-4.34E-02	1.00E+00
ENSBTAG00000018589	-1.03E-01	1.00E+00	-4.93E-01	3.80E-02	-4.85E-01	4.00E-02	-1.90E-01	5.90E-01	2.23E-01	8.10E-01
ENSBTAG00000045767	-3.37E-01	7.06E-01	-4.03E-02	1.00E+00	1.37E-01	8.69E-01	5.30E-02	1.00E+00	2.96E-03	1.00E+00
ENSBTAG00000047249	-4.75E-02	1.00E+00	1.60E-01	6.47E-01	3.14E-01	1.91E-01	4.59E-01	3.69E-02	-9.94E-02	1.00E+00
ENSBTAG00000046551	0.00E+00	1.00E+00	0.00E+00	1.00E+00	-5.13E-15	1.00E+00	0.00E+00	1.00E+00	0.00E+00	1.00E+00
ENSBTAG00000044083	-2.80E-02	1.00E+00	-1.13E+00	1.39E-04	1.61E-01	8.24E-01	7.46E-02	1.00E+00	-1.51E-01	1.00E+00
ENSBTAG00000019517	-7.79E-01	3.94E-01	-4.57E-01	4.66E-01	-4.78E-01	4.29E-01	-5.54E-01	3.44E-01	-2.64E-01	1.00E+00
ENSBTAG00000014810	0.00E+00	1.00E+00	0.00E+00	1.00E+00	-5.13E-15	1.00E+00	3.39E+00	8.39E-02	0.00E+00	1.00E+00
ENSBTAG00000001374	3.68E-01	7.65E-01	-6.93E-01	3.65E-02	-1.55E+00	4.36E-07	-1.97E+00	2.74E-10	3.13E-01	8.00E-01
ENSBTAG00000026278	4.58E-01	1.00E+00	-1.45E+00	5.09E-02	-1.54E-01	1.00E+00	1.14E+00	1.21E-01	1.20E-01	1.00E+00
ENSBTAG00000040432	-8.96E-01	7.25E-01	-1.11E+00	2.00E-01	-2.04E+00	1.01E-02	-7.40E-01	4.40E-01	-1.40E+00	3.88E-01
ENSBTAG00000010339	-1.95E-01	1.00E+00	-8.71E-01	3.84E-02	8.72E-01	2.02E-02	8.74E-01	2.24E-02	-3.95E-01	8.00E-01
ENSBTAG00000017075	-2.34E-01	1.00E+00	-7.90E-01	2.96E-01	-1.18E+00	7.64E-02	-7.54E-01	3.16E-01	-1.32E+00	2.97E-01
ENSBTAG00000017067	-1.56E-01	1.00E+00	-7.41E-02	1.00E+00	6.21E-01	2.41E-02	8.55E-01	1.29E-03	-5.27E-01	3.30E-01
ENSBTAG00000044466	0.00E+00	1.00E+00	0.00E+00	1.00E+00	-5.13E-15	1.00E+00	0.00E+00	1.00E+00	0.00E+00	1.00E+00
ENSBTAG00000009022	-5.37E-01	3.40E-01	-1.22E+00	2.81E-05	-6.68E-01	2.83E-02	-2.34E-01	6.26E-01	-1.45E-01	1.00E+00
ENSBTAG00000008487	-6.03E-01	4.85E-01	-2.38E+00	8.26E-09	-1.79E+00	3.23E-06	-1.70E+00	1.19E-05	-9.22E-01	1.62E-01
ENSBTAG00000009153	-8.82E-03	1.00E+00	-5.55E-01	1.60E-02	-5.43E-01	1.88E-02	-1.16E+00	5.73E-08	2.17E-01	8.34E-01
ENSBTAG00000000321	1.86E-01	9.99E-01	6.59E-01	5.35E-03	7.36E-01	1.48E-03	1.40E+00	2.18E-10	3.87E-01	5.03E-01
ENSBTAG00000020544	-1.56E-01	1.00E+00	-4.71E-01	7.24E-02	2.96E-01	3.24E-01	4.23E-01	1.15E-01	6.08E-02	1.00E+00
ENSBTAG00000005197	-1.18E-01	1.00E+00	8.26E-03	1.00E+00	5.50E-02	1.00E+00	6.37E-02	1.00E+00	7.45E-02	1.00E+00
ENSBTAG00000044552	0.00E+00	1.00E+00	0.00E+00	1.00E+00	-5.13E-15	1.00E+00	0.00E+00	1.00E+00	0.00E+00	1.00E+00

ENSBTAG00000020742	3.92E-01	1.00E+00	2.42E+00	5.32E-04	2.37E+00	6.47E-04	2.18E+00	2.47E-03	-1.25E+00	7.43E-01
ENSBTAG00000002253	-5.15E-01	1.00E+00	-4.31E-01	1.00E+00	-1.44E+00	4.79E-01	-6.92E-01	8.81E-01	5.54E-03	1.00E+00
ENSBTAG00000005798	6.02E-03	1.00E+00	-7.23E-01	9.45E-01	-1.49E-01	1.00E+00	-2.71E+00	2.05E-01	-2.71E+00	6.01E-01
ENSBTAG00000017941	3.17E-01	6.75E-01	1.60E+00	4.00E-13	1.85E+00	2.37E-17	8.21E-01	4.72E-04	5.23E-02	1.00E+00
ENSBTAG00000017939	3.03E-01	8.42E-01	1.61E+00	1.73E-08	1.94E+00	6.40E-12	1.44E+00	6.00E-07	5.47E-01	4.28E-01
ENSBTAG00000000781	1.50E-01	1.00E+00	-1.30E-01	1.00E+00	2.11E-01	7.62E-01	5.94E-03	1.00E+00	1.41E-01	1.00E+00
ENSBTAG00000046507	0.00E+00	1.00E+00	0.00E+00	1.00E+00	-5.13E-15	1.00E+00	0.00E+00	1.00E+00	0.00E+00	1.00E+00
ENSBTAG00000044875	0.00E+00	1.00E+00	0.00E+00	1.00E+00	-5.13E-15	1.00E+00	0.00E+00	1.00E+00	0.00E+00	1.00E+00
ENSBTAG00000026275	4.55E+00	2.12E-26	6.59E+00	8.58E-51	7.78E+00	9.34E-66	7.73E+00	3.77E-65	2.60E+00	1.03E-07
ENSBTAG00000005089	5.90E-01	6.37E-02	-1.68E-01	6.98E-01	7.24E-02	1.00E+00	3.13E-01	2.75E-01	3.32E-01	6.01E-01
ENSBTAG00000017082	-1.45E-01	1.00E+00	-8.30E-01	6.34E-04	-1.79E+00	1.91E-14	-1.72E+00	2.24E-13	4.10E-02	1.00E+00
ENSBTAG00000016139	-1.47E-01	1.00E+00	7.07E-02	1.00E+00	4.06E-01	3.78E-01	6.46E-01	1.01E-01	-7.68E-02	1.00E+00
ENSBTAG00000009461	-1.71E-01	1.00E+00	-8.36E-01	3.93E-03	-9.32E-01	9.92E-04	-1.36E+00	1.29E-06	-3.13E-01	8.00E-01
ENSBTAG00000009462	-1.47E-01	1.00E+00	-5.74E-01	1.08E-01	-9.35E-01	3.70E-03	-6.88E-01	4.87E-02	-6.93E-02	1.00E+00
ENSBTAG00000007879	1.10E-02	1.00E+00	-1.65E-01	1.00E+00	-3.09E-01	9.72E-01	1.09E-01	1.00E+00	2.84E-01	1.00E+00
ENSBTAG00000011969	9.09E-02	1.00E+00	1.17E+00	1.74E-03	6.18E-01	1.46E-01	3.44E-01	5.35E-01	-1.65E-02	1.00E+00
ENSBTAG00000037454	0.00E+00	1.00E+00	2.79E+00	2.10E-01	1.78E+00	4.79E-01	3.03E+00	1.50E-01	0.00E+00	1.00E+00
ENSBTAG00000004077	5.75E-02	1.00E+00	9.31E-01	8.20E-04	8.26E-01	3.37E-03	1.10E+00	7.26E-05	2.90E-01	8.00E-01
ENSBTAG00000003020	-2.77E-01	8.92E-01	-1.14E+00	1.23E-04	-1.90E+00	7.64E-11	-1.25E+00	2.14E-05	1.00E+00	1.01E-02
ENSBTAG00000014834	-4.35E-01	1.00E+00	7.74E-01	4.10E-01	-6.25E-01	6.42E-01	-8.37E-01	4.79E-01	-6.30E-01	9.78E-01
ENSBTAG00000001470	4.43E-01	2.05E-01	1.10E+00	2.81E-08	5.12E-01	1.81E-02	2.75E-01	3.08E-01	6.91E-02	1.00E+00
ENSBTAG00000021430	7.76E-01	7.54E-02	1.33E+00	5.02E-06	2.30E-01	6.77E-01	-7.76E-01	2.66E-02	-1.27E-01	1.00E+00
ENSBTAG00000020524	3.92E+00	8.84E-04	4.33E+00	6.15E-06	2.61E+00	4.53E-02	1.50E+00	4.52E-01	-1.91E+00	8.00E-01
ENSBTAG00000005448	1.95E+00	4.29E-04	1.62E+00	9.38E-04	-2.52E-01	8.97E-01	-5.48E-01	4.44E-01	-5.34E-01	8.00E-01
ENSBTAG00000005446	-1.95E-01	9.59E-01	-8.61E-02	1.00E+00	5.26E-02	1.00E+00	-1.04E-01	9.51E-01	-2.38E-01	8.00E-01
ENSBTAG00000014742	-1.19E-01	1.00E+00	-9.29E-02	9.77E-01	3.80E-02	1.00E+00	-2.13E-01	5.12E-01	2.45E-01	8.00E-01
ENSBTAG00000003617	-2.71E-01	8.90E-01	-2.74E-01	5.13E-01	-4.92E-02	1.00E+00	-3.53E-02	1.00E+00	-2.15E-01	9.93E-01
ENSBTAG00000012050	1.09E+00	4.17E-02	1.22E+00	2.00E-03	9.42E-02	1.00E+00	-3.51E-01	6.06E-01	-2.08E-01	1.00E+00
ENSBTAG00000004106	5.40E-01	3.56E-01	9.03E-01	2.00E-03	8.55E-01	3.30E-03	4.41E-01	2.20E-01	2.99E-01	8.24E-01
ENSBTAG00000047266	4.77E-01	9.13E-01	9.39E-01	9.02E-02	1.05E+00	4.16E-02	6.94E-01	2.70E-01	-1.05E+00	4.87E-01
ENSBTAG00000044279	0.00E+00	1.00E+00	0.00E+00	1.00E+00	-5.13E-15	1.00E+00	0.00E+00	1.00E+00	0.00E+00	1.00E+00
ENSBTAG00000009691	1.64E+00	3.62E-07	1.83E+00	6.85E-10	7.85E-01	1.76E-02	-4.59E-02	1.00E+00	-2.78E-01	9.37E-01
ENSBTAG00000010659	6.99E-02	1.00E+00	-1.58E-01	7.91E-01	-3.85E-01	1.99E-01	-2.62E-01	4.74E-01	3.39E-01	6.71E-01
ENSBTAG00000044580	0.00E+00	1.00E+00	0.00E+00	1.00E+00	-5.13E-15	1.00E+00	0.00E+00	1.00E+00	0.00E+00	1.00E+00
ENSBTAG00000036637	0.00E+00	1.00E+00	0.00E+00	1.00E+00	-5.13E-15	1.00E+00	0.00E+00	1.00E+00	0.00E+00	1.00E+00

ENSBTAG00000026273	7.02E-01	1.00E+00	7.59E-01	7.78E-01	1.90E-01	1.00E+00	1.86E+00	1.10E-01	-1.31E+00	9.13E-01
ENSBTAG00000011818	-6.24E-01	6.61E-01	9.58E-03	1.00E+00	-2.83E-02	1.00E+00	-2.50E-01	8.40E-01	-7.21E-01	5.80E-01
ENSBTAG00000047808	0.00E+00	1.00E+00	0.00E+00	1.00E+00	-5.13E-15	1.00E+00	0.00E+00	1.00E+00	0.00E+00	1.00E+00
ENSBTAG00000014343	-3.08E-01	8.56E-01	-1.69E+00	3.59E-07	-1.00E+00	1.78E-03	8.26E-02	1.00E+00	1.49E-01	1.00E+00
ENSBTAG00000007900	-1.82E-01	9.65E-01	-7.00E-01	1.38E-03	-8.91E-01	2.81E-05	-6.63E-01	3.02E-03	-1.07E-01	1.00E+00
ENSBTAG00000000345	-7.83E-01	5.75E-01	-1.88E+00	3.45E-04	-4.24E+00	1.08E-14	-3.75E+00	4.66E-12	-3.16E-01	1.00E+00
ENSBTAG00000000343	-2.62E-01	7.47E-01	-1.02E+00	6.55E-07	-1.78E+00	2.42E-18	-1.50E+00	1.66E-13	-2.90E-01	6.49E-01
ENSBTAG00000000342	1.04E-01	1.00E+00	-2.16E-01	4.59E-01	-2.97E-01	2.40E-01	-2.29E-02	1.00E+00	1.07E+00	2.85E-06
ENSBTAG00000020157	3.60E-01	9.82E-01	-3.50E-01	6.67E-01	-1.69E+00	5.17E-04	-2.92E+00	3.72E-08	1.36E+00	2.66E-02
ENSBTAG00000047767	-2.28E+00	2.79E-02	-2.21E+00	6.15E-03	-3.89E+00	1.74E-05	-6.44E+00	1.22E-07	-3.74E+00	1.58E-03
ENSBTAG00000009868	1.68E+00	7.73E-01	8.75E-01	8.53E-01	1.51E+00	4.28E-01	-1.54E-01	1.00E+00	7.93E-01	1.00E+00
ENSBTAG00000014468	-9.38E-02	1.00E+00	-1.09E+00	4.05E-06	-1.16E+00	6.54E-07	-6.37E-01	1.20E-02	-1.05E-01	1.00E+00
ENSBTAG00000014465	1.52E+00	2.06E-03	2.66E+00	1.42E-10	2.03E+00	7.87E-07	2.89E+00	5.16E-12	6.58E-01	5.60E-01
ENSBTAG00000012038	5.42E-01	8.23E-01	2.60E+00	4.52E-08	1.46E+00	2.83E-03	1.05E+00	4.79E-02	5.62E-01	8.00E-01
ENSBTAG00000047125	1.92E+00	8.23E-01	1.98E+00	4.43E-01	1.78E+00	4.79E-01	0.00E+00	1.00E+00	0.00E+00	1.00E+00
ENSBTAG00000031247	0.00E+00	1.00E+00	0.00E+00	1.00E+00	-5.13E-15	1.00E+00	0.00E+00	1.00E+00	0.00E+00	1.00E+00
ENSBTAG00000001139	-1.82E-01	1.00E+00	-2.05E-01	1.00E+00	-7.75E-01	4.79E-01	-3.55E-01	9.30E-01	-6.70E-02	1.00E+00
ENSBTAG00000001138	-3.41E-01	8.74E-01	-2.01E-01	8.42E-01	-7.20E-02	1.00E+00	-2.94E-01	6.06E-01	1.61E-01	1.00E+00
ENSBTAG00000001134	1.05E-02	1.00E+00	1.40E-01	7.01E-01	6.80E-01	3.34E-04	5.32E-01	7.88E-03	1.25E-01	1.00E+00
ENSBTAG00000024772	2.16E-01	1.00E+00	-1.41E+00	1.18E-03	-7.56E-01	9.35E-02	-3.92E-01	4.97E-01	-4.51E-01	8.00E-01
ENSBTAG00000001131	-6.56E-02	1.00E+00	-1.02E+00	1.44E-05	-5.94E-01	1.58E-02	-1.54E-01	7.66E-01	-2.66E-02	1.00E+00
ENSBTAG00000003446	-1.07E+00	8.59E-03	-2.68E+00	6.58E-16	-1.06E+00	1.43E-03	-1.95E-01	8.24E-01	2.11E-01	1.00E+00
ENSBTAG00000003432	-2.42E+00	1.76E-02	-2.34E+00	3.98E-03	-2.06E+00	5.67E-03	-9.18E-01	2.41E-01	-8.91E-01	6.57E-01
ENSBTAG00000003430	5.18E+00	7.43E-25	1.94E+00	5.68E-03	1.55E+00	3.86E-02	2.74E+00	5.04E-06	4.97E-01	1.00E+00
ENSBTAG00000026248	9.50E-03	1.00E+00	-1.30E+00	2.24E-07	-9.14E-01	3.28E-04	-5.21E-01	6.82E-02	-5.37E-01	2.97E-01
ENSBTAG00000015738	1.26E-01	1.00E+00	-8.77E-01	4.11E-04	-1.74E-01	7.15E-01	-1.05E-01	9.76E-01	3.68E-01	6.01E-01
ENSBTAG00000006495	1.13E-01	1.00E+00	-1.79E-01	6.73E-01	-1.09E-01	9.38E-01	5.99E-02	1.00E+00	-2.20E-01	8.75E-01
ENSBTAG00000008295	8.59E-01	3.30E-01	-2.16E-01	9.45E-01	-6.54E-01	2.62E-01	-1.96E-01	9.78E-01	3.74E-01	9.49E-01
ENSBTAG00000011738	-4.31E-02	1.00E+00	-8.64E-01	1.28E-04	-1.36E+00	5.62E-10	-1.31E+00	3.47E-09	4.56E-01	3.33E-01
ENSBTAG00000026246	-2.13E-01	9.32E-01	-7.77E-01	1.23E-03	-1.21E+00	1.64E-07	-1.09E+00	3.47E-06	2.53E-03	1.00E+00
ENSBTAG00000020528	-1.77E-01	9.43E-01	-1.75E+00	5.90E-19	-1.94E+00	4.78E-23	-1.26E+00	1.12E-10	-3.53E-02	1.00E+00
ENSBTAG00000003169	-4.97E-01	4.30E-01	-2.38E+00	2.48E-16	-2.05E+00	8.15E-13	-1.99E+00	4.36E-12	-1.07E-01	1.00E+00
ENSBTAG00000003160	-9.81E-02	1.00E+00	-1.14E+00	3.00E-06	-9.40E-01	1.27E-04	-6.24E-01	1.82E-02	5.17E-03	1.00E+00
ENSBTAG00000039601	-2.64E-01	1.00E+00	-1.06E+00	9.41E-03	-1.17E+00	3.22E-03	-1.26E+00	1.72E-03	-2.92E-01	9.86E-01
ENSBTAG00000046753	5.61E-01	1.00E+00	-7.94E-01	7.85E-01	-1.55E-01	1.00E+00	1.71E+00	6.74E-02	-8.64E-01	1.00E+00

ENSBTAG00000000713	2.03E-01	1.00E+00	-2.21E+00	9.98E-13	-1.29E+00	3.33E-05	-5.98E-01	9.65E-02	5.55E-01	4.45E-01
ENSBTAG00000042717	0.00E+00	1.00E+00	0.00E+00	1.00E+00	-5.13E-15	1.00E+00	0.00E+00	1.00E+00	0.00E+00	1.00E+00
ENSBTAG00000017456	1.55E-01	1.00E+00	-5.63E-01	7.89E-01	-3.81E-01	9.55E-01	-8.62E-01	5.09E-01	9.25E-01	8.00E-01
ENSBTAG00000015272	-4.14E-02	1.00E+00	-1.19E+00	4.37E-06	-9.26E-01	3.98E-04	-5.76E-01	4.62E-02	-1.94E-02	1.00E+00
ENSBTAG00000038577	-6.14E-01	6.14E-01	-1.59E+00	7.60E-04	-1.33E+00	3.72E-03	-1.10E+00	1.98E-02	-6.74E-01	5.85E-01
ENSBTAG00000016765	5.31E-01	1.00E+00	-2.70E+00	2.42E-01	-9.16E-01	8.10E-01	6.99E-01	8.54E-01	1.18E+00	8.33E-01
ENSBTAG00000026243	-3.61E-01	6.97E-01	-6.17E-01	3.74E-02	-3.90E-01	2.47E-01	-4.85E-01	1.30E-01	-3.88E-01	6.28E-01
ENSBTAG00000016763	-4.77E-01	2.29E-01	-7.86E-01	6.91E-04	3.29E-01	2.35E-01	4.98E-01	4.61E-02	1.00E-01	1.00E+00
ENSBTAG00000001068	-3.07E-01	8.23E-01	-1.63E+00	1.33E-07	-1.55E+00	2.21E-07	-2.14E+00	6.12E-12	-3.49E-01	7.90E-01
ENSBTAG00000042708	0.00E+00	1.00E+00	0.00E+00	1.00E+00	-5.13E-15	1.00E+00	0.00E+00	1.00E+00	0.00E+00	1.00E+00
ENSBTAG00000001066	-9.39E-01	3.71E-03	-2.53E+00	1.07E-18	-7.66E-02	1.00E+00	2.54E-01	5.02E-01	-3.76E-01	6.27E-01
ENSBTAG00000010521	-1.16E-01	1.00E+00	-8.68E-01	1.27E-02	-7.27E-01	3.75E-02	-8.58E-01	1.34E-02	-3.25E-01	8.24E-01
ENSBTAG00000010496	-8.78E-01	3.16E-01	-1.85E+00	1.23E-04	-2.46E+00	3.39E-07	-2.99E+00	2.75E-09	-2.66E-01	1.00E+00
ENSBTAG00000008869	-8.99E-01	9.63E-01	-8.21E-01	6.67E-01	-3.98E-01	9.79E-01	2.08E-01	1.00E+00	8.60E-01	8.01E-01
ENSBTAG00000015017	-2.00E-01	1.00E+00	6.91E-01	2.56E-01	2.14E+00	1.27E-06	1.19E+00	1.86E-02	-3.71E-01	1.00E+00
ENSBTAG00000015011	-4.54E-01	3.75E-01	3.12E-01	3.31E-01	8.48E-01	4.73E-04	4.16E-01	1.52E-01	-2.61E-02	1.00E+00
ENSBTAG00000015009	-1.57E-01	1.00E+00	-5.12E-01	2.04E-02	-5.23E-01	1.71E-02	-7.90E-01	1.65E-04	-7.58E-02	1.00E+00
ENSBTAG00000011470	3.95E-01	1.00E+00	-4.20E-01	1.00E+00	-6.55E-01	8.97E-01	-1.74E-01	1.00E+00	9.30E-01	9.12E-01
ENSBTAG00000012668	-5.55E-01	7.22E-01	-2.71E+00	4.26E-07	-2.67E-02	1.00E+00	5.76E-01	2.42E-01	1.22E-01	1.00E+00
ENSBTAG00000037549	-6.50E-01	1.79E-01	-1.23E+00	3.22E-05	-1.49E+00	2.98E-07	-1.31E+00	9.87E-06	-2.31E-02	1.00E+00
ENSBTAG00000007016	-1.13E-01	1.00E+00	-7.11E-01	4.48E-03	-9.17E-01	1.45E-04	-2.02E-01	6.17E-01	2.69E-02	1.00E+00
ENSBTAG00000030966	-4.05E-02	1.00E+00	-4.17E-02	1.00E+00	-1.07E-01	9.22E-01	-2.62E-01	3.95E-01	1.13E-01	1.00E+00
ENSBTAG00000002881	-1.31E-01	1.00E+00	2.72E-02	1.00E+00	2.03E-01	6.03E-01	2.38E-01	5.02E-01	-3.82E-02	1.00E+00
ENSBTAG00000030965	-1.47E-01	1.00E+00	5.05E-01	7.36E-02	4.88E-01	8.71E-02	6.26E-01	2.24E-02	-1.40E-01	1.00E+00
ENSBTAG00000036362	0.00E+00	1.00E+00	0.00E+00	1.00E+00	-5.13E-15	1.00E+00	0.00E+00	1.00E+00	0.00E+00	1.00E+00
ENSBTAG00000029817	0.00E+00	1.00E+00	0.00E+00	1.00E+00	-5.13E-15	1.00E+00	0.00E+00	1.00E+00	0.00E+00	1.00E+00
ENSBTAG00000029920	0.00E+00	1.00E+00	0.00E+00	1.00E+00	-5.13E-15	1.00E+00	0.00E+00	1.00E+00	0.00E+00	1.00E+00
ENSBTAG00000002871	-4.70E-02	1.00E+00	-3.06E-01	3.97E-01	-2.74E-01	4.64E-01	-2.29E-01	5.92E-01	-8.19E-02	1.00E+00
ENSBTAG00000007012	2.47E-01	8.23E-01	1.49E+00	3.13E-12	1.19E+00	3.02E-08	3.28E-01	2.44E-01	-6.00E-02	1.00E+00
ENSBTAG00000030956	2.69E-01	9.49E-01	-3.35E-02	1.00E+00	-3.92E-01	3.33E-01	-7.51E-01	2.62E-02	-2.76E-02	1.00E+00
ENSBTAG00000016757	-1.02E+00	1.47E-02	-1.48E-01	9.47E-01	-2.94E-01	5.53E-01	4.09E-01	3.46E-01	5.75E-01	4.46E-01
ENSBTAG00000026236	2.85E-01	1.00E+00	2.71E-01	7.43E-01	-4.48E-01	4.16E-01	-5.98E-01	2.24E-01	9.41E-01	1.88E-01
ENSBTAG00000038074	-3.99E-01	1.00E+00	7.18E-01	3.36E-01	-9.51E-01	2.40E-01	-2.04E+00	9.85E-03	-4.17E-02	1.00E+00
ENSBTAG00000012201	1.18E-01	1.00E+00	-1.80E-01	5.15E-01	-8.73E-01	1.70E-06	-1.48E+00	1.59E-16	1.85E-01	8.38E-01
ENSBTAG00000009840	6.95E-01	9.39E-01	1.57E+00	4.20E-02	-1.97E+00	1.14E-01	-1.13E+00	4.02E-01	1.09E+00	6.13E-01

ENSBTAG00000045180	-1.49E-01	1.00E+00	-8.32E-01	5.91E-01	-2.73E+00	3.50E-02	-2.75E+00	3.68E-02	1.17E+00	5.50E-01
ENSBTAG00000047379	3.30E-02	1.00E+00	1.43E-01	8.28E-01	-2.33E-01	5.05E-01	-4.54E-01	1.03E-01	5.65E-01	1.97E-01
ENSBTAG00000005450	5.30E-02	1.00E+00	-4.51E-01	2.91E-01	-1.28E+00	1.28E-04	-1.90E+00	1.12E-08	-1.77E-01	1.00E+00
ENSBTAG00000019435	-2.70E+00	5.76E-01	-2.70E+00	2.42E-01	-9.18E-01	8.09E-01	-9.34E-01	8.08E-01	-2.70E+00	6.01E-01
ENSBTAG00000039512	1.48E-01	1.00E+00	6.80E-01	7.93E-02	6.58E-01	9.11E-02	6.40E-01	1.11E-01	6.68E-01	3.81E-01
ENSBTAG00000039720	-7.65E-02	1.00E+00	5.54E-02	1.00E+00	-1.40E+00	3.04E-05	-1.63E+00	1.46E-06	6.93E-02	1.00E+00
ENSBTAG00000030951	1.10E-01	1.00E+00	3.93E-01	2.30E-01	7.87E-01	3.27E-03	1.01E+00	1.15E-04	-2.46E-01	8.99E-01
ENSBTAG00000047572	2.53E-01	1.00E+00	4.19E-01	4.69E-01	2.18E-01	8.70E-01	8.85E-02	1.00E+00	1.14E-01	1.00E+00
ENSBTAG00000040568	1.14E-01	1.00E+00	-2.24E-01	4.48E-01	1.07E-01	8.96E-01	-2.47E-01	4.01E-01	2.25E-01	8.00E-01
ENSBTAG00000016190	0.00E+00	1.00E+00	0.00E+00	1.00E+00	-5.13E-15	1.00E+00	0.00E+00	1.00E+00	0.00E+00	1.00E+00
ENSBTAG00000002094	1.71E-02	1.00E+00	5.28E-02	1.00E+00	-4.48E-02	1.00E+00	1.42E-01	9.10E-01	3.37E-01	7.85E-01
ENSBTAG00000002090	3.08E-01	7.36E-01	1.05E+00	8.46E-06	1.07E+00	4.64E-06	4.49E-01	1.07E-01	1.35E-02	1.00E+00
ENSBTAG00000020440	-8.73E-02	1.00E+00	-4.72E-01	1.25E-01	5.07E-01	8.14E-02	5.21E-01	7.75E-02	3.72E-01	6.28E-01
ENSBTAG00000020439	4.22E-02	1.00E+00	4.45E-01	8.83E-02	6.67E-01	5.18E-03	3.09E-01	3.08E-01	-3.49E-03	1.00E+00
ENSBTAG00000044040	-6.17E-02	1.00E+00	6.55E-01	1.23E-03	1.11E+00	8.35E-09	9.89E-01	4.85E-07	-2.98E-02	1.00E+00
ENSBTAG00000046248	5.72E-02	1.00E+00	2.44E-02	1.00E+00	1.78E-01	8.81E-01	4.36E-01	3.31E-01	-1.85E-01	1.00E+00
ENSBTAG00000004242	-1.67E-01	1.00E+00	-4.99E-01	2.65E-02	-3.96E-01	9.58E-02	-3.35E-01	1.92E-01	-1.97E-01	8.75E-01
ENSBTAG00000015737	-1.23E-01	1.00E+00	-9.56E-01	2.68E-01	-2.06E-01	1.00E+00	4.69E-01	7.21E-01	1.61E-01	1.00E+00
ENSBTAG00000007118	-1.46E-01	1.00E+00	-1.87E+00	5.66E-16	-1.94E+00	3.07E-17	-1.55E+00	1.77E-11	2.43E-01	8.06E-01
ENSBTAG00000007113	5.88E-02	1.00E+00	-6.40E-01	3.53E-03	-2.19E-01	4.75E-01	1.25E-02	1.00E+00	3.78E-01	4.44E-01
ENSBTAG00000005679	-8.84E-01	1.00E+00	-3.01E-01	1.00E+00	-3.60E+00	4.49E-02	-3.60E+00	4.85E-02	-3.60E+00	3.09E-01
ENSBTAG00000045896	-1.41E+00	7.69E-01	5.05E-01	8.50E-01	1.44E+00	8.76E-02	5.28E-01	8.03E-01	-1.42E+00	7.68E-01
ENSBTAG00000019181	2.95E-01	6.59E-01	4.66E-01	4.76E-02	-2.67E-01	3.50E-01	-3.42E-01	1.97E-01	-6.43E-02	1.00E+00
ENSBTAG00000022825	6.43E-01	7.26E-02	6.15E-01	1.91E-02	4.68E-01	9.50E-02	2.75E-01	4.39E-01	1.15E-02	1.00E+00
ENSBTAG00000046010	4.89E+00	4.29E-23	6.81E+00	1.00E-39	6.58E+00	5.47E-38	6.09E+00	9.18E-34	2.07E+00	4.85E-04
ENSBTAG00000004135	1.25E+00	1.19E-04	1.92E+00	1.24E-11	2.14E+00	4.16E-14	1.23E+00	2.36E-05	4.19E-01	6.21E-01
ENSBTAG00000046943	0.00E+00	1.00E+00	0.00E+00	1.00E+00	-5.13E-15	1.00E+00	0.00E+00	1.00E+00	0.00E+00	1.00E+00
ENSBTAG00000003288	-4.96E-02	1.00E+00	-2.20E-01	4.41E-01	5.05E-01	1.43E-02	2.47E-01	3.51E-01	-7.48E-02	1.00E+00
ENSBTAG00000005908	4.66E-01	5.19E-01	4.75E-01	1.74E-01	-1.93E-01	7.82E-01	-5.21E-01	1.60E-01	5.49E-01	4.08E-01
ENSBTAG00000007364	-6.08E-01	1.85E-01	-6.46E-01	3.10E-02	-1.50E-01	8.55E-01	-3.92E-01	2.60E-01	2.38E-01	9.07E-01
ENSBTAG00000007367	1.52E-01	1.00E+00	2.11E+00	7.32E-29	1.85E+00	7.29E-23	1.09E+00	1.55E-08	-1.87E-01	8.94E-01
ENSBTAG00000007370	2.15E-02	1.00E+00	8.02E-01	5.41E-06	7.91E-01	6.80E-06	4.29E-01	2.92E-02	3.78E-02	1.00E+00
ENSBTAG00000007372	0.00E+00	1.00E+00	0.00E+00	1.00E+00	-5.13E-15	1.00E+00	0.00E+00	1.00E+00	0.00E+00	1.00E+00
ENSBTAG00000000725	3.04E-02	1.00E+00	1.71E+00	1.10E-11	1.74E+00	2.93E-12	1.28E+00	6.26E-07	8.07E-02	1.00E+00
ENSBTAG00000007384	3.77E-01	7.94E-01	1.49E+00	1.23E-06	1.12E+00	3.54E-04	9.51E-01	3.54E-03	2.40E-01	9.98E-01

ENSBTAG00000046517	-1.04E+00	3.53E-01	2.89E-01	8.29E-01	1.23E-01	1.00E+00	-5.68E-01	4.40E-01	-1.13E+00	3.49E-01
ENSBTAG00000009233	5.12E-01	2.60E-01	1.69E+00	6.66E-12	1.44E+00	4.66E-09	8.58E-01	9.44E-04	1.18E-01	1.00E+00
ENSBTAG00000009236	2.34E-01	8.23E-01	5.30E-01	1.58E-02	1.03E+00	3.32E-07	8.43E-01	4.91E-05	4.71E-01	2.31E-01
ENSBTAG00000009237	6.89E-01	1.44E-02	1.53E+00	6.04E-13	1.68E+00	2.02E-15	1.64E+00	1.25E-14	5.21E-01	1.93E-01
ENSBTAG00000003308	-1.30E+00	7.16E-01	-8.71E-02	1.00E+00	-1.10E+00	3.90E-01	-5.85E-01	7.61E-01	4.04E-02	1.00E+00
ENSBTAG00000009547	4.36E-02	1.00E+00	-1.49E-01	6.68E-01	-9.84E-02	8.96E-01	-2.41E-01	3.53E-01	-1.30E-01	1.00E+00
ENSBTAG00000021248	-6.86E-02	1.00E+00	5.80E-01	3.79E-03	2.81E-01	2.46E-01	-1.87E-01	5.32E-01	1.85E-01	8.71E-01
ENSBTAG00000003953	9.89E-02	1.00E+00	1.50E+00	2.90E-07	1.53E+00	1.27E-07	1.07E+00	4.43E-04	2.62E-01	9.19E-01
ENSBTAG00000038613	1.74E-01	1.00E+00	7.24E-01	5.68E-01	1.05E+00	2.63E-01	1.45E-01	1.00E+00	9.91E-01	7.42E-01
ENSBTAG00000039782	4.36E-01	6.81E-01	6.17E-01	9.20E-02	7.97E-01	1.99E-02	2.47E-01	6.83E-01	2.05E-01	1.00E+00
ENSBTAG00000047163	0.00E+00	1.00E+00	0.00E+00	1.00E+00	-5.13E-15	1.00E+00	0.00E+00	1.00E+00	0.00E+00	1.00E+00
ENSBTAG00000040014	5.37E-03	1.00E+00	-1.91E+00	4.69E-01	6.45E-01	9.78E-01	-1.48E-01	1.00E+00	6.36E-03	1.00E+00
ENSBTAG00000010291	2.97E-01	6.66E-01	1.72E+00	3.77E-17	1.55E+00	2.26E-14	7.54E-01	5.59E-04	-2.32E-01	8.01E-01
ENSBTAG00000005600	1.26E-02	1.00E+00	7.71E-02	1.00E+00	-1.25E-01	1.00E+00	-1.91E+00	4.39E-01	-1.91E+00	8.00E-01
ENSBTAG00000038627	-1.89E+00	1.58E-01	-7.18E-01	4.82E-01	-1.53E+00	7.31E-02	-1.36E+00	1.17E-01	-5.10E-01	1.00E+00
ENSBTAG00000048231	1.91E+00	8.23E-01	0.00E+00	1.00E+00	-5.13E-15	1.00E+00	0.00E+00	1.00E+00	0.00E+00	1.00E+00
ENSBTAG00000017175	-5.50E-02	1.00E+00	3.36E-02	1.00E+00	6.37E-01	5.84E-03	5.47E-01	2.48E-02	4.39E-03	1.00E+00
ENSBTAG00000003191	5.59E-01	1.70E-01	9.70E-01	8.95E-05	2.56E-01	4.79E-01	-5.77E-01	3.82E-02	-5.16E-01	3.25E-01
ENSBTAG00000047781	-1.91E+00	8.23E-01	6.91E-02	1.00E+00	-1.91E+00	4.28E-01	-1.91E+00	4.39E-01	-1.91E+00	8.00E-01
[truncated: 550,047 more chars]
